# Centaur antibodies: Engineered chimeric equine-human recombinant antibodies

**DOI:** 10.3389/fimmu.2022.942317

**Published:** 2022-08-19

**Authors:** Ronit Rosenfeld, Ron Alcalay, Anat Zvi, Alon Ben-David, Tal Noy-Porat, Theodor Chitlaru, Eyal Epstein, Ofir Israeli, Shirley Lazar, Noa Caspi, Ada Barnea, Eyal Dor, Inbar Chomsky, Shani Pitel, Efi Makdasi, Ran Zichel, Ohad Mazor

**Affiliations:** ^1^ Department of Biochemistry and Molecular Genetics, Israel Institute for Biological Research, Ness Ziona, Israel; ^2^ Department of Biotechnology, Israel Institute for Biological Research, Ness Ziona, Israel; ^3^ Veterinary Center for Preclinical Research, Israel Institute for Biological Research, Ness Ziona, Israel

**Keywords:** antibody engineering, phage display, equine V-genes, anti-toxins, anti-venoms, botulinum

## Abstract

Hyper-immune antisera from large mammals, in particular horses, are routinely used for life-saving anti-intoxication intervention. While highly efficient, the use of these immunotherapeutics is complicated by possible recipient reactogenicity and limited availability. Accordingly, there is an urgent need for alternative improved next-generation immunotherapies to respond to this issue of high public health priority. Here, we document the development of previously unavailable tools for equine antibody engineering. A novel primer set, EquPD v2020, based on equine V-gene data, was designed for efficient and accurate amplification of rearranged horse antibody V-segments. The primer set served for generation of immune phage display libraries, representing highly diverse V-gene repertoires of horses immunized against botulinum A or B neurotoxins. Highly specific scFv clones were selected and expressed as full-length antibodies, carrying equine V-genes and human Gamma1/Lambda constant genes, to be referred as “Centaur antibodies”. Preliminary assessment in a murine model of botulism established their therapeutic potential. The experimental approach detailed in the current report, represents a valuable tool for isolation and engineering of therapeutic equine antibodies.

## Introduction

Therapeutic life-saving polyclonal antitoxin and antivenom antibodies (Abs) are mostly produced in large animals, in particular horses (Equus caballus), possessing a robust immune system and large blood volume (1). Nevertheless, the use of native sera raises concerns regarding post-treatment complications such as serum sickness and anaphylaxis, as well as complications owing to limited availability, poor regulation, lack of standardization and other bio-technological relevant aspects. Consequently, alleviation of these concerns by improvement of antisera therapeutic products represents an issue of high clinical priority (https://www.who.int/teams/control-of-neglected-tropical-diseases/snakebite-envenoming/antivenoms; https://www.who.int/news/item/19-08-2018-who-issues-new-recommendation-on-antivenom-for-snakebites (2, 3);).

Development of potent recombinant monoclonal Abs (mAbs) may represent a beneficial substitute for polyclonal antisera (4–6), as they were already proven to be essential molecules for diagnosis and treatment of a variety of human oncologic, autoimmune and metabolic diseases as well as infections and intoxications (7). As of 2022, more than 100 monoclonal Abs were licensed by the US FDA and hundreds more are being investigated in clinical studies ( (8); https://www.antibodysociety.org/resources/approved-antibodies).

Hence, several anti-toxin recombinant mAbs were isolated from hyper-immune animals but not yet from horses, probably due to a lack of molecular tools appropriate for engineering Abs of equine origin. As a general rule, all the strategies employed for Ab generation [including phage display (PD), yeast display, single B cell sorting, humanization, *in vitro* affinity maturation (9)], require a primary step consisting of the amplification of desired Ab coding genes. Particularly, species-specific primers directed for amplifying rearranged B-cell mRNA sequences coding Ab variable regions, are essential. The design of such primers is therefore of outmost importance and requires broad genetic information regarding the immunoglobulin (Ig) repertoire of the organism.

The organization and diversity of the equine Ig gene repertoire was previously addressed by several studies. Accordingly, the IGH locus on the horse chromosome 24 includes at least 52 IGHV, 40 IGHD, 8 IGHJ and 11 IGHC (representing mu, alpha, delta, epsilon and 7 gamma chain genes); the IGK locus on chromosome 15 includes at least 60 IGKV, 5 IGKJ and 1 IGKC; the IGL locus on chromosome 8 (which in horses, is prevalently expressed compared to the IGKV) includes at least 144 IGLV, 7 IGLJ, and 7 IGLC (10–15). The above detailed information was consolidated here for the generation of a unique novel set of primers, designed to enable efficient and accurate PCR amplification of equine variable gene segments.

The PD technology is widely used for *in vitro* selection of specific mAbs (9). Universal PD libraries are generated from naïve organisms and therefore include a broad range of V-gene segments irrespective to antigen specificity. Yet, libraries constructed on the basis of lymphocytes collected from immunized organisms are highly beneficial for the direct isolation of high affinity mAbs of a desired antigen specificity. As of today, antibody-PD was applied for the generation of mAbs originating from several species, including human, camel, llama, alpaca, chimpanzee, macaque, pig, rabbit, mice, chicken and shark (16), however PD has not been reported for the isolation of horse-derived mAbs in spite of their potential therapeutic value.

The current report documents the generation of PD immune-horse libraries using a novel unique primer set (EquPD v2020), the use of these libraries for isolation of equine recombinant mAbs directed to botulinum toxin A and B, the generation of chimeric horse-human mAbs (to be referred as “Centaur Abs” in analogy to the mythological creature), and finally assessment of their neutralization potential in a murine model. To the best of our knowledge, this study exemplifies, for the first time, the successful development of equine recombinant mAbs.

## Materials and methods

### Animal studies

Animals were treated in accordance with regulations outlined in the U.S. Department of Agriculture (USDA) Animal Welfare Act and the conditions specified in the Guide for Care and Use of Laboratory Animals, National Institute of Health, 2011. Animal studies were approved by the local ethics committee for animal experiments (protocol numbers H-02-14 and M-05-22).

### Horse blood samples

The horses in this study were used for the production of pharmaceutical botulinum antitoxin preparation (17). The horses were hyper-immunized with toxoid of BoNT/A or BoNT/B, prepared by dialyzing the toxins against 0.14% formalin at 35°C for two weeks.

Sera samples were collected using Vacutte^®^ (Greiner bio-one, 455071). Peripheral PBMCs were obtained from blood samples collected from immunized horses using BD Vacutainer^®^ CPT™ (BD, 362782).

### Cells

ExpiCHO-S (Thermo scientific, A29127) cells were used for expression of recombinant chimeric full-length Abs as detailed below/above.

### Bacterial strains

Bacterial of the TG1 strain [F’ traD36 proAB lacIqZ DM15] supE thi-1 D(lac-proAB) D(mcrB-hsdSM)5(rK -mK -)] were used for phage display (PD) library construction and handling.

### Recombinant proteins and toxins

The following BoNT-related proteins were used as target antigens in the described study. BoNT/A heavy chain (HC) C-terminal domain (H_C_/A; amino acids 872-1296, 49.6 kDa); BoNT/B HC C-terminal domain (H_C_/B; amino acids 859-1290, 51.9 kDa); BoNT/B light chain (LC/B; amino acids 1-433, 50.1 kDa); BoNT/A and BonT/B LC-H_N_ proteins, consist of the LC and the N-terminal translocation domain (H_N_) of the heavy chain (LC-H_N_/A; amino acids 1-871, 101.1 kDa; LC-H_N_/B; amino acids 1-861, 100 kDa). The sequences of BoNT/A and BoNT/B antigens corresponds to GenBank accession numbers BAH79821.1 and M8116.1, respectively. All recombinant antigens contained 6xhis tag. The proteins were expressed and purified as described previously (18–20).

BoNT/A and BoNT/B native toxins prepared as previously described (21).

### Single chain Fv (scFv-) PD library construction

All amplification PCR reactions were performed using Advantage 2 DNA polymerase mix and buffer (Takara, 639202); 10 mM dNTPs (Promega,C1145) and 0.5 µM of each primer [Desalted oligonucleotides (Sigma)] in a total of 25 µl volume.

Total RNA was purified from PBMCs using RNeasy mini kit (Qiagen GmbH, 74104) and used for cDNA synthesis (Verso cDNA synthesis kit; Thermo scientific, AB-1453). Heavy and light Ig variable domains (VH and VK/VL) amplification was performed directly from cDNA in two successive PCR reactions. First PCR amplification performed using FR1- and J-specific primers (indicated by upper case in **Table 1**; 1 min 95°CC; 30 cycles of: 20 s, 94°CC; 30 s, 55°CC; 1 min, 72°CC; 5 min, 72°CC). The resulted PCR products were gel-purified [using QIAquick Gel extraction kit (Qiagen GmbH, 28706)] and used as a template for a second amplification (1 min 95°CC; 20 cycles of: 20 s, 94°CC; 30 s, 57°CC; 1 min, 72°CC; 5 min, 72°CC), using primers that adding flanking sequences (indicated by lower case) introducing linker-coding sequence as well as restriction recognition sites for subsequent cloning into pCC16 phagemid vector (22, 23). Following purification [using QIAquick PCR purification kit (Qiagen GmbH, 28104)], an equal amount of VH and Vκ/Vλ fragments (~200 ng each) were assembled into scFv fragment by two-step PCR. First 10 assembly PCR cycles were performed without primers addition (95°CC, 1 min; 10 cycles of: 95°CC, 15 s; 60°CC, 30 s; 72°CC, 30 s; 72°CC, 5 min). Next, products were diluted 1:10 in fresh reaction mixture including: ASS1_For (CTTTCTATGCGGCCCAGC) and ASS1_Rev (GATACCGGTGTATTTGCGCC) primers pair, for an additional 30 cycles (95°CC, 1 min; 30 cycles of: 95°CC, 15 s; 65°CC, 20 s; 72°CC, 1 min; 72°CC, 5 min).

**Table 1:** EquPD v2020 primer set for equine V-genes amplification used for PD libraries generation.

Primer name	Primer sequence [5’-3’]
**Heavy chain variable region (V_H_) amplification primers**	
Equ-V_H_-PD_For1	ctttctatgcggcccagccggccatggcc CAGGTGCAACTGAAGGAGTC
Equ-V_H_-PD_For2	ctttctatgcggcccagccggccatggcc CAGGTGCAACTGCAGGAGTC
Equ-V_H_-PD_For3	ctttctatgcggcccagccggccatggcc CAGGTGCAGCTGAAG**R**AGTC
Equ-V_H_-PD_For4	ctttctatgcggcccagccggccatggcc CAGGTGCAGCTGCAGGAGTC
Equ-V_H_-PD_For5	ctttctatgcggcccagccggccatggcc CAGGTGCAATTGAAGGAGTC
Equ-V_H_-PD_For6	ctttctatgcggcccagccggccatggcc CAGGT**H**CAACTGAAGGAGTC
Equ-V_H_-PD_For7	ctttctatgcggcccagccggccatggcc CAGGTGCTACTGAAGGAGTC
Equ-V_H_-PD_Rev1.2	accaccaccaccggatcctcctcctcctgctgagcc TGAGGAGACGGTGACCAGG
Equ-V_H_-PD_Rev3	accaccaccaccggatcctcctcctcctgctgagcc TGAGGAGACGAAGACCAGGATG
**Kappa chain variable region (Vκ) amplification primers**	
Equ-Vκ-PD_For1.3	ggatccggtggtggtggttctggcggcggcggctcc GAC**R**TCGTGATGAC**S**CAGTCTCC
Equ-Vκ-PD_For2	ggatccggtggtggtggttctggcggcggcggctcc GACATCGTGTTGACCCAGTCTCC
Equ-Vκ-PD_For4	ggatccggtggtggtggttctggcggcggcggctcc GACGTCGTGTTGACCCAGTCTCC
Equ-Vκ-PD_For5	ggatccggtggtggtggttctggcggcggcggctcc GAGATCCAGATGACCCAGTCTCC
Equ-Vκ-PD_For6	ggatccggtggtggtggttctggcggcggcggctcc GATATTGTGATGACCCAGACTCC
Equ-Vκ-PD_For7	ggatccggtggtggtggttctggcggcggcggctcc GATGTTGTG**W**TGACCCAGACTCC
Equ-Vκ-PD_For9	ggatccggtggtggtggttctggcggcggcggctcc GAC**R**TCATGATGACCCAGTCTCC
Equ-Vκ-PD_Rev1	gataccggtgtatttgcgccacctgcggccgc TTTGAT**Y**TCCAG**Y**TTGGTCCC
Equ-Vκ-PD_Rev2	gataccggtgtatttgcgccacctgcggccgc TTTGAT**Y**TCCACCTTGGTCCC
Equ-Vκ-PD_Rev3	gataccggtgtatttgcgccacctgcggccgc TTTGAT**Y**TCCAACTTGGTCCC
Equ-Vκ-PD_Rev4	gataccggtgtatttgcgccacctgcggccgc TTTAATGTCCAGACGCGTCCC
**Lambda chain variable region (V_L_) amplification primers**	
Equ-V_L_-PD_For1	ggatccggtggtggtggttctggcggcggcggctcc CAGTCTGTGACCCAGCCCGC
Equ-V_L_-PD_For2	ggatccggtggtggtggttctggcggcggcggctcc CAGTCTGTGACTCAGCCCGC
Equ-V_L_-PD_For3	ggatccggtggtggtggttctggcggcggcggctcc CAGTCTCTGACCCAGCCCGC
Equ-V_L_-PD_For4	ggatccggtggtggtggttctggcggcggcggctcc CAGTCTCTGACTCAGCCCGC
Equ-V_L_-PD_For5	ggatccggtggtggtggttctggcggcggcggctcc CAGTCTGTGACGCAGCCCGC
Equ-V_L_-PD_For6	ggatccggtggtggtggttctggcggcggcggctcc CAGTCTGCCCTGACTCAGCC
Equ-V_L_-PD_For7	ggatccggtggtggtggttctggcggcggcggctcc TCTTCTAAGCTGACTCAGCC
Equ-V_L_-PD_For8	ggatccggtggtggtggttctggcggcggcggctcc TCTTCTATGCTGACTCAGCC
Equ-V_L_-PD_For9	ggatccggtggtggtggttctggcggcggcggctcc TCTTCTGCAGTGACTCAGCC
Equ-V_L_-PD_For10	ggatccggtggtggtggttctggcggcggcggctcc TCCTTGGAGCTGACTCAGCC
Equ-V_L_-PD_For12	ggatccggtggtggtggttctggcggcggcggctcc TCTGCCCTGACTCAGCC
Equ-V_L_-PD_For13	ggatccggtggtggtggttctggcggcggcggctcc CAAAGTAACCTGACTCAGCCGG
Equ-V_L_-PD_For14	ggatccggtggtggtggttctggcggcggcggctcc TCCTCCGCCCTGACTCAG
Equ-V_L_-PD_Rev1.2	gataccggtgtatttgcgccacctgcggccgc GA**Y**GGTCA**R**GTGGGTGCC
Equ-V_L_-PD_Rev3	gataccggtgtatttgcgccacctgcggccgc TAGGACGGTCAGGGTTGTC

Upper case letters indicate V-gene specific primer sequences, corresponding to the FR1 [forward (For) primers] and the J segment [reverse (Rev) primers]. Lower case letters indicate sequences engineered in the amplicons enabling their assembly into scFv and cloning into the pCC16 phagemid vector. Degenerative bases are designated by bold letters (R=A/G; H=A/C/T; S=C/G; Y=C/T; W=A/T).

For PD library construction, pCC16 plasmid and assembled scFvs were digested with *Nco*I and *Not*I restriction enzymes (Thermo Scientific, FD0574 and FD0595; reaction performed by 1U of each enzyme per 1 μg DNA in 20 μl reaction incubated 2-3 hrs at 37°CC). Both resulted pCC16 vector and scFv inserts, were gel-purified and then used in ligation reaction, performed at 5:1 vector:insert molar ratio [using 6U of T4 HC ligase (Thermo Scientific, EL0013) per 200 ng vector in 15 μl reaction volume with 2 hr incubation at RT and further overnight incubation at 4°CC] followed by inactivation at 70°CC for 5 min. Ligated DNA was introduced into E. coli TG1 cells (Lucigen, 60502) by electroporation (each ligation reaction was transformed to 30 µL TG1 cells). A total of 24 cloning reactions were performed for each constructed library. The transformed bacteria, representing the final scFv library were plated on YPD agar (BD, 242720) supplemented with 100 µg/mL ampicillin and 100 mM glucose and incubated overnight at 30°CC. PD library size was determined based on number of transformed colonies. Library/TG1 glycerol-stocks performed for harvested transformed colonies supplemented with 20% glycerol and stored at -80°CC.

The scFv-PD library integrity and complexity were analyzed by sequencing of random scFv fragments, amplified and sequenced using the vector-specific primers: TAB-RI-For (CCATGATTACGCCAAGCTTTGGAGCC) and CBD-As-Rev (GAATTCAACCTTCAAATTGCC).

### Libraries Ab repertoire sequencing and mapping

Profiling of the amplified V-genes represented in each equine-PD library was performed by HTS and subsequent V-gene bioinformatic analyses. Standard Illumina sequencing primers and Miseq Reagent Kit v3 paired-end (600 cycles) were used for the amplicon DNA libraries preparation. These libraries were sequenced on Illumina Miseq platform in the presence of 25% PhiX library. FastQC (https://www.bioinformatics.babraham.ac.uk/projects/fastqc) and Trim Galore! v0.6.3 (http://www.bioinformatics.babraham.ac.uk/projects/trim_galore/) were used for quality control of the raw DNA sequence reads using default parameters. The pre-processing of the data was conducted using the pRESTO software pipeline (version 0.7.0) (24). A Phred quality score of 10 was applied as quality filtering. Ab V(D)J segments assignment was determined using the IMGT/HighV Quest version 1.8.5 (25). Subsequent clonotype analysis of the library sequences was carried out using Change-O clonal assignment kit (26), implementing the hamming distance model as a distance metric between junction nucleotide sequences (hamming distance histograms are provided in **Supplementary Figure S1**).

### Phage Ab enrichment (Panning)

Libraries were panned against several BoNT-related antigens as detailed in the result section. All routine PD techniques and panning procedure performed essentially as described (27). Polystyrene 96-well plates (F96 Maxisorp Nunc-immuno plates; Thermo scientific, 442404) were used to directly absorb target antigens (at concentration of 5 μg/ml); M13KO7 helper phage (New England Biolabs, N0315) used to rescue library and enriched phages; 3% BSA or 2% skimmed milk in PBS were alternatively used as blocking solution (used to block both phages and panning plates); x10 washing steps were performed at the first panning cycle and x20 washing steps performed at the second and third cycles. In each cycle, the first wash performed using blocking solution following by x5 washing steps with PBST solution (PBS containing 0.05% Tween20) and remain washing steps performed by PBS. 1-5x10^10^ phages were used at each cycle as panning input. Elution of panning output phages performed by 100 mM Triethylamine (Sigma, T0886; 30 min at RT) following neutralization with 1/5 volume of 1M Tris-HCl, pH 7.4.

For output phages enrichment logarithmic TG1 culture were infected by eluted phages (30 min at 37°C without shaking followed by 30 min at 120 rpm).

Single colonies were randomly picked from the third cycle output and screened for specific binders isolation, using phage-ELISA against the target antigen.

### Single-chain Fv (scFv) individual clone diversity and sequence verification

TAB-RI_For and CBD-AS_Rev pCC16 phagemid-specific primers were used to amplify scFv fragments by colony PCR. Amplified fragments integrity and variability were evaluated by agarose gel and by restriction fragment size polymorphism (RFLP) using MvaI (Thermo scientific, FD0554; 0.5 μl MvaI used to digest 8.5 μl PCR product at 37°C for 1 hr). 1% and 3% GTG agarose gels used resolved each scFv fragment size and its related RFLP pattern, respectively. Nucleic acid sequence analysis of individual scFv fragments, was performed to the colony PCR product, using SeqStudio Genetic Analyzer (AppliedBiosystems).

### Production of full-length IgG horse-human mAbs

Phagemid DNA of the desired clones were isolated using QIAprep spin Miniprep kit (Qiagen, GmbH, 27106). Horse-derived VH and VL fragments of each mAb were separately amplified and cloned into a pcDNA3.1+ based expression vector including either human gamma1 constant region for the heavy variable fragments, or lambda constant regions for the light variable fragments. In these expression vectors, secretion of the Ab heavy/light chains is mediated by human Ig heavy and light leader sequences (coding the amino acid sequences MELGLSWVFLVALLRGVQC and MDMRAHVHLLGLLLLWLPGAKC, respectively). The resulted horse-human IgG1/Lambda chimeric mAbs were expressed using ExpiCHO™ Expression system (Thermo scientific, A29133) and purified on HiTrap Protein-A column (GE healthcare, 17-0403-03). The integrity and purity of the mAbs were analyzed using SDS-PAGE **Supplementary Figure S2**).

### ELISA

Standard ELISA was performed using microtiter plates coated with 5 μg/ml of relevant recombinant protein.

For detection of equine Abs in sera samples, AP-conjugated Rabbit anti-horse IgG (Sigma, A6063; used at 1:4000 working dilution) was applied following detection using PNPP substrate (Sigma, N1891).

For phage-ELISA, anti-M13 Ab (Sino Biological, 11973-MM05T; used at 300 ng/nl) followed by HRP-conjugated sheep anti-mouse (GE, LNA931V/AG; used at 1:2000 working dilution) were used, Detection performed with TMB substrate (Millipore, ES001).

For detection of recombinant chimeric Abs, AP-conjugated Donkey anti-human IgG (Jackson ImmunoResearch, 709-055-149; used at 1:2000 working dilution) was applied following detection using PNPP substrate.

Specificity ELISA of the Abs performed at least 3 times for each Ab in serial concentrations. Representative results are shown at constant Ab concentration (3 μg/ml). Plotted signals represent OD [405 nm] values of each Ab against its cognate specific antigen.

For BoNT native toxins binding evaluation, Indirect ELISA performed. Anti-BoNT/A or anti-BoNT/B rabbit polyclonal Abs (28) or anti-BoNT-HC murine mAbs (21) at a concentration of 5 μg/ml used for toxins [10 ng/ml] capturing. Tested mAbs were then reacted with captured toxins and detected as detailed above.

### Biolayer interferometry measurements

Biolayer interferometry (BLI) analysis was carried out using the Octet system (ForteBio, USA, Version8.1, 2015)). All steps were performed at 30°C with shaking at 1500 rpm in a black 96-well plates containing 200 μl solution in each well.

For affinity measurements, streptavidin-coated biosensors were loaded with biotinylated chimeric IgG mAb (5 μg/ml) to reach 2.5-3 nm wavelength shift followed by a wash. The sensors were then incubated for 300 sec with increasing concentrations of target antigen (association phase) and then transferred to buffer-containing wells for another 600 s (dissociation phase). Binding and dissociation were measured as changes over time in light interference after subtraction of parallel measurements from unloaded biosensors. Sensorgrams were fitted with a 1:1 binding model using the Octet data analysis software 8.1 (Fortebio, USA, 2015) and the presented values are an average of at least two repeated measurements.

For the binning experiments of anti-LC-H_N_/A mAbs pairs, streptavidin-coated biosensors were loaded with biotinylated chimeric IgG mAb (5 μg/ml) to reach 2.5-3 nm wavelength shift followed by a wash. Sensors were then incubated with a fixed antigen concentration (100 nM), washed and incubated with the mAb counterpart (non-biotinylated). Background signals were obtained from two parallel sensors incubated either with the tested Ab itself (non-biotinylated) or non-specific Ab.

### BoNT neutralization test in mice

Protection evaluation of the selected mAbs, was performed essentially as previously described (21). The methodology used in the current study involved an assay for anti-BoNT antibody potency recommended by the pharmacopeia. In this mouse neutralization assay, a toxin test dose is pre-incubated with the tested antibody *in vitro* and then the antibody-toxin mixture is injected to mice for *in vivo* evaluation of protection (29). Since the assay combines *in vitro* and *in vivo* procedures, we referred to it in the text as *in vitro*-*in vivo*. Briefly, 5 LD_50_ (determined by the Karber method (30)) of BoNT/A or BoNT/B were pre-mixed with 50 μg of each mAb in gelatin buffer (0.2% w/v gelatin in phosphate buffer, pH=6.4) in a total volume of 1 ml/animal. Toxin : Ab mixtures were incubated for 1 hr at 25°C and then intraperitoneally-administered to CD-1 females (Charles River, UK). Control groups were administered with solution containing only toxin (without Ab) or a mixture of toxin with isotype control anti-ricin MH75 mAb, that contains the same human Fc but targets a non-relevant antigen (31). Animal survival was monitored throughout 10 days post-administration.

## Results

### Equine V-gene amplification primer set design

As of today, Ab engineering tools and PD in particular, were broadly implemented for the generation of recombinant mAbs originating from various mammal species, yet they were not applied for the development of equine recombinant mAbs (16). To apply this technology for the isolation of equine Abs, we first designed a universal primer set which may provide an extensive coverage of the equine Ig-V-gene repertoire. All available information and datasets pertaining to equine Ab sequences were implemented, to develop an updated tool for engineering equine Abs. A total of 920, 119 and 353 NCBI GenBank sequences [provided in **Supplementary Datas 1–3**], annotated as equine Ig VH, VK and VL loci, respectively (10, 11, 14) were employed as the genomic basis for designing a unique primer set (to be referred as EquPD v2020) enabling equine V-gene amplification for the purpose of single-chain Fv (scFv)-PD libraries construction. Although equine light chains are largely dominated by lambda type (32, 33), amplification of both kappa and lambda light chain genes was intended to be enabled by the EquPD v2020 primer set.

The EquPD v2020 primer set was designed essentially as previously described (34). Primer set design criteria included broad coverage, minimum number of non-silent degenerative bases (in particular in the 5’ region of the primers, proximal to the template amplicon), and calculated Tm values above 58°C for the majority of primers (and never under 53°C Tm) to mitigate nonspecific targeting.

In order to enrich for productive primers, each primer was evaluated for its efficiency to serve in generation of PCR products using equine PBMCs-cDNA (obtained from mixed blood samples collected from five individual horses) as a template. Following this selection step, 4 out of 39 designed primers were eliminated. The final EquPD v2020 primer set, listed in **Table 1**, included only primers that enabled amplification of the desired products, as judged by size analysis and DNA sequencing. This newly-designed set comprised 7, 7 and 13 forward primers directed to the framework 1 (FR1) region of VH, Vκ and Vλ, respectively and 2, 4 and 2 reverse primers directed to the JH, Jκ and Jλ segments.

### PD libraries construction and complexity evaluation

**Figure 1:**
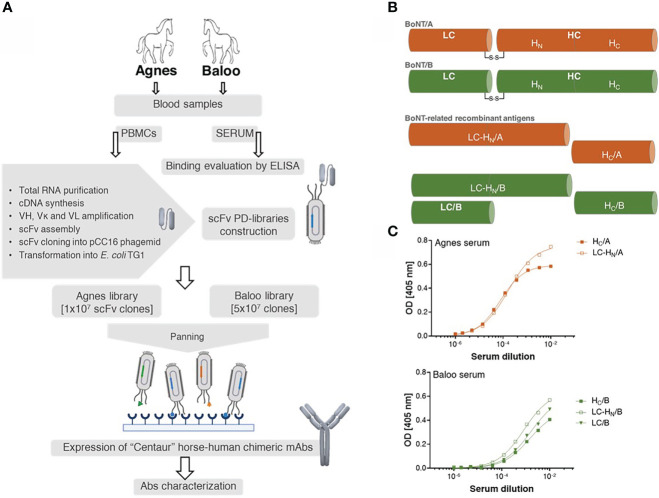
Study outline. **(A)** Schematic workflow of the development of horse-derived recombinant monoclonal antibodies (mAbs). Two horses, Agnes (♀) and Baloo (♂), immunized by administration of (*C*) *botulinum* inactivated neurotoxin (toxoid) serotype A or B (BoNT/A or BoNT/B, respectively) served as a source of PBMCs-cDNA. Two scFv-PD libraries were constructed based on Ab V-segments that were amplified using a newly designed primer set. Following enrichment by panning, specific mAbs were isolated, cloned and expressed in CHO cells as recombinant horse-human chimeric Centaur mAbs. **(B)** Schematic representation of BoNT/A, BoNT/B and their related recombinant antigens. BoNT protein is composed of a 50 kDa light chain (LC), which encodes the effector molecule (responsible for the BoNT toxicity) connected by a disulfide bridge to a 100 kDa heavy chain (HC). Receptor-mediated endocytosis followed by translocation of the light chain across the membrane into the neuronal cytosol is facilitated by the two HC functional domains, H_C_ and H_N_, correspondingly. **(C)** Binding capacity of Agnes’s and Baloo’s sera, was evaluated by ELISA against indicated BoNT/A- and BoNT/B- recombinant antigens. Serially diluted serum samples were tested against the indicated antigens.

To demonstrate the efficacy of the novel EquPD v2020 primer set in amplifying diverse Ab repertoires essential for high quality PD libraries, two scFv Ab libraries were constructed. The Ig genes were amplified from B-cells collected from two horses, Agnes and Baloo, immunized against Clostridium botulinum neurotoxin serotype A and B (BoNT/A and BoNT/B), respectively, by administration of inactivated toxoid (21)). In their native state, the BoNT neurotoxins represent the most potent known toxin, causing botulism that is mainly treated by polyclonal antitoxins Abs prepared in horses (4, 35). The general outline of the current study is schematically depicted in **Figure 1A**. Twenty-one days post-boost, sera samples were collected from the two horses and evaluated using ELISA for their binding capacity towards BoNT-related antigens (**Figures 1B, C**). Specifically, binding capacity was assessed against the following recombinant antigens: (i) the toxin heavy chain C-terminal domain of serotype A (HC/A), (ii) the toxin heavy chain C-terminal domain of serotype B (HC/B), (iii) BoNT/B light chain (LC/B), (iv) protein consisting of serotype A toxin LC and the translocation domain (HN) of the heavy chain (LC-HN/A), and (v) protein consisting of serotype B toxin LC and the translocation domain (HN) of the heavy chain (LC-HN/B). As depicted in **Figure 1C**, both Agnes and Baloo sera exhibited significant binding to their cognate antigens: Agnes-serum exhibited strong binding to both BoNT/A HC and LC-HN (DIL50 of approx. 13,000 and 6,000, respectively). Baloo-serum reacted against BoNT/B HC, LC-HN and LC (DIL50 of approx. 700, 1,200 and 600, respectively).

PBMCs obtained from peripheral blood collected from the two horses were used for RNA preparation and subsequent cDNA synthesis. The resulted cDNA samples were used as templates for the amplification of DNA molecules encoding for Ab V-segments using the newly-designed EquPD v2020 primer set. Finally, two PD libraries were generated, each representing 10 and 50 million distinct scFv Abs, derived from Agnes and Baloo PBMCs-cDNA, respectively.

**Figure 2:**
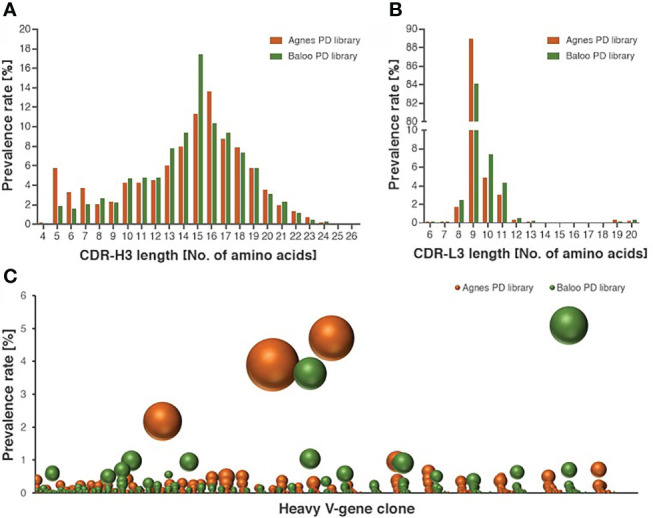
Characterization of the diversity of the equine PD library repertoires. **(A, B)** Length distribution of CDR-H3 **(A)** and CDR-L3 **(B)**, represented by V-segment sequences of Agnes- and Baloo-PD libraries. The histograms depict the amino-acid length of library sequences exhibiting a prevalence higher than 0.1%. **(C)** Prevalence rate of V-segment sequences (on the ordinate axis) assigned to each cluster (on the abscissa axis), as calculated by the clonotype analysis. Clusters composed of less than 100 sequences were not included in the chart presentation. The number of unique sequences present in each cluster is reflected by the sphere size (see also **Supplementary Table S1** for the detailed cluster analysis data).

To demonstrate the comprehensive nature of the libraries, high-throughput sequencing (HTS) analysis was applied to map the Ab repertoires of the horses-derived V-segments represented in the libraries. The analysis included a total of 5,086,173, 4,262,125, 3,837,731 and 1,770,165 HTS-obtained sequences for the heavy and light V-segments derived from Agnes-PD library and for the heavy and light V-segments derived from Baloo-PD library, respectively. Inference of the V(D)J segments that were rearranged to generate the library Ab repertoire, was conducted by applying the IMGT/HighV-Quest analysis (25). A total of 236,211, 145,198, 158,850, and 78,099 V-domain coding productive unique sequences were determined for the Agnes heavy and light chains and for the Baloo heavy and light chains, respectively. Of note, each unique sequence represents more than a single assembled HTS-generated sequences (up to thousands), consequently yielding a total of 746,073, 659,579, 501,845 and 283,255 counted sequences identified in the Agnes heavy and light chains and the Baloo heavy and light chains repertoires, respectively. The IMGT/HighV-Quest analysis enabled the delimitation of the FR and complementarity determining regions (CDR) segments of the assembled V-domain, in particular permitting the allocation of the CDR-3 of each V-segment and the determination of their length distributions. As shown in **Figure 2A**, a typical mammalian length distribution ranging between 4-26 amino acids was observed for the CDR-H3 of both libraries, while CDR-L3 length distribution emerged as highly restricted (with high prevalence for CDR-L3 segments of 9 amino acids in length, **Figure 2B**). A subsequent clonotype analysis of the heavy chain V-domain productive sequences, which classifies sequences into B-cell clusters, was conducted. This analysis enables the distinction of common ancestor origin of clones, suggestive of a shared primordial lymphocyte (26). The analysis disclosed 45,836 and 43,585 clusters identified in the Agnes and Baloo heavy chain repertoires, respectively. The data in **Figure 2C** demonstrates the prevalence rate of V-segment sequences (on the ordinate axis) assigned to each cluster (on the abscissa axis), as well as the number of unique sequences represented by each cluster (reflected by the sphere size). As low as 3 and 2 clusters in the Agnes and Baloo repertoire, respectively, were found to predominate with respect to both the fraction of the sequences assigned to each cluster and the number of unique sequences covered (accounting approx. 9 and 10% of all heavy chain sequences, respectively).

### PD libraries panning and specific mAbs isolation

**Figure 3:**
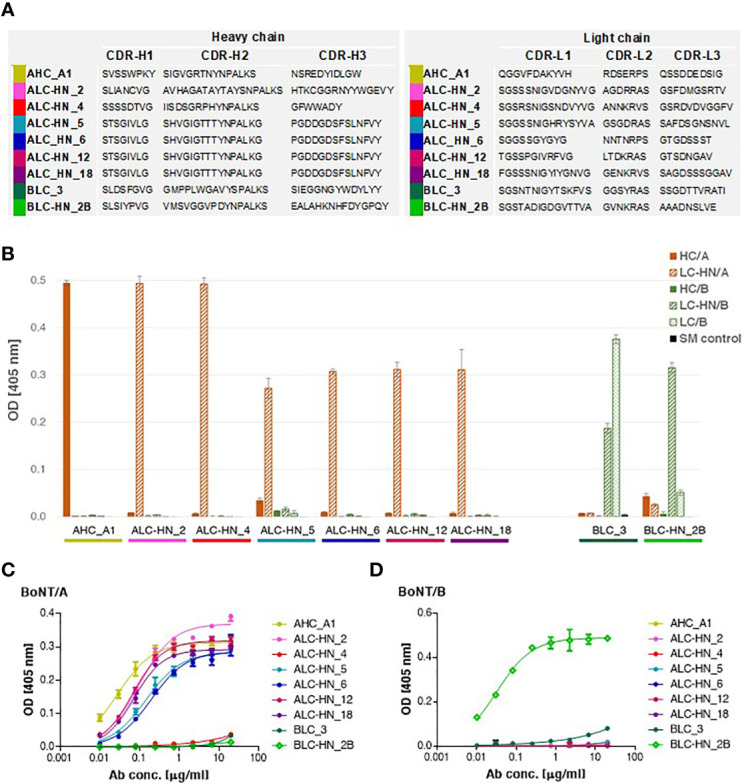
Characterization of the anti-BoNT Centaur mAbs. **(A)** Amino acid sequences of the VH and VL CDR segments of the anti-BoNT selected mAbs. CDR positions are indicated according to the Kabat system (36). **(B)** Specificity of the nine selected mAbs determined by ELISA against the indicated BoNT subunits. Data represent average of triplicates ± SD. Skim milk (SM) was used as control protein, as indicated in the in-set legend. **(C, D)** Binding ability of the selected mAbs was evaluated against BoNT native toxins. Each indicated mAb was serially diluted and tested in duplicates by indirect ELISA against BoNT/A **(C)** and BoNT/B **(D)**. Data represent average ± SD.

With the objective of enriching for equine specific antigen binding mAbs, three consecutive panning steps were performed for Agnes-PD library against BoNT/A HC and LC-HN and for Baloo-PD library against BoNT/B LC and LC-HN. Following enrichment, single clones were individually screened by phage-ELISA to determine their ability to bind their cognate antigens. Clones identified as specific binders were further subjected to scFv diversity analysis by restriction fragment size polymorphism (RFLP; using MVAI restriction enzyme) and direct DNA sequencing. All four panning procedures resulted in high frequency of positively binding clones. Panning against HC/A, LC/B and LC-HN/B antigens yielded a single dominant specific binder per each antigen, whereas panning against LC-HN/A yielded six specific binders. The amino acid sequences of the VH and VL CDRs of the nine selected mAbs are presented in **Figure 3A**. Sequence analysis revealed that the VH of LC-HN/A-specific mAbs: ALC-HN_5, 6, 12 and 18, although originating from amplification mediated by disparate primer pairs, share common coding sequence. Yet, these mAbs are distinct since their VL fragments are unique. This observation owes to the inherent tendency of the Ab PD libraries not to preserve the physiological VH VL pairing of native antibodies but rather enable creation of novel pairings resulting in augmented variability. The results strongly suggest that in the ALC-HN_5, 6, 12 and 18 mAbs the common VH mainly contributes to the LC-HN/A antigen binding while the distinct VL may impact the mAbs conformational and stability characteristics.

Interestingly, although the two libraries were constructed with commensurate amounts of kappa and lambda type light chains, the specific binders selected carried only lambda light chains. The propensity for selection of mAbs which include lambda type, may be expected on the basis of the documented high frequency of lambda chain expression in horses lymphocytes (32, 33).

### Generation and characterization of full-length Centaur mAbs

The nine specific isolated binders were sub-cloned and expressed in mammalian cells as a horse-human chimeric full-length IgG1/Lambda mAbs, comprising of equine Ig variable segments and human constant domains. These mAbs represent a novel type of recombinant chimeric Abs described here for the first time, termed Centaur mAbs. The full-length mAbs were purified from the cell cultures and further analyzed. Binding analysis by ELISA against various antigens confirmed the mAbs specificity for their cognate antigens, in line with the panning antigens employed for their selection (**Figure 3B**). As expected, BLC_3 Ab, selected against LC/B, also efficiently bound LC-HN/B.

Next, binding of the Centaur mAbs to their cognate target was interrogated in the context of the native toxins. Accordingly, an indirect ELISA was employed, in which each tested mAb was reacted with the BoNT/A (**Figure 3C**) and BoNT/B (**Figure 3D**) toxins that were specifically immobilized by rabbit polyclonal Abs. As shown, binding of 7 out of 9 tested mAbs to their respective native toxin serotype was confirmed. Only weak binding could be detected for the 2 Centaur mAbs ALC-HN_4 and BLC_3. To determine whether this observation is indeed related to poor binding, these two mAbs were inspected also by a modified assay using anti-BoNT/A HC and anti-BoNT/B HC mAbs for the capturing of each toxin. The assay yielded similar results (not shown), confirming that in spite of their high binding ability for the respective toxin subunits, they exhibit low binding towards the native toxins.

**Figure 4:**
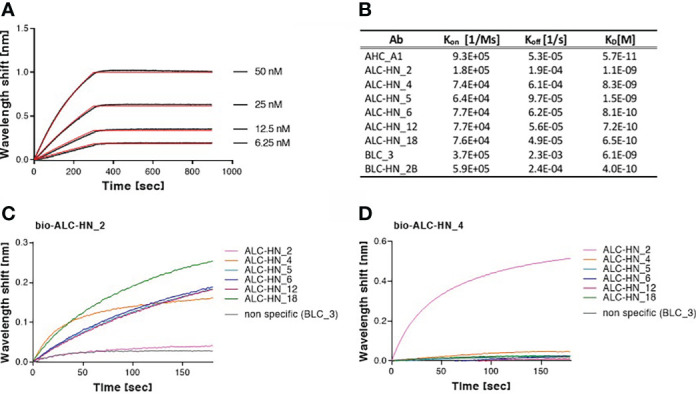
Biolayer interferometry (BLI) analyses of Centaur mAbs. **(A, B)** Binding kinetic of the selected mAbs. Each tested mAb was biotinylated, immobilized on a streptavidin sensor and incubated with increasing amounts of targeted antigen. Binding kinetics were fitted using the 1:1 binding model. Each BLI analysis was independently-repeated at least two times, resulting in highly-similar calculated values. **(A)** Representative assay results are shown for ALC-HN_18 mAb. Gray sensograms represent binding profiles in the presence of each indicated antigen concentration. Fitting curves are depicted in red. Binding BLI curves of the nine tested mAbs are provided in **Supplementary Figure S3**. **(B)** Table of the binding kinetic parameters of the nine selected mAbs, presented as average values. **(C, D)** Identification of two distinct epitopes, recognized by the six selected anti-LC-H_N_/A mAbs by epitope binning. Each mAb was biotinylated (bio-mAb), immobilized on streptavidin sensor and saturated with LC-H_N_/A recombinant protein. The complex was then incubated with each one of the indicated mAbs. Background signals were obtained from parallel sensors incubated with the tested mAb itself (non-biotinylated) or non-specific mAb. **(C)** Time 0 represent the binding to bio-ALC-HN_2-LC-H_N_/A complex, demonstrating a unique epitope targeted by this Ab. **(D)** Time 0 represent the binding to bio-ALC-HN_4-LC-H_N_/A complex. This representative data set, indicate that ALC-HN_4, 5, 6, 12 and 18 recognize a competitive epitope, (see **Supplementary Figure S4** for all binning data).

To further characterize the Abs, their binding kinetic parameters (K_on_, K_off_ and K_D_) were next determined using biolayer interferometry (BLI). BLI results and the binding kinetic parameters of the nine selected mAbs are shown in **Figures 4A, B** and **Supplementary Figure S3**. The data established high affinity binding of all mAbs. BLC_3 and ALC-HN_4 mAbs (directed against the LC/B and the LC-H_N_/A domains, respectively) exhibited lower affinity values (average K_D_ of 6.1 and 8.3 nM, respectively) than that determined for the remaining 7 mAbs (average K_D_ values of 1.5 nM to 0.06 nM).

To define the epitope recognition of the six selected anti-LC-H_N_/A Centaur mAbs, BLI-binning competitive analysis was performed. Accordingly, each individual mAb was biotinylated, immobilized to a streptavidin sensor, loaded with recombinant LC-H_N_/A, and then competed with each of the other mAbs (non-biotinylated). In this assay, the ability of two Abs to bind LC-H_N_/A concomitantly (as reflected by a significant wavelength shift) indicates that they bind non-overlapping distinct epitopes (37). Of note, the inability of two mAbs to bind simultaneously (thus competing Abs) does not necessarily indicate that they bind the same epitope, as their competition may suggest steric interference (due to adjacency of their binding sites) or allosteric conformational disruption. Thus, the assay allows classification of Ab-binding sites on the LC-H_N_/A, based on the proximity of their respective epitopes. Sensograms of the 6 mAbs interactions with the pre-complex bio-ALC-HN_2-LC-H_N_/A, depicted in **Figure 4C**, demonstrate a marked wavelength shift manifested by each of the tested mAbs. This result clearly confirmed that the ALC-HN_2 mAb recognizes a unique epitope that does not compete with epitopes recognized by either one of the other five tested mAbs. No wavelength shift was obtained by non-specific Ab or by the ALC-HN_2 mAb itself (non-biotinylated), confirming the accuracy of the data. BLI analysis was then performed for the next five anti-LC-H_N_/A mAbs: ALC-HN_4, 5, 6, 12 and 18 (a representative result is shown for ALC-HN_4 in **Figure 4D** and the results of the entire binning experiment are provided in **Supplementary Figure S4**). While a marked wavelength shift was demonstrated by ALC-HN_2 mAb when tested on LC-H_N_/A attached to either of the five biotinylated mAbs, none of these five mAbs could bind LC-H_N_/A complexed with either one of them. This result could be explained by the fact that mAbs: ALC-HN_5, 6, 12 and 18 share the same heavy chain and probably recognize an identical epitope and that ALC-HN_4 (encoded by a unique sequence) recognizes an overlapping epitope. In summary, the six tested mAbs recognize two distinct epitopes on the LC-H_N_/A protein.

### *In vitro-in vivo* protection experiments

**Figure 5:**
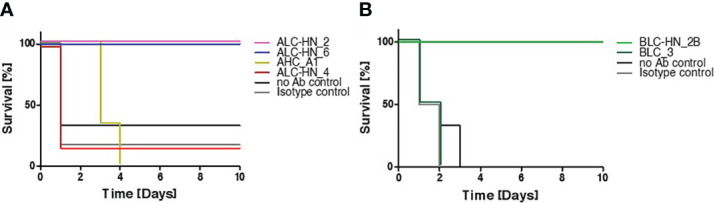
Protection of mice against BoNT intoxication by the Centaur mAbs. Mice (n = 6) were intraperitoneally-injected with 5xLD_50_ of BoNT/A **(A)** or BoNT/B **(B)** pre-incubated with 50 μg of each indicated Centaur mAb. Control groups included “no Ab control” and “non-specific isotype control” (anti-ricin mAb). Mice survival was monitored for 10 days post-administration.

In the current study, BoNT-specific recombinant mAbs were developed as a proof of concept for demonstrating the feasibility of the PD approach for generation of chimeric Centaur mAbs. To substantiate the therapeutic value of the novel mAbs, they were further assessed for their potential to countermeasure the detrimental effect of the toxin in a murine model of BoNT intoxication (**Figure 5**), essentially as previously described (21). Mice were intraperitoneal-administrated with 5xLD_50_ serotypes A or B that were individually pre-incubated with each of the tested mAbs. Control groups included mice injected with toxin only (no Ab control) and mice injected with toxin pre-incubated with a non-specific isotype control (the anti-ricin MH75 mAb (31)). Mice were monitored for 10 days post-administration. Approx. 70-80% of the mice in the control groups succumbed within the first day post-intoxication with BoNT/A (**Figure 5A**) and 100% succumbed within 2-3 days post-intoxication with the BoNT/B toxin (**Figure 5B**).

Since mAbs ALC-HN_5, 6, 12 and 18 were similar with respect to their heavy-chain germline origin (**Figure 3A**) as well as the antigen targeted epitope (**Figure 4D**) and affinities (**Figure 4B**), only one of them (ALC-H_N__6) was tested. As shown in **Figure 5A**, full protection was obtained upon pre-incubation of BoNT/A with either mAb ALC-HN_2 or ALC-HN_6, recognizing distinct epitopes on the LC-H_N_/A protein. A delayed MTTD was observed following pre-incubation of the toxin with AHC_A1. No protection was observed following pre-incubation of the toxin with ALC-HN_4. With regard to BoNT/B neutralization, mAb BLC-HN_2B fully prevented the toxin lethal effect, while the mAb BLC_3 had no protective effect (**Figure 5B**). Of note, the mAbs ALC-HN_4 and BLC_3 that failed to prevent toxicity of BoNT/A and BoNT/B, respectively, exhibited also poor binding to the native forms of the toxins (see **Figures 3C, D**).

## Discussion

The development of efficient neutralizing Abs for antitoxin and antivenom treatments mostly relies on sera collected from large animals (often horses) previously hyper-immunized with the respective substance. In spite of the possibility to apply lifesaving treatment using polyclonal antisera, there is an urgent need for developing available antitoxins that exhibit therapeutic consistency, improved safety, efficacy, and that are compatible with large-scale biotechnological processes (https://www.who.int/teams/control-of-neglected-tropical-diseases/snakebite-envenoming/antivenoms (1–3);). Recombinant Abs meet all these requirements and therefore represent the substitute of choice for polyclonal antisera (4–6).

Amongst the current available tools for recombinant Ab generation, PD is considered the most powerful and widely used platform. Specifically, PD was frequently used for the successful selection of diagnostic as well as neutralizing mAbs targeting various CDC class A categorized select agents (38) as botulinum, anthrax and ricin toxins (16, 39, 40) as well as animals venoms (3).

In the study documented in the current report, we aimed to meet the need for horse-derived new-generation recombinant immunotherapy agents by implementing the powerful PD technology. To the best of our knowledge, PD has not been used before for the isolation of horse-derived mAbs. The development of equine-therapeutic mAbs requires detailed information of the Ig gene repertoire of this animal, which possesses significant distinctiveness compared to other mammals used for designing recombinant therapeutic Abs. Of note, Ab expression mechanisms and germline repertoires have extensively been studied in humans and mice, while limited information is available for other organisms, including horses (41, 42).

The available (yet incomplete) information pertaining to equine V-gene repertoire in genetic databases was successfully used in the current study to design the EquPD v2020 primer set for the amplification of equine V-segments. A recent report documenting HTS for the characterization of expressed Ab repertoire in two non-immunized horses (43), indicated prevalently expressed V-gene subgroups (IGHV2 and IGLV8) which are also covered by the current EquPD v2020 primer set.

The implementation of the PD technology for the generation of horse-human chimeric Centaur mAbs was exemplified in the current work for the generation of anti BoNT specific mAbs. Interestingly, three of the four panning procedures in the course of PD library construction yielded only a single binder directed to the cognate target antigen and one panning resulted in the isolation of several distinct specific Abs. One plausible explanation for the observed restricted variability of selected binders is that following repeated and prolonged exposure to the same target antigen, the Ab repertoire diversity (as well as affinity maturation) is limited and dominated by a few B cell clones (44, 45). Furthermore, additional non-mutually exclusive explanations for the observed limited variability, includes the high stringency of the panning procedure and the fact that the panning employed antigens representing recombinant bacterial-expressed versions of portions of the inactivated holotoxins which served for the immunization of the horses. Thus, theoretically, panning may have exerted enrichment of a rare subset of specific antibodies from the original immunoglobulin anti toxin repertoire.

Although the PD libraries constructed in this work represented both kappa and lambda light chain types in similar ratio, all selected binders carried lambda chains. This result together with the prevalence reported for lambda chains in the equine expressed Ab repertoire (32, 33) may suggest that in the future, it will be sufficient to include only lambda light chain specific primers for construction of libraries used for isolation of horse-derived mAbs. A similar situation of strong propensity for the use of a distinct light chain [the kappa chain in this case (46)] is known in the generation of murine PD libraries, consequently in the case of murine libraries, the primers are specifically directed for amplification of kappa segments.

The main neutralization mechanism of anti-BoNT Abs is inhibition of the interaction of the toxin with its neuron receptor by targeting the HC domain (5). However, neutralization may also be mediated by targeting other domains, such as the translocation domain (HN) or the catalytic domain (LC) (4, 16). The data presented here, clearly demonstrated the protection potential achieved by 2 of the 3 LC-HN/A-specific Centaur mAbs (ALC-HN_2 and ALC-HN_6 (**Figure 5A**), directed to separate epitopes on the targeted protein and by LC-HN/B-specific BLC-HN_2B mAb. Conversely, AHC_A1 mAb, directed to the HC/A domain was only efficient in delaying, but not preventing the mortality of the animals, in spite of its high affinity. Therefore, the performance of AHC_A1 apparently contradicts the expected efficacy of Abs directed against the receptor binding domain of the toxin as explained above. Future studies will be necessary to assess the value of this mAb as well as to identify the neutralizing epitopes, protection and therapeutic potential of other mAbs described here. Furthermore, combinations of the selected mAbs may also be assessed in line with the previously demonstrated synergistic neutralization effect of anti-BoNT Abs (47, 48).

In conclusion, the EquPD v2020 primer set, designed for the accurate amplification of equine Ig genes, may be applied in various Ab engineering approaches, including PD, single B cell, humanization, *in vitro* affinity maturation (9). Equine recombinant mAbs and Centaur horse-human chimeric recombinant mAbs in particular, may serve in the future as an alternative improved lifesaving treatment, which could circumvent some of the disadvantages of native horse antisera.

## Data availability statement

The datasets presented in this study can be found in online repositories. The names of the repository/repositories and accession number(s) can be found below: NCBI databank under accession number PRJNA848968.

## Author contributions

Conceptualization, RR and OM; methodology and investigation, RR, RA, AZ, AB-D, TN-P, EE, OI, SL, NC, AB, ED, IC, SP, and EM; writing, review and editing, RR, AZ, AB-D, TC, RZ, and OM; supervision, RZ and OM. All authors have read and agreed to the published version of the manuscript.

## Acknowledgments

We wish to express our gratitude to our colleagues Dr. Emanuelle Mamroud, Dr. Amir Rosner and Dr. Tseela David for fruitful discussions and support. We would like to thank Moshe Mantzur, Moshe Aftalion and Miki Saraf for skillful and devoting technical assistance. Icons included in **Figure 1** were created with BioRender.com.

## Conflict of interest

The authors declare that the research was conducted in the absence of any commercial or financial relationships that could be construed as a potential conflict of interest.

## Publisher’s note

All claims expressed in this article are solely those of the authors and do not necessarily represent those of their affiliated organizations, or those of the publisher, the editors and the reviewers. Any product that may be evaluated in this article, or claim that may be made by its manufacturer, is not guaranteed or endorsed by the publisher.
