# Supplementary material for: Centaur antibodies: Engineered chimeric equine-human recombinant antibodies
Source: Front Immunol. 2022 Aug 19;13:942317. doi: 10.3389/fimmu.2022.942317 (PMC9437483; doi:10.3389/fimmu.2022.942317)
Supplement: Supplementary file 2 [file DataSheet_1.pdf]

Supplementary Material

1 Supplementary Data

NCBI Gene Bank equine Ig V-segments sequences employed for designing the EquPD v2020 primer set are provided in Supplementary Data 1-3. Of note, the use of the NCBI Gene Bank-derived dataset rather than the IMGT database (usually serving for Ig gene sequence retrieval) owes to the fact that the IMGT database did not include any equine relevant information at the time of the primer set design. The IMGT database was updated posteriorly yet, bioinformatic confrontation of the EquPD v2020 primer set with the IMGT database equine sequences failed to detect any sequence homology. The reason for this discrepancy is elusive as of today and was not further investigated.

1.1 **Supplementary Data 1. NCBI Genbank Equine Ig VH mRNA.** A total of 920 NCBI Genbank sequences, annotated as Equus caballus Ig VH sequences which constituted the dataset employed for the designing of the EquPD v2020 primer set.

```
>HQ403631.1 Equus caballus clone VDJ6 dev-stage foal immunoglobulin heavy
chain variable region mRNA, partial cds
CAGGTGCAGCTGCAGGAGTCGGGACCTGGCCTGGTGAAGCCCTCGCAGACCCTCTCCCTCACCTGCACTGTCTCTGG
ATTCTCTTTGAGCAGTTACGGTGTAGGCTGGGTCCGCCAGGCTCCAGGAAAAGGGCTGGAATATGTTGGTGGTATAG
CTAGTAGTGGAAGTGCAAACTACAACCCAGCCCTGAAGTCCCGAGCCAGCATCACCAAGGACACCTCAAAGAGCCAA
GTTTATCTGACGCTGAACAGCCTGACAGGCGAGGACACGGCCGTCTATTACTGTGCGAGAACCTTTATATATGGTTA
CTATGCTGGTAGTTACTATGCGGGTTATGATATAGACTACTGGGGCCAGGGTATCCTGGTCACCGT
>HQ403622.1 Equus caballus clone VDJ6 dev-stage neonate immunoglobulin heavy
chain variable region mRNA, partial cds
CAGGTGCAGCTGCAGGAGTCGGGACCTGGCCTGGTGAAGCCCTCGCAGACCCTGTCCCTCACCTGCACTGTCTCTGG
ATTATCTTTGAGCAGTAATGCTGTAGGCTGGGTCCGCCAGGCTCCAGGAAAAGGGCTGGAGTGGGTTGGTGTTATAT
ATGGTAGTGAAAGTACATACTACAACCCAGCCCTGAAGTCCCGAGCCAGCATCACCAAGGACACCTCAAAGAGCCAA
GTTTATCTGACGCTGAACAGCCTGACAGGCGAAGACACGGCCGTCTATTACTGTGCAGGATATGGTTTCTATGATAC
ATATCATGATTATTATGAGCTAGACTACTGGGGCCAGGGTATCCTGGTCACCGT
>HQ403617.1 Equus caballus clone VDJ1 dev-stage neonate immunoglobulin heavy
chain variable region mRNA, partial cds
CAGGTGCAGCTGAAGGAGTCGGGACCTGGCCTGGTGAAGCCCTCGCAGACCCTCTCCCTCACCTGCACTGTCTCTGG
ATTCTCTTTGAGCAGTTATGCTGTATACTGGGTCCGCCAGGCTCCAGGAAAAGGGCTGGAATATGTTGGTAATATAT
ATGGTAGTTCAACTGCAGACTACAACCCAGCCCTGAAGTCCCGAGCCAGCATCACCAAGGACACCTCAAAGAGCCAA
GTTTATCTGACGCTGAACACCCTGACAGGCGAGGACACGGCCGTCTATTACTGTGCGAGAGAGGGGGACGATATGTG
GTATGCTTACGCCTACTGGGGCCAGGGTATCCTGGTCACCGT
>HQ403616.1 Equus caballus clone VDJ9 dev-stage fetus immunoglobulin heavy
chain variable region mRNA, partial cds
CAGGTGCAGCTGAAGGAGTCAGGACCTGGCCTGGTGAAGCCCTCGCAGACCCTGTCCCTCACCTGCACTGTCTCTGG
ATTCTCTTTGAGCAGTTACGGTGTAGGCTGGGTCCGCCAGGCTCCAGGAAAAGGGCTGGAATATGTTGGTGGTATAG
CTAGTAGTGGAAGTGCAAACTACAACCCAGCCCTGAAGTCCCGAGCCAGCATCACCAAGGACACCTCAAAGAGCCAA
GTTTATCTGACGCTGAACAGCCTGACAGGCGAGGACACGGCCGTCTATTACTGTGCGAGATCCGGTTATGGTTATGC
TTATGGTATAAACTACTGGGGCCAGGGTATCCTGGTCACCGT
>HQ403614.1 Equus caballus clone VDJ7 dev-stage fetus immunoglobulin heavy
chain variable region mRNA, partial cds
CAGGTGCAGCTGAAGGAGTCGGGACCTGGCCTGGTGAAGCCCTCGCAGACCCTCTCCCTCACCTGCACTGTCTCTGG
ATTCTCTTTGAGCAGTTATGCTGTATACTGGGTCCGCCAGGCTCCAGGAAAAGGGCTGGAATATGTTGGTGCTATAG
```

CTAGTAGTGGAAGTGCAAACTACAACCCAGCCCTGAAGTCCCGAGCCAGCATCACCAAGGACACCTCCAAGAGCCAA
GTTTATCTGACGCTGAACAGCCTGACAGGCGAGGACACGGCCGTCTATTACTGTGCGAAAGATCAAGCTACGGTAGC
GGTTGGTCACTATTTTGGCTACTGGGGCCAGGGTATCCTGGTCACCGT
>HQ403612.1 Equus caballus clone VDJ5 dev-stage fetus immunoglobulin heavy chain variable region mRNA, partial cds
CAGGTGCAGCTGAAGGAGTCGGGACCTGGCCTGGTGAAGCCCTCGCAGACCCTGTCCCTCACCTGCACTGTCTCTGG
ATTATCTTTGAGCAGTAATGCTGTAGGCTGGGTCCGCCAGGCTCCAGGAAAAGGGCTGGAGTGGGTTGGTGTTATAT
ATGGTAGTGAAAGTACATACTACAACCCAGCCCTGAAGTCCCGAGCCAGCATCACCAAGGACACCTCAAAGAGCCAA
GTTTATCTGACGCTGAACAGCCTGACAGGCGAAGACACGGCCGTCTATTACTGTGCAGGATGCATAGGCAGCTATGC
TTATGGTATAAACTACTGGGGCCAGGGTATCCTGGTCACCGT
>HQ403610.1 Equus caballus clone VDJ3 dev-stage fetus immunoglobulin heavy chain variable region mRNA, partial cds
CAGGTGCAGCTGAAGGAGTCGGGACCTGGCCTGGTGAAGCCCTCGCAGACCCTGTCCCTCACCTGCACTGTCTCTGG
ATTATCTTTGAGCAGTAATGCTGTAGGCTGGGTCCGCCAGGCTCCAGGAAAAGGGCTGGAGTGGGTTGGTGTTATAT
ATGGTAGTGAAAGTACATACTACAACCCAGCCCTGAAGTCCCGAGCCAGCATCACCAAGGACACCTCAAAGAGCCAA
GTTTATCTGACGCTGAACAGCCTGACAGGCGAAGACACGGCCGTCTATTACTGTGCAGGATGTATGACTGTACTGGT
CATGGATGTGTCTACATATTATGGTATAAACTACTGGGGCCAGGGTATCCTGGTCACCGT
>HQ403609.1 Equus caballus clone VDJ2 dev-stage fetus immunoglobulin heavy chain variable region mRNA, partial cds
CAGGTGCAGCTGAAGGAGTCAGGACCTGGCCTGGTGAAGCCCTCGCAGACCCTCTCCCTCACCTGCACTGTCTCTGG
ATTATCTTTGAGCAGTAATGCTGTAGGCTGGGTCCGCCAGGCTCCAGGAAAAGGGCTGGAATTTGTTGGTGCTATAT
ATGGTAGTGCAAGTGCAAACTACAACCCAGCCCTGAAGTCCCGAGCCAGCATCACCAAGGACACCTCAAAGAGCCAA
GTTTATCTGACGCTGAACAGCCTGACAAGCGAGGACACGGCCGTCTATTACTGTGCAGGAGGCTATGATACTTTCTA
TTTTGGCTACTGGGGCCAGGGTATCCTGGTCACCGT
>HQ403608.1 Equus caballus clone VDJ1 dev-stage fetus immunoglobulin heavy chain variable region mRNA, partial cds
CAGGTGCAGCTGAAGGAGTCGGGACCTGGCCTGGTGAAGCCCTCGCAGACCCTCTCCCTCACCTGCACTGTCTCTGG
ATTATCTTTGAGCAGTAATGCTGTAGGCTGGGTCCGCCAGGCTCCAGGAAAAGGGCTGGAATTTGTTGGTGCTATAT
ATGGTAGTGCAAGTGCAAACTACAACCCAGCCCTGAAGTCCCGAGCCAGCATCACCAAGGACACCTCAAAGAGCCAA
GTTTATCTGACGCTGAACAGCCTGACAAGCGAGGACACGGCCGTCTATTACTGTGCAGGAGGTTTTGACTGTACTGG
TCATGGATGTGTCTACATCGTAAACTACTGGGGCCAGGGTATCCTGGTCACCGT
>DQ125456.1 Equus caballus clone 4-12 immunoglobulin heavy chain variable region mRNA, partial cds
CAGGTGCAGCTGAAGGAGTCAGGACCTGGCCTGGTGAAGCCCTCGCAGACCCTCTCCCTCACCTGCACTGTCTCTGG
ATTATCTTTGAGCAGTTATGGTGTGGGCTGGGTCCGCCAGGCTCCAGGAAAAGGTCTGGAATTTGTTGGCGGTATAC
TTAGTAGTGGAAGGGCAAACTACAACCCAGCCCTGAAGTCCCGAGCCAGCATCACCAGGGATACAACAAAGAACCAA
GTTTATCTGACGCTGAACAGCCTGACAGGCGAGGACACGTCCGTCTATTACTGTGCGAGATCATTTGCTAGTGGTGG
TTCTTACTACGACTATGCGATAAACTTCTGGGGCCAGGGTATCCTGGTCACCGTCTCG
>DQ125448.1 Equus caballus clone 4-4 immunoglobulin heavy chain variable region mRNA, partial cds
CAGGTGCAACTGAAGGAGTCAGGACCTGGCCTGGTGAAGCCCTCGCAGACCCTGTCCCTCACCTGCACTATCTCTGG
ATTCTCTTTGACCAGTGCCAGTGTAGACTGGGTCCGCCAGGCTCCAGGAAAAGGGCTGGAATTTGTTGGTGGTATAG
CGACTAGTGGGCGTGCAAATTACAACCCAGTCCTGAAGTCCCGCGCCACTATCACCAGAGACACCTCAAAGAGCCAA
GTTTATCTGACGCTGAACAGTCTGACAGGCGAGGACACGGCCGTCTATTACTGTGCGGAATCCTACTATGATGGTGT
TGGTGGTAATTACTACTTTTGGGGCCAGGGTATCCTGGTCACCGTCTCG
>DQ125445.1 Equus caballus clone 4-1 immunoglobulin heavy chain variable region mRNA, partial cds
CAGGTGCAGCTGCAGGAGTCAGGACCTGGCCTGGTGAAGCCCTCGCAGACCCTGTCCCTCACCTGCACTGTCTCTGG
ACTCTCTTTGAACAGTTACGATGTAAACTGGGTCCGCCAGGCTCCAGGAAAAGGGCTGGAAGTAGTTGGTAGTATAA
GTGACAGTGGAATTGCGGTGTACAACCCAGCCCTGAAGTCCCGAGCCAGCATCACCAAGGACACCTCAAACAGTCAA
GTTTATCTGACGCTGAACAGCCTGACAGGCGAGGACACGGCCGTCTATTACTGTGCGAGAGGGAATTTTGCGTTTGA
CTACTGGGGCCAGGGTATCCTGGTCACCGTCTCG
>DQ125444.1 Equus caballus clone 3-15 immunoglobulin heavy chain variable region mRNA, partial cds

CAGGTGCAACTGAAGGAGTCAGGACCTGGCCTGGTGAACCCCTCGCAGACCCTGTCCCTCACCTGCTTTGTCTCTGG
ATTCTCTTTGACGAGTTGGCATGTAGGCTGGGTCCGCCAGGCTCCAGGAAAAGGGCTGGAATTTGTCGGTGGTATAC
CTGTCATCGGAGAGGCATACTACAACCCAGTGCTGAAGTCCCGAATCAGCATTACTAAGGACACCTCGAAGAGCCAA
GTTTATCTGACGCTGAACAGCCTGACAGACGAGGACACGGCCGTCTATGCCTGTGCGAGGTTAAGGAACTGGTATGG
TGATTACTACAGTGACATGGACTATTGGGGCCAGGGTATCCTGGTCACCGTCTCG
>DQ125413.1 Equus caballus clone 1-2 immunoglobulin heavy chain variable region mRNA, partial cds
CAGGTGCAACTGAAGGAGTCAGGACCTGGCCTGGTGAAGCCCTCGCAGACCCTCTCCCTCACCTGCACTGTCTCTGG
ATTATCAGTGAGCAGTAATGGTGTGGCCTGGGTCCGCCAGGCTCCAGGAAAAGGGCTGGAATTTGTCGGTGTTATAC
ATACTGATGGAGGTGTTGACTACAACCCAGCCCTGAAGTCCCGAGGCAGCATCACTAGGGACATCTCAAAGAGCCAA
CTTTATCTGACGCTGAACACACTGACAGGCGAGGACACGGCCGTCTATTACTGTGCGCGACATGCTAGTACTGGTGC
TTACCTTTACCCCTTTGACTATTGGGGCCAGGGTATCCTGGTCACCGTCTCG
>KF748754.1 Equus caballus clone IGVDJ57 immunoglobulin heavy chain variable region (IGH) mRNA, partial cds
ATGAGTCACCTGTGGTTCTTCCTCTTTCTGGTGGCCGCTCCTACATGTGTCCTGTCCCAGGTGCAACTGAAGGAGTC
AGGACCTGGCCTGGTGAAGCCCTCGCAGACCCTGTCCCTCACCTGCACTGTCTCTGGATTCTCTTTGAGCAGTTACG
GTGTAGGCTGGGTCCGCCAGGCTCCAGGAAAAGGGCTGGAATATGTTGGTGGTATAGCTAGTAGTGGAAGTGCAAAC
TACAACCCAGCCCTGAAGTCCCGAGCCAGCATCACCAAGGACACCTCAAAGAGCCAAGTTTATCTGACGCTGAACAG
CCTGACAGGCGAGGACACGGCCGTCTATTACTGTGCGAGAAGGCGGGGGGCCGATTATTATGGTGCTATTGACTACA
CAAATTATGGTATAAACTACTGGGGCCAGGGCATC
>KF748751.1 Equus caballus clone IGVDJ54 immunoglobulin heavy chain variable region (IGH) mRNA, partial cds
ATGAGTCACCTGTGGTTCTTCCTCTTTCTGGTGGCCGCTCCTACATGTGTCCTGTCCCAGGTGCAACTGAAGGAGTC
AGGACCTGGCCTGGTGAAGCCCTCGCAGACCCTCTCCCTCACCTGCACTGTCTCTGGATTATCTTTGAGCAGTAATG
CTGTAGGCTGGGTCCGCCAGGCTCCAGGAAAAGGGCTGGAATTTGTTGGTGCTATATATGGTAGTGCAAGTGCAAAC
TACAACCCAGCCCTGAAGTCCCGAGCCAGCATCACCAAGGACACCTCAAAGAGCCAAGTTTATCTGACGCTGAACAG
CCTGACAGGCGAGGACACGGCCGTCTATTACTGTGCGAGATACTACGGTTATGGTTATGCTACCTTTTTTGGCTACT
GGGGCCAGGGCACC
>KF748739.1 Equus caballus clone IGVDJ42 immunoglobulin heavy chain variable region (IGH) mRNA, partial cds
ATGAATCACCTGTGGTTCTTCCTCTTTCTGGTGACCGCTCCTACATGTGTCCTGTCCCAGGTGCAACTGAAGGAGTC
GGGACCTGGCCTGGTGAAGCCCTCGCAGACCCTGTCCCTCACCTGCACTGTCTCTGGATTATCTTTGAGCAGTAATG
CTGTAGGCTGGGTCCGCCAGGCTCCAGGAAAAGGGCTGGAGTGGGTTGGTGTTATATATGGTAGTGAAAGTACATAC
TACAACCCAGCCCTGAAGTCCCGAGCCAGCATCACCAAGGACACCTCAAAGAGCCAAGTTTATCTGACGCTGAACAG
CCTGACAGGCGAGGACACGGCCGTCTATTACTGTGCGAGAAACTACGGTTATGGTTATGCTTTTGGCTACTGGGGCC
AGGGCACC
>KF748737.1 Equus caballus clone IGVDJ40 immunoglobulin heavy chain variable region (IGH) mRNA, partial cds
ATGAATCACCTGTGGTTCTTCCTCTTTCTGGTGGCCGCTCCTACATGTGTCCTGTCCCAGGTGCAACTGAAGGAGTC
AGGACCTGGCCTGGTGAAGCCCTCGCAGACCCTCTCCCTCACCTGCACTGTCTCTGGATTCTCTTTGAGCAGTTATG
CTGTATACTGGGTCCGCCAGGCTCCAGGAAAAGGGCTGGAATATGTTGGTGCTATATATGGTAGTGCAAGTGCAAAC
TACAACCCAGCCCTGAAGTCCCGAGCCAGCATCACCAAGGACACCTCAAAGAGCCAAGTTTATCTGACGCTGAACAG
CCTGACAGGCGAGGACACGGCCGTCTATTACTGTGCGAGGCATAGCAGCTATGCTTTTGGCTACTGGGGCCAGGGCA
CC
>KF748729.1 Equus caballus clone IGVDJ32 immunoglobulin heavy chain variable region (IGH) mRNA, partial cds
ATGAGTCACCTGTGGTTCTTCCTCTTTCTGGTGGCCGCTCCTACATGTGTCCTGTCCCAGGTGCAACTGAAGGAGTC
AGGACCTGGCCTGGTGAAGCCCTCGCAGACCCTGTCCCTCACCTGCACTGTCTCTGGATTCTCTTTGAGCAGTTACG
GTGTAGGCTGGGTCCGCCAGGCTCCAGGAAAAGGGCTGGAATATGTTGGTGGTATAGCTAGTAGTGGAAGTGCAAAC
TACAACCCAGCCCTGAAGTCCCGAGCCAGCATCACCAAGGACACCTCAAAGAGCCAAGTTTATCTGACGCTGAACAG
CCTGACAGGCGAGGACACGGCCGTCTATTACTGTGCGAGTTTAGGCGGTAGTTCCTGGTACTCTTATGGCTACGTGG
ATCACTGGGGCCAGGGCACC
>KF748714.1 Equus caballus clone IGVDJ17 immunoglobulin heavy chain variable region (IGH) mRNA, partial cds
ATGAATCACCTGTGGTTCTTCCTCTTTCTGGTGGCCGCTCCTACATGTGTCCTGTCCCAGGTGCAACTGAAGGAGTC
AGGACCTGGCCTGGTGAAGCCCTCGCAGACCCTCTCCCTCACCTGCACTGTCTCTGGATTATCTTTGAGCAGTTATG

CTGTAGGCTGGGTCCGCCAGGCTCCAGGAAAAGGGCTGGAATATGTTGGTGCTATATATGGTAGTGCAAGTGCAAAC
TACAACCCAGCCCTGAAGTCCCGAGCCAGCATCACCAAGGACACCTCAAAGAGCCAAGTTTATCTGACGCTGAACAG
CCTGACAGGCGAGGACACGGCCGTCTATTACTGTGCGAGATATAGTTATGGTAGTTACTATGGTATAGACTACTGGG
GCCAGGGCATC
>KF748708.1 Equus caballus clone IGVDJ11 immunoglobulin heavy chain variable region (IGH) mRNA, partial cds
ATGAATCACCTGTGGTTCTTCCTCTTTCTGGTGACCGCTCCTACATGTGTCCTGTCCCAGGTGCAACTGAAGGAGTC
GGGACCTGGCCTGGTGAAGCCCTCGCAGACCCTGTCCCTCACCTGCACTGTCTCTGGATTATCTTTGAGCAGTAATG
CTGTAGGCTGGGTCCGCCAGGCTCCAGGAAAAGGGCTGGAGTGGGTTGGTGTTATATATGGTAGTGAAAGTACATAC
TACAACCCAGCCCTGAAGTCCCGAGCCAGCATCACCAAGGACACCTCAAAGAGCCAAGTTTATCTGACGCTGAACAG
CCTGACAGGCGAAGACACGGCCGTCTATTACTGTGCAGCTGCTATAGCAGCTATGCTTACTTATGGTATAGACTACT
GGGGCCAGGGCATC
>KF748707.1 Equus caballus clone IGVDJ10 immunoglobulin heavy chain variable region (IGH) mRNA, partial cds
ATGAATCACCTGTGGTTCTTCCTCTTTCTGGTGGCCGCTCCTACATGTGTCCTGTCCCAGGTGCAACTGAAGGAGTC
AGGACCTGGCCTGGTGAAGCCCTCGCAGACCCTCTCCCTCACCTGCACTGTCTCTGGATTCTCTTTGAGCAGTTATG
CTGTAGGCTGGGTCCGCCAGGCTCCAGGAAAAGGGCTGGAATATGTTGGTGCTATATATGGTAGTGCAAGTGCAAAC
TACAACCCAGCCCTGAAGTCCCGAGCCAGCATCACCAAGGACACCTCAAAGAGCCAAGTTTATCTGACGCTGAACAG
CCTGACAGGCGAGGACACGGCCGTCTATTACTGTGCGACCCCTACGGACGGTTATGGTTATTATGGTATAAACTACT
GGGGCCAGGGCATC
>KF748706.1 Equus caballus clone IGVDJ9 immunoglobulin heavy chain variable region (IGH) mRNA, partial cds
ATGAATCACCTGTGGTTCTTCCTCTTTCTGGTGGCCGCTCCTACATGTGTCCTGTCCCAGGTGCAACTGAAGGAGTC
AGGACCTGGCCTGGTGAAGCCCTCGCAGACCCTCTCCCTCACCTGCACTGTCTCTGGATTCTCTTTGAGCAGTTATG
CTGTATACTGGGTCCGCCAGGCTCCAGGAAAAGGGCTGGAATATGTTGGTGCTATATATGGTAGTGCAAGTGCAAAC
TACAACCCAGCCCTGAAGTCCCGAGCCAGCATCACCAAGGACACCTCAAAGAGCCAAGTTTATCTGACGCTGAACAG
CCTGACAGGCGAGGACACGGCCGTCTATTACTGTGCGCGCTATGGTTATGGTGGTGCTTACTACTATGGCTACGTGG
ATCACTGGGGCCAGGGCACC
>KF748705.1 Equus caballus clone IGVDJ8 immunoglobulin heavy chain variable region (IGH) mRNA, partial cds
ATGAATCACCTGTGGTTCTTCCTCTTTCTGGTGGCCGCTCCTACATGTGTCCTGTCCCAGGTGCAACTGAAGGAGTC
GGGACCTGGCCTGGTGAAGCCCTCGCAGACCCTGTCCCTCACCTGCACTGTCTCTGGATTCTCTTTGAGCAGTTACG
GTGTAGGCTGGGTCCGCCAGGCTCCAGGAAAAGGGCTGGAATTTGTTGGTGCTATATATGGTAGTGCAAGTGCAAAC
TACAACCCAGCCCTGAAGTCCCGAGCCAGCATCACCAAGGACACCTCAAAGAGCCAAGTTTATCTGACGCTGAACAG
CCTGACAAGCGAGGACACGGCCGTCTATTACTGTGCAGGAGAGATACGGTTATGGTATTATTATGGTATAAACTACT
GGGGCCAGGGCATC
>KF748699.1 Equus caballus clone IGVDJ2 immunoglobulin heavy chain variable region (IGH) mRNA, partial cds
ATGAATCACCTGTGGTTCTTCCTCTTTCTGGTGGCCGCTCCTACATGTGTCCTGTCCCAGGTGCAACTGAAGGAGTC
AGGACCTGGCCTGGTGAAGCCCTCGCAGACCCTCTCCCTCACCTGCACTGTCTCTGGATTCTCTTTGAGCAGTTATG
CTGTAGGCTGGGTCCGCCAGGCTCCAGGAAAAGGGCTGGAATATGTTGGTGCTATATATGGTAGTGCAAGTGCAAAC
TACAACCCAGCCCTGAAGTCCCGAGCCAGCATCACCAAGGACACCTCAAAGAGCCAAGTTTATCTGACGCTGAACAG
CCTGACAGGCGAGGACACGGCCGTCTATTACTGTGCGAGAGAGAGGAGTGGTAGTTCCTGGTACTTACGTTATGGCT
ACGTGGATCACTGGGGCCAGGGCACC
>KF748698.1 Equus caballus clone IGVDJ1 immunoglobulin heavy chain variable region (IGH) mRNA, partial cds
ATGAATCACCTGTGGTTCTTCCTCTTTCTGGTGGCCGCTCCTACATGTGTCCTGTCCCAGGTGCAACTGAAGGAGTC
GGGACCTGGCCTGGTGAAGCCCTCGCAGACCCTGTCCCTCACCTGCACTGTCTCTGGATTATCTTTGAGCAGTAATG
CTGTAGGCTGGGTCCGCCAGGCTCCAGGAAAAGGGCTGGAGTGGGTTGGTGTTATATATGGTAGTGAAAGTACATAC
TACAACCCAGCCCTGAAGTCCCGAGCCAGCATCACCAAGGACACCTCAAAGAGCCAAGTTTATCTGACGCTGAACAG
CCTGACAGGCGAAGACACGGCCGTCTATTACTGTGCAGGAGGCTGTATCTCATATGGTTACTATAGTAGGAGTTGCT
ATTTTTTTGGCTACTGGGGCCAGGGCACC
>HQ403643.1 Equus caballus clone VDJ9 dev-stage adult immunoglobulin heavy chain variable region mRNA, partial cds

CAGGTGCAGCTGCAGGAGTCGGGACCTGGCCTGGTGAAGCCCTCGCAGACCCTGTCCCTCACCTGCACTGTCTCTGA
ATACTCTGTAGGCAGGAATGTTATAGGCTGGGTCCGCCAGGCTCCAGGAAAAGGGCTGGAGTGGGTTGCAATAATAT
ACGCTAGTGAGAGGGTAAAGTACAACCCAGCCCTGAAGAACCGAGCCAGCATCACCAAGGACACCTCAAAGAGCCAG
GTTCATCTGACGCTGAATAGCCTGACAGGCGAAGACACGGCCGTCTATTACTGTGCAGGGCTTGATAACAACTATTT
ATTAGGAGTACTCTATTGGGGCCAGGGTATCCTGGTCACCGT
>HQ403642.1 Equus caballus clone VDJ8 dev-stage adult immunoglobulin heavy chain variable region mRNA, partial cds
CAGGTGCAGCTGCAGGAGTCGGGACGTGGCCTGGTGAAGTCCTCGCAGACCCTCTCCCTCACCTGTAGTATTTCTGG
ATTCTCTTTGACGAGTGAAACTGTGGACTGGGTCCGCCAGGCTCCAGGAAAAGGGCTGGAATATGTTGGTGGCTTAG
CCGACCGTGGAAAGGGTGGCGTTGGCCCATATAATCCAGCCCTGAAGTCCCGAGCCAGAGTCACCAAGGACGACTCA
AAGAACGAAGTTTATCTGACGCTGGACGCCGTGACAAGGGAGGACACGGCCGTCTATTACTGTGCGAGAGGCGGCTA
TGCTTATGAAGATTACTACTACCAAGGGACTTATGGGTTCCTGGATCACTGGGGCCAGGGTATCCTGGTCACCGT
>HQ403641.1 Equus caballus clone VDJ7 dev-stage adult immunoglobulin heavy chain variable region mRNA, partial cds
CAGGTGCAGCTGAAGGAGTCGGGACCTGGCCTGGTGAAGCCCTCGCAGACCCTCTCCCTCACCTGCACTGTCTCTGG
GTTCTCTTTGAGCGATAGCGTTGTAGGCTGGGTCCGCCAGGCTCCAGGAAAAGGTCTGGAAGAGGTTGGATTGATGG
GTCCTAATGGAAGACCAGAGTACAAACCAGCCCTGAAGTCCCGAGCCAGCATCACCAAGGACACCTCCGAGAGTCAA
GTTTATCTGACGCTGAGCGGACTGACAGGCGAGGACACGGCCGTGTATTACTGTGCGAGATGGAAGTCTACTGGTGG
TGAAAGTTACTACCGTGATTCCATTTCTAACTGGGGCCAGGGTATCCTGGTCACCGT
>HQ403640.1 Equus caballus clone VDJ6 dev-stage adult immunoglobulin heavy chain variable region mRNA, partial cds
CAGGTGCAGCTGAAGGAGTCAGGACCTACCCTGGTGAAGCCCGCGCAGACCCTCTCCCTCACCTGCGCCGTCTCCGG
TCTGAATTTGAGCGGCAGTGCGGTAGGCTGGATCCGCCAGGCTCCAGAAAAAGGGCTGGAATGGGTGGGTGATATTC
TTAGTGATGGAAGTACATTCTACAGACCAGACCTGAAGTCCCGTGTCAGCATCGGCAAGGACACCTCAAAGAGCCAA
GTTTATCTGACGCTGAACACTGTGACAACGGAAGACACGGCCGTCTATTACTGTACGTTGTACACAGATCTTTGGGG
CCAGGGTATCCTGGTCACCGT
>HQ403639.1 Equus caballus clone VDJ5 dev-stage adult immunoglobulin heavy chain variable region mRNA, partial cds
CAGGTGCAGCTGCAGGAGTCAGGAGATGGCCGAATGAAGCCCTCAGAGACCCTCGCCCTCACCTGCATTGTCTCTGG
ACTAAGAAGTAGTGCCGGTGTGGCCTGGGTCCGCCAGGCTCCAGGAGGTGGTCTGGAATTTGTAGGCAGTAGAGAGA
GGACTGGACATGAAAACTACAACGCAGACCTGAAGTCCCGAGCCAGAGTCACCAAGGACGCCTACGGCCATAACGTT
TATCTGACGTTGACCGGCCTGACAAGTGACGACACGGCCGTCTATTACTGTGCACAATTAACGAATGGTGGAAATTC
GCCTTACGAAATACTGGAATGGGGCCAGGGTATCCTGGTCACCGT
>HQ403638.1 Equus caballus clone VDJ4 dev-stage adult immunoglobulin heavy chain variable region mRNA, partial cds
CAGGTGCAGCTGAAGGAGTCAGGAGATGGCCGAATGAAGCCCTCAGAGACCCTCGCCCTCACCTGCATTGTCTCTGG
ACTGCGAAGTAGTGCCGGTGTGGCCTGGGTCCGCCAGGCTCCAGGAGGTGGTCTGGAATTTGTAGGCAGTAGAGAGA
GGACTGGACATGAAAACTACAACGCAGACCTGAAGTCCCGAGCCAGAGTCACCAAGGACGCCTACGGCCATAACGTT
TATCTGACGTTGACCGGCCTGACAAGTGACGACACGGCCGTCTATTATTGTGTAGGAGGGGACATTTTGTTATGGCC
TTATGGCATAGACTATTGGGGCCAGGGTATCCTGGTCACCGA
>HQ403637.1 Equus caballus clone VDJ3 dev-stage adult immunoglobulin heavy chain variable region mRNA, partial cds
CAGGTGCAGCTGCAGGAGTCGGGACCTGGCCTGGTGAAGCCCTCGCAGACCCTGTCCCTCACCTGCACTGTCTCTGG
ATTCTCTTTGACCAGTTACGGTGTAGCCTGGGTCCGCCAGGCTCCAGGAAAAGGGCCAGAATTTGTAGCTGGAATGG
CTGCTAGTGGAAGTGATAACTACAGTCCAGTCCTGAAGGGACGAGTCAATATTTCTAAGGACAATGGAAAGAACCAG
GTTTATCTGACGCTGTACGACCTGACAAGCGAGGACACGGCCGTCTATTATTGTGTAGGACCGCTTGGTTGGGGTGG
TTTTTACTTTGAAAACTATTTCGGCCAGGGTATCCTGGTCACCGT
>HQ403636.1 Equus caballus clone VDJ2 dev-stage adult immunoglobulin heavy chain variable region mRNA, partial cds
CAGGTGCAGCTGCAGGAGTCAGGATCTGATCTCGTGAATCCCTCGCAGACCCTGTTCCTCACCTGCTCTGTTTCTGG
GCTCCCCTTGTTGGGGAACGAAGTAGTGTGGGTCCGTCAGAAGCCAGGCAAAGGGTTGGAATTCATTGGCCGGAGTG
CGGATGATGGAGGTGAGGTATATAATCCAGGTCTGGACCCCCGAGTCGTGATCACCAAGGACTATTCACAGAGTCAA
GTATTTCTTTGGATAAATGATGTGACGGGCGAGGACACGGCCGTCTATTTTTGCCTGACATTGATGACCACGAGAGA
CGACGCCTATTTTGACAATTGGGGCCAGGGTATCCTGGTCACCGT
>HQ403635.1 Equus caballus clone VDJ1 dev-stage adult immunoglobulin heavy chain variable region mRNA, partial cds

CAGGTGCAGCTGCAGGAGTCAGGACCTGGCCTGGTGAAGCCCTCGCAGACCCTCTCCCTCATCTGTACTGTCTCTGG
ATTATCTTTGTCGCGTAATGGAATAGGCTGGGTCCGCCAGGCTCCAGGAAAGGGTTTGGAATTTGTTGGTGTTATAC
TTCCTAGTAGAGTTGCAGACTACAACCCAGCCCTGAAGTCCCGAGCCAGCATCACCCAGGACACCTCAAAGACCCAA
GTTTATCTGACGCTGAACAGCCTGACAAGCGAGGACGAGGCCGTCTATTACTGTGCGGGAGGCTCGGCTTTAGGTGG
TGGTGTGACTTTTGACAGCTATCATATTATGTACTGGGGCCAGGGTATCCTGGTCACCGT
>HQ403634.1 Equus caballus clone VDJ9 dev-stage foal immunoglobulin heavy chain variable region mRNA, partial cds
CAGGTGCAACTGCAGGAGTCGGGCCCAGGACTGGTGCAGCCCTCACAGACCCTGTCCCTCACCTGCACTGTCACTGG
AGGCTCCATCACAAGCAGCTATTCTACCTGGAGCTGGTTACGCCAGCCTCCAGGGAAGGGGCTGGAGTACATGGGAG
ACATACTTTATGATGGTAGATCTTACTACAATCCTTCCTTCAAGAGCCGCACCTCCATCTCCAGAGACACCTCCAGG
AACCAGTTCTCCCTGCAGCTGAGCTCCGTGACCGCTGAGGACGCGGCCGTGTATTACTGTGCAGGGGACTACGGTTA
TGGACACGACTGGGGCCAGGGTATCCTGGTCACCGT
>HQ403633.1 Equus caballus clone VDJ8 dev-stage foal immunoglobulin heavy chain variable region mRNA, partial cds
CAGGTGCAGCTGAAGGAGTCGGGCCCAGGACAGGTGAAGCCCTCACAGACCCTCTCCCTCACCTGCTCTGTGTCTGG
AGTCTCCATCACAAGCAGTGGTGACTGGTGGAACTGGATCCGCCAGCCCCCAGGGAAGGGGCTGGAATGGATGGGGT
ACATAAGTTATAGTGGTAGCGCTTACTACACCACATCCCTCAAGAGCCGACTCTCCATCTCCAGAGACACGTCCAAG
GACCAGTTCTCCCTGCAGCTGAGCTCCGTGACCGCCGAGGACACGGCCGTTTATTACTGTGCCCCTATAACAGCTAT
GGTTACTGAGGGCTTAAACTACTGGGGACAGGGTATCCTGGTCACCGT
>HQ403632.1 Equus caballus clone VDJ7 dev-stage foal immunoglobulin heavy chain variable region mRNA, partial cds
CAGGTGCAGCTGCAGGAGTCGGGACCTGGCCTGGTGAAGCCCTCGCAGACCCTGTCCCTCACCTGCACTGTCTCTGG
ATTCTCTTTGAGCAGTTACGATGTAGGCTGGGTCCGCCAGGCTCCAGGAAAAGGGCTGGAATATGTTGGTGGTATTT
GGCGTGATGGAAGTGCAATGTACAACCCAGCCCTGAAGTCCCGAGCCAGCATTACCAAGGACACCTCAAAGAGCCAG
GTTTATCTGACGCTGAACAGCCTGACAGGCGAGGACACGGCCGTCTATTACTGTTGGTTATTGCGAGGGAGTTACTG
GGGCCAGGGTATCCTGGTCACCGT
>HQ403630.1 Equus caballus clone VDJ5 dev-stage foal immunoglobulin heavy chain variable region mRNA, partial cds
CAGGTGCAGCTGCAGGAGTCGGGACCTGGCCTGGTGAAGCCCTCGCAGACCCTGTCCCTCACCTGCACTGTCTCTGG
ATTCTCTTTGAGCAGTTACGGTGTAGGCTGGGTCCGCCAGGCTCCAGGAAAAGGGCTGGAATTTGTTGGTGGTTTAG
CGAGTAGTGGAAGTGAAAACTACAACCCAGCCCTGAAGTCCCGAGCCAGCATCACCAAGGACACCTCAAAGAGCCAA
GTTTATCTGACGCTGAACAGCCTGACAGGCGAAGACACGGCCGTCTATTACTGTGGGGATGGTGGTGCTTACTACAG
TGGTATAAACTACTGGGGCCAGGGTATCCTGGTCACCGT
>HQ403629.1 Equus caballus clone VDJ4 dev-stage foal immunoglobulin heavy chain variable region mRNA, partial cds
CAGGTGCAACTGCAGGAGTCAGGACCTGGCCTGGTGAAGCCCTCGCAGACCCTCTCCCTCACCTGCACTGTCTCTGG
ATTCTCTTTGAGCAGTTTTGCTGTAGGCTGGGTCCGCCAGGCTCCAGGAAAAGGGCTGGAATATGTTGGTGCTATAT
ATGGTAGTGCAAGTGCAAACTACAACCCAGCCCTGAAGTCCCGAGCCAGCATCACCAAGGACACCTCAAAGAGCCGA
ATTTATCTGACGCTGAACAGCCTGACAGGCGAGGACACGGCCGTCTATTACTGTGCGAGTAGTTACTATGATTTTGG
TGGTGCTTACTACTTTGGCGACTGGGGCCAGGGTATCCTGGTCACCGT
>HQ403628.1 Equus caballus clone VDJ3 dev-stage foal immunoglobulin heavy chain variable region mRNA, partial cds
CAGGTGCAGCTGCAGGAGTCAGGACCTGGCCTGGTGAAGCCCTCGCAGACCCTCTCCCTCACCTGCACTGTCTCTGG
ATTATCTTTGAGCAGTAATGCTGTAGGCTGGGTCCGCCAGGCTCCAGGAAAAGGGCTGGAATTTGTTGGTGCAATAT
ACGGTAGTGCAAGTGCAAGGTACAACCCAGCCCTGAAGTCCCGAGCCAGCATCACCAAGGACACCTCAAAGAGCCAA
GTCTATCTGACGCTGAACAGCCTGACAAGCGAGGACACGGCCGTCTATTACTGTGCAGGAGTTTATGATGATACTTT
CTACCACTGGGGCCAGGGTATCCTGGTCACCGT
>HQ403627.1 Equus caballus clone VDJ2 dev-stage foal immunoglobulin heavy chain variable region mRNA, partial cds
CAGGTGCAGCTGAAGGAGTCGGGACCTGGCCTGGTGAAGCCCTCGCAGACCCTGTCCCTCACCTGCACTGTCTCTGG
ATTATCTTTGAGCAGTAATACTGTAGGCTGGGTCCGCCAGGCTCCAGGAAAAGGGCTGGAGTGGGTTGGTAGTCTTA
TATATGGTAGTGAAAGTACATACTACAACCCAGCCCTGAAGTCCCGAGCCAGCATCACCAAGGACACCTCAAAGAGC
CAAGCTTATCTGACGCTGAACAGCCTGACAGGCGAAGACACGGCCGTCTATTACTGTGCAGGATGGGGAGATAGTTA
TGGTACTTACTATGGAGGGGATTCTCATTGTATAAACTACTGGGGCCAGGGTATCCTGGTCACCGT

>HQ403626.1 Equus caballus clone VDJ1 dev-stage foal immunoglobulin heavy chain variable region mRNA, partial cds
CAGGTGCAGCTGAAGGAGTCGGGACCTGGCCTGGTGAAGCCCTCGCAGACCCTCTCCCTCACCTGCACTGTCTCTGG
ATTATCTTTGAGCAGTAATGCTGTAGGCTGGGTCCGCCAGGCTCCAGGAAAAGGGCTGGAATACGTTGGTATTATAT
ATGCCAGTGCAAGTGAAAACTACAACCCAGCCCTGAAGTCCCGAGCCAGCATCACCAAGGACACCTCAAAGAGCCAA
GTTTATCTGACGCTGAACAGCCTGACAAGCGAGGACACGGCCGTCTATTACTGTGCAGGAGTCGTCGATGACTATGC
AGATACTTTCTACTATACGGCGTATTTTGGCTACTGGGGCCAGGGTATCCTGGTCACCGT

>HQ403625.1 Equus caballus clone VDJ9 dev-stage neonate immunoglobulin heavy chain variable region mRNA, partial cds
CAGGTGCAACTGCAGGAGTCGGGACCTGGCCTGGTGAAGCCCTCGCAGACCCTGTCCCTCACCTGCACTGTCTCTGG
ATTATCTTTGAGCAGTAATGTTGTAGGCTGGGTCCGCCAGGCTCCAGGAAAAGGGCTGGAGTGGGTTGGTAAAATAT
ATGGTAGTGGAAGTGCAAACTACAACCCAGCCCTGAAGTCCCGAGCCAGCATCACCAAGGACACCTCAAAGAGCCAA
GTTTATCTGACGCTGAACAGCCTGACAAGCGAGGACACGGCCGTCTATTACTGTGCAGGTCATGGTTACTATGGTGG
TTACTGGACTAGTTATTATGATTATGGTGTAGACTACTGGGGCCAGGGTATCCTGGTCACCGT

>HQ403624.1 Equus caballus clone VDJ8 dev-stage neonate immunoglobulin heavy chain variable region mRNA, partial cds
CAGGTGCAGCTGCAGGAGTCGGGACCTGGCCTGGTGAAGCCCTCGCAGACCCTGTCCCTCACCTGCACTGTCTCTGG
ATTATCTTTGAGCAGTAATGCTGTAAACTGGGTCCGCCAGGCTCCAGGAAAAGGGCTGGAGTGGGTTGGTGTTATAG
TTGGTAGTGAAAGTACATACTACAACCCAGCCCTGAAGTCCCGAGCCAGCATCACCAAGGACACCTCAAAGAGCCAA
GTTTATCTGACGCTGAACAGCCTGACAGGCGAAGACACGGCCGTCTATTACTGTGCAGGATTTGATGGTGGTAGTTC
CTGGTACTCGCAACTATATTATTATGCTATAAACTACTGGGGCCAGGGTATCCTGGTCACCGT

>HQ403623.1 Equus caballus clone VDJ7 dev-stage neonate immunoglobulin heavy chain variable region mRNA, partial cds
CAGGTGCAGCTGCAGGAGTCGGGACCTGGCCTGGTGAAGCCCTCGCAGACCCTCTCCCTCACCTGCACTGTCTCTGG
ATTGTCTTTGAGCAGTAATGTTGTGGGCTGGGTCCGCCAGGCTCCAGGAAAAGGGCTGGAATACATTGGCTCTATAT
ATGGTAGTGCAAGTCCGTGGTACAACCCAGCCCTGAAGTCCCGAGCCAGCATCACCAAGGACACCTCAAAGAGCCAA
GTTTATCTGACGCTGAACAGCCTGACAAGCGAGGACACGGCCGTCTATTACTGTTCAGGAGGCATTGGTTCCTATGC
TGGAATTTACAAATACTGTAGTATAGCGTACTGGGGCCAGGGTATCCTGGTCACCGT

>HQ403621.1 Equus caballus clone VDJ5 dev-stage neonate immunoglobulin heavy chain variable region mRNA, partial cds
CAGGTGCAGCTGCAGGAGTCGGGACCTGGCCTGGTGAAGCCCTCGCAGACCCTGTCCCTCACCTGCACTGTCTCTGG
ATTATCTTTGAGCAGTAATGCTGTAGGCTGGGTCCGCCAGGCTCCCGGAAAAGGGCTGGAGTGGGTTGGCGCTATAT
ATGGTAGTGAAAGTCCATACTACAACACAGCCCTGAAGTCCCGAGCCAACATCACCAAGGACACCTCAAAGAGCCAA
GTTTATCTGACGCTGAACAGCCTGACAGGCGAAGACACGGCCGTCTATTACTGTGCAGGATATTCCGGTAGTTCCTG
GTTTTCTTATGGCTACGTGGATCACTGGGGCCAGGGTATCCTGGTCACCGT

>HQ403620.1 Equus caballus clone VDJ4 dev-stage neonate immunoglobulin heavy chain variable region mRNA, partial cds
CAGGTGCAGCTGCAGGAGTCGGGACCTGGCCTGGTGAAGCCCTCGCAGACCCTGTCCCTCACCTGCACTGTCTCTGG
ATTATCTTTGAGCAGTTATGCTGTAGGCTGGGTCCGCCAGGCTCCAGGAAAAGGGCTGGAGTGGGTTGGTGTTATAA
GCGGTAGTACATACTACAACCCAGCCCTGAAGTCCCGAGCCAGCATCACCAAGGACACCTCAAAGAGCCAAGTTTAT
CTGACGCTGAACAGCCTGACAGGCGAAGACACGGCCGTCTATTACTGTGCAGGATACTATAGCAGCTATCTATACTA
CACCGATAATGGTATAAAGTACTGGGGCCAGGGTATCCTGGTCACCGT

>HQ403619.1 Equus caballus clone VDJ3 dev-stage neonate immunoglobulin heavy chain variable region mRNA, partial cds
CAGGTGCAGCTGAAGGAGTCGGGACCTGGCCTGGTGAAGCCCTCGCAGACCCTCTCCCTCACCTGCACTGTCTCTGG
ATTATCTTTGAGCAGTTATGCTGTAGGCTGGGTCCGCCAGGCTCCAGGAAAAGGGCTGGAATATGTTGGTGCTATAT
ATGGTAGTGCAAGTGCAAACTACAACCCAGCCCTGAAGTCCCGAGCCAGCATCACCAAGGACACCTCAAAGAGCCAA
GTTTATCTGACGCTGAACAGCCTGACAGGCGAGGACACGGCCGTCTATTACTGTGCGAGAGGAAGAATTACTATAGT
AGGAGTTGCTACATTTAGCTACTGGGGCCAGGGTATCCTGGTCACCGT

>HQ403618.1 Equus caballus clone VDJ2 dev-stage neonate immunoglobulin heavy chain variable region mRNA, partial cds
CAGGTGCAGCTGAAGGAGTCAGGACCTGGCCTGGTGAAGCCCTCGCAGACCCTGTCCCTCACCTGCACTGTCTCTGG
ATTCTCTTTGAGCAGTTACGGTGTAGGCTGGGTCCGCCAGGCTCCAGGAAAAGGGCTGGAATTTGTTGGTGATATAG
CTAGTAGTGGAAGTGCAAACTACAACCCAGCCCTGAAGTCCCGAGCCAGCATCACCAAGGACACCTCAAAGAGCCAA
GTTTATCTGACGCTGAACAGCCTGACAGGCGAAGACACGGCCGTCTATTACTGTGCAGGATGGGAAGACGATGACGG
ATACTATTATGGTATAAACTACTGGGGCCAGGGTATCCTGGTCACCGT

```
>HQ403615.1 Equus caballus clone VDJ8 dev-stage fetus immunoglobulin heavy
chain variable region mRNA, partial cds
CAGGTGCAGCTGCAGGAGTCGGGACCTGGCCTGGTGAAGCCCTCGCAGACCCTGTCCCTCACCTGCACTGTCTCTGG
ATTCTCTTTGAGCAGTTACGGTGTAGGCTGGGTCCGCCAGGCTCCAGGAAAAGGGCTGGAATTTGTTGGTGGTATAG
CTAGTAGTGGAAGTGCAAACTACAACCCAGCCCTGAAGTCCCGAGCCAGCATCACCAAGGACACCTCAAAGAGCCAA
GTTTATCTGACGCTGAACAGCCTGACAGGCGAGGACACGGCCGTCTATTACTGTGCGATCTATGGTTATGGTGGTGC
TTACTATTTTTATTATAATGCTATGGACCCCTGGGGCCAGGGTATCCTGGTCACCGT
>HQ403613.1 Equus caballus clone VDJ6 dev-stage fetus immunoglobulin heavy
chain variable region mRNA, partial cds
CAGGTGCAGCTGCAGGAGTCGGGACCTGGCCTGGTGAAGCCCTCGCAGACCCTGTCCCTCACCTGCACTGTCTCTGG
ATTCTCTTTGAGCAGTTACGGTGTAGGCTGGGTCCGCCAGGCTCCAGGAAAAGGGCTGGAATATGTTGGTGGTATAG
CTAGTAGTGGAAGTGCAAACTACAACCCAGCCCTGAAGTCCCGAGCCAGCATCACCAAGGACACCTCAAAGAGCCAA
GTTTATCTGACGCTGAACAGCCTGACAGGCGAGGACACGGCCGTCTATTACTGTGCGATAGCCTACGGTTATGGTTA
TGCTTTTGGCTACTGGGGCCAGGGTATCCTGGTCACCGT
>HQ403611.1 Equus caballus clone VDJ4 dev-stage fetus immunoglobulin heavy
chain variable region mRNA, partial cds
CAGGTGCAGCTGCAGGAGTCGGGACCTGGCCTGGTGAAGCCCTCGCAGACCCTGTCCCTCACCTGCACTGTCTCTGG
ATTATCTTTGAGCAGTAATGCTGTAGGCTGGGTCCGCCAGGCTCCAGGAAAAGGGCTGGAGTGGGTTGGTGTTATAT
ATGGTAGTGAAAGTACATACTACAACCCAGCCCTGAAGTCCCGAGCCAGCATCACCAAGGACACCTCAAAGAGCCAA
GTTTATCTGACGCTGAACAGCCTGACAGGCGAAGACACGGCCGTCTATTACTGTGCAGGATGGGGTTACTATGGTGG
TAGTTCCTGGTACTCCGGTCGGTATTATGGTATAAACTACTGGGGCCAGGGTATCCTGGTCACCGT
>DQ125458.1 Equus caballus clone 4-15 immunoglobulin heavy chain variable
region mRNA, partial cds
CAGGTGCAACTGCAGGAGTCGGGCCCAGGACAGGTGAAGCCCTCACAGACCCTCTCCCTCACCTGCACTGTCACTGG
AGGATCCATCACAAACAAGTATTCTAGCTGGACCTGGTTACGCCAGCCTCCAGGGAAGGGCCTGGAATTTATCGGAT
ACATATATTATGATGGTAGACGTTACTACAATCCTTCCTTCAAGAGCCGCACCTCCATCTCCAGAGACACCTCCAGG
AACGAGTTCTCCCTGCAGCTGAGCTCCGTGACCGATGAGGACGCGGCCGTATATTTTTGTGCAGGGGATTATGGTTA
TGGCGGTGTTTGGTACTCAGATGGTGAAAACTACTGGGGCCAGGGTATCCTGGTCACCGTCTCG
>DQ125457.1 Equus caballus clone 4-14 immunoglobulin heavy chain variable
region mRNA, partial cds
CAGGTGCAGCTGAAGGAGTCAGGACCTGGCCTGGTGAAGCCCTCGCAGACCCTCTCCCTCACCTGCGCTGTCTCTGG
ATTACCTTTGAGAGATGCTGCCGTAGGCTGGGTCCGCCAGGCTCCAGGAAAGGGTCTGGAATATATTGGTTCTATGT
ATAATGAAGAAGACTACAATCCAGACCTGAAGTCCCGAGCCAGCGTCACCAAGGACACCTCAAAGAGCCGAGTCACT
CTGACGCTGAACAGTCTGACAAGTGAGGACACGGCCGTCTATTACTGTGTAGGGGACGGTGGCTCTGGATACTACTG
GGGCCAGGGTATCCTGGTCACCGTCTCG
>DQ125455.1 Equus caballus clone 4-11 immunoglobulin heavy chain variable
region mRNA, partial cds
CAGGTGCAGCTGCAGGAGTCAGGACCTGGCCTGGTGAAGCCCTCGCAGACCCTCTCCCTCACCTGTACTGTCTCTGG
ATTATCACTGAGCAGTAATGTGTTAGGCTGGGTCCGCCAGGCTCCCGGAAAAGGGCTGGAATGGATTGGTGGAATAT
ATGGAAGTGCAAGTCCAAACTATAATCTAACCCTGAAGGCCCGAGGCAGCATCACCAAGGACACCTCAAAGAGCCAA
GTGTATCTGACGCTAACTGGGATGACAGAGGAGGACACGGCCGTCTATTACTGTGCAGGAGGGGCTCCCTATAATTA
TGCCGGTGGTAACATTGGAAGAATGAAGTATTGGGGCCAGGGTATCCTGGTCACCGTCTCG
>DQ125454.1 Equus caballus clone 4-10 immunoglobulin heavy chain variable
region mRNA, partial cds
CAGGTGCAACTGAAGGAGTCGGGACCTGGCCTGGTGAAGCCCTCGCAGACCCTGTCCCTCACCTGCACTGTCTCTGG
ATTCCCTTTGAGCAGTTACGGTGTAGGCTGGGTCCGCCAGGCTCCAGGAAAAGGGCTGGAATCGGTTGGTGAAATAG
CTAGTAGTGGAAGTGCAAACTACAACCCAGCCCTGAAGTCCCGAGCCAGCATCACCAAGGACACCTCAAAGAGCCAA
GTTTATCTGACGCTGAACAGCCTGACAAGCGAGGACACGGCCGTCTATTATTGTACAGGATGGGGACTGAGACTGTA
CTACTGGGGCCAGGGTATCCTGGTCACCGTCTCG
>DQ125453.1 Equus caballus clone 4-9 immunoglobulin heavy chain variable
region mRNA, partial cds
CAGGTGCAACTGCAGGAGTCAGGACCTGGCCTGGTGAAGCCCTCGCAGACCCTCTCCCTCACCTGCACTGTCTCTGG
ATTATCTTTGAGCAGTAATGCTGTAGGCTGGGTCCGCCAGGCTCCAGGAAAAGGGCTGGAATATGTTGATAGTATAG
GCAACAGTGAAAGTGCAAACTTTAACCCAGCCCTGAAGTCCCGAGCCAGCATCACCGAGGACACCTCAAAGAGCCGA
```

GTTTATCTGACGCTGAACAGCCTGACAAGCGAGGACACGGCCGTCTATTACTGTGCAGCCCAATATGATTACTTTGC
TGGTGCTTATGGCCTCATCCCTTATGCTATAAAGTACTGGGGCCAGGGTATCCTGGTCACCGTCTCG
>DQ125452.1 Equus caballus clone 4-8 immunoglobulin heavy chain variable region mRNA, partial cds
CAGGTGCAACTGAAGGAGTCAGGACCTGGCCTGGTGAAGCCCTCGCAGACCCTCTCCCTCACCTGCACTGTCTCTGC
GTTATCTTTGAGCAGTGCTGGTGTGGGCTGGGTCCGCCAGGCTCCAGGAAAAGGGCTGGAATTTGTTGCTGGTATAG
TTGGTGATGGTGGTACGTACGCCAACCCAGCCCTGAGGTCCCGAGCCAGCATCACCAAGGACACCTCAAAGAGCCAA
GTTTATCTGACGCTGAACATGCTGACAAGCGAGGACACGGCCGTCTATTACTGTGCAGGAAGCTTGGAGTTTAGTGG
CTGGGGAGTTATGCGCTACGGTATAAACTACTGGGGCCAGGGTATCCTGGTCACCGTCTCG
>DQ125451.1 Equus caballus clone 4-7 immunoglobulin heavy chain variable region mRNA, partial cds
CAGGTGCAGCTGAAGGAGTCAGGACCTGGCCTGGTGAAGCCCTCGCAGACCCTGTCCCTCACCTGCACTGTCTCTGG
GAACGATTTGAGAAGTTTTGGCGTAGCCTGGGTCCGCCAGGCTCCAGGAAAAGGCCTGGAATTTGTTGGTGGTGTAG
CCAGGTTTGGCAGCCCTTACTACAACCCAGCCCTGAAGTCCCGGGCCATCATCACCAAGGACACCTCAAAGAAGGAA
AGTGTGCTGACGTTAAATAGCGTGACAGGCGAGGACACGGCCGTCTATTGGTGTGCAGGGGGATATGGTGATGAATC
CTGGGGACCCTGGGGCCAGGGTATCCTGGTCACCGTCTCG
>DQ125450.1 Equus caballus clone 4-6 immunoglobulin heavy chain variable region mRNA, partial cds
CAGGTGCAACTGCAGGAGTCAGGACCTGGCCTGGTGAAGCCCTCGCAGACCCTCTCCCTCACCTGCACTGTCTCTGG
AATCTCTTTGACCGATTACAATGTAGACTGGGTCCGCCAGGCTCCAGGAAAAGGGCTGGAATTTGTTGGTGGACTAT
GGACTAATGGACAATCGAACTACAATCCAGCCCTGAAGTCCCGAGCCAGAATCACCAAGGACACCTCAAAGAGTCAA
GTTTATCTGACGCTGAACAGCCTGACAAGCGAGGACACGGCCGTCTATTACTGTGAGGGTTATGGTAATTCCTGGCA
GCCACCGCACTACTGGGGCCAGGGTATCCTGGTCACCGTCTCG
>DQ125449.1 Equus caballus clone 4-5 immunoglobulin heavy chain variable region mRNA, partial cds
CAGGTGCAACTGAAGGAGTCAGGACCTGGCCTGGTGAAGCCCTCGCAGACCCTTTCCCTCACCTGCACCGTCTCTGG
AATGTCTTTGAGCACCAACACTGTAGGCTGGGTCCGCCAGGCTCCAGGAAAAGGGCTGGAATACGTTGGTCTAATCT
ATGGTATGAAAAGTGCAGAGTACAATCCAGCCCTGAAGTCCCGAGCCAGTATCACCAAGGACACCTCAAATAGTCAA
GTTCTTCTGACGCTGAATAGCCTGACAAGCGAGGACACGGCCGTCTACTACTGTGCAGGGGGTGAAGCCTGGGGTCC
AATGTATAGTTCGAACGAAGAAAAAAATGGTGTGGAATACTGGGGCCAGGGTATCCTGGTCACCGTCTCG
>DQ125447.1 Equus caballus clone 4-3 immunoglobulin heavy chain variable region mRNA, partial cds
CAGGTGCAGCTGAAGGAGTCGGGACCTGGCCTGGTGAAGCCCTCGCAGACCCTCTCCCTCACCTGCACTGTCTCTGG
ATTCTCTTTGAGCACCACCGGTGTGGGCTGGGTCCGCCAGGCTCCAGGAAAAGGGCTGGAATTTGTTGGTGGTGTAC
CTAGTAGTGGAAGTGCAAACTACAATCCAGCCCTGAAGTCCCGATGCAGCATCACCAAGGACGAATCAAAGAGCCAA
GTTTATCTGACGCTGAACAGCCTGACAAGCGAGGACACGGCCGTCTATATATGTGCAGGAGGCTTCTACAATACATT
GGATAAGGGGATAAACTATTGGGGCCAGGGTATCCTGGTCACCGTCTCG
>DQ125446.1 Equus caballus clone 4-2 immunoglobulin heavy chain variable region mRNA, partial cds
CAGGTGCAACTGAAGGAGTCAGGACCTGGCCTGGTGAAGCCCTCGCAGACTCTCTCCCTCACCTGCGCTGTCTCTGG
ATTCTCTTTGAGAGATGCCGCCATGGGCTGGGTCCGCCAGGCTCCAGGAAGGGGTCTGGAATACATCGGTTCTATGT
ATATTAGAGAAGACTACAATCCAGCCCTGAAGTCCCGAGCCAGCGTCACCAAGGACACAAAGGAGAGCCGAAGTTAT
CTGACACTGAACGCGCTGACAAGTGAGGACACGGCCGTCTATTGGTGTGTAGGGGATGTTGGCACTGGATACTACTG
GGGCCAGGGTATCCTGGTCACCGTCTCG
>DQ125443.1 Equus caballus clone 3-13 immunoglobulin heavy chain variable region mRNA, partial cds
CAGGTGCAGCTGAAGGAGTCAGGACCTGGCCTGGTGAAGCCCGGGCAGACCCTCTCCCTCTCCTGCACTGTCTCTGG
ATTGTCATTGAGCACAAATACTGTAGGTTGGGTCCGCCAGGCTCCAGGAAAAGGATGGGAATATGTTGCTGCGTTAT
ACGCGGATGCAGATGGAGATTATAATCCAGTCCTTCAGTCCCGAGCCAGCATCACGAAGGACACCTCCAAGAACCAG
GTCTTTCTGACGCTAGACACACTGACGAGCGAGGACACGGCCGTCTATTACTGCACAGGGGGAGTCTTCTCCGTCCC
CGTCGGTACTGGATATACTTACTATGAATCGGGAATACTATACTGGGGCCAGGGTATCCTGGTCACCGTCTCG
>DQ125442.1 Equus caballus clone 3-12 immunoglobulin heavy chain variable region mRNA, partial cds
CAGGTGCAGCTGAAGGAGTCAGGACCTGGCCTGGTGAAGCCCTCGCAGACCCTCTCCCTCGTCTGCACTGTCTCTGG
ATTCAGTTTGAACAGTTGGGGTGTAGGCTGGGTCCGCCAGGCTCCAGGAAAAGGGCTGGAGGAAGTTGGTGGAAGTC
AGATTGGTGGGAATGCAAACTACAATCCAGCCCTGGAGTCCCGAGCCAGCATCACCAAGGACGCCTCAAAGAGCCAA

GTTTATCTGACGCTGAACAGCCTGACAGAAGAGGACACGGCCGTCTATTACTGTACAGGAGGTTACAACTGGAATCT
TGGGACTAATAGGGACCGTATAACGTACTGGGGCCAGGGTATCCTGGTCACCGTCTCG
>DQ125441.1 Equus caballus clone 3-9 immunoglobulin heavy chain variable region mRNA, partial cds
CAGGTGCAACTGAAGGAGTCGGGACCTGGCCTGGTGAAGCCCTCGCAGGCCCTGTCCCTCACCTGTACTATCTCTGG
GTTCTCTTTGACCAGTCACGGTGTAGGCTGGGTCCGCCAGGCTCCAGGAAAAGGGCTGGAATTTGTCGGTAGTATAT
GGACTACGGGACAGACAATCAACAATCCAACCCTGAAGTCCCGAGTCAGCATCACTAGGGACACCGGGCTGAACCAA
GTTTCACTGACGTTGAATGAGTTGACAAGCGAGGACACGGCCGTCTATTATTGTGCAGGAGGCGCGATTTCCGATTA
CGACTTTTTCGGTTTTCGTGGTATGTTCAGCATTTATGATGTGCAGTATTGGGGCCAGGGTATCCTGGTCACCGTCT
CG
>DQ125440.1 Equus caballus clone 3-8 immunoglobulin heavy chain variable region mRNA, partial cds
CAGGTGCAGCTGAAGGAGTCAGGGCCTGGCCTGGTGAAGCCCGCGCAGACCCTTACCCTTACCTGCACTGTCTCTGG
ATTACACTTGAACAGTGACGCGGTAGTGGGCTGGGTCCGTCAGGCTCCAGGAAAGGGGCTGGAATTTGTTGGTGGAT
TGTCTAATACAGGACGTGCAAACTACAATCCAGCCCTGAAGTCCCGAGCCATCATCACCAAGGACACCTCAAAGAGC
CAGGTTTATCTGACCCTGAACAGCCTGACAAGCGAGGACACGGCCGACTATTTTTGTGCAGGAGGTAGAATGTTCGA
TTATGTTTATGGCGGCTATTACGAAATACAATATTGGGGCCAGGGTATCCTGGTCACCGTCTCG
>DQ125439.1 Equus caballus clone 3-7 immunoglobulin heavy chain variable region mRNA, partial cds
CAGGTGCAGCTGAAGGAGTCGGGACCTGGCCTGGTGAAGCCCTCGCAGACCCTCTCCCTCACCTGCACTGTCTCTGG
ATTATCTTTGAGCAGTAGTTGTGTACAATGGGTCCGCCAGGTTCCAGGAAAAGGGCTGGAATACGTCGGTAGGATAG
TTAGTAGTGGTGGTGGTCTAACCTACAACCCGGCCCTGAAGTCCCGAGCCAGCATCACCAGAGACACTTCAAAGAGC
CAGGTTTATCTGACGCTGAACAGCCTGACAGACGAGGACACGGCCGTCTATTACTGTACAGGGGCCCTGAATACTCA
CTACAGTTCATACGCGGGTTATGGTATAGACTACTGGGGCCAGGGTATCCTGGTCACCGTCTCG
>DQ125438.1 Equus caballus clone 3-6 immunoglobulin heavy chain variable region mRNA, partial cds
CAGGTGCAACTGCAGGAGTCAGGTCCTGGCCTGGTGAGGCCAGCAGAGACCCTCTCCCTCACCTGCACTGTCTCTGG
ATTGGACTTGAGCAGTGGTACGATAATCTGGGTCCGCCAGGCTCCAGGAAAAGGGCTGGAGAGAGTCGGTGAAATAG
TTGGTGAGGGAAGTGGATTCTACAATCCAGCCCTGAAGTCCCGAGCCATGATCACCAAGGACACCTCGAAGAATGAG
ATTTATCTGACACTGAAGAGCCTGACAAGCGAGGACACGGCCGTCTATTACTGTGCAGGAGCCTGGGGCGGAAATTA
CTACGAAAATTTTTTTATTAATGGTGTAGAGAATTGGGGCCAGGGTATCCTGGTCACCGTCTCG
>DQ125437.1 Equus caballus clone 3-5 immunoglobulin heavy chain variable region mRNA, partial cds
CAGGTGCAACTGAAGGAGTCAGGACCTGGCCTGGTGAAGCCCTCGCAGACCCTCTCCCTCACCTGCGCTGTCTCTGG
ATTCTCTTTGAGCAGTGACGGTATAAACTGGGTCCGCCAGGCTCCAGGAAAAGGGCTGGAATTCGTGGGTTCTATAT
ATACTAGTGCAAGTACAATCTACAACCCAGCCCTGAAGTCCCGAGCCAGCATCACCAAGGACACCTCAAAGAGCCAA
GTTTATCTGACGCTGAACAGCCTGACAAGTGAGGACACGGCCGTCTATTACTGTTCAGGAGGCAGTGAAGAATATTG
GGGCCAGGGTATCCTGGTCACCGTCTCG
>DQ125436.1 Equus caballus clone 3-4 immunoglobulin heavy chain variable region mRNA, partial cds
CAGGTGCAGCTGAAGGAGTCAGGACCTGGCCTGGTGAAGCCCTCGCAGACCCTCTCCCTCACCTGCACTGTCTCTGG
ATTATCTTTGAGAGGTAATGTTGTAGGCTGGGTCCGCCAGGCTCCAGGAAAAGGGCTGGAACACGTTGGCGAAAACG
TTAGTAGTGGAGGTGCGTTCTACAGCCCAGCCCTAAAGTCCCGAGCCAGCATCACCAGGGACACCTCAAAGAGCCAA
ATTTATCTGACGCTGAACAGCCTGACAAGGGAGGACACGGCCGTCTATTACTGTGCAGCATGGAAGGTTAGCAGTCG
CTCTTACTTGGATGGTATAAACTACTGGGGCCAGGGTATCCTGGTCACCGTCTCG
>DQ125435.1 Equus caballus clone 3-3 immunoglobulin heavy chain variable region mRNA, partial cds
CAGGTGCAACTGAAGGAGTCGGGACCGGGCCTGGTGAAGCCCTCGCAGACCCTCTCCCTCACCTGCACTGTCTCTGG
ATTAAGTTTGAGCAGTTATGGTGCAGGCTGGGTCCGCCAGTCTCCAGGAAAAGGGCTGGAATATGTTGGTGGGGTGG
GTAAAAGTGGAAGTTCAAATTACAATTCAGCCCTGAAGCCCCGAGCCAGTATCACCAAGGACTCCTCAAAGAGTCAG
ATTTCTCTGACGCTGAGAAGCCTGACAGGCGAGGACACGGCCGTCTATTACTGTGCGATCTACGATAGTTATCTTCG
TGGTTGGTCAGTTGTCTACTGGGGCCAGGGTATCCTGGTCACCGTCTCG
>DQ125434.1 Equus caballus clone 3-2 immunoglobulin heavy chain variable region mRNA, partial cds

CAGGTGCAACTGAAGGAGTCCGGACCTGACCTGGTGAAGCCCTCGGAGACCCTCTCCCTCGTCTGCTCCGTCTCTGG
ACAATCTTTGAGCAGTTATGATGTGGGCTGGGTTCGCCAGGCTCCAGGCTGGGGACTGGAATTCGTTGGTGTAACGG
CGCATTATGGAGGTATAGACTACAATCCAGCCCTGAAGTCCCGAGCCAGCATCACCAAGGACACCTCAAAGAACCAA
CTTACTCTGATACTGAATAGTCTGACAAGCGAGGACACGGCCGTCTATTACTGTACAGGAGAAGCGCAGACTAATTG
TGACTTTGGCGTCAGTTGTTTGGGCTACTGGGGCCAGGGTATCCTGGTCACCGTCTCG
>DQ125433.1 Equus caballus clone 3-1 immunoglobulin heavy chain variable region mRNA, partial cds
CAGGTGCAACTGAAGGAGTCAGGACCTGGCCTGGTGAAGCCCTCGCAGACCCTCTCCCTCATCTGCACTGTCTCTGG
ATTCTCTTTGAGCAGTGACAGTGTAGGCTGGGTCCGCCAGGCTCCAGGAAAAGGGCTGGAATTTGTTGGAGTGGTAC
ATAGTAGTGGAAGGGCAAGAAACCCAGCCCTGAAGTCCCGAGCCAGCATCACCAAGGACACCTCAGAGAGCCAAGTT
TATCTGACGCTGAACAGCCTGACAAGCGAGGACACGGCCGTCTATTACTGTGCAGGGGGGCGTAGTGGCTACAGTTA
TTACGCTGGGATGGTAGATGGTATAAACTACTGGGGCCAGGGTATCCTGGTCACCGTCTCG
>DQ125432.1 Equus caballus clone 2-15 immunoglobulin heavy chain variable region mRNA, partial cds
CAGGTGCAGCTGCAGGAGTCAGGACCTGGCCTGGTGAAGCCCTCGCAGACCCTCTCCCTCACCTGCAGTGTCTCTGG
ATTGTCTTTGAGCAGTGTTTTTGTATACTGGGTCCGCCAGGCTCCAGGAAAAGGGCTGGAATATGTTGGTTTTATAG
GTAATAGTGGAAGTACAATAGGTAATAGTGGAAAAACAAACTACAACTACAACCCAGTCCTGAAGTCCCGAGCCAGC
ATCAGCAAGGACACCTCAAAGAGCCAAGTTCTTCTGACGCTGAACAGCCTGACAAGCGAGGACACGGCCGTCTATTA
CTGTGCAGGAGACAATATAAAGTATTGGGGCCAGGGTATCCTGGTCACCGTCTCG
>DQ125431.1 Equus caballus clone 2-14 immunoglobulin heavy chain variable region mRNA, partial cds
CAGGTGCAACTGAAGGAGTCAGGACCTGGCCTGGTGAAGCCCTCGCAGACCCTCTCCCTCACCTGCACTGTCTCTGG
ATTATCTTTGAGCAGTTATGGTGTGGACTGGGTCCGCCAGGCTCCAGGAAAAGGACTTGAATGGGTTGGTGGTATAA
CTAGTAGTGGAGGTTCAGGTTACAACCCAGCCCTGAAGTCCCGAGCCAGCATCACCAAGGACACCTCAAAGAGCCAA
GTTTATCTGACGCTGAACAGCCTGACAAGCGAGGACACGGCCGTCTATTACTGTGCAGGAGAGGAGGAAGGCTACGT
TTATGGTTTTACTCGTTATTATGGTAACTACTACTGGGGCCAGGGTATCCTGGTCACCGTCTCG
>DQ125430.1 Equus caballus clone 2-11 immunoglobulin heavy chain variable region mRNA, partial cds
CAGGTGCAGCTGAAGGAGTCAGGACCTGACCTGATGAAGCCCTCGCAGACCCTCTCCCTCACCTGCACTGTCTCTGG
ATTCTCTTTGAGCAGTTATGGTGTAGGCTGGGTCCGCCAGGCTCCAGGTAAAGGCCTGGAGTTTGTTGGCGGGTTAC
CTGGTAGTGGAAGTGCAGACTACAGCCCAGCCCTGAGGTCCCGAGCCAGCATCACCAAGGACACCTCAAAGAGCCAA
GTTTATGTGACGCTGAACAGCCTGACAAGCGAGGACACGGCCGTCTATTACTGTGCAAGATTCTATAACTGGAATAG
TGGTGTTGTCAGTTATACTGGTATTGACTACTGGGGCCAGGGTATCCTGGTCACCGTCTCG
>DQ125429.1 Equus caballus clone 2-9 immunoglobulin heavy chain variable region mRNA, partial cds
CAGGTGCAGCTGAAGGAGTCGGGACCTGGCCTAGTGAAGCCCTCGCAGACCCTGTCCCTCGTCTGCACTGTCAGTGG
ATTCTCCCTGACCGACCGGGGTGTAGGCTGGGTCCGCCAGGCGCCAGGAAAAGGACTGGAATTTGTGAGTTATATAC
TAACCAGTGGAGCCCAAGACGGGAATCCAGCCCTAAGGTCCCGAGTCAGCATCACCAGGGACACCTCACTGAGTCAA
GTTTATCTGACAATGAACAGCGTGACAGGCGAGGACACGGCCGTCTACTATTGTGGGAGGCATGGACCGAATCTTCA
TGGAACTTTTGACTATTGGGGCCAGGGTATCCTGGTCACCGTCTCG
>DQ125428.1 Equus caballus clone 2-8 immunoglobulin heavy chain variable region mRNA, partial cds
CAGGTGCAGCTGAAGGAGTCAGGACCTGGCCTGGTGAAGCCCTCGCAGACCCTCTCCCTCACCTGCACTGTCTCTGG
ATTATCTTTGACAGATTATGGTGTGGGCTGGGTCCGCCAGGCTCCAGGAAAAGGGCTGGAATTTGTTGCCAGAATAG
ATAGTGATGGAAGTAAAAACTTTAACCCAGCGCTGAAGTCCCGAGCCAACATCATCAAGGACACCTCAAAGAGCCAA
GTTTATCTGACGCTGAACAGCCTGACAAGTGAAGACACGGCCGTCTATTACTGTGCAGGGTATGGTTACAGTGGTCG
TTACTCCACACCGGGGAATTTATACTGGTGGGGCCAGGGTATCCTGGTCACCGTCTCG
>DQ125427.1 Equus caballus clone 2-7 immunoglobulin heavy chain variable region mRNA, partial cds
CAGGTGCAGCTGAAGGAGTCAGGACCTGGCCTGGTGAAGCCCTCGCAGACCCTCTCCCTCACCTGCACTGTCTCTGG
ATTGTCTTTGACAGGTAGTCAGATAGCTTGGGTCCGCCAGGCTCCAGGAAAAGGACTGGAATATATTAGTGGAAGTT
CAATGTACAACCCAGCCCTGAAGTTCCGAGCCAGCATCACCAAGGACACCTCCAAGAATCAAGTTACTCTGACGCTG
AATAAGCTGACAGGCGAGGACACGGCCGTCTATTACTGTGTGGCGACAGCTTTTTGGGGCGGTTATGGCGGTATCCA
ATACTGGGGCCAGGGTATCCTGGTCACCGTCTCG
>DQ125426.1 Equus caballus clone 2-6 immunoglobulin heavy chain variable region mRNA, partial cds

CAGGTGCAACTGAAGGAGTCAGGACCTGGCCTGGTGAAGCCCTCGCAGACCCTTTCCCTCACCTGCACTGTCTCTGG
ATTACTTTTGAACAGTAATTGTGTAGGTTGGGTCCGCCAGGCTCCAGGAAAACGACTGGAATACGTTGGTTCTATAT
ATGGGACGTTAACAAACTACAACTCAGCCCTGAGGTCCCGAGCCAGAATCACCAGCGACTACTCAAAGAGCCAAGTT
CTTCTGACGCTGAACAGCCTGACAAGCGAGGATACGGCCGTCTATTACTGTGCAGCACTCGATTATGGTGTGACGAT
TAGTCGCGATATAAATGATTGGGGCCAGGGTATCCTGGTCACCGTCTCG
>DQ125425.1 Equus caballus clone 2-5 immunoglobulin heavy chain variable region mRNA, partial cds
CAGGTGCAGCTGAAGGAGTCAGGACCTGGCCTGGTGAAGATCTCGCAGACCCTCTCCCTCACCTGCACTGTGTCTGG
ATTATCTTTGAGCAGTAATGATGTAGGCTGGGTCCGCCAGGCTCCAGGAAAAGGGCTGGAATACGTGGCTCGTATAT
GGGGTGGTGCAAATGAACACTACAACCCAGCCCTGAAGTCGCGAGCCAGCATCACCAAGGACACCTCAAAGAGCCAA
GTTTATCTGACGCTGAACAGCCTGACAAGCGAGGACACGGCCGTCTATTACTGTGGAGGAACACCTGGTTTCTATAA
TAGTGCTTACGAGACGTTTGCCTACTGGGGCCAGGGTATCCTGGTCACCGTCTCG
>DQ125424.1 Equus caballus clone 2-4 immunoglobulin heavy chain variable region mRNA, partial cds
CAGGTGCAACTGAAGGAGTCAGGACCTGGCCTGGTGAAGCCCTCGCAGACCCTCTCCCTCACCTGCACTGTCTCTGG
ATTATCTTTGAGCAGTGTTGATGTAGGCTGGGTCCGCCAGGCTCCAGGAAAAGGACTGGAATACGTTAGTTGGATAG
GTAGAAGTACTAGCTACAAGCCGGCCCTGAAGTCCCGAGCCAGCATCACCAAGGACACCTCAAAGAGCCAAGCTTAT
CTGACGCTGAACAGTCTGACGAGCGAGGACACGGCCGTCTATTACTGTGTAGGAGGTTACGCGGACGGTATAGATTA
CTGGGGCCAGGGTATCCTGGTCACCGTCTCG
>DQ125423.1 Equus caballus clone 2-3 immunoglobulin heavy chain variable region mRNA, partial cds
CAGGTGCAGCTGAAGGAGTCAGGACCTGGCCTGGTGAAGCCCTCGCAGACCCTCTCCCTCACCTGCACTATCTCTGG
ATTATCTTTGAGCAGCTATGGTGTGGGCTGGGTCCGCCAGGCTCCAGGAAAAGGGCTGGAATTCGTTGGTGGAATAC
GTAGTAGTGGAAGTGCAAACTACAATCCAGCCCTGAAGTCCCGAGCCAGCATCACCAAGGACACCTCACAGAGCCAT
GTTTATCTGACGCTGAACAGCCTGACAAGCGAGGACACGGCCGTCTATTACTGTGCAGGAGGGACAGAACAACGTGA
TTATATTGACGTTGGTGTGAAGTTCTGGGGCCAGGGTATCCTGGTCACCGTCTCG
>DQ125422.1 Equus caballus clone 2-1 immunoglobulin heavy chain variable region mRNA, partial cds
CAGGTGCAACTGAAGGAGTCGGGACCTGGCCTGGTGAAGCCCTCGCAGACCCTCAGCCTGACATGCAGTGTCTCTGG
ATTGAATTTGAACGAAGATATTGTAGGGTGGGTCCGCCAGGCTCCAGGAAAAGGGCCGGAATACGTCGGAAGTATAT
GGGGAGATAGAAGCCCAAAATACAATCCAGACGTGAAGTCCCGAGCCAGTATCAGTAAGGACACCTCGAAACGCCAG
GTTTATCTTCAACTGAACAGCCTGAGTGACGAGGACACGGCCGTCTATTACTGTGCAGGAGGACTTACAATTTTAGG
CGTCATGAAGGATGAGACGTTCGTGGATCACTGGGGCCCGGGTATCCTGGTCACCGTCTCG
>DQ125421.1 Equus caballus clone 1-14 immunoglobulin heavy chain variable region mRNA, partial cds
CAGGTGCAGCTGAAGGAGTCAGGACCTGGCCTGGTGAAGCCCTCGCAGACCCTCTCCCTCACCTGCACTGTCTCTGG
ATTATCTTTGAGCAGTAATACTGTAGGGTGGGTCCGCCAGGCTCCAGGAAAAGGGCTGGAGTACGTCGGTATTATCT
ATGGTAGTGCAAGTACATTGTACAACCCAGCCCTGAAGTCCCGAGCCAGCATCACCAAGGAATCCTCAAAGAGCCAA
GTTTATTTGACGCTGAACAGCCTGACAAGCGAGGACACGGCCGTCTATTATTGTGCAGGAGGCTTTAGCGGCTTTGA
TTGGTTCGATAGAGGTATAAACTACTGGGGCCAGGGTATCCTGGTCACCGTCTCG
>DQ125420.1 Equus caballus clone 1-13 immunoglobulin heavy chain variable region mRNA, partial cds
CAGGTGCAACTGCAGGAGTCAGGACCTGGCCTGGTGATGCCCTCGCAGACCCTCTCCCTCACCTGCACTGTCTCTGG
ATTATCTCTGGTGAACAGGAATGCTGTGCGCTGGGTCCGCCAGGCTCCGGGAAAAGGGCTGGAATACGTTGGTTCAA
TATACGGTGTTGAAGAACGAAACTACAACCCAGTCCTGAAGTCCCGAGTCGATATCACCAAGGACACCTCAAAGAGT
CAAGTTTATCTGACGCTGAATAGCGTGACAAGCGGGGACACGGCCGTCTATTACTGTGCGAGAAATGAATATGGTAT
TGTGGAATGGGGCCAGGGTATCCTGGTCACCGTCTCG
>DQ125419.1 Equus caballus clone 1-11 immunoglobulin heavy chain variable region mRNA, partial cds
CAGGTGCAACTGAAGGAGTCAGGACCTGGCCTGGTGAAGCCCTCGCAGACCCTGTCCCTCACTTGCACTGTCTCTGG
ATTCTCTTTGATCACTGACAGTGTAGGCTGGGTCCGCCAGGCTCCAGGGAAAGGGCTGGAATTTGTTGGTGGACTTT
CTAGTTTTGGAAGTGCAAATTACAACCCAGGCCTGAACTCCCGAGCCAGCATCACCAAGGACACCTCAAAGGGCCAA
GTCGTTCTGACGCTGAACAGCCTGACAAGCGACGACACGGCCGTCTATTACTGTGTGTCATTTTCGGGCCAGGGTGA
AGCGTTCGCTTTCGCTTACCTTTATTATGGAATAACCTACTGGGGCCAGGGTATCCTGGTCACCGTCTCG

>DQ125418.1 Equus caballus clone 1-10 immunoglobulin heavy chain variable region mRNA, partial cds
CAGGTGCAACTGAAGGAGTCAGGACCTGGCCTGGTGAAGTCCTCGCAGACCCTCTCCCTCACCTGTACTGTCTCTGG
GGCGTCCTTGAACGACATTGCTGTGGGTTGGGTCCGCCAGGCTCCAGGAAAAGGACTGGAATACGTTGGTTGTGTTT
ATGATGGTACCGGAGAAAACTATAACCCAGCCCTGAAGTCCCGAGCCAGCATCACCAGGGACACCTCAAAGAGCCAG
GTTTATCTGGCGCTGAACAGCTTGACGAGTGAGGACACGGCCGTCTATTATTGTACAGGAGGCAAGGGTGACTATGG
TAGATACTGGAATAGTTACGCTGAGGATGGAATAACCAACTGGGGCCAGGGTATCCTGGTCACCGTCTCG
>DQ125417.1 Equus caballus clone 1-7 immunoglobulin heavy chain variable region mRNA, partial cds
CAGGTGCAGCTGAAGGAGTCGGGACCTGGCCTAGTGAAGCCCTCGCAGACCCTCTCCCTCACCTGCACTGTCTCTGG
ATTATCTGACAACAGTAACGCTGTGGGCTGGGTCCGCCAGGCTCCAGGAAAAGGACTGGAATTTGTGGCTGATCTAA
CGGATAGTGACAGTAACCCAGCCCTGAAGTCGCGAGTCAGGATCACCAAGGAACCCTCAAAGAGCCAAGTTCGCCTG
ATTATGAACAGCCTGACAGAAGAGGACACGGCCGTCTATTACTGTATTCATGGTTACTACAATAGTTTTATGGTGGG
AGCGATAAAATATTGGGGCCAGGGTATCCTGGTCACCGTCTCG
>DQ125416.1 Equus caballus clone 1-5 immunoglobulin heavy chain variable region mRNA, partial cds
CAGGTGCAGCTGCAGGAGTCAGGACCTGGCCTGGTGAAGCCCTCGCAGACCCTCTCCCTCACCTGCACTGTCTCTGG
GTTATCTTTGAGCAGTAATACTGTAGGCTGGGTCCGCCAGGCTCCAGGAAAAGGACTGGAATACGTTGGTGCTATAT
ATGGTAGTGCAAGTGCAGCGTACAACCCAGCCCTGAAGTCCCGAGCCAGCATCACCAAGGACACCTCAAAGAGCCAA
GTTTATCTGACGCTGAACAGCCTGACAAGCGAGGACACGGCCGTCTATTACTGTGCAGGAGGAGGCGGTGGTTGGAT
TGGTTATGACTACTTAGGATATTATGATATAAACTACTGGGGCCAGGGTATCCTGGTCACCGTCTCG
>DQ125415.1 Equus caballus clone 1-4 immunoglobulin heavy chain variable region mRNA, partial cds
CAGGTGCAACTGAAGGAGTCAGGACCTGGCCTGGTGAAGCCCTCGCAGACCCTCTCCCTCACCTGCACTGTCTCTGG
GTCATCTTCGGAGGGTTATGGTGTGGGCTGGGTCCGCCAGGCTCCAGGACGAGGACTAGAGTTTGTAGGGGGTATAA
CCAATAGTGGTAGTGCAAGATTTAATCCAGGACTGGCGTCCCGAGCCAGCATTCTCAAGAACACCGAAAAGAGCCAA
GTTTACCTGACGCTGACCGACCTGACAGGCGAGGACACGGCCGTCTATTATTGTGCGAAGGATTCCGAGAGTGGCTT
TCTTTATTGGGGACATTACGGTGTAGAATATTGGGGCCAGGGTATCCTGGTCACCGTCTCG
>DQ125414.1 Equus caballus clone 1-3 immunoglobulin heavy chain variable region mRNA, partial cds
CAGGTGCAACTGAAGGAGTCGGGACCTGGCCTGGTGAAGCCCTCGCAGACCCTCTCCCTCACCTGCACTGTCTCTGG
ATTCTCTTTGAACACTTACGCAGTGGGATGGGTCCGCCAGGCTCCAGGAAAAGGCCTGGAATTTGTTGGTAGTATTT
ATAGTATTGGAAGTGCGACGTACAATTTAGACCTGAAGTCCCGAGTCAGCATCACCAAGGACACCTCAAAGAGCCAA
GTTTATCTGACGGTGAATAGTCTGACAAGTGAGGACACGGCCGTCTATTATTGTGGAAGACGAGTCAATGAAATTGA
CTACTGGGGCCAGGGTATCCTGGTCACCGTCTCG
>KF748792.1 Equus caballus clone IGVDJ95 immunoglobulin heavy chain variable region (IGH) mRNA, partial cds
ATGAGTCACCTGTGGTTCTTCCTCTTTCTGGTGGCCGCTCCTACATGTGTCCTGTCCCAGGTGCAACTGAAGGAGTC
AGGACCTGGCCTGGTGAAGCCCTCGCAGACCCTCTCCCTCACCTGCACTGTCTCTGGCTTATCTTTGAGCGAAAGAG
GTGTAGGATGGGTCCGCCAGGCTCCAGGAAAAGGACTGGAATGGCTTGGTGACATGAATACTAGTGGAGGTGCAAAG
TTCAACCCAGCCCTGAAGTCCCGAGCCAGCATCACCAAGGACACCTCAAAGAGTCAGTTTTATCTGACGCTGAACAG
CATGACAAGTGAGGACACGGCCGTCTATTACTGTGCAGGGGACGTTAGTCCTGGAGGCCTTTTTGATATAGACTGGT
GGGGCCAGGGCATC
>KF748791.1 Equus caballus clone IGVDJ94 immunoglobulin heavy chain variable region (IGH) mRNA, partial cds
ATGAGTCACCTGTGGTTCTTCCTCTTTCTGGTGGCCGCTCCTACATGTGTGGTGTCCCAGGTGCAATTGAAGGAGTC
AGGACCTGGCCTGGTGAAGCCGGCGCAGACCCTCTCCCTCGCCTGCACTGTCTCTGGATTCTCTTTGAGCAGTTACG
GTGTAGGCTGGGTCCGCCAGGCTCCAGGAAAAGGGCTGGAATTGGTTGGTAGGATATTCTCTGATGGAAGCACAGAC
TACAACCCAGCCCTGAAGTCCCGAGCCAGCGTCACCAGAGACACCGGAAAGAGCCAAGTTTATCTGACGCTGAACAG
CCTGACAGGCGAGGACACGGCCGTCTATTACTGTGCGAGAGGCACTGATGGGAGTGCTAGTGGAGATGATATGTACT
TTTGGGGCCAGGGCATC
>KF748790.1 Equus caballus clone IGVDJ93 immunoglobulin heavy chain variable region (IGH) mRNA, partial cds
ATGAGTCACCTGTGGTTCTTCCTCTTTCTGGTGGCCGCTCCTACATGTGTCCTGTCCCAGGTGCAACTGAAGGAGTC
AGGACCCGGCCTGGTGAAGCCCTCGCAGACCCTCTCCCTCAGCTGCGGTGTCTCTGGATTATCTTTGGGCAGTAAGA
CTATAGGCTGGGTCCGCCAGGCTCCAGGAAAAGGGCTGGAATATGTAGGTGCTATATCTGGTGAGAAGTCAAACTAC

AATCCAGCCCTACAGTCCCGATGTAGAATCACCAAGGACACTTCAAAGAGCCAAGTCTATCTGACGCTGGAAGGCCT
GACAAGTGAGGACACGGCCGTCTATTATTGTACAGGAGGGGCCAGTGCTGATGATATGGTCCTTGACGCATTTTACT
TCAGGCTAAGTCACTGGGGCCAGGGCATC
>KF748789.1 Equus caballus clone IGVDJ92 immunoglobulin heavy chain variable region (IGH) mRNA, partial cds
ATGAGTCACCTGTGGTTCTTCCTCTTTCTGGTGGCCGCTCCTACATGTGTCCTGTCCCAGGTGCACTTGAAGGAGTC
AGGACCTGGCCTGGTGAAGCCCTCGCAGACCCTCTCCCTCACCTGCACTGTCTCTGGATTATCTTTGAACACTTATG
GTGTGGGCTGGGTCCGCCAGGCTCCGGGAAAAGGGCTGGAATTTGTTGGTGGTATAGCCCGTAGTGGAATTGCAAAG
TACAATCCAGACCTGAAGTCCCGAGCCAGCATCACCAAGGACATCTCAAAGAGCCAAAGTTACCTGACGCTGAACAG
CCTGACAATAGAGGACACGGCCGTCTATTACTGTACAGGGGGAGATGGTTACGGTACTTTTTACTTTGACAATGATG
GAATATTGTATTGGGCCCAGGGCATC
>KF748788.1 Equus caballus clone IGVDJ91 immunoglobulin heavy chain variable region (IGH) mRNA, partial cds
ATGAGTCACCTGTGGTTCTTCCTCTTTCTGGTGGCCGCTCCTACATGTGTCCTGTCCCAGGTGCTACTGAAGGAGTC
AGGACCTGGCCTGGTGAAGCCCTCGCAGACCCTGTCCCTCACCTGCACTGTCTCTGGATTAGATTTGACTACTAATA
CTGTCACCTGGGTCCGCCAGGCTCCAGGAAAAGGGCTGCAATATGTTGGTGAACTGACAACTCTCGGAAATCTATAC
GTGAACCCAGCCCTCAAGTCCCGAGCCAGCATCACCAAGGACACCTCAAAGAGCGAAGTCACTCTGACGCTGAGAAG
CCTGACAAGCGAGGACACGGCCCTCTATTTTTGTACAGCAGAACTGGGGAACAACGATTATTATTTCATGAACTACT
GGGGCCAGGGAATC
>KF748787.1 Equus caballus clone IGVDJ90 immunoglobulin heavy chain variable region (IGH) mRNA, partial cds
ATGAGTCACCTGTGGTTCTTCCTCTTACTGGTGGCCGCTCCTACATGTGTCCTGTCCCAGGTGCAATTGGAGGAGTC
AGGACCTGGCCTGGTGAAGCCCTCGCAGTCTCTCTCCCTCACTTGCACTGTCTCTGAGTGGAATTTGAAGAGTTGGG
CTGTGAGCTGGGTCCGCCAGACGCCAGAAAAAGGCCTGGAATTCATTGTTGGCAGTTGGGAGGGCGGGAAGCCTAAT
TACAATCCAGCCCTGAGGTCCCGCGTTGTGATTACCCTCGACACCTCGAAGGACCAAGTTTATCTAACACTGAATAA
CCTGGCAAGCGAAGACACGGCCGTCTATTATTGTGCAGGAGGAAGCGACCACCCTGATAATCTTCACTACTGGGGCC
CGGGCATC
>KF748786.1 Equus caballus clone IGVDJ89 immunoglobulin heavy chain variable region (IGH) mRNA, partial cds
ATGAGTCACCTGTGGTTCTTCCTCTTTCTGGTGGCCGCTCCTACATGTGTCCTGTCCCAGGTGCAACTGAGGGAGAA
AGGACCTGGCCTGGTGAAGCCCTCGCAGAGCCTGACCCTCACCTGCACTGTCTCTGGATTATCCTTGACCAGTAATG
CTGTAGGCTGGGTCCGCCAGGCTCCAGGAAAAGGTCTGGAGTTCGTTGGTAGTGTTCTAAATGATTTCAATGGAAAG
TACAATCCAGACCTTCAGTCCCGAGCCAGCATCACCAAGGACACCGAAAGGGGCCAGTCTTATTTGACGCTGAACAG
CCTGACAAGCGAGGACACGGCCGTCTATTACTGTGCAAAATTCTTCAAGAATGGTATTGACCACTGGGGCCAGGGCA
TC
>KF748785.1 Equus caballus clone IGVDJ88 immunoglobulin heavy chain variable region (IGH) mRNA, partial cds
ATGAGTCACCTGTGGTTCTTCCTCTTTCTGGTGGCCGCTCCTACATGTGTCCTGTCCCAGGTGCAACTGAAGGAGTC
AGGACCTGGCCTGGTGCAGCCCTCGCAAACCCTCTCCCTCACCTGCACTGTCTCTGGATTATCTTTGAGAGACGACG
TTCTGGGCTGGGTCCGCCAGGCTCCAGGAACAGGGCTGGAATTTGTTGCTACAATCTATAATAGTGGAAGTTCAAAG
TACAACCCAGCCCTGAAGTCCCGAGCCAGCATCACCCTGGACACCTCAAAGAGCGAAGTGCATCTGACACTGAACAG
CCTGACAAGCGAGGACACGGCCGTCTACTACTGTGCAGGAGGAGGGGGTATGTGGGGTTATGGTCCGTCATCTTTTG
GCTACTGGGGCCAGGGCACC
>KF748784.1 Equus caballus clone IGVDJ87 immunoglobulin heavy chain variable region (IGH) mRNA, partial cds
ATGAGTCACCTGTGGTTCTTCCTCTTTCTGGTGGCCGCTCCTACATGTGTCCTATCCCAGGTGCAACTGAAGGAGTC
AGGACCTGGCCTGGTGAAACCCTCGCAGACCCTGTCCCTCACCTGCACTGTCTCGGGGTTCTCTTTGAGCAGTACCG
GTGTAGGCTGGGTCCGCCAGGCTCCTGGAAGCGGGCTGGAATATGTTGGTGTGATACGTAGTGATGGAACCACGTTC
TTCAATCACGCCCTGAAGTCCCGAGCCAGCATCTCCAAGGACGCCTCAAAGGGCCAAGTGGTTCTGACGCTGAACAG
TCTGACAGGCGAGGACACGGCCGTCTATTATTGTTGGGCATCAGGTTACGGCTATTGGGGCCAGGGCATC
>KF748783.1 Equus caballus clone IGVDJ86 immunoglobulin heavy chain variable region (IGH) mRNA, partial cds
ATGAGTCACCTGTGGTTCTTCCTCTTTCTGGTGGCCGCTCCTACATGTGTCCTGTCCCAGGTGCCACTGAAGGAGTC
AGGACCTGGCCTGGTGAAGCCCTCGCAGACCCTCTCCCTCACCTGTACTGTCTCTGGAGTGGATTTGAGTAGGAATG
CTGTAGGCTGGGTCCGCCAGGCTCCAGGAAAAGGGCTGGAGTATGTTGGTGGTTTATCTACTAGTGGGCTTACAAGA

TATGGTTCAGTCCTGAAGTCCCGAGTCAGCATCATCAAGGACGACGATAAGAGCCAAGTTATTCTGACGCTCAACAG
CCTGACAATCGAAGACACGGCCGTCTATTTTTGTGCAGGAGGTTCATTGGACGGTGGTTTTGGTGGTGTAAGTTTTT
TCTTGCCTAATGGTATAAAGTTCTGGGGCCAGGGCATC
>KF748782.1 Equus caballus clone IGVDJ85 immunoglobulin heavy chain variable region (IGH) mRNA, partial cds
ATGAGTCACCTGTGGTTCTTCCTCTTTCTGGTGGCCACTCCTACATGTGTCCTGTCCCAGGTGCAACTGAAGGAGTC
AGGACCTGGCCTGGTGAAGCCCTCGCAGACCCTGTCCCTCACCTGCCTTGTCTCCGGATTCTCTTTGAGTAGTTACG
CTGTAGGCTGGGTCCGCCAGGCTCCAGGAAAAGGGCTGGAATTTGTTGGTGGTCAGAATAAAGATGGAGGTCGCGCA
TACAATCCAGCCCTGAAGTCCCGAGCCAGCATCTCCGTGGACACCTCAAAGAGTCAAGTTTATCTGACGCTGAACGA
TCTTACAGTCGAGGACACGGCCGTCTATTATTGTGTGAGATATTGTCCGGAGGGAAGTGAACATTATTATGGTGTTT
ACTCTGGTGATTACTATGTTCCTTTCGGCTACTGGGGCCAGGGCACC
>KF748781.1 Equus caballus clone IGVDJ84 immunoglobulin heavy chain variable region (IGH) mRNA, partial cds
ATGAATCACCTGTGGTTCTTCCTCTTTCTGGTGGCCGCTCCTACATGTGTCCTGTCCCAGGTGCAACTGAAGGAGTC
AGGACCTGGCCTGGTGAAGCCCTCGCAGACCCTGTCCCTCACCTGCACTGTCTCTGGATTCTCTTTGAGTGATTGGG
CTGTATATTGGGTCCGCCAGACTCCAGGAAAAGGGCTGGAATATGTCGGTTTAATAAGTGGTGGTTCGTTTTCAAGT
TACAGCCCATCCCTGAAGTCCCGAGCCAGCATCACCAAGGACCCCTCAAAGAACCAAGTTTATCTGACGCTGAACAG
CCTGACAGGCGAGGACACGGCCGTCTATTGGTGTGCGAGAGCCCTATTAGACTGGGGCCAGGGCATC
>KF748780.1 Equus caballus clone IGVDJ83 immunoglobulin heavy chain variable region (IGH) mRNA, partial cds
ATGAGTCACCTGTGGTTCTTCCTCTTTCTGGTGGCCGCTCCTACATGTGTCCTGTCCCAGGTGCAACTGAAGGAGTC
AGGAACTGGCCTGGTGAAGCCCTCGCAGACCCTCTCCCTCACCTGCACTGTCTCTGGATTAAGTCCCCAGAGAGATG
GTGTGGGCTGGGTCCGCCAGGCTCCAGGAAAAGGGCTGGAATTTATTGGTGCAATACCTTGGCCTGGAAATCCATAC
TACAACCCAGCCCTGAAGTCCCGAGCCAGCATCACCGAGGACACCTCAAAGAGCCAAGTGACGCTCACGCTGGACAG
CCTGACAAGCGAGGACACGGCCGTCTATTATTGTGCAGCCCGTCAGGGCGTTGGTCATTTTTTCTGGGGAAATCTTG
GAATAGACTACTGGGGCCAGGGCATC
>KF748779.1 Equus caballus clone IGVDJ82 immunoglobulin heavy chain variable region (IGH) mRNA, partial cds
ATGAGTCACCTGTGGTTCTTCCTCTTTCTGGTGGCCGCTCCTACATGTGTCCTGTCCCAGGTGCAGCTGAAGGAGTC
GGGACCTGGCCTGGTGAAACCCACGCAGACCCTGTCCCTCACCTGCACTTTGTCTGGATTATCTTTGAGTTCTACGG
ATGTAGGCTGGGTCCGCCAGGCTCCAGGAAAAGGGTTGGAATTTGTTGGTGCTGGTGTGGAATCAGGTAGTGAGGAA
GTTAACTACAACCCAGCCCTGAAGTCCCGAGCCAGCATCACCAAGGACACCTCAAAGAGCGTAGTTTATCTGACGCT
GAACAGCCTGACAAGCGAGGACACGGCCGTCTATTATTGTGGATTAGGGAACTACTACAGTTATGGAGGTTACTATG
CCGGCGATATAAACTACTGGGGCCAGGGCATC
>KF748778.1 Equus caballus clone IGVDJ81 immunoglobulin heavy chain variable region (IGH) mRNA, partial cds
ATGAGTCACCTGTGGTTCTTCCTCTTTCTGGTGGCCGCTCCTACATGTGTCCTGTCCCAGGTGCAACTGAAGGAGTC
AGGACCTGGCCTGGTGAATCCCTCGCAGACCCTCTCCCTCACCTGCACTGTCTCTGGATTATCTTTGAGTAGTTATA
GTGTGGGCTGGGTCCGCCAGGCGCCAGGAAAAGGACTGGCATTTGTTGGTGATATAAGCGCCGGTGGGGATACTGAC
TACAACCCGTCCCTGAAGTCCCGAGCAAGCATTACCGCGGACACCTCAAGGAGCCAAATTTATCTGACGCTCGACAG
CCTGACGGAGGAGGACACGGCCGTCTATTTCTGTGCAGGAGGGACCCATGTAGGAAACCTATACTCAAACGGAATAA
AGTATTGGGGCCAGGGCATC
>KF748777.1 Equus caballus clone IGVDJ80 immunoglobulin heavy chain variable region (IGH) mRNA, partial cds
ATGAATCACCTGTGGTTCTTCCTCTTTCTGGTGGCCGCTCCTACATGTGTCCTGTCCCAGGTGCAATTGAAGGAGTC
AGGACCTGGCCTGGTGAAGCCCTCGCAGGGTCTGTCCCTCACCTGCACTGTCTCTGGCTTATCTTTGAGCAGTTTTG
GTGTGGCCTGGGTCCGCCAGGCTCCAGGAAAAGGGCTGGAATTTGTTGGTACTTCGGATAGTGATGGAGGTGCGTGG
TACAACCCAGTCCTGAAGTCCCGAGCCAACATCACCAGGGACACCTCAAAGAGCCAAGTCTATCTGACGCTGAACAG
CCTGACAAGCGAGGACACGGCCGTCTATTTTTGTGCGGGATGTTCTTCAATGTATTACGCTATAGCGATGTGTGAGA
CCCAGTGGGGCCAGGGTATT
>KF748776.1 Equus caballus clone IGVDJ79 immunoglobulin heavy chain variable region (IGH) mRNA, partial cds
ATGAGTCACCTGTGGTTCTTCCTCTTTCTGGTGGCCGCTCCTACATGTGTCCTGTCCCAGGTGCAACTGAAGGAGTC
AGGACCTGGCCTGGTGAAGCCCTCGCAGACCCTCTCCCTCACCTGCACTGTCTCTGGATTATCTTTGAGCGTTTATG
GTGTGGCCTGGGTCCGCCAGGCTCCAGGAAAAGGGTTGGAATTTATTGGTGGTAAATCTGATAGTGGTACGTCAAAC
GCGAACCCAGCCCTGAAGTCCCGAGCCATGATTACCAAGGACACCTCAAAGAGCCAAGTTTATCTGACGCTGAACAG

CCTGACAAAAGAGGACACGGCCGTCTATTACTGTGCAGGACGACGTTGGGGCGATGCAAATTACTATGGATTGGCCT
TTGACTACTGGGGCCAGGGCACC
>KF748775.1 Equus caballus clone IGVDJ78 immunoglobulin heavy chain variable region (IGH) mRNA, partial cds
ATGAGTCACCTGTGGTTCTTCCTCTTTCTGGTGGCCGCTCCTACATGTGTCCTGTCCCAGGTACAACTGAAGGAGTC
GGGACCTGGCCTGCTGAAGCCCTCGCAGACCCTGACCCTCACCTGCGAGGTGGCTGGATTCTCTTTGACCAGTTGGG
CGGTAGGATGGGTCCGCCACTCTCCAGGGAAAGGGCTGGATTTTGTTGGCGGCGTAGGTTATGGTGGGAAGTCTGAA
TACAACCCAGCCCTAGAGTCCCGAGCCACAATTACGAGGGACACTTCAAAGAGTCAAGTCACGCTGACGCTGTACGG
AATGACAGGCGAGGACACGGCCGTCTATTATTGTGTCAGAAGCGAGACTTTTTTCTTCCGATTAGGCAGCGTGGATC
AATGGGGCCAGGGCACC
>KF748774.1 Equus caballus clone IGVDJ77 immunoglobulin heavy chain variable region (IGH) mRNA, partial cds
ATGAATCACCTGTGGTTCTTCCTCTTTCTGGTGGCCGCTCCTACATGTGTCTTGTCCCAGGTGCAACTGAAGGAGTC
GGGACCTGCACTGGTGAAGCCCTCGCAGACCCTGTCCCTCACCTGCACTGTTTCTGGATTCGATTTGAGCTCCAACA
CAGTGGGCTGGGTCCGCCAGGCTCCAGGAAAAGGGCTGGAGTGGGTTGCTAGGGTATATGATAATGAGGCAACATAT
TACAATCCAGTCCTGAGGTCCCGAGCCAGCATCACCAAGGACTACTCAAAGAGCGCAGTAATCCTGACGCTGAACAG
CCTGACAGGCGAAGACACGGCCGTCTATTACTGTGCAGGATATGTCGTTGGTGCTGGTGGGACTATCTACTTTGGGA
ATTGGGGTATTCGACACTGGGGCCAGGGCATC
>KF748773.1 Equus caballus clone IGVDJ76 immunoglobulin heavy chain variable region (IGH) mRNA, partial cds
ATGAATCACCTGTGGTTCTTCCTCTTTCTAGTGGCCGCTCCTACATGTGTCCGAGCCCAGGTTGAACTGAAGGAGTC
GGGACCTGGCCTGGTGAAGCCCTCGCAGACCCTGTCCCTCACCTGTTCTGTCTCTGGTTATTCTTTGAACGGTGGGG
AGGTGAACTGGGTCCGCCAGGCTCCAGGAAGGGGGCTGGAGTGGATTGGTGAAATACGCGGTGATGATCATCGCACG
AATTACAATTTGACCCTGAAGTCCCGAGCCAGGCTCACTATGGACAATGCAAAGAGCCAGGTTTACCTGACTCTAAA
CAGCCTGACAGAAGAAGACGCGGCCGTCTATCTGTGTGTTATTGCCGGAGAGGGGCTTTTTGGAGCTTACCTCTACT
GGGGCCAGGGCATC
>KF748772.1 Equus caballus clone IGVDJ75 immunoglobulin heavy chain variable region (IGH) mRNA, partial cds
ATGAATCACCTGTGGTTCTTCCTCTTTCTGGTGGCCGCTCCTACATGTGCCCTGTCCCAGGAACAACTGAAGGAGTC
GGGACCTGACCTGGTGAAGCCCTCGCAGACCCTGTCTCTCACTTGCACTGTCTCTGGACCAGGGTTGTTTAGTAATG
CCCTAGGCTGGGTCCGCCAGGCTCCAGGAAAAGGGCTGGAGTGGATAGGAGAATTGTGGAAAGATGATTTTACAGGA
AACGGGAATACGTATATACATCCAGGGCTGAAGTCGCGAGCCTTCATCTACAAAGACCGCTCCAAGAGTCAAGTAAG
TCTGACACTGAACAGCCTGACAGAAGAGGACACGGCCGTCTATTATTGTGTAGCATATGATAATAGTGGGGCCAACT
GGATGTCCGAAGAAACGATCGTCTACTGGGGCCAGGGCATC
>KF748771.1 Equus caballus clone IGVDJ74 immunoglobulin heavy chain variable region (IGH) mRNA, partial cds
ATGAATCACCTGTGGTTCTTCCTCTTTCTGGTGGCCGCTCCTACATGTGTCCTGTCCCAGGTTCAACTGAAGGAGTC
AGGACCTGGCCTGGTGAAGCCCTCGCAGGCCCTCTCCCTCACCTGCACGGTCTCTGGATCCTTGCCGAGAGGTGGAG
CTGTAGGCTGGATCCGCCAGGCTCCAGGAAAAGGCCTGAAATATGTTGGATTTGCAACTGATGATGGACGTGTACAC
TTGACTCCAGCCCTGAGCACCCGAGCCAGCATCACCAGCGACACCTCAAAGAACGAAGTTTATCTGACACTGAATAG
CCTGACAATCGAGGACACGGCCGTCTATTACTGTGCAGCCTTCTTCCGTGAGGGTGTAGGTTATTGGGGCCAGGGCA
TC
>KF748770.1 Equus caballus clone IGVDJ73 immunoglobulin heavy chain variable region (IGH) mRNA, partial cds
ATGAGTCACCTGTGGTTCTTCCTCTTTCTGGTGGCCGCTCCTACATGTGTCCTGTCCCAGGTCCAACTGAAGGAGTC
AGGACCTGGCCTGGTGAAGCCCTCGCAGACCCTCTCCCTCACCTGCACTGTCTCTGGAGCTTCTCTGAGCAGTAATG
CTGTAGGCTGGGTCCGCCAGGCTCCAGGAAAAGGGCTGGAATATGTTGGTGATATGTCTAGTGGAGGGAGTCCAAGA
TACGACCCAGCCCTGAAGTCCCGAGCCAGCATCACCAGGGATGCCTCAAAGAGCCAAGTTTATCTGACGCTGAACAG
CCTGACAAGCGAGGACACGGCCGTCTATTACTGTGCAGGAGGCGGTGAATATGGAGGCGCTTTGTTTGATAGTTCTT
ATGTGTGGGAAATAGACTATTGGGGCCAGGGCATC
>KF748769.1 Equus caballus clone IGVDJ72 immunoglobulin heavy chain variable region (IGH) mRNA, partial cds
ATGAGTCACCTGTGGTTCTTCCTCTTTCTGGTGGCCGCTCCTACATGTGTCCTGTCCCAGGTGCAACTGAAGGAGTC
AGGACCTGGCCTGGTGAAGCCCTCGCAGACCCTCTCCCTCACCTGCACTGTCTCTGGATTTCCTTTGAGCAGTTATA
CTGTAGGCTGGGTCCGCCAGGCTCCAGGAAAAGGGCTGGAATATGTTGGTATTATAGAAAGTACTGCAAGTCAAATG

TACAATCCAGCCCTGAAGTCCCGAGCCAGCATCACCAAGGACACCTCAAAGAGCCAAGTTTATCTGACGCTGAACAG
CCTGACAGGCGAGGACACGGCCGTCTATTACTGTGCGGGATTGATGTGGTGGGGTGCTGATGGTGGTCGGCGATACT
ACTATGATATACCCTACTGGGGCCAGGGCACC
>KF748768.1 Equus caballus clone IGVDJ71 immunoglobulin heavy chain variable region (IGH) mRNA, partial cds
ATGAGTCACCTGTGGTTCTTCCTCTTTCTGGTGGCCGCTCCTACATGTGTCCTGTCCCAGGTGCAACTGAAGGAGTC
AGGACCTGGCCTGGTGAAGCCCTCGCAGACCCTCTCCCTCACCTGTACTGTCTCCGGATTATCTTTGACCGACAATC
ATGTAGGCTGGGTCCGCCAGGGTCCAGGAAAAGGTCTGGAATATGTTGGTTATATACATAGTCATGGAGAGACACTC
TACAATCCAAACCTGAAGTCCCGAGCGCTCATCACCAAGGACGACTCAAAGAGCCAAGTTTATCTGACGCTGAACAG
TCTGACGAGCGAGGACACGGCCGTCTATGTCTGTGCGAAGTGGGGTAATGGTGGTGCTTACTACGCTTCGTATGGTG
AGGCTTATTGGGGCCAGGGCATC
>KF748767.1 Equus caballus clone IGVDJ70 immunoglobulin heavy chain variable region (IGH) mRNA, partial cds
ATGAGTCACCTGTGGTTCTTCCTCTTTCTGGTGGCCGCTCCTACATGTGTCCTGTCCCAGGTAGAACTGAAGGAGTC
AGGACCTGGCCTGGTGAAGCCCTCGCAGACCCTCTCCCTCACCTGCGCTGTCTCTGGATTATCTTTGAGCAATAATG
TTGTGGCGTGGGTCCGCCAGGCTCCAGGAAAAGGGCTGGAATTTGTTGGTTCGATATATGGAACGAGAGGTGCCCAC
TACAACCCGGCCCTAGAGTCCCGAGCCAGCATCACCAGGGACATTGGGGCGAGCCGTATTGGTCTGACGCTGAACAG
CCTGACAAGCGACGACACGGCCGTCTATTACTGTGCAGGGGGCATTTCCTCTTCTCTACCTACGGGGATGCTGAGAC
GGACACAGCCTACGCCCTTGCACTTATGGGGCCAGGGCATC
>KF748766.1 Equus caballus clone IGVDJ69 immunoglobulin heavy chain variable region (IGH) mRNA, partial cds
ATGAGTCACCTGTGGTTCTTCCTCTTTCTGGTGGCCGATCCTACATGTGTCCTGTCCCAGGTGCAACTGAAGGAGTC
AGGACCTGGCCTGGTGAAGCCCTCGCAGACCCTCTCCCTCACCTGCACTGTCTCTGGATTATCTTTGAGCAGTAACG
CTGTGGCCTGGGTCCGTCAGGCTCCAGGAAAAGGGCTAGAAGAGGTCGGTAGTATAGCGAGTCGTGGAAGAACAAGT
TACAACCCAGCCCTGAAGTCCCGAGCCAGCATCACCAAGGACACCTCAAAGAGCCAAGTTTATCTGACGCTGAACAG
CCTGACAATGGAGGACACGGCCGTCTATTACTGTGCAGGAGACAGCCACTCTGGTGAGAGTGTCCAACATTTGATCG
GTGAAAACTACTGGGGCCAGGGCATC
>KF748765.1 Equus caballus clone IGVDJ68 immunoglobulin heavy chain variable region (IGH) mRNA, partial cds
ATGAGTCACCTGTGGTTCTTCCTCTTTCTGGTGGCCGTTCCTACATGTGTCCCGTCCCAGGTGCAATTGAAGGAGTC
AGGACCTGGCCTGGTGAAGCCCTCGCAGACCCTCAGCCTCACCTGCACTGTCCGTGGCTTATCTTTGAGCAGCAGGG
CTGTAGGCTGGGTCCGCCAGGCTCCAGGAAAAGGTCTGGAATTTGTTGCTGACATAAGTGCTGGTGATGGTACAATG
TACAACCCAGCCCTGAAGTCCCGAGCCAGCATCACTAAGGACACCTCAAAGAGCCAAGTTTATCTGACGTTGAACAG
CCTGACAAGCGAGGACACGGCCGTCTATTATTGTGCAGGAGGCCTAGATTTTCACGATGCCATCGGTCCTGATAATA
CGTACTGGGGCCAAGGCATC
>KF748764.1 Equus caballus clone IGVDJ67 immunoglobulin heavy chain variable region (IGH) mRNA, partial cds
ATGAGTCACCTGTGGTTCTTCCTCTTTCTGGTGGCCGCTCCTACATGTGTCCTGTCCCAGGTGGAACTGAAGGAGTC
AGGACCTGGCCTGGTGAAGCCCTCTCAGACCCTGTCCCTCATCTGCACTGTCTCTGGCTTCACTTTGAACGAATACG
GTGTAGCCTGGGTCCGCCAGGCTCCAGGAAAAGGACTGGAGTATGTTGCAGGGATACGTAAAGATGGGGAGGCAGTG
TACAACCCAGCCCTGAAGTCCCGAGCCAGCATCACCAAGGACGCCTCAAAGAGTGAAGTTTATTTGTCACTGAACAG
CCTGACAGGCGAGGACACGGCCGTCTATTACTGTGCGAGATCCTGGTATATTGGGGACTACTGGGGCCAGGGCACC
>KF748763.1 Equus caballus clone IGVDJ66 immunoglobulin heavy chain variable region (IGH) mRNA, partial cds
ATGAATCACCTGTGGTTCTTCCTCTTTCTGGTGACCGCTCCTACATGTGTCCTGTCCCAGGTACAACTGAGCGAGTC
GGGACCTGGCCTGATGAAGCCCTCGCAGACCCTGTCCCTCACCTGCACGATCTCTGGCTTGCCACTAACGGCGAATG
GTGTAGGCTGGGTCCGCCAGATTCAGGGAAAAGGCCTTGAGTGGATTGGGATAATATATTATCCCGGAAATACGGAC
TATAACCCAGCCCTGAAGTCCCGAGCTCTGATTACAAGGGACACCTCTAAGAACATTGTGTGGCTGACGCTGAGCGG
CCTGACAGTGGAAGACACGGCCGTCTACTACTGTGCAGGGATGGGACATGAATATAAGATACATACATGGGGCCAGG
GCAAC
>KF748762.1 Equus caballus clone IGVDJ65 immunoglobulin heavy chain variable region (IGH) mRNA, partial cds
ATGAATCACCTGTGGTTCTTCCTCTTTCTGGTGGCCGCTCCTACATGTGTCCTGTCCCAGGTGGTACTGAAGGAGTC
GGGACCTGGCCTGGTGAAGCCCTCGCAGACCCTGTCCCTCACCTGCACTGTCTCTGGCTTATCTTTGAACACTGACA
CCATAGGCTGGGTCCGCCAGGCTCCAGGAAAAGGGCTGGAATGGGTTGGTGTGATAGGGAATGGGGAGAATCGATGG
TACAACCCAGCCCTGAAGTCCCGAGCCAGCGTCGACAAGGACACCTCAAAGACTGAAGTTTATCTGACGCTGAATAG

CCTGACAGGCGAAGACAGTGGCGTCTATTTCTGCGCAGGATCCATTGGGAGTTTTGCCGATGGTATAGACAGATGGG
GCCAGGGCATT
>KF748761.1 Equus caballus clone IGVDJ64 immunoglobulin heavy chain variable region (IGH) mRNA, partial cds
ATGAATCACCTGTGGCTTTTCCTCTTTCTGGTGGCCGCTCCTACATGTGTCCTGTCCCAGGTGCAACTGAAGGAGTC
GGGACCTGGCCTGGTGAAGCCCTCGCAGACCCTGTCCCTCACCTGCACTGTCTCTGGCTTCTCTTTGAGCAGTAATG
CTGTAGGCTGGGTCCGCCAGGCCCCAGGAAAAGGGCTGGAGTGGGTTGGTGTTATAGGCGTTGATGGTTCTACATAC
TACAGCCCAGCCCTGAGGTCCCGAGCCAGCATCACCAAGGACACCTCAAAGAGCCAAGTTTATCTGACGCTGAACAG
CCTGACAGGCGAAGACACGGCCGTCTATTACTGTGTAGGGAGTGATTACGGTACTTATTACTTTCTCGCGAATATAA
ACTACTGGGGTCAGGGCATC
>KF748760.1 Equus caballus clone IGVDJ63 immunoglobulin heavy chain variable region (IGH) mRNA, partial cds
ATGAATCACCTGTGGTTCTTCCTCTTTCTGGTGGCCGCTCCTACATGTGTCCTGTCCCAGGTGCAACTGAAGGAGTC
AGGACCTGGCCTGGTGAAGCCCTCGCAGACCCTCTCCCTCACCTGCACTGTCTCTGGATTCTCTTTGAGCAGTTATG
CTGTATACTGGGTCCGCCAGGCTCCAGGAAAAGGGCTGGAATATGTTGGTGCTATATATGGTAGTGCAAGTGCAAAC
TACAACCCAGCCCTGAAGTCCCGAGCCAGCATCACCAAGGACACCTCAAAGAGCCAAGTTTATCTGACGCTGAACAG
CCTGACAGGCGAGGACACGGCCGTCTATTACTGTGCGAGACTTGATGACTATGGTGATACTTTCTACTACAGACAAA
ACCCTCTTGGCTACTGGGGCCAGGGCACC
>KF748759.1 Equus caballus clone IGVDJ62 immunoglobulin heavy chain variable region (IGH) mRNA, partial cds
ATGAGACTCTTGTGTCTTCTCCTTTTCCTGGTGACGGCTCCCCAAGGTGTCCTGTCCCAGGTGCAGCTGCAGGAGTC
GGGCCCAGGACTGGTGCAGCCCTCACAGACCCTCTCCCTCACCTGCACTGTCACTGGAGGCTCCATCACAAGCAGCT
ATTCTAGCTGGAGCTGGTTACGCCAGGCTCCAGGGAAGGGGCTGGAGTACATGGGGTACATATATTATGATGGTAGA
ACTTACTACAATCCTTCCTTCAAGAGCCGCACCTCCATCTCCAGAGACACCTCCAGGAACCAGTTCTCCCTGCAGCT
GAGCTCCGTGACCGCTGAGGACGCGGCCGTGTATTACTGTGCTGAAGGGTGCTTACTACTTGGCTACTGGGGCCAGG
GCACC
>KF748758.1 Equus caballus clone IGVDJ61 immunoglobulin heavy chain variable region (IGH) mRNA, partial cds
ATGAGTCACCTGTGGTTCTTCCTCTTTCTGGTGGCCGCTCCTACATGTGTCCTGTCCCAGGTGCAACTGAAGGAGTC
AGGACCTGGCCTGGTGAAGCCCTCGCAGACCCTCTCCCTCACCTGCACTGTCTCTGGATTATCTTTGAGCAGTAATG
CTGTGGGCTGGGTCCGCCAGGCTCCAGGAAAAGGGCTGGAATATGTTGGTGGTATAGCTAGTAGTGGAAGTGCAAAC
TACAACCCAGCCCTGAAGTCCCGAGCCAGCATCACCAAGGACACCTCAAAGAGCCAAGTTTATCTGACGCTGAACAG
CCTGACAAGCGAGGACACGGCCGTCTATTACTGTGCAGGAGTACGTTATTATGGTGCTATTGACTACATGGGGTATT
ATGGTATAAACTACTGGGGCCAGGGCATC
>KF748757.1 Equus caballus clone IGVDJ60 immunoglobulin heavy chain variable region (IGH) mRNA, partial cds
ATGAGTCACCTGTGGTTCTTCCTCTTTCTGGTGGCCGCTCCTACATGTGTCCTGTCTCAGGTGCAACTGAAGGAGTC
AGGACCTGGCCTGGTGAAGCCCTCGCAGACCCTCTCCCTCACCTGCACTGTCTCTGGATTATCTTTGAGCAGTAATG
CTGTAGGCTGGGTCCGCCAGGCTCCAGGAAAAGGGCTGGAATATGTTGGTGGTATAGCTAGTAGTGGAAGTGCAAAC
TACAACCCAGCCCTGAAGTCCCGAGCCAGCATCACCAAGGACACCTCAAAGAGCCAAGTTTATCTGACGCTGAACAG
CCTGACAAGCGAGGACACGGCCGTCTATTACTGTGCAGGAGGACTTGACTATGGTGATACTTTCTACTTTTTGGGCT
TATATGGTATAAACTACTGGGGCCAGGGCATC
>KF748756.1 Equus caballus clone IGVDJ59 immunoglobulin heavy chain variable region (IGH) mRNA, partial cds
ATGAGTCACCTGTGGTTCTTCCTCTTTCTGGTGGCCGCTCCTACATGTGTCCTGTCCCAGGTGCAACTGAAGGAGTC
AGGACCTGGCCTGGTGAAGCCCTCGCAGACCCTCTCCCTCACCTGCACTGTCTCTGGATTATCTTTGAGCAGTAATG
CTGTAGGCTGGGTCCGCCAGGCTCCAGGAAAAGGGCTGGAATGGGTTGGTGCTATATATGGTAGTGCAAGTGCAAAC
TACAACCCAGCCCTGAAGTCCCGAGCCAGCATCACCAAGGACACCTCAAAGAGCCAAGTTTATCTGACGCTGAACAG
CCTGACAAGCGAGGACACGGCCGTCTATTACTGTGCAGGAGGCTTTCGTCATAACTACGGTTATGGTTATGCTACTT
ATGGTATAAACTACTGGGGCCAGGGCATC
>KF748755.1 Equus caballus clone IGVDJ58 immunoglobulin heavy chain variable region (IGH) mRNA, partial cds
ATGAGTCACCTGTGGTTCTTCCTCTTTCTGGTGGCCGCTCCTACATGTGTCCTGTCCCAGGTGCAACTGAAGGAGTC
AGGACCTGGCCTGGTGAAGCCCTCGCAGACCCTCTCCCTCACCTGCACTGTCTCTGGATTATCTTTGAGTAGTTATG
GTGTGGGCTGGGTCCGCCAGGCTCCAGGAAAAGGGCTGGAATTTGTTGGTGGTATAGCTAGTAGTGGAAGTGCAAAC

TACAACCCAGCCCTGAAGTCCCGAGCCAGCATCACCAAGGACACCTCAAAGAGCCAAGTTTATCTGACGCTGAACAG
CCTGACAAGCGAGGACACGGCCGTCTATTACTGTGCAGGAGGCTTCTATAGCAGCTATGCTTATTATTATGGTATAA
ACTACTGGGGCCAGGGCATC
>KF748753.1 Equus caballus clone IGVDJ56 immunoglobulin heavy chain variable region (IGH) mRNA, partial cds
ATGAGTCACCTGTGGTTCTTCCTCTTTCTGGTGGCCGCTCCTACATGTGTCCTGTCTCAGGTGCAACTGAAGGAGTC
AGGACCTGGCCTGGTGAAGCCCTCGCAGACCCTCTCCCTCACCTGCACTGTCTCTGGATTATCTTTGAGCAGTAATG
CTGTAGGCTGGGTCCGCCAGGCTCCAGGAAAAGGGCTGGAATATGTTGGTGGTATAGCTAGTAGTGGAAGTGCAAAC
TACAACCCAGCCCTGAAGTCCCGAGCCAGCATCACCAAGGACACCTCAAAGAGCCAAGTTTATCTGACGCTGAACAG
CCTGACAAGCGAGGACACGGCCGTCTATTACTGTGCAGGAGTCTGTTTGGATTATTATGGTGCTATTGACTACATAA
ACTACTGGGGCCAGGGCATC
>KF748752.1 Equus caballus clone IGVDJ55 immunoglobulin heavy chain variable region (IGH) mRNA, partial cds
ATGAGTCACCTGTGGTTCTTCCTCTTTCTGGTGGCCGCTCCTACATGTGTCCTGTCCCAGGTGCAACTGAAGGAGTC
AGGACCTGGCCTGGTGAAGCCCTCGCAGACCCTCTCCCTCACCTGCACTGTCTCTGGATTATCTTTGAGCAGTAATG
CTGTAGGCTGGGTCCGCCAGGCTCCAGGAAAAGGGCTGGAATTTGTTGGTGCTATATATGGTAGTGCAAGTGCAAAC
TACAACCCAGCCCTGAAGTCCCGAGCCAGCATCACCAAGGACACCTCAAAGAGCCAAGTTTATCTGACGCTGAACAG
CCTGACAAGCGAGGACACGGCCGTCTATTACTGTGCAGGAGCGCCGATAGCAGCTATGCTTACTACGTACTATTTTG
GCTACTGGGGCCAGGGCACC
>KF748750.1 Equus caballus clone IGVDJ53 immunoglobulin heavy chain variable region (IGH) mRNA, partial cds
ATGAGTCACCTGTGGTTCTTCCTCTTTCTGGTGGCCGCTCCTACATGTGTCCTGTCCCAGGTGCAACTGAAGGAGTC
AGGACCTGGCCTGGTGAAGCCCTCGCAGACCCTGTCCCTCACCTGCACTGTCTCTGGATTCTCTTTGAGCAGTTACG
GTGTAGGCTGGGTCCGCCAGGCTCCAGGAAAAGGGCTGGAATATGTTGGTGGTATAGCTAGTAGTGGAAGTGCAAAC
TACAACCCAGCCCTGAAGTCCCGAGCCAGCATCACCAAGGACACCTCAAAGAGCCAAGTTTATCTGACGCTGAACAG
CCTGACAGGCGAGGACACGGCCGTCTATTACTGTGCGAGTTTTCGATGTTATGGTTATGGTGGTGCTTACTACGCCT
ATGGCTACGTGGATCACTGGGGCCAGGGCACC
>KF748749.1 Equus caballus clone IGVDJ52 immunoglobulin heavy chain variable region (IGH) mRNA, partial cds
ATGAATCACCTGTGGTTCTTCCTCTTTCTGGTGGCCGCTCCTACATGTGTCCTGTCCCAGGTGCAACTGAAGGAGTC
GGGACCTGGCCTGGTGAAGCCCTCGCAGACCCTGTCCCTCACCTGCACTGTCTCTGGATTATCTTTGAGCAGTAATG
CTGTAGGCTGGGTCCGCCAGGCTCCAGGAAAAGGGCTGGAGTGGGTTGGTGTTATATATGGTAGTGAAAGTACATAC
TACAACCCAGCCCTGAAGTCCCGAGCCAGCATCACCAAGGACACCTCAAAGAGCCAAGTTTATCTGACGCTGAACAG
CCTGACAGGCGAAGACACGGCCGTCTATTACTGTGCAGGATGCTTGTATGACTGTACTGGTCATGGATGTGTCTACA
TAGATTATTATGGTATAAACTACTGGGGCCAGGGCATC
>KF748748.1 Equus caballus clone IGVDJ51 immunoglobulin heavy chain variable region (IGH) mRNA, partial cds
ATGAGGCTGTTGGGTCTTCTCCTTTGCCTGGTGACGGCTCCCCAAGGTGTCCTGTCCCAGGTGCAGCTGAAGGAGTC
GGGCCCAGGACAGGTGAAGCCCTCACAGACCCTCTCCCTCACCTGCACTGTCACTGGAGGCTCCATCACAAGCAGGT
ATTATGGCTGGAGCTGGATCCGCCAGACCCCAGGGAAGGGGCTGGAGTACATTGGGAGCATAGCTTATAGTGGTAGC
ACTTACTACAGCCCATCCCTCAAGAGCCACGCCTCCATCTCCAGAGACACGTCCAAGAACCAGTTCTCCCTGCAGCT
GAGCTCCGTGACCACCGAGGACACGGCCGTTTATTACTGTGCAAGTGTATATGGTTACTATGCTGGTAGTTACTATT
ATGGTATAAACTACTGGGGCCAGGGCATC
>KF748747.1 Equus caballus clone IGVDJ50 immunoglobulin heavy chain variable region (IGH) mRNA, partial cds
ATGAGGCTGTTGGGTCTTCTCCTTTGCCTGGTGACGGCTCCCCAAGGTGTCCTGTCCCAGGTGCAGCTGAAGGAGTC
GGGCCCAGGACAGGTGAAGCCCTCACAGACCCTCTCCCTCACCTGCACTGTCACTGGAGGCTCCATCACAAGCAGGT
ATTATGGCTGGAGCTGGATCCGCCAGACCCCAGGGAAGGGGCTGGAGTACATTGGGAGCATAGCTTATAGTGGTAGC
ACTTACTACAGCCCATCCCTCAAGAGCCACGCCTCCATCTCCAGAGACACGTCCAAGAACCAGTTCTCCCTGCAGCT
GAGCTCCGTGACCACCGAGGACACGGCCGTTTATTACTGTGCAAGTTACTATGGTGGTAGTTCCTGGTATTATTATG
GTATAAACTACTGGGGCCAGGGCATC
>KF748746.1 Equus caballus clone IGVDJ49 immunoglobulin heavy chain variable region (IGH) mRNA, partial cds
ATGAGGCTGTTGGGTCTTCTCCTTTGCCTGGTGACGGCTCCCCAAGGTGTCCTGTCCCAGGTGCAGCTGAAGGAGTC
GGGCCCAGGACAGGTGAAGCCCTCACAGACCCTCTCCCTCACCTGCACTGTCACTGGAGGCTCCATCACAAGCAGGT
ATTATGGCTGGAGCTGGATCCGCCAGACCCCAGGGAAGGGGCTGGAGTACATTGGGAGCATCACTTATAGTGGTAGC

ACTTACTACAGCCCATCCTTCAAGAGCCGCGCCTCCATCTCCAGAGACACGCCCAAGAACCAGTTCTCCCTGCAGCT
GAGCTCCGTGACCACAGAGGACACGGCCGTTTATTACTGTGCAAGTGTTAGCAGCTATGCTTATGGCTACTGGGGCC
AGGGCACC
>KF748745.1 Equus caballus clone IGVDJ48 immunoglobulin heavy chain variable region (IGH) mRNA, partial cds
ATGAGACTCTTGTGTCTTCTCCTTTTCCTGGTGACGGCTCCCCAAGGAGTCCTGTCCCAGGTGCAGCTGCAGGAGTC
GGGCCCAGGACTGGTGCAGCCCTCACAGACCCTGTCCCTCACCTGCACTGTCACTGGAGGCTCCATCACAAGCAGCT
ATTCTAGCTGGAGCTGGTTACGCCAGCCTCCAGGGAAGGGGCTGGAGTACATGGGGTACATATATTATGATGGTAGA
ACTTACTACAATCCTTCCTTCAAGAGCCGCACCTCCATCTCCAGAGACACCTCCAAGAACCAGTTCTCCCTGCAGCT
GAGCTCCGTGACCACCGAGGACGCGGCCGTGTATTACTGTGCAAGCTATAGCAGCTATGCTTATGGTATAGACTACT
GGGGCCAGGGCATC
>KF748744.1 Equus caballus clone IGVDJ47 immunoglobulin heavy chain variable region (IGH) mRNA, partial cds
ATGAGACTCTTGTGTCTTCTCCTTTTCCTGGTGACGGCTCCCCAAGGAGTCCTGTCCCAGGTGCAGCTGCAGGAGTC
GGGCCCAGGACTGGTGCAGCCCTCACAGACCCTGTCCCTCACCTGCACTGTCACTGGAGGCTCCATCACAAGCAGCT
ATTCTAGCTGGAGCTGGTTACGCCAGCCTCCAGGGAAGGGGCTGGAGTACATGGGGTACATATATTATGATGGTAGA
ACTTACTACAATCCTTCCTTCAAGAGCCGCACCTCCATCTCCAGAGACACCTCCAAGAACCAGTTCTCCCTGCAGCT
GAGCTCCGTGACCACCGAGGACGCGGCCGTGTATTACTGTGCAAGACATAGCAGCTATGCTTTTGGCTACTGGGGCC
AGGGCACC
>KF748743.1 Equus caballus clone IGVDJ46 immunoglobulin heavy chain variable region (IGH) mRNA, partial cds
ATGGGCTGGAGCTGGAGAATCCTCTTCTTGGTGGCAGTAGCTTCAGGTGTCTCCTCCGAGGGTCAGCTGGAACAGTC
GGGGCCGGAGTTGAAGAAGCCTGGGTCATCAGTGAAGATCTCCTGCAAGGCTTCTGGATACACCTTCAGTAGCTATG
CTGTGCACTGGGTGCGACAGGCCAATGGAAAAGGGATTGAGTGGATGGGATCTATCTATGCTGAATATGATGATACA
AGCTACGCACCGAAGTTCCAGGGCAGAGTCACCATGACTGCGGACAAGTCCACGAGCACAGTCTACATGGAGCTGAG
CAGTCTGACATCTGAGGACACGGCCGTGTATTACTGTGCAACACGGTTATGGTTATTTGGCTACTGGGGCCAGGGCA
CC
>KF748742.1 Equus caballus clone IGVDJ45 immunoglobulin heavy chain variable region (IGH) mRNA, partial cds
ATGAGTCACCTGTGGTTCTTCCTCTTTCTGGTGGCCGCTCCTACATGTGTCCTGTCCCAGGTGCAACTGAAGGAGTC
AGGACCTGGCCTGGTGAAGCCCTCGCAGACCCTCTCCCTCACCTGCACTGTCTCTGGATTATCTTTGAGCAGTAATG
CTGTAGGCTGGGTCCGCCAGGCTCCAGGAAAAGGGCTGGAATTTGTTGGTGCTATATATGGTAGTGCAAGTGCAAAC
TACAACCCAGCCCTGAAGTCCCGAGCCAGCATCACCAAGGACACCTCAAAGAGCCAAGTTTATCTGACGCTGAACAG
CCTGACAAGCGAGGACACGGCCGTCTATTACTGTGCAGGATACGGTTATGGTTATTATGATATAGACTACTGGGGCC
AGGGCACC
>KF748741.1 Equus caballus clone IGVDJ44 immunoglobulin heavy chain variable region (IGH) mRNA, partial cds
ATGAGTCACCTGTGGTTCTTCCTCTTTCTGGTGGCCGCTCCTACATGTGTCCTGTCCCAGGTGCAACTGAAGGAGTC
AGGACCTGGCCTGGTGAAGCCCTCGCAGACCCTCTCCCTCACCTGCACTGTCTCTGGATTATCTTTGAGCAGTAATG
CTGTAGGCTGGGTCCGCCAGGCTCCAGGAAAAGGGCTGGAATACGTTGGTGCTATATATGGTAGTGCAAGTGCAAAC
TACAACCCAGCCCTGAAGTCCCGAGCCAGCATCACCAAGGACACCTCAAAGAGCCAAGTTTATCTGACGCTGAACAG
CCTGACAAGCGAGGACACGGCCGTCTATTACTGTGCAGGATATGGTTCCTATGCTGGTAGTTACTATTATGGTATAG
ACTACTGGGGCCAGGGCATC
>KF748740.1 Equus caballus clone IGVDJ43 immunoglobulin heavy chain variable region (IGH) mRNA, partial cds
ATGAGTCACCTGTGGTTCTTCCTCTTTCTGGTGGCCGCTCCTACATGTGTCCTGTCCCAGGTGCAACTGAAGGAGTC
AGGACCTGGCCTGGTGAAGCCCTCGCAGACCCTCTCCCTCACCTGCACTGTCTCTGGATTATCTTTGAGCAGTAATG
CTGTAGGCTGGGTCCGCCAGGCTCCAGGAAAAGGGCTGGAATACGTTGGTGCTATATATGGTAGTGCAAGTGCAAAC
TACAACCCAGCCCTGAAGTCCCGAGCCAGCATCACCAAGGACACCTCAAAGAGCCAAGTTTATCTGACGCTGAACAG
CCTGACAAGCGAGGACACGGCCGTCTATTACTGTGCAGGATATGGTTACTATGCTAGTGGTTATGACTATTTTAGCT
ACTGGGGCCAGGGCACC
>KF748738.1 Equus caballus clone IGVDJ41 immunoglobulin heavy chain variable region (IGH) mRNA, partial cds
ATGAATCACCTGTGGTTCTTCCTCTTTCTGGTGGCCGCTCCTACATGTGTCCTGTCCCAGGTGCAACTGAAGGAGTC
AGGACCTGGCCTGGTGAAGCCCTCGCAGACCCTCTCCCTCACCTGCACTGTCTCTGGATTCTCTTTGAGCAGTTATG

CTGTAGGCTGGGTCCGCCAGGCTCCAGGAAAAGGGCTGGAATATGTTGGTGCTATATATGGTAGTGCAAGTGCAAAC
TACAACCCAGCCCTGAAGTCCCGAGCCAGCATCACCAAGGACACCTCAAAGAGCCAAGTTTATCTGACGCTGAACAG
CCTGACAGGCGAGGACACGGCCGTCTATTACTGTGCGAGATCCGCGGGTTATAACTACGGTTATGGTTATGGTATAA
ACTACTGGGGCCAGGGCAT
>KF748736.1 Equus caballus clone IGVDJ39 immunoglobulin heavy chain variable region (IGH) mRNA, partial cds
ATGAGTCACCTGTGGTTCTTCCTCTTTCTGGTGGCCGCTCCTACATGTGTCCTGTCCCAGGTGCAACTGAAGGAGTC
AGGACCTGGCCTGGTGAAGCCCTCGCAGACCCTCTCCCTCACCTGCACTGTCTCTGGATTATCTTTGAGCAGTAATG
CTGTAGGCTGGGTCCGCCAGGCTCCAGGAAAAGGGCTGGAATTTGTTGGTGCTATATATGGTAGTGCAAGTGCAAAC
TACAACCCAGCCCTGAAGTCCCGAGCCAGCATCACCAAGGACACCTCAAAGAGCCAAGTTTATCTGACGCTGAACAG
CCTGACAAGCGAGGACACGGCCGTCTATTACTGTGCAGGAGGCATTGGTGGTGCTTATTATTATGGTATAAACTACT
GGGGCCAGGGCATC
>KF748735.1 Equus caballus clone IGVDJ38 immunoglobulin heavy chain variable region (IGH) mRNA, partial cds
ATGAGTCACCTGTGGTTCTTCCTCTTTCTGGTGGCCGCTCCTACATGTGTCCTGTCCCAGGTGCAACTGAAGGAGTC
AGGACCTGGCCTGGTGAAGCCCTCGCAGACCCTCTCCCTCACCTGCACTGTCTCTGGATTATCTTTGAGTAGTTATG
GTGTGGGCTGGGTCCGCCAGGCTCCAGGAAAAGGGCTGGAATTTGTTGGTGGTATAGCTAGTAGTGGAAGTGCAAAC
TACAACCCAGCCCTGAAGTCCCGAGCCAGCATCACCAAGGACACCTCAAAGAGCCAAGTTTATCTGACGCTGAACAG
CCTGACAAGCGAGGACACGGCCGTCTATTACTGTGCAGGATATGAAGCTTTATATAGCAGCTATGCTTACTACGATG
GTATAAACTACTGGGGCCAGGGCATC
>KF748734.1 Equus caballus clone IGVDJ37 immunoglobulin heavy chain variable region (IGH) mRNA, partial cds
ATGAATCACCTGTGGTTCTTCCTCTTTCTGGTGGCCGCTCCTACATGTGTCCTGTCCCAGGTGCAACTGAAGGAGTC
AGGACCTGGCCTGGTGAAGCCCTCGCAGACCCTGTCCCTCACCTGCACTGTCTCTGGATTCTCTTTGAGCAGTTACG
GTGTAGGCTGGGTCCGCCAGGCTCCAGGAAAAGGGCTGGAATATGTTGGTGGTATAGCTAGTAGTGGAAGTGCAAAC
TACAACCCAGCCCTGAAGTCCCGAGCCAGCATCACCAAGGACACCTCAAAGAGCCAAGTTTATCTGACGCTGAACAG
CCTGACAGGCGAGGACACGGCCGTCTATTACTGTGCGAGATCTCGCGGTTATGGTATAAACTACTGGGGCCAGGGCA
TC
>KF748733.1 Equus caballus clone IGVDJ36 immunoglobulin heavy chain variable region (IGH) mRNA, partial cds
ATGAGTCACCTGTGGTTCTTCCTCTTTCTGGTGGCCGCTCCTACATGTGTCCTGTCCCAGGTGCAACTGAAGGAGTC
AGGACCTGGCCTGGTGAAGCCCTCGCAGACCCTCTCCCTCACCTGCACTGTCTCTGGATTATCTTTGAGTAGTTATG
GTGTGGGCTGGGTCCGCCAGGCTCCAGGAAAAGGGCTGGAATTTGTTGGTGGTATAGCTAGTAGTGGAAGTGCAAAC
TACAACCCAGCCCTGAAGTCCCGAGCCAGCATCACCAAGGACACCTCAAAGAGCCAAGTTTATCTGACGCTGAACAG
CCTGACAAGCGAGGACACGGCCGTCTATTACTGTGCAGGTTTAATTCCTATGCTGGTAGTTACTTATACTACATATT
ATTATGGTATAAACTACTGGGGCCAGGGCAT
>KF748732.1 Equus caballus clone IGVDJ35 immunoglobulin heavy chain variable region (IGH) mRNA, partial cds
ATGAGTCACCTGTGGTTCTTCCTCTTTCTGGTGGCCGCTCCTACATGTGTCCTGTCTCAGGTGCAACTGAAGGAGTC
AGGACCTGGCCTGGTGAAGCCCTCGCAGACCCTCTCCCTCACCTGCACTGTCTCTGGATTCTCTTTGAGCAGTTATG
CTGTAGGCTGGGTCCGCCAGGCTCCAGGAAAAGGGCTGGAATATGTTGGTGGTATAGCTAGTAGTGGAAGTGCAAAC
TACAACCCAGCCCTGAAGTCCCGAGCCAGCATCACCAAGGACACCTCAAAGAGCCAAGTTTATCTGACGCTGAACAG
CCTGACAAGCGAGGACACGGCCGTCTATTACTGTGCAGGAGGCGGAAGAGTTGGAGTAAGGGTTGGCTACTGGGGCC
AGGGCACC
>KF748731.1 Equus caballus clone IGVDJ34 immunoglobulin heavy chain variable region (IGH) mRNA, partial cds
ATGAGTCACCTGTGGTTCTTCCTCTTTCTGGTGGCCGCTCCTACATGTGTCCTGTCCCAGGTGCAACTGAAGGAGTC
AGGACCTGGCCTGGTGAAGCCCTCGCAGACCCTCTCCCTCACCTGCACTGTCTCTGGATTATCTTTGAGCAGTAATG
CTGTAGGCTGGGTCCGCCAGGCTCCAGGAAAAGGGCTGGAATTTGTTGGTGCTATATATGGTAGTGCAAGTGCAAAC
TACAACCCAGCCCTGAAGTCCCGAGCCAGCATCACCAAGGACACCTCAAAGAGCCAAGTTTATCTGACGCTGAACAG
CCTGACAAGCGAGGACACGGCCGTCTATTACTGTGCAGGAGGAATCTTCTATTACTATAGTAGGAGTTGCTACTATT
TTGGCTACTGGGGCCAGGGCACC
>KF748730.1 Equus caballus clone IGVDJ33 immunoglobulin heavy chain variable region (IGH) mRNA, partial cds
ATGAGTCACCTGTGGTTCTTCCTCTTTCTGGTGGCCGCTCCTACATGTGTCCTGTCCCAGGTGCAACTGAAGGAGTC
AGGACCTGGCCTGGTGAAGCCCTCGCAGACCCTCTCCCTCACCTGCACTGTCTCTGGATTATCTTTGAGCAGTTATG

GTGTGGGCTGGGTCCGCCAGGCTCCAGGAAAAGGGCTGGAATTTGTTGGTGGTATAGCTAGTAGTGGAAGTGCAAAC
TACAACCCAGCCCTGAAGTCCCGAGCCAGCATCACCAAGGACACCTCAAAGAGCCAAGTTTATCTGACGCTGAACAG
CCTGACAAGCGAGGACACGGCCGTCTATTACTGTGCAGGAGCTATAGCAGCTATGCTTGGCCACTGGGGCCAGGGCA
CC
>KF748728.1 Equus caballus clone IGVDJ31 immunoglobulin heavy chain variable region (IGH) mRNA, partial cds
ATGAATCACCTGTGGTTCTTCCTCTTTCTGGTGGCCGCTCCTACATGTGTCCTGTCCCAGGTGCAACTGAAGGAGTC
GGGACCTGGCCTGGTGAAGCCCTCGCAGACCCTGTCCCTCACCTGCACTGTCTCTGGATTATCTTTGAGCAGTAATG
CTGTAGGCTGGGTCCGCCAGGCTCCAGGAAAAGGGCTGGAGTGGGTTGGTGTTATATATGGTAGTGAAAGTACATAC
TACAACCCAGCCCTGAAGTCCCGAGCCAGCATCACCAAGGACACCTCAAAGAGCCAAGTTTATCTGACGCTGAACAG
CCTGACAGGCGAAGACACGGCCGTCTATTACTGTGCAGGATGGTCACGGTTATGGTTATATTATAATGCTATGGACC
CCTGGGGCCAGGGCACC
>KF748727.1 Equus caballus clone IGVDJ30 immunoglobulin heavy chain variable region (IGH) mRNA, partial cds
ATGAATCACCTGTGGTTCTTCCTCTTTCTGGTGGCCGCTCCTACATGTGTCCTGTCCCAGGTGCAACTGAAGGAGTC
AGGACCTGGCCTGGTGAAGCCCTCGCAGACCCTCTCCCTCACCTGCACTGTCTCTGGATTCTCTTTGAGCAGTTATG
CTGTATACTGGGTCCGCCAGGCTCCAGGAAAAGGGCTGGAATATGTTGGTGCTATATATGGTAGTGCAAGTGCAAAC
TACAACCCAGCCCTGAAGTCCCGAGCCAGCATCACCAAGGACACCTCAAAGAGCCAAGTTTATCTGACGCTGAACAG
CCTGACAGGCGAGGACACGGCCGTCTATTACTGTGCGAGTGACGGTAGCGGTTGGCCGGGGGGTATAAACTACTGGG
GCCAGGGCATC
>KF748726.1 Equus caballus clone IGVDJ29 immunoglobulin heavy chain variable region (IGH) mRNA, partial cds
ATGAATCACCTGTGGTTCTTCCTCTTTCTGGTGGCCGCTCCTACATGTGTCCTGTCCCAGGTGCAACTGAAGGAGTC
AGGACCTGGCCTGGTGAAGCCCTCGCAGACCCTCTCCCTCACCTGCACTGTCTCTGGATTCTCTTTGAGCAGTTATG
CTGTATACTGGGTCCGCCAGGCTCCAGGAAAAGGGCTGGAATATGTTGGTGCTATATATGGTAGTGCAAGTGCAAAC
TACAACCCAGCCCTGAAGTCCCGAGCCAGCATCACCAAGGACACCTCAAAGAGCCAAGTTTATCTGACGCTGAACAG
CCTGACAGGCGAGGACACGGCCGTCTATTACTGTGCGAGAACAGCTATGCTTACTATAAACTACTGGGGCCAGGGCA
TC
>KF748725.1 Equus caballus clone IGVDJ28 immunoglobulin heavy chain variable region (IGH) mRNA, partial cds
ATGAGTCACCTGTGGTTCTTCCTCTTTCTGGTGGCCGCTCCTACATGTGTCCTGTCCCAGGTGCAACTGAAGGAGTC
AGGACCTGGCCTGGTGAAGCCCTCGCAGACCCTGTCCCTCACCTGCACTGTCTCTGGATTCTCTTTGAGCAGTTACG
GTGTAGGCTGGGTCCGCCAGGCTCCAGGAAAAGGGCTGGAATATGTTGGTGGTATAGCTAGTAGTGGAAGTGCAAAC
TACAACCCAGCCCTGAAGTCCCGAGCCAGCATCACCAAGGACACCTCAAAGAGCCAAGTTTATCTGACGCTGAACAG
CCTGACAGGCGAGGACACGGCCGTCTATTACTGTGCGAGATCAGGACAAAGAGTTGGAGTAAAGAGTTACTGGGGCC
AGGGCATC
>KF748724.1 Equus caballus clone IGVDJ27 immunoglobulin heavy chain variable region (IGH) mRNA, partial cds
ATGAGTCACCTGTGGTTCTTCCTCTTTCTGGTGGCCGCTCCTACATGTGTCCTGTCCCAGGTGCAACTGAAGGAGTC
AGGACCTGGCCTGGTGAAGCCCTCGCAGACCCTCTCCCTCACCTGCACTGTCTCTGGATTATCTTTGAGCAGTAATG
CTGTAGGCTGGGTCCGCCAGGCTCCAGGAAAAGGGCTGGAATTTGTTGGTGCTATATATGGTAGTGCAAGTGCAAAC
TACAACCCAGCCCTGAAGTCCCGAGCCAGCATCACCAAGGACACCTCAAAGAGCCAAGTTTATCTGACGCTGAACAG
CCTGACAAGCGAGGACACGGCCGTCTATTACTGTGCAGGATACGGTTATGTTTATGGTATAAACTACTGGGGCCAGG
GCATC
>KF748723.1 Equus caballus clone IGVDJ26 immunoglobulin heavy chain variable region (IGH) mRNA, partial cds
ATGAGTCACCTGTGGTTCTTCCTCTTTCTGGTGGCCGCTCCTACATGTGTCCTGTCCCAGGTGCAACTGAAGGAGTC
AGGACCTGGCCTGGTGAAGCCCTCGCAGACCCTCTCCCTCACCTGCACTGTCTCTGGATTATCTTTGAGCAGTAATG
CTGTAGGCTGGGTCCGCCAGGCTCCAGGAAAAGGGCTGGAATATGTTGGTGGTATAGCTAGTAGTGGAAGTGCAAAC
TACAACCCAGCCCTGAAGTCCCGAGCCAGCATCACCAAGGACACCTCAAAGAGCCAAGTTTATCTGACGCTGAACAG
CCTGACAAGCGAGGACACGGCCGTCTATTACTGTGCAGGAATCTACGGTTATGGTTATCGGCTGGGTTATTATGGTA
TAAACTACTGGGGCCAGGGCATC
>KF748722.1 Equus caballus clone IGVDJ25 immunoglobulin heavy chain variable region (IGH) mRNA, partial cds

ATGAGTCACCTGTGGTTCTTCCTCTTTCTGGTGGCCGCTCCTACATGTGTCCTGTCTCAGGTGCAACTGAAGGAGTC
AGGACCTGGCCTGGTGAAGCCCTCGCAGACCCTCTCCCTCACCTGCACTGTCTCTGGATTATCTTTGAGCAGTAATG
CTGTAGGCTGGGTCCGCCAGGCTCCAGGAAAAGGGCTGGAATATGTTGGTGGTATATATGGTAGTGCAAGTGCAAAC
TACAACCCAGCCCTGAAGTCCCGAGCCAGCATCACCAAGGACACCTCAAAGAGCCAAGTTTATCTGACGCTGAACAG
CCTGACAAGCGAGGACACGGCCGTCTATTACTGTGCAGGAGGCGAGGCACAGGATAGTTATGGTAGTTACTATGGTA
TAAACTACTGGGGCCAGGGCATC
>KF748721.1 Equus caballus clone IGVDJ24 immunoglobulin heavy chain variable region (IGH) mRNA, partial cds
ATGAATCACCTGTGGTTCTTCCTCTTTCTGGTGGCCGCTCCTACATGTGTCCTGTCCCAGGTGCAACTGAAGGAGTC
AGGACCTGGCCTGGTGAAGCCCTCGCAGACCCTCTCCCTCACCTGCACTGTCTCTGGATTCTCTTTGAGCAGTTATG
CTGTATACTGGGTCCGCCAGGCTCCAGGAAAAGGGCTGGAATATGTTGGTGCTATATATGGTAGTGCAAGTGCAAAC
TACAACCCAGCCCTGAAGTCCCGAGCCAGCATCACCAAGGACACCTCAAAGAGCCAAGTTTATCTGACGCTGAACAG
CCTGACAGGCGAGGACACGGCCGTCTATTACTGTGCGAGATCGGAGGGCCCCTCGTTCCCCACACAATTCCTGAGTT
ATGGTAGTTACTATGGTATAAACTACTGGGGCCAGGGCATC
>KF748720.1 Equus caballus clone IGVDJ23 immunoglobulin heavy chain variable region (IGH) mRNA, partial cds
ATGAGTCACCTGTGGTTCTTCCTCTTTCTGGTGGCCGCTCCTACATGTGTCCTGTCCCAGGTGCAACTGAAGGAGTC
AGGACCTGGCCTGGTGAAGCCCTCGCAGACCCTCTCCCTCACCTGCACTGTCTCTGGATTATCTTTGAGCAGTAATG
CTGTAGGCTGGGTCCGCCAGGCTCCAGGAAAAGGGCTGGAATATGTTGGTGGTATAGCTAGTAGTGGAAGTGCAAAC
TACAACCCAGCCCTGAAGTCCCGAGCCAGCATCACCAAGGACACCTCAAAGAGCCAAGTTTATCTGACGCTGAACAG
CCTGACAAGCGAGGACACGGCCGTCTATTACTGTGCAGGAGGCTGGTATAACTACGGTTATGGTTATGCCCATTTTG
GCTACTGGGGCCAGGGCACC
>KF748719.1 Equus caballus clone IGVDJ22 immunoglobulin heavy chain variable region (IGH) mRNA, partial cds
ATGAGTCACCTGTGGTTCTTCCTCTTTCTGGTGGCCGCTCCTACATGTGTCCTGTCCCAGGTGCAACTGAAGGAGTC
AGGACCTGGCCTGGTGAAGCCCTCGCAGACCCTCTCCCTCACCTGCACTGTCTCTGGATTATCTTTGAGTAGTTATG
GTGTGGGCTGGGTCCGCCAGGCTCCAGGAAAAGGGCTGGAATTTGTTGGTGGTATAGCTAGTAGTGGAAGTGCAAAC
TACAACCCAGCCCTGAAGTCCCGAGCCAGCATCACCAAGGACACCTCAAAGAGCCAAGTTTATCTGACGCTGAACAG
CCTGACAAGCGAGGACACGGCCGTCTATTACTGTGCAGGATGTGCTATAGCAGCTATGCTTGGCTACTGGGGCCAGG
GCACC
>KF748718.1 Equus caballus clone IGVDJ21 immunoglobulin heavy chain variable region (IGH) mRNA, partial cds
ATGAGTCACCTGTGGTTCTTCCTCTTTCTGGTGGCCGCTCCTACATGTGTCCTGTCTCAGGTGCAACTGAAGGAGTC
AGGACCTGGCCTGGTGAAGCCCTCGCAGACCCTCTCCCTCACCTGCACTGTCTCTGGATTATCTTTGAGCAGTAATG
CTGTAGGCTGGGTCCGCCAGGCTCCAGGAAAAGGGCTGGAATATGTTGGTGGTATAGCTAGTAGTGGAAGTGCAAAC
TACAACCCAGCCCTGAAGTCCCGAGCCAGCATCACCAAGGACACCTCAAAGAGCCAAGTTTATCTGACGCTGAACAG
CCTGACAAGCGAGGACACGGCCGTCTATTACTGTGCAGGATTCTACGGTTATGGTTATGCTACCTATTTTGGCTACT
GGGGCCAGGGCACC
>KF748717.1 Equus caballus clone IGVDJ20 immunoglobulin heavy chain variable region (IGH) mRNA, partial cds
ATGAGTCACCTGTGGTTCTTCCTCTTTCTGGTGGCCGCTCCTACATGTGTCCTGTCCCAGGTGCAACTGAAGGAGTC
AGGACCTGGCCTGGTGAAGCCCTCGCAGACCCTCTCCCTCACCTGCACTGTCTCTGGATTATCTTTGAGCAGTAATG
CTGTAGGCTGGGTCCGCCAGGCTCCAGGAAAAGGGCTGGAATATGTTGGTGGTATAGCTAGTAGTGGAAGTGCAAAC
TACAACCCAGCCCTGAAGTCCCGAGCCAGCATCACCAAGGACACCTCAAAGAGCCAAGTTTATCTGACGCTGAACAG
CCTGACAAGCGAGGACACGGCCGTCTATTACTGTGCAGGAGATCTTATCGACTTATATGGTAGTAGTTCCTGGTACT
GTATAGACTACTGGGGCCAGGGCATC
>KF748716.1 Equus caballus clone IGVDJ19 immunoglobulin heavy chain variable region (IGH) mRNA, partial cds
ATGAATCACCTGTGGTTCTTCCTCTTTCTGGTGGCCGCTCCTACATGTGTCCTGTCCCAGGTGCAACTGAAGGAGTC
AGGACCTGGCCTGGTGAAGCCCTCGCAGACCCTCTCCCTCACCTGCACTGTCTCTGGATTATCTTTGAGCAGTTATG
CTGTAGGCTGGGTCCGCCAGGCTCCAGGAAAAGGGCTGGAATATGTTGGTGCTATATATGGTAGTGCAAGTGCAAAC
TACAACCCAGCCCTGAAGTCCCGAGCCAGCATCACCAAGGACACCTCAAAGAGCCAAGTTTATCTGACGCTGAACAG
CCTGACAGGCGAGGACACGGCCGTCTATTACTGTGCGAGATATAGCAGCTATGCTTATGGTATAGACTACTGGGGCC
AGGGCATC
>KF748715.1 Equus caballus clone IGVDJ18 immunoglobulin heavy chain variable region (IGH) mRNA, partial cds

ATGAATCACCTGTGGTTCTTCCTCTTTCTGGTGGCCGCTCCTACATGTGTCCTGTCCCAGGTGCAACTGAAGGAGTC
AGGACCTGGCCTGGTGAAGCCCTCGCAGACCCTCTCCCTCACCTGCACTGTCTCTGGATTATCTTTGAGCAGTTATG
CTGTAGGCTGGGTCCGCCAGGCTCCAGGAAAAGGGCTGGAATATGTTGGTGCTATATATGGTAGTGCAAGTGCAAAC
TACAACCCAGCCCTGAAGTCCCGAGCCAGCATCACCAAGGACACCTCAAAGAGCCAAGTTTATCTGACGCTGAACAG
CCTGACAGGCGAGGACACGGCCGTCTATTACTGTGCGAGATGGGTATGGTATGGTATAGACTACTGGGGCCAGGGCA
TC
>KF748713.1 Equus caballus clone IGVDJ16 immunoglobulin heavy chain variable region (IGH) mRNA, partial cds
ATGAGTCACCTGTGGTTCTTCCTCTTTCTGGTGGCCGCTCCTACATGTGTCCTGTCCCAGGTGCAACTGAAGGAGTC
AGGACCTGGCCTGGTGAAGCCCTCGCAGACCCTCTCCCTCACCTGCACTGTCTCTGGATTATCTTTGAGCAGTAATG
CTGTAGGCTGGGTCCGCCAGGCTCCAGGAAAAGGGCTGGAATACGTTGGTGCTATATATGGTAGTGCAAGTGCAAAC
TACAACCCAGCCCTGAAGTCCCGAGCCAGCATCACCAAGGACACCTCAAAGAGCCAAGTTTATCTGACGCTGAACAG
CCTGACAAGCGAGGACACGGCCGTCTATTACTGTGCAGGAGGCAGCTATGCTTATGGTATAGACTACTGGGGCCAGG
GCATC
>KF748712.1 Equus caballus clone IGVDJ15 immunoglobulin heavy chain variable region (IGH) mRNA, partial cds
ATGAATCACCTGTGGTTCTTCCTCTTTCTGGTGGCCGCTCCTACATGTGTCCTGTCCCAGGTGCAACTGAAGGAGTC
AGGACCTGGCCTGGTGAAGCCCTCGCAGACCCTCTCCCTCACCTGCACTGTCTCTGGATTATCTTTGAGCAGTAATG
CTGTAGGCTGGGTCCGCCAGGCTCCAGGAAAAGGGCTGGAATACGTTGGTGCTATATATGGTAGTGCAAGTGCAAAC
TACAACCCAGCCCTGAAGTCCCGAGCCAGCATCACCAAGGACACCTCAAAGAGCCAAGTTTATCTGACGCTGAACAG
CCTGACAAGCGAGGACACGGCCGTCTATTACTGTGCAGGAATAGTAGTTCCTGGTACTTATGGTATAGACTACTGGG
GCCAGGGCATC
>KF748711.1 Equus caballus clone IGVDJ14 immunoglobulin heavy chain variable region (IGH) mRNA, partial cds
ATGAGTCACCTGTGGTTCTTCCTCTTTCTGGTGGCCGCTCCTACATGTGTCCTGTCCCAGGTGCAACTGAAGGAGTC
AGGACCTGGCCTGGTGAAGCCCTCGCAGACCCTCTCCCTCACCTGCACTGTCTCTGGATTATCTTTGAGCAGTAATG
CTGTAGGCTGGGTCCGCCAGGCTCCAGGAAAAGGGCTGGAATACGTTGGTGCTATATATGGTAGTGCAAGTGCAAAC
TACAACCCAGCCCTGAAGTCCCGAGCCAGCATCACCAAGGACACCTCAAAGAGCCAAGTTTATCTGACGCTGAACAG
CCTGACAAGCGAGGACACGGCCGTCTATTACTGTGCAGGATATGGTTACTATGCTAGTGGTTATGACTATTTTAGCT
ACTGGGGCCAGGGCACC
>KF748710.1 Equus caballus clone IGVDJ13 immunoglobulin heavy chain variable region (IGH) mRNA, partial cds
ATGAATCACCTGTGGTTCTTCCTCTTTCTGGTGGCCGCTCCTACATGTGTCCTGTCCCAGGTGCAACTGAAGGAGTC
AGGACCTGGCCTGGTGAAGCCCTCGCAGACCCTCTCCCTCACCTGCACTGTCTCTGGATTATCTTTGAGCAGTTATG
CTGTAGGCTGGGTCCGCCAGGCTCCAGGAAAAGGGCTGGAATATGTTGGTGCTATATATGGTAGTGCAAGTGCAAAC
TACAACCCAGCCCTGAAGTCCCGAGCCAGCATCACCAAGGACACCTCAAAGAGCCAAGTTTATCTGACGCTGAACAG
CCTGACAGGCGAGGACACGGCCGTCTATTACTGTGCGAGATATGGTAGTTACTACAGTAGTTACTATGGCTACGTGG
ATCACTGGGGCCAGGGCACC
>KF748709.1 Equus caballus clone IGVDJ12 immunoglobulin heavy chain variable region (IGH) mRNA, partial cds
ATGAATCACCTGTGGTTCTTCCTCTTTCTGGTGGCCGCTCCTACATGTGTCCTGTCCCAGGTGCAACTGAAGGAGTC
GGGACCTGGCCTGGTGAAGCCCTCGCAGACCCTGTCCCTCACCTGCACTGTCTCTGGATTATCTTTGAGCAGTAATG
CTGTAGGCTGGGTCCGCCAGGCTCCAGGAAAAGGGCTGGAGTGGGTTGGTGTTATATATGGTAGTGAAAGTACATAC
TACAACCCAGCCCTGAAGTCCCGAGCCAGCATCACCAAGGACACCTCAAAGAGCCAAGTTTATCTGACGCTGAACAG
CCTGACAGGCGAAGACACGGCCGTCTATTACTGTGCAGGATATAGCTATAGCAGCTATGCTTACTATTATGGTATAA
ACTACTGGGGCCAGGGCATC
>KF748704.1 Equus caballus clone IGVDJ7 immunoglobulin heavy chain variable region (IGH) mRNA, partial cds
ATGAGTCACCTGTGGTTCTTCCTCTTTCTGGTGGCCGCTCCTACATGTGTCCTGTCCCAGGTGCAACTGAAGGAGTC
AGGACCTGGCCTGGTGAAGCCCTCGCAGACCCTGTCCCTCACCTGCACTGTCTCTGGATTCTCTTTGAGCAGTTACG
GTGTAGGCTGGGTCCGCCAGGCTCCAGGAAAAGGGCTGGAATATGTTGGTGGTATAGCTAGTAGTGGAAGTGCAAAC
TACAACCCAGCCCTGAAGTCCCGAGCCAGCATCACCAAGGACACCTCAAAGAGCCAAGTTTATCTGACGCTGAACAG
CCTGACAGGCGAGGACACGGCCGTCTATTACTGTGCGAGATCCTACTACGGTTATGGTTATGCTTATGGTATAAACT
ACTGGGGCCAGGGCATC

>KF748703.1 Equus caballus clone IGVDJ6 immunoglobulin heavy chain variable region (IGH) mRNA, partial cds
ATGAGTCACCTGTGGTTCTTCCTCTTTCTGGTGGCCGCTCCTACATGTGTCCTGTCTCAGGTGCAACTGAAGGAGTC
AGGACCTGGCCTGGTGAAGCCCTCGCAGACCCTCTCCCTCACCTGCACTGTCTCTGGATTATCTTTGAGCAGTAATG
CTGTAGGCTGGGTCCGCCAGGCTCCAGGAAAAGGGCTGGAATATGTTGGTGGTATAGCTAGTAGTGGAAGTGCAAAC
TACAACCCAGCCCTGAAGTCCCGAGCCAGCATCACCAAGGACACCTCAAAGAGCCAAGTTTATCTGACGCTGAACAG
CCTGACAAGCGAGGACACGGCCGTCTATTACTGTGCAGGGTGGAATCTACGGTTATGGTCAATGGGGCATTTTGGCT
ACTGGGGCCAGGGCACC
>KF748702.1 Equus caballus clone IGVDJ5 immunoglobulin heavy chain variable region (IGH) mRNA, partial cds
ATGAGTCACCTGTGGTTCTTCCTCTTTCTGGTGGCCGCTCCTACATGTGTCCTGTCCCAGGTGCAACTGAAGGAGTC
AGGACCTGGCCTGGTGAAGCCCTCGCAGACCCTCTCCCTCACCTGCACTGTCTCTGGATTATCTTTGAGCAGTAATG
CTGTAGGCTGGGTCCGCCAGGCTCCAGGAAAAGGGCTGGAATTTGTTGGTGCTATATATGGTAGTGCAAGTGCAAAC
TACAACCCAGCCCTGAAGTCCCGAGCCAGCATCACCAAGGACACCTCAAAGAGCCAAGTTTATCTGACGCTGAACAG
CCTGACAAGCGAGGACACGGCCGTCTATTACTGTGCAGGAAGAGATAACTACGGTTATGGTTATGCTACGTACTATT
TTGGCTACTGGGGCCAGGGCACC
>KF748701.1 Equus caballus clone IGVDJ4 immunoglobulin heavy chain variable region (IGH) mRNA, partial cds
ATGAGTCACCTGTGGTTCTTCCTCTTTCTGGTGGCCGCTCCTACATGTGTCCTGTCTCAGGTGCAACTGAAGGAGTC
AGGACCTGGCCTGGTGAAGCCCTCGCAGACCCTCTCCCTCACCTGCACTGTCTCTGGATTATCTTTGAGCAGTAATG
CTGTAGGCTGGGTCCGCCAGGCTCCAGGAAAAGGGCTGGAATATGTTGGTGGTATAGCTAGTAGTGGAAGTGCAAAC
TACAACCCAGCCCTGAAGTCCCGAGCCAGCATCACCAAGGACACCTCAAAGAGCCAAGTTTATCTGACGCTGAACAG
CCTGACAAGCGAGGACACGGCCGTCTATTACTGTGCAGGAGGTCGGAACCACTTACACCCGCAGAAAAAGCACTATG
GTGGTAGTTCCTGGTACTATTTTGGCTACTGGGGCCAGGGCACC
>KF748700.1 Equus caballus clone IGVDJ3 immunoglobulin heavy chain variable region (IGH) mRNA, partial cds
ATGAGTCACCTGTGGTTCTTCCTCTTTCTGGTGGCCGCTCCTACATGTGTCCTGTCCCAGGTGCAACTGAAGGAGTC
AGGACCTGGCCTGGTGAAGCCCTCGCAGACCCTCTCCCTCACCTGCACTGTCTCTGGATTATCTTTGAGCAGTAATG
CTGTAGGCTGGGTCCGCCAGGCTCCAGGAAAAGGGCTGGAATTTGTTGGTGCTATATATGGTAGTGCAAGTGCAAAC
TACAACCCAGCCCTGAAGTCCCGAGCCAGCATCACCAAGGACACCTCAAAGAGCCAAGTTTATCTGACGCTGAACAG
CCTGACAAGCGAGGACACGGCCGTCTATTACTGTGCAGGAGTTAATGGCGACTACGGTTATGGTTATGCTTATGGCT
ACGTGGATCACTGGGGCCAGGGCACC
>KY437667.1 Equus caballus clone IGHG mRNA clone 24 immunoglobulin mu heavy chain G (IGHM) mRNA, partial cds
CTCTCCCTCACCTGCACTGTCTCTGGATCATCTTTGAGTGCGTATGCAGTGGGCTGGGTCCGCCAGGCTCCCGGAAA
AGGGCTGGAATTTGTTGGCGGGTTGTCTAGAAAGGGAGGAACACTCTACAACCCAGCCCTGAAGTCCCGAGCCACTA
TCACCAGGGACACCTCAAAGAATGAAGTTTATCTGACGATGGACAGCCTGACAAGCGAGGACACGGCCGTCTATTAC
TGTGCTGGAAGTTCGTGGAATGGTTATTGGGTTGGATTAATAGAGGATTGGGGCCAGGGCACCCTGGTCACCGTCTC
CTCAGCCTCCACCACGCCCCCGAAGGTCTTCCAGCTGGCCTCACACTCTGCGGGCACATCTGACTCCACGGTGGCCC
TGGGCTGCCTGGTCTCCAGCTACATCCCAGAGCCGGTGACCGTGTCCTGGAACTCAGGCACGCTGACCAGCGGCGTG
CACACCTTCCCATCTGTCCGGCAGTCCTCGGGGCTCTACTC
>KY437666.1 Equus caballus clone IGHG mRNA clone 23 immunoglobulin mu heavy chain G (IGHM) mRNA, partial cds
CTCTCCCTCACCTGCACTGTCTCTAAATTATCTTTGAGCAGTAATGCTGTAGGCTGGGTCCGCCAGGCTCCAGGAAA
AGGGCTGGAATATGTTGGTGATATAAGTGCTGCTGGAAGTGCAAACTACAACCCAGCCCTGAAGTCCCGAGCCAGCA
TCACCAAGGACACCTCAAAGAGCCAAGTTTATCTGACGCTGAACAGCCTGACAATTGAGGACACGGCCGTCTATTAC
TGTCTAGGAGGCGGCAACAACTGGCTTGTCTACGCCATCCATTATTATGGTATAAACTACTGGGGCCAGGGCATCCT
GGTCACCGTCTCCTCAGCCTCCACCACCGCCCCGAAGGTCTTCGCGCTGGCCCCCGGCTGTGGGACCACATCTGACT
CCACGGTGGCCCTGGGCTGCCTTGTCTCCGGATACTTCCCCGAGCCAGTGAAGGTGTCCTGGAACTCGGGCTCCCTG
ACCAGTGGCGTGCACACCTTCCCTTCCGTCCTGCAGTCCTCAGGGTTCTACTC
>KY437665.1 Equus caballus clone IGHG mRNA clone 22 immunoglobulin mu heavy chain G (IGHM) mRNA, partial cds
CTGTCCCTCACCTGCACTGTCTCTGGATTATCTTTGAGCAGTAATGATGTGGTCTGGGTCCGCCAGGCTCCAGGAAA
GGGGCTGGAGTGGATTGGTAAGATAAGTAGCAGTGAAAGTACATACTACAACCCAGCCCTGAAGTCCCGAGCCAGCA
TCACCAAGGACACCTCAAAGAGCCAAGTTTATCTGACGCTGAACAGCCTGACAGGCGAAGACACGGCCGTCTATTAC
TGTAAAGGACCACGACTGTCCAATGGTATGGACCCCTGGGGCCAGGGCACCCTGGTCACCGTCTCCTCAGCCTCCAC

CACCGCCCCGAAGGTCTTCGCGCTGGCCCCCGGCTGTGGGACCACATCTGACTCCACGGTGGCCCTGGGCTGCCTTG
TCTCCGGATACTTCCCCGAGCCAGTGAAGGTGTCCTGGAACTCGGGCTCCCTGACCAGTGGCGTGCACACCTTCCCT
TCCGTCCTGCAGTCCTCAGGGTTCTACTC
>KY437664.1 Equus caballus clone IGHG mRNA clone 21 immunoglobulin mu heavy chain G (IGHM) mRNA, partial cds
CTCTCCCTCACCTGCACTGTCTCTAAATTATCTTTGAGCAGTAATGCTGTAGGCTGGGTCCGCCAGGCTCCAGGAAA
AGGGCTGGAATATGTTGGTGATATAAGTGCTGCTGGAAGTGCAAACTACAACCCAGCCCTGAAGTCCCGAGCCAGCA
TCACCAAGGACACCTCAAAGAGCCAAGTTTATCTGACGCTGAACAGCCTGACAATTGAGGACACGGCCGTCTATTAC
TGTCTAGGAGGCGGCAACAACTGGCTTGTCTACGCCATCCATTATTATGGTATAAACTACTGGGGCCAGGGCATCCT
GGTCACCGTCTCCTCAGCCTCCACCACCGCCCCGAAGGTCTTCGCGCTGGCCCCCGGCTGTGGGACCACATCTGACT
CCACGGTGGCCCTGGGCTGCCTTGTCTCCGGATACTTCCCCGAGCCAGTGAAGGTGTCCTGGAACTCGGGCTCCCTG
ACCAGTGGCGTGCACACCTTCCCTTCCGTCCTGCAGTCCTCAGGGTTCTACTC
>KY437663.1 Equus caballus clone IGHG mRNA clone 20 immunoglobulin mu heavy chain G (IGHM) mRNA, partial cds
CTCTCCCTCACCTGCACTGTCTCTGGATTGTCTTTGAGCAGTTATGCTGTATACTGGGTCCGCCAGGCTCCAGGAAA
AGGGCTGGAATATGTCGGTGCTATAGGTATCAGAGGACGTGCAAACTACAACCCAGCCCTGAAGTCCCGAGCCAGCA
TCACCAAGGACACCTCCAAGAGCCAAGTACATCTGACGCTGAACAGCCTGACAGGCGAGGACACGGCCGTCTATTAC
TGTGCGAAAGATGGTTATGGTGGTGCTTACTACCCTTATGGCTACGTGAGCCACTGGGGCCAGGGCACCCTGGTCAC
CGTCTCCTCAGAGAGCCCTAAGGCCCCAGACGTCTTCCCGCTGACCATCTGTGGGAACACACCTGACCCCACGGTGC
CCGTGGGCTGCCTGGTCTCCAACTACTTCCCAGAGCCAGTGACCGTGTCCTGGAACTGTGATGCCCTGAAAGGCGAC
ATACACACCTTTCCGCTGGACCTGAGCAACTCGGCTCACCACTC
>KY437662.1 Equus caballus clone IGHG mRNA clone 19 immunoglobulin mu heavy chain G (IGHM) mRNA, partial cds
CTCTCCCTCACCTGCACTGTCTCTGGATTGTCTTTGAGCAGTTATGCTGTATACTGGGTCCGCCAGGCTCCAGGAAA
AGGGCTGGAATATGTCGGTGCTATAGGTATCAGAGGACGTGCAAACTACAACCCAGCCCTGAAGTCCCGAGCCAGCA
TCACCAAGGACACCTCCAAGAGCCAAGTACATCTGACGCTGAACAGCCTGACAGGCGAGGACACGGCCGTCTATTAC
TGTGCGAAAGATGGTTATGGTGGTGCTTACTACCCTTATGGCTACGTGAGCCACTGGGGCCAGGGCACCCTGGTCAC
CGTCTCCTCAGAGAGCCCTAAGGCCCCAGACGTCTTCCCGCTGACCATCTGTGGGAACACACCTGACCCCACGGTGC
CCGTGGGCTGCCTGGTCTCCAACTACTTCCCAGAGCCAGTGACCGTGTCCTGGAACTGTGATGCCCTGAAAGGCGAC
ATACACACCTTTCCGCTGGACCTGAGCAACTCGGCTCACCACTC
>KY437661.1 Equus caballus clone IGHG mRNA clone 18 immunoglobulin mu heavy chain G (IGHM) mRNA, partial cds
CTCTCCCTCACCTGCACTGTCTCTGGATTCTCTTTGAGCAGTTACGGTGTAGGCTGGGTCCGCCAGGCTCCAGGAAA
AGGGCTGGAATTTGTTGGTGATATACCTAGTAGTGGAAGTGCAAACTACAACCCAGCCCTGAAGTCCCGAGCCAGCA
TCACCAAGGACACCTCAAAGAGCCAAGTTTATCTGACGCTGAACAGCCTGACGACCGAGGACACGGCCGTCTATTAC
TGTGCAGGAGCGGGTGTTTATGACAGTTACAACAGCAATTACAATGTTTATGGCTACGTGCGTCATTGGGGCCAGGG
CACCCTGGTCACCGTCTCCTCCGCCTCCACCACCGCCCCGAAGGTCTTCGCGCTGGCCCCCGGCTGTGGGACCACAT
CTGACTCCACGGTGGCCCTGGGCTGCCTTGTCTCCGGATACTTCCCCGAGCCAGTGAAGGTGTCCTGGAACTCGGGC
TCCCTGACCAGTGGCGTGCACACCTTCCCTTCCGTCCTGCAGTCCTCAGGGTTCTACTC
>KY437660.1 Equus caballus clone IGHG mRNA clone 17 immunoglobulin mu heavy chain G (IGHM) mRNA, partial cds
CTGTCCCTCACCTGCACTGTCTCTGGATTATCTTTGAGCAGTAATGGTGTCGGCTGGGTCCGCCAGGCTCCAGGAAA
AGGGCTGGAGTGGGTTGGTGTTATAAATGGGCGTGAAAGTACTTTCTACAACCCAGCCCTGAAGTCCCGAGCCAGCA
TCACCAAGGACACCTCAAAGAGTCAAGTTTATCTGACGCTGAACAGCCTGACAGGCGAAGACACGGCCGTCTATTAC
TGTGCGGGGACTATACTAGGAGTTGCCGTGAACTGGGGCCAGGGCACCCTGGTCACCGTCTCCTCAGCCTCCACCAC
CGCCCCGAAGGTCTTCGCGCTGGCCCCCGGCTGTGGGACCACATCTGACTCCACGGTGGCCCTGGGCTGCCTTGTCT
CCGGATACTTCCCCGAGCCAGTGAAGGTGTCCTGGAACTCGGGCTCCCTGACCAGTGGCGTGCACACCTTCCCTTCC
GTCCTGCAGTCCTCAGGGTTCTACTC
>KY437659.1 Equus caballus clone IGHG mRNA clone 16 immunoglobulin mu heavy chain G (IGHM) mRNA, partial cds
CTCTCCCTCACCTGCACTGTCTCTGGATTCTCTTTGAGCAGTTATCATGTAGGCTGGGTCCGCCAGGCTCCAGGAAG
AGGGCTGGAATTTGTTGGTTCACAAAGCGGTAACGGGGAAAGTGTAAAGTATAATCCAGCCCTGAAGTCCCGAGCCA
AAATCACCAAGGACACCTCCAAGAGTCAAGTTTATCTGACGTTGAGCAGCCTGACAAGCGAGGACACGGCCGTCTAT
TTCTGTGCGCAGCACGGTTATGGTGTGCGGCGGGCATATTGGTATGGTATAAAGTATTGGGGCCAGGGCATCCTGGT
CACCGTCTCCTCAGCCTCCACCACCGCCCCGAAGGTCTTCGCGCTGGCCCCCGGCTGTGGGACCACATCTGACTCCA

CGGTGGCCCTGGGCTGCCTTGTCTCCGGATACTTCCCCGAGCCAGTGAAGGTGTCCTGGAACTCGGGCTCCCTGACC
AGTGGCGTGCACACCTTCCCTTCCGTCCTGCAGTCCTCAGGGTTCTACTCCCTCAGCAGCATGGTTACATCT
>KY437658.1 Equus caballus clone IGHG mRNA clone 15 immunoglobulin mu heavy chain G (IGHM) mRNA, partial cds
CTGTCCCTCACCTGCACTGTCTCTGGATTCTCTTTGAGCAACTACGGTGTGAACTGGGTCCGCCAGGCTCCAGGAAA
AGGGCTGGAAGTAGTTGGTACTATAGGTAAAGGTGGCAGTGCAACGTACAACCCAGCCCTGAGGTCCCGAGCCAGCA
TCACTAGGGACACCTCAAAGAGCCAAGTTTATCTGACGCTGAACAGCCTGACAGGCGAGGACACGGCCGTCTATTAC
TGCTGGAGGAGTTCGAACTACTGGGGCCAGGGCATCCTGGTCACCGTCTCCTCAGCCTCCACCACCGCCCCGAAGGT
CTTCGCGCTGGCCCCCGGCTGTGGGACCACATCTGACTCCACGGTGGCCCTGGGCTGCCTTGTCTCCGGATACTTCC
CCGAGCCAGTGAAGGTGTCCTGGAACTCGGGCTCCCTGACCAGTGGCGTGCACACCTTCCCTTCCGTCCTGCAGTCC
TCAGGGTTCTACTCCCTCAGCAGCATGGTGACCGTGCCTGCCAGCACCTGGACCAGCGA
>KY437657.1 Equus caballus clone IGHG mRNA clone 14 immunoglobulin mu heavy chain G (IGHM) mRNA, partial cds
CTCTCCCTCCCCTGCACTGTCTCTGGATTATCTTTGAGCAGTAGTGCTGTGGGCTGGGTCCGCCAGGCTCCAGGTAA
AGGGCTGGAATTTGTTGGTGGTGTAGGTAGTGATGGACTTACAAGGCACAACCCAGCCCTGAAGTCCCGAGCCAGCA
TCACCAAGGACACCTCAAAGAGCCAAGTTTATCTGACGCTGAACAGCCTGACAAGCGAGGACACGGCCGTCTATTAC
TGTGTAGGAGGCTCTTATAGTGATTATCTGTATTACGTTTCTACAATAACGTACTGGGGCCAGGGCATCCTGGTCAC
CGTCTCCTCAGCCTCCACCACCGCCCCGAAGGTCTTCGCGCTGGCCCCCGGCTGTGGGACCACATCTGACTCCACGG
TGGCCCTGGGCTGCCTTGTCTCCGGATACTTCCCCGAGCCAGTGAAGGTGTCCTGGAACTCGGGCTCCCTGACCAGT
GGCGTGCACACCTTCCCTTCCGTCCTGCAGTCCTTAGGGTTCTACTC
>KY437656.1 Equus caballus clone IGHG mRNA clone 13 immunoglobulin mu heavy chain G (IGHM) mRNA, partial cds
CTCTCCCTCACCTGCACTGTCTCTGGATTCTCTTTGAGCACGTATGCTGTAGGCTGGGTCCGCCAGGCTCCAGGAAA
AGGGCTGGAATATGTTGGTGAAATATATGGTAGTGCAAGTGCAAACTACAACCCAGCCCTGAAGTCCCGAGCCAGCA
TCACCAAGGACACCTCAAAGAGCCAAGTTTATCTGACGCTGAACAGCCTGACAGGCGAGGACACGGCCGTCTATTAC
TGTGCGAGAGTTAGGGTGGATGGTATAAACTACTGGGGCCAGGGCATCCTGGTCACCGTCTCCTCAGCCTCCACCAC
CGCCCCGAAGGTCTTCGCGCTGGCCCCCGGCTGTGGGACCACATCTGACTCCACGGTGGCCCTGGGCTGCCTTGTCT
CCGGATACTTCCCCGAGCCAGTGAAGGTGTCCTGGAACTCGGGCTCCCTGACCAGTGGCGTGCACACCTTCCCTTCC
GTCCTGCAGTCCTCAGGGTTCTACTC
>KY437655.1 Equus caballus clone IGHG mRNA clone 12 immunoglobulin mu heavy chain G (IGHM) mRNA, partial cds
CTGTCCCTCACCTGCACTGTCTCTGGATTATCTTTGAGCAGTAATGCTGTAGGCTGGGTCCGCCAGGCTCCAGGAAA
AGGGCTGGAGTGGGTTGGTGTTATATATGGTAGTGAAAGTACATACTACAACCCAGCCCTGAAGTCCCGAGCCAGCA
TCACCAAGGACACCTCAAAGAGCCAAGTTTATCTGACGCTGAACAGCCTGACAGGCGAAGACACGGCCGTCTATTAC
TGTGCAGGATTATTTCTGGATATGAGCAGCTATGCTTACTACCTAAGGGGTGGATATTATTATGGTATAAACTACTG
GGGCCAGGGCATCCTGGTCACCGTCTCCTCAGCCTCCACCACCGCCCCGAAGGTCTTCGCGCTGGCCCCCGGCTGTG
GGACCACATCTGACTCCACGGTGGCCCTGGGCTGCCTTGTCTCCGGATACTTCCCCGAGCCAGTGAAGGTGTCCTGG
AACTCGGGCTCCCTGACCAGTGGCGTGCACACCTTCCCTTCCGTCCTGCAGTCCTCAGGGTTCTACTC
>KY437654.1 Equus caballus clone IGHG mRNA clone 11 immunoglobulin mu heavy chain G (IGHM) mRNA, partial cds
CTCTCCCTCACCTGCACTGTCTCTGGATTCTCTTTGAGCAGTGATGCTGCATACTGGGTCCGCCAGGCTCCAGGAAA
AGGGCTGGAATATGTTGGTACTATATATGCTGGTGCAAGTACAAACTACAACCCAGCCCTGAAGTCCCGAGCCAGCA
TCACCAAGGACACCTCAAAGAGCCAAGTTTATCTGACCCTGAACAGCCTGACAGGCGAGGACACGGCCGTCTATTAC
TGTGCGAGATCATATGGTGGTGGTAGTGCTTACTTCGCTTTTGGCTACTGGGGCCAGGGCACCCTGGTCACCGTCTC
CTCAGCCTCCACCACCGCCCCGAAGGTCTTCGCGCTGGCCCCCGGCTGTGGGACCACATCTGACTCCACGGTGGCCC
TGGGCTGCCTTGTCTCCGGATACTTCCCCGAGCCAGTGAAGGTGTCCTGGAACTCGGGCTCCCTGACCAGTGGCGTG
CACACCTTCCCTTCCGTCCTGCAGTCCTCAGGGTTCTACTC
>KY437653.1 Equus caballus clone IGHG mRNA clone 10 immunoglobulin mu heavy chain G (IGHM) mRNA, partial cds
CTCTCCCTCACCTGCACTGTCTCTGGATTATCTTTGAGTAGTTATGGTGTGGGCTGGGTCCGCCAGGCTCCAGGAAA
AGGGCTGGAATTTGTTGGTGGTATACGTAGTAGTGGAAGTGCAAACTACAACCCAGCCCTGAAGTCCCGAGCCAGCA
TCACCAAGGACACCTCAAAGAGCCAAGTTTATCTGACGCTGAACAGCCTGACAAGCGAGGACACGGCCGTCTATTAT
TGTGCGAGGAAAATGGTGATACTTTCTACTATCCCTAATTCTATGGACCCCTGGGGCCAGGGCACCCTGGTCACCGT
CTCCTCAGCCTCCACCACCGCCCCGAAGGTCTTCGCGCTGGCCCCCGGCTGTGGGACCACATCTGACTCCACGGTGG
CCCTGGGCTGCCTTGTCTCCGGATACTTCCCCGAGCCAGTGAAGGTGTCCTGGAACTCGGGCTCCCTGACCAGTGGC
GTGCACACCTTCCCTTCCGTCCTGCAGTCCTCAGGGTTCTACTC

>KY437652.1 Equus caballus clone IGHG mRNA clone 9 immunoglobulin mu heavy chain G (IGHM) mRNA, partial cds
CTCTCCCTCACCTGCACTGTCTCTGGATTCTCTTTGAGCAGTTATGCTGTAGGCTGGGTCCGCCAGGCTCCAGGAAA
AGGGCTGGAATATGTTGCTTCTATATACGGTAGTGTAAGTGCAAACTTCAACCCAGCCCTGAAGTCCCGAGCCAGCA
TCACCAAGGACACCTCAAAGAGCCAAGTGATTCTGACGCTGAACAGCCTGACAGGCGAGGACACGGCCGTCTATAGA
TGTGCGAGTGGTGTTGGTAATACTCGTTGGTTTGATTATGGTATAAGGTACTGGGGCCAGGGCATCCTGGTCACCGT
CTCCTCAGAGAGCCCTAAGGCCCCAGACGTCTTCCCGCTGACCATCTGTGGGAACACACCTGACCCCACGGTGCCCG
TGGGCTGCCTGGTCTCCAACTACTTCCCAGAGCCAGTGACCGTGTCCTGGAACTGTGATGCCCTGAAAGGCGACATA
CACACCTTTCCGCTGGACCTGAGCAACTCGGCTCACCACTCCCTCAGCAGCATGATGGCTGTGCCTAGGAGCAGCTT
GAACCA
>KY437651.1 Equus caballus clone IGHG mRNA clone 8 immunoglobulin mu heavy chain G (IGHM) mRNA, partial cds
CTCTCCCTCACCTGCACTGTCTCTGGATTATCTTTGAGCAGTAATGCTGTAGGCTGGGTCCGCCAGGCTCCAGGCAA
AGGGCTGGAATTTGTTGGTGCTATATATGGTAGTGCAAGTGCAAAGTACAACCCAGCCCTGAAGTCCCGAGCCAGCC
TCACCAAGGACACCTCAAAGAGCCAAGTTTATTTGACGCTGAACAGCCTGACAAGCGAGGACACGGCCGTCTATTAT
TGTGTAGGAGGGGCATATATAGCTGCTTTCGCAGCTATGCTTGGCTACTGGGGCCAGGGCACCCTGGTCACCGTCTC
CTCAGCCTCCACCACCGCCCCGAAGGTCTTCGCGCTGGCCCCCGGCTGTGGGACCACATCTGACTCCACGGTGGCCC
TGGGCTGCCTTGTCTCCGGATACTTCCCCGAGCCAGTGAAGGTGTCCTGGAACTCGGGCTCCCTGACCAGTGGCGTG
CACACCTTCCCTTCCGTCCTGCAGTCCTCAGGGTTCTACTCCCTCAGCAGCATGGTGACCGTGCCTGCCAGCACCTG
GACCAGCGA
>KY437650.1 Equus caballus clone IGHG mRNA clone 7 immunoglobulin mu heavy chain G (IGHM) mRNA, partial cds
CTCTCCCTCACCTGCACTGTCTCTGGATTATCTTTGAGTAGTTATGGTGTGGGCTGGGTCCGCCAGGCTCCAGGAAA
AGGGCTGGAATTTGTTGGTGGTATACGTAGTAGTGGAAGTGCAAACTACAACCCAGCCCTGAAGTCCCGAGCCAGCA
TCACCAAGGACACCTCAAAGAGCCAAGTTTATCTGACGCTGAACAGCCTGACAAGCGAGGACACGGCCGTCTATTAT
TGTGCGAGGAAAATGGTGATACTTTCTACTATCCCTAATTCTATGGACCCCTGGGGCCAGGGCACCCTGGTCACCGT
CTCCTCAGCCTCCACCACCGCCCCTAAGGTCTTCGCGCTGGCCCCCGGCTGTGGGACCACATCTGACTCCACGGTGG
CCCTGGGCTGCCTTGTCTCCGGATACTTCCCCGAGCCAGTGAAGGTGTCCTGGAACTCGGGCTCCCTGACCAGTGGC
GTGCACACCTTCCCTTCCGTCCTGCAGTCCTCAGGGTTCTACTC
>KY437649.1 Equus caballus clone IGHG mRNA clone 6 immunoglobulin mu heavy chain G (IGHM) mRNA, partial cds
CTGTCCCTCACCTGCACTGTCTCTGGATTCTCTTTGAGCAGTTACGGTGTAGGCTGGGTCCGCCAGGCTCCAGGAAA
AGGGCTGGAATATGTTGGTAGTATAACTAGTAGTGGAAGTGCAAACTACAACCCAGTCCTGAAGTCCCGAGCCAACA
TCACCAAGGACACCTCAAAGAGCCAAGTTTATATGACGCTGAACAGCCTGACAGGCGAGGACACGGCCGTCTATTAC
TGTGCGAGATTCTACGGTGTTGAGTGTTTTGGCTACTGGGGCCAGGGCACCCTGGTCACCGTCTCCTCAGCCTCCAC
CACCGCCCCGAAGGTCTTCGCGCTGGCCCCCGGCTGTGGGACCACATCTGACTCCACGGTGGCCCTGGGCTGCCTTG
TCTCCGGATACTTCCCCGAGCCAGTGAAGGTGTCCTGGAACTCGGGCTCCCTGACCAGTGGCGTGCACACCTTCCCT
TCCGTCCTGCAGTCCTCAGGGTTCTACTC
>KY437648.1 Equus caballus clone IGHG mRNA clone 5 immunoglobulin mu heavy chain G (IGHM) mRNA, partial cds
CTCTCCCTCACCTGCACTGTCTCTGGATTCTCTTTGAGCAGTAATGCTGTAGGCTGGGTCCGCCAGGCTCCAGGAAA
AGGGCTGGAATACGTTGGTGCTATATATGGTAGTGCAAGTGCAAACTACAACCCAGCCCTGAAGTCCCGAGCCAGCA
TCACCAAGGACACCTCAAAGAGCCAAGTTTATCTGACGCTGAACAGCCTGACAAGCGAGGACACGGCCGTCTATTAC
TGTGCAGGAGGCCCTATAGCAGCTATGCACCTGCCTTATTGGTTCGAATACGTCGTATTTGGCTACTGGGGCCAGGG
CACCCTGGTCACCGTCTCCTCAGCCTCCACCACAGCCCCGAAGGTCTTCCCTCTGGCTCCCAGCTGTGGGACCACAT
CTGACTCCACGGTGGCCCTGGGCTGCCTGGTCTCCAGCTACTTCCCAGAGCCAGTGACCGTGTCCTGGAACTCGGGC
ACGCTGACCAGCGGTGTGCGCACCTTCCCGTCCGTCCTGCAGTCCTCGGGGCTCTACTC
>KY437647.1 Equus caballus clone IGHG mRNA clone 4 immunoglobulin mu heavy chain G (IGHM) mRNA, partial cds
CTCTCCCTCACCTGCACTGTCTCTGGATTATCTTTGAGAAATAATGCTGTAGGCTGGGTCCGCCAGGCTCCAGGAAA
AGGGCTGGAATACGTTGTTCGTATATATGATAAAAGTACAAACTACAACCCAGCCCTGAAGTCCCGAGCCAGCATCA
GCAAGGACACCTCAAAGAGTCAAGTTTATCTGACGCTGAACAGCCTGACAAGCGAGGACACGGCCGTCTATTACTGT
GCAGGAGGCTACGCTTATGTGTACTGGGGCCAGGGCATCCTGGTCACCGTCTCCTCAGCCTCCACCACCGCCCCGAA
GGTCTTCGCGCTGGCCCCCGGCTGTGGGACCACATCTGACTCCACGGTGGCCCTGGGCTGCCTTGTCTCCGGATACT

TCCCCGAGCCAGTGAAGGTGTCCTGGAACTCGGGCTCCCTGACCAGTGGCGTGCACACCTTCCCTTCCGTCCTGCAG
TCCTCAGGGTTCTACTCCCTCAGCAGCATGGTGACCGTGCCTGCCAGCACCTGGACCAGCGA
>KY437646.1 Equus caballus clone IGHG mRNA clone 3 immunoglobulin mu heavy chain G (IGHM) mRNA, partial cds
CTGTCCCTCACCTGCACTGTCTCTGGATTATCTTTGAGCAGTAATGCTGTAGGCTGGGTCCGCCAGGCTCCAGGAAA
AGGGCTGGAGTGGGTTGGTGTTATATATGGTAGTGAAAGTACATACTACAACCCAGCCCTGAAGTCCCGAGCCAGCA
TCACCAAGGACACCTCAAAGAGCCAAATTTATCTGACGCTGAACAGCCTGACAGGCGAAGACACGGCCGTCTATTAC
TGTGCAGGATTCATCGATTCGGATGCTGGTAGTTACTCGAAACAGTTTTATGGCTACGTGGATCACTGGGGCCAGGG
CACCCTGGTCACCGTCTCCTCAGCCTCCACCACCGCCCCGAAGGTCTTCGCGCTGGCCCCCGGCTGTGGGACCACAT
CTGACTCCACGGTGGCCCTGGGCTGCCTTGTCTCCGGATACTTCCCCGAGCCAGTGAAGGTGTCCTGGAACTCGGGC
TCCCTGACCAGTGGCGTGCACACCTTCCCTTCCGTCCTGCAGTCCTCAGGGTTCTACTCCCTCAGCAGCATGGTGAC
CGTGCCTGCCAGCACCTGGACCAGCGA
>KY437645.1 Equus caballus clone IGHG mRNA clone 2 immunoglobulin mu heavy chain G (IGHM) mRNA, partial cds
CTGTCCCTCACCTGCACTGTCTCTGGATTATCTTTGACTGGTAGTGATGTAGGCTGGGTCCGCCAGGCTCCAGGAAA
AGGGCTGGAGTGGGTTGGTAGAATATATGATAGTGAAAATATATACTACAACCCAGCCCTGAAGTCCCGAGCCAGCA
TCACCAAGGACACCTCAAAGAGCCAAGTTTATCTGACGCTGAACAGCCTGACAGGCGAAGACACGGCCGTCTATTAC
TGTGCAGCATACGGTTATGAATATGCGATATACTTTTGGGGCCAGGGCATCCTGGTCACCGTCTCCTCAGCCTCCAC
CACCGCCCCGAAGGTCTTCGCGCTGGCCCCCGGCTGTGGGACCACATCTGACTCCACGGTGGCCCTGGGCTGCCTTG
TCTCCGGATACTTCCCCGAGCCAGTGAAGGTGTCCTGGAACTCGGGCTCCCTGACCAGTGGCGTGCACACCTTCCCT
TCCGTCCTGCAGTCCTCAGGGTTCTACTCCCTCAGCAGCATGGTGACCGTGCCTGCCAGCACCTGGACCAGCGA
>KY437644.1 Equus caballus clone IGHG mRNA clone 1 immunoglobulin mu heavy chain G (IGHM) mRNA, partial cds
CTCTCCCTCACCTGCACTGTCTCTGGATTATCTTTGAACACTTATGGTGTGGCCTGGGTCCGCCAGGCTCCAGGAAA
AGGGCTGGAATGGATTGGTGGTATAGCTAGTAGTGGAAGTGCGAGCAACAACCCAGCCCTGAAGTCCCGAGCCAGCA
TCACCAAGGACACCTCAAAGAGCCAAGTTTATCTGACGCTGAACAGCCTGACAAGCGAGGACACGGCCGTCTATTAC
TGTGCGGGTGCTTTTTACTACTATTGGGGCCAGGGCATCCTAGTCACCGTCTCCTCAGAGAGCCCTAAGGCCCCAGA
CGTCTTCCCGCTGAGCATCTGTGGGAACACACCTGACCCCAAGGTGCCCGTGGGCTGCCTGGTCTCCAACTACTTCC
CAGAGCCAGTGACTGTGTCCTGGAACTGTGATGCCCTGAAAGGCGACATACACACCTTTCCGTTGGACCTGAGCAAC
TCGGCTCACCACTC
>KC549764.1 Equus caballus clone 5'RACE Foal MLN T10 immunoglobulin heavy chain mRNA, partial cds
ATTTTCAGGGAGTCGTGAATCTCATCTGCAAGAACATGAATCACCTGTGGTTCTTCCTCTTTCTGGTGGCCGCTCCT
ACATGTGTCCTGTCCCAGGTGCAACTGAAGGAGTCGGGACCTGGCCTGGTGAAGCCCTCGCAGACCCTGTCCCTCAC
CTGCACTGTCTCTGGATTATCTTTGAGCAGTAATCATGTAGGCTGGGTCCGCCAGGCTCCAGGAAAAGGGCTGGAGT
GGGTTGCTGTTATATATGGTGGTGAAAGTACATACTACAACCCAGCCCTGAAGTCCCGAGCCAGCATCACCAAGGAC
ACCTCAAAGAGCCAAGTTTATCTGACGCTGAACAGCCTGACAGGCGAAGACACGGCCGTCTATTACTGTGCAGGATT
CGGTAATATGGTTATGTATGGCTGGGTGGATCACTGGGGCCAGGGCACC
>KC549762.1 Equus caballus clone 5'RACE Foal MLN T12 immunoglobulin heavy chain mRNA, partial cds
ATTTTCAGGGAGTCGTGAATCTCATCTGCAAGAACATGAGTCACCTGTGGTTCTTCCTCTTTCTGGTGGCCGCTCCT
ACATGTGTCCTGTCCCAGGTGCAACTGAAGGAGTCAGGACCTGGCCTGGTGAAGCCCTCGCAGACCCTCTCCCTCAC
CTGCACTGTCTCTGGATTATCTTTGAGCAGTAATGCTGTAGGCTGGGTCCGCCAGGCTCCAGGAAAAGGGCTGGAGT
GGGTTGGTGTTATATGGGGTAGTGAAAGTACATACTACAACCCAGCCCTGAAGTCCCGAGCCAGCATCACCAAGGAC
TCCTCAAAGAGCCAAGTTTATCTGACGCTGAACAGCCTGACAGGCGAAGACACGGCCGTCTATTACTGTGCAGGATC
TTATAGCAACGATGGCGAGTATGGTATAAACTACTGGGGCCAGGGCATC
>KC549761.1 Equus caballus clone 5'RACE Foal MLN T01 immunoglobulin heavy chain mRNA, partial cds
ATTTTCAGGGAGTCGTGAATCTCATCTGCAAGAACATGAATCACCTGTGGTTCTTCCTCTTTCTGGTGGCCGCTCCT
ACATGTGTCCTGTCCCAGGTGCAACTGAAGGAGTCGGGACCTGGCCTGGTGAAGCCCTCGCAGACCCTGTCCCTCAC
CTGCACTGTCTCTGGATTATCTTTGAGCAGTAATGCTGTAGGCTGGGTCCGCCAGGCTCCAGGAAAAGGGCTGGAGT
GGGTTGGTGTTATATATGGTAGTGAAAGTACATACTACAACCCAGCCCTGAAGTCCCGAGCCAGCATCACCAAGGAC
ACCTCAAAGAGCCAAGTTTATCTGACGCTGAACAGCCTGACAGGCGAAGACACGGCCGTCTATTACTGTGCAGGGGA
GGACTATGCTGGTAGTTACTATGGAAGCGATGGTATAAACTACTGGGGCCAGGGCATC
>KC549752.1 Equus caballus clone 5'RACE Foal MLN T31 immunoglobulin heavy chain mRNA, partial cds

ATTTTCAGGGAGTCGTGAATCTCATCTGCAAGAACATGAATCACCTGTGGTTCTTCCTCTTTCTGGTGGCCGCTCCT
ACATGTGTCCTGTCCCAGGTGCAACTGAAGGAGTCAGGACCTGGCCTGGTGAAGCCCTCGCAGACCCTCTCCCTCAC
CTGCACTGTCTCTGGATTATCTTTGAGCAGTTATGCTGTAGGCTGGGTCCGCCAGGCTCCAGGAAAAGGGCTGGAAT
ATGTTGGTGTTATATATGGTAGTGCAAGTGCAAACTACAACCCAGCCCTGAAGTCCCGAGCCAGCATCACCAAGGAC
ACCTCAAAGAGCCAAGTTTATCTGACGATGAACAGCCTGACAGGCGAGGACACGGCCGTCTATTACTGTGCGAGATT
GAACGGTTGGAATTATGCTACAGAGGTTGGCTACTGGGGCCAGGGCACC
>KC549747.1 Equus caballus clone 5'RACE Foal MLN T21 immunoglobulin heavy
chain mRNA, partial cds
ATTTTCAGGGAGTCGTGAATCTCATCTACAAGAACATGAGTCACCTGTGGTTCTTCCTCTTTCTGGTGGCCGCTCCT
ACATGTGTCCTGTCCCAGGTGCAACTGAAGGAGTCAGGACCTGGCCTGGTGAAGCCCTCGCAGACCCTGTCCCTCAC
CTGCACTGTCTCTGGATTCTCTTTGAGCAGTTACGGTGTACGCTGGGTCCGCCAGGCTCCAGGAAAAGGGCTGGAAT
TTGTTGGTGGTATTGGTAGTGATGGAAGTGTGTATTACAATCCAGCCCTGAAGTCCCGAGCCAGCATCACCAAGGAC
ACCTCAAAGAGCCAAGTTTATCTGACGCTGAACAGCCTGACAGGCGAGGACACGGCCGTCTATTACTGTGCGAGACG
TTACACTTTATCCGGGTATTGGGTTATGGACCCCTGGGGCCAGGGCACC
>KC549746.1 Equus caballus clone 5'RACE Foal MLN T02 immunoglobulin heavy
chain mRNA, partial cds
ATTTTCAGGGAGTCGTGAATCTCATCTACAAGAACATGAGTCACCTGTGGTTCTTCCTCTTTCTGGTGGCCGCTCCT
ACATGTGTCCTGTCCCAGGTGCAACTGAAGGAGTCAGGACCTGGCCTGGTGAAGCCCTCGCAGACCCTGTCCCTCAC
CTGCACTGTCTCTGGATTCTCTTTGAGCAGTTACGGTGTAGGCTGGGTCCGCCAGGCTCCAGGAAAAGGGCTGGAAA
TTGTTGGTGCTATAACTAATAGTGGAAGTGCAAACTACAACCCAGCCCTGAAGTCCCGAGCCAGCATCACCAAGGAC
ACCTCAAAGAGCCAAGTTTATCTGACGCTGAACAGCCTGACAGGCGAGGACACGGCCGTCTATTACTGTGCGAGAAA
CTACGGTGCTGGTTTTGCTACGTTTGGCTACTGGGGCCAGGGCACC
>KC549743.1 Equus caballus clone 5'RACE Foal MLN T13 immunoglobulin heavy
chain mRNA, partial cds
AGGGAGTCGTGAATCTCATCTACAAGAACATGAGTCACCTGTGGTTCTTCCTCTTTCTGGTGGCCGCTCCTACATGT
GTCCTGTCCCAGGTGCAACTGAAGGAGTCAGGACCTGGCCTGGTGAAGCCCTCGCAGACCCTGTCCCTCACCTGCAC
TGTCTCTGGATTCTCTTTGAGCAGTTACGGTGTAGGCTGGGTCCGCCAGGCTCCAGGAAAAGGGCTGGAATTTGTTG
GTGGTATAGCTAGTAGTGGAAGTGCAAACTACAACCCAGCCCTGAAGTCCCGAGCCAGCATCACCAAGGACACCTCA
AAGAGCCAAGTTTATCTGACGCTGAACAGCCTGACAGGCGAGGACACGGCCGTCTATTACTGTGCGAGGGGTTATGG
GCATGGTATAACATACTGGGGCCAGGGCATC
>KC549741.1 Equus caballus clone 5'RACE Neo MLN C07 immunoglobulin heavy
chain mRNA, partial cds
AGCATCACCCAAACATCACACTCCCTCCCCTACAGAAGCCCTGAGACCACAGTGCCTCACAATGGGCTGGAGCTGGA
GAATCCTCTTCTTGGTGGCAGTAGCTTCAGGTGTCTCCTCCGAGGTCCAGCTAATACAGTCGGGGCCAGAGTTGAAG
AAGCCTGGGTCATCAGTGAAGATCTCCTGCAAGGCTTCTGGATACACCTTCACTGAATATGCTATGCACTGGGTGCG
ACAGGCCAATGGAAAAGGGATTGAATGGATGGGATCTATCAGTCCTCATGATGATGATACGAGCTACGCACCGAAGT
TCCAAGGCAGAGTCACCATCACCGTGGACAAGTCCACGAGCACAGTCTACATGGAGCTGAGCAGTCTGACATCTGAG
GACACGGCCATGTATTACTGTGCGAAAGAGCCTTATGGTTACTATAGTAGGAGCCTTTACTATTTTAGCTACTGGGG
CCAGGGCACC
>KC549736.1 Equus caballus clone 5'RACE Neo MLN C05 immunoglobulin heavy
chain mRNA, partial cds
ATTTTCAGGGAGTCATGAATCTCATCTGCAAGAACATGAATCACCTGTGGTTCTTCCTCTTTCTGGTGGCCGCTCCT
AGATGTGTCCTGTCCCAGGTGCAACTGAAGGAGTCAGGACCTGGCCTGGTGAAGCCCTCGCAGACCCTCTCCCTCAC
CTGCACTGTCTCTGGATTCTCTTTGAGCAGTTATGCTGTAGGCTGGGTCCGCCAGGCTCCAGGAAAAGGGCTGGAAT
ATGTTGGTGAAATATATGGTAGTGAAAGTGCAAACTACAACCCAGCCCTGAAGTCCCGAGCCAGCATCACCAAGGAC
ACCTCCAAGAGCCAAGTTTATCTGACGCTGAACAGCCTGACAGGCGAGGACACGGCCGTCTATTACTGCGCGAAAGA
TAGCAATGGTAATACTGACCACTATACATTGGATGGTATAGACTACTGGGGCCAGGGCATT
>KC549735.1 Equus caballus clone 5'RACE Neo MLN B07 immunoglobulin heavy
chain mRNA, partial cds
ATTTTCAGGGAGTCATGAATCTCATCTGCAAGAACATGAATCACCTGTGGTTCTTCCTCTTTCTGGTGGCCGCTCCT
AGATGTGTCCTGTCCCAGGTGCAACTGAAGGAGTCAGGACCTGGCCTGGTGAAGCCCTCGCAGACCCTCTCCCTCAC
CTGCACTGTCTCTGGATTCTCTTTGAGCAGTTATGCTGTAGGCTGGGTCCGCCAGGCTCCAGGAAAAGGGCTGGAAT
ATGTTGGTGCTATATATGGTAGTGCAAGTGCAAACTACAACCCAGCCCTGAAGTCCCGAGCCAGCATCACCAAGGAC
ACCTCCAAGAGCCAAGTTTATCTGACGCTGAACAGCCTGACAGGCGAGGACACGGCCGTCTATTACTGTGCGAAAGA
GGGAGCTGCTATAGCAGCTATGCTTACTTATGGTATAGACTACTGGGGCCAGGGCATC

>KC549733.1 Equus caballus clone 5'RACE Neo MLN B03 immunoglobulin heavy chain mRNA, partial cds
ATTTTCAGGGAGTCGTGAATCTCATCTGCAAGAACATGAATCACCTGTGGTTCTTCCTCTTTCTGGTGGCCGCTCCT
ACATGTGTCCTGTCCCAGGTGCAACTGAAGGAGTCGGGACCTGGCCTGGTGAAGCCCTCGCAGACCCTGTCCCTCAC
CTGCACTGTCTCTGGATTATCTTTGAGCAGTAATGCTGGCGGCTGGGTCCGCCAGGCTCCAGGAAAAGGGCTGGAGT
GGGTTGGTTGGGTATATGGTGGTGGCAGTACATACTACAACCCAGCCCTGAAGTCCCGAGCCAGCATCACCAAGGAC
ACCTCAAAGAGCCAAGTTTATCTGACGCTGAACAGCCTGACAGGCGAAGACACGGCCGTCTATTACTGTGCAGGCCG
ACATGGTTATGGTGGTGCTTACTATGGTATTGACTACTGGGGCCAGGGCATC
>KC549732.1 Equus caballus clone 5'RACE Neo MLN A11 immunoglobulin heavy chain mRNA, partial cds
ATTTTCAGGGAGTCGTGAATCTCATCTGCAAGAACATGAATCACCTGTGGTTCTTCCTCTTTCTGGTGGCCGCTCCT
ACATGTGTCCTGTCCCAGGTGCAACTGAAGGAGTCGGGACCTGGCCTGGTGAAGCCCTCGCAGACCCTGTCCCTCAC
CTGCACTGTCTCTGGATTATCTTTGAGCAGTAATAATGTAGGCTGGGTCCGCCAGGCTCCAGGAAAAGGGCTGGAAT
GGGTTGGTACTGCATGGGGTAGTGAAGGTACATACTACAACCCAGCCCTGAAGTCCCGAGCCAGCATCACCAAGGAC
ACCTCAAAGGCCCAAGTTTATCTGACGCTGAACAGCCTGACAGGCGAAGACACGGCCGTCTATTACTGTGCAAAGCG
GGACTACCCAGCTGCTTATTTTGCCTACTGGGGCCAGGGCACC
>KC549726.1 Equus caballus clone 5'RACE Neo MLN A10 immunoglobulin heavy chain mRNA, partial cds
ATTTTCAGGGAGTCGTGAATCTCATCTGCAAGAACATGAATCACCTGTGGTTCTTCCTCTTTCTGGTGACCGCTCCT
ACATGTGTCCTGTCCCAGGTGCAACTGAAGGAGTCGGGACCTGGCCTGGTGAAGCCCTCGCAGACCCTGTCCCTCAC
CTGCACTGTCTCTGGATTATCTTTGAGCAGTAATGCTGTAGGCTGGGTCCGCCAGGCTCCAGGAAAAGGGCTGGAGT
GGGTTGGTGTAATATATGGTAGTGAAAGTACATACTACAACCCAGCCCTGAAGTCCCGAGCCAGCATCACCAAGGAC
ACCTCAAAGAGCCAAGTTTATCTGACGCTGAACAGCCTGACAGGCGAAGACACGGCCGTCTATTATTGTGCAGCAGG
GGGCCTCCTGTATGGTTACTATGCTGATGTTTACAGTAACTACTGGGGTCAGGGCACC
>KC549712.1 Equus caballus clone 5'RACE Neo MLN B11 immunoglobulin heavy chain mRNA, partial cds
AGGGAGTCGTGAATCTCATCTGCAAGAACATGAGTCACCTGTGGTTCTTCCTCTTTCTGGTGGCCGCTCCTACATGT
GTCCTGTCCCAGGTGCAACTGAAGGAGTCAGGACCTGGCCTGGTGAAGCCCTCGCAGACCCTGTCCCTCACCTGCAC
TGTCTCTGGATTCTCTTTGAGCAGTTACGGTGTAGGCTGGGTCCGCCAGGCTCCAGGAAAAGGGCTGGAATTTGTTG
GTGGTATATTTAATAGTGGAAGTGCAGAATACAACCCAGCCCTGAAGTCCCGAGCCAGCATCACCAAGGACACCTCA
AAGAGCCAAGTTTATCTGACGCTGAACAGCCTGACAGGCGAGGACACGGCCGTCTATTACTGTGCGAGTTCCCGATA
CGGCTATGATAGTGCTTACTCGTATGACTACGTGGATCACTGGGGCCAGGGCACC
>KC549704.1 Equus caballus clone 5'RACE Eq F Spl C02 immunoglobulin heavy chain mRNA, partial cds
ATTTTCAGGGAGTCATGAATCTCATCTGCAAGAACATGAATCACCTGTGGTTCTTCCTCTTTCTGGTGGCCGCTCCT
AGATGTGTCCTGTCCCAGGTGCAACTGAAGGAGTCAGGACCTGGCCTGGTGAAGCCCTCGCAGACCCTCTCCCTCAC
CTGCACTGTCTCTGGATTCTCTTTGAGCAGTTATGCTGTATACTGGGTCCGCCAGGCTCCAGGAAAAGGGCTGGAAT
ATGTTGGTGCTATAGCTAGTAGTGGAAGTGCAAACTACAACCCAGCCCTGAAGTCCCGAGCCAGCATCACCAAGGAC
ACCTCCAAGAGCCAAGTTTATCTGACGCTGAACAGCCTGACAGGCGAGGACACGGCCGTCTATTACTGTGCGAAAGA
TCTTAGCAGCTATGCTTACTACAACGAGTTGGATTACTGGGGCCAGGGCACCC
>KC549703.1 Equus caballus clone 5'RACE Eq F Spl-02 immunoglobulin heavy chain mRNA, partial cds
ATTTTCAGGGAGTCGTGAATCTCATCTGCAAGAACATGAATCACCTGTGGTTCTTCCTCTTTCTGGTGGCCGCTCCT
ACATGTGTCCTGTCCCAGGTGCAACTGAAGGAGTCGGGACCTGGCCTGGTGAAGCCCTCGCAGACCCTCTCCCTCAC
CTGCACTGTCTCTGGATTCTCTTTGAGCAGTTATGCTGTATACTGGGTCCGCCAGGCTCCAGGAAAAGGGCTGGAAT
ATGTTGGTGCTATATATGGTAGTGCAAGTGCAAACTACAACCCAGCCCTGAAGTCCCGAGCCAGCATCACCAAGGAC
ACCTCAAAGAGCCAAGTTTATCTGACGCTGAACAGCCTGACAGGCGAGGACACGGCCGTCTATTACTGTGCGAGATC
GGCTGCTGGTAGTTACTATAATGCTATGGACCCCTGGGGCCAGGGCACCC
>KC549702.1 Equus caballus clone 5'RACE Eq F Spl C08 immunoglobulin heavy chain mRNA, partial cds
ATTTTCAGGGAGTCGTGAATCTCATCTGCAAGAACATGAATCACCTGTGGTTCTTCCTCTTTCTGGTGGCCGCTCCT
ACATGTGTCCTGTCCCAGGTGCAACTGAAGGAGTCAGGACCTGGCCTGGTGAAGCCCTCGCAGACCCTCTCCCTCAC
CTGCACTGTCTCTGGATTCTCTTTGAGCAGTTATGCTGTAGGCTGGGTCCGCCAGGCTCCAGGAAAAGGGCTGGAAT
ATGTTGGTGCTATATATGGTAGTGCAAGTGCAAACTACAACCCAGCCCTGAAGTCCCGAGCCAGCATCACCAAGGAC
ACCTCAAAGAGCCAAGTTTATCTGACGCTGAACAGCCTGACAGGCGAGGACACGGCCGTCTATTACTGTGCGAGATC
CGACTATAGCAGCTATGCTTATTATGGCTACGTGGATCACTGGGGCCAGGGCACCC

>KC549701.1 Equus caballus clone 5'RACE Eq F Spl A09 immunoglobulin heavy chain mRNA, partial cds
ATTTTCAGGGAGTCGTGAATCTCATCTGCAAGAACATGAATCACCTGTGGTTCTTCCTCTTTCTGGTGGCCGCTCCT
ACATGTGTCCTGTCCCAGGTGCAACTGAAGGAGTCAGGACCTGGCCTGGTGAAGCCCTCGCAGACCCTCTCCCTCAC
CTGCACTGTCTCTGGATTCTCTTTGAGCAGTTATGCTGTAGGCTGGGTCCGCCAGGCTCCAGGAAAAGGGCTGGAAT
ATGTTGGTGCTATATATGGTAGTGCAAGTGCAAACTACAACCCAGCCCTGAAGTCCCGAGCCAGCATCACCAAGGAC
ACCTCAAAGAGCCAAGTTTATCTGACGCTGAACAGCCTGACAGGCGAGGACACGGCCGTCTATTACTGTGCGAGATC
TTATTACTATGGTGGTAGTTCCTGGTTTGGCTACTGGGGCCAGGGCACCC
>KC549697.1 Equus caballus clone 5'RACE Eq F Spl-07 immunoglobulin heavy chain mRNA, partial cds
ATTTTCAGGGAGTCGTGAATCTCATCTGCAAGAACATGAATCACCTGTGGTTCTTCCTCTTTCTGGTGGCCGCTCCT
ACATGTGTCCTGTCCCAGGTGCAACTGAAGGAGTCGGGACCTGGCCTGGTGAAGCCCTCGCAGACCCTGTCCCTCAC
CTGCACTGTCTCTGGATTATCTTTGAGCAGTAATGCTGTAGGCTGGGTCCGCCAGGCTCCAGGAAAAGGGCTGGAGT
GGGTTGGTGTTATATATGGTAGTGAAAGTACATACTACAACCCAGCCCTGAAGTCCCGAGCCAGCATCACCAAGGAC
ACCTCAAAGAGCCAAGTTTATCTGACGCTGAACAGCCTGACAGGCGAAGACACGGCCGTCTATTACTGTGCAGGATC
TGGTGGTAGTTCCTGGTACTCCGACGATAATGCTATGGACCCCTGGGGCCAGGGCACCC
>KC549694.1 Equus caballus clone 5'RACE Eq F Spl B11 immunoglobulin heavy chain mRNA, partial cds
ATTTTCAGGGAGTCGTGAATCTCATCTGCAAGAACATGAATCACCTGTGGTTCTTCCTCTTTCTGGTGGCCGCTCCT
ACATGTGTCCTGTCCCAGGTGCAACTGAAGGAGTCGGGACCTGGCCTGGTGAAGCCCTCGCAGACCCTGTCCCTCAC
CTGCACTGTCTCTGGATTATCTTTGAGCAGTAATGCTGTAGGCTGGGTCCGCCAGGCTCCAGGAAAAGGGCTGGAGT
GGGTTGGTGTTATATATGGTAGTGAAAGTACATACTACAACCCAGCCCTGAAGTCCCGAGCCAGCATCACCAAGGAC
ACCTCAAAGAGCCAAGTTTATCTGACGCTGAACAGCCTGACAGGCGAAGACACGGCCGTCTATTACTGTGCAGGGGA
GGACTATGCTGGTAGTTACTATGGAAGCGATGGTATAAACTACTGGGGCCAGGGCATCC
>KC549692.1 Equus caballus clone 5'RACE Eq F Spl C03 immunoglobulin heavy chain mRNA, partial cds
ATTTTCAGGGAGTCGTGAATCTCATCTGCAAGAACATGAATCACCTGTGGTTCTTCCTCTTTCTGGTGGCCGCTCCT
ACATGTGTCCTGTCCCAGGTGCAACTGAAGGAGTCGGGACCTGGCCTGGTGAAGCCCTCGCAGACCCTGTCCCTCAC
CTGCACTGTCTCTGGATTATCTTTGAGCAGTAATGCTGTAGGCTGGGTCCGCCAGGCTCCAGGAAAAGGGCTGGAGT
GGGTTGGTGTTATATATGGTAGTGAAAGTACATACTACAACCCAGCCCTGAAGTCCCGAGCCAGCATCACCAAGGAC
ACCTCAAAGAGCCAAGTTTATCTGACGCTGAACAGCCTGACAGGCGAAGACACGGCCGTCTATTACTGTGCAGGATG
GTGGTACGGTTATGGTTATGCTTATGATATAGACTACTGGGGCCAGGGCACC
>KC549691.1 Equus caballus clone 5'RACE Eq F Spl B08 immunoglobulin heavy chain mRNA, partial cds
ATTTTCAGGGAGTCGTGAATCTCATCTGCAAGAACATGAGTCACCTGTGGTTCTTCCTCTTTCTGGTGGCCGCTCCT
ACATGTGTCCTGTCCCAGGTGCAACTGAAGGAGTCAGGACCTGGCCTGGTGAAGCCCTCGCAGACCCTCTCCCTCAC
CTGCACTGTCTCTGGATTATCTTTGAGCAGTAATGCTGTAGGCTGGGTCCGCCAGGCTCCAGGAAAAGGGCTGGAAT
TTGTTGGTGCTATATATGGTAGTGCAAGTGCAAACTACAACCCAGCCCTGAAGTCCCGAGCCAGCATCACCAAGGAC
ACCTCAAAGAGCCAAGTTTATCTGACGCTGAACAGCCTGACAAGCGAGGACACGGCCGTCTATTACTGTGCAGGAGG
CGAAGGAGATGATTATTATGGTGCTATTGACTACATAACCTATTTTGGCTACTGGGGCCAGGGCACCC
>KC549689.1 Equus caballus clone 5'RACE Eq F Spl A05 immunoglobulin heavy chain mRNA, partial cds
ATTTTCAGGGAGTCGTGAATCTCATCTGCAAGAACATGAGTCACCTGTGGTTCTTCCTCTTTCTGGTGGCCGCTCCT
ACATGTGTCCTGTCCCAGGTGCAACTGAAGGAGTCAGGACCTGGCCTGGTGAAGCCCTCGCAGACCCTCTCCCTCAC
CTGCACTGTCTCTGGATTATCTTTGAGCAGTTATGGTGTGGGCTGGGTCCGCCAGGCTCCAGGAAAAGGGCTGGAAT
TTGTTGGTGGTATAGCTAGTAGTGGAAGTGCAAACTACAACCCAGCCCTGAAGTCCCGAGCCAGCATCACCAAGGAC
ACCTCAAAGAGCCAAGTTTATCTGACGCTGAACAGCCTGACAAGCGAGGACACGGCCGTCTATTACTGTGCAGGAGG
TAGTTCCTGGTACTATGGCTACGTGGATCACTGGGGCCAGGGCACCC
>KC549686.1 Equus caballus clone 5'RACE Eq F Spl B02 immunoglobulin heavy chain mRNA, partial cds
ATTTTCAGGGAGTCGTGAATCTCATCTGCAAGAACATGAGTCACCTGTGGTTCTTCCTCTTTCTGGTGGCCGCTCCT
ACATGTGTCCTGTCCCAGGTGCAACTGAAGGAGTCAGGACCTGGCCTGGTGAAGCCCTCGCAGACCCTGTCCCTCAC
CTGCACTGTCTCTGGATTCTCTTTGAGCAGTTACGGTGTAGGCTGGGTCCGCCAGGCTCCAGGAAAAGGGCTGGAAT
TTGTTGGTGGTATAGCTAGTAGTGGAAGTGCAAACTACAACCCAGCCCTGAAGTCCCGAGCCAGCATCACCAAGGAC

ACCTCAAAGAGCCAAGTTTATCTGACGCTGAACAGCCTGACAGGCGAGGACACGGCCGTCTATTACTGTGCGAGATT
TTACTATGGTGGTAGTTCCTGGTACTCCTATGGCTACGTGGATCACTGGGGCCAGGGCACC
>KC549685.1 Equus caballus clone 5'RACE Eq F Spl A07 immunoglobulin heavy chain mRNA, partial cds
ATTTTCAGGGAGTCGTGAATCTCATCTGCAAGAACATGAGTCACCTGTGGTTCTTCCTCTTTCTGGTGGCCGCTCCT
ACATGTGTCCTGTCCCAGGTGCAACTGAAGGAGTCAGGACCTGGCCTGGTGAAGCCCTCGCAGACCCTGTCCCTCAC
CTGCACTGTCTCTGGATTCTCTTTGAGCAGTTACGGTGTAGGCTGGGTCCGCCAGGCTCCAGGAAAAGGGCTGGAAT
TTGTTGGTGGTATAGCTAGTAGTGGAAGTGCAAACTACAACCCAGCCCTGAAGTCCCGAGCCAGCATCACCAAGGAC
ACCTCAAAGAGCCAAGTTTATCTGACGCTGAACAGCCTGACAGGCGAGGACACGGCCGTCTATTACTGTGCGAGGTC
TAATTATTATGGTGCTATTGACTACATCTATTTGGCTACTGGGGCCAGGGCACCC
>KC549684.1 Equus caballus clone 5'RACE Eq F Spl A10 immunoglobulin heavy chain mRNA, partial cds
ATTTTCAGGGAGTCGTGAATCTCATCTGCAAGAACATGAGTCACCTGTGGTTCTTCCTCTTTCTGGTGGCCGCTCCT
ACATGTGTCCTGTCCCAGGTGCAACTGAAGGAGTCAGGACCTGGCCTGGTGAAGCCCTCGCAGACCCTGTCCCTCAC
CTGCACTGTCTCTGGATTCTCTTTGAGCAGTTACGGTGTAGGCTGGGTCCGCCAGGCTCCAGGAAAAGGGCTGGAAT
TTGTTGGTGGTATAGCTAGTAGTGGAAGTGCAAACTACAACCCAGCCCTGAAGTCCCGAGCCAGCATCACCAAGGAC
ACCTCAAAGAGCCAAGTTTATCTGACGCTGAACAGCCTGACAGGCGAGGACACGGCCGTCTATTACTGTGCGAGGGA
CGAATATGGTTATGGTGGTGCTTACTACTATGGTATAAACTACTGGGGCCAGGGCATCC
>KC549682.1 Equus caballus clone 5'RACE Eq F Spl C09 immunoglobulin heavy chain mRNA, partial cds
ATTTTCAGGTAGTCGTGAATCTCATCTGCAAGAACATGAGTCACCTGTGGTTCTTCCTCTTTCTGGTGGCCGCTCCT
ACATGTGTCCTGTCCCAGGTGCAACTGAAGGAGTCAGGACCTGGCCTGGTGAAGCCCTCGCAGACCCTGTCCCTCAC
CTGCACTGTCTCTGGATTCTCTTTGAGCAGTTACGGTGTAGGCTGGGTCCGCCAGGCTCCAGGAAAAGGGCTGGAAT
ATGTTGGTGGTATAGCTAGTAGTGGAAGTGCAAACTACAACCCAGCCCTGAAGTCCCGAGCCAGCATCACCAAGGAC
ACCTCAAAGAGCCAAGTTTATCTGACGCTGAACAGCCTGACAGGCGAGGACACGGCCGTCTATTACTGTGCGAGATC
TTCTCGTGGTGCTTACTACTATTTGGCTACTGGGGCCAGGGCACCC
>U17041.1 Equus caballus Ig epsilon heavy chain mRNA, partial cds
GGCACGAGGAATCACCTGTGGTTCTTCCTAGTTCTGGTGGCCGCTCCTACATGTGCCCCGTCCCAGGTGCAACTGAA
GGAGTCAGGACCTGGCCTGGTGAAGCCCTCGCAGACCCTGTCCCTCACCTGCACTGTCTCTGGATTCTCTTTGACTA
GTTACACTGTAGGCTGGGTCGCCAGGCTCCAGGAAAAGGACTGGAATATGTTGCGGCTATACGTAATAGTGGGAGTG
CAAACTTCAACCCAGACCCTGAAGTCCCGAGCCAGCATCACCAGGGACGCCTCAAAGAGCCAAGTTTATCTGACGCT
GAACAGCCTGACAGTCGAGGACACGGCCGTCTATTACTGTGCGAGGTCTCTGCCAGTCTCTGGTAATGGTTATAGTT
TGGTGAACTATTGGGGCCAGGCATCTGTCACCGTCTCATCAGTCTCCAAGCAGAGCCCCATTATCTTGCCCTTGGCT
GCCTGCTGCAAAGACACCAAGACTACTAACATCACACTGGGCTGCCTGGTCAAGGGCTACTTCCCGGAGCCAGTGAC
CGTGACCTGGGATGCAGGGTCCCTTAACCGGAGCACCATAACCTTCCCTGCCGTCTTTGACCAAACCTCTGGCCTCT
ACACCACCATCAGCAGGGTGGTCGCCTCGGGGAAGTGGGCCAAGCAGAAGTTCACCTGCAACGTGGTGCACTCCCAG
GAGACCTTCAACAAGACCTTCAACGCATGCATCGTGACCTTCACCCCACCCACCGTGAAGCTCTTCCACTCCTCCTG
CGACCCCGGCGGCGACTCCCATACCACCATCCAGCTCCTGTGCCTCATCTCCGACTACACCCCTGGCGACATCGACA
TCGTTTGGCTGATAGACGGGCAGAAGGTCGACGAGCAGTTCCCTCAACACGGCCTCGTGAAGCAGGAGGGCAAGCTG
GCCTCCACACACAGCGAGCTCAACATCACCCAGGGCCAGTGGGCGTCCGAAAACACCTACACCTGCCAGGTCACTTA
CAAAAGACATGATCTTTTAAGGACCAGGCCCCGCAAGTGCACAGAGTCTGAACCCCGCGGTGTGAGCGTCTACCTGA
GCCCGCCCAGCCCCCTGGACCTGTACGTCTCTAAATCGCCCAAGATCACCTGCCTGGTGGTGGACCTGGCCAACGTG
CAGGGCTTAAGCCTGAACTGGTCCCGGGAGAGCGGGGAGCCCCTGCAGAAGCACACACTGGCGACCAGCGAACAATT
TAACAAGACATTCTCGGTCACGTCCACCCTGCCTGTGGACACCACCGACTGGATCGAGGGCGAGACTTACAAGTGCA
CCGTGTCCCACCCAGACCTGCCCAGGGAAGTCGTGCGCTCCATCGCCAAGGCCCCTGGCAAGCGTTTGTCCCCCGAG
GTCTACGTGTTCCTGCCGCCTGAGGAGGACCAGAGCTCCAAGGACAAGGTCACCCTCACCTGCCTGATCCAGAACTT
CTTCCCCGCGGACATCTCCGTACAGTGGCTGCGTAACAATGTCCTAATCCAGACAGACCAGCAAGCCACCACACGCC
CCAAAAGCCAATGGCCCCGACCCGCCTTCTTCGTCTTCAGCCGCCTAGAGGTCAGCCGGGCGGAATGGGAGCAGAAG
AACAAATTTGCCTGCAAGGTGGTCCACGAGGCGCTGTCCCAAAGGACCCTCCAGAAAGAGGTGTCCAAAGACCCTGG
TAAATGATGCCCTCTGCCCGCCGCCTCCCACCCTCCCAGGCTCCCTCCTGCTGGGGTGGAGGTGGGGGCCAGCCAGA
CCTGCTCCGGTC
>U15150.1 Equus caballus IgE heavy chain mRNA, partial cds
CCTCTAAAGCGTGAATCTCATCTGCAAGAACATGAATCACCTGTGGTTCTTCCTCTTTCTGGTGACCGCCCTACATG
TGTCCTGTCCCAGGTGCAGCTGAAGGAGTCGGGACCTGGCCTGGTGAAGCCCTCGCAGACCCTGTCCCTCACCTGCA
CTGTCTCTGGATTATCTTTGAGCAGTAATTCTGTAGGCTGGGTCCGCCAGGCTCCAGGAAAAGGGCTGGAGTGGGTT
GGTTTTAGATCTGGTGGTGAGGAGGAATACTACAACCCAGCCCTGAAGTCCCGAGCCACTATCACCGAGGACGCCGC

GAAGAGCCAAGTTTATCTGGACGCTGAACAAGTGACAGGCGAAGCAACGGCCGTCTATTACTGTGCAGAAGTTTATA
ACAACTACCTTTATTACGGTATAAAGGAACTAGGGGCCAGGGGACTTCTGGTCACCGTCTCTTCAGTCTCCAAGCAA
GCCCCATTAATCTTTCCCTTGGCTGCCTGCTGCAAAGACACCAAGACTACTAACATCACACTGGGCTGCCTCGTCAA
GGGCTACTTCCCGGGAGCCTGGGATGCAGGGCCCCTTAACCCGAGCACCATGACCTTCCCTGCCGTCTTTGACCAAA
CCTCTGGCCTCTACACCACCATCAGCAGGGTGGTCGCCTCGGGGAAGTGGGCCAAGCAGAAGTTCACCTGCGGCGTG
GTGCACTCCCAGGAGACCTTCAACAAGACCTTCAACGCATGCATCGTGACCTTCACCCCACCCACCGTGAAGCTCTT
CCACTCCTCCTGCGACCCCGGCGGCGACTCCCATACCACCATCCAGCTCCTGTGCCTCATCTCCGACTACACCCCTG
GCGACATCGACATCGTTTGGCTGATAGAAGGGCAGAAGGTCGACGAGCAGTTCCCTACACAGGCCTCGATGAAGCAG
GAGGGAAGCTGGCCTCCAACACACAGCGAGCTCAACATCAACCAGGGCCAGTGGGCGTCCGAAAACACCTACACCTG
CCAGGTTACTTACAAAGACATGATCTTTAACCAGGCCCGCAAGTGCACAGAGTCTGACCCCCCCGGTGTGAGCGTCT
ACCTGAGCCCGCCCAGCCCCCTCGACCTGTACGTCTCTAAAACGCCCAAGATCACCTGCCTGGTGGTGGACCTGGCC
AACGTGCAGGGCTTAAGCCTGAACTGGTCCCGGGAGAGCGGGGAGCCCCTGCAGAAGCACACACTGGCCACCAGCGA
ACAATTTAACAAGACATTCTCAGTCACGTCCACCCTGCCTGTGGACACCACCGACTGGATCGAGGGCGAGACTTACA
AGTGCACCGTGTCCCACCCAGACCTGCCCAGGGAAGTCGTGCGCTCCATCGCCAAGGCCCCTGGCAAGCGTTTGTCC
CCCGAGGTCTACGTGTTCCTGCCGCCTGAGGAGGACCAGAGCTCCAAGGACAAGGTCACCCTCACCTGCCTGATCCA
GAACTTCTTCCCCGCGGACATCTCCGTACAGTGGCGTCGTAACAATGTCCTAATCCAGACAGACCAGCAAGCCACCA
CACGGCCCCAAAAGGCCAATGGCCCCGACCCCGCCTTCTTCGTCTTCAGCCGCCTAGAGGTCAGCCGGGCGGAATGG
GAGCAGAAGAACAAATTTGCCTGCAAGGTGGTCCACGAGGCGCTGTCCCAAAGGACCCTCCAGAAAGAGGTGTCCAA
AGACCCTGGTAAATGATGCCCTCTGCCCGCCGCCTGCCCACCCTCCCAGGGCTCCCTCCTGCTGGGGTGGAGGTGGG
GGCCAGCCAGACCTGCTCCGGTCATTGTTCGTTGTCAATAAACACTCCGGTGCCTGCT
>HM176092.1 Equus caballus clone 1HE113 immunoglobulin heavy chain V-D-J region mRNA, partial cds
ATGAGTCACCTGTGGTTCTTCCTCTTTCTGGTGGCCGCTCCTACATGTGTCCTCTCCCAGATGCAACTGAAGGAGTC
AGGACCTGGCCTGGTGAAGCCCTCGCAGACCCTCTCCCTCACCTGCACTGTCTCTGGATTATCTTTGAACGCGAATG
CTGTAGGCTGGGTCCGCCAGGCTCCAGGAAAAGGGCTGGAACTTGTTGCCTTTATATGGCAGATTGCAAGCTACAAT
CCAGCCCTGAAGTCCCGAGCCAGCATCACCAAGGACACCTCAAAGAGCCAAGTTTATCTGACCCTGAACAGCCTGAC
AAGCGAGGACACGGCCGTCTATTACTGTGCAAGGGGCAGTGTTGGTGGCTACACGACTTACTATGCCGATGCTATGG
ACCCCTGGGGCCAGGGCACCCTGGTCACCGTCTCCTCAGAGAGTACGATGACCCCAGATCTCTTCCCCCTTGTCTCC
TGTGGGCCCTCTCTTGATGAGAGCCTGGTGGCTGTGGGCTGCCTAGCCCGGGACTTCCTACCCAAG
>HM176091.1 Equus caballus clone 2H971 immunoglobulin heavy chain V-D-J region mRNA, partial cds
ATGAATCACCTGTGGTTCTTCCTCTTTCTGGTGGCCGCTCCTACATGTGTCCTGTCCCAGGTGCAACTGAAGGAGTC
AGGACCTGGCCTGGTGAAGCCCTCGCAGACCCTCTCCCTCACCTGCACTGTCTCTGGATTATCTTTGAATAGTTATG
CTGTAGGCTGGGTCCGCCAGGCTCCAGAAAAAGGGCTGGAATATGTCGGTGTAATATATGGTACGCAAAGTCCAGTC
TATAATCCGGCCCTGAAGTCCCGTGCCAGCATCACCAAGGACACCTCAAAGAGCCAAGTATATCTGACGCTGAACAG
CCTGACAGGCGAGGACACGGCTGTCTATTACTGTGCGAGCCATCCATTGTCTGGTTGGTATGCTGGTGGGGATGAGA
ATTACTGGGGCCAGGGCATCCTGGTCTTCGTCTCCTCAGAGAGTACGGTGACCCCAGATCTCTTCCCCCTCGTCTCC
TGTGGGCCCTCTCTTGATGAGAGCCTGGTGGCTGTGGGCTGCCTAGCCCGGGACTTCCTACCCAAG
>HM176090.1 Equus caballus clone 2H1-27 immunoglobulin heavy chain V-D-J region mRNA, partial cds
ATGAATCACCTGTGGTTCTTCCTCTTTCTGGTGGCCGCTCCTACATGTGTCCTGTCCCAGGTGCAACTGAAGGAGTC
AGGACCTGGCCTGGTGCAGCCCTCGCAGACCCTCTCCCTCACCTGCACTATCTCTGGATTATCTTTGAGTAGTTATG
CTGTAGGCTGGGTCCGCCAGGCTCCAGAAAAAGGGCTGGAATATGTCGGTGTAATATATGGTACGCAAAGTCCAGTC
TATAATCCGGCCCTGAAGTCCCGTGCCAGCATCACCAAGGACACCTCAAAGAGCCAAGTATATCTGACGCTGAACAG
CCTGACAGGCGAGGACACGGCTGTCTATTACTGTGCGAGCCATTCATTGTCTGGTTGGTATGCTGGTGGGGATGAGA
ATTACTGGGGCCAGGGCATCCTGGTCTTCGTCTCCTCAGAGAGTACGATGACCCCAGATCTCTTCCCCCTCGTCTCC
TGTGGGCCCTCTCTTGATGAGAGCCTGGTGGCTGTGGGCTGCCTAGCCCGGGACTTCCTACCCAAG
>HM176086.1 Equus caballus clone 2H549 immunoglobulin heavy chain V-D-J region mRNA, partial cds
ATGAGTCACCTGTGGTTCTTCCTCTTTCTGGTGGCCGCTCCTACATGTGTCCTGTCCCAGGTGCAACTGAAGGAGTC
AGGACCTGGCCTGGTGAAGCCCTCGCAGACCCTGTCCCTCACCTGCACTGTCTCTGGATTATCTTTGACCAGTAATC
TTATAGGCTGGGTCCGCCAGGCTCCAGGAAAAGGTCTGGAATGGGTGGGTGGTGTATCCAGTGATGGAACACCGGTG
TACAATCCAGTCCTGAAGACCCGAACCACCATCGGCAAGGACACCTCAAAGAGCCAAGTTTATTTGACGCTGAACAG
CATGACAAGCGAGGACACGGCCGTCTATTATTGTACACGATGCGGAGAATATGGCTACCATGGTGCGGTGGACCCCT

GGGGCCAGGGCACCCTGGTCACCGCCTCCTCAGAGAGTACGATGACCCCAGATCTCTTCCCCCTCGTCTCCTGTGGG
CCCTCTCTTGATGAGAGCCTGGTGGCTGTGGGCTGCCTAGCCCGGGACTTCCTACCCAAG
>HM176082.1 Equus caballus clone 1H48 immunoglobulin heavy chain V-D-J region mRNA, partial cds
ATGAGTCACCTGTGGTTCTTCCTCTTTCTGGTGGCCGCTCCTACATGTGTCCTGTCTCAGGTGCAACTGAAGGAGTC
AGGACCTGGCCTGGTGAAGCCCTCACAGACCCTCTCCCTCACCTGCACTGTCTCTGGATTATCTTTGAGCAGTTATG
CTGTAGGCTGGGTCCGCCAGACTCCAGGAAAAGGGCTGGAATATATTACTGGTATAAGTAGTGATGGAAGGCTAGAC
ACCAAGCCAGCCCTGAAGTCCCGAGCCAGCGTCACCAAGGACACCTCAAAGAGCCAAGTATATCTGACGCTGAACAG
CCTGACAAGCGAGGACACGGCCGTCTATTATTGTGCAGGGGGTGACCGTGGTTGGGTAAGGGATGGTATAAACTACT
GGGGCCAGGGCATCCTGGTCACCGTCTCCTCAGAGAGTACGATGACCCCAGATCTCTTCCCCCTCGTCTCCTGTGGG
CCCTCTCTTGATGAGAGCCTGGTGGCTGTGGGCTGCCTAGCCCGGGACTTCCTACCCAAG
>HM176081.1 Equus caballus clone 2H761 immunoglobulin heavy chain V-D-J region mRNA, partial cds
ATGAGTCACCTGTGGTTCTTCCTCTTTCTGGTGGCCGCTCCTACATGTGTCCTGTCCCAGGTGCAACTGAAGGAGTC
AGGACCTGGCCTGGTGAAGCCCTCGCAGACCCTCTCCCTCACCTGCACTGTCTCTGGATTATCTTTGAGCAGTAATC
ATGTACGCTGGGTCCGCCAGGCTCCAGGAAAAGGGCTGGAATGGGTTGGTGGTATACTTAGTGATGGAAGTGCAAAC
TACAATCCAGCCCTGAAGTCCCGAGCCAGCATCACCAAGGACACCTCAAAGACCCAAGTTTATCTGACGCTGAACAG
CCTGACAAGCGAGGACACGGCCGTCTATTACTGTACAGGAAGTACCTATCACCGCTACTGGGGCCAGGGCACCCTGG
TCACCGTCTCCTCAGAGAGTACGATGACCCCAGATCTCTTCCCCCTCGTCTCCTGTGGGCCCTCTCTTGATGAGAGC
CTGGTGGCTGTGGGCTGCCTAGCCCGGGACTTCCTACCCAAG
>HM176080.1 Equus caballus clone 2H606 immunoglobulin heavy chain V-D-J region mRNA, partial cds
ATGAATCACCTGTGGTTCTTCCTCTTTCTGGTGGCCGCTCCTACATGTGTCCTGTCCCAGGTGCAACTGAAGGAGTC
AGGACCTGGCCTGGTGAAGCCCTCGCAGACCCTCTCCCTCACCTGCACTGTCTCTGGATTATCTTTGAATAGTTATG
CTGTAGGCTGGGTCCGCCAGGCTCCAGAAAAAGGGCTGGAATATGTCGGTGTAATATATGGTACGCAAAGTCCAGTC
TATAATCCGGCCCTGAAGTCCCGTGCCAGCATCACCAAGGACACCTCAAAGAGCCAAGTATATCTGACGCTGAACAG
CCTGACAGGCGAGGACACGGCTGTCTATTACTGTGCGAGCCATCCATTGTCTGGTTGGTATGCTGGTGGGGATGAGA
ATTACTGGGGCCAGGGCATCCTGGTCTTCGTCTCCTCAGAGAGTACGGTGACCCCAGATCTCTTCCCCCTCGTCTCC
TGTGGGCCCTCTCTTGATGAGAGCCTGGTGGCTGTGGGCTGCCTAGCCCGGGACTTCCTACCCAAG
>HM176079.1 Equus caballus clone 2H993 immunoglobulin heavy chain V-D-J region mRNA, partial cds
ATGAATCACCTGTGGTTCTTCCTCTTTCTGGTGGCCGCTCCTACATGTGTCCTGTCCCAGGTGCAACTGAAGGAGTC
AGGACCTGGCCTGGTGAAGCCCTCGCAGACCCTCTCCCTCACCTGCACTGTCTCTGGATTATCTTTGAATAGTTATG
CTGTAGGCTGGGTCCGCCAGGCTCCAGAAAAAGGGCTGGAATATGTCGGTGTAATATATGGTACGCAAAGTCCAGTC
TATAATCCGGCCCTGAAGTCCCGTGCCAGCATCACCAAGGACACCTCAAAGAGCCAAGTATATCTGACGCTGAACAG
CCTGACAGGCGAGGACACGGCTGTCTATTACTGTGCGAGCCATCCATTGTCTGGTTGGTATGCTGGTGGGGATGAGA
ATTACTGGGGCCAGGGCATCCTGGTCTTCGTCTCCTCAGAGAGTACGGTGACCCCAGATCTCTTCCCCCTCGTCTCC
TGTGGGCCCTCTCTTGATGAGAGCCTGGTGGCTGTGGGCTGCCTAGCCCGGGACTTCCTACCCAAG
>HM176078.1 Equus caballus clone 2H682 immunoglobulin heavy chain V-D-J region mRNA, partial cds
ATGAATCACCTGTGGTTCTTCCTCTTTCTGGTGGCCGCTCCTACATGTGTCCTGTCCCAGGTGCAACTGAAGGAGTC
AGGACCTGGCCTGGTGAAGCCCTCGCAGACCCTCTCCCTCACCTGCACTGTCTCTGGATTATCTTTGAATAGTTATG
CTGTAGGCTGGGTCCGCCAGGCTCCAGAAAAAGGGCTGGAATATGTCGGTGTAATATATGGTACGCAAAGTCCAGTC
TATAATCCGGCCCTGAAGTCCCGTGCCAGCATCACCAAGGACACCTCAAAGAGCCAAGTATATCTGACGCTGAACAG
CCTGACAGGCGAGGACACGGCTGTCTATTACTGTGCGAGCCATTCATTGTCTGGTTGGTATGCTGGTGGGGATGAGA
ATTACTGGGGCCAGGGCATCCTGGTCTTCGTCTCCTCAGAGAGTACGATGACCCCAGATCTCTTCCCCCTCGTCTCC
TGTGGGCCCTCTCTTGATGAGAGCCTGGTGGCTGTGGGCTGCCTAGCCCGGGACTTCCTACCCAAG
>HM176077.1 Equus caballus clone 2H651 immunoglobulin heavy chain V-D-J region mRNA, partial cds
ATGAATCACCTGTGGTTCTTCCTCTTTCTGGTGGCCGCTCCTACATGTGTCCTGTCCCAGGTGCAACTGAAGGAGTC
AGGACCTGGCCTGGTGAAGCCCTCGCAGACCCTCTCCCTCACCTGCACTGTCTCTGGATTATCTTTGAATAGTTATG
CTGTAGGCTGGGTCCGCCAGGCTCCAGAAAAAGGGCTGGAATATGTCGGTGTAATATATGGTACGCAAAGTCCAGTC
TATAATCCGGCCCTGAAGTCCCGTGCCAGCATCACCAAGGACACCTCAAAGAGCCAAGTATATCTGACGCTGAACAG
CCTGACAGGCGAGGACACGGCTGTCTATTACTGTGCGAGCCATTCATTGTCTGGTTGGTATGCTGGTGGGGATGAGA
ATTACTGGGGCCAGGGCATCCTGGTCTTCGTCTCCTCAGAGAGTACGATGACCCCAGATCTCTTCCCCCTCGTCTCC
TGTGGGCCCTCTCTTGATGAGAGCCTGGTGGCTGTGGGCTGCCTAGCCCGGGACTTCCTACCCAAG

>HM176076.1 Equus caballus clone 2H559 immunoglobulin heavy chain V-D-J region mRNA, partial cds
ATGAATCACCTGTGGTTCTTCCTCTTTCTGGTGGCCGCTCCTACATGTGTCCTGTCCCAGGTGCAACTGAAGGAGTC
AGGACCTGGCCTGGTGAAGCCCTCGCAGACCCTCTCCCTCACCTGCACTGTCTCTGGATTATCTTTGAATAGTTATG
CTGTAGGCTGGGTCCGCCAGGCTCCAGAAAAAGGGCTGGAATATGTCGGTGTAATATATGGTACGCAAAGTCCAGTC
TATAATCCGGCCCTGAAGTCCCGTGCCAGCATCACCAAGGACACCTCAAAGAGCCAAGTATATCTGACGCTGAACAG
CCTGACAGGCGAGGACACGGCTGTCTATTACTGTGCGAGCCATTCATTGTCTGGTTGGTATGCTGGTGGGGATGAGA
ATTACTGGGGCCAGGGCATCCTGGTCTTCGTCTCCTCAGAGAGTACGATGACCCCAGATCTCTTCCCCCTCGTCTCC
TGTGGGCCCTCTCTTGATGAGAGCCTGGTGGCTGTGGGCTGCCTAGCCCGGGACTTCCTACCCAAG
>HM176075.1 Equus caballus clone 2H501 immunoglobulin heavy chain V-D-J region mRNA, partial cds
ATGAATCACCTGTGGTTCTTCCTCTTTCTGGTGGCCGCTCCTACATGTGTCCTGTCCCAGGTGCAACTGAAGGAGTC
AGGACCTGGCCTGGTGAAGCCCTCGCAGACCCTCTCCCTCACCTGCACTGTCTCTGGATTATCTTTGAATAGTTATG
CTGTAGGCTGGGTCCGCCAGGCTCCAGAAAAAGGGCTGGAATATGTCGGTGTAATATATGGTACGCAAAGTCCAGTC
TATAATCCGGCCCTGAAGTCCCGTGCCAGCATCACCAAGGACACCTCAAAGAGCCAAGTATATCTGACGCTGAACAG
CCTGACAGGCGAGGACACGGCTGTCTATTACTGTGCGAGCCATTCATTGTCTGGTTGGTATGCTGGTGGGGATGAGA
ATTACTGGGGCCAGGGCATCCTGGTCTTCGTCTCCTCAGAGAGTACGATGACCCCAGATCTCTTCCCCCTCGTCTCC
TGTGGGCCCTCTCTTGATGAGAGCCTGGTGGCTGTGGGCTGCCTAGCCCGGGACTTCCTACCCAAG
>HM176074.1 Equus caballus clone 2H300 immunoglobulin heavy chain V-D-J region mRNA, partial cds
ATGAATCACCTGTGGTTCTTCCTCTTTCTGGTGGCCGCTCCTACATGTGTCCTGTCCCAGGTGCAACTGAAGGAGTC
AGGACCTGGCCTGGTGAAGCCCTCGCAGACCCTCTCCCTCACCTGCACTGTCTCTGGATTATCTTTGAATAGTTATG
CTGTAGGCTGGGTCCGCCAGGCTCCAGAAAAAGGGCTGGAATATGTCGGTGTAATATATGGTACGCAAAGTCCAGTC
TATAATCCGGCCCTGAAGTCCCGTGCCAGCATCACCAAGGACACCTCAAAGAGCCAAGTATATCTGACGCTGAACAG
CCTGACAGGCGAGGACACGGCTGTCTATTACTGTGCGAGCCATTCATTGTCTGGTTGGTATGCTGGTGGGGATGAGA
ATTACTGGGGCCAGGGCATCCTGGTCTTCGTCTCCTCAGAGAGTACGATGACCCCAGATCTCTTCCCCCTCGTCTCC
TGTGGGCCCTCTCTTGATGAGAGCCTGGTGGCTGTGGGCTGCCTAGCCCGGGACTTCCTACCCAAG
>HM176073.1 Equus caballus clone 2H161 immunoglobulin heavy chain V-D-J region mRNA, partial cds
ATGAATCACCTGTGGTTCTTCCTCTTTCTGGTGGCCGCTCCTACATGTGTCCTGTCCCAGGTGCAACTGAAGGAGTC
AGGACCTGGCCTGGTGAAGCCCTCGCAGACCCTCTCCCTCACCTGCACTGTCTCTGGATTATCTTTGAATAGTTATG
CTGTAGGCTGGGTCCGCCAGGCTCCAGAAAAAGGGCTGGAATATGTCGGTGTAATATATGGTACGCAAAGTCCAGTC
TATAATCCGGCCCTGAAGTCCCGTGCCAGCATCACCAAGGACACCTCAAAGAGCCAAGTATATCTGACGCTGAACAG
CCTGACAGGCGAGGACACGGCTGTCTATTACTGTGCGAGCCATTCATTGTCTGGTTGGTATGCTGGTGGGGATGAGA
ATTACTGGGGCCAGGGCATCCTGGTCTTCGTCTCCTCAGAGAGTACGATGACCCCAGATCTCTTCCCCCTCGTCTCC
TGTGGGCCCTCTCTTGATGAGAGCCTGGTGGCTGTGGGCTGCCTAGCCCGGGACTTCCTACCCAAG
>HM176072.1 Equus caballus clone 2H128 immunoglobulin heavy chain V-D-J region mRNA, partial cds
ATGAATCACCTGTGGTTCTTCCTCTTTCTGGTGGCCGCTCCTACATGTGTCCTGTCCCAGGTGCAACTGAAGGAGTC
AGGACCTGGCCTGGTGAAGCCCTCGCAGACCCTCTCCCTCACCTGCACTGTCTCTGGATTATCTTTGAATAGTTATG
CTGTAGGCTGGGTCCGCCAGGCTCCAGAAAAAGGGCTGGAATATGTCGGTGTAATATATGGTACGCAAAGTCCAGTC
TATAATCCGGCCCTGAAGTCCCGTGCCAGCATCACCAAGGACACCTCAAAGAGCCAAGTATATCTGACGCTGAACAG
CCTGACAGGCGAGGACACGGCTGTCTATTACTGTGCGAGCCATTCATTGTCTGGTTGGTATGCTGGTGGGGATGAGA
ATTACTGGGGCCAGGGCATCCTGGTCTTCGTCTCCTCAGAGAGTACGATGACCCCAGATCTCTTCCCCCTCGTCTCC
TGTGGGCCCTCTCTTGATGAGAGCCTGGTGGCTGTGGGCTGCCTAGCCCGGGACTTCCTACCCAAG
>HM176071.1 Equus caballus clone 2H1105 immunoglobulin heavy chain V-D-J region mRNA, partial cds
ATGAATCACCTGTGGTTCTTCCTCTTTCTGGTGGCCGCTCCTACATGTGTCCTGTCCCAGGTGCAACTGAAGGAGTC
AGGACCTGGCCTGGTGAAGCCCTCGCAGACCCTCTCCCTCACCTGCACTGTCTCTGGATTATCTTTGAATAGTTATG
CTGTAGGCTGGGTCCGCCAGGCTCCAGAAAAAGGGCTGGAATATGTCGGTGTAATATATGGTACGCAAAGTCCAGTC
TATAATCCGGCCCTGAAGTCCCGTGCCAGCATCACCAAGGACACCTCAAAGAGCCAAGTATATCTGACGCTGAACAG
CCTGACAGGCGAGGACACGGCTGTCTATTACTGTGCGAGCCATTCATTGTCTGGTTGGTATGCTGGTGGGGATGAGA
ATTACTGGGGCCAGGGCATCCTGGTCTTCGTCTCCTCAGAGAGTACGATGACCCCAGATCTCTTCCCCCTCGTCTCC
TGTGGGCCCTCTCTTGATGAGAGCCTGGTGGCTGTGGGCTGCCTAGCCCGGGACTTCCTACCCAAG

>HM176070.1 Equus caballus clone 2H1012 immunoglobulin heavy chain V-D-J region mRNA, partial cds
ATGAATCACCTGTGGTTCTTCCTCTTTCTGGTGGCCGCTCCTACATGTGTCCTGTCCCAGGTGCAACTGAAGGAGTC
AGGACCTGGCCTGGTGAAGCCCTCGCAGACCCTCTCCCTCACCTGCACTGTCTCTGGATTATCTTTGAATAGTTATG
CTGTAGGCTGGGTCCGCCAGGCTCCAGAAAAAGGGCTGGAATATGTCGGTGTAATATATGGTACGCAAAGTCCAGTC
TATAATCCGGCCCTGAAGTCCCGTGCCAGCATCACCAAGGACACCTCAAAGAGCCAAGTATATCTGACGCTGAACAG
CCTGACAGGCGAGGACACGGCTGTCTATTACTGTGCGAGCCATTCATTGTCTGGTTGGTATGCTGGTGGGGATGAGA
ATTACTGGGGCCAGGGCATCCTGGTCTTCGTCTCCTCAGAGAGTACGATGACCCCAGATCTCTTCCCCCTCGTCTCC
TGTGGGCCCTCTCTTGATGAGAGCCTGGTGGCTGTGGGCTGCCTAGCCCGGGACTTCCTACCCAAG
>HM176069.1 Equus caballus clone 2H1008 immunoglobulin heavy chain V-D-J region mRNA, partial cds
ATGAATCACCTGTGGTTCTTCCTCTTTCTGGTGGCCGCTCCTACATGTGTCCTGTCCCAGGTGCAACTGAAGGAGTC
AGGACCTGGCCTGGTGAAGCCCTCGCAGACCCTCTCCCTCACCTGCACTGTCTCTGGATTATCTTTGAATAGTTATG
CTGTAGGCTGGGTCCGCCAGGCTCCAGAAAAAGGGCTGGAATATGTCGGTGTAATATATGGTACGCAAAGTCCAGTC
TATAATCCGGCCCTGAAGTCCCGTGCCAGCATCACCAAGGACACCTCAAAGAGCCAAGTATATCTGACGCTGAACAG
CCTGACAGGCGAGGACACGGCTGTCTATTACTGTGCGAGCCATTCATTGTCTGGTTGGTATGCTGGTGGGGATGAGA
ATTACTGGGGCCAGGGCATCCTGGTCTTCGTCTCCTCAGAGAGTACGATGACCCCAGATCTCTTCCCCCTCGTCTCC
TGTGGGCCCTCTCTTGATGAGAGCCTGGTGGCTGTGGGCTGCCTAGCCCGGGACTTCCTACCCAAG
>HM176068.1 Equus caballus clone 2H948 immunoglobulin heavy chain V-D-J region mRNA, partial cds
ATGAATCACCTGTGGTTCTTCCTCTTTCTGGTGGCCGCTCCTACATGTGTCCTGTCCCAGGTGCAACTGAAGGAGTC
AGGACCTGGCCTGGTGAAGCCCTCGCAGACCCTCTCCCTCACCTGCACTGTCTCTGGATTATCTTTGAATAGTTATG
CTGTAGGCTGGGTCCGCCAGGCTCCAGAAAAAGGGCTGGAATATGTCGGTGTAATATATGGTACGCAAAGTCCAGTC
TATAATCCGGCCCTGAAGTCCCGTGCCAGCATCACCAAGGACACCTCAAAGAGCCAAGTATATCTGACGCTGAACAG
CCTGACAGGCGAGGACACGGCTGTCTATTACTGTGCGAGCCATTCATTGTCTGGTTGGTATGCTGGTGGGGATGAGA
ATTACTGGGGCCAGGGCATCCTGGTCTTCGTCTCCTCAGAGAGTACGATGACCCCAGATCTCTTCCCCCTCGTCTCC
TGTGGGCCCTCTCTTGATGAGAGCCTGGTGGCTGTGGGCTGCCTAGCCCGGGACTTCCTACCCAAG
>HM176067.1 Equus caballus clone 2H278 immunoglobulin heavy chain V-D-J region mRNA, partial cds
ATGAATCACCTGTGGTTCTTCCTCTTTCTGGTGGCCGCTCCTACATGTGTCCTGTCCCAGGTGCAACTGAAGGAGTC
AGGACCTGGCCTGGTGAAGCCCTCGCAGACCCTCTCCCTCACCTGCACTGTCTCTGGGTTATCTTTGAATAGTTATG
CTGTAGGCTGGGTCCGCCAGGCTCCAGAAAAAGGGCTGGAATATGTCGGTGTAATATATGGTACGCAAAGTCCAGTC
TATAATCCGGCCCTGAAGTCCCGTGCCAGCATCACCAAGGACACCTCAAAGAGCCAAGTATATCTGACGCTGAACAG
CCTGACAGGCGAGGACACGGCTGTCTATTACTGTGCGAGCCATTCATTGTCTGGTTGGTATGCTGGTGGGGATGAGA
ATTACTGGGGCCAGGGCATCCTGGTCTTCGTCTCCTCAGAGAGTACGATGACCCCAGATCTCTTCCCCCTCGTCTCC
TGTGGGCCCTCTCTTGATGAGAGCCTGGTGGCTGTGGGCTGCCTAGCCCGGGACTTCCTACCCAAG
>HM176066.1 Equus caballus clone 2H1350 immunoglobulin heavy chain V-D-J region mRNA, partial cds
ATGAATCACCTGTGGTTCTTCCTCTTTCTGGTGGCCGCTCCTACATGTGTCCTGTCCCAGGTGCAACTGAAGGAGTC
AGGACCTGGCCTGGTGAAGCCCTCGCAGACCCTCTCCCTCACCTGCACTGTCTCTGGATTATCTTTGAATAGTTATG
CTGTAGGCTGGGTCCGCCAGGCTCCAGAAAAAGGGCTGGAATATGTCGGTGTAATATATGGTACGCAAAGTCCAGTC
TATAGTCCGGCCCTGAAGTCCCGTGCCAGCATCACCAAGGACACCTCAAAGAGCCAAGTATATCTGACGCTGAACAG
CCTGACAGGCGAGGACACGGCTGTCTATTACTGTGCGAGCCATTCATTGTCTGGTTGGTATGCTGGTGGGGATGAGA
ATTACTGGGGCCAGGGCATCCTGGTCTTCGTCTCCTCAGAGAGTACGATGACCCCAGATCTCTTCCCCCTCGTCTCC
TGTGGGCCCTCTCTTGATGAGAGCCTGGTGGCTGTGGGCTGCCTAGCCCGGGACTTCCTACCCAAG
>HM176064.1 Equus caballus clone 2H154 immunoglobulin heavy chain V-D-J region mRNA, partial cds
ATGAATCACCTGTGGTTCTTCCTCTTTCTGGTGGCCGCTCCTACATGTGTCCTGTCCCAGGTGCAACTGAAGGAGTC
AGGACCTGGCCTGGTGAAGCCCTCGCAGACCCTCTCCCTCACCTGCACTGTCTCTGGATTATCTTTGAATAGTTATG
CTGTAGGCTGGGTCCGCCAGGCTCCAGAGAAAGGGCTGGAATATGTCGGTGTAATATATGGTACGCAAAGTCCAGTC
TATAATCCGGCCCTGAAGTCCCGTGCCAGCATCACCAAGGACACCTCAAAGAGCCAAGTATATCTGACGCTGAACAG
CCTGACAGGCGAGGACACGGCTGTCTATTACTGTGCGAGCCATTCATTGTCTGGTTGGTATGCTGGTGGGGATGAGA
ATTACTGGGGCCAGGGCATCCTGGTCTTCGTCTCCTCAGAGAGTACGATGACCCCAGATCTCTTCCCCCTCGTCTCC
TGTGGGCCCTCTCTTGATGAGAGCCTGGTGGCTGTGGGCTGCCTAGCCCGGGACTTCCTACCCAAG
>HM176063.1 Equus caballus clone 2H1175 immunoglobulin heavy chain V-D-J region mRNA, partial cds

ATGAATCACCTGTGGTTCTTCCTCTTTCTGGTGGCCGCTCCTACATGTGTCCTGTCCCAGGTGCAACTGAAGGAGTC
AGGACCTGGCCTGGTGAAGCCCTCGCAGACCCTCTCCCTCACCTGCACTGTCTCTGGATTATCTTTGAATAGTTATG
CTGTAGGCTGGGTCCGCCAGGCTCCAGAAAAAGGGCTGGAATATGTCGGTGTAATATATGGTACGCAAAGTCCAGTC
TATAATCCGGCCCTGAAGTCCCGTGCCAGCATCACCAAGGACACCTCGAAGAGCCAAGTATATCTGACGCTGAACAG
CCTGACAGGCGAGGACACGGCTGTCTATTACTGTGCGAGCCATTCATTGTCTGGTTGGTATGCTGGTGGGGATGAGA
ATTACTGGGGCCAGGGCATCCTGGTCTTCGTCTCCTCAGAGAGTACGATGACCCCAGATCTCTTCCCCCTCGTCTCC
TGTGGGCCCTCTCTTGATGAGAGCCTGGTGGCTGTGGGCTGCCTAGCCCGGGACTTCCTACCCAAG
>HM176062.1 Equus caballus clone 2H238 immunoglobulin heavy chain V-D-J
region mRNA, partial cds
ATGAATCACCTGTGGTTCTTCCTCTTTCTGGTGGCCGCTCCTACAAGTGTCCTGTCCCAGGTGCAACTGAAGGAGTC
AGGACCTGGCCTGGTGAAGCCCTCGCAGACCCTCTCCCTCACCTGCACTGTCTCTGGATTATCTTTGAATAGTTATG
CTGTAGGCTGGGTCCGCCAGGCTCCAGAAAAAGGGCTGGAATATGTCGGTGTAATATATGGTACGCAAAGTCCAGTC
TATAATCCGGCCCTGAAGTCCCGTGCCAGCATCACCAAGGACACCTCAAAGAGCCAAGTATATCTGACGCTGAACAG
CCTGACAGGCGAGGACACGGCTGTCTATTACTGTGCGAGCCATTCATTGTCTGGTTGGTATGCTGGTGGGGATGAGA
ATTACTGGGGCCAGGGCATCCTGGTCTTCGTCTCCTCAGAGAGTACGATGACCCCAGATCTCTTCCCCCTCGTCTCC
TGTGGGCCCTCTCTTGATGAGAGCCTGGTGGCTGTGGGCTGCCTAGCCCGGGACTTCCTACCCAAG
>HM176061.1 Equus caballus clone 2H136 immunoglobulin heavy chain V-D-J
region mRNA, partial cds
ATGAATCACCTGTGGTTCTTCCTCTTTCTGGTGGCCGCTCCTGCATGTGTCCTGTCCCAGGTGCAACTGAAGGAGTC
AGGACCTGGCCTGGTGAAGCCCTCGCAGACCCTCTCCCTCACCTGCACTGTCTCTGGATTATCTTTGAATAGTTATG
CTGTAGGCTGGGTCCGCCAGGCTCCAGAAAAAGGGCTGGAATATGTCGGTGTAATATATGGTACGCAAAGTCCAGTC
TATAATCCGGCCCTGAAGTCCCGTGCCAGCATCACCAAGGACACCTCAAAGAGCCAAGTATATCTGACGCTGAACAG
CCTGACAGGCGAGGACACGGCTGTCTATTACTGTGCGAGCCATTCATTGTCTGGTTGGTATGCTGGTGGGGATGAGA
ATTACTGGGGCCAGGGCATCCTGGTCTTCGTCTCCTCAGAGAGTACGATGACCCCAGATCTCTTCCCCCTCGTCTCC
TGTGGGCCCTCTCTTGATGAGAGCCTGGTGGCTGTGGGCTGCCTAGCCCGGGACTTCCTACCCAAG
>HM176060.1 Equus caballus clone 2H807 immunoglobulin heavy chain V-D-J
region mRNA, partial cds
ATGAATCACCTGTGGTTCTTCCTCTTTCTGGTGGCCGCTCCTACATGTGTCCTGTCCCAGGTGCAACTGAAGGAGTC
AGGACCTGGCCTGGTGAAGCCCTCGCAGACCCTCTCCCTCACCTGCACTGTCTCTGGATTATCTTTGAATAGTTATG
CTGTAGGCTGGGTCCGCCAGGCTCCAGAAAAAGGGCTGGAATATGTCGGTGTAATATATGGTACGCAAAGTCCAGTC
TATAATCCGGCCCTGAAGTCCCGTGCCAGCATCACCAAGGACACCTCAAAGAGCCAAGTATATCTGACGCTGAACAG
CCTGACAGGCGAGGACACGGCTGTCTATTACTGTGCGAGCCATTCATTGTCTGGTTGGTATGCTGGTGGGGATGAGA
ATTACTGGGGCCAGGGCATCCTGGTCTTTGTCTCCTCAGAGAGTACGATGACCCCAGATCTCTTCCCCCTCGTCTCC
TGTGGGCCCTCTCTTGATGAGAGCCTGGTGGCTGTGGGCTGCCTAGCCCGGGACTTCCTACCCAAG
>HM176059.1 Equus caballus clone 2H1384 immunoglobulin heavy chain V-D-J
region mRNA, partial cds
ATGAATCACCTGTGGTTCTTCCTCTTTCTGGTGGCCGCTCCTACATGTGTCCTGTCCCAGGTGCAACTGAAGGAGTC
AGGACCTGGCCTGGTGAAGCCCTCGCAGACCCTCTCCCTCACCTGCACTGTCTCTGGATTATCTTTGAATAGTTATG
CTGTAGGCTGGGTCCGCCAGGCTCCAGAAAAAGGGCTGGAATATGTCGGTGTAATATATGGTACGCAAAGTCCAGTC
TATAATCCGGCCCTGAAGTCCCGTGCCAGCATCACCAAGGACACCTCAAAGAGCCAAGTATATCTGACGCTGAACAG
CCTGACAGGCGAGGACACGGCTGTCTATTACTGTGCGAGCCATTCATTGTCTGGTTGGTATGCTGGTGGGGATGAGA
ATTACTGGGGCCAGGGCATCCTGGTCTTCGTCTCCTCAGAGAGTACGATGACCCCAGATCTCTTCCCCCTCGTCTCC
TGTGGGCCCTCTCTTGATGAGAGCCTGGTGGCTGTGGGCTGCCTAGCCCGGGACTTCCTACACAAG
>HM176058.1 Equus caballus clone 2H915 immunoglobulin heavy chain V-D-J
region mRNA, partial cds
ATGAATCACCTGTGGTTCTTCCTCTTTCTGGTGGCCGCTCCTACATGTGTCCTGTCCCAGGTGCAACTGAAGGAGTC
AGGACCTGGCCTGGTGAAGCCCTCGCAGACCCTCTCCCTCACCTGCACTGTCTCTGGATTATCTTTGAATAGTTATG
CTGTAGGCTGGGTCCGCCAGGCTCCAGAAAAAGGGCTGGAATATGTCGGTGTAATATATGGTACGCAAAGTCCAGTC
TATAATCCGGCCCTGAAGTCCCGTGCCAGCATCACCAAGGACACCTCAAAGAGCCAAGTATATCTGACGCTGAACAG
CCTGACAGGCGAGGACACGGCTGTCTATTACTGTGCGAGCCATTCATTGTCTGGTTGGTATGCTGGTGGGGATGAGA
ATTACTGGGGCCAGGGCATCCTGGTCTTCGTCTCCTCAGGGAGTACGATGACCCCAGATCTCTTCCCCCTCGTCTCC
TGTGGGCCCTCTCTTGATGAGAGCCTGGTGGCTGTGGGCTGCCTAGCCCGGGACTTCCTACCCAAG
>HM176057.1 Equus caballus clone 2H564 immunoglobulin heavy chain V-D-J
region mRNA, partial cds

ATGAATCACCTGTGGTTCTTCCTCTTTCTGGTGGCCGCTCCTACATGTGTCCTGTCCCAGGTGCAACTGAAGGAGTC
AGGACCTGGCCTGGTGAAGCCCTCGCAGACCCTCTCCCTCACCTGCACTGTCTCTGGATTATCTTTGAATAGTTATG
CTGTAGGCTGGGTCCGCCAGGCTCCAGAAAAAGGGCTGGAATATGTCGGTGTAATATATGGTACGCAAAGTCCAGTC
TATAATCCGGCCCTGAAGTCCCGTGCCAGCATCACCAAGGACACCTCAATGAGCCAAGTATATCTGACGCTGAACAG
CCTGACAGGCGAGGACACGGCTGTCTATTACTGTGCGAGCCATTCATTGTCTGGTTGGTATGCTGGTGGGGATGAGA
ATTACTGGGGCCAGGGCATCCTGGTCTTCGTCTCCTTAGAGAGTACGATGACCCCAGATCTCTTCCCCCTCGTCTCC
TGTGGGCCCTCTCTTGATGAGAGCCTGGTGGCTGTGGGCTGCCTAGCCCGGGACTTCCTACCCAAG
>HM176056.1 Equus caballus clone 2H13 immunoglobulin heavy chain V-D-J region mRNA, partial cds
ATGGATCACCTGTGGTTCTTCCTCTTTCTGGTGGCCGCTCCTACATGTGTCCTGTCCCAGGTGCAACTGAAGGAGTC
AGGACCTGGCCTGGTGAAGCCCTCGCAGACCCTCTCCCTCACCTGCACTGTCTCTGGATTATCTTTGAATAGTTATG
CTGTAGGCTGGGTCCGCCAGGCTCCAGAAAAAGGGCTGGAATATGTCGGTGTAATATATGGTACGCAAAGTCCAGTC
TATAATCCGGCCCTGAAGTCCCGTGCCAGCATCACCAAGGACACCTCAAAGAGCCAAGTATATCTGACGCTGAACAG
TCTGACAGGCGAGGACACGGCTGTCTATTACTGTGCGAGCCATTCATTGTCTGGTTGGTATGCTGGTGGGGATGAGA
ATTACTGGGGCCAGGGCATCCTGGTCTTCGTCTCCTCAGAGAGTACGATGACCCCAGATCTCTTCCCCCTCGTCTCC
TGTGGGCCCTCTCTTGATGAGAGCCTGGTGGCTGTGGGCTGCCTAGCCCGGGACTTCCTACCCAAG
>HM176055.1 Equus caballus clone 2H876 immunoglobulin heavy chain V-D-J region mRNA, partial cds
ATGAATCACCTGTGGTTCTTCCTCTTTCTGGTGGCCGCTCCTACATGTGTCCTGTCCCAGGTGCAACTGAAGGAGTC
AGGACCTGGCCTGGTGAAGCCCTCGCAGACCCTCTCCCTCACCTGCACTGTCTCTGGATTATCTTTGAATAGTTATG
CTGTAGGCTGGGTCCGCCAGGCTCCAGAAAAAGGGCTGGAATATGTCGGTGTAATATATGGTACGCAAAGTCCAGTC
TATAATCCGGCCCTGAAGTCCCGTGCCAGCATCACCAAGGACACCTCAAAGAGCCAAGTATATCTGACGCTGAACAG
CCTGACAGGCGAGGACACGGCTGTCTATTACTGTGCGAGCCATTCATTGTCTGGTTGGTATGCTGGTGGGGATGAGA
ATTACTGGGGCCAGGGCATCCTGGTCTTCGTCTCCTCAGAGAGTACGATGACCCCAGATCCCTTCCCCCTCGTCTCC
TGTGGGCCCTCTCTTGATGAGAGCCTGGTGGCTGTGGGCTGCCTAGCCCGGGACTTCCTACCCAAG
>HM176054.1 Equus caballus clone 2H384 immunoglobulin heavy chain V-D-J region mRNA, partial cds
ATGAATCACCTGTGGTTCTTCCTCTTTCTGGTGGCCGCTCCTACATGTGTCCTGTCCCAGGTGCAACTGAAGGAGTC
AGGACCTGGCCTGGTGAAGCCCTCGCAGACCCTCTCCCTTACCTGCACTGTCTCTGGATTATCTTTGAATAGTTATG
CTGTAGGCTGGGTCCGCCAGGCTCCAGAAAAAGGGCTGGAATATGTCGGTGTAATATATGGTACGCAAAGTCCAGTC
TATAATCCGGCCCTGAAGTCCCGTGCCAGCATCACCAAGGACACCTCAAAGAGCCAAGTATATCTGACGCTGAACAG
CCTGACAGGCGAGGACACGGCTGTCTATTACTGTGCGAGCCATTCATTGTCTGGTTGGTATGCTGGTGGGGATGAGA
ATTACTGGGGCCAGGGCATCCTGGTCTTCGTCTCCTCAGAGAGTACGATGACCCCAGATCCCTTCCCCCTCGTCTCC
TGTGGGCCCTCTCTTGATGAGAGCCTGGTGGCTGTGGGCTGCCTAGCCCGGGACTTCCTACCCAAG
>HM176053.1 Equus caballus clone 2H132 immunoglobulin heavy chain V-D-J region mRNA, partial cds
ATGAATCACCTGTGGTTCTTCCTCTTTCTGGTGGCCGCTCCTACATGTGTCCTGTCCCAGGTGCAACTGAAGGAGTC
AGGACCTGGCCTGGTGAAGCCCTCGCAGACCCTCTCCCTTACCTGCACTGTCTCTGGATTATCTTTGAATAGTTATG
CTGTAGGCTGGGTCCGCCAGGCTCCAGAAAAAGGGCTGGAATATGTCGGTGTAATATATGGTACGCAAAGTCCAGTC
TATAATCCGGCCCTGAAGTCCCGTGCCAGCATCACCAAGGACACCTCAAAGAGCCAAGTATATCTGACGCTGAACAG
CCTGACAGGCGAGGACACGGCTGTCTATTACTGTGCGAGCCATTCATTGTCTGGTTGGTATGCTGGTGGGGATGAGA
ATTACTGGGGCCAGGGCATCCTGGTCTTCGTCTCCTCAGAGAGTACGATGACCCCAGATCCCTTCCCCCTCGTCTCC
TGTGGGCCCTCTCTTGATGAGAGCCTGGTGGCTGTGGGCTGCCTAGCCCGGGACTTCCTACCCAAG
>HM176052.1 Equus caballus clone 2H688 immunoglobulin heavy chain V-D-J region mRNA, partial cds
ATGAATCACCTGTGGTTCTTCCTCTTTCTGGTGGCCGCTCCTACATGCGTCCTGTCCCAGGTGCAACTGAAGGAGTC
AGGACCTGGCCTGGTGAAGCCCTCGCAGACCCTCTCCCTCACCTGCACTGTCTCTGGATTATCTTTGAATAGTTATG
CTGTAGGCTGGGTCCGCCAGGCTCCAGAAAAAGGGCTGGAATATGTCGGTGTAATATATGGTACGCAAAGTCCAGTC
TATAATCCGGCCCTGAAGTCCCGTGCCAGCATCACCAAGGACACCTCAAAGAGCCAAGTATATCTGACGCTGAACAG
CCTGACAGGCGAGGACACGGCTGTCTATTACTGTGCGAGCCATTCATTGTCTGGTTGGTATGCTGGTGGGGATGAGA
ATTACTGGGGCCAGGGCATCCTGGTCTTCGTCTCCTCGGAGAGTACGATGACCCCAGATCTCTTCCCCCTCGTCTCC
TGTGGGCCCTCTCTTGATGAGAGCCTGGTGGCTGTGGGCTGCCTAGCCCGGGACTTCCTACCCAAG
>HM176051.1 Equus caballus clone 2H217 immunoglobulin heavy chain V-D-J region mRNA, partial cds
ATGAATCACCTGTGGCTCTTCCTCTTTCTGGTGGCCGCTCCTACATGTGTCCTGTCCCAGGTGCAACTGAAGGAGTC
AGGACCTGGCCTGGTGAAGCCCTCGCAGACCCTCTCCCTCACCTGCACTGTCTCTGGATTATCTTTGAATAGTTATG

CTGTAGGCTGGGTCCGTCAGGCTCCAGAAAAAGGGCTGGAATATGTCGGTGTAATATATGGTACGCAAAGTCCAGTC
TATAATCCGGCCCTGAAGTCCCGTGCCAGCATCACCAAGGACACCTCAAAGAGCCAAGTATATCTGACGCTGAACAG
CCTGACAGGCGAGGACACGGCTGTCTATTACTGTGCGAGCCATTCATTGTCTGGTTGGTATGCTGGTGGGGATGAGA
ATTACTGGGGCCAGGGCATCCTGGTCTTCGTCTCCTCAGAGAGTACGATGACCCCAGATCTCTTCCCCCTCGTCTCC
TGTGGGCCCTCTCTTGATGAGAGCCTGGTGGCTGTGGGCTGCCTAGCCCGGGACTTCCTACCCAAG
>HM176046.1 Equus caballus clone 1HF19 immunoglobulin heavy chain V-D-J region mRNA, partial cds
ATGAGTCACCTGTGGTTCTTCCTCTTTCTGGTGGCCGCTCCTACCTATGTCCTGTCCCAGGTGCAACTGAAGGAGTC
AGGACCTGGCCTGGTGAAGCCCCCGCAGACCCTGTCCCTCACCTGCACTGTCTCTGGATTCTCTTTGAGCAGTTACG
ATGTAGGCTGGGTCCGCCAGGCTCCAGGTAAAGGGCTGGAATTCATAGGTGGTATAGGTAAAAGTGGAAATACAGTC
TACAACCCAGCCCTGAGGTCCCGAGCCAGCATCACCGAGGACACCTCAAAGAGCCAAGTTTATCTGACGCTGAACAG
CCTGACAGGCGAGGACACGGCGGTCTATTACTGTGGGAGAGATGTTGTTGGTAATAATTGGTTTCATCCCGGCGACG
TGGATCACTGGGGCCAGGGCACCCTGGTCACCGTCTCCTCAGAGAGTACGATGACCCCAGATCTCTTCCCCCTCGTC
TCCTGTGGGCCCTCTCTTGATGAGAGCCTGGTGGCTGTGGGCTGCCTAGCCCGGGACTTCCTACCCAAG
>HM176044.1 Equus caballus clone 1HA2 immunoglobulin heavy chain V-D-J region mRNA, partial cds
ATGAGTCACCTGTGGTTCTTCCTCTCTCTGGTGGCCGCTCCTACATGTGTCCTCTCCCAGGTGCAACTGAAGGAGTC
AGGACCTGGCCTGGTGAAGCCCTCGCAGACCCTGTCACTCACCTGCACTGTCTCTGGATTCACTTTGAGCGGTTACG
GTATAGGCTGGGTCCGCCAGGCTCCAGGAAAAGGACTAGAATATGTTGGGGGAATGACTATAAGTGGAAGTGAATCC
TGGAACCCAGCCCTGAGGAACCGAGCCAGCATCACCAAGGACACCTCAGAGACCCAAGTTTATCTGACGCTGAGAAG
CCTGACAAGCGAGGACACGGCCATCTATTACTGTGCGAGATCGGAAAAGGCAACGTATGGTGGCACCTACTTATGGA
GGAATGTTGATTACTGGGGCCAGGGCATCCTGGTCACCGTCTCCTCAGAGAGTACGATGACCCCAGATCTCTTCCCC
CTCGTCTCCTGTGGGCCCTCTCTTGATGAGAGCCTGGTGGCTGTGGGCTGCCTAGCCCGGGACTTCCTACCCAAG
>HM176043.1 Equus caballus clone 1HB70 immunoglobulin heavy chain V-D-J region mRNA, partial cds
ATGAGTCACCTGTGGTTCTTCCTCTTTCTGGTGGCCGCTCCTACATGTGTCCTATCCCAGGTGCAACTGAAGGAGTC
AGGACCTGGCCTGGTGAAGCCCTCGCAGACCCTGTCCCTCACCTGCACTGTCTCTGGATTCTCTTTGAGCAGTTACA
GTGTAGGCTGGGTCCGCCAGGCTCCAGGAAAAGGGCTGGAATATGTTGGTGCTATGCGTAGTGGTGGAAGTGCGGTG
TACAACCCAGCCCTGAAGTCCCGAGCCAGCATCACCAAGGACACCTCAAAGAGCCAAGTTTATCTGACAATGAGTAG
CCTGACAGGCGAGGACACGGCCGTCTATTACTGTGCGACGACCACTTCTGACTGTAGTGATTGCTGGAACATCGATT
ATGATTACTGGGGCCAGGGCACCCTGGTCACCGTCTCCTCAGAGAGTACGATGACCCCAGATCTCTTCCCCCTCGTC
TCCTGTGGGCCCTCTCTTGATGAGAGCCTGGTGGCTGTGGGCTGCCTAGCCCGGGACTTCCCACCCAAG
>HM176041.1 Equus caballus clone 2H1014 immunoglobulin heavy chain V-D-J region mRNA, partial cds
ATGAGTCACCTGTGGTTCTTCCTCTTTCTGGTGGCCGCTCCTACATGTGTCCTGTCCCAGGTGCAACTTCAGGAGTC
AGGACCTGGCCTGGTGAAGCCCTCGCAGACCCTGTCCCTCACCTGCACTGTCTCCGGATTCTCTTTGAGCAGTTGGG
GTGTAGGCTGGGTCCGCCAGGCTCCAGGAAAAGGATTGGAACATGTTGGTATGATAGCTGCTGGTGGAAGGCCATAC
TATAACCCAGCCCTGAAGTCCCGAGCCATCATCACCAAGGACACCTCAAAGAGCGAAATTGTTCTGACGCTGAACAG
CCTGACAGGCGAGGACACGGCCGTCTATTACTGTGCGAGAGTCCTTTATAGTTCGTATGTTAGTGGACTTGACAATT
ATTATTCTGTTTTGGACCCCTGGGGCCAGGGCATTCTGGTCACCGTCTCCTCAGAGAGTACGATGACCCCAGATCTC
TTCCCCCTCGTCTCCTGTGGGCCCTCTCTTGATGAGAGCCTGGTGGCTGTGGGCCGCCTAGCCCGGGACTTCCTACC
CAAG
>HM176040.1 Equus caballus clone 1HC20 immunoglobulin heavy chain V-D-J region mRNA, partial cds
ATGAGTCGTCTGTGGTTCTTCCTCTTTCTGGTGGCCGCTCCTAGATGTGTCCGGTCCCAGGTGCAATTGAAGGAGTC
AGGACCTGGCCTGGTGAAGCCCTCGCAGACCCTCTCCCTCGTCTGCACTGTCTCTGGATTCTCTTTGAGCAGTTATT
CTGTAGGGTGGGTCCGCCAGGCTCCAGGGAAAGGGCTGGAAATTGTTGGCGCCTTACATGGTAGTAGTGGGAATTCG
TATTACAATGAAGCCCTGAAGTCCCGAGCCAGCATCACCAAGGACACCTCCCAGAACCAAGTTTATCTGACGCTGAA
CAGCCTGACAGTGGAGGACACGGGCGTCTATTACTGTGCAAAAGATCAAAACAGTATCAATTTTTATTCTGGTATAG
ATTACTGGGGTCGGGGCATCCTGGTCACCGTCTCCTCAGAGAGTACGAAGACCCCAGATCTCTTCCCCCTCGTCTCC
TGTGGGCCCTCTCTTGATGAGGGCCTGGTGGCTGTGGGCTGCCTAGCCCGGGACTTCCTACCCAAG
>HM176039.1 Equus caballus clone 1HD100 immunoglobulin heavy chain V-D-J region mRNA, partial cds
ATGAGTCACCTGTGGTTCTTCCTCTTTCTGGTGGCCGCTCCTACATGTGTCCTGTCCCAGGTGCAACTGAAGGAGTC
AGGACCTGACCTGGTGAAGCCCTCGCAGACCCTGTCCCTCACCTGCACTGTCTCTGGATTCTCTTTGAACAGTTACG

GTATATCATGGGTCCGCCAGGCTCCAGGAAAAGGACTGGAATGGGTTGGTGATATAATTGGCAGTGAAAGTGCGCGC
TACAACCCAGCCCTGAGGTCCCGAGGCAGCATCACCAAGGACACCTCAAAGAGCCAAGTTTATCTGACGCTGAACAG
CCTGACAGGCGACGACACGGCCGTCTATTACTGTGCGAGATCGTTGAATAATGATTATTTCATCCTAGATGATATAC
TCTACTGGGGCCAGGGCATCCTGGTCACCGTCGCCTCAGAGAGTACGATGACCCCAGATCTCTTCCCCCTCGTCTCC
TGTGGGCCCTCTCTTGATGAGAGCCTGGTGGCTGTGGGCTGCCTAGCCCGGGACTTCCTACCCAAG
>HM176038.1 Equus caballus clone 1HA34 immunoglobulin heavy chain V-D-J
region mRNA, partial cds
ATGAGTCACCTGTGGTTCTTCCTCTTTCTGGTGGCCGCTCCTACATGTGTCCTGTCCCAGGTCCAACTGAGGGAGTC
AGGACCTGGCCTGGTGAAGCCCTCGCAGACCCTGTCCCTCACCTGCACTGTCTCTGGATTCTCTTTGACGGGGACCG
AAGTGGGCTGGGTCCGCCAGGCTCCAGGAAAAGGGCTGGAATGTGTTGGTCGCATAACGGCTAGTGGCGGTGCAAAC
CTCAACCCAGCCCTGAGGTCCCGAGCCACAATCACCAAGGACAACTCAAGGAGCCAAGTTTATCTGACGCTGAACAG
CCTGACAGAAGAGGACACGGCCGTCTATTACTGTGCGAGAGCCGACCCTGTCTATCTTTGGGATTGGGCGGGAGGTA
TAGATTACTGGGGCCAGGGCATCCTGGTCACCGTCTCCTCAGAGAGTACGAAGACCCCAGATCTCTTCCCCCTCGTC
TCCTGTGGGCCCTCTCTTGATGAGAGCCTGGTGGCTGTGGGCTGCCTAGCCCGGGACTTCCTACCCAAG
>HM176035.1 Equus caballus clone 1HF27 immunoglobulin heavy chain V-D-J
region mRNA, partial cds
ATGAGTCACCTGTGGTTCTTCCTCTTTCTGGTGGCCGCTCCTACATGTGTCCTGTCCCAGGTGCAACTGAAGGAGTC
AGGACCTGGCCTGGTGAAGCCCTCGCAGACCCTGTCCCTCACCTGCACTGTCTCTGGATTCTCTTTGAGCAGTTACG
GTGTAGGCTGGGTCCGCCAGGCTCCAGGAAGGGGGCTGGAATGGGTTGGTGCGCTAGATAGTAGTGGAAGTAAAAAC
TACAACCCATCCCTGAAGTCCCGAGCCACCATCACCAAGGACACCTCAAAGAGCCAAGTGTATCTGACGCTGAACAG
CCTGACAGGCGAGGACACGGCCGTCTATTACTGTGCGAGGAGCTATGGCACCTGGGCTTTCGACAAGATAGCCTACT
GGGGCCAGGGAATCCTGGCCCACGTCTCCTCAGAGAGTACGATGACCCCAGATCTCTTCCCCCTCGTCTCCTGTGGG
CCCTCTCTTGATGAGAGCCTGGTGGCTGTGGGCTGCCTAGCCCGGGACTTCCTACCCAAG
>HM176033.1 Equus caballus clone 1HC36 immunoglobulin heavy chain V-D-J
region mRNA, partial cds
ATGAGTCACCTGTGGTTCTTCCTCTTTCTGGTGGCCGCTCCTACATGCGTCCTGTCCCAGGTACAACTGAAGGAATC
AGGACCTGGCCTGGTGAAGCCCTCGCAGACCCTGTCCCTCACCTGCACTGTCTCTGAATTCTCTTTGAGCGGTTTTG
GTGTAGGCTGGGTCCGCCAGACTCCAGGAAAAGGACTGGAATGGATTGGTGCCATAACTGGTAGTAGAGAAGAACGT
TACAATCCAGCCCTGAAGTCCCGAGCCGGCGTCACCAAGGACAGCTCAAAGAGCCAAGTTTATCTGACGTTGAACAG
CCTGACAGGCGAGGACACGGCCGTCTACTACTGTGCGAAATACGACTCTTATGCCGGGGGTACTCCAGACTACTGGG
GCCAGGGCATCCTGGTCACCGTCTCCTCAGAGAGTACGAAGACCCCAGATCTCTTCCCCCTCGTCTCCTGTGGGCCC
TCTCTTGATGAGAGCCTGGTGGCTGTGGGCTGCCTAGCCCGGGACTTCCTACCCAAG
>HM176029.1 Equus caballus clone 1HB5 immunoglobulin heavy chain V-D-J region
mRNA, partial cds
ATGAGTCACCTGTGGTTCTTCCTCTTTCTGGTGGCCGTTCCTACATGTGTCCTGTCCCAGGTGCAACTGAAGGAGTC
AGGACCTAGCCTGGTGAAGCCCTCGCAGACCCTGTCCCTCACCTGCACTGTCTCTGGATTCTCTTTGAGCAGTGCCG
GTGTAGGCTGGGTCCGCCAGGCTCCAGGAAAAGGGCTGGAATATGTTGGTACTGTGTCTAGTAGTGGAACAACAAAC
TACAATCCAGCCCTGAAGTCCCGATGCAGCATCACCAAGGACACCTCAAAGAGCCAAGTTTATCTGACGCTGAACAG
CCTGACAGGCGAGGACACGGCCGTCTATTACTGTGCGAGATCTCGTCCCTACAGTGGTAGTGCAGACTTTTGGGCCG
AGTTAAATGGTATAGAGTCCTGGGGCCAGGGCACCCTGGTCACCGTCTCCTCAGAGAGTACGATGACCCCAGATCTC
TTCCCCCTCGTCTCCTGTGGGCCCTCTCTTGATGAGAGCCTGGTGGCTGTGGGCTGCCTAGCCCGGGACTTCCTACC
CAAG
>HM176028.1 Equus caballus clone 1HC70 immunoglobulin heavy chain V-D-J
region mRNA, partial cds
ATGAATCACCTGTGGTTCTTCCTCTTTCTGGTGGCCGCTCCTAGATGTGTCCTGTCCCAGGTGCAACTGAAGGAGTC
AGGACCTGGCCTGGTGAAGTCCTCGCAGACCCTCTCCCTCACCTGCACTGTCTCTGGATTCTCTTTGAGCGGTTATA
GTGTAGTGTGGGTCCGCCAGGCTCCAGGAAAAGGGCTGGAATATGTTGGTGGCATAGCCGGTGGTGGGAGAGTAACA
TACAGCCCAGCCCTGCAGTCCCGAGCCAGCATCGCCAGGGACACCTCCAAGAGCCTAGTTTATCTTGCGCTGAACAG
CCTGACAGGCGAGGACACGGCCGTCTATTATTGTGCGAGAGAGTGGGACGGCGATGCAGTTGCGACAGATGATATCG
ACTACTGGGGCCAGGGCATCCTGGTCACTGTCTCGTCAGAGAGTACGAAGACCCCAGATCTCTTCCCCCTCGTCTCC
TGTGGGCCCTCTCTTGATGAGAGCCTGGTGGCTGTGGGCTGCCTAGCCCGGGACTTCCTACCCAAG
>HM176026.1 Equus caballus clone 1HF33 immunoglobulin heavy chain V-D-J
region mRNA, partial cds
ATGAGTCACCTGTGGTTCTTCCTCTTTCTGGTGGCCGCTCCTACATGTGTCCTGTCCCAGGTGCAACTGAAGGAGTC
AGGACCTGGCCTGGTGAACCCCTCGCAGACCCTGTCCCTCACCTGCACTGTCTCTGGATCCTCTTTGAGTCATAACG
GTGTAGGCTGGGTCCGCCAGGCTCCAGGAAAGGGGCTGGAATATGTTGGTGTTATAATTAGTGATGGAAGTACAGAC

TACAATCCGGCCCTGAAGTCCCGAGCCAGCATCACCAAGGACACCTCAAAGAACCAAGTTTATCTGACGCTGAACAG
CCTGACAGGCGAGGACACGGCCGTCTATTATTGTGTGAGGGATGCGAATGGTCGGAATCTTTACCTGGCGTCTGATC
TTGACTACTGGGGCCAGGGCATCCTGGTCACCGTCTCCTCAGAGAGTACGAAGACCCCAGATCTCTTCCCCCTCGTC
TCCTGTGGGCCCTCCCTTGATGAGAGCCTGGTGGCTGTGGGCTGCCTAGCCCGGGACTTCCTACCCAAG
>HM176025.1 Equus caballus clone 1HF62 immunoglobulin heavy chain V-D-J region mRNA, partial cds
ATGAGTCACCTGTGGTTCTTCCTCTTTCTGGTGGCCGCTCCTACATGTGTCCTCTCCCAGGTGCAACTGAAGGAGTC
AGGACCTGGCCTGGTGAAGCCCTCGCAGCTCCTCTCCCTCGTCTGCACTGTCTCTGGATTCTCCTTGAGCAGTTACG
GAATAGGCTGGGTCCGCCAGGCTCCGGGAAAAGGTCTGGAATGGGTTGCTGCGATATCTCCTAGTCACGCCGCAAGG
TACAATCCAGACCTGAAGTCCCGTACCAGCATCATCAAGGACACCTCCCAGAACCAGGTTTATCTGACGCTGAACAG
CCCGACAGGCGAGGACACGGCCGTCTATTACTGTGCGCGAAGTTACTACGAAATTCATTATTGGTTCGGGGGTGATA
TAGACGTCTGGGGCCAGGGCATCCTGGTCACCGTCTCCTCAGAGAGTACGAAGACCCCAGATCTCTTCCCCCTCGTC
TCCTGTGGGCCCTCTCTTGATGAGAGCCTGGTGGCTGTGGGCTGCCTAGCCCGGGACTTCCTACCCAAG
>HM176021.1 Equus caballus clone 1HF81 immunoglobulin heavy chain V-D-J region mRNA, partial cds
ATGAATCACCTGTGGTTCTTCCTCTTTCTGGTGGCCGCTCCTAGATGTGTCCTGTCCCAGGTGCAAATGAAGGAGTC
GGGACCTGGCCTGGTGAAGCCCTCGCAGACCCTGTCCCTCACCTGCACTCATTCTGGATTATCTTTGAACAGTGGTG
CTGTACACTGGATCCGCCAGGCTCCAGGAAAAGGGCTGGAGTGGGTTGGGCGTATATGGAGTGGTGAGAATACACTC
TACAACCTAGCCCTGAAGTCCCGAGCCAGCATCACCAAGGACACCTCAAAGAGCCAAGTTTATCTGACGCTGAGCAG
CCTGACAGGCGAAGACACGGCCGTCTATTACTGTGCAGGATCCCAAGGCTCTAGTAGTTACTGGGATATACTCTACT
GGGGCCAGGGCATCCTGGTCACCGTCTCCTCAGAGAGTACGAAGACCCCAGATCTCTTCCCCCTCGTCTCCTGTGGG
CCCTCTCTCGATGAGAGCCTGGTGGCTGTGGGCTGCCTAGCCCGGGACTTCCTACCCAAG
>HM176020.1 Equus caballus clone 1HB68 immunoglobulin heavy chain V-D-J region mRNA, partial cds
ATGAGTCACCTGTGGTTCTTCCTCTTTCTGGTGGCCGCTCCTACATGTGTCCTGTCCCAGGTGCAACTGAAGGAGTC
AGGACCTGGCCTGGTGAAGCCCTCGCAGACCCTGCCCCTCACCTGCACTGTCTCTGGATTCTCTTTGAGCAGTGACG
ATGTAGGCTGGGTCCGCCAGGCTCCAGGAAAAGGTCTGGAGCGTGTGGGTGAAATAACCAGTAGTGGAAGTGCAAAC
TACAACCCAGCCCTGAAGTCCCGAGCCAGCATCACCAAGGACATCTCAAAGAGCCATGTTTATCTGACGCTGAACAG
CCTGACAGGCGAGGACACGGCCGTCTATTACTGTGTGAGAGACGAAGATCACTATCTCTCCATGGATGGTATAATGT
ACTGGGGCCAGGGCATCCTGGTCACCGTCTCCTCAGAGAGTACGATGACCCCAGATCTCTTCCCCCTCGTCTCCTGT
GGGCCCTCTCTTGATGAGAGCCTGGTGGCTGTGGGCTGCCTAGCCCGGGACTCCCTACCCAAG
>HM176016.1 Equus caballus clone 1HA55 immunoglobulin heavy chain V-D-J region mRNA, partial cds
ATGAGTCACCTGTGGTTCTTCCTCTTTCTGGTGGCCGCTCCTACATGTGTTCTGTCCCAGGTGCAACTGAAGGAGTC
AGGACCTGGCCTGGTGAAGCCCTCGCAGACCCTGTCCCTCACCTGCACTGTCTCTGGATTCTCTTTGAGCAATTACG
GTGTACACTGGGTCTGCCAGGCTCCAGGAAAAGGGCTGGAATATGTTGGTATTACGGCTATTGGTGGCACTGCAGAC
CTCAACCCAGCCCTGAAGTCCCGAGTCAGCATCACCAGGGACACCTCAAAGAGCCTAGTTATTCTGACGCTGAACAG
CCTGACAGGCGAGGACACGGCCGTCTATTACTGTGCGTGGGACAATTGGTATGGTAGTAGTTTCGGGTATAGTGGAG
TTGGTAGTATAGACTACTGGGGCCAGGGCATCCTGGTCACCGTCTCCTCAGAGAGTACGAAGACCCCAGATCTCTTC
CCCCTCGTCTCCTGTGGGCCCTCTCTTGATGAGAGCCTGGTGGCTGTGGGCTGCCTAGCCCGGGACTTCCTACCCAA
G
>HM176014.1 Equus caballus clone 1HF78 immunoglobulin heavy chain V-D-J region mRNA, partial cds
ATGAGTCACCTGTGGTTCTTCCTCTTTCTGGTGGCCGCTCCTACATGTGTCCTGTCCCAGGTTCAACTGAAGGAGTC
AGGACCTGGGCTGGTGAAGCCCTCGCAGACCCTGTCCCTCACCTGTACTGTCTCTGGATTCTCTTTGAGCAGTTACG
GTGTAGGGTGGTTCCGCCAGGCTCCAGGAAAAGGGCTGGAATGGGTTGGTGGTCCACGTGGTGGTGTAGGTATGGTG
TCGAACCCAGCCCTGAGGTCCCGAACCAGTATCACCAAGGACACCTCAAAGAGTCAAGTTTATCTGACGCTGAGCAG
CCTGACAGGCGAGGACACGGCCGTCTATTACTGTGCGAGAAGTGATTGCGCAAATGGTGTCTGGTACTGTGGTGGTA
TAATGCACTGGGGCCAGGGCATCCTGGTCACCGTCTCCTCAGAGAGTACGATGACCCCAGATCTCTTCCCCCTCGTC
TCCTGTGGGCCCTCTCTTGATGAGAGCCTGGTGGCTGTGGGCTGCCTAGCCCGGGACTTCCTACCCAAG
>HM176013.1 Equus caballus clone 1HF1 immunoglobulin heavy chain V-D-J region mRNA, partial cds
ATGAGTCCCCTGTGGTTCTTCCTCTTTCTGGTGGCCGCTCCTACATGTGTCCTGTCCCAGGTGCAACTGAAGGAGTC
AGGACCTGGCCTGGTGAAGCCCTCGCAGACCCTCTCCCTCGTCTGCACTGTCGCTGGATTTTCTTTGAGCAGTTATG
GTGTGGGCTGGGTCCGCCAGGCTCCAGGAAAGGGACCGGAATGTGTTGGTGGTATGGGAGGAAGTGTAGACTACAAC

CCAACCCTGAAGTCCCGAGCCAGCATCACCAAGGACACCTCGCAGAGCCAAGTTTATCTGACGCTGAACAGCCTGAC
AAGTGAGGACACGGCCGTCTATTACTGTGCAAGAGATAACGACCTTTTGGTAGATGCTGCAGCTATACTGGACTGGG
GCCAGGGCATCCTGGTCACCGTCTCCTCAGAGAGTACGATGACCCCAGATCTCTTCCCCCTCGTCTCCTGTGGGCCC
TCTCTTGATGAGAGCCTGGTGGCTGTGGGCTGCCTAGCCCGGGACTTCCTACCCAAG
>HM176011.1 Equus caballus clone 1HE82 immunoglobulin heavy chain V-D-J
region mRNA, partial cds
ATGAGTCACCTGTGGTTCTTCCTCTTTCTGGTGGCCGCTCCTACATGTGTCCTGTCCCAGGTGCAACTGAAGGAGTC
AGGACCTGGCCTGGTGAAGCCCTCGCAGACCCTCTCCCTCACCTGCACTGTCTCTGGATTATCTTTGACGACTTATG
CTGTGGGCTGGGTCCGCCAGGCTCCAGGAAAAGGGCTGGAATTTGTTGGTGGCATAGCTGGTAGTGGAGGTGCAGAC
TACAACCCAGCCCTGAAGTCCCGAGCCAGAATCACCAAGGACACCTCAGAGAGCCAAGTTTATCTGACGCTGAACAC
GCTGACAGACGAAGACACGGCCGTCTATTACTGTGCAGGAATTAATGGTGACAGTACGCTGGGTAATATTAACTACT
GGGGCCAGGGCATTCTGGTCACCGTCTCCTCAGAGAGTACGATGACCCCAGATCTCTTCCCCCTCGTCTCCTGTGGG
CCCTCTCTTGATGAGAGCCTGGTGGCTGTGGGCTGCCTAGCCCGGGACTTCCTACCCAAG
>HM176009.1 Equus caballus clone 1HF6 immunoglobulin heavy chain V-D-J region
mRNA, partial cds
ATGAATCACCTGTGGTTCTTCCTCTTTCTGGTGGCCGCTCCTAGATGTGTCCTGTCCCAGGTGGAACTGAAGGAGTC
AGGACCTGGCCTGGTGAAGCCCTCGCAGACCCTCTCCCTCACCTGCACTGTCTCTGGATTCTCTTTGAGCAGTTATG
CTGTGGGCTGGGTCCGCCAGGCTCCAGGAAAAGGGCTGGAATATGTTGGTGGAATATTTGGTAGTGCAAGTGTAAAG
TACAACCCAGCCCTGAAGTCCCGAGCCAGCATCACCCAGGACGTCTCCAAGAGCCAAGTTTATCTGACGCTGAACAG
CCTGACAGGCGAGGACACGGCCGTCTATTACTGTGCGAAAGGGGAAGACAACTATGTGTTCTGCGATGGTATAGTAG
ATTGGGGCCAGGGCCTCCTGGTCACCGTCTCCTCAGAGAGTACGAAGACCCCAGATCTCTTCCCCCTCGTCTCCTGT
GGGCCCTCTCTTGATGAGAGCCTGGTGGCTGTGGGCTGCCTAGCCCGGGACTTCCTACCCAAG
>HM176008.1 Equus caballus clone 1HA85 immunoglobulin heavy chain V-D-J
region mRNA, partial cds
ATGAATCACCTGTGGTTCTTCCTCTTTCTGGTGGCCGCTCCTAGATGTGTCCTGTCCCAGGTGCAACTGAAGGAGTC
AGGACCTGGCCTGGTGAAGCCCTCGCAGACCCTCTCCCTCACCTGCACCGTCTCTGGATTCTCTTTGAGCAGTTATG
CTGTAGGCTGGGTCCGCCAGGCTCCAGGAAAAGGGCTGGAGTGGATTGGTGGTCCATTCGGTGGATCAAGTATGAAC
ATTAATCCACTCCTGAAGTCCCGAACCACAATCACCGTGGACACCTCCAAGAGCCAAGTGCTTTTGACGCTGAATAG
CCTGACAGGCGAGGACACGGCCGTCTATTACTGTGCGAAAGATACGAACGCATACTGGAAATCGAATGGTATAGACT
ACTGGGGCCAGGGCATCCTGGTCACCGTCTCCTCAGAGAGTACGAAGACCCCAGATCTCTTCCCCCTCGTCTCCTGT
GGGCCCTCTCTTGATGAGAGCCTGGTGGCTGTGGGCTGCCTAGCCCGGGACTTCCTACCCAAG
>HM176007.1 Equus caballus clone 1HC18 immunoglobulin heavy chain V-D-J
region mRNA, partial cds
ATGAGTCACCTGTGGTTCTTCCTCTTTCTGGTGGCCGCTCCTACATGTGTCCTGTCCCAGGTGCAACTGAAGGAGTC
AGGACCTGGCCTGGTGAAGCCCTCGCAGACCCTCTCCCTCACCTGCACTGTCTCTGGATTATCTTTGAGCAGTTTCG
GTGTGGGCTGGGTCCGCCAGGCTCCAGGAAAAGGGCTGGAATTTGTTGGTGGTATGGTGACCAGTGGAAGTACTGAC
CACAACCCAGCCCTTAAGTCCCGAGCCAGCATCACCAGGGACGCCTCAAAGAGCCAAGTTTATCTGTCGCTGAACAG
TCTGACAAGCGAGGACACGGCCGTCTACTACTGTGCGGTTGTGGGCTGGCGTGCGGTAATCTGGTGGGGCCAGGGCA
TCCTCGTCACCGTCTCCTCGGAGAGTACGATGACCCCAGATCTCTTCCCCCTCGTCTCCTGTGGGCCCTCTCTTGAT
GAGAGCCTGGTGGCTGTGGGCTGCCTAGCCCGGGACTTCCTACCCAAG
>HM176005.1 Equus caballus clone 2H1082 immunoglobulin heavy chain V-D-J
region mRNA, partial cds
ATGAATCACCTGTGGTTCTTCCTCTTTCTGGTGGCCGCTCCTACATGTGTCCTGTCCCAGGTGCAACTGAAGGAGTC
AGGACCTGGCCTGGTGGAGCCCTCGCAGACCCTCTCCCTCACCTGCACTGTCTCTGGATTATCTTTGAATAGTTATG
CTGTAGGCTGGGTCCGCCAGGCTCCAGAAAAAGGGCTGGAATATGTCGGTGTAATATATGGTACGCAAAGTCCAGTC
TATAATCCGGCCCTGAAGTCCCGTGCCAGCATCACCAAGGACACCTCAAAGAGCCAAGTATATCTGACGCTGAACAG
CCTGACAGGCGAGGACACGGCTGTCTATTACTGTGCGAGCCATTCATTGTCTGGTTGGTATGCTGGTGGGGATGAGA
ATTACTGGGGCCAGGGCATCCTGGTCTTCGTCTCCTCAGAGAATACGATGACCCCAGATCTCTTCCCCCTCGTCTCC
TGTGGGCCCTCTCTTGATGAAAGCCTGGTGGCTGTGGGCTGCCTAGCCCGGGACTTCCTACCCAAG
>HM176003.1 Equus caballus clone 2H87 immunoglobulin heavy chain V-D-J region
mRNA, partial cds
ATGAGTCACCTGTGGTTCTTCCTCTTTCTGGTGGCCGCTCCTACATGTGTCCTGTCCCAGGTGCAACTGAAGGAGTC
AGGACCTGGCCTGGTGAAGCCCCCGCAGACCCTGTCCCTCACCTGCACTGTCTCTGGATTCTCTTTGAGCGGTTACC
CGGTAGTCTGGGTCCGCCAGGCTCCAGGAAAAGGGCTGGACTATGTGGGTCTTATACATGGTGCTGGAAATACGGTC
TACAACCCAGCCCTGAAGTCCCGAGCCAGCATCACCAGGGACACCTCAAAGAGCCAAGTTTATCTGACGCTGAACAG
CCTGACAGGCGAGGACACGGCCGTCTATTACTGTGCGAGATCCGCCACTGCTTTGTATGGTGGTAATTGGTATGTGG

ATGGTAGTGACTACTGGGGCCAGGGCATCCTGGTCACCGTCTCCTCAGAGAGTACGATGACCCCAGATCTCTTCCCC
CTCGTCTCCTGTGGGCCCTCTCTTGATGAGAGCCTGGTGGCTGTGGGCTGCCTAGCCCGGGACTTCCTACCCAAG
>HM176000.1 Equus caballus clone 1HC28 immunoglobulin heavy chain V-D-J region mRNA, partial cds
ATGAGTCACCTGTGGTTCTTCCTCTTTCAGGTGGCCGCTCCTACATGTGTCCTGTCCCAGGTGCAACTGAAGGAGTC
AGGACCTGGCCTGGTGAAGCCCTCGCAGACCCTGTCCCTCACCTGCACTGTGTCTGGATTCTCTTTGAGCGGTTACG
ATGTAGGCTGGGTCCGCCAGGCTCCAGGAAAAGGGCTGGAATATGTTGGTGAGATAACTAGGAGCGGAAGTGCAAAC
TACAACCCAGCCCTGAAGTCCCGAGCCAGCATCACCAAGGACACCTCAAAGAGCCAAGTTTATCTGACGCTAGACAG
CCTGACAGGCGAGGACACGGCCGTCTATTACTGTGCGAGACACTATATGGATAGTTTTTTGTTTGAGTACTGGGGCC
AGGGCACCCTGGTCACCGTCTCCTCAGAGAGTACGATGACCCCAGATCTCTTCCCCCTCGTCTCCTGTGGGCCCTCT
CTTGATGAGAGCCTGGTGGCTGTGGGCTGCCTAGCCCGGGACTTCCTACCCAAG
>HM175999.1 Equus caballus clone 1HA92 immunoglobulin heavy chain V-D-J region mRNA, partial cds
ATGAGTCACCTGTGGTTCTTCCTCTTTCTGGTGGCCGCTCCTACATGTGTCCTGTCCCAGGTGCAACTGAAGGAGTC
AGGACCTGGCCTGGTGAAGCCCTCGCAGACCCTGTCCCTCATCTGCACTGTCTCTGGATTTTCTTTGAGCAGTGACG
GGGTAGGCTGGGTCCGCCAGGCTCCAGGAAAAGGGCTGGAATGGGTTGGTGTTAGTCGGAGTAGTGGAAGTGTAAAC
TACAATCCAGCCCTGAAGTCCCGAGCCAGCATCACCAAGGACACCTCAAAGAGCCAAGTTTATCTGACGCTGAACAG
CCTGACAGGCGAGGACACGGCCGTCTATTACTGTTTCGGTTACCGTGGTGGTGGAGTTGTTCTTAGTACCTTTGAGA
CCTACTGGGGCCAGGGCACCCTGGTCACCGTCTCCTCAGAGAGTACGAAGACCCCAGATCTCTTCCCCCTCGTCTCC
TGTGGGCCCTCTCTTGATGAGAGCCTGGTGGCTGTGGGCTGCCTAGCCCGGGACTTCCTACCCAAG
>HM175998.1 Equus caballus clone 1HB94 immunoglobulin heavy chain V-D-J region mRNA, partial cds
ATGAGTCACCTGTGGTTCTTCCTCTTTCTGGTGGCCGCTCCTACATGTGTCCTGTCCCAGGTGCAACTGAAGGAGTC
AGGACCTGGCCTGGTGAAGCCCTCGCAGACCCTGTCCCTCACCTGCACTGTCTCTGGATTCTCTTTGAGTAGTTACG
CTGTAGGGTGGGTCCGCCAGGCTCCAGGAAAAGGTCTACAATATGTTGGCACTATACGTATGGATGGAAGTACCTAC
TACAACCCAGACCTGAAGTCCCGAGCCAGCATCACCAAGGACACCTCAAACAGTCAGGTGTATCTGACGCTGAACAG
TCTGACGGGCGAGGACGCGGCCTTCTATTATTGTACGAGGGTCCTAGATGGTGGCGATTGGTGGTTCCCCTTTGGCT
ACTGGGGCCAGGGCACCCTGGTCACCGTCTCCTCAGAGAGTACGATGACCCCAGATCTCTTCCCCCTCGTCTCCTGT
GGGCCCTCTCTTGATGAGAGCCTGGTGGCTGTGGGCTGCCTAGCCCGGGACTTCCTACCCAAG
>HM175996.1 Equus caballus clone 1HB3 immunoglobulin heavy chain V-D-J region mRNA, partial cds
ATGAATCACCTGTGGTTCTTCCTCTTTCTGGTGGCCGCTCCTACATGTGTTCTGTCCCAGGTGCAACTGAAGGAGTC
AGGACCTGGCCTGGTGAAGCCCTCGCAGACCCTCTCCCTCACCTGCACTGTCTCTGGAGTCTCTTTGAGCAGTTATG
CTGTGTTTTGGGTCCGCCAGGCTCCAGGAAAAGGGCTGGAATTCGTTGGTAGGATACGCGTTAGTGGAACTATATAT
TACAACCCGGCCCTGAAGTCCCGAGCCAGTATCACCAAGGACACCTCAAAGAGCCAAGTTTATCTGACGCTGAACAG
CCTGACAGGCGAGGACACGGCCGTCTATTACTGTGCGAGCAGTGGAGACGCTGACAATAATTTCTCAAATTATGCCT
ACTGGGGCCAGGGCATCCTGGTCACCGTCTCCTCAGAGAGTACGATGACCCCAGATCTCTTCCCCCTCGTCTCCTGT
GGGCCCTCTCTTGATGAGAGCCTGGTGGCTGTGGGCTGCCTAGCCCGGGACTTCCTACCCAAG
>HM175991.1 Equus caballus clone 1HF36 immunoglobulin heavy chain V-D-J region mRNA, partial cds
ATGAGTCACCTGTGGTTCTTCCTCTTTCTGGTGGCCGCTCCTACATGTGTCCTGTCTCAGGTGACACTGAAGGAGTC
CGGACCTGGCCTGGTGAAGCCCTCACAGACCCTCTCCCTCACCTGCATTGTCTCTGGATTATCTTGGAACAATATTG
CTGTAGGCTGGGTCCGCCAGGCTCCAGGAAAAGGGCTGGAATTTGTTGGTGGTATAGGTGGAAGTGGAATTGCAAAC
TACAACCCAGCCCTGAAGTCCCGAGCCAGCATCACCAAGGACACCTCAAAGAGCCAAGTTTACCTGACGCTGAACAG
CCTGACAAGCGAGGACACGGCCGTCTATTACTGTACAGGAGGCTATGCCGGGACATATGTCATTGGCAGAATGGAAC
ACTGGGGCCAGGGCACCCTGGTCACCGTGTCCTCAGAGAGTACGATGACCCCAGATCTCTTCCCCCTCGTCTCCTGT
GGGCCCTCTCTTGATGAGAGCCTGGTGGCTGTGGGCTGCCTAGCCCGGGACTTCCTACCCAAG
>HM175985.1 Equus caballus clone 1HF40 immunoglobulin heavy chain V-D-J region mRNA, partial cds
ATGAGTCACCTGTGGTTCTTCCTCTTTCTGGTGGCCGCTCCTACATGTGTCCTCTCCCAGATGCAACTGAAGGAGTC
AGGACCTGGCCTGGTGAAGCCCTCGCAGACCCTCTCCCTCACCTGCACTGTCTCTGGATTATCTTTGAACGAGAATG
CTGTAGGCTGGGTCCGCCAGGCTCCAGGAAAAGGGCTGGAATTTGTTGCCTTTATATGGCAGATTGCAAGCTACAAT
CCAGCCCTGAAGTCCCGAGCCGGCATCACCAAGGACACCTCAAAGAGCCAAGTTTATCTGACCCTGAACAGCCTGAC
AAGCGAGGACACGGCCGTCTATTACTGTGCAAGAGGCAGTGTTGGTGGCTACACGACTTACTATGCCGATGCTATGG

ACCCCTGGGGCCATGGCACCCTGGTCACCGTCTCCTCAGAGAGTACGATGACCCCAGATCTCTTCCCCCTCGTCTCC
TGTGGGCCCTCTCTTGATGAGAGCCTGGTGGCTGTGGGCTGCCTAGCCCGGGACTTCCTACCCAAG
>HM175984.1 Equus caballus clone 2H534 immunoglobulin heavy chain V-D-J region mRNA, partial cds
ATGAGTCACCTGTGGTTCTTCCTCTTTCTGGTGGCCGCTCCTACATGTGTCCTGTCCCAGGTGCAACTGAAGGAGTC
AGGACCTGGCCTGGTGAAGCCCTCGCAGACCCTGTCCCTCACCTGCACTGTCTCTGGATTATCTTTGACCAGTAATC
TTATAGGCTGGGTCCGCCAGGCTCCAGGAAAAGGTCTGGAATGGGTGGGTGGTGTATCCAGTGATGGAACACCGGTG
TACAATCCAGTCCTGAAGACCCGAACCACCATCGGCAAGGACACCTCAAAGAGCCAAGTTTATTGACGCTGAACAG
CATGACAAGCGAGGACACGGCCGTCTATTATTGTACACGATGCGGAGAATATGGCTACCATGGTGCGGTGGACCCCT
GGGGCCAGGGCACCCTGGTCACCGCCTCCTCAGAGAGTACGATGACCCCAGATCTCTTCCCCCTCGTCTCCTGTGGG
CCCTCTCTTGATGAGAGCCTGGTGGCTGTGGGCTGCCTAGCCCGGGACTTCCTACCCAAG
>HM175983.1 Equus caballus clone 2H208 immunoglobulin heavy chain V-D-J region mRNA, partial cds
ATGAGTCACCTGTGGTTCTTCCTCTTTCTGGTGGCCGCTCCTACCTGTGTCCTGTCCCAGGTGCAGCTGAAGGAGTC
AGGACCTGGCCTGGTGAAGCCCTCGCAGACCCTCTCCCTCACCTGCACTGTCTCTGGATTATCTTTGAGCAGTAATG
TAGGCTGGGTCCGCCAGGCTCCAGGAAAAGGGCTGGAATATGTTGGTGGACTGATGTGGAATGGAATTGCAGACTAC
AACCCAGCCCTGAAGTCCCGAGCCAGCATCACCGTGGACACCTCAAAGAGCCAAGTTTATCTGACGCTGAACAGCCT
GACAAGTGAGGACACGGCCGTCTATTTTTGTGCAGGGAATCCTGGTTGGGGTGCGACGGCCGTCAGTAATTACGAAG
GGGCTATGGACCCCTGGGGCCAGGGCACCCTGGTCACCGTCTCCTCAGAGAGTACGATGACCCCAGATCTCTTCCCC
CTCGTCTCCTGTGGGCCCTCTCTTGATGAGAGCCTGGTGGCTGTGGGCTGCCTAGCCCGGGACTTCCTACCCAAG
>HM175980.1 Equus caballus clone 1HC56 immunoglobulin heavy chain V-D-J region mRNA, partial cds
ATGAGTCACCTGTGGTTCTTCCTCTTTCTGGTGGCCGCCCCTACACGTGTCCTGTCCCAGGTGCAACTGAAGGAGTC
AGGACCTGGCCTGGTGAAGCCCTCGCAGACCCTGTCCCTCACCTGCACTGTCTCTGGCTTATCTGTGACAAGTTATG
GTGTGGCCTGGGTCCGCCAGGCTCCAGGAAAAGGGCTGGAATTTGTTGGTGGTGTGGTTGCCAGTGCAAGCTACAAC
CCAGCCCTGAAGTCCCGAGCCAGCATCACCAGGGACACCTCAAAGAGCCAAGTTTATCTGACGTTGAATAGCCTGAC
AAGCGAGGACACGGCCGTCTATCATTGTGCAGGGGGGGAACTAGGTGGTTGGTATGGTTTCAATAACGAACACTATT
GGGGCCAGGGCATCCTGGTCACCGTCTCCTCAGAGAGTACGATGACCCCAGATCTCTTCCCCCTCGTCTCCTGTGGG
CCCTCTCTTGATGAGAGCCTGGTGGCTGTGGGCTGCCTAGCCCGGGACTTCCTACCCAAG
>HM175979.1 Equus caballus clone 1HB2 immunoglobulin heavy chain V-D-J region mRNA, partial cds
ATGAGTCACCTGTGGTTCTTCCTCTTTCTGGTGGCCGCTCCTACATGTGTCCTCTCCCAGGTGCAACTGAAGGAATC
AGGACCTGGCCTGGTGAAGCCCTCGCAGACCCTCTCCCTCACCTGCACTGTCTCTGGATTATCTTTGAGCAGTAATT
CTGTAGGCTGGGTCCGCCAGGCTCCAGGAAAAGGGCTGGAATTTGTTGGTGTTAAAAGTAGAAGTGTAAACTACAAC
CCAGCCCTGAAGTCCCGAGCCAGCATCACCAAGGACACCTCAAAGAGCCAAGTCTATCTGACGCTGAACAGCCTGAC
AAGCGAGGACACGGCCGTCTATTACTGTGCAGGAGACGCGCAGAATAACAATATATTGGTTCCGTCTTCTATAGACT
ACTGGGGCCAGGGCATCCTGGTCACCGTCTCCTCAGAGAGTACGAAGACCCCAGATCTCTTCCCCCTCGTCTCCTGT
GGGCCCTCTCTTGATGAGAGCCTGGTGGCTGTGGGCTGCCTAGCCCGGGACTTCCTACCCAAG
>HM175978.1 Equus caballus clone 1HE81 immunoglobulin heavy chain V-D-J region mRNA, partial cds
ATGAGTCACCTGTGGTTCTTCCTCTTTCTGGTGGCCGCTCCTACATGTGTCCTGTCCCAGGTGCAACTGAAGGAGTC
AGGACCTGGCCTGGTGAAGCCCTCGCAGACCCTCTCCCTCACCTGCATTGTCTCTGGATTTTCTTTGAGCAATTATT
ATGTAGGGTGGGTCCGCCAGGCTCCAGGAAAAGGGCTGGAAACGGTTGGTGGTGTAGAGCCTAGTGGAAGTTCAAAC
TACAACCCAGCCCTGAAGTCCCGAGCCAGCATCACCAAGGACACCTCAAAGAGCCAATCTTATCTGACGCTGAACAG
TCCAACAAGCGAGGACACGGCCGTCTATTACTGTGCAGCGTCGGCTCGCAGTGGCTGGTGGTATGCTATAGACTACT
GGGGACAGGGCATCCTGGTCACCGTCTCCTCAGAGAGTACGAAGACCCCAGATCTCTTCCCCCTCGTCTCCTGTGGG
CCCTCTCTTGTTGAGAGCCTGGTGGCTGTGGGCTGCCTAGCCCGGGACTTCCTACCCAAG
>HM175976.1 Equus caballus clone 1HC10 immunoglobulin heavy chain V-D-J region mRNA, partial cds
ATGAGTCACCTGTGGTTCTTCCTCTTTCTGGTGGCCGCTCCTACATGTGTCCTGTCCCAGGTGCAGCTGAAGGAGTC
AGGACCTGGCCTGGTGAAGCCCTCGCAGACCCTCTCCCTCACCTGCACTGTCTCTGGGATATCTTTGAGCAGTATTG
GTGTAGGGTGGGTCCGCCAGGCTCCAGGAAAAGGGCTGGAATATGTTGGTGCCATTGATGGAAGTACAAACTACAAC
CCAGACCTGAAGTCCCGAGCCACCATCACCCAGGACACCTCAAAGAGCCAAGTTTATCTGACGCTGAACAGCCTGAC
AAGCGAGGACACGGCCGTCTATTACTGTGCAGGGCAAGATGGCAGCAGGAATTACTTGGGGGGGGATGATATAGACT
ACTGGGGCCAGGGCATCCTGGTCACCGTCTCCTCAGAGAGTACGAAGACCCCAGATCTCTTCCCCCTCGTCTCCTGT
GGGCCCTCTCTTGATGAGAGCCTGGTGGCTGTGGGCTGCCTAGCCCGGGACTTCCTACCCAAG

>HM175972.1 Equus caballus clone 1HA9 immunoglobulin heavy chain V-D-J region mRNA, partial cds
ATGAGTCACCTGTGGTTCTTCCTCTTTCTGGTGGCCGCTCCTACATGTGTCCTGTCCCAGGTGCAACTGAAGGAGTC
AGGACCTGGCCTGGTGAAGCCCTCGCAGACCCTCTCCCTCACCTGCACTGTCTCTGGATTGGATTTGAGCGGAAAG
CTGTGGGCTGGGTCCGCCAGGCTCCAGGAAAAGGACTGGAAAATGTTGCCAGTATAGGTAGTAGTGGAATGTGCGA
ATCAACCCAGCCCTGAAGTCCCGAGCCAGCATCACCAAGGACACCTCAAAGAGCCAAGTTTATTTGACGCTGAGCAG
CCTGACAAGTGAGGACACGGCCGTCTATTACTGTGCAGGAGGTGTGGGCGGTAATTACCTAGTCTTCTTTGACTACT
GGGGCCAGGGCACCTTGGTCGACGTCTCCTCAGAGAGTACGAAGACCCCAGATCTCTTCCCCCTCGTCTCCTGTGGG
CCCTCTCTTGATGAGAGCCTGGTGGCTGTGGGCTGCCTAGCCCGGGACTTCCTACCCAAG
>HM175971.1 Equus caballus clone 1HC77 immunoglobulin heavy chain V-D-J region mRNA, partial cds
ATGAGTCACCTGTGGTTCTTCCTCTTTCTGGTGGCCGCTCCTACATGTGTCCTGTCCCAGGTGCAACTGAAGGAGTC
AGGACCTGGCCTGGTGAAGCCCTCGCAGACCCTCTCCCTCACCTGCACTGTCTCTGGATTATCTTTGAACAGTAATG
GTGTGCACTGGGTCCGCCAGGCTCCAGGAAAAGGGCTGGAATTTGTTGGTGGTGTAGCGCATGATGGGAGTGTAGAC
TACAATCCAGTCCTGAAGTCTCGAGCCAGCGTCACCAAGGACACCTCAAAGAAGCAAGTTTATCTGACGTTAAACAG
CCTGACAAGCGAGGACACGGCGTTCTATTACTGTGCAGCTGGCTATGGTTACGGTCGCAAGGGTTATGACTACATCC
CGAGTATAGATTCTGGATACTGGGGCCAGGGCACCCTGGTCACCGTCTCCTCAGAGAGTACGATGACCCCAGATCTC
TTCCCCCTCGTCTCCTGTGGGCCCTCTCTTGATGAGAGCCTGGTGGCTGTGGGCTGCCTAGCCCGGGACTTCCTACC
CAAG
>HM175969.1 Equus caballus clone 1HA83 immunoglobulin heavy chain V-D-J region mRNA, partial cds
ATGAGTCACCTGTGGTTCTTCCTCTTTCTGGTGGCCGCTCCTACATGTGTCCTGTCCCAGGTGCAACTGAAGGAGTC
AGGACCTGGCCTGGTGAAGCCCTCGCAGACCCTCTCCCTCACCTGCACTGTCTCTGGATTACCTTTGAGCAGTAACG
CTGTAGGCTGGGTCCGCCAGGCTCCAGGAAAAGGGCTGGAATATGTTGGTGGTGTGGCTACGACAGGAAGTGTAAAG
TACAACCCAGACCTGAAGTCCCGAGCCAGCATCACCAAGGACACCTCAAAGAGCCAAGTTTATCTGACGCTGAACAG
CCTGACAAGCGAGGACACGGCCGTCTATTACTGTGCAGGAGGCGGTGGTCCTAATCACGTGGTGGGGTTATCCACCT
ACTGGGGCCAGGGCACCCTGATCACCGTCTCCTCAGAGAGTACGAAGACCCCAGATCTCTTCCCCCTCGTCTCCTGT
GGGCCCTCTCTTGATGAGAGCCTGGTGGCTGTGGGCTGCCTAGCCCGGGACTTCCTACCCAAG
>HM175968.1 Equus caballus clone 1HA13 immunoglobulin heavy chain V-D-J region mRNA, partial cds
ATGAGTCACCTGTGGTTCTTCCTCTTTCTGGTGGCCGCTCCTACATGTGTCCTGTCTCAGGTGCAACTGAAGGAGTC
TGGACCTGGCCTGGTGAAGCCCTCACAGACCCTCTCCCTCACCTGCACTGTCTCTGGATTATCTTTGAGCAGTAGGG
CTGTAGCCTGGGCCCGCCAGGCTCCAGGAAAAGGGCTGGAATGTGTCGGTGGTATTACTGATAGTGGAAGTCCGTAC
TACGGCCCAGCCCTGGAGTCCCGAGCCAGTATCACCAAGGACACCTCAAAGAGCCAGGTTTATCTGACGCTGAACAG
CCTGACAGAAGAGGACACGGCCGTCTACTTCTGTGCAGGCACGGATTACAGGAATCCGTGGGGGGACAATTTTGGTT
ACTGGGGCCAGGGCACCCTGGTCACCGTCTCCTCAGAGAGTACGATGACCCCAGATCTCTTCCCCCTCGTCTCCTGT
GGGCCCTCTCTTGATGAGAGCCTGGTGGCTGTGGGCTGCCTAGCCCGGGACTTCCTACCCAAG
>HM175966.1 Equus caballus clone 1HB18 immunoglobulin heavy chain V-D-J region mRNA, partial cds
ATGAGTCACCTGTGGTTCTTCCTCTTTCTGGTGGCCGCTCCTACATGTGTCCTCTCCCAGGTGCAACTGAAGGAGTC
AGGACCTGGCCTGGTGAAGCCCTCGCAGACCCTCTCCCTCGTCTGCACTGTCTCTGGATTATCTTTGAGCAGTAATG
TTGTAGGCTGGGTCCGCCAGGCTCCAGGAAAAGGGCTGGAATTTGTTGGTAATATGTGGACAAGTGCAAACTACAAC
CCAGCCCTGAAGTCCCGAGCCAGCATCACCAAGGACGACTCACAGAGCCAAGTTTATCTGACGCTGAACAGCCTGAC
AAGCGAGGACACGGCCGTCTATTACTGTGCAGGAGCTGCACAGAGTCAAGGGTGTGGTCGTGGTGGTGCAGACTGTT
TTGGCTACTGGGGCCAGGGCACCCTGGTCACCGTCTCCTCAGAGAGTACGATGACCCCAGATCTCTTCCCCCTCGTC
TCCTGTGGGCCCTCTCTTGATGAGAGCCTGGTGGCTGTGGGCTGCCTAGCCCGGGACTTCCTACCCAAG
>HM175965.1 Equus caballus clone 1HF80 immunoglobulin heavy chain V-D-J region mRNA, partial cds
ATGAGTCACCTGTGGTTCTTCCTCTTTCTGGTGGCCGCTCCTACATGTGTCCTGTCCCAGGTGCAACTGAAGGAGTC
AGGACCTGGCCTGGTGAAGCCCTCGCAGACCCTCTCCCTCGTCTGCACTGTCTCTGGATTATCTTTGAGCAGTGTTG
GTGTGGGCTGGGTCCGCCAGGCTCCAGGAAAAGGGCTGGAATTTGTTGGTGGTATACGTGATAGTGCAAACTACAAC
CCAGCCCTGAAGTCCCGAGCCAGCATCACCAAAGACACCTCTCAGAGCCAAGTTTATCTGACTCTGAACAGCCTGAC
AAGCGAGGACACGGCCGTCTATTACTGTCACGGAGGCTCAGAGAACAGGGACGGTGGTGGTGTTTTCTGGTTTAATT
TTGGCTACTGGGGCCAGGGCACCCTGGTCACCGTCTCCTCAGAGAGTACGATGACCCCAGATCTCTTCCCCCTCGTC
TCCTGTGGGCCCTCTCTTGATGAGAGCCTGGTGGCTGTAGGCTGCCTAGCCCGGGACTTCCTACCCAAG

>HM175963.1 Equus caballus clone 1HC60 immunoglobulin heavy chain V-D-J region mRNA, partial cds
ATGAGTCACCTGTGGTTCTTCCTCTTTCTGGTGGCCGCTCCTACATGTGTCCTGTCCCAGATACAACTGAAGGAGTC
AGGACCTGGCCTGGTGAAGCCCTCGCAGACCCTCTCCCTCACCTGCACTGTCTCTGGATTGACTGGGAACACTTATT
CTGTAGGCTGGGTCCGCCGGGCTCCAGGAAAAGGGCTGGAATGGATTGGTTCAACGTATGGTGATAGAGGTTACAAC
CCAGCCCTGAAGTCCCGAACCAGCATCACCAAGGACGCCTCAAAGAGCCAAGTTTATCTGACGCTGAACAGCCTGAC
AAGCGAGGACACGGCCGTCTATTACTGTGCAGGGAAGGACGGCGAATGGGCTTGGTACGGTGACAGTGACTACTGGG
GCCAGGGCACCCTGGTCACCGTCTCCTCAGAGAGTACGAAGACCCCAGATCTCTTCCCCCTCGTCTCCTGTGGGCCC
TCTCTTGATGAGAGCCTGGTGGCTGTGGGCTGCCTAGCCCGGGACTTCCTACCCAAG
>HM175962.1 Equus caballus clone 1HF85 immunoglobulin heavy chain V-D-J region mRNA, partial cds
ATGAGTCACCTGTGGTTCTTCCTCTTTCTGGTGGCCGCTCCTACATGTGTCTTCTCCCAGGTGCAACTGAAGGAGTC
AGGACCTGGCCTGGTGAAGCCCTCGCAGACCCTCTCCCTCACCTGCACTGTCTCTGGATTATCTTTGACAAGTAATG
CTGTAGCCTGGGTCCGCCAGGCTCCAGGAAAAGGGCTGGAATTTGTTGGTAATATATATGATAGTGCAAACTACAAT
CCAGACCTGAAGTCCCGAGCCAGCATCACCAAGGACACCTCAAAGAGCCAAGTTTATCTGACGCTGAACAGCCTGAC
AAGCGAGGACACGGCCGTCTATTATTGTGCAGGCTATCGAGAGACGTGGGGTAATTGGTTTTACATCGAATTTGGCT
ACTGGGGCCAGGGCACCCTGGTCACCGTCTCCTCAGAGAGTACGATGACCCCAGATCTCTTCCCCCTCGTCTCCTGT
GGGCCCTCTCTTGATGAGAGCCTGGTGGCTGTGGGCTGCCTAGCCCGGGACTTCCTACCCAAG
>HM175959.1 Equus caballus clone 2H43 immunoglobulin heavy chain V-D-J region mRNA, partial cds
ATGAATCACCTGTGGTTCTTCCTCTTTCTGGTGACCGCTCCTGCATGTGTCCTGTCCCAGGTGCAACTGAAGGAGTC
GGGACCTGGCCTGGTGAAGCCCTCGCAGTCCCTGTCCCTCATCTGCACGGTCTCTGGGTTATCTTTAAACAGTAATT
CTGTGAACTGGGTCCGCCAGGCTCCAGGGAAAGGGCTGGAGTGGATTGGTGCGCTACATGTTAGTGCAGGTACATTC
TACAACCCAGCCCTGAAGTCCCGAGCCAGCATCACCAAGGACGCCGCGCAGAGCCAAGTTTATCTGACGCTGAACAG
CCTGACAGGCGAAGACACGGCCGTCTATTACTGTGCAGCTAATTACGAAGCTAATTATGCCAAGATTGGCTACTGGG
GCCAGGGCGCCCTGGTCACCGTCTCCTCAGAGAGTACGATGACCCCAGATCTCTTCCCCCTCGTCTCCTGTGGGCCC
TCTCTTGATGAGAGCCTGGTGGCTGTGGGCTGCCTAGCCCGGGACTTCCTACCCAAG
>HM175958.1 Equus caballus clone 2H700 immunoglobulin heavy chain V-D-J region mRNA, partial cds
ATGAGTCACCTGTGGTTCTTCCTCTTTCTGGTGGCCGCTCCTACATGTGTCCTGTCCCAGGTGCAACTGAAGGAGTC
AGGACCTGGCCTGGTGAAGCCCTCGCAGACCCTCTCCCTCACCTGCACTGTCTCTGGATTATCTTGGAGCGGTAATG
ATGTAGGCTGGGTCCGCCGGGCTCCAGGAAAAGGGCTGGAACACATTGGTCGTATATGGGATACTTCAAGTACAAAC
TACAACCCAGCCCTGAAGTCCCGAGCCGCCATCACCAAGGACACCTCAAAGAGCCAAGTTTATCTGACGCTGAACAG
CCTGACAAGCGAGGACACGGCCGTCTATTACTGTGCAGGAGGCGGAGGTGTAAGTCGGTATTTGCGGGAATTCTATG
CCTACTGGGGCCAGGGCACCCTGGTCACCGTCTCCTCAGAGAGTACGATGACCCCAGATCTCTTCCCCCTCGTCTCC
TGTGGGCCCTCTCTTGATGAGAGCCTGGTGGCTGTGGGCTGCCTAGCCCGGGACTTCCTACCCAAG
>HM175956.1 Equus caballus clone 2H1101 immunoglobulin heavy chain V-D-J region mRNA, partial cds
ATGAGTCACCTGTGGTTCTTCCTCTTTCTGGTGGCCGCTCCTACATGTGTCCTGTCCCAGGTGCAACTGAAGGAGTC
AGGACCTGGCCTGGTGAAGCCCCCGCAGACCCTCTCCCTCACCTGCACTGTCTCTGGATTATCTTTGTGCAGTAATG
CTGTAGGCTGGGTCCGCCAGGCTCCAGGAAAAGGGCTGGAATACGTTGGTGCTATATATGACAGTGCAAGTGCAAAC
TACAACCCAGCCCTGAAGTCCCGAGCCAGCATCACCAAGGACACCTCAAAGAGCCAAGTTTATCTGACGCTGAACAG
CCTGACAAGCGAGGACACGGCCGTCTATTACTGTGCAGCCCTGGATGACTATGGTAGAACGTACACCAGTTACTATG
CCGGGGATTTTGCCTACTGGGGCCAGGGCACCCTGGTCACCGTCTCCTCAGAGAGTACGATGACCCCAGATCTCTTC
CCCCTCGTCTCCTGTGGGCCCTCTCTTGATGAGAGCCTGGTGGCTGTGGGCTGCCTAGCCCGGGACTTCCTACCCAA
G
>HM175955.1 Equus caballus clone 2H1331 immunoglobulin heavy chain V-D-J region mRNA, partial cds
ATGAGTCACCTGTGGTTCTTCCTCTTTCTGGTGGCCGCTCCTACATGTGTCCTGTCCCAGGTGCAACTGAAGGAGTC
AGGACCTGGCCTGGTGAAGCCCTCGCAGACCCTCTCCCTCACCTGCACTGTCTCTGGGTTATCTGTGAGCAGTAATG
GTATAGGCTGGGTCCGCCAGGCTCCAGGAAAAGGGCTGGAATATGTTGGTGGTATGCATAGTACTGGAAGTACAGAC
TACAACCCAACCCTGAAGTCCCGAGCCAGCATCACCAAGGACACCTCAAAGAGCCAGGTGTATCTGACGCTGAACAG
CCTGACAAGCGAGGACACGGCCGCCTATTATTGTGCGGCCGACACAAACTTCCACTATAGTGCTGGTTCCATTGGCT
ACTGGGGCCAGGGCACCCTGGTCACCGTCTCCTCAGAGAGTACGATGACCCCAGATCTCTTCCCCCTCGTCTCCTGT
GGGCCCTCTCTTGATGAGAGCCTGGTGGCTGTGGGCTGCCTAGCCCGGGACTTCCTACCCAAG

>HM175954.1 Equus caballus clone 2H878 immunoglobulin heavy chain V-D-J region mRNA, partial cds
ATGAGTCACCTGTGGTTCTTCCTCTTTCTGGTGGCCGCTCCTACATGTGTCCTGTCCCAGGTGCAACTGAAGGAGTC
AGGACCTGGCCTGGTGAAGCCCTCGCAGACCCTCTCCCTCACCTGCACTGTCTCTGGATTATCTTTGACCAGTAATG
GTGTGGTGTGGTTCCGCCAGGCTCCAGGCATGGGGCTGGAACGTGTTGGTGGTATTCATAATAGTGGAAGTCAAGAC
TACAACCCAGCCCTGAAGTCCCGAGCCACAATCACCAAGGACACCTCAAGGAGCCAAGTTTATCTGACGCTGAACAG
CCTGACAAGCGAGGACACGGCCGTCTATTACTGTGCAGGAAACATGAATGGTTGGGCTGCACCATCCAATATTGGCT
ACTGGGGCCAGGGCACCCTGGTCACCGTCTCCTCAGAGAGTACGATGACCCCAGATCTCTTCCCCCTCGTCTCCTGT
GGGCCCTCTCTTGATGAGAGCCTGGTGGCTGTGGGCTGCCTAGCCCGGGACTTCCTACCCAAG
>HM175953.1 Equus caballus clone 2H1368 immunoglobulin heavy chain V-D-J region mRNA, partial cds
ATGAGTCACCTGTGGTTCTTCCTCTTTCTGGTGGCCGCTCCTACATGTGTCCTGTCCCAGGTGCAACTGAAGGAGTC
AGGACCTGGCCTGGTGAAGCCCTCGCAGACCCTCTCCCTCACCTGCACTGTCTCTGGATTATCTTTGAGCAGTAATC
ATGTACGCTGGGTCCGCCAGGCTCCAGGAAAAGGGCTGGAATGGGTTGGTGGTATACTTAGTGATGGAAGTGCAAAC
TACAATCCAGCCCTGAAGTCCCGAGCCAGCATCACCAAGGACACCTCAAAGACCCAAGTTTATCTGACGCTGAACAG
CCTGACAAGCGAGGACACGGCCGTCTATTACTGTACAGGAAGTACCTATCACCGCTACTGGGGCCAGGGCACCCTGG
TCACCGTCTCCTCAGAGAGTACGATGACCCCAGATCTCTTCCCCCTCGTCTCCTGTGGGCCCTCTCTTGATGAGAGC
CTGGTGGCTGTGGGCTGCCTAGCCCGGGACTTCCTACCCAAG
>HM175952.1 Equus caballus clone 2H922 immunoglobulin heavy chain V-D-J region mRNA, partial cds
ATGAGTCACCTGTGGTTCTTCCTCTTTCTGGTGGCCGCCCCTACATGTGTCCTGTCCCAGGTGCAACTGAAGGAGTC
AGGACCTGGCCTGGTGAAGCCCTCGCAGACCCTCTCCCTCACCTGCACTGTCTCTGGATTAACTTTGAGCAGTGCAC
ATGTAGGCTGGGTCCGCCAGGCTCCAGGAAAAGGGCTGGAACATGTTGGTGGTATATCTACCACAGGAGATAAAGAC
CGCTACAATCCAGCCCTGAGGTCCCGAGTCAGCGTCGCCACGGACACCTCAAAGAGCCAAGTTTATCTGACGCTGAA
CAGCCTGACAAGCGAGGACACGGCCGTCTATTACTGTGTAGGAACGCAACGCGGTGAGGGTTATGCTACGTCCACGT
TTGGCTACTGGGGCCAGGGCACCCTGGTCACCGTCTCCTCAGAGAGTACGATGACCCCAGATCTCTTCCCCCTCGTC
TCCTGTGGGCCCTCTCTTGATGAGAGCCTGGTGGCTGTGGGCTGCCTAGCCCGGGACTTCCTACCCAAG
>HM175948.1 Equus caballus clone 1HC16 immunoglobulin heavy chain V-D-J region mRNA, partial cds
ATGAGTCACCTGTGGTTCTTCCTCTTTCTGGTGGCCGCTCCTACATGTGTCCTGTCTCAGGTGCAACTGAAGGAGTC
AGGACCTGGCCTGGTGAAGCCCTCACAGACCCTCTCCCTCACCTGCACTGTCTCTGGATTATCTTTGAGCAGTAAAG
GTGTAGGCTGGGTCCGCCAGACTCCAGGAAAAGGGCTGGAATATATTACTGGTATAAGTAGTGATGGAAGGCTAGAC
ACCAAGCCAGCCCTGAAGTCCCGAGCCAGCGTCACCAAGGACACCTCAAAGAGCCAAGTATATCTGACGCTGAACAG
CCTGACAAGCGAGGACACGGCCGTCTATTATTGTGCAGGGGGTGACCGTGGTTGGGTAAGGGATGGTATAAACTACT
GGGGCCAGGGCATCCTGGTCACCGTCTCCTCAGAGAGTACGATGACCCCAGATCTCTTCCCCCTCGTCTCCTGTGGG
CCCTCTCTTGATGAGAGCCTGGTGGCTGTGGGCTGCCTAGCCCGGGACTTCCTACCCAAG
>HM175939.1 Equus caballus clone 1HA32 immunoglobulin heavy chain V-D-J region mRNA, partial cds
ATGAGTCACCTGTGGTTCTTCCTCTTTCTGGTGGCCGCTCCTACATGTGTCCTGTCCCAGGTGCAACTGAAGGAGTC
AGGACCTGGCCTGGTGAAGCCCTCGCAGACCCTCTCCCTCGTCTGCACTGTCTCTGGATTATCTTTGAGCAGTAATG
CTGTAGGCTGGGTCCGCCAGGCTCCAGGAAAAGGGCTAGAATATGTTGGTAATATATATGGTAGTGCAAACTACAAC
CCAGCCCTGAAGTCCCGAGCCAGCATCACCAGGGACACCTCAAAGAGCCAAGTTTATCTGACGCTGAACAGCCTGAC
AAGCGAGGACACGGCCGTCTATTACTGTGCAGGAAGTGTGAATGGTGATGGTGCCTGGAACTTATATGGTATAACGT
ACTGGGGCCAGGGCATCCTGGTCACCGTCTCCTCAGAGAGTACGATGACCCCAGATCTCTTCCCCCTCGTCTCCTGT
GGGCCCTCTCTTGATGAGAGCCTGGTGGCTGTGGGCTGCCTAGCCCGGGACTTCCTACCCAAG
>HM175937.1 Equus caballus clone 1HF94 immunoglobulin heavy chain V-D-J region mRNA, partial cds
ATGAATCACCTGTGGTTCTTCCTCTTTCTGGTGGCCGCTCCTACATGTGTCCTGTCCCAGGTGCAACTGAAGGAGTC
GGGACCTGGCCTGGTGAAGCCCTCGCGGACCCTGTCCCTCACCTGCACTGTCTCTGGACTATCTTTGGGGAGCAATG
GTGTAGGCTGGGTCCGCCAGGCTCCAGGAAAAGGGCTGGAGTGGGTTGGTATGTATTGGACTGATGGTAGGGGATAC
TACAATCCAGTCCTGAAGTCCCGAGTCACCATCGCCAAGGACACCTCAAAGAACCAAGTTTATCTGACGCTGACCAG
CCTGACAGACGAAGACACGGCCGTCTATTATTGTGCAGTGAACTGGGGTCCCTATGCTGGTAGTTACATATGGGAGG
TCTACTGGGGCCAGGGCATCCTGGTCACCGTCTCCTCAGAGAGTACGAAGACCCCAGATCTCTTCCCCCTCGTCTCC
TGTGGGCCCTCTCTTGATGAGAGCCTGGTGGCTGTGGGCTGCCTAGCCCGAGACTTCCTACCCAAG

>HM175932.1 Equus caballus clone 1HB67 immunoglobulin heavy chain V-D-J region mRNA, partial cds
ATGAGTCACCTGTGGTTCTTCCTCTTTCTGGTGGCCGCTCCTACATGTGTCCTCTCCCAGGTGCAACTGAAGGAGTC
AGGACCTGGCCTGGTGAAGCCCTCGCAGACCCTCTCCCTCACCTGCACTGTCTCTGGATTATCTTTGAGCAACAATG
CTGTAGGCTGGGTCCGCCAGGCTCCAGGAAAAGCGCTGGATTGGGTTGGTGCTATATGGAGTAGTGCAAAGTACGAC
CCAGCCCTGAAGTCCCGAGCCGAAATCACCAAGGACACCTCAAAGAGTCAAGTTTATCTGACGCTGGACAGCCTGAC
AAGCGAGGACACGGCCGTCTATTACTGTGCAGGAGGTTCGATTTTTGGCTATAGGTTTTTTATGGTGAAACATACT
GGGGCCAGGGCATCCTGGTCACCGTCTCCTCAGAGAGTACGATGACCCCAGATCTCTTCCCCCTCGTCTCCTGTGGG
CCCTCTCTTGATGAGAGCCTGGTGGCTGTGGGCTGCCTAGCCCGGGACTTCCTACCCAAG
>HM175925.1 Equus caballus clone 1HB21 immunoglobulin heavy chain V-D-J region mRNA, partial cds
ATGAGTCACCTGTGGTTCTTCCTCTTTCTGGTGGCCGCTCCTACATGTGTCCTCTCCCAGGTGCAACTGAAGGAGTC
AGGACCTGGCCTGGTGAAGCCCTCGCAGACCCTCTCCCTCACCTGCACTGTCTCTGGATTATCTTTGGAAGGTAATG
TTGTAGGCTGGGTCCGCCAGGCTCCAGGAAAAGGGCTGGAATTTGTTGGCGGTATAAGTAATACTGCAGCCTACAAC
CCAGCCCTGAAGTCCCGAGCCAGCATCACCAAGGACACCTCAAAGAGCCAAGTTTATCTGACGCTGAGCAGCCTGAC
AGAGGAGGACACGGCCGTCTATTACTGTGTGAGAGACCATGATCATTACCTCTCCATGGATGGCATAATGTACTGGG
GCCAGGGCATCCTGGTCACCGTCTCCTCAGAGAGTACGATGACCCCAGATCTCTTCCCCCTCGTCTCCTGTGGGCCC
TCTCTTGATGAGAGCCTGGTGGCTGTGGGCTGCCTAGCCCGGGACTTCCTACCCAAG
>HM175923.1 Equus caballus clone 2H398 immunoglobulin heavy chain V-D-J region mRNA, partial cds
ATGAATCACCTGTGGTTCTTCCTCTTTCTGGTGGCCGCTCCTACATGTGTCCTGTCCCAGGTGCAACTGAAGGAGTC
GGGACCTGGCCTGGTGAAGCCCTCGCAGACCCTGTCCCTCACCTGCTCTGTCTCTGGATTATCTTTGAGCAGTAATG
GTGCAGGCTGGGTCCGCCAGGCTCCAGGAAAAGGGCTGGAGTGGGTGGGTAATATCAGAAATAGTGAGGAGACAGTG
TACAACCCAGTCCTGAAGTCCCGAGCCAGCATCACCAAGGACACCTCAAAGAGCCAGCTTTATCTGACGGTGAACAG
CCTGACAGGCGAAGACACGGCCGTCTATTACTGTGCAGGACGTACGGGCTGGGGTTTGATCGATGGTGTAGTCTACT
GGGGCCAGGGCATCCTGGTCACCGTCTCCTCAGAGAGTACGATGACCCCAGATCTCTTCCCCCTCGTCTCCTGTGGG
CCCTCTCTTGATGAGAGCCTGGTGGCTGTGGGCTGCCTAGCCCGGGACTTCCTACCCAAG
>HM175922.1 Equus caballus clone 2H934 immunoglobulin heavy chain V-D-J region mRNA, partial cds
ATGAATCACCTGTGGTTCTTCCTCTTTCTGGTGGCCGCTCCTACATGTGTCCTGTCCCAGGTGCAACTGAAGGAGTC
GGGACCTGGCCTGGTGAAGCCCTCGCAGACCCTGTCCCTCACCTGCACTGTCTCTGGATTATCTTTGAGCGAAAATG
GTGCGGGCTGGGTCCGCCAGGCTCCAGGAAAAGGGCTGGAGTGGATTGGGCGTATATTTAATAGTGATAGTACAACG
TACAACCCAGCCCTGAAGTCCCGAGCCAGCATCACCAAGGACGCCTCAAAGAGCCAAGTTTATCTGACGCTGAGCAG
CCTGACAGGCGAAGACACGGCCGTCTATTACTGTGCAGGATCAACGGATAAATGGAGAAATATTGACTACATAATCT
ACTGGGGCCAGGGCACCCTGGTCACCGTCTCCTCAGAGAGTACGATGACCCCAGATCTCTTCCCCCTCGTCTCCTGT
GGGCCCTCTCTTGATGAGAGCCTGGTGGCTGTGGGCTGCCTAGCCCGGGACTTCCTACCCAAG
>HM175919.1 Equus caballus clone 2H1354 immunoglobulin heavy chain V-D-J region mRNA, partial cds
ATGAATCACCTGTGGTTCTTCCTCTTTCTGGTGACCGCTCCTACATGTGTCCTGTCCCAGGTGCAACTGAAGGAGCC
GGGACCTGGCCTGGTGAAGCCCTCGCAGACCCTGTCCCTCACCTGCACTGTCTCTGGATTATCTTTGAGCAGTAATA
CTGTAGGCTGGGTCCGCCAGGCTCCAGGAAAAGGGCTGGACTGGGTTGGTGATATAAATGGTGGTGGAAGCTTATAC
TACAACCCAGCCCTGAAGTCCCGAGCCAGCATCACCAGGGACACTTCAAAGAGCACAGTGGCTCTCACGCTGAACAG
CCTGACAGGCGAAGACACGGCCGTCTATTACTGTACAGGATGGGGTGGTAGTGATTTGTGGTATGCTATTCACTACT
GGGGCCGGGGCGTGCTGGTCACCGTCTCCTCAGAGAGTACGATGACCCCAGATCTCTTCCCCCTCGTCTCCTGTGGG
CCCTCTCTTGATGAGAGCCTGGTGGCTGTGGGCTGCCTAGCCCGGGACTTCCTACCCAAG
>HM175918.1 Equus caballus clone 2H375 immunoglobulin heavy chain V-D-J region mRNA, partial cds
ATGAATCACCTGTGGTTCTTCCTCTTTCTGGTGACCGCTCCTACATGTGTCCTGTCCCAGGTCCGACTGAAGGAGTC
GGGACCTGTCCTGGTCAAGCCGTCGCAGACCCTGTCCCTCACCTGCACTGTCTCTGGATTGTCTTTGAGAAGTAATG
CTGTAGGCTGGGTCCGTCAGGCTCCAGGAAAGGGGCTGGAGTGGGTTGCCGGTATACATGGCAATGGTGAAACATAC
TACAATCCAGCCCTGAAGTCCCGAGCCAGCGTCACCAGGGACACCTCAAAGGGCCAAGTAGATCTGACGCTGAACAG
CCTGACAGGCGAAGACACGGCCGTCTATTACTGTCATGGTAATTATCAAGAACGTATCCGTGCCTCTGATGCACTCT
ACTGGGGCCAGGGCATCCTGGTCACCGTCTCCTCAGAGAGTACGATGACCCCAGATCTCTTCCCCCTCGTCTCCTGT
GGGCCCTCTCTTGATGAGAGCCTGGTGGCTGTGGGCTGCCTAGCCCGGGACTTCCTACCCAAG
>HM175917.1 Equus caballus clone 2H369 immunoglobulin heavy chain V-D-J region mRNA, partial cds

ATGAATCACCTGTGGTTCTTCCTCTTTCTGGTGACTGCTCCTACATGTGTCCTGTCCCAGGTGCAACTGAAGGAGTC
GGGACCTGGCCTGGTCGAGCCCTCGCAGACCCTGTCCCTCACCTGCACTGTCTCTGGGCTATCTTTGAGCAATAATG
CTGTAGGCTGGGTCCGCCAGGCTCCCGGAAAAGGGCTGGAGTGGGTTGGTACGATATACCGAAATGGAAATAGTCAT
TACAATCCAGCCCTGAAGTCCCGAGCCGAAATCCCCAGGGACACCTCAAAGAGTCAAGTTTATCTGGTGCTGAACAG
CCTGACAGGCGAAGACACGGCCGTCTACTACTGTGCAGGATCTCCAGGGTTTGGTTATGGAGATAAGGGCTTCTTGA
ACGGCATAAACCAATGGGGCCAGGGCATCCTGGTCACCGTCTCTTACGAGAGTACGATGACCCCAGATCTCTTCCCC
CTCGTCTCCTGTGGGCCCTCTCTTGATGAGAGCCTGGTGGCTGTGGGCTGCCTAGCCCGGGACTTCCTACCCAAG
>HM175916.1 Equus caballus clone 2H1200 immunoglobulin heavy chain V-D-J region mRNA, partial cds
ATGAATCACCTGTGGTTCTTCCTCTTTCTGGTGACCGCTCCTACATGTGTCCTGTCACAGGTGCAACTGAAGGAGTC
GGGACCTGGCCTGGTGAAGCCCTCGCAGACCCTGTCCCTCACCTGCACTGTCTCTGGATTATCTTTGACGAGATATC
ATGTAGGCTGGGTCCGCCAGGCTCCAGGAAAGGGCCTGGAGTGGGTTGGTGCTACATATTTCACAGTGGGTACATAC
TACAACCCAGCCCTGAAGTCCCGAGCCACCATCACCAGGGACAACTCAAAGAGCCGAGTTGTTCTGACTGTGAACAG
CCTGACAGGCGAAGACACGGCCGTCTATTACTGTGCAGGACTGCCTGGTATACGTGAGGGTATAACTTATTGGGGCC
AGGGCATCCTGGTCACCGTCTCCTCAGAGAGTACGATGACCCCAGATCTCTTCCCCCTCGTCTCCTGTGGGCCCTCT
CTTGATGAGAGCCTGGTGGCTGTGGGCTGCCTAGCCCGGGACTTCCTACCCAAG
>HM175914.1 Equus caballus clone 2H520 immunoglobulin heavy chain V-D-J region mRNA, partial cds
ATGAGTCACCTGTGGTTCTTCCTCTTTCTGGTGGCCGCTCCTACATGTGTCCTGTCCCAGGTGCAACTGAAGGAGTC
AGGACCTGGCCTGGTGAAGCCCTCGCAGACCCTCTCCCTCACCTGCACTATCTCTGGATTATCTTTGATCAGTAATG
CTGCAGGCTGGGTCCGCCAGGCTCCAGGAAAAGGGCTGGAATACGTTGGTGTTATACTTGATAGTGGTAGTGTCGGA
TACAACCCAGCCCTGAAGTCCCGAGCCACCATCACCAGGGACACCCCAAAGAGCCAAGTTTATCTGACGCTGAACAG
CCTGACAGGCGAGGACACGGCCGTCTATTACTGTGCAGGAGGCTCCGACGGTGGAGGTGATGATCAATATTCTATGA
ACTATTGGGGCCAGGGCATCCTGGTCACCGTCTCCTCAGAGAGTACGATGACCCCAGATCTCTTCCCCCTCGTCTCC
TGTGGGCCCTCTCTTGATGAGAGCCTGGTGGCTGTGGGCTGCCTAGCCCGGGACTTCCTACCCAAG
>HM175913.1 Equus caballus clone 2H944 immunoglobulin heavy chain V-D-J region mRNA, partial cds
ATGAGTCACCTGCGGTTCTTCCTCTTTCTGGTGGCCGCTCCTACATGTGTCCTGTCCCAGGTGCAACTGAAGGAGTC
AGGACCTGGCCTGGTGAAGCCCTCGCAGACCCTCTCCCTCACCTGCACTGTCTCTGGATTATCTTTGAGCAGTAATG
CTGTAGGCTGGGTCCGCCAGGCTCCAGGAAGAGGGCTGGAATACGTTGGTGCAATAGAAGTGAGTGAAAGTGCGTGG
TACAACCCAGCCCTGCAGTCCCGAGTTATTATCACCAGGGACACCTCAAAGAGCCAAGTTTATCTGACGATGAACAG
CCTGACAAGCGAGGACACGGCAGTCTATTACTGTGCAGGACTTGGTGGTGATGAATACTGGGACTTTTTTTTCGGAC
AAAACTACTGGGGCCAGGGCATCCTGGTCACCGTCTCCTCAGAGAGTACGATGACCCCAGATCTCTTCCCCCTCGTC
TCCTGTGGGCCCTCTCTTGATGAGAGCCTGGTGGCTGTGGGCTGCCTAGCCCGGGACTTCCTACCCAAG
>HM175907.1 Equus caballus clone 2H576 immunoglobulin heavy chain V-D-J region mRNA, partial cds
ATGAGACACCTGTGGTTCTTCCTCTTTCTGGTGGCCGCTCCTACATGTGTCCTGTCCCAGGTGCAACTGAAGGAGTC
AGGACCTGGCCTGGTGAAGCCCTCGCAGACCCTCTCCCTCACCTGCACTGTCTCTGGATTATCTTGGAGTGACAATG
TTGCAAGATGGGTCCGCCAGGCTCCAGGAAAAGGGCTGGAATATGTTGGTACTATACACAGTAGTGGAAGTACGTTC
TACAACCCAGCCCTGAAGTCCCGAGCCAGCATCACCAAGGACACCTCAAAGAGCCAAGTTTATCTGACGCTGAACAG
CCTGACAAGCGAGGACACGGCCGTCTATTACTGTGCAGGGGGTGGTAAATGGCTGAATTTCGCTGAAATGACCTATT
GGGGCCAGGGCATCTTGGTCACCGTCTCCTCAGAGAGTACGATGACCCCAGATCTCTTCCCCCTCGTCTCCTGTGGG
CCCTCTCTTGATGAGAGCCTGGTGGCTGTGGGCTGCCTAGCCCGGGACTTCCTACCCAAG
>HM175905.1 Equus caballus clone 2H27 immunoglobulin heavy chain V-D-J region mRNA, partial cds
ATGAGTCACCTGTGGTTCTTCCTCTTTCTGGTGGCCGCTCCTACATGTGTCCTGTCCCAGGTGCAACTGAAGGAGTC
AGGACCTGGCCTGGTGAAGCCCTCGCAGACCCTGTCCCTCACCTGCACTGTCTCTGGATTCTCTTTGAGCAGTTACG
GTGTAGGCTGGGTCCGCCAGGCTCCAGGAAGGGGGCTGGAATGGGTTGGTGCGCTAGATAGTAGTGGAAGTAAAAAC
TACAACCCATCCCTGAAGTCCCGAGCCACCATCACCAAGGACACCTCAAAGAGCCAAGTGTATCTGACGCTGAACAG
CCTGACAGGCGAGGACACGGCCGTCTATTACTGTGCGAGGAGCTATGGCACCTGGGCTTTCGACAAGATAGCCTACT
GGGGCCAGGGAATCCTGGCCCACGTCTCCTCAGAGAGTACGATGACCCCAGATCTCTTCCCCCTCGTCTCCTGTGGG
CCCTCTCTTGATGAGAGCCTGGTGGCTGTGGGCTGCCTAGCCCGGGACTTCCTACCCAAG
>HM175904.1 Equus caballus clone 2H196 immunoglobulin heavy chain V-D-J region mRNA, partial cds

ATGAGTCACCTGTGGTTCTTCCTCTTTCTGGTGGCCGCTCCTACATGTGTCCTGTCCCAGGTACAACTGAAGGAGTC
AGGACCTGGCCTGGTGAAGCCCTCGCAGACCCTCTCCCTCACCTGCACTGTCTCTGGATTATATTTGAGCACGAATG
GTGTAGGCTGGGTCCGCCAGGCTCCAGGAAAAGGGCTGGAACGTGTTGGTGACATAATTGATGAGGGAAAAGCGTTC
TACTCATCAGTCCTGAAGTCCCGAGCCATCATCACCAGGGACACCTCAAAGAGCCAAGTTTATCTGACGCTGAACAG
CCTGACAAGCGAGGACACGGCCGTCTATTACTGCACAGCCGTTAGGGCTACGGATAACAGTGGCTTTGCCGGTAAAT
TTTACTCCAATATAAACTTCTGGGGCCAGGGCATCCTGGTCACCGTCTCCTCAGAGAGTACGATGACCCCAGATCTC
TTCCCCCTCGTCTCCTGTGGGCCCTCTCTTGATGAGAGCCTGGTGGCTGTGGGCTGCCTAGCCCGGGACTTCCTACC
CAAG
>HM175899.1 Equus caballus clone 2H533 immunoglobulin heavy chain V-D-J
region mRNA, partial cds
ATGAGTCCCCTGTGGTTCTTCCTCTTTCTGGTGGCCGCTCCTACATGTGTCCTGTCCCAGGTGCAACTGAAGGAGTC
AGGACCTGGCCTGGTGAAGCCCTCGCAGACCCTCTCCCTCACCTGCACTGTCTCTGGATTTTCTTTGAGAAATAATG
TTGTAGGCTGGGTCCGCCAGGCTCCAGGAAAAGGACTGGAATATGTTGGTGAAATGCCTTATAGTGGAGGTGCAAAC
TACAACCCAGCCCTGAAGTCCCGAGCCAGCATCAGCAAGGACACCTCAAAGAGCCAAGTTTATCTGACGCTGAACAG
CCTGACAAGCGAGGACACGGCCGTCTATCACTGTGCAGGCAACGCGTTGTTGAACGAAGACTGGTCAATCGCGGGCG
GAATAAACTACTGGGGCCAGGGCATCCTGGTCACCGTCTCCTCAGAGAGTACGATGACCCCAGATCTCTTCCCCCTC
GTCTCCTGTGGGCCCTCTCTTGATGAGAGCCTGGTGGCTGTGGGCTGCCTAGCCCGGGACTTCCTACCCAAG
>HM175896.1 Equus caballus clone 2H267 immunoglobulin heavy chain V-D-J
region mRNA, partial cds
ATGAGTCACCTGTGGTTCTTCCTCTTTCTGGTGGCCGCTCCTACATGTGTCCTGTCCCAGGTACAACTGAAGGAGTC
AGGACCTGGCCTGGTGAAGCCCTCGCAGACCCTCTCCCTCACCTGCACTGTCTCTGGATTATCTTTGAGCAGTAAGG
TTGTAGGCTGGGTCCGCCAGGCTCCAGGAAAAGGACTGGAATATGTTGGTGGTGTGTCTAATACTGGAATCGCGTAC
TACAACCCAGCCCTGAAGTCCCGAGCCAGCATCGCCAAGGACGCCTCAAAAAGCCAAGTTTATCTGACGCTGAACAG
CCTGACAACGGAGGACACGGCCGTCTATTACTGTGCAGGAAATGAACACACTACGACGCTTGCTGGAATAAGGTACT
GGGGCCAGGGCATCCTGGTCACCGTCTCCTCAGAGAGTACGATGACCCCAGATCTCTTCCCCCTCGTCTCCTGTGGG
CCCTCTCTTGATGAGAGCCTGGTGGCTGTGGGCTGCCTAGCCCGGGACTTCCTACCCAAG
>HM175895.1 Equus caballus clone 2H140 immunoglobulin heavy chain V-D-J
region mRNA, partial cds
ATGAGTCACCTGTGGTTCTTCCTCTTTCTGGTGGCCGCTCCTACATGTGTCCTGTCCCAGGTGCAACTGAAGGAGTC
AGGACCTGGCCTGGTGAAGCCCTCGCAGACCCTCTCCCTCACCTGCACTGTCTCTGGATTATCTTTGAGTAGTAATG
CTATAAGCTGGGTCCGCCAGGCTCCAGGAAAAGGGCTGGAACATGTTGGTGACATACGTTTTAATGGAGGTGAAAGG
TACGTCCCAGCCCTGAAGTCCCGAGCCCGCATCACCAAGGACACCTCAAAGAGCCAAGTTTATCTGACGCTGAACAG
CCTGACAAGCGAGGACACGGCCATCTATTACTGTGCAGGGGGTACTGATACAAACTCGTATCCCGTTGATGGCATAA
ACTACTGGGGCCAGGGCATCCTGGTCACCGTCTCCGCAGAGAGTACGATGACCCCAGATCTCTTCCCCCTCGTCTCC
TGTGGGCCCTCTCTTGATGAGAGCCTGGTGGCTGTGGGCTGCCTAGCCCGGGACTTCCTACCCAAG
>HM175892.1 Equus caballus clone 2H1422 immunoglobulin heavy chain V-D-J
region mRNA, partial cds
ATGAGTCACCTGTGGTTCTTCCTCTTTCCGGTGGCCGCTCCTACATGTGTCCTGTCCCAGGTGCAACTGAAGGAGTC
AGGACCTGGCCTGGTGAAGCCCTCGCAGACCCTCTCCCTCACCTGCACTGTCTCTGGATTGTCTTTGAGCAGTAATG
GTGTAGGCTGGGTCCGCCAGGCTCCAGGAAAAGGGCTGGAATATGTTGGTGGTATACATAATAATGGAGGTACAATG
TACGACCCAGCCCTGAAGTCCCGAGCCAGCGTCACCAAGGACACCTCAAAGAGCCAAGTTTATCTGACGCTGAACAG
CCTGACAAGCGAGGACACGGCCGTCTATTACTGTGCAGTACGAGCAGGACCGTGGTTTACTAGGGACGGTATAAACT
ACTGGGGCCAGGGCATCCTGGTCACCGTCTCCTCAGAGAGTACGATGACCCCAGATCTCTTCCCCCTCGTCTCCTGT
GGGCCCTCTCTTGATGAGAGCCTGGTGGCTGTGGGCTGCCTAGCCCGGGACTTCCTACCCAAG
>HM175891.1 Equus caballus clone 2H1142 immunoglobulin heavy chain V-D-J
region mRNA, partial cds
ATGAGTCACCTGTGGTTCTTCCTCTTTCTGGTGGCCGCTCCTACATGTGTCCTGTCCCAGGTGCAACTGAAGGAGTC
AGGACCTGGCCTGGTGAAGCCCTCGCAGACCCTCTCCCTCACCTGCACTGTCTCTGGATTATCTTTGAACAGTAATG
GTGTAGGCTGGGTCCGCCAGGCTCCAGGAAAAGGGCTGGATTATGTTGGTGGCATACGTGGAATAGAAAGTCCAATC
TACAACCCGGCCCTGAAGTCCCGAGCCAGCATCACCAAGGACACCTCAAAGAGCCAAGTTTATCTGACGCTGAACAG
CCTGACAAGTGAGGACACGGCCGTCTATTACTGTGCAGGACGTCCTGGTTACTATTCGACTTTGGCTGATGCGATAA
GTTACTGGGGTCAGGGCATCCTGGTCACCGTCTCCTCAGAGAGTACGATGACCCCAGATCTCTTCCCCCTCGTCTCC
TGTGGGCCCTCTCTTGATGAGAGCCTGGTGGC
>HM175889.1 Equus caballus clone 2H1251 immunoglobulin heavy chain V-D-J
region mRNA, partial cds

ATGAGTCACCTGTGGTTCTTCCTCTTTCTGGTGGCCGCTCCTACATGTGTCCTGTCCCAGGTGCAACTGAAGGAGTC
AGGACCTGGCCTGGTGAAGCCCTCGCAGACCCTCTCCCTCGTCTGCGTGGTCTCTGGATTATCACTGAGGACTAAAA
CTGTGACCTGGGTCCGCCAGGCTCCAGGAAAAGGGCTGGAATATGTTGGTGGTATCACTAGTGGTGGTGATGTAGAC
TACCACCCAGCCCTGCAGTCCCGAGCCAGCATCACCAAGGACACCTCCAAGAGCCAAGTTTATCTGACGCTGAACAG
CCTGACAAGCGAGGACACGGCCGTCTATTACTGTGCAGGACGAGAGTATGGTGACACTTACTTTTACCACGTAAACT
TCTGGGGCCAGGGCATCCCGGTCACCGTCTCCTCAGAGAGTACGATGACCCCAGATCTCTTCCCCCTCGTCTCCTGT
GGGCCCTCTCTTGATGAGAGCCTGGTGGCTGTGGGCTGCCTAGCCCGGGACTTCCTACCCAAG
>HM175888.1 Equus caballus clone 2H1443 immunoglobulin heavy chain V-D-J
region mRNA, partial cds
ATGAGTCACCTGTGGTTCTTCCTCTTTCTGGTGGCCGCTCCTACATGTGTCCTGTCCCAGGTGCAACTGAAGGAGTC
AGGACCTGGCCTGGTGAAGCCCTCGCAGACCCTCTCCCTCACCTGCACTGTCTCTGGATTATCTTTGAGCAGCCATG
CTGTAGGCTGGGTCCGCCAGGCTCCAGGGAAAGGACTGGAATATGTTGGTGGAATACGTAGTAGTGGAGTGGTGGAC
TATAACCCAGCCCTGAAGTCCCGAGCCAGCATCACCAAGGACACCTCAAAGAGCCAAGTTTATCTGACGCTGAACAG
CCTGACAACAGAGGACACGGTCGTCTATTACTGTTTAGGAGGCTCCGAAGACAATTATGTCGCTCATGCAATGATCT
ACTGGGGCCAGGGCATCCTGGTCACCGTCTCCTCAGAGAGTACGATGACCCCAGATCTCTTCCCCCTCGTCTCCTGT
GGGCCCTCTCTTGATGAGAGCCTGGTGGCTGTGGGCTGCCTAGCCCGGGACTTCCTACCCAAG
>HM175887.1 Equus caballus clone 2H1013 immunoglobulin heavy chain V-D-J
region mRNA, partial cds
ATGAGTCACCTGTGGTTCTTCCTCTTTCTGGTGGCCGCTCCTACATGTGTCCTGTCCCAGGTGCAACTGAAGGAGTC
AGGACCTGGCCTGGTGCAGCCCTCGCAGACCCTCTCCCTCACCTGCACTGTCTCTGGCTTATCTTTGAGCAGCACTG
ATGTAGGCTGGGTCCGCCAGGCTCCAGGAAAAGGGCTGGAATATGTTGGTGCTATAGCTAATAGTGGAGGTGCAAAC
TACAACTCAGCCCTGAGGTCCCGAGCCACCATCACTAAGGACGCCTCAAAGAGCCAAGTCTATCTGACGCTGAACAG
CCTGACAAGCGAGGACACGGCCGTCTATTACTGTGCAGGAGGAGGCACTGGTGGTTATATTGCCGATGATGTGCACT
ACTGGGGCCAGGGCATCCTGGTCACCGTCTCCTCAGAGAGTACGATGACCCCAGATCTCTTCCCCCTCGTCTCCTGT
GGGCCCTCTCTTGATGAGAGCCTGGTGGCTGTGGGCTGCCTAGCCCGGGACTTCCTACCCAAG
>HM175886.1 Equus caballus clone 2H108 immunoglobulin heavy chain V-D-J
region mRNA, partial cds
ATGAGTCACCTGTGGTTCTTCCCCTCTCTGGTGGCCGCTCCTACATGTGTCCTGTCCCAGGTGCAACTGAAGGAGTC
AGGACCTGGCCTGGTGAAGCCCTCGCAGACCCTCTCCCTCACCTGCACTGTCTCTGGATTATCTTTGAGCAGTAATG
CTGTAGGCTGGGTCCGCCAGGCTCCAGGAAAAGGGCTGGAACGTGTTGGTGGTATGCACAGTAGTGGTAGTGCGTTC
TACAACCCAGCCCTGAAGTCCCGAGCCAGCATCACCAAGGACACCTCAAAGAGCCAAGTTTATCTGACGCTGAACAG
CCTGACAAGCGAGGACACGGCCGTCTATTACTGTGCAGGGTCGGGGAACGGCTATGCTAATCCCGATGGTATAAAAT
ACTGGGGCCAGGGCATCCTGGTCACCGTCTCCTCAGAGAGTACGATGACCCCAGATCTCTTCCCCCTCGTCTCCTGT
GGGCCCTCTCTTGATGAGAGCCTGGTGGCTGTGGGCTGCCTAGCCCGGGACTTCCTACCCAAG
>KC549798.1 Equus caballus clone 5'RACE Adult MLN T33 immunoglobulin heavy
chain mRNA, partial cds
GTGAATCTCATCTGCAAGAACATGAATCACCTGTGGTTCTTCCTCTTTCTGGTGGCCGCTCCTATATGTGTCCTGTC
CCAGGTGCAACTGAAGGAGTCGGGACCTGGCCTGGTGAAGCCCTCGCAGACCCTCTCCCTCACCTGCTTTGTCGCTC
AATCCCCTGACAGCCGTTATCCTGTACACTGGGTCCGCCAGGCTCCAGGAAGAGGGCTGGAATATGTTGCTGGCGTG
TACGGAACTGGAGTGCCAATGTACAATCCAGCCCTGAAGTCCCGAGCCAGCATCACCAAGGACACAACAAAGAGCCA
GGTTTATCTGACGCTGAACAGCCTGACAGGCGAGGACACGGCCGTCTATTTTTGTGCAGCCCGGGGTGAAAATATGG
TAGCGGGATATGGCTACTGGGGCCAGGGCACC
>KC549797.1 Equus caballus clone 5'RACE Adult MLN T18 immunoglobulin heavy
chain mRNA, partial cds
GTGAATCTCATCTGCAAGAACATGAATCACCTGTGGTTCTTCCTCTTTCTGGTGGCCGCTCCTACATGTGTCCTGTC
CCAGGTGCTACTGAAGGAGTCAGGACCTGGCCTGATGAAGCCCTCGCAGACCCTCTCCCTCACCTGTACTGTGTCTG
GATTCTCTTTGAGTGATTATAATGTAGCCTGGGTCCGCCAGGCTCCAGGAAAAGGGCTGGAATACTTAGGTAGTGTG
GAGAGGGATGGAGATAAAAACTACAATCCAGTCCTGAAGTCCCGAGTCATAGTCAATAAGGACACCTCAAAGAGCCA
AGTCATTCTGACGTTAATGGAGCTGACAGGCTCGGACACGGCCGTCTATCACTGTGCGAGACGTCGTTCGAATCTGC
TCGACGGGATCTATTACGACTACTGGGGCCAGGGCCTC
>KC549796.1 Equus caballus clone 5'RACE Adult MLN T17 immunoglobulin heavy
chain mRNA, partial cds
GTGAATCTCATCTGCAAGAACATGAATCACCTGTGGTTCTTCCTCTTTCTGGTGGCCGCTCCTACATGTGTCCGATC
CCAGGTGGAACTGAAGGAGTCGGGACCTGGCCTGATGAAGCCCTCGCAGACCCTCTCCCTCGCCTGCACTGTCTCTG
GATTCTCTTTGAGCACCTACGGTGTACACTGGGTCCGCCAGGCTCCAGGAAAAGGGCTGGAATACGTTGCCGGCATG

GCTCCTAGTGGTAGTGAGAGATACAGCCCAGAGCATAAGTCCCGAGCCAGCATCACCAGAGACACCTCAAAGAGCCA
AGTTTATCTGACGGTGAACAGCCTGACAGGCGAGGACACGGCCGTCTATTACTGTGCGACGAAGGACAGTGAGGGTT
ATACCTACTACTCCTGGGGCCAGGGCATC
>KC549795.1 Equus caballus clone 5'RACE Adult MLN T14 immunoglobulin heavy chain mRNA, partial cds
GTGAATCTCATCTGCAAGAACATGAATCACCTGTGGTTCTTCCTCTTTCTGGTGGCCGCTCCTACATGTGTCCTGTC
CCAGGTGCAACTGAAGGAGTCAGGACCTGGCCTGGTGAAGCCCTCGCAGACCCTCTCCCTCACCTGCACTGTCTCTG
GATTCTCTTTGAGCTTTTATGGTGTAGGCTGGGTCCGCCAGGCTCCAGGAAAAGGACTGGAATATGTTGGATCTATG
ATGAATGATGGAAGTACGTGGTACAACCCAGCCCTGAAGTCCCGAGCCAGCATCACCAAGGACACCTCAAGGAGCCA
AGTTTATCTGACGCTGAACAGCCTGACAGGCGAGGACACGGCCGTCTATTTTTGTGTGAGAACGTATGGTTATGAAA
GTGCTGAATACTCTGGCTACTGGGGCCAGGGCACC
>KC549794.1 Equus caballus clone 5'RACE Adult MLN T02 immunoglobulin heavy chain mRNA, partial cds
GGGAGTCGTGAATCTCATCTGCAAGAACATGAATCACCTGTGGTTCTTCCTCTTGCTGGTGGCCGCTCCTACATGTG
TCCTGTCCCAGGTGCAACTGAAGGAGTCGGGACCTGGCCTGGTGAAGCCCTCGCAGACCCTGTCCCTCACCTGTACT
GTCTCTGGATACTCTCTGAGCAGACTGGTAGTGGGTTGGGTCCGCCAGGCTCCAGGAAGAGGATTGGAGTGGGTTGC
CGCAGAGTATTCTGATGGACGGACATATTACAATGCAGCCCTGAAGTCCCGAACCAGTATCACCAAGGACGCCTCAA
GGAGACAAGTCGCACTGACGTTGAACAGCCTGACAGGCGAAGACACGGCCGTCTATTACTGTCTAGGAGATGACACA
GACGGGGAGGAATTTGCCTACTGGGGCCAGGGCTCC
>KC549793.1 Equus caballus clone 5'RACE Adult MLN T20 immunoglobulin heavy chain mRNA, partial cds
GGGAGTCGTGAATCTCATCTGCAAGAACATGAATCACCTGTGGTTCTTCCTCTTTCTGGTGGCCGCTCCTACATGTG
TCCTGTCCCAGGTGCAACTGAAGGAGTCGGGACCTGGCCTGGTGAAGCCCTCGCAGACCCTGTCCCTCACCTGCACA
GTCTCTGGACTATCTTTGACTAATAATGCTGTAGGCTGGGCCCGCCAGGCTCCAGGAAAAGCGCTGGAGTGGGTTGG
TGTATTGTATGGTGGTAGGAATACGCTATACAACCCAGCCCTGAAGTCCCGAGCCATCATCACCGTCACCGAGGACA
CCCGGAAGAGCCAAGTTCATCTGACGCTGAACAGTCTGACAGGCGAAGACACGGCCGTCTATTACTGTGCAGCCGAA
ACAGAGTTTGGCGTGTCAAACTCATACTGGATTAGTAGATTTTATGGTTACGTGGATCACTGGGGCCAGGGCACC
>KC549792.1 Equus caballus clone 5'RACE Adult MLN T27 immunoglobulin heavy chain mRNA, partial cds
GGGAGTCGTGAATCTCATCTGCAAGAACATGAATCACCTGTGGTTCTTCCTCTTTCTGGTGGCCGCTCCTACATGTG
TCCTGTCCCAGGTGCAACTGAAGGAGTCGGGACCTGGCCTGGTGAAGCCCTCGCAGACCCTCTCCCTCACCTGCACT
GTCTCTGGATTGTCTTTGAGCAGTGAAACTGTAGCCTGGGTCCGCCAGGCTCCAGGAAAAGGGCTGGAAAGCGTTGG
TCGTGTGGTTGTTGGTGGTGTAACAGTATACAACCCAGCCCTAAAGTCCCGAGTCAGCGTCACCAAGGACACCTCAA
AGAGCCAAGTTTATCTGACGCTGAACAGCCTGACAGGCGAGGACACGGCAGTCTATTACTGTGCGAGAATAACTTTG
GATAAGGTTGGCTATTGGGGCCAGGGTATC
>KC549791.1 Equus caballus clone 5'RACE Adult MLN T05 immunoglobulin heavy chain mRNA, partial cds
TTTTTCAGGGAGCCGTGAATCTCATCTGCAAGAACATGAGTCACCTGTGGTTCTTCCTCTTTCTGGTGGCCGCTCCT
ACATGTGTCCAGTCCCAGGTGTATCTGGAGGAGTCAGGGCCCGGCCTGGTGAAGCCCGGGCAGACCCTCTCCCTCAT
CTGTGCTGTCTCTGGATATTCTTTGACGGAATATGGAGTGGCCTGGGTCCGCCAGGCTCCGGGAAAAGGGCTGGAGT
TTGTTGCTGGCATAGTTAGCGGTGGACAGGAATTCTACAGTCCAGCCCTGAAGGCCCGAGTAGAAATCACCAGGGAC
GTTTCAAAGAGTCAGCTTCGCCTGACTCTGAACACCCTAACGGGCGAGGACACGGCCGTCTATTACTGTGCAGGAGG
CTCCGGACACGGAGTGTCCGTCTTTGGCTACTGGGGCCAGGGCACC
>KC549790.1 Equus caballus clone 5'RACE Adult MLN T03 immunoglobulin heavy chain mRNA, partial cds
ATTTTCAGGGAGTCGTGAATCTCATCTGCAAGAACATGAGTCACCTGTGGTTCTTCCTCTTTCTGGTGGCCGCTCCT
ACATGTGTCCTGTCCGTGGTGAAACTGAAGGAGTCAGGACCTGGCCTGGTGAAGCCCTCGCAGACCCTCTCCCTAGT
CTGCACTGTCTCTGGAACATCTTTGAGTAGTAATGCGGTCGGCTGGGTCCGCCAGGCTCCAGGAAAGGGGCTTCAAT
ATGTTGGTGGAATAGCTAGTGACGGAGATGCAAACTTCAACCCAGCCCTGAAGTCCCGCGCCAGCATCACCAGGGAC
ACCTCAAAGAGCCTAGTTTATCTGACACTAGACAGCCTGACAGGCGAGGACACGGCCGTCTATTACTGTGCGTCAGA
TGCCGCCTACTACTCCTACTATTTTACGGGTATAGTCTATTGGGGCCCGGGCATC
>KC549789.1 Equus caballus clone 5'RACE Adult MLN T23 immunoglobulin heavy chain mRNA, partial cds
ATTTTCAGGGAGTCGTGAATCTCATCTGCAAGAACATGAGTCACCTGTGGTTCTTCCTCTTTCTGGTGGCCGCTCCT
ACATGTGTCCTGTCCCAGGTTCAACTGAAGGAGTCAGGACCTGGCCTGGTGAAGCCCTCGCAGACCCTGTCCCTCAC
ATGCACTGTCTCCGGAATGTCTTTGACAAGTCACGGTATAGGCTGGGTCCGTCAGGCTCCAGGAAAAGGCCTGGAAT

TTGTAGGTGGCACGACTAGTAGTGGAACAGTGTACAATCCAGCCCTGAAGTCCCGAGCCAGTATCACCAAGGACACC
TCAAAGAGCCAAGTTTATCTGACGCTGAACAGCCTGACAGTTCTCGACACGGCCGTCTATTACTGTGCGAGGACCCA
GGATTACTGGGGTTATTCCATCACCGCATACTATGGTTACGGCTACGTGGACTACTGGGGCCAGGGCACC
>KC549788.1 Equus caballus clone 5'RACE Adult MLN T01 immunoglobulin heavy chain mRNA, partial cds
ATTTTCAGGGAGTCGTGAATCTCATCTGCAAGAACATGAGTCACCTGTGGTTCTTCCTCTTTCTGGTGGCCGCTCCT
ACATGTGTCCTGTCCCAGGTGCAACTGAAGGACTCAGGACCTGGCCTGGTGAAGCCCTCGCAGACCCTCTCCCTCAC
CTGCACTGTCTCCGGATTAACATTGAACAATATTGGTGTGGGCTGGGTCCGCCAGGCTCCAGGAAAAGGCTTGGAGT
TCCTTGGCGGTGTTGCAAGTAGAGGTGGTGGTGTACCAGCGAGTGGAAGTGTAAACTTAAGCCCAGACCTGAAGTCC
CGAGCCAGCATCACCACGGACACTTCACAGAGCCAAGTGTATCTGACGCTGAACAGTCTGACAAGCGAGGACACGGC
CGTCTATTATTGTGCAGGTTATTTAGGGGGTGCTCATTCCGTGTATCCCTTATTTAGGGTGGATTATTGGGGCCAAG
GCGTC
>KC549787.1 Equus caballus clone 5'RACE Adult MLN T13 immunoglobulin heavy chain mRNA, partial cds
ATTTTCAGGGAGTCGTGAATCTCATCTGCAAGAACATGAGTCACCTGTGGTTCTTCCTCTTTCTGGTGGCCGCTCCT
ACATGTGTCCTGTCCCAGGTGCAACTGAAGGAGTCAGGACCTGGCCTGATGAAGCCCTCGCAGACCCTCTCCCTCAT
CTGCACTGTCTCTGGATTGGATGTGATGAGTTATGGTGTGGGCTGGGTCCGCCAGGCTCCAGGAAAAGGGCTGGAGT
GGATTGCTGCGCATTATGGTAGACTAAGTTCCGGCGGTGAAAGACAGTACTACAATCCATCCCTGAAGTCCCGAGCC
AGCATCACCAAGGACTACTCAATGAGCCGAGTTTATCTGACGCTCTACAGCCTGACGGACGAGGACGCGGCCGTCTA
TTACTGTGTGGGAGGAATGGCATCCGGTTATCATCAACCTGACTCTTTTGCCTTGTGGGGCCAGGGCACC
>KC549786.1 Equus caballus clone 5'RACE Adult MLN T06 immunoglobulin heavy chain mRNA, partial cds
ATTTTCAGGGAGTCGTGAATCTCATCTGCAAGAACATGAGTCACCTGTGGTTCTTCCTCTTTCTGGTGGCCGCTCCT
ACATGTGTCCTGTCCCAGGTGCAACTAAAGGAATCAGGACCTGGCCTGGTGAAGCCCTCGCAGACCCTCTCCCTCAC
CTGCTCTGTCTCTGGTTTATCAATGGCCGAAGATACGATAGTATGGGTCCGCCAGGCTCCAGGAAAAGGTCTGGAAT
TTCTTGGTGAGATATATAGCACTACTGGCGGTGGAAGTCGCAACCCAGCTCTGGAGTCCCGACTCATTATCACCAGG
GACACCGGAAAGAGCCAAGTTTATCTGTCACTGGACAGCCTGACAAGCGAAGACACGGCCGTCTATTACTGTGCAGG
ATCACTGGGTACATCCTGGTATGCGGGCCTTGGCCGCTGGGGCCAGGGCATT
>KC549785.1 Equus caballus clone 5'RACE Adult MLN T28 immunoglobulin heavy chain mRNA, partial cds
TTTCAGGGAGTCGTGAAGCTCATCTGCAAGAACATGAATCACCTGTGGTTCTTCCTCTTTCTGGTGGCCGCTCCTAC
ATGTGTCCTGTCCCAGGTGCAACTGAAGGAGTCAGGACCTGGCCTGGTGAAGCCCTCGCAGACCCTCTCCCTCACCT
GCACTGTCTCTGGATTATCTGTGTACAGTGGTGCTGTGGGCTGGGTCCGCCAGGCTCCAGGAAAAGGGCTGGAATTT
GTGGGTGCCTGTTATGGTAGTACAGTTAAAAATTACAACCCAGCCCTGAAGTCCCGGGCCAGCATCACCAAGGACGA
CTCAAAGGGCCAAGTTTATCTGACGCTGAACAGCCTGACAGGCGAGGACACGGCCGTCTATTACTGTGCGAGAGCCT
ATAGCGGTTATGTTTATACTGCCGGCTATTTCGGGCTGGTGTTTTGGGGCCAGGGAATC
>KC549784.1 Equus caballus clone 5'RACE Adult MLN T12 immunoglobulin heavy chain mRNA, partial cds
CGTGAATCTCATCTGCAAGAACATGAGTCACCTGTGGTTCTTCCTCTTTCTGGTGGCCGCTCCTACATGTGTCCTGT
CCCAGGTGCAATTGAAGGAGTCAGGACCTGGCCTGGTGAAGCCCTCCCAGACCCTCTCCCTCACCTGCACTGTCTCT
GGACTCACTTTGAGCAATAGCGCTGTGAGCTGGGTCCGCCAGGTTCCAGGAAAAGGGCTTGAATATGTCGGTGGTAT
AACTGGTGATGGAAGTACAATGCACAATCCCGCCCTGAAGTCCCGAGCCAAGGTCACCAAGGACACCTCAAAGAGTC
AAGTGTATCTGACTCTGAACAGCCTGACAAGCGAGGACACGGCCGTCTATTTTTGTGCAGGAGGAGTCTTTGTCAAG
ACCATGGATAGCGAGTGGCCTGACGGTATACTCTACTGGGGCCAGGGCATC
>KC549783.1 Equus caballus clone 5'RACE Adult MLN T32 immunoglobulin heavy chain mRNA, partial cds
ATTTTCAGGGAGTCGTGAATCTCATCTGCAAGAACATGAGTCACCTGTGGTTCTTCCTCTTTCTGGTGGCCGCTCCT
ACATGTGTCCTGTCCCAGGTGCAACTGAAGGAGTCAGGACCTGGCCTGGTGAAGCCCTCGCAGACCCTGTCCCTCAC
TTGCACTGTCTCTGGACTGTCTTTGAGGAGTTACGCTGTACACTGGGTCCGCCAGGTTCCAGGAAAAGGGCTGGAAT
TTGTTGGTGTTATGGGTACCGGTGGTAGTGCAGCATACAATCCAGCCCTGAAGTCCCGAGCCAGCATCACCGTGGAC
ACCAAGAAGAGCCAAGTTTATCTGACGCTGAACAGCCTGACAGGCGAGGACACGGCCGTCTATTTTTGTGCGGGGTC
AAGTGACTTTACCGGCTGGGAGATAAACTCCTGGGGCCAGGGCATC
>KC549782.1 Equus caballus clone 5'RACE Adult MLN T10 immunoglobulin heavy chain mRNA, partial cds

ATTTTCAGGGAGTCGTGAGTCTCATCTGCAAGAACATGAGTCACCTGTGGTTCTTCCTCTTTCTGGTGGCCGCTCCT
ACATGTGCCCTATCCCAGGTGCAACTGAAGGAGTCAGGACCTGGCCTGGTGAAGCCCTCGCAGACCCTCTCCCTCAC
CTGCACTGTCTCCGGATTATCTTTGAGCGACACAGCTGTAGGCTGGGTCCGCCAGGCTCCAGGAAAAGGGTTAGAAT
TCATTGGTGGGATTGCCACTAGTGGGGTACTGAAGTACAACCCAGCCCTAGAACCCCGAGTCAGTATCACCAGGGAC
ACCTTAAAGAACATAGTTTATCTGACGCTGAAGGACGTGACAACCGAAGACACGGCCGTCTATTATTGTGCGGGGGG
CGGAAAGAATTACTATCATGAATGGTACAGCAATTACTATGATGGCTACGTGGATCACTGGGGCCAGGGCACC
>KC549781.1 Equus caballus clone 5'RACE Adult MLN T26 immunoglobulin heavy chain mRNA, partial cds
ATTTTCAGGGAGTCGTGAATCTCATCTGCAAGAACATGAATCACCTGTGGTTCTTCCTCTTTCTGGTGGCCGCTCCT
ACATGTGTCCTGTCCCAGGTGCAACTGAAGGAGTCCGGACCTGGCCTGCTGAAGCCCTCGCAGACCCTCTCCCTCTC
CTGCAGTGTCTCTGGATTATCTGTGAGCAGTAATGCTGTAGGCTGGGTCCGCCAGGCTCCAGGAAAAGGGCTGGAAT
TTGTTGCCACTATATATGAGTGGCATGGTATGTCTGCAGACTACAACCCAGCCCTGAAGTCCCGAGCCATCATCACC
AAGGACACCTCAAAGAACGTGGTTGCTCTGACGCTGAACGATCTGACAAGCGAAGACGAGGCCGTCTATTACTGTGC
AGGAGTCATCGATGGTTACCGTGGTTATTTCCTCCAATATCAATTTGGCTACTGGGGCCAGGGCACC
>KC549780.1 Equus caballus clone 5'RACE Adult MLN T22 immunoglobulin heavy chain mRNA, partial cds
ATTTTCAGGGAGTCGTGAATCTCATCTGCAAGAACATGAGTCACCTGTGGTTCTTCCTCTTTCTGGTGGCCGCTCCT
ACATGTGTCCTGTCCCAGGTGCAACTGAAGGAGTCAGGACCTGGCCTGGTGAAGCCCTCGCAGACCCTCTCCCTCAC
CTGCTATGTCTCTGGATTATCTTTGAGCAGTTATGCGGTGACCTGGTTCCGCCAGGCTCCAGGAAAAGGGCTGGAAT
TTGTTGGTGAAATACCTGACAGTGGAAGTAATGTCTACAACCCAGCCCTGAAGTCCCGAACCAGCATCACCAAGGAC
ACCTCAAAGAGCCAGGTTTATCTGACGCTGAACAGCCTGACAAGCGAGGACACGGCCGTCTATTATTGTGCTGCGTC
GCTTTTCTGGGGCCAGGGCATC
>KC549779.1 Equus caballus clone 5'RACE Adult MLN T24 immunoglobulin heavy chain mRNA, partial cds
ATTTTCAGGGAGTCGTGAATCTCATCTGCAAGAACATGAGTCACCTGTGGTTCTTCCTCTTTCTGGTGGCCGCTCCT
ACATGTGTCCTGTCCCAGGTACAACTGAGGGAGTCAGGACCTGGCCTGGTGAAGCCCTCGCAGACCCTGTCCCTCAC
CTGCACTGTCTCTGGATTATCTTTGAGCAGCAGTGTTGTAGCGTGGGTCCGCCAGGCTCCAGGAAAAGGACTGGAGT
GGGTTGGTAATATACATGGTAGCGAAATGACATACTACAATCCTGCTCTGAAGTCCCGAGCCAGCATCACCAAGGAC
GTTGCAAAGGGTCAAGTTTATCTGACGCTGAACAAGATGACAGGCGAAGACACGGCCGTCTATTACTGTACAGGGGA
TAGTACTTTTCTTTACTACGCGGGAGGCTTAAGTTACTGGGGCCAGGGCATC
>KC549778.1 Equus caballus clone 5'RACE Adult MLN T19 immunoglobulin heavy chain mRNA, partial cds
CAAGAACATGAGTCACCTGTGGTTCTTCCTCTTTCTGGTGGCCGCTCCTACATGTGTCCTGTCCCAGGTGCAACTGA
AGGAGTCAGGACCTGGCCTGGTGAAGCCCTCGCAGACCCTCTCCCTCACCTGCAATGTCTCTGGATTATCTTTGAGC
AGTCATGGCGTAGGCTGGGTCCGCCAGGCTCCAGGAAAAGGGCTGGAATATGTTGGTGGTATGGCACAAAGTGGAAA
TCTTAAGCTCAATCCAGCCCTGAAGGACCGGGTCAGTATCACCAAGGACGTTTCAAAGAGCCAAGTTTACCTGACAC
TGAATCGCCTGACAAGCGAGGACACGGCCGTCTATTACTGTGCAGGAGGCGAGTCGTTGTTCAATAATTATCATAGT
GGCTGGGTCGGTCTTGACATAAAGTATTGGGGCCAGGGCATC
>KC549777.1 Equus caballus clone 5'RACE Adult MLN T29 immunoglobulin heavy chain mRNA, partial cds
ATTTTCAGGGAGTCGTGAATCTCATCTGCAAGAACATGAGTCACCTGTGGTTCTTCCTCTTTCTGGTGGCCGCTCCT
ACATGTGTCCTGTCCCAGGTACAACTGAAGGAGTCAGGACCTGGCCTGGTGAAGCCCTCGCAGACCCTCTCCCTCAC
CTGCACTGTGTCTGGATTATCTTTGAGCAGTAATACTGTAGGCTGGGTCCGCCAGGCTCCAGGAAAAGGGCTGGAAT
TTGTTGGTAACATAGATGGATATGAAAGATTAAGATTCAACCCAGCCCTGAAGCCCCGAGCCAGCATCACCAAGGAC
ACCTCAAAGAGACGGGTTTATCTGACGCTGAACAGCCTGACAAGCGAGGACACGGCCGTCTATTTCTGTACAGGAGC
CCAGTCCGATGGTGGGAGTTATTGGGCGGTCAACGGTGTGAACGTCTGGGGCCAGGGCATC
>KC549776.1 Equus caballus clone 5'RACE Adult MLN T11 immunoglobulin heavy chain mRNA, partial cds
ATTTTCAGGGAGTCGTGAGGATCTCATCTGCAAGAACATGAGTCACCTGTGGTTCTTCCTCTTTCTGGTGGCCGCTC
CTACATGTGTCCTGTCCCAGGTGCAACTGAAGGAGTCAGGACCTGGCCTGGTGAAGCCCTCGCAGACCCTCTCCCTC
ACCTGCACTGTCTCTGGATTATCTTTGAGCAGTTATAATGTGGGCTGGGTCCGCCAGGCTCCAGGAAAAGGGCTGGA
ATTTGTTGCGGATATAGGCACTAGTGGAGATAGATGGAACAACCCCGCCCTGAAGTCCCGAGCCAGCATCACCAAGG
ACACCTCAAAGAGCCAAGTTTATCTGACGCTGAACAGCCTGTTAATAGAGGACACGGCCGTCTATTATTGTATAGGA
AGTGGCAGACCTCCAGTTTACTGGGGCCAGGGCATC
>KC549775.1 Equus caballus clone 5'RACE Adult MLN T31 immunoglobulin heavy chain mRNA, partial cds

```
ATTTTCAGGGAGTCGTGAATCTCATCTGCAAGAACATGAGTCACCTGTGGTTCTTCCTCTTTCTGGTGGCCGCTCCT
ACATGTGTCCTGTCCCAGGTGCAACTGAAGGAGTCAGGACCTGGCCTGGTGAAGCCCTCGCAGACCCTGTCCCTCAC
CTGCGATGTCTCTGGATTCTCTTTGAGAGACTACGGTGTAGGCTGGGTCCGCCAGGCTCCAGGAAAAGGGCTGGAAC
TTGTGGGTGATGTGTCCAGTAGTGGGAAGGGGTTCTACAACCCAGTCCTGAAGTCTCGAGTCAGCATCACCAAGGAC
ATTTCAAGGGGCCTTTTCTATCTGACGCTGAACAGCCTGACTGGCGAGGACACGGCCGTCTATTATTGTGCTAGCGC
TTATAACGGAGATGGTAGTGTGGCGGATGGTATCGTCTACTGGGGCCAGGGCATC
>KC549774.1 Equus caballus clone 5'RACE Adult MLN T34 immunoglobulin heavy
chain mRNA, partial cds
ATTTTCAGGGAGTCGTGAATCTCATCTGCAAGAACATGAATCACCTGTGGTTCTTCCTCTTTCTGGTGGCCGCTCCT
ACATGTGTCCTGTCCGAGGTGCAACTGAAGGAGTCGGGACCTGGCCTGGTGAAGCCCTCGCAGAGCCTGTCCCTCAC
CTGCACTGTCTCTGGATTCTCTTTGAACGAGTACGCTGTAGGCTGGGTCCGCCAGGCTCCAGGAAAAGGGCTTGAAT
ATGTTGGTGATATACCTGCCTTTGGAAGTGCGAACTACAATCCAGCCCTGAAGTCCCGAGCCAGCATCACCAAGGAC
ACCTCAAAGGAGCTACTTTATCTGACGGTGAACAGCCTGACAGGCGAAGACACGGCCGTCTATTACTGTGTAGGATC
CCTTTACGGTTCTGGTCTCTATGGCATTCTTCACTGGGGCCAGGGCATC
>KC549773.1 Equus caballus clone 5'RACE Adult MLN T04 immunoglobulin heavy
chain mRNA, partial cds
ATTTTCAGGGAGTCGTGAATCTCATCTGCAAGAACATGAGTCACCTGTGGTTCTTCCTCTTTCTGGTGGCCGCTCCT
ACATGTGTCCTGTCCCAGGTGCAACTGAAGGAGTCAGGACCTGGCCTGGTGAAGCCCTCGCAGACCCTGTCCCTCAC
CTGCACTGTCTCTGGATTCTCTTTGAACAGCCACGGTGTCTACTGGGTCCGCCAGGCTCCAGGAAAGGGGCTGGAAT
TTGTTGGGATGGTTGGTCGCAGCGGAACACCGTACTACAACCCAGTCCTGAAGTCCCGAACATCCATTACCAGGGAC
ACTTCAAAGGCCAGAGTTTCCCTGACGCTGGATAGTCTGACAGGCGAGGACACGGCCGTCTATTACTGTGCGAGTTC
CTTGTGGTCAATGTTTAGGACTTATGGTATAAGTGACTGGGGCCAGGGCATC
>KC549772.1 Equus caballus clone 5'RACE Adult MLN T07 immunoglobulin heavy
chain mRNA, partial cds
ATTTTCAGGGAGTCGTGAATCTCATCTGCAAGAACATGAGTCACCTGTGGTTCTTCCTCTTTCTGGTGGCCGCTCCT
ACATGTGTCCTGTCCCAGGTGCAACTGAAGGAGTCAGGACCTGGCCTGGTGAAGCCCTCGCAGACCCTGTCCCTCAC
CTGCACTGTCTCTGGATTCTCTTTGGACAGTTACGGTGTGGGGTGGGTCCGCCAGGCTCCAGGAAAAGGGCTGGAGA
TGGTTGGTGGTATAACTAGTAAAAAGGGTGCAGACTACAATCCAGCCCTGAAGTCCCGAGCCAACATCACCAAGGAC
ACCTCAAAGAATCAAGTTTACTTGACACTGAACAGCCTGACAGGCGAGGACACGGCGGTCTATTACTGTACATTATT
GTATAGTCTGGGATACACTTTACGTGATGACTTTCGGCCCCCGGAATCTGGTGGCAATTACTGGGGCCAGGGCATC
>KC549771.1 Equus caballus clone 5'RACE Adult MLN T25 immunoglobulin heavy
chain mRNA, partial cds
ATTTTCAGGGAGTCGTGAATCTCATCTGCAAGAACATGAGTCACCTGTGGTTCTTCCTCTTTCTGGTGGCCGCTCCT
ACATGTGTCCTGTCCCAGGTGCAACTGAAGGAGTCAGGACCTGGCCTGGTGAAGCCCTCGCAGACCCTGTCCCTCAC
CTGCACTGTCTCTGGATTCTCTTTGAGCAGTTACGGTGTAGGCTGGGTCCGCCAGGCTCCAGGAAAAGGGCTGGAAT
ATGTTGGTGGTATTCATGGTAGTGGAAGTACAATCTACAACCCAGCCCTGAAGTCCCGAGCCAGCATCACCAAGGAC
ACCTCAAAGACCCAAGTTTATCTGACGCTGAACAGCCTGACAGGCGACGACACGGCCGTCTATTACTGTGCAAAATC
CACCTTGGAATGGGGTAACAGTGCTGATAATTACGCAAACTGGGGCCAGGGCACC
>KC549770.1 Equus caballus clone 5'RACE Adult MLN T15 immunoglobulin heavy
chain mRNA, partial cds
ATTTTCAGGGAGTCGTGAATCTCATCTGCAAGAACATGAGTCACCTGTGGTTCTTCCTCTTTCTGGTGGCCGCTCCT
ACATGTGTCCTGTCCCAGGTGCAACTGAAGGAGTCAGGACCTGGCCTGGTGAAGCCCTCGCAGACCCTGTCCCTCAC
CTGCACTGTCTCTGGATTCTCTTTGAGCAGTTACGGTGTAGGCTGGGTCCGCCAGGCTCCGGGAAAAGGGCTGGAAT
TTGTTGGTGGAATATATAGTGGTGGAAGTGTAAAGTACAAACCAGCCCTGAAGTCCCGAGCCAGCATCACCAAGGAC
ACCTCAAAGAGCCAAGTTTATCTGACGCTGAACAGCCTGACAGGCGAGGACACGGCCGTCTATTACTGTGCGAGTAG
CAGCACTACTTACTATTCTACTGTAACAACCTGGGGCCAGGGCATC
>KC549769.1 Equus caballus clone 5'RACE Foal MLN T30 immunoglobulin heavy
chain mRNA, partial cds
CATCACCCAAACATCACACTCCCTCCCCTACAGAAGCCCTGAGACCACAGTGCCTCACAATGGGCTGGAGCTGGAGA
ATCCTCTTCTTGGTGGCAGTAGCTTCAGGTGTCTCCTCCGAGGGTCAGCTGGAACAGTCGGGGCCGGAGTTGAAGAA
GCCTGGGTCATCAGTGAAGATCTCCTGCAAGGCTTCTGGATACACCTTCAGTAGCTATGCTGTGCACTGGGTGCGAC
AGGCCAATGGAAAAGGGATTGAGTGGATGGGATCTATCTATGCTGAATATGATGATACAAGCTACGCACCGAAGTTC
CAGGGCAGAGTCACCATGACTGCGGACAAGTCCACGAGCACAGTCTACATGGAGCTGAGCAGTCTGACATCTGAGGA
CACGGCCGTGTATTACTGTGCGTCAACGTATATGGTGACTGATTATTATGGTGCTATTGACTACTATTTTGGCTACT
GGGGCCAGGGCACC
```

>KC549768.1 Equus caballus clone 5'RACE Foal MLN T16 immunoglobulin heavy chain mRNA, partial cds
ACAGTTGTCTCCTCAGACAGGGCTGAGCTCTCTGGGGAAGGCAGAGTGTAATCTAGAAGAAGATGAGACTCTTGTGT
CTTCTCCTTTTCCTGGTGACGGCTCCCCAAGGAGTCCTGTCCCAGGTGCAGCTGCAGGAGTCGGGCCCAGGACTGGT
GCAGCCCTCACAGACCCTGTCCCTCACCTGCACTGTCACTGGAGGCTCCATCACAAGCAGCTATTCTAGCTGGAGCT
GGTTACGCCAGCCTCCAGGGAAGGGGCTGGAGTACATGGGATACATATATTATGATGGTAGAACTTACTACAATCCT
TCCTTCAAGAGCCGCACCTCCATCTCCAGAGACACCTCCAAGAACCAGTTCTCCCTGCAGCTGAGCTCCGTGACCAC
CGAGGACGCGGCCGTGTATTACTGTGCAAGAGAGGTCATAAATACTTATGGTGGTGCTTACTACCATTATTATGGTA
TAGCTTACTGGGGCCAGGGCATC
>KC549767.1 Equus caballus clone 5'RACE Foal MLN T23 immunoglobulin heavy chain mRNA, partial cds
ATTTTTCAGGGAGTCGTGAATCTCATCTGCAAGAACATGAATCACCTGTGGTTCTTCCTCTTTCTGGTGGCCGCTCC
TACATGTGTCCTGTCCCAGGTGCAACTGAAGGAGTCAGGACCTGGCCTGGTGAAGCCCTCGCAGACCCTCTCCCTCA
CCTGCACTGTCTCTGGATACTCTTTGAGCACATATAATGTAGGCTGGGTCCGCCAGGCTCCAGGAAAAGGGCTGGAG
TATGTTGGTGCTATATGGGAAAGTGGAACTACAAACTACAACCCAGCCCTGAAGTCCCGAGCCAGCATCACCAAGGA
CACCTCAAAGAGCCAAGTTTATCTGACGCTGAACAGCCTGACAGGCGAGGACACGGCCGTCTATTACTGTGCGCGCC
TATGGACAAACTACTGGGGCCAGGGCATC
>KC549766.1 Equus caballus clone 5'RACE Foal MLN T06 immunoglobulin heavy chain mRNA, partial cds
ATTTTCAGGGAGTCGTGAATCTCATCTGCAAGAACATGAGTCACCTGTGGTTCTTCCTCTTTCTGGTGGCCGCTCCT
ACATGTGTCCTGTCCCAGGTGCAACTGAAGGAGTCAGGACCTGGCCTGGTGAAGCCCTCGCAGACCCTCTCCCTCAC
CTGCACTGTCTCTGGATTCTCTTTGAGCAGTTTTGATGTAGGTTGGGTCCGCCAGGCTCCAGGAAAAGGGCTGGAAT
ATGTTGGTGCTATGGCTTATAGTGGAAGTGCAGACTACAACCCAGCCCTGAAGTCCCGAGCCAGCATCACCAGGGAC
ACCTCAAAGAGCCAAGTTTATCTGGCGCTAGACAGCCTGACAAGCGAGGACACGGCCGTCTATTATTGTGCAGCATT
GGTGGCAGTTCCTGATAACTGGTGGGGCCAGGGCATC
>KC549765.1 Equus caballus clone 5'RACE Foal MLN T08 immunoglobulin heavy chain mRNA, partial cds
ATTTTCAGGGAGTCGTGAATCTCATCTGCAAGAACATGAGTCACCTGTGGTTCTTCCTCTTTCTGGTGGCCGCTCCT
ACATGTGTCCTGTCCCAGGTGCAACTGAAGGAGTCAGGACCTGGCCTGGTGAAGCCCTCGCAGACCCTCTCCCTCAC
CTGCACTGTCTCTGGATTCTCTTTGAGCAGTTACGGTGTAGGCTGGGTCCGCCAGGCTCCAGGAAAAGGGCTGGAAT
TTGTTGGGGGTATAGTTAGTAGTGGAAGTGCAAACTACAACCCAGCCCTGAAGTCCCGAGCCAGCATCACCAAGGAC
ACCTCAAAGAGCCAAGTTTATCTGACGCTGAACAGCCTGACAGGAGAGGACACGGCCGCCTATTACTGTGCAGGAGT
TTATGACGATAGTAGGACTTACAATCCCTTTGGCTACTGGGGCCAGGGCACC
>KC549763.1 Equus caballus clone 5'RACE Foal MLN T20 immunoglobulin heavy chain mRNA, partial cds
TCTTTGAGCAGTAATGCTGTAGGCTGGGTCCGCCAGGCTCCAGGAAAAGGGCTGGAGTGGGTTGGTGTTATATATGG
TAGTGAAAGTACATACTACAACCCAGCCCTGAAGTCCCGAGCCAGCATCACCAAGGACACCTCAAAGAGCCAAGTTT
ATCTGACGCTGAACAGCCTGACAGGCGAAGACACGGCCGTCTATTACTGTGCAGGGATGGAAAATGATGACTATGGT
GATACTGCCTACTATACGTCGTTTTTTGGCTACTGGGGCCAGGGCACC
>KC549760.1 Equus caballus clone 5'RACE Foal MLN T19 immunoglobulin heavy chain mRNA, partial cds
ATTTTCAGGGAGTCGTGAATCTCATCTGCAAGAACATGAGTCACCTGTGGTTCTTCCTCTTTCTGGTGGCCGCTCCT
ACATGTGTCCTGTCCCAGGTGCAACTGAAGGAGTCAGGACCTGGCCTGGTGAAGCCCTCGCAGACCCTCTCCCTCAC
CTGCACTGTCTCTGGATTATCTTTGAGCAGTAATCTTGTAGGCTGGGTCCGCCAGGCTCCAGGAAAAGGGCTGGAAT
ATGTTGGTGGTATAGCTAGTAGTGGAAGTGCAAACTACAACCCAGCCCTGAAGTCCCGACCCAGCATCACCAAGGAC
ACCTCAAAGAGCCAAGTTTATCTGACGCTGAACAGCCTGACAAGCGAGGACACGGCCGTCTATTACTGTGCAGGAAG
GAGGGACTATAGCGGCTATGACAATGATATAAACTACTGGGGCCAGGGCATC
>KC549759.1 Equus caballus clone 5'RACE Foal MLN T32 immunoglobulin heavy chain mRNA, partial cds
ATTTTCAGGGAGTCGTGAATCTCATCTGCAAGAACATGAGTCACCTGTGGTTCTTCCTCTTTCTGGTGGCCGCTCCT
ACATGTGTCCTGTCCCAGGTGCAACTGAAGGAGTCAGGACCTGGCCTGGTGAAGCCCTCGCAGACCCTCTCCCTCAC
CTGCACTGTCTCTGGATTATCTTTGAGCAGTAATGCTGTAGGCTGGGTCCGCCAGGCTCCAGGAAAAAGGCTGGAAT
ATGTTGGTGGTATAGCTAGTAGTGGAAGTGCAAACTACAACCCAGCCCTGAAGTCCCGAGCCAGCATCACCAAGGAC
ACCTCAAAGAGCCAAGTTTATCTGACGCTGAACAGCCTGACAAGCGAGGACACGGCCGTCTATTACTGTGCAGGAGG
AGGGGACTACGGTTATGGTTATGAGCTGGGGGGTATAAACTACTGGGGCCAGGGCATC

>KC549758.1 Equus caballus clone 5'RACE Foal MLN T29 immunoglobulin heavy chain mRNA, partial cds
ATTTTCAGGGAGTCGTGAATCTCATCTGCAAGAACATGAGTCACCTGTGGTTCTTCCTCTTTCTGGTGGCCGCTCCT
ACATGTGTCCTGTCCCAGGTGCAACTGAAGGAGTCAGGACCTGGCCTGGTGAAGCCCTCGCAGACCCTCTCCCTCAC
CTGCACTGTCTCTGGATTATCTTTGAGCAGTAATGCTGTAGGCTGGGTCCGCCAGGCTCCAGGAAAAGGGCTGGAAT
ACGTTGGTGAAATAGTTGGTAGTGTAAGTGCAATGTACAATCCAGCCCTGAAGTCCCGAGCCAGCATCACCAAGGAC
ACCTCAAAGAGCCAAGTTTATCTGACGCTGAATAGTCTGACAAGCGAGGACACGGCCGTCTATTACTGTGCAGGAGG
CAGCTATGATTACGACGAATTTGGCTACTGGGGCCAGGGCACC
>KC549757.1 Equus caballus clone 5'RACE Foal MLN T28 immunoglobulin heavy chain mRNA, partial cds
ATTTTCAGGGAGTCGTGAATCTCATCTGCAAGAACATGAGTCACCTGTGGTTCTTCCTCTTTCTGGTGGCCGCTCCT
ACATGTGTCCTGTCCCAGGTGCAACTGAAGGAGTCAGGACCTGGCCTGGTGAAGCCCTCGCAGACCCTCTCCCTCAC
CTGCACTGTCTCTGGATTATCTTTGAGCAGTAATGCTGTAGGCTGGGTCCGCCAGGCTCCAGGAAAAGGGCTGGAAT
TTGTTGCTTATATACGCGGTAGTGCAAGTGCAAACTACAACCCAGCCCTGAAGTCCCGAGCCAGCATCACCAAGGAC
ACCTCAAAGAGCCAAGTTTATCTGACGCTGAACAGCCTGACAAGCGAGGACACGGCCGTCTATTACTGTGCAGGACG
AGAGAGAATAGAATATGAGCTTACTTTAGACGGTATAAACTACTGGGGCCAGGGCATC
>KC549756.1 Equus caballus clone 5'RACE Foal MLN T27 immunoglobulin heavy chain mRNA, partial cds
ATTTTCAGGGAGTCGTGAATCTCATCTGCAAGAACATGAGTCACCTGTGGTTCTTCCTCTTTCTGGTGGCCGCTCCT
ACATGTGTCCTGTCCCAGGTGCAACTGAAGGAGTCAGGACCTGGCCTGGTGAAGCCCTCGCAGACCCTCTCCCTCAC
CTGCACTGTCTCTGGATTATCTTTGAGCAGTAATGCTGTAGGCTGGGTCCGCCAGGCTCCAGGAAAAGGGCTGGAAT
TTGTTGGTGCTATAGCTGGTAGTACAAGCTACAACCCAGCCCTGAAGTCCCGAGCCAGCATCACCAAGGACACCTCA
AAGAGCCAAGTTTATCTGACGCTGAACAGCCTGACAAGCGAGGACACGGCCGTCTATTACTGTGCAGGAAAGATCCC
CGTAGCTGATATAGCATCTATGCTCCTGTTTGGCTACTGGGGCCAGGGCACC
>KC549755.1 Equus caballus clone 5'RACE Foal MLN T18 immunoglobulin heavy chain mRNA, partial cds
ATTTTCAGGGAGTCGTGAATCTCATCTGCAAGAACATGAGTCACCTGTGGTTCTTCCTCTTTCTGGTGGCCGCTCCT
ACATGTGTCCTGTCCCAGGTGCAACTGAAGGAGTCAGGACCTGGCCTGGTGAAGCCCTCGCAGACCCTCTCCCTCAC
CTGCACTGTCTCTGGATTATCTTTGAGCAGTAATGCTGTAGGCTGGGTCCGCCAGGCTCCAGGAAAAGGGCTGGAAT
ACGTTGGTAGAATATGGGGTAGTGCAAGTGAAAACTACAACCCAGCCCTGAAGTCCCGAGCCAGCATCACCAAGGAC
ACCTCAAAGAGCCAAGTTTATCTGACGCTGAACAGCCTGACAAGCGAGGACACGGCCGTCTATTTTTGTGTAGGATA
CGGTTTTGGTTATGCTCAGGATTACTGGGGCCAGGGCACC
>KC549754.1 Equus caballus clone 5'RACE Foal MLN T09 immunoglobulin heavy chain mRNA, partial cds
ATTTTCAGGGAGTCGTGAATCTCATCTGCAAGAACATGAGTCACCTGTGGTTCTTCCTCTTTCTGGTGGCCGCTCCT
ACATGTGTCCTGTCCCAGGTGCAACTGAAGGAGTCAGGACCTGGCCTGGTGAAGCCCTCGCAGACCCTCTCCCTCAC
CTGCACTGTCTCTGGATTATCTTTGAGCAGTAATGCTGTAGGCTGGGTCCGCCAGGCTCCAGGAAAAGGGCTGGAAT
ACGTTGGTGATATATATGGTAGTGCAAGTGCAAACTACAACCCAGCCCTGAAGTCCCGAGCCAGCATCACCAAGGAC
ACCTCAAAGAGCCAAGTTTATCTGACGCTGAACAGCCTGACAAGCGAGGACACGGCCGTCTATTACTGTGCAGGATG
TAGTTACTGGAGTAGGAGTTGCTATCTGGATGGTATAAACTACTGGGGCCAGGGCATC
>KC549753.1 Equus caballus clone 5'RACE Foal MLN T03 immunoglobulin heavy chain mRNA, partial cds
AGTCGTGAATCTCATCTGCAAGAACATGAGTCACCTGTGGTTCTTCCTCTTTCTGGTGGCCGCTCCTACATGTGTCC
TGTCCCAGGTGCAACTGAAGGAGTCAGGACCTGGCCTGGTGAAGCCCTCGCAGACCCTCTCCCTCACCTGCACTGTC
TCTGGATTATCTTTGAGCAGTAATGCTGTAGGCTGGGTCCGCCAGGCTCCAGGAAAAGGGCTGGAATTTGTTGGTGT
TATACATAGTGATGCAAGTGCAGACTACAACCCAACCCTGAAGTCCCGAGCCAGCATCACCAAGGACACCTCAAAGA
GCCAAGTTTATCTGACGCTGAACAGCCTGACAAGCGAGGACACGGCCGTCTATTACTGTGCAGGACTACGGTCATGG
TTATGCTACTCCTGGGGCCAGGGCACC
>KC549751.1 Equus caballus clone 5'RACE Foal MLN T11 immunoglobulin heavy chain mRNA, partial cds
ATTTTCAGGGAGTCGTGAATCTCATCTGCAAGAACATGAGTCACCTGTGGTTCTTCCTCTTTCTGGTGGCCGCTCCT
ACATGTGTCCTGTCCCAGGTGCAACTGAAGGAGTCAGGACCTGGCCTGGTGAAGCCCTCGCAGACCCTGTCCCTCAC
CTGCACTGTCTCTGGACTCTCTTTGAGCAGTTACGGTGTAGGCTGGGTCCGCCAGGCTCCAGGAAAAGGGCTGGAAT
ATGTTGGTGGTATAGATAGCAGTGGTATAGATAGCAGTGGAAGAGAATACTACAACCCAGCCCTGAAGTCCCGAGCC

AGCATCACCAAGGACACCTCAAAGAGCCAAGTTTATCTGACACTGAACAGCCTGACAGGCGAGGACACGGCCGTCTA
TTTTTGTGTCAAGGGGTTACAGGGTGAATTTGGCTACTGGGGCTGGGGCCAGGGCACC
>KC549750.1 Equus caballus clone 5'RACE Foal MLN T25 immunoglobulin heavy chain mRNA, partial cds
ATTTTCAGGGAGTCGTGAATCTCATCTGCAAGAACATGAGTCACCTGTGGTTCTTCCTCTTTCTGGTGGCCGCTCCT
ACATGTGTCCTGTCCCAGGTGCAACTGAAGGAGTCAGGACCTGGCCTGGTGAAGCCCTCGCAGACCCTCTCCCTCAC
CTGCACTGTCTCTGGATTCTCTTTGAGCACATATGCTGTAGGCTGGGTCCGCCAGGCTCCAGGAAAAGGGCTGGAAT
ATGTTGGTGGTATAGCTAGTAGTGGAAGTGCAAACTACAACCCAGCCCTGAAGTCCCGAGCCAGCATCACCAAGGAC
ACCTCAAAGAGCCAAGTTTATCTGACGCTGAACAGCCTGACAAGCGAGGACACGGCCGTCTATTACTGTTCAGGACC
AGATATTGCAGCTACTGTCGCTTACGGAACTGGTACTAACTACTGGGGCCAGGGCATC
>KC549749.1 Equus caballus clone 5'RACE Foal MLN T26 immunoglobulin heavy chain mRNA, partial cds
ATTTTCAGGGAGTCGTGAATCTCATCTGCAAGAACATGAGTCACCTGTGGTTCTTCCTCTTTCTGGTGGCCGCTCCT
ACATGTGTCCTGTCCCAGGTGCAACTGAAGGAGTCAGGACCTGGCCTGGTGAAGCCCTCGCAGACCCTCTCCCTCAC
CTGCACTGTCTCTGGATTCTCTTTGAGCAGTTACGGTGGTGTAGGCTGGGTCCGCCAGGCTCCAGGAAAAGGGCTGG
AATTTGTTGGTGCTATAGCTGGTAGTGGAAGTGCAAACTACAACCCAGCCCTGAAGTCCCGAGCCAGCATCACCAAG
GACACCTCAAAGAGTCAAGTTTATCTGACGCTGAACAGCCTGACAAGCGAGGACACGGCCGTCTATTACTGTGCGGG
GGGTGGTGCTGGTGGTGATTACTATTACTGGGGCCAGGGCATC
>KC549748.1 Equus caballus clone 5'RACE Foal MLN T07 immunoglobulin heavy chain mRNA, partial cds
ATTTTCAGGGAGTCGTGAATCTCATCTACAAGAACATGAGTCACCTGTGGTTCTTCCTCTTTCTGGTGGCCGCTCCT
ACATGTGTCCTGTCCCAGGTGCAACTGAAGGAGTCAGGACCTGGCCTGGTGAAGCCCTCGCAGACCCTGTCCCTCAC
CTGCACTGTCTCTGGATTCTCTTTGAGCAGTAACAATGTAGCCTGGGTCCGCCAGGCTCCAGGAAAAGGGCTGGAAT
TTGTTGGTGGTATAGGTAGTGAGGGAAGTACATACTACAACCCAGCCCTGAGGTCCCGAGCCAGCATCACCAAGGAC
ACCTCAAAGAGCCAAGTTTATCTGACGCTGAACAGCCTGACAGGCGAGGACACGGCCGTCTATTACTGTGCGAGATT
CGCTTGGGGATGGTTTGGCTACTGGGGCCAGGGCACC
>KC549745.1 Equus caballus clone 5'RACE Foal MLN T33 immunoglobulin heavy chain mRNA, partial cds
ATTTTCAGGGAGTCGTGAATCTCATCTGCAAGAACATGAGTCACCTGTGGTTCTTCCTCTTTCTGGTGGCCGCTCCT
ACATGTGTCCTGTCCCAGGTGCAACTGAAGGAGTCAGGACCTGGCCTGGTGAAGCCCTCGCAGACCCTCTCCCTCAC
CTGCACTGTCTCTGGATTCTCTTTGAGCAGTTACGGTGTAGGCTGGGTCCGCCAGGCTCCAGGAAAAGGCCTGGAAT
TTGTTGGTGGTATGGCTAGTGATGGAAGTGCGGTCTACAATCCAGCCCTGAAGTCCCGAGCCAGCATCACCAAGGAC
ACCTCAAAGAGCCAAGTTTATCTGACGCTGAACAGCCTGACAAGCGAGGACACGGCCGTCTATTACTGTGCAGGAGG
CCTTTCAAGTGACTACCGTTATGGTTATGTTTCAAACTACTGGGGCCAGGGCATC
>KC549744.1 Equus caballus clone 5'RACE Foal MLN T15 immunoglobulin heavy chain mRNA, partial cds
ATTTTTTCAGGGAGTCGTGAATCTCATCTGCAAGAACATGAGTCACCTGTGGTTCTTCCTCTTTCTGGTGGCCGCTC
CTACATGTGTCCTGTCCCAGGTGCAACTGAAGGAGTCAGGACCTGGCCTGGTGAAGCCCTCGCAGACCCTCTCCCTC
ACCTGCACTGTCTCTGGATTCTCTTTGAGCAGTTACGGTGTAGGCTGGGTCCGCCAGGCTCCAGGAAAAGGGCTGGA
ATTTGTTGGTGGTATAGCTAGTAGTGGAAGTGCAAACTACAACCCAGCCCTGAAGTCCCGAGCCAGCATCACCAAGG
ACACCTCAAAGAGCCAAGTTTATCTGACGCTGAACAGCCTGACAAGCGAGGACACGGCCGTCTATTACTGTGCAGGA
CAACCGGATGCTATAAAGTACTGGGGCCAGGGCATC
>KC549742.1 Equus caballus clone 5'RACE Foal MLN T22 immunoglobulin heavy chain mRNA, partial cds
ATTTTCAGGGAGTCGTGAATCTCATCTGCAAGAACATGAGTCACCTGTGGTTCTTCCTCTTTCTGGTGGCCGCTCCT
ACATGTGTCCTGTCCCAGGTGCAACTGAAGGAGTCAGGACCTGGCCTGGTGAAGCCCTCGCAGACCCTGTCCCTCAC
CTGCACTGTCTCTGGATTCTCTTTGAGCAGTTACGGTGTAGGCTGGGTCCGCCAGGCTCCAGGAAAAGGGCTGGAAT
ATGTTGGTGGTATAGCTAGTAGTGGAAGTGCAAACTACAACCCAGCCCTGAAGTCCCGAGCCAGCATCACCAAGGAC
ACCTCAAAGAGCCAAGTTTATCTGACGCTGAACAGCCTGACAGGCGAGGACACGGCCGTCTATTACTGTGCGAGTCA
CGCTACGGTTAATGTTATGCTACCTCATGGTATAAACTACTGGGGCCAGGGCATC
>KC549740.1 Equus caballus clone 5'RACE Neo MLN D03 immunoglobulin heavy chain mRNA, partial cds
ATTTTCAGGGAGTCGTGAATCTCATCTGCAAGAACATGAATCACCTGTGGTTCTTCCTCTTTCTGGTGGCCGCTCCT
ACATGTGTCCTGTCCCAGGTGCAACTGAAGGCGTCGGGACCTGGCCTGGTGAAGCCCTCGCAGACCCTCTCCCTTAC
CTGCACTGTCTCTGGATTCTCTTTGAGCAGTTGGCATGTATACTGGGTCCGCCAGGCTCCAGGAAAAGGGCTGGAAT
ATGTTGCTTCTATCGATGGTAGTGCAAGTGCAAACTACAACCCAGCCCTGAAGTCCCGAGCCAGCATCACCAGGGAC

ACCTCAAAGAGCCAAGTTTATCTGACGCTGAACAGCCTGACAGGCGAGGACACGGCCGTCTATTACTGTGCGAGTTC
TGACTATGGTACATATGATTTTGGTATAAACTACTGGGGCCAGGGCATC
>KC549739.1 Equus caballus clone 5'RACE Neo MLN C01 immunoglobulin heavy chain mRNA, partial cds
ATTTTCAGGGAGTCGTGAATCTCATCTGCAAGAACATGAATCACCTGTGGTTCTTCCTCTTTCTGGTGGCCGCTCCT
ACATGTGTCCTGTCCCAGGTGCAACTGAAGGAGTCGGGACCTGGCCTGGTGAAGCCCTCGCAGACCCTCTCCCTCAC
CTGCACTGTCTCTGGATTCTCTTTGAGCAGTTATGTTGTATACTGGGTCCGCCAGGCTCCAGGAAAAGGGCTGGAAT
ATGTTGGTTGTATATATGGTAGTGCAAGTGCAAACTACAACCCAGCCCTGAAGTCCCGAGCCAGCGTCACCAAGGAC
ACCTCAAAGAGCCAGGTTTATCTGACGCTGAACAGCCTGACAGGCGAGGACACGGCCGTCTATTACTGTGCGAGTCA
TCTAATCGGTTATGATACAGGTGGCTATTGGGGCCAGGGCACC
>KC549738.1 Equus caballus clone 5'RACE Neo MLN D02 immunoglobulin heavy chain mRNA, partial cds
ATTTTCAGGGAGTCGTGAATCTCATCTGCAAGAACATGAATCACCTGTGGTTCTTCCTCTTTCTGGTGGCCGCTCCT
ACATGTGTCCTGTCCCAGGTGCAACTGAAGGAGTCGGGACCTGGCCTGGTGAAGCCCTCGCAGACCCTCTCCCTCAC
CTGCACTGTCTCTGGATTCTCTTTGAGCAGTTATGCTGTATACTGGGTCCGCCAGGCTCCAGGAAAAGGGCTGGAAT
ATGTTGGTGATATAGATGATAGTGCAAGTGCAAAGTACAGCCCAGCCCTGAAGTCCCGAGCCAGCATCACCAAGGAC
ACCTCAAAGAGCCAAGTTTATCTGACGCTGAACAGCCTGACAGGCGAGGACACGGCCGTCTATTACTGTGCGAGATC
TAGGATTTACTATGGTGGTAGTTCCTGGTACTCCAACGAGTTGGATTACTGGGGCCAGGGCACC
>KC549737.1 Equus caballus clone 5'RACE Neo MLN B02 immunoglobulin heavy chain mRNA, partial cds
ATTTTCAGGGAGTCATGAATCTCATCTGCAAGAACATGAATCACCTGTGGTTCTTCCTCTTTCTGGTGGCCGCTCCT
AGATGTGTCCTGTCCCAGGTGCAACTGAAGGAGTCAGGACCTGGCCTGGTGAAGCCCTCGCAGACCCTCTCCCTCAC
CTGCACTGTCTCTGGATTCTCTTTGAGCACTTATGCTGTAGGCTGGGTCCGCCAGGCTCCAGGAAAAGGGCTGGAAT
ATGTTGGTGCTGGATGGGATAGTGCAAGTGCAAACTACAACCCAGCCCTGAAGTCCCGAGCCAGCATCACCAAGGAC
ACCTCCAAGAGCCAAGCTTATCTGACGCTGAACAGCCTGACAGGCGAGGACACGGCCGTCTATTACTGTGCGAAGCT
CACGTACTGGGACAACTATGATTACCAATATTATTATGATATAGACTACTGGGGCCAGGGCATC
>KC549734.1 Equus caballus clone 5'RACE Neo MLN B05 immunoglobulin heavy chain mRNA, partial cds
ATTTTCAGGGAGTCGTGAATCTCATCTGCAAGAACATGAATCACCTGTGGTTCTTCCTCTTTCTGGTGACCGCTCCT
ACATGTGTCCTGTCCCAGGTGCAACTGAAGGAGTCGGGACCTGGCCTGGTGAAGCCCTCGCAGACCCTGTCCCTCAC
CTGCACTGTCTCTGGATTATCTTTGAGCGATAATGCTGTAGGCTGGGTCCGCCAGGCTCCAGGAAAAGGGCTGGAGT
GGGTTGGTGTTTCATATGGTAGTGAAGATACATACTACAACCCAGCCCTGAAGTCCCGAGCCAGCATCACCAAGGAC
ACCTCAAAGAGCCAAGTTTATCTGACGCTTAACAGCCTGACAGGCGAAGACACGGCCGTCTATTACTGTGCAGGATA
TACCACTGCTAGTAGTGTCTATCCCGATTTCGGTATAGACTACTGGGGCCAGGGCATC
>KC549731.1 Equus caballus clone 5'RACE Neo MLN A09 immunoglobulin heavy chain mRNA, partial cds
ATTTTCAGGGAGTCGTGAATCTCATCTGCAAGAACATGAATCACCTGTGGTTCTTCCTCTTTCTGGTGGCCGCTCCT
ACATGTGTCCTGTCCCAGGTGCAACTGAAGGAGTCGGGACCTGGCCTGGTGAAGCCCTCGCAGACCCTGTCCCTCAC
CTGCACTGTCTCTGGATTATCTTTGAGCGTTAATAATGTAGGCTGGGTCCGCCAGGCTCCAGGAAAAGGGCTGGAGT
GGATTGGTAATAGCGATGGTAGTGAAGGTACATACTACAACCCAGCCCTGAAGTCCCGAGCCAGCATCACCAAGGAC
ACCTCAAAGAGCCAAGTTTATCTGACGCTGAACAGCCTGACAGGCGAAGACACGGCCGTCTATTACTGTGCAGGATC
ACAATACTACGTTTATGGTCATGTCCTCTATGACTTTGGCTACTGGGGCCAGGGCACC
>KC549730.1 Equus caballus clone 5'RACE Neo MLN A01 immunoglobulin heavy chain mRNA, partial cds
ATTTTCAGGGAGTCGTGAATCTCATCTGCAAGAACATGAATCACCTGTGGTTCTTCCTCTTTCTGGTGACCGCTCCT
ACATGTGTCCTGTCCCAGGTGCAACTGAAGGAGTCGGGACCTGGCCTGGTGAAGCCCTCGCAGACCCTGTCCCTCAT
CTGCACTGTCTCTGGATTATCTTTGAGGAGTAGGACTGTAGGCTGGGTCCGCCAGGCTCCAGGAAAAGGGCTGGAGT
GGGTTGGTTCTATAGATGGCGGTGAAAGTACAGCCTACAATCCAGCCCTGAAGTCCCGAGCCAGCATCACCAAGGAC
ACCTCAAAGAGCCAGATTTACCTGACGCTGAACAGCCTGACAGGCGAAGACACGGCCGTCTATTACTGTGCAGGATA
TGATGGCTATGCTGGTGGTTATCACTGGGGCCAGGGCACC
>KC549729.1 Equus caballus clone 5'RACE Neo MLN D01 immunoglobulin heavy chain mRNA, partial cds
GAAGGAGTCGGGCCCTGGCCTGGTGAAGCCCTCGCAGACCCTGTCCCTCACCTGCACTGTCTCTGGATTATCTTTGA
GCCGTAATGATGTAGGCTGGGTCCGCCAGGCTCCAGGAAAAGGGCTGGAGTGGGTTGGTGTTATACATAGAAGTGAA
AGTACACTGTACAACCCAGCCCTGAAGTCCCGAGCCAGCATCACCAAGGACACCTCAAAGAGCCAAGTTTATCTGAC

GCTGAACAGCCTGACAGGCGAAGACACGGCCGTCTATTACTGTGCAGGATGGGATGCATTTGGTTATGCTGGTGTAA
ACTACTGGGGCCAGGGCATC
>KC549728.1 Equus caballus clone 5'RACE Neo MLN C03 immunoglobulin heavy chain mRNA, partial cds
ATTTTCAGGGAGTCGTGAATCTCATCTGCAAGAACATGAATCACCTGTGGTTCTTCCTCTTTCTGGTGGCCGCTCCT
ACATGTGTCCTGTCCCAGGTGCAACTGAAGGAGTCGGGACCTGGCCTGGTGAAGCCCTCGCAGACCCTGTCCCTCAC
CTGCACTGTCTCTGGATTATCTTTGAGCAGTAATGCTGTAGGCTGGGTCCGCCAGGCTCCAGGAAAAGGGCTGGAGT
GGGTTGGTGTTATATATGGTAGTGAAAGTACATACTACAACCCAGCCCTGAAGTCCCGAGCCAGCATCACCAAGGAC
ACCTCAAAGAGCCAAGTTTATCTGACGCTGAACAGCCTGACAGGCGAAGACACGGCCGTCTATTACTGTGCAGGATG
TATGACTGTACTGGTCATGGATGTGTCTACATATTATGGTATAAACTACTGGGGCCAGGGCATC
>KC549727.1 Equus caballus clone 5'RACE Neo MLN B08 immunoglobulin heavy chain mRNA, partial cds
ATTTTCAGGGAGTCGTGAATCTCATCTGCAAGAACATGAATCACCTGTGGTTCTTCCTCTTTCTGGTGACCGCTCCT
ACATGTGTCCTGTCCCAGGTGCAAGTGAAGGAGTCGGGACCTGGCCTGGTGAAGCCCTCGCAGACCCTGTCCCTCAC
CTGCACTGTCTCTGGATTATCTTTGAGCAGTAATGCTGTAGGCTGGGTCCGCCAGGCTCCAGGAAAAGGGCTGGAGT
GGGTTGGTGTTATATATGGTGGTGAAAGTACATACTACAACCCAGCCCTGAAGTCCCGAGCCAGCATCACCAAGGAC
ACCTCAAAGAGCCAAGTTTATCTGACGCTGAACAGCCTGACAGGCGAAGACACGGCCGTCTATTACTGTGTAGGATA
CGACGTCGGTTATGGTCACTGGGGCCAGGGCACC
>KC549725.1 Equus caballus clone 5'RACE Neo MLN C12 immunoglobulin heavy chain mRNA, partial cds
ATTTTCAGGGAGTCGTGAATCTCATCTGCAAGAACATGAATCACCTGTGGTTCTTCCTCTTTCTGGTGACCGCTCCT
ACATGTGTCCTGTCCCAGGTGCAACTGAAGGAGTCGGGACCTGGCCTGGTGAAGCCCTCGCAGACCCTGTCCCTCAC
CTGCACTGTCTCTGGATTATCTTTGAGCAGTTATGGTGTGGGCTGGGTCCGCCAGGCTCCAGGAAAAGGGCTGGAAT
TTGTTGGTGATGAAGCTAGTAGTGGAAGTGCAAACTACAACCCAGCCCTGAAGTCCCGAGCCAGCATCACCAAGGAC
ACCGCAAAGAGCCAAGTTTATCTGACGCTGAACAGCCTGACAAGCGAGGACACGGCCGTCTATTACTGTACGAGTAC
TATAATAAGAGTTGTCATCCATTGGGGCCAGGGCATC
>KC549724.1 Equus caballus clone 5'RACE Neo MLN B10 immunoglobulin heavy chain mRNA, partial cds
ATTTTCAGGGAGTCGTGAATCTCATCTGCAAGAACATGAGTCACCTGTGGTTCTTCCTCTTTCTGGTGGCCGCTCCT
ACATGTGTCCTGTCCCAGGTGCAACTGAAGGAGTCAGGACCTGGCCTGGTGAAGCCCTCGCAGACCCTCTCCCTCAC
CTGCACTGTCTCTGGATTATCTTTGAGCAGTTATGGTGTGGGCTGGGTCCGCCAGGCTCCAGGAAAAGGGCTGGAGT
TTGTTGGTGGTATAGCTAGAAGTGGAAGTGCAAACTACAACCCAGCCCTGAAGTCCCGAGCCAGCATCACCAAGGAC
ACCTCAAAGAGCCAAGTTTATCTGACGCTGAACAGCCTGACAAGCGAGGACACGGCCGTCTATTACTGTGCAGGAGG
CCTGAATTATTATGGTGCTATTGACTATGGTATAAACTACTGGGGCCAGGGCATC
>KC549723.1 Equus caballus clone 5'RACE Neo MLN A12 immunoglobulin heavy chain mRNA, partial cds
ATTTTCAGGGAGTCGTGAATCTCATCTGCAAGAACATGAGTCACCTGTGGTTCTTCCTCTTTCTGGTGGCCGCTCCT
ACATGTGTCCTGTCCCAGGTGCAACTGAAGGAGTCAGGACCTGGCCTGGTGAAGCCCTCGCAGACCCTCTCCCTCAC
CTGCACTGTCTCTGGATTATCTTTGAGCAGTTATGGTGTGGGCTGGGTCCGCCAGGCTCCAGGAAAAGGGCTGGAAT
TTGTTGGTGGTATAGCTAGTAGTGGAAGTGCAAACTACAACCCAGCCCTGAAGTCCCGAGCCAGCATCACCAAGGAC
ACCTCAAAGAGCCAAGTTTATCTGACGCTGAACAGCCTGACAAGCGAGGACACGGCCGTCTATTACTGTGCAGGAGA
GCCGTGGTTGGAAAGGAGATATGGTTATGGTGGTGCTTACTACGGACGAAATTATGGTATAAACTACTGGGGCCAGG
GCATC
>KC549722.1 Equus caballus clone 5'RACE Neo MLN C10 immunoglobulin heavy chain mRNA, partial cds
AGTCACCTGTGGTTCTTCCTCTTTCTGGTGGCCGCTCCTACATGTGTCCTGTCCCAGGTGCAACTGAAGGAGTCAGG
ACCTGGCCTGGTGAAGCCCTCGCAGACCCTCTCCCTCACCTGCACTGTCTCTGGATTATCTTTGAACAGTAATGAAG
TAGGCTGGGTCCGCCAGGCTCCAGGAAAAGGGCTGGAATATGTTGGTGGTATAGCTATTAGTGGAAGTGCAAACTAC
AACCCAGCCCTGAAGTCCCGAGCCAGCATCACCAAGGACACCTCAAAGAGCCAAGTTTATCTGACGCTGAACAGCCT
GACAAGCGAGGACACGGCCGTCTATTACTGTGCAGGGCCGGGAATCTACGGTAGAAGTTATGCTACGAGGTTTACAT
ACTGGGGCCAGGGCACC
>KC549721.1 Equus caballus clone 5'RACE Neo MLN A07 immunoglobulin heavy chain mRNA, partial cds
ATTTTCAGGGAGTCGTGAATCTCATCTGCAAGAACATGAGTCACCTGTGGTTCTTCCTCTTTCTGGTGGCCGCTCCT
ACATGTGTCCTGTCCCAGGTGCAACTGAAGGAGTCAGGACCTGGCCTGGTGAAGCCCTCGCAGACCCTCTCCCTCAC
CTGCACTGTCTCTGGATTATCTTTGAGCACAAATTCTGTAGGCTGGGTCCGCCAGGCTCCAGGAAAAGGGCTGGAAT

ACGTTGGTCTTATATATGGTAGTGCAAGTGCAAACTACAACCCAGCCCTGAAGTCCCGAGCCAGCATCACCAAGGAC
ACCTCAAAGAGCCAAGTTTATCTGACGCTGAACAGCCTGACAAGCGAGGACACGGCCGTCTATTACTGTGCAGGATA
TGGTAGTTATGATGAGGTCTACTGGGGCCAGGGCACC
>KC549720.1 Equus caballus clone 5'RACE Neo MLN B04 immunoglobulin heavy
chain mRNA, partial cds
ATTTTCAGGGAGTCGTGAATCTCATCTGCAAGAACATGAGTCACCTGTGGTTCTTCCTCTTTCTGGTGGCCGCTCCT
ACATGTGTCCTGTCCCAGGTGCAACTGAAGGAGTCAGGACCTGGCCTGGTGAAGCCCTCGCAGACCCTCTCCCTCAC
CTGCACTGTCTCTGGATTATCTTTGAGCAGTAATTTTGTAGGCTGGGTCCGCCAGGCTCCAGGAAAAGGGCTGAAAT
ATGTTGGTGGTATAACTAGTAGTGGAAGTGCAAGGTACAACCCAGCCCTGAAGTCCCGAGCCAGCATCACCAAGGAC
ACCTCAAAGAGCCAAGTTTATCTGACGCTAAACAGCCTGACAAGCGAGGACACGGCCGTCTATTACTGTAGAGGAGC
CGCGGATGGTGTATACTACTGGGGCCAGGGCATC
>KC549719.1 Equus caballus clone 5'RACE Neo MLN A06 immunoglobulin heavy
chain mRNA, partial cds
ATTTTCAGGGAGTCGTGAATCTCATCTGCAAGAACATGAGTCACCTGTGGTTCTTCCTCTTTCTGGTGGCCGCTCCT
ACATGTGTCCTGTCCCAGGTGCAACTGAAGGAGTCAGGACCTGGCCCGGTGAAGCCCTCGCAGACCCTCTCCCTCAC
CTGCACTGTCTCTGGATTATCTTTGAGCAGTAATACTGTAGGCTGGGTCCGCCAGGCTCCAGGAAAAGGGCTGGAAT
ACGTTGGTGCTATAGATTTCAGTGCAAGTGCAGACTACAACCCAGCCCTGAAGTCCCGAGCCAGCATCACCAAGGAC
ACCTCAAAGAGCCAAGTTTATCTGACGCTGAACAGCCTGACAAGCGAGGACACGGCCGTCTATCACTGTGCAGGAGG
CTCCCATGTTGGTATTTACTATGCGGGTGGTATAGAATACTGGGGCCAGGGCATC
>KC549718.1 Equus caballus clone 5'RACE Neo MLN B06 immunoglobulin heavy
chain mRNA, partial cds
ATTTTCAGGGAGTCGTGAATCTCATCTGCAAGAACATGAGTCACCTGTGGTTCTTCCTCTTTCTGGTGGCCGCTCCT
ACATGTGTCCTGTCCCAGGTGCAACTGAAGGAGTCAGGACCTGGCCTGGTGAAGCCCTCGCAGACCCTCTCCCTCAC
CTGCACTGTCTCTGGATTATCTTTGAGCAGTAATGCTGTAGGCTGGGTCCGCCAGGCTCCAGGAAAAGGGCTGGAAT
ACGTTGGTGACATAGATGGCAGTAGAAGTGCAAACTACAACCCAGCCCTGAAGTCCCGAGCCAGCATCACCAAGGAC
ACCTCAAAGAGCCAAGTTTATCTGACGCTGAACAGCCTGACAAGCGAGGACACGGCCGTCTATTACTGTGCAGGAGT
ACTGGGGGGTTCCTATGGTGGTAGTTACCACTGGGGCCAGGGCACC
>KC549717.1 Equus caballus clone 5'RACE Neo MLN A08 immunoglobulin heavy
chain mRNA, partial cds
ATTTTCAGGGAGTCGTGAATCTCATCTGCAAGAACATGAGTCACCTGTGGTTCTTCCTCTTTCTGGTGGCCGCTCCT
ACATGTGTCCTGTCCCAGGTGCAACTGAAGGAGTCAGGACCTGGCCTGGTGAAGCCCTCGCAGACCCTCTCCCTCAC
CTGCACTGTCTCTGGATTATCTTTGAGCAGTAATGCTGTAGGCTGGGTCCGCCAGGCTCCAGGAAAAGGGCTGGAAT
ATGTTGGTGGTATTGGTAGTAGTGGAAGTGCAAACTGCGACCCAGCCCTGAAGTCCCGAGCCAGCATCACCAAGGAC
ACCTCAAAGAGCCAAGTTTATCTGACGCTGAACAGCCTGACAAGCGAGGACACGGCCGTCTATTACTGTGCAGGAGG
CGGATGGGATATTATACTAGATGTGCCCCGGGATTTTACCTACTGGGGCCAGGGCACC
>KC549716.1 Equus caballus clone 5'RACE Neo MLN A05 immunoglobulin heavy
chain mRNA, partial cds
ATTTTCAGGGAGTCGTGAATCTCATCTGCAAGAACATGAGTCACCTGTGGTTCTTCCTCTTTCTGGTGGCCGCTCCT
ACATGTGTCCTGTCCCAGGTGCAACTGAAGGAGTCAGGACCTGGCCTGGTGAAGCCCTCGCAGACCCTCTCCCTCAC
CTGCACTGTCTCTGGATTATCTTTGAGCAGTAATGCTGTAGGCTGGGTCCGCCAGGCTCCAGGAAAAGGGCTGGAAT
ATGTTGGTGGTATACGTAGTAGTGGAAGTGCAGACTACAACCCAGCCCTGAAGTCCCGAGCCAGCATCACCAAGGAC
ACCTCAAAGAGCCAAGTTTATCTGACGCTGAACAGCCTGACAAGCGAGGACACGGCCGTCTATTACTGTGCAGGAGG
GATTGACTATGAAGATACTTTCTACGATGGTATACAGTACTGGGGCCAGGGCATC
>KC549715.1 Equus caballus clone 5'RACE Neo MLN C08 immunoglobulin heavy
chain mRNA, partial cds
ATTTTCAGGGAGTCGTGAATCTCATCTGCAAGAACATGAGTCACCTGTGGTTCTTCCTCTTTCTGGTGGCCGCTCCT
ACATGTGTCCTGTCCCAGGTGCAACTGAAGGAGTCAGGACCTGGCCTGGTGAAGCCCTCGCAGACCCTCTCCCTCAC
CTGCACTGTCTCTGGATTATCTTTGAGCAGTAATGCTGTAGGCTGGGTCCGCCAGGCTCCAGGAAAAGGGCTGGAAT
TTGTTGGTGCTATATATGGTAGTGCAAGTGCAAACTACAACCCAGCCCTGAAGTCCCGAGCCAGCATCACCAAGGAC
ACCTCAAAGAGCCAAGTTTATCTGACGCTGAACAGCCTGACAAGCGAGGACACGGCCGTCTATTACTGTGCAGGAGG
CTTAGACAGTTACTATGGTGGTAGTTCCTGGTGGAGGAGTTATGGTATAAACTACTGGGGCCAGGGCATC
>KC549714.1 Equus caballus clone 5'RACE Neo MLN C04 immunoglobulin heavy
chain mRNA, partial cds
ATTTTCAGGGAGTCGTGAATCTCATCTGCAAGAACATGAATCACCTGTGGTTCTTCCTCTTTCTGGTGGCCGCTCCT
ACATGTGTCCTGTCCCAGGTGCAACTGAAGGAGTCGGGACCTGGCCTGGTGAAGCCCTCGCAGACCCTCTCCCTCAC

CTGCACTGTCTCTGGATTCTCTTTGAGCAGTTATACTGTATACTGGGTCCGCCAGGCTCCAGGAAAAGGGCTGGAAT
ATGTTGGTGGTATATATGGTAGTGCAAGTGCAAACTACAACCCAGCCCTGAAGTCCCGAGCCAGCATCACCAAGGAC
ACCTCAAAGAGCCAAGTTTATCTGACACTGAACAGCCTGACAGGCGAGGACACGGCCGTCTATTACTGTGCGAGACC
TTCCTATGTAGGTGGTGAGTTATTGTACGGTATAAACTACTGGGGCCAGGGCATC
>KC549713.1 Equus caballus clone 5'RACE Neo MLN C02 immunoglobulin heavy chain mRNA, partial cds
ATTTTCAGGGAGTCGTGAATCTCATCTGCAAGAACATGAGTCACCTGTGGTTCTTCCTCTTTCTGGTGGCCGCTCCT
ACATGTGTCCTGTCCCAGGTGCAACTGAAGGAGTCAGGACCTGGCCTGGTGAAGCCCTCGCAGACCCTGTCCCTCAC
CTGCACTGTCTCTGGATTCTCTTTGAGCAGTTACAGTGTAGACTGGGTCCGCCAGGCTCCAGGAAAAGGGCTGGAAT
TTGTTGGTGGTATAGCTGATAGTGGAAGTGGATACTACAACCCAGCCCTGAAGTCCCGAGCCAGCATCACCAAGGAC
ACCTCAAAGAGCCAAGTTTATCTGACGCTGAACAGCCTGACAGGCGAGGACACGGCCGTCTATTACTGTGCGAGTCT
AAGCTATTTTGGCTACTGGGGCCAGGGCACC
>KC549711.1 Equus caballus clone 5'RACE Neo MLN A04 immunoglobulin heavy chain mRNA, partial cds
ATTTTTTCAGGGAGTCGTGAATCTCATCTGCAAGAACATGAGTCACCTGTGGTTCTTCCTCTTTCTGGTGGCCGCTC
CTACATGTGTCCTGTCCCAGGTGCAACTGAAGGAGTCAGGACCTGGCCTGGTGAAGCCCTCGCAGACCCTCTCCCTC
ACCTGCACTGTCTCTGGATTCTCTTTGAGCAGTTATGGTGTGGGCTGGGTCCGCCAGGCTCCAGGAAAAGGGCTGGA
ATTTGTTGGTGGTATACCTAGTAGTGGAAGTGCAAACTACAACCCAGCCCTGAAGTCCCGAGCCAGCATCACCAAGG
ACACCTCAAAGAGCCAAGTTTATCTGACACTGAACAGCCTGACAATGGAAGACACGGCCGTCTATTACTGTGCAGGA
GGCGGCGGTTATGATGACGCTTGGTATCCGTCGGGAGGCCAATGGGGCCAGGGCACC
>KC549710.1 Equus caballus clone 5'RACE Eq F Spl C11 immunoglobulin heavy chain mRNA, partial cds
ACAGCTCTCTCCTAAGATGAGGCTGAGGTCTCTGGGGAAGGCAGAGTGTAGTCTAGAAGAAGATGAGGCTGTTGGGT
CTTCTCCTTTGCCTGGTGACGGCTCCCCAAGGTGTCCTGTCCCAGGTGCAGCTGAAGGAGTCGGGCCCAGGACAGGT
GAAGCCCTCACAGACCCTCTCCCTCACCTGCACTGTCACTGGAGGCTCCATCACAAGCAGGTATTATGGCTGGAGCT
GGATCCGCCAGACCCCAGGGAAGGGGCTGGAGTACATTGGGAGCATCACTTATAGTGGTAGCACTTACTACAGCCCA
TCCTTCAAGAGCCGCGCCTCCATCTCCAGAGACACGCCCAAGAACCAGTTCTCCCTGCAGCTGAGCTCCGTGACCAC
AGAGGACACGGCCGTTTATTACTGTGCAAGTGATGACTATGGTGATACTTTCTACTATACTGGTATAAACTACTGGG
GCCAGGGCATCC
>KC549709.1 Equus caballus clone 5'RACE Eq F Spl-04 immunoglobulin heavy chain mRNA, partial cds
ACAGCTCTCTCCTAAGACGAGGCTGAGGTCTCTGGGGAAGGCAGAGTGTAGTCTAGAAGAAGATGAGGCTGTTGGGT
CTTCTCCTTTGCCTGGTGACGGCTCCCCAAGGTGTCCTGTCCCAGGTGCAGCTGAAGGAGTCGGGCCCAGGACAGGT
GAAGCCCTCACAGACCCTCTCCCTCACCTGCACTGTCACTGGAGGCTCCATCACAAGCAGGTATTATGGCTGGAGCT
GGATCCGCCAGACCCCAGGGAAGGGGCTGGAGTACATTGGGAGCATAGCTTATAGTGGTAGCACTTACTACAGCCCA
TCCCTCAAGAGCCACGCCTCCATCTCCAGAGACACGTCCAAGAACCAGTTCTCCCTGCAGCTGAGCTCCGTGACCAC
CGAGGACACGGCCGTTTATTACTGTGCAAGTTTGGGGGGTTATGGTTATGCCGAGAATGCTATGGACCCCTGGGGCC
AGGGCACCC
>KC549708.1 Equus caballus clone 5'RACE Eq F Spl B10 immunoglobulin heavy chain mRNA, partial cds
ACAGCTCTCTCCTAAGATGAGGCTGAGGTCTCTGGGGAAGGCAGAGTGTAGTCTAGAAGAAGATGAGGCTGTTGGGT
CTTCTCCTTTGCCTGGTGACGGCTCCCCAAGGTGTCCTGTCCCAGGTGCAGCTGAAGGAGTCGGGCCCAGGACAGGT
GAAGCCCTCACAGACCCTCTCCCTCACCTGCACTGTCACTGGAGGCTCCATCACAAGCAGGTATTATGGCTGGAGCT
GGATCCGCCAGACCCCAGGGAAGGGGCTGGAGTACATTGGGAGCATAGCTTATAGTGGTAGCACTTACTACAGCCCA
TCCCTCAAGAGCCGCGCCTCCATCTCCAGAGACACGTCCAAGAACCAGTTCTCCCTGCAGCTGAGCTCCGTGACCAC
CGAGGACACGGCCGTTTATTACTGTGCAAGTGTCCTGAGGAGTTGCTATGGCTACTGGGGCCAGGGCACCC
>KC549707.1 Equus caballus clone 5'RACE Eq F Spl A08 immunoglobulin heavy chain mRNA, partial cds
ATCACCCAAACATCACACTCCCTCCCCTACAGAAGCCCTGAGACCACAGTGCCTCACAATGGGCTGGAGCTGGAGAA
TCCTCTTCTTGGTGGCAGTAGCTTCAGGTGTCTCCTCCGAGGGTCAGCTGGAACAGTCGGGGCCGGAGTTGAAGAAG
CCTGGGTCATCAGTGAAGATCTCCTGCAAGGCTTCTGGATACACCTTCAGTAGCTATGCTGTGCACTGGGTGCGACA
GGCCAATGGAAAAGGGATTGAGTGGATGGGATCTATCTATGCTGAATATGATGATACAAGCTACGCACCGAAGTTCC
AGGGCAGAGTCACCATGACTGCGGACAAGTCCACGAGCACAGTCTACATGGAGCTGAGCAGTCTGACATCTGAGGAC
ATGGCCGTGTATTACTGTGCAACGGTTATGGTTATGCTACGTGCTATGGACCCCTGGGGCCAGGGCACCC
>KC549706.1 Equus caballus clone 5'RACE Eq F Spl-09 immunoglobulin heavy chain mRNA, partial cds

AGTTGTCTCCTCAGACAGGGCTGAGCTCTCTGGGGAAGGCAGAGTGTAATCTAGAAGAAGATGAGACTCTTGTGTCT
TCTCCTTTTCCTGGTGACGGCTCCCCAAGGAGTCCTGTCCCAGGTGCAGCTGCAGGAGTCGGGCCCAGGACTGGTGC
AGCCCTCACAGACCCTGTCCCTCACCTGCACTGTCACTGGAGGCTCCATCACAAGCAGCTATTCTAGCTGGAGCTGG
TTACGCCAGCCTCCAGGGAAGGGGCTGGAGTACATGGGGTACATATATTATGATGGTAGAACTTACTACAATCCTTC
CTTCAAGAGCCGCACCTCCATCTCCAGAGACACCTCCAAGAACCAGTTCTCCCTGCAGCTGAGCTCCGTGACCACCG
AGGACGCGGCCGTGTATTACTGTGCAAGAAGGAATAACTACGGTTATGGTTATGCTAATGCTATGGACCCCTGGGGC
CAGGGCACCC
>KC549705.1 Equus caballus clone 5'RACE Eq F Spl-10 immunoglobulin heavy chain mRNA, partial cds
AGTTGTCTCCTCAGACAGGGCTGAGCTCTCTGGGGAAGGCAGAGTGTAATCTAGAAGAAGATGAGACTCTTGTGTCT
TCTCCTTTTCCTGGTGACGGCTCCCCAAGGAGTCCTGTCCCAGGTGCAGCTGCAGGAGTCGGGCCCAGGACTGGTGC
AGCCCTCACAGACCCTGTCCCTCACCTGCACTGTCACTGGAGGCTCCATCACAAGCAGCTATTCTAGCTGGAGCTGG
TTACGCCAGCCTCCAGGGAAGGGGCTGGAGTACATGGGGTACATATATTATGATGGTAGAACTTACTACAATCCTTC
CTTCAAGAGCCGCACCTCCATCTCCAGAGACACCTCCAAGAACCAGTTCTCCCTGCAGCTGAGCTCCGTGACCACCG
AGGACGCGGCCGTGTATTACTGTGCAAGAGAATACTATGGTTATGGTGGTGCTTACTATTATGGTATAAACTACTGG
GGCCAGGGCATCC
>KC549700.1 Equus caballus clone 5'RACE Eq F Spl-03 immunoglobulin heavy chain mRNA, partial cds
ATTTTCAGGGAGTCGTGAATCTCATCTGCAAGAACATGAATCACCTGTGGTTCTTCCTCTTTCTGGTGGCCGCTCCT
ACATGTGTCCTGTCCCAGGTGCAACTGAAGGAGTCAGGACCTGGCCTGGTGAAGCCCTCGCAGACCCTCTCCCTCAC
CTGCACTGTCTCTGGATTCTCTTTGAGCAGTTATGCTGTAGGCTGGGTCCGCCAGGCTCCAGGAAAAGGGCTGGAAT
ATGTTGGTGCTATATATGGTAGTGCAAGTGCAAACTACAACCCAGCCCTGAAGTCCCGAGCCAGCATCACCAAGGAC
ACCTCAAAGAGCCAAGTTTATCTGACGCTGAACAGCCTGACAGGCGAGGACACGGCCGTCTATTACTGTGCGAGGAG
GAGGACTACTGGTATAAACTACTGGGGCCAGGGCATCC
>KC549699.1 Equus caballus clone 5'RACE Eq F Spl-08 immunoglobulin heavy chain mRNA, partial cds
ATTTTCAGGGAGTCGTGAATCTCATCTGCAAGAACATGAATCACCTGTGGTTCTTCCTCTTTCTGGTGGCCGCTCCT
ACATGTGTCCTGTCCCAGGTGCAACTGAAGGAGTCGGGACCTGGCCTGGTGAAGCCCTCGCAGACCCTGTCCCTCAC
CTGCACTGTCTCTGGATTATCTTTGAGCAGTAATGATGTAGGATGGGTCCGCCAGGCTCCAGGAAAAGGGCTGGAGT
GGGTTGGTGTTATATATGGTAGTGAAAGTACATACTACAACCCAGCCCTGAAGTCCCGAGCCAGCATCACCAAGGAC
ACCTCAAAGAGCCAAGTTTATCCGACGCTGAACAGCCTGACAGGCGAAGACACGGCCGTCTATTACTGTGCAGGAAC
TACGGTTATGGTTATGCTTGGCTACTGGGGCCAGGGCACC
>KC549698.1 Equus caballus clone 5'RACE Eq F Spl C05 immunoglobulin heavy chain mRNA, partial cds
ATTTTCAGGGAGTCGTGAATCTCATCTGCAAGAACATGAATCACCTGTGGTTCTTCCTCTTTCTGGTGGCCGCTCCT
ACATGTGTCCTGTCCCAGGTGCAACTGAAGGAGTCGGGACCTGGCCTGGTGAAGCCCTCGCAGACCCTGTCCCTCAC
CTGCACTGTCTCTGGATTATCTTTGAGCAGTAATGCTGTAGGCTGGGTCCGCCAGGCTCCAGGAAAAGGGCTGGAGT
GGGTTGGTGTTATATATGGTAGTGAAAGTACATACTACAACCCAGCCCTGAAGTCCCGAGCCAGCATCACCAAGGAC
ACCTCCAAGAGCCAAGTTTATCTGACGCTGAACAGCCTGACAGGCGAGGACACGGCCGTCTATTACTGTGCGTACGG
TTCCTATGCTGGTAGTTACTTATACTACTGGGGCCAGGGCACC
>KC549696.1 Equus caballus clone 5'RACE Eq F Spl C04 immunoglobulin heavy chain mRNA, partial cds
ATTTTCAGGGAGTCGTGAATCTCATCTGCAAGAACATGAATCACCTGTGGTTCTTCCTCTTTCTGGTGGCCGCTCCT
ACATGTGTCCTGTCCCAGGTGCAACTGAAGGAGTCGGGACCTGGCCTGGTGAAGCCCTCGCAGACCCTGTCCCTCAC
CTGCACTGTCTCTGGATTATCTTTGAGCAGTAATGCTGTAGGCTGGGTCCGCCAGGCTCCAGGAAAAGGGCTGGAGT
GGGTTGGTGTTATATATGGTAGTGAAAGTACATACTACAACCCAGCCCTGAAGTCCCGAGCCAGCATCACCAAGGAC
ACCTCAAAGAGCCAAGTTTATCTGACGCTGAACAGCCTGACAGGCGAAGACACGGCCGTCTATTACTGTGCAGGATC
CGGTTTTGGCTACTGGGGCCAGGGCACCC
>KC549695.1 Equus caballus clone 5'RACE Eq F Spl A03 immunoglobulin heavy chain mRNA, partial cds
ATTTTCAGGGAGTCGTGAATCTCATCTGCAAGAACATGAATCACCTGTGGTTCTTCCTCTTTCTGGTGGCCCAGGTG
CAACTGAAGGAGTCGGGACCTGGCCTGGTGAAGCCCTCGCAGACCCTGTCCCTCACCTGCACTGTCTCTGGATTATC
TTTGAGCAGTAATGCTGTAGGCTGGGTCCGCCAGGCTCCAGGAAAAGGGCTGGAGTGGGTTGGTGTTATATATGGTA
GTGAAAGTACATACTACAACCCAGCCCTGAAGTCCCGAGCCAGCATCACCAAGGACACCTCAAAGAGCCAAGTTTAT

CTGACGCTGAACAGCCTGACAGGCGAAGACACGGCCGTCTATTACTGTGCAGGATGTATGACTGTACTGGTCATGGA
TGTGTCTACATATTATGGTATAAACTACTGGGGCCAGGGCATCC
>KC549693.1 Equus caballus clone 5'RACE Eq F Spl C07 immunoglobulin heavy chain mRNA, partial cds
ATTTTCAGGGAGTCGTGAATCTCATCTGCAAGAACATGAATCACCTGTGGTTCTTCCTCTTTCTGGTGGCCGCTCCT
ACATGTGTCCTGTCCCAGGTGCAACTGAAGGAGTCGGGACCTGGCCTGGTGAAGCCCTCGCAGACCCTGTCCCTCAC
CTGCACTGTCTCTGGATTATCTTTGAGCAGTAATGCTGTAGGCTGGGTCCGCCAGGCTCCAGGAAAAGGGCTGGAGT
GGGTTGGTGTTATATATGGTAGTGAAAGTACATACTACAACCCAGCCCTGAAGTCCCGAGCCAGCATCACCAAGGAC
ACCTCAAAGAGCCAAGTTTATCTGACGCTGAACAGCCTGACAGGCGAAGACACGGCCGTCTATTACTGTGCAGGGTA
TGGTTATGGTGGTGCTTACTACGGGTCCTATGGTATAAACTACTGGGGCCAGGGCATCC
>KC549690.1 Equus caballus clone 5'RACE Eq F Spl B04 immunoglobulin heavy chain mRNA, partial cds
ATTTTCAGGGAGTCGTGAATCTCATCTGCAAGAACATGAGTCACCTGTGGTTCTTCCTCTTTCTGGTGGCCGCTCCT
ACATGTGTCCTGTCCCAGGTGCAACTGAAGGAGTCAGGACCTGGCCTGGTGAAGCCCTCGCAGACCCTCTCCCTCAC
CTGCACTGTCTCTGGATTATCTTTGAGCAGTAATGCTGTAGGCTGGGTCCGCCAGGCTCCAGGAAAAGGGCTGGAAT
TTGTTGGTGCTATATATGGTAGTGCAAGTGCAAACTACAACCCAGCCCTGAAGTCCCGAGCCAGCATCACCAAGGAC
ACCTCAAAGAGCCAAGTTTATCTGACGCTGAACAGCCTGACAAGCGAGGACACGGCCGTCTATTACTGTGCAGGAGG
CTTAGACAGTTACTATGGTGGTAGTTCCTGGTGGAGGAGTTATGGTATAAACTACTGGGGCCAGGGCATCC
>KC549688.1 Equus caballus clone 5'RACE Eq F Spl B07 immunoglobulin heavy chain mRNA, partial cds
ATTTTCAGGGAGTCGTGAATCTCATCTGCAAGAACATGAGTCACCTGTGGTTCTTCCTCTTTCTGGTGGCCGCTCCT
ACATGTGTCCTGTCCCAGGTGCAACTGAAGGAGTCAGGACCTGGCCTGGTGAAGCCCTCGCAGACCCTCTCCCTCAC
CTGCACTGTCTCTGGATTATCTTTGAGCAGTTATGGTGTGGGCTGGGTCCGCCAGGCTCCAGGAAAAGGGCTGGAAT
TTGTTGGTGGTATAGCTAGTAGTGGAAGTGCAAACTACAACCCAGCCCTGAAGTCCCGAGCCAGCATCACCAAGGAC
ACCTCAAAGAGCCAAGTTTATCTGACGCTGAACAGCCTGACAAGCGAGGACACGGCCGTCTATTACTGTGCAGGAGG
CCGGAGAGTTGGAGTAAACTACTGGGGCCAGGGCATCC
>KC549687.1 Equus caballus clone 5'RACE Eq F Spl-06 immunoglobulin heavy chain mRNA, partial cds
ATTTTCAGGGAGTCGTGAATCTCATCTGCAAGAACATGAGTCACCTGTGGTTCTTCCTCTTTCTGGTGGCCGCTCCT
ACATGTGTCCTGTCCCAGGTGCAACTGAAGGAGTCAGGACCTGGCCTGGTGAAGCCCTCGCAGACCCTGTCCCTCAC
CTGCACTGTCTCTGGATTCTCTTTGAGCAGTTACGGTGTAGGCTGGGTCCGCCAGGCTCCAGGAAAAGGGCTGGAAT
TTGTTGGTGGTATAGCTAGTAGTGGAAGTGCAAACTACAACCCAGCCCTGAAGTCCCGAGCCAGCATCACCAAGGAC
ACCTCAAAGAGCCAAGTTTATCTGACGCTGAACAGCCTGACAGGCGAGGACACGGCCGTCTATTACTGTGCGAGATA
TGGTTACTATGGTAGTTACTACAGTAGTTACTATGGCTACGTGGATCACTGGGGCCAGGGCACCC
>KC549683.1 Equus caballus clone 5'RACE Eq F Spl C01 immunoglobulin heavy chain mRNA, partial cds
TATTTTCAGGTAGTCGTGAATCTCATCTGCAAGAACATGAGTCACCTGTGGTTCTTCCTCTTTCTGGTGGCCGCTCC
TACATGTGTCCTGTCCCAGGTGCAACTGAAGGAGTCAGGACCTGGCCTGGTGAAGCCCTCGCAGACCCTGTCCCTCA
CCTGCACTGTCTCTGGATTCTCTTTGAGCAGTTACGGTGTAGGCTGGGTCCGCCAGGCTCCAGGAAAAGGGCTGGAA
TATGTTGGTGGTATAGCTAGTAGTGGAAGTGCAAACTACAACCCAGCCCTGAAGTCCCGAGCCAGCATCACCAAGGA
CACCTCAAAGAGCCAAGTTTATCTGACGCTGAACAGCCTGACAGGCGAGGACACGGCCGTCTATTACTGTGCGAGCG
GTTATGGTTATGCTTTTGGCTACTGGGGCCAGGGCACCC
>KC549681.1 Equus caballus clone 5'RACE Eq F Spl A11 immunoglobulin heavy chain mRNA, partial cds
ATTTTCAGGTAGTCGTGAATCTCATCTGCAAGAACATGAGTCACCTGTGGTTCTTCCTCTTTCTGGTGGCCGCTCCT
ACATGTGTCCTGTCCCAGGTGCAACTGAAGGAGTCAGGACCTGGCCTGGTGAAGCCCTCGCAGACCCTGTCCCTCAC
CTGCACTGTCTCTGGATTCTCTTTGAGCAGTTACGGTGTAGGCTGGGTCCGCCAGGCTCCAGGAAAAGGGCTGGAAT
ATGTTGGTGGTATAGCTAGTAGTGGAAGTGCAAACTACAACCCAGCCCTGAAGTCCCGAGCCAGCATCACCAAGGAC
ACCTCAAAGAGCCAAGTTTATCTGACGCTGAACAGCCTGACAGGCGAGGACACGGCCGTCTATTACTGTGCGAGATA
TAGCAGCTATGCTTACTATGGTATAAACTACTGGGGCCAGGGCATCC
>KC549680.1 Equus caballus clone 5'RACE Eq F Spl B09 immunoglobulin heavy chain mRNA, partial cds
ATTTTCAGGTAGTCGTGAATCTCATCTGCAAGAACATGAGTCACCTGTGGTTCTTCCTCTTTCTGGTGGCCGCTCCT
ACATGTGTCCTGTCCCAGGTGCAACTGAAGGAGTCAGGACCTGGCCTGGTGAAGCCCTCGCAGACCCTGTCCCTCAC
CTGCACTGTCTCTGGATTCTCTTTGAGCAGTTACGGTGTAGGCTGGGTCCGCCAGGCTCCAGGAAAAGGGCTGGAAT
ATGTTGGTGGTATAGCTAGTAGTGGAAGTGCAAACTACAACCCAGCCCTGAAGTCCCGAGCCAGCATCACCAAGGAC

ACCTCAAAGAGCCAAGTTTATCTGACGCTGAACAGCCTGACAGGCGAGGACACGGCCGTCTATTACTGTGCGATAGC
CTACGGTTATGGTTATGCTTTTGGCTACTGGGGCCAGGGCACCC
>KY437643.1 Equus caballus clone 487 immunoglobulin mu heavy chain (IGHM)
mRNA, partial cds
CTCTCCCTCACCTGCACTGTCTCTGGATTATCTTTGAGCAGTAATGCTGTAGGCTGGGTCCGCCAGGCTCCAGGAAA
AGGGCTGGAATTTGTTGGTACTATAGCTAGTAGTGGAAGTGCAAACTACAACCCAGCCCTGAAGTCCCGAGCCAGCA
TCACCAAGGACACCTCAAAGAGCCAAGTTTATCTGACGCTGAACAGCCTGACAAGCGAGGACACGGCCGTCTATTAC
TGTTGTGCAGGAATGGATGGTGGTAGTTCCTGGTACCTCCTAGGCTACGTGGATCACTGGGGCCAGGGCACCCTGGT
CACCGTCTCCTCAGAGAGTACGATGACCCCAGATCTCTTCCCCCTCGTCTCCTGTGGGCCCTCTCTTGATGAGAGCC
TGGTGGCTGTGGGCTGCCTAGCCCGGGACTTCCTACCCAA
>KY437642.1 Equus caballus clone 486 immunoglobulin mu heavy chain (IGHM)
mRNA, partial cds
CTCTCCCTCACCTGCACTGTCTCTGGATTATCTTTGAGCAGTAATGCTGTAGGCTGGGTCCGCCAGGCTCCAGGAAA
AGGGCTGGAATTTGTTGGTGCTATATATGGTAGTGCAAGTGCAAACTACAACCCAGCCCTGAAGTCCCGAGCCAGCA
TCACCAAGGACACCTCAAAGAGCCAAGTTTATCTGACGCTGAACAGCCTGACAAGCGAGGACACGGCCGTCTATTAC
TGTGTAGGAGGCGTTTATTGGGGCCAGGGCACCCTGGTCACCGTCTCCTCAGAGAGTACGATGACCCCAGATCTCTT
CCCCCTCGTCTCCTGTGGGCCCTCTCTTGATGAGAGCCTGGTGGCTGTGGGCTGCCTAGCCCGGGACTTCCTACCCA
A
>KY437641.1 Equus caballus clone 485 immunoglobulin mu heavy chain (IGHM)
mRNA, partial cds
CTCTCCCTCACCTGCACTGTCTCTGGATTATCTTTGAGCAGTAATGCTGTAGGCTGGGTCCGCCAGGCTCCAGGAAA
AGGGCTGGAATATGTTGGTGGTATAGCACGTAGTGGAAGTGCAAACTACAACCCAGCCCTGAAGTCCCGAGCCAGCA
TCACCAAAGACACCTCAAAGAGCCAAGTTTATCTGACGCTGAACAGCCTGACAAGCGAGGACACGGCCGTCTATTAC
TGTGCAGGAGGGGACTATGGTTATGGTAGTACTTACTACTACGCCCCTTATGGTATAAACTACTGGGGCCAGGGCAT
CCTGGTCACCGTCTCCTCAGAGAGTACGATGACCCCAGATCTCTTCCCCCTCGTCTCCTGTGGGCCCTCTCTTGATG
AGAGCCTGGTGGCTGTGGGCTGCCTAGCCCGGGACTTCCTACCCAA
>KY437640.1 Equus caballus clone 484 immunoglobulin mu heavy chain (IGHM)
mRNA, partial cds
CTGTCCCTCACCTGCACTGTCTCTGGATTCTCTTTGAGCAGTTACGGTGTAGGCTGGGTCCGCCAGGCTCCAGGAAA
AGGGCTGGAATATGTTGGTGGTATAGCTAGTAGTGGAAGTGCAAACTACAACCCAGCCCTGAAGTCCCGAGCCAGCA
TCACCAAGGACACCTCAAAGAGCCAAGTTTATCTGACGCTGAACAGCCTGACAGGCGAGGACACGGCCGTCTATTAC
TGTGTGTCCACTTACTATGGTGGTAGTTCCTCCGATTTTGGCTACTGGGGCCAGGGCACCCTGGTCACCGTCTCCTC
AGAGAGTACGATGACCCCAGATCTCTTCCCCCTCGTCTCCTGTGGGCCCTCTCTTGATGAGAGCCTGGTGGCTGTGG
GCTGCCTAGCCCGGGACTTCCTACCCAA
>KY437639.1 Equus caballus clone 483 immunoglobulin mu heavy chain (IGHM)
mRNA, partial cds
CTGTCCCTCACCTGCACTGTCTCTGGATTATCTTTGAGCAGTAATGCTGTAGGCTGGGTCCGCCAGGCTCCAGGAAA
AGGGCTGGAGTGGGTTGGTGTTATATATGGTAGTGAAAGTACATACTACAACCCAGCCCTGAAGTCCCGAGCCAGCA
TCACCAAGGACACCTCAAAGAGCCAAGTTTATCTGACGCTGAACAGCCTGACAGGCGAAGACACGGCCGTCTATTAC
TGTGCAGGATCGGGGAGTGGTTATGGCTACAGTTATTATGATATAAACTACTGGGGCCAGGGCATCCTGGTCACCGT
CTCCTCAGAGAGTACGATGACCCCAGATCTCTTCCCCCTCGTCTCCTGTGGGCCCTCTCTTGATGAGAGCCTGGTGG
CTGTGGGCTGCCTAGCCCGGGACTTCCTACCCAA
>KY437638.1 Equus caballus clone 482 immunoglobulin mu heavy chain (IGHM)
mRNA, partial cds
CTCTCCCTCACCTGCACTGTCTCTGGATTATCTTTGAACAGTAATGCTGTAGGCTGGGTCCGCCAGGCTCCAGGAAA
AGGGCTGGAATATGTTGGTGATATAGCTGGTAGTGGAAGTGCAAACTACAACCCAGCCCTGAAGTCCCGAGCCAGCA
TCACCAAGGACACCTCAAAGAGCCAAGTTTATCTGACGCTGAACAGCCTGACAAGCGAGGACACGGCCGTCTATTAC
TGTGCAGGAGGCGGGGGTTACTATGCTAGTGGTTATGGTCTTGACTACTGGGGCCAGGGCACCCTGGTCACCGTCTC
CTCAGAGAGTACGATGACCCCAGATCTCTTCCCCCTCGTCTCCTGTGGGCCCTCTCTTGATGAGAGCCTGGTGGCTG
TGGGCTGCCTAGCCCGGGACTTCCTACCCAA
>KY437637.1 Equus caballus clone 481 immunoglobulin mu heavy chain (IGHM)
mRNA, partial cds
CTCTCCCTCACCTGCACTGTCTCTGGATTATCTTTGAGCAGTAGTGCTGTAGGCTGGGTCCGCCAGGCTCCAGGAAA
AGGGCTGGAATATGTTGGTGGTATAGCCGGTAGTGGAACAGCAAGCTACAACCCAGCCCTGAAGTCCCGAGCCAGCA
TCACCAAGGACACCTCAAAGAGCCAAGTTTATCTGACGCTGAACAGCCTGACAGGCGAGGACACGGCCGTCTATTAC

TGTGCGAGATTCGCTGGTGATGCTAGTAGTAACTTGTACTGGGGCCAGGGCACCCTGGTCACCGTCTCCTCAGAGAG
TACGATGACCCCAGATCTCTTCCCCCTCGTCTCCTGTGGGCCCTCTCTTGATGAGAGCCTGGTGGCTGTGGGCTGCC
TAGCCCGGGACTTCCTACCCAA
>KY437636.1 Equus caballus clone 480 immunoglobulin mu heavy chain (IGHM) mRNA, partial cds
CTCTCCCTCACCTGCACTGTCTCTGGATTATCTTTGAGCAGTAATGCTGTAGGCTGGGTCCGCCAGGCTCCAGGAAA
AGGGCTGGAATTTGTTGGTGCTATAGGTGGTAGTGCAAGTGCAAACTACAACCCAGCCCTGAAGTCCCGAACCAGCA
TCACCAAGGACACCTCAAAGAGCCAAGTTTATCTGACGCTGAACAGCCTGACAAGCGAGGACACGGCCGTCTATTAC
TGTGCAGGAGGAGTACAACTTTATGGTGGTGCTTACTACCCTTTTGGCTACTGGGGCCAGGGCACCCTGGTCACCGT
CTCCTCAGAGAGTACGATGACCCCAGATCTCTTCCCCCTCGTCTCCTGTGGGCCCTCTCTTGATGAGAGCCTGGTGG
CTGTGGGCTGCCTAGCCCGGGACTTCCTACCCAA
>KY437635.1 Equus caballus clone 479 immunoglobulin mu heavy chain (IGHM) mRNA, partial cds
CTGTCCCTCACCTGCACTGTCTCTGGATTATCTTGGAGCAGTAATGCTGTAGGCTGGGTCCGCCAGGCTCCAGGAAA
AGGGCTGGAGTGGGTTGGTGTTATATATGGTAGTGAAAGTACATACTACAACCCAGCCCTGAAGTCCCGAGCCAGCA
TCACCAAGGACACCTCAAAGAGCCAAGTTTATCTGACGCTGAACAGCCTGACAGGCGAAGACACGGCCGTCTATTAC
TGTGCAGGATATGATCTCTATGGTTATGGTGGTGCTTACTACTCTCTCGGTTATTATGGTATAAACTACTGGGGCCA
GGGCATCCTGGTCACCGTCTCCTCAGAGAGTACGATGACCCCAGATCTCTTCCCCCTCGTCTCCTGTGGGCCCTCTC
TTGATGAGAGCCTGGTGGCTGTGGGCTGCCTAGCCCGGGACTTCCTACCCAA
>KY437634.1 Equus caballus clone 478 immunoglobulin mu heavy chain (IGHM) mRNA, partial cds
CTCTCCCTCACCTGCACTGTCTCTGGATTATCTTTGAGCAGTAATGCTGTAGGCTGGGTCCGCCAGGCTCCAGGAAA
AGGGCTGGAATATGTTGGTGGTATAGTTAGTAGTGGAAGTGCAAACTACAACCCAGCCCTGAAGTCCCGAGCCAGCA
TCACCAAGGACACCTCAAAGAGCCAAGTTTATCTGACGCTGAACAGCCTGACAAGCGAGGACACGGCCGTCTATTAC
TGTGCAGGGATATCGGGTTATGGTGGTGCTTACTACTGGGGGGATGGTATAAACTACTGGGGCCAGGGCATCCTGGT
CACCGTCTCCTCAGAGAGTACGATGACCCCAGATCTCTTCCCCCTCGTCTCCTGTGGGCCCTCTCTTGATGAGAGCC
TGGTGGCTGTGGGCTGCCTAGCCCGGGACTTCCTACCCAA
>KY437633.1 Equus caballus clone 477 immunoglobulin mu heavy chain (IGHM) mRNA, partial cds
CTGTCCCTCACCTGCACTGTCTCTGGATTATCTTTGAGCAGTAATGCTGTAGGCTGGGTCCGCCAGGCTCCAGGAAA
AGGGCTGGAGTGGGTTGGTGTTATATATGGTAGTGAAAGTACATACTACAACCCAGCCCTGAAGTCCCGAGCCAGCA
TCACCAAGGACACCTCAAAGAGCCAAGTTTATCTGACGCTGAACAGCCTGACAGGCGAAGACACGGCCGTCTATTAC
TGTGCAGGATTTTATAGCAGCTATGCTTACTACACCTTTCGGGGCTACGTGGATCACTGGGGCCAGGGCACCCTGGT
CACCGTCTCCTCAGAGAGTACGATGACCCCAGATCTCTTCCCCCTCGTCTCCTGTGGGCCCTCTCTTGGTGAGAGCC
TGGTGGCTGTGGGCTGCCTAGCCCGGGACTTCCTACCCAA
>KY437632.1 Equus caballus clone 476 immunoglobulin mu heavy chain (IGHM) mRNA, partial cds
CTGTCCCTCACCTGCACTGTCTCTGGATTATCTTTGAGCAGTAATTCTGTAGGCTGGGTCCGCCAGGCTCCAGGAAA
AGGGCTGGAGTGGGTTGGTGTTATATATGGTAGTGAAAGTACATACTACAACCCAGCCCTGAAGTCCCGAGCCAGCA
TCACCAAGGACACCTCAAAGAGCCAAGTTTATCTGACGCTGAACAGTCTGACAAGCGAGGACACGGCCGTCTATTAC
TGTGCAGGAGGCAGCGACTATGCTTACTACGTTATCTCTTATGGTATAAACTACTGGGGCCAGGGCATCCTGGTCAC
CGTCTCCTCAGAGAGTACGATGACCCCAGATCTCTTCCCCCTCGTCTCCTGTGGGCCCTCTCTTGATGGGAGCCTGG
TGGCTGTGGGCTGCCTAGCCCGGGACTTCCTACCCAA
>KY437631.1 Equus caballus clone 475 immunoglobulin mu heavy chain (IGHM) mRNA, partial cds
CTCTCCCTCACCTGCACTGTCTCTGGATTATCTTTGAGCAGTAATGCTGTAGGCTGGGTCCGCCAGGCTCCAGGAAA
AGGGCTGGAATATGTTGGTGGTATAGCTAGTAGTGGAAGTGCAAACTACAACCCAGCCCTGAAGTCCCGAGCCAGCA
TCACCAAGGACACCTCAAAGAGCCAAGTTTATCTGACGCTGAACAGCCTGACAAGCGAGGACACGGCCGTCTATTAC
TGTGCAGGATATTATGGTAGTTACTATTACTACGTGGATCACTGGGGCCAGGGCACCCTGGTCACCGTCTCCTCAGA
GAGTACGATGACCCCAGATCTCTTCCCCCTCGTCTCCTGTGGGCCCTCTCTTGATGAGAGCCTGGTGGCTGTGGGCT
GCCTAGCCCGGGACTTCCTACCCAA
>KY437630.1 Equus caballus clone 474 immunoglobulin mu heavy chain (IGHM) mRNA, partial cds
CTCTCCCTCACCTGCACTGTCTCTGGATTATCTTTGAGCAGTAATGCTGTAGGCTGGGTCCGCCAGGCTCCAGGAAA
AGGGCTGGAATTTGTTGGTGGAATATATGGTAGTGCAAGTGCAAACTACAACCCAGCCCTGAAGTCCCGAGCCAGCA
TCACCAAGGACACCTCAAAGAGCCAAGTTTATCTGACGCTGAACAGCCTGACGAGCGAGGACACGGCCGTCTATTAC

TGTGCAGGAACGGAATATAGTTATGAAAGTTACTATGCCTATTATGGTATGTGCTACTGGGGCCAGGGCATCCTGGT
CACCGTCTCCTCAGAGAGTACGATGACCCCAGATCTCTTCCCCCTCGTCTCCTGTGGGCCCTCTCTTGATGAGAGCC
TGGTGGCTGTGGGCTGCCTAGCCCGGGACTTCCTACCCAA
>KY437629.1 Equus caballus clone 473 immunoglobulin mu heavy chain (IGHM)
mRNA, partial cds
CTGTCCCTCACCTGCACTGTCTCTGGATTATCTTTGAGCAGTAATTCTGTAGGCTGGGTCCGCCAGGCTCCAGGAAA
AGGGCTGGAGTGGGTTGGTAGAAGCTATGGTAGTGAAATTACAGTCTACAACCCAGCCCTGAAGTCCCGAGTCAGCA
TCACCGTGGACACCTCAAAGAGCCAAGTTTATCTGACGCTGAACAGCCTGACAGGCGAAGACACGGCCGTCTATTAC
TGTGCAGGATGGCAGCTCTATAGTGTTGGTAGTCACTACTCGGCTGAATTTGCCTACTGGGGCCAGGGCACCCTGGT
CACCGTCTCCTCAGAGAGTACGATGACCCCAGATCTCTTCCCCCTCGTCTCCTGTGGGCCCTCTCTTGATGAGAGCC
TGGTGGCTGTGGGCTGCCTAGCCCGGGACTTCCTACCCAA
>KY437628.1 Equus caballus clone 472 immunoglobulin mu heavy chain (IGHM)
mRNA, partial cds
CTGTCCCTCACCTGCACTGTCTCTGGATTATCTTTGAGCAGTAATGCTGTAGGCTGGGTCCGCCAGGCTCCAGGAAA
AGGGCTGGAGTGGGTTGCTTGTATATATGGTGACGGAAGTACAAGATACAACCCAGCCCTGAAGTCCCGAGCCAGCA
TCACCAAGGACACCTCAAAGAGCCAAGTTTATCTGACGCTGAACAGCCTGACAAGCGAGGACACGGCCGTCTATTAC
TGTGCAGGAGTCCTCTATGGTTATGGTGGTGCTTACTACTGGTCTCCCCCGGAGAATGCTATGGACCCCTGGGGCCA
GGGCACCCTGGTCACCGTCTCCTCAGAGAGTACGATGACCCCAGATCTCTTCCCCCTCGTCTCCTGTGGGCCCTCTC
TTGATGAGAGCCTGGTGGCTGTGGGCTGCCTAGCCCGGGACTTCCTACCCAA
>KY437627.1 Equus caballus clone 471 immunoglobulin mu heavy chain (IGHM)
mRNA, partial cds
CTCTCCCTCACCTGCACTGTCTCTGGATTATCTTTGAGCAGTAATGCTGTAGGCTGGGTCCGCCAGGCTCCAGGAAA
AGGGCTGGAATATGTTGGTGGTATAGCTAGTAGTGGAAGTGCGAACTACAACCCAGCCCTGAAGTCCCGAGCCAGCA
TCACCAAGGACACCTCAAAGAGCCAAGTTTATCTGACGCTGAACAGCCTGACAGGCGAGGACACGGCCGTCTATTAC
TGTGCGAGATCATCGATTGTGCTGAATACTAATGTAGGAGTTTTTGTACCGGGGGCTATGGACCCCTGGGGCCAGGG
CACCCTGGTCACCGTCTCCTCAGAGAGTACGATGACCCCAGATCTCTTCCCCCTCGTCTCCTGTGGGCCCTCTCTTG
ATGAGAGCCTGGTGGCTGTGGGCTGCCTAGCCCGGGACTTCCTACCCAA
>KY437626.1 Equus caballus clone 470 immunoglobulin mu heavy chain (IGHM)
mRNA, partial cds
CTCTCCCTCACCTGCACTGTCTCTGGATTATCTTTGAGCAGTAATGCTGTAGGCTGGGTCCGCCAGGCTCCAGGAAA
AGGGCTGGAATTTGTTGGTGCTATATATGGTAGTGCAAGTGCAAACTACAACCCAGCCCTGAAGTCCCGAGCCAGCA
TCACCAAGGACACCTCAAAGAGCCAAGTTTATCTGACGCTGAACAGCCTGACAAGCGAGGACACGGCCGTCTATTAC
TGTGCAGGAGGCTCCCCAAGATTATATGGTTACTATGCTAGTGGTTATGGGAATGCTATGGACCCCTGGGGCCAGGG
CACCCTGGTCACCGTCTCCTCAGAGAGTACGATGACCCCAGATCTCTTCCCCCTCGTCTCCTGTGGGCCCTCTCTTG
ATGAGAGCCTGGTGGCTGTGGGCTGCCTAGCCCGGGACTTCCTACCCAA
>KY437625.1 Equus caballus clone 469 immunoglobulin mu heavy chain (IGHM)
mRNA, partial cds
CTCTCCCTCACCTGCACTGTCTCTGGATTATCTTTGAGCAGTAATGCTGTAGGCTGGGTCCGCCAGGCTCCAGGAAA
AGGGCTGGAATTTGTTGGTGCTATATATGGTAGTGCAAGTGCAAACTACAACCCAGCCCTGAAGTCCCGAGCCAGCA
TCACCAAGGACACCTCAAAGAGCCAAGTTTATCTGACGCTGAACAGCCTGACAAGCGAGGACACGGCCGTCTATTAC
TGTGCAGGAGTTAGGGGTAGTTATGGTAGTTACTATGCCGTAACTTATGGCTACGTGGATCACTGGGGCCAGGGCAC
CCTGGTCACCGTCTCCTCAGAGAGTACGATGACCCCAGATCTCTTCCCCCTCGTCTCCTGTGGGCCCTCTCTTGATG
AGAGCCTGGTGGCTGTGGGCTGCCTAGCCCGGGACTTCCTACCCAA
>KY437624.1 Equus caballus clone 468 immunoglobulin mu heavy chain (IGHM)
mRNA, partial cds
CTCTCCCTCACCTGCACTGTCTCTGGATTATCTTTGAGCAGTAATGCTGTAGGCTGGGTCCGCCAGGCTCCAGGAAA
AGGGCTGGAATACGTTGGTGCTATATATGGTAGTGCAAGTGCAAACTACAACCCAGCCCTGAAGTCCCGAGCCAGCA
TCACCAAGGACACCTCAAAGAGCCAAGTTTATCTGACGCTGAACAGCCTGACAAGCGAGGACACGGCCGTCTATTAC
TGTGCAGGAGCCGACTACGGTTATGGTTATGCTTTTCGATATTATTATGGTATAAACTACTGGGGCCAGGGCATCCT
GGTCACCGTCTCCCCAGAGAGTACGATGACCCCAGATCTCTTCCCCCTCGTCTCCTGTGGGCCCTCTCTTGATGAGA
GCCTGGTGGCTGTGGGCTGCCTAGCCCGGGACTTCCTACCCAA
>KY437623.1 Equus caballus clone 467 immunoglobulin mu heavy chain (IGHM)
mRNA, partial cds
CTGTCCCTCACCTGCACTGTCTCTGGATTCTCTTTGAGCAGTTACGGTGTAGGCTGGGTCCGCCAGGCTCCAGGAAA
AGGGCTGGAATATGTTGGTGGTATAGCTAGTAGTGGAAGTGCAAACTACAACCCAGCCCTGAAGTCCCGAGCCAGCA

TCACCAAGGACACCTCAAAGAGCCAAGTTTATCTGACGCTGAACAGCCTGACAGGCGAGGACACGGCCGTCTATTAC
TGTGCGGGATCGTTACGAAATTTTAGGAGTCAGCGACCTTATGGTATAAACTACTGGGGCCAGGGCATCCTGGTCAC
CGTCTCCTCAGAGAGTACGATGACCTCAGATCTCTTCCCCCTCGTCTCCTGTGGGCCCTCTCTTGATGAGAGCCTGG
TGGCTGTGGGCTGCCTAGCCCGGGACTTCCTACCCAA
>KY437622.1 Equus caballus clone 466 immunoglobulin mu heavy chain (IGHM) mRNA, partial cds
CTGTCCCTCACCTGCACTGTCTCTGGATTATCTTTGAGCAGTAATGCTGTAGGCTGGGTCCGCCAGGCTCCAGGAAA
AGGGCTGGAATATGTTGGTGGTATAGCTAGTAGTGGAAGTGCAAACTACAACCCAGCCCTGAAGTCCCGAGCCAGCA
TCACCAAGGACACCTCAAAGAGCCAAGTTTATCTGACGCTGAACAGCCTGACAAGCGAGGACACGGCCGTCTATTAC
TGTGCAGGATATGGTTACTATGGTAGTTACTACAGTAGTTACTATGCCCTAAACTACTGGGGCCAGGGCATCCTGGT
CACCGTCTCCTCAGAGAGTACGATGACCCCAGATCTCTTCCCCCTCGTCTCCTGTGGGCCCTCTCTTGATGAGAGCC
TGGTGGCTGTGGGCTGCCTAGCCCGGGACTTCCTACCCAA
>KY437621.1 Equus caballus clone 465 immunoglobulin mu heavy chain (IGHM) mRNA, partial cds
CTCTCCCTCACCTGCACTGTCTCTGGATTATCTTTGAGCAGTAATGCTGTAGGCTGGGTCCGCCAGGCTCCAGGAAA
AGGGCTGGAATTTGTTGGTGCTATATATGGTAGTGCAAGTGCAAACTACAACCCAGCCCTGAAGTCCCGAGCCAGCA
TCACCAAGGACACCTCAAAGAGCCAAGTTTATCTGACGCTGAACAGCCTGACAAGCGAGGACACGGCCGTCTATTAC
TGTGCAGGAGGCTTCTATGGTTTTGGTGGTGCTTGGTATCCTGATGGTATAAACTACTGGGGCCAGGGCATCCTGGT
CACCGTCTCCTCAGAGAGTACGATGACCCCAGATCTCTTCCCCCTCGTCTCCTGTGGGCCCTCTCTTGATGAGAGCC
TGGTGGCTGTGGGCTGCCTAGCCCGGGACTTCCTACCCAA
>KY437620.1 Equus caballus clone 464 immunoglobulin mu heavy chain (IGHM) mRNA, partial cds
CTCTCCCTCACCTGCACTGTCTCTGGACTCTCTTTGAGCAGTTATACTGTATACTGGGTCCGCCAGGCTCCCGGAAA
AGGGCTGGAACATGTTGCTTATATAGCAAGTAGTGGAAGTACAAATTACAACCCAGCCCTGAAGTCCCGAGCCAGCA
TCACCAAGGACACCTCCAAGAGCCAAGTTTATCTGACGCTGAACAGCCTGACAGGCGAGGACACGGCCGTCTATTAT
TGTAATTGTGGCGACTACTGGGGCCAGGGCATCCTGGTCACCGTCTCCTCAGAGAGTACGATGACCCCAGATCTCTT
CCCCCTCGTCTCCTGTGGGCCCTCTCTTGATGAGAGCCTGGTGGCTGTGGGCTGCCTAGCCCGGGACTTCCTACCCA
A
>KY437619.1 Equus caballus clone 463 immunoglobulin mu heavy chain (IGHM) mRNA, partial cds
CTGTCCCTCACCTGCACTGTCTCTGGATTATCTTTGAGCAGTAATGCTGTAGGCTGGGTCCGCCAGGCTCCAGGAAA
AGGGCTGGAATATGTTGGTGGTATACGTAGGAGTGGAAGTGCAAACTACAACCCAGCCCTGAAGTCCCGAGCCAGCA
TCACCAAGGACACCTCAAAGAGCCAAGTTTATCTGACGCTGAACAGCCTGACAAGCGAGGACACGGCCGTCTATTAC
TGTGCGCGATATTCTTACTATGATGGTATAAGGTACTGGGGCCAGGGCATCCTGGTCACCGTCTCCTCAGAGAGTAC
GATGACCCCAGATCTCTTCCCCCTCGTCTCCTGTGGGCCCTCTCTTGATGAGAGCCTGGTGGCTGTGGGCTGCCTAG
CCCGGGACTTCCTACCCAA
>KY437618.1 Equus caballus clone 462 immunoglobulin mu heavy chain (IGHM) mRNA, partial cds
CTCTCCCTCACCTGCACTGTCTCTGGATTATCTTTGAGCAGTAATGCTGTAGGCTGGGTCCGCCAGGCTCCAGGAAA
AGGGCTGGAATACGTTGGTCATATATATGGTAGTCAAAGTGCAATTTACAACCCAGCCCTGAAGTCCCGAGCCAGCA
TCACCAAGGACACCTCAAAGAGCCAAGTTTATCTGACGCTGAACAGCCTGACAAGCGAGGACACGGCCGTCTATTAC
TGTGAAGGAGGCAGGAATGGTTATGGTGGTGCTTACTACCCTATTTATGGTATAAACTACTGGGGCCAGGGCATCCT
GGTCACCGTCTCCTCAGAGAGTACGATGACCCCAGATCTCTTCCCCCTCGTCTCCTGTGGGCCCTCTCTTGATGAGA
GCCTGGTGGCTGTGGGCTGCCTAGCCCGGGACTTCCTACCCAA
>KY437617.1 Equus caballus clone 461 immunoglobulin mu heavy chain (IGHM) mRNA, partial cds
CTCTCCCTCACCTGCACTGTCTCTGGATTATCTTTGAGCAGTAATGCTGTAGGCTGGGTCCGCCAGGCTCCAGGAAA
AGGGCTGGAATATGTTGGTGGTATAGCTAGTAGTGGAAGTGCAAACTACAACCCAGCCCTGAAGTCCCGAGCCAGCA
TCACCAAGGACACCTCAAAGAGCCAAGTTTATCTGACGCTGAACAGCCTGACAAGCGAGGACACGGCCGTCTATTAC
TGTGCAGGAGCGTTCGGTTCCTGGTACTCCCCTGCCTACTTTGGCTACTGGGGCCAGGGCACCCTGGTCACCGTCTC
CTCAGAGAGTACGATGACCCCAGATCTCTTCCCCCTCGTCTCCTGTGGGCCCTCTCTTGATGAGAGCCTGGTGGCTG
TGGGCTGCCTAGCCCGGGACTTCCTACCCAA
>KY437616.1 Equus caballus clone 460 immunoglobulin mu heavy chain (IGHM) mRNA, partial cds
CTCTCCCTCACCTGCACTGTCTCTGGATTATCTTTGAGCAGTAACTCTGTAGGCTGGGTCCGCCAGGCTCCAGGAAA
AGGGCTGGAATATGTTGGTGGTATAGCTAGTAGTGGAGGTGCAAACTACAACCCAGCCCTGAAGTCCCGAGCCAGCA

TCACCAAGGGCACCTCAAAGAGCCAAGTTTATCTGACGCTGAACAGCCTGACAAGCGAGGACACGGCCGTCTATTAC
TGTGCAGGAGGGGCGGGTTCCTATGCTGGTAGTTACTTATACTATTTTCCTGCCGGGGGTATACGCTACTGGGGCCA
GGGCATCCTGGTCACCGTCTCCTCAGAGAGTACGAAGACCCCAGATCTCTTCCCCCTCGTCTCCTGTGGGCCCTCTC
TTGATGAGAGCCTGGTGGCTGTGGGCTGCCTAGCCCGGGACTTCCTACCCAA
>KY437615.1 Equus caballus clone 459 immunoglobulin mu heavy chain (IGHM) mRNA, partial cds
CTCTCCCTCACCTGCACTGTCTCTGGATTATCTTTGAGCAGTAATGCTGTAGGCTGGGTCCGCCAGGCTCCAGGAAA
AGGGCTGGAATATGTTGGTGGTATAGCTAGTAGTGGAAGTGCAAACTACAACCCAGCCCTGAAGTCCCGAGCCAGCA
TCACCAAGGACACCTCAAAGAGCCAAGTTTATCTGACGCTGAACAGCCTGACAGGCGAGGACACGGCCGTCTATTAC
TGTGCGAAATCCTCTTACTACTTTGGTAGTTACCATTATTATGATATAGACTACTGGGGCCAGGGCACCCTGGTCAC
CGTCTCCTCAGAGAGTACGAAGACCCCAGATCTCTTCCCCCTCGTCTCCTGTGGGCCCTCTCTTGATGAGAGCCTGG
TGGCTGTGGGCTGCCTAGCCCGGGACTTCCTACCCAA
>KY437614.1 Equus caballus clone 458 immunoglobulin mu heavy chain (IGHM) mRNA, partial cds
CTCTCCCTCACCTGCACTGTCTCTGGATTATCTTTGAGCAGTAATGCTGTAGGCTGGGTCCGCCAGGCTCCAGGAAA
AGGGCTGGAATATGTTGGTGGTATAGCTAGTAGTGGAAGTGCAAACTACAACCCAGCCCTGAAGTCCCGAGCCAGCA
TCACCAAGGACACCTCAAAGAGCCAAGTTTATCTGACGCTGAACAGCCTGACAAGCGAGGACACGGCCGTCTATTAC
TGTGCAGGAGGCTATGGTTATGGTGGTGCTTACTACTACATGAGAGAGGGGTTTGGCTACTGGGGCCAGGGCACCCT
GGTCACCGTCTCCCCAGAGAGTACGATGACCCCAGATCTCTTCCCCCTCGTCTCCTGTGGGCCCTCTCTTGATGAGA
GCCTGGTGGCTGTGCGCTGCCTAGCCCGGGACTTCCTACCCAA
>KY437613.1 Equus caballus clone 457 immunoglobulin mu heavy chain (IGHM) mRNA, partial cds
CTGTCCCTCACCTGCACTGTCTCTGGATTATCTTTGAGCAGAGATACTGTAGGCTGGGTCCGCCAGGCTCCAGGAAA
AGGGCTGGAGTGGGTTGGTGTTATATATGAGAGTGGAACTGTATACTACAATACAGCCCTGAAGTCCCGAATCAGCA
TCACCAAGGACACCTCAAAGAGCCAAGTTTATCTGACGCTGAACAGCCTGACAGGCGAAGACACGGCCGTCTATTAC
TGTGCAGTATGTGGTTATGGTGGTTCTTGTAACTCTGGTACAAACTACTGGGGCCAGGGCATCCTGGTCACCGTCTC
CTCAGAGAGTACGATGACCCCAGATCTCTTCCCCCTCGTCTCCTGTGGGCCCTCTCTTGATGAGAGCCTGGTGGCTG
TGGGCTGCCTAGCCCGGGGCTTCCTACCCAA
>KY437612.1 Equus caballus clone 456 immunoglobulin mu heavy chain (IGHM) mRNA, partial cds
CTCTCCCTCACCTGCACTGTCACTGGAGGCTCCATCACAAGCAGCTATTCTAGCTGGAGCTGGTTACGCCAGCCTCC
AGGGAAGGGGCTGGAGTACATGGGGTACATATATTATGATGGTAGAACTTACTACAATCCTTCCTTCAAGAGCCGCA
CCTCCATCTCCAGAGACACCTCCAGGAACCAGTTCTCCCTGCAGCTGAGCTACGTGACCGCTGAGGACGCGGCCGTG
TATTACTGTGCAGGAACTAACTACGGTTATGGTTATGCTCTGGCACTACCCCTCACGATGTACTATTTTGGCTACTG
GGGCCAGGGCACCCTGGTCACCGTCTCCTCAGAGAGTACGATGACCCCAGATCTCTTCCCCCTCGTCTCCTGTGGGC
CCTCTCTTGATGAGAGCCTGGTGGCTGTGGGCTGCCTAGCCCGGGACTTCCTACCCAA
>KY437611.1 Equus caballus clone 455 immunoglobulin mu heavy chain (IGHM) mRNA, partial cds
CTCTCCCTCACCTGCACTGTCTCTGGATTATCTTTGAGCAGTAATGCTGTAGGCTGGGTCCGCCAGGCTCCAGGAAA
AGGGCTGGAATTCGTTGGTGGTATATATGGTAGTGCAAGTGCAAACTACAACCCAGCCCTGAAGTCCCGAGCCAGCA
CCACCAAGGACACCTCAAAGAGCCAAGTTTATCTGACGCTGAACAGCCTGACAAGCGAGGACACGGCCGTCTATTAC
TGTGCAGGAGGCCTCCCCCGCACGACTTACTATGGTAGTTACCGCAGTAGTTACTATGGTATAGACTACTGGGGCCA
GGGCACCCTGGTCACCGTCTCCTCAGAGAGTACGAAGACCCCAGATCTCTTCCCCCTCGTCTCCTGTGGGCCCTCTC
TTGATGAGAGACTGGTGGCTGTGGGCTGCCTAGCCCGGGACTTCCTACCCAA
>KY437610.1 Equus caballus clone 454 immunoglobulin mu heavy chain (IGHM) mRNA, partial cds
CTCTCCCTCACCTGCACTGTCTCTGGATTCTCTTTGAGCAGTTACACCGTAGGCTGGGTCCGCCAGGCTCCAGGAAA
AGGGCTGGAATATGTTGGTGGTATAGCGAGTAGTGGAAGTGCAAACTACAACCCAGCCCTGAAGTCCCGAGTCAGCA
TCACCATGGACACCTCAAAGAGCAGAGTTTATCTGACGCTGAACGGCCTGACAGGCGAGGACACGGCCGTCTATTAC
TGTGCGAGATTAGGGAGTTCCTGGTACTCTACCCCTGTCTACTGGGGCCAGGGCACCCTGGTCACCGTCTCCTCAGA
GAGTACGAAGACCCCAGATCTCTTCCCCCTCGTCTCCTGTGGGCCCTCTCTTGATGAGAGCCTGGTGGCTGTGGGCT
GCCTAGCCCGGGACTTCCTACCCAA
>KY437609.1 Equus caballus clone 453 immunoglobulin mu heavy chain (IGHM) mRNA, partial cds

CTCTCCCTCACCTGCACTGTCTCTGGATTCTCTTTGAGCAGTTATGCTGTATACTGGGTCCGCCAGGCTCCAGGAAA
AGGGCTGGAATATGTTGGTGCTATAGCTAGTAGTGGAAGTGCAAACTACAACCCAGCCCTGAAGTCCCGAGCCAGCA
TCACCAAGGACACCTCCAAGAGCCAAGTTTATCTGACGCTGAACAGCCTGACAGGCGAGGACACGGCCGTCTATTAC
TGTGCGAAAGGGGATTGGAGATATGGTTATGGTGGTGCTTACTACGATTATGGTATAAACTACTGGGGCCAGGGCAT
CCTGGTCACCGTCTCCTCAGAGAGTACGATGACCCCAGATCTCTTCCCCCTCGTCTCCTGTGGGCCCTCTCTTGATG
AGAGCCTGGTGGCTGTGGGCTGCCTAGCCCGGGACTTCCTACCCAA
>KY437608.1 Equus caballus clone 452 immunoglobulin mu heavy chain (IGHM) mRNA, partial cds
CTCTCCCTCACCTGCACTGTCTCTGGATTATCTTTGAGCAGTAATCGTGTAGGCTGGGTCCGCCAGGCTCCAGGAAA
AGGGCTGGAATACGTTGGTGCTATATATTTTAGTGCAAGTGCAAACTACAACCCAGCCCTGAAGTCCCGAGCCAGCA
TCACCAAGGACACCTCAAAGAGCCAAGTTTATCTGACGCTGAACAGCCTGACAAGCGAGGACACGGCCGTCTATTAC
TGTACAGGAGGTCCTTTTTATGGAAATTACTATGAAGGAGGCTATTTTACCTACTGGGGCCAGGGCACCCTGGTCAC
CGTCTCCTCAGAGAGTACGAAGACCCCAGATCTCTTCCCCCTCGTCTCCTGTGGGCCCTCTCTTGATGAGAGCCTGG
TGGCTGTGGGCTGCCTAGCCCGGGACTTCCTACCCAA
>KY437607.1 Equus caballus clone 451 immunoglobulin mu heavy chain (IGHM) mRNA, partial cds
CTCTCCCTCACCTGCACTGTCTCTGGATTCTCTTTGAGCAGTTACGGTGTAGGCTGGGTCCGCCAGGCTCCAGGAAA
AGGGCTGGAATATGTTGGTGGTATAGCTAATAGTGGAAGTGCAAACTACAACCCAGCCCTAAAGTCCCGAGCCAGCG
TCACCAAGGACACCTCAAAGAGCCAAGTTTATCTGACGCTGAACAGCCTGACAAGCGAGGACACGGCCGTCTATTAC
TGTGCAGGAGGTCCATATGGTTACTATGGTAGTTACTACAGTAGTTACTATAGGGATCATTATGGCTACGTGGATCA
CTGGGGCCAGGGCACCCTGGTCACCGTCTCCTCAGAGAGTACGATGACCCCAGATCTCTTCCCCCTCGTCTCCTGTG
GGCCCTCTCTTGATGAGAGCCTGGTGGCTGTGGGCTGCCTAGCCCGGGACTTCCTACCCAA
>KY437606.1 Equus caballus clone 450 immunoglobulin mu heavy chain (IGHM) mRNA, partial cds
CTCTCCCTCACCTGCACTGTCTCTGGATTATCTTTGAGCAGTAATGCTGTAGGCTGGGTCCGCCAGGCTCCAGGAAA
AGGGCTGCAATACGTTGGTGCTATATATGGTAGTGCAAGTGCAAACTACAGCCCAGCCCTGAAGTCCCGAGCCAGCA
TCACCAAGGACACCTCAAAGAGCCAAGTTTATCTGACGCTGAACAGCCTGACAAGCGAGGACACGGCCGTCTATTAC
TGTGCAGGAGGCATACCTGAATATAGTTCCTATGCTGGTACATACGGAGTCCTTTATGGAATAAAATACTGGGGCCA
GGGCATCCTGGTCACCGTCTCCTCAGAGAGTACGAAGACCCCAGATCTCTTCCCCCTCGTCTCCTGTGGGCCCTCTC
TTGATGAGAGCCTGGTGGCTGTGGGCTGCCTAGCCCGGGACTTCCTACCCAA
>KY437605.1 Equus caballus clone 449 immunoglobulin mu heavy chain (IGHM) mRNA, partial cds
CTTTCCCTCACCTGCACTGTCTCTGGATTATCTTTGAGCAGTAATGCTGTAGGCTGGGTCCGCCAGGCTCCAGGAAA
AGGGCTGGAGTGGGTTGGTGTTATATATGGTAGTGAAAGTACATACTACAACCCAGCCCTGAAGTCCCGGGCCAGCA
TCACCAAGGACACCTCAAAGAGCCAAGTTTATCTGACGCTGAACAGCCTGACAGGCAAAGACACGGCCGTCTATTAC
TGTGCAGGATACGGCAGCTATGCTTACTACGAAGGGGTGAGCTACTGGGGCCAGGGCACCCTGGTCACCGTCTCCTC
AGAGAGTACGAAGACCCCAGATCTCTTCCCCCTCGTCTCCTGTGGGCCCTCTCTTGATGAGAGCCTGGTGGCTGTGG
GCTGCCTAGCCCGGGACTTCCTACCCAA
>KY437604.1 Equus caballus clone 448 immunoglobulin mu heavy chain (IGHM) mRNA, partial cds
CTCTCCCTCACCTGCACTGTCTCTGGATTATCTTTGAGCAGTAATTATGTAGGCTGGGTCCGCCAGGCTCCAGGAAA
AGGGCTGGAATGGGTTGGATCCATAGGTAGTAGTGGAGGCACGTGGTACAGCCCAGCCCTGAGGTCCCGAGCCAGCA
TCACCAAGGACACCTCAAAGAGCCAAATTAGTCTGACGCTGAACAGCCTGACAAGCGAGGACACGGCCGTCTATTAC
TGTATAGGAGGGGACTACTGGGGCCAGGGCATCCTGGTCACCGTCTCCGCAGAGAGTACGATGACCCCAGATCTCTT
CCCCCTCGTCTCCTGTGGGCCCTCTCTTGATGAGAGCCTGGTGGCTGTGGGCTGCCTAGCCCGGGACTTCCTACCCA
A
>KY437603.1 Equus caballus clone 447 immunoglobulin mu heavy chain (IGHM) mRNA, partial cds
CTGTCCCTCACCTGCACTGTCTCTGGATTATCTTTGAGCAGTTATCCTGTAGGCTGGGTCCGCCAGACTCCAGGAAA
AGGGCTGGAATATGTTGGTGATATATATGGTAGTGGACGTGCGTACTACAACCCAGCCCTGAGGTCCCGAGCCAGCA
TCACCAAGGACACCTCAAAGAGCCAAGTTTATCTGACGCTGAACAGCCTGACAGGCGAGGACACGGCCGTCTATTAC
TGTGCGTCCGCATTGACAAATTTGGCTGATGGTGTGCAGTACTGGGGCCCGGGCATCCTGGTCACCGTCTCCTCAGA
GAGTACGATGACCCCAGATCTCTTCCCCCTCGTCTCCTGTGGGCCCTCTCTTGATGAGAGCCTGGTGGCTGTGGGCT
GCCTAGCCCGGGACTTCCTACCCAA
>KY437602.1 Equus caballus clone 446 immunoglobulin mu heavy chain (IGHM) mRNA, partial cds

CTCTCCCTCACCTGCACTGTCTCTGGATTATCTTTGAGCAGGAATGATATAACGTGGGTCCGCCAGGCTCCAGGAAA
AGGGCTGGAATACGTTGCTGAAATACAAGCCAGTGCAAGTGCATTGTACAACCCGGCCCTGAAGTCCCGAGCCAGCA
TCACCAAGGACACCTCAAAGAGCCAAGTTTATCTGACGCTGAACAGCCTGACAAGCGAGGACACGGCCGTCTATTAC
TGTATAGGAGGTCTAATTGAGTTTTGGGGCCAGGGCACCCTGGTCACCGTCTCCTCAGAGAGTACGAAGACCCCAGA
TCTCTTCCCCCTCGTCTCCTGTGGGCCCTCTCTTGATGAGAGCCTGGTGGCTGTGGGCTGCCTAGCCCGGGACTTCC
TACCCAA
>KY437601.1 Equus caballus clone 445 immunoglobulin mu heavy chain (IGHM) mRNA, partial cds
CTCTCCCTCACCTGCACTGCCTCTGGATTATCTTTGAGCAGGAATAATGTAGGCTGGGTCCGCCAGGTTTCAGGAAA
AGGGCTGGAATATGTTGGTGGTATAGCTACTAGTGGAAGTGCAAACTACAACCCAGCCCTGAAGTCCCGAGCCAGCA
TCACCAAGGACACCTCAAAGAGCCAAGTTGATCTGACGCTGAACAGCCTGACAAGCGAGGACACGGCCGTCTATTAC
TGTGCAGGCCAGACCGAATACTATGGTTATGGTAGTGCTTACTACTGGGATATGTACTACTGGGGCCAGGGCATCCT
GGTCACCGTCTCCTCAGAGAGTACGAAGACCCCAGATCTCTTCCCCCTCGTCTCCTGTGGGCCCTCTCTTGATGAGA
GCCTGGTGGCTGTGGGCTGCCTAGCCCGGGACTTCCTACCCAA
>KY437600.1 Equus caballus clone 444 immunoglobulin mu heavy chain (IGHM) mRNA, partial cds
CTCTCCCTCACCTGCACTGTCTCTGGATTATCTTTGAGCAGTAATGCTGTAGGCTGGGTCCGCCAGGCTCCAGGAAA
AGGGCTGGAATATGTTGGTGGTATAGGTAGTAGTGGAAGTGCAAACTACAACCCAGCCCTGAAGTCCCGAGCCAGCA
TCACCAAGGACACCTCAAAGAGCCAAGTTTATCTGACGCTGAACAGCCTGACAAGCGAGGACACGGCCGTCTATTAC
TGTACAGGAGGCCTACTTTATGGTTACTATGCTAGTGGTTACTACTGGGGCCAGGGCACCCTGGTCACCGTCTCCTC
AGAGAGTACGATGACCCCAGATCTCTTCCCCCTCGTCTCCTGTGGGCCCTCTCTTGATGAGAGCCTGGTGGCTGTGG
GCTGCCTAGCCCGGGACTTCCTACCCAA
>KY437599.1 Equus caballus clone 443 immunoglobulin mu heavy chain (IGHM) mRNA, partial cds
CTCTCCCTCACCTGCACTGTCTCTGGATTATCTTTGAGCAGTAATGCTGTAGGCTGGGTCCGCCAGGCTCCAGGAAA
AGGGCTGGAATATGTTGGTGGTATAGCTAGTAGTGGAAGTGCAAACTACAACCCAGCCCTGAAGTCCCGAGCCAGCA
TCACCAAGGACACCTCAAAGAGCCAAGTTTATCTGACGCTGAACAGCCTGACAAGCGAGGACACGGCCGTCTATTAC
TGTGCAGTCTACGGTTATGGTTATGTCTTTAGCTACTGGGGCCAGGGTACCCCGGTCACCGTCTCCTCAGAGAGTAC
GAAGACCCCAGATCTCTTCCCCCTCGTCTCCTGTGGGCCCTCTCTTGATGAGAGCCTGGTGGCTGTGGGCTGCCTAG
CCCGGGACTTCCTACCCAA
>KY437598.1 Equus caballus clone 442 immunoglobulin mu heavy chain (IGHM) mRNA, partial cds
CTCTCCCTCACCTGCACCGTCTCTGGATTCTCTTTGAGCAGTTATGCTGTAGGCTGGGTCCGCCAGGCTCCAGGAAA
AGGGCTGGAATATGTTGGTGCTATATATGGTAGTACAAGTGCAAACTACAACCCAGCCCTGAAGTCCCGAGCCAGCA
TCACCAAGGACACCTCCAAGAGCCAAGTTTATCTGACGCTGAACAGCCTGACAGGCGAGGACACGGCCGTCTATTAC
TGTGCAGGAGGCTGGCCTAACTACTGGGGCCAGGGCACCCTGGTCACCGTCTCCTCAGAGAGTACGAAGACCCCAGA
TCTCTTCCCCCTCGTCTCCTGTGGGCCCTCTCTTGATGAGAGCCTGGTGGCTGTGGGCTGCCTAGCCCGGGACTTCC
TACCCAA
>KY437597.1 Equus caballus clone 441 immunoglobulin mu heavy chain (IGHM) mRNA, partial cds
CTGTCCCTCACCTGCACTGTCTCTGGATTATCTTTGAGCAGTAATGCTGTAGGCTGGGTCCGCCAGGCTCCAGGAAA
AGGGCTGGAGTGGGTTGGTGCTATATATGGTAGTGAAAGTACATACTACAACCCAGCCCTGAAGTCCCGAGCCAGCA
TCACCAAGGACACCTCAAAGAGCCAAGTTTATCTGACGCTGAACAGCCTGACAGGCGAAGACACGGCCGTCTATTAC
TGTGCAGGAGGTAACAGATACTGGGGTGGTAGTTCCTGGTTGTGGAACTATTTTGGCTACTGGGGCCAGGGCACCCT
GGTCACCGTCTCCTCAGAGAGTACGATGACCCCAGATCTCTTCCCCCTCGTCTCCTGCGGGCCCTCTCTTGATGAGA
GCCTGGTGGCTGTGGGCTGCCTAGCCCGGGACTTCCTACCCAA
>KY437596.1 Equus caballus clone 440 immunoglobulin mu heavy chain (IGHM) mRNA, partial cds
CTCTCCCTCACCTGCACTGTCTCTGGATTATCTTTGAGCAGTAGCTCTGTAGGCTGGGTCCGCCAGGCTCCAGGAAA
AGGGCTGGAATATGTTGGTGGTATAGCTAGTAGTGGAAGTACAAACTACAACCCAGCCCTGAAGTCCCGAGCCAGCA
TCACCAAGGACACCTCAAAGAGCCAAGTTTATCTGACGCTGAACAGCCTGACAAGCGAGGACACGGCCGTCTATTAC
TGTGCAGGAGCTCCCCTCCTCTATGCTAGTGTTTATTATGGTGTAGAATACTGGGGCCAGGGCATCCTGGTCACCGT
CTCCTCAGAGAGTACGATGACCCCAGATCTCTTCCCCCTCGTCTCCTGTGGGCCCTCTCTTGATGAGAGCCTGGTGG
CTGTGGGCTGCCTAGCCCGGGACTTCCTACCCAA

>KY437595.1 Equus caballus clone 439 immunoglobulin mu heavy chain (IGHM) mRNA, partial cds
CTCTCCCTCACCTGCACTGTCTCTGGATTATCTTTGAGCAGTTATGCTGTAGGCTGGGTCCGCCAGGCTCCAGGAAA
AGGGCTGGAATATGTTGGTGCTATATATGGTAGTGCAAGTGCAAACTACAACCCAGCCCTGAAGTCCCGAGCCAGCA
TCACCAAGGACACCTCAAAGAGCCAAGTTTATCTGACGCTGAACAGCCTGACAAGCGAGGACACGGCCGTCTATTAC
TGTGCAGGAGGGTACGGTTCCTATTGGTTATACTACATTCAACTTAGCTACTGGGGCCAGGGCACCCTGGTCACCGT
CTCCTCAGAGAGTACGAAGACCCCAGATCTCTTCCCCCTCGTCTCCTGTGGGCCCTCTCTTGATGAGAGCCTGGTGG
CTGTGGGCTGCCTAGCCCGGGACTTCCTACCCAA
>KY437594.1 Equus caballus clone 438 immunoglobulin mu heavy chain (IGHM) mRNA, partial cds
CTCTCCCTCACCTGCACTGTCTCTGGATTATCTTTGAGCAGTAATGCTGTAGGCTGGGTCCGCCAGGCTCCAGGAAA
AGGGCTGGAATACGTTGGTAATATATATGGTAGTGCAAGTGCAAACTACAACCCAGCCCTGAAGTCCCGAGCCAGCA
TCACCAAGGACACCTCAAAGAGCCAAGTTTATCTGACGCTGAACAGCCTGACAAGCGAGGACACGGCCGTCTATTAC
TGTGCAGCAGCAGTAAACGGTTATGGTTTGACACTCTTATACGGAGCTGGTATAAGCTACTGGGGCCAGGGCATCCT
GGTCACCGTCTCCTCAGAGAGTACGAAGACCCCAGATCTCTTCCCCCTCGTCTCCTGTGGGCCCTCTCTTGATGAGA
GCCTGGTGGCTGTGGGCTGCCTAGCCCGGGACTTCCTACCCAA
>KY437593.1 Equus caballus clone 437 immunoglobulin mu heavy chain (IGHM) mRNA, partial cds
CTCTCCCTCACCTGCACTGTCTCTGGATTATCTTTGAGCAGTTATGCTGTAGGCTGGGTCCGCCAGGCTCCAGGAAA
AGGGCTGGAATATGTTGGTACTATATACGGTAGTGCAAGTGCAAACTACAAGCCAGCCCTGAAGTCCCGAGCCAGCA
TCACAAAGGACACCTCAAAGAGCCAAGTTTATCTGACGCTGAACACCCTGACAGGCGAGGACACGGCCGTCTATTAC
TGTGCGAGAATCCCCTATTACTATGCTACAGGTGGTATAGACTACTGGGGCCAGGGCATCCTGGTCACCGTCTCCTC
AGAGAGTACGAAGACCCCAGATCTCTTCCCCCTCGTCTCCTGTGGGCCCTCTCTTGATGAGAGCCTGGTGGCTGTGG
GCTGCCTAGCCCGGGACTTCCTACCCAA
>KY437592.1 Equus caballus clone 436 immunoglobulin mu heavy chain (IGHM) mRNA, partial cds
CTCTCCCTCACCTGCACTGTCTCTGGATTATCTTTGAGCAGTAATGCTGTAGGCTGGGTCCGCCAGGCTCCAGGAAA
AGGGCTGGAATACGTTGGTGTTATATATTTCAGTACAAGTGCAAACTACAACCCAGCCCTGAAGTCCCGAGCCAGCA
TCACCAAGGACACCTCAAAGAGTCAAGTTTATCTGACGCTGAACAGCCTGACAAGCGAGGACACGGCCGTCTATTAC
TGTGCATGTTCGGAAACATACTATGTCGGAGATTTTAGCTACTGGGGCCAGGGCACCCTGGTCACCGTCTCCTCAGA
GAGTACGAAGACCCCAGATCTCTTCCCCCTCGTCTCCTGTGGGCCCTCTCTTGATGAGGGCCTGGTGGCTGTGGGCT
GCCTAGCCCGGGACTTCCTACCCAA
>KY437591.1 Equus caballus clone 435 immunoglobulin mu heavy chain (IGHM) mRNA, partial cds
CTGTCCCTCACCTGCACTGTCTCTGGATTATCTTTGAACAGTGATCTTGTAGGCTGGGTCCGCCAGGCTCCAGGAAA
AGGGCTGGAGTGGGTTGGTGATATACGTGCTGGTGAAAGTCCGTACTACAACCCAGCCCTGAAGTCCCGAGCCAGCA
TCACCAAGGACACCTCAAAGAGCCAAGTTTATCTGACGCTGAACAGCCTGACAGGCGAAGACACGGCCGTCTATTAC
TGTACAGGAGCGGGAAAGGAAGACTATGCTTTAACCTTTGCCTATTGGGGCCAGGGCACCCTGGTCACCGTCTCCTC
AGAGAGTACGATGACCCCAGATCTCTTCCCCCTCGTCTCCTGTGGGCCCTCTCTTGATGAGAGCCTGGTGGCTGTGG
GCTGCCTAGCCCGGGACTTCCTACCCAA
>KY437590.1 Equus caballus clone 434 immunoglobulin mu heavy chain (IGHM) mRNA, partial cds
CTCTCCCTCACCTGCACTGTCTCTGGATTATCTTTGAGCAGTAACGCTGTAGGCTGGGTCCGCCAGGCTCCAGGAAA
AGGGCTGGAATATGTTGGTGGTATAGCTAGTAGTGGAAGTGCAAACTACAACCCAGCCCTGAAGTCCCGAGCCAGCA
TCACCAAGGACACCGCAAAGAGCCAAGTTTATCTGACGCTGAACAGCCTGACAGGCGAAGACACGGCCGTCTATTAC
TGTGCGAGACATATTCGGTACTATGGTTATGGTATGGCTTACTACTACCACGATATAGACTATTGGGGCCAGGGCAT
CCTGGTCACCGTCTCCTCAGAGAGTACGAAGACCCCAGATCTCTTCCCCCTCGTCTCCTGTGGGCCCTCTCTTGATG
AGAGCCTGGTGGCTGTGGGCTGCCTAGCCCGGGACTTCCTACCCAA
>KY437589.1 Equus caballus clone 433 immunoglobulin mu heavy chain (IGHM) mRNA, partial cds
CTCTCCCTCACCTGCACTGTCTCTGGATTATCTTTGAGCAGTAATGCTGTAGGCTGGGTCCGCCAGGCTCCAGGAAA
AGGGCTGGAATACGTTGGTGCTATATATGATAGTGCAAGTGCAAACTACAACCCAGCCCTGAAGTCCCGAGCCAGCA
TCACCAAGGACACCTCAAAGAGCCAAGTTTATCTGACGCTGAACAGCCTGACAAGTGAGGACACGGCCGTCTATTAC
TGTGTAGGAGGAGGACTCTACTGGGGCCAGGGCATCCTGGTCACCGTCTCCTCAGAGAGTACGATGACCCCAGATCT
CTTCCCCCTCGTCTCCTGTGGGCCCTCTCTTGATGAGAGCCTGGTGGCTGTGGGCTGCCTAGCCCGGGACTTCCTAC
CCAA

>KY437588.1 Equus caballus clone 432 immunoglobulin mu heavy chain (IGHM) mRNA, partial cds
CTCTCCCTCACCTGCACTGTCTCTGGATTATCTTTGAGCAGTAATGCTGTAGGCTGGGTCCGCCAGGCTCCAGGAAA
AGGGCTGGAATACGTTGGTACTATATATGGTAGTGCAGGTGCAAACTACAACCCAGCCCTGAAGTCCCGAGCCAGCA
CCACCAAGGACACCTCAAAGAGCCAAGTTTATCTGACGCTGAACAGCCTGACAAGCGAGGACACGGCCGTCTATTAC
TGTGCAGGAGGGTCCACCGGTTACTATGGTAGTGGTTATGACTACACCCGATATAATCAAGACTACTGGGGCCAGGG
CATCCTGGTCACCGTCTCCTCAGAGAGTACGAAGACCCCAGATCTCTTCCCCCTCGTCTCCTGTGGGCCCTCTCTTG
ATGAGAGCCTGGTGGCTGTGGGCTGCCTAGCCCGGGACTTCCTACCCAA
>KY437587.1 Equus caballus clone 431 immunoglobulin mu heavy chain (IGHM) mRNA, partial cds
CTCTCCCTCACCTGCACTGTCTCTGGATTATCTTTGAGCAGTAATGCTGTAGGCTGGGTCCGCCAGGCTCCAGGAAA
AGGGCTGGAATACGTTGCTAATATAGCTGGTAGTGCAAGTGCAAGCTACAACCCAGCCCTGAAGTCCCGAGCCAGCA
TCACCAAGGACACCTCAAAGAGCCAAGTTTATCTGACGCTGAACAGCCTGACAAGCGAGGACACGGCCGTCTATTAC
TGTGGGAGATATTATTATGGTATAAACTACTGGGGCCAGGGCATCCTGGTCACCGTCTCCTCAGAGAGTACGATGAC
CCCAGATCTCTTCCCCCTCGTCTCCTGTGGGCCCTCTCTTGATGAGAGCCTGGTGGCTGTGGGCTGCCTAGCCCGGG
ACTTCCTACCCAA
>KY437586.1 Equus caballus clone 430 immunoglobulin mu heavy chain (IGHM) mRNA, partial cds
CTCTCCCTCACCTGCACTGTCTCTGGATTATCTTTGAGCAGTAATGCTGTAGGCTGGGTCCGCCAGGCTCCAGGAAA
AGGGCTGGAATATGTTGGTGGTATAGCTAGTAGTGGAAGTGCAAACTACAACCCAGCCCTGAAGTCCCGAGCCAGCA
TCACCAAGGACACCTCCAAGAGCCAAGTTTATCTGACGCTGAACAGCCTGACAGGCGAGGACACGGCCGTCTATTAC
TGTGCGAAAGATGGCGCATATGGTTCCTATGCTGGTAATTTTTATTATGGTATAGACTACTGGGGCCAGGGCATCCT
GGTCACCGTCTCCTCAGAGAGTACGAAGACCCCAGATCTCTTCCCCCTCGTCTCCTGTGGGCCCTCTCTTGATGAGA
GCCTGGTGGCTGTGGGCTGCCTAGCCCGGGACTTCCTACCCAA
>KY437585.1 Equus caballus clone 429 immunoglobulin mu heavy chain (IGHM) mRNA, partial cds
CTCTCCCTCACCTGCACTGTCTCTGGATTATCTTTGAGCAGTAATGCTGTAGGCTGGGTCCGCCAGGCTCCAGGAAA
AGGGCTGGAATATGTTGGTGCTATATATGGTAGTGCAAGTGCAAACTACAACCCAGCCCTGAAGTCCCGAGCCAGCA
TCACCAAGGACACCTCAAAGAGCCAAGTTTATCTGACGCTGAGCAGCCTGACAAGCGAGGACACGGCCGTCTATTAC
TGTGCAGGAGGGCGAAGTGGTGTTGCACTGATAGACTACTGGGGCCAGGGCATCCTGGTCACCGTCTCCTCAGAGAG
TACGAAGACCCCAGATCTCTTCCCCCTCGTCTCCTGTGGGCCCTCTCTTGATGAGAGCCTGGTGGCTGTGGGCTGCC
TAGCCCGGGACTTCCTACCCAA
>KY437584.1 Equus caballus clone 428 immunoglobulin mu heavy chain (IGHM) mRNA, partial cds
CTCTCCCTCACCTGCACTGTCTCTGGATTCTCTTTGAGCAGTTATGCTGTAGGCTGGGTCCGCCAGGCTCCAGGAAA
AGGGCTGGAATATGTTGGTTATATATATGGTAGTGGAAGTGCAAACTACAACCCAGCCCTGAAGTCCCGAGCCAGCA
TCACCAAGGACACCTCAAAGAGCCAGATTTATCTGACGCTGAACAGCCTGACAAGCGAGGACACGGCCGTCTATTAC
TGTGCAGGAGGCCCCTATGCTGGTAGTTACTATTGGAATTATTATAGAGTAGACTACTGGGGTCAGGGCATCCTGGT
CACCGTCTCCTCAGAGAGTACGAAGACCCCAGATCTCTTCCCCCTCGTCTCCTGTGGGCCCTCTCTTGATGAGAGCC
TGGTGGCTGTGGGCTGCCTAGCCCGGGACTTCCTACCCAA
>KY437583.1 Equus caballus clone 427 immunoglobulin mu heavy chain (IGHM) mRNA, partial cds
CTGTCCCTCACCTGCACTGTCTCTGGATTATCTTTGAGCAGTAATGCTGTAGGCTGGGTCCGCCAGGCTCCAGGAAA
AGGGCTGGAGTGGGTTGGTGTTATATATGGTAGTGAAAGTACATACTACAACCCAGCCCTGAAGTCCCGAGTCAGCA
TCACCAAGGACACCTCAAAGAGCCAAGTTTATCTGACGCTGAACAGCCTGACAAGCGAGGACACGGCCGTCTATTAC
TGTGCAGGACAGGGTAGTTACTATGCGTGGTGGTCTTGGGGCCAGGGCATCCTGGTCACCGTCTCCTCAGAGAGTAC
GAAGACCCCAGATCTCTTCCCCCTCGTCTCCTGTGGGCCCTCTCTTGATGAGAGCCTGGTGGCTGTGGGCTGCCTAG
CCCGGGACTTCCTACCCAA
>KY437582.1 Equus caballus clone 426 immunoglobulin mu heavy chain (IGHM) mRNA, partial cds
CTCTCCCTCACCTGCACTGTCTCTGGATTATCTTTGAGCAGTAATGCTGTAGGCTGGGTCCGCCAGGCTCCAGGAAA
AGGGCTGGAATATGTTGGTGGTATAGCTAGTAGTGGAAGTGCAAACTACAACCCAGCCCTGAAGTCCCGAGCCAGCA
TCACCAAGGACACCTCAAAGAGCCAAATTTATCTGACGCTGAACAGCCTGACAAGCGAGGACACGGCCGTCTATTAC
TGTGCAGGAGGCCCGGAGGGTTACTGGGGCCAGGGCATCCTGGTCACCGTCTCCTCAGAGAGTACGATGACCCCAGA

TCTCTTCCCCCTCGTCTCCTGTGGGCCCTCTCTTGATGAGAGCCTGGTGGCTGTGGGCTGCCTAGCCCGGGACTTCC
TACCCAA
>KY437581.1 Equus caballus clone 425 immunoglobulin mu heavy chain (IGHM)
mRNA, partial cds
CTCTCCCTCACCTGCACTGTCTCTGGATTATCTTTGAGCAGTAATGCTGTAGGCTGGGTCCGCCAGGCTCCAGGAAA
AGGGCTGGAATATGTTGGTGGTATAGCTAGTAGTGGAAGTGCAAACTACAACCCAGCCCTGAAGTCCCGAGCCAGCA
TCACCAAGGACACCTCAAAGAGCCAAGTTTATCTGACGCTGAACAGCCTGACAAGCGAGGACACGGCCGTCTATTAC
TGTGCAGGAGAACCTAATGGTTATGGTGGTATTTACTATCCTTTTCGCTACTGGGGCCAGGGCACCCTGGTCACCGT
CTCCTCAGAGAGTACGATGACCCCAGATCTCTTCCCCCTCGTCTCCTGTGGGCCCTCTCTTGATGAGAGCCTGGTGG
CTGTGGGCTGCCTAGCCCGGGACTTCCTACCCAA
>KY437580.1 Equus caballus clone 424 immunoglobulin mu heavy chain (IGHM)
mRNA, partial cds
CTCTCCCTCACCTGCACTGTCTCTGGATTATCTTTGAGCAGTAATGCTGTAGGCTGGGTCCGCCAGGCTCCAGGAAA
AGGGCTGGAATCTGTTGGTGCTATATATGGTAGTGCAAGTGCAAACTACAACCCAGCCCTGAAGTCCCGAGCCAGCA
TCACCAAGGACACCTCAAAGAGCCGAGTTTATCTGACGCTGAACAGCCTGACAAGCGAGGACACGGCCGTCTATTAC
TGTGCAGGAGACCTATATGGTTCCTATGCTGGTAGTTGGTATGCGATCCCGTTTAGCTACTGGGGCCAGGGCACCCT
GGTCACCGTCTCCTCAGAGAGTACGAAGACCCCAGATCTCTTCCCCCTCGTCTCCTGTGGGCCCTCTCTTGATGAGA
GCCTGGTGGCTGTGGGCTGCCTAGCCCGGGACTTCCTACCCAA
>KY437579.1 Equus caballus clone 423 immunoglobulin mu heavy chain (IGHM)
mRNA, partial cds
CTCTCCCTCACCTGCACTGTCTCTGGATTATCTTTGAACGGTAATGCTGTAGGCTGGGTCCGCCAGGCTCCAGGAAA
AGGGCTGGAATATGTTGGTGGTATAGCTAAAAGTGGAAGTGCAAACTACAACCCAGCCCTGAAGTCCCGAGCCAGCA
TCACCAAGGACACCTCAAAGAGCCAAGTTTATCTGACGCTGAACAGCCTGACAAGCGAGGACACGGCCGTCTATTAC
TGTGCAGGAGGAAATCCGGAAGATACTTGGAATAATCACTATAGTAGTTACTCGTGGGGCCAGGGCACCCTGGTCAC
CGTCTCCTCAGAGAGTACGAAGACCCCAGATCTCTTCCCCCTCGTCTCCTGTGGGCCCTCTCTTGATGAGAGCCTGG
TGGCTGTGGGCTGCCTAGCCCGGGACTTCCTACCCAA
>KY437578.1 Equus caballus clone 422 immunoglobulin mu heavy chain (IGHM)
mRNA, partial cds
CTGTCCCTCACCTGCACTGTCTCTGGATTCTCTTTGAGCAGTTACGGTGTAGGCTGGGTCCGCCAGGCTCCAGGAAA
AGGGCTGGAATTTGTTGGTGGTATAGCTAGTAGTGGAAGTGCAAACTACAACCCAGCCCTGAAGTCCCGAGCCAGCA
TCACCAAGGACACCTCAAAGAGCCAAGTTTATCTGACGCTGAACAACCTGACAGGCGAGGACACGGCCGTCTATTAC
TGTGCGAGAGCCACCGGGGGGGCAGCTATGCTTTATGGGGCTTCTGGCTACTGGGGCCAGGGCACCCTGGTCACCGT
CTCCTCAGAGAGTACGAAGACCCCAGATCTCTTCCCCCTCGTCTCCTGTGGGCCCTCTCTTGATGAGAGCCTGGTGG
CTGTGGGCTGCCTAGCCCGGGACTTCCTACCCAA
>KY437577.1 Equus caballus clone 421 immunoglobulin mu heavy chain (IGHM)
mRNA, partial cds
CTCTCCCTCACCTGCACTGTCTCTGGATTATCTTTGAGCAGTTATGGTGTGGGCTGGGTCCGCCAGGCTCCAGGAAA
AGGGCTGGAATTTGTTGGTGGTATAGCTAGTAGTGGAAGTGCAAACTACAACCCAGCCCTGAAGTCCCGAGCCAGCA
TCACCAAGGACACCTCAAAGAGCCAAGTTTATCTGACGCTGAACAGCCTGACAAGCGAGGACACGGCCGTCTATTAC
TGTGCACTATCCGGCGGGGGTTATGGTAGCAATGGTGCTTACTACCTCTCCACGTTTGGCTACTGGGGCCAGGGCAC
CCTGGTCACCGTCTCCTCAGAGAGTACGAAGACCCCAGATCTCTTCCCCCTCGTCTCCTGTGGGCCCTCTCTTGATG
AGAGCCTGGTGGCTGTGGGCTGCCTAGCCCGGGACTTCCTACCCAA
>KY437576.1 Equus caballus clone 420 immunoglobulin mu heavy chain (IGHM)
mRNA, partial cds
CTCTTTCTCACCTGCACCGTCTCTGGATTCTCTTTGAGCAGTTACGGTGTAGGCTGGGTCCGCCAGGCTCCAGGAAA
AGGGCTGGAATTTGTTGGTAATATAGGTAGTAGTGGAAGTACAAACTACAACCCAGCCCTGAAGTCCCGAGCCAGCA
TCACCAAGGACACCTCAAAGAGCCAAGTTTATCTGACGCTGAACAGCCTGACAAGCGAGGACACGGCCGTCTATTAC
TGTGCAGGAGGGCCAATATATGCGTACTATATTAGTGGTTTCGGTTATGGTATAGAGTACTGGGGCCAGGGCATCCT
GGTCACCGTCTCCTCAGAGAGTACGAAGACCCCAGATCTCTTCCCCCTCGTCTCCTGTGGGCCCTCTCTTGATGAGA
GCCTGGTGGCTGTGGGCTGCCTAGCCCGGGACTTCCTACCCAA
>KY437575.1 Equus caballus clone 419 immunoglobulin mu heavy chain (IGHM)
mRNA, partial cds
CTCTCCCTCACCTGCACTGTCTCTGGATTATCTTTGAGCAGTTATGGTGTGGGCTGGGTCCGCCAGGCTCCAGGAAA
AGGGCTGGAATTTGTTGGTAGTATACCTAGTAGTGGAAGTGCAAGCTACAACCCAGCCCTGAAGTCCCGAGCCAGCA
TCACCAAGGACACCTCAAAGAGCCAAGTTTATCTGACGCTGAACAGCCTGACAAGCGAGGACACGGCCGTCTATTAC
TGTGCAGGAGAGGGTTACAGAGGTAGTTACTACAGCTATTACTATGGACTGATGGGCCTTTTTGGCTACTGGGGCCA

GGGCACCCTGGTCACCGTCTCCTCAGAGAGTACGAAGACCCCAGATCTCTTCCCCCTCGTCTCCTGTGGGCCCTCTC
TTGATGAGAGCCTGGTGGCTGTGGGCTGCCTAGCCCGGGACTTCCTACCCAA
>KY437574.1 Equus caballus clone 418 immunoglobulin mu heavy chain (IGHM) mRNA, partial cds
CTGTCCCTCACCTGCACTGTCTCTGGATTCTCTTTGAACAGTAACGGTGTAGGCTGGGTCCGCCAGGCTCCAGGAAA
AGGGCTGGAATTTGTTGGTGATATAGGTAGTAGTGGAAGTGCAAACTACAACCCAGCCCTGAAGTCCCGAGCCAGCA
TCACCAAGGACACCTCAAAGAGCCAAGTTTATCTGACGCTGAACAGCCTGACAGGCGAGGACACGGCCGTCTATTAC
TGTGCGAGATGGCATTANTATGACTCAGGGTACTATTTTGGCTACTGGGGCCAGGGCACCCTGGTCACCGTCTCCTC
AGAGAGTACGAAGACCCCAGATCTCTTCCCCCTCGTCTCCTGTGGGCCCTCTCTTGATGAGAGCCTGGTGGCTGTGG
GCTGCCTAGCCCGGGACTTCCTACCCAA
>KY437573.1 Equus caballus clone 417 immunoglobulin mu heavy chain (IGHM) mRNA, partial cds
CTGTCCCTCACCTGCACTGTCTCTGGATTCTCTTTGAGCAGTTACGGTGTGGGCTGGGTCCGCCAGGCTCCAGGAAA
AGGGCTGGAATTTGTTGGTGGTATAGCTAGTAGTGGAAGTGCAAACTACAACCCAGCCCTGAAGTCCCGAGCCAGCA
TCACCAAGGACACCTCAAAGAGCCAAGTTTATCTGACGCTGAACAGCCTGACAAGCGAGGACACGGCCGTCTATTAC
TGTGCAGGAGGCGGGATATACGGGGACGGTTCCTATGCTGGTAGTTACTTATACTACGGCCGAGTCCGTCGTAATGG
TATAAACTACTGGGGCCAGGGCATCCTGGTCACCGTCTCCTCAGAGAGTACGAAGACCCCAGATCTCTTCCCCCTCG
TCTCCTGTGGGCCCTCTCTTGATGAGAGCCTGGTGGCTGTGGGCTGCCTAGCCCGGGACTTCCTACCCAA
>KY437572.1 Equus caballus clone 416 immunoglobulin mu heavy chain (IGHM) mRNA, partial cds
CTGTCCCTCACCTGCACTGTCTCTGGATTCTCTTTGAGCAGTTACGGTGTAGGCTGGGTCCGCCAGGCTCCAGGAAA
AGGGCTGGAATTTGTTGGTAATATACCTAGTAGTGCAAGTGCAAACTACAACCCAGCCCTGAAGTCCCGAGCCAGCA
TCACCAAGGACACCTCAAAGAGCCAAGTTTATCTGACGCTGAACAGCCTGACAAGCGAGGACACGGCCGTCTATTAC
TGTGCAGGAACTTGGAACACAGGTTATAGTTATGCTACGGCGAGCTACTGGGGCCAGGGCACCCTGGTCACCGTCTC
CTCAGAGAGTACGAAGACCCCAGATCTCTTCCCCCTCGTCTCCTGTGGGCCCTCTCTTGATGAGAGCCTGGTGGCTG
TGGGCTGCCTAGCCCGGGACTTCCTACCCAA
>KY437571.1 Equus caballus clone 415 immunoglobulin mu heavy chain (IGHM) mRNA, partial cds
CTCTCCCTCACCTGCACTGTCTCTGGATTATCTTTGAGCAGTAATGCTGTAGGCTGGGTCCGCCAGGCTCCAGGAAA
AGGGCTGGAATACGTTGGTACTATATATGGTAGTGCAAGTGCAAACTACAACCCAGCCCTGAAGTCCCGAGCCAGCA
TCACCAAGGACACCTCAAAGAGCCAAGTTTATCTGACGCTGAACAGCCTGACAAGCGAGGACACGGCCGTCTATTAC
TGTGCAGGAGGCCGAGTATCCTATAGTTATGGTAGTTACTATTACTTTCTTGTCATAGACTACTGGGGCCAGGGCAT
CCTGGTCACCGTCTCCTCAGAGAGTACGAAGACCCCAGATCTCTTCCCCCTCGTCTCCTGTGGGCCCTCTCTTGATG
AGAGCCTGGTGGCTGTGGGCTGCCTAGCCCGGGACTTCCTACCCAA
>KY437570.1 Equus caballus clone 414 immunoglobulin mu heavy chain (IGHM) mRNA, partial cds
CTCTCCCTCACCTGCACTGTCTCTGGAGTCTCTTTGAGGAGTTACGGTGTAGAATGGGTCCGCCAGGCTCCAGGAAA
AGGGCTGGAATTTGTCGGTAGTATAACTAATAGTGGAAGTGCAAACTACAACCCAGCCCTGAAGTCCCGAGCCAGCA
TCACCAAGGACACCTCAAAGAGCCAAGTTTATCTGACGCTGAACAGCCTGACAAGCGAGGACACGGCCGTCTATTAC
TGTGCAGGAGGCGGGATATACGGGGACGGTTCCTATGCTGGTAGTTACTTATACTACGGCCGAGTCCGTCGTAATGG
TATAAACTACTGGGGCCAGGGCATCCTGGTCACCGTCTCCTCAGAGAGTACNAAGACCCCAGATCTCTTCCCCCTCG
TCTCCTGTGGGCCCTCTCTTGATGAGAGCCTGGTGGCTGTGGGCTGCCTAGCCCGGGACTTCCTACCCAA
>KY437569.1 Equus caballus clone 413 immunoglobulin mu heavy chain (IGHM) mRNA, partial cds
CTGTCCCTCACCTGCACTGTCTCTGGATTCTCTTTGAGTAGTTACGGTGTAGGCTGGGTCCGCCAGGCTCCAGGAAA
AGGACTGGAATTTGTTGGTGGTGGCCCTAGTAGTGGAAGTGCAAACTACAACCCAGCCCTGAAGTCCCGAGCCAGCA
TCACCAAGGACACCTCAAAGAGCCAAGTTTATCTGACGCTGAACAGCCTGACAGGCGAGGACACGGCCGTCTATTAT
TGTGCGAGATACAATACTTATGGTAGTTACTATGCCTATGGTATAGACTACTGGGGCCAGGGCATCCTGGTCACCGT
CTCCTCAGAGAGTACNAAGACCCCAGATCTCTTCCCCCTCGTCTCCTGTGGGCCCTCTCTTGATGAGAGCCTGGTGG
CTGTGGGCTGCCTAGCCCGGGACTTCCTACCCAA
>KY437568.1 Equus caballus clone 412 immunoglobulin mu heavy chain (IGHM) mRNA, partial cds
CTCTCCCTCACCTGCACTGTCTCTGGATTATCTTTGAGCAGTTATGGTGTGGGCTGGGTCCGCCAGGCTCCAGGAAA
AGGGCTGGAATTTGTTGGTGGAGGAGCTAGTAGTGGAAGTGCAAACTACAACCCAGCCCTGAAGTCCCGAGCCAGCA
TCACCAAGGACACCTCAAAGAGCCAAGTTTATCTGACGCTGAACAGCCTGACAAGCGAGGACACGGCCGTCTATTTT

TGTGCAGGAGGCCGGGGTTACCCTGGTGGTAGTTCCTGGTTGTCTATGCTTGGCTACTGGGGCCAGGGCACCCTGGT
CACCGTCTCCTCAGAGAGTACNAAGACCCCAGATCTCTTCCCCCTCGTCTCCTGTGGGCCCTCTCTTGATGAGAGCC
TGGTGGCTGTGGGCTGCCTAGCCCGGGACTTCCTACCCAA
>KY437567.1 Equus caballus clone 411 immunoglobulin mu heavy chain (IGHM) mRNA, partial cds
CTCTCCCTCACCTGCACTGTCTCTGGATTATCTTTGAGCAGTTATGGTGTGGGCTGGGTCCGCCAGGCTCCAGGAAA
AGGGCTGGAATTTGTTGGTGGTATAGCTAGTAGTGGAAGTGCAAACTACAACCCAGCCCTGAAGTCCCGAGCCAGCA
TCACCAAGGACACCTCAAAGAGCCAAGTTTATCTGACGCTGAACAGCCTGACAAGCGAGGACACGGCCGTCTATTAC
TGTGCAGGAGCTCGAGAGGAATACAGTTACTATGGTGGTAGTTCCTGGTACGGGGGTTTTTTTGGCTACTGGGGCCA
GGGCACCCTGGTCACCGTCTCCTCAGAGAGTACGAAGACCCCAGATCTCTTCCCCCTCGTCTCCTGTGGGCCCTCTC
TTGATGAGAGCCTGGTGGCTGTGGGCTGCCTAGCCCGGGACTTCCTACCCAA
>KY437566.1 Equus caballus clone 410 immunoglobulin mu heavy chain (IGHM) mRNA, partial cds
CTGTCCCTCACCTGCACTGTCTCTGGATTCTCTTTGAGCAGTTACGGTGTAGGCTGGGTCCGCCAGGCTCCAGGAAA
AGGGCTGGAATTTGTTGGTGGTATAGCTAGTAGTGGAAGTGCAAACTACAACCCAGCCCTGAAGTCCCGAGCCAGCA
TCACCAAGGACACCTCAAAGAGCCAAGTTTATCTGACGCTGAACAGCCTGACAGGCGAGGACACGGCCGTCTATTAC
TGTGCGAAATTTAGAGGTTCCTATGCTGGTAGCACAGGGGGTGATGTAGACTACTGGGGCCAGGGCATCCTGGTCAC
CGTCTCCTCAGAGAGTACGAAGACCCCAGATCTCTTCCCCCTCGTCTCCTGTGGGCCCTCTCTTGATGAGAGCCTGG
TGGCTGTGGGCTGCCTAGCCCGGGACTTCCTACCCAA
>KY437565.1 Equus caballus clone 409 immunoglobulin mu heavy chain (IGHM) mRNA, partial cds
CTCTCCCTCACCTGCACTGTCTCTGGATTCTCTTTGAGCAGTTACGGTGTAGGCTGGGTCCGCCAGGCTCCAGGAAA
AGGGCTGGAATTTGTTGGTGGTATAGCTAGTAGTGGAAGTGCAAACTACAACCCAGCCCTGAAGTCCCGAGCCAGCA
TCACCAAGGACACCTCAAAGAGCCAAGTTTACCTGACGCTGAACAGCCTGACAAGCGAGGACACGGCCGTCTATTAC
TGTGCAGGAGAAATCCTTAGGTACTATACCTATGGTAGTTACTATGCAGAGGGTTTTGGCTACTGGGGCCAGGGCAC
CCTGGTCACCGTCTCCTCAGAGAGTACGAAGACCCCAGATCTCTTCCCCCTCGTCTCCTGTGGGCCCTCTCTTGATG
AGAGCCTGGTGGCTGTGGGCTGCCTAGCCCGGGACTTCCTACCCAA
>KY437564.1 Equus caballus clone 408 immunoglobulin mu heavy chain (IGHM) mRNA, partial cds
CTCTCCCTCACCTGCACTGTCTCTGGATTATCTTTGAGCAGTAATGGTGTGGGCTGGGTCCGCCAGGCTCCAGGAAA
AGGGCTGGAATTTGTTGGTGGTATAGTTAGTAGTGGAAGTACAAACTACAACCCAGCCCTGAAGTCCCGAGCCAGCA
TCACCAAGGACACCTCAAAGAGCCAAGTTTATCTGACGCTGAACAGCCTGACAAGCGAGGACACGGCCGTCTATTAC
TGTGCAGGGGCTCTATATGGTTACTATGTTGGCTACTGGGGCCAGGGCACCCTGGTCACCGTCTCCTCAGAGAGTAC
GAAGACCCCAGATCTCTTCCCCCTCGTCTCCTGTGGGCCCTCTCTTGATGAGAGCCTGGTGGCTGTGGGCTGCCTAG
CCCGGGACTTCCTACCCAA
>KY437563.1 Equus caballus clone 407 immunoglobulin mu heavy chain (IGHM) mRNA, partial cds
CTGTCCCTCACCTGCACTGTCTCTGGATTCTCTTTGAGCAGTTACGGTGTAGGCTGGGTCCGCCAGGCTCCAGGAAA
AGGGCTGGAATTTGTTGGTGGTATAGCTAGTAGTGGAAGTGCAAACTACAACCCAGCCCTGAAGTCCCGAGCCAGCA
TCACCAAGGACACCTCAAAGAGCCAAGTTTATCTGACGCTGAACAGCCTGACAGGCGAGGACACGGCCGTCCATTAC
TGTGCGAGATTCGATGGTTCCTATGTTGGTGGTAAATATTATGGTATAGACTACTGGGGCCAGGGCATCCTGGTCAC
CGTCTCCTCAGAGAGTACGAAGACCCCAGATCTCTTCCCCCTCGTCTCCTGTGGGCCCTCTCTTGATGAGAGCCTGG
TGGCTGTGGGCTGCCTAGCCCGGGACTTCCTACCCAA
>KY437562.1 Equus caballus clone 406 immunoglobulin mu heavy chain (IGHM) mRNA, partial cds
CTCTCCCTCACCTGCACTGTCTCTGGATTCTCTTTGAGCAGTTACGGTGTAGGCTGGGTCCGCCAGGCTCCAGGAAA
AGGGCTGGAATCTGTTGGTGGTATAGGTAGTAGTGGAAGTGCAAACTACAACCCAGCCCTGAAGTCCCGAGCCAGCA
TCACCAAGGACACCTCAAAGAGCCAAGTTTATCTGACGCTGAACAGCCTGACAAGCGAGGACACGGCCGTCTATTAC
TGTGCAGGATATGGTTATGGTAGTCATTACTACTACGGGCCTTATGGTATACACTACTGGGGCCAGGGCATCCTGGT
CACCGTCTCCTCAGAGAGTACGAAGACCCCAGATCTCTTCCCCCTCGTCTCCTGTGGGCCCTCTCTTGATGAGAGCC
TGGTGGCTGTGGGCTGCCTAGCCCGGGACTTCCTACCCAA
>KY437561.1 Equus caballus clone 405 immunoglobulin mu heavy chain (IGHM) mRNA, partial cds
CTGTCCCTCACCTGCACTGTCTCTGGATTCTCTTTGAGCAGTTACGGTGTAAAATGGGTCCGCCAGGCTCCAGGAAA
AGGGCTGGAATTTGTTGGTGGTATACCTAGTAGTGGAAGTGCAAACTACAACCCAGCCCTGAAGTCCCGAGCCAGCA
TCACCAAGGACACCTCAAAGAGCCAAGTTTATCTGACGCTGAACAGCCTGACAGGCGAGGACACGGCCGTCTATTAC

TGTGCGAGATCCCCCTATAGCAGCTATGCTTACTACGATAGTGGTATAGACTACTGGGGCCAGGGCATCCTGGTCAC
CGTCTCCTCAGAGAGTACGAAGACCCCAGATCTCTTCCCCCTCGTCTCCTGTGGGCCCTCTCTTGATGAGAGCCTGG
TGGCTGTGGGCTGCCTAGCCCGGGACTTCCTACCCAA
>KY437560.1 Equus caballus clone 404 immunoglobulin mu heavy chain (IGHM)
mRNA, partial cds
CTGTCCCTCACCTGCACTGTCTCTGGATTCTCTTTGAGCAGTTACGGTGTAGGCTGGGTCCGCCAGGCTCCAGGAAA
AGGGCTGGAAATTGTTGGTGGTATAGCTAGTAGTGGAAGTGCAAACTACAACCCAGCCCTGAAGTCCCGAGCCAGCA
TCACCAAGGACACCTCAAAGAGCCAAGTTTATCTGACGCTGAACAGCCTGACAGGCGAGGACACGGCCGTCTATTAC
TGTGCGAGATCCCCTCCGGCTATATTAGATTCATTCCCCGATTATTATGGTATAGACTACTGGGGCCAGGGCATCCT
GGTCACCGTCTCCTCAGAGAGTACGAAGACCCCAGATCTCTTCCCCCTCGTCTCCTGTGGGCCCTCTCTTGATGAGA
GCCTGGTGGCTGTGGGCTGCCTAGCCCGGGACTTCCTACCCAA
>KY437559.1 Equus caballus clone 403 immunoglobulin mu heavy chain (IGHM)
mRNA, partial cds
CTCTCCCTCACCTGCACTGTCTCTGGATTGTCTTTGAGCAGTTATGCTGTATACTGGGTCCGCCAGGCTCCAGGAAA
AGGGCTGGAATATGTTGGTCGTATAGCTAGTAGTGGAAGTAGAAACTACAACCCAGCCCTGAAGTCCCGAGCCAGCA
TCACCAAGGACACCTCCAAGAGCCAAGTTTATCTGACGCTGAACAGCCTGACAGGCGAGGACACGGCCGTCTATTAC
TGTGCGAAAGATGGTTACTATGATAGTGGTTATGGGTTCTATTATTATGGTATAGACTACTGGGGCCAGGGCATCCT
GGTCACCGTCTCCTCAGAGAGTACGAAGACCCCAGATCTCTTCCCCCTCGTCTCCTGTGGGCCCTCTCTTGATGAGA
GCCTGGTGGCTGTGGGCTGCCTAGCCCGGGACTTCCTACCCAA
>KY437558.1 Equus caballus clone 402 immunoglobulin mu heavy chain (IGHM)
mRNA, partial cds
CTCTCCCTCACCTGCACTGTCTCTGGATTATCTTTGAGCAGTTATGGTGTGGGCTGGGTCCGCCAGGCTCCAGGAAA
AGGGCTGGAATTTGTTGGTGGTATAGCTAGTAGTGGAAGTGCAAACTACAACCCAGCCCTGAAGTCCCGAGCCAGCA
TCACCAAGGACACCTCAAAGAGCCAAGTTTATCTGACGCTGAACAGCCTGACAAGCGAGGACACGGCCGTCTATTAC
TGTGTATTATTCCTGGGTAGCAACTACTATTTTGGCTACTGGGGCCAGGGCACCCTGGTCACCGTCTCCTCAGAGAG
TACGAAGACCCCAGATCTCTTCCCCCTCGTCTCCTGTGGGCCCTCTCTTGATGAGAGCCTGGTGGCTGTGGGCTGCC
TAGCCCGGGACTTCCTACCCAA
>KY437557.1 Equus caballus clone 401 immunoglobulin mu heavy chain (IGHM)
mRNA, partial cds
CTGTCCCTCACCTGCACTGTCTCTGGATTCTCTTTGAGCAGTTACGGTGTAGGCTGGGTCCGCCAGGCTCCAGGAAA
AGGGCTGGAATTTGTTGGTGGTATAGCTAGTAGTGGAAGTGCAAGGTACAACCCAGCCCTGAAGTCCCGAGCCAGCA
TCACCAAGGACACCTCAAAGAGCCAAGTTTATCTGACGCTGAACAGCCTGACAGGCGAGGACACGGCCGTCTATTAC
TGTGCGAGATCTTACCGGCCCAATGGGTATAGTGCTATGGACCCCTGGGGCCAGGGCACCCTGGTCACCGTCTCCTC
AGAGAGTACGAAGACCCCAGATCTCTTCCCCCTCGTCTCCTGTGGGCCCTCTCTTGATGAGAGCCTGGTGGCTGTGG
GCTGCCTAGCCCGGGACTTCCTACCCAA
>KY437556.1 Equus caballus clone 400 immunoglobulin mu heavy chain (IGHM)
mRNA, partial cds
CTCTCCCTCACCTGCACTGTCTCTGGATTATCTTTGAGCAGTAATGCTGTAGGCTGGGTCCGCCAGGCTCCAGGAAA
AGGGCTGGAATACGTTGGTGCTATATATGGTAGTGCAAGTGCAAACTACAACCCAGCCCTGAAGTCCCGAGCCAGCA
TCACCAAGGACACCTCAAAGAGCCAAGTTTATCTGACGCTGAACAGCCTGACAAGCGAGGACACGGCCGTCTATTAC
TGTGCAGGAGACGATAGACGCTACGGTTATGGTTATGCTACGATAGGCTACTGGGGCCAGGGCACCCTGGTCACCGT
CTCCTCAGAGAGTACGATGACCCCAGATCTCTTCCCCCTCGTCTCCTGTGGGCCCTCTCTTGATGAGAGCCTGGTGG
CTGTGGGCTGCCTAGCCCGGGACTTCCTACCCAA
>KY437555.1 Equus caballus clone 399 immunoglobulin mu heavy chain (IGHM)
mRNA, partial cds
CTCTCCCTCACCTGCACTGTCTCTGGATTATCTTTGAGCAGTTATGGTGTAGGCTGGGTCCGCCAGGCTCCAGGAAA
AGGGCTGGAATTTGTTGGTGGTATAGCTAGTAGTGGAAGTGCAAACTACAACCCAGCCCTGAAGTCCCGAGCCAGCA
TCACCAAGGACACCTCAAAGAGCCAAGTTTATCTGACGCTGAACAGCCTGACAAGCGAGGACACGGCCGTCTATTAC
TGTGCAGGAGGTTATGATGGAGGTTATGGTGGTGGTTATTCCTACTATTTTGACTACTGGGGCCAGGGCACCCTGGT
CACCGTCTCCTCAGAGAGTACGATGACCCCAGATCTCTTCCCCCTCGTCTCCTGTGGGCCCTCTCTTGATGAGAGCC
TGGTGGCTGTGGGCTGCCTAGCCCGGGACTTCCTACCCAA
>KY437554.1 Equus caballus clone 398 immunoglobulin mu heavy chain (IGHM)
mRNA, partial cds
CTGTCCCTCACCTGCACTGTCTCTGGATTCTCTTTGAGCAGTTACGGTGTAGGCTGGGTCCGCCAGGCTCCAGGAAA
AGGGCTGGAATTTGTTGGTGGTATAGTTAGTAGTGGAAGTGCAATGTACAACCCAGCCCTGAAGTCCCGAGCCAGCA

TCACCAAGGACACCTCAAAGAGCCAAGTTTATCTGACGCTGAACAGCCTGACAGGCGAGGACACGGCCGTCTATTAC
TGTGCGAGATCGGTGTCATATAGTTATGGTGGTGGTTACCTATACTATTTTGACTACTGGGGCCAGGGCACCCTGGT
CACCGTCTCCTCAGAGAGTACGATGACCCCAGATCTCTTCCCCCTCGTCTCCTGTGGGCCCTCTCTTGATGAGAGCC
TGGTGGCTGTGGGCTGCCTAGCCCGGGACTTCCTACCCAA
>KY437553.1 Equus caballus clone 397 immunoglobulin mu heavy chain (IGHM) mRNA, partial cds
CTCTCCCTCATCTGCACTGTCTCTGGATTATCTTTGAGCAGTTATGGTGTGGGCTGGGTCCGCCAGGCTCCAGGAAA
AGGGCTGGAATTTGTTGGTGGTATAGCTAGTAGTGGAAGTGCAAACTACAACCCAGCCCTGAAGTCCCGAGCCAGCA
TCACCAAGGACACCTCAAAGAGCCAAGTTTATCTGACGCTGAACAGCCTGACAAGCGAGGACACGGCCGTCTATTAC
TGTGCAGGAGACAATTACTATGGTGGTAGTTCCTGGTACTCCGTCTCTAGCTTTGACTACTGGGGCCAGGGCACCCT
GGTCACCGTCTCCTCAGAGAGTACGAAGACCCCAGATCTCTTCCCCCTCGTCTCCTGTGGGCCCTCTCTTGATGAGA
GCCTGGTGGCTGTGGGCTGCCTAGCCCGGGACTTCCTACCCAA
>KY437552.1 Equus caballus clone 396 immunoglobulin mu heavy chain (IGHM) mRNA, partial cds
CTCTCCCTCACCTGCACTGTCTCTGGATTATCTTTGAGCAGTAATGCTGTAGGCTGGGTCCGCCAGGCTCCAGGAAA
AGGGCTGGAATTTGTTGGTACTATATATGGTAGTGCAAGTGCAAACTACAACCCAGCCCTGAAGTCCCGAGCCAGCA
TCACCAAGGACACCTCAAAGAGCCAAGTTTATCTGACGCTGAACAGCCTGACAAGCGAGGACACGGCCGTCTATTAC
TGTGCAGGAGGTTACTATGCTAGTTACTACTATGCGAATTATGGTATAAACTACTGGGGCCAGGGCATCCTGGTCAC
CGTCTCCTCAGAGAGTACGATGACCCCAGATCTCTTCCCCCTCGTCTCCTGTGGGCCCTCTCTTGATGAGAGCCTGG
TGGCTGTGGGCTGCCTAGCCCGGGACTTCCTACCCAA
>KY437551.1 Equus caballus clone 395 immunoglobulin mu heavy chain (IGHM) mRNA, partial cds
CTCTCCCTCACCTGCACTGTCTCTGGATTATCTTTGAGCAGTAATGCTGTAGGCTGGGTCCGCCAGGCTCCAGGAAA
AGGGCTGGAATTTGTTGGTGATATATATGGTAGTGCAAGTGCAAACTACAACCCAGCCCTGAAGTCCCGAGCCAGCA
TCACCAAGGACACCTCAAAGAGCCAAGTTTATCTGACGCTGAACAGCCTGACAAGCGAGGACACGGCCGTCTATTAC
TGTGCAGGAGGGATGAGGAATGTGGTGCTTATCCCGTACTATTTTGTCTACTGGGGCCAGGGCACCCTGGTCACCGT
CTCCTCAGAGAGTACGATGACCCCAGATCTCTTCCCCCTCGTCTCCTGTGGGCCCTCTCTTGATGAGAGCCTGGTGG
CTGTGGGCTGCCTAGCCCGGGACTTCCTACCCAA
>KY437550.1 Equus caballus clone 394 immunoglobulin mu heavy chain (IGHM) mRNA, partial cds
CTCTCCCTCACCTGCACTGTCTCTGGATTATCTTTGAGCAGTAATGCTGTAGGCTGGGTCCGCCAGGCTCCAGGAAA
AGGGCTGGAATACGTTGGTGGTTCATATGGTAGTGAAAGTGCCTACTACAACCCAGCCCTGAAGTCCCGAGCCAGCA
TCACCAAGGACACCTCAAAGAGCCAAGTTTATCTGACGCTGAACTACCTGACAAGCGAGGACACGGCCGTCTATTAC
TGTGCAGGATTGACGGTTATGAACTGGGGCCAGGGCACCCTGGTCACCGTCTCCTCAGAGAGTACGATGACCCCAGA
TCTCTTCCCCCTCGTCTCCTGTGGGCCCTCTCTTGATGAGAGCCTGGTGGCTGTGGGCTGCCTAGCCCGGGACTTCC
TACCCAA
>KY437549.1 Equus caballus clone 393 immunoglobulin mu heavy chain (IGHM) mRNA, partial cds
CTCTCCCTCACCTGCACTGTCTCTGGATTATCTTTGAGCAGTAATGGTGTAGGCTGGGTCCGCCAGGCTCCAGGAAA
AGGGCTGGAATATGTTGGTGGTATAGCTAGTAGTGGAAGTGCAAACTACAACCCAGCCCTGAAGTCCCGAGCCAGCA
TCACCAAGGACACCTCAAAGAGCCAAGTTTATCTGACGCTGAACAGCCTGACAAGCGAGGACACGGCCGTCTATTAC
TGTGCAGGGGTTGACTATGGTTATAGTGGTGGTTGGTACTACTATTTTGGCTACTGGGGCCAGGGCACCCTGGTCAC
CGTCTCCTCAGAGAGTACGATGACCCCAGATCTCTTCCCCCTCGTCTCCTGTGGGCCCTCTCTTGATGAGAGCCTGG
TGGCTGTGGGCTGCCTAGCCCGGGACTTCCTACCCAA
>KY437548.1 Equus caballus clone 392 immunoglobulin mu heavy chain (IGHM) mRNA, partial cds
CTCTCCCTCACCTGCACTGTCTCTGGATTATCTTTGAGTAGTTATGGTGTGGGCTGGGTCCGCCAGGCTCCAGGAAA
AGGGCTGGAATTTGTTGGTGGCATAGCTACTGGTGGAAGTGCAAACTGCAACCCAGCCCTGAAGTCCCGAGCCAGCA
TCACCAAGGACACCTCAAAGAGCCAAGTTTATCTGACGCTGAACAGCCTGACAAGCGAGGACACGGCCGTCTATTAC
TGTGCAGGAGGTTATGGTGGAGGTTATGGTGGTGGTTACTACTACTATTTTGACTACTGGGGCCAGGGCACCCTGGT
CACCGTCTCCTCAGAGAGTACGATGACCCCAGATCTCTTCCCCCTCGTCTCCTGTGGGCCCTCTCTTGATGAGAGCC
TGGTGGCTGTGGGCTGCCTAGCCCGGGACTTCCTACCCAA
>KY437547.1 Equus caballus clone 391 immunoglobulin mu heavy chain (IGHM) mRNA, partial cds
CTCTCCCTCACCTGCACTGTCTCTGGATTATCTTTGAGCAGTAATGCTGTAGGCTGGGTCCGCCAGGCTCCAGGAAA
AGGGCTGGAATATGTTGGTGGTATAACTAGTAGTGGAAGTGAAAGGTACAACCCAGCCCTGAAGTCCCGAGCCAGCA

TCACCAAGGACACCTCAAAGAGCCAAGTTTATCTGACGCTGAACAGCCTGACAAGCGAGGACACGGCCGTCTATTAC
TGTGCAGGAACTAATGGTTATGGTGGTTATTACTACTATTACTATTTTGGCTACTGGGGCCAGGGCACCCTGGTCAC
CGTCTCCTCAGAGAGTACGATGACCCCAGATCTCTTCCCCCTCGTCTCCTGTGGGCCCTCTCTTGATGAGAGCCTGG
TGGCTGTGGGCTGCCTAGCCCGGGACTTCCTACCCAA
>KY437546.1 Equus caballus clone 390 immunoglobulin mu heavy chain (IGHM) mRNA, partial cds
CTCTCCCTCACCTGCACTGTCTCTGGATTCTCTTTGAGCAGTTACGGTGTAGGCTGGGTCCGCCAGGCTCCAGGAAA
AGGGCTGGAATTTATTGGTGGTATACCTAGTAGTGGAAGTGCAAACTACAACCCAGCCCTGAAGTCCCGAGCCAGCA
TCACCAAGGACACCTCAAAGAGCCAAGTTTATCTGACGCTGAACAGCCTGACAAGCGAGGACACGGCCGTCTATTAC
TGTGCAGGAGGCTATGATCGTTACTATGGTAGTTACTATGCCGGGTATTTTGGCTACTGGGGCCAGGGCACCCTGGT
CACCGTCTCCTCAGAGAGTACGATGACCCCAGATCTCTTCCCCCTCGTCTCCTGTGGGCCCTCTCTTGATGAGAGCC
TGGTGGCTGTGGGCTGCCTAGCCCGGGACTTCCTACCCAA
>KY437545.1 Equus caballus clone 389 immunoglobulin mu heavy chain (IGHM) mRNA, partial cds
CTCTCCCTCACCTGCACTGTCTCTGGATTATCTTTGAGCAGTAATGGTGTAGGCTGGGTCCGCCAGGCTCCAGGAAA
AGGGCTGGAATATGTTGGTGGTATAGCTAGTAGTGGAAGTGCAAACTACAACCCAGCCCTGAAGTCCCGAGCCAGCA
TCACCAAGGACACCTCAAAGAGCCAAGTTTATCTGACGCTGAACAGCCTGACAAGCGAGGACACGGCCGTCTATTAC
TGTGCAGGAATTCTTGGTTACTATGCTGGTAGTTACTATTATGTATACTATTTTGACTACTGGGGCCAGGGCACCCT
GGTCACCGTCTCCTCAGAGAGTACGATGACCCCAGATCTCTTCCCCCTCGTCTCCTGTGGGCCCTCTCTTGATGAGA
GCCTGGTGGCTGTGGGCTGCCTAGCCCGGGACTTCCTACCCAA
>KY437544.1 Equus caballus clone 388 immunoglobulin mu heavy chain (IGHM) mRNA, partial cds
CTGTCCCTCACCTGCACTGTCTCTGGATTCTCTTTGAGCAGTTACGGTGTAGGCTGGGTCCGCCAGGCTCCAGGAAA
AGGGCTGGAATTTGTTGGTGGTATAGCTAGTAGTGGAAGTGCAAACTACAACCCAGCCCTGAAGTCCCGAGCCAGCA
TCAATGAGGACACCTCAAAGAGTCAAGTTTATCTGACGCTGAACAGCCTGACAGGCGAGGACACGGCCGTCTATTAC
TGTGCGAGATTCAGTGGTGGTACTTACTACTCGACCAATTATAATGAGATAGACTACTGGGGCCAGGGCACCCTGGT
CACCGTCTCCTCAGAGAGTACGATGACCCCAGATCTCTTCCCCCTCGTCTCCTGTGGGCCCTCTCTTGATGAGAGCC
TGGTGGCTGTGGGCTGCCTAGCCCGGGACTTCCTACCCAA
>KY437543.1 Equus caballus clone 387 immunoglobulin mu heavy chain (IGHM) mRNA, partial cds
CTGTCCCTCACCTGCACTGTCTCTGGATTCTCTTTGAGCAGTTACGCTGTAGGCTGGGTCCGCCAGGCTCCAGGAAA
AGGGCTGGAATATGTTGGTGGTATAGCTAGTAGTGGAAGTGCAAACTACAACCCAGCCCTGAAGTCCCGAGCCAGCA
TCACCAAGGACACCTCAAAGAGCCAAGTTTATCTGACGCTGAACAGCCTGACAGGCGAGGACACGGCCGTCTATTAC
TGTGCAGGAGGGATGTTTTGGGATAGTTATGTTAGTAACTATGCCCCGTATGGTATAAACTACTGGGGCCAGGGCAT
CCTGGTCACCGTCTCCTCAGAGAGTACGATGACCCCAGATCTCTTCCCCCTCGTCTCCTGTGGGCCCTCTCTTGATG
AGAGCCTGGTGGCTGTGGGCTGCCTAGCCCGGGACTTCCTACCCAA
>KY437542.1 Equus caballus clone 386 immunoglobulin mu heavy chain (IGHM) mRNA, partial cds
CTCTCCCTCACCTGCACTGTCTCTGGATTATCTTTGAGCAGTAATGGTGTAGGCTGGGTCCGCCAGGCTCCAGGAAA
AGGGCTGGAATATGTTGGTGGTATAGCTAGTAGTGGAAGTGCAAACTACAACCCAGCCCTGAAGTCCCGAGCCAGCA
TCACCAAGGACACCTCAAAGAGCCAAGTTTATCTGACGCTGAACAGCCTGACAAGCGAGGACACGGCCGTCTATTAC
TGTGCAGGAGACGATTATTATGGTGCTATTGACTACAGCCTTATTGCCTACTGGGGCCAGGGCACCCTGGTCACCGT
CTCCTCAGAGAGTACGATGACCCCAGATCTCTTCCCCCCCGTCTCCTGTGGGCCCTCTCTTGATGAGAGCCTGGTGG
CTGTGGGCTGCCTAGCCCGGGACTTCCTACCCAA
>KY437541.1 Equus caballus clone 385 immunoglobulin mu heavy chain (IGHM) mRNA, partial cds
CTCTCCCTCACCTGCACTGTCTCTGGATTATCTTTGAGTAGTTATGGTGTGGGCTGGGTCCGCCAGGCTCCAGGAAA
AGGGCTGGAATATGTTGGTGGTATAGCGCATAGTGGAAGTACAAACTACAACCCAGCCCTGAAGTCCCGAGCCAGCA
TCACCAAGGACACCTCAAAGAGCCAAGTTTATCTGACGCTGGACAGCCTGACAAGCGAGGACACGGCCGTCTATTAC
TGTACAGGAGGACTAAATTACTATGGTGGTTACTGGAGTAGTTACTATGCCGACTATTTTGGCTACTGGGGCCAGGG
CACCCTGGTCACCGTCTCCTCAGAGAGTACGATGACCCCAGATCTCTTCCCCCTCGTCTCCTGTGGGCCCTCTCTTG
ATGAGAGCCTGGTGGCTGTGGGCTGCCTAGCCCGGGACTTCCTACCCAA
>KY437540.1 Equus caballus clone 384 immunoglobulin mu heavy chain (IGHM) mRNA, partial cds

CTCTCCCTCACCTGCACTGTCTCTGGATTATCTTTGAGTAGTTATGGTGTGGGCTGGGTCCGCCAGGCTCCAGGAAA
AGGGCTGGAATTTGTTGGTGGTATAGCTAGTAGTGGAAGTGCAAACTACAACCCAGCCCTGAAGTCCCGAGCCAGCA
TCACCAAGGACACCTCAAAGAGCCAAGTTTATCTGACGCTGAACAGCCTGACAAGCGAGGACACGGCCGTCTATTAC
TGTGCAGGGCAAAATAGTTATGGTAGTTACTGGGACTATTTTGCCTACTGGGGCCAGGGCACCCTGGTCACCGTCTC
CTCAGAGAGTACGATGACCCCAGATCTCTTCCCCCTCGTCTCCTGTGGGCCCTCTCTTGATGAGAGCCTGGTGGCTG
TGGGCTGCCTAGCCCGGGACTTCCTACCCAA
>KY437539.1 Equus caballus clone 383 immunoglobulin mu heavy chain (IGHM) mRNA, partial cds
CTCCCCCTCACCTGCACTGTCTCTGGATTATCTTGGAGCAGTAATGCTGTAGGCTGGGTCCGCCAGGCTCCAGGAAA
AGGGCTGGAATACGTTGGTGCTGTATATGGTAGTGCAAGTGCAAACTACAACCCAGCCCTGAAGTCCCGAGCCAGCA
TCACCAAGGACACCTCAAAGAGCCAAGTTTATCTGACGCTGAACAGCCTGACAAGCGAGGACACGGCCGTCTATTAC
TGTGCAGGAGAACGCGGCGAATATGGTTACTATGGTAGTGGTTATGGGACATATTATGGTATAACGTACTGGGGCCA
GGGCATCCTGGTCACCGTCTCCTCAGAGAGTACGATGACCCCAGATCTCTTCCCCCTCGTCTCCTGTGGGCCCTCTC
TTGATGAGAGCCTGGTGGCTGTGGGCTGCCTAGCCCGGGACTTCCTACCCAA
>KY437538.1 Equus caballus clone 382 immunoglobulin mu heavy chain (IGHM) mRNA, partial cds
CTCTCCCTCACCTGCACTGTCTCTGGATTCTCTTTGAGCAGTTACGGTGTAGGCTGGGTCCGCCAGGCTCCAGGAAA
AGGGCTGGAATTTGTTGGTGGTATAGCTAGTAGTGGAAGTGCAAGGTACAACCCAGCCCTGAAGTCCCGAGCCAGCA
TCACCAAGGACACCTCAAAGAGCCAAGTTTATCTGACGCTGAACAGCCTGACAAGCGAGGACACGGCCGTCTATTAC
TGTGCAGGAGGATCAGGCTATAGCAGCTATGCTTCAATGAGCCGCTATATGAATATAAACTACTGGGGCCAGGGCAT
CCTGCTCACCGTCTCCTCAGAGAGTACGATGACCCCAGATCTCTTCCCCCTCGTCTCCTGTGGGCCCTCTCTTGATG
AGAGCCTGGTGGCTGTGGGCTGCCTAGCCCGGGACTTCCTACCCAA
>KY437537.1 Equus caballus clone 381 immunoglobulin mu heavy chain (IGHM) mRNA, partial cds
CTCTCCCTCACCTGCACTGTCTCTGGATTATCTTTGAGTAGTTATGGTGTGGGCTGGGTCCGCCAGGCTCCAGGAAA
AGGGCTGGAATTTGTTGGACGTATAGCTAGTAGTGGAAGTGCAAACTACAACCCAGCCCTGAAGTCCCGAGCCAGCA
TCACCAAGGACACCTCAAAGAGCCAAGTTTATCTGACGCTGAACAGCCTGACAAGCGAGGACACGGCCGTCTATTAC
TGTGCCCTTCTCTATGGTTATGGTGGTGCTTACTACGAGTATTTTGGCTACTGGGGCCAGGGCACCCTGGTCACCGT
CCCCTCAGAGAGTACGATGACCCCAGATCTCTTCCCCCTCGTCTCCTGTGGGCCCTCTCTTGATGAGAGCCTGGTGG
CTGTGGGCTGCCTAGCCCGGGACTTCCTACCCAA
>KY437536.1 Equus caballus clone 380 immunoglobulin mu heavy chain (IGHM) mRNA, partial cds
CTGTCCCTCACCTGCACTGTCTCTGGATTCTCTTTGAGCAGTTACGGTGTAGGCTGGGTCCGCCAGGCTCCAGGAAA
AGGGCTGGAATTTGTTGGTGGTATAACTAGTAGTGGAAGTGTAAACTACAACCCAGCCCTGAAGTCCCGAGCCAGCA
TCACCAAGGACACCTCAAAGAGCCAGGTTTATCTGACGCTGAACAGCCTGACAGGCGAGGACACGGCCGTCTATTAC
TGTAGAACTATAGACTACTGGGGCCAGGGCACCCTGGTCACCGTCTCCTCAGAGAGTACGATGACCCCAGATCTCTT
CCCCCTCGTCTCCTGTGGGCCCTCTCTTGATGAGAGCCTGGTGGCTGTGGGCTGCCTAGCCCGGGACTTCCTACCCA
A
>KY437535.1 Equus caballus clone 379 immunoglobulin mu heavy chain (IGHM) mRNA, partial cds
CTCTCCCTCACCTGCACTGTCTCTGGATTCTCTTTGAGCAGTTATGCTGTAGGCTGGGTCCGCCAGGCTCCAGGAAA
AGGGCTGGAATATGTTGGTCTTATATATGGTAGTGCAAGTGCAGCCTACAACCCAGCCCTGAAGTCCCGAGCCAGCA
TCACCAAGGACACCTCAAAGAGCCAAGTTTATCTGACGTTGAACAGCCTGACAGGCGAGGACACGGCCGTCTATTAC
TGTGCAGGAGGAACGGCGGGAACTGCTGTAGGAGCTATACTTACTAATGGCTACTGGGGCCAGGGCACCCTGGTCAC
CGTCTCCTCAGAGAGTACGATGACCCCAGATCTCTTCCCCCTCGTCTCCTGTGGGCCCTCTCTTGATGAGAGCCTGG
TGGCTGTGGGCTGCCTAGCCCGGGACTTCCTACCCAA
>KY437534.1 Equus caballus clone 378 immunoglobulin mu heavy chain (IGHM) mRNA, partial cds
CTGTCCCTCACCTGCACTGTCTCTGGATTCTCTTTGAGCAGTTACGGTGTAGGCTGGGTCCGCCAGGCTCCAGGAAA
AGGGCTGGAATTTGTTGGTGGTATAGCTAGTAGTGGAAGTGCAAACTACAACCCAGCCCTGAAGTCCCGAGCCAGCA
TCACCAAGGACACCTCAAAGAGCCAAGTTTATCTGACGCTGAACAGCCTGACAAGCGAGGACACGGCCGTCTATTAC
TGTGCAGGAGGCCGGGGGGAATATGGTTACTATGGTAGTTACTACTATTATGGTATAAACTACTGGGGCCAGGGCAT
CCTGGTCACCGTCTCCTCAGAGAGTACGATGACCCCAGATCTCTTCCCCCTCGTCTCCTGTGGGCCCTCTCTTGATG
AGAGCCTGGTGGCTGTGGGCTGCCTAGCCCGGGACTTCCTACCCAA
>KY437533.1 Equus caballus clone 377 immunoglobulin mu heavy chain (IGHM) mRNA, partial cds

CTCTCCCTCACCTGCACTGTCTCTGGATTCTCTTTGAGCAGTTACGGTGTAGGCTGGGTCCGCCAGGCTCCAGGAAA
AGGGCTGGAATATGTTGGTAGTATAGCTAGTAGTGGAAGTACAAACTACAACCCAGCCCTGAAGTCCCGAGCCAGCA
TCACCAAGGACACCTCAAAGAGCCAAGTTTATCTGACGCTGAACAGCCTGACAAGCGAGGACACGGCCGTCTATTTG
TGTATTTATGGTAGTTATTATGATGTAATAACCTACTGGGGCCAGGGCACCCTGGTCACCGTCTCCTCAGAGAGTAC
GATGACCCCAGATCTCTTCCCCCTCGTCTCCTGTGGGCCCTCTCTTGATGAGAGCCTGGTGGCTGTGGGCTGCCTAG
CCCGGGACTTCCTACCCAA
>KY437532.1 Equus caballus clone 376 immunoglobulin mu heavy chain (IGHM) mRNA, partial cds
CTCTCCCTCACCTGCACTGTCTCTGGATTCTCTTTGAGCAGTGGCGGTGTAGGCTGGGTCCGCCAGGCTCCAGGAAA
AGGGCTGGAATTTGTTGGTAGTATAGCTAGTAGTGGAAGTGCAAACTACAACCCAGCCCTGAAGTCCCGAGCCAGCA
TCACCAAGGACACCTCAAAGAGCCAAGTTTATCTGACGCTGAACAGCCTGACAAGCGAGGACACGGCCGTCTATTAC
TGTGCAGGAGGCAGGAATGGTTACTATACTCTTAGTTACTATGATAGTGCCATTGACTACTGGGGCCAGGGCATCCT
GGTCACCGTCTCCTCAGAGAGTACGATGACCCCAGATCTCTTCCCCCTCGTCTCCTGTGGGCCCTCTCTTGATGAGA
GCCTGGTGGCTGTGGGCTGCCTAGCCCGGGACTTCCTACCCAA
>KY437531.1 Equus caballus clone 375 immunoglobulin mu heavy chain (IGHM) mRNA, partial cds
CTGTCCCCCACCTGCACTGTCTCTGGATTCTCTTTGAGCAGTTACGGTGTAGGCTGGGTCCGCCAGGCTCCAGGAAA
AGGGCTGGAATACGTTGGTGCTATATATGGTGGTGCAAGTGCAAACTACAACCCAGCCCTGAAGTCCCGAGCCAGCA
TCACCAAGGACACCTCAAAGAGCCAAGTTTATCTGACGCTGAACAGCCTGACAGGCGAGGACACGGCCGTCTATTAC
TGTGCGAGATCGAGTAGCAGCTATTATTACTACTATACAATCTGTGATATAGACTACTGGGGCCAGGGCACCCTGGT
CACCGTCTCCTCAGAGAGTACGATGACCCCAGATCTCTTCCCCCTCGTCTCCTGTGGGCCCTCTCTTGATGAGAGCC
TGGTGGCTGTGGGCTGCCTAGCCCGGGACTTCCTACCCAA
>KY437530.1 Equus caballus clone 374 immunoglobulin mu heavy chain (IGHM) mRNA, partial cds
CTCTCCCTCACCTGCACTGTCTCTGGATTCTCTTTGAGCAATTACGGTGTAGGCTGGGTCCGCCAGGCTCCAGGAAA
AGGGCTGGAATTTGTTGGTGGTATAACTAGTAGTGGAAGTGCAAACTACAACCCAGCCCTGAAGTCCCGAGCCAGCA
TCACCAAGGACACCTCAAAGAGCCAAGTTTATCTGACGCTGAACAGCCTGACAAACGAGGACACGGCCGTCTATTAC
TGTACAAGAGGGACTACTATGGACCCCTGGGGCCAGGGCACCCTGGTCACCGTCTCCTCAGAGAGTACGATGACCCC
AGATCTCTTCCCCCTCGTCTCCTGTGGGCCCTCTCTTGATGAGAGCCTGGTGGCTGTGGGCTGCCTAGCCCGGGACT
TCCTACCCAA
>KY437529.1 Equus caballus clone 373 immunoglobulin mu heavy chain (IGHM) mRNA, partial cds
CTCTCCCTCACCTGCACTGTCTCTGGATTATCTTTGAGCAGTTCTACTGTAGGCTGGGTCCGCCAGGCTCCAGGAAA
AGGGCTGGAAAAGGTTGGTGCTATATATGGTAGTGCAAGTGCAAACTACAACCCAGCCCTGAAGTCCCGAGCCAGCA
TCACCAAGGACACCTCCAAGAGCCAAGTTTATCTGACGCTGAACAGCCTGACAGGCGAGGACACGGCCGTCTATTAC
TGTGCGAGATCGAGTGGTAGTGGTGGTGGTTACTACTATGGTGAAAACTACTGGGGCCAGGGCATCCTGGTCACCGT
CTCCTCAGAGAGTACGATGACCCCAGATCTCTTCCCCCTCGTCTCCTGTGGGCCCTCTCTTGATGAGAGCCTGGTGG
CTGTGGGCTGCCTAGCCCGGGACTTCCTACCCAA
>KY437528.1 Equus caballus clone 372 immunoglobulin mu heavy chain (IGHM) mRNA, partial cds
CTGTCCCTCACCTGCACTGTCTCTGGATTCTCTTTGAGCAGTTACGGTGTAGGCTGGGTCCGCCAGGCTCCAGGAAA
AGGGCTGGAATATGTTGGTGGTATAACTAGTAGTGGAAGTGCAAACTACAACCCAGCCCTGAAGTCCCGAGCCAGCA
TCACCAAGGACACTTCAAAGAGCCAAGTTTATCTGACGCTGAACAGCCTGACAGGCGAGGACACGGCCGTCTATTAC
TGTGCGAGAAATGGTTACTATACTTATAGTTACTGGGAGGGCCTCTATTATTATGACATAGACTACTGGGGCCAGGG
CACCCTGGTCACCGTCTCCTCAGAGAGTACGATGACCCCAGATCTCTTCCCCCTCGTCTCCTGTGGGCCCTCTCTTG
ATGAGAGCCTGGTGGCTGTGGGCTGCCTAGCCCGGGAACTTCCTACCCAA
>KY437527.1 Equus caballus clone 371 immunoglobulin mu heavy chain (IGHM) mRNA, partial cds
CTCTCCCTCACCTGCACTGTCTCTGGATTATCTTTGAGCAGTAATGCTGTAGGCTGGGTCCGCCAGGCTCCAGGAAA
AGGGCTGGAATACGTTGGTGCTATATATGGTAGTGCAAGTGCAAACTACAACCCAGCCCTGAAGTCCCGAGCCAGCA
TCACCAAGGACACCTCAAAGAGCCAAGTTTCTCTGACGCTGAACAGCCTGACAAGCGAGGACACGGCCGTCTATTAC
TGTGCAGGAGAGACGGTTTATAGCAGCAGTGCTTACTACGTCGTCCCGGGGTTTGGCTACTGGGGCCAGGGCACCCT
GGTCACCGTCTCCTCAGAGAGTACGATGACCCCAGATCTCTTCCCCCTCGTCTCCTGTGGGCCCTCTCTTGATGAGA
GCCTGGTGGCTGTGGGCTGCCTAGCCCGGGACTTCCTACCCAA

>KY437526.1 Equus caballus clone 370 immunoglobulin mu heavy chain (IGHM) mRNA, partial cds
CTCTCCCTCACCTGCACTGTCTCTGGATTCTCTTTGAGCAGTTATGCTGTAGGCTGGGTCCGCCAGGCTCCAGGAAA
AGGGCTGGAATATGTTGGTGATATATATGGTAGTGCAAGTGCAAACTACAACCCAGCCCTGAAGTCCCGAGCCAGCA
TCACCAAGGACACCTCAAAGAGCCAAGTTTATCTCACGCTGAACAGCCTGACAGGCGAGGACACGGCCGTCTATTAC
TGTGCAGGAGGAACGGATTATGGTTACTATGCTGGTGGTTACTATGGATTTGGCTACTGGGGCCAGGGCACCCTGGT
CACCGTCTCCTCAGAGAGTACGATGACCCCAGATCTCTTCCCCCTCGTCTCCTGTGGGCCCTCTCTTGATGAGAGCC
TGGTGGCTGTGGGCTGCCTAGCCCGGGACTTCCTACCCAA
>KY437525.1 Equus caballus clone 369 immunoglobulin mu heavy chain (IGHM) mRNA, partial cds
CTGTCCCTCACCTGCACTGTCTCTGGATTCTCTTTGAGCAGTTACGGTGTAGGCTGGGTCCGCCAGGCTCCAGGAAA
AGGGCTGGAATATGTTGGTGGTATACTTAGTAGTGGAAGTGCAAACTACAACCCAGCCCTGAAGTCCCGAGCCAGCA
TCACCAAGGACACCTCAAAGAGCCAAGTTTATCTGACGCTGAACAGCCTGACAAGCGAGGACACGGCCGTCTATTAC
TGTGCAGGATTTTACTATGCGGGATATGAACTCTTTGGCTACTGGGGCCAGGGCACCCTGGTCACCGTCTCCTCAGA
GAGTACGATGACCCCAGATCTCTTCCCCCTCGTCTCCTGTGGGCCCTCTCTTGATGAGAGCCTGGTGGCTGTGGGCT
GCCTAGCCCGGGACTTCCTACCCAA
>KY437524.1 Equus caballus clone 368 immunoglobulin mu heavy chain (IGHM) mRNA, partial cds
CTCTCCCTCACCTGCACTGTCTCTGGATTCTCTTTGAGCAGTAATGGTGTGAGCTGGGTCCGCCAGGCTCCAGGAAA
AGGGCTGGAATTTGTTGGTACTATATATGGTAGTGCAAGTGCAAACTACAACCCAGCCCTGAAGTCCCGAGCCAGCA
TCACCAAGGACACCTCAAAGAGCCAAGTTTATCTGACGCTGAACAGCCTGACAAGCGAGGACACGGCCGTCTATTAC
TGTGCGAGATGGGTACCGGGTTATGTTGGTGCTTACGGCACTGCCTACGTGGATCACTGGGGCCAGGGCACCCTGGT
CACCGTCTCCTCAGAGAGTACGATGACCCCAGATCTCTTCCCCCTCGTCTCCTGTGGGCCCTCTCTTGATGAGAGCC
TGGTGGCTGTGGGCTGCCTAGCCCGGGACTTCCTACCCAA
>KY437523.1 Equus caballus clone 367 immunoglobulin mu heavy chain (IGHM) mRNA, partial cds
CTCTCCCTCACCTGCACTGTCTCTGGATTCTCTTTGAATGGTTATGGTGTAGGCTGGGTCCGCCAGGCTCCAGGAAA
AGGGCTGGAATTTGTTGGTGGTATAGCTAGTAGTGGAAGTACAAACTACAACCCAGCCCTGAAGTCCCGAGCCAGCA
TCACCAAGGACACCTCAAAGAGCCAAGTTTATCTGACGCTGAACAGCCCGACAGGCGAGGACACGGCCGTCTATTAC
TGTGCAGGAGGGGATAATTCCTGGTCTTTTGGCTACTGGGGCCAGGGCACCCTGGTCACCGTCTCCTCAGAGAGTAC
GATGACCCCAGATCTCTTCCCCCTCGTCTCCTGTGGGCCCTCTCTTGATGAGAGCCTGGTGGCTGTGGGCTGCCTAG
CCCGGGACTTCCTACCCAA
>KY437522.1 Equus caballus clone 366 immunoglobulin mu heavy chain (IGHM) mRNA, partial cds
CTCTCCCTCACCTGCACTGTCTCTGGATTATCTTTGAGCAGTTATGCTGTAGGCTGGGTCCGCCAGGCTCCAGGAAA
AGGGCTGGAATATGTTGGTGCTATATATGGTAGTGCAAGTGCAAACTACAACCCAGCCCTGAAGTCCCGAGCCAGCA
TCACCAAGGACACCTCAAAGAGCCAAGTTTATCTGACGCTGAACAGCCTGACAGGCGAGGACACGGCCGTCTATTAC
TGTGCGAGATATGGTTACTATGGTAGTTACTACAGTAGTTACTATGATTTTGGCTACTGGGGCCAGGGCACCCTGGT
CACCGTCTCCTCAGAGAGTACGATGACCCCAGATCTCTTCCCCCTCGTCTCCTGTGGGCCCTCTCTTGATGAGAGCC
TGGTGGCTGTGGGCTGCCTAGCCCGGGACTTCCTACCCAA
>KY437521.1 Equus caballus clone 365 immunoglobulin mu heavy chain (IGHM) mRNA, partial cds
CTGTCCCTCACCTGCACTGTCTCTGGATTCTCTTTGAGCAGTTACGGTGTAGGCTGGGTCCGCCAGGCTCCAGGAAA
AGGGCTGGAATTTGTTGGTAGTATAGCTAGTAGTGGAAGTGCAAACTACAACCCAGCCCTGAAGTCCCGAGCCAGCA
TCACCAAGGACACCTCAAAGAGCCAAGTTTATCTGACGCTGAACAGCCTGACAGGCGAGGACACGGCCGTCTATTAC
TGTGCGAGACCCTACGGTTATGGTAGTGCTACGATTTACTACTGGGGCCAGGGCATCCTGGTCACCGTCTCCTCAGA
GAGTACGATGACCCCAGATCTCTTCCCCCTCGTCTCCTGTGGGCCCTCTCTTGATGAGAGCCTGGTGGCTGTGGGCT
GCCTAGCCCGGGACTTCCTACCCAA
>KY437520.1 Equus caballus clone 364 immunoglobulin mu heavy chain (IGHM) mRNA, partial cds
CTGTCCCTCACCTGCAGTGTCTCTGGATTCTCTTTGAGCAGTTACGGTGTAGGCTGGGTCCGCCAGGCTCCAGGAAA
AGGGCTGGAATTTGTTGGTGGTATAGCTAGGAGTGGAAGTGCAAACTACAACCCAGCCCTGAAGTCCCGAGCCAGCA
TCACCAAGGACACCTCAAAGAGCCAAGTTTATCTGACGCTGAACAGCCTGACAGGCGAGGACACGGCCGTCTATTAC
TGTGCGAGATATGCTTATGGTGGTGACTACTGGGGCCAGGGCATCCTGGTCACCGTCTCCTCAGAGAGTACGATGAC
CCCAGATCTCTTCCCCCTCGTCTCCTGTGGGCCCTCTCTTGATGAGAGCCTGGTGGCTGTGGGCTGCCTAGCCCGGG
ACTTCCTACCCAA

```
>KY437519.1 Equus caballus clone 363 immunoglobulin mu heavy chain (IGHM)
mRNA, partial cds
CTGTCCCTCACCTGCACTGTCTCTGGATTATCTTTGAGCAGTAATCGTGTAGGCTGGGTCCGCCAGGCTCCAGGAAA
AGGGCTGGAATACGTTGGTGCTATATATGGTAGTGCAAGTGCAAACTACAACCCAGCCCTGAAGTCCCGAGCCAGCA
TCACCAAGGACACCTCAAAGAGCCAAGTTTATCTGACGCTGAACAGCCTGACAAGCGAGGACACGGCCGTCTATTAC
TGTGTGAGATCAGCATATGGTTATGGTGGTGCTTACTACGGCGGTATAAACTACTGGGGCCAGGGCATCCTGGTCAC
CGTCTCCTCAGAGAGTACGATGACCCCAGATCTCTTCCCCCTCGTCTCCTGTGGGCCCTCTCTTGATGAGAGCCTGG
TGGCTGTGGGCTGCCTAGCCCGGGACTTCCTACCCAA
>KY437518.1 Equus caballus clone 362 immunoglobulin mu heavy chain (IGHM)
mRNA, partial cds
CTCTCCCTCACCTGCACTGTCTCTGGATTCTCTTTGAGCAGTTACGGTGTAGGCTGGGTCCGCCAGGCTCCAGGAAA
AGGGCTGGAATTGGTTGGTGGTATAGCTAAGAGTGGAACTACAAACTACAACCCAGCCCTGAAGTCCCGAGCCAGCA
TCACCAAGGACACCTCAAAGAGCCAAGTTTATCTGACGCTGAACAGCCTGACAAGCGAGGACACGGCCGTCTATTAC
TGTGCAGGATACGTGTATGGTTATGGTAGTGCTTACTACTACCCCTTTGGCTACTGGGGCCAGGGCACCCTGGTCAC
CGTCTCCTCAGAGAGTACGATGACCCCAGATCTCTTCCCCCTCGTCTCCTGTGGGCCCTCTCTTGATGAGAGCCTGG
TGGCTGTGGGCTGCCTAGCCCGGGACTTCCTACCCAA
>KY437517.1 Equus caballus clone 361 immunoglobulin mu heavy chain (IGHM)
mRNA, partial cds
CTCTCCCTCACCTGCACTGTCTCTGGATTATCTTGGAGCAGTAATTATGTAGGCTGGGTCCGCCAGGCTCCAGGAAA
AGGGCTGGAATATGTTGGTGGTATAGGTAGTAGTGGAGGTGCAGCATACAACCCAGCCCTGAAGTCCCGAGCCAGCA
TCACCAAGGACACCTCAAAGAGCCAAGTTTATCTGACGCTGAACAGCCTGACAAGCGAGGACACGGCCGTCTATTAC
TGTGCAGGAGGAATCTATGGTTGGAGTGGTGCTTACTTTTACTGGGGCCAGGGCATCCTGGTCACCGTCTCCTCAGA
GAGTACGATGACCCCAGATCTCTTCCCCCTCGTCTCCTGTGGGCCCTCTCTTGATGAGAGCCTGGTGGCTGTGGGCT
GCCTAGCCCGGGACTTCCTACCCAA
>KY437516.1 Equus caballus clone 360 immunoglobulin mu heavy chain (IGHM)
mRNA, partial cds
CTGTCCCTCACCTGCACTGTCTCTGGATTATCTTTGAGCAGTAACGCTGTAGGCTGGGTCCGCCAGGCTCCAGGAAA
AGGGCTGGAATATGTTGGTGGTATAGGTAGTGGAAGTGGAAACTACAACCCAGCCCTGAAGTCCCGAGCCAGCATCA
CCAAGGACACCTCAAAGAGCCAAGTTTATCTGACGCTGAACAGCCTGACAAGCGAGGACGCGGCCGTCTATTACTGT
GCAGGATTGCATGGTTATGGTGGTGCTTACTACATGTCTTTTGGCTACTGGGGCCAGGGCACCCTGGTCACCGTCTC
CTCAGAGAGTACGATGACCCCAGATCTCTTCCCCCTCGTCTCCTGTGGGCCCTCTCTTGATGAGAGCCTGGTGGCTG
TGGGCTGCCTAGCCCGGGACTTCCTACCCAA
>KY437515.1 Equus caballus clone 359 immunoglobulin mu heavy chain (IGHM)
mRNA, partial cds
CTCTCCCTCACCTGCACTGTCTCTGGATTATCTTTGAGCAGTAATACTATAGGCTGGGTCCGCCAGGCTCCAGGAAA
AGGGCTGGAATTTGTTGGTATATATGGTAGTGCAAGTGCAAACTACAACCCAGCCCTGAAGTCCCGAGCCAGCATCA
CCAAGGACACCTCAAAGAGCCAAGTTTATCTGACGCTGAACAGCCTGACAAGCGAGGACACGGCCGTCTATTACTGT
GCGAGCCCATCAATGTACTGGGGCCAGGGCACCCTGGTCACCGTCTCCTCAGAGAGTACGATGACCCCAGATCTCTT
CCCCCTCGTCTCCTGTGGGCCCTCTCTTGATGAGAGCCTGGTGGCTGTGGGCTGCCTAGCCCGGGACTTCCTACCCA
A
>KY437514.1 Equus caballus clone 358 immunoglobulin mu heavy chain (IGHM)
mRNA, partial cds
CTGTCCCTCACCTGCACTGTCTCTGGATTCTCTTTGAGCAGTGGTGCTGTAGGCTGGGTCCGCCAGGCTCCAGGAAA
AGGACTGGAGTGGGTGGGTACTATAAGTGAGATTGGAATTACATACTACAACCCAGCCCTGAAGTCCCGAGCCAGCA
TCACCAAGGACACCTCAAAGAGCCAAGTGCATCTGACGCTGAACAGCCTGACAGGCGAAGACACGGCCGTCTATTAC
TGTAGGCTCCAAGTGACTTGGGGCCAGGGCACCCTGGTCACCGTCTCCTCAGAGAGTACGATGACCCCAGATCTCTT
CCCCCTCGTCTCCTGTGGGCCCTCTCTTGATGAGAGCCTGGTGGCTGTGGACTGCCTAGCCCGGGACTTCCTACCCA
A
>KY437513.1 Equus caballus clone 357 immunoglobulin mu heavy chain (IGHM)
mRNA, partial cds
CTGTCCCTCACCTGCACTGTCTCTGGATTATCTTTGAGCAGTAATGCTGTAGGCTGGGTCCGCCAGGCTCCAGGAAA
AGGGCTGGAGTGGGTTGGTGTTATATATGGTAGTGAAAGTACAGGCTACAACCCAGCCCTGAAGTCCCGAGCCAGCA
TCACCAAGGACACCTCAAAGAGCCAAGTTTATCTGACGCTGAACAGCCTGACAAGCGAGGACACGGCCGTCTATTAC
TGTGCGAGTGGTGTCTTGGGCCATGATAGTTATGGTACTTACTATGCCGTTGATTATGGTATAAAGTACTGGGGCCA
```

GGGCATCCTGGTCACCGTCTCCTCAGAGAGTACGATGACCCCAGATCTCTTCCCCCTCGTCTCCTGTGGGCCCTCTC
TTGATGAGAGCCTGGTGGCTGTGGGCTGCCTAGCCCGGGACTTCCTACCCAA
>KY437512.1 Equus caballus clone 356 immunoglobulin mu heavy chain (IGHM)
mRNA, partial cds
CTCTCCCTCACCTGCACTGTCTCTGGATTATCTTTGAGCAGTAATGCTGTAAGGTGGGTCCGCCAGGCTCCAGGAAA
AGGGCTGGAATATGTTGCTGGAATGGATAATAGTGGAAGTGCGTACTACAACCCAGCCCTGAAGTCCCGAGCCAGCA
TCACTAAGGACGCCTCAAAGAGCCAAGTTTATCTGACGCTGAACAGCCTGACAAGCGAGGACACGGCCGTCTATTAC
TGTGCAGGAGGTGGTGATTACTCCTGGGGCCAGGGCATCCTGGTCACCGTCTCCTCAGAGAGTACGATGACCCCAGA
TCTCTTCCCCCTCGTCTCCTGTGGGCCCTCTCTTGATGAGAGCCTGGTGGCTGTGGGCTGCCTAGCCCGGGACTTCC
TACCCAA
>KY437511.1 Equus caballus clone 355 immunoglobulin mu heavy chain (IGHM)
mRNA, partial cds
CTGTCCCTCACCTGCACTGTCTCTGGATTATCTTTGAGCAGTAACGCTGTAGGCTGGGTCCGCCAGGCTCCAGGAAA
AGGGCTGGAGTGGGTTGGTGTTATATATGGTAGTGAAAGTACATACTACAACCCAGCCCTGAAGTCCCGAGCCAGCA
TCACCAAGGACACCTCAAAGAGCCAAGTTTATCTGACGCTGAACAGCCTGACAGGCGAAGACACGGCCGTCTATTAC
TGTCTAGGATCGGAAGAAGTGACCTGGGGCCAGGGCACCCTGGTCACCGTCTCCTCAGAGAGTACGATGACCCCAGA
TCTCTTCCCCCTCGTCTCCTGTGGGCCCTCTCTTGATGAGAGCCTGGTGGCTGTGGGCTGCCTAGCCCGGGACTTCC
TACCCA
>KY437510.1 Equus caballus clone 354 immunoglobulin mu heavy chain (IGHM)
mRNA, partial cds
CTCTCCCTCACCTGCACTGTCTCTGGATTATCTTTGAGCAGTAATGCTGTAGGCTGGGTCCGCCAGGCTCCAGGAAA
AGGGCTGGAATATGTTGGTGGTATTTATGGTAGTGGAAGTGCAAACTACAACCCAGCCCTGAAGTCCCGAGCCAGCA
TCACCAAGGACACCCCAAAGAGCCAAGTTTATCTGACGCTGAACAGCCTGACAAGCGAGGACACGGCCGTCTATTAC
TGTGCAGGAACTTACTCTGGTAGTAGGTGGAGTAGTTACTATTCATGGTATTTTGGCTACTGGGGCCAGGGCACCCT
GGTCACCGTCTCCTCAGAGAGTACGATGACCCCAGATCTCTTCCCCCTCGTCTCCTGTGGGCCCTCTCTTGATGAGA
GCCTGGTGGCTGTGGGCTGCCTAGCCCGGGACTTCCTACCCAA
>KY437509.1 Equus caballus clone 353 immunoglobulin mu heavy chain (IGHM)
mRNA, partial cds
CTCTCCCTCACCTGCACTGTCTCTGGATTATCTTTGAGCAGTAATGCTGTAGGCTGGGTCCGCCAGGCTCCAGGAAA
AGGGCTGGAATTTGTTAGTGCTATATATGGTAGTGTAAGTGTAAAGTACAACCCAGCCCTGAAGTCCCGAGCCAGCA
TCACCAAGGACACCTCAAAGAGCCAAGTTTATCTGACGCTGAACAGCCTGACAAGCGAGGACACGGCCGTCTACTAC
TGTGCAGGAGGAGGACGATACGGTTACTATGTTGATAGTTACTATGCGAATGATATAAACTACTGGGGCCAGGGCAT
CCTGGTCACCGTCTCCTCAGAGAGTACGATGACCCCAGATCTCTTCCCCCTCGTCTCCTGTGGGCCCTCTCTTGATG
AGAGCCTGGTGGCTGTGGGCTGCCTAGCCCGGGACTTCCTACCCAA
>KY437508.1 Equus caballus clone 352 immunoglobulin mu heavy chain (IGHM)
mRNA, partial cds
CTCTCCCTCACCTGCACTGTCTCTGGATTCTCTTTGAGCAGTTATACTGTAGGCTGGGTCCGCCAGGCTCCAGGAAA
AGGGCTGGAATATGTTGGTCATATATATGGTAGTGCAAGTGCAAACTACAACCCAGCCCTGAAGTCCCGAGCCAGCA
TCACCAAGGACACCTCAAAGAGCCAAGTTTATCTGACGCTGAACAGCCTGACAGGCGAGGACACGGCCGTCTATTAC
TGTGCGAGAACCCTCTTAAGTGGTAGTTATTCTACTTTTGGCTACTGGGGCCAGGGCACCCTGGTCACCGTCTCCTC
AGAGAGTACGATGACCCCAGATCTCTTCCCCCTCGTCTCCTGTGGGCCCTCTCTTGATGAGAGCCTGGTGGCTGTGG
GCTGCCTAGCCCGGGACTTCCTACCCAA
>KY437507.1 Equus caballus clone 351 immunoglobulin mu heavy chain (IGHM)
mRNA, partial cds
CTGTCCCTCACCTGCACTGTCTCTGGATTATCTTTGAGCAATAGTGATGTAGGCTGGGTCCGCCAGGCTCCAGGAAA
AGGGCTGGAGTGGGTTGGTATTATATATGGTAGTGAAAGTACAAGTACATACTACAACCCAGCCCTGAGGTCCCGAG
CCAGCATCACCAAGGACACCTCAAGGAGCCAAGTTTATCTGACGCTGAACAGCCTGACAGGCGAAGACACGGCCGTC
TATTACTGTGCAGGACTAGCGCAGCTGGTTATTGGGGGGCTGGACATTGGCCATTCCGTTATAGACTACTGGGGCCA
GGGCATCCTGGCCACCGTCTCCTCAGAGAGTACGATGACCCCAGATCTCTTCCCCCTCGTCTCCTGTGGGCCCTCTC
TTGATGAGAGCCTGGTGGCTGTGGGCTGCCTAGCCCGGGACTTCCTACCCAA
>KY437506.1 Equus caballus clone 350 immunoglobulin mu heavy chain (IGHM)
mRNA, partial cds
CTGTCCCTCACCTGCACTGTCTCTGGATTCTCTTTGAGCAGTTACGGTGTAGGCTGGGTCCGCCAGGCTCCAGGAAA
AGGGCTGGAATATGTTGGTGGTATAGCTAGTAGTGGAAGTGCAAACTACAACCCAGCCCTGAAGTCCCGAGCCAGCA
TCACCAAGGACACCTCAAAGAGCCAAGTTTATCTGACGCTGAACAGCCTGACAGGCGAGGACACGGCCGTCTATTAC
TGTGCGAGATCTTATAACTACGATTATCCCTATTATGGTATAAACTACTGGGGCCAGGGCATCCTGGTCACCGTCTC

CTCAGAGAGTACGATGACCCCAGATCTCTTCCCCCTCGTCTCCTGTGGGCCCTCTCTTGATGAGAGCCTGGTGGCTG
TGGGCTGCCTAGCCCGGGACTTCCTACCCAA
>KY437505.1 Equus caballus clone 349 immunoglobulin mu heavy chain (IGHM) mRNA, partial cds
CTCTCCCTCACCTGCACTGTCTCTGGATTATCTTTGAGCAGTAATGGTGTAGCCTGGGTCCGCCAGGCTCCAGGAAA
AGGGCTGGGATATGTTGGTGCTATAGCTACGAGTGGAAGTACAGACTACAACCCAGCCCTGAAGTCCCGAGCCAGCA
TCACCAAGGACACCTCAAAGAGCCAAGTTTATCTGACGCTGAACAGCCTGACAGGTGAAGACACGGCCGTCTATTAC
TGTGCAGGATTAGGGGACATCCTTGCATATGGTAGCTATGCTGGTAGTTACTATGGCTACGTGGATCACTGGGGCCA
GGGCACCCTGGTCACCGTCTCCTCAGAGAGTACGATGACCCCAGATCTCTTCCCCCTCGTCTCCTGTGGGCCCTCTC
TTGATGAGAGCCTGGTGGCTGTGGGCTGCCTAGCCCGGGACTTCCTACCCAA
>KY437504.1 Equus caballus clone 348 immunoglobulin mu heavy chain (IGHM) mRNA, partial cds
CTCTCCCTCACCTGCACTGTCTCTGGATTATCTTTGAGCAGTAATGCTGTAGCCTGGGTCCGCCAGGCTCCAGGAAA
AGGGCTGGAGTGGGTTGGTGGTATATATGCTAGTGAAAGTGCATGGTACGACTTAGTCCTGAAGTCCCGAGCCAGCA
TCACCAAGGACACCTCAAAGAGCCAAGTTTATCTGACGCTGAACAGCCTGACAAGCGAGGACACGGCCGTCTATTTT
TGTGTAGGAGACTTAGTCCGCTGGGGCCAGGGCATCCTGGTCACCGTCTCCTCAGAGAGTACGATGACCCCAGATCT
CTTCCCCCTCGTCTCCTGTGGGCCCTCTCTTGATGAGAGCCTGGTGGCTGTGGGCTGCCTAGCCCGGGACTTCCTAC
CCAA
>KY437503.1 Equus caballus clone 347 immunoglobulin mu heavy chain (IGHM) mRNA, partial cds
CTGTCCCTCACCTGCACTGTCTCTGGATTCTCTTTGAGCAGTTACGGTGTAGGCTGGGTCCGCCAGGCTCCAGGAAA
AGGGCTGGAATATGTTGGTCGTATAGCTAGTAGTGGAAGTGCAAACTACAACCCAGCCCTGAAGTCCCGAGCCAGCA
TCACCAAGGACACCTCAAAGAGCCAAGTTTATCTGACGCTGAACAGCCTGACAGGCGAGGACACGGCCGTCTATTAC
TGTGCGAGATTCCTCTTTTATAGCATCAATGGTTACTTTGAATACCCTTATGGTAGCAACTACTGGGGCCAGGGCAT
CCTGGTCACCGTCTCCTCAGAGAGTACGATGACCCCAGATCTCTTCCCCCTCGTCTCCTGTGGGCCCTCTCTTGATG
AGAGCCTGGTGGCTGTGGGCTGCCTAGCCCGGGACTTCCTACCCAA
>KY437502.1 Equus caballus clone 346 immunoglobulin mu heavy chain (IGHM) mRNA, partial cds
CTCTCCCTCACCTGCACTGTCTCTGGATTATCTTGGAGCAGTAATTATGTAGGCTGGGTCCGCCAGGCTCCAGGAAA
AGGGCTGGAATTTGTTGGTACTATATATGGTAGTGCAAGTACAAACTACAACCCAGCCCTGAAGTCCCGAGCCAGCA
TCACCAAGGACACCTCAAAGAGCCAAGTTTATCTGACGCTGAACAGCCTGACAAGCGAGGACACGGCCGTCTATTAC
TGTGTAGGAGGAGCATATGGGTATGGTATAAACTACTGGGGCCAGGGCATCCTGGTCACCGTCTCCTCAGAGAGTAC
GATGACCCCAGATCTCTTCCCCCTCGTCTCCTGTGGGCCCTCTCTTGATGAGAGCCTGGTGGCTGTGGGCTGCCTAG
CCCGGGACTTCCTACCCAA
>KY437501.1 Equus caballus clone 345 immunoglobulin mu heavy chain (IGHM) mRNA, partial cds
CTGTCCCTCACCTGCACTGTCTCTGGATTCTCTTTGAGCAGTTACGGTGTAGGCTGGGTCCGCCAGGCTCCAGGAAA
AGGGTTGGAATATGTTGGTGGTATAACTAGTAGTGGAAGTGCAAACTACAACCCAGCCCTGAAGTCCCGAGCCAGCA
TCACCAAGGACACCTCAAAGAGCCAAGGCTATCTGACGCTGAACAGCCTGACAGGCGAGGACACGGCCGTCTATTAC
TGTGCGGGAAGAAGTGGTGCTTCGTACTTAGGTCCGAGTGGTCTAAACTACTGGGGCCAGGGCATCCTGGTCGCCGT
CTCCTCAGAGAGTACGATGACCCCAGATCTCTTCCCCCTCGTCTCCTGTGGGCCCTCTCTTGATGAGAGCCTGGTGG
CTGTGGGCTGCCTAGCCCGGGACTTCCTACCCAA
>KY437500.1 Equus caballus clone 344 immunoglobulin mu heavy chain (IGHM) mRNA, partial cds
CTCTCCCTCACCTGCACTGTCTCTGGATTATCTTTGAGCAGTAATTTTGTAGGCTGGGTCCGCCAGGCTCCAGGAAA
AGGGCTGGAATATGTTGGTGTTATAAGTAGTAGTGGAAGTTTAACGTACAACCCAGCCCTGAAGTCCCGAGCCAGCA
TCACCAAAGACACCTCAAAGAGCCAAGTTTATCTGACGCTGAACAGCCTGACAGGCGAGGACACGGCCGTCTATTAC
TGTACGAGATGGGATTGGGGTGGTAGTTCCTGGTACTCGGGGGGATTTGGCTACTGGGGCCAGGGCACCCTGGTCAC
CGTCTCCTCAGAGAGTACGATGACCCCAGATCTCTTCCCCCTCGTCTCCTGTGGGCCCTCTCTTGATGAGAGCCTGG
TGGCTGTGGGCTGCCTAGCCCGGGACTTCCTACCCAA
>KY437499.1 Equus caballus clone 343 immunoglobulin mu heavy chain (IGHM) mRNA, partial cds
CTCTCCCTCACCTGCACTGTCTCTGGATTATCTTTGAGCAGTAATCCTGTAGCCTGGGTCCGCCAGGCTCCAGGAAA
AGGGCTGGAATATGTTGGTGGTATAGCTAGTAGTGGAAGTGCAAACTACAACCCAGCCCTGAAGTCCCGAGCCAGCA
TCACCGCGGACACCTCAAAGAGCCAAGTTTATCTGACGCTGAACAGCCTGACAAGCGAGGACACGGCCGTCTATTAC

TGTACAGGAGACTACGATTCCTATGCTGGTGGTATAAAGTATTGGGGCCAGGGCATCCTGGTCACCGTCTCCTCAGA
GAGTACGATGACCCCAGATCTCTTCCCCCTCGTCTCCTGTGGGCCCTCTCTTGATGAGAGCCTGGTGGCTGTGGGCT
GCCTAGCCCGGGACTTCCTACCCAA
>KY437498.1 Equus caballus clone 342 immunoglobulin mu heavy chain (IGHM) mRNA, partial cds
CTCTCCCTCACCTGCACTGTCTCTGGATTGTCTTTGAGCGCTAATGCTGTAGGCTGGGTCCGCCAGGCTCTAGGAAA
AGGGCTGGAATATGTTGGTGGTATAGGTAGTAGTGGAAGTGCAATGTACAACCCAGCCCTGAAGTCCCGAGCCAGCA
TCACCAAGGACACCTCAAAGAGCCAAGTTTATCTGACGCTGAACAGCCTGACAAGCGAGGACACGGCCGTCTATTAC
TGTGCGACTTTTCCCTACTGGGGCCAGGGCACCCTGGTCACCGTCTCCTCAGAGAGTACGATGACCCCAGATCTCTT
CCCCCTCGTCTCCTGTGGGCCCTCTCTTGATGAGAGCCTGGTGGCTGTGGGCTGCCTAGCCCGGGACTTCCTACCCA
A
>KY437497.1 Equus caballus clone 341 immunoglobulin mu heavy chain (IGHM) mRNA, partial cds
CTCTCCCTCACCTGCACTGTCTCTGAATTATCTTTGAGCAGTAATGCTGTAGGCTGGGTCCGCCAGGCTCCAGGAAA
AGGGCTGGAATGGGTTGGTGGTATAGCTAGTAGTGGAAGTGCAGCCTACAACCCAGCCCTGAAGTCCCGAGCCAGCA
TCACCAAGGACACCTCAAAGAGCCAAGTTTATCTGACGCTGAACAGCCTGACAAGCGAGGACACGGCCGTCTATTAC
TGTGCAGGAGACGCAGATGGCTACGTGGGTCACTGGGGCCAGGGCACCCTGGTCACCGTCTCCTCAGAGAGTACGAA
GACCCCAGATCTCTTCCCCCTCGTCTCCTGTGGGCCCTCTCTTGATGAGAGCCTGGTGGCTGTGGGCTGCCTAGCCC
GGGACTTCCTACCCAA
>KY437496.1 Equus caballus clone 340 immunoglobulin mu heavy chain (IGHM) mRNA, partial cds
CTGTCCCTCACCTGCACTGTCTCTGGATTATCTTTGAGCAGTAATGCTGTAGGCTGGGTCCGCCAGGCTCCAGGAAA
AGGGCTGGAATTTGTTGGTTCTATATATGGTAGTGCAAGTGCAAACTACAACCCAGCCCTGAGGTCCCGAGCCAGCA
TCACCAAGGACACCTCAAAGAGCCAAGTTTATCTGACGCTGAACAGCCTGACAAGCGAGGACACGGCCGTCTATTAC
TGTGCAGGAGGCCTAGGCTATATCAGCTATGCTTACTACGAAGTACCCCGCTGGGGCCAGGGCACCCTGGTCACCGT
CTCCTCAGAGAGTACGATGACCCCAGATCTCTTCCCCCTCGTCTCCTGTGGGCCCTCTCTTGATGAGAGCCTGGTGG
CTGTGGGCTGCCTAGCCCGGGACTTCCTACCCAA
>KY437495.1 Equus caballus clone 339 immunoglobulin mu heavy chain (IGHM) mRNA, partial cds
CTGTCCCTCACCTGCACTGTCTCTGGATTCTCTTTGAGCAGTTACGGTGTAGGCTGGGTCCGCCAGGCTCCAGGAAA
AGGGCTGGAATATGTTGGTGGTATAGCTGTTAGTGGAAGTGCAAACTACAACCCAGCCCTGAAGTCCCGAGCCAGCA
TCACCAAGGACACCTCAAAGAGCCAAGTTTATCTGACGCTGAACAGCCTGACAGGCGAGGACACGGCCGTCTATTAC
TGTGCGAGACTCTGGTATGCTTATGGGCGTGATCACTACTACGTGTATGATATAGACTACTGGGGCCAGGGCACCCT
GGTCACCGTCTCCTCAGAGAGTACGATGACCCCAGATCTCTTCCCCCTCGTCTCCTGTGGGCCCTCTCTTGATGAGA
GCCTGGTGGCTGTGGGCTGCCTAGCCCGGGACTTCCTACCCAA
>KY437494.1 Equus caballus clone 338 immunoglobulin mu heavy chain (IGHM) mRNA, partial cds
CTCTCCCTCACCTGCACTGTCTCTGGATTATCTTTGAGCAGTAATGCTGTAGGCTGGGTCCGCCAGGCTCCAGGAAA
AGGGCTGGAATTTGTTTGTGACGTGGATAATAGTGGAAGTACAAACTACAACCCAGCCCTGAAGTCCCGAGCCAGCA
TCACCAAGGACACCTCAAAGAGCCAAGTTTATCTGACGCTGAACAGCCTGACAAGCGAGGACACGGCCGTCTATTAT
TGTGTAGGCGTTCCTTTTGCGGGTGCTTACTATTATTGGGGCCAGGGCACCCTGGTCACCGTCTCCTCAGAGAGTAC
GAAGACCCCAGATCTCTTCCCCCTCGTCTCCTGTGGGCCCTCTCTTGATGAGAGCCTGGTGGCTGTGGGCTGCCTAG
CCCGGGACTTCCTACCCAA
>KY437493.1 Equus caballus clone 337 immunoglobulin mu heavy chain (IGHM) mRNA, partial cds
CTCTCCCTCACCTGCACTGTCTCTGGATTATCTTTGAGCAGTAATGCTGTAGGCTGGGTCCGCCAGGCTCCAGGAAA
AGGGCTGGAATTTGTTGGTTATATATATGGTAGTGCAAGTGCAACTTACAACCCAGCCCTGAAGTCCCGAGCCAGCA
TCACCAAGGACACCTCAAAGAGCCAAGTTTATCTGACGCTGAACACGCTGACAAGCGAGGACACGGCCGTCTATTAC
TGTGTAAAATACTATAGTTATGATGATAACTATCAGGATGGCCACGTGCATCACTGGGGCCAGGGCACCCTGGTCAC
CGTCTCCTCAGAGAGTACGATGACCCCAGATCTCTTCCCCCTCGTCTCCTGTGGGCCCTCTCTTGATGAGAGCCTGG
TGGCTGTGGGCTGCCTAGCCCGGGACTTCCTACCCAA
>KY437492.1 Equus caballus clone 336 immunoglobulin mu heavy chain (IGHM) mRNA, partial cds
CTCTCCCTCACCTGCACTGTCTCTGGATTATCTTTGAGCAGTAATGCTGTAGGCTGGGTCCGCCAGGCTCCAGGAAA
AGGGCTGGAATATGTTGGTTGTATAGATAGTAGTGGAGGTGCAGACTACAACCCAGCCCTGGAGTCCCGAGCCAGCA
TCACCAAGGACACCTCAAAGAGCCAAGTTTATCTGACGCTGAACAGCCTGACAAGCGAGGACACGGCCGTCTATTAC

TGTGCAGGAGGCCTGGGGATGATTCTGCAGCCCTATAATATAGCCTACTGGGGCCAGGGCATCCTGGTCACCGTCTC
CTCAGAGAGTACGATGACCCCAGATCTCTTCCCCCTCGTCTCCTGTGGGCCCTCTCTTGATGAGAGCCTGGTGGCTG
TGGGCTGCCTAGCCCGGGACTTCCTACCCAA
>KY437491.1 Equus caballus clone 335 immunoglobulin mu heavy chain (IGHM)
mRNA, partial cds
CTCTCCCTCACCTGCACTGTCTCTGGATTCTCTTTGAGCAGTTATACTGTATACTGGGTCCGCCAGGCTCCAGGAAA
AGGGCTGGAATATGTTGGTATCATATATGTTAGTGGAAGTGCAAACTACAACCCAGCCCTGAAGTCCCGAGCCAGCA
TCACCAAGGACACCTCAAAGAGCCAAGTTTATCTGACGCTGAACAGCCTGACAGGCGAGGACACGGCCGTCTATTAC
TGTGCGAGATCTATCTTCGGCGAATTTGGCTACTGGGGCCAGGGCACCCTGGTCACCGTCTCCTCAGAGAGTACGAT
GACCCCAGATCTCTTCCCCCTCGTCTCCTGTGGGCCCTCTCTTGATGAGAGCCTGGTGGCTGTGGGCTGCCTAGCCC
GGGACTTCCTACCCAA
>KY437490.1 Equus caballus clone 334 immunoglobulin mu heavy chain (IGHM)
mRNA, partial cds
CTCTCCCTCACCTGCACTGTCTCTGGATTATCTTTGAGTAGTAATGGTGTGGTCTGGGTCCGCCAGGCTCCAGGAAA
AGGGCTGGAATTTGTTGGTGCTATAGCTGGTAGTGGAATTACATACTACAACCCAGCCCTGAAGTCCCGAGCCAGCA
TCACCAAGGACACCTCAAAGAGCCAAGTTTTTCTGACGCTGAACAGCCTGACAAGCGAATACACGGCCGTCTATTAC
TGTGGTCGTAACATTGTTACTATGTCGTATCCTGCCTACTGGGGCCAGGGCACCCTGGTCACCGTCTCCTCAGAGAG
TACGATGACCCCAGATCTCTTCCCCCTCGTCTCCTGTGGGCCCTCTCTTGATGAGAGCCTGGTGGCTGTGGGCTGCC
TAGCCCGGGACTTCCTACCCAA
>KY437489.1 Equus caballus clone 333 immunoglobulin mu heavy chain (IGHM)
mRNA, partial cds
CTCTCCCTCACCTGCACTGTCTCTGGATTATCTTTGAGTAGTATTGGTGTGGGCTGGGTCCGCCAGGCTCCAGGAAA
AGGGCTGGAATTTGTTGGTTATAGTGCTATTAGTGAAGGTGCAGGGTACAACCCAGCCCTGAAGTCCCGAGCCAGTG
TCACCAAGGACACCTCAAAGAGCCAAGTTTATCTGACGCTGAACAGCCTGACAACTGAGGACACGGCCGTCTATTAC
TGTGCAGGAGATCTGTATTCAAGTGGTGGTTTCCATGATATAGACTACTGGGGCCAGGGCACCCTGGTTACCGTCTC
CTCAGAGAGTACGATGACCCCAGATCTCTTCCCCCTCGTCTCCTGTGGGCCCTCTCTTGATGAGAGCCTGGTGGCTG
TGGGCTGCCTAGCCCGGGACTTCCTACCCAA
>KY437488.1 Equus caballus clone 332 immunoglobulin mu heavy chain (IGHM)
mRNA, partial cds
CTCTCCCTCACCTGCACTGTCTCTGGATTATCTTTGAGTAGTTATGGTGTGGGCTGGGTCCGCCAGGCTCCAGGAAA
AGGGCTGGAATTTGTAGGTGGTATGACTAGTAGTGGGAGTGCAAACTACAACCCAGCCCTGAAGTCCCGAGCCAGCA
TCACCAAGGACACCTCAAAGAGCCAACTTTATCTGACGCTGAACAGCCTGACAAGCGAGGACACGGCCGTCTATTAC
TGTACAGGAACTGGTTATGGTATTGACTCCTACTGGGGCCAGGGCACCCTGGTCACCGTCTCCTCAGAGAGTACGAT
GACCCCAGATCTCTTCCCCCTCGTCTCCTGTGGGCCCTCTCTTGATGAGAGCCTGGTGGCTGTGGGCTGCCTAGCCC
GGGACTTCCTACCCAA
>KY437487.1 Equus caballus clone 331 immunoglobulin mu heavy chain (IGHM)
mRNA, partial cds
CTCTCCCTCACCTGCACTGTCTCTGGATTCTCTTTGAGCAGTTATGAAGTATACTGGGTCCGCCAGGCTCCAGGAAA
AGGGCTGGAATATGTTGGTGATATACTGGACAGTGGAGGTGCACTATACAACCCAGCCCTGAAGTCCCGAGCCAGCA
TCACCAAGGACACCTCAAAGAGCCAAGTTTATCTGACGCTGAACAGCCTGACAGGCGAGGACACGGCCGTCTATTAC
TGTGCGAGATATGATTCCCTTGCTAGTAGTGGAATTGGTGCTATGGACCCCTGGGGCCAGGGCACCCTGGTCACCGT
CTCCTCAGAGAGTACGATGACCCCAGATCTCTTCCCCCTCGTCTCCTGTGGGCCCTCTCTTGATGAGAGCCTGGTGG
CTGTGGGCTGCCTAGCCCGGGACTTCCTACCCAA
>KY437486.1 Equus caballus clone 330 immunoglobulin mu heavy chain (IGHM)
mRNA, partial cds
CTCTCCCTCACCTGCACTGTCTCTGGATTCTCTTTGAGCAGTTATGCTGTATACTGGGTCCGCCAGGCTCCAGGAAA
AGGGCTGGAATATGTTGGTGACATATATGGTAGTGCAAACTACAACCCAGCCCTGAAGTCCCGAGCCAGCATCACCA
AGGACACCTCAAAGAGCCAAGTTTATCTGACGCTGAACAGCCTGACAAGCGAGGACACGGCCGTCTATTACTGTGCA
GGCTCTGATTATGGTACTATCGTTTATGGTATAAACTACTGGGGCCAGGGCATCCTGGTCACCGTCTCCTCAGAGAG
TACGATGACCCCAGATCTCTTCCCCCTCGTCTCCTGTGGGCCCTCTCTTGATGAGAGCCTGGTGGCTGTGGGCTGCC
TAGCCCGGGACTTCCTACCCAA
>KY437485.1 Equus caballus clone 329 immunoglobulin mu heavy chain (IGHM)
mRNA, partial cds
CTCGCAGACCCTCTCCCTCACCTGCACTGTCTCTGGATTATCTTTGAGCAGTAATGCTGTACACTGGGTCCGCCAGG
CTCCAGGAAAAGGGCTGGAATATGTTGGCGGTATAGCTCGTAGTGGAAGTGCAAACTACAACCCAGCCCTGAAGTCC

CGAGCCAGCATCACCAAGGACACCTCAAAGAGCCAAGTTTATCTGACGCTGAACAGCCTGACAAGCGAGGACACGGC
CGTCTATTACTGTGTAGGAGGTTATGGGCACTATGCTGGTAGTTACTTCTACTTCCTTGATATAAACTACTGGGGCC
AGGGCATCCTGGTCACCGTCTCCTCAGAGAGTACGATGACCCCAGATCTCTTCCCCCTCGTCTCCTGTGGGCCCTCT
CTTGATGAGAGCCTGGTGGCTGTGGGCTGCCTAGCCCGGGACTTCCTACCCAA
>KY437484.1 Equus caballus clone 328 immunoglobulin mu heavy chain (IGHM) mRNA, partial cds
CTCTCCCTCACCTGCACTGTCTCTGGATTATCTTTGAACAGTAATGCTGTAGGCTGGGTCCGCCAGGCTCCAGGAAA
AGGGCTGGAATTTGTTGGTGTTATATATGGTAGTGCAAGTGCAAACTACAACCCAGCCCTGAAGTCCCGAGCCAGCA
TCACCAAGGACACCTCAAAGAGCCAAGTTTATCTGACGCTGAACAGCCTGACAAGCGAGGACACGGCCGTCTATTAC
TGTGCAGGAGTCCCCTATGGTGATATTTTCTGGAATATGGAGTTTATAAACTACTGGGGCCAGGGCATCCTGGTCAC
CGTCTCCTCAGAGAGTACGATGACCCCAGATCTCTTCCCCCTCGTCTCCTGTGGGCCCTCTCTTGATGAGAGCCTGG
TGGCTGTGGGCTGCCTAGCCCGGGACCTCCTACCCAA
>KY437483.1 Equus caballus clone 327 immunoglobulin mu heavy chain (IGHM) mRNA, partial cds
CTCTCCCTCACCTGCACTGTCTCTGGATTCTCTTTGAGCAGTGTTAATGTATGGTGGGTCCGCCAGGCTCCAGGAAA
AGGGCTGGAATATGTTGGTCAAATAAGTGGTGTTGGAGGTACAACTTACAACCCGTCCCTGAAGTCCCGAGCCAGCA
TCACCAAGGACACCTCAAAGAGCCAAGTTTATCTGACGCTGAACAGCCTGACAGGCGAGGACACGGCCGTCTATTAC
TGTGCGGGACCCTATGTTGATGGAGGTCGTTACTACGGACTTGGCTACTGGGGCCAGGGCACCCTGGTCACCGTCTC
CTCAGAGAGTACGATGACCCCAGATCTCTTCCCCCTCGTCTCCTGTGGGCCCTCTCTTGATGAGAGCCTGGTGGCTG
TGGGCTGCCTAGCCCGGGACTTCCTACCCAA
>KY437482.1 Equus caballus clone 326 immunoglobulin mu heavy chain (IGHM) mRNA, partial cds
CTCTCCCTCACCTGCTCTGTGTCTGGAGTCTCCATCACAAGCAGTGGTGACTGGTGGAGCTGGATCCGCCAGCCCCC
AGGGAAGGGGCTGGAATGGATGGGGTACATAAGTTATAGTGGTAGCGCTTACTACACCACATCCCTCAAGAGCCGAC
TCTCCATCTCCAGAGACACGTCCAAGGACCAGTTCTCCCTGCAGCTGAGCTCCGTGACCGCCGAGGACACGGCCGTT
TATTACTGTGCAAGGAAGCTGACCGGTTATGGTGGTGCTTATTATAATGCTATGGACCCCTGGGGCCAGGGCACCCT
GGTCACCGTCTCCTCAGAGAGTACGATGACCCCAGATCTCTTCCCCCTCGTCTCCTGTGGGCCCTCTCTTGATGAGA
GCCTGGTGGCTGTGGGCTGCCTAGCCCGGGACTTCCTACCCAA
>KY437481.1 Equus caballus clone 325 immunoglobulin mu heavy chain (IGHM) mRNA, partial cds
CTCTCCCTCACCTGCACTGTCTCTGGATTCTCTTTGAGCAGTTATGCTGTACACTGGGTCCGCCAGGCTCCAGGAAA
AGGGCTGGAATATGTTGGTAGTTTCGATGATGGTGGACGTGAAAACTACAACCCAGCCCTGAAGTCCCGAGCCAGCA
TCACCAAGGACACCTCAAAGGGCCAAGTTTATCTGACGCTGCACAGCCTGACAAGCGAGGACACGGCCGTCTATTAC
TGTACGAATTTGGGAGGCTACTGGGGCCAGGGCACCCTGGTCACCGTCTCCTCAGAGAGTACGATGACCCCAGATCT
CTTCCCCCTCGTCTCCTGTGGGCCCTCTCTTGATGAGAGCCTGGTGGCTGTGGGCTGCCTAGCCCGGGACTTCCTAC
CCAA
>KY437480.1 Equus caballus clone 324 immunoglobulin mu heavy chain (IGHM) mRNA, partial cds
CTCTCCCTCACCTGCACTGTCTCTGGATTATCTTTGAGCAATCGTGCTGTAGGCTGGGTCCGCCAGGCTCCAGGAAA
AGGGCTGGAATATGTGGGTACTAAAATTAGTAGTGGCAGTGAAGTGTACAACCCAGCCCTGAAGTCCCGAGCCAGCA
TCACCAAGGACACCTCAAAGAGCCAAGTTTATCTGACGCTGAACAGCCTGACAAGCGAGGACACGGCCGTCTATTAC
TGTAGAGTTGTGGATGGTGGTGCTTACTTGTGGGGCCAGGGCACCCTGGTCACCGTCTCCTCAGAGAGTACGATGAC
CCCAGATCTCTTCCCCCTCGTCTCCTGTGGGCCCTCTCTTGATGAGAGCCTGGTGGCTGTGGGCTGCCTAGCCCGGG
ACTTCCTACCCAA
>KY437479.1 Equus caballus clone 323 immunoglobulin mu heavy chain (IGHM) mRNA, partial cds
CTCTCCCTCATCTGCACTGTCTCTGGAATATCTTTGAGTAGTTATGGTGTGGGCTGGGTCCGCCAGGCTCCCGGAAA
AGGGCTGGAATTTGTTGCCGCATTAGGTAGTAGTGGAAGTGCTTACTACAACCCAGCCCTGAAGTCCCGAGCCAGCA
TCACCAAGGACACCTCAAAGAGCCAAGTTTATCTGACGCTGAACAGCCTGACAAGCGAGGATACGGCCGTCTATTAC
TGTGCAGGAGGAATGCTTCATTATGAAGGCTACTGGGGCCAGGGCACCCTGGTCACCGTCTCCTCAGAGAGTACGAT
GACCCCAGATCTCTTCCCCCTCGTCTCCTGTGGGCCCTCTCTTGATGAGAGCCTGGTGGCTGTGGGCTGCCTAGCCC
GGGACTTCCTACCCAA
>KY437478.1 Equus caballus clone 322 immunoglobulin mu heavy chain (IGHM) mRNA, partial cds
CTCTCCCTCACCTGTACTGTCTCTGGATTTTCTTTGAGTAGTTATGGTGTGGGCTGGGTCCGCCAGGCTCCAGGAAA
AGGGCTGGAATTTGTTAGTGAAATAGGTGGTAGTGGAAAACTAAGGTACAATCCAGCCCTGAAGTCCCGAGTCAGTA

TCACCAAGGACACCTCAAAGAGTCAAGCCTATCTGACGCTGAACAGCCTGACAAGCGAGGACACGGCCGTCTATTAC
TGTGTAGGAGGTGGTTCGATTATGAATGGACTTGACTACTGGGGCCAGGGCACCCTGGTCACCGTCTCCTCAGAGAG
TACGATGACCCCAGATCTCTTCCCCCTCGTCTCCTGTGGGCCCTCTCTTGATGAGAGCCTGGTGGCTGTGGGCTGCC
TAGCCCGGGACTTCCTACCCAA
>KY437477.1 Equus caballus clone 321 immunoglobulin mu heavy chain (IGHM) mRNA, partial cds
CTCTCCCTCACCTGCACTGTCTCTGGATTATCTTTGAGCAGTAATGCTGTAGGCTGGGTCCGCCAGGCTCCAGGAAA
AGGGCTGGAATTTGTTGGTGCTATATATGGTAGTGCAAGTGCAAACTACAACCCAGCCCTGAAGTCCCGAGCCAGCA
TCACCAAGGACACCTCAAAGAGCCAAGTTTATCTGACGCTGAACAGCCTGACAAGCGAGGACACGGCCGTCTATTAC
TGTGCAGGATGGTTGTCCGATGACTATGGTGATACTTTCTACCACACCGAATATGGTATAAACTACTGGGGCCAGGG
CATCCTGGTCACCGTCTCCTCAGAGAGTACGATGACCCCAGATCTCTTCCCCCTCGTCTCCTGTGGGCCCTCTCTTG
ATGAGAGCCTGGTGGCTGTGGGCTGCCTAGCCCGGGACTTCCTACCCAA
>KY437476.1 Equus caballus clone 320 immunoglobulin mu heavy chain (IGHM) mRNA, partial cds
CTCTCCCTCACCTGCACTGTCTCTGGATTATCTTTGATCAGTAATACTGTAGGCTGGGTCCGCCAGGCTCCAGGAGA
AGGGCTGGAATATGTTGGTGGTATACCTACTGGTGGAAGTGCAAACTACAACCCAGCCCTGAAGTCCCGAGCCAGCA
TCACCAAGGACACCTCAAAGAGCCAAGTTTATCTGACGCTGAACAGCCTGACAAGCGAGGACACGGCCGTCTATTAC
TGTACAGGAGGCCTTATGTACGATAGTGGATATGTTATAAAGTACTGGGGCCAGGGCATCCTGGTCACCGTCTCCTC
AGAGAGTACGATGACCCCAGATCTCTTCCCCCTCGTCTCCTGTGGGCCCTCTCTTGATGAGAGCCTGGTGGCTGTGG
GCTGCCTAGCCCGGGACTTCCTACCCAA
>KY437475.1 Equus caballus clone 319 immunoglobulin mu heavy chain (IGHM) mRNA, partial cds
CTCTCCCTCACCTGCACTGTCTCTGGATTCTCTTTGAGCAGTTATGATATATACTGGGTCCGCCAGGCTCCAGGAAA
AGGGCTGGAATATGTTGGTTTTATACATGCTAGTGCAAGTGCAATCTACAACCCAGCCCTGAAGTCCCGAGCCAGCA
TCACCGAGGACACCTCAAAGAGCCAAGTTTATCTGACGCTGAACAGCCTGACAGGCGAGGACACGGCCGTCTATTAC
TGTGCGAGACGGAAAAACTTGTACGGTTATGGTCTTGATTTTGGCTACTGGGGCCAGGGCACCCTGGTCACCGTCTC
CTCAGAGAGTACGATGACCCCAGATCTCTTCCCCCTCGTCTCCTGTGGGCCCTCTCTTGATGAGAGCCTGGTGGCTG
TGGGCTGCCTAGCCCGGGACTTCCTACCCAA
>KY437474.1 Equus caballus clone 318 immunoglobulin mu heavy chain (IGHM) mRNA, partial cds
CTCTCCCTCACCTGCACTGTCTCTGGATTCTCTTTGAGCAGTTATGATGTATACTGGGTCCGCCAGGCTTCAGGAAA
AGGGCTGGAATATGTTGGTGCAGTATATGGTAGTGGAAGTGCAAACTACAACCCAGCCCTGAAGTCCCGAGCCAGCA
TCACCAAGGACACCTCAAAGAGCCAAGTTTATCTGACGCTGAACAGCCTGACAGGCGAGGACACGGCCGTCTATTAC
TGTACAAGACCTGCTTCGTTTTACTGGGGCCAGGGCACCCTGGTCACCGTCTCCTCAGAGAGTACGATGACCCCAGA
TCTCTTCCCCCTCGTCTCCTGTGGGCCCTCTCTTGATGAGAGCCTGGTGGCTGTGGGCTGCCTAGCCCGGGACTTCC
TACCCAA
>KY437473.1 Equus caballus clone 317 immunoglobulin mu heavy chain (IGHM) mRNA, partial cds
CTCTCCCTCACCTGCACTGTCTCTGGATTATCTTTGAGCAGTAATGCTGTAGGCTGGGTCCGCCAGGCTCCAGGAAA
AGGGCTGGAATATGTTGGTCGTATAGCTAGTAGTGGAAGTGCTTACTACAACCCAGCCCTGAAGTCCCGAGCCAGCA
TCACCAAGGACACCTCAAAGAGCCAAGTTTATCTGACGCTGCACAGCCTGACAAGCGAGGACACGGCCGTCTATTAC
TGTACGAATTTGGGAGGCTACTGGGGCCAGGGCACCCTGGTCACCGTCTCCTCAGAGAGTACGATGACCCCAGATCT
CTTCCCCCTCGTCTCCTGTGGGCCCTCTCTTGATGAGAGCCTGGTGGCTGTGGGCTGCCTAGCCCGGGACTTCCTAC
CCAA
>KY437472.1 Equus caballus clone 316 immunoglobulin mu heavy chain (IGHM) mRNA, partial cds
CTCTCCCTCACCTGCACTGTCTCTGGATTATCTTTGAGCAGTAATGCTGTAGGCTGGGTCCGCCAGGCTCCAGGAAA
AGGGCTGGAATACATTGGTGATATATATGGTAGTGTGAGTGCAGACTATAACCCAGCCCTGAAGTCCCGAGCCAGCA
TCACCAAGGGCACCTCAAAGAGCCAAGCTTACCTGACGCTGAACAGCCTGACAAGTGAAGACACGGCCGTCTATTAC
TGTGCAGGAGGCGACGAATACGGGTCTATAAAATCGTGGGGCCAGGGCATCCTGGTCACCGTCTCCTCAGAGAGTAC
GATGACCCCAGATCTCTTCCCCCTCGTCTCCTGTGGGCCCTCCCTTGATGAGAGCCTGGTGGCTGTGGGCTGCCTAG
CCCGGGACTTCCTACCCAA
>KY437471.1 Equus caballus clone 315 immunoglobulin mu heavy chain (IGHM) mRNA, partial cds

CTCTCCCTCACCTGCACTGTCTCTGGATTATCTTTGAGCAGTAATTCTATAAAGTGGGTCCGCCAGGCTCCAGGAAA
AGGGCTGGAATATGTGGGTAAGATGAGTAGTAGTGGAAGTGCTTGGTACAACCCAGCCCTGAAGTCCCGAGCCAGCA
TCACCAAGGACACCTCAAAGAGCCAAGTTTATCTGACGCTGAACAGCCTGACAAGCGAGGACACGGCCGTCTATTAC
TGTGCGGGTGTTGTAAGCTGGGGCCAGGGCACCCTGGTCACCGTCTCCTCAGAGAGTACGATGACCCCAGATCTCTT
CCCCCTCGTCTCCTGTGGGCCCTCTCTTGATGAGAGCCTGGTGGCTGTGGGCTGCCTAGCCCGGGACTTCCTACCCA
A
>KY437470.1 Equus caballus clone 314 immunoglobulin mu heavy chain (IGHM)
mRNA, partial cds
CTCTCCCTCACCTGCACTGTCTCTGGATTATCTTTGAGTAGTAATGGTGTGGGCTGGGTCCGCCAGGCTCCAGGAAA
AGGGCTGGAATTTGTTGGTGGTATAGCTGCTAGTGGAGGTGCCAACTACAACCCAGCCCTGAAGTCCCGAGCCAGCA
TCACCAAGGACACCTCAAAGAGCCAAGTTTATCTGACGCTGAACAGCCTGACAAGCGAGGACACGGCCGTCTATTAC
TGTGTAGAAATAGACGCTTATGGTTATGTGGCTGACTACGTGGATCACTGGGGCCAGGGCACCCTGGTCACCGTCTC
CTCAGAGAGTACGATGACCCCAGATCTCTTCCCCCTCGTCTCCTGTGGGCCCTCTCTTGATGAGAGCCTGGTGGCTG
TGGGCTGCCTAGCCCGGGACTTCCTACCCAA
>KY437469.1 Equus caballus clone 313 immunoglobulin mu heavy chain (IGHM)
mRNA, partial cds
CTCTCCCTCACCTGCACTGTCTCTGGATTCTCTTTGACCAGTAGTATTATATACTGGGTCCGCCAGGCTCCAGGAAA
AGGGCTGGAATATGTTGGTGCGATGGATGGTAGTGCAAATACAGTGTACAACCCAGCCCTGAAGTCCCGAACCAGCA
TCAGTAAGGACACCTCAAAGAGCCAAGTTTATCTGACGCTGAACAGCCTGACAAGCGAGGACACGGCCGTCTATTTC
TGTGCAGGAGTGCTAAACGATTATTACTGCGTGAATCACTGGGGCCAGGGCACCCTGGTCACCGTCTCCTCAGAGAG
TACGATGACCCCAGATCTCTTCCCCCTCGTCTCCTGTGGGCCCTCTCTTGATGAGAGCCTGGTGGCTGTGGGCTGCC
TAGCCCGGGACTTCCTACCCAA
>KY437468.1 Equus caballus clone 312 immunoglobulin mu heavy chain (IGHM)
mRNA, partial cds
CTCTCCCTCACCTGCACTGTCTCTGGATTATCTTTGAGCAGTAGTGCTGTAGGCTGGGTCCGCCAGGCTCCAGGAAA
AGGGCTGGAATTTGTTGGTGGTATAGTTAGTAGTGGAAGTGCAAAGTACAACCCAGCCCTGAAGTCCCGAGCCAGCA
TCACCAAGGACACCTCAAAGAGCCAAGTTTATCTGACGCTGAATAGCCTGACAAGCGAGGACACGGCCGTCTATTAC
TGTGCAGGAGGAAACGGTTATGGTTATTCCGCGTTATTCGGCTACTGGGGCCAGGGCACCCTGGTCACCGTCTCCTC
AGAGAGTACGATGACCCCAGATCTCTTCCCCCTCGTCTCCTGTGGGCCCTCTCTTGATGAGAGCCTGGTGGCTGTGG
GCTGCCTAGCCCGGGACTTCCTACCCAA
>KY437467.1 Equus caballus clone 311 immunoglobulin mu heavy chain (IGHM)
mRNA, partial cds
CTCTCCCTCACCTGCACTGTCTCTGGATTATCTTTGAGCAGTAATGCTGTAGGCTGGGTCCGCCAGGCTCCAGGAAA
AGGGCTGGAATTTGTTGGTGCTATATATGGTGATTTAAGTACAAACTACAACCCAGCCCTGAAGTCCCGAGCCAGCA
TCACCAAGGACACCTCAAAGAGCCAAGTTTATCTGACGCTGAACAGCCTGACAAGCGAGGACACGGCCGTCTACTAC
TGTCGAGGAGGCACTTACTGGGGCCAGGGCACCCTGGTCACCGTCTCCTCAGAGAGTACGATGACCCCAGATCTCTT
CCCCCTCGTCTCCTGTGGGCCCTCTCTTGATGAGAGCCTGGTGGCTGTGGGCTGCCTAGCCCGGGACTTCCTACCCA
A
>KY437466.1 Equus caballus clone 310 immunoglobulin mu heavy chain (IGHM)
mRNA, partial cds
CTCTCCCTCACCTGCACTGTCTCTGGATTATCTTTGAGTAGTTATGGTGTGGGCTGGGTCCGCCAGGCTCCAGGAAA
AGGGCTGGAATTTGTTGGTGGTATAGCTAGTAGTGGAAGTGCAAACTACAACCCAGCCCTGAAGTCCCGAGCCAGCA
TCACCAAGGACACCTCAAAGAGCCAAGTTTATCTGACGCTGAACAGCCTGACAAGCGAGGACACGGCCGTCTATTAC
TGTGCTCTAGGTGACGGATCGTCTCATTTTGAATACTGGGGCCAGGGCACCCTGGTCACCGTCTCCTCAGAGAGTAC
GATGACCCCAGATCTCTTCCCCCTCGTCTCCTGTGGGCCCTCTCTTGATGAGAGCCTGGTGGCTGTGGGCTGCCTAG
CCCGGGACTTCCTACCCAA
>KY437465.1 Equus caballus clone 309 immunoglobulin mu heavy chain (IGHM)
mRNA, partial cds
CTCTCCCTCACCTGCACTGTCTCTGGATTATCTTTGAGCAGTAATGCTGTAGGCTGGGTCCGCCAGGCTCCAGGAAA
AGGGCTGGAATTTGTTGGTGCTATATATGGTAGTGCAAGTGCAAACTACAACCCAGCCCTGAAGTCCCGAGCCAGCA
TCACCAAGGACACCTCAAAGAGCCAAGTTTATCTGACGCTGAACAGCCTGACAAGCGAGGACACGGCCGTCTATTAC
TGTGCAGGAGCCTACGGTTATGGTTATGCTACGGATGCTATGGACCCCTGGGGCCAGGGCACCCTGGTCACCGTCTC
CTCAGAGAGTACGATGACCCCAGATCTCTTCCCCCTCGTCTCCTGTGGGCCCTCTCTTGATGAGAGCCTGGTGGCTG
TGGGCTGCCTAGCCCGGGACTTCCTACCCAA
>KY437464.1 Equus caballus clone 308 immunoglobulin mu heavy chain (IGHM)
mRNA, partial cds

CTCTCCCTCACCTGCACTGTCTCTGGATTATCTTTGAGCAGTAATGCTGTAGGCTGGGTCCGCCAGGCTCCAGGAAA
AGGGCTGGAATATGTTGGTAGTATACGTAGTAGTGGAAGTGCAAACTACAACCCAGCCCTGAAGTCCCGAGCCAGCA
TCACCAAGGACACCTCAAAGAGCCAAGTCTATCTGACGCTGAACAGCCTGACAAGCGAGGACACGGCCGTCTATTAC
TGTGCAGATGGTTATGGGCGTCCTTACTACTACTGGGGCCAGGGCATCCTGGTCACCGTCTCCTCAGAGAGTACGAT
GACCCCAGATCTCTTCCCCCTCGTCTCCTGTGGGCCCTCTCTTGATGAGAGCCTGGTGGCTGTGGGCTGCCTAGCCC
GGGACTTCCTACCCAA
>KY437463.1 Equus caballus clone 307 immunoglobulin mu heavy chain (IGHM)
mRNA, partial cds
CTCTCCCTCACCTGCTCTGTCTCTGGATTATCTTTGAGCAGTAATGCTGTAGGCTGGGTCCGCCAGGCTCCAGGAAA
AGGGCTGGAATTTGTTGGTGCTATACATGGTAGTGCAAGTGCAAACTACAACCCAGCCCTGAAGTCCCGAGCCAGCA
TCACCAAGGACACCTCAAAGAGCCAAGTTTATCTGACGCTGAACAGCCTGACAAGCGAGGACACGGCCGTCTATTAC
TGTGTAGGAGGAGTCGCGGATTTCTTCTATGGTTATGGTGGCCAGGGCACCCTGGTCACCGTCTCCTCAGAGAGTAC
GATGACCCCAGATCTCTTCCCCCTCGTCTCCTGTGGGCCCTCTCTTGATGAGAGCCTGGTGGCTGTGGGCTGCCTAG
CCCGGGACCTCCTACCCAA
>KY437462.1 Equus caballus clone 306 immunoglobulin mu heavy chain (IGHM)
mRNA, partial cds
CTCTCCCTCACCTGCACTGTCTCTGGATTATCTTTGAGTAGTTATGGTGTGGGCTGGGTCCGCCAGGCTCCAGGAAA
AGGGCTGGAATTTGTTGGTGGTATAGCTAGTAGTGGAAGTGCAAACTACAACCCAGCCCTGAAGTCCCGAGCCAGCA
TCACCAAGGACACCTCAAAGAGCCAAGTTTATCTGACGCTGAACAGCCTGACAAGCGAGGACACGGCCGTCTATTAC
TGTGCAGGAGGCCGCCTAGAAATGGTTACTATGCTGGTAGTTACTATCTCGAATTTTGGCTACTGGGGCCAGGGCAC
CCTGGTCACCGTCTCCTCAGAGAGTACGATGACCCCAGATCTCTTCCCCCTCGTCTCCTGTGGGCCCTCTCTTGATG
AGAGCCTGGTGGCTGTGGGCTGCCTAGCCCGGGACTTCCTACCCAA
>KY437461.1 Equus caballus clone 305 immunoglobulin mu heavy chain (IGHM)
mRNA, partial cds
CTCTCCCTCACCTGCACTGTCTCTGGATTATCCGCGAGCAGAGATGATGTAGGCTGGGTCCGCCAGGCTCCAGGAAA
AGGGCTGGAATTTGTTGGTTTTATACAAGATGATGCAAGTGCAAGCTACAACCCAGCCCTGAAGTCCCGAGCCAGCA
TCACCAAGGACACCTCAAAGAGCCAAGTTTATCTGACGCTGAACAGCCTGACAAGCGAGGACACGGCCGTCTATTAT
TGTGCAGGAAGTCAGTACTACGTCTATGGTATAGACTACTGGGGCCAGGGCATCCTGGTCACCGTCTCCTCAGAGAG
TACGATGACCCCAGATCTCTTCCCCCTCGTCTCCTGTGGGCCCTCTCTTGATGAGAGCCTGGTGGCTGTGGGCTGCC
TAGCCCGGGACTTCCTACCCAA
>KY437460.1 Equus caballus clone 304 immunoglobulin mu heavy chain (IGHM)
mRNA, partial cds
CTCTCCCTCACCTGCACTAGCTCTGGATTATCTTTGACTAGTTATGAAGTGGGCTGGGTCCGCCAGGCTCCAGGAAA
AGGGCTGGAATTTGTTGGTGCCTTAGGTAGTAGTGGAAATACCTACTACAACCCAGCCCTGAAGTCCCGAGCCAGCA
TCACCAAGGACACCTCAAAGAGCCAAGTTTATCTGACGCTGAACAGCCTGACAAGCGAGGACACGGCCGTCTATTAC
TGTGGAGGAGGCGGTTTTGTCTACTGGGGCCAGGGCACCCTGGTCACCGTCTCCTCAGAGAGTACGATGACCCCAGA
TCTCTTCCCCCTCGTCTCCTGTGGGCCCTCTCTTGATGAGAGCCTGGTGGCTGTGGGCTGCCTAGCCCGGGACTTCC
TACCCAA
>KY437459.1 Equus caballus clone 303 immunoglobulin mu heavy chain (IGHM)
mRNA, partial cds
CTCTCCCTCACCTGCACTGTCTCTGGATTATCTTTGAGTAGTTATGGTGTGGGCTGGGTCCGCCAGGCTCCAGGAAA
AGGGCTGGAATTTGTTGGTGGTATAGCTAGTAGTGGAAGTGCAAACTACAACCCAGCCCTGAAGTCCCGAGCCAGCA
TCACCAAGGACACCTCAAAGAGCCAAGTTTATCTGACGCTGAACAGCCTGACAAGCGAGGACACGGCCGTCTATTAC
TGTGCAGGAGCCTACGGTTATGGTTATGCTACGGATGCTATGGACCCCTGGGGCCAGGGCACCCTGGTCACCGTCTC
TTCAGAGAGTACGATGACCCCAGATCTCTTCCCCCTCGTCTCCTGTGGGCCCTCTCTTGATGAGAGCCTGGTGGCTG
TGGGCTGCCTACCCGGGACTTCCTACCCAA
>KY437458.1 Equus caballus clone 302 immunoglobulin mu heavy chain (IGHM)
mRNA, partial cds
CTCTCCCTCACCTGCACTGTCTCTGGATTATCTTTGAGTGTTTATGGTGTGGGCTGGGTCCGCCAGGCTCCAGGAAA
AGGGCTGGAATTTGTTGGTGGTATAGCTAGAAGTGGAAGTGCAAACTACAACCCAGCCCTGAAGTCCCGAGCCAGCG
TCACCAAGGACACCTCAAAGAGCCAAGTTTATCTGACGCTGAACAGCCTGACAAGCGAGGACACGGCCGTCTATTAC
TGTGCAGGATACGGTAGTTACTATTTTGACTACTGGGGCCAGGGCACCCTGGTCACCGTCTCCTCAGAGAGTACGAT
GACCCCAGATCTCTTCCCCCTCGTCTCCTGTGGGCCCTCTCTTGATGGGAGCCTGGTGGCTGTGGGCTGCCTAGCCC
GGGACTTCCTACCCAA

>KY437457.1 Equus caballus clone 301 immunoglobulin mu heavy chain (IGHM) mRNA, partial cds
CTCTCCCTCACCTGCACTGTCTCTGGATTATCTTTGATGAGTAATCCTGCAGGCTGGGTCCGCCAGGCTCCAGGAAG
AGGGCTGGAATATGTTGGTTCAATAGGTAGTGCTGGAAGTACAGAGTACAACCCAGCCCTGAAGTCCCGAGCCAGCA
TCACCAAGGACACCTCAAAGAGCCAAGTTTATCTGACGCTGAATAGCCTGACAAGCGAGGACACGGCCGTCTATTAT
TGTCGTGCCGGGGGCGCCTGGGGCCAGGGCACCCTGGTCACCGTCTCCTCAGAGAGTACGATGACCCCAGATCTCTT
CCCCCTCGTCTCCTGTGGGCCCTCTCTTGATGAGAGCCTGGTGGCTGTGGGCTGCCTAGCCCGGGACTTCCTACCCA
A
>KY437456.1 Equus caballus clone 300 immunoglobulin mu heavy chain (IGHM) mRNA, partial cds
CTCTCCCTCACCTGCACTGTCTCTGGATTATCTTTGAGTAGTTGGAGTGTGGGCTGGGTCCGCCAGGCTCCAGGAAA
AGGGCTGGAATTTGTTGGTGGTATAGAAAGTAGTGGAAGTGCAAACTACAACCCAGCCCTGAAGTCCCGAGCCAGCA
TCACCAAGGACACCTCAAAGAGCCAAGTTTATCTGACGCTGCACAGCCTGACAAGCGAGGACACGGCCGTCTATTAC
TGTGCAGGAGGCGGTATTGCGAGCAGCAATGCTTGGTTTGCCTACTGGGGCCAGGGCACCCTGGTCACCGTCTCCTC
AGAGAGTACGATGACCCCAGATCTCTTCCCCCTCGTCTCCTGTGGGCCCTCTCTTGATGAGAGCCTGGTGGCTGTGG
GCTGCCTAGCCCGGGACTTCCTACCCAA
>KY437455.1 Equus caballus clone 299 immunoglobulin mu heavy chain (IGHM) mRNA, partial cds
CTCTCCCTCACCTGCACTGTCTCTGGATTATCTTTGAGTAGTTATGGTGTGGGCTGGGTCCGCCAGGCTCCAGGAAA
AGGGCTGGAATTTGTTGGTGGTATAGCTAGTAGTGGAAGTGCAAACTACAACCCAGCCCTGAAGTCCCGAGCCAGCA
TCACCAAGGACACCTCAAAGAGCCAAGTTTATCCGACGCTGAACAGCCTGACAAGCGAGGACACGGCCGTCTATTAC
TGTGCAGGAGAAGTTACTATGGTGGTAGTTCCTGGTACTCTCGGATTTGGCTACTGGGGCCAGGGCACCCTGGTCAC
CGTCTCCTCAGAGAGTACGATGACCCCAGATCTCTTCCCCCTCGTCTCCTGTGGGCCCTCTCTTGATGAGAGCCTGG
TGGCTGTGGGCTGCCTAGCCCGGGACTTCCTACCCAA
>KY437454.1 Equus caballus clone 298 immunoglobulin mu heavy chain (IGHM) mRNA, partial cds
CTCTCCCTCACCTGCACTGTCTCTGGATTATCTTTGAGCAGTAATTATGTAGGCTGGGTCCGCCAGGCTCCAGGAAA
AGGGCTGGAATATGTTGGTGTTATACGTAGTGGTGGAAGTGCAGCGTACAACCCAGCCCTGAAGTCCCGAGCCAGCA
TCACCCAGGACACCTCAAAGAGCCAAGTTTATCTGACGCTGAACAGCCTGACAAGCGAGGACACGGCCGTCTATTAC
TGTGCAGGAGTCTACGGTTATGATTATTGTTTTGGCTACTGGGGCCAGGGCACCCTGGTCACCGTCTCCTCAGAGAG
TACGATGACCCCAGATCTCTTCCCCCTCGTCTCCTGTGGGCCCTCTCTTGATGAGAGCCTGGTGGCTGTGGGCTGCC
TAGCCCGGGACTTCCTACCCAA
>KY437453.1 Equus caballus clone 297 immunoglobulin mu heavy chain (IGHM) mRNA, partial cds
CTCTCCCTCACCTGCACTGTCTCTGGATTAACTTTGAGCAGTAACGCTGTAGGCTGGGTCCGCCAGGTTCCAGGAAA
AGGGCTGGAATTGGTTGGTGCAGTATATGGTGGTGCAGGTACAACGTACAACCCAGCCCTGAAGTCCCGAGCCAGCA
TCACCAAGGACACCTCAAAGAGCCAAGTTTATCTGACGCTGAACAGCCTGACAAGCGAGGACACGGCCGTCTATTAC
TGTGCAGGTAGACATCAAAAGGCAGCTATGCTTACTCGGGATGGTATAAACTACTGGGGCCAGGGCATCCTGGTCAC
CGTCTCCTCAGAGAGTACGATGACCCCAGATCTCTTCCCCCTCGTCTCCTGTGGGCCCTCTCTTGATGAGAGCCTGG
TGGCTGTGGGCTGCCTAGCCCGGGACTTCCTACCCAA
>KY437452.1 Equus caballus clone 296 immunoglobulin mu heavy chain (IGHM) mRNA, partial cds
CTCTCCCTCACCTGCACTGTCTCTGGATTATCTTTGGGCAGTGATGCTGTAGGCTGGGTCCGCCAGGCTCCAGGAAA
AGGGCTGGAATATGTTGGTGGTATGCTAAGTTTCAGTGAAAGTGCAAAGTACAACCCAGCCCTGAAGTCCCGAGCCA
GCATCATTAAGGACACCTCAAAGAGCCAAGTTTATCTGACAATGAACAGCCTGACAAGCGAGGACACGGCCGTCTAT
TACTGTGTAGGAGGTTTGGAAACGGTTCTGCGACTGCTACCTGGTATCAGTTATTGGGGCCAGGGCATCCTGGTCAC
CGCCTCGCCAGAGAGTACGATGACCCCAGATCTCTTCCCCCTCGTCTCCTGTGGGCCCTCTCTTGATGAGAGCCTGG
TGGCTGTGGGCTGCCTAGCCCGGGACTTCCTACCCAA
>KY437451.1 Equus caballus clone 295 immunoglobulin mu heavy chain (IGHM) mRNA, partial cds
CTCTCCCTCACCTGCACTGTCTCTGGATTATCTTTGAGCAGTAATGCTGTAGGCTGGGTCCGCCAGGCTCCAGGAAA
AGGGCTGGAATATGTTGGTGGTATAGCTAGTAGTGGAAGTGCAAGCTACAACCCAGCCCTGAAGTCCCGAGCCAGCA
TCACCAAGGACACGGCAAAGAGCCAAGTTTATCTGACGCTGAACAGCCTGACAAGCGAGGACACGGCCGTCTATTAC
TGTGCAGGACCTAGCTACCTTACGGTTATGCTACCTGCCTACTGGGGCCAGGGCACCCTGGTCACCGTCTCCTCAGA
GAGTACGATGACCCCAGATCTCTTCCCCCTCGTCTCCTGTGGGCCCTCTCTTGATGAGAGCCTGGTGGCTGTGGGCT
GCCTAGCCCGGGACTTCCTACCCAA

>KY437450.1 Equus caballus clone 294 immunoglobulin mu heavy chain (IGHM) mRNA, partial cds
CTGTCCCTCACCTGCACTGTCTCTGGATTCTCTTTGAGCAGTTACGGTGTAGGCTGGGTCCGCCAGGCTCCAGGAAA
AGGGCTGGAATATGTTGGTGTTATAGCTAGTAGTGGAAGTGCAAACTACAACCCAGCCCTGAAGTCCCGAGCCAGCA
TCACCAAGGACACCTCAAAGAGCCAAGTTTATCTGACGCTGAGCAGCCTGACAGGCGAGGACACGGCCGTCTATTAC
TGTGCGACATCGGAGATCAGCTATACTTACTACGTGGGCCAAACCTACTATTTTGGCTACTGGGGCCAGGGCACCCT
GGTCACCGTCTCCTCAGAGAGTACGATGACCCCAGATCTCTTCCCCCTCGTCTCCTGTGGGCCCTCTCTTGATGAGA
GCCTGGTGGCTGTGGGCTGCCTAGCCCGGGACTTCCTACCCAA
>KY437449.1 Equus caballus clone 293 immunoglobulin mu heavy chain (IGHM) mRNA, partial cds
CTCTCCCTCACCTGCACTGTCTCTGGATTATCTTTGAGCAGTAATAATGTAGGCTGGGTCCGCCAGGCTCCAGGAAA
AGGGCTGGAATATGTTGGTGGTATAGCTAGTAGTGGAAGTGCAAACTACAACCCAGCCCTGAAGTCCCGAGCCAGCA
TCACCAAGGACACCTCAAAGAGCCAAGTTTATCTGACGCTGAACAGCCTGACAAGCGAGGACACGGCCGTCTATTAC
TGTGCAAGAGAGTATGGTTACTGGGCTGGTAATTTTGGCTACTGGGGCCAGGGCACCCTGGTCACCGTCTCCTCAGA
GAGTACGATGACCCCAGATCTCTTCCCCCTCGTCTCCTGTGGGCCCCCTCTTGATGAGAGCCTGGTGGCTGTGGGCT
GCCTAGCCCGGGACTTCCTACCCAA
>KY437448.1 Equus caballus clone 292 immunoglobulin mu heavy chain (IGHM) mRNA, partial cds
CTCTCCCTCACCTGCACTGTCTCTGGATTATCTTTGAGCAGTTATGCTGTAGGCTGGGTCCGCCAGGCTCCAGGAAA
AGGGCTGGAATCAGTTGGTGCTATATATGGTAGTGCAAGTGCAAACTACAACCCAGCCCTGAAGTCCCGAGCCAGCA
TCACCAAGGACACCTCAAAGAGCCAAGTTTATCTGACGCTGAACAGCCTGACAGGCGAGGACACGGCCGTCTATTAC
TGTGCGAGTAGCCGAAGTGATGATTGGTATGGTGCTATTGACTACATAATCTATTATTATGGTATAAACTACTGGGG
CCAGGGCATCCTGGTCACCGTCTCCTCAGAGAGTACGATGACCCCAGATCTCTTCCCCCTCGTCTCCTGTGGGCCCT
CTCTTGATGAGAGCCTGGTGGCTGTGGGCTGCCTAGCCCGGGACTTCCTACCCAA
>KY437447.1 Equus caballus clone 291 immunoglobulin mu heavy chain (IGHM) mRNA, partial cds
CTCTCCCTCACCTGCACTGTCTCTGGATTATCTTTGAGCAGTAATACTGTAGGCTGGGTCCGCCAGGCTCCAGGAAA
AGGGCTGGAATATGTTGGTGGTATAGCTGGTAGTGGAAGTGCAAACTACAATTCAGCCCTGAAGTCCCGAGCCAGCA
TCACCAAGGACACCTCAAAGAGCCAAGTTTATCTGACGCTGAACAGCCTGACAAGCGAGGACACGGCCGTCTATTAC
TGTGCAGGAGGTCCTCATTTTGGCTACTGGGGCCAGGGCACCCTGGTCACCGTCTCCTCAGAGAGTACGATGACCCC
AGATCTCTTCCCCCTCGTCTCCTGTGGGCCCTCTCTTGATGAGAGCCTGGTGGCTGTGGGCTGCCTAGCCCGGGACT
TCCTACCCAA
>KY437446.1 Equus caballus clone 290 immunoglobulin mu heavy chain (IGHM) mRNA, partial cds
CTCTCCCTCACCTGCACTGTCTCTGGATTATCTTTGAGCAGTAATGCTGTAGGCTGGGTCCGCCAGGCTCCAGGAAA
AGGGCTGGAATATGTTGGTGGTATAGCTAGTAGTGGAAGTGCAAACTACAACCCAGCCCTGAAGTCCCGAGCCAGCA
TCACCAAGGACACCTCAAAGAGCCAAGTTTATCTGACGCTGAACAGCCTGACAAGCGAGGACACGGCCGTCTATTAC
TGTGCAAGAGAGTATGGTTACTGGGCTGGTAATTTTGGCTACTGGGGCCAGGGCACCCTGGTCACCGTCTCCTCAGA
GAGTACGATGACCCCAGATCTCCTCCCCCTCGTCTCCTGTGGGCCCTCTCTTGATGAGAGCCTGGTGGCTGTGGGCT
GCCTAGCCCGGGACTTCCTACCCAA
>KY437445.1 Equus caballus clone 289 immunoglobulin mu heavy chain (IGHM) mRNA, partial cds
CTCTCCCTCACCTGCACTGTCTCTGGATTATCTTTGAGCAGTAATGCTGTAGGCTGGGTCCGCCAGGCTCCAGGAAA
AGGGCTGGAATATGTTGGTGATATAGGTAGAAGTGGAAGTACAAACTACAACCCAGCCCTGAAGTCCCGAGCCAGCA
TCACCAAGGACACCTCAAAGAGTCAAGTTTATCTGACGCTGAACAGCCTGACAAGCGAAGACACGGCCGTCTATTAC
TGTGCAGGAGGCCTCTGGGGTGATGGTTATTTTTGCGGCTACGTGTTTCACTGGGGCCAGGGCACCCTGGTCACCGT
CTCCTCAGAGAGTACGATGACCCCAGATCTCTTCCCCCTCGTCTCCTGTGGGCCCTCTCTTGATGAGAGCCTGGTGG
CTGTGGGCTGCCTAGCCCGGGACTTCCTACCCAA
>KY437444.1 Equus caballus clone 288 immunoglobulin mu heavy chain (IGHM) mRNA, partial cds
CTGTCCCTCACCTGCACTGTCTCTGGATTATCTTTGAGCAGTGATGGTGTAAACTGGGTCCGCCAGGCTCCAGGAAA
AGGGCTGGAGTGGGTTGGTGCTGATTATGGAAGTGGAATTACATACTACAACCCAGCCCTGAAGTCCCGAGCCAGCA
TCACCAAGGACACCTCAAAGAGCCAAGTTTATCTGACGCTGAACAGCCTGACAGGCGAAGACACGGCCGTCTATTAC
TGTGTAGTTTTAGGTTTTGTCTACTGGGGCCAGGGCACCCTGGTCACCGTCTCCTCAGAGAGTACGATGACCCCAGA

TCTCTTCCCCCTCGTCTCCTGTGGGCCCTCTCTTGATGAGAGCCTGGTGGCTGTGGGCTGCCTAGCCCGGGACTTCC
TACCCAA
>KY437443.1 Equus caballus clone 287 immunoglobulin mu heavy chain (IGHM) mRNA, partial cds
CTCTCCCTCACCTGCACTGTCTCTGGATTCTCTTTGAGTAGTTATCCTGTAGGCTGGGTCCGCCAGGCTCCAGGAAA
AGGGCTGGAATATGTTGGTTCTATAGATGATGATGCAGAGACAAACTACAACCCAGCCCTGAAGTCCCGAGCCAGCA
TCACCAAGGACACCTCAAAGAGCCAAGTTTATCTGACGCTGAACAGCCTGACAGGCGAGGACACGGCCGTCTATTAC
TGTGTAAGAGACAGCTACGAATATATGTATGCACTTTTTGCCTACTGGGGCCAGGGCACCCTGGTCACCGTCTCCTC
AGAGAGTACGATGACCCCAGATCTCTTCCCCCTCGTCTCCTGTGGGCCCTCCCTTGATGAGAGCCTGGTGGCTGTGG
GCTGCCTAGCCCGGGACTTCCTACCCAA
>KY437442.1 Equus caballus clone 286 immunoglobulin mu heavy chain (IGHM) mRNA, partial cds
CTGTCCCTCACCTGCACTGTCTCTGGATCATCTTTGAGCAGTACGGCTGTAGGCTGGGTCCGCCAGGCTCCAGGAAA
AGGGCTGGAGTGGGTTGGTGCTATATATGGTAGTGAAAGTACATACTACAACCCAGCCCTGAAGTCCCGAGCCAGCA
TCACCAAGGACACCTCAAAGAGCCAAGTTTATCTGACGCTGAACAGCCTGACAGGCGAAGACACGGCCGTCTATTAC
TGTGCAGATGAAAGAGGCTACTGGGGCCAGGGCATCCTGGTCACCGTCTCCTCAGAGAGTACGATGACCCCAGATCT
CTTCCCCCTCGTCTCCTGTGGGCCCTCTCTTGATGAGAGCCTGGTGGCTGTGGGCTGCCTAGCCCGGGACTTCCTAC
CCAA
>KY437441.1 Equus caballus clone 285 immunoglobulin mu heavy chain (IGHM) mRNA, partial cds
CTCTCCCTCACCTGCACTGTCTCTGGATTATCTTTGAGCAGTAAGGTTGTAGGCTGGATCCGCCAGGCTCCAGGAAA
AGGGCTGGAATGGGTTGGTGATATAAGTAGTAGTGGAAGTACAGAGTACAACCCAGCCCTGAAGTCCCGAGCCAGCA
TCACCAAGGACACCTCAAGCAGCCAAGTTTATCTGACGCTGAACAGCCTGACAAGCGAGGACACGGCCGTCTATTAC
TGTGCAGGAGACGTCGCTTTTGGCTACTGGGGCCAGGGCACCCTGGTCACCGTCTCCCCAGAGAGTACGATGACCCC
AGATCTCTTCCCCCTCGTCTCCTGTGGGCCCTCTCTTGATGAGAGCCTGGTGGCTGTGGGCTGCCTAGCCCGGGACT
TCCTACCCAA
>KY437440.1 Equus caballus clone 284 immunoglobulin mu heavy chain (IGHM) mRNA, partial cds
CTCTCCCTCACCTGCACTGTCTCTGGATTATCTTTGAACAGTAATGCTGTAGACTGGGTCCGCCAGGCTCCAGGAAA
AGGGCTGGAATATGTTGGTGAGATATTTAGTAGTGGAAGTGCAAACTACAACCCAGCCCTGAAGTCCCGAGCCAGCA
TCACCAAGGACACCTCAAAGAGCCAAGTTTATCTGACGCTGAACAGCCTGACAGGCGAAGACACGGCCGTCTATTAC
TGTGCAAGTAGTCTACCTGATGGTATAAACTACTGGGGCCAGGGCATCCTGGTCACCGTCTCCTCAGAGAGTACGAT
GACCCCAGATCTCTTCCCCCTCGTCTCCTGTGGGCCCTCTCTTGATGAGAGCCTGGTGGCTGTGGGCTGCCTAGCCC
GGGACTTCCTACCCAA
>KY437439.1 Equus caballus clone 283 immunoglobulin mu heavy chain (IGHM) mRNA, partial cds
CTCTCCCTCACCTGCACTGTCTCTGGATTATCTTTGAGCAGTAATTCTGTAGGCTGGGTCCGCCAGGCTCCAGGAAA
AGGGCTGGAATTTGTTGGTGCTATAACTGGTAGTTCAAGTGCACGCTACAACCCAGCCCTGAAGTCCCGAGCCAGCA
TCACCAAGGACACCTCAAAGAGCCAAGTTTATCTGACGCTGAACAGCCTGACAAGCGAGGACACGGCCGTCTATTAC
TGTGCAGGATGGATACCAACTATGCTTTTTGGCTACTGGGGCCAGGGCACCCTGGTCACCGTCTCCTCAGAGAGTAC
GATGACCCCAGATCTCTTCCCCCTCGTCTCCTGTGGGCCCTCTCTTGATGAGAGCCTGGTGGCTGTGGGCTGCCTAG
CCCGGGACTTCCTACCCAA
>KY437438.1 Equus caballus clone 282 immunoglobulin mu heavy chain (IGHM) mRNA, partial cds
CTCTCCCTCACCTGCACTGTCTCTGGATTATCTTTGAGCAGTTATAGTGTGGGCTGGGTCCGCCAGGCTCCAGGAAA
AGGGCTGGAATTTGTTGGTATGATTACTACCAGTGGAAGTGCAGACTACAATCCAGTCCTGAAGTCCCGAGCCAGCA
TCACCAAGGACACCTCGAGGAGCCAAGTTTATCTGACGCTGAACAGCCTGACAAGCGAGGACACGGCCGTCTATTAC
TGTGTAGGACGTTACCATAGTGGTAGTTACTACTACTGGGGCCAGGGCACCCTGGTCACCGTCTCCTCAGAGAGTAC
GAAGACCCCAGATCTCTTCCCCCTCGTCTCCTGTGGGCCCTCTCTTGATGAGAGCCTGGTGGCTGTGGGCTGCCTAG
CCCGGGACTTCCTACCCAA
>KY437437.1 Equus caballus clone 281 immunoglobulin mu heavy chain (IGHM) mRNA, partial cds
CTCTCCCTCACCTGCACTGTCTCTGGATTATCTTTGAGTAGTTATGGTGTGGGCTGGGTCCGCCAGGCTCCAGGAAA
AGGGCTGGAATTTGTTGGTGGTATAGCTGATAGTGGAAGTACAATGTACAACCCAGCCCTGAAGTCCCGAGCCAGCA
TCACCAAGGACACCTCAAAGAGCCAAGTTTATCTGACGCTGAACAGCCTGACAAGCGAGGACACGGCCGTCTATTAC
TGTGCAGGAGACAATTACTATGGTGGTAGTTCCTGGTACTCCGTCTCTAGCTTTGACTACTGGGGCCAGGGCACCCT

GGTCACCGTCTCCTCAGAGAGTACGAAGACCCCAGATCTCTTCCCCCTCGTCTCCTGTGGGCCCTCTCTTGATGAGA
GCCTGGTGGCTGTGGGCTGCCTAGCCCGGGACTTCCTACCCAA
>KY437436.1 Equus caballus clone 280 immunoglobulin mu heavy chain (IGHM) mRNA, partial cds
CTCTCCCTCACCTGCACTGTCTCTGGATTATCTTTGAGCAGTTATGGTGTGGGCTGGGTCCGCCAGGCTCCAGGAAA
AGGACTGGAATTTGTTGGTGGTATAGCTGGTAGTGGAACTGCAACCTACAACCCAGCCCTGAAGTCCCGAGCCAGCA
TCACCAAGGACACCTCAACGAGCCAAGTTTATCTGACGCTAAACAGCCTGACAAGCGAGGACACGGCCGTCTATTAC
TGTGCAGGAGGCTTCGACGCTGGTGATGGTGGTCGTTACTACGCGGATTATGGAATAAACTACTGGGGCCAGGGCAT
CCTGGTGACCGTCTCCTCAGAGAGTACGAAGACCCCAGATCTCTTCCCCCTCGTCTCCTGTGGGCCCTCTCTTGATG
AGAGCCTGGTGGCTGTGGGCTGCCTAGCCCGGGACTTCCTACCCAA
>KY437435.1 Equus caballus clone 279 immunoglobulin mu heavy chain (IGHM) mRNA, partial cds
CTCTCCCTCACCTGCACTGTCTCTGGATTATCTTTGAGCAGTAATGCTGTAGGCTGGGTCCGCCAGGCTCCAGGAAA
AGGGCTGGAATTTGTTGGTGCCGTATATGGTAGTGCAAGTACAAACTACAACCCAGCCCTGAAGTCCCGAGCCAGCA
TCACCAAGGACACCTCAAAGAGCCAAGTTTATCTGACGCTGAACAGCCTGACAAGCGAGGACACGGCCGTCTATTAC
TGTGCAGGAGAAAAACGCGTTAGTGCCGATGCCTCTTATTATTATGGTATAGACTACTGGGGCCAGGGCATCCTGGT
CACCGTCTCCTCAGAGAGTACGATGACCCCAGATCTCTTCCCCCTCGTCTCCTGTGGGCCCTCTCTTGATGAGAGCC
TGGTGGCTGTGGGCTGCCTAGCCCGGGACTTCCTACCCAA
>KY437434.1 Equus caballus clone 278 immunoglobulin mu heavy chain (IGHM) mRNA, partial cds
CTCTCCCTCACCTGCACTGTCTCTGGATTATCTTTGAGCAGCTATCTTATAGAGTGGGTCCGCCAGGCTCCAGGAAA
AGGGCTGGAATATGTTGGTGCTATCGCTAGTAGTGGAAGTACAAACTACAACCCAGCCCTGAAGTCCCGAGCCAGCA
TCACCAAGGACACCTCAAAGAGCCAAGTTTATCTGACGCTGAACAGCCTGACAAGCGAGGACACGGCCGTCTATTAC
TGTCGAGGAGGCTCCAAGTGGGGCCAGGGCATCCTGGTCACCGTCTCCTCAGAGAGTACGATGACCCCAGATCTCTT
CCCCCTCGTCTCCTGTGGGCCCTCTCTTGATGAGAGCCTGGTGGCTGTGGGCTGCCTAGCCCGGGACTTCCTACCCA
A
>KY437433.1 Equus caballus clone 277 immunoglobulin mu heavy chain (IGHM) mRNA, partial cds
CTGTCCCTCACCTGCACTGTCTCTGGATTCTCTTTGAGCAGTTACGGTGTAGGCTGGGTCCGCCAGGCTCCAGGAAA
AGGGCTGGAATTTGTTGGTGGTATAGCTAGTAGTGGAAGTGCAAACTACAACCCAGCCCTGAAGTCCCGAGCCAGCA
TCACCAAGGACACCTCAAAGAGCCAAGTTTATCTGACGCTGAACAGCCTGACAGGCGAGGACACGGCCGTCTATTAC
TGTGCGAGAGATATGGTTATGGTGGTGCTTACTTGGGGTATAAACTACTGGGGCCAGGGCATCCTGGTCACCGTCTC
CTCAGAGAGTACGAAGACCCCAGATCTCTTCCCCCTCGTCTCCTGTGGGCCCCCTCTTGATGAGAGCCTGGTGGCTG
TGGGCTGCCTAGCCCGGGACTTCCTACCCAA
>KY437432.1 Equus caballus clone 276 immunoglobulin mu heavy chain (IGHM) mRNA, partial cds
CTCTCCCTCACCTGCACTGTCTCTGGATTATCTTTGAGCAGTTATGGTGTGGGCTGGGTCCGCCAGGCTCCAGGAAA
AGGGCTGGAATATGTTGGTGCTATAGCTAGTACAGGAAGTGCAAACTACAACCCAGCCCTGAAGTCCCGAGCCAGCA
TCACCAAGGACACCTCAAAGAGCCAAGTTTATCTGACGCTGAACAGCCTGACAAGCGAGGACACGGCCGTCTATTAC
TGTGCAGGATGCTATGGGGATTATGGTGAGAACTACTGGGGCCAGGGCATCCTGGTCACCGTCTCCTCAGAGAGTAC
GATGACCCCAGACCTCTTCCCCCTCGTCTCCTGTGGGCCCTCTCTTGATGAGAGCCTGGTGGCTGTGGGCTGCCTAG
CCCGGGACTTCCTACCCAA
>KY437431.1 Equus caballus clone 275 immunoglobulin mu heavy chain (IGHM) mRNA, partial cds
CCTCTCCCTCACCTGCACTGTCTCTGGATTATCTTTGAGTAGTTATGGTGTGGGCTGGGTCCGCCAGGCTCCAGGAA
AAGGGCTGGAATTAGTTGGTGAGTGGGATAGTAGTGGAAGTATAATCTACAATCCAGCCCTGAAGTCCCGAGCCAGC
ATCACCAAGGACACCTCAAAGAGCCAACTTTATCTGACGCTGAACAGCCTGTCAAGCGAGGACACGGCCGTCTATTA
CTGTATAGGATTCACTATGGCTATGTCATGGGGCCAGGGCACCCTGGTCACCGCCTCCTCAGAGAGTACGATGACCC
CAGATCTCTTCCCCCTCGTCTCCTGTGGGCCCTCTCTTGATGAGAGCCCGGTGGCTGTGGGCTGCCTAGCCCGGGAC
TTCCTACCCAA
>KY437430.1 Equus caballus clone 274 immunoglobulin mu heavy chain (IGHM) mRNA, partial cds
CTCTCCCTCACCTGCACTGTCTCTGGATTATCTTTGAGCAGTTATGGTGTGGGCTGGGTCCGCCAGGCTCCAGGAAA
AGGGCTGGAATTTGTTGGTGGTATAGCTAGTAGTGGAAGTGCAAACTACAACCCAGCCCTGAAGTCCCGAGCCAGCA
TCACCAAGGACACCTCAAAGAGCCAAGTTTATCTGACGCTGAACAGCCTGACAAGCGAGGACACGGCCGTCTATTAC

TGTGCAGGAGGCCGTATGTACTATGGTGATACTTTCTACTATTCGTTTGGCTACTGGGGCCAGGGCACCCTGGTCAC
CGTCTCCTCAGAGAGTACGAAGACCCCAGATCTCTTCCCCCTCGTCTCCTGTGGGCCCTCTCTTGATGAGAGCCTGG
TGGCTGTGGGCTGCCTAGCCCGGGACTTCCTACCCAA
>KY437429.1 Equus caballus clone 273 immunoglobulin mu heavy chain (IGHM) mRNA, partial cds
CTCTCCCTCACCTGCACTGTCTCTGGATTATCTTTGAGTAGTTATGTTGTATGGTGGGTCCGCCAGGCTCCAGGAAA
AGGGCTGGAATATGTTGGTTCAGCATATGGCAGTGCAAGTCCAAGGTACAACCCAGCCCTGAAGTCCCGAGCCAGCA
TCACCAAGGACACCTCAAAGAGCCAAGTTTATCTGACGCTGAACAGCCTGACAGGCGAGGACACGGCCGTCTATTAC
TGTGCGAGATGGGGGGGTGACTACGTGGATCACTGGGGCCAGGGCACCCTGGTCACCGTCTCCTCAGAGAGTACGAT
GACCCCAGATCTCTTCCCCCTCGTCTCCTGTGGGCCCTCTCTTGATGAGAGCCTGGTGGCTGTGGGCTGCCTAGCCC
GGGACTTCCTACCCAA
>KY437428.1 Equus caballus clone 272 immunoglobulin mu heavy chain (IGHM) mRNA, partial cds
CTCTCCCTCACCTGCACTGTCTCTGGATTATCTTTGAGCAGTAATGCTGTAGGCTGGGTCCGCCAGGCTCCAGGAAA
AGGGCTGGAATTTGTTGGTGATGCAAGTGCAAACTACAACCCAGCCCTGAAGTCCCGAGCCAGCATCACCAAGGACA
CCTCAAAGAGCCAAGTTTATCTGACGCTGAACAGCCTGACAAGCGAGGACACGGCCGTCTATTACTGTGCAGGAGGC
TCTTTCTATGGCATTCAAGGAGACTGGGGCCAGGGCATCCTGGTCACCGTCTCCTCAGAGAGTACGATGACCCCAGA
TCTCTTCCCCCTCGTCTCCTGTGGGCCCTCTCTTGATGAGAGCCTGGTGGCTGTGGGCTGCCTAGCCCGGGACTTCC
TACCCAA
>KY437427.1 Equus caballus clone 271 immunoglobulin mu heavy chain (IGHM) mRNA, partial cds
CTCTCCCTCACCTGCACTGTCTCTGGATTATCTTTGAGCAGTAATGTTGTAGGCTGGGTCCGCCAGGCTCCAGGAAA
AGGGCTGGAATATGTTGGTGGTATAGCGGGTAGTGGAGGTACAAACTACAACCCAGCCCTGAAGTCCCGAGCCACCA
TCACCAAGGACACCTCAAAGAGCCAAGTTTATCTGACGCTGAACAGCCTGACAAGCGAGGACACGGCCGTCTATTAC
TGTGCAGAAAGGTTTTTTGGCTACTGGGGCCAGGGCACCCTGGTCACCGTCTCCTCAGAGAGTACGATGACCCCAGA
TCTCTTCCCCCTCGTCTCCTGTGGGCCCTCTCTTGATGAGAGCCTGGTGGCTGTGGGCTGCCTAGCCCGGGACTTCC
TACCCAA
>KY437426.1 Equus caballus clone 270 immunoglobulin mu heavy chain (IGHM) mRNA, partial cds
CTCTCCCTCACCTGCACTGTCTCTGGATTCTCTTTGAGTAGTAATGGTGTGCAATGGGTCCGCCAGGCTCCAGGAAA
AGGGCTGGAATTTGTTGGTTTTATAGCTAGCAGTGGAAGTGCAAACTACAACCCAGCCCTGAAGTCCCGAGCCAGCA
TCACCAAGGACACCTCAAAGAGCCAAGTTTATCTGACGCTGAACAGCCTGACAAGCGAGGACACGGCCGTCTATTAC
TGTATAGGAATGCCCTACTGGGGCCAGGGCATCCTGGTCACCGTCTCCTCAGAGAGTACGATGACCCCAGATCTCTT
CCCCCTCGTCTCCTGTGGGCCCTCTCTTGATGAGAGCCTGGTGGCTGTGGGCTGCCTAGCCCGGGACTTCCTACCCA
A
>KY437425.1 Equus caballus clone 269 immunoglobulin mu heavy chain (IGHM) mRNA, partial cds
CTCTCCCTCACCTGCACTGTCTCTGGATTATCTTTGAGCAGTGATGCTGTAGGCTGGGTCCGCCAGGCTCCAGGAAA
AGGGCTGGAATTTGTTGGTGTCATAGTTGGTAGTGGAAGTGCAAACTACAACCCAGCCCTGAAGTCCCGAGCCAGCA
TCACCAAGGACACCTCAAAGAGCCAAGTTTATCTGACGCTGAACAGCCTGACAAGCGAGGACACGGCCGTCTATTAC
TGTGCAGGAGATGGCTATGGTGATACTTTCTACTTTAAGTACAGTTTTGGCTACTGGGGCCAGGGCACCCTGGTCAC
CGTCTCCTCAGAGAGTACGATGACCCCAGATCTCTTCCCCCTCGTCTCCTGTGGGCCCTCTCTTGATGAGAGCCTGG
TGGCTGTGGGCTGCCTAGCCCGGGACTTCCTACCCAA
>KY437424.1 Equus caballus clone 268 immunoglobulin mu heavy chain (IGHM) mRNA, partial cds
CTGTCCCTCACCTGCACTGTCTCTGGATTCTCTTTGAGCAGTTACGGTGTAGGCTGGGTCCGCCAGGCTCCAGGAAA
AGGGCTGGAATTTGTTGGTGGTATAGCTAGTAGTGGAAGTGCAAACTACAACCCAGCCCTGAAGTCCCGAGCCAGCA
TCACCAAGGACACCTCAAAGAGCCAAGTTTATCTGACGCTGAACAGCCTGACAGGCGAGGACACGGCCGTCTATTAC
TGTGCGAGATTCGTCAAAGATGGTTACTATGGTAGTTACTACAGTAGTTACTATGGTATAAACTACTGGGGCCAGGG
CATCCTGGTCACCGTCTCCTCAGAGAGTACGAAGACCCCAGATCTCTTCCCCCTCGTCTCCTGTGGGCCCTCTCTTG
ATGAGAGCCTGGTGGCTGTGGGCTGCCTAGCCCGGGACTTCCTACCCAA
>KY437423.1 Equus caballus clone 267 immunoglobulin mu heavy chain (IGHM) mRNA, partial cds
CTCTCCCTCACCTGCACTGTCTCTGGATTATCTTTGAGCAGTAATGTTGTAGGCTGGGTCCGCCAGGCTCCAGGAAA
AGGGCTGGAATATGTTGGTGGTATAGCGGGTAGTGGAGGTACAAACTACAACCCAGCCCTGAAGTCCCGAGCCACCA
TCACCAAGGACACCTCAAAGAGCCAAGTTTATCTGACGCTGAACAGCCTGACAAGCGGGGACACGGCCGTCTATTAC

TGTGCAGAAAGGTTTTTTGGCTACTGGGGCCAGGGCACCCTGGTCACCGTCTCCTCAGAGAGTACGATGACCCCAGA
TCTCTTCCCCCTCGTCTCCTGTGGGCCCTCTCTTGATGAGAGCCTGGTGGCTGTGGGCTGCCTAGCCCGGGACTTCC
TACCCAA
>KY437422.1 Equus caballus clone 266 immunoglobulin mu heavy chain (IGHM) mRNA, partial cds
CCGTCCCTCACCTGCACTGTCTCTGGATTATCTTTGAGCAGTAATGCTGTAGGCTGGGTCCGCCAGGCTCCAGGAAA
AGGGCTGGAGTGGGTTGGTGTTATATATGGTAGTGAAAGTACATACTACAACCCAGCCCTGAAGTCCCGAGCCAGCA
TCACCAAGGACACCTCAAAGAGCCAAGTTTATCTGACGCTGAACAGCCTGACAGGCGAAGACACGGCCGTCTATTAC
TGTGCAGGGGGGTCATATGGTTATGGTGGTGCTTACTACTCGTATTATTATGGTATAAACTACTGGGGCCAGGGCAT
CCTGGTCACCGTCTCCTCAGAGAGTACGATGACCCCAGATCTCTTCCCCCTCGTCTCCTGTGGGCCCTCTCTTGATG
AGAGCCTGGTGGCTGTGGGCTGCCTAGCCCGGGACTTCCTACCCAA
>KY437421.1 Equus caballus clone 265 immunoglobulin mu heavy chain (IGHM) mRNA, partial cds
CTCTCCCTCACCTGCACTGTCTCGGGAGTATCTTTGAGCAGTTATACTGTGGGCTGGGTCCGCCAGGCTCCAGGAAA
AGGGCTGGAATTTGTTGGTGGTGTAGCTAGTAGTGGAAGTGCAAACTACAACCCAGCCCTGAAGTCCCGAGCCAGCA
TCACCAAGGACACCTCAAAGAGCCAAGTTTATCTGACGCTGAACAGCCTGACAAGCGAGGACACGGCCGTCTATTAC
TGTGCAGGAGGGGGTTACTATCTCAATACTTACTATGGTAAATTTGACTACTGGGGCCAGGGCACCCTGGTCACCGT
CTCCTCAGAGAGTACGAAGACCCCAGATCTCTTCCCCCTCGTCTCCTGTGGGCCCTCTCTTGATGAGAGCCTGGTGG
CTGTGGGCTGCCTAGCCCGGGACTTCCTACCCAA
>KY437420.1 Equus caballus clone 264 immunoglobulin mu heavy chain (IGHM) mRNA, partial cds
CTCTCCCTCACCTGCACTGTCTCTGGATTATCTTTGAGCAGTAATACTGTAGGCTGGGTCCGCCAGGCTCCAGGAAA
AGGGCTGGAATATGTTGGTACGATAGCTAGTAGTGGAAGTGCAAACTACAACCCAGCCCTGAAGTCCCGAGCCAGCA
TCACCAAGGACACCTCAAAGAGCCAAGTTTATCTGACGCTGAACAGCCTGACAAGCGAGGACACGGCCGTCTATTAC
TGTGCAGGAGGAGGAGACCGACATGACTATAGTGATACTTTCTACTACTCTGGCTACTGGGGCCAGGGCACCCTGGT
CACCGTCTCCTCAGAGAGTACGATGACCCCAGATCTCTTCCCCCTCGTCTCCTGTGGGCCCTCTCTTGATGAGAGCC
TGGTGGCTGTGGGCTGCCTAGCCCGGGACTTCCTACCCAA
>KY437419.1 Equus caballus clone 263 immunoglobulin mu heavy chain (IGHM) mRNA, partial cds
CTCTCCCTCACCTGCACTATCTCTGGATTCTCTTTGAGCAGTTATGCTGTATACTGGGTCCGCCAGGCTCCAGGAAA
AGGGCTGGAATATGTTGGTACTATAGATGGTAGTGCAAGTGCAGACTACAACCCAGCCCTGAAGTCCCGAGCCAGCA
TCACCAAGGACACCTCAAAGAGCCAAGTTTATCTGACGCTGAACAGCCTGACAGGCGAGGACACGGCCGTCTATTAC
TGTGCGAGATCGGATATCCGGAGCATTTATGGTAAATCCGTTAATGCTGTGGACCCCTGGGGCCAGGGCACCCTGGT
CACCGTCTCCTCAGAGAGTACGATGACCCCAGATCTCTTCCCCCTCGTCTCCTGTGGGCCCTCTCTTGATGAGAGCC
TGGTGGCTGTGGGCTGCCTAGCCCGGGACTTCCTACCCAA
>KY437418.1 Equus caballus clone 262 immunoglobulin mu heavy chain (IGHM) mRNA, partial cds
CTCTCCCTCACCTGCACTGTCTCTGGATTATCTTTGAGCAGTAATTCTGTAGACTGGGTCCGCCAGGCTCCAGGAAA
AGGGCTGGAATACGTTGGTGCTATATATGGTAGTATAAGTCCTATATACAACCCAGCCCTGAAGTCCCGAGCCAGCA
TCACCAAGGACACCTCAAAGAGCCAAGTTTATCTGACGCTGAACAGCCTGACAAGCGAGGACACGGCCGTCTATTAC
TGTGCAGGAGGCCAGGCAAACACTGTACTTACCTACTGGGGCCAGGGCACCCTGGTCACCGTCTCCTCAGAGAGTAC
GAAGACCCCAGATCTCTTCCCCCTCGTCTCCTGTGGGCCCTCTCTTGATGAGAGCCTGGTGGCTGTGGGCTGCCTAG
CCCGGGACTTCCTACCCAA
>KY437417.1 Equus caballus clone 261 immunoglobulin mu heavy chain (IGHM) mRNA, partial cds
CTGTCCCTCACCTGCACTGTCTCTGGATTCTCTTTGAGCAGTTACGGTGTAAAGTGGGTCCGCCAGGCTCCAGGAAA
AGGGCTGGAATATGTTGGCGGTATAGCTACTAGTGGAAGTATAAACTACAACCCAGCCCTGAAGTCCCGAGCCAGCA
TCACCAAGGACACCTCAAAGAGCCAAGTTTATCTGACGCTGAACAGCCTGACAGGCGAGGACACGGCCGTCTATTAC
TGTGCGAGGCTGGACTATGGTGGTAGTCCCTGGCGCTCAACGTACTACTTTGCCTACTGGGGCCAGGGCACCCTGGT
CACCGTCTCCTCAGAGAGTACGATGACCCCAGATCTCTTCCCCCTCGTCTCCTGTGGGCCCTCTCTTGATGAGAGCC
TGGTGGCTGTGGGCTGCCTAGCCCGGGACTTCCTACCCAA
>KY437416.1 Equus caballus clone 260 immunoglobulin mu heavy chain (IGHM) mRNA, partial cds
CTGTCCCTCACCTGCACTGTCTCTGGATTCTCTTTGAGCAGTTACGGTGTAGGCTGGGTCCGCCAGGCTCCAGGAAA
AGGGCTGGAATATGTTGGTGGTATAACTAATAGTGGAAGTGCAAACTACAACCCAGCCCTGAAGTCCCGAGCCAGCA

TCACCAAGGACACCTCAAAGAGCCAGGTTTATCTGACGCTGAACAGCCTGACAGGCGAAGACACGGCCGTCTATTAC
TGTGCGAGAGGTCAATCTTTTGGCAGCTGGGGCCAGGGCACCCTGGTCACCGTCTCCTCAGAGAGTACGAAGACCCC
AGATCTCTTCCCCCTCGTCTCCTGTGGGCCCTCTCTTGATGAGAGCCTGGTGGCTGTGGGCTGCCTAGCCCGGGACT
TCCTACCCAA
>KY437415.1 Equus caballus clone 259 immunoglobulin mu heavy chain (IGHM)
mRNA, partial cds
CTGTCCCTCACCTGCACTGTCTCTGGATTCTCTTTGAGCAGTTACGGTGTAGGCTGGGTCCGCCAGGCTCCAGGAAA
AGGGCTGGAATATGTTGCTGGTATAGCTAGTAGTGGAAGTGCAAACTACAACCCAGCCCTGAAGTCCCGAGCCAGCA
TCACCAAGGACACCTCAAAGAGCCAAGTTCTTCTGACGCTGAACAGCCTGACAGGCGAGGACACGGCCGTCTATTAC
TGTGCGAGAGGAGAGAGATCCCAGTATAGTGACTATGCGGATGTCTACGTGGATCACTGGGGCCAGGGCACCCTGGT
CACCGTCTCCTCAGAGAGTACGATGACCCCAGATCTCTTCCCCCTCGTCTCCTGTGGGCCCTCTCTTGATGAGAGCC
TGGTGGCTGTGGGCTGCCTAGCCCGGGACTTCCTACCCAA
>KY437414.1 Equus caballus clone 258 immunoglobulin mu heavy chain (IGHM)
mRNA, partial cds
CTCTCCCTCACCTGCACTGTCTCTGGATTCTCTTTGAGCAGTTATGCTGTAGGCTGGGTCCGCCAGGCTCCAGGAAA
AGGGCTGGAATATGTTGGTGCTATATATGGTAGTGCAAGTGCAAACTACAACCCAGCCCTGAAGTCCCGAGCCAGCA
TCACCAAGGACACCTCAAAGAGCCAAGTTTATCTGACGCTGAACAGCCTGACAGGCGAGGACACGGCCGTCTATTAC
TGTGCGAGACTGAGACGGGGGGATAGTTATGGTAGTTACTATGGCTTTGGCTACTGGGGCCAGGGCACCCTGGTCAC
CGTCTCCTCAGAGAGTACGATGACCCCAGATCTCTTCCCCCTCGTCTCCTGTGGGCCCTCTCTTGATGAGAGCCTGG
TGGCTGTGGGCTGCCTAGCCCGGGACTTCCTACCCAA
>KY437413.1 Equus caballus clone 257 immunoglobulin mu heavy chain (IGHM)
mRNA, partial cds
CTGTCCCTCACCTGCACTGTCTCTGGATTCTCTTTGAGCAGTTACGGTGTAGGCTGGGTCCGCCAGGCTCCAGGAAA
AGGGCTGGAATATGTTGGTGGTATAGCTAGTAGTGGAAGTGCAAACTACAACCCAGCCCTGAAGTCCCGAGCCAGCA
TCACCAAGGACACCTCAAAGAGCCAAGTTTATCTGACGCTGAACAGCCTGACAGGCGAGGACACGGCCGTCTATTAC
TGTGCGAGCCAGGCATATAGTTATGCTAGTAGCTATGGTTTTACGTCTGAGTTTGGCTACTGGGGCCAGGGCACCCT
GGTCACCGTCTCCTCAGAGAGTACGATGACCCCAGATCTCTTCCCCCTCGTCTCCTGTGGGCCCTCTCTTGATGAGA
GCCTGGTGGCTGTGGGCTGCCTAGCCCGGGACTTCCTACCCAA
>KY437412.1 Equus caballus clone 256 immunoglobulin mu heavy chain (IGHM)
mRNA, partial cds
CTGTCCCTCACCTGCACTGTCTCTGGATTCTCTTTGAGCAGTTACGGTGTAGGCTGGGTCCGCCAGGCTCCAGGAAA
AGGGCTGGAATATGTTGGTGGTATAGCTAGTAGTGGAAGTGCAAACTACAACCCAGCCCTGAAGTCCCGAGCCAGCA
TCACCAAGGACACCTCAAAGAGCCAAGTTTATCTGACGCTGAACAGCCTGACAGGCGAGGACACGGCCGTCTATTAC
TGTGCGAGACTTAATTACTGGGCGGATGGTATAAACTACTGGGGCCAGGGCATCCTGGTCACCGTCTCCTCAGAGAG
TACGATGACCCCAGATCTCTTCCCCCTCGTCTCCTGTGGGCCCTCTCTTGATGAGAGCCCGGTGGCTGTGGGCTGCC
TAGCCCGGGACTTCCTACCCAA
>KY437411.1 Equus caballus clone 255 immunoglobulin mu heavy chain (IGHM)
mRNA, partial cds
CTGTCCCTCACCTGCACTGTCTCTGGATTATCTTTGAGCAGTGATGCTGTAGGCTGGGTCCGCCAGGCTCCAGGAAA
GGGGCTGGAGTGGGTTGGTGTTATATATGTTAGTGAGCGTACACACTACAACCCAGCCCTGAAGTCCCGAGCCAGCA
TTACCACGGACACCTCAAAGAGCCAAGTTTATCTGACGCTGGACAGCCTGACAGGCGAAGACACGGCCGTCTATTAC
TGTGCAGGTTCCGAGGAGACCATGCGCATGGTGGTGTTCACTCGTCTCCCGTACTATTTTGGTTACTGGGGCCAGGG
CACCCTGGTCACCGTCTCCTCAGAGAGTACGATGACCCCAGATCTCTTCCCCCTCGTCTCCTGTGGGCCCTCTCTTG
ATGAGAGCCTGGTGGCTGTGGGCTGCCTAGCCCGGGACTTCCTACCCAA
>KY437410.1 Equus caballus clone 254 immunoglobulin mu heavy chain (IGHM)
mRNA, partial cds
CTCTCCCTCACCTGCACTGTCTCTGGATTATCTTTGAGCAGTAATGCTGTAGGCTGGGTCCGCCAGGCTCCAGGAAA
AGGGCTGGAATATGTTGGTGGTATAGCTAGTAGTGGAAGTGCAAACTACAACCCAGCCCTGAAGTCCCGAGCCAGCA
TCACCAAGGACACCTCAAAGAGCCAAGTTTATCTGACGCTGAACAGCCTGACAAGCGAGGACACGGCCGTCTATTAC
TGTGCAGGTTCTTATGGTTCCTATGCTGGTAGTTACTATGCGGCCATAATTTATGATATAGACTACTGGGGCCAGGG
CACCCTGGTCACCGTCTCCTCAGAGAGTACGAAGACCCCAGATCTCTTCCCCCTCGTCTCCTGTGGGCCCTCTCTTG
ATGAGAGCCTGGTGGCTGTGGGCTGCCTAGCCCGGGACTTCCTACCCAA
>KY437409.1 Equus caballus clone 253 immunoglobulin mu heavy chain (IGHM)
mRNA, partial cds
CTCTCCCTCACCTGCACTGTCTCTGGATTATCTTTGAGCAGTAATGGTGTAGTCTGGGTCCGCCAGGCTCCAGGAAA
AGGGCTGGAATATGTGGGTGCTATGCGTAGTAGTGGAAGTGCAAACTACAACCCAGCCCTGAAGTCCCGAGCCGCCA

TCACCAAGGACACCTCAAAGAGCCAAGTTTATCTGACGCTGAACAGCCTGACAAGCGAGGACACGGCCGTCTATTAC
TGTGCAGATATAGCAGATGCTTTACCAGACTCCTGGGGCCAGGGCATACTGGTCACCGTCTCCTCAGAGAGTACGAA
GACCCCAGATCTCTTCCCCCTCGTCTCCTGTGGGCCCTCTCTTGATGAGAGCCTGGTGGCTGTGGGCTGCCTAGCCC
GGGACTTCCTACCCAA
>KY437408.1 Equus caballus clone 252 immunoglobulin mu heavy chain (IGHM) mRNA, partial cds
CTCTCCCTCACCTGCACTGTCTCTGGATTATCTTTGAGCAGTAATTCTGTAGGCTGGGTCCGCCAGGCTCCAGGAAA
AGGGCTGGAATATGTTGGTGCTATAGATAGGAGTGGAAGTTTAGTATACAACCCAGCCCTGAAGTCCCGAGCCAGCA
TCACCAAGGACACCTCAAAGAGCCAAGTTTATCTGACGCTGAACAGCCTGACAAGCGAGGACACGGCCGTCTATCTT
TGTGCAGGAGGAGGCCATAATTATGGTACTTATTTTGCTTTTGGCTACTGGGGCCAGGGCACCCTGGTCACCGTCTC
CTCAGAGAGTACGATGACCCCAGATCTCTTCCCCCTCGTCTCCTGTGGGCCCTCTCTTGATGAGAGCCTGGTGGCTG
TGGGCTGCCTAGCCCGGGACTTCCTACCCAA
>KY437407.1 Equus caballus clone 251 immunoglobulin mu heavy chain (IGHM) mRNA, partial cds
CTCTCCCTCACCTGCACTATCTCTGGATTATCTTTGAGCAGTAATACTGTAGCCTGGGTCCGCCAGGCTCCAGGAAA
AGGGCTGGAATATGTTGGTGCCATGACTGGTAGTGGAAGTGCGTTTTACAACCCAGCCCTGAAGTCCCGAGCCAGCA
TCACCAAAGACACCTCAAAGAGCCAAGTTTATCTGACGCTGAACAGCCTGACAAGCGAGGACACGGCCGTCTATTAC
TGTTTAGGAGGCCCAGCTATAGCAGATATGCTTATAGAGTACTGGGGCCAGGGCATCCTGGTCACCGTCTCCTCAGA
GAGTACGAAGACCCCAGATCTCTTCCCCCTCGTCTCCTGTGGGCCCTCTCTTGATGAGAGCCTGGTGGCTGTGGGCT
GCCTAGCCCGGGACTTCCTACCCAA
>KY437406.1 Equus caballus clone 250 immunoglobulin mu heavy chain (IGHM) mRNA, partial cds
CTCTCCCTCACCTGCACTGTCTCTGGATTATCTTTGAGCAGTAATGCTGTAGGCTGGGTCCGCCAGGCTCCAGGAAA
AGGGCTGGAATATGTTGGTGGTATAGCTAGTAGTGGAAGTGCAAACTACAACCCAGCCCTGAAGTCCCGAGCCAGCA
TCACCAAGGACACCTCAAAGAGCCAAGTTTATCTGACGCTGAACAGCCTGAGAAGCGAGGACACGGCCGTCTATTAC
TGTGTAGGAGGCGTCTACTGGGGCCAGGGCACCCTGGTCACCGTCTCCTCAGAGAGTACGAAGACCCCAGATCTCTT
CCCCCTCGTCTCCTGTGGGCCCTCTCTTGATGAGAGCCTGGTGGCTGTGGGCTGCCTAGCCCGGGACTTCCTACCCA
A
>KY437405.1 Equus caballus clone 249 immunoglobulin mu heavy chain (IGHM) mRNA, partial cds
CTCTCCCTCACCTGCACTGTCTCTGGATTCTCTTTGAGCAGTTATGCTGTATACTGGGTCCGCCAGGCTCCAGGAAA
AGGGCTGGAATATGTTGGTGCTATAGCTAAAAGTGGAAATACAAACTACAACCCAGCCCTGAAGTCCCGAGCCAGCA
TCACCAAGGACACCTCCAAGAGCCAAGTTTATCTGACGCTGAACAGCCTGACAGGCGAGGACACGGCCGTCTATTAC
TGTGCAGGAGGCGTCTACTGGGGCCAGGGCACCCTGGTCACCGTCTCCTCAGAGAGTACGAAGACCCCAGATCTCTT
CCCCCTCGTCTCCTGTGGGCCCTCTCTTGATGAGAGCCTGGTGGCTGTGGGCTGCCTAGCCCGGGACTTCCTACCCA
A
>KY437404.1 Equus caballus clone 248 immunoglobulin mu heavy chain (IGHM) mRNA, partial cds
CTCTCCCTCACCTGCACTGTCTCTGGATTATCTTTGAGCAGTGTTGGTGTGGAGTGGGTCCGTCAGGCTCCAGGAAA
AGGGCTGGAATATGTTGCTGGTATACCTAGTAGTGGAAGTTTGGCCTACAATTCAGCCCTGAAGTCCCGAGCCAGCA
TCACCAAGGACACCTCAAAGAGCCAAGTTTATCTGACGCTGAACAGCCTGACAAGCGAGGACACGGCCGTCTATTAC
TGTGTAGGATCCCTGGGCTACTGGGGCCAGGGCATCCTGGTCACCGTCTCCTCAGAGAGTACGATGACCCCAGATCT
CTTCCCCCTCGTCTCCTGTGGGCCCTCTCTTGATGAGAGCCTGGTGGCTGTGGGCTGCCTAGCCCGGGACTTCCTAC
CCAA
>KY437403.1 Equus caballus clone 247 immunoglobulin mu heavy chain (IGHM) mRNA, partial cds
CTCTCCCTCACCTGCACTGTCTCTGGATTCTCTTTGAGCAGTTATGCTGTATACTGGGTCCGCCAGGCTCCAGGAAA
AGGGCTGGAATATGTTGGTGCTATAGCTAAAAGTGGAAATACAAACTACAACCCAGCCCTGAAGTCCCGAGCCAGCA
TCACCAAGGACACCTCCAAGAGCCAAGTTTATCTGACGCTGAACAGCCTGACAGGCGAGGACACGGCCGTCTATTAC
TGTGCGAAAGATTATTTTGGTTATGGTGGTACTTTGTACTATGATATAGACTACTGGGGCCAGGGCACCCTGGTCAC
CGTCTCCTCAGAGAGTACGATGACCCCAGATCTCTTCCCCCTCGTCTCCTGTGGGCCCTCTCTTGATGAGAGCCTGG
TGGCTGTGGGCTGCCTAGCCCGGGACTTCCTACCCAA
>KY437402.1 Equus caballus clone 246 immunoglobulin mu heavy chain (IGHM) mRNA, partial cds

CTCTCCCTCACCTGCACTGTCTCTGGGGTATCTTTGAGCAGTAATAATGTAGGCTGGGTCCGCCAGGCTCCAGGAAA
AGGGCTGGAATTTGTTGCCAAGATAACTGATAGTGGAAGTGTGTACTACAACCCAGCCCTGAAGTCCCGAGCCAGCA
TCACCAAGGACACCTCAAAGAGCCAACATTATCTGACGCTGAACAGCCTGAGAAGCGAGGACACGGCCGTCTATTAC
TGTGTAGTCTACGATTATGGTAAACCTGGCTACTGGGGCCAGGGCACCCTGGTCACCGTCTCCTCAGAGAGTACGAT
GACCCCAGATCTCTTCCCCCTCGTCTCCTGTGGGCCCTCTCTTGATGAGAGCCTGGTGGCTGTGGGCTGCCTAGCCC
GGGACTTCCTACCCAA
>KY437401.1 Equus caballus clone 245 immunoglobulin mu heavy chain (IGHM) mRNA, partial cds
CTCTCCCTCACCTGCACTGTCTCTGGATTATCTTTGAGCAGTAATACTGTAGGCTGGGTCCGCCAGGCTCCAGGAAA
AGGGCTGGAATATGTTGGTGGAGTAGTGCCTAGTGGAAGTGCAAGGTACAACCCAGCCCTGAAGTCCCGAGCCAGCA
TCACCGAGGACACCTCAAAGAGCCAAGCCTATCTGACGCTGAACAGCCTGACAAGCGAGGACACGGCCGTCTATTAC
TGTGCAGGAGGTAGTGGTGATAGCTACCATTATGGTATCCACTACTGGGGCCAGGGCATCCTGGTCACCGTCTCCTC
AGAGAGTACGATGACCCCAGATCTCTTCCCCCTCGTCTCCTGTGGGCCCTCTCTTGATGAGAGCCTGGTGGCTGTGG
GCTGCCTAGCCCGGGACTTCCTACCCAA
>KY437400.1 Equus caballus clone 244 immunoglobulin mu heavy chain (IGHM) mRNA, partial cds
CTGTCCCTCACCTGCACTGTCTCTGGATTCTCTTTGAGCAGTTACGGTGTAGGCTGGGTCCGCCAGGCTCCAGGAAA
AGGGCTGGAATATGTTGGTGGTATAGTTAGTAGTGGAAGTGCAAACTACAACCCAGCCCTGAAGTCCCGAGCCAGCA
TCACCAAGGACACCTCAAAGAGCCAAGTTTATCTGACGCTGAACAGCCTGACAGGCGAGGACACGGCCGTCTATTAC
TGTGCGAGAGGGTGGTCTGATTATGGTTATGGTGGTGCTTACTACTATTTTGGCTACTGGGGCCAGGGCACCCTGGT
CACCGTCTCCTCAGAGAGTACGATGACCCCAGATCTCTTCCCCCTCGTCTCCTGTGGGCCCTCTCTTGATGAGAGCC
TGGTGGCTGTGGGCTGCCTAGCCCGGGACTTCCTACCCAA
>KY437399.1 Equus caballus clone 243 immunoglobulin mu heavy chain (IGHM) mRNA, partial cds
CTCTCCCTCACCTGCACTGTCTCTGGATTATCTTTGAGCAGTAATACTGTAGGCTGGGTCCGCCAGGCTCCAGGAAA
AGGGCTGGAATATGTTGGTGGTATACATAGTAGTGGAAGTGTTAGTGGAAGTGCAAACTACAACCCAGCCCTGAAGT
CCCGAGCCAGCATCACCAAGGACACCTCAAAGAGCCAAGTTTATCTGACGCTGAACAGCCTGACAAGCGAGGACACG
GCCGTCTATTACTGTGCAGGAGGCCCCTATATCGGTTATATAATGGGTGGTGCGGACTACTGGGGCCAGGGCATCCT
GGTCACCGTCTCCTCAGAGAGTACGAAGACCCCAGATCTCTTCCCCCTCGTCTCCTGTGGGCCCCCTCTTGATGAGA
GCCTGGTGGCTGTGGGCTGCCTAGCCCGGGACTTCCTACCCAA
>KY437398.1 Equus caballus clone 242 immunoglobulin mu heavy chain (IGHM) mRNA, partial cds
CTCTCCCTCACCTGCACTGTCTCTGGATTATCTGCGAGCAGTAATGCTCTAGGCTGGGTCCGCCAGGCTCCAGGAAA
AGGGCTGGAATATGTTGGTGGCGTAGCTAAAAGTGGAAGTGCGTACTACAACCCAGCCCTGAAGTCCCGAGCCACCA
TCACCAAGGACACCTCAAAGAGCCAACTTTATCTGACGCTGAACAGCCTGACAAGCGAGGACACGGCCGTCTATTAC
TGTGCAGGAGGCGAGTATGTCGGCGATTTTACCTACTGGGGCCAGGGCACCCTGGTCACCGTCTCCTCAGAGAGTAC
GATGACCCCAGATCTCTTCCCCCTCGTCTCCTGTGGGCCCTCTCTTGGTGAGAGCCTGGTGGCTGTGGGCTGCCTAG
CCCGGGACTTCCTACCCAA
>KY437397.1 Equus caballus clone 241 immunoglobulin mu heavy chain (IGHM) mRNA, partial cds
CTCTCCCTCACCTGCACTGTCTCTGGATTATCTTTGAGCAGTACTGTTGTAGGCTGGGTCCGCCAGGCTCCAGGAAA
AGGGCTGGTTTTTGTTGGTTTCGCAGTTGGTTATGCAAGTGCAAAGTACAACCCAGCCCTGAAGTCCCGAGCCAGCA
TCACCGAGGACACCTCAAAGAGCCAAGTTTATCTGACGCTGAACAGCCTGACAAGCGAGGACACGGCCGTCTATTAC
TGTGCAGGAGTCGACGTTAATGAGGGCTATTTTGGCTACTGGGGCCAGGGCACCCTGGTCACCGTCTCCTCAGAGAG
TACGATGACCCCAGATCTCTTCCCCCTCGTCTCCTGTGGGCCCTCTCTTGATGAGAGCCTGGTGGCTGTGGGCTGCC
TAGCCCGGGACTTCCTACCCAA
>KY437396.1 Equus caballus clone 240 immunoglobulin mu heavy chain (IGHM) mRNA, partial cds
CTCTCCCTCACCTGCACTGTCTCTGGATTCTCTTTGAGCAGTTACGCAGTAGGCTGGGTCCGCCAGGCTCCAGGAGA
AGGGCTGGAATTTGTTGGTATGATAGGTAACAGCGGAAGTACATACTACAACCCAGCCCTGAAGTCCCGAGCCAGCA
TCACCAAGGACACCTCAAAGAGCCAAGTTTCTCTGACGCTGAACAGCCTGACAGGCGAGGACACGGCCGTCTATTAC
TGTGCGAGATTTTCGAGGAGCTATAATAATGGCATATCGGATTGGGGCCAGGGCATCCTGGTCACCGTCTCCTCAGA
GAGTACGATGACCCCAGATCTCTCCCCCCTCGTCTCCTGTGGGCCCTCTCTTGATGAGAGCCTGGTGGCTGTGGGCT
GCCTAGCCCGGGACTTCCTACCCAA
>KY437395.1 Equus caballus clone 239 immunoglobulin mu heavy chain (IGHM) mRNA, partial cds

CTCTCCCTCACCTGCACTGTCTCTGGATTATCTTTGAGCAGTAATGCTGTAGGCTGGGTCCGCCAGGCTCCAGGAAA
AGGGCTGGAATTTGTTGGTGCTATATATGGTAGTGCAAGTGCAAACTACAACCCAGCCCTGAAGTCCCGAGCCAGCA
TCACCAAGGACACCTCAAAGAGCCAAGTTTATCTGACGCTGAACAGCCTGACAAGCGAGGACACGGCCGTCTATTAC
TGTGCAGGAGGCCTTGGCTCTATGGTAGTTACTACAGTAGTTACTATGCCGAATTTTGGCTACTGGGGCCAGGGCAC
CCTGGTCACCGTCTCCTCAGAGAGTACGATGACCCCAGATCTCTTCCCCCTCGTCTCCTGTGGGCCCTCTCTTGATG
AGAGCCTGGTGGCTGTGGGCTGCCTAGCCCGGGACTTCCTACCCAA
>KY437394.1 Equus caballus clone 238 immunoglobulin mu heavy chain (IGHM) mRNA, partial cds
CTCTCCCTCACCTGCACTGTCTCTGGATTATCTTTGAGCAGTAATGGTGTAGGCTGGGTCCGCCAGGCTCCAGGAAA
AGGGCTGGAATGGGTTGGTGCAATATATGAAAGTGCAAGTGCGAACTACAACCCAGCCCTGAAGTCCCGAGCCAGCA
TCACCAAGGACACCACAAAGAGCCAAGTTTATCTGACGCTGAACAGCCTGACAAGCGAGGACACGGCCGTCCATTAC
TGTGCAGGAGAACGAAGACTGAACTACGGCTATGGTCCTATAAACTACTGGGGCCAGGGCATCCTGGTCACCGTCCC
CTCAGAGAGTACGATGACCCCAGATCTCTTCCCCCTCGTCTCCTGTGGGCCCTCTCTTGATGAGAGCCTGGTGGCTG
TGGGCTGCCTAGCCCGGGACTTCCTACCCAA
>KY437393.1 Equus caballus clone 237 immunoglobulin mu heavy chain (IGHM) mRNA, partial cds
CTCTCCCTCACCTGCACTGTCTCTGGATTATCTTTGAGCAGTAATGCTGTAGGCTGGGTCCGCCGGGCTCCAGGAAA
AGGGCTGGAATTTGTTGGTGCTATATATGGTAGTGCAAGTCCAGACTACAACCCAACCCTGAAGTCCCGAGCCAGCC
TCACCAAGGACACCTCAAAGAGCCAAGTTTATCTGACGCTGAACAGCCTGACAAGCGAGGACACGGCCGTCTATTAC
TGTACAGAAGGGGGTTATAGATACTGGGGCCAGGGCACCCTGGTCACCGTCTCCTCAGAGAGTACGATGACCCCAGA
TCTCTTCCCCCTCGTCTCCTGTGGGCCCTCTCTTGATGAGAGCCTGGTGGCTGTGGGCTGCCTAGCCCGGGACTTCC
TACCCAA
>KY437392.1 Equus caballus clone 236 immunoglobulin mu heavy chain (IGHM) mRNA, partial cds
CTCTCCCTCACCTGCACTGTCTCTGGATTATCTTTGAGCAGTAATGTTGTAGCATGGGTCCGCCAGGCTCCAGGAAA
AGGGCTGGAATATGTTGGAAGTATAGCTAGTAGTGGAAGTGCAATGTACAACCCAGCCCTGAAGTCCCGAGCCAGCA
TCACCAGGGACACCTCAAAACGCCAACTTTATCTGACGCTGAACAGCCTGACAAGCGAGGACACGGCCGTCTATTAC
TGTAGAGGAGTTGTCTCGTGGGGCCAGGGCACCCTGGTCACCGTCTCCTCAGAGAGTACGATGACCCCAGATCTCTT
CCCCCTCGTCTCCTGTGGGCCCTCTCTTGATGAGAGCCTGGTGGCTGTGGGCTGCCTAGCCCGGGACTTCCTACCCA
A
>KY437391.1 Equus caballus clone 235 immunoglobulin mu heavy chain (IGHM) mRNA, partial cds
CTCTCCCTCACCTGCACTGTCTCTGGATTATCTTTGAGCAGTAATGGTGTAGGCTGGGTCCGCCAGGCTCCAGGAAA
AGGGCTGGAATGGGTTGGTGCAATATATGAAAGTGCAAGTGCGAACTACAACCCAGCCCTGAAGTCCCGAGCCAGCA
TCACCAAGGACACCACAAAGAGCCAAGTTTATCTGACGCTGAACAGCCTGACAAGCGAGGACACGGCCGTCTATTAC
TGTGCAGGAGAACGAAGACTGAACTACGGTTATGGTCCTATAAACTACTGGGGCCAGGGCATCCTGGTCACCGTCTC
CTCAGAGAGTACGATGACCCCAGATCTCTTCCCCCTCGTCTCCTGTGGGCCCTCTCTTGATGAGAGCCTGGTGGCTG
TGGGCTGCCTAGCCCGGGACTTCCTACCCAA
>KY437390.1 Equus caballus clone 234 immunoglobulin mu heavy chain (IGHM) mRNA, partial cds
CTGTCCCTCACCTGCACTGTCTTTGGATTATCTTTGAGCAGTGGTGCTGTAGGCTGGGTCCGCCAGGCTCCAGGAAA
AGGGCTGGAGTGGGTTGCTGATCTATATGGTAGTGGTAATACATACTACAACCCAGCCCTGAAGTCCCGAGCCAGCA
TCACCAAGGACACCTCAAAGAGCCAAGTTTATCTGACGCTAAACAGCCTGACAGGCGAAGACACGGCCGTCTATTAC
TGTGCAGGACGGTCCTATGATTATGGTGGTGCTTTTGCCTACTGGGGCCAGGGCACCCTGGTCACCGTCTCCTCAGA
GAGTACGATGACCCCAGATCTCTTCCCCCTCGTCTCCTGTGGGCCCTCTCTTGATGAGAGCCTGGTGGCTGTGGGCT
GCCTAGCCCGGGACTTCCTACCCAA
>KY437389.1 Equus caballus clone 233 immunoglobulin mu heavy chain (IGHM) mRNA, partial cds
CCCTCCCTCACCTGCACTGTCTCTGGATTATCTTTGAGCAGTAATGCTGTAGGCTGGGTCCGCCAGGCTCCAGGAAA
AGGGCTGGAATACGTTGGTAGTATATCGCGTAGTGCAAGTGGTGCTATATCGGGTAGTGCAAGTGCAAACTACAACC
CAGCCCTGAAGTCCCGAGCCAGCATCACCAAGGACCCCTCAAAGAGCCAAGTTTATCTGACGCTGAACAGCCTGACA
GGCGAGGACACGGCCGTCTATTACTGTGCGAGCTCTGGTGAAGGCTATTTTGGCTACTGGGGCCAGGGCACCCTGGT
CACCGTCTCCTCAGAGAGTACGATGACCCCAGATCTCTTCCCCCTCGTCTCCTGTGGGCCCTCTCTTGATGAGAGCC
TGGTGGCTGTGGGCTGCCTAGCCCGGGACTTCCTACCCAA

>KY437388.1 Equus caballus clone 232 immunoglobulin mu heavy chain (IGHM) mRNA, partial cds
CTCTCCCTCACCTGCACTGTCTCTGGATTATCTTTGAGCAGTAATACTGTAGGCTGGGTCCGCCAGGCTCCAGGAAA
AGGGCTGGAATATGTTGGTGAAATAGCTAGTAGTGGAAGTGCAAACTACAACCCAGCCCTGAAGTCCCGAGCCAGCA
TCACCAAGGACACCTCAAAGAGCCAAGTTTATCTGACGCTGAACAGCCTGACAAGCGAGGACACGGCCGTCTATTAC
TGTGCAGGATCAACAGCTATGCTAGATTATGGTATAGACTACTGGGGCCAGGGCATCCTGGTCACCGTCTCCTCAGA
GAGTACGAAGACCCCAGATCTCTTCCCCCTCGTCTCCTGTGGGCCCTCTCTCGATGAGAGCCTGGTGGCTGTGGGCT
GCCTAGCCCGGGACTTCCTACCCAA
>KY437387.1 Equus caballus clone 231 immunoglobulin mu heavy chain (IGHM) mRNA, partial cds
CTCTCCCTCACCTGCACTGTCTCTGGATTCTCTTTGAGCAGTTATGCTGTAGGCTGGGTCCGCCAGGCTCCAGGAAA
AGGGCTGGAATATGTTGGTGCTATATATGGTAGTGCAAGTGCAAACTACAACCCAGCCCTGAAGTCCCGAGCCAGCA
TCACCAAGGACACCTCAAAGAGCCAAGTTTATCTGACGCTGAACAGCCTGACAGGCGAGGACACGGCCGTCTATTAC
TGTGCGAGAGGAGGCTTCATCTCATCGGGGGATGGTTATGGTGGTGCTTACTACTCGCCCTTTGGCTACTGGGGCCA
GGGCACCCTGGTCACCGTCTCCTCAGAGAGTACGATGACCCCAGATCTCTTCCCCCTCGTCTCCTGTGGGCCCTCTC
TTGATGAGAGCCTGGTGGCTGTGGGCTGCCTAGCCCGGGACTTCCTACCCAA
>KY437386.1 Equus caballus clone 230 immunoglobulin mu heavy chain (IGHM) mRNA, partial cds
CTCTCCCTCATCTGCACTGTCTCTGGATTATCTTTGAGCAGTTATGGTGTGGGCTGGGTCCGCCAGGCTCCAGGAAA
AGGGCTGGAATTTGTTGGTGGTATAGCTAGTAGTGGAAGTGCAAACTACAACCCAGCCCTGAAGTCCCGAGCCAGCA
TCACCAAGGACACCTCAAAGAGCCAAGTTTATCTGACGCTGAACAGCCTGACAAGCGAGGACACGGCCGTCTATTAC
TGTGCGAGAACGAACGGTTATGCATATATGGTTTTGGCTACTGGGGCCAGGGCACCCTGGTCACCGTCTCCTCAGA
GAGTACGATGACCCCAGATCTCTTCCCCCTCGTCTCCTGTGGGCCCTCTCTTGATGAGAGCCTGGTGGCTGTGGGCT
GCCTAGCCCGGGACTTCCTACCCAA
>KY437385.1 Equus caballus clone 229 immunoglobulin mu heavy chain (IGHM) mRNA, partial cds
CTCTCCCTCACCTGCACTGTCTCTGGATTATCTTTGAGCAGTAATGCTGTAGGCTGGGTCCGCCAGGCTCCAGGAAA
AGGGCTGGAATTTGTTGGTGCTATATATGGTAGTGCAAGTGCAAACTACAACCCAGCCCTGAAGTCCCGAGCCAGCA
TCACCAAGGACACCTCAAAGAGCCAAGTTTATCTGACGCTGAACAGCCTGACAAGCGAGGACACGGCCGTCTATTAC
TGTGCAGGAAGTAATAGAGTGAGAGCTTACGGGGAGTCCATTTATGGTATAAACTACTGGGGCCAGGGCATCCTGGT
CACCGTCTCCTCAGAGAGTACGATGACCCCAGATCTCTTCCCCCTCGTCTCCTGTGGGCCCTCTCTTGATGAGAGCC
TGGTGGCTGTGGGCTGCCTAGCCCGGGACTTCCTACCCAA
>KY437384.1 Equus caballus clone 228 immunoglobulin mu heavy chain (IGHM) mRNA, partial cds
CTCTCCCTCACCTGCACTGTCTCTGGATTATCTTTGAGCAGTGCTACTGTAGGCTGGGTCCGCCAGGCTCCAGGAAA
AGGGCTGGAATATGTTGGTCATATCGCAGGTAGTGGAAGTGCGTACTACAACCCAGCCCTGAAGTCCCGAGCCAGCA
TCACCAAGGACACCTCAAAGAGCCAAGTTTATCTGACGCTGAACAGCCTGACAAGCGAGGACACGGCCGTTTATTAC
TGTGCGAGATTTTTCTATAGTAATGGTAATTACATCACATATTTTGACTACTGGGGCCAGGGCACCCTGGTCACCGT
CTCCTCAGAGAGTACGATGACCCCAGATCTCTTCCCCCTCGTCTCCTGTGGGCCCTCTCTTGATGAGAGCCTGGTGG
CTGTGGGCTGCCTAGCCCGGGACTTCCTACCCAA
>KY437383.1 Equus caballus clone 227 immunoglobulin mu heavy chain (IGHM) mRNA, partial cds
CTGTCCCTCACCTGCACTGTCTCTGGCTTATCTTTGAGCAGTAATGGTGTAGCCTGGGTCCGCCAGGCTCCAGGAAA
AGGGCTGGAGTGGGTTGGTGATATATATAGTGAAAGTACATCGTACAACCCAGCCCTGAAGTCCCGAGCCAGCATCA
CCAAGGACACCTCAAAGAGCCAAGTTTATCTGACGCTGAACAGCCTGACAGGCGAAGACACGGCCGTCTATTACTGT
GCAGGATGGCCCAGTGATTATGGTGGTGCTTACTACTACTGGGGCCAGGGCACCCTGGTCACCGTCTCCTCAGAGAG
TACGATGACCCCAGATCTCTTCCCCCTCGTCTCCTGTGGGCCCTCTCTTGATGAGAGCCTGGTGGCTGTGGGCTGCC
TAGCCCGGGACTTCCTACCCAA
>KY437382.1 Equus caballus clone 226 immunoglobulin mu heavy chain (IGHM) mRNA, partial cds
CTCTCCCTCACCTGCACTGTCTCTGGATTATCTTTGAGCAGTTATGCTGTAACCTGGGTCCGCCAGGCTCCAGGAAA
AGGGCTGGAATATGTTGGTAGTATAGATAGAAGTGGAAGTGCAAACTACCTCCCAGCCCTGAAGTCCCGAGCCAGCA
TCACCAAGGACACCTCAAAGAGCCAAGTTTATCTGACGCTGAACAGCCTGACAAGCGAGGACACGGCCGTCTATTTT
TGTAGAGGAGGGCTGCACTGGGGCCAGGGCACCCTGGTCACCGTCTCCTCAGAGAGTACGAAGACCCCAGATCTCTT
CCCCCTCGTCTCCTGTGGGCCCTCTCTTGATGAGAGCCTGGTGGCTGTGGGCTGCCTAGCCCGGGACTTCCTACCCA
A

>KY437381.1 Equus caballus clone 225 immunoglobulin mu heavy chain (IGHM) mRNA, partial cds
CTGTCCCTCACCTGCACTGTCTCTGGATTATCTTTGAGCAGTAATGCTGTAGGCTGGGTCCGCCAGACTCCAGGAAA
AGGGCTGGAAAGAGTTGGTGATATAACGAGTCTCGGAACGGCATACTACAACTCAGCCCTGAAGTCCCGAGCCAGCA
TCACCAAGGACACCTCAAAGAGCCAAGTTTATCTGACGCTGAATAGCCTGACGAACGAAGACTCGGCCGTCTATTAC
TGTGCAGGAGGGGAGTGGGGTAATACTTACTTCTATGCCTACGTGGAGCACTGGGGCCAGGGCATCCTGGTCACCGT
CTCCTCAGAGAGTACGAAGACCCCAGATCTCTTCCCCCTCGTCTCCTGTGGGCCCTCTCTTGATGAGAGCCTGGTGG
CTGTGGGCTGCCTAGCCCGGGACTTCCTACCCAA
>KY437380.1 Equus caballus clone 224 immunoglobulin mu heavy chain (IGHM) mRNA, partial cds
CTCTCCCTCACCTGCACTGTCTCTGGATTATCTTTGAGCAGTAATGCTGTAGGCTGGGTCCGCCAGGCTCCAGGAAA
AGGGCTGGAATATGTTGGTGGTATAGCTAGTAGTGGAAGTGCAAACTACAACCCAGCCCTGAAGTCCCGAGCCAGCA
TCACCAAGGACACCTCAAAGAGCCAACTTTATCTGACGCTGAACAGCCTGACAAGCGAGGACACGGCCGTCTATTAC
TGTGCAGGAAGGCAGGATAACTACGGTTCTGGTTATGCTACTAATTATTTTGGTATAAACTACTGGGGCCAGGGCAT
CCTGGTCACCGTCTCCTCAGAGAGTACGAAGACCCCAGATCTCTTCCCCCTAGAAACCTGTGGGCCCTCTCTTGATG
AGAGCCTGGTGGCTGTGGGCTGCCTAGCCCGGGACTTCCTACCCAA
>KY437379.1 Equus caballus clone 223 immunoglobulin mu heavy chain (IGHM) mRNA, partial cds
CTCGCCCTCACCTGCACTGTCTCTGGATTATCTTTGAACAGTAATGCTGTAGGCTGGGTCCGCCAGGCTCCAGGAAA
AGGGCTGGAATATGTTGGTGACATAGAGAGTAGTGGAAGTGCAAACTACAACCCAGCCCTGAAGTCCCGAGCCAGCA
TCACCAAGGACACCTCAAAGAGCCAAGTTTATCTGACGCTGAACAGCCTGACAAGCGAGGACACGGCCGTCTATTAC
TGTGCAGGCCACATCGTGCCTTACGTAGATGGTTGGCAGATAGGCTATTTTGCCTACTGGGGCCAGGGCACCCTGGT
CACCGTCTCCTCAGAGAGTACGATGACCCCAGATCTCTTCCCCCTCGTCTCCTGTGGGCCCTCTCTTGATGAGAGCC
TGGTGGCTGTGGGCTGCCTAGCCCGGGACTTCCTACCCAA
>KY437378.1 Equus caballus clone 222 immunoglobulin mu heavy chain (IGHM) mRNA, partial cds
CTCTCCCTCACCTGCACTGTCTCTGGATTATCTTTGAGCAGTAATCGTGTAGCCTGGGTCCGCCAGGCTCCAGGAAA
AGGGCTGGAATGTGTTGGTTCTATATATGGTAGTGCAAGTGCAAACTACAACCCAGCCCTGAAGTCCCGAGCCAGCA
TCACCAAGGACACCTCAAAGAGCCAAGTTCATCTGGCGCTGAACAGCCTGACAAGCGAGGACACGGCCGTCTATTAC
TGTACAGGTGTCGGATTAAAATACTGGGGCCAGGGCGCCCTGGTCACCGTCTCCTCAGAGAGTACGAAGACCCCAGA
TCTCTTCCCCCTCGTCTCCTGTGGGCCCTCTCTTGATGAGAGCCTGGTGGCTGTGGACTGCCTAGCCCGGGACTTCC
TACCCAA
>KY437377.1 Equus caballus clone 221 immunoglobulin mu heavy chain (IGHM) mRNA, partial cds
CTCTCCCTCACCTGCACTGTCTCTGGATTATCTTTGAGCAGTAATGCTGTAGGCTGGGTCCGCCAGGCTCCAGGAAA
AGGGCTGGAATATGTTGGTAGTATAGCTAGTAGTGGAAGTGCAAACTACAACCCAGCCCTGAAGTCCCGAGCCAGCA
TCACCAAGGACACCTCAAAGAGCCAAGTTTATCTGACGCTGAACAGCCTGACAAGCGAGGACACGGCCGTCTATTAC
TGTGCAGGAAACGAGTATGGTTATGCTACAGTATCGAGTATAAACTACTGGGGCCAGGGCATCCTGGTCACCGTCTC
CTCAGAGAGTACGATGACCCCAGATCTCTTCCCCCTCGTCTCCTGTGGGCCCTCTCTTGATGAGAGCCTGGTGGCTG
TGGGCTGCCTAGCCCGGGACTTCCTACCCAA
>KY437376.1 Equus caballus clone 220 immunoglobulin mu heavy chain (IGHM) mRNA, partial cds
CTCTCCCTCACCTGCACTGTCTCTGGATTATCTTTGAGAGGTAGTGCTGTAGGCTGGGTCCGCCAGGCTCCAGGAAA
AGGGCTGGAATATGTTGGTGTTATAAGTAGTAGTGGAAGTGCAAACTACAACCCAGCCCTGAAGTCCCGAGCCAGCA
TCACCAGGGACACCTCAAAGAGTCAACTTTATCTGACGCTGAACAGCCTGACAAGCGAGGACACGGCCGTCTATTAC
TGTGCAGGAAACGAGTATGGTTATGCTACAGTATCGAGTATAAACTACTGGGGCCAGGGCATCCTGGTCACCGTCTC
CTCAGAGAGTACGATGACCCCAGATCTCTTCCCCCTCGTCTCCTGTGGGCCCTCTCTTGATGAGAGCCTGGTGGCTG
TGGGCTGCCTAGCCCGGGACTTCCTACCCAA
>KY437375.1 Equus caballus clone 219 immunoglobulin mu heavy chain (IGHM) mRNA, partial cds
CTCTCCCTTACTTGCACTGTCTCTGGATTATCTTTGAGCAGTAATATCGTTTTCTGGGTCCGCCAGGCTCCAGGAAA
AGGGCTGGAATATGTTGGTATAATAGACACTAGTGGAAATTTAAACTACAACCCAGCCCTGAAGTCCCGAGCCAGCA
TCACCAGGGACACCTCAAAGAGCCAAGTTTATCTGACCCTGAACAGCCTGACAAGCGAGGACACGGCCGTCTATTAC
TGTGTAGGAGGGCTGTATTGGGGCCAGGGCACCCTGGTCACCGTCTCCTCAGAGAGTACGAAGACCCCAGATCTCTT

CCCCCTCGTCTCCTGTGGGCCCTCTCTTGATGAGAGCCTGGTGGCTGTGGGCTGCCTAGCCCGGGACTTCCTACCCA
A
>KY437374.1 Equus caballus clone 218 immunoglobulin mu heavy chain (IGHM)
mRNA, partial cds
CTGTCCCTCACCTGCACTGTCTCTGGATTATCTTTGAGCGGTAACGCTGTAAACTGGGTCCGCCAGACTCCAGGAAA
AGGGCTGGAGTGGGTTGCCATGGTACGTGGTAGTGGAAATACATACTACAACCCAGCCCTGAAGTCCCGAGCCAGCA
TCACCAAGGACACCTCAAAGAGCCAAGTTTATCTGACGCTGAACAGCCTGACAGGCGAGGACACGGCCGTCTATTAC
TGTGTGACCATAAGCAGGACATCTTTTGGCTACAGCAGGGAATCTTTTGGCTACTGGGGCCAGGGCACCCTGGTCAC
CGTCTCCTCAGAGAGTACGATGACCCCAGATCTCTTCCCCCTCGTCTCCTGTGGGCCCTCTCTTGATGAGAGCCTGG
TGGCTGTGGGCTGCCTAGCCCGGGACTTCCTACCCAA
>KY437373.1 Equus caballus clone 217 immunoglobulin mu heavy chain (IGHM)
mRNA, partial cds
CTCTCCCTCACCTGCACTGTCTCTGGATTATCTTTGAGCAGTAATAATGTAGCATGGGTCCGCCAGGCTNCAGGAAA
AGGGCTGGAATATGTTGGTGTTATAACTAACAGTGGAAGTACAGCCTACAACACAGCCCTGAAGTCCCGAGCCAGCA
TCACCAAGGACACCTCAAAGAGCCAAGTTTATCTGACGCTGAACAGCTTGACAAGCGAGGACACGGCCGTCTATTAC
TGTACAACTCTTTTGCGGTACTGGGGCCAGGGCACCCTGGTCACCGTCTCCTCAGAGAGTACGAAGACCCCAGATCT
CTTCCCCCTCGTCTCCTGTGGNCCCTCTCTTGATGAGAGCCTGGTGGCTGTGGGCTGCCTAGCCCGGGACTTCCTAC
CCAA
>KY437372.1 Equus caballus clone 216 immunoglobulin mu heavy chain (IGHM)
mRNA, partial cds
CTCTCCCTCACCTGCACTGTCTCTGGATTATCTTTGAGCAGTAAATCTGTGCGCTGGGTCCGCCAGGCTCCAGGAAA
AGGGCTGGAATATGTTGGTGGTATAGCCTCTAGTGGAAGTGCTTTCTACAACCCAGCCCTGAAGTCCCGAGCCAGCA
TCACCAAGGACACCTCAAAGAGCCAAGTTTATCTGACGCTGAACAGCCTGACAAGCGAGGACACGGCCGTCTATTAC
TGTCAGTCCGGGAACTACTGGGGCCAGGGCATCCTGGTCACCGTCTCCTCAGAGAGTACGATGACCCCAGATCTCTT
CCCCCTCGTCTCCTGTGGGACCTCTCTTGATGAGAGCCTGGTGGCTGTGGGCTGCCTAGCCCGGGACTTCCTACCCA
A
>KY437371.1 Equus caballus clone 215 immunoglobulin mu heavy chain (IGHM)
mRNA, partial cds
CTGTCCCTCACCTGCACTGTCTCTGGATTATCTTTGAGCAGTAATGCTGTAGGCTGGGTCCGCCAGGCTCCAGGAAA
AGGGCTGGAATATGTCGGTATTATGGGGGCGAGTGGAAGTACAAACTACAACCCAGCCCTGAAGTCCCGAGCCAGCA
TCACCAAGGACACCTCAAAGAGCCAAGTTTATCTGACGCTGACCAGCCTGACAAGCGAGGACACGGCCGTCTATTAC
TGTAAAGGAAGCAGTAACGCTGGCCTCGAAGAAATAAAGTACTGGGGCCAGGGCATCCTGGTCACCGTCTCCTCAGA
GAGTACGATGACCCCAGATCTCTTCCCCCTCGTCTCCTGTGGGCCCTCTCTTGATGAGAGCCTGGTGGCTGTGGGCT
GCCTAGCCCGGGACTTCCTACCCAA
>KY437370.1 Equus caballus clone 214 immunoglobulin mu heavy chain (IGHM)
mRNA, partial cds
CTCTCCCTCACCTGCACTGTCTCTGGATACTCTTTGAGCAGTAATGCTGTAGGCTGGGTCCGCCAGGCTCCAGGAAA
AGGGCTGGAATATGTTGGTGGTATACCTAGTAGTGGAGGTGCGTGGTACAACCCAGCCCTGAAGTCCCGAGCCAGCA
TCACCAAGGACACCTCAAAGAGCCAACATTATCTGACGCTGAACAGCCTGACAAGCGAGGACACGGCCGTCTATTAC
TGTGCAGGATATGACTATGGTGATGATTTCTACAGTATTGTGGACTACTGGGGCCAGGGCATCCTGGTCACCGTCTC
CTCAGAGAGTACGATGACCCCAGATCTCTTCCCCCTCGTCTCCTGTGGGCCCTCTCTTGATGAGAGCCTGGTGGCTG
TGGGCTGCCTAGCCCGGGACTTCCTACCCAA
>KY437369.1 Equus caballus clone 213 immunoglobulin mu heavy chain (IGHM)
mRNA, partial cds
CTCTCCCTCACCTGCACTGTCTCTGGATTCTCTTTGAGCAGTTATGCTGTAGGCTGGGTCCGCCAGGCTCCAGGAAA
AGGGCTGGAATGTGTTGGTTATGCATATGGTAGTGTAAGTACAAAGTACAACCCAGCCCTGAAGTCCCGAGCCAGCA
TCACCAAGGACACCTCAAAGAGCCAAGTTTATCTGACGCTGAACAGCCTGACAAGCGAGGACACGGCCGTCTATTAC
TGCTCAATATACGGCTACTGGGGCCAGGGCACCCTGGTCACCGTCTCCTCAGAGAGTACGATGACCCCAGATCTCTT
CCCCCTCGTCTCCTGTGGGCCCTCTCTTGATGAGAGCCTGGTGGCTGTGGGCTGCCTAGCCCGGGACTTCCTACCCA
A
>KY437368.1 Equus caballus clone 212 immunoglobulin mu heavy chain (IGHM)
mRNA, partial cds
CTGTCCCTCACCTGCACTGTCTCTGGATACTCTTTGAGCAGTGCTACTGTAGGCTGGGTCCGCCAGGCTCCAGGAAA
AGGGCTGGAATATGTTGGTGGTATAGCTAGTAGTGAAAGTGCACACTACAACCCAGCCCTGAAGTCCCGAGCCAGCA
TCACCAAGGACACCTCAAAGAGCCAAGTTTATCTGACGCTGAACAGCCTGACAGGCGAAGACACGGCCGTCTATTAC
TGTGCTTTCTTTATTTCCCCGGGGTATTTTGACTACTGGGGCCAGGGCACCCTGGTCACCGTCTCCTCAGAGAGTAC

GATGACCCCAGATCTCTTCCCCCTCGTCTCCTGTGGGCCCTCTCTTGATGAGAGCCTGGTGGCTGTGGGCTGCCTAG
CCCGGGACTTCCTACCCAA
>KY437367.1 Equus caballus clone 211 immunoglobulin mu heavy chain (IGHM) mRNA, partial cds
CTCTCCCTCACCTGCACTGTCTCTGGATTATCTTTGAGCAGTAATGATGTAGGCTGGGTCCGCCAGGCTCCAGGAAA
AGGGCTGGAATACGTTGGTTTTTTTTATAATGATAGAAGTGCGTACTACAACCCAGCCCTGAAGTCCCGAGCCAGCA
TCACCAAGGACACCTCAAAGAGCCAAGTTTATCTGACGCTGAACAGCCTGACAAGCGAGGACACGGCCGTCTATTAC
TGTGCAGGTCCCGACACGATTACGAATCGGTTGCGAGATGGTATAAACTACTGGGGCCAGGGCATCCTGGTCACCGT
CTCCTCAGAGAGTACGAAGACCCCAGATCTCTTCCCCCTCGTCTCCTGTGGGCCCTCTCTTGATGAGAGCCTGGTGG
CTGTGGGCTGCCTAGCCCGGGACTTCCTACCCAA
>KY437366.1 Equus caballus clone 210 immunoglobulin mu heavy chain (IGHM) mRNA, partial cds
CTCTCCCTCACCTGCACTGTCTCTGGATTATCTTTGAGCAGTAATGCTGTGGGCTGGGTCCGCCAGGCTCCAGGAAA
AGGGCTGGAATACGTTGGTGCTATATATGGTAGTGGTAGTGCAAACTACAACCCAGCCCTGAAGTCCCGAGCCAGCA
TCACCAAGGACACCTCAAAGAGCCAAGTTTATCTGACGCTGAACAGCCTGACAAGCGAGGACACGGCCGTCTACCAC
TGTGCAGGTGTACAGGGTAGTGTCTACACTACTCTCTATGAGGGCTACTGGGGCCAGGGCACCCTGGTCACCGTCTC
CTCAGAGAGTACGAAGACCCCAGATCTCTTCCCCCTCGTCTCCTGTGGGCCCTCTCTTGATGAGAGCCTGGTGGCTG
TGGGCTGCCTAGCCCGGGACTTCCTACCCAA
>KY437365.1 Equus caballus clone 209 immunoglobulin mu heavy chain (IGHM) mRNA, partial cds
CTCTCCCTCACCTGCACTGTCTCTGGATTATCTTTGAGCAGTAATGCTGTAGGCTGGGTCCGCCAGGCTCCAGGAAA
AGGGCTGGAATATGTTGGTGGTATAACTAGTAGTGGAAGTGAAAACTACAACCCAGCCCTGAAGTCCCGAGCCAGCA
TCACCAAGGACACCTCAAAGAGCCAAGTTTATCTGACGCTGAACAGCCTGACAAGCGAGGACACGGCCGTCTATTAC
TGTGCAGGAGGCCGGTCTATGATTATGGTGGTGCTGTTTACAGGGGGTATAAACTACTGGGGCCAGGGCATCCTGGT
CACCGTCTCCTCAGAGAGTACGATGACCCCAGATCTCTTCCCCCTCGTCTCCTGTGGGCCCTCTCTTGATGAGAGCC
TGGTGGCTGTGGGTTGCCTAGCCCGGGACTTCCTACCCAA
>KY437364.1 Equus caballus clone 208 immunoglobulin mu heavy chain (IGHM) mRNA, partial cds
CTCTCCCTCACCTGCACTGTCTCTGGATTATCTTTGAGGCGTAATGCTGTAGGCTGGGTCCGCCAGGCTCCAGGAAA
AGGACTGGAATATGTTGGTCATATATTTAGTAGTGGAAGTACAAACTACAACCCAGCCCTGAAGTCCCGAGCCAGCA
TCACCAAGGACACCTCAAAGAGCCAAGTTTATCTGACACTGAACAGCCTGACAGGCGAAGACACGGCCGTCTATTAC
TGTGCAGTATGCACTAAAAATGGTGCTACTTACTACTTCGGTATAGCCTACTGGGGCCAGGGCATCCTGGTCACCGT
CTCCTCAGAGAGTACGATGACCCCAGATCTCTTCCCCCTCGTCTCCTGTGGGCCCTCTCTTGATGAGAGCCTGGTGG
CTGTGGGCTGCCTAGCCCGGGACTTCCTACCCAA
>KY437363.1 Equus caballus clone 207 immunoglobulin mu heavy chain (IGHM) mRNA, partial cds
CTCTCCCTCACCTGCACTGTCTCTGGATTATCTTTGAGCAGTAATGCTGTAGGCTGGGTCCGCCAGGCTCCAGGAAA
AGGGCTGGAATATGTTGGTGGTATCGCGGCTAGTGGGAGTGCAAACTACAACCCAGCCCTGAAGTCCCGAGCCAGCA
TCACCAAGGACACCTCAAAGAGCCAAGTTTATCTGACGCTGAACAGCCTGACAGGCGAAGACACGGCCGTCTATTAC
TGTGCAGGTTATGGTGTTGGTGAAACTCACTGGGGCCAGGGCATCCTGGTCACCGTCTCCTCAGAGAGTACGATGAC
CCCAGATCTCTTCCCCCTCGTCTCCTGTGGGCCCTCTCTTGATGAGAGCCTGGTGGCTGTGGGCTGCCTAGCCCGGG
ACTTCCTACCCAA
>KY437362.1 Equus caballus clone 206 immunoglobulin mu heavy chain (IGHM) mRNA, partial cds
CTCTCCCTCACCTGCACTGTCTCTGGATTATCTTTGAGCAGTAATACTGTAGGCTGGGTCCGCCAGGCTCCAGGAAA
AGGGCTGGAATATGTTGGTGGTATCACTAATAGTGGAAGTACAGTAGACAACCCAGTCCCGAAGTCCCGAGCCAGCA
TCACCAAGGACACCTCAAAGAGCCAAGTTTATCTGACGCTGAACAGCCTGACAAGCGAGGACACGGCCGTCTATTAC
TGTAGAGGAGGTAGTTACTGGGGCCAGGGCATCCTGGTCACCGTCTCCTCAGAGAGTACGATGACCCCAGATCTCTT
CCCCCTCGTCTCCTGTGGGCCCTCTCTTGATGAGAGCCTGGTGGCTGTGGGCTGCCTAGCCCGGGACTTCCTACCCA
A
>KY437361.1 Equus caballus clone 205 immunoglobulin mu heavy chain (IGHM) mRNA, partial cds
CTCTCCCTCACCTGCACTGTCTCTGGATTATCTTTGAGCAGTAATACTGTAGGCTGGGTCCGCCAGGCTCCAGGAAA
AGGGCTGGAATATGTTGGTGGTATCACTAATAGTGGAAGTACAGTAGACAACCCAGTCCCGAAGTCCCGAGCCAGCA
TCACCAAGGACACCTCAAAGAGCCAAGTTTATCTGACGCTGAACAGCCTGACAAGCGAGGACACGGCCGTCTATTAC

TGTAGAGGAGGTAGTTACTGGGGCCAGGGCATCCTGGTCACCGTCTCCTCAGAGAGTACGATGACCCCAGATCTCTT
CCCCCTCGTCTCCTGTGGGCCCTCTCTTGATGAGAGCCTGGTGGCTGTGGGCTGCCTAGCCCGGGACTTCCTACCCA
A
>KY437360.1 Equus caballus clone 204 immunoglobulin mu heavy chain (IGHM) mRNA, partial cds
CTCTCCCTCACCTGCACTGTCTCTGGATTATCTTTGAGCAGTAATACTGTAGGCTGGGTCCGCCAGGCTCCAGGAAA
AGGGCTGGAATATGTTGGTAGAATATTTAGTGGAGGAAGTGCAGCCTACAACCCAGACCTGAAGCCCCGAGCCAGCA
TCACCAAGGACACCTCAAAGAGCCAAGTTTATCTGACGCTGAACAGCCTGACAGGCGAGGACACGGCCGTCTATTAC
TGTGCAGGATACTTTGCCCCACTAGCTCGCTTTACGGGTGACTTTGTCTACTGGGGCCAGGGCACCCTGGTCACCGT
CTCCTCAGAGAGTACGATGACCCCAGATCTCTTCCCCCTCGTCTCCTGTGGGCCCTCTCTTGATGAGAGCCTGGTGG
CTGTGGGCTGCCTAGCCCGGGACTTCCTACCCAA
>KY437359.1 Equus caballus clone 203 immunoglobulin mu heavy chain (IGHM) mRNA, partial cds
CTCTCCCTCACCTGCACTGTCTCTGGACTCTCTTTGAGCAGTAATTATGTGGCCTGGGTCCGCCAGGCTCCAGGAAA
AGGGCTGGAATATGTTGGTGGTATAAGCAGTAGTGGAAGTGCATACTACAACCCAGCCCTGAAGTCCCGAGCCAGCA
TCACCAAGGACACCTCAAAGAGCCAAGTCGATCTGACGCTGAACAGCCTGACAGGCGAAGACACGGCCGTCTATTAC
TGTGCAGGGGTCGACGGTGATGATTATGATTATTATGGTATAAAGTACTGGGGCCAGGGCATCCTGGTCACCGTCTC
CTCAGAGAGTACGAAGACCCCAGATCTCTTCCCCCTCGTCTCCTGTGGGCCCTCTCTTGATGAGAGCCTGGTGGCTG
TGGGCTGCCTAGCCCGGGACTTCCTACCCAA
>KY437358.1 Equus caballus clone 202 immunoglobulin mu heavy chain (IGHM) mRNA, partial cds
CTGTCCCTCACCTGCACTGTCTCTGGATTATCTTTGAGCGGTAACGCTGTAAACTGGGTCCGCCAGGCTCCAGGAAA
AGGGCTGGAGTGGGTTGCCATGATACGTGGTAGTGGAAATACATACTACAACCCAGCCCTGAAGTCCCGAGCCAGCA
TCACCAAGGACACCTCAAAGAGCCAAATTTATCTGACGCTGAACAGCCTGACAAGCGAGGACACGGCCGTCTATTAC
TGTACAGGAGGCTTTGGACTTTCCTATTATGGTGATTACTTATATAAGACCGCAAGCTACTGGGGCCAGGGCACCCT
GGTCACCGTCTCCTCAGAGAGTACGAAGACCCCAGATCTCTTCCCCCTCGTCTCCTGTGGGCCCTCTCTTGATGAGA
GCCTGGTGGCTGTGGGCTGCCTAGCCCGGGACTTCCTACCCAA
>KY437357.1 Equus caballus clone 201 immunoglobulin mu heavy chain (IGHM) mRNA, partial cds
CTCTCCCTCACCTGCACTGTCTCTGGATTATCTTTGAGCAGTAATGCTGTAGGCTGGGTCCGCCAGGCTCCAGGAAA
AGGGCTGGAATATGTTGGTGGTATAAGTAGTAGTGGAAGTGCACGGTACAACCCAGCCCTGAAGTCCCGAGCCAGCA
TCACCAAGGACACCTCAAAGAGCCAAGTTTATCTGACGCTGAACAGCCTGACAACCGAGGACACGGCCGTCTATCAC
TGTAGAGGAGGAACGTACTGGGGCCAGGGCACCCTGGTCACCGTCTCCTCAGAGAGTACGATGACCCCAGATCTCTT
CCCCCTCGTCTCCTGTGGGCCCTCTCTTGATGAGAGCCTGGTGGCTGTGGGCTGCCTAGCCCGGGACTTCCTACCCA
A
>KY437356.1 Equus caballus clone 200 immunoglobulin mu heavy chain (IGHM) mRNA, partial cds
CTGTCCCCCACCTGCACTGTCTCTGGATTATCTTTGAGCAGTAATGCTGTAGGCTGGGTCCGCCAGGCTCCAGGAAA
AGGGCTGGAGTGGGTTGGTGTTATATATGGTAGTGAAAGTACATACTACAACCCAGCCCTGAAGTCCCGAGCCAGCA
TCACCAAGGACACCTCAAAGAGCCAAGTTTATCTGACGCTGAACAGCCTGACAGGCAAAGACACGGCCGTCTATTAC
TGTGCAGGATTGGTTTCACGGTTATGGTTATTCGGTATAGACTACTGGGGCCAGGGCATCCTGGTCACCGTCTCCTC
AGAGAGTACGAAGACCCCAGATCTCTTCCCCCTCGTCTCCTGTGGGCCCTCTCTTGATGAGAGCCTGGTGGCTGTGG
GCTGCCTAGCCCGGGACTTCCTACCCAA
>KY437355.1 Equus caballus clone 199 immunoglobulin mu heavy chain (IGHM) mRNA, partial cds
CTCTCCCTCACCTGCACTGTCTCTGGATTATCTTTGAGCAGTAATGCTGTAGGCTGGGTCCGCCAGGCTCCAGGAAA
AGGGCTGGAATATGTTGCTGGCATACCTAGTAGTGGGATGACAAGGTACAACCCAGCCCTGAAGTCCCGAGCCAGCA
TCACCAAGGACACCTCAAAGAGCCAAGTTTATCTGACGCTGAACAGCCTGACAAGCGAGGACACGGCCGTCTATTAC
TGTAGAGGAGGTGAATACTGGGGCCAGGGCACCCTGGTCACCGTCTCCTCAGAGAGTACGATGACCCCAGATCTCTT
CCCCCTCGTCTCCTGTGGGCCCTCTCTTGATGAGAGCCTGGTGGCTGTGGGCTGCCTAGCCCGGGACTTCCTACCCA
A
>KY437354.1 Equus caballus clone 198 immunoglobulin mu heavy chain (IGHM) mRNA, partial cds
CTCTCCCTCACCTGCACTGTCTCTGGATTATCTTTGAGCAGTAATTATGTAGGCTGGGTCCGCCAGGCTCCAGGAAA
AGGGCTGGAATATGTTGGTGCTATATATGGTAGTGCAAGTGCAAACTACAACCCAGCCCTGAAGTCCCGAGCCAGCA
TCACCAAGGACACCTCAAAAAGCCAAGTTTATCTGACGCTGAACAGCCTGACAGGCGAGGACACGGCCGTCTATTAC

TGTGCGGGAGGTCCGTACAGTTACTATGGTGGTAGTTCCTGGGCCGACTGGGGCCAGGGCACCCTGGTCACCGTCTC
CTCAGAGAGTACGAAGACCCCAGATCTCTTCCCCCTCGTCTCCTGTGGGCCCTCTCTTGATGAGAGCCTGGTGGCTG
TGGGCTGCCTAGCCCGGGACTTCCTACCCAA
>KY437353.1 Equus caballus clone 197 immunoglobulin mu heavy chain (IGHM) mRNA, partial cds
CTCTCCCTCACCTGCATTGTCTCTGGATTATCTTTGAGCAGTGATGCTGTAGGCTGGGTCCGCCAGGCTCCAGGAAA
AGGGCTGGAATACGTTGGTAAGTTATATGGTAGTGCAAGTCCAAACTACAACCCAGCCCTGAAGTCCCGAGCCAGCA
TCACCAAGGACACCTCAAAGAGCCAAGTTTATCTGACGCTGAACAGCCTGACAAGCGAGGACACGGCCGTCTATTAC
TGTGTAGGAAGCCGGGGACTTGGCTACTGGGGCCAGGGCACCCTGGTCACCGTCTCCTCAGAGAGTACGAAGACCCC
AGATCTCTTCCCCCTCGTCTCCTGTGGGCCCTCTCTTGATGAGAGCCTGGTGGCTGTGGGCTGCCTAGCCCGGGACT
TCCTACCCAA
>KY437352.1 Equus caballus clone 196 immunoglobulin mu heavy chain (IGHM) mRNA, partial cds
CTGTCCCTCACCTGCACTGTCTCTGGATTATCTTTGAGCAGTGATGCTGTAGGCTGGGTCCGCCAGGCTCCAGGAAA
AGGGCTGGAGTGGGTTGGTGTTATATATGGTAGTGAAAGTACATACTACAACCCAGCCCTGAAGTCCCGAGCCAGCA
TCACCAAGGACACCTCAAAGAGCCAAGTTTATCTGACGCTGAACAGCCTGACAGGCGAGGACACGGCCGTCTATTAC
TGTGCAGGGGCAGTTTATGGTAGTTACTATGCCCCAAATTATTATGGAGAAACGTACTGGGGCCAGGGCATCCTGGT
CACCGTCTCCTCAGAGAGTACGATGACCCCAGATCTCTTCCCCCTCGTCTCCTGTGGGCCCTCTCTTGATGAGAGCC
TGGTGGCTGTGGGCTGCCTAGCCCGGGACTTCCTACCCAA
>KY437351.1 Equus caballus clone 195 immunoglobulin mu heavy chain (IGHM) mRNA, partial cds
CTCTCCCTCACCTGCACTGTCTCTGGATTATCTTTGAGCAGTAATTTTGTAGGCTGGGTCCGCCAGGCTCCAGGAAA
AGGGCTGGAATATGTTGGTGGTATAGGTAAAAGTGGAAGTGCATACTACAACCCAGCCCTGAAGTCCCGAGCCAGCA
TCACCAAGGACACCTCAAAGAGCCAAGTTTATCTGACGCTGAACAGCCTGACAAGCGAGGACACGGCCGTCTATTAC
TGTGCAGGACTTTTGTCGTTTGGCTACTGGGGCCAGGGCACCCTGGTCACCGTCTCCTCAGAGAGTACGATGACCCC
AGATCTCTTCCCCCTCGTCTCCTGTGGGCCCTCTCTTGATGGGAGCCTGGTGGCTGTGGGCTGCCTAGCCCGGGACT
TCCTACCCAA
>KY437350.1 Equus caballus clone 194 immunoglobulin mu heavy chain (IGHM) mRNA, partial cds
CTCCCCCTCACCTGCACTGTCTCTGGATTATCTTTGAGCAGTAATGGTGTAGCTTGGGTCCGCCAGGCTCCAGGAAA
AGGGCTGGAATATGTTGGTAATATACGTAGTAGTGGAAGTGTAAACTACAACCCAGCCCTGAAGTCCCGAGTCAGCA
TCACCAAGGACATCTCAAAGAGCCAAGTTTATCTGACGCTGAACAGCCTGACAAGCGAGGACACGGCCGTCTATTAC
TGCCTAAGTGGGGGATTTGGCTACTGGGGCCAGGGCACCCTGGTCACCGTCTCCTCAGAGAGTACGATGACCCCAGA
TCTCTTCCCCCTCGTCTCCTGTGGGCCCTCTCTTGATGAGAGCCTGGTGGCTGTGGGCTGCCTAGCCCGGGACTTCC
TACCCAA
>KY437349.1 Equus caballus clone 193 immunoglobulin mu heavy chain (IGHM) mRNA, partial cds
CTCTCCCTCACCTGCACTGTCTCTGGATTATCTTGGAGCAGTAGTGGTGTGGGCTGGGTCCGCCAGGCTCCAGGAAA
AGGGCTGGAATATGTTGGTGGTATACCTAAGAGTGGCAGTGCAATGTACAACCCAGCCCTGAAGTCCCGAGCCAGCA
TCACCAAGGACACCTCAAAGAGCCAAGTTTATCTGACGCTGAACAGCCTGACAAGCGAGGACACGGCCGTCTATTAC
TGTAGAGGATTCACGTACTGGGGCCAGGGCACCCTGGTCACCGTCTCCTCAGAGAGTACGATGACCCCAGATCTCTT
CCCCCTCGTCTCCTGTGGGCCCTCTCTTGATGAGAGCCTGGTGGCTGTGGGCTGCCTAGCCCGGGACTTCCTACCCA
A
>KY437348.1 Equus caballus clone 192 immunoglobulin mu heavy chain (IGHM) mRNA, partial cds
CTGTCCCTCACCTGCACTGTCTCTGGATTATCTTTGAGCAGTAATGCTGTAGGCTGGGTCCGCCAGGCTCCAGGAAA
AGGGCTGGAGTGGGTTGGTGTTATATATGGTAGTGAAAGTACATACTACAATCCAGCCCTGAAGTCCCGAGCCAGCA
TCACCAAGGACACCTCAAAGAGCCAAGTTTATCTGACGCTGAACAGCCTGACAGGCGAAGACACGGCCGTCTATTAC
TGTGTAGGAGCCGTCTACTGGGGCCAGGGCATCCTGGTCACCGTCTCCTCAGAGAGTACGATGACCCCAGATCTCTT
CCCCCTCGTCTCCTGTGGGCCCTCTCTTGATGAGAGCCTGGTGGCTGTGGGCTGCCTAGCCCGGGACTTCCTACCCA
A
>KY437347.1 Equus caballus clone 191 immunoglobulin mu heavy chain (IGHM) mRNA, partial cds
CCCTCCCTCACCTGCACTGTCTCTGGATTATCTTTGAGCAGTAATGCTGTAGGCTGGGTCCGCCAGGCTCCAGGAAA
AGGGCTGGAATATGTTGGTGGTATAGCTAGTAGTGGAAGTGCAAACTACAACCCAGCCCTGAAGTCCCGAGCCAGCA

TCACCAAGGACACCTCAAAGAGCCAAGTTTATCTGACGCTGAACAGCCTGACAAGCGAGGACACGGCCGTCTATTAC
TGTGCAGGAGGCTCAATTTACTATGCTAGTAGATTTTACTATTATGATGAAGACTACTGGGGCCAGGGCATCCTGGT
CACCGTCTCCTCAGAGAGTACGAAGACCCCAGATCTCTTCCCCCTCGTCTCCTGTGGGCCCTCTCTTGATGAGAGCC
TGGTGGCTGTGGGCTGCCTAGCCCGGGACTTCCTACCCAA
>KY437346.1 Equus caballus clone 190 immunoglobulin mu heavy chain (IGHM) mRNA, partial cds
CTCTCCCTCACCTGCACTGTCTCTGGATTATCTTTGAGCAGTAATGCTGTAGGCTGGGTCCGCCAGGCTCCAGGAAA
AGGGCTGGAAAATGTTGGTGCGTTAGCTAGTAGTGGAAGTGCAAACTACAACCCAGCCCTGAAGTCCCGAGCCAGCA
TCACCAAGGACACCTCAAAGAGCCAAGTTTATCTGACGCTGAACAGCCTGACAAGCGAGGACACGGCCGTCTATTAC
TGTGCAGGGTATAATTATTATAGTACCATTAGCTACTGGGGCCAGGGCACCCTGGTCACCGTCTCCTCAGAGAGTAC
GAAGACCCCAGATCTCTTCCCCCTCGTCTCCTGTGGGCCCTCTCTTGATGAGAGCCTGGTGGCTGTGGGCTGCCTAG
CCCGGGACTTCCTACCCAA
>KY437345.1 Equus caballus clone 189 immunoglobulin mu heavy chain (IGHM) mRNA, partial cds
CTCTCCCTCACCTGCACTGTCTCTGGATTATCTTTGAGCAGTAATGCTGTAGGCTGGGTCCGCCAGGCTCCAGGAAA
AGGGCTGGAATATGTTGGTACTATATATGTCGGTGCAAGTGCAAACTACAACCCAGCCCTGAAGTCCCGAGCCAGCA
TCACCAAGGACACCTCAAAGAGCCAAGTTTATCTGACGCTGAACAGCCTGACAAGCGAGGACACGGCCGTCTATTAC
TGTGCAGGAGGCCTACTGATGGTAACTCCTGATACCGTGGACTGGGGCCAGGGCATCCTGGTCACCGTCTCCTCAGA
GAGTACGAAGACCCCAGATCTCTTCCCCCTCGTCTCCTGTGGGCCCTCTCTTGATGAGAGCCTGGTGGCTGTGGGCT
GCCTAGCCCGGGACTTCCTACCCAA
>KY437344.1 Equus caballus clone 188 immunoglobulin mu heavy chain (IGHM) mRNA, partial cds
CTCTCCCTCACCTGCACTGTCTCTGGATTATCTTTGAGCAGTAATACTGTAGGCTGGGTCCGCCAGGCTCCAGGAAA
AGGGCTGGAATATGTTGGTGTTATAGTTAGTAGTGGAAGTGTAGAATACAACCCAGCCCTGAAGTCCCGAGCCAGCA
TCACCAAGGACGCCTCAAAGAGCCAAGTTTATCTGACGCTGAACAGCCTGACAAGCGAGGACACGGCCGTCTATTAC
TGTGCAGGAGGCCTCATTATAGACGGAGTTTGGAAAACTAGATTTGGCTACTGGGGCCAGGGCACCCTGGTCACCGT
CTCCTCAGAGAGTACGATGACCCCAGATCTCTTCCCCCTCGTCTCCTGTGGGCCCTCCCTTGATGAGAGCCTGGTGG
CTGTGGGCTGCCTAGCCCGGGACTTCCTACCCAA
>KY437343.1 Equus caballus clone 187 immunoglobulin mu heavy chain (IGHM) mRNA, partial cds
CTCTCCCTCACCTGCACTGTCTCTGGATTATCTTTGAACAGTAATAATGTAGGCTGGGTCCGCCAGGCTCCAGGAAA
AGGGCTGCAATACGTGGGTTCAATACATGGCGATGGAAGTATAAACTACAACCCAGCCCTGAAGTCCCGAGCCAGCA
TCACCAAGGACACCTCAAAGAGCCAAGTTTATCTGACGCTGAACAGCCTGACAAGCGAGGACACGGCCGTCTATTAC
TGTGCAAGAATCACCAACTCGGAGGCCACCTACTGGGGCCAGGGCACCCTGGTCACCGTCTCCTCAGAGAGTACGAA
GACCCCAGATCTCTTCCCCCTCGTCTCCTGTGGGCCCTCTCTTGATGAGAGCCTGGTGGCTGTGGGCTGCCTAGCCC
GGGACTTCCTACCCAA
>KY437342.1 Equus caballus clone 186 immunoglobulin mu heavy chain (IGHM) mRNA, partial cds
CTCTCCCTCACCTGCACTGTCTCTGGATTATCTTTGAGCAGGTATACTGTAGGCTGGGTCCGCCAGGCTCCAGGAAA
AGGGCTGGAGTGGGTTGGCTATATATATAACAATGGTGCTACACACTACAACCCAGCCCTGAAGTCCCGAGCCAGCA
TCACCAAGGACACCTCAAAGAGCCAAGTTTATCTGACGCTGAACAGCCTGACAGGCGAAGACACGGCCGTCTATTAC
TGTGCAGGAAACGAGTTGGATTACTGGGGCCAGGGCACCCTGGTCACCGTCTCCTCAGAGAGTACGATGACCCCAGA
TCTCTTCCCCCTCGTCTCCTGTGGGCCCTCTCTTGATGAGAGCCTGGTGGCTGTGGGCTGCCTAGCCCGGGACTTCC
TACCCAA
>KY437341.1 Equus caballus clone 185 immunoglobulin mu heavy chain (IGHM) mRNA, partial cds
CTCTCCCTCATCTGCACTGTCTCTGGATTAGCTTTGAGCAGTGACGGTGTAGGCTGGGTCCGCCAGGCTCCAGGAAA
AGGGCTGGAATTTGTTGGTGGTATAGCTAGTAGTGGAAGTGCAAACTACAACCCAGCCCTGAAGTCCCGAGCCAGCA
TCACCAAGGACACCTCAAAGAGTCAAGTTTATCCGACGCTGAACAGCCTGACAGGCGAGGACACGGCCGTCTATTAC
TGTAGAGGGGGGCGGCTCATGGGGCCACGGCACCCTGGTCACCGTCTCCTCAGAGAGTACGAAGACCCCAGATCTCTT
CCCCCTCGTCTCCTGTGGGCCCTCTCTTGATGAGAGCCTGGTGGCTGTGGGCTGCCTAGCCCGGGACTTCCTACCCA
A
>KY437340.1 Equus caballus clone 184 immunoglobulin mu heavy chain (IGHM) mRNA, partial cds
CTCTCCCTCACCTGCACTGTCTCTGGATTATCTTTGAGCACGAGTGCTGTAGGCTGGGTCCGCCAGGCTCCAGGAAA
AGGGCTGGAATACGTTGGTGCCCTATCAGTCTACAACCCAGCCCTGAAGTCCCGAGCCAGCATCACCAAGGACACCT

CAAAGAGCCAAGTTTATCTGACGCTGAACAGCCTGACAAGCGAGGACACGGCCGTCTATTACTGTGCAGGATGGAAC
AGTATAACACCGCGGTTTGGTAACTATTTTACAAGCTGGGGCCAGGGCACCCTGGTCACCGTCTCCTCAGAGAGTAC
GAAGACCCCAGATCTCTTCCCCCTCGTCTCCTGTGGGCCCTCTCTTGATGAGAGCCTGGTGGCTGTGGGCTGCCTAG
CCCGGGACTTCCTACCCAA
>KY437339.1 Equus caballus clone 183 immunoglobulin mu heavy chain (IGHM) mRNA, partial cds
CTGTCCCTCACCTGCACTGTCTCTGGATTATCTTTGAGCAGTGCTGCTGTAGGCTGGGTCCGCCAGGCTCCAGGAAA
AGGGCTGGAATTTGTTGGTGCTATATCTGGTAGTGGAAGTGCAAACTACAACCCAGCCCTGAAGTCCCGAGCCAGCA
TCACCAAGGACACCTCAAAGAGCCAAGTTTATCTGACGCTGAACAGCCTGACAGGCGAGGACACGGCCGTCTATTAC
TGTGCGAGATCTTATGGTTATGGTAGTACTTACTACGGGTATTATGATATAGACTACTGGGGCCAGGGCACCCTGGT
CACCGTCTCCTCAGAGAGTACGATGACCCCAGATCTCTTCCCCCTCGTCTCCTGTGGGCCCTCTCTTGATGAGAGCC
TGGTGGCTGTGGGCTGCCTAGCCCGGGACTTCCTACCCAA
>KY437338.1 Equus caballus clone 182 immunoglobulin mu heavy chain (IGHM) mRNA, partial cds
CTGTCCCTCACCTGCACTGTCTCTGGATTATCTTTGAGCAGTAATAGTGTAGGCTGGGTCCGCCAGGCTCCAGGAAA
AGGGCTGGAGTGGGTTGGTGTTATACATGGTAGTGAAAGTACATACTACAACCCAGCCCTGAAGTCCCGAGCCAGCA
TCACCAAGGACACCTCAAAGAGCCAAGTTTATCTGACGCTGAACAGCCTGACAGGCGAAGACACGGCCGTCTATTAC
TGTTTAGGATATGGCTACTGGGGCCAGGGCACCCTGGTCACCGTCTCCTCAGAGAGTACGATGACCCCAGATCTCTT
CCCCCTCGTCTCCTGTGGGCCCTCTCTTGATGAGAGCCTGGTGGCTGTGGGCTGCCTAGCCCGGGACTTCCTACCCA
A
>KY437337.1 Equus caballus clone 181 immunoglobulin mu heavy chain (IGHM) mRNA, partial cds
CTCTCCCTCACCTGCACTGTCTCTGGATTATCTTTGAGCAGTGTTTCTGTAGGCTGGGTCCGCCAGGCTCCAGGAAA
AGGGCTGGAAAGTGTTGGTGGTATACGTAGTGATGGAAGTGCACACTACAACCCAGCCCTGAAGTCCCGAGCCAGCA
TCACCAAGGACACCTCAAAGAGCCAAGTTTATCTGACGCTGAACAGCCTGACAAGCGAGGACACGGCCGTCTATTGG
TGTAGAGGAGACGGCTATTTCTACTGGGGCCAGGGCACCCTGGTCACCGTCTCCTCAGAGAGTACGATGACCCCAGA
TCTCTTCCCCCTCGTCTCCTGTGGGCCCTCTCTTGATGAGAGCCTGGTGGCTGTGGGCTGCCTAGCCCGGGACTTCC
TACCCAA
>KY437336.1 Equus caballus clone 180 immunoglobulin mu heavy chain (IGHM) mRNA, partial cds
CTCTCCCTCACCTGCACTGCCTCTGGATTATCTTTGAGCAGTAATGCTGTAGGCTGGGTCCGCCAGGCTCCAGGAAA
AGGGCTGGAATATGTCGGTGCTATAGCTAGTAGTGGAAGTGCCTACTACAACCCAGCCCTGAAGTCCCGAGCCAGCA
TCACCAAGGACACCTCAAAGAGCCAAGTTTATCTGACGCTGAACAGCCTGACAAGCGAGGACACGGCCGTCTATTAC
TGTGCAGGAGAGGTTCTAGGGGACTCGAGTCGTATGCCGGATACTATGGACCCCTGGGGCCAGGGCACCCTGGTCAC
CGTCTCCTCAGAGAGTACGATGACCCCAGATCTCTTCCCCCTCGTCTCCTGTGGGCCCTCTCTTGATGAGAGCCTGG
TGGCTGTGGGCTGCCTAGCCCGGGACTTCCTACCCAA
>KY437335.1 Equus caballus clone 179 immunoglobulin mu heavy chain (IGHM) mRNA, partial cds
CTGTCCCTCACCTGCACTGTCTCTGGATTCTCTTTGAGCAGTGCCGCTGTAGGCTGGGTCCGCCAGGCTCCAGGAAA
AGGGCTGGAATATGTTGCTGGATTCTCTACTAGTGGAAGTGCAATGTACAACCCAGCCCTGAAGTCCCGAGCCAGCA
TCACCAAGGACACCTCAAAGAGCCAAGTTTATCTGACGCTGAACAGCCTGACAAGCGAGGACACGGCCGTCTATTAC
TGTGCAGGATGTCGCGGGGGCGGTTATGGTGTTGCTTACTACTTTTATGGTGAGAGCTACTGGGGCCAGGGCATCCT
GGTCACCGTCTCCTCAGAGAGTACGATGACCCCAGATCTCTTCCCCCTCGTCTCCTGTGGGCCCTCTCTTGATGAGA
GCCTGGTGGCTGTGGGCTGCCTAGCCCGGGACTTCCTACCCAA
>KY437334.1 Equus caballus clone 178 immunoglobulin mu heavy chain (IGHM) mRNA, partial cds
CTCTCCCTCACCTGCACTGTCTCTGGATTATCTTTGAGCAGTAATAATGTAGGCTGGGTCCGCCAGGCTCCAGGAAA
AGGGCTGGAATATGTTGGTAATATACATAGCAGTGGAAGTGCAAACTACAACCCAGCCCTGAAGTCCCGAGCCAGCA
TCACCAAGGACACCTCAAAGAGCCAAGTTTATCTGACGCTGAACAGCCTGACAAGCGAGGACACGGCCGTCTATTAC
TGTGCAGGAGGTACTGGTTATGGGTACTGGGGCCAGGGCACCCTGGTCACCGTCTCCTCAGAGAGTACGATGACCCC
AGATCTCTTCCCCCTCGTCTCCTGTGGGCCCTCTCTTGATGAGAGCCTGGTGGCTGTGGGCTGCCTAGCCCGGGACT
TCCTACCCAA
>KY437333.1 Equus caballus clone 177 immunoglobulin mu heavy chain (IGHM) mRNA, partial cds

CTGTCCCTCACCTGCACTGTCTCTGGATTCTCTTTGAGCAGTTACGGTGTAGGCTGGGTCCGCCAGGCTCCAGGAAA
AGGGCTGGAATATGTTGGTGGTATAGCTAGTAGTGGAAGTGCAAACTACAACCCAGCCCTGAAGTCCCGAGCCAGCA
TCACCAAGGACACCTCAAAGAGCCAAGTCACTCTGACGCTGAACAGCCTGACAGGCGAGGACACGGCCGTCTATTAC
TGTGCGAGATTGGGGTACAACTACGGTTATGGTTATGCTACTTTTGTTGATTTTATGGACCCCTGGGGCCAGGGCAC
CCTGGTCACCGTCTCCTCAGAGAGTACGATGACCCCAGATCTCTTCCCCCTCGTCTCCTGTGGGCCCTCTCTTGATG
AGAGCCTGGTGGCTGTGGGCTGCCTAGCCCGGGACTTCCTACCCAA
>KY437332.1 Equus caballus clone 176 immunoglobulin mu heavy chain (IGHM)
mRNA, partial cds
CTGTCCCTCACCTGCACTGTCTCTGGATTCTCTTTGAGCAGTTACGGTGTAGGCTGGGTCCGCCAGGCTCCAGGAAA
AGGGCTGGAATATGTTGGTGGTATTCTTAAGAGTGGAAGTGCAGTCTACAACCCAGCCCTGAAGTCCCGAGCCAGCA
TCACCAAGGACACCTCAAAGAGCCAAGTTTATCTGACGCTGAACAGCCTGACAGGCGAGGACACGGCCGTCTATTAC
TGTGCGAGACTCGACTACTGGGGCCAGGGCACCCTGGTCACCGTCTCCTCAGAGAGTACGATGACCCCAGATCTCTT
CCCCCTCGTCTCCTGTGGGCCCTCTCTTGATGAGAGCCTGGTGGCTGTGGGCTGCCTAGCCCGGGACTTCCTACCCA
A
>KY437331.1 Equus caballus clone 175 immunoglobulin mu heavy chain (IGHM)
mRNA, partial cds
CTGTCCCTCACCTGCACTGTCTCTGGATTATCTTTGAGCAGTAATGCTGTAGGCTGGGTCCGCCAGGCTCCAGGAAA
AGGGCTGGAATATGTTGGTGGTATAGCTAGTAGTGGAAGTGCAAACTACAACCCAGCCCTGAAGTCCCGAGCCAACA
TCACCAAGGACACCTCAAAGAGCCAAGTTTATCTGACGCTGAACAGCCTGACAAGCGAGGACACGGCCGTCTATTAC
TGTGCAGGAGGCTTGAGAATGTGGAATTATTATGGATTTATTGACTACTATTTTGGCTACTGGGGCCAGGGCACCCT
GGTCACCGTCTCCTCAGAGAGTACGATGACCCCAGATCTCTTCCCCCTCGTCTCCTGTGGGCCCTCTCTTGATGAGA
GCCTGGTGGCTGTGGGCTGCCTAGCCCGGGACTTCCTACCCAA
>KY437330.1 Equus caballus clone 174 immunoglobulin mu heavy chain (IGHM)
mRNA, partial cds
CTGTCCCTCACCTGCACTGTCTCTGGATTCTCTTTGAGCAGTTACGGTGTAGGCTGGGTCCGCCAGGCTCCAGGAAA
AGGGCTGGAATATGTTGGTGGTATAGCTAGTAGTGGAAGTGCAAACTACAACCCAGCCCTGAAGTCCCGAGCCAGCA
TCACCAAGGACACCTCAAAGAGCCAAGTTTATCTGACGCTGAACAGCCTGACAAGCGAGGACACGGCCGTCTATTAC
TGTGCAGGTTATAGCAGCACCACTTATGACTACTGGGGCCAGGGCACCCTGGTCACCGTCTCCTCAGAGAGTACGAT
GACCCCAGATCTCTTCCCCCTCGTCTCCTGTGGGCCCTCTCTTGATGAGAGCCTGGTGGCTGTGGGCTGCCTAGCCC
GGGACTTCCTACCCAA
>KY437329.1 Equus caballus clone 173 immunoglobulin mu heavy chain (IGHM)
mRNA, partial cds
CTCTCCCTCACCTGCACTGTCTCTGGATTATCTTTGAGCAGTTATGCTGTAGGCTGGGTCCGCCAGGCTCCAGGAAA
AGGGCTGGAATATGTTGGTGCTATATATGGTGTTGCAAGTGCAAACTACAACCCAGCCCTGAAGTCCCGAGCCAGCA
TCACCAAGGACACCTCAAAGAGCCAAGTTTATCTGACGCTGAACAGCCTGACAGGCGAGGACACGGCCGTCTATTAC
TGTGCGAGATGGCGCTATGGTTATGGTGGTACTTACTACCTTTATGATATAGACTACTGGGGCCAGGGCACCCTGGT
CACCGTCTCCTCAGAGAGTACGATGACCCCAGATCTCTTCCCCCTCGTCTCCTGTGGGCCCTCTCTTGATGAGAGCC
TGGTGGCTGTGGGCTGCCTAGCCCGGGACTTCCTACCCAA
>KY437328.1 Equus caballus clone 172 immunoglobulin mu heavy chain (IGHM)
mRNA, partial cds
CTCTCCCTCACCTGCACTGTCTCTGGATCATCTTGGAGCAGTTATGGTGTAGGCTGGGTCCGCCAGGCTCCAGGAAA
AGGGCTGGAATATGTTGGTGCTATATATGGTGTTGCAAGTGCAAACTACAACCCAGCCCTGAAGTCCCGAGCCAGCA
TCACCAAGGACACCTCAAAGAGCCAAGTTTATCTGACGCTGAACAGCCTGACAGGCGAGGACACGGCCGTCTATTAC
TGTGCGAGATGGCGCTATGGTTATGGTGGTACTTACTACCTTTATGATATAGACTACTGGGGCCAGGGCACCCTGGT
CACCGTCTCCTCAGAGAGTACGATGACCCCAGATCTCTTCCCCCTCGTCTCCTGTGGGCCCTCTCTTGATGAGAGCC
TGGTGGCTGTGGGCTGCCTAGCCCGGGACTTCCTACCCAA
>KY437327.1 Equus caballus clone 171 immunoglobulin mu heavy chain (IGHM)
mRNA, partial cds
CTCTCCCTCACTTGCACTGTCTCTGGATTATCTCTGAGCAGTGTTATTGTAGGCTGGGTCCGCCAGGCTCCAGGAAA
AGGGCTGGAACATGTTGGTGGTATAATGAGTAGTGGAGTTGCAGAGTACAACCCAGCCCTGAAGTCCCGAGCCAGCA
TCACCAAGGACACCTCAAAGAGCCAGATTTATCTGACGCTGAACAGCCTGACAAGCGAGGACACGGCCGTCTATTAC
TGTGCAGGAGAGATACCACGGCTCGGGTATTTTGGCTACTGGGGCCAGGGCACCCTGGTCACCGTCTCCTCAGAGAG
TACGATGACCCCAGATCTCTTCCCCCTCGTCTCCTGTGGGCCCTCTCTTGATGAGAGCCTGGTGGCTGTGGGCTGCC
TAGCCCGGGACTTCCTACCCAA
>KY437326.1 Equus caballus clone 170 immunoglobulin mu heavy chain (IGHM)
mRNA, partial cds

CTCTCCCTCACCTGCACTGTCTCTGGATTACCTTTGAGCAGTAATACTGTAGTTTGGGTCCGCCAGGCTCCAGGAAA
AGGGCTGGAATATGTTGGTGGTATTGTTAGTAGTGGAAGTGCAATGTACAACCCAGCCCTGAAGTCCCGAGCCAGCA
TCACCAAGGACACCTCAAAGAGCCAAGTATATCTGACGCTGAACAGCCTGACAAGCGAGGACACGGCCGTCTATTAC
TGTAGAGGAGGTGGTTCCTGGGGCCAGGGCATCCTGGTCACCGTCTCCTCAGAGAGTACGAAGACCCCAGATCTCTT
CCCCCTCGTCTCCTGTGGGCCCTCTCTTGATGAGAGCCTGGTGGCTGTGGGCTGCCTAGCCCGGGACTTCCTACCCA
A
>KY437325.1 Equus caballus clone 169 immunoglobulin mu heavy chain (IGHM) mRNA, partial cds
CTCTCCCTCACCTGCACTGTCTCTGGATTATCTTTGAGCAGTAATGCTGTAGGCTGGGTCCGCCAGGCTCCAGGAAA
AGGGCTGGAATATGTTGGTGGTATAGGGAGTAGTGGAAGTAGAACGTACAACCCAGCCCTGAAGTCCCGAGCCAGCA
TCACCAAGGACACCTCAAAGAGCCAAGTTTATCTGACGCTGAGCAGCCTGACAAGCGAGGACACGGCCGTCTATTAC
TGTGCAGGAGGCAGTTGGGGTGGTGATGAGTACTGGGGTGATATTATAAACTACTGGGGCCAGGGCATCCTGGTCAC
CGTCTCCTCAGAGAGTACGATGACCCCAGATCTCTTCCCCCTCGTCTCCTGTGGGCCCTCTCTTGATGAGAGCCTGG
TGGCTGTGGGCTGCCTAGCCCGGGACTTCCTACCCAA
>KY437324.1 Equus caballus clone 168 immunoglobulin mu heavy chain (IGHM) mRNA, partial cds
CTCTCCCTCACCTGCACTGTCTCTGGATTCTCTTTGAGCACTTATGCTGTAGGCTGGGTCCGCCAGGCTCCAGGAAA
AGGGCTGGAAGCTGTTGGTGATATATTAAATGGTGGAAGTGCATACTACAACCCAGCCCTGAAGTCCCGAGCCAGCA
TCACCAAGGACACCTCCAAGAGCCAAGTTTATCTGACGCTGAACAGCCTGACAGACGAGGACACGGCCGTCTATTAC
TGTGCGATCGTTTATGATTTTGATGGTGACCACTACTACAACTATTTGGGCGTCTGGGGCCAGGGCACCCTGGTCAC
CGTCTCCTCAGAGAGTACGATGACCCCAGATCTCTTCCCCCTCGTCTCCTGTGGGCCCTCTCTTGATGAGAGCCTGG
TGGCTGTGGGCTGCCTAGCCCGGGACTTCCTACCCAA
>KY437323.1 Equus caballus clone 167 immunoglobulin mu heavy chain (IGHM) mRNA, partial cds
CTGTCCCTCACCTGCACTGTCTCTGGATTCTCTTTGAGCAGTGACGGTGTAGGCTGGGTCCGCCAGGCTCCAGGAAA
AGGGCTGGAATTTGTTGGTGGTATAGCTAGTAGTGGAAGTGCAAACTACAACCCAGCCCTGAAGTCCCGAGCCAGCA
TCACCAAGGACACCTCAAAGAGTCAAGTTTATCTGACGCTGAACAGCCTGACAGGCGAGGACACGGCCGTCTATTGG
TGTGCGAGCGCGGATAGCAGCTATTCTTGGTATTCCTATGGTATAACCTACTGGGGCCAGGGCATCCTGGTCACCGT
CTCCTCAGAGAGTACGAAGACCCCAGATCTCTTCCCCCTCGTCTCCTGTGGGCCCTCTCTTGATGAGAGCCTGGTGG
CTGTGGGCTGCCTAGCCCGGGACTTCCTACCCAA
>KY437322.1 Equus caballus clone 166 immunoglobulin mu heavy chain (IGHM) mRNA, partial cds
CTCTCCCTCATCTGCACTGTCTCTGGATTATCTTTGAGCAGTTATGGTGTGGGCTGGGTCCGCCAGGCTCCAGGAAA
AGGGCTGGAATTTGTTGGTGGTATAGCTAGTAGTGGAAGTGCAAACTACAACCCAGCCCTGAAGTCCCGAGCCAGCA
TCACCAAGGACACCTCAAAGAGCCAAGTTTATCTGACGCTGAACAGCCTGACAAGCGAGGACACGGCCGTCTATTAC
TGTGCAGGAGACAATTACTATGGTGGTAGTTCCTGGTACTCCGTCTCTAGCTTTGACTACTGGGGCCAGGGCCCCCT
GGTCACCGTCTCCTCAGAGGGTACGAAGACCCCAGATCTCTTCCCCCTCGTCTCCTGTGGGCCCTCTCTTGATGAGA
GCCTGGTGGCTGTGGGCTGCCTAGCCCGGGACTTCCTACCCAA
>KY437321.1 Equus caballus clone 165 immunoglobulin mu heavy chain (IGHM) mRNA, partial cds
CTCTCCCTCATCTGCACTGTCTCTGGATTATCTTTGAGCAGTTATGGTGTGGGCTGGGTCCGCCAGGCTCCAGGAAA
AGGGCTGGAATTTGTTGGTGGTATAGCTAGTAGTGGAAGTGCAAACTACAACCCAGCCCTGAAGTCCCGAGCCAGCA
TCACCAAGGACACCTCAAAGAGCCAAGTTTATCTGACGCTGAACAGCCTGACAAGCGAGGACACGGCCGTCTATTAC
TGTGCAGGAGACAATTACTATGGTGGTAGTTCCTGGTACTCCGTCTCTAGCTTTGACTACTGGGGCCAGGGCACCCT
GGTCACCGTCTCCTCAGAGAGTACGAAGACCCCAGATCTCTTCCCCCTCGTCTCCTGTGGGCCCTCTCTTGATGAGA
GCCTGGTGGCTGTGGGCTGCCTAGCCCGGGACTTCCTACCCAA
>KY437320.1 Equus caballus clone 164 immunoglobulin mu heavy chain (IGHM) mRNA, partial cds
CTGTCCCTCACCTGCACTGTCTCTGGATTCTCTTTGAGCAGTTACGGTGTAGGCTGGGTCCGCCAGGCTCCAGGAAA
AGGGCTGGAATTTGTTGGTGGAATACGTAGTACTGGAAGTGCAAACTACAACCCAGCCCTGAAGTCCCGAGCCAGCA
TCACCAAGGACACCTCAAAGAGCCAAGTTTATCTGACGCTGAACAGCCTGACAGGCGAGGACACGGCCGTCTATTAC
TGTGCGAGAATAAGTGCCATAGACGACTGGGGCCAGGGCATCCTCGTCCCCGTCTCCTCAGAGAGTACGAAGACCCC
AGATCTCTTCCCCCTCGTCTCCTGTGGGCCCTCTCTTGATGAGAGCCTGGTGGCTGTGGGCTGCCTAGCCCGGGACT
TCCTACCCAA

>KY437319.1 Equus caballus clone 163 immunoglobulin mu heavy chain (IGHM) mRNA, partial cds
CTCTCCCTCACCTGCACTGTCTCTGGATTATCTTTGAGCAGTTATGGTGTGGGCTGGGTCCGCCAGGCTCCAGGAAA
AGGGCTGGAATTTGTTGGCGGTATACGTAGTAGTGGAAGTGCAGACTACAACCCAGCCCTGAAGTCCCGAGCCAGCA
TCACCAAGGACACCTCAAAGAGCCAAGTTTATCTGACGCTGAACAGCCTGACAGGCGAAGACACGGCCGTCTATTAC
TGTCGCGGTTCTGGTTATTGGGGCCAGGGCACCCTGGTCACCGTCTCCTCAGAGAGTACGAAGACCCCAGATCTCTT
CCCCCTCGTCTCCTGTGGGCCCTCTCTTGATGAGAGCCTGGTGGCTGTGGGCTGCCTAGCCCGGGACTTCCTACCCA
A
>KY437318.1 Equus caballus clone 162 immunoglobulin mu heavy chain (IGHM) mRNA, partial cds
CTGTCCCTCACCTGCACTGTCTCTGGATTCTCTTTGACCAGTTACACTGTATATTGGGTCCGCCAGGCTCCAGGAAA
AGGGCTGGAATTTGTTGGTCGTATTTCTAGTAGTGGAGCTGTAAACTACAACCCAGCCCTGAAGTCCCGAGCCAGCA
TCACCAAGGACACCTCAAAGAGCCAAGTTTATCTGACGCTGAACAACCTGACAGGCGAGGACACGGCCGTCTATTAC
TGTGCGAGAAGTGTTTTCGGTAGCAATGCTGCTGGCTACTGGGGCCAGGGCACCCTGGTCACCGTCTCCTCAGAGAG
TACGAAGACCCCAGATCTCTTCCCCCTCGTCTCCTGTGGGCCCTCTCTTGATGAGAGCCTGGTGGCTGTGGGCTGCC
TAGCCCGGGACTTCCTACCCAA
>KY437317.1 Equus caballus clone 161 immunoglobulin mu heavy chain (IGHM) mRNA, partial cds
CTGTCCCTCACCTGCACTGTCTCTGGATTCTCTTTGAGCAGTTTTGGTGTAGGCTGGGTCCGCCAGGCTCCAGGAAA
AGGGCTGGAATTTGTTGGTGGTATAGCTAGTAGTGGAAGTGCAAATTACAACCCAGCCCTGAAGTCCCGAGCCAGCA
TCACCAAGGACACCTCAAAGAGCCAAGTTTATCTGACGCTGATCAGCCTGACAGGCGAGGACACGGCCGTCTATTAC
TGTGCGAGATATTATATTGTGGCAGTGGTTATGGTGCGTTTGGCTACTGGGGCCAGGGCACCCTGGTCACCGTCTC
CTCAGAGAGTACGAAGACCCCAGATCTCTTCCCCCTCGTCTCCTGTGGGCCCTCTCTTGATGAGAGCCTGGTGGCTG
TGGGANGCGTAGCCCGGGACTTCCTACCCAA
>KY437316.1 Equus caballus clone 160 immunoglobulin mu heavy chain (IGHM) mRNA, partial cds
CTGTCCCTCACCTGCACTGTCTCTGGATTCTCTTTGAGCAGTCACGGTGTAGGCTGGGTCCGCCAGGCTCCAGGAAA
AGGGCTGGAATTTGTTGGTGTTATAGCTAGTAGTGGAAGTGCAAACTACAACCCAGCCCTGAAGTCCCGAGCCAGCA
TCACCAAGGACACCTCAAAGAGCCAAGTTTATCTGACGCTGAACAGCCTGACAGGCGAGGACACGGCCGTCTATTAC
TGTGCGACGGTATACGGTTCCTATGCTGGTAGTTACTTTAACTGGGGCCAGGGCACCCTGGTCACCGTCTCCTCAGA
GAGTACNAAGACCCCAGATCTCTTCCCCCTCGTCTCCTGTGGGCCCTCTCTTGATGAGAGCCTGGTGGCTGTGGGCT
GCCTAGCCCGGGACTTCCTACCCAA
>KY437315.1 Equus caballus clone 159 immunoglobulin mu heavy chain (IGHM) mRNA, partial cds
CTCTCCCTCACCTGCACTGTCTCTGGATTTCCTTTGAGCAGTAGTACTGTAAGCTGGGTCCGCCAGGCTCCAGGAAA
AGGGCTGGAATTTGTTGGTGCTATAAGTGGTAGTGGAAGTACAGGGTACAACCCAGCCCTGAAGTCCCGAGCCAGCA
TCACCAAGGACACCTCCAAGAGCCAAGTTTATCTGACGCTGAACAGCCTGACAGGCGAGGACACGGCCGTCTATTAC
TGTGCCTGGCCTCTTATATACTGGGGCCAGGGCACCCTGGTCACCGTCTCCTCAGAGAGTACNAAGACCCCAGATCT
CTTCCCCCTCGTCTCCTGTGGGCCCTCTCTTGATGAGAGCCTGGTGGCTGTGGGCTGCCTAGCCCGGGACTTCCTAC
CCAA
>KY437314.1 Equus caballus clone 158 immunoglobulin mu heavy chain (IGHM) mRNA, partial cds
CTGTCCCTCACCTGCACTGTCTCTGGATTCTCTTTGAGCAGTTACGGTGCAACTTGGGTCCGCCAGGCTCCAGGAAA
AGGGCTGGAATTTGTTGGTTATATTACTAGTAGTGGAAGTGCCGCCTACAACCCAGCCCTGAAGTCCCGAGCCAGCA
TCACCAAGGACACCTCAAAGAGCCAAGTTTATCTGACGCTGAACAGCCTGACAGGCGAGGACACGGCCGTCTATTAC
TGTGTGAGTCAGGCATTGAACAGCTGGGGCCAGGGCATCCTGGTCACCGTCTCCTCAGAGAGTACNAAGACCCCAGA
TCTCTTCCCCCTCGTCTCCTGTGGGCCCTCTCTTGATGAGAGCCTGGTGGCTGTGGGCTGCCTAGCCCGGGACTTCC
TACCCAA
>KY437313.1 Equus caballus clone 157 immunoglobulin mu heavy chain (IGHM) mRNA, partial cds
CTGTCCCTCACCTGCACTGTCTCTGGATTATCTTTGAGCAGTAATACTGTAGGCTGGGTCCGCCAGGCTCCAGGAAA
AGGGCTGGAGTGGGTTGGTGTTATATATGGTAGTGAAAGTACATCCTACAACCCAGCCCTGAAGTCCCGAGCCAGCA
TCACCAAGGACACCTCAAAGAGCCAAGTTTATCTGACGCTGAACAGCCTGACAGGCGAAGACACGGCCGTCTATTAC
TGTGCAGGATGCACTGGTAGTTACTATGTCCCGTATGGTATAGTCTACTGGGGCCAGGGCATCCTGGTCACCGTCTC
CTCAGAGAGTACGAAGACCCCAGATCTCTTCCCCCTCGTCTCCTGTGGGCCCTCTCTTGATGAGAGCCTGGTGGCTG
TGGGCTGCCTAGCCCGGGACTTCCTACCCAA

>KY437312.1 Equus caballus clone 156 immunoglobulin mu heavy chain (IGHM) mRNA, partial cds
CTGTCCCTCACCTGCACTGTCTCTGGATTCTCTTTGAGCAGTTACTATGTAGCCTGGGTCCGCCAGGCTCCAGGAAA
AGGGCTGGAATTTGTTGGTGGTATACCTGCTAGTGGAAGTACAAACTACAACCCAGCCCTGAAGACCCGAGCCAGCA
TCACCAAGGACACCTCAAAGAGCCAAGTTTATCTGACGCTGAACAGCCTGACAGGCGAGGACACGGCCGTCTATTAC
TGTGCGCGATCGTCGTATGGTAACTACGACTGGGGCCAGGGCATCCTGGTCACCGTCTCCTCAGAGAGTACGAAGAC
CCCAGATCTCTTCCCCCTCGTCTCCTGTGGGCCCTCTCTTGATGAGAGCCTGGTGGCTGTGGGCTGCCTAGCCCGGG
ACTTCCTACCCAA
>KY437311.1 Equus caballus clone 155 immunoglobulin mu heavy chain (IGHM) mRNA, partial cds
CTCTCCCTCACCTGCACTGTCTCTGGATTGTCTTTGAGCAGTTATGCTGTATACTGGGTCCGCCAGGCTCCAGGAAA
AGGGCTGGAATATGTTGGTGCTATAGCGCGTAGTGGAAGTGCAAACTACAACCCAGCCCTGAAGTCCCGAGCCAGCA
TCACCAAGGACACCTCCAAGAGCCAAGTTTATCTGACGCTGAACAGCCTGACAGGCGAGGACACGGCCGTCTATTAC
TGTGCGAAAGATGCTGGTTACTATGGTAGTAGTTCCTGGAACTCCGAATATTATTATGGTATAGACTACTGGGGCCA
GGGCATCCTGGTCACCGTCTCCTCAGAGAGTACGAAGACCCCAGATCTCTTCCCCCTCGTCTCCTGTGGGCCCTCTC
TTGATGAGAGCCTGGTGGCTGTGGGCTGCCTAGCCCGGGACTTCCTACCCAA
>KY437310.1 Equus caballus clone 154 immunoglobulin mu heavy chain (IGHM) mRNA, partial cds
CTGTCCCTCACCTGCACTGTCTCTGGATTATCTTTGAGCAGTAATGCTGTGCGCTGGGTCCGCCAGGCTCCAGGAAA
AGGGCTGGAGTGGGTTGCTGTTATATATGGTACTGAAAGTACATACTACAACCCAGCCCTGAAGTCCCGAGCCAGCA
TCACCAAGGACACCTCAAAGAGCCAAGTTTATCTGACGCTGAACAGCCTGACAGGCGAAGACACGGCCGTCTATTAC
TGTGCAGGATGGGGGGCTATTTATGAGCATGCCATGGGCCCCTGGGGCCAGGGCACCCTGGTCACCGTCTCCTCAGA
GAGTACGAAGACCCCAGATCTCTTCCCCCTCGTCTCCTGTGGGCCCTCTCTTGATGAGAGCCTGGTGGCTGTGGGCT
GCCTAGCCCGGGACTTCCTACCCAA
>KY437309.1 Equus caballus clone 153 immunoglobulin mu heavy chain (IGHM) mRNA, partial cds
CTCTCCCTCACCTGCACTGTCTCTGGATTATCTTTGAGCAGTTATGGTGTGGGCTGGGTCCGCCAGGCTCCAGGAAA
AGGGCTGGAATTTGTTGGTGAAATAGCTAGTAGTGGAAGTGCAAACTACAATCCAGCCCTGAAGTCCCGAGCCAGCA
TCACCAAGGACACCTCAAAGAGCCAAGTTTATCTGACGCTGAACAGCCTGACAAGCGAGGACACGGCCGTCTATTAC
TGTGCGAGACCGATGCGAGATTACTATGGTAGTTACTCAAGTAGTTACTATGAGTCGTATTCTGGCTACTGGGGCCA
GGGCACCCTGGTCACCGTCTCCTCAGAGAGTACGAAGACCCCAGATCTCTTCCCCCTCGTCTCCTGTGGGCCCTCTC
TTGATGAGAGCCTGGTGGCTGTGGGCTGCCTAGCCCGGGACTTCCTACCCAA
>KY437308.1 Equus caballus clone 152 immunoglobulin mu heavy chain (IGHM) mRNA, partial cds
CTCTCCCTCACCTGCACTGTCTCTGGATTCTCTTTGAGCAGTTACGGTGTAGGCTGGGTCCGCCAGGCTCCAGGAAA
AGGGCTGGAATTTGTTGGTGGTATAGCTAGTAGTGGAAGTGCAAACTACAACCCAGCCCTGAAGTCCCGAGCCAGCA
TCACCAAGGACACCTCAAAGAGCCAAGTTTATCTGACGCTGAACAGCCTGACAGGCGAGGACACGGCCGTCTATTAC
TGTGTTTCTGGGACCTATGGTTATGGTGGTGCTTTGTCCGAGTACTATTTTGGCTACTGGGGCCAGGGCACCCTGGT
CACCGTCTCCTCAGAGAGTACGAAGACCCCAGATCTCTTCCCCCTCGTCTCCTGTGGGCCCTCTCTTGATGAGAGCC
TGGTGGCTGTGGGCTGCCTAGCCCGGGACTTCCTACCCAA
>KY437307.1 Equus caballus clone 151 immunoglobulin mu heavy chain (IGHM) mRNA, partial cds
CTCTCCCTCACCTGCACTGTCTCTGGATTCTCTTTGAGCAGTTACGGTGTAGGCTGGGTCCGCCAGGCTCCAGGAAA
AGGGCTGGAAATTGTTGGTTATATAGCTAAGAGTGGAATTACAGGCTACAACCCAGCCCTGAAGTCCCGAGCCAGCA
TCACCAAGGACACCTCAAAGAGCCAAGTTTATCTGACGCTGAACAGCCTGACAAGCGAGGACACGGCCGTCTATTAC
TGTGCAGGAGGCCCGGAAGTGTATGGTGATACTTTCTGGGGCCAGGGCACCCTGGTCACCGTCTCCTCAGAGAGTAC
GAAGACCCCAGATCTCTTCCCCCTCGTCTCCTGTGGGCCCTCTCTTGATGAGAGCCTGGTGGCTGTGGGCTGCCTAG
CCCGGGACTTCCTACCCAA
>KY437306.1 Equus caballus clone 150 immunoglobulin mu heavy chain (IGHM) mRNA, partial cds
CTCTCCCTCACCTGCACTGTCTCTGGATTATCTTTGAGCAGAGATGCTGTAGGCTGGGTCCGCCAGGCTCCAGGAAA
AGGGCTGGAATACGTTGGTGCAGTATATGGTAGTGATAGTGCAAACTACAACCCAGCCCTGAAGTCCCGAGCCAGCA
TCACCAAGGACACCTCAAAGAGCCAAGTTTATCTGACGCTGAACAGCCTGACAAGCGAGGACACGGCCGTCTATTAC
TGTGCAGGAGGCTATGGTTATGGTAGTGCTTACTACTATTTAGCTACTGGGGCCAGGGCACCCTGGTCACCGTCTC

CTCAGAGAGTACGAAGACCCCAGATCTCTTCCCCCTCGTCTCCTGTGGGCCCTCTCTTGATGAGAGCCTGGTGGCTG
TGGGCTGCCTAGCCCGGGACTTCCTACCCAA
>KY437305.1 Equus caballus clone 149 immunoglobulin mu heavy chain (IGHM)
mRNA, partial cds
CTCTCCCTCACCTGCACTGTCTCTGGATTATCTTTGAGCAGTTATGGTGTGGGCTGGGTCCGCCAGGCTCCAGGAAA
AGGGCTGGAATTTGTTGGTGAAATAGCTAGTAGTGGAAGTGCAAACTACAATCCAGCCCTGAAGTCCCGAGCCAGCA
TCACCAAGGACACCTCAAAGAGCCAAGTTTATCTGACGCTGAACAGCCTGACAAGCGAGGACACGGCCGTCTATTAC
TGTACAGGAGGCCCTTATGGTGGTGATTACTACTCCTATGTTGGTATAAACTACTGGGGCCAGGGCATCCTGGTCAC
CGTCTCCTCAGAGAGTACGAAGACCCCAGATCTCTTCCCCCTCGTCTCCTGTGGGCCCTCTCTTGATGAGAGCCTGG
TGGCTGTGGGCTGCCTAGCCCGGGACTTCCTACCCAA
>KY437304.1 Equus caballus clone 148 immunoglobulin mu heavy chain (IGHM)
mRNA, partial cds
CTGTCCCTCACCTGCACTGTCTCTGGATTCTCTTTGAGCAGTTACGGTGTAGGCTGGGTCCGCCAGGCTCCAGGAAA
AGGGCTGGAATTTGTTGGTGGTATAGCTAGTAGTGGAAGTGCAAACTACAACCCAGCCCTGAAGTCCCGAGCCAGCA
TCACCAAGGACACCTCAAAGAGCCAAGTTTATCTGACGCTGAACAGCCTGACAGGCGAGGACACGGCCGTCTATTAC
TGTGCGAGATCGGGTACCGAGGATGGTTACTATGGTAGTTACTACAGTAGCCACTATGTGGGCTATTTTGGCTACTG
GGGCCAGGGCACCCTGGTCACCGTCTCCTCAGAGAGTACGAAGACCCCAGATCTCTTCCCCCTCGTCTCCTGTGGGC
CCTCTCTTGATGAGAGCCTGGTGGCTGTGGGCTGCCTAGCCCGGGACTTCCTACCCAA
>KY437303.1 Equus caballus clone 147 immunoglobulin mu heavy chain (IGHM)
mRNA, partial cds
CTGTCCCTCACCTGCACTGTCTCTGGATTCTCTTTGAGCAGTTACGGTGTAGGCTGGGTCCGCCAGGCTCCAGGAAA
AGGGCTGGAATTTGTTGGTGCAATAGATAGTAGTGGAAGTGCAAACTACAACCCAGCCCTGAAGTCCCGAGCCAGCA
TCACCAAGGACACCTCAAAGAGCCAAGTTTATCTGACGCTGAACAGCCTGACAGGCGAGGACACGGCCGTCTATTAC
TGTGCGAGGATTTTAGTCGAGAAGACGTGGAGTACGAATCTTTTTGGCTACTGGGGCCAGGGCACCCTGGTCACCGT
CTCCTCAGAGAGTACGAAGACCCCAGATCTCTTCCCCCTCGTCTCCTGTGGGCCCTCTCTTGATGAGAGCCTGGTGG
CTGTGGGCTGCCTAGCCCGGGACTTCCTACCCAA
>KY437302.1 Equus caballus clone 146 immunoglobulin mu heavy chain (IGHM)
mRNA, partial cds
CTCTCCCTCACCTGCACTGTCTCTGGATTATCTTTGAGCAGTTATGCTGTAGGCTGGGTCCGCCAGGCTCCAGGAAA
AGGGCTGGAATATGTTGGTGCTATAGCTAGTAGTGGAAGTGCAAACTACAACCCAGCCCTGAAGTCCCGAGCCAGCA
TCACCAAGGACACCTCAAAGAGCCAAGTTTATCTGACGCTGAACAGCCTGACAGGCGAGGACACGGCCGTCTATTAC
TGTGCGAGCCGTAATGCTGGTAGTTACTATAACAGTCTGATGTACTGGGGCCAGGGCACCCTGGTCACCGTCTCCTC
AGAGAGTACGAAGACCCCAGATCTCTTCCCCCTCGTCTCCTGTGGGCCCTCTCTTGATGAGAGCCTGGTGGCTGTGG
GCTGCCTAGCCCGGGACTTCCTACCCAA
>KY437301.1 Equus caballus clone 145 immunoglobulin mu heavy chain (IGHM)
mRNA, partial cds
CTGTCCCTCACCTGCACTGTCTCTGGATTCTCTTTGAGCAGTGTCGGTGTAGGCTGGGTCCGCCAGGCTCCAGGAAA
AGGGCTGGAATTTGTTGGTTATATAGCTAGTAGTGGAACTGCAAACTACAACCCAGCCCTGAAGTCCCGAGCCAGCA
TCACCAAGGACACCTCAAAGAGCCAAGTTTATCTGACGCTGAACAGCCTGACAGGCGAGGACACGGCCGTCTATTGG
TGTGCGAGCGCGGATAGCAGCTATTCTTGGTATTCCTATGGTATAACCTACTGGGGCCAGGGCATCCTGGTCACCGT
CTCCTCAGAGAGTACGAAGACCCCAGATCTCTTCCCCCTCGTCTCCTGTGGGCCCTCTCTTGATGAGAGCCTGGTGG
CTGTGGGCTGCCTAGCCCGGGACTTCCTACCCAA
>KY437300.1 Equus caballus clone 144 immunoglobulin mu heavy chain (IGHM)
mRNA, partial cds
CTGTCCCTCACCTGCACTGTCTCTGGATTCTCTTTGAGCAGTTACGGTGTAGGCTGGGTCCGCCAGGCTCCAGGAAA
AGGGCTGGAATTTGTTGGTGCTATATATGGTAGTGCAAGTGCAAACTACAACCCAGCCCTGAAGTCCCGAGCCAGCA
TCACCAAGGACACCTCAAGGAGCCAAGTTTATCTGACGCTGAACAGCCTGACAGGCGAGGACACGGCCGTCTATTAC
TGTGCGAGATCCATATATAGTGTTGGTAGTAGCTATGCCCCTAGTTATGGCTACGTGGATCACTGGGGCCAGGGCAC
CCTGGTCACCGTCTCCTCAGAGAGTACGAAGACCCCAGATCTCTTCCCCCTCGTCTCCTGTGGGCCCTCTCTTGATG
AGAGCCTGGTGGCTGTGGGCTGCCTAGCCCGGGACTTCCTACCCAA
>KY437299.1 Equus caballus clone 143 immunoglobulin mu heavy chain (IGHM)
mRNA, partial cds
CTGTCCCTCACCTGCACTGTCTCTGGATTATCTTTGAGCAGTAATGCTGTAAACTGGGTCCGCCAGGCTCCAGGAAA
AGGGCTGGAGTGGGTTGGTGTTATATATGGTCGCGGCGCTACATACTACAGCCCAGCCCTGAAGTCCCGAGCCAGCA
TCACCAAGGACACCTCAAAGAGCCAAGTTTATCTGACGCTGAACAGCCTGACAGGCGAAGACACGGCCGTCTATTAC
TGTGCAGATTACATTAGTGGTAGTTTTGGCAACTGGGGCCAGGGCACCCTGGTCACCGTCTCCTCAGAGAGTACGAA

GACCCCAGATCTCTTCCCCCTCGTCTCCTGTGGGCCCTCTCTTGATGAGAGCCTGGTGGCTGTGGGCTGCCTAGCCC
GGGACTTCCTACCCAA
>KY437298.1 Equus caballus clone 142 immunoglobulin mu heavy chain (IGHM)
mRNA, partial cds
CTCTCCCTCACCTGCACTGTCTCTGGATTCTCTTTGAGCAATTACGGTGCAGGCTGGGTCCGCCAGGCTCCAGGAAA
AGGGCTGGAATTTGTTGGTGGTATAGCTAGTAGTGGAAGTGCAAACTACAACCCAGCCCTGAAGTCCCGAGCCAGCA
TCACCAAGGACACCTCAAAGAGCCAAGTTTATCTGACGCTGAACAGCCTGACAAGCGAGGACACGGCCGTCTATTAC
TGTGCAGGACAGAATACCGGACTTATAGACTACTGGGGCCAGGGCATCCTGGTCACCGTCTCCTCAGAGAGTACGAA
GACCCCAGATCTCTTCCCCCTCGTCTCCTGTGGGCCCTCTCTTGATGAGAGCCTGGTGGCTGTGGGCTGCCTAGCCC
GGGACTTCCTACCCAA
>KY437297.1 Equus caballus clone 141 immunoglobulin mu heavy chain (IGHM)
mRNA, partial cds
CTCTCCCTCACCTGCACTGTCTCTGGATTATCTTTGAGCAGTTATGCTGTAGGCTGGGTCCGCCAGGCTCCAGGAAA
AGGGCTGGAATATGTTGGTGCTATATATGGTAGTGCAAGTGCAAACTACAACCCAGCCCTGAAGTCCCGAGCCAGCA
TCACCAAGGACACCTCAAAGAGCCAAGTTTATCTGACGCTGAACAGCCTGACAGGCGAGGACACGGCCGTCTATTAC
TGTGCGAGATCCGGGGTTTACGATGGTTCCTATGCTGGTAGTTACTATGCCGATGGTATAGACTACTGGGGCCAGGG
CATCCTGGTCACCGTCTCCTCAGAGAGTACGAAGACCCCAGATCTCTTCCCCCTCGTCTCCTGTGGGCCCTCTCTTG
ATGAGAGCCTGGTGGCTGTGGGCTGCCTAGCCCGGGACTTCCTACCCAA
>KY437296.1 Equus caballus clone 140 immunoglobulin mu heavy chain (IGHM)
mRNA, partial cds
CTGTCCCTCACCTGCACTGTCTCTGGATTCTCTTTGAGCAGTTACGGTGTAGGCTGGGTCCGCCAGGCTCCAGGAAA
AGGGCTGGAATTTGTTGGTGATATACCTAGTAGTGGAAGTGCAAACTACAACCCAGCCCTGAAGTCCCGAGCCAGCA
TCACCAAGGACACCTCAAAGAGCCAAGTTTATCTGACGCTGAACAGCCTGACAGGCGAGGACACGGCCGTCTATTAC
TGTGCGAGATACGCGGTGCAAGCCTACGGTTATGGTTATACTACTGGCTCCTTTGCCTACTGGGGCCAGGGCACCCT
GGTCACCGTCTCCTCAGAGAGTACGAAGACCCCAGATCTCTTCCCCCTCGTCTCCTGTGGGCCCTCTCTTGATGAGA
GCCTGGTGGCTGTGGGCTGCCTAGCCCGGGACTTCCTACCCAA
>KY437295.1 Equus caballus clone 139 immunoglobulin mu heavy chain (IGHM)
mRNA, partial cds
CTCTCCCTCACCTGCACTGTCTCTGGATTATCTTTGAGCAGTAATGATGTAGGCTGGGTCCGCCAGGCTCCAGGAAA
AGGGCTGGAATACGTTGGTCTTATACGCGGTAGTGCAAGTGCAAACTACAACCCAGCCCTGAAGTCCCGAGCCAGCA
TCACCAAGGACACCTCAAAGAGCCAAGTTTATCTGACGCTGAACAGCCTGACAAGCGAGGACACGGCCGTCTATTAC
TGTGCAGGACAAGACTATGCCGCAGCTGGTACATACTATAGTGGCGAGGGGGTTTACTGGGGCCAGGGCACCCTGGT
CACCGTCTCCTCAGAGAGTACGAAGACCCCAGATCTCTTCCCCCTCGTCTCCTGTGGGCCCTCTCTTGATGAGAGCC
TGGTGGCTGTGGGCTGCCTAGCCCGGGACTTCCTACCCAA
>KY437294.1 Equus caballus clone 138 immunoglobulin mu heavy chain (IGHM)
mRNA, partial cds
CTGTCCCTCACCTGCACTGTCTCTGGATTCTCTTTGAGCAGTTACGGTGTAGTCTGGGTCCGCCAGGCTCCAGGAAA
AGGGCTGGAATTTGTTGGTGGTATACGTAGTAGTGGAAGTGCAAACTACAACCCAGCCCTGAAGTCCCGAGCCAGCA
TCACCAAGGACACCTCAAAGAGCCAAGTTTATCTGACGCTGAACAGCCTGACAGGCGAGGACACGGCCGTCTATTAC
TGTGTGAGGTCTATGGTTAGTGGAGCAGTGGATATAGGATGGTTCAGCGACTGGGGCCAGGGCACCCTGGCCACCGT
CTCCTCAGAGAGTACGAAGACCCCAGATCTCTTCCCCCTCGTCTCCTGTGGGCCCTCTCTTGATGAGAGCCTGGTGG
CTGTGGGCTGCCTAGCCCGGGACTTCCTACCCAA
>KY437293.1 Equus caballus clone 137 immunoglobulin mu heavy chain (IGHM)
mRNA, partial cds
CTGTCCCTCACCTGCACTGTCTCTGGATTATCTTTGAGCAGTAATGCTGTAGGCTGGGTCCGCCAGGCTCCAGGAAA
AGGGCTGGAGTGGGTTGGTGTTATATATGGTAGTGAAAGTACATACTACAACCCAGCCCTGAAGTCCCGAGCCAGCA
TCACCAAGGACACCTCAAAGAGCCAAGTTTATCTGACGCTGAACAGCCTGACAGGCGAAGACACGGCCGTCTATTAC
TGTGCAGGATCAATCGATGGTGGTAGTTCCTTTTTCCGCTTGGGAGGTGATGGGTACTATTTTGGCTACTGGGGCCA
GGGCACCCTGGTCACCGTCTCCTCAGAGAGTACGAAGACCCCAGATCTCTTCCCCCTCGTCTCCTGTGGGCCCTCTC
TTGATGAGAGCCTGGTGGCTGTGGGCTGCCTAGCCCGGGACTTCCTACCCAA
>KY437292.1 Equus caballus clone 136 immunoglobulin mu heavy chain (IGHM)
mRNA, partial cds
CTCTCCCTCACCTGCACTGTCTCTGGATTATCTTTGAGCAGTAATGCTGTAGGCTGGGTCCGCCAGGCTCCAGGAAA
AGGGCTGGAATACGTTGGTGCTATATATGGTAGTGCAAGTGCAAACTACAACCCAGCCCTGAAGTCCCGAGCCAGCA
TCACCAAGGACACCTCAAAGAGCCAAGTTTATCTGACGCTGAACAGCCTGACAGGCGAGGACACGGCCGTCTATTAC

TGTGCGAAACGAAGTGGTGATATTTTCTGGTATTTTACGTACTGGGGCCAGGGCACCCTGGTCACCGTCTCCTCAGA
GAGTACGAAGACCCCAGATCTCTTCCCCCTCGTCTCCTGTGGGCCCTCTCTTGATGAGAGCCTGGTGGCTGTGGGCT
GCCTAGCCCGGGACTTCCTACCCAA
>KY437291.1 Equus caballus clone 135 immunoglobulin mu heavy chain (IGHM) mRNA, partial cds
CTGTCCCTCACCTGCACTGTCTCTGGATTATCTTTGAGCAGCTATGCTGTAGGCTGGGTCCGCCAGGCTCCAGGAAA
AGGGCTGGAGTGGATTGGTGTTATAGATGGTAGTGAAAGTACAGTCTACAACCCAGCCCTGAAGTCCCGAGCCAGCA
TCACCAAGGACACCTCAAAGAGCCAAGTTTATCTGACGCTGAACAGCCTGACAGGCGAAGACACGGCCGTCTATTAC
TGTGCAGGATGTTCCGACGATGCTACTAGTGTGTATAGGGGTAATATGGACCCCTGGGGCCAGGGCACCCTGGTCAC
CGTCTCCTCAGAGAGTACGAAGACCCCAGATCTCTTCCCCCTCGTCTCCTGTGGGCCCTCTCTTGATGAGAGCCTGG
TGGCTGTGGGCTGCCTAGCCCGGGACTTCCTACCCAA
>KY437290.1 Equus caballus clone 134 immunoglobulin mu heavy chain (IGHM) mRNA, partial cds
CTCTCCCTCACCTGCACTGTCTCTGGATTCTCTTTGAGCAGTTACGGTGTAGGCTGGGTCCGCCAGGCTCCAGGAAA
AGGGCTGGAATTTGTTGGTGGTATACATAGTAGTGGAAGTGCAAACTACAACCCAGCCCTGAAGTCCCGAGCCAGCA
TCACCAAGGACACCGCAAAGAGCCAAGTTTATCTGACGCTGAACAGCCTGACAAGCGAGGACACGGCCGTCTATTAC
TGTGCAGGAGGGGGGCAAATTTCCTATGCTGGTAATTACTATGGGACTAACTACTGGGGCCAGGGCACCCTGGTCAC
CGTCTCCTCAGAGAGTACGAAGACCCCAGATCTCTTCCCCCTCGTCTCCTGTGGGCCCTCTCTTGATGAGAGCCTGG
TGGCTGTGGGCTGCCTAGCCCGGGACTTCCTACCCAA
>KY437289.1 Equus caballus clone 133 immunoglobulin mu heavy chain (IGHM) mRNA, partial cds
CTCTCCCTCACCTGCACTGTCTCTGGATTCTCTTTGAGCAGTTATGCTGTATACTGGGTCCGCCAGGCTCCAGGAAA
AGGGCTGGAATATGTTGGTGCTATATATGATAGTGCAAGTGCAAACTACAACCCAGCCCTGAAGTCCCGAGCCAGCA
TCACCAAGGACACCTCAAAGAGCCAAGTTTATCTGACGCTGAACAGCCTGACAGGCGAGGACACGGCCGTCTATTAC
TGTGCGAGACGCTATGGTTATGGTGCAACTTACTCTTTTGGTTATGATATAGACTACTGGGGCCAGGGCACCCTGGT
CACCGTCTCCTCAGAGAGTACGAAGACCCCAGATCTCTTCCCCCTCGTCTCCTGTGGGCCCTCTCTTGATGAGAGCC
TGGTGGCTGTGGGCTGCCTAGCCCGGGACTTCCTACCCAA
>KY437288.1 Equus caballus clone 132 immunoglobulin mu heavy chain (IGHM) mRNA, partial cds
CTCTCCCTCACCTGCACTGTCTCTGGATTATCTTTGAGCAGTAATGCTGTAGGCTGGGTCCGCCAGGCTCCAGGAAA
AGGGCTGGAATATGTTGGTGGTATAGTTAGTAGTGAAAGTGCAAACTACAACCCAGCCCTGAAGTCCCGAGCCAGCA
TCACCAAGGACACCTCAAAGAGCCAAGTTTACCTGACGCTGAGCAGCCTGACAAGGGAGGACACGGCCGTCTATTAC
TGTTTAGGACGGAATGGTTATGGTAGTGCTTACTACAGTGGTATAGACTACTGGGGCCAGGGCATCCTGGTCACCGT
CTCCTCAGAGAGTACGAAGACCCCAGATCTCTTCCCCCTCGTCTCCTGTGGGCCCTCTCTTGATGAGAGCCTGGTGG
CTGTGGGCTGCCTAGCCCGGGACTTCCTACCCAA
>KY437287.1 Equus caballus clone 131 immunoglobulin mu heavy chain (IGHM) mRNA, partial cds
CTCTCCCTCACCTGCACTGTCTCTGGATTATCTTTGAGCAGTTATGCTGTAGGCTGGGTCCGCCAGGCTCCAGGAAA
AGGGCTGGAATATGTTGGTGCTATATATGGTAGTTCAAGTGCAAACTACAACCCAGCCCTGAAGTCCCGAGCCAGCA
TCACCAAGGACACCTCAAAGAGCCAAGTTTATCTGACGCTGAACAGCCTGACAGGCGAGGACACGGCCGTCTATTAC
TGTGCGAGATACAATGGTTATGGTAGTGCTTACTACTCCCAATATGGTATAGACTACTGGGGCCAGGGCATCCTGGT
CACCGTCTCCTCAGAGAGTACGAAGACCCCAGATCTCTTCCCCCTCGTCTCCTGTGGGCCCTCTCTTGATGAGAGCC
TGGTGGCTGTGGGCTGCCTAGCCCGGGACTTCCTACCCAA
>KY437286.1 Equus caballus clone 130 immunoglobulin mu heavy chain (IGHM) mRNA, partial cds
CTGTCCCTCACCTGCACTGTCTCTGGATTCTCTTTGAGCAGTTACAGTGTAGGCTGGGTCCGCCAGGCTCCAGGAAA
AGGGCTGGAATTTGTTGGTGGTATACGTAGTAGTGGAAGTGCAAACTACAACCCAGCCCTTAAGTCCCGAGCCAGCA
TCACCAAGGACACCTCAAAGAGCCAAGTTTATCTGACGCTGAACAGCCTGACAGGCGAGGACACGGCCGTCTATTAC
TGTGCGAGATTTTTCTATGAATCCTATGATGGTATTTGGTATTATGCTATACATTACTGGGGCCAGGGCATCCTGGT
CACCGTCTCCTCAGAGAGTACGAAGACCCCAGATCTCTTCCCCCTCGTCTCCTGTGGGCCCTCTCTTGATGAGAGCC
TGGTGGCTGTGGGCTGCCTAGCCCGGGACTTCCTACCCAA
>KY437285.1 Equus caballus clone 129 immunoglobulin mu heavy chain (IGHM) mRNA, partial cds
CTGTCCCTCACCTGCACTGTCTCTGGATTCTCTTTGAGCAGTGTCGGTGTAGGCTGGGTCCGCCAGGCTCCAGGAAA
AGGGCTGGAATTTGTTGGTTATATAGCTAGTAGTGGAACTGCAAACTACAACCCAGCCCTGAAGTCCCGAGCCAGCA
TCACCAAGGACACCTCAAAGAGCCAAGTTTATCTGACGCTGAACAGCCTGACAGGCGAGGACACGGCCGTCTATTGG

TGTGCGAGCGCGGATAGCAGCTATTCTTGGTATTCCTATGGTATAACCTACTGGGGCCAGGGCATCCTGGTCACCGT
CTCCTCAGAGAGTACGAAGACCCCAGATCTCTTCCCCCTCGTCTCCTGTGGGCCCTCTCTTGATGAGAGCCTGGTGG
CTGTGGGCTGCCTAGCCCGGGACTTCCTACCCAA
>KY437284.1 Equus caballus clone 128 immunoglobulin mu heavy chain (IGHM) mRNA, partial cds
CTGTCCCTCACCTGCACTGTCTCTGGATTCTCTTTGAGCACGAATGGTGTGGGCTGGGTCCGCCAGGCTCCAGGAAA
AGGGCTGGAATTTGTTGGTGGTATAGGTAATAGTGGAACAGTATACTACAACCCAGCCCTGAAGTCCCGAGCCAGCA
TCACCAAGGACACCTCAAAGAGCCAGCTTTATCTGACGCTGAACAGCCTGACAAGCGAGGACACGGCCGTCTATTAC
TGTGCAGGATACTACTATCGCAGCTATTTTGTCTACTGGGGCCAGGGCACCCTGGTCACCGTCTCCTCAGAGAGTAC
GAAGACCCCAGATCTCTTCCCCCTCGTCTCCTGTGGGCCCTCTCTTGATGAGAGCCTGGTGGCTGTGGGCTGCCTAG
CCCGGGACTTCCTACCCAA
>KY437283.1 Equus caballus clone 127 immunoglobulin mu heavy chain (IGHM) mRNA, partial cds
CTCTCCCTCACCTGCACTGTCTCTGGATTCTCTTTGAGCAGTTACGGTGTAGGCTGGGTCCGCCAGGCTCCAGGAAA
AGGGCTGGAATTTGTTGGTGGTATAGCTAGTAGTGGAAGTGCAAACTACAACCCAGCCCTGAAGTCCCGAGCCAGCA
TCACCAAGGACACCTCAAAGAGCCAAGTTTATCTGACGCTGAACAGCCTGACAAGCGAGGACACGGCCGTCTATTAC
TGTGTGAGGACCGGATTTGGCGACTGGGGCCAGGGCACCCTGGTCACCGTCTCCTCAGAGAGTACGAAGACCCCAGA
TCTCTTCCCCCTCGTCTCCTGTGGGCCCTCTCTTGATGAGAGCCTGGTGGCTGTGGGCTGCCTAGCCCGGGACTTCC
TACCCAA
>KY437282.1 Equus caballus clone 126 immunoglobulin mu heavy chain (IGHM) mRNA, partial cds
CTCTCCCTCACCTGCACTGTCTCTGGATTCTCTTTGAGCAGTTATGCTGTATACTGGGTCCGCCAGGCTCCAGGAAA
AGGGCTGGAATATGTTGGTGCTATATATGATAGTGCAAGTGCAAACTACAACCCAGCCCTGAAGTCCCGAGCCAGCA
TCACCAAGGACACCTCAAAGAGCCAAGTTTATCTGACGCTGAACAGCCTGACACGCGAGGACACGGCCGTCTATTAC
TGTGCAGGAAACTACGCCTTTGGTACTGCTCCGTACTGGGGCCAGGGCACCCTGGTCACCGTCTCCTCAGAGAGTAC
GAAGACCCCAGATCTCTTCCCCCTCGTCTCCTGTGGGCCCTCTCTTGATGAGAGCCTGGTGGCTGTGGGCTGCCTAG
CCCGGGACTTCCTACCCAA
>KY437281.1 Equus caballus clone 125 immunoglobulin mu heavy chain (IGHM) mRNA, partial cds
CTGTCCCTCACCTGCACTGTCTCTGGATTCTCTTTGGGCAGTTACGGTGTAGGCTGGGTCCGCCAGGCTCCAAGAAA
AGGGCTGGAATTTGTTGGTGGTATAGCTACTAGTGGAAGTGCAAACTACAACCCAGCCCTGAAGTCCCGAGCCAGCA
TCACCAAGGACACCTCAAAGAGCCAAGTTTATCTGACGCTGAACAGCCTGACAGGCGAGGACACGGCCGTCTATTAC
TGTGCGAGACAGATTGCCTATGGTGGTAAATACTACTACAGTTTTGGCTACTGGGGCCAGGGCACCCTGGTCACCGT
CTCCTCAGAGAGTACGAAGACCCCAGATCTCTTCCCCCTCGTCTCCTGTGGGCCCTCTCTTGATGAGAGCCTGGTGG
CTGTGGGCTGCCTAGCCCGGGACTTCCTACCCAA
>KY437280.1 Equus caballus clone 124 immunoglobulin mu heavy chain (IGHM) mRNA, partial cds
CTCTCCCTCACCTGCACTGTCTCTGGATTATCTTTGAGCACGAATGGTGTGGGCTGGGTCCGCCAGGCTCCAGGAAA
AGGGCTGGAATTTGTTGGTGGTATAGGTAATAGTGGAACAGTATACTACAACCCAGCCCTGAAGTCCCGAGCCAGCA
TCACCAAGGACACCTCAAAGAGCCAGCTTTATCTGACGCTGAACAGCCTGACAAGCGAGGACACGGCCGTCTATTAC
TGTGCAGGATACTACTATCGCAGCTATTTTGTCTACTGGGGCCAGGGCACCCTGGTCACCGTCTCCTCAGAGAGTAC
GAAGACCCCAGATCTCTTCCCCCTCGTCTCCTGTGGGCCCTCTCTTGATGAGAGCCTGGTGGCTGTGGGCTGCCTAG
CCCGGGACTTCCTACCCAA
>KY437279.1 Equus caballus clone 123 immunoglobulin mu heavy chain (IGHM) mRNA, partial cds
CTGTCCCTCACCTGCACTGTCTCTGGATTCTCTTTGAGCAGTTACGGTGTAGGCTGGGTCCGCCAGGCTCCAGGAAA
AGGGCTGGAATTTGTTGGTGGTATAGGTAGTAGTGGAAGTGCAAACTACAACCCAGCCCTGAAGTCCCGAGCCAGCA
TCACCAAGGACACCTCAAAGAGCCAAGTTTATCTGACGCTGAACAGCCTGACAGGCGAGGACACGGCCGTCTATTAC
TGTGCGAGAATGATGGGTGGTTATGGTCCATACTGGGGCCAGGGCACCCTGGTCACCGTCTCCTCAGAGAGTACGAA
GACCCCAGATCTCTTCCCCCTCGTCTCCTGTGGGCCCTCTCTTGATGAGAGCCTGGTGGCTGTGGGCTGCCTAGCCC
GGGACTTCCTACCCAA
>KY437278.1 Equus caballus clone 122 immunoglobulin mu heavy chain (IGHM) mRNA, partial cds
CTCTCCCTCACCTGCACTGTCTCTGAGTTGTCAGAGAGCAGTTATGCTGTATACTGGGTCCGCCAGGCTCCAGGAAA
AGGGCTGGAATATGTAGGTGATATAACTAATAGTGGAAGTACAATGTACAACCCTGCCCTGAAGTCCCGAGCCAGCG

TCACCAAGGACACCTCCAAGAGCCAAGTTTATCTGACGCTGAACAGCCTGACAGGCGAGGACACGGCCGTCTATTAC
TGTGCGAAACGAAGTGGTGATATTTTCTGGTATTTTACGTACTGGGGCCAGGGCACCCTGGTCACCGTCTCCTCAGA
GAGTACGAAGACCCCAGATCTCTTCCCCCTCGTCTCCTGTGGCCCTCTCTTGATGAGAGCCTGGTGGCTGTGGGCT
GCCTAGCCCGGGACTTCCTACCCAA
>KY437277.1 Equus caballus clone 121 immunoglobulin mu heavy chain (IGHM) mRNA, partial cds
CTGTCCCTCACCTGCACTGTCTCTGGATTCTCTTTGAGCGTTTACGGTGTAGGCTGGGTCCGCCAGGCTCCAGGAAA
AGGGCTGGAATTTGTTGGTAGTATAGATAGTAGTGGTAATGCAAACTACAATACAGCCCTGAAGTCCCGAGCCAGCA
TCACCAAGGACACCTCAAAGAGCCAAGTTTATCTGACGCTGAACAGCCTGACAGGCGAGGACACGGCCGTCTATTAC
TGTGCGAGATCTCTCTTCGATACGACTATGGTTGGGTCTTTTGGCTACTGGGGCCAGGGCACCCTGGTCACCGTCTC
CTCAGAGAGTACGAAGACCCCAGATCTCTTCCCCCTCGTCTCCTGTGGCCCTCTCTTGATGAGAGCCTGGTGGCTG
TGGGCTGCCTAGCCCGGGACTTCCTACCCAA
>KY437276.1 Equus caballus clone 120 immunoglobulin mu heavy chain (IGHM) mRNA, partial cds
CTGTCCCTCACCTGCACTGTCTCTGGATTCTCTTTGAGCAGTTACGGTGTAGGCTGGGTCCGCCAGGCTCCAGGAAA
AGGGCTGGAATTTGTTGGTGGTATAGCTAGTAGTGGAAGTGCAAACTACAACCCAGCCCTGAAGTCCCGAGCCAGCA
TCACCAAGGACACCTCAAAGAGCCAAGTTTATCTGACGCTGAACAGCCTGACAAGCGAGGACACGGCCGTCTATTAC
TGTGCAGGAGACTATGGTTGGGGTGGTGCTTACTACTACTTTCGTATATACTACTGGGGCCAGGGCATCCTGGTCAC
CGTCTCCTCAGAGAGTACGAAGACCCCAGATCTCTTCCCCCTCGTCTCCTGTGGCCCTCTCTTGATGAGAGCCTGG
TGGCTGTGGGCTGCCTAGCCCGGGACTTCCTACCCAA
>KY437275.1 Equus caballus clone 119 immunoglobulin mu heavy chain (IGHM) mRNA, partial cds
CTCTCCCTCACCTGCACTGTCTCTGGATTCTCTTTGAGCAGTTATGCTGTATACTGGGTCCGCCAGGCTCCAGGAAA
AGGGCTGGAATATGTTGGTACTATATATAATAGTGCAAGTGCAAACTACAACCCAGCCCTGAAGTCCCGAGCCAGCA
TCACCAAGGACACCTCAAAGAGCCAAGTTTATCTGACGCTGAACAGCCCGACAGGCGAGGACACGGCCGTCTATTAC
TGTGCGAGAGGGGAAGACTGGACTTATGGTTATGCACCGTTGTATTTTAGCTACTGGGGCCAGGGCACCCTGGTCAC
CGTCTCCTCAGAGAGTACGAAGACCCCAGATCTCTTCCCCCTCGTCTCCTGTGGCCCTCTCTTGATGAGAGCCTGG
TGGCTGTGGGCTGCCTAGCCCGGGACTTCCTACCCAA
>KY437274.1 Equus caballus clone 118 immunoglobulin mu heavy chain (IGHM) mRNA, partial cds
CTCTCCCTCACCTGCACTGTCTCTGGATTATCTTTGAGCAGTTATGGTGTGGGCTGGGTCCGCCAGGCTCCAGGAAA
AGGGCTGGAGTGGGTTGGTGTTATAGATGTTATATATGGTAGTGAAAGTACATACTACAACCCAGCCCTGAAGTCCC
GAGCCAGCATCACCAAGGACACCTCAAAGAGCCAAGTTTATCTGACGCTGAACAGCCTGACAGGCGAAGACACGGCC
GTCTATTACTGTGCAGGATGTCTCTCTGGTTACTATGCTGGTTATTACTACAACCCCGCTTATGGTATAAACTACTG
GGGCCAGGGCATCCTGGTCACCGTCTCCTCAGAGAGTACGAAGACCCCAGATCTCTTCCCCCTCGTCTCCTGTGGGC
CCTCTCTTGATGAGAGCCTGGTGGCTGTGGGCTGCCTAGCCCGGGACTTCCTACCCAA
>KY437273.1 Equus caballus clone 117 immunoglobulin mu heavy chain (IGHM) mRNA, partial cds
CTCTCCCTCACCTGCACTGTCTCTGGAGTATCTTTGAGCAGTTATGGTGTGGGCTGGGTCCGCCAGGCTCCAGGAAA
AGGACTGGAATTTGTTGGTGGTATACTTAGTAGTGGAAGTCCAAACTACAACCCAGCCCTGAAGTCCCGAGCCAGCA
TCACCAAGGACACCTCAAAGAGCCGACTTTATTTGACGCTGAACAGCCTGACAAGCGAGGACACGGCCGTCTATTAC
TGTACAGGAGGTGCTGTAGTAAATGGACTTGGCTACTGGGGCCAGGGCACCCTGGTCACCGTCTCCTCAGAGAGTAC
GAAGACCCCAGATCTCTTCCCCCTCGTCTCCTGTGGCCCTCTCTTGATGAGAGCCTGGTGGCTGTGGGCTGCCTAG
CCCGGGACTTCCTACCCAA
>KY437272.1 Equus caballus clone 116 immunoglobulin mu heavy chain (IGHM) mRNA, partial cds
CTCTCCCTCACCTGCACTGTCTCTGGATTATCTTTGAGCAGTTATGGTGTGGGCTGGGTCCGCCAGGCTCCAGGAAA
AGGGCTGGAATTTGTTGGTGGTATAGGTAGTAGTGGAAGTGCAAACTACAACCCAGCCCTGAAGTCCCGAGCCAGCA
TCACCAAGGACACCTCAAAGAGCCAAGTTTATCTGACGCTGAACAGCCTGACAAGCGAGGACACGGCCGTCTATTAC
TGTGCAGGAGGGCGGTCCTATGCTGGTAGTTATTTTGGCTACTGGGGCCAGGGCACCCTGGTCACCGTCTCCTCAGA
GAGTACGAAGACCCCAGATCTCTTCCCCCTCGTCTCCTGTGGCCCTCTCTTGATGAGAGCCTGGTGGCTGTGGGCT
GCCTAGCCCGGGACTTCCTACCCAA
>KY437271.1 Equus caballus clone 115 immunoglobulin mu heavy chain (IGHM) mRNA, partial cds
CTCTCCCTCACCTGCACTGTCTCTGGAGTCTCTTTGAGGAGTTACGGTGTAGAATGGGTCCGCCAGGCTCCAGGAAA
AGGGCTGGAATTTGTCGGTAGTATAACTAATAGTGGAAGTGCAAACTACAACCCAGCCCTGAAGTCCCGAGCCAGCA

TCACCAAGGACACCTCAAAGAGCCAAGTTTATCTGACGCTGAACAGCCTGACAAGCGAGGACACGGCCGTCTATTAC
TGTGCAGGAGGCGGGATATACGGGGACGGTTCCTATGCTGGTAGTTACTTATACTACGGCCGAGTCCGTCGTAATGG
TATAAACTACTGGGGCCAGGGCATCCTGGTCACCGTCTCCTCAGAGAGTACGAAGACCCCAGATCTCTTCCCCCTCG
TCTCCTGTGGGCCCTCTCTTGATGAGAGCCTGGTGGCTGTGGGCTGCCTAGCCCGGGACTTCCTACCCAA
>KY437270.1 Equus caballus clone 114 immunoglobulin mu heavy chain (IGHM) mRNA, partial cds
CTCTCCCTCACCTGCACTGTCTCTGGATTATCTTTGAGCAGTGTTGCTGTAGGCTGGGTCCGCCAGGCTCCAGGAAA
AGGGCTGGAATTTGTTGGTGGTATAGTGGGTAGTGGAAGTGCAAACTACAACCCAGCCCTGAAGTCCCGAGCCAGCA
TCACCAAGGACACCTCAAAGAGCCAAGTTTATCTGACGCTGAACAGCCTGACAGGCGAGGACACGGCCGTCTATTAC
TGTGTTTCTGGGACCTATGGTTATGGTGGTGCTTTGTCCGAGTACTATTTTGGCTACTGGGGCCAGGGCACCCTGGT
CACCGTCTCCTCAGAGAGTACGAAGACCCCAGATCTCTTCCCCCTCGTCTCCTGTGGGCCCTCTCTTGATGAGAGCC
TGGTGGCTGTGGGCTGCCTAGCCCGGGACTTCCTACCCAA
>KY437269.1 Equus caballus clone 113 immunoglobulin mu heavy chain (IGHM) mRNA, partial cds
CTGTCCCTCACCTGCACTGTCTCTGGATTCTCTTTGACCAGTAACGGTGTATTCTGGGTCCGCCAGGCTCCAGGAAA
AGGGCTGGAATTTGTTAGTAGGATGGCTATTAGTGGAAGTGCGTACTACAACCCAGCCCTGAAGTCCCGAGCCAGCA
TCACCAAGGACACCTCAAAGAGCCAAGTTTATCTGACGCTGAACAGCCTGACAGGCGAGGACACGGCCGTCTATTAC
TGTGCGAATTCGCGTTATGGTTGGGGCCAGGGCACCCTGGTCACCGTCTCCTCAGAGAGTACGAAGACCCCAGATCT
CTTCCCCCTCGTCTCCTGTGGGCCCTCTCTTGATGAGAGCCTGGTGGCTGTGGGCTGCCTAGCCCGGGACTTCCTAC
CCAA
>KY437268.1 Equus caballus clone 112 immunoglobulin mu heavy chain (IGHM) mRNA, partial cds
CTGTCCCTCACCTGCACTGTCTCTGGATTCTCTTTGAGCAGTTACAAGGTAGGCTGGGTCCGCCAGGCTCCAGGAAA
AGGGCTGGAATTCGTTGGTGTAATAGATCGTGATGGAAGTTCGCACTACAACCCAGCCCTGAAGTCCCGAGCCAGCA
TCACCAAGGACACCTCAAAGAGCCAAGTTTATCTGACGCTGAACAGCCTGACAGGCGAGGACACGGCCGTCTATTAC
TGTGCGAGAGTGATTAGTGTAGTGGGAGATGTTATACGGAGTTATTGGGGCCAGGGCACCCTGGTCACCGTCTCCTC
AGAGAGTACGAAGACCCCAGATCTCTTCCCCCTCGTCTCCTGTGGGCCCTCTCTTGATGAGAGCCTGGTGGCTGTGG
GCTGCCTAGCCCGGGACTTCCTACCCAA
>KY437267.1 Equus caballus clone 111 immunoglobulin mu heavy chain (IGHM) mRNA, partial cds
CTCTCCCTCACCTGCACTGTCTCTGGATTATCTTTGAGCAGTTATGGTGTGGGCTGGGTCCGCCAGGCTCCAGGAAA
AGGGCTGGAATTTGTTGGTGGTATAGCTAGTAGTGGAAGTGCAAACTACAACCCAGCCCTGAAGTCCCGAGCCAGCA
TCACCAAGGACACCTCAAAGAGCCAAGTTTATCTGACGCTGAACAGCCTGACAAGCGAGGACACGGCCGTCTATTAC
TGTGCAGGAGGCTATTCGAGGGCGGTGGCCGTGGATCACTGGGGCCAGGGCACCCTGGTCACCGTCTCCTCAGAGAG
TACGAAGACCCCAGATCTCTTCCCCCTCGTCTCCTGTGGGCCCTCTCTTGATGAGAGCCTGGTGGCTGTGGGCTGCC
TAGCCCGGGACTTCCTACCCAA
>KY437266.1 Equus caballus clone 110 immunoglobulin mu heavy chain (IGHM) mRNA, partial cds
CTCTCCCTCACCTGCACTGTCTCTGGATTCTCTTTGAGCAGTTACGGTGTAGGCTGGGTCCGCCAGGCTCCAGGAAA
AGGGCTGGAATTTGTTGGTGGTATAGCTAGTAGTGGAAGTGCAAACTACAACCCAGCCCTGAAGTCCCGAGCCAGCA
TCACCAAGGACACCTCAAAGAGCCAAGTTTATCTGACGCTGAACAGCCTGACAGGCGAGGACACGGCCGTCTATTAC
TGTGTTTCTGGGACCTATGGTTATGGTGGTGCTTTGTCCGAGTACTATTTTGGCTACTGGGGCCAGGGCACCCTGGT
CACCGTCTCCTCAGAGAGTACGAAGACCCCAGATCTCTTCCCCCTCGTCTCCTGTGGGCCCTCTCTTGATGAGAGCC
TGGTGGCTGTGGGCTGCCTAGCCCGGGACTTCCTACCCAA
>KY437265.1 Equus caballus clone 109 immunoglobulin mu heavy chain (IGHM) mRNA, partial cds
CTGTCCCTCACCTGCACTGTCTCTGGATTCTCTTTGAGCAGTTATGCTGTATACTGGGTCCGCCAGGCTCCAGGAAA
AGGGCTGGAATATGTTGGACTTAGCACTGGTAGTGGTAGTGCAAAGTACAACCCAGCCCTGAAGTCCCGAGCCAGCA
TCACCAAGGACACCTCAAAGAGCCAAGTTTATCTGACTCTGAACAGCCTGACAGGCGAGGACACGGCCGTCTATTAC
TGTGCGAGATCCCACGATAGTACAAATAATTGGTGGGCCTACTGGGGCCAGGGCACCCTGGTCACCGTCTCCTCAGA
GAGTACGAAGACCCCAGATCTCTTCCCCCTCGTCTCCTGTGGGCCCTCTCTTGATGAGAGCCTGGTGGCTGTGGGCT
GCCTAGCCCGGGACTTCCTACCCAA
>KY437264.1 Equus caballus clone 108 immunoglobulin mu heavy chain (IGHM) mRNA, partial cds

CTGTCCCTCATCTGCACTGTCTCTGGTTTCTCTTTGAGCAATTACGGTGTAGGCTGGGTCCGCCAGGCTCCAGGAAA
AGGGCTGGAATTTGTTGGTGGTATAGGTAGTAACAGAGGTACAATCTACAACCCAGCCCTGAAGTCCCGAGCCAGCA
TCACCGAGGACACCTCAAAGAGCCAAGTTTATCTGACGCTGAACAGCCTGACAGGCGAGGACACGGCCGTCTATTAC
TGTGTGAGATCGAGTGTATCAGCCATGTCGATCGGAGCTAGCAACTATTGGGGCCAGGGCATCCTGGTCACCGTCTC
CTCAGAGAGTACGAAGACCCCAGATCTCTTCCCCCTCGTCTCCTGTGGGCCCTCTCTTGATGAGAGCCTGGTGGCTG
TGGGCTGCCTAGCCCGGGACTTCCTACCCAA
>KY437263.1 Equus caballus clone 107 immunoglobulin mu heavy chain (IGHM) mRNA, partial cds
CTGTCCCTCACCTGCACTGTCTCTGGATTATCTTTGAGCAGTAATGCTGTAGGCTGGGTCCGCCAGGCTCCAGGAAA
AGGGCTGGAATATGTTGGTAGTATAGCTAGTAGTGGAAGTGCAAACTACAACCCAGCCCTGAAGTCCCGAGCCAGCA
TCACCAAGGACACCTCAAAGAGCCAAGTTTATCTGACGCTGAACAGCCTGACAGGCGGGGACACGGCCGTCTATTAC
TGTGTGCGAGATGGTTATAATAGTAGGAGTTGCTCGCTGACCTCAATGTATGGACCCTGGGGCCAGGGCACCCTGGT
CACCGTCTCCTCAGAGAGTACGATGACCCCAGATCTCTTCCCCCTCGTCTCCTGTGGGCCCTCTCTTGATGAGAGCC
TGGTGGCTGTGGGCTGCCTAGCCCGGGACTTCCTACCCAA
>KY437262.1 Equus caballus clone 106 immunoglobulin mu heavy chain (IGHM) mRNA, partial cds
CTGTCCCTCACCTGCACTGTCTCTGGATTCTCTTTGAGCAATAGCGCAGTAGGCTGGGTCCGCCAGGCTCCAGGAGG
AGGGCTGGAATTTGTTGGTGGCGTACTGACTAGTGGAAGTGCAAACTACAACCCAGCCCTGAAGTCCCGAGCCAGCA
TCACCAAGGACACCTCAAAGAGCCAAGTTTATCTGACGCTGAACAGCCTGACAGGCGAGGACACGGCCGTCTATTAC
TGTGTAACGAATCTAGCAGCTTATGCCTACTGGGGCCAGGGCACCCTGGTCACCGTCTCCTCAGAGAGTACGAAGAC
CCCAGATCTCTTCCCCCTCGTCTCCTGTGGGCCCTCTCTTGATGAGAGCCTGGTGGCTGTGGGCTGCCTAGCCCGGG
ACTTCCTACCCAA
>KY437261.1 Equus caballus clone 105 immunoglobulin mu heavy chain (IGHM) mRNA, partial cds
CTGTCCCTCACCTGCGCTGTCTCTGGTTTATCTTTGAGCATGAGCAGTGACGCTGTAGGCTGGGTCCGCCAGGCTCC
AGGAAAAGGGCTGGAGTGGGTTGGTATGATATATGGTGATGAAATGACATACTACAACCCAGCCCTGAAGTCCCGAG
CCAGCATCACCAAGGACACCTCAAAGAGCCAACATTATCTGACGCTGAACAGCCTGACAAGCGAGGACACGGCCGTC
TATTACTGTGCAGGAACAAGAGACAGTTACTATGGTGGTAGATCCTGGTACCCCGACGTTGGTGGTGAGAACTACTG
GGGCCAGGGCATCCTGGTCACCGTCTCCTCAGAGAGTACGATGACCCCAGATCTCTTCCCCCTCGTCTCCTGTGGGC
CCTCTCTTGATGAGAGCCTGGTGGCTGTGGGCTGCCTAGCCCGGGACTTCCTACCCAA
>KY437260.1 Equus caballus clone 104 immunoglobulin mu heavy chain (IGHM) mRNA, partial cds
CTGTCCCTCACCTGCACTGTCTCTGGATTATCTTTGAGCAGTAATGCTGTAGGCTGGGTCCGCCAGGCTCCAGGAAA
AGGGCTGGAGTGGGTTGGTTATATATATGGTAGTGAAAGTACATACTACAACCCAGCCCTGAAGTCCCGAGCCAGCA
TCACCAAGGACACCTCAAAGAGCCAAGTTTATCTGACGCTGAACAGCCTGACAGGCGAAGACACGGCCGTCTATTAT
TGTGTAGGCGATGCTGGTAGTTACTATGGGAGGATTGTCTACTGGGGCCAGGGCACCCTGGTCACCGTCTCCTCAGA
GAGTACGAAGACCCCAGATCTCTTCCCCCTCGTCTCCTGTGGGCCCTCTCTTGATGAGAGCCTGGTGGCTGTGGGCT
GCCTAGCCCGGGACTTCCTACCCAA
>KY437259.1 Equus caballus clone 103 immunoglobulin mu heavy chain (IGHM) mRNA, partial cds
CTCTCCCTCACCTGCACTGTCTCTGGATTATCTTTGAGCAGTAATGCTGTAGGCTGGGTCCGCCAGGCTCCAGGAAA
AGGGCTGGAATATGTTGGTATGATAGGTAGAGGTGGAAGTGCAAACTACAACCCAGCCCTGAAGTCCCGAGCCAGCA
TCACCAAGGACACCTCAGAGAGCCAAGTTTATCTGACGCTGAACAGCCTGACAAGCGAGGACACGGCCGTCTATTAC
TGTGCAGGAGGCGCTACTACGATAGTGATTACCAATTATGGTCTAAGGTACTGGGGCCAGGGCATCCTGGTCACCGT
CTCCTCAGAGAGTACGATGACCCCAGATCTCTTCCCCCTCGTCTCCTGTGGGCCCTCTCTTGATGAGAGCCTGGTGG
CTGTGGGCTGCCTAGCCCGGGACTTCCTACCCAA
>KY437258.1 Equus caballus clone 102 immunoglobulin mu heavy chain (IGHM) mRNA, partial cds
CTGTCCCTCACCTGCACTGTCTCTGGATTCTCTTTGAGCAGTTATGTTGTGAACTGGGTCCGCCAGGCTCCAGGAAA
AGGGCTGGAATATATTGGTGCAAGCTATGATTCCGCAGACTACAACCCAGTCCTGAAGTCCCGAGCCAGCATCACCA
AGGACACCTCAAAGAGCCAAGTTTATCTGACGCTGAGCAGCCTGACAGGCGAGGACACGGCCGTCTATTACTGTATG
TCGGGGGTCTACTGGGGCCAGGGCACCCTGGTCACCGTCTCCTCAGAGAGTACGAAGACCCCAGATCTCTTCCCCCT
CGTCTCCTGTGGGCCCTCTCTTGATGAGAGCCTGGTGGCTGTGGGCTGCCTAGCCCGGGACTTCCTACCCAA
>KY437257.1 Equus caballus clone 101 immunoglobulin mu heavy chain (IGHM) mRNA, partial cds

CTCTCCCTCACCTGCACTGTCTCTGGATTATCTTTGAGCAGTAATGCTGTAGGCTGGGTCCGCCAGGCTCCAGGAAA
AGGGCTGGAATATGTTGGTGGTATAGCTAGTAGTGGAAGTGCAAACTACAACCCAGCCCTGAAGTCCCGAGCCAGCA
TCACCAAGGACACCTCAAAGAGCCAAGTTTATCTGACGCTGAACAGCCTGACAAGCGAGGACACGGCCGTCTATTAC
TGTGCAGGAGGCCTATATGGTAGTACTTTCTACCTCTCTTCGATTGCCTACTGGGGCCAGGGCACCCTGGTCACCGT
CTCCTCAGAGAGTACGATGACCCCAGATCTCTTCCCCCTCGTCTCCTGTGGGCCCTCTCTTGATGAGAGCCTGGTGG
CTGTGGGCTGCCTAGCCCGGGACTTCCTACCCAA
>KY437256.1 Equus caballus clone 100 immunoglobulin mu heavy chain (IGHM) mRNA, partial cds
CTCTCCCTCACCTGCACTGTCTCTGGATTATCTTTGAGCAGTAATGCTGTAGGCTGGGTCCGCCAGGCTCCAGGAAA
AGGGCTGGAATATGTTGGTGGTATAGCTAGTAGTGGAAGTGCAAACTACAACCCAGCCCTGAAGTCCCGAGCCAGCA
TCACCAAGGACACCTCAAAGAGCCAAGTTTATCTGACGCTGAACAGCCTGACAAGCGAGGACACGGCCGTCTATTAC
TGTGCAGGAGGCTTCGAAAACCCATTTATTCCGATTCTTACTGCTACACACAGCTACTGGGGCCAGGGCACCCTGGT
CACCGTCTCCTCAGAGAGTACGAAGACCCCAGATCTCTTCCCCCTCGTCTCCTGTGGGCCCTCTCTTGATGAGAGCC
TGGTGGCTGTGGGCTGCCTAGCCCGGGACTTCCTACCCAA
>KY437255.1 Equus caballus clone 99 immunoglobulin mu heavy chain (IGHM) mRNA, partial cds
CTCTCCCTCACCTGCACTGTCTCTGGATTATCTTTGAGCAGTAATACTGTAGGCTGGGTCCGCCAGGCTCCAGGAAA
AGGGCTGGAATATGTTGGTGGTATAACAGGCAGTGGAAGTGCAGCCTACAACCCAGCCCTGAAGTCCCGAGCCAGCA
TCACCAAGGACACCTCAAAGAGCCAAATTTATCTGACGCTGAACAGCCTGACAAGCGAGGACACGGCCGTCTATTAC
TGTCGTACGGGTGATTTTGCCTACTGGGGCCAGGGCACCCTGGTCACCGTCTCCTCAGAGAGTACGATGACCCCAGA
TCTCTTCCCCCTCGTCTCCTGTGGGCCCTCTCTTGATGAGAGCCTGGTGGCTGTGGGCTGCCTAGCCCGGGACTTCC
TACCCAA
>KY437254.1 Equus caballus clone 98 immunoglobulin mu heavy chain (IGHM) mRNA, partial cds
CTGTCCCTCACCTGCACTGTCTCTGGATTCTCTTTGAGCAGTTACGGTGTAGGCTGGGTCCGCCAGGCTCCAGGAAA
AGGGCTGGAATTTGTTGGTGGTATAGCTAGTAGTGGAAGTGCAAACTACAACCCAGCCCTGAAGTCCCGAGCCAGCA
TCACCAAGGACACCTCAAAGAGCCAAGTTTATCTGACGCTGAACAGCCTGACAAGCGAGGACACGGCCGTCTATTAC
TGTGCAGGAGGCTTCGAAAACCCATTTATTCCGATTCTTACTGCTACACACAGCTACTGGGGCCAGGGCACCCTGGT
CACCGTCTCCTCAGAGAGTACNAAGACCCCAGATCTCTTCCCCCTCGTCTCCTGTGGGCCCTCTCTTGATGAGAGCC
TGGTGGCTGTGGGCTGCCTAGCCCGGGACTTCCTACCCAA
>KY437253.1 Equus caballus clone 97 immunoglobulin mu heavy chain (IGHM) mRNA, partial cds
CTGTCCCTCAGCTGCACTGTCTCTGGATTCTCTTTGAGCAGTTACGGTGTAGGCTGGGTCCGCCAGGCTCCAGGAAA
AGGGCTGGAATGGGTTGGTGGTATAATAATTACTGGAACTACAATCTACAACCCAGCCCTGGAGTCCCGAGCCAGCA
TCACCAAGGACACCTCAAAGAACCAACTTTATCTGACGCTGAACAGCCTGACAAGCGAGGACACGGCCGTCTATTAC
TGTGCAGGACTAGATTATGGTAATGTCCTTGACTATTGGGGCCAGGGCACCCTGGTCACCGTCTCCTCAGAGAGTAC
GATGACCCCAGATCTCTTCCCCCTCGTCTCCTGTGGGCCCTCTCTTGATGAGAGCCTGGTGGCTGTGGGCTGCCTAG
CCCGGGACTTCCTACCCAA
>KY437252.1 Equus caballus clone 96 immunoglobulin mu heavy chain (IGHM) mRNA, partial cds
CTGTCCCTCACCTGCACTGTCTCTGGATTCTCTTTGAGCAGTTACGGTGTAGGCTGGGTCCGCCAGGCTCCAGGAAA
AGGGCTGGAATTTGTTGGTGGTATAGCTGGTAGTGGAAGTGCAAACTACAACCCAGCCCTGAAGTCCCGAGCCAGCA
TCACCAAGGACACCTCAAAGAGCCAAGTTTATCTGACGCTGAACAGCCTGACAGGCGAGGACACGGCCGTCTATTAC
TGTGCGAGATCGGAGGTTTCTTATGATAATTACTGGGCTCAGATTGGCTCTTGGGGCCAGGGCACCCTGGTCACCGT
CTCCTCAGAGAGTACGAAGACCCCAGATCTCTTCCCCCTCGTCTCCTGTGGNCCCTCTCTTGATGAGAGCCTGGTGG
CTGTGGGCTGCCTAGCCCGGGACTTCCTACCCAA
>KY437251.1 Equus caballus clone 95 immunoglobulin mu heavy chain (IGHM) mRNA, partial cds
CTCTCCCTCACCTGCACTGTCTCTGGATTATCTTTGAGCAGTTATGGTGTGGCCTGGGTCCGCCAGGCTCCAGGAAA
AGGGCTGGAATTTGTTGGTGGTATAGCTGGTAGTGGAAGTGCAAACTACAACCCAGCCCTGAAGTCCCGAGCCAGCA
TCACCAAGGACACCTCAAAGAGCCAAGTTTATCTGACGCTGAACAGCCTGACAGGCGAGGACACGGCCGTCTATTAC
TGTGCAGGAGGGCCATATCGGAACTACGGTTATGGTTATGCTACGGAGTTTGGCTACTGGGGCCAGGGCACCCTGGT
CACCGTCTCCTCAGAGAGTACGAAGACCCCAGATCTCTTCCCCCTCGTCTCCTGTGGGCCCTCTCTTGATGAGAGCC
TGGTGGCTGTGGGCTGCCTAGCCCGGGACTTCCTACCCAA

>KY437250.1 Equus caballus clone 94 immunoglobulin mu heavy chain (IGHM) mRNA, partial cds
CTGTCCCTCACCTGCACTGTCTCTGGATTCTCTTTGAGCAGTGACGGTGTAGGCTGGGTCCGCCAGGCTCCAGGAAA
AGGGCTGGAATTTGTTGGTGGTATAGTTAGTAGTGGAAGTGCAAACTACAACCCAGCCCTGAAGTCCCGAGCCAGCA
TCACCAAGGACACCTCAAAGAGCCAAGTTTATCTGACGCTGAACAGCCTGACAGGCGAGGACACGGCCGTCTATTAC
TGTGCGACCTATAGGGTTACGGTGGTGATTACTATGGATGTCTATTTTGACTACTGGGGCCAGGGCACCCTGGTCAC
CGTCTCCTCAGAGAGTACNAAGACCCCAGATCTCTTCCCCCTCGTCTCCTGTGGGCCCTCTCTTGATGAGAGCCTGG
TGGCTGTGGGCTGCCTAGCCCGGGACTTCCTACCCAA
>KY437249.1 Equus caballus clone 93 immunoglobulin mu heavy chain (IGHM) mRNA, partial cds
CTGTCCCTCACCTGCACTGTCTCTGGATTCTCTTTGAGCAGTTACGGTGTAGGCTGGGTCCGCCAGGCTCCAGGAAA
AGGGCTGGAATTTGTTGGTGGTATAGCTAGTAGTGGAAGTGCAAACTACAACCCAGCCCTGAAGTCCCGAGCCAGCA
TCACCAAGGACACCTCAAAGAGCCAAGTTTATCTGACGCTGAACAGCCTGACAGGCGAGGACACGGCCGTCTATTAC
TGTGCGAGATTTTATGGTTATGGTTCTTCTTACTTAGACTGGGGCCAGGGCACCCTGGTCACCGTCTCCTCAGAGAG
TACGATGACCCCAGATCTCTTCCCCCTCGTCTCCTGTGGGCCCTCTCTTGATGAGAGCCTGGTGGCTGTGGGCTGCC
TAGCCCGGGACTTCCTACCCAA
>KY437248.1 Equus caballus clone 92 immunoglobulin mu heavy chain (IGHM) mRNA, partial cds
CTCTCCCTCACCTGCACTGTCTCTGGATTATCTTTGAGCAGTTATGGTGTGGGCTGGGTCCGCCAGGCTCCAGGAAA
AGGGCTGGAATTTGTTGGTAGTATAGCTAGTAGTGGAAGTGCAAACTACAACCCAGCCCTGAAGTCCCGAGCCAGCA
TCACCAAGGACACCTCAAAGAGCCAAGTTTATCTGACGCTGAACAGCCTGACAAGCGAGGACACGGCCGTCTATTAC
TGTGCAGGAGGCGTTAATGATTATAGTTATGGTAGTTACTATGTGAGCCTTCGCTACTGGGGCCAGGGCACCCTGGT
CACCGTCTCCTCAGAGAGTACGAAGACCCCAGATCTCTTCCCCCTCGTCTCCTGTGGGCCCTCTCTTGATGAGAGCC
TGGTGGCTGTGGGCTGCCTAGCCCGGGACTTCCTACCCAA
>KY437247.1 Equus caballus clone 91 immunoglobulin mu heavy chain (IGHM) mRNA, partial cds
CTCTCCCTCACCTGCACTGTCTCTGGATTATCTTTGAGCAGTTATGGTGTGGGCTGGGTCCGCCAGGCTCCAGGAAA
AGGGCTGGAATTTGTTGGTGGTATATCTAGTAGTGGAAGTGCAAACTACAACCCAGCCCTGAAGTCCCGAGCCAGCA
TCACCAAGGACACCTCAAAGAGCCAAGTTTATCTGACGCTGAACAGCCTGACAAGCGAGGACACGGCCGTCTATTAC
TGTGCAGGGAGCTTTGACATTGGATGGAGTGTTGGTCCTTACTCCCTTGGCTACTGGGGCCAGGGCACCCTGGTCAC
CGTCTCCTCAGAGAGTACGAAGACCCCAGATCTCTTCCCCCTCGTCTCCTGTGGGCCCTCTCTTGATGAGAGCCTGG
TGGCTGTGGGCTGCCTAGCCCGGGACTTCCTACCCAA
>KY437246.1 Equus caballus clone 90 immunoglobulin mu heavy chain (IGHM) mRNA, partial cds
CTCTCCCTCACCTGCACTGTCTCTGGATTATCTTTGAGCAGTAATGCTGTAGGCTGGGTCCGCCAGGCTCCAGGAAA
AGGGCTGGAATATGTTGGTCTTATAGCTAGTAGTGGAAGTGCAAACTACAACCCAGCCCTGAAGTCCCGAGCCAGTA
TCACCAAGGACACCTCAAAGAGCCAAGTTTATCTGACGCTGAACAGCCTGACAAGCGAGGACACGTCCGTCTATTAC
TGCGCAGGAGGTCTCCTAAGAGATAATGGGGGTGCTTACTGGTGGGGCCAGGGCACCCTGGTCACCGTCTCCTCAGA
GAGTACGAAGACCCCAGATCTCTTCCCCCTCGTCTCCTGTGGGCCCTCTCTTGATGAGAGCCTGGTGGCTGTGGGCT
GCCTAGCCCGGGACTTCCTACCCAA
>KY437245.1 Equus caballus clone 89 immunoglobulin mu heavy chain (IGHM) mRNA, partial cds
CTGTCCCTCACCTGCACTGTCTCTGGATTATCTTTGAGCAGTAGTGCTGTAGCGTGGGTCCGCCAGGCTCCAGGAAA
AGGGCTGGAGTGGGTTGGTGTCGTATGGGGTAGTGAAGGTATATACGTCAACCCAGCCCTGAAGTCCCGAGCCAGCA
TCACCAAGGACACCTCAAAGAGCCAAGTTTATCTGACGCTGAACAGCCTGACAGGCGAAGACACAGCCGTCTATTAC
TGTGAAGGATTTATTTATAGCAGCGATGCAATGGCTATAAACTACTGGGGCCAGGGCATCCTGGTCACCGTCTCCTC
AGAGAGTACGATGACCCCAGATCTCTTCCCCCTCGTCTCCTGTGGGCCCTCTCTTGATGAGAGCCTGGTGGCTGTGG
GCTGCCTAGCCCGGGACTTCCTACCCAA
>KY437244.1 Equus caballus clone 88 immunoglobulin mu heavy chain (IGHM) mRNA, partial cds
CTCTCCCTCACCTGCACTGTCTCTGGATTATCTTTGAGCAGTAATGCTGTAGGCTGGGTCCGCCAGGCTCCAGGAAA
AGGGCTGGAATATGTTGGTGGTATAGAGGCAAGTGGAAGTGCAAACTACAACCCAGCCCTGAATTCCCGAGCCAGCA
TCACCAGGGACACCTCAAAGAGCCAAGTTTATCTGACGCTGAACAGTCTGACAAGCGAGGACACGGCCGTCTATTAC
TGTGCAGATTACTATCTTTATGCTATAAACTATTGGGGCCAGGGCACCCTGGTCACCGTCTCCTCAGAGAGTACGAT
GACCCCAGATCTCTTCCCCCTCGTCTCCTGTGGGCCCTCTCTTGATGAGAGCCTGGTGGCTGTGGGCTGCCTAGCCC
GGGACTTCCTACCCAA

>KY437243.1 Equus caballus clone 87 immunoglobulin mu heavy chain (IGHM) mRNA, partial cds
CTGTCCCTCACCTGCACTGTCTCTGGATTCTCTTTGAGCAGTTACGGTGTGGGCTGGGTCCGCCAGGCTCCAGGAAA
AGGGCTGGAATTTGTTGGTGGTATAGCTAGTAGTGGAAGTGCAAACTACAACCCAGCCCTGAAGTCCCGAGCCAGCA
TCACCAAGGACACCTCAAAGAGCCAAGTTTATCTGACGCTGAACAGCCTGACAAGCGAGGACACGGCCGTCTATTAC
TGTGCAGGAGACAATTACTATGGTGGTAGTTCCTGGTACTCCGTCTCTAGCTTTGACTACTGGGGCCAGGGCACCCT
GGTCACCGTCTCCTCAGAGAGTACGAAGACCCCAGATCTCTTCCCCCTCGTCTCCTGTGGGCCCTCTCTTGATGAGA
GCCTGGTGGCTGTGGGCTGCCTAGCCCGGGACTTCCTACCCAA
>KY437242.1 Equus caballus clone 86 immunoglobulin mu heavy chain (IGHM) mRNA, partial cds
CTGTCCCTCACCTGCACTGTCTCTGGATTCTCTTTGAGCAGTTACGGTGTAGGCTGGGTCCGCCAGGCTCCAGGAAA
AGGGCTGGAATATGTTGGTGGTATAGCTAGTAGTGGAAGTGCAAACTACAACCCAGCCCTGAAGTCCCGAGCCAGCA
TCACCAAGGACACCTCAAAGAGCCAAGTTTATCTGACGCTGAACAGCCTGACAAGCGAGGACACGGCCGTCTATTAC
TGTGCAGGACGCCACCATCCTTGGGACGATGACTATGGTGATACTTTCTACCAGGGAAAAACGGCATTTAACTATTT
TGGCTACTGGGGCCAGGGCACCCTGGTCACCGTCTCCTCAGAGAGTACGATGACCCCAGATCTCTTCCCCCTCGTCT
CCTGTGGGCCCTCTCTTGATGAGAGCCTGGTGGCTGTGGGCTGCCTAGCCCGGGACTTCCTACCCAA
>KY437241.1 Equus caballus clone 85 immunoglobulin mu heavy chain (IGHM) mRNA, partial cds
CTGTCCCTCACCTGCACTGTCTCTGGATTATCTTTGAGCAGTAATGCTGTAGGCTGGGTCCGCCAGGCTCCAGGAAA
AGGGCTGGAGTGGGTTGGTGTTATATGGGGTAGTGAAAGTACATACTACAACCCAGCCCTGAAGTCCCGAGCCAGCA
TCACCAAGGACACCTCAAAGAGCCAAGTTTATCTGGCGCTGAACAGCCTGACAAGCGAGGACACGGCCGTCTATTAC
TGTGCCACTGGTTACTACGGTGAAAATGGCTACTGGGGCCAGGGCACCCTGGTCACCGTCTCCTCAGAGAGTACGAT
GACCCCAGATCTCTTCCCCCTCGTCTCCTGTGGGCCCTCTCTTGATGAGAGCCTGGTGGCTGTGGGCTGCCTAGCCC
GGGACTTCCTACCCAA
>KY437240.1 Equus caballus clone 84 immunoglobulin mu heavy chain (IGHM) mRNA, partial cds
CTGTCCCTCACCTGCACTGTCTCTGGATTCTCTTTGAGCAGTGACGGTGTAGGCTGGGTCCGCCAGGCTCCAGGAAA
AGGGCTGGAATTTGTTGGTGGTATAGCTACTAGTGGAAGTGCAAACTACAACCCAGCCCTGAAGTCCCGAGCCAGCA
TCACCAAGGACACCTCAAAGAGCCAAGTTTATCTGACGCTGAACAGCCTGACGGGCGAAGACACGGCCGTCTATTAC
TGTGCAGGATCCCGGCATATGGTTACTATAGTAGGAGTTGCTCCTCCGCCTACCGCTTATGGTATAAACTACTGGGG
CCAGGGCATCCTGGTCACCGTCTCCTCAGAGAGTACNAAGACCCCAGATCTCTTCCCCCTCGTCTCCTGTGGGCCCT
CTCTTGATGAGAGCCTGGTGGCTGTGGGCTGCCTAGCCCGGGACTTCCTACCCAA
>KY437239.1 Equus caballus clone 83 immunoglobulin mu heavy chain (IGHM) mRNA, partial cds
CTGTCCCTCACCTGCACTGTCTCTGGATTCTCTTTGAGCAGTTACGGTGTAGGCTGGGTCCGCCAGGCTCCAGGAAA
AGGGCTGGAATTTGTTGGTGGTATCGCTAGTAGTGGAAGTGCAAACTACAACCCAGCCCTGAAGTCCCGAGCCAGCA
TCACGAAGGACACCTCAAAGAGCCAAGTTTATCTGACGCTGAACAGCCTGACAGGCGAGGACACGGCCGTCTATTAC
TGTGCGAGATCTCACTATGGTGGTAGTTCCTGGTACTCGTCGGATTATGGTATAAACTACTGGGGCCAGGGCATCCT
GGTCACCGTCTCCTCAGAGAGTACNATGACCCCAGATCTCTTCCCCCTCGTCTCCTGTGGGCCCTCTCTTGATGAGA
GCCTGGTGGCTGTGGGCTGCCTAGCCCGGGACTTCCTACCCAA
>KY437238.1 Equus caballus clone 82 immunoglobulin mu heavy chain (IGHM) mRNA, partial cds
CTGTCCCTCACCTGCACTGTCTCTGGATTATCTTTGAGCAGTAATGCTGTAGGCTGGGTCCGCCAGGCTCCAGGAAA
AGGGCTGGAGTGGGTTGGTGTTATATATGGTAGTGAAAGTACATACTACAACCCAGCCCTGAAGTCCCGAGCCAGCA
TCACCAAGGACACCTCAAAGAGCCAAGTCTATCTGACGCTGAACAGCCTGACAGGCGAAGACACGGCCGTCTATTAC
TGTGCAGGAACAGCGGATTCGTATGGTAGCTACTGGGGCCAGGGCACCCTGGTCACCGTCTCCTCAGAGAGTACGAA
GACCCCAGATCTCTTCCCCCTCGTCTCCTGTGGGCCCTCTCTTGATGAGAGCCTGGTGGCTGTGGGCTGCCTAGCCC
GGGACTTCCTACCCAA
>KY437237.1 Equus caballus clone 81 immunoglobulin mu heavy chain (IGHM) mRNA, partial cds
CTGTCCCTCACCTGCACTGTCTCTGGATTCTCTTTGAGCAGTTACGGTGTAGGCTGGGTCCGCCAGGCTCCAGGAAA
AGGGCTGGAATTTGTTGGTGGTATAACTAGTAGTCGAAGTGCAAACTACAACCCAGCCCTGAAGTCCCGAGCCAGCA
TCACCAAGGACATCTCAAACAGCCAAGTTTATCTGACGCTGAACAGCCTGACAGGCGAGGACACGGCCGTCTATTAC
TGTGCGAGAATTGGCCAATATTATTATGGTATGCACTACTGGGGCCAGGGCATCCTGGTCACCGTCTCCTCAGAGAG

TACGAAGACCCCAGATCTCTTCCCCCTCGTCTCCTGTGGGCCCTCTCTTGATGAGAGCCTGGTGGCTGTGGGCTGCC
TAGCCCGGGACTTCCTACCCAA
>KY437236.1 Equus caballus clone 80 immunoglobulin mu heavy chain (IGHM) mRNA, partial cds
CTGTCCCTCACCTGCACTGTCTCCGGATTATCTTTGAGCACTAATTCTGTAGGCTGGGTCCGCCAGGCTCCAGGAAA
AGGGCTGGAGTGGATTGTTTATCTTCATGGTAGTGCGAGTGCACACCGCAACCCAGCCCTCGAGTCCCGAGCCAGCA
TCGCCAAGGACACCTCAAAGAGCCAAGTTTATCTGACGCTGAACAGCCTGACAGGCGAAGACACGGCCGTCTATTAC
TGTGTAGGATACATGGGTGGTGTTATGGGTGGTAGTTCCTGGTTCTATTTTGCCTACTGGGGCCAGGGCACCCTGGT
CACCGTCTCCTCAGAGAGTACGAAGACCCCAGATCTCTTCCCCCTCGTCTCCTGTGGGCCCTCTCTTGATGAGAGCC
TGGTGGCTGTGGGCTGCCTAGCCCGGGACTTCCTACCCAA
>KY437235.1 Equus caballus clone 79 immunoglobulin mu heavy chain (IGHM) mRNA, partial cds
CTCTCCCTCACCTGCACTGTCTCTGGATTATCTTTGAGCAGTAATGCTGTAGGCTGGGTCCGCCAGGCTCCAGGGAA
AGGGCTGGAATATGTTGGTGGTATAGGTAGTAGTGGAAGTGTAATTTACAACCCAGCCCTGAAGTCCCGAGCCAGCA
TCACCAAGGACACCTCAAAGAGCCAAGTTTATCTGACGCTGAACAGCCTGACAAGCGAGGACACGGCCGTCTATTAC
TGTGCAGGAGGCGAATATGGTTACTATGCTGGTAGTTACTATCCAGGCTTTGGCTACTGGGGCCAGGGCACCCTGGT
CACCGTCTCCTCAGAGAGTACGAAGACCCCAGATCTCTTCCCCCTCGTCTCCTGTGGGCCCTCTCTTGATGAGAGCC
TGGTGGCTGTGGGCTGCCTAGCCCGGGACTTCCTACCCAA
>KY437234.1 Equus caballus clone 78 immunoglobulin mu heavy chain (IGHM) mRNA, partial cds
CTCTCCCTCACCTGCACTGTCTCTGGATTATCTTTGAGCAGTTATGGTGTGGGCTGGGTCCGCCAGGCTCCAGGAAA
AGGGCTGGAATACGTTGCCGGTATAAGTAAAAGTGGAAGTGCAAAATACAATTTAGCCCTGAAGTCCCGAGCCAGCA
TCACCAAGGACACCTCAAAGAGCCAAGTTTATCTGACGCTGAACAGCCTGACAGGCGAGGACACGGCCGTCTATTAC
TGTGCGAGATTTGGAGGGGGCTACTGGGGCCAGGGCACCCTGGTCACCGTCTCCTCAGAGAGTACGAAGACCCCAGA
TCTCTTCCCCCTCGTCTCCTGTGGGCCCTCTCTTGATGAGAGCCTGGTGGCTGTGGGCTGCCTAGCCCGGGACTTCC
TACCCAA
>KY437233.1 Equus caballus clone 77 immunoglobulin mu heavy chain (IGHM) mRNA, partial cds
CTCTCCCTCACCTGCACTGTCTCTGGATTATCTTTGAGCAGTTATAATGTAGGCTGGGTCCGCCAGGCTCCAGGAAA
AGGGCTGGAATATGTTGGTGCTATACTTGCCAGTGCAAGTGCAAACTACAACCCAGCCCTGAAGTCCCGAGCCAGCA
TCACCAAGGACACCTCAAAGAGCCAAGTTTATCTGACGCTGAACAGCCTGACAGGCGAGGACACGGCCGTCTATTAC
TGTGCGAGTGATTACCTTGGATATAGCAGCTATTTTGCCCTGACCTTTGGCTACTGGGGCCAGGGCACCCTGGTCAC
CGTCTCCTCAGAGAGTACGATGACCCCAGATCTCTTCCCCCTCGTCTCCTGTGGGCCCTCTCTTGATGAGAGCCTGG
TGGCTGTGGGCTGCCTAGCCCGGGACTTCCTACCCAA
>KY437232.1 Equus caballus clone 76 immunoglobulin mu heavy chain (IGHM) mRNA, partial cds
CTCTCCCTCACCTGCACTGTCTCTGGATTATCTTTGAGCAGTTATCTTGTGAGCTGGGTCCGCCAGGCTCCAGGAAA
AGGGCTGGAGTATGTCGGTGGTGCATATGGTAGTGCAAGTGCAATGTACAACCCAGCCCTGAAGTCCCGAGCCAGCA
TCACCAAGGACGCCTCAAAGAGCCAAGTTTATCTGACGCTGAACAGCCTGACAGGCGAGGACACGGCCGTCTATTGG
TGTGCGATTTGGAGGATGGGTGCGGCTATAAACTATTGGGGCCAGGGCACCCTGGTCACCGTCTCCTCAGAGAGTAC
GATGACCCCAGATCTCTTCCCCCTCGTCTCCTGTGGGCCCTCTCTTGATGAGAGCCTGGTGGCTGTGGGCTGCCTAG
CCCGGGACTTCCTACCCAA
>KY437231.1 Equus caballus clone 75 immunoglobulin mu heavy chain (IGHM) mRNA, partial cds
CTCTCCCTCACCTGCACTGTCTCTGGATTATCTTTGAGCAGTTATCTTGTGAGCTGGGTCCGCCAGGCTCCAGGAAA
AGGGCTGGAATATGTCGGTGGTGCATATGGTAGTGCAAGTGCAATGTACAACCCAGCCCTGAAGTCCCGAGCCAGCA
TCACCAAGGACGCCTCAAAGAGCCAAGTTTATCTGACGCTGTACAGCCTGACAGGCGAGGACACGGCCGTCTATTGG
TGTGCGATTTGGAGGATGGGTGCGGCTATAAACTATTGGGGCCAGGGCATCCTGGTCACCGTCTCCTCAGAGAGTAC
GATGACCCCAGATCTCTTCCCCCTCGTCTCCTGTGGGCCCTCTCTTGATGAGAGCCTGGTGGCTGTGGGCTGCCTAG
CCCGGGACTTCCTACCCAA
>KY437230.1 Equus caballus clone 74 immunoglobulin mu heavy chain (IGHM) mRNA, partial cds
CTCTCCCTCACCTGCACTGTCTCTGGATTATCTTTGAGTAGTATTGGTGTGGGCTGGGTCCGCCAGGCTCCAGGAAA
AGGGCTGGAATTTGTTGGTAGAATACCTAGTAGTGGAAGTGCAAACTACAACCCAGCCCTGAAGTCCCGAGCCAGCA
TCACCAAGGACACCTCAAAGAGCCAAGTTTATCTGACGCTGAACAGCCTGACAAGCGAGGACACGGCCGTCTATTAC
TGTGCAGGAGGCCTGGGTAGTTGGGCTTATGATAGAGACTACTGGGGCCAGGGCACCCTGGTCACCGTCTCCTCAGA

GAGTACGATGACCCCAGATCTCTTCCCCCTCGTCTCCTGTGGGCCCTCTCTTGATGAGAGCCTGGTGGCTGTGGGCT
GCCTAGCCCGGGACTTCCTACCCAA
>KY437229.1 Equus caballus clone 73 immunoglobulin mu heavy chain (IGHM) mRNA, partial cds
CTCTCCCTCACCTGCACTGTCTCTGGATTATCTTGGAGCAGTTATCCTGTAGGCTGGGTCCGCCAGGCTCCAGGAAA
AGGGCTGGAATACGTTGGTGCTATATATGGTAGTGCAAGTGCAAACTACAACCCAGCCCTGAAGTCCCGAGCCAGCA
TCACCAAGGACACCTCAAAGAGCCAAGTTTATCTGACGCTGAACAGCCTGACAAGCGAGGACACGGCCGTCTATTAC
TGTGCAGGAGCCGATGAATTGAATCATGGTACAAACTATGCATTTGGCTACTGGGGCCAGGGCACCCTGGTCACCGT
CTCCTCAGAGAGTACGATGACCCCAGATCTCTTCCCCCTCGTCTCCTGTGGGCCCTCTCTTGATGAGAGCCTGGTGG
CTGTGGGCTGCCTAGCCCGGGACTTCCTACCCAA
>KY437228.1 Equus caballus clone 72 immunoglobulin mu heavy chain (IGHM) mRNA, partial cds
CTCTCCCTCACCTGCACTGTCTCTGGATTATCTTTGAGCAGTAATGCTGTAGGCTGGGTCCGCCAGGCTCCAGGAAA
AGGGCTGGAATACGTTGGTGCTATATATGGTAGTGCAAGTGCAAACTACAACCCAGCCCTGAAGTCCCGAGCCAGCA
TCACCAAGGACACCTCAAAGAGCCAAGTTTATCTGACGCTGAACAGCCTGACAAGCGAGGACACGGCCGTCTATTAC
TGTGCAGGAGGTTACTATGGTAATTCGTACAGTCGTTTGTATGGTGCCTTTGGCGACTGGGGCCAGGGCATCCTGGT
CACCGTCTCCTCAGAGAGTACGATGACCCCAGATCTCTTCCCCCTCGTCTCCTGTGGGCCCTCTCTTGATGAGAGCC
TGGTGGCTGTGGGCTGCCTAGCCCGGGACTTCCTACCCAA
>KY437227.1 Equus caballus clone 71 immunoglobulin mu heavy chain (IGHM) mRNA, partial cds
CTCTCCCTCACCTGCACTGTCTCTGGATTCTCTTTGAGCAGTAATGCTGTAGGCTGGGTCCGCCAGGCTCCAGGAAA
AGGGCTGGAATACGTTGGTGCTATATATGGTAGTGCAAGTGCAAACTACAACCCAGCCCTGAAGTCCCGAGCCAGCA
TCACCAAGGACACCTCAAAGAGCCAAGTTTATCTGACGCTGAACAGCCTGACAAGCGAGGACACGGCCGTCTATTAC
TGTGCAGGAGGTGGTGTTAGCTACGGGGCTGATCTTTACGGTTACTGGGGCCAGGGCACCCTGGTCACCGTCTCCTC
AGAGAGTACGATGACCCCAGATCTCTTCCCCCTCGTCTCCTGTGGGCCCTCTCTTGATGAGAGCCTGGTGGCTGTGG
GCTGCCTAGCCCGGGACTTCCTACCCAA
>KY437226.1 Equus caballus clone 70 immunoglobulin mu heavy chain (IGHM) mRNA, partial cds
CTCTCCCTCACCTGCACTGTCTCTGGATTATCTTTGAGCAGTAATGCTGTAGGCTGGGTCCGCCAGGCTCCAGGAAA
AGGGCTGGAATACGTTGGTGCTATATATGGTAGTGCAAGTGCAAACTACAACCCAGCCCTGAAGTCCCGAGCCAGCA
TCACCAAGGACACCTCAAAGAGCCAAGTTTATCTGACGCTGAACAGCCTGACAAGCGAGGACACGGCCGTCTATTAC
TGTGCAGGATTGAGTCATGGTAGTCCCTCCTGGGGCCAGGGCACCCTGGTCACCGTCTCCTCAGAGAGTACGATGAC
CCCAGATCTCTTCCCCCTCGTCTCCTGTGGGCCCTCTCTTGATGAGAGCCTGGTGGCTGTGGGCTGCCTAGCCCGGG
ACTTCCTACCCAA
>KY437225.1 Equus caballus clone 69 immunoglobulin mu heavy chain (IGHM) mRNA, partial cds
CTCTCCCTCACCTGCACTGTCTCTGGATTATCTTTGAGCAGTTATGATGTAGGCTGGGTCCGCCAGGCTTCGGGAAA
AGGGCTGGAATATGTTGGTGTTTTATATGGTAGTGCAAGTGCAAAGTACAACCCAGCCCTGAAGTCCCGAGCCAGCA
TCACCAAGGACACCTCAAAGAGCCAAGTTTCTCTGACGCTGAACAGCCTGACAGGCGAGGACACGGCCGTCTATTAC
TGTGCAGGAGGCCTCGATTACTATGGTGGTAGTTCCTGGTACTGGGACTGGGGCCAGGGCACCCTGGTCACCGTCTC
CTCAGAGAGTACGATGACCCCAGATCTCTTCCCCCTCGTCTCCTGTGGGCCCTCTCTTGATGAGAGCCTGGTGGCTG
GGGGCTGCCTAGCCCGGGACTTCCTACCCAA
>KY437224.1 Equus caballus clone 68 immunoglobulin mu heavy chain (IGHM) mRNA, partial cds
CTCTCCCTCACCTGCACTGTCTCTGGATTATCTTTGAGCAGTAATGCTGTAGGCTGGGTCCGCCAGGCTCCAGGAAA
AGGGCTGGAATTTGTTGGTGCTATATATGGTAGTGCAAGTGCAAACTACAACCCAGCCCTGAAGTCCCGAGCCAGCA
TCACCAAGGACACCTCAAAGAGTCAAGTTTATCTGACGCTGAACAGCCTGACAAGCGAGGACACGGCCGTCTATTAC
TGTGCAGGAGGCAGAATAGTTCCTGGTACTCCGAGTGTTGGCTACTGGGTCCAGGGCACCCTGGTCACCGTCTCCTC
AGAGAGTACGATGACCCCAGATCTCTTCCCCCTCGTCTCCTGTGGGCCCTCTCTTGATGAGAGCCTGGTGGCTGTGG
GCTGCCTAGCCCGGGACTTCCTACCCAA
>KY437223.1 Equus caballus clone 67 immunoglobulin mu heavy chain (IGHM) mRNA, partial cds
CTCTCCCTCACCTGCACTGTCTCTGGATTCTCTTTGAGCAGTGTTGCTGTAGGCTGGGTCCGCCAGGCTCCAGGAAA
AGGGCTGGAATATGTTGGTTATATAGCTAGTAGTGGAAGTGCAAACTACAACCCAGCCCTGAAGTCCCGAGCCAGCA
TCACCAAGGACACCTCAAAGAGCCAAGTTTATCTGACGCTGAACAGCCTGACAAGCGAGGACACGGCCGTCTATTAC

TGCGCAGGAGGCTGGTACGCTTATGGTTATAATCCCGGCATGATTGGATACTGGGGCCAGGGCACCCTGGTCACCGT
CTCCTCAGAGAGTACGATGACCCCAGATCTCTTCCCCCTCGTCTCCTGTGGGCCCTCTCTTGATGAGAGCCTGGTGG
CTGTGGGCTGCCTAGCCCGGGACTTCCTACCCAA
>KY437222.1 Equus caballus clone 66 immunoglobulin mu heavy chain (IGHM) mRNA, partial cds
CTCTCCCTCACCTGCACTGTCTCTGGATTATCTGTGAGCAGTAATGCTGTAGGCTGGGTCCGCCAGGCTCCAGGAAA
AGGGCTGGAATACGTTGGTGCTATATATGGTAGTGGAAGTGCAAACTACAACTCAGCCCTGAAGTCCCGAGCCAGCA
TCACCAAGGACACCTCAAAGAGCCAAGTTTATCTGACGCTGAACAGCCTGACAAGCGAGGACACGGCCGTCTATTAC
TGTGCAGGAGGCCCTGAGGGTTTATGGGGCCAGGGCATCCTGGTCACCGTCTCCTCAGAGAGTACGATGACCCCAGA
TCTCTTCCCCCTCGTCTCCTGTGGGCCCTCTCTTGATGAGAGCCTGGTGGCTGTGGGCTGCCTAGCCCGGGACTTCC
TACCCAA
>KY437221.1 Equus caballus clone 65 immunoglobulin mu heavy chain (IGHM) mRNA, partial cds
CTCTCCCTCACCTGCACTGTCTCTGGATTCTCTTTGAGCAGTTACGGTGTAGGCTGGGTCCGCCAGGCTCCAGGAAA
AGGGCTGGAATTTGTTGGTGGTATAGCTAGTAGTGGAAGTGCAAACTACAACCCAGCCCTGAAGTCCCGAGCCAGCA
TCACCAAGGACACCTCAAAGAGCCAAGTTTATCTGACGCTGAACAGCCTGACAAGCGAGGACACGGCCGTCTATTAC
TGTGCAGGAGGAATTGGCTACTGGGGCCAGGGCACCCTGGTCACCGTCTCCTCAGAGAGTACGATGACCCCAGATCT
CTTCCCCCTCGTCTCCTGTGGGCCCTCTCTTGATGAGAGCCTGGTGGCTGTGGGCTGCCTAGCCCGGGACTTCCTAC
CCAA
>KY437220.1 Equus caballus clone 64 immunoglobulin mu heavy chain (IGHM) mRNA, partial cds
CTCTCCCTCACCTGCACTGTCTCTGGATTCTCTTTGAGCAGTTACGGTGTAGGCTGGGTCCGCCAGGCTCCAGGAAA
AGGGCTGGAATTTGTTGGTGTTATAGATAAGAGTGGAAGTGCAAACTACAACCCAGCCCTGAAGTCCCGAGCCAGCA
TCACCAAGGACACCTCAAAGAGCCAAGTTTATCTGACGCTGAACAGCCTGACAAGCGAGGACACGGCCGTCTATTAC
TGTGCAGGAGTGGACTACGGTTATGGTTATCATTATGGTATAAACTACTGGGGCCAGGGCATCCTGGTCACCGTCTC
CTCAGAGAGTACGATGACCCCAGATCTCTTCCCCCTCGTCTCCTGTGGGCCCTCTCTTGATGAGAGCCTGGTGGCTG
TGGGCTGCCTAGCCCGGGACTTCCTACCCAA
>KY437219.1 Equus caballus clone 63 immunoglobulin mu heavy chain (IGHM) mRNA, partial cds
CTGTCCCTCACCTGCACTGTCTCTGGATTCTCGTTGAGCAGTTACGGTGTAAGCTGGGTCCGCCAGGCTCCAGGAAA
AGGGCTGGAATTTGTTGGTGGTATAGATATGACTGGAAGAGCAATTTACAACCCAGCCCTGAAGTCCCGAAGCAGCA
TCACCAAGGACACCTCAAAGAGCCAAGTTTATCTGACGCTGAACAGCCTGACAAGCGAGGACACGGCCGTCTATTAC
TGTGCAGGAGGCGACTACGGTTACTGGGGCCAGGGCATCCTGGTCACCGTCTCCTCAGAGAGTACGATGACCCCAGA
TCTCTTCCCCCTCGTCTCCTGTGGGCCCTCTCTTGATGAGAGCCTGGTGGCTGTGGGCTGCCTAGCCCGGGACTTCC
TACCCAA
>KY437218.1 Equus caballus clone 62 immunoglobulin mu heavy chain (IGHM) mRNA, partial cds
CTCTCCCTCACCTGCACTGTCTCTGGATTCTCTTTGAGCAGTTACGGTGTAGGCTGGGTCCGCCAGGCTCCAGGAAA
AGGGCTGGAATTTGTTGGTGGTATAGCTATAAGTGGAAGTGCAAACTACAACCCAGCCCTGAAGTCCCGAGCCAGCA
TCACCAAGGACACCTCAAAGAGCCAAGTTTATCTGACGCTGAACAGCCTGACAAGCGAGGACACGGCCGTCTATTAC
TGTGCAGGAGGGGATGGTTACTATGGTAGTTACTACAGTACATACTGGTATTATGGTATAAACTACTGGGGCCAGGG
CATCCTGGTCACCGTCTCCTCAGAGAGTACGATGACCCCAGATCTCTTCCCCCTCGTCTCCTGTGGGCCCTCTCTTG
ATGAGAGCCTGGTGGCTGTGGGCTGCCTAGCCCGGGACTTCCTACCCAA
>KY437217.1 Equus caballus clone 61 immunoglobulin mu heavy chain (IGHM) mRNA, partial cds
CTGTCCCTCACCTGCACTGTCTCTGGATTCTCTTTGAGCAGTTACGGTGTAGGCTGGGTCCGCCAGGCTCCAGGAAA
AGGGCTGGAATATGTTGGTGGTATAGTTAGTAGTGGAAGTGCAAACTACAACCCAGCCCTGAAGTCCCGAGCCAGCA
TCACCAAGGACACCTCAAAGAGCCAAGTTTATCTGACGCTGAACAGCCTGACAGGCGAGGACACGGCCGTCTATTAC
TGTGCGAGATCGTACTATGGTTACTATGGTAGTGGATACAAAAGTGACATTCTAAACTACTGGGGCCAGGGCATCCT
GGTCACCGTCTCCTCAGAGAGTACGATGACCCCAGATCTCTTCCCCCTCGTCTCCTGTGGGCCCTCTCTTGATGAGA
GCCTGGTGGCTGTGGGCTGCCTAGCCCGGGACTTCCTACCCAA
>KY437216.1 Equus caballus clone 60 immunoglobulin mu heavy chain (IGHM) mRNA, partial cds
CTCTCCCTCACCTGCACTGTCTCTGGATTATCTTTGAGCAGTAATAGTGTAGGCTGGGTCCGCCAGGCTCCAGGAAA
AGGGCTGGAATATGTTGGTGGTATAGCTAGTAGTGGAAGTGCAAACTACAACCCAGCCCTGAAGTCCCAAGCCAGCA
TCACCAAGGACACCTCAAAGAGCCAAGTTTATCTGACGCTGAACAGCCTGACAAGCGAGGACACGGCCGTCTATTAC

TGTGCAGGAATGAGGTATAGCAGCTATGATCAGTACTACTGGGGCCAGGGCACCCTGGTCACCGTCTCCTCAGAGAG
TACGATGACCCCAGATCTCTTCCCCCTCGTCTCCTGTGGGCCCTCTCTTGATGAGAGCCTGGTGGCTGTGGGCTGCC
TAGCCCGGGACTTCCTACCCAA
>KY437215.1 Equus caballus clone 59 immunoglobulin mu heavy chain (IGHM) mRNA, partial cds
CTCTCCCTCACCTGCACTGTCTCTGGATTCTCTTTGAGCAGTTACGGTGTAGGCTGGGTCCGCCAGGCTCCAGGAAA
AGGGCTGGAATTTGTTGGTGGTATAGCTAGTAGTGGAAGTGCAAACTACAACCCAGCCCTGAAGTCCCGAGCCAGCA
TCACCAAGGACACCTCAAAGAGCCAAGTTTATCTGACGCTGAACAGCCTGACAAGCGAGGACACGGCCGTCTATTAC
TGTACAGGAGGCATATATGGTTCTGGTTATAATACAGCATTTGGCTACTGGGGCCAGGGCACCCTGGTCACCGTCTC
CTCAGAGAGTACGATGACCCCAGATCTCTTCCCCCTCGTCTCCTGTGGGCCCTCTCTTGATGAGAGCCTGGTGGCTG
TGGGCTGCCTAGCCCGGGACTTCCTACCCAA
>KY437214.1 Equus caballus clone 58 immunoglobulin mu heavy chain (IGHM) mRNA, partial cds
CTCTCCCTCACCTGCACTGTCTCTGGATTCTCTTTGAGCAGTTACGCTGTAGGCTGGGTCCGCCAGGCTCCAGGAAG
AGGGCTGGAATTTGTTGGTGGTATAGCTAGTAGTGGAAGTGCAATGTACAACCCAGCCCTGAAGTCCCGAGCCAGCA
TCACCAAGGACACCTCAAAGAGCCAAGTTTATCTGACGCTGAACAGCCTGACAAGCGAGGACACGGCCGTCTATTAC
TGTGCAGGAGGCAATGACTACTGGGGCCAGGGCATCCTGGTCACCGTCTCCTCAGAGAGTACGATGACCCCAGATCT
CTTCCCCCTCGTCTCCTGTGGGCCCTCTCTTGATGAGAGCCTGGTGGCTGTGGGCTGCCTAGCCCGGGACTTCCTAC
CCAA
>KY437213.1 Equus caballus clone 57 immunoglobulin mu heavy chain (IGHM) mRNA, partial cds
CTCTCCCTCACCTGCACTGTCTCTGGATTATCTTTGAGCAGTAATCCTGTAGGCTGGGTCCGCCAGGCTCCAGGAAA
AGGGCTGGAATTTGTTGGTGCTATATATGGTAGTGCAAGTGCAAACTACAACCCAGCCCTGAAGTCCCGAGCCAGCA
TCACCAAGGACACCTCAAAGAGCCAAGTTTATCTGACGCTGAACAGCCTGACAAGCGAGGACACGGCCGTCTATTAC
TGTGCAGGAGGCTTAGGTTATGGTGGTGCTTACATTTATGGTCCAAACTACTGGGGCCAGGGCATCCTGGTCACCGT
CTCCTCAGAGAGTACGATGACCCCAGATCTCTTCCCCCTCGTCTCCTGTGGGCCCTCTCTTGATGAGAGCCTGGTGG
CTGTGGGCTGCCTAGCCCGGGACTTCCTACCCAA
>KY437212.1 Equus caballus clone 56 immunoglobulin mu heavy chain (IGHM) mRNA, partial cds
CTCTCCCTCACCTGCACTGTCTCTGGATTATCTTTGAGCAGTAATGCTGTAGGCTGGGTCCGCCAGGCTCCAGGAAA
AGGGCTGGAATTGGTTGGTTACATATATGGTAGTGCAAGTGCAAACTACAACCCAGCCCTGAAGTCCCGAGCCAGCA
TCACCAAGGACACCTCAAAGAGCCAAGTTTATCTGACGCTGAACAGCCTGACAAGCGAGGACACGGCCGTCTATTAC
TGTGCAGGAAGGACATATAGCAGTTATGATTACAATGAGTCAGGCCCGGTCTACTGGGGCCAGGGCATCCTGGTCAC
CGTCTCCTCAGAGAGTACGATGACCCCAGATCTCTTCCCCCTCGTCTCCTGTGGGCCCTCTCTTGATGAGAGCCTGG
TGGCTGTGGGCTGCCTAGCCCGGGACTTCCTACCCAA
>KY437211.1 Equus caballus clone 55 immunoglobulin mu heavy chain (IGHM) mRNA, partial cds
CTCTCCCTCACCTGCACTGTCTCTGGATTATCTTTGAGCAGTAATGCTGTAGGCTGGGTCCGCCAGGCTCCAGGAAA
AGGGCTGGAATATGTTGGTGGTATAGCTAGTAGTGGAGGTGCAAACTACAACCCAGCCCTGAAGTCCCGAGCCAGCA
TCACCAAGGACACCTCAAAGAGCCAAGTTTATCTGACGCTGAACAGCCTGACAGGCGAGGACACGGCCGTCTATTAC
TGTGCGAGCGAATATGAGAGGGGCAGCGCTGCTGCTGGCTACTGGGGCCAGGGCACCCTGGTCACCGTCTCCTCAGA
GAGTACGATGACCCCAGATCTCTTCCCCCTCGTCTCCTGTGGGCCCTCTCTTGATGAGAGCCTGGTGGCTGTGGGCT
GCCTAGCCCGGGACTTCCTACCCAA
>KY437210.1 Equus caballus clone 54 immunoglobulin mu heavy chain (IGHM) mRNA, partial cds
CTGTCCCTCACCTGCACTGTCTCTGGATTCTCTTTGAGCAGTTACGATGTAGGCTGGGTCCGCCAGGCTCCAGGAAA
AGGGCTGGAATATGTTGGTGGTATAACTAGTAGTGGAAGTGCAAACTACAACCCAGCCCTGAAGTCCCGAGCCAGCA
TCACCAAGGACACCTCAAAGAGCCAAGTTTATCTGACGCTGAACAGCCTGACAGGCGAGGACACGGCCGTCTATTAC
TGTGCAGGAGTAGTCTACTGGGGCCAGGGCACCCTGGTCACCGTCTCCTCAGAGAGTACGATGACCCCAGATCTCTT
CCCCCTCGTCTCCTGTGGGCCCTCTCTTGATGAGAGCCTGGTGGCTGTGGGCTGCCTAGCCCGGGACTTCCTACCCA
A
>KY437209.1 Equus caballus clone 53 immunoglobulin mu heavy chain (IGHM) mRNA, partial cds
CTGTCCCTCACCTGCACTGTCTCTGGATTCTCTTTGAGCAGTTACGGTGTAGGCTGGGTCCGCCAGGCTCCAGGAAA
AGGGCTGGAATTTGTTGGTGGTATAGCTAGTAGTGGAAGTGCGGGCTACAACCCAGCCCTGAAGTCCCGAGCCAGCA

TCACCAAGGACACCTCAAAGAGCCAAGTTTATCTGACGCTGAACAGCCTGACAAGCGAGGACACGGCCGTCTATTAC
TGTGCAGGAGGGGATGGTGGTGCTTACTGGGGCCAGGGCATCCTGGTCACCGTCTCCTCAGAGAGTACGATGACCCC
AGATCTCTTCCCCCTCGTCTCCTGTGGGCCCTCTCTTGATGAGAGCCTGGTGGCTGTGGGCTGCCTAGCCCGGGACT
TCCTACCCAA
>KY437208.1 Equus caballus clone 52 immunoglobulin mu heavy chain (IGHM)
mRNA, partial cds
CTGTCCCTCACCTGCACTGTCTCTGGATTCTCTTTGAGCAGTTACGGTGTAGGCTGGGTCCGCCAGGCTCCAGGAAA
AGGGCTGGAATTTGTTGGTGGTATAGCTAGTAGTGGAAGTGCAAACTACAACCCAGCCCTGAAGTCCCGAGCCAGCA
TCACCAAGGACACCTCAAAGAGCCAAGTTTATCTGACGCTGAACAGCCTGACAGGCGAGGACACGGCCGTCTATTAC
TGTGCGAGATCCCCCTCGCCCTATGGTTATGTTCCTTACTACCATTATGATATAGACTACTGGGGCCAGGGCACCCT
GGTCACCGTCTCCTCAGAGAGTACGATGACCCCAGATCCCTTCCCCCTCGTCTCCTGTGGGCCCTCTCTTGATGAGA
GCCTGGTGGCTGTGGGCTGCCTAGCCCGGGACTTCCTACCCAA
>KY437207.1 Equus caballus clone 51 immunoglobulin mu heavy chain (IGHM)
mRNA, partial cds
CTCTCCCTCACCTGCACTGTCTCTGGATTCTCTTTGAGCAGTTACGGTGTAGGCTGGGTCCGCCAGGCTCCAGGAAA
AGGGCTGGAATTTGTTGGTGGTATAGCTATAAGTGGAAGTGCAAACTACAACCCAGCCCTGAAGTCCCGAGCCAGCA
TCACCAAGGACACCTCAAAGAGCCAAGTTTATCTGACGCTGAACAGCCTGACAAGCGAGGACACGGCCGTCTATTAC
TGTGCAGGAGGCGACTACGGTTACTGGGGCCAGGGCATCCTGGTCACCGTCTCCTCAGAGAGTACGATGACCCCAGA
TCTCTTCCCCCTCGTCTCCTGTGGGCCCTCTCTTGATGAGAGCCTGGTGGCTGTGGGCTGCCTAGCCCGGGACTTCC
TACCCAA
>KY437206.1 Equus caballus clone 50 immunoglobulin mu heavy chain (IGHM)
mRNA, partial cds
CTCTCCCTCACCTGCACTGTCTCTGGATTATCTTTGAGCAGTAATGCTGTAGGCTGGGTCCGCCAGGCTCCAGGAAA
AGGGCTGGAATATGTTGGTGGTATAGCTAGTAGTGGAAGTGCAAACTACAACCCAGCCCTGAAGTCCCGAGCCAGCA
TCGCCAAGGACACCTCAAAGAGCCAAGTTTATCTGACGCTGAACAGCCTGACAAGCGAGGACACGGCCGTCTATTAC
TGTGCAGGAGGCGAGGGTTTTTCCTGGAGTTCCTATGCTGGTAGTTACTTATACTACGGCCACTTTGGGGCTATGGA
CCCCTGGGGCCAGGGCACCCTGGCCACCGTCTCCTCAGAGAGTACGATGACCCCAGATCTCTTCCCCCTCGTCTCCT
GTGGGCCCTCTCTTGATGAGAGCCTGGTGGCTGTGGGCTGCCTAGCCCGGGACTTCCTACCCAA
>KY437205.1 Equus caballus clone 49 immunoglobulin mu heavy chain (IGHM)
mRNA, partial cds
CTCTCCCTCACCTGCACTGTCTCTGGATTATCTTTGAACTCTGTAGGCTGGGTCCGCCAGGCTCCAGGAAAAGGGCT
GGAATTTGTTGGTGTTCTATATGGTGGTGGAAGTGCAAACTACAACCCAGCCCTGAAGTCCCGAGCCAGCATCACCA
AGGACACCTCAAAGAGCCAAGTTTCACTGACGCTGAACCTGAACAGCCTGACAAGCGAGGACACGGCCGTCTATTAC
TGTGCAGGAGGTACCAACGATTATAATAATGCTTTTGCCTACTGGGGCCAGGGCACCCTGGTCACCGTCTCCTCAGA
GAGTACGATGACCCCAGATCTCTTCCCCCTCGTCTCCTGTGGGCCCTCTCTTGATGAGAGCCTGGTGGCTGTGGGCT
GCCTAGCCCGGGACTTCCTACCCAA
>KY437204.1 Equus caballus clone 48 immunoglobulin mu heavy chain (IGHM)
mRNA, partial cds
CTCTCCCTCACCTGCACTGTCTCTGGATTCTCTTTGAGCAGTTACGGTGTAGGCTGGGTCCGCCAGGCTCCAGGAAA
AGGGCTGGAATTTGTTGGTGCGATACCTAGTAGTGGAAGTGCAAACTACAACCCAGCCCTGAAGTCCCGAGCCAGCA
TCACCAAGGACACCTCAAAGAGCCAAGTTTATCTGACGCTGAACAGCCTGACAAGCGAGGACACGGCCGTCTATTAC
TGTGCAGCAGGTGGGGGATATAGCACGTACTTTGGCCAATGGGGCCAGGGCACCCTGGTCACCGTCTCCTCAGAGAG
TACGATGACCCCAGATCTCTTCCCCCTCGTCTCCTGTGGGCCCTCTCTTGATGAGAGCCTGGTGGCTGTGGGCTGCC
TAGCCCGGGACTTCCTACCCAA
>KY437203.1 Equus caballus clone 47 immunoglobulin mu heavy chain (IGHM)
mRNA, partial cds
CTGTCCCTCACCTGCACTGTCTCTGGATTCTCTTTGAGCAGTTACGGTGTAGGCTGGGTCCGCCAGGCTCCAGGAAA
AGGGCTGGAATTTGTTGGTGGTATAGCTAGTAGTGGAAGTGCAAACTACAACCCAGCCCTGAAGTCCCGAGCCAGCA
TCACCAAGGACACCTCAAAGAGCCAAGTTTATCTGACGCTGAACAGCCTGACAGGCGAGGACACGGCCGTCTATTAC
TGTGCGAGGGTGGTTACTATAGTAGGAGTTGCCGATTATTTCGGTATAAACTACTGGGGCCAGGGCATCCTGGTCAC
CGTCTCCTCAGAGAGTACGATGACCCCAGATCTCTTCCCCCTCGCCTCCTGTGGGCCCTCTCTTGATGAGAGCCTGG
TGGCTGTGGGCTGCCTAGCCCGGGACTTCCTACCCAA
>KY437202.1 Equus caballus clone 46 immunoglobulin mu heavy chain (IGHM)
mRNA, partial cds
CTCTCCCTCACCTGCACTGTCTCTGGATTCTCTTTGAGCAGTTATGCTGTAGGCTGGGTCCGCCAGGCTCCAGGAAA
AGGGCTGGAATATGTTGGTGCTATATATGGTAGTGCAAGTGCAAACTACAACCCAGCCCTGAAGTCCCGAGCCAGCA

TCACCAAGGACACCTCAAAGAGCCAAGTCTATCTGACGCTGAACAGCCTGACAGGCGAGGACACGGCCGTCTATTAC
TGTGCGAGGTATGGTTATGGTGGTGCTTACTACTACGGTATAAACTACTGGGGCCAGGGCATCCTGGTCACCGTCTC
CTCAGAGAGTACGATGACCCCAGATCTCTTCCCCCTCGTCTCCTGTGGGCCCTCTCTTGATGAGAGCCTGGTGGCTG
TGGGCTGCCTAGCCCGGGACTTCCTACCCAA
>KY437201.1 Equus caballus clone 45 immunoglobulin mu heavy chain (IGHM) mRNA, partial cds
CTCTCCCTCACCTGCACTGTCTCTGGATTATCTTTGAGCAGTAATCATGTAGGCTGGGTCCGCCAGGCTCCAGGAAA
AGGGCTGGAATATGTTGGTATGATATATGGTAGTGCAATTGAAAACTACAACCCAGCCCTGAAGTCCCGAATCCGCA
TCACCAAGGACACCTCAAAGAGCCTAGTTTATCTGACGCTGAACAGCCTGACAAGCGAGGACACGGCCGTCTATTAC
TGTGCAGGATTTTTTGTACCTATGAAATTCGACTACTGGGGCCAGGGCACCCTAGTCACCGTCTCCTCAGAGAGTAC
GATGACCCCAGATCTCTTCCCCCTCGTCTCCTGTGGGCCCTCTCTTGATGAGAGCCTGGTGGCTGTGGGCTGCCTAG
CCCGGGACTTCCTACCCAA
>KY437200.1 Equus caballus clone 44 immunoglobulin mu heavy chain (IGHM) mRNA, partial cds
CTGTCCCTCACCTGCACTGTCTCTGGATTCTCTTTGAGCAGTTACGGTGTAGGCTGGGTCCGCCAGGCTCCAGGAAA
AGGGCTGGAATTTGTTGGTGGTATAGCTAGTAGTGGAAGTGCAAACTACAACCCAGCCCTGAAGTCCCGAGCCAGCA
TCACCAAGGACACCTCAAAGAGCCAAGTTTATCTGACGCTGAACAGCCTGACAGGCGAGGACACGGCCGTCTATTAC
TGTGCGAGCATTGGTTTTCTGTACTGGGGCCAGGGCACCCTGGTCACCGTCTCCTCAGAGAGTACGATGACCCCAGA
TCTCTTCCCCCTCGTCTCCTGTGGGCCCTCTCTTGATGAGAGCCTGGTGGCTGTGGGCTGCCTAGCCCGGGACTTCC
TACCCAA
>KY437199.1 Equus caballus clone 43 immunoglobulin mu heavy chain (IGHM) mRNA, partial cds
CTGTCCCTCACCTGCACTGTCTCTGGATTCTCTTTGAGCAGTTACGGTGTAGGCTGGGTCCGCCAGGCTCCAGGAAA
AGGGTGGGAATATGTTGGTGAAATAGCTGGTAGTGGAAGTGCAAACTACAGCCCAGCCCTGAAGTCCCGAGCCAGCA
TCACCAAGGACACCTCAAAGAGCCAAGTTTATCTGACGCTGAACAGCCTGACAGGCGAGGACACGGCCGTCTATTAC
TGTGTAAAAGTGTTTGGTTATGGTGATACTAATCACTTTGGCTACTGGGGCCAGGGCACCCTGGTCACCGTCTCCTC
AGAGAGTACGATGACCCCAGATCTCTTCCCCCTCGTCTCCTGTGGGCCCTCTCTTGATGAGAGCCTGGTGGCTGTGG
GCTGCCTAGCCCGGGACTTCCTACCCAA
>KY437198.1 Equus caballus clone 42 immunoglobulin mu heavy chain (IGHM) mRNA, partial cds
CTGTCCCTCACCTGCACTGTCTCTGGATTCTCTTTGAGCAGTTACCGTGTAGGCTGGGTCCGCCAGGCTCCAGGAAA
AGGGCTGGAATATGTTGGTGGTATAACTACAATTGGAAAAACATACTGGAACCCAGCCCTGAAGTCCCGAGCCAGCA
TCACCAAGGACACCTCAAAGAGCCAAGTTTATCTGACGCTGAACAGCCTGACAGGCGAGGACACGGCCGTCTATTAC
TGTGCGAGGGGCGCTTCTCAGGCGTTTGAATACTGGGGCCAGGGCATCCTGGTCACCGTCTCCTCAGAGAGTACGAT
GACCCCAGATCTCTTCCCCCTCGTCTCCTGTGGGCCCTCTCTTGATGAGAGCCTGGTGGCTGTGGGCTGCCTAGCCC
GGGACTTCCTACCCAA
>KY437197.1 Equus caballus clone 41 immunoglobulin mu heavy chain (IGHM) mRNA, partial cds
CTGTCCCTCACCTGCACTGTCTCTGGATTCTCTTTGAGCAGTTACGCTGTAGGCTGGGTCCGCCAGGCTCCAGGAAA
AGGGCTGCAATATGTTGGTGTGAGCGTGAGTAGTGGAAGTTCATACTACAACCCACCCCTGGCGTCCCGAGCCCGCA
TCACCAAGGACACTTCAAAGAGCCAAGTTTATCTGACGCTGAACAGCCTGACAGGCGAGGACACGGCCGTCTATTAC
TGTGCGAGAGCCGAATATGGTGGTGGCACTGCTGATATTTACTACTGGGGCCAGGGCATCCTGGTCACCGTCTCCTC
AGAGAGTACGATGACCCCAGATCTCTTCCCCCTCGTCTCCTGTGGGCCCTCTCTTGATGAGAGCCTGGTGGCTGTGG
GCTGCCTAGCTCGGGACTTCCTACCCAA
>KY437196.1 Equus caballus clone 40 immunoglobulin mu heavy chain (IGHM) mRNA, partial cds
CTCTCCCTCACCTGCACTGTCTCTGGATTGTCTTTGAGCAGTCGTGCTGTATCATGGGTCCGCCAGGCTCCAGGAAA
AGGGCTGGAATATGTTGGTACTATAGGTGACAGTGGAAGTGCAAAGTACAACCCAGTCCTGAAGTCCCGAGCCAGCA
TCACCAAGGACACCTCCAAGAGCCAAGTTTATCTGACGCTGAACAGCCTGACAGGCGAGGACACGGCCGTCTATTAC
TGTGCGGCCCGATTGTACTATTTTGGCTATTGGGGCCAGGGCACCCTGGTCACCGTCTCCTCAGAGAGTACGATGAC
CCCAGATCTCTTCCCCCTCGTCTCCTGTGGGCCCTCTCTTGATGAGAGCCTGGTGGCTGTGGGCTGCCTAGCCCGGG
ACTTCCTACCCAA
>KY437195.1 Equus caballus clone 39 immunoglobulin mu heavy chain (IGHM) mRNA, partial cds

CTCTCCCTCACCTGCACTGTCTCTGGATTGTCTTTGAGCAGTAGTACTATACACTGGGTCCGCCAGGCTCCAGGAAA
AGGGCTGGAATATGTTGGTGGTATAGCTGGTAGTGGAAGTTCTTACTACAACCCAGCCCTGAAGTCCCGGGCCAGCA
TCACCAAGGACACCTCCAAGAGTCAAGTTTATCTGACGCTGAACAGCCTGACAGGCGAGGACACGGCCGTCTATTAC
TGTGCGAGCATTGGTTTTCTGTACTGGGGCCAGGGCACCCTGGTCACCGTCTCCTCAGAGAGTACGATGACCCCAGA
TCTCTTCCCCCTCGTCTCCTGTGGGCCCTCTCTTGATGAGAGCCTGGTGGCTGTGGGCTGCCTAGCCCGGGACTTCC
TACCCAA
>KY437194.1 Equus caballus clone 38 immunoglobulin mu heavy chain (IGHM) mRNA, partial cds
CTGTCCCTCACCTGCACTGTCTCTGGATTATCTTTGAGCAGTAATGCTGTAGGCTGGGTCCGCCAGGCTCCAGGAAA
AGGGCTGGAGTGGGTTGGTGTTATATCTGGTGATGAAAGTACATACTACAACCCAGCCCCGAAGTCCCGAGCCAGCA
TCACCAAGGACACCTCAAAGAGCCAAGTTTATCTGACGCTGAACAGCCTGACAGGCGAGGACACGGCCGTCTATTAC
TGTGCGAGATATGGTTACTATGGTGGTAGTTACTATGCCGCTGCTATAGACTACTGGGGCCAGGGCACCCTGGTCAC
CGTCTCCTCAGAGAGTACGATGACCCCAGATCTCTTCCCCCTCGTCTCCTGTGGGCCCTCTCTTGATGAGAGCCTGG
TGGCTGTGGGCTGCCTAGCCCGGGACTTCCTACCCAA
>KY437193.1 Equus caballus clone 37 immunoglobulin mu heavy chain (IGHM) mRNA, partial cds
CTCTCCCTCACCTGCACTGTCTCTGGATTATCTTTGAGCAGTAATACTGTACACTGGGTCCGCCAGGCTCCAGGAAA
AGGGCTGGAATATGTTGGTGGTATAGCTACTAGTGGAAGTGCAAACTACAACCCAGCCCTGAAGTCCCGAGCCAGCA
TCACCAAGGACACCTCAAAGAGCCAAGTTTATCTGACGCTGAACAGCCTGACAAGCGAGGACACGGCCGTCTATTAC
TGTGCAGGACATAATCGTTATTACTATGGTAGTTACTACAGTGGTTACTATGTATTTGTCTACTGGGGCCAGGGCAC
CCTGGTCACCGTCTCCTCAGAGAGTACGATGACCCCAGATCTCTTCCCCCTCGTCTCCTGTGGGCCCTCTCTCGATG
AGAGCCTGGTGGCTGTGGGCTGCCTAGCCCGGGACTTCCTACCCAA
>KY437192.1 Equus caballus clone 36 immunoglobulin mu heavy chain (IGHM) mRNA, partial cds
CTGTCCCTCACCTGCACTGTCTCTGGATTATCTTTGAGCAGTAATGCTGTAGGCTGGGTCCGCCAGGCTCCAGGAAA
AGGGCTGGAGTGGGTTGGTGTTAGATATGGTAGTGAAAGTACATACTACAACCCAGCCCTGAAGTCCCGAGCCAGCA
TCACCAAGGACACCTCAAAGAGCCAAGTTTATCTGACGCTGAACAGCCTGACAGGCGAAGACACGGCCGTCTATTAC
TGTGCAGGATGGGATCGTAATGGTGGTGCGCTTGGCTACTGGGGCCAGGGCACCCTGGTCACCGTCTCCTCAGAGAG
TACGATGACCCCAGATCTCTTCCCCCTCGTCTCCTGTGGGCCCTCTCTTGATGAGAGCCTGGTGGCTGTGGGCTGCC
TAGCCCGGGACTTCCTACCCAA
>KY437191.1 Equus caballus clone 35 immunoglobulin mu heavy chain (IGHM) mRNA, partial cds
CTGTCCCTCACCTGCACTGTCTCTGGATTATCTTTGAGCAGTAATCATGTAGGCTGGGTCCGCCAGGCTCCAGGAAA
AGGGCTGGAGTGGGTTGGTGTTATATATGGTAGTGAAAGTACACAGTACAACCCAGCCCTGAAGTCCCGAGCCAGCA
TCACCAAGGACACCTCAAAGAGCCAAGTTTATCTGACGCTGAACAGCCTGACAGGCGAAGACACGGCCGTCTATTAC
TGTGCAGGATGTAGGGGGGACTATGGTTATTACATTGTTGGCTACTGGGGCCAGGGCACCCTGGTCACCGTCTCCTC
AGAGAGTACGATGACCCCAGATCTCTTCCCCCTCGTCTCCTGTGGGCCCTCTCTTGATGAGAGCCTGGTGGCTGTGG
GCTGCCTAGCCCGGGACTTCCTACCCAA
>KY437190.1 Equus caballus clone 34 immunoglobulin mu heavy chain (IGHM) mRNA, partial cds
CTGTCCCTCACCTGCACTGTCTCTGGATTATCTTTGAGCACTAATGCTGTAGGCTGGGTCCGCCAGGCTCCAGGAAA
AGGGCTGGAGTGGGTTGGTGTTATATATGCCCGTGAAAGTACATACTACAACCCAGCCCTGAAGTCCCGAGCCAGCA
TCACCAAGGACACCTCAAAGAGCCAAGTTTATCTGACGCTGAACAGCCTGACAGGCGAAGACACGGCCGCCTATTAC
TGTGCAGGATGGAGAGCGGGTTATGACGGTGCTTACTACTGGGGCCAGGGCACCCTGGTCACCGTCTCCTCAGAGAG
TACGATGACCCCAGATCTCTTCCCCCTCGTCTCCTGTGGGCCCTCTCTTGATGAGAGCCTGGTGGCTGTGGGCTGCC
TAGCCCGGGACTTCCTACCCAA
>KY437189.1 Equus caballus clone 33 immunoglobulin mu heavy chain (IGHM) mRNA, partial cds
CTCTCCCTCACCTGCACTGTCTCTGGATTATCTTTGAGCAGTAATGCTGTAGGCTGGGTCCGCCAGGCTCCAGGAAA
AGGGCTGGAATATGTTGGATATATAGCTAGTAGTGGAAGTGCAGACTACAACCCAGCCCTGAAGTCCCGAGCCAGCA
TCACCAAGGACACCTCAAAGAGCCAAGTTTATCTGACGCTGAACAGCCTGACAGGCGAAGACACGGCCGTCTATTAC
TGTGCAGGAGGGGACGGAGAAGAAGTGGTGTGGGGCCAGGGCACCCTGGTCACCGTCTCCTCAGAGAGTACGATGAC
CCCAGATCTCTTCCCCCTCGTCTCCTGTGGGCCCTCTCTTGATGAGAGCCTGGTGGCTGTGGGCTGCCTAGCCCGGG
ACTTCCTACCCAA
>KY437188.1 Equus caballus clone 32 immunoglobulin mu heavy chain (IGHM) mRNA, partial cds

CTCTCCCTCACCTGCACTGTCTCTGGATTATCTTTGAGCAGTCTAGCTGTAGGCTGGGTCCGCCAGGCTCCAGGAAA
AGGGCTGGAATATGTTGGTGATATGAGTAGTGTTGGAAGTACAAACTACAACCCAGCCCTGAAGTCCCGAGCCAGCA
TCACCAAGGACACCTCAAAGAGCCAAGTTTATCTGACGCTGAACAGCCTGACAAGCGAGGACACGGCCGCCTATTAC
TGTGCAGGAGCGTTCATTACTGAGACTTTTAGTAGTTACTATTTGAACTACTGGGGCCAGGGCACCCTGGTCACCGT
CTCCTCAGAGAGTACGATGACCCCAGATCTCTTCCCCCTCGTCTCCTGTGGGCCCTCTCTTGATGAGAGCCTGGTGG
CTGTGGGCTGCCTAGCCCGGGACTTCCTACCCAA
>KY437187.1 Equus caballus clone 31 immunoglobulin mu heavy chain (IGHM)
mRNA, partial cds
CTGTCCCTCACCTGCACTGTCTCTGGATTATCTTTGAGCAGTAATGCTGTAGGCTGGGTCCGCCAGGCTCCAGGAAA
AGGGCTGGAGTGGGTTGGTGCTATATATGGTAGTGAAAGTACATACTACAACCCAGCCCTGAAGTCCCGAGCCAGCA
TCACCAAGGACACCTCAAAGAGCCAAGTTTATCTGACGCTGAACAGCCTGACAGGCGAAGACACGGCCGTCTATTAC
TGTGCAGGAGAACAAATCTGGGGCCAGGGCACCCTGGTCACCGTCTCCTCAGAGAGTACGATGACCCCAGATCTCTT
CCCCCTCGTCTCCTGTGGGCCCTCTCTTGATGAGAGCCTGGTGGCTGTGGGCTGCCTAGCCCGGGACTTCCTACCCA
A
>KY437186.1 Equus caballus clone 30 immunoglobulin mu heavy chain (IGHM)
mRNA, partial cds
CTCTCCCTCACCTGCACTGTCTCTGGATTATCTTTGAGCAGTAATGCTGTAGGCTGGGTCCGCCAGGCTCCAGGAAA
AGGGCTGGAATACGTTGGTGCTATATATGGTAGTGCAAGTGCAAACTACAACCCAGCCCTGAAGTCCCGAGCCGGCA
TCACCAAGGACACCTCAAAGAGCCAAGTTTATCTGACGCTGAACAGCCTGACAAGCGAGGACACGGCCGTCTATTAC
TGTGCAGGAGGCCTAATCATGGTTATGGTGGTGCACCGCTATTATGGTATAAACTACTGGGGCCAGGGCATCCTGGT
CACCGTCTCCTCAGAGAGTACGAAGACCCCAGATCTCTTCCCCCTCGTCTCCTGTGGGCCCTCTCTTGATGAGAGCC
TGGTGGCTGTGGGCTGCCTAGCCCGGGACTTCCTACCCAA
>KY437185.1 Equus caballus clone 29 immunoglobulin mu heavy chain (IGHM)
mRNA, partial cds
CTCTCCCTCATCTGCACTGTCTCTGGATTATCTTTGAGCAGTTATGGTGTGGGCTGGGTCCGCCAGGCTCCAGGAAA
AGGGCTGGAATTTGTTGGTGGTATAGCTAGTAGTGGAAGTGCAAACTACAACCCAGCCCTGAAGTCCCGAGCCAGCA
TCACCAAGGACACCTCAAAGAGCCAAGTTTATCTGACGCTGAACAGCCTGACAAGCGAGGACACGGCCGTCTATTAC
TGTGCAGGAGACAATTACTATGGTGGTAGTTCCTGGTACTCCGTCTCTAGCTTTGACTACTGGGGCCAGGGCACCCT
GGTCACCGTCTCCTCAGAGAGTACGAAGACCCCAGATCTCTTCCCCCTCGTCTCCTGTGGGCCCTCTCTTGATGAGA
GCCTGGTGGCTGTGGGCTGCCTAGCCCGGGACTTCCTACCCAA
>KY437184.1 Equus caballus clone 28 immunoglobulin mu heavy chain (IGHM)
mRNA, partial cds
CTGTCCCTCACCTGCACTGTCTCTGGATATTCTTTGAGCGGAAATTCTGTAGGCTGGGTCCGCCAGGCTCCAGGAAA
AGGGCTGGAATGGGTTGGTGTTATAGAAGACAGTGAAAGTACATACTATAATCCAGCCCTGAAGTCCCGAGCCAGCA
TCACTAAGGACACCTCAAAGAGCCAAGTTTATCTGACGCTGAACAGCCTGACAGGCGAAGACACGGCCGTCTATTAC
TGTGCAGGATCCGACTATTACTGGGGTGCCGGTATAAACTATTGGGGCCAGGGCATCCTGGTCACCGTCTCCTCAGA
GAGTACGATGACCCCAGATCTCTTCCCCCTCGTCTCCTGTGGGCCCTCTCTTGATGAGAGCCTGGTGGCTGTGGGCT
GCCTAGCCCGGGACTTCCTACCCAA
>KY437183.1 Equus caballus clone 27 immunoglobulin mu heavy chain (IGHM)
mRNA, partial cds
CTCTCCCTCACCTGCACTGTCTCTGGATTATCTTTGAGCAGTAATGCTGTAGGCTGGGTCCGCCAGGCTCCAGGAAA
AGGGCTGGAATATGTTGGTGGTATAGCTAGTAGTGGAAGTGCAAACTACAACCCAGCCCTGAAGTCCCGAGCCAGCA
TCACCAAGGACACCTCAAAGAGCCAAGTTTATCTGACGCTGAACAGCCTGACAAGCGAGGACACGGCCGTCTATTAC
TGTGCAGGAGCCGGGTTTGACGGTTATGGTTATGCTACAGAGGATAATGCTATGGACCCCTGGGGCCAGGGCACCCT
GGTCACCGTCTCCTCAGAGAGTACGAAGACCCCAGATCTCTTCCCCCTCGTCTCCTGTGGGCCCTCTCTTGATGAGA
GCCTGGTGGCTGTGGGCTGCCTAGCCCGGGACTTCCTACCCAA
>KY437182.1 Equus caballus clone 26 immunoglobulin mu heavy chain (IGHM)
mRNA, partial cds
CTCTCCCTCACCTGCACTGTCTCTGGATTATCTTTGAGCAGTAATACTGTAGGCTGGGTCCGCCAGGCTCCAGGAAA
AGGGCTGGAATATGTTGGTAAAATAGCTAGTAGTGGAAGTGCAAACTACAACCCAGCCCTGAAGTCCCGAGCCAGCA
TCACCAAGGACACCTCAAAGAGCCAAGTTTATCTGACGCTGAACAGCCTGACAAGCGAGGACACGGCCGTCTATTAC
TGTGCAGGAGGGAATATCTATGGTTATGGTTATGCTACGGGTGGTATAAACTACTGGGGCCAGGGCATTCTGGTCAC
CGTCTCCTCAGAGAGTACGAAGACCCCAGATCTCTTCCCCCTCGTCTCCTGTGGGCCCTCTCTTGATGAGAGCCTGG
TGGCTGTGGGCTGCCTAGCCCGGGACTTCCTACCCAA

>KY437181.1 Equus caballus clone 25 immunoglobulin mu heavy chain (IGHM) mRNA, partial cds
CTCTCCCTCACCTGCACTGTCTCTGGATTATCTTTGAGCAGTAATGCTGTAGGCTGGGTCCGCCAGGCTCCAGGAAA
AGGGCTGGAATACGTTGGTGCGATATATGGTAGTGCAAACTACAACCCAGCCCTGAAGTCCCGAGCCAGCATCACTC
AGGACACCTCAAAGAGCCAAGTTTATCTGACGCTGAACAGCCTGACAAGCGAGGACACGGCCGTCTATTACTGTGCA
GGAGGAAACTACTGGGGCCAGGGCATCCTGGTCACCGTCTCCTCAGAGAGTACGAAGACCCCAGATCTCTTCCCCCT
CGTCTCCTGTGGGCCCTCTCTTGATGAGAGCCTGGTGGCTGTGGGCTGCCTAGCCCGGGACTTCCTACCCAA
>KY437180.1 Equus caballus clone 24 immunoglobulin mu heavy chain (IGHM) mRNA, partial cds
CTCTCCCTCACCTGCACTGTCTCTGGATTATCTTTGAGCAGTAATGCTGTAGGCTGGGTCCGCCAGGCTCCAGGAAA
AGGGCTGGAATACGTTGGTGCGATATATGGTAGTGCAAACTACAACCCAGCCCTGAAGTCCCGAGCCAGCATCACTC
AGGACACCTCAAAGAGCCAAGTCTATCTGACGCTGAACAGCCTGACAAGCGAGGACACGGCCGTCTATTACTGTGCA
GGAGGAAACTACTGGGGCCAGGGCATCCTGGTCACCGTCTCCTCAGAGAGTACGAAGACCCCAGATCTCTTCCCCCT
CGTCTCCTGTGGGCCCTCTCTTGATGAGAGCCTGGTGGCTGTGGGCTGCCTAGCCCGGGACTTCCTACCCA
>KY437179.1 Equus caballus clone 23 immunoglobulin mu heavy chain (IGHM) mRNA, partial cds
CTCTCCCTCACCTGCACTGTCTCTGGATTCTCTTTGAGCAGTTATGCTGTAGGCTGGGTCCGCCAGGCTCCAGGAAA
AGGGCTGGAATATGTTGGTGCTATATATGGTAGTGCAAGTGCAAACTACAACCCAGCCCTGAAGTCCCGAGCCAGCA
TCACCAAGGACACCTCAAAGAGCCAAGTTTATCTGACGCTGAACAGCCTGACAAGCGAGGACACGGCCGTCTATTAC
TGTGCAGGAGTGTATGGTTACTATGCTAGTGGTTATGACGTCTATGGTATAAACTACTGGGGCCAGGGCATCCTGGT
CACCGTCTCCTCAGAGAGTACGAAGACCCCAGATCTCTTCCCCCTCGTCTCCTGTGGGCCCTCTCTTGATGAGAGCC
TGGTGGCTGTGGGCTGCCTAGCCCGGGACTTCCTACCCAA
>KY437178.1 Equus caballus clone 22 immunoglobulin mu heavy chain (IGHM) mRNA, partial cds
CTCTCCCTCACCTGCACTGTCTCTGGATTATCTTTGAGCAGTAATGCTGTAGGCTGGGTCCGCCAGGCTCCAGGAAA
AGGGCTGGAATATGTTGGTGGTATAGATAGTAGTGGAAGTGCAAACTACAACCCAGCCCTGAAGTCCCGAGCCAGCA
TCACCAAGGACACCTCAAAGAGCCAAGTTTATCTGACGCTGAACAGCCTGACAAGCGAGGACACGGCCGTCTATTAC
TGTGCAGGAGGCGTTACTATAGACTGGGGCCAGGGCATCCTGGTCACCGTCTCCTCAGAGAGTACGATGACCCCAGA
TCTCTTCCCCCTCGTCTCCTGTGGGCCCTCTCTTGATGAGAGCCTGATGGCTGTGGGCTGCCTAGCCCGGGACTTCC
TACCCAA
>KY437177.1 Equus caballus clone 21 immunoglobulin mu heavy chain (IGHM) mRNA, partial cds
CTCTCCCTCACCTGCACTGTCTCTGGATTATCTTTGAGCAGTAATGCTGTAGGCTGGGTCCGCCAGGCTCCAGGAAA
AGGGCTGGAATATGTTGGTGGTATAGCTAGTAGTGGAAGTGCAAACTACAACCCAGCCCTGAAGTCCCGAGCCAGCA
TCACCAAGGACACCTCAAAGAGCCAAGTTTATCTGACGCTGAACAGCCTGACAAGCGAGGACACGGCCGTCTATTAC
TGTGCAGGAGTGTATGGTTACTATGCTAGTGGTTATGACGTCTATGGTATAAACTACTGGGGCCAGGGCATCCTGGT
CACCGTCTCCTCAGAGAGTACGAAGACCCCAGATCTCTTCCCCCTCGTCTCCTGTGGGCCCTCTCTTGATGAGAGCC
TGGTGGCTGTGGGCTGCCTAGCCCGGGACTTCCTACCCAA
>KY437176.1 Equus caballus clone 20 immunoglobulin mu heavy chain (IGHM) mRNA, partial cds
CTGTCCCTCACCTGCACTGTCTCTGGATTTCCTTTGAGCAGTTACGGTGTAGGCTGGGTCCGCCAGGCTCCAGGAAA
AGGGCTGGAACATGTTGGTAGTATAGCTAGTAGTGGAAGTGCAGTCTACAACCCAGCCCTGAAGTCCCGAGCCAGCG
TCAGCAAGGACACCTCAAAGAGCCAAGTTTATCTGACGCTGAACACCCTGACAAGCGAGGACACGGCCGTCTATTAC
TGTGCAGGAGGCGGGAACTGGCGTGACTATGGTGATTATTTCTACTATACGAAATTTTATGGTATAAACTACTGGGG
CCAGGGCATCCTGGTCACCGTCTCCTCAGAGAGTACGAAGACCCCAGATCTCTTCCCCCTCGTCTCCTGTGGGCCCT
CTCTTGATGAGAGCCTGGTGGCTGTGGGCTGCCTAGCCCGGGACTTCCTACCCAA
>KY437175.1 Equus caballus clone 19 immunoglobulin mu heavy chain (IGHM) mRNA, partial cds
CTCTCCCTCACCTGCACTGTCTCTGGATTATCTTTGAGCAGTAATGCTGTAGGCTGGGTCCGCCAGGCTCCAGGAAA
AGGGCTGGAATATGTTGGTGGTATAGCTAGTAGTGGAAGTGCAAACTACAACCCAGCCCTGAAGTCCCGAGCCAGCA
TCACCAAGGACACCTCAAAGAGACAAGTTTATCTGACGCTGAACAGCCTGACAAGCGAGGACACGGCCGCCTATTAC
TGTGCAGGAGGCTCCGATTATTATGCCAGAAACTACTGGGGCCAGGGCATCCTGGTCACCGTCTCCTCAGAGAGTAC
GAAGACCCCAGATCTCTTCCCCCTCGTCTCCTGTGGGCCCTCTCTTGATGAGAGCCTGGTGGCTGTGGGCTGCCTAG
CCCGGGACTTCCTACCCAA
>KY437174.1 Equus caballus clone 18 immunoglobulin mu heavy chain (IGHM) mRNA, partial cds

CTGTCCCTCACCTGCACTGTCTCTGGATTATCTTTGAGCAGTAATGCTGTAGGCTGGGTCCGCCAGGCTCCAGGAAA
AGGGCTGGAGTGGGTTGGTGTTATATATGGTAGTGAAAGTACATACTACAACCCAGCCCTGAAGTCCCGAGCCAGCA
TCACCAAGGACACCTCAAAGAGCCAAGTTTATCTGACGCTGAACAGCCTGACAGGCGAAGACACGGCCGTCTATTAC
TGTGCAGGATACGGAGATAATTATGGTAGTTACTATGCTTATTTTGCCTACTGGGGCCAGGGCACCCTGGTCACCGT
CTCCTCAGAGAGTACGAAGACCCCAGATCTCTTCCCCCTCGTCTCCTGTGGGCCCTCTCTTGATGAGAGCCTGGTGG
CTGTGGGCTGCCTAGCCCGGGACTTCCTACCCAA
>KY437173.1 Equus caballus clone 17 immunoglobulin mu heavy chain (IGHM) mRNA, partial cds
CTGTCCCTCACCTGCACTGTCTCTGGATTATCTTTGAGCAGTAATGCTGTAGGCTGGGTCCGCCAGGCTCCAGGAAA
AGGGCTGGAATATGTTGGTGGTATACGTAGTAGTGGAAGTGCAAACTACAATTCAGCCCTGAAGTCCCGAGCCAGCA
TCACCAAGGACACCTCAAAGAGCCAAGTTTATCTGACGCTGAACAGCCTGACAAGCGAGGACACGGCCGTCTATTAC
TGTGCAGGAGGAACGGGGAGTTTTTTTGGTTACTATGCTGGTACTTACTATCCCCCCGATTATTATGGTGAAAACTA
CTGGGGCCAGGGCATCCTGGTCACCGTCTCCTCAGAGAGTACGATGACCCCAGATCTCTTCCCCCTCGTCTCCTGTG
GGCCCTCTCTTGATGAGAGCCTGGTGGCTGTGGGCTGCCTAGCCCGGGACTTCCTACCCAA
>KY437172.1 Equus caballus clone 16 immunoglobulin mu heavy chain (IGHM) mRNA, partial cds
CTCTCCCTCACCTGCACTGTCTCTGGATTATCTTTGAGCAGTAATGCTGTAGGCTGGGTCCGCCAGGCTCCAGGAAA
AGGGCTGGAATATGTTGGTGGTATAGCTAGTAGTGGAAGTGCAAACTGGAACCCAGCCCTGAAGTCCCGAGCCAGCA
TCACCAAGGACACCTCAAAGAGCCAAGTTTATCTGACGCTGAACAGCCTGACAAGCGAGGACACGGCCGTCTATTAC
TGTGCAGGCGCGAGAGATCGTCCCTATTATGGTATAAACTACTGGGGCCAGGGCATCCTGGTCACCGTCTCCTCAGA
GAGTACGAAGACCCCAGATCTCTTCCCCCTCGTCTCCTGTGGGCCCTCTCTTGATGAGAGCCTGGTGGCTGTGGGCT
GCCTAGCCCGGGACTTCCTACCCAA
>KY437171.1 Equus caballus clone 15 immunoglobulin mu heavy chain (IGHM) mRNA, partial cds
CTGTCCCTCACCTGCACTGTCTCTGGATTCTCTTTGAGCAGTTACGGTGTAGGCTGGGTCCGCCAGGCTCCAGGAAA
AGGGCTGGAATATGTTGGTCGTATAGCAAGTAGTGGAAGTGCAAACTACAACCCAGCCCTGAAGTCCCGAGCCAGCA
TCACCAAGGACACCTCAAAGAGCCAAGTTTATCTGACGCTGAACAGCCTGACAGGCGAGGACACGGCCGTCTATTAC
TGTGCGTCGGATGGTGGTAGGGACTACTCTTCATTTGTCTACTGGGGCCAGGGCACCCTGGTCACCGTCTCCTCAGA
GAGTACGATGACCCCAGATCTCTTCCCCCTCGTCTCCTGTGGGCCCTCTCTTGATGAGAGCCTGGTGGCTGTGGGCT
GCCTAGCCCGGGACTTCCTACCCAA
>KY437170.1 Equus caballus clone 14 immunoglobulin mu heavy chain (IGHM) mRNA, partial cds
CTCTCCCTCACCTGCACTGTCTCTGGATTATCTTTGAGCAGTAATGCTGTAGGCTGGGTCCGCCAGGCTCCAGGAAA
AGGGCTGGAATACGTTGGTCATATATATGGTAGTGCAAGTGCAAACTACAACCCAGCCCTGAAGTCCCGAGCCAGCA
TCACCAAGGACACCTCAAAGAGCCAAGTTTATCTGACGCTGAACAGCCTGACAAGCGAGGACACGGCCGTCTATTAC
TGTGCAGGATCTGATCCTATCGAGTGGGGCCAGGGCATCCTGGTCACCGTCTCCTCAGAGAGTACGAAGACCCCAGA
TCTCTTCCCCCTCGTCTCCTGTGGGCCCTCTCTTGATGAGAGCCTGGTGGCTGTGGGCTGCCTAGCCCGGGACTTCC
TACCCAC
>KY437169.1 Equus caballus clone 13 immunoglobulin mu heavy chain (IGHM) mRNA, partial cds
CTGTCCCTCACCTGCACTGTCTCTGGATTATCTTTGGGCAGTTATTCTGTAGGCTGGGTCCGCCAGGCTCCAGGAAA
ACGGCTGGAGTGGGTTGGTCATATAGATGGTAGTGGAAGTTTAGAATACCACCCAGCCCTGAAGTCCCGAGTCAGCA
TCACCAAGGACACCTCAAAGAGCCAAGTTTATCTGACGCTGAACAGCCTGACAGGCGAAGACACGGCCGTCTATTAC
TGTGCAGGTGACGTATATGAGTACTGGGGCCAGGGCACCCTGGTCACCGTCTCCTCAGAGAGTACGAAGACCCCAGA
TCTCTTCCCCCTCGTCTCCTGTGGGCCCTCTCTTGATGAGAGCCTGGTGGCTGTGGGCTGCCTAGCCCGGGACTTCC
TACCCAA
>KY437168.1 Equus caballus clone 12 immunoglobulin mu heavy chain (IGHM) mRNA, partial cds
CTCTCCCTCACCTGCACTGTCTCTGGATTATCTTTGAGCAGTAATGCTGTAGGCTGGGTCCGCCAGGCTCCAGGAAA
AGGGCTGGAATACGTTGGTGCTATATATGGTAGTGCAAGTGCAAACTACAACCCAGCCCTGAAGTCCCGAGCCAGCA
TCACCAAGGACACCTCAAAGAGCCAAGTTTATCTGACGCTGAACAGCCTGACAAGCGAGGACACGGCCGTCTATTAC
TGTGCAGGAAATGGAAACGGTTATGGTTATGTTACCTTATCGGGGGATTATTATGGTATAAACTACTGGGGCCAGGG
CATCCTGGTCACCGTCTCCTCAGAGAGTACGAAGACCCCAGATCTCTTCCCCCTCGTCTCCTGTGGGCCCTCTCTTG
ATGAGAGCCTGGTGGCTGTGGGCTGCCTAGCCCGGGACTTCCTACCCAA

>KY437167.1 Equus caballus clone 11 immunoglobulin mu heavy chain (IGHM) mRNA, partial cds
CTCTCCCTCACCTGCACTGTCTCTGGATTATCTTTGAGCAGTAATGCTGTAGGCTGGGTCCGCCAGGCTCCAGGAAA
AGGGCTGGAATATGTTGGTGATATAGATAGTAGTGGAACAGCAAGGTATAACCCAGCCCTGAAGTCCCGAGCCAGCA
TCACCAAGGACACCTCAAAGAGCCAAGTTTATCTGACGCTGAACAGCCTGACAAGCGAGGACACGGCCGTCTATTAC
TGTGCAGGAGCGAGAGTTGCTATACGCTATGGCTACGTGGATCACTGGGGCCAGGGCACCCTGGTCACCGTCTCCTC
AGAGAGTACGATGACCCCAGATCTCTTCCCCCTCGTCTCCTGTGGGCCCTCTCTTGATGAGAGCCTGGTGGCTGTGG
GCTGCCTAGCCCGGGACTTCCTACCCAA
>KY437166.1 Equus caballus clone 10 immunoglobulin mu heavy chain (IGHM) mRNA, partial cds
CTCTCCCTCACCTGCACTGTCTCTGGATTATCTTTGAGCGGTAATTCTGTAGGCTGGGTCCGCCAGGCTCCAGGAAA
AGGGCTGGAATGGGTTGCTCATATATATGATAGTACAAGTCCGAGCTACAACCCAGCCCTGAAGTCCCGAGCCAGCA
TCACCAAGGACACCTCAAAGAGCCAAGTTTATCTGACGCTGAACAGCCTGACAAGCGAGGACACGGCCGTCTATTAC
TGTGCAGGAGGATATGGTGGAGCTTATGCATACTATTTTGGCTACTGGGGCCAGGGCACCCTGGTCACCGTCTCCTC
AGAGAGTACGAAGACCCCAGATCTCTTCCCCCTCGTCTCCTGTGGGCCCTCTCTTGATGAGAGCCTGGTGGCTGT
>KY437165.1 Equus caballus clone 9 immunoglobulin mu heavy chain (IGHM) mRNA, partial cds
CTCTCCCTCACCTGCACTGTCTCTGGATTATCTTTGAGCAGTAATGCTGTAGGCTGGGTCCGCCAGGCTCCAGGAAA
AGGGCTGGAATATGTTGGTGGTATAGCTAGTAGTGGAAGTACAAACTACAACCCAGCCCTGAAGTCCCGAGCCAGCA
TCACCAAGGACACCTCAAAGAGCCAAGTTTATCTGACGCTGAACAGCCTGACAAGCGAGGACACGGCCGTCTATTAC
TGTGCAGGAGGCTTAGACTACGGTTATGATTATGTGCGGTATGGTATAGTGTACTGGGGCCAGGGCATCCTGGTCAC
CGTCTCCTCAGAGAGTACGAAGACCCCAGATCTCTTCCCCCTCGTCTCCTGTGGGCCCTCTCTTGATGAGAGCCTGG
TGGCTGTGGGCTGCCTAGCCCGGGACTTCCTACCCAA
>KY437164.1 Equus caballus clone 8 immunoglobulin mu heavy chain (IGHM) mRNA, partial cds
CTGTCCCTCACCTGCACTGTCTCTGGATTCTCTTTGAGCAGTTACAGTGTATTCTGGGTCCGCCAGGCTCCAGGAAA
AGGGCTGGAATATGTTGGTGTAATACGTGATAGTGGAAGTACAAACTACAACCCAGCCCTGAAGTCCCGAGCCAGCA
TCACCAAGGACACCTCAAAGAGCCAAGTTTATCTGACGCTGAACAGCCTGACAGGCGAGGACACGGCCGTCTATTAC
TGTGCGAGCAGCGGTTTCTCCTACTGGGGCCAGGGCACCCTGGTCACCGTCTCCTCAGAGAGTACGATGACCCCAGA
TCTCTTCCCCCTCGTCTCCTGTGGGCCCTCTCTTGATGAGAGCCTGGTGGCTGTGGGCTGCCTAGCCCGGGACTTCC
TACCCAA
>KY437163.1 Equus caballus clone 7 immunoglobulin mu heavy chain (IGHM) mRNA, partial cds
CTCTCCCTCACCTGCACTGTCTCTGGATTATCTTTGAGCAGTAATGCTGTAGGCTGGGTCCGCCAGGCTCCAGGAAA
AGGGCTGGAATATGTTGGTGGTATAGCTAGTAGTGGAAGTGCAAACTACAACCCAGCCCTGAAGTCCCGAGCCAGCA
TCACCAAGGACACCTCAAAGAGCCAAGTTTATCTGACGCTGAACAGCCTGACAAGCGAGGACACGGCCGTCTATTAC
TGTGCAGGAGGCACCAGCTACTATAGTTATGGTAGTCACTATCATGTTCCATTGGTCTACTGGGGCCAGGGCACCCT
GGTCACCGTCTCCTCAGAGAGTACGATGACCCCAGATCTCTTCCCCCTCGTCTCCTGTGGGCCCTCTCTTGATGAGA
GCCTGGTGGCTGTGGGCTGCCTAGCCCGGGACTTCCTACCCAA
>KY437162.1 Equus caballus clone 6 immunoglobulin mu heavy chain (IGHM) mRNA, partial cds
CTGTCCCTCACCTGCACTGTCTCTGGATTCTCTTTGAGCAGTTACGGTGTAGGCTGGGTCCGCCAGGCTCCAGGAAA
AGGGCTGGAATATGTTGGTGGTATAGCTAGTAGTGGAAGTGCAAACTACAACCCAGCCCTGAAGTCCCGAGCCAGCA
TCACCAAGGACACCTCAAAGAGCCAAGTTTATCTGACGCTGAACAGCCTGACAGGCGAGGACACGGCCGTTTATTAC
TGTGCGAGAACTCGATATGGTTATGGTGGTGCTTACTACTATTTTGGCTACTGGGGCCGGGGCACCCTGGTCACCGT
CTCCTCAGAGAGTACGATGACCCCAGATCTCTCCCCCCTCGTCTCCTGTGGGCCCTCTCTTGATGAGAGCCTGGTGG
CTGTGGGCTGCCTAGCCCGGGACTTCCTACCCAA
>KY437161.1 Equus caballus clone 5 immunoglobulin mu heavy chain (IGHM) mRNA, partial cds
CTGTCCCTCACCTGCACTGTCTCTGGATTCTCTTTGAGCAGTTATGGTGTAGGCTGGGTCCGCCAGGCTCCAGGAAA
AGGGCTGGAATATGTTGGTGGTATAGCTAGTAGTGGAAGTGCAAACTACAACCCAGCCCTGAAGTCCCGAGCCAGCA
TCACCAAGGACACCTCAAAGAGCCAAGTTTATCTGACGCTGAACAGCCTGACAGGCGAGGACACGGCCGTCTATTAC
TGTGCGAGAACTCGATATGGTTATGGTGGTGCTTACTACTATTTTGGCTACTGGGGCCAGGGCACCCTGGTCACCGT
CTCCTCAGAGAGTACGATGACCCCAGATCTCTTCCCCCTCGTCTCCTGTGGGCCCTCTCTTGATGAGAGCCTGGTGG
CTGTGGGCTGCCTAGCCCGGGACTTCCTACCCAA

```
>KY437160.1 Equus caballus clone 4 immunoglobulin mu heavy chain (IGHM) mRNA,
partial cds
CTCTCCCTCACCTGCTCTGTGTCTGGAGTCTCCATCACAAGCAGTGGTGACTGGTGGAGCTGGATCCGCCAGCCCCC
AGGGAAGGGGCTGGAATGGATGGGGTACATAAGTTATAGTGGTAGCGCTTACTACACCACATCCCTCAAGAGCCGAC
TCTCCATCTCCAGAGACACGTCCAAGGACCAGTTCTCCCTGCAGCTGAGCTCCGTGACCGCCGAGGACACGGCCGTT
TATTACTGTGCAAGGATGCAGACATATGGTTATGGTGGTGCTTACCCCCATAATGCTATGGACCCCTGGGGCCAGGG
CACCCTGGTCACCGTCTCCTCAGAGAGTACGAAGACCCCAGATCTCTTCCCCCTCGTCTCCTGTGGGCCCTCTCTTG
ATGAGAGCCTGGTGGCTGTGGGCTGCCTAGCCCGGGACTTCCTACCCAA
>KY437159.1 Equus caballus clone 3 immunoglobulin mu heavy chain (IGHM) mRNA,
partial cds
CTGTCCCTCACCTGCACTGTCTCTGGATTCTCTTTGATCAATAACCGAGTATTTTGGTTCCGCCAGGCTCCCGGAAA
AGGGCTGGAATATCTTGGTGATATAGACAGTAGTGGAAATTCAATATACAACCCAGCCCTGAAGTCCCGAGGTAGCA
TCACCAGGGACACCTCAAAGAGCCAAGTTTATCTGTCGCTGAACAGCCTGACAGGCGAGGACACGGCCGTCTATTAC
TGTGCGAAAAGGCTGGATGGTGGTGGTGGTGCTTACTGGTATTTTGCCTACTGGGGCCAGGGCACCCTGGTCACCGT
CTCCTCAGAGAGTACGATGACCCCAGATCTCTTCCCCCTCGTCTCCTGTGGGCCCTCTCTTGATGAGAGCCTGGTGG
CTGTGGGCTGCCTAGCCCGGGACTTCCTACCCAG
>KY437158.1 Equus caballus clone 2 immunoglobulin mu heavy chain (IGHM) mRNA,
partial cds
CTGTCCCTCACCTGCACTGTCTCTGGATTCTCTTTGAGCAGTTACGGTGTAGGCTGGGTCCGCCAGGCTCCAGGAAA
AGGGCTGGAATATGTTGGTGGTATAGCTAGTAGTGGAAGTGCAAACTACAACCCAGCCCTGAAGTCCCGAGCCAGCA
TCACCAAGGACACCTCAAAGAGCCAAGTTTATCTGACGCTGAACAGCCTGACAGGCGAGGACACGGCCGTCTATTAC
TGTGCCTCTTTCCCCCAGTATGGTTACTATGCTGGTAGTTACTATGTCAGTTATTTTGGCTACTGGGGCCAGGGCAC
CCTGGTCACCGTCTCCTCAGAGAGTACGATGACCCCAGATCTCTTCCCCCTCGTCTCCTGTGGGCCCTCTCTTGATG
AGAGCCTGGTGGCTGTGGGCTGCCTAGCCCGGGACTTCCTACCCAA
>KY437157.1 Equus caballus clone 1 immunoglobulin mu heavy chain (IGHM) mRNA,
partial cds
CTGTCCCTCACCTGCACTGTCTCTGGATTATCTTTGAGCAGTAATGTTGTAGGCTGGGTCCGCCAGGCTCCAGGAAA
AGGGCTGGAGTGGGTTGGTGTTATATATGGTAGTGAAAGTGTATTGTACAACCCAGCCCTGAAGTCCCGAGCCAGCA
TCACCAAGGACACCTCAAAGAGCCAAGTTTATCTGACGCTGAATAGCCTGACAGGCGAAGACACGGCCGTCTATTAC
TGTGCAGGATCCCCATGGAACTACGGCACTAGCTTTAACGAGTTGGATTACTGGGGCCAGGGCACCCTGGTCACCGT
CTCCTCAGAGAGTACGAGGACCCCAGATCTCTTCCCCCTCGTCTCCTGTGGGCCCTCTCTTGATGAGAGCCTGGTGG
CTGTGGGCTGCCTAGCCCGGGACTTCCTACCCAA
```

1.2 **Supplementary Data 2. NCBI Genbank Equine Ig VK mRNA.** A total of 119 NCBI Genbank sequences, annotated as Equus caballus Ig VK sequences which constituted the dataset employed for the designing of the EquPD v2020 primer set.

```
>KJ741386.1 Equus caballus immunoglobulin kappa light chain (IGK) mRNA,
partial cds
ATGATGTCGCTGACAAAGGTCCTTATATCTGTGTTGCTCTGGGTCTCAGGTGCCTGTGGGGACATCGTGTTGACCCA
GTCTCCAGGGTCCTTGGCAGTGTCTCTAGGACAGAGGGTCGAGATGAAGTGCAAGGCCAGTCAGAGTGCTAGCAGCT
ACTTAGCTTGGTACCAGCAGAAACCAGGACAGGCTCCTAAGCAGCTCATCTACAGAGCATCCAGCAGAGCGTCTGGG
GTCCCTGACCGATTCAGTGGCAGTGGATCTGGGACAGATTTCACTCTCACCATCAGCAGCCTCCAGGCTGAAGATGT
GGCCGTTTATTACTGTCAGCAGTATAATAGTGCTCCTCCGGTTACGTTCGGCCAGGGGACCAAGCTGGAGATCAAAC
GGGATGATGCTAAGCCATCTGCCTTCATCTTCCCACCGTCTTCTGAGGAGTTAAGCAGTGGAAGTGCCTCTGTCGTA
TGCTTGGTGTATGGCTTCTACCCCAGTGGAGCCACTATCAACTGGAAAGTGGATGGTCTTGCCAAAACAAGTAGCTT
CCACAGCAGTCTGACGGAGCAGGACAGCAAGGACAACACCTACAGCCTCAGCAGCACCCTGACGCTGC
>HM176272.1 Equus caballus clone 1K272 immunoglobulin kappa light chain V-J
region mRNA, partial cds
ATGTCGCTGACAAAGGTCCTTATATCTGTGTTGCTCTGGGTCTCAGGTGCCTGTGGGGACATCGTGTTGACCCAGTC
TCCAGAGTCCTTGGCAGTGTCTCTAGGACAGAGGGTCGAGATGAAGTGCAAGGCCAGTCAGAGTTTTAGCAGGTACT
TAAACTGGTACCAGCAGAAACCAGGGCAGGCTCCGAAGCAGATCATCTATGATGCGTCCAGCAGAGCATCTGGCGTC
CCTGACCGATTCAGTGGCAGTGGATCTGGGACAGATTTCACTCTCACCATCAGCAGCCTCCAGGCTGAAGATGTGGC
CACTTATTACTGTCAGCAGGTTAATAGTCTTCCTCTTACGTTCGGCCAGGGGACCAAGCTGGAGATCAAACGGGATG
ATGCTAAGCCATCTGCCTTCATCTTCCCACCGTCTTCTGAGGAGTTAAGCAGTGGAAGTGCCTCCGTCGTATGCTTG
GTGTATGGCTTCTACCCCAGTGGAGCCACTATCAACTGGAAAGTGGATGGTCTTGCCAAAACAAGTAGC
>HM176271.1 Equus caballus clone 1K288 immunoglobulin kappa light chain V-J
region mRNA, partial cds
ATGATGTCGCTGACAAAGGTCCTTATATCTGTGTTGCTCTGGGTCTCAGGAGCCTGTGGGGACAATGTGTTGACCCA
GTCTCCAGAGTCCTTGGCAGTGTCTCTAGGACAGAGGGTCGAGATGAAGTGCAAGGCCAGTCCGGGTGTTAGTTTCT
ACTTAGCTTGGTACCAGCAGAAACCAGGACAGGCTCCTGAACAGCTCATCTATGCTGCATCCAGCAGAGCATCTGGC
GTCCCTGACCGATTCAGTGGCAGTGGATCTGGGACAGATTTCACTCCCACCATAAGCAGCCTCCAGGCTGAAGATGT
GGCCACTTATTACTGTCAGCAGTATTACAGTTGGCCTCTTACGTTCGGCCAGGGGACCAAGCTGGAGATCAAACGGG
ATGATGCTAAGCCACCTGCCTTCATCTTCCCACCGTCTTCTGAGGAGTTAAGCAGTGGAAGTGCCTCTGTCGTATGC
TTGGTGTATGGCTTCTACCCCAGTGGAGCCACTATCAACTGGAAAGTGGATGGTCTTGCCAAAACAAGTAGC
>HM176270.1 Equus caballus clone 1K108 immunoglobulin kappa light chain V-J
region mRNA, partial cds
ATGCTGACGCAGACGCAGGTCCTTATATCTGTGTTGCTCTGGGTCTCAGGAGCCTGTGGGGACGTCGTGATGACCCA
GTCTCCAGAGTCCTTGGCAGTGTCTCTAGGACAGAGGGTCGAGATGAAGTGCAAGGCCAGTCAGAGTGTAAGCCCCT
CCTTAGCTTGGTACCAGCAGAAACCAGGACAGGCTCCTAGGCGGCTCATCCAACTTGCATCCAGCAGAGATCCTGGG
GTCCCTGACCGATTCAGTGGCAGTGGATCTGGGACGGATTTCACTCTCACCATCAGCAGCCTCCAGGCTGAAGATGT
GGCCGTTTATTACTGTATGCAGTTTTTTGTCCTTCCTTTCACGTTCGGCCAGGGGACCAAGCTGGAGATCAAACGGG
ATGATGCTAAGCCATCTGCCTTCATCTTCCCACCGTCTTCTGAGGAGTTAAGCAGTGGAAGTGCCTCTGTCGTATGC
TTGGTGTATGGCTTCTACCCCAGTGGAGCCACTATCAACTGGAAAGTGGATGGTCTTGCCAAAACAAGTAGC
>HM176269.1 Equus caballus clone 1K351 immunoglobulin kappa light chain V-J
region mRNA, partial cds
ATGCTGACGCAGACGCAGGTCCTTATATCTGTGTTGCTCTGGGTCTCAGGAGCCTGTGGGGACGTCGTGATGACCCA
GTCTCCAGAGTCCTTGGCAGTGTCTCTAGGACAGAGGGTCGAGATGCAGTGCAAGGCCAGTCAGAGTGTACGCAGCA
ACTTAGCTTGGTACCAGCAGAAACCAGGACAGGCTCCTAAGGAGCTCATCTATGGTGCATCCAAGAGACGTTCTGGG
GTCCCTGACCGGTTCAGTGGCAGTGGATCTGGGACGGATTTCACTCTCACCATCAGCAGCCTCCAGGCTGAAGATGT
GGCCATTTATTACTGTATGCAGAATAATAATGTTCCATACGCGTTCGGCCAAGGGACCAAGCTGGAGATCAAAAGGG
ATGATGCTAAGCCATCTGCCTTCATCTTCCCACCGTCTTCTGAGGAGTTAAGCAGTGGAAGTGCCTCTGTCGTATGC
TTGGTGTATGGCTTCTACCCCAGTGGAGCCACTATCAACTGGAAAGTGGATGGTCTTGCCAAAACAAGTAGC
>HM176268.1 Equus caballus clone 1K373 immunoglobulin kappa light chain V-J
region mRNA, partial cds
ATGCTGACGCAGACGCAGGTCCTTATATCTGTGTTGCTCTGGGTCTCAGGAGCCTGTGGGGACGTCGTGATGACCCA
GTCTCCATCATCCTTGGCAGTGTCTCTAGGACAGAGGGTCGAGATGAAGTGCAGGGCCAGTCAGAGTGTTAGCAAAT
ATTTACATTGGTACCAGCAGAAACCTGGACAGGCTCCTAAGCGGATCATCTATCAGGCAGAGAAGAGACCATCTGGG
```

GTCCCTGACCGATTCAGTGGCAGTCGATCTGGGACGGATTTCACTCTCACCATCAGCAGCCTCCAGGCTGAAGATGC
GGCGGTTTATTATTGTATGCAGGGTGGTGTTAATCCATTCACGTTCGGCCAAGGGACCAAGCTGGAGATCAAAAGGG
ATGATGCTAAGCCATCTGCCTTCATCTTCCCACCGTCTTCTGAGGAGTTAAGCAGTGGAAGTGCCTCTGTCGTATGC
TTGGTGTATGGCTTCTACCCCAGTGGAGCCACTATCAACTGGAAAGTGGATGGTCTTGCCAAAACAAGTAGC
>HM176267.1 Equus caballus clone 1K133 immunoglobulin kappa light chain V-J
region mRNA, partial cds
ATGCTGACGCAGACGCAGGTCCTTATATCTGTGTTGCTCTGGGTCTCAGGAGCCTGTGGGGACGTCGTGATGACCCA
GTCTCCAGAGTCCTTGGCAGTGTCTCTAGGACAGAGGGTCGAGATGAAGTGCAGGGCCAGTCAGAGTGTTAGCAGCT
ACTTAGCTTGGTACCAGCAGAAACCAGGACAGGCTCCTAAGCGGCTCATCTATGCTGAGTCCAGCAGAGCATCTGGG
GTCCCTGACCGATTCAGTGGCAGTGGATCTGGGACGGATTTCACTCTCACCATCACTAGCCTCCAGGCTGAAGATGT
GGCCATTTATTACTGTATGCAGCATTATAACTATCCATTTACGTTCGGCCAAGGGACCAAGCTGGAGATCAAAAGGG
ATGATGCTAAGCCATCTGCCTTCATCTTCCCACCGTCTTCTGAGGAGTTAAGCAGTGGAAGTGCCTCTGTCGTATGC
TTGGTGTATGGCTTCTACCCCAGTGGAGCCACTATCAACTGGAAAGTGGATGGTCTTGCCAAAACAAGTAGC
>HM176266.1 Equus caballus clone 1K196 immunoglobulin kappa light chain V-J
region mRNA, partial cds
ATGCTGACGCAGACGCAGGTCCTTATATCTGTGTTGCTCTGGGTCTCAGGAGCCTGTGGGGACGTCGTGATGACCCA
GTCTCCAGAAACCTTGGCAGTGTCTCTAGGACAGAGGGTCGAGATGAAGTGTAAGGCCAGTCAGAGTGTTAGTACCT
GGTTAAATTGGTACCAGCAGAAACCAGGGCAGGCTCCTAAGCGGCTCATCGATGATGCATCCAGCAGAGCATTTGGG
GTCCCTGACCGATTCAGTGGCAGTGGATCTGGGACGGATTTCACTCTCACCATCAGCAGCCTCCAGGCTGAAGATGT
GGCCATTTACTACTGTATGCATTATTATAATAATCCATACATGTTCGGCCAAGGGACCAAGCTGGAGATCAAAAGGG
ATGATGCTAAGCCATCTGCCTTCATCTTCCCACCGTCTTCTGAGGAGTTAAGCAGTGGAAGTGCCTCTGTCGTATGC
TTGGTGTATGGCTTCTACCCCAGTGGAGCCACTATCAACTGGAAAGTGGATGGTCTTGCCAAAACAAGTAGC
>HM176265.1 Equus caballus clone 1K310 immunoglobulin kappa light chain V-J
region mRNA, partial cds
ATGCTGACGCAGACGCAGGTCCTTATATCTGTGTTGCTCTGGGTCTCAGGAGCCTGTGGGGACGTCGTGATGACCCA
GTCTCCAGAGTCCTTGGCAGTGTCTCTAGGACAGAGGGTCGAGATGAAGTGCAAGGCCAGTCAGAGTGTTAACACGT
ACTTAGCTTGGTACCAACAGAAACCAGGACAGGCTCCTAAGCGGCTCATCTATTCTGCATCCAAAAGAGCATCTGGG
GTCCCTGACCGATTCAGTGGCAGTGGATCTGGGACGGATTTCACTCTCACCATCAGCAGCCTCCAGGCTGAAGATGT
GGCCGGGTATTACTGTATGCAGCATGTTGTTGATCCCCTTACGTTCGGCCAGGGGACCAAGCTGGAGATCAAACGGG
ATGATGCTAAGCCATCTGCCTTCATCTTCCCACCGTCTTCTGAGGAGTTAAGCAGTGGAAGTGCCTCTGTCGTATGC
TTGGTGTATGGCTTCTACCCCAGTGGAGCCACTATCAACTGGAAAGTGGATGGTCTTGCCAAAACAAGTAGC
>HM176263.1 Equus caballus clone 1K182 immunoglobulin kappa light chain V-J
region mRNA, partial cds
ATGCTGACGCAGACGCAGGTCCTTATATCTGTGTTGCTCTGGGTCTCAGGAGCCTGTGGGGACGTCGTGATGACCCA
GTCTCCAGAGTCCTTGGCAGTGTCTCTGGGACAGAGGGTCGAGATGAAGTGCAAGGCCAGTCAGAGTGTTGAGAGAT
ACTTAGCTTGGTACCAAAAGAAACCAGGACAGGCTCCTAAGCGACTCATCTATTCTGCATCCAGCAGAGCATCTGGG
GTCCCTGGCCGGTTCAGTGGCAGTGGATCTGGGACGGATTTCACTCTCACCATCAGCAGCCTCCAGGCTGAAGATGT
GGCAGTTTATTACTGTATGCAGCATGTTAATGATCCCCTTACGTTCGGCCAGGGGACCAAGCTGGAGATCAAACGGG
ATGATGCTAAGCCATCTGCCTTCATCTTCCCACCGTCTTCTGAGGAGTTAAGCAGTGGAAGTGCCTCTGTCGTATGC
TTGGTGTATGGCTTCTACCCCAGTGGAGCCACTATCAACTGGAAAGTGGATGGTCTTGCCAAAACAAGTAGC
>HM176262.1 Equus caballus clone 1K317 immunoglobulin kappa light chain V-J
region mRNA, partial cds
ATGCTGACGCAGACGCAGGTCCTTATATCTGTGTTGCTCTGGGCCTCAGGAGCCTGTGGGGACGTCGTGATGACCCA
GTCTCCGGAGTCCTTGGCCCTGTCTCTAGGACAGAGGGTCGAGATGAAGTGCAGGGCCAGTCAGAATGTTGGTAGTT
ACGTTGCTTGGTACCAGCAGAAACCAGGACAGGCTCCTAAGCGGCTCATCTATGCTACATCCAAGAGGAGTGCTGGA
GTCCCTGACCGATTCAGTGGCGAAGCATTTGGGACGGATTTCACTCTCATCATCAGCAGCCTCCAGGCTGAAGATGT
GGCCATTTATTATTGTATGCAGCATTATCATGATCCGGTCACGTTCGGCCAGGGGACCAAACTGGAGATCAAACGGG
ATGATGCTAAGCCATCTGCCTTCATCTTCCCACCGTCTTCTGAGGAGTTAAGCAGTGGAAGTGCCTCTGTCGTATGC
TTGGTGTATGGCTTCTACCCCAGTGGAGCCACTATCAACTGGAAAGTGGATGGTCTTGCCAAAACAAGTAGC
>HM176261.1 Equus caballus clone 1K95 immunoglobulin kappa light chain V-J
region mRNA, partial cds
ATGCTGACGCAGACGCAGGTCCTTATATCTGTGTTGCTCTGGGTCTCAGGAGCCTGTGGGGACGTCGTGATGACTCA
GTCTCCAGAGTCCTTGGCAGTGTCTCTAGGACAGAGGGTCGAGATGAAGTGTAAGGCCAGTCAGAGTGTTGGTAGTG
ACTTGATTTGGTATCAACAGAAGCCTGGACAGGCTCTTAAGCGGCTCATCTATAATGGTTCCAGAAGACAGTCTGGG
GTCCCTGACCGATTCAGTGGCGATGGATCTGGGACGGAATTCACTCTCATCATCAGCAGCCTCCAGTCTGAGGACGT

GGCCGACTATTATTGTATGCAACATTATACAAATCCGATTACGTTCGGCCAGGGGACCAAACTGGAGATCAAACGAG
ATGATGCTAAGCCATCTGCCTTCATCTTCCCACCGTCTTCTGAGGAGTTAAGCAGTGGAAGTGCCTCTGTCGTATGC
TTGGTGTATGGCTTCTACCCCAGTGGAGCCACTATCAACTGGAAAGTGGATGGTCTTGCCAAAACAAGTAGC
>HM176260.1 Equus caballus clone 1K63 immunoglobulin kappa light chain V-J region mRNA, partial cds
ATGCTGACGCAGACGCAGGTCCTTATATCTGTGTTGTTCTGGGTCTCAGGAGCCTGTGGGGACGTCGTGATGACCCA
GTCTCCAGAGTCCTTGGCAGTGTCTCTAGGACAGAGGGTCGAGATGAAGTGCAAGGCCAGTCAGAGTGTTAGCAGCG
AATTATCTTGGTACCAGCAGAAACCAGGACAGGCTCCTAAGCGGCTCATCGTTACTGCATCCAGCAGAAGGCCTGGG
GTCCCTGACCGATTCAGTGGCAGTGGATCTGGGACGGATTTCACTCTCACCATCAGCAGCCTCCAGGCTGAAGATGT
GGCCATTTATTACTGTATGCAGCATTATAATAATCCGTTTACGTTCGGCCAGGGGACCAAGCTGGAGATCAAACGGG
ATGATGCTAAGCCATCTGCCTTCATCTTCCCACCGTCTTCTGAGGAGTTAAGCAGTGGAAGTGCCTCTGTCGTATGC
TTGGTGTATGGCTTCTACCCCAGTGGAGCCACTATCAACTGGAAAGTGGATGGTCTTGCCAAAACAAGTAGC
>HM176259.1 Equus caballus clone 1K107 immunoglobulin kappa light chain V-J region mRNA, partial cds
ATGCTGACGCAGACGCAGGTCCTTATATCTGTGTTGCTCTGGGTCTCAGGAGCCTGTGGGGACGTCGTGATGACCCA
GTCTCCAGAGTCCTTGGCAGTGTCTCTAGGACAGAGGGTCGAGATGAAGTGCAAGGCCAGTCAGAGTGTTGATAAAT
ACTTAGCTTGGTACCAGCAGAAACCAGGACAGGCTCCTAAGCGGCTCATCTATGGTGTATCCAGCAGAGCATCTGGG
GTCCCTGACCGATTCGGTGGCAGTGGATCTGGGACGGATTTCACTCTCACCATCAGCAGCCTCCAGGCTGAAGATGT
GGCAGTTTATTACTGTATGCAGCATAATGACTATCCGTTTACGTTAGGCCAGGGGACCAAGCTGGAGATCAAACGGG
ATGATGCTAAGCCATCTGCCTTCATCTTCCCACCGTCTTCTGAGGAGTTAAGCAGTGGAAGTGCCTCTGTCGTATGC
TTGGTGTATGGCTTCTACCCCAGTGGAGCCACTATCAACTGGAAAGTGGATGGTCTTGCCAAAACAAGTAGC
>HM176258.1 Equus caballus clone 1K83 immunoglobulin kappa light chain V-J region mRNA, partial cds
ATGCTGACGCAGACGCAGGTCCTTATATCTGTGTTGCTCTGGGTCTCAGGAGCCCGCGGGGACGTCGTGATGACCCA
GTCTCCAGAGTCCTTGGCAGTGTCTCTGGGACAGAGGGTCGAGATGAAGTGCAAGGCCAGTCAGACTATTAGCACGT
ACTTGAGTTGGTTTCAACAGAAACCGGGACAGGCTCCTGAGCGGCTCATCGTTGATGCATCCAGCAGAGAGGTTGGG
GTCCCTGACCGATTCAGTGGCAGTGGATCTGGGACGGATTTCACTCTCACCATCAGTAATCTGCAGGCTGAAGATGT
GGCCGTTTATTACTGTATGCAGGGTTATGACGATCCGCTTACGTTCGGCCAGGGGACCAAGCTGGAGATCAAACGGG
ATGATGCTAAGCCATCTGCCTTCATCTTCCCACCGTCTTCTGAGGAGTTAAGCAGTGGAAGTGCCTCTGTCGTATGC
TTGGTGTATGGCTTCTACCCCAGTGGAGCCACTATCAACTGGAAAGTGGATGGTCTTGCCAAAACAAGTAGC
>HM176257.1 Equus caballus clone 1K223 immunoglobulin kappa light chain V-J region mRNA, partial cds
ATGCTGACGCAGACGCAGGTCCTTATATCTGTGTTGCTCTGGGTCTCAGGAGCCTGTGGGGACGTCGTGATGACCCA
GTCTCCAGAGTCCTTGGTACAGTCTCTAGGACAGAAGGTCGAGATGAAGTGCAAGGCCAGTCAGAGTATTAGCACGT
TCTTGTCCTGGTACCAGCAGAAACCAGGACAGGCTCCTAAGCGGCTCATCTATTTCGTATCCATCAGAGCGCCTGGG
GTCCCTGACCGATTCAGTGGCAGTGGAACTGGGACGGATTTCACTCTCACCATCAGCAGCCTCCAGGCTGAAGATGT
GGCCATTTATTACTGTATGCAAGGTTATGCGTATCCGCTTACGTTCGGCCAGGGGACCAAGCTGGAGATCAAACGGG
ATGATGCTAAGCCATCTGCCTTCATCTTCCCACCGTCTTCTGAGGAGTTAAGCAGTGGAAGTGCCTCTGTCGTATGC
TTGGTGTATGCCTTCTACCCCAGTGGAGCCACTATCAACTGGAAAGTGGATGGTCTTGCCAAAACAAGTAGC
>HM176256.1 Equus caballus clone 1K240 immunoglobulin kappa light chain V-J region mRNA, partial cds
ATGCTGACGCAGACGCAGGTCCTTATATCTGTGTTGCTCTGGGTCTCAGGAGCCTGTGGGGACGTCGTGATGACCCA
GTCTCCAGAGTCCTTGGCAGTGTCTCTAGGACAGAGGGTCGAGATGAAGTGCAAGGCCAGTCAGAGTGTTGGCAGCC
AGTTAAGTTGGTACCAGCAGAAACCAGGACAGGCTCCTAAGCGGCTCATCTATCATTCATCCAGCAGAGCATCTGGG
GTCCCTGACCGATTCAGTGGCAGTGGATCTGGGACGGATTTCACTCTCACCATCAGCAGCCTCCAGGCTGAAGATGT
GGCCATTTATTACTGTATGCACAATTATGATTGGCCGCTTACGTTCGGCCAGGGGACCAAGCTGGAGATCAAACGGG
ATGATGCTAAGCCATCTGCCTTCATCTTCCCACCGTCTTCTGAGGAGTTAAGCAGTGGAAGTGCCTCTGTCGTATGC
TTGGTGTATGGCTTCTACCCCAGTGGAGCCACTATCAACTGGAAAGTGGATGGTCTTGCCAAAACAGGTAGC
>HM176255.1 Equus caballus clone 1K45 immunoglobulin kappa light chain V-J region mRNA, partial cds
ATGCTGACGCAGACGCAGGTCCTTATATCTGTGTTGCTCTGGGTCTCAGGAGCCTGTGGGGACGTCGTGATGACCCA
GTCTCCAGAGTCCTTGGCAGTGTCTCTAGGACAGAGGGTCGAGATGAGGTGCAAGGCCAGTCAGAGTGTTAACACGT
GGGTAGTTTGGTACCAGCAGAAACCAGGACAGGCTCCTAAGCGGCTCATCTATCGTGCATCCAGCAGAGCATCTGGG
GTCCCTGACCGATTCAGTGGCAGTGGATCTGGGACGGATTTCACTCTCACCATCAGCAGCCTCCAGGCTGAAGATGT
GGCCATTTATTACTGTATGCAGCATAATGTAAATCCGCTTACGTTCGGCCAGGGGACCAAGCTGGAGATCGCACGGG

ATGATGCTAAGCCATCTGCCTTCATCTTCCCACCGTCTTCTGAGGAGTTAAGCAGTGGAAGTGCCTCTGTCGTATGC
TTGGTGTATGGCTTCTACCCCAGTGGAGCCACTATCAACTGGAAAGTGGATGGTCTTGCCAAAACAAGTAGC
>HM176253.1 Equus caballus clone 1K313 immunoglobulin kappa light chain V-J region mRNA, partial cds
ATGCTGACGCAGACGCAGGTCCTTATATCTGTGTTGCTCTGGGTCTCAGGAGCCTGTGGGGACGTCGTGATGACCCA
GTCTCCAGAGTCCTTGGCAGTGTCTCTAGGACAGAGGGTCGACATGAAGTGCAAGGCCAGTCAGAGTGTGATCACCT
ACTTAGCTTGGTACCAGCAAAAACCAGGACAGGCTCCTAAGCGGCTCATCTGGGGTGCCTCCAACAGAGCGTCTGGG
GTCCCTGACCGATTCAGTGGCAGTGGATCTGGGACGGATTTCACTCTCACCATCAGCAGCCTCCAGGCTGAGGATGT
GGCCATTTATTACTGTATGCAGCACGCTACTCATCCGCTTACGTTCGGCCAGGGGACCAAGCTGGAGATCAGACGGG
ATGATGCTAAGCCATCTGCCTTCATCTTCCCACCGTCTTCTGAGGAGTTAAGCAGTGGAAGTGCCTCTGTCGTATGC
TTGGTGTATGGCTTCTACCCCAGTGGAGCCACTATCAACTGGAAAGTGGATGGTCTTGCCAAAACAAGTAGC
>HM176252.1 Equus caballus clone 1K269 immunoglobulin kappa light chain V-J region mRNA, partial cds
ATGCTGACGCAGACGCAGGTCCTTATATCTGTGTTGCTCTGGGTCTCAGGAGCCTGTGGGGACGTCGTGATGACCCA
GTCTCCAGAGTCCTTGGCAGTGTCTCTAGGACAGAGGGTCGAGATGAAGTGCAAGGCCAGTCAGAGTATTAGCAATT
TAGTTTGGTACCAGCAGAAACCAGGACAGGCTCCTAAGCGGCTCATCTATGCTGCATCCAGCAGACCATCTGGGGTC
CCTGACCGATTCAGTGGCAGTGGATCTGGGACGGATTTCACTCTCACCATCAGCAGCCTCCAGGCTGAAGATGTGGC
CATTTATTACTGTATGCAAGATTCTAATCATCCGCTTACGTTCGGCCAGGGGACCAAGCTGGAGATCAAACGGGATG
ATGCTAAGCCATCTGCCTTCATCTTCCCACCGTCTTCTGAGGAGTTAAGCAGTGGAAGTGCCTCTGTCGTATGCTTG
GTGTATGGCTTCTACCCCAGTGGAGCCACTATCAACTGGAAAGTGGATGGTCTTGCCAAAACAAGTAGC
>HM176250.1 Equus caballus clone 1K312 immunoglobulin kappa light chain V-J region mRNA, partial cds
ATGCTGACGCAGACGCAGGTCCTTATATCTGTGTTGCTCTGGGTCTCAGGAGCCTGTGGGGACGTCGTGATGACCCA
GTCTCCAGAGTCCTTGGCAGTGTCTCTAGGACAGAGGGTCGAGATGAAGTGCAAGGCCAGTCAGAGTGTTAGCGCTT
ACGTGGCTTGGTTCCAGCAGAAACCAGGACAGGCTCCTAAGCGGCTCATCTACAGAGCATCCAGCAGAGAATCTGGG
GTCCCTGACCGATTCAGTGGCAGTGGATCTGGGACGGATTTCACTCTCACCATCAGCAGCCTCCAGGCTGAAGATGT
GGCCATTTATTACTGTATGCAGCATTTTCATAAGCCGCTTACGTTCGGCCAGGGGACCAAGCTGGAGATCAAACGGG
ATGATGCTAAGCCATCTGCCTTCATCTTCCCACCGTCTTCTGAGGAGTTAAGCAGTGGAAGTGCCTCTGTCGTATGC
TTGGTGTATGGCTTCTACCCCAGTGGAGCCACTATCAACTGGAAAGTGGATGGTCTTGCCAAAACAAGTAGC
>HM176249.1 Equus caballus clone 1K242 immunoglobulin kappa light chain V-J region mRNA, partial cds
ATGCTGACGCAGACGCAGGTCCTTATATCTGTGTTGCTCTGGGTCTCAGGAGCCTGTGGGGACGTCGTGATGACCCA
GTCTCCAGAGTCCTTGGCAGTGTCTCTAGGACAGAGGGTCGAGATGAAGTGCAAGGCTAGTCAAGGTGTTGGCAGAT
ACTTAACTTGGTATCAGCAGAAACCAGGACAGGCTCCTAAGCGGCTCATCTATGCTGCATCCAACCGAGCATCTGGG
GTCCCTGACCGATTCAGTGGCAGTGGATCTGGGACGGATTTCACTCTCACCATCAGCAGCCTCCAGGCTGAAGATGT
GGCCATTTATTACTGTATGCAGAATTATAACAATCCGCTTACGTTCGGCCAGGGGACCAAGCTGGAGATCACGCGGG
ATGATGCTAAGCCATCTGCCTTCATCTTCCCACCGTCTTCTGAGGAGTTAAGCAGTGGAAGTGCCTCTGTCGTATGC
TTGGTGTATGGCTTCTACCCCAGTGGAGCCACTATCAACTGGAAAGTGGATGGTCTTGCCAAAACAAGTAGC
>HM176248.1 Equus caballus clone 1K102 immunoglobulin kappa light chain V-J region mRNA, partial cds
ATGCTGACGCAGACGCAGGTCCTTATATCTGTGTTGCTCTGGGTCTCAGGAGCCTGTGGGGACGTCGTTATGACCCA
GTCTCCAGAGTCCTTGGCAGTGTCTCTGGGACAGAGGGTCGAGGTGAAGTGTAAGGCCAGTCACAATATTGGCACTG
CCTTATCTTGGTACCAGCAGAAAGCAGGACAGGCTCCTAAGCGGCTCATCTATTTTGCATCCAGCAGAGCATCTGGG
GCCCCTGACCGATTCAGTGGCAGTGGATCTGGGACGGATTTCACTCTCACCATCAGCAGCCTCCAGGCTGAAGATGT
GGCCATTTATTATTGTATGCAAAATTCTAATTATCCGCTTACGTTCGGCCAGGGGACCAAGCTGGAGATCAAACGGG
ATGATGCTAAGCCATCTGCCTTCATCTTCCCACCGTCTTCTGAGGAGTTAAGCAGTGGAAGTGCCTCTGTCGTATGC
TTGGTGTATGGCTTCTACCCCAGTGGAGCCACTATCAACTGGAAAGTGGATGGTCTTGCCAAAACAAGTAGC
>HM176247.1 Equus caballus clone 1K143 immunoglobulin kappa light chain V-J region mRNA, partial cds
ATGCTGACGCAGACGCAGGTCCTTATATCTGTGTTGCTCTGGGTCTCAGGAGCCTGTGGGGACGTCGTGATGACCCA
GTCTCCAGAGTCCTTGGCAGTGTCTCTAGGACAGAGGGTCGAGATGGAGTGCAAGGCCAGCAAGAGTGTTAGCAACT
GGTTAGTTTGGTACCAGCAGAAACCAGGACAGGCTCCTAAGCAGCTCATCTATCGTGCATCCAGCAGAGCATCTGGG
GTCCCTGACCGCTTCAGTGGCAGTGGATCTGGGACGGATTTCACTCTCACCATCAGCAGCCTCCAGGCTGAAGATGT
GGCCACGTATTACTGTATGCAGAACAATGAGGCTCCGCTTACGTTCGGCCAGGGGACCAAGCTGGAGATCAAACGGG

ATGATGCTAAGCCATCTGCCTTCATCTTCCCACCGTCTTCTGAGGAGTTAAGCAGTGGAAGTGCCTCTGTCGTATGC
TTGGTGTATGGCTTCTACCCCAGTGGAGCCACTATCAACTGGAAAGTGGATGGTCTTGCCAAAACAAGTAGC
>HM176246.1 Equus caballus clone 1K29 immunoglobulin kappa light chain V-J
region mRNA, partial cds
ATGCTGACGCAGACGCAGGTCCTTATATCTGTGTTGCTCTGGGTCTCAGGAGCCTGTGGGGACGTCGTGATGACCCA
GTTTCCAGAGTCCTTGGCAGTGTCTCCAGGACAGAGGGTCGAGATGAAGTGCAAGGCCAGTCAGCGTATTAGTGCCT
ACCACTTAGATTGGTACCAGCAGAAACCAGGACAGGCTCCTAAGAGGCTCATCTATGCTGCATCCAACAGACAATCT
GGGGTCCCTGACCGATTCAGTGGCAGTGGATCTGGGACGGATTTCACTTTCACCATCAGCAGCCTCCAGGCTGAGGA
TGTGGCCATTTATTACTGTATGCAGTATTCCAGTAATCCGCTCACGTTCGGCCAGGGGACCAAGCTGGAGATCAAAC
GGGATGATGCTAAGCCATCTGCCTTCATCTTCCCACCGTCTTCTGAGGAGTTAAGCAGTGGAAGTGCCTCTGTCGTA
TGCTTGGTGTATGGCTTCTACCCCAGTGGAGCCACTATCAACTGGAAAGTGGATGGTCTTGCCAAAACAAGTAGC
>HM176245.1 Equus caballus clone 1K278 immunoglobulin kappa light chain V-J
region mRNA, partial cds
ATGCTGACGCAGACGCAGGTCCTTATATCTGTGTTGCTCTGGGTCTCAGGAGCCTGTGGGGGCGTCGTGATGACCCA
GTCTCCAGAGTCCTTGGCAGTGTCTCTAGGACAGAGGGTCGAGATGAAGTGCAAGGCCAGTCAGAGTGTTAGCATAT
ACTTAGCTTGGTACCAGCAGAAACCAGGACAGGCTCCTAAGCGGCTCATCTATGACGCATCCAGTAGAGCATCTGGA
GTCCCTGACCGATTCAGTGGCAGTGGATCTGGGACGGATTTCACTCTCACCATCAGCAGCCTCCAGGCTGAAGATGT
GGCCATCTATTACTGTATGACACATATTAATAATCTTCCGAAGTTCGGCCAAGGGACCAAGCTGGAGATCAAAAGGG
ATGATGCTAAGCCATCTGCCTTCATCTTCCCACCGTCTTCTGAGGAGTTAAGCAGTGGAAGTGCCTCTGTCGTATGC
TTGGTGTATGGCTTCTACCCCAGTGGAGCCACTATCAACTGGAAAGTGGATGGTCTTGCCAAAACAAGTAGC
>HM176244.1 Equus caballus clone 1K232 immunoglobulin kappa light chain V-J
region mRNA, partial cds
ATGCTGACGCAGACGCAGGTCCTTATATCTGTGTTGCTCTGGGTCTCAGGAGCCTGTGGGGACGTCGTGATGACCCA
GTCTCCAGAGTCCTTGGCAGTGTCTCTAGGACAGAGGGTCGAGATGAAGTGCAAGGCCAGTCAGAGTGTTAGCATAT
ACTTAGCTTGGTACCAGCAGAAACCAGGACAGGCTCCTAAGCGGCTCATCTATGACGCATCCAGTAGAGCATCTGGA
GTCCCTGACCGATTCAGTGGCAGTGGATCTGGGACGGATTTCACTCTCACCATCGGCAGCCTCCAGGCTGAAGATGT
GGCCATCTATTACTGTATGACACATATTAATAATCTTCCGAAGTTCGGCCAAGGGACCAAGCTGGAGATCAAAAGGG
ATGATGCTAAGCCATCTGCCTTCATCTTCCCACCGTCTTCTGAGGAGTTAAGCAGTGGAAGTGCCTCTGTCGTATGC
TTGGTGTATGGCTTCTACCCCAGTGGAGCCACTATCAACTGGAAAGTGGATGGTCTTGCCAAAACAAGTAGC
>HM176243.1 Equus caballus clone 1K349 immunoglobulin kappa light chain V-J
region mRNA, partial cds
ATGCTGACGCAGACGCAGGTCCTTATATCTGTGTTGCTCTGGGTCTCAGGAGCCTGTGGGGACGTCGTGATGACCCA
GTCTCCAGAGTCCTTGGCAGTGTCTCTGGGACAGAGGGTCGAGATGAAGTGCAAGGCCAGTCAGAGTGTTAGTAGCG
AATTAGGTTGGTACCAGCAGAAACCAGGACAGGCTCCTAAGGGGCTCATCTTTGCTGCATCCACTAGAGCATCTGGG
GTCCCTGACCGATTCAGTGGCAGTGGATCTGGGACGGATTTCACTCTCACTATCAGCAGCCTCCAGGCTGAAGATGT
GGCCATTTATTACTGTATGCAGCATAGAATTGGCGCACCTACGTTCGGCCAGGGGACCAAGCTGGAGATCACACGGG
ATGATGCTAAGCCATCTGCCTTCATCTTCCCACCGTCTTCTGAGGAGTTAAGCAGTGGAAGTGCCTCTGTCGTATGC
TTGGTGTATGGCTTCTACCCCAGTGGAGCCACTATCAACTGGAAAGTGGATGGTCTTGCCGAAACAAGTAGC
>HM176242.1 Equus caballus clone 1K327 immunoglobulin kappa light chain V-J
region mRNA, partial cds
ATGCTGACGCAGACGCAGGTCCTTATATCTGTGTTGCTCTGGGTCTCAGGAGCCTGTGGGGACGTCGTGATGACCCA
GTCTCCAGAGTCCTTGGCAGTGTCTCGCGGAGAGAGGGTCGAGATGAAGTGCAAGGCCAGTCAGAGTGTTATCAACA
CTTACTTAGTTTGGTACCAGCAGAAACCAGGACAGGCTCCTAAGCGGCTCATCTATGCTACATCCAGCAGAGCATCT
GGGGTCCCTGACCGATTCAGTGGCAGTGGATCTGGGACGGATTTCACTCTCACCATCAGCAGCCTCCAGGCTGAAGA
TGTGGCCATTTATTACTGTATGCAGCATCATCATCATCCTCCTACGTTCGGCCAGGGGACCAAGCTGGAGATCAAAC
GGGATGATGCTAAGCCATCTGCCTTCATCTTCCCACCGTCTTCTGAGGAGTTAAGCAGTGGAAGTGCCTCTGTCGTA
TGCTTGGTGTATGGCTTCTACCCCAGTGGAGCCACTATCAACTGGAAAGTGGATGGTCTTGCCAAAACAAGTAGC
>HM176241.1 Equus caballus clone 1K69 immunoglobulin kappa light chain V-J
region mRNA, partial cds
ATGCTGACGCAGACGCAGGTCCTTATATCTGTGTTGCTCTGGGTCTCAGGAGCCTGTGGGGACGTCGTGATGACCCA
GTCTCCGGAGTCCGTGGCAGTGTCTCTAGGACAGAGGGTCGAGATGAAGTGCAAGGCCAGCCAGAGTGTTAGGGAAT
ACTTAGCTTGGTACCAGCAGAAACCAGGTCAGGCTCCTAAGCGGCTCATCTATGCTGCATCCAGCAGAACATCTGGG
GTCCCTGACCGATTCAGTGGCAGTGGATCTGGGACGGATTTCACTCTCACCATCAGCAGCCTCCAGGCTGAAGATGT
GGCCATTTATTACTGTATGCAGCATTTGCCATATCCTCGGACGTTCGGCCAAGGGACCAAGGTGGAGATCAAAAGGG
ATGATGCTAAGCCATCTGCCTTCATCTTCCCACCGTCTTCTGAGGAGTTAAGCAGTGGAAGTGCCTCTGTCGTATGC
TTGGTGTATGGCTTCTACCCCAGTGGAGCCACTATCAACTGGAAAGTGGATGGTCTTGCCAAAACAAGTAGC

>HM176240.1 Equus caballus clone 1K213 immunoglobulin kappa light chain V-J region mRNA, partial cds
ATGCTGACACAGACGCAGGTCCTACTATCTGTGTTGCTCTGGGTCTCAGGAGCCTGTGGGGACGTCGTGATGACCCA
GTCTCCAGAGTCCTTGGCAGTGTCTCTAGGACAGAGGGTCGAGATGAAGTGCAAGGCCAGTCAGAATATTAACAGTA
ACTTAGATTGGTACCAGCAGAAACCAGGACAGGTTCCTAAGCGCATCATCTATACTGCATCCAGCAGAGCATCTGGG
GTCCCTGACCGGTTCAGTGGCAGTGGATCTGGGACGGATTTCACTCTCACCATCAGCAGCCTCCAGGCTGAAGATGT
GGCCATTTATTACTGTATGCAGCGCAGTGCATTCCCTCTTACGTTCGGCCAGGGGACCAAACTGGAGATCAAACGGG
ATGATGCTAAGCCATCTGCCTTCATCTTCCCACCGTCTTCTGAGGAGTTAAGCAGTGGAAGTGCCTCTGTCGTATGC
TTGGTGTATGGCTTCTACCCCAGTGGAGCCACTATCAACTGGAAAGTGGATGGTCTTGCCAAAACAAGTAGC
>HM176239.1 Equus caballus clone 1K110 immunoglobulin kappa light chain V-J region mRNA, partial cds
ATGCTGACGCAGACGCAGGTCCTTATATCTGTGTTGCTCTGGGTCTCAGGAGCCTGTGGGGACGTCGTGATGACCCA
GTCTCCAGAGTCCTTGGCAGTGTCTCTAGGACAGAGGGTCGAGATGAAGTGCAAGGCCAGTCAGAGTGTTAGCGCCT
ACTTAGCTTGGTTCCAGCAGAAACCAGGACAGGCTCCTAAGCGGCTCATCTATTATGGATCCAAGAGAGCATCTGGG
GTCCCTGACCGATTCAGTGGCAGTGGATCTGGGACGGATTTCACTCTCACCATCAGCAGCCTCCAGGCTGAAGATGT
GGCCATTTATTACTGTATGCAGAATTATAATAATCCTCTTACGTTCGGCCAGGGGACCAAGCTGGAGATCAAACGGG
ATGATGCTAAGCCATCTGCCTTCATCTTCCCACCGTCTTCTGAGGAGTTAAGCAGTGGAAGTGCCTCTGTCGTATGC
TTGGTGTATGGCTTCTACCCCAGTGGAGCCACTATCAACTGGAAAGTGGATGGTCTTGCCAAAACAAGTAGC
>HM176238.1 Equus caballus clone 1K119 immunoglobulin kappa light chain V-J region mRNA, partial cds
ATGCTGACGCAGACGCAGGTCCTTATATCTGTGTTGCTCTGGGTCTCAGGAGCCTGTGGGGAGATCGTGATGACCCA
GTCTCCAGAGTCCTTGGCAGTGTCTCTAGGACAGAGGGTCGAGATGAAGTGCAAGGCCAGTCAGAGTATTGGCAGCT
ACTTAGCTTGGTACCAGCAGAAACCAGGACAGGCTCCTAAGCGACTCATCTATCCTGCATCCACTAGAGCATCTGGG
GTCCCTGACCGATTCAGTGGCAGTGGATCTGGGACGGATTTCACTCTCACCATCAGCAGCCTCCAGGCTGAAGATGT
GGCCGATTATTACTGTATGCAATGTAATAGTAGTCCTCTTACGTTCGGCCAGGGGACCAAGCTGGAGATCAAACGGG
ATGATGCTAAGCCATCTGCCTTCATCTTCCCACCGTCTTCTGAGGAGTTAAGCAGTGGAAGTGCCTCTGTCGTATGC
TTGGTGTATGGCTTCTACCCCAGTGGAGCCACTATCAACTGGAAAGTGGATGGTCTTGCCAAAACAAGTAGC
>HM176236.1 Equus caballus clone 1K2 immunoglobulin kappa light chain V-J region mRNA, partial cds
ATGCTGACGCAGACGCAGGTCCTTATATCTGTGTTGCTCTGGGTCTCAGGAGCCTGTGGGGACGTCGTGATGACCCA
GTCTCCAGAGTCCTTGGCAGTGTCTCTAGGACAGAGGGTCGAGATGAAGTGCAAGGCCAGTCAGACTGTTAGCAGTC
ACTTAGCTTGGTACCAGCAGAAACCAGGACAGGCTCCTAAGCGGCTCATCTATGCTGCATCCAGCAGAGCATCTGGG
GTCCCTGACCGATTCAGTGGCAGTGGATCTGGGACGGATTTCACTCTCACCATCAGCAGCCTCCAGGCTGAAGATGT
GGCCATTTATTATTGTATGCAGCATTATACCTATCCTCTTACGTTCGGCCAGGGGACCAAGCTGGAGATCAAACGGG
ATGATGCTAAGCCATCTGCCTTCATCTTCCCACCGTCTTCTGAGGAGTTAAGCAGTGGAAGTGCCTCTGTCGTATGC
TTGGTGTATGGCTTCTACCCCAGTGGAGCCACTATCAACTGGAAAGTGGATGGTCTTGCCAAAACAAGTAGC
>HM176235.1 Equus caballus clone 1K361 immunoglobulin kappa light chain V-J region mRNA, partial cds
ATGCTGACGCAGACGCAGGTCCTTATATCTGTGTTGCTCTGGGTCTCAGGAGCCTGTGGGGACGTCGTGATGACCCA
GTCTCCAGAGTCCTTGGCAGTGTCTCTAGGACAGAGGGTCGAGATGAAGTGCAAGGCCAGTCAGACTATTAGCAGCC
GCTTAGCTTGGTACCAGCAGAAACCAGGACAGGCTCCTAAGCGGCTCATCTATGCTGCATCCAGCAGAGCATCTGGG
GTCCCTGATCGATTCAGTGGCAGTCGATCTGGGACGGATTTCACTCTCACCATCAGCAGCCTCCAGGCTGAAGATGT
GGCCGTTTATTACTGTATGCAATCGAATTACCATCCTCTTACGTTCGGCCAGGGGACCAAGCTGGAGATCAAACGGG
ATGATGCTAAGCCATCTGCCTTCATCTTCCCACCGTCTTCTGAGGAGTTAAGCAGTGGAAGTGCCTCTGTCGTATGC
TTGGTGTATGGCTTCTACCCCAGTGGAGCCACTATCAACTGGAAAGTGGATGGTCTTGCCAAAACAAGTAGC
>HM176234.1 Equus caballus clone 1K364 immunoglobulin kappa light chain V-J region mRNA, partial cds
ATGCTGACGCAGACGCAGGTCCTTATATCTGTGTTGCTCTGGGTCTCAGGAGCCTGTGGGGACGTCGTGATGACCCA
GTCTCCAGAGTCCTTGGCAGTGTCTCTAGGACAGAGGGTCGAGATGAAGTGCAGAGCCAGTCAGAGTATTGGACGAG
CGTTAGATTGGTACCAGCAGAAACCAGGACAGGCTCCTAAGCGGCTCATCTATACTGCATCCAGTAGACCATCTGGG
GTCCCTGACCGATTCAGTGGCAGTGGATCTGGGACGGATTTCACTCTCACCATCAGCAGCCTCCAGGCTGAAGATGT
GGCCATTTATTACTGTATGCAGCGTACTACGTTTCCTCTTACGTTCGGCCAGGGGACCAAGCTGGAGATCAAACGGG
ATGATGCTAAGCCATCTGCCTTCATCTTCCCACCGTCTTCTGAGGAGTTAAGCAGTGGAAGTGCCTCTGTCGTATGC
TTGGTGTATGGCTTCTACCCCAGTGGAGCCACTATCAACTGGAAAGCGGATGGTCTTGCCAAAACAAGTAGC

>HM176232.1 Equus caballus clone 1K380 immunoglobulin kappa light chain V-J region mRNA, partial cds
ATGCTGACGCAGACGCAGGTCCTTATATCTGTGTTGCTCTGGGTCTCAGGAGCCTGTGGGGACGTCGTGATGACCCA
GTCTCCAGAGTCCTTGGCAGTGTCTCTAGGACAGAGGGTCGAGATGAAGTGCAAGGCCAGTCAGAGTGTTAGGACGT
ACTTAGCTTGGTACCAGCAGAAACCAGGACAGGCTCCTAAGCGGCTCATCTATGCTGCATCCAGCAGAGCATCTGGG
GTCCCTGACCGATTCAGTGGCAGTGGATCTGGGACGGATTTCACTCTCACCATCAGCGACCTCCAGGCTGAAGATGT
GGCCATTTATTACTGTATGCAGAATTATGTTGCTCCTCTTACGTTCGGCCAGGGGACCAAGCTGGAGATCGAACGGG
ATGATGCTAAGCCATCTGCCTTCATCTTCCCACCGTCTTCTGAGGAGTTAAGCAGTGGAAGTGCCTCTGTCGTATGC
TTGGTGTATGGCTTCTACCCCAGTGGAGCCACTATCAACTGGAAAGTGGATGGTCTTGCCAAAACAAGTAGC
>HM176231.1 Equus caballus clone 1K320 immunoglobulin kappa light chain V-J region mRNA, partial cds
ATGCTGACGCAGACGCAGGTCCTTATATCTGTGTTGCTCTGGGTCTCAGGAGCCTGTGGGGACGTCGTGATGACCCA
GTCTCCAGAGTCCTTGGCAGTGTCTCTAGGACAGAGGGTCGAGATGAAGTGCAAGGCCAGTCAGAGTGTTAGCAACA
ACTTAGCTTGGTACCAGCAGAAACCAGGACAGGCTCCTAAGCGGCTCATCTATGCTGCATCCAGCAGAGCATCTGGG
GTCCCTGACCGATTCAGTGGCAGTGGATCTGGGACGGATTTCACTCTCACCATCAGCAGCCTCCAGGCTGAAGATGT
GGCCATTTATTACTGTATGCAGCATTATGATAATCCTCTTACGTTCGGCCAGGGGACCAAGCTGGAGATCAAACGGG
ATGATGCTAAGCCATCTGCCTTCATCTTCCCACCGTCTTCTGAGGAGTTAAGCAGTGGAAGTGCCTCTGTCGTATGC
TTGGTGTATGGCTTCTACCCCAGTGGAGCCACTATCAACTGGAAAGTGGATGGTCTTGCCAAAACAAGTAGC
>HM176228.1 Equus caballus clone 1K309 immunoglobulin kappa light chain V-J region mRNA, partial cds
ATGCTGACGCAGACGCAGGTCCTTATATCTGTGTTGCTCTGGGTCTCAGGAGCCTGTGGGGACGTCGTGATGACCCA
GACTCCAGAGTCCTTGGCAGTGTCTCTAGGACAGAGGGTCGAGATGAAGTGCAAGGCCAGTCAGAGTATTAACTTCT
ACCTAGCTTGGTATCAGCAGAAACCAGGACAGGCTCCTAAGCGGCTCATTTATGCTGCATCCAGCAGAGCATCTGGG
GTCCCTGACCGATTCAGTGGCAGTGGATCTGGGACGGATTTCACTCTCACCATCAGCAGCCTCCAGGCTGAAGATGT
GGCCATTTATTACTGTATGCAGTATGAGCATAATCCTCTTACGTTCGGCCAGGGGACCAAGCTGGAGATCAAACGGG
ATGATGCTAAGCCATCTGCCTTCATCTTCCCACCGTCTTCTGAGGAGTTAAGCAGTGGAAGTGCCTCTGTCGTATGC
TTGGTGTATGGCTTCTACCCCAGTGGAGCCACTATCAACTGGAAAGTGGATGGTCTTGCCAAAACAAGTAGC
>HM176227.1 Equus caballus clone 1K212 immunoglobulin kappa light chain V-J region mRNA, partial cds
ATGCTGACGCAGACGCAGGTCCTTATATCTGTGTTGCTCTGGGTCTCAGGAGCCTGTGGGGACGTCGTGATGACCCA
GTCTCCAGAGTCCTTGGCAGTGTCTCTAGGACAGAGGGTCGAGATGAAGTGCAAGGCCAGTCAGAGTATTGTCAGCA
AATTATCTTGGTACCAGCAGAAACCAGGACAGGCTCCTAAGCGGCTCATCTATGATGCATCCAGCAGATCATCTGGG
GTCCCTGACCGATTCAGTGGCAGTGGATCTGGGACGGATTTCACTCTCACCATCAGCAGCCTCCAGGCTGAAGATGT
GGCCATTTATTACTGTATGCAGTATGATAATAATCCTCTTACGTTCGGCCAGGGGACCAAGCTGGAGATCAAACGGG
ATGATGCTAAGCCATCTGCCTTCATCTTCCCACCGTCTTCTGAGGAGTTAAGCAGTGGAAGTGCCTCTGTCGTATGC
TTGGTGTATGGCTTCTACCCCAGTGGAGCCACTATCAACTGGAAAGTGGATGGTCTTGCCAAAACAAGTAGC
>HM176226.1 Equus caballus clone 1K209 immunoglobulin kappa light chain V-J region mRNA, partial cds
ATGCTGACGCAGACGCAGGTCCTTATATCTGTGTTGCTCTGGGTCTCAGGAGCCTGTGGGGACGTCGTGATGACCCA
GTCTCCAGAGCTCTTGGCAGTGTCTCTAGGACAGAGGGTCGAGATGAAGTGCAAGGCCAGTCAGAGTATAGGTGTGT
GGGTAACTTGGTACCAGCAGAAACCAGGACAGGCTCCTAAGCGGCTCATCTATACTGCATCCAACAGAGCATCTGGG
GTCCCAGACCGATTCAGTGGCAGCGGATCTGGGACGGATTTCACTCTCACCATCAGCAGCCTCCAGGCTGAAGATGG
GGCCATTTATTACTGTATGCAGCATTCTACCTATCCTCTTACGTTCGGCCAGGGGACCAAGCTGGAGATCAAACGGG
ATGATGCTAAGCCATCTGCCTTCATCTTCCCACCGTCTTCTGAGGAGTTAAGCAGTGGAAGTGCCTCTGTCGTATGC
TTGGTGTATGGCTTCTACCCCAGTGGAGCCACTATCAACTGGAAAGTGGATGGTCTTGCCAAAACAAGTAGC
>HM176225.1 Equus caballus clone 1K98 immunoglobulin kappa light chain V-J region mRNA, partial cds
ATGCTGACGCAGACGCAGGTCCTTATATCTGTGTTGCTCTGGGTCTCAGGAGCGTGTGGGGACGTCGTGATGACCCA
GTCTCCAGAGTCCTTGGCAGTGTCTCTGGGACAGAGGGTCGAGATGAAGTGCGTGGCCAGTCAGAGTGTTAGCAGCT
ACTTAGCTTGGTACCAGCAGAAACCAGGACAGGCTCCTAAGCGGCTCATCTATGCTGCATCCATCAGATCATCTGGG
GTCCCTGACCGATTCAGTGGCAGTGGATCTGGGACGGATTTCGCTCTCACCATCAGCAGCCTCCAGGCTGAAAATGT
GGCCATTTATTTCTGTATGCAGAATAGCGAGTGGCCTCTTACGTTCGGCCAGGGGACCAAGCTGGAGATCAAACGGG
ATGATGCTAAGCCATCTGCCTTCATCTTCCCACCGTCTTCTGAGGAGTTAAGCAGTGGAAGTGCCTCTGTCGTATGC
TTGGTGTATGGCTTCTACCCCAGTGGAGCCACTATCAACTGGAAAGTGGATGGTCTTGCCAAAGCAAGTAGC
>HM176224.1 Equus caballus clone 1K154 immunoglobulin kappa light chain V-J region mRNA, partial cds

ATGCTGACGCAGACGCAGGTCCTTATATCTGTGTTGCTCTGGGTCTCAGGAGCCTGTGGGGACGTCGTGATGACCCA
GTCTCCAGAGTCCTTGGCAGTGTCTCTAGGACAGAGGGTCGAGATGAAGTGCAAGGCCAGTCAGAGTATTGGCAAGT
ACTTAGCTTGGTACCAGCAGAAACCAGGACAGGCTCCTAAGCGACTCATCTATCCTGCATCCATAAGAGCATCTGGG
GTCCCTGACCGATTCAGTGGCAGTGGATCTGGGACGGATTTCACTCTCACCATCAGCAGCCTCCGGGCTGAAGATGT
GGCCGATTATTACTGTATGCAATGTAATAGTAGTCCTCTTACGTTCGGCCAGGGGACCAAGCTGGAGATCAAACGGG
ATGATGCTAAGCCATCTGCCTTCATCTTCCCACTGTCTTCTGAGGAGTTAAGCAGTGGAAGTGCCTCTGTCGTATGC
TTGGTGTATGGCTTCTACCCCAGTGGAGCCACTATCAACTGGAAAGTGGATGGTCTTGCCAAAACAAGTAGC
>HM176223.1 Equus caballus clone 1K41 immunoglobulin kappa light chain V-J
region mRNA, partial cds
ATGCTGACGCAGACGCAGGTCCTTATATCTGTGTTGCTCTGGGTCTCAGGAGCCTGTGGGGACGTCGTGATGACCCA
GTCTCCAGAGTCCTTGGCAGTGTCTCTAGGACAGAGGGTCGAGATGAAGTGTAAGGCCAGTCAGAGTGTTAGCAACT
ACTTAGCTTGGTACCAGCAGAAACCAGGACAGGCTCCTAAGTGGGTCATCTATCGTGCATCCAACAGAGTATCAGGG
GTCCCTGACCGATTCAGTGGCAGTGGATCTGGGACGGATTTCACTCTCACCATCAGCAGCCTCCAGGCTGAAGATGT
GGCCGAATATTATTGTATGCAACATTATAATAATCCTCTTACGTTCGGCCAGGGGACCAAGCTGGAGATCAAACGGG
ATGATGCTAAGCCATCTGCCTTCATCTTCCCACCGTCTTCTGAGGAGTTAAGCAGTGGAAGTGCCTCTGTCGTATGC
TTGGTGTATGGCTTCTACCCCAGTGGAGCCACTATCAACTGGAAAGTGGATGGTCTTGCCAAAACAAGTAGC
>HM176222.1 Equus caballus clone 1K9 immunoglobulin kappa light chain V-J
region mRNA, partial cds
ATGCTGACGCAGACGCAGGTCCTTATATCTGTGTTGCTCTGGGTCTCAGGAGCCTGTGGGGACGTCGTGATGACCCA
GTCTCCAGAGTCCTTGGCAGTGTCTCTAGGACAGGGGGTCGAGATGAAGTGCAAGGCCAGTCAGAGTGTTAGCAGCT
TTTTAGCTTGGTACCAGCAGAAACCAGGACAGGCTCCTAAGCGGCTCATCTATGCTGCATCCAGCAGAGCATCTGGG
GTCCCTGACCGATTCAGTGGCAGTGGATCTGGGACGGATTTCACTCTCACCATCAGCAGCCTCCAGGCTGAAGATGT
GGCCATTTATTACTGTATGCAGCATTATAATAATCCTCTTACGTTCGGCCAGGGGACCAAGCTGGAGATCAAACGGG
ATGATGCTAAGCCATCTGCCTTCATCTTCCCACCGTCTTCTGAGGAGTTAAGCAGTGGAAGTGCCTCTGTCGTATGC
TTGGTGTATGGCTTCTACCCCAGTGGAGCCACTATCAACTGGAAAGTGGATGGTCTTGCCAAAACAAGTAGC
>HM176221.1 Equus caballus clone 1K21 immunoglobulin kappa light chain V-J
region mRNA, partial cds
ATGCTGACGCAGACGCAGGTCCTTATCGCTGTGTTGCTCTGGGTCTCAGGAGCCTGTGGGGACGTCGTGATGACCCA
GTCTCCAGAGTCCTTGGCAGTGTCTCTAGGACAGAGGGTCGAGATGAAGTGCAAGGCCAGTCAGAGTGTTGGCACTA
ACTTGGATTGGTACCAGCAGAAACCAGGACAGGCTCCTAAGGGGCTCATCTCTGGTGCATCCAGGAGAGCATCTGGG
GTCCCTGACCGATTCAGTGGCAGTGGATCTGGGACGGATTTCACTCTCACCATCAGCAGCCTCCAGGCTGAAGATGT
GGCCAACTATTACTGTATGCAGCGTAGCAGCTATCCTCTTACGTTCGGCCAGGGGACCAAGCTGGAGATCAAACGGG
ATGATGCTAAGCCATCTGCCTTCATCTTCCCACCGTCTTCTGAGGAGTTAAGCAGTGGAAGTGCCTCTGTCGTATGC
TTGGTGTATGGCTTCTACCCCAGTGGAGCCACTATCAACTGGAAAGTGGATGGTCTTGCCAAAACAAGTAGC
>HM176220.1 Equus caballus clone 1K48 immunoglobulin kappa light chain V-J
region mRNA, partial cds
ATGCTGACGCAGACGCAGGTCCTTATATCTGTGTTGCTCTGGGTCTCAGGTGCCTGTGGGGACGTCGTGATGACCCA
GTCTCCAGAGTCCTTGGCAGTGTCTCTAGGACAGAGGGTCGAGATGAAGTGCAAGGCCAGTGAGTATGTAAGCAGCC
ACTTAGCTTGGTACCAGCAGAAACCAGGACAGGCTCCTAAGCGAATGATCTATCTTGGATCCAGCAGAGGATCTGGG
GTCCCTGACCGATTCAGTGGCAGTGGATCTGGGACGGATTTCACTCTCACCATCAGCAGCCTCCAGGCTGAAGATGT
GGCCATTTATTACTGTATGCAGAGTTATATAGAACCTCTTACGTTCGGCCAGGGGACCAAGCTGGAGATCAAACGGG
ATGATGCTAAGCCATCTGCCTTCATCTTCCCACCGTCTTCTGAGGAGTTAAGCAGTGGAAGTGCCTCTGTCGTATGC
TTGGTGTATGGCTTCTACCCCAGTGGAGCCACTATCAACTGGAAAGTGGATGGTCTTGCCAAAACAAGTAGC
>HM176219.1 Equus caballus clone 1K126 immunoglobulin kappa light chain V-J
region mRNA, partial cds
ATGCTGACGCAGACGCAGGTCCTTATATCTGTGTTGCTCTGGGTCTCAGGAGCCTGTGGGGACGTCGTGATGACCCA
GTCTCCAGAGTCCTTGGCAGTGTCTCTGGGACAGAGGGTCGAGATGAAGTGCAAGGCCAGTCAGAGTGTTAGCGGCT
GGTTAGCTTGGTACCAGCAGAAACCAGGACAGGCTCCTAAGGCGCTCATGTATGCTACATCCAGCAGAGCATCTGGG
GTCCCTGACCGATTCAGTGGCAGTGGATCTGGGACGGATTACACTCTCACCATCAGCAGCCTCCAGGCTGAAGATGT
GGCCATTTATTACTGTATGCAGTATAGTAATAATCCTCTTACGTTCGGCCAGGGGACCAACCTGGAGGTCAAACGGG
ATGATGCTAAGCCATCTGCCTTCATCTTCCCACCGTCTTCTGAGGAGTTAAGCAGTGGAAGTGCCTCTGTCGTATGC
TTGGTGTATGGCTTCTACCCCAGTGGAGCCACTATCAACTGGAAAGTGGATGGTCTTGCCAAAACAAGTAGC
>HM176218.1 Equus caballus clone 1K70 immunoglobulin kappa light chain V-J
region mRNA, partial cds

ATGCTGACGCAAACGCAGGTCCTTATATCTGTGTTGCTCTGGGTCTCAGGAACCTGTGGGGACGTCGTGATGACCCA
GTCTCCAGAGTCCTTGGCAGTGTCTCTGGGACAGAGGGTCGAGATGAAGTGCAAGGCCAGTCAGAGTGTTAGCAATC
GCTTAGCTTGGTACCAACAGAAACCAGGACAGGCTCCTAAGCGGCTCATCTATGGTACATCCAGCAGAGAATCTGGG
GTCCCTGACCGATTCAGTGGCAGTGGATCTGGGACGGATTTCACTCTCACCATCAGCAGCCTCCAGGCTGAAGATGT
GGCCATTTATTACTGTATGCAGCATTATAATAATCCTCTTACGTTCGGCCAGGGGACCAAGCTGGAGATCAAACGGG
ATGATGCTAAGCCATCTGCCTTCATCTTCCCACCGTCTTCTGAGGAGTTAAGCAGTGGAAGTGCCTCTGTCGTATGC
TTGGTGTATGGCTTCTACCCCAGTGGAGCCACTATCAACTGGAAAGTGGATGGTCTTGCCAAAACAAGTAGC
>HM176214.1 Equus caballus clone 2K153 immunoglobulin kappa light chain V-J region mRNA, partial cds
ATGGGGTCAAGGGCTTCACTGCTGTGGATCCTGCTGCTCTGGGTTCCAGACGCTATTGGGGACATTGTGTTGACCCA
GTCTCCAGCCTCCTTGACGGTGTCTCCAGGTCAGAGCGCCACGATTTCCTGCAGGGCCAGTGAGAATATTGGTTCTT
TTGGCGTGATTTATCGCCAAGTAGTAAATAACCTTCACTGGTATCAACAGAAACCAGGACAACGTCCTAAACTCCTG
ATCTCTTCAGCATCCGAGCTAGGGTCTGGGGTCCCAGCCAGGTTCAGTGGCAGTGGGTCTGGGACTGATTTCACCCT
CACAATTAATCCTGTGGAGGAGGGCGATGCTGCAACGTACTACTGCCAGCAGGGTAAGGAGTCTCCATACACGTTCG
GCCAAGGGACCAAGCTGGAGATGGAGATCAAAAGGGATGATGCTAAGCCATCTGCCTTCATCTTCCCACCGTCTTCT
GAGGAGTTAAGCAGTGGAAGTGCCTCTGTCGTATGCTTGGTGTATGGCTTCTACCCCAGTGGAGCCACTATCAACTG
GAAAGTGGATGGTCTTGCCAAAACAAGTAGC
>HM176204.1 Equus caballus clone 2K68 immunoglobulin kappa light chain V-J region mRNA, partial cds
ATGATGTCGCTGACAAAGGTCCTTATATCTGTGTTGCTCTGGGTCTCAGGTGCCTGGGGGGACTTCGTGTTGACCCA
GTCTCCAGAGTCCTTGGCAGTGTCTCTGGGACAGAGGGTCGAGATGAAGTGCAGGGCCAGTCAAGATGTTAGCAGCT
ACTTAGCTTGGTACCAGCAAAAACCAGGCCAGGCTCCTAAGAGACTCATCTACGGTGCATCCGACAGAGCGTCTGGG
GTCCCTGACCGATTCAGTGGCAGAGGGTTTGGGACAGATTTCACTCTCACCATCAGCAGCCTCCAGGCTGAAGATGT
GGCCGTTTATTACTGTCAGCAATATAATACGGTTCCTCGGGAATACACGTTCGGCCAAGGGACCAAACTGGAGATCA
AAAGGGATGATGCTAAGCCATCTGCCTTCATCTTCCCACCGTCTTCTGAGGAGTTAAGCAGTGGAAGTGCCTCTGTC
GTATGCTTGGTGTATGGCTTCTACCCCAGTGGAGCCACTATCAACTGGAAAGTGGATGGTCTTGCCAAAACAAGTAG
C
>HM176203.1 Equus caballus clone 2K439 immunoglobulin kappa light chain V-J region mRNA, partial cds
ATGATGTCGCTGACAAAGGTCCTTATATCTGTGTTGCTCTGGGTCTCAGGTGCCTGTCGGGACATCGTGTTGACCCA
GTCTCCAGAGTCCTTGGCAGTGTCTCTAGGACAGAGGGTCGAGATGAAGTGCAAGGCCAGTCAGAGTTTTGAAGATA
AGTTAGCTTGGTACCAGCAGAAACCAGGACAGTCTCCTAAGCATATCATCTACAGTGCATCCAGCAGAGCGTCTGGG
GTCCCTGACCGATTCAGTGGCAGTGGATCTGGGACAGATTTCACTCTCACCATCAGTAGCCTCCAGGCTGAAGATGT
GGCCGTTTATTACTGTCAGCAGTATAATTTCGATCCTCTTACGTTCGGCCAGGGGACCAAGCTGGAGATCAAACGGG
ATGATGCTAAGCCATCTGCCTTCATCTTCCCACCGTCTTCTGAGGAGTTAAGCAGTGGAAGTGCCTCTGTCGTATGC
TTGGTGTATGGCTTCTACCCCAGTGGAGCCACTATCAACTGGAAAGTGGATGGTCTTGCCAAAACAAGTAGC
>HM176201.1 Equus caballus clone 2K298 immunoglobulin kappa light chain V-J region mRNA, partial cds
ATGATGTCGCTGACAAAGGTCCTTATATCTGTGTTGCTCTGGGTCTCAGGTGCCTGTGGGGACATCGTCGTGACCCA
GTCTCCAGAGTCCTTGGCAGTGTCTCTAGGACAGAGGGTCGAAATGAAGTGCAGGGCCAGTCACAGTGTTAGCATTT
ACTTTGCTTGGTACCAGCAGAAACCAGGACAGGTTCCTAAGCAGCTCATCTACGAGGCAACCAGAAGAGCGTCTGGG
GTCCCTGACCGATTCAGTGGCGGTGGATCTGGGACAGATTTCACTCTCACCATCGACAGCCTCCAGGCTGAAGATGT
GGCCGTTTACTACTGTCAGCAGTATAATTACTTTCCTCTTACGTTCGGCCAGGGGACCAAGGTGGAGATCAGACGGG
ATGATGCTAAGCCATCTGCCTTCATCTTCCCACCGTCTTCTGAGGAGTTAAGCAGTGGAAGTGCCTCTGTCGTATGC
TTGGTGTATGGCTTCTACCCCAGTGGAGCCACTATCAACTGGAAAGTGGATGGTCTTGCCAAAACAAGTAGC
>HM176200.1 Equus caballus clone 2K170 immunoglobulin kappa light chain V-J region mRNA, partial cds
ATGATGTCGCTGACAAAGGTCCTTATATCTGTGTTGCTCTGGGTCTCAGGTGCCTGCGGGGACATCATGTTGACCCA
GTCTCCAGAGTCCTTGGCAGTGGCTCTAGGACAGAGGGTCGAGATGAAGTGTAAGGCCAGTCAGAGTGCTGAGAATT
ACTTGGCTTGGTACCAGCAGAAGCCAGGACAGGCTCCTAAACAGGTCATCTACGATACATCCGGCAGAGCGGCTGGG
TTCCCTGACCGATTCAGTGGCAGTGGATCTGGGACTGATTTCACTCTCACCATCAGTAGCCTCCAGGCTGAAGATGT
GGCCGTTTATTATTGTCAGCAGTATAAAAGCCTACCTCTTACGTTCGGCCAGGGGACCAAGTTGGAGATCAAACGGG
ATGATGCTAAGCCATCTGCCTTCATCTTCCCACCGTCTTCTGAGGAGTTAAGCAGTGGAAGTGCCTCTGTCGTATGC
TTGGTGTATGGCTTCTACCCCAGTGGAGCCACTATCAACTGGAAAGTGGATGGTCTTGCCAAAACAAGTAGC
>HM176199.1 Equus caballus clone 2K151 immunoglobulin kappa light chain V-J region mRNA, partial cds

ATGATGTCGCTGACAAAGGTCCTTATATCTGTGTTGCTCTGGGTCTCAGGTGCCTGTGGGGACATCGTGTTGACCCA
GTCTCCAGAGTCCTTGGCAGTGTCTCTAGGGCAGAGGGTCGAGATGAAGTGTAAGGCCAGTCAGAGTGCTGAAATAA
AATTAGCTTGGTACCAGCAGAAACCAGGACAGGCTCCTAAGCAGCTCATCTACGGTTCGTCCAACAGAGCGTCTGGG
GTCCCTGACCGATTCAGCGGCAGTGGATCTGGGACAGATTTCACTCTCACCATCAGCAGCCTCCAGGCTGAAGATGT
GGCCGATTATTACTGTCAGCAGTATAAAAGTGATCCTCTTACGTTCGGCCAGGGGACCAAGCTGGAGATCAAACGGG
ATGATGCTAAGCCATCTGCCTTCATCTTCTCACCGTCTTCTGAGGAGTTAAGCAGTGGAAGTGCCTCTGTCGTATGC
TTGGTGTATGGCTTCTACCCCAGTGGAGCCACTATCAACTGGAAAGTGGATGGTCTTGCCAAAACAAGTAGC
>HM176198.1 Equus caballus clone 2K130 immunoglobulin kappa light chain V-J
region mRNA, partial cds
ATGATGTCGCTGACAAAGGTCCTTATATCTGTGTTGCTCTGGGTCTCAGGTGCCTGTCGGGACATCGTGTTGACCCA
GTCTCCAGAGTCCTTGGCAGTGTCTCTAGGACAGAGGGTCGAGATGAAGTGCAAGGCCAGTCAGAGTTTTGAAGATA
AGTTAGCTTGGTACCAGCAGAAACCAGGACAGTCTCCTAAGCATATCATCTACAGTGCATCCAGCAGAGCGTCTGGG
GTCCCTGACCGATTCAGTGGCAGTGGATCTGGGACAGATTTCACTCTCACCATCAGTAGCCTCCAGGCTGAAGATGT
GGCCGTGTATTACTGTCAGCAGTATCATTTCGATCCTCTTACGTTCGGCCAGGGGACCAAGCTGGAGATCAAACGGG
ATGATGCTAAGCCATCTGCCTTCATCTTCCCACCGTCTTCTGAGGAGTTAAGCAGTGGAAGTGCCTCTGTCGTATGC
TTGGTGTATGGCTTCTACCCCAGTGGAGCCACTATCAACTGGAAAGTGGATGGTCTTGCCAAAACAAGTAGC
>HM176197.1 Equus caballus clone 2K284 immunoglobulin kappa light chain V-J
region mRNA, partial cds
ATGATGTCGCTGACAAAGGTCCTTATTTCTGTGTTGCTCTGGGTCTCAGGCACCTGTGGGGACATCGTGTTGACCCA
GTCTCCAGAGTCCTTGGCAGTGTCTCTAGGACAGAGGGTCGAGATGAAGTGCGTGGCCAGTCGGAGTGCCGTGACCT
GGTTAGCTTGGTATCAGCAGAAACCAGGACAGGCTCCTAAGCGACTCATCTATGGTGCGTCCAGGAGAGGGTCGGGG
GTCCCTGACCGATTCAGTGGAAGTGGATCTGGGACAGATTTCACTCTCACCATCAGCGAACCCCAGGCTGAAGATGT
GGCCGTTTATTACTGTCATCAGTATAATGAGGAACCGTGGACGTTCGGTGCCGGGACCAAGCTGGAAATCAAACGGG
ATGATGCTAAGCCATCTGCCTTCATCTTCCCACCGTCTTCTGAGGAGTTAAGCAGTGGAAGTGCCTCTGTCGTATGC
TTGGTGTATGGCTTCTACCCCAGTGGAGCCACTATCAACTGGAAAGTGGATGGTCTTGCCAAAACAAGTAGC
>HM176195.1 Equus caballus clone 2K426 immunoglobulin kappa light chain V-J
region mRNA, partial cds
ATGTCGCTGACAAAGGTCCTTATATCTGTGTTGCTCTGGGTCTCAGGTGCCTGTGGGGACATCGTGTTGACCCAGTC
TCCAGAGTCCTTGGCAGTGTCTCTAGGACAGAGGGTCGAGATGAACTGCAAGGCCAGTCAGAGTGCTAACATCTACT
TAACTTGGTACCAGCAGAAACCAGGACAGGCTCCTAAACAGATCATCTATAGTGCATCCAGCAGAGCGACTGGGGTC
CCTGACCGCTTCTGTGGCAGTGGATCTGGGACAGATTTCACTCTCACCATCAGCAGCCTCCAGGCTGAAGATGTGGC
CGTTTATTACTGTCAGCAGTATAATAGTGTTCCGCTCTCGTTCGGCCAGGGGACCAAGCTGGAGATCAAACGGGATG
ATGCTAAGCCATCTGCCTTCATCTTCCCACCGTCTTCTGAGGAGTTAAGCAGTGGAAGTGCCTCTGTCGTATGCTTG
GTGTATGGCTTCTACCCCAGTGGAGCCACTATCAACTGGAAAGTGGATGGTCTTGCCAAAACAAGTAGC
>HM176192.1 Equus caballus clone 2K257 immunoglobulin kappa light chain V-J
region mRNA, partial cds
ATGATGTCGCTGACAAAGGTCCTTATATCTGTGTTGCTCTGGGTCTCAGGTGCCTGTGGGGACATCGTGTTGACCCA
GTCTCCAGAGTCCTTGGCGGTGTCTCTAGGACAGAGGGTCGAGATGGAGTGCAAGGCCAGTCAGAGTGCTGGCATCT
ACTTAGCTTGGTACCAGCAGAAACCAGGACAGGCTCCTAAGCAGCTCATCGAAGTTGCATCCAGCAGAGCGTCTGGG
GTCCCCGACCGATTCAGTGGCAGTGGATCTGGGACAGATTTCACTCTCACCATCAGCAGCCTCCAGGCTGAAGATGT
GGCCGTTTATTATTGTCAGCAGTATGTTCAATATCCGCTTACGTTCGGCCAGGGGACCAAGCTGGAGATCAAACGGG
ATGATGCTAAGCCATCTGCCTTCATCTTCCCACCGTCTTCTGAGGAGTTAAGCAGTGGAAGTGCCTCTGTCGTATGC
TTGGTGTATGGCTTCTACCCCAGTGGAGCCACTATCAACTGGAAAGTGGATGGTCTTGCCAAAACAAGTAGC
>HM176191.1 Equus caballus clone 2K52 immunoglobulin kappa light chain V-J
region mRNA, partial cds
ATGATGTCGCTGACAAAGGTCCTTATATCTGTGTTGCTCTGGGTCTCAGGTGCCTGTGGGGACATCGTGTTGACCCA
GTCTCCAGAGTCCTTGGCAGTGTCTCTAGGACAGAGGGTCGAGATGAAGTGCAAGGCCAGTCAGAGTGTTAGCAGCT
ACTTAGCTTGGTACCAGCAGAAACCAGGACAGACTCCTAAGCAGCTCTTCTACTACGCATCCAGCAGAGCGTCTGGG
GTCCCTGACCGATTCAGTGGCAGTGGATCTGGGACAGATTTCACTCTCACCATCAGCAGTTTCCAGGCTGAAGATGT
GGCCGTTTATTACTGTCAGCAGTATATCACCTATCCGCTTACGTTCGGCCAGGGGACCAAGCTGGAGATCAAACGGG
ATAATGCTAAGCCATCTGCCTTCATCTTCCCACCGTCTTCTGAGGAGTTAAGCAGTGGAAGTGCCTCTGTCGTATGC
TTGGTGTATGGCTTCTACCCCAGTGGAGCCACTATCAACTGGAAAGTGGATGGTCTTGCCAAAACAAGTAGC
>HM176190.1 Equus caballus clone 2K17 immunoglobulin kappa light chain V-J
region mRNA, partial cds

ATGATGTCGCTGACAAAGGTCCTTATATCTGTGTTGCTCTGGGTCTCAGGTGCCTGTGGGGACACAGTGTTGACCCA
GTCTCCAGAGTCCTTGGCAGTGTCTCTAGGACAGAGGGTCGAGATGAAGTGCAAGGCCAGTCAGAGTCCGCGCAGCA
AAATAGCTTGGTACCAGCAGAAACCAGGACAGGCTCCTAAGCAGGTCATCTACAGTGCATCCAGCAGAGCGTCTGGG
GTCCCTGACCGATTCAGTGGCAGTGGATCTGGGACAGATTTCACTCTCACCATCAGCAGCCTCCAGGCTGAGGATGT
GGCCGTTTATTACTGTCAACAGTACTATGATGATCCGCTTACGTTCGGCCAGGGGACCAAGCTGGAGATCAAACGGG
ATGATGCTAAGCCATCTGCCTTCATCTTCCCACCGTCTTCTGAGGAGTTAAGCAGTGGAAGTGCCTCTGTCGTATGC
TTGGTGTATGGCTTCTACCCCAGTGGAGCCACTATCAACTGGAAAGTGGATGGTCTTGCCAAAACAAGTAGC
>HM176189.1 Equus caballus clone 2K316 immunoglobulin kappa light chain V-J region mRNA, partial cds
ATGCTGACGCAGACGCAGGTCTTTATATCTGTGTTGCTCTGGGTCTCGGGAGCCTGTGGGGACGTCGTGATGACCCA
GTCTCCAGACTCCTTGGCAGCGTCTCTAGGACAGAGAGTCGAGATGAAGTGCAAGGCCAGTCAGAGTATTAGCAGCA
GCTTAGCTTGGTACCAGCAGAAATCAGGAGAGGCTCCTAAGCGGCTCATCTATGCTGCATCCAGGAGAGCATCTGGG
GTCCCTGACCGATTCAGTGGCAGTGGATCTGGGACGGATTTCACTCTCACCATCAGCAGCCTCCAGGCTGAAGATGT
GGCCATTTATTACTGTCTGCAGAATATAGCCTATCCTCTTACGTTCGGCCAGGGGACCAAGCTGGAGATCAAACGGG
ATGATGCTAAGCCATCTGCCTTCATCTTCCCACCGTCTTCTGAGGAGTTAAGCAGTGGAAGTGCCTCTGTCGTATGC
TTGGTGTATGGCTTCTACCCCAGTGGAGCCACTATCAACTGGAAAGTGGATGGTCTTGCCAAAACAAGTAGC
>HM176188.1 Equus caballus clone 2K231 immunoglobulin kappa light chain V-J region mRNA, partial cds
ATGCTGACGCAGACGCAGGTCTTTATATCTGTGTTGCTCTGGGTCTCAGGAGCCTGTGGGGACGTCGTGATGACCCA
GTCTCCAGACTCCTTGGCAGCGTCTCTAGGACAGAGAGTCGAGATGAAGTGCAAGGCCAGTCAGAGTGTTAGCAGCT
ACTTAACTTGGTACCAGCAGAAACCAGGACAGGCTCCTAAGCGACTCATCTATGCTTCATCCAGCAGAGAATCTGGG
GTCCCTGACCGATTCAGTGGCAGTGGATCTGGGACGGATTTCACTCTCACCATCAGCAGCCTCCAGGCTGAAGATGT
GGCGATTTATTACTGCCTACAGCATGGTGTTGATCCTCTTACGTTCGGCCAGGGGACCAAGCTGGAGATCAAACGGG
ATGATGCTAAGCCATCTGCCTTCATCTTCCCACCGTCTTCTGAGGAGTTAAGCAGTGGAAGTGCCTCTGTCGTATGC
TTGGTGTATGGCTTCTACCCCAGTGGAGCCACTATCAACTGGAAAGTGGATGGTCTTGCCAAAACAAGTAGC
>HM176187.1 Equus caballus clone 2K173 immunoglobulin kappa light chain V-J region mRNA, partial cds
ATGCTGACGCAGACGCAGGTCTTTATATCTGTGCTGCTCTGGGTCTCAGGAGCCTGTGGGGACGTCGTGATGACCCA
GTCTCCAGACTCCTTGGCAGCGTCTCTAGGACAGAGAGTCGAGATGAAGTGCAAGGCCAGTCAGAGTGTTAGCAGCC
GCTTATCTTGGTACCAGCAGAAACCAGGACAGGCTCCTAAGCGGCTCATCGTTGCTGCATCCGGCAGAGAATCTGGG
GTCCCTGACCGATTCAGTGGCAGTGGATCTGGGACGGATTTCACGCTCACCATCAGCAGTTTCCAGGCTGAAGATGT
GGCCATTTATTTTTGTCTGCAGCAGTATAAATATCCTCTTACGTTCGGCCAGGGGACCAAGCTGGAGATCAAACGGG
ATGATGCTAAGCCATCTGCCTTCATCTTCCCACCGTCTTCTGAGGAGTTAAGCAGTGGAAGTGCCTCTGTCGTATGC
TTGGTGTATGGCTTCTACCCCAGTGGAGCCACTATCAACTGGAAAGTGGATGGTCTTGCCAAAACAAGTAGC
>HM176186.1 Equus caballus clone 2K277 immunoglobulin kappa light chain V-J region mRNA, partial cds
ATGCTGACGCAGACGCAGGTCTTTATATCTGTGTTGCTCTGGGTCTCAGGAGCCTGTGGGGACGTCGTGATGACCCA
GTCTCCAGACTCCTTGGCAGCGTCTCTAGGACAGAGAGTCGAGATGAAGTGCAAGGCCAGTCAGAGTGTTAGCAACT
ACTTAACTTGGTACCAGCAGAAACCAGGACAGGCTCCTAAGCGGCTCATCTATGCTGCATCCAGCAGAGCAGCTGGG
GTCCCTGACCGATTCAGCGGCAGTGGATCTGGGACGGATTTCACTCTCACCATCAGCAGCCTCCAGGCTGAAGATGT
GGCCATTTATTATTGTCTGCAGAATTATTACAATCCGCTTATGTTCGGCCAGGGGACCAAGCTGGAGATCAAGCGGG
ATGATGCTAAGCCATCTGCCTTCATCTTCCCACCGTCTTCTGAGGAGTTAAGCAGTGGAAGTGCCTCTGTCGTATGC
TTGGTGTATGGCTTCTACCCCAGTGGAGCCACTATCATCTGGAAAGTGGATGGTCTTGCCAAAACAAGTAGC
>HM176185.1 Equus caballus clone 2K179 immunoglobulin kappa light chain V-J region mRNA, partial cds
ATGCTGACGCAGACGCAGGTCTTTATATCTGTGTTGCTCTGGGTCTCAGGAGCCTGTGGGGACGTCGTGATGACCCA
GTCTCCAGACTCCTTGGCAGCGTCTCTAGGACAGAGAGTCGAGATGAAGTGTAAGGCCAGTCAGAGTGTTAGGAACC
ACTTAACTTGGTACCAGCAGAAACCAGGACAGGCTCCTAAGCGGCTCATCTACGCTGCATCCAACAGAGCATCTGGG
GTCCCTGACCGATTCAGTGGCAGCGGATCTGGGACGGATTTCACTCTCACCATCAGCAGCCTCCAGGCTGAAGATGT
GGCCATTTATTACTGTCTGCAGTATTCGTATAATCCGTTTACGTTCGGCCAGGGGACCAAGCTGGAGATCAAACGGG
ATGATGCTAAGCCATCTGCCTTCATCTTCCCACCGTCTTCTGAGGAGTTAAGCAGTGGAAGTGCCTCTGTCGTATGC
TTGGTGTATGGCTTCTACCCCAGTGGAGCCACTATCAACTGGAAAGTGGATGGTCTTGCCAAAACAAGTAGC
>HM176183.1 Equus caballus clone 2K443 immunoglobulin kappa light chain V-J region mRNA, partial cds
ATGCTGACGCAGACGCAGGTCCTTATATCTGTGTTGCTCTGGGTCTCAGGAGCCTGTGGGGACGTCGTGATGACCCA
GTCTCCAGACTCCTTGGCAGCGTCTCTAGGACAGAGAGTCGAGATGAAGTGCAAGGCCAGTCAGAGTGTTAACAACT

ACTTAGCTTGGTACCAGCACAAACCAGGACAGGCTCCTAAGCGGGTCATCTATGCTGCGTCCACCAGAGAATCTGGG
GTCCCTGACCGATTCAGTGGCAGTGGATCTGGGACGGATTTCACTCTCACCATCAGCAGCCTCCAGGCTGGAGATGT
GGCCGTTTATTACTGTATGCAGGTTTATAGTAATCCTCCTACGTTCGGCCAGGGGACCAAGCTGGAGATCAAACGGG
ATGATGCTAAGCCATCTGCCTTCATCTTCCCACCGTCTTCTGAGGAGTTAAGCAGTGGAAGTGCCTCTGTCGTATGC
TTGGTGTATGGCTTCTACCCCAGTGGAGCCACTATCAACTGGAAAGTGGATGGTCTTGCCAAAACAAGTAGC
>HM176182.1 Equus caballus clone 2K377 immunoglobulin kappa light chain V-J region mRNA, partial cds
ATGCTGACGCAGACGCAGGTCCTTATATCTGTGTTGCTCTGGGTCTCAGGGGCCTGCGGGGACGTCGTGATGACCCA
GTCTCCAGACTCCTTGGCGGCGTCTCTAGGACAGAGAGTCGAGATGGAGTGCAAGGCCAGTCAGTATGTTGAAAACT
ACGTAGATTGGTACCAGCAGAAACCAGGACAGGCTCCTAAGCGAATCATCTATGCTGCAAATGGCAGAGCGACTGGG
GTCCCTGACCGATTCAGTGGCAGTGGATCTGGGTGGGATTTCACTCTCACCATCAGCAGCCTCCAGGCTGAAGATGT
GGCCATTTATTACTGTATGCATTATTATTATAATCCTCCTACGTTCGGCCAGGGGACCAAGCTGGAGATCACACGGG
ATGATGCTAAGCCATCTGCCTTCATCTTCCCACCGTCTTCTGAGGAGTTAAGCAGTGGAAGTGCCTCTGTCGTATGC
TTGGTGTATGGCTTCTACCCCAGTGGAGCCACTATCAACTGGAAAGTGGATGGTCTTGCCAAAACAAGTAGC
>HM176181.1 Equus caballus clone 2K152 immunoglobulin kappa light chain V-J region mRNA, partial cds
ATGCTGACGCAGACGCAGGTCCTTATATCTGTGTTGCTCTGGGTCTCAGGAGCCTGTGGGGACGTCGTGATGACCCA
GTCTCCAGACTCCTTGGCAGCGTCTCTAGGACAGAGAGTCGAGATGAAGTGCGAGGCCAGTCAGAGTGTTCTCAGTT
ATTTGTCTTGGTACCAACATAAACCAGGACAGGCTCCTAAGCGGGTCATCTATACTGCATCCAGTAGAACACCTGGG
GTCCCTGACCGATTCAGTGGCAGTGGATCTGGGACGGATTTCACTCTTACCATCAGCAGCCTCCAGGCTGAAGATGT
GGCCATTTATTACTGTATGCAGTATCGTATTGATCCTCCTACATTCGGCCAGGGGACCAAGCTGGAGATCAAACGGG
ATGATGCTAAGCCATCTGCCTTCATCTTCCCACCGTCTTCTGAGGAGTTAAGCAGTGGAAGTGCCTCTGTCGTATGC
TTGGTGTATGGCTTCTACCCCAGTGGAGCCACTATCAACTGGAAAGTGGATGGTCTTGCCAAAACAAGTAGC
>HM176178.1 Equus caballus clone 2K183 immunoglobulin kappa light chain V-J region mRNA, partial cds
ATGCTGACGCAGACGCAGGTCCTTATATCTGTGTTGCTCTGGGTCTCAGGAGCCTGTGGGGACGTCGTGATGACCCA
GTCTCCAGACTCCTTGGCAGCGTCTCTAGGACAGAGAGTCGAGATGAAGTGCAAGGCCAGTCAGAGTGTTGGCACCT
GGTTACTTTGGTACCAGCACAAACCAGGACAGGCTCCTAAGCGGGTCATCTATGGTGCATCCAGTAGAGCATCTGGG
GTCCCTGACCGATTCAGTGGCAGTGGATCTGGGACGGATTTCACTCTCACCATCAGCAGCCTCCAGGCTGAAGATGT
GGCCATTTATTACTGTATGCAGCATTCTAGTTATCCTCTTACGTTCGGCCAGGGGACCAAGCTGGAGATCAAACGGG
ATGATGCTAAGCCATCTGCCTTCATCTTCCCACCGTCTTCTGAGGAGTTAAGCAGTGGAAGTGCCTCTGTCGTATGC
TTGGTGTATGGCTTCTACCCCAGTGGAGCCACTATCAACTGGAAAGTGGATGGTCTTGCCAAAACAAGTAGC
>HM176177.1 Equus caballus clone 2K140 immunoglobulin kappa light chain V-J region mRNA, partial cds
ATGCTGACGCAGACGCAGGTCCTTATATCTGTGTTGCTCTGGGTCTCAGGAGCCTGTGGGGACGTCGTGATGACCCA
GTCTCCAGACTCCTTGGCAGCGTCTCTAGGACAGAGAGTCGAGATGAAGTGCAAGGCCAGCCAGAGTGTTAGCGTTT
ACTTAGCTTGGTACCAGCACAAACCAGGACAGGCTCCTAAGCGGGTCATCTATTCTGCATCCAGCAGAGAATCTGGG
GTCCCTGACCGATTCAGTGGCAGTGGAGCTGGGACGGATTTCACTCTCACCATCAGCAGCCTCCAGGCTGAAGATGT
GGCCATCTATTACTGTATGCAGCATTATAATAATCCTCTTACGTTCGGCCAGGGGACCAAGGTGGAGATCAAACGGG
ATGATGCTAAGCCATCTGCCTTCATCTTCCCACCGTCTTCTGAGGAGTTAAGCAGTGGAAGTGCCTCTGTCGTATGC
TTGGTGTATGGCTTCTACCCCAGTGGAGCCACTATCAACTGGAAAGTGGATGGTCTTGCCAAAACAAGTAGC
>HM176176.1 Equus caballus clone 2K139 immunoglobulin kappa light chain V-J region mRNA, partial cds
ATGCTGACGCAGACGCAGGTCCTTATATCTGTGTTGCTCTGGGTCTCAGGAGCCTGTGGGGACGTCGTGATGACCCA
GTCTCCAGACTCCTTGGCAGCGTCTCTAGGACAGAGAGTCGAGATGAAGTGCAAGGCCAGTCAGAGTGTTAGCACCT
ACTTAGCTTGGTACCAGCACAAACCAGGACAGGCTCCTAAGCGGGTCATCTATGCTGCATCCAGCAGAGCATCTGGG
GTCCTTGACCGATTCAGTGGCAGTGGATCTGGGACGGATTTCACTCTCACCATCAGCAGCCTCCAGGCTGAAGATGT
GGCGCTTTATTACTGTATGCAGCATTATAGTAATCCTCTTACGTTCGGCCAGGGGACCAAGCTGGAGATCAAACGGG
ATGATGCTAAGCCATCTGCCTTCATCTTCCCACCGTCTTCTGAGGAGTTAAGCAGTGGAAGTGCCTCTGTCGTATGC
TTGGTGTATGGCTTCTACCCCAGTGGAGCCACTATCAACTGGAAAGTGGATGGTCTTGCCAAAACAAGTAGC
>HM176172.1 Equus caballus clone 2K319 immunoglobulin kappa light chain V-J region mRNA, partial cds
ATGCTGACGCAGACGCAGGTCCTTATATCTGTGTTGCTCTGGGTCTCAGGAGCCTGTGGGGACGTCGTGATGACCCA
GTCTCCAGACTCCTTGGCAGCGTCTCTAGGACAGAGAGTCGAGATGAAGTGCAAGGCCAGTCAGAGTGTTAGCAACG
ACTTAGCTTGGTACCAACACAAACCAGGACAGGCTCCTAAGCGGGTCATCTATGCTGCATCCAGCAGAGCATATGGG

GTCCCTGAGCGATTCAGTGGCAGTGGATCTGGGACGGATTTCACTCTCACCATCAGCAGCCTCCAGGCTGAAGATGT
GGCCATTTATTACTGTATGCATTATTATAACTATCCAATTACGTTCGGCCAGGGGACCAAGCTGGAGATCAAACGGG
ATGATGCTAAGCCATCTGCCTTCATCTTCCCACCGTCTTCTGAGGAGTTAAGCAGGGGAAGTGCCTCTGTCGTATGC
TTGGTGTATGGCTTCTACCCCAGTGGAGCCACTATCAACTGGAAAGTGGATGGTCTTGCCAAAACAAGTAGC
>HM176171.1 Equus caballus clone 2K63 immunoglobulin kappa light chain V-J region mRNA, partial cds
ATGCTGACGCAGACGCAGGTCCTTATATCTGTGTTGCTCTGGGTCTCAGGAGCCTGTGGGGACGTCGTGATGACCCA
GTCTCCAGACTCCTTGGCAGCGTCTCTAGGACAGAGAGTCGAGATGAAGTGCAAGGCCAGTCAGAGTGTTAGCAGTT
ACTTAGCTTGGTACCAGCACAAACCAGGACAGGCTCCTAAGCGGGTCATCTATGCTGCATCCAGCAGAGCATCTGGG
GTCCCTGACCGATTCAGTGGCAGTGGATCTGGGACGGATTTCACTCTCACCATCAGCAGCCTCCAGGCTGAAGATGT
GGCCATTTATTACTGTATGCAGCATTCTACTAATCCGTTTACGTTCGGCCAGGGGACCAAGCTGGAGATCAAACGGG
ATGATGCTAAGCCATCTGCCTTCATCTTCCCACCGTCTTCTGAGGAGTTAAGCAGTGGAAGTGCCTCTGTCGTATGC
TTGGTGTATGGCTTCTACCCCAGTGGAGCCACTATCAACTGGAAAGTGGATGGTCTTGCCAAAACAAGTAGC
>HM176170.1 Equus caballus clone 2K392 immunoglobulin kappa light chain V-J region mRNA, partial cds
ATGCTGACGCAGACGCAGGTCCTTATATCTGTGTTGCTCTGGGTCTCAGGAGCCTGTGGGGACGTCGTGATGACCCA
GTCTCCAGACTCCTTGGCAGCGTCTCTAGGACAGAGAGTCGAGATGAGGTGCAAGGCCAGTCAGAGTGTGAGCAACA
CCTTAGTTTGGTTCCAGCACAAACCAGGACAGGCTCCTAAGCGGGTCATCTATGCTGCATCCAGCAGAGCATCTGGG
GTCCCTGACCGATTCAGTGGCAGTGGATCTGGGACGGAGTTCACTCTCACCATCAGCAGCCTCCAGGCTGAAGATGT
GGCCATTTATTACTGTATGCAGCATGGTTATAATCCGTTTACGTTCGGCCAGGGGACCAAGCTGGAGATCAAACGGG
ATGATGCTAAGCCATCTGCCTTCATCTTCCCACCGTCTTCTGAGGAGTTAAGCAGTGGAAGTGCCTCTGTCGTATGC
TTGGTGTATGGCTTCTACCCCAGTGGAGCCACTATCAACTGGAAAGTGGATGGTCTTGCCAAAACAAGTAGC
>HM176169.1 Equus caballus clone 2K455 immunoglobulin kappa light chain V-J region mRNA, partial cds
ATGCTGACGCAGACGCAGGTCCTTATATCTGTGTTGCTCTGGGCCTCAGGAGCCTGTGGGGACGTCGTGATGACCCA
GTCTCCAGACTCCTTGGCAGCGTCTCTAGGACAGAGAGTCGAGATGAAGTGCAAGGCCAGTCAGAGTGTTAACAACT
ACTTGGCTTGGTACCAGCACAAACCAGGACAGGCTCCTAAGCGGGTCATCTATGCTGCATCCAGCAGAGCATCTGGG
GTCCCTGACCGGTTCAGTGGCAGTGGATACGGGACGGATTTCACTCTCACCATCAGCAGCCTCCAGGCTGAAGATGT
GGCCGTCTATACCTGTATGCAAAATTATAAAAATCCGCTTACGTTCGGCCAGGGGACCAAGCTGGAGATCAAACAAC
GGGATGATGCTAAGCCATCTGCCTTCATCTTCCCACCGTCTTCTGAGGAGTTAAGCAGTGGAAGTGCCTCTGTCGTA
TGCTTGGTGTATGGCTTCTACCCCAGTGGAGCCACTATCAACTGGAAAGTGGATGGTCTTGCCAAAACAAGTAGC
>HM176168.1 Equus caballus clone 2K448 immunoglobulin kappa light chain V-J region mRNA, partial cds
ATGCTGACGCAGACGCAGGTCCTTATATCTGTGTTGCTCTGGGTCTCAGGAACCTGTGGGGACGTCGTGATGACCCA
GTCTCCAGACTCCTTGGCAGCGTCTCTAGGACAGAGAGTCGAGATGGAGTGCAAGGCCAGTCAGAGTATTAGCAACA
GATTAGCTTGGTACCAGCACAAACCAGGACAGGCTCCTAAGCGGGTCATCTACGCTGCATCCATCCGGTCATCTGGG
GTCCCTGACCGATTCAGTGGCAGTGGATCTGGGACGGATTTCACTCTCACCATCAGCAGCCTCCAGGCTGAAGATGT
GGCCATTTATTACTGTATGCAGCATCACTGGGATCCGCTTACGTTCGGCCAGGGGACCAAGCTGGAGCTCAAACGGG
ATGATGCTAAGCCATCTGCCTTCATCTTCCCACCGTCTTCTGAGGAGTTAAGCAGTGGAAGTGCCTCTGTCGTATGC
TTGGTGTATGGCTTCTACCCCAGTGGAGCCACTATCAACTGGAAAGTGGATGGTCTTGCCAAAACAAGTAGC
>HM176167.1 Equus caballus clone 2K101 immunoglobulin kappa light chain V-J region mRNA, partial cds
ATGCTGACGCAGACGCAGGTCCTTATATCTGTGTTGCTCTGGGTCTCAGGAGCCTGTGGGGAGATCGTGATGACCCA
GTCTCCAGACTCCTTGGCAGCGTCTTTAGGACAGAGAGTCGAGATGAAGTGCAAGTCCAGTCAGACTGTAATTGGCA
GTATAGCTTGGTACCAGCAGAAACTAGGACAGGCTCCTAAGCGGGTCATCTATGCTACATCCAGGAGACAATCTGGG
GTCCCTGACCGATACAGTGGCAGTGGATCTGGGACGGATTTCACTCTCACCATCAGCAGACTCCAGGCTGAAGATGT
GGCAGTTTATTACTGTATGCAGTATCATGACTATCCGCTTACGTTCGGCCAGGGGACCAAGTTGGAGATCAAACGGG
ATGATGCTAAGCCATCTGCCTTCATCTTCCCACCGTCTTCTGAGGAGTTAAGCAGTGGAAGTGCCTCTGTCGTATGC
TTGGTGTATGGCTTCTACCCCAGTGGAGCCACTATCAACTGGAAAGTGGATGGTCTTGCCAAAACAAGTAGC
>HM176166.1 Equus caballus clone 2K122 immunoglobulin kappa light chain V-J region mRNA, partial cds
ATGCTGACGCAGACGCAGGTCCTTATATCTGTGTTGCTCTGGGTCTCAGGAGCCTGTGGGGACGTCGTGATGACCCA
GTCTCCAGACTCCTTGGCAGCGTCTCTAGGACAGAGAGTCGAGATGAAGTGCAAGGCCAGTCAGAGTGTAGGCAGCT
ACTTAGCTTGGTACCAGCACAAACCAGGACAGGCTCCTAAGCGGGTCATCTATGCTGCATCCACCAGAGCATCTGGG
GTCCCTGACCGATTCAGTGGCAGTGGATCTGGGACGGATTTCACTCTCGCCATCAGCAGCCTCCAGGCTGAAGATGT
GGCCATTTATTACTGTATGCAGCATTATGCCAGTCCGCTTACGTTCGGCCAGGGGACCAGGCTGAAGATCAAACGGG

ATGATGCTAAGCCATCTGCCTTCATCTTCCCACCGTCTTCTGAGGAGTTAAGCAGTGGAAGTGCCTCTGTCGTATGC
TTGGTGTATGGCTTCTACCCCAGTGGAGCCACTATCAACTGGAAAGTGGATGGTCTTGCCAAAACAAGTAGC
>HM176159.1 Equus caballus clone 1K14 immunoglobulin kappa light chain V-J region mRNA, partial cds
ATGGGGTCAAGGGCTTCACTGCCGTGGATCCTGCTGCTCTGGGTTCCAGACATTACTGGGGACATTGTGCTGACCCA
GTCTCCAGCCTCCTTGACGGTGTCTCCAGGTCAGAGCGCCACAATTTCCTGCAGGGCCAGTGAAAGTATTGGTTCTT
TTGACATGTATCCAGGCAAACTCACTCACAACCTTCATTGGTATCAAGTGAAACCAGGACAGCGTCCTAAACTCCTG
ATCTCTTCAGCCTCGGGTCTACGGTCTGGGGTCCCAGACAGGTTCAGTGCCAGTGGGTCTGGGACTGATTTCACCCT
CACAATTGATCCTGTGGAGGAGGCCGATGCTGCAGTTTACTACTGCCAGCAGGGTAAGGAGTCTCCGCTTACGTTCG
GCCAGGGGACCAAGCTGGAGATCAAACGGGATGATGCTAAGCCATCTGCCTTCATCTTCCCACCGTCTTCTGAGGAG
TTAAGCAGTGGAAGTGCCTCTGTCGTATGCTTGGTGTATGGCTTCTACCCCAGTGGAGCCACTATCAACTGGAAAGT
GGATGGTCTTGCCAAAACAAGTAGC
>HM176153.1 Equus caballus clone 1K74 immunoglobulin kappa light chain V-J region mRNA, partial cds
ATGATGTCGCTGACAAAGGTCCTTATATCTGTGTTGCTCTGGGTCTCAGGTGCCTGTGGGGACATCGTGTTGACCCA
GTCTCCAGAGTCCTTGGCAGTGTCTCTAGGACAGAGGGTCGAGATGAAGTGCAAGGCCAGTCAGACAATTAGTAGTT
ACTTAACTTGGTACCAGCAGAAACCAGGACAGGCTCCTAAACAGCTCGTCTATGCTGCATCTATTAGAGAATCTGGC
GTCCCCGAGCGATTCAGTGGCAGTGGATACGGGACAGACTTCACTCTCACCATCAGCAGCCTCCAGGCTGAAGATGT
GGCCATTTATTACTGTCAACAGCGGAATACTTTTCCTCTAACGTTCGGTGCCGGGACCAAGGTGGAAATCAAACGGG
ATGATGCTAAGCCATCTGCCTTCATCTTCCCACCGTCTTCTGAGGAGTTAAGCAGTGGAAGTGCCTCTGTCGTATGC
TTGGTGTATGGCTTCTACCCCAGTGGAGCCACTATCAACTGGAAAGTGGATGGTCTTGCCAAAACAAGTAGC
>HM176151.1 Equus caballus clone 1K277 immunoglobulin kappa light chain V-J region mRNA, partial cds
ATGATGTCGCTGACAAAGGTCCTTATATCTGTGTTGCTCTGGGTCTCAGGTGCCTGTGGGGACATCGTGTTGACCCA
GTCTCCAGAGTCCTTGGCAGTGTCTCTAGGACAGAGGGTCGAGATGAAGTGCAAGGCCAGTCAGAGTGTTAGCGGCT
ACTTAGCTTGGTACCAGCAGAAACCAGGACAGGCTCCTAAGTGGCTCATCTCGGCTGCATCCAGCAGAGCATCTGGC
GTCCCTGACCGATTCAGTGGCAGTGGATCTGGGACAGATTTCACTCTCACAATCAGCAGCCTCCAGGCTGAAGATGT
GGCCATTTATTACTGTCAGCAGTATAATAGTGCTCCTCCTACGTTCGGCCAGGGGACCAAACTGGAGATCATTCGGG
ATGATGCTAAGCCATCTGCCTTCATCTTCCCACCGTCTTCTGAGGAGTTAAGCAGTGGAAGTGCCTCTGTCGTATGC
TTGGTGTATGGCTTCTACCCCAGTGGAGCCACTATCAACTGGAAAGTGGATGGTCTTGCCAAAACAAGTAGC
>HM176148.1 Equus caballus clone 1K59 immunoglobulin kappa light chain V-J region mRNA, partial cds
ATGATGTCGCTGACAAAGGTCCTTATATCTGTGTTGCTCTGGGTCTCAGGTGCCTGTGGGGACATCGTGTTGACCCA
GTCTCCAGAGTCCTTGGCAGTGTCTCTAGGACAGAGGGTCGAGATGAAGTGTAAGGCCAGTCAGAGTGTTAACAGCT
ACTTATGTTGGTACCAGCAGAAACCAGGACAGGCTCCTAAGCAACTCATCTATGCTGCATCCAGCAGAGCATCTGGC
GTCCCTGACCGATTCTTTGGCAGTGGATCTGGGACAGATTTCACTCTCACCATCAGCAGCCTCCAGGCGGAAGATGT
GGCCATTTATTACTGTCAGCAGTATGAAGATAGTCCCACGTTCGGCCAAGGGACCAAACTGGAGATCAAAAGGGATG
ATGCTAAGCCATCTGCCTTCATCTTCCCACCGTCTTCTGAGGAGTTAAGCAGTGGAAGTGCCTCTGTCGTATGCTTG
GTGTATGGCTTCTACCCCAGTGGAGCCACTATCAACTGGAAAGTGGATGGTCTTGCCAAAACAAGTAGC
>HM176147.1 Equus caballus clone 1K208 immunoglobulin kappa light chain V-J region mRNA, partial cds
ATGATGTCGCTGACAAAGGTCCTTATATCTGTGTTGCTCTGGGTCTCAGGTGCCTATGGGAAGATCATGTTGACCCA
GTCTCCAGACACCTTGGCAGTGTCGCCGGGACAGAGGGTCGAGATGCAGTGCAGGGCCAGTCAGAGTGTGTCCAGAA
ACTTACATTGGTACCAGCAGAAACCAGGACAGTCTCCTAGGCAACTCATCTATCAGGCATCCCGCAGACCGTCTGGC
GTCCCTGACCGTTTCCGTGGCAGTGGATCTGGGACGGATTTCACTCTCACCATCACTAGCCTCCAGGCTGAAGATGT
GGCCATTTATTACTGTATGCAGCATTATAACTATCCATTTACGTTCGGCCAAGGGACCAAGCTGGAGATCAAAAGGG
ATGATGCTAAGCCATCTGCCTTCATCTTCCCACCGTCTTCTGAGGAGTTAAGCAGTGGAAGTGCCTCTGTCGTATGC
TTGGTGTATGGCTTCTACCCCAGTGGAGCCACTATCAACTGGAAAGTGGATGGTCTTGCCAAAACAAGTAGC
>HM176145.1 Equus caballus clone 1K4 immunoglobulin kappa light chain V-J region mRNA, partial cds
ATGATGTCGCTGACAAAGGTCCTTATATCTGTGTTGCTCTGGGTCTCAGGTGCCTGTGGGGACATCGTGTTGACCCA
GTCTCCAGAGTCCTTGGCAGTGTCTCTAGGACAGAGGGTCGAGATGAAGTGCAAGGCCAGTCAGAGTGTTAGCACGT
ACTTAAATTGGTACCAGGTGAAACCAGGACAGGCTCCTAAGCAGTTCATCACCGCTGCATCCAGCAGAAGATCTGGC
GTCCCTGACCGATTCAGTGGCAGTGGATCTGGGACAGATTTCACTCTCACCATCAGCAGCCTCCAGGCTGAAGATGT
GGCCACTTATTACTGTCAGCAGTATAATGTTGCCCTCTACACGTTCGGCCAAGGGACCAAGCTGGAGATCAAACGGG

ATGATGCTAAGCCATCTGCCTTCATCTTCCCACCGTCTTCTGAGGAGTTAAGCAGTGGAAGTGCCTCTGTCGTATGC
TTGGTGTATGGCTTCTACCCCAGTGGAGCCACTATCAACTGGAAAGTGGATGGTCTTGCCAAAACAAGTAGC
>HM176143.1 Equus caballus clone 2K444 immunoglobulin kappa light chain V-J region mRNA, partial cds
ATGATGTCGCTGACAAAGGTCCTTATATCTGTGTTGCTCTGGGTCTCAGGTGCCTGTGGGGACATCGTGTTGACCCA
GTCTCCAGAGTCCTTGGCAGTGTCTCTAGGACAGAGGGTCGAGATGAAGTGCAAGACCAGTCAGAGTGCTAACACCT
ACTTAGCTTGGTACCAGCAGAAACCAGGACAGGCTCCTAAACAGCTCATCTACGGTGCATCCAGCAGAGCGTCTGGG
GTCCCTGACCGATTCAGTGGCAGTGGATCTGGGACAGATTTCACTCTCTCCATCAGCAGCCTCCAGGCTGACGATGC
GGCCGTTTATTACTGTCAGCAGTATAACGGCGCTCCTCCGGCGTTCGGCCAAGGGACCAAGCTGGAGATCAAAAGGG
ATGATGCTAAGCCATCTGCCTTCATCTTCCCACCGTCTTCTGAGGAGTTAAGCAGTGGAAGTGCCTCTGTCGTATGC
TTGGTGTATGGCTTCTACCCCAGTGGAGCCACTATCAACTGGAAAGTGGATGGTCTTGCCAAAACAAGTAGC
>HM176142.1 Equus caballus clone 2K60 immunoglobulin kappa light chain V-J region mRNA, partial cds
ATGATGTCGCTGACAAAGGTCCTTATATCTGTGTTGCTCTGGGTCTCAGCTGCCTGTGGGGACATCGTGTTGACCCA
GTCTCCAGAGTCCTTGGCGGTGTCTCTAGGACAGAGGGTCGAGATGAAGTGCAAGGCCAGTCAAAGTGTTAGCAATT
ACTTAGTCTGGTACCAGCAGGTACCAGGACAGGCTCCGAAGCAGCTCATCTACGATGCATCCAGCAGAGCGTCTGGG
GTCCCTGACCGATTCAGTGGCAGTGGATCTGGGACAGACTTCACTCTCTCCATCAGCAGCCTCCAGGCTGAAGATGT
GGCCGTTTATTTCTGTCAGCAGGTCAGGAGTGATCCTCCCACTTTCGGCCAAGGGACCAAGCTGGAGATCAAAAGGG
ATGATGCTAAGCCATCTGCCTTCATCTTCCCACCGTCTTCTGAGGAGTTAAGCAGTGGAAGTGCCTCTGTCGTATGC
TTGGTGTATGGCTTCTACCCCAGTGGAGCCACTATCAACTGGAAAGTGGATGGTCTTGCCAAAACAAGTAGC
>HM176141.1 Equus caballus clone 2K205 immunoglobulin kappa light chain V-J region mRNA, partial cds
ATGATGTCGCTGACAAAGGTCCTTATATCTGTGTTGCTCTGGGTCTCAGGTGCCTGTGGGGACATCGTGTTGACCCA
GTCTCCAGAGTCCTTGGCAGTGTCTCTAGGACAGAGGGTCGAGATGAAGTGCAAGGCCAGTCAGAGTGTTAGAAGCT
ACTTAGCTTGGCACCAGCAGAAACCAGGACAGGCTCCTAAGCAGGTCATCTACAGTGCCACCAGCAGAGCGTCTGGG
GTCCCTGACCGATTCAGTGGCAGTGGATCTGGGACAGATTTCACTCTCACCATCAGCAGCCTCCAGGCTGAAGATGT
GGCCGTTTATTCCTGTCAGCAGTACTATGCAGTTCCTCTCACGTTCGGCCAAGGGACCAAGCTGGAGCTCAAAAGGG
ATGATGCTAAGCCATCTGCCTTCATCTTCCCACCGTCTTCTGAGGAGTTAAGCAGTGGAAGTGCCTCTGTCGTATGC
TTGGTGTATGGCTTCTACCCCAGTGGAGCCACTATCAACTGGAAAGTGGATGGTCTTGCCAAAACAAGTAGC
>HM176140.1 Equus caballus clone 2K375 immunoglobulin kappa light chain V-J region mRNA, partial cds
ATGATGTCGCTGACAAAGGTCCTTATATCTGTGTTGCTCTGGGTCTCAGGTGCCTGGGGGGACATCGTGTTGACCCA
GTTTCCAGAGTCCTTGGCAGTGTCTCTGGGACAGAGGGCCGAGATGAAGTGCAGGGCCAGTCAAGATGTTAGCAGCT
ACTTAGCCTGGTACCAGCAGAAACCAGGCCAGGCTCCTAAGAGACTCATCTACGGTGCATCCGACAGAGCGTCTGGG
GTCCCTGACCGATTCAGTGGCAGAGGGTTTGGGACAGATTTCACTCTCACCATCAGCAGCCTCCAGGCTGAAGATGT
GGCCGTTTATTACTGTCAGCAATATAATACGGTTCCTCGGGAATACACGTTCGGCCAAGGGACCAAACTGGAGATCA
AAAGGGATGATGCTAAGCCATCTGCCTTCATCTTCCCACCGTCTTCTGAGGAGTTAAGCAGTGGAAGTGCCTCTGTC
GTATGCTTGGTGTATGGCTTCTACCCCAGTGGAGCCACTATCAACTGGAAAGTGGATGGTCTTGCCAAAACAAGTAG
C
>HM176139.1 Equus caballus clone 2K373 immunoglobulin kappa light chain V-J region mRNA, partial cds
ATGATGTCGCTGACAAAGGTCCTTATATCTGTGTTGCTCTGGGTCTCAGGTGCCTGTGGGGACATCGTCTTGACCCA
GTCTCCAGAGTCCTTGGCAGTGTCTCTAGGACAGAGGGTCGAGATGAAGTGCAGAACCAGTCACGATGTTAGCACAA
ATTTAGCTTGGTACCAGCAGAAACCAGGACAGGCTCCTAAGCAGCTCATCTACAGTGTATCCAGCAGACGGTCTGGG
GTCCCTGACCGATTCAGTGGCAGTGGATCTGGGACAGATTTCACGATCACCATCAGCAGCCTCCAGGCTGAAGATGT
GGCCGTTTATTACTGCCAGCAGTATAATTTCGGTTGGACGTTCGGTGCCGGGACCAAGTTGGAAATCAAACGGGATG
ATGCTAAGCCATCTGCCTTCATCTTCCCACCGTCTTCTGAGGAGTTAAGCAGTGGAAGTGCCTCTGTCGTATGCTTG
GTGTATGGCTTCTACCCCAGTGGAGCCACTATCAACTGGAAAGTGGATGGTCTTGCCAAAACAAGTAGC
>HM176138.1 Equus caballus clone 2K371 immunoglobulin kappa light chain V-J region mRNA, partial cds
ATGATGTCGCTGACAAAGGTCCTTATATCTGTGTTGCTCTGGGTCTCAGGTGCCTGTGGGGACATCGTGTTGACCCA
GTCTCCAGAGTCCTTGGCAGTGTCTCTAGGACAGAGGGTCGAGATGAAGTGCAAGGCCAGTCAGAGTGCTAGCAGCT
GGGTAAATTGGTACCAGCAGAAACCAGGACAGGCTCCTAAGCAACTCATCTACAAATCATCCAGCAGAGCGTCTGGG
GTCCCTGACCGATTCAGTGGCAGTGGATCTGGGACAGATTTCACTCTCACCATCAGCAGCCTCCAGGCTGAAGATGT
GGCCGTTTATTACTGTCAGCAGTATAATAGTGCTCCTTGGACGTTCGGTGCCGGGACCAAGCTGGAAATCAAACGGG

ATGATGCTAAGCCATCTGCCTTCATCTTCCCACCGTCTTCTGAGGAGTTAAGCAGTGGAAGTGCCTCTGTCGTATGC
TTGGTGTATGGCTTCTACCCCAGTGGAGCCACTATCAACTGGAAAGTGGATGGTCTTGCCAAAACAAGTAGC
>HM176136.1 Equus caballus clone 2K209 immunoglobulin kappa light chain V-J region mRNA, partial cds
ATGATGTCGCTGACAAAGGTCCTTATATCTGTGTTGCTCTGGGTCTCAGGTGCCTGTGGGGACATCGTGTTGACCCA
GTCTCCAGAGTCCTTGGCAGTGTCTCTAGGACAGAGGGTCGAGATGAAGTGCAAGGCCAGTCAGAGTGCTAGCAGCT
ACTTAAATTGGTACCAACAGAAACCAGGACAGGCTCCTAAGCAGCTCATCTACAGTGCATCCAGCAGAGCGTCTGGG
GTCCCTGACCGATTCAGTGGCAGTGGATCGGAGACAGATTTCACTCTCACCATCAGCAGCCTCCAGGCTGAAGATGT
GGCCGTTTATTACTGTCATCAATATAAAGATGCTCCGTGGACGTTCGGTGCCGGGACCAAGCTGGAAATCAAACGGG
ATGATGCTAAGCCATCTGCCTTCATCTTCCCACCGTCTTCTGAGGAGTTAAGCAGTGGAAGTGCCTCTGTCGTATGC
TTGGTGTATGGCTTCTACCCCAGTGGAGCCACTATCAACTGGAAAGTGGATGGTCTTGCCAAAACAAGTAGC
>HM176135.1 Equus caballus clone 2K23 immunoglobulin kappa light chain V-J region mRNA, partial cds
ATGATGTCGCTGACAAAGGTCCTTATATCTGTGTTGCTCTTGGTCTCAGGTGCCTGTGGGGACATCGTGTTGACCCA
GTCTCCAGAGTCCTTGGCAGTGTCTCTAGGACAGAGGGTCGAGATGAAGTGCAAGGCCAGTCAGAGTGCTAGCCGAT
ACTTAGCTTGGTACCAGCAGAAACCAGGACAGACTCCTAAGCAGCTCATCTACGGTATATCCAGCAGAGCGTCTGGG
GTCCCTGACCGATTCAGTGGCAGTGGATCTGGGACAGATTTCACTCTCACCATCAGCAGCCTCCAGGCTGAAGATGT
GGCCATTTATTACTGTCAGCAGTATCTTAGTTTTCCTATTACGTTCGGCCAGGGGACCAAGCTGGAGATCAAACGGG
GTGATGCTAAGCCATCTGCCTTCATCTTCCCACCGTCTTCTGAGGAGTTAAGCAGTGGAAGTGCCTCTGTCGTATGC
TTGGTGTATGGCTTCTACCCCAGTGGAGCCACTATCAACTGGAAAGTGGATGGTCTTGCCAAAACAAGTAGC
>HM176133.1 Equus caballus clone 2K133 immunoglobulin kappa light chain V-J region mRNA, partial cds
ATGATGTCGCTGACAAAGGTCCTTATATCTGTGTTGCTCTGGGTCTCAGGTGCCTGTGGGGACATCGTGTTGACCCA
GTCTCCAGAGTCCTTGGCAGTGTCTCTAGGGCAGAGGGTCGAGATGAAGTGTAGGGCCAGTCAGAGTGCTAGCAGAT
ACTTAGCTTGGTACCAGAAGAAACCAGGACAGGCTCCTAAGCATCTCATCTACAGCGCGTCCAGCAGAGCGTCTGGG
GTCCCTGACCGATTCAGTGGCAGTGGATCTGGGACAGATTTCACTCTCATCATCAGCAGCCTCCAGGCTGAAGATGT
GGCCGTTTATTACTGTCAGCAGTATCATAGTGCTCCTCTTACGTTCGGCCAGGGGACCAAGCTGGAGCTCAAACGGG
ATGATGCTAAGCCATCTGCCTTCATCTTCCCACCGTCTTCTGAGGAGTTAAGCAGTGGAAGTGCCTCTGTCGTATGC
TTGGTGTATGGCTTCTACCCCAGTGGAGCCACTATCAACTGGAAAGTGGATGGTCTTGCCAAAACAAGTAGC
>HM176132.1 Equus caballus clone 2K40 immunoglobulin kappa light chain V-J region mRNA, partial cds
ATGCTGACGCAGACGCAGGTCTTTATATCTGTGTTGCTCTGGGTCTCAGGTGCCTGTGGGGACATCGTGTTGACCCA
GTCTCCAGAGTCCTTGGCAGTGTCTCTGGGACAGAGGGTCGAGATGAAGTGCAAGGCCAGTCAGAGTACTGACATCT
ACTTAGCTTGGTACCAGCAGAAACCAGGACAGGCTCCTAAGTTGCTCACGTACAGTGCATCCAGCAGAGCGTTTGGG
GTCCCTGACCGATTCAGTGGCAGTGGATCTGGGACAGATTTCACTCTCACTATCAGCAGCCTCCAGGCTGAAGATGT
GGCCGTTTATTACTGTCAGCAGTATCACAGTTTTCCCCTTACGTTCGGCCAGGGGACCAAGCTGGAAGTCAAACGGG
ATGATGCTAAGCCATCTGCCTTCATCTTCCCACCGTCTTCTGAGGAGTTAAGCAGTGGAAGTGCCTCTGTCGTATGC
TTGGTGTATGGCTTCTACCCCAGTGGAGCCACTATCAACTGGAAAGTGGATGGTCTTGCCAAAACAAGTAGC
>HM176131.1 Equus caballus clone 2K309 immunoglobulin kappa light chain V-J region mRNA, partial cds
ATGATGTCGCTGACAAAGGTCCTTATATCTGTGTTGCTCTGGGTCTCAGGTGCCTGTGGGGACATCGTGTTGACCCA
GTCTCCAGAGTCCTTGGCAGTGTCTCTAGGACAGAGGGTCGAGATGAACTGCAAGGCCAGTCAGAGTGCTAACATCT
ACTTAGCTTGGTACCAGCAGAAACCAGGACAGGCTCCTAAGCAGATCATCTATAGTGCATCCAGCAGAGCGACTGGG
GTCCCTGACCGCTTCAGTGGCAGTGGATCTGGGACAGATTTCACTCTCACCATCAGCAGCCTCCAGGCTGAAGATGT
GGCCGTTTATTACTGTCAGCAGTATAATAGTGTTCCGCTCTCGTTCGGCCAGGGGACCAAGCTGGAGATCAAACGGG
ATGATGCTAAGCCATCTGCCTTCATCTTCCCACCGTCTTCTGAGGAGTTAGGCAGTGGAAGTGCCTCTGTCGTATGC
TTGGTGTATGGCTTCTACCCCAGTGGAGCCACTATCAACTGGAAAGTGGATGGTCTTGCCAAAACAAGTAGC
>HM176127.1 Equus caballus clone 1K104 immunoglobulin kappa light chain V-J region mRNA, partial cds
ATGCTGACGCAGACGCAGGTCCTTATATCTGTGTTGCTCTGGGTCTCAGGAACCTGTGGGGACGTCGTGATGACCCA
GTCTCCAGACTCCTTGGCAGCGCCTCTAGGACAGAGGGTCGAGCTGAAGTGCAAGGCCAGTCAGGGTGTTGGAAGCA
GATTAGCATGGTACCAGAGCAAACCAGGACAGGCTCCTAAGCGGCTCATTTATGCTACGTCCAACCGAGCACCTGGG
GTCCCTGACCGATTCAGTGGCAGTGGATCTGGGACGGATTTCACTCTCACCATCAGCAGCCTCCAGGCTGAAGATGT
GGCCATGTATTACTGTCTGCAGCATTATAATAATCCTCCTACGTTCGGCCAGGGGACCAAGCTGGAGATCAAACGGG

ATGATGCTAAGCCATCTGCCTTCATCTTCCCACCGTCTTCTGAGGAGTTAAGCAGTGGAAGTGCCTCTGTCGTATGC
TTGGTGTATGGCTTCTACCCCAGTGGAGCCTCTATCAACTGGAAAGTGGATGGTCTTGCCAAAACAAGTAGC
>HM176126.1 Equus caballus clone 1K281 immunoglobulin kappa light chain V-J region mRNA, partial cds
ATGCTGACGCAGACGCAGGTCCTTATATCTGTGTTGCTCTGGGTCTCAGGAGCCTGTGGGGACGTCGTGATGACCCA
GTCTCCAGAGTCCTTGGCAGTGTCTCTAGGACAGAGGGTCGAGATGAAGTGCAAGGCCAGTCAGAGTGTTAGCACCT
ACTTAGCTTGGTTCCAGCAGAAACCAGGACAGGCTCCCTTGTTGCTCATTTATGCTGCATCCAGCAGAGCATCTGGG
GTCCCTGACCGATTCAGTGGCAGTGGATCTGGGACGGATTTCACTCTCACCATCAGCAGCCTCCAGGCTGAAGATGT
GGCCATTTATTACTGTATGCAGTTAGGTGCCAATCCTCGGACGTTCGGTGCCGGGACCAAGCTGGAAATCAAGCGGG
ATGATGCTAAGCCATCTGCCTTCATCTTCCCACCGTCTTCTGAGGAGTTAAGCAGTGGAAGTACCTCTGTCGTATGC
TTGGTGTATGGCTTCTACCCCAGTGGAGCCACTATCAACTGGAAAGTGGGTGGTCTTGCCAAAACAAGTAGC
>HM176124.1 Equus caballus clone 1K346 immunoglobulin kappa light chain V-J region mRNA, partial cds
ATGCTGACGCAGACGCAGGTCCTTATAACTGTGTTGCTCTGGGTCTCAGGAGCCTGCGGGGACGTCGTGTTGACCCA
GTCTCCAGAGTCCTTGGCAGTGTCTCTAGGACAGAGGGTCGAGATGAAGTGCAAGGCCAGTCAGATGATTAGAGACT
ACGTAGCTTGGTACCAACAGAAACCAGGACAGGCGCCTAAGCGGCTCATCTATCGCGCATCCACCAGGACATCTGGG
GTCCCTGACCGATTCAGTGGCAGTGGATCTGGGACGGATTTCACTCTCACCATCAGCAGCCTCCAGGCTGAAGATGT
GGCCATTTATTACTGTATGCAGGTTTATTACAATCCATTCACGTTCGGCCAAGGGACCAAGCTGGAGATCAAAAGGG
ATGATGCTAAGCCATCTGCCTTCATCTTCCCACCGTCTTCTGAGGAGTTAAGCAGTGGAAGTGCCTCTGTCGTATGC
TTGGTGTATGGCTTCTACCCCAGTGGAGCCACTATCAACTGGAAAGTGGATGGTCTTGCCAAAACAAGTAGC
>HM176123.1 Equus caballus clone 1K330 immunoglobulin kappa light chain V-J region mRNA, partial cds
ATGCTGACGCAGACGCAGGTCCTTATATCTGTGTTGCTCTGGGTCTCAGGAGCCTGTGGGGACGTCGTGATGACCCA
GTCTCCAGAGACATTGGCAGTGTCTCTAGGACAGAGGGTCGAGATGACTTGTAAGGCCAGTCAGAGTGTTGGTAATT
ACTTAGGTTGGTACCAGCAGAAACCAGGGCAGGCTCCTAAGGCGCTCATCGTTGTTGCATCCAGAAGAGCAGTCGGG
GTCCCTGACCGATTCAGTGGCAGCGGATCTGGGACGGAATTCACTCTCACCATCAGCGAAGTCCAGGCTGAAGATGT
GGCCATTTATTACTGTATGCAGCATTCCTCCCTATATACTTTCGGCCAAGGGACCAAGCTGGAGATCAAAAGGGATG
ATGCTAAGCCATCTGCCTTCATCTTCCCACCGTCTTCTGAGGAGTTAAGCAGTGGAAGTGCCTCTGTCGTATGCTTG
GTGTATGGCTTCTACCCCAGTGGAGCCACTATCAACTGGAAAGTGGATGGTCTTGCCAAAACAAGTAGC
>HM176121.1 Equus caballus clone 1K11 immunoglobulin kappa light chain V-J region mRNA, partial cds
ATGCTGACGCAGACGCAGGTCCTTATATCTGTGTTGCTCTGGGTCTCAGGAGCCTGTGGGGACGTCGTGATGACCCA
GTCTCCAGAGTCCTTGGCAGTGTCTCTAGGACAGAGGGTCGAGATGAAGTGCAAGGCCAGTCAGAATGTTTTCAGCT
ACTTAGCTTGGTACCAGCGGAAGCCAGGACAGGCTCCTAAGCGACTCATCTATAGTGCATACAGCAGACCATCTGGG
GTCCCTGACCGATTCAGTGGCAGTGGATCTGGGACGGATTTCACTCTCACCATCAGCAGCCTCCAGGCTGAAGATGT
GGCAGACTATGTCTGTATGCAGCATTATTACCATCCCCCCACGTTCGGCCAGGGGACCAAGCTGGAGATCAAAAGGG
ATGATGCTAAGCCATCTGCCTTCATCTTCCCACCGTCTTCTGAGGAGTTAAGCAGTGGAAGTGCCTCTGTCGTATGC
TTGGTGTATGGCTTCTACCCCAGTGGAGCCACTATCAACTGGAAAGTGGATGGTCTTGCCAAAACAAGTAGC
>HM176119.1 Equus caballus clone 1K241 immunoglobulin kappa light chain V-J region mRNA, partial cds
ATGCTGACGCAGACGCAGGTCCTTATATCTGTGTTGCTCTGGGTCTCAGGAGCCTGTGGGGACGTCGTGATGACCCA
GTCTCCAGAGTCCTTGGCAGTGTCTCTAGGACAGAGGGTCGAGATGAAGTGCAAGGCCAGTCAGAGTGTTAGCGTAT
ATTTAGCTTGGTACCAGCAGAAACCAGGACAGGCTCCTAAGCGGCTCATCTACGGTGCATCCAGCAGAGCATCTGGG
GTCCCTGACCGATTCAGTGGCAGTGGATCTGGGACGGATTTCACTCTCACCATCAGCAGCCTCCAGGCTGAAGATGT
GGCCATTTATTACTGTATGCAGCATTATACCAATCCTCGGACGTTCGGCCAAGGGACCAAACTGGCTATCAAAAGGG
ATGATGCTAAGCCATCTGCCTTCATCTTCCCACCGTCTTCTGAGGAGTTAAGCAGTGGAAGTGCCTCTGTCGTATGC
TTGGTGTATGGCTTCTACCCCAGTGGAGCCACTATCAACTGGAAAGTGGATGGTCTTGCCAAAACAAGTAGC
>HM176117.1 Equus caballus clone 1K96 immunoglobulin kappa light chain V-J region mRNA, partial cds
ATGCTGACGCAGACGCAGGTCCTTATATCTGTGTTGCTCTGGGTCTCAGGAGCCTGTGGGGACGTCGTGATGACCCA
GTCTCCAGAGTCCTTGGCAGTGTCTCTAGGGCAGAGGGTCGAGATGAAGTGTAAGGCCAGTCAGAGTATTGGCGTAA
GTGTATCTTGGTTCCAACAGAAACCAGGACAGGCTCCTAAGCGGCTCATCTATTATGCATCCAGCAGACAATCTGGG
GTCCCTGACCGATTCAGTGGCAGTGGATCTGGGACGGATTTCACTCTCACCATCAGCAGCCTCCAGGCTGAAGATGT
GGCCATTTATTACTGTATGCAGCAATATAATGCGCTTACGTTCGGCCAGGGGACCAAGCTGGAGATCAAACGGGATG
ATGCTAAGCCATCTGCCTTCATCTTCCCACCGTCTTCTGAGGAGTTAAGCAATGGAAGTGCCTCTGTCGTATGCTTG
GTGTATGGCTTCTACCCCAGTGGAGCCACTATCAACTGGAAAGTGGATGGTCTTGCCAAAACAAGTAGC

>HM176116.1 Equus caballus clone 1K124 immunoglobulin kappa light chain V-J region mRNA, partial cds
ATGCTGACGCAGACGCAGGTCCTTATATCTGTGTTGCTCTGGGTCTCAGGAGCCTGTGGGGACGTCGTGATGACCCA
GTCTCCAGAGTCCTTGGCAGTGTCTCTAGGACAGAGGGTCGAGATGAAGTGCAAGGCCAGTCAGAGTGTTAGCAGCA
AGTTAGCTTGGTACCAGCAGAAACCAGGACAGGCTCCTAAGCGGCTCATCTATGCTGCATCCAGCAGAGCATCTGGG
GTCCCTGACCGATTCAGTGGCAGTGGATCTGGGACGGATTTCACTCTCACCATCAGCAGCCTCCAGGCTGAAGATGT
GGCCAGTTATTACTGTATGCAGCATTGGACACATCCTATTACGTTCGGCCAGGGGACCAAGCTGGAGATCGTACGGG
ATGATGCTAAGCCATCTGCCTTCATCTTCCCACCGTCTTCTGAGGAGTTAAGCAGTGGAAGTGCCTCTGTCGTATGC
TTGGTGTATGGCTTCTACCCCAGTGGAGCCACTATCAACTGGAAAGTGGATGGTCTTGCCAAAACAAGTAGC
>HM176114.1 Equus caballus clone 1K363 immunoglobulin kappa light chain V-J region mRNA, partial cds
ATGCTGACGCAGACGCAGGTCCTTATATCTGTGTTGCTCTGGGTCTCAGGAGCCTGTGGGGACGTCGTGATGACCCA
GTCTCCAGAGTCCTTGGCAGTGTCTCTAGGACAGAGGGTCGAGATGAAGTGCAAGGCCAGTCAGAGTGTTAGCAGCT
ACTTAGCTTGGTACCAGCAGAAACCAGGACAGGCTCCTAAGCGAATCATCTATGCTGCATCCAGCAGAGCATCTGGG
GTCCCTGACCGATTCAGTGGCAGTGGATCTGGGACGGATTTCACTCTCACCATCAGCAGCCTCCAGGCTGAAGATGT
GGCCATTTATTACTGTATGCACAATCTTAATAATCCAATTACGTTCGGCCAGGGGACCAAGCTGGAGATCAAACGGG
ATGATGCTAAGCCATCTGCCTTCATCTTCCCACCGTCTTCTGAGGAGTTAAGCAGTGGAAGTGCCTCTGTCGTATGC
TTGGTGTATGGCTTCTACCCCAGTGGAGCCACTATCAACTGGAAAGTGGATGGTCTTGCCAAAACAAGTAGC
>HM176113.1 Equus caballus clone 1K24 immunoglobulin kappa light chain V-J region mRNA, partial cds
ATGCTGACGCAGACGCAGGTCCTTATATCTGTGTTGCTCTGGGTCTCAGGAGCCTGTGGGGACGTCGTGATGACCCA
GTCTCCAGAGTCCTTGGCAGTGTCTCTAGGACAGAGGGTCGAGATGAAGTGCAAGGCCAGTCAGAGTGTTGAGAGAT
ACTTAGCTTGGTACCAGCAGAAGCCAGGACAGGCTCCTAAGCGACTCATCTATTCTGCATCCACTAGATCATCTGGG
GTCCCTGACCGATTCAGTGGCAGTGGATCTGGGACGGATTTCACTCTCACCATCAGCAGCCTCCAGGCTGAGGATGT
GGCCAATTATTACTGTATGCAGCATGTTGTTGATCCCCTTACGTTCGGCCAGGGGACCAAGCTGGAGATCAAACGGG
ATGATGCTAAGCCATCTGCCTTCATCTTCCCACCGTCTTCTGAGGAGTTAAGCAGTGGAAGTGCCTCTGTCGTATGC
TTGGTGTATGGCTTCTACCCCAGTGGAGCCACTATCAACTGGAAAGTGGATGGTCTTGCCAAAACAAGTAGC
>HM176111.1 Equus caballus clone 1K355 immunoglobulin kappa light chain V-J region mRNA, partial cds
ATGCTGACGCAGACGCAGGTCCTTATATCTGTGTTGCTCTGGGTCTCAGGAGCCTGTGGGGACGTCGTGATGACCCA
GTCTCCAGAGTCCTTGGCAGTGTCTCTAGGACAGAGGGTCGAGATGAAGTGCAAGGCCAGTCAGAGTGTTAGCAGCG
GCTTATCTTGGTACCAGCAGAAACCAGGACAGGCTCCTAAGCGGCTCATCGTTAATGCATCCAACAGAAGGCCTGGG
GTCCCCGACCGATTCAGTGGCAGTGGATCTGGGACGGATTTCACTCTCACCATCAGCAGCCTCCAGGCTGAAGATGT
GGCCATTTATTACTGTATGCAGCATGGTAATGATCCGTTTACGTTCGGCCAGGGGACCAAGCTGGAGATCAAACGGG
ATGATGCTAAGCCATCTGCCTTCATCTTCCCACCGTCTTCTGAGGAGTTAAGCAGTGGAAGTGCCTCTGTCGTATGC
TTGGTGTATGGCTTCTACCCCAGTGGAGCCACTATCAACTGGAAAGTGGATGGTCTTGCCAAAACAAGTAGC
>HM176110.1 Equus caballus clone 1K183 immunoglobulin kappa light chain V-J region mRNA, partial cds
ATGCTGACGCAGACGCAGGTCCTTATATCTGTGTTGCTCTGGGTCTCAGGAGCCTGTGGGGACGTCGTGATGACCCA
GTCTCCAGAGTCCTTGGCAGTGTCTCTAGGACAGAGGGTCGAAGTGAAGTGCAAGGCCAGTCAGAGTGTGCGCAATT
TTTTAGTTTGGTACCAACAAAAACCAGGACAGGCTCCTAAGCGAATCATCTATGGTGCATCCAGCAGAGCATCTGGG
GTCCCTGACCGATTCAGTGGCAGTGGATCGGGGACGGATTTCACCCTCACCATCAGCAGTCTCCAGGCTGAAGATTC
GGCCATTTATTACTGTATGCAGAGTTATAGTAATCCGCCTACGTTCGGCCAGGGGACCAAGCTGGAGATCAAACGGG
ATGATGCTAAGCCATCTGCCTTCATCTTCCCACCGTCTTCTGAGGAGTTAAGCAGTGGAAGTGCCTCTGTCGTATGC
TTGGTGTATGGCTTCTACCCCAGTGGAGCCACTATCAACTGGAAAGTGGATGGTCTTGCCAAAACAAGTAGC
>HM176109.1 Equus caballus clone 1K73 immunoglobulin kappa light chain V-J region mRNA, partial cds
ATGCTGACGCAGACGCAGGTCCTTATATCTGTGTTGCTCTGGGTCTCAGGAGCCTGTGGGGACGTCGTGATGACCCA
GTCTCCAGAGTCCTTGGCAGTGTCTCTAGGACAGAGGGTCGAGATGAGGTGCAAGGCCAGTCAGAGTATTACCACGT
ACTTAGCTTGGTACCAGCAGAAACCAGGACAGGCTCCTAAGCGGCTCATCTATTATGCATCCATCAGAGCATCTGGG
GTCCCTGACCGATTCAGTGGCAGTGGATCTGGGACGGATTTCACTCTCACCATCAGCAGCCTCCAGGCTGAAGATGT
GGCCATTTATTACTGTATGCAGAATTATAATAATCCTCTTACGTTCGGCCAGGGGACCAAGCTGGAGATCAAACGGG
ATGATGCTAAGCCATCTGCCTTCATCTTCCCACCGTCTTCTGAGGAGTTAAGCAGTGGAAGTGCCTCTGTCGTATGC
TTGGTGTATGGCTTCTACCCCAGTGGAGCCACTATCAACTGGAAAGTGGATGGTCTTGCCAAAACAAGTAGC

>HM176108.1 Equus caballus clone 2K308 immunoglobulin kappa light chain V-J region mRNA, partial cds
ATGCTGACGCAGACGCAGGTCTTTATATCTGTGTTGCTCTGGGTCTCAGGAGCCTGTGGGGACGTCGTGATGACCCA
GTCTCCAGACTCCTTGGCAGCGTCTCTAGGACAGAGAGTCGAGATGAAGTGCAGGGCCAGTCAGAGTGTGAGCAGCT
ACTTAGTTTGGTACCAGCAGAAACCAGGACAGGCTCCTAAGCGGCTCATCTATGCTGCATCCAGCAGAGCATCTGGG
GTCCCTGACCGATTCAGTGGCAGTGGATCTGGGGCGGATTTCACTCTCACCATCAGCAGCCTCCAGGCTGAAGATGT
GGCCATTTATTACTGTCTGCAAACTCTTGTTAATCCGTACACTTTCGGCCAAGGGACCAAACTGGAGATCAAAAGGG
ATGATGCTAAGCCATCTGCCTTCATCTTCCCACCGTCTTCTGAGGAGTTAAGCAGTGGAAGTGCCTCTGTCGTATGC
TTGGTGTATGGCTTCTACCCCAGTGGAGCCACTATCAACTGGAAAGTGGATGGTCTTGCCAAAACAAGTAGC
>HM176107.1 Equus caballus clone 2K49 immunoglobulin kappa light chain V-J region mRNA, partial cds
ATGCTGACACAGACGCAGGTCTTTATATCTGTGTTGCTCTGGGTCTCAGGAGCCTGTGGGGACGTCGTGATGACCCA
GTCTCCAGACTCCTTGGCAGCGTCTCTAGGTCAGAGAGTCGAGATGAAGTGCAAGGCCAGTCAGAGTGTTAGCAGCT
ACTTAACTTGGTACCAGCAGAAACCAGGACAGGCTCCTAAGCGACTCATCTATGCTGCATCCAGCAGAGCATCTGGG
GTCCCTGACCGATTCAGTGGCAGTGGATCTGGGACGGATTTCACTCTCACCATCAGCAGCCTCCAGGCTGAAGATGT
GGCGATTTATTACTGTCTGCAGCATGGTGTTGATCCTCTTACGTTCGGCCAGGGGACCAAGCTGGAGATCAAACGGG
ATGATGCTAAGCCATCTGCCTTCATCTTCCCACCGTCTTCTGAGGAGTTAAGCAGTGGAAGTGCCTCTGTCGTATGC
TTGGTGTATGGCTTCTACCCCAGTGGAGCCACTATCAACTGGAAAGTGGATGGTCTTGCCAAAACAAGTAGC
>HM176106.1 Equus caballus clone 2K10 immunoglobulin kappa light chain V-J region mRNA, partial cds
ATGCTGACGCAGACGCAGGTCTTTATATCTGTGTTGCTCTGGGTCTCAGGAGCCTGTGGGGACATCGTGATGACCCA
GTCTCCAGACTCCTTGGCAGCGTCTCTAGGACAGAGAGTCGAGATGAAGTGCAAGGCCAGTCAGAGTGTTAGCAACA
GGTTGACTTGGTACCAGCAGAAACCAGGACAGGCTCCTAAGCGGATCATCTATGCTGCATCCACAAGAGAATCTGGG
GTCTCTGACCGATTCAGTGGCAGTGGATCTGGGACGGATTTCACGCTCACCATCAGCAGCCTCCAGGCTGAAGATGT
GGCCTCTTATTACTGTCTGCAGAGTACTAGTAATCCGCTTACGTTCGGCCAGGGGACCAAGCTGGAGATCAAGCGGG
ATGATGCTAAGCCATCTGCCTTCATCTTCCCACCGTCTTCTGAGGAGTTAAGCAGTGGAAGTGCCTCTGTCGTATGC
TTGGTGTATGGCTTCTACCCCAGTGGAGCCACTATCAACTGGAAAGTGGATGGTCTTGCCAAAACAAGTAGC
>HM176104.1 Equus caballus clone 2K388 immunoglobulin kappa light chain V-J region mRNA, partial cds
ATGATGTCGCTGACAAAGGTCCTTATATCTGTGTTGCTCTGGGTCTCAGGAGCCTGTGGGGACGTCGTGATGACCCA
GTCTCCAGACTCCTTGGCAGCGTCTCTAGGACAGAGAGTCGAGATGAAGTGTAAGGCCAGTCAGAGTGTTAGCACAT
ACTTAGCTTGGTACCAGCACAAACCAGGTCAGGCTCCTAAGCGGGTCATCTATGCTGCATCCAGCAGAGCATCTGGG
GTCCCTGACCGATTCAGTGGCAGTGGATCTGGGACGGATTTCACTCTCACCATCAGCAGCCTCCAGGCTGAAGATGT
GGCCATTTATTACTGTGTGCAGCATTATGCCAGACCTCCCCTGTTCGGCCAAGGGACCAAGCTGGAGATCAAAAGGG
ATGATGCTAAGCCATCTGCCTTCATCTTCCCACCGTCTTCTGAGGAGTTAAGCAGTGGAAGTGCCTCTGTCGTATGC
TTGGTGTATGGCTTCTACCCCAGTGGAGCCACTATCAACTGGAAAGTGGATGGTCTTGCCAAAACAAGTAGC
>HM176101.1 Equus caballus clone 2K250 immunoglobulin kappa light chain V-J region mRNA, partial cds
ATGCTGACGCAGACGCAGGTCCTTATATCTGTGTTGCTCTGGGTCTCAGGAGCCTGTGGGGACGTCGTGATGACCCA
GTCTCCAGACTCCTTGGCAGCGTCTCTGGGACAGAGAGTCGAGATGAAGTGCAAGGCCAGTCAGAGTGTTAGCAGCT
ACTTAGCTTGGTACCAGCACAAACCAGGACAGGCTCCTAAGCGGGTCATCTATAGTGCATCCAGCAGAGCATCTGGG
GTCCCTGACCGATTCACGGGCAGTGGATCTGGGACGGATTTCACTCTCACCATTAGCAGCCTCCAGGCTGAAGATGT
GGCCATTTATTACTGTATGCAGCATTATAATAAGCCCCACACGTTCGGCCAAGGGACCAAGTTGGAGATCAAAAGGG
ATGATGCTAAGCCATCTGCCTTCATCTTCCCACCGTCTTCTGAGGAGTTAAGCAGTGGAAGTGCCTCTGTCGTATGC
TTGGTGTATGGCTTCTACCCCAGTGGAGCCACTATCAACTGGAAAGTGGATGGTCTTGCCAAAACAAGTAGC
>HM176100.1 Equus caballus clone 2K372 immunoglobulin kappa light chain V-J region mRNA, partial cds
ATGCTGACGCAGACGCAGGTCCTTATATCTGTGTTGCTCTGGGTCTCAGGAGCCTGTGGGGACGTCGTGATGACCCA
GTCTCCAGACTCCTTGGCAGCGTCTCTAGGACAGAGAGTCGAGATGAAGTGCAAGGCCAGTCAGAGTGTTAGCAGTT
ACTTAGCTTGGTACCAGCACAAACCAGGACAGGCTCCTAAGCGGGTCATCTATTCTGCATCCAGCAGAGCATCTGGG
GTCCCTGACCGATTCAGTGGCAGTGGATCTGGGACGGATTTCACTCTCACCATCAGCAGCCTCCAGGCTGAAGATGT
GGCCATTTATTACTGTACGCAGCATTATACTTCTCCTTATACGTTCGGCCAGGGGACCAAGCTGGAGATCAAACGGG
ATGATGCTAAGCCATCTGCCTTCATCTTCCCACCGTCTTCTGAGGAGTTAAGCAGTGGAAGTGCCTCTGTCGTATGC
TTGGTGTATGGCTTCTACCCCAGTGGAGCCACTATCAACTGGAAAGTGGATGGTCTTGCCAAAACAAGTAGC
>HM176099.1 Equus caballus clone 2K352 immunoglobulin kappa light chain V-J region mRNA, partial cds

```
ATGCTGACGCAGACGCAGGTCCTTATATCTGTGTTGCTCTGGGTCTCAGGAGCCTGTGGGGACGTCGTGATGACCCA
GTCTCCAGACTCCTTGGCAGCGTCTCTAGGACAGAGAGTCGAGATGAAGTGCAAGGCCAGTCAGAGTGTTAGCAGCA
ACTTAGCTTGGTACCAGCACAAACCAGGACAGGCTCCTAAGCGGGTCATCTATGATGCATCCAGCAGAGCATCTGGG
GTCCCTGACCGATTCAGTGGCAGTGGATCTGGGACGGATTTCACTCTCACCATCAGCAGCCTCCAGGCTGAAGATGT
GGCCATTTATTACTGTATGCAGCATTATAATAATCCTCCTACGTTCGGCCAGGGGACCAAGCTGGAGATCAAACGGG
ATGATGCTAAGCCATCTGCCTTCATCTTCCCACCGTCTTCTGAGGAGTTAAGCAGTGGAAGTGCCTCTGTCGTATGC
TTGGTGTATGGCTTCTACCCCAGTGGAGCCACTATCAACTGGAAAGTGGATGGTCTTGCCAAAACAAGTAGC
>HM176098.1 Equus caballus clone 2K99 immunoglobulin kappa light chain V-J
region mRNA, partial cds
ATGCTGACGCAGACGCAGGTCCTTATATCTGTGTTGCTCTGGGTCTCAGGAGCCTGTGGGGACGTCGTGATGACCCA
GTCTCCAGACTCCTTGGCAGCGTCTCTAAGACAAAGAGTCGAGATGAAGTGCAAGGCCAGTCAGAGTGTTGGCCGAT
ACTTAGCTTGGTACCAGCACAAACCAGGACAGGCTCCTAAGCGGGTCATCTATTCTGCATCCAGCAGACCATCTGGG
GTCCCTGACCGATTCAGTGGCAGTGGATCTGGGACGGATTTCACTCTCACCATAAGCAGCCTCCAGGCTGAAGATGT
GGCCATTTATTACTGTATGCAGTATCAAAGTAATCTCCCCACGTTCGGCCAGGGGACCAAGCTGGAGATCAAACGGG
ATGATGCTAAGCCATCTGCCTTCATCTTCCCACCGTCTTCTGAGGGGTTAAGCAGTGGAAGTGCCTCTGTCGTATGC
TTGGTGCATGGCTTCTACCCCAGTGGAGCCACTATCAACTGGAAAGTGGATGGTCTTGCCAAAACAAGTAGC
>HM176096.1 Equus caballus clone 2K193 immunoglobulin kappa light chain V-J
region mRNA, partial cds
ATGCTGACGCAGACGCAGGTCCTTATATCTGTGTTGCTCTGGGTCTCAGGAGCCTGTGGGGACGTCGTGATGACCCA
GTCTCCAGACTCCTTGGCAGCGTCTCTAGGACAGAGAGTCGAGATGAAGTGCAAGGCCAGTCAGAGTATTGACAACT
ACTTAGCTTGGTACCAGCACAAACCAGGACAGGCTCCTAAGCGGGTCATCGCTGCTGCATCCAGAAGAGCATCTGGG
GTCCCTGACCGATTCAGTGGCAGTGGATCTGGGACGGATTTCACTCTCACCATCAGCAGCCTCCAGGCTGAAGATGT
GGCCATTTATTACTGTATGCAGCATTATAATTCTCCGCTTACGTTCGGCCAGGGGACCAAGCTGGAGATCAAACGGG
ATGATGCTAAGCCATCTGCCTTCATCTTCCCACCGTCTTCTGAGGAGTTAAGCAGTGGAAGTGCCTCTGTCGTATGC
TTGGTGTATGGCTTCTACCCCAGTGGAGCCACTATCAACTGGAAAGTGGATGGTCTTGCCAAAACAAGTAGC
>HM176094.1 Equus caballus clone 2K41 immunoglobulin kappa light chain V-J
region mRNA, partial cds
ATGCTGACGCAGACGCAGGTCCTTATATCTGTGTTGCTCTGGGTCTCAGGAGCCTGTGGGGACGTCGTGATGACCCA
GTCTCCAGACTCCTTGGCAGCGTCTCTAGGACAGAGAGTCGAGATGAAGTGCAAGGCCAGTCAGAGTGTTAGCAACC
ACTTAGCTTGGTACCAACACAAACCAGGACAGGCTCCTAAGCGGCTGATGTTTGGTGCATCTACCAGACAGTCTGGG
GTCCCTGACCGTTTCAGTGGCAGTGGATCTGGGACGGATTTCACTCTCACCATCAGCAGCCTCCAGGCTGAAGATGT
GGCCATTTATTACTGTATGCAGCATTGGTATAATCCCCTTACGTTCGGCCAGGGGACCAAGCTGGAGATCAAACGGG
ATGATGCTAAGCCATCTGCCTTCATCTTCCCACCGTCTTCTGAGGAGTTAAGCAGTGGAAGTGCCTCTGTCGTATGC
TTGGTGTATGGCTTCTACCCCAGTGGAGCCACTATCAACTGGAAAGTGGATGGTCTTGCCAAAACAAGTAGC
```

1.3 **Supplementary Data 3. NCBI Genbank Equine Ig VL mRNA.** A total of 353 NCBI Genbank sequences, annotated as Equus caballus Ig VL sequences which constituted the dataset employed for the designing of the EquPD v2020 primer set.

```
>KF985142.1 Equus caballus clone AdultB02 immmunoglobulin lambda light chain
variable region (IGL) mRNA, partial cds
AGCTCTGCTTCAGCTGTGGGGCCACAGAAGGCAGGACTCGGTGAAGATCTCCACCATGGCCTGGTCCCCT
CTCCTCCTCACCCTCATCGCTCTCTGCACAGGATCCTGGGCCCAGTCTGTGACCCAGCCCGCCTCAGTGT
CTGTGACCCCGGGCCAGACAGTCACCATCTCCTGCACTGGAAGCAGCACCAACATCGGACATTATAATGT
AGGCTGGTTCCAACAGAACCCAGGAACAGCCCCCAAAACCCTCATCTATGGTGTGAACGAACGAGGCTCA
GGGGTCCCAGATCGATTCTCTGGCTCCAAGTCTGGCAACACAGCCACCCTGACCATCGCTGGGGTCCAGA
CTGAGGACGAGGCCGATTATTACTGTGGTGTCTATGACACCGACGCTACTACTAACCTGTTCGGCGGAGG
CACCCACTTGACCATCGCAGGTGGTCCCACGTCTACACCCTCGGTCTCTCTCTTCCCGCCCTCCTCTGAG
GAGCTCAGCGCCAACAAGGCCACAGTGGTGTGTCTCATCAGCGACTTCTCCCCCAGCGGCCTGGAGGTGA
TCTGGAAGGTAAATGACGCTGTCACCACCGACGGCGTCCAGACCACCAGGTCCTCGAAACAGAGCAACGG
CAAGTACGCGGCCAGCAGCTACCTGACGCGGACTTCCGCACAGTGGAAATCGTACAGCAGCGTCAGCTGC
CAGGTCAAGCACCAAGGGAAAACC
>KF985125.1 Equus caballus clone FoalT09 immmunoglobulin lambda light chain
variable region (IGL) mRNA, partial cds
AGCTCTGCTTCGGCTGTGGGGCCACAGAAGGCAGGACTCGGTGACGATCTCCACCATGGCCTGGTCCCCT
CTCCTCCTCACCCTCATCGCTCTCTGCACAGGATCCTGGGCCCAGTCTCTGACCCAGCCCGCCTCAGTGT
CTGGGACCCTGGGCCAGACAGTCACCATCTCCTGCTCTGGAAGCAGCTCCAACATCGGGTATACATATAG
TGCTGTGGGCTGGTACCAACAGATCCCAGGAACAGCCCCCAAAACCCTCATCTATGGTAATAACAAACGA
GCCTCAGGGGTCCCAGATCGATTCTCTGGCTCCAAGTCTGGCAACACAGCCACCCTGACCATCTCTGGGC
TTCAGGCTGAGGACGAGGCCGATTATTACTGTGGTTCCTATTACAGCAGTGATGGTGCATTCGGCGGAGG
CACCCACCTGACCATCGCAGGTGGTCCCACGTCTACACCCTCGGTCTCTCTCTTCCCGCCCTCCTCTGAG
GAGCTCAGCGCCAACAAGGCCACAGTGGTGTGTCTCATCAGTGACTTCTCCCCCAGCGGCTTGGAGGTGA
TCTGGAAGGTAAATGACGCTGTCACCACCGACGGCGTCCAGACCACCAGGTCCTCGAAACAGAGCAACGG
CAAGTACGCGGCCAGCAGCTACCTGACGCGGACTTCCGCACAGTGGAAATCGTACAGCAGCGTCAGCTGC
CAGGTCAAGCACCAAGGGAAAACC
>KF985120.1 Equus caballus clone FoalT35 immmunoglobulin lambda light chain
variable region (IGL) mRNA, partial cds
AAGCTCTGCTTCAGCTGTGGGGACACAGAAGGCAGGACTCGGTGACGATCTCCACCATGGCCTGGTCCCC
TCTCCTCCTCACCCTCATCGCTCTCTGCACAGGATCCTGGGCCCAGTCTCTGACTCAGCCCGCCTCAGTG
TCTGGGACCCTGGGCCAGACAGTCACCATCTCCTGCACTGGAAGCAGCTCCAACATCGGAGATAGTAAAG
GTTATGTGGGCTGGTACCAACAGATCCCAGGAACAGCCCCCAAACTCCTCATCTATCGTAGTAACAAACG
ACCCTCAGGGGTCCCAGATCGATTCTCTGGCTCCAAGTCTGGGAACACAGGCACCCTGACCATCTCTGGG
GTCCAGGCTGAGGACGAGGCCGATTATTACTGTGCAGCAGGAGACAGCAGCCTGAGTAGTGTTGTATTCG
GCGGAGGCACCCACCTGACCATCGCAGGTGGTACCCCGTCTGCACCCTCGGTCTCTCTCTTCCCGCCCTC
CTCTGAGGAGCTCAGCGCCAACAAGGCCACAGTGGTGTGTCTCATCAGTGACTTCTCCCCCAGCGACTTG
ACGGTGAGCTGGAAGGTAAATGGCGCCGCCACCACCCAGGGCGTCCAGACCACCAAGCCCTCGAAACAGA
GCAACGGCAAGTACGCAGCCAGCAGCTACCTGTCGCTGACCCCCAGCCAGTGGAAATCGTCCAGCAGCGT
CAGCTGCCAGGTCACGCACCAAGGGAAAACC
>KF985113.1 Equus caballus clone FoalT22 immmunoglobulin lambda light chain
variable region (IGL) mRNA, partial cds
AAGCTCTGCTTCAGCTGTGGGGCCACAGAAGGCAGGACTCGGTGACAATCTCCACCATGGCCTGGTCCCC
TCTCCTCCTCACCCTCATCGCTCTCTGCACAGGATCCTGGGCCCAGTCTCTGACTCAGCCCGCCTCAGTG
TCTGGGACCCTGGGCCAGACAGTCACCATCTCCTGCACTGGAAGCAGCTCCAACATAGGTCTTTATATGG
GCTGGTACCAACAGATCCCAGGAACAGCCCCCAAAACCCTCATCTATGGTAATAACAAACGAGCCTCAGG
GGTCCCAGATCGATTCTCTGGCTCCAAGTCTGGCAACACAGCCACCCTGACCATCTCTGGGCTTCAGGCT
GAGGACGAGGCCGATTATTACTGTGGTTCCTATTACAACAGTGATAATAGTGCTGTATTCGGCGGAGGCA
CCCACCTGACCATCGCAGGTGGTCCCACGTCTACACCCTCGGTCTCTCTCTTCCCGCCCTCCTCTGAGGA
GCTCAGCGCGAACAAGGCCACAGTGGTGTGTCTCATCAGTGACTTCTCCCCCAGCGACTTGACGGTGAGC
TGGAAGGTAAATGGCGCCGCCACCACCCAGGGCGTCCAGACCACCAAGCCCTCGAAACAGAGCAACGGCA
AGTACGCAGCCAGCAGCTACCTGTCGCTGACCCCCAGCCAGTGGAAATCGTCCAGCAGCGTCAGCTGCCA
```

GGTCACGCACCAAGGGAAAACC
>KF985096.1 Equus caballus clone NeonateT21 immmunoglobulin lambda light chain variable region (IGL) mRNA, partial cds
GGGCCACAGAAGGCAGGACTCGGTGAAGATCTCCACCATGGCCTGGTCCCCTCTCCTCCTCACCCTCATC
GCTCTCTGCACAGGATCCTGGGCCCAGTCTGTGACTCAGCCCGCCTCAGTGTCTGGGACCCTGGGCCAGA
CAGTCACCATCTCCTGCACTGGAAGCATCTCCAGCATAGGTGCTTATGTGGACTGGTACCAACAGATCCC
AGGAACAGCCCCCAAAACCGTCATCTATGCTACTAACAGCCAACCCTCAGGGGTCCCAGATCGATTCTCT
GGCTCCAAGTCTGGCAACACAGCCACCCTGACCATCACTGGGCTCCAGGCTGAGGACGAGGCTGATTATT
ACTGTGGTATCTATGACAACAGCCTGAGTAGTGTTGTATTCGGCGGAGGCACCCACCTGACCATCGCAGG
TGGTCCCACGTCTACACCCTCGGTCTCTCTCTTCCCGCCCTCCTCTGAGGAGCTCAGCGCCAACAAGGCC
ACAGTGGTGTGTCTCATCAGTGACTTCTCCCCCAGCGGCCTGGAGGTGATCTGGAAGGTAAATGACGCTG
TCACCACCGACGGCGTCCAGACCACCAGGTCCTCGAAACAGAGCAACGGCAAGTACGCGGCCAGCAGCTA
CCTGACGCGGACTTCCGCACAGTGGAAATCGTACAGCAGCGTCAGCTGCCAGGTCAAGCACCAAGGGAAA
ACC
>KF985088.1 Equus caballus clone NeonateT23 immmunoglobulin lambda light chain variable region (IGL) mRNA, partial cds
CTGCTTCAGCTGTGGGGCCACAGAAGGCGGGACTCGGTGAAGATCTCCACCATGGCCTGGTCCCCTCTCC
TCCTCACCCTCATCGCTCTCTGCACAGGATCCTGGGCCCAGTCTCTGACCCAGCCCGCCTCAGTGTCTGG
GACCCTGGGCCAGACAGTCACCATCTCCTGCTCTGGAAGCAGCTCCAACATCGGGTATAGTTATAGTGCT
GTGGGCTGGTACCAACAGATCCCAGGAACAGCCCCCAAAACCCTCATCTATGGTAATAACAAACGAGCCT
CAGGGGTCCCAGATCGATTCTCTGGCTCCAAGTCTGGCAACACAGCCACCCTGACCATCTCTGGGCTTCA
GGCTGAGGACGAGGCCGATTATTACTGTGGTTCCTATTACAGCAGTGATAGTAGTGCTGTATTCGGCGGA
GGCACCCACCTGACCATCGCAGGTGGTCCCACGTCTACACCCTCGGTCTCTCTCTTCCCGCCCTCCTCTG
AGGAGCTCAGCGCCAACAAGGCCACAGTGGTGTGTCTCATCAGCGACTTCTCCCCCAGCGGCCTGGAGGT
GATCTGGAAGGTAAATGACGCTGTCACCACCGACGGCGTCCAGACCACCAGGTCCTCGAAACAGAGCAAC
GGCAAGTACGCGGCCAGCAGCTACCTGACGCGGACTTCCGCACAGTGGAAATCGTACAGCAGCGTCAGCT
GCCAGGTCAAGCACCAAGGGAAAACC
>KF985087.1 Equus caballus clone NeonateT05 immmunoglobulin lambda light chain variable region (IGL) mRNA, partial cds
AGCTGTGGGGCCACAGAAGGCAGGACTCGGTGAAGATCTCCACCATGGCCTGGTCCCCTCTCCTCCTCAC
CCTCATCGCTCTCTGCACAGGATCCTGGGCCCAGTCTCTGACTCAGCCCGCCTCAGTGTCTGGGACCCTG
GGCCAGACAGTCACCATCTCCTGCTCTGGAAGCAGCTCCAACATCGGGTATAGTTATAGTGCTGTGGGCT
GGTACCAACAGATCCCAGGGACAGCCCCCAAAACCCTCATCTATGGTAATAACAAACGAGCCTCAGGGGT
CCCAGATCGATTCTCTGGCTCCAAGTCTGGCAACACAGCCACCCTGACCATCTCTGGGCTTCAGGCTGAG
GACGAGGCCGATTATTACTGTGGTTCCTCATACAGCAGTGATAGTGCTGTATTCGGCGGAGGCACCCACC
TGACCATCGCAGGTGGTCCCACGTCTACACCCTCGGTCTCTCTCTTCCCGCCCTCCTCTGAGGAGCTCAG
CGCCAACAAGGCCACAGTGGTGTGTCTCATCAGCGACTTCTCCCCCAGCGGCCTGGAGGTGATCTGGAAG
GTAAATGACGCTGTCACCACCGACGGCGTCCAGACCACCAGGTCCTCGAAACAGAGCAACGGCAAGTACG
CGGCCAGCAGCTACCTGACGCGGACTTCCGCACAGTGGAAATCGTACAGCAGCGTCAGCTGCCAGGTCAA
GCACCAAGGGAAAACC
>KF985077.1 Equus caballus clone NeonateT16 immmunoglobulin lambda light chain variable region (IGL) mRNA, partial cds
GGGCTCAGAGGCAGAGCTCTGGGGCATCTCCACCATGGCCTGGACCCCTCTCCTGCTCCCCTTCCTCACT
CTCTGTATAGGTTCTGTGGTCTCCTTGGAGCTGACTCAGCCAGCTTCAGTGTCTGTGGCCTTAGGACAGA
CTGCCACAATCACCTGCCAGGGAGGAATCTTTGACAAAGAGTATGTGACATGGTACCAACAGAAGCCTGG
CGGGCCCCCTGTGACAGTGATTTATGGGGATAGTGAACGGCCCTCCGGGATCCCTGAACGATTCTCTGGC
TCCAGCTCAGGAGACACAGCCACGCTGACCATCAGCGGGGCCCAGGCTGAGGACGAGGCTGACTATTACT
GTCTGGCAGCAGCAGCAGATGCTTCTGATTATATCTTCGGCGGCGGGACCCACCTCAGCGTCCTGGGTGG
TCCCACGTCTGCACCCTCGGTCTCTCTCTTCCCGCCCTCCTCTGAGGAGCTCAGCGCCAACAAGGCCACA
GTGGTGTGTCTCATCAGTGACTTCTCCCCCAGCGACTTGACGGTGAGCTGGAAGGTAAATGGCGCCGCCA
TCAGCCAGGGAGTCCAGACCACCAAGCCCTCGAAACAGAGCAATGGCAAGTACGCGGCTAGCAGCTACCT
GACGCTGACCCCCGCCCAGTGGAAATCGTCCAGCAGCGTCAGCTGCCAGGTCACGCACCAAGGGAAAACC
>KF985061.1 Equus caballus clone FetusB07 immmunoglobulin lambda light chain variable region (IGL) mRNA, partial cds
GCTTCAGCTGTGGGGCCACAGAAGGCAGGACTCGGTGACAATCTCCACCATGGCCTGGTGCCCTCTCCTC

CTCACCCTCATCGCTCTCTGCACAGGATCCTGGGCCCAGTCTGTGACTCAGCCCGCCTCAGTGTCTGGGA
CCCTGGGCCAGACAGTCACCATCACCTGCACTGGAAGCAGCTCCAACATAGTTGCTTATGTGGGCTGGTA
CCAACAGATCCCAGGAACAGCCCCCAAAACCCTCATCTACGCTAATAACAAACGAGCCTCAGGGGTCCCA
GATCGATTCTCTGGCTCCAAGTCTGGCAGCACAGCCACCCTGACCATCACTGGGCTCCAGGCTGAGGACG
AGGCCGATTATTACTGTGGTACCTCTAGCAGCAGTGGTAGTGCTGTATTCGGCGGAGGCACCCACCTGAC
CATCGCAGGTGGTCCCCTGTCTCCACCCTCGGTCTCTCTCTTCCCGCCCTCCTCTGAGGAGCTCAGCGCC
AACAAGGCCACAGTGGTGTGTCTCATCAGTGACTTCTCCCCCAGCGGCTTGGAGGTGATCTGGAAGGTAA
ATGACGCTGTCACCAACGACCGCGTCCAGACCACCAGGCCCTCGAAACAGAGCAACGGCAAGTACGCGGC
CAGCAGCTACCTGACACGGACCTCCACAGAGTGGAAATCGTACAGCAGCGTCAGCTGCCAGGTCACGCAC
CAAGGGAAAACC
>KF985042.1 Equus caballus clone FetusC04 immmunoglobulin lambda light chain
variable region (IGL) mRNA, partial cds
CTTCAGCTGTGGGGCCACAGAAGGCAGGACTCGGTGAAGATCTCCACCATGGCCTGGTCCCCTCTCCTCC
TCACCCTCATCGCTCTCTGCACAGGATCCTGGGCCCAGTCTCTGACTCAGCCCGCCTCAGTGTCTGGGAC
CCTGGGCCAGACAGTCACCATCTCCTGCTCTGGAAGCAGCTCCAACATCGGGTATAGTTATAGTGCTGTG
GGCTGGTACCAACAGATCCCAGGGACAGCCCCCAAAACCCTCATCTATGCTACTAACAAACGAGCCTCAG
GGGTCCCAGATCGATTCTCTGGCTCCAAGTCTGGCAACACAGCCACCCTGACCATCTCTGGGCTTCAGGC
TGAGGACGAGGCCGATTATTACTGTGGTTCCTCATACAGCAGTGATAGTATCTTCGGCGGCGGGACCCAC
CTCAGCGTCCTGGGTGGTCCCCCGTCTGCACCCTCGGTCTCTCTCTTCCCGCCCTCCTCTGAGGAGCTCA
GCACCAACAAGGCCACAGTGGTGTGTCTCATCAGTGACTTCTCCCCCAGCGACTTGACGGTGAGCTGGAA
GGGAAATGGCGCCGCCATCAGCCAGGGCGTCCAGACCACCAAGCCCTCGAAACAGAGCAATGGCAAGTAC
GCGGCTAGCAGCTACCTGACGCTGACCCCCGCCCAGTGGAAATCGTACAGCAGCGTCAGCTGCCAGGTCA
CGCACCAAGGGAAAACC
>KF748671.1 Equus caballus clone IGLVJ60 immunoglobulin lambda light chain
variable region (IGL) mRNA, partial cds
ATGGCCTGGTCCCCTCTCCTCCTCACCCTCATCGCTCTCTGCACAGGATCCTGGGCCCAGTCTGTCACTC
AGCCCGCCTCAGTGTCTGGGACCCTGGGCCAGACAGTCACCATCACCTGCACTGGAAGCGGCGCGACCCT
TCCGACCGTTGTGGCTTACGTGGCCTGGTATAAACAGGTCCCAGGAACTCCCCCCAAAACCCTCATCTAT
GATAAAAACAAGCGAGCCGCAGGAGTCCCAGATCGATTCTCTGGCTCCGTGTCTGGCAACACAGCCACCC
TGACCATCTCTGGGGTCCAGGCTGAGGACGAGGCCGATTATTATTGTAGTTCCTATGACGCAGACAGTTT
TCTATTCGGCGGAGGCACCCACCTGACCGTCG
>KF748649.1 Equus caballus clone IGLVJ38 immunoglobulin lambda light chain
variable region (IGL) mRNA, partial cds
ATGGCCTGGTCCCCTCTCCTCCTCACCCTCATCGCTCTCTGCACAGGATCCTGGGCCCAGTCTCTGACTC
AGCCCGCCTCAGTGTCTGGGACCCTGGGCCAGACAGTCACCATCTCCTGCACTGGAAGCAGCTCCAGCAT
AGGTTCTTATATGGGCTGGTACCAACAGATCCCAGGGACAGCCCCCAAAACCCTCATCTATGCTACTAAC
AAACGAGCCTCAGGGGTCCCAGATCGATTCTCTGGCTCCAAGTCTGGCAACACAGCCACCCTGACCATCA
CTGGGCTTCAGGCTGAGGACGAGGCCGATTATTACTGTGGTTCCTATTACAGCAGTGATGCTGTATTCGG
CGGAGGCACCCACCTGACCATCGCAG
>KF748643.1 Equus caballus clone IGLVJ32 immunoglobulin lambda light chain
variable region (IGL) mRNA, partial cds
ATGGCCTGGTCCCCTCTCCTCCTCACCCTCATCGCTCTCTGCACAGGATCCTGGGCCCAGTCTCTGACCC
AGCCCGCCTCAGTGTCTGGGACCCTGGGCCAGACAGTCACCATCTCCTGCTCTGGAAGCAGCTCCAACAT
CGGGTATAGTTATAGTGCTGTGGGCTGGTACCAACAGATCCCAGGAACAGCCCCCAAAACCCTCATCTAT
GGTAATAACAAACGAGCCTCAGGGGTCCCAGATCGATTCTCTGGCTCCAAGTCTGGCAACACAGCCACCC
TGACCATCTCTGGGCTTCAGGCTGAGGACGAGGCCGATTATTACTGTGGTTCCTATTACAGCAGTGATGG
TGCATTCGGCGGAGGCACCCACCTGACCATCGCAG
>KF748640.1 Equus caballus clone IGLVJ29 immunoglobulin lambda light chain
variable region (IGL) mRNA, partial cds
ATGGCCTGGTCCCCTCTCCTCCTCACCCTCATCGCTCTCTGCACAGGATCCTGGGCCCAGTCTGTGACTC
AGCCCGCCTCAGTGTCTGGGACCCTGGGCCAGACAGTCACCATCTCCTGCACTGGAAGCATCTCCAACAT
AGGTGTTTATGTGGACTGGTACCAACAGATCCCAGGAACAGCCCCCAAAACCATCATCTATGCTACTAAC
AAACAACCCTCAGGGGTCCCAGATCGATTCTCTGGCTCCAAGTCTGGCAACACAGCCACCCTGACCATCA
CTGGGCTCCAGGCTGAGGACGAGGCTGATTATTACTGTGGTATCTATGACAGCAGCCTGAGTAGTGCATT
CGGCGGAGGCACCCACCTGACCATCGCAG

>KF985160.1 Equus caballus clone AdultB05 immmunoglobulin lambda light chain variable region (IGL) mRNA, partial cds
AGCTCTGCTTCAGCTGTGGGGCCACAGAAGGCAGGACTCGGTGAAGATCTCCACCATGGCCTGGTCCCCT
CTCCTCCTCACCCTCATCGCTCTCTGCACAGGATCCTGGGCCCAGTCTCTGACTCAGCCCGCCTCAGTGT
CTGGGACCCTGGGCCAGACAGTCACCATCTCCTGCTCTGGAAGCAGCTCCAACATCGGACCTAATTCTGT
GGGCTGGTACCAACAGGTCCCAGGAAAAGCCCTCAAAACCCTCATCACCGATACTGTCATACGTTCCTCA
GGGGTCCCAGATCGATTCTCTGCCTCCAGGTCTGGCAACACAGCCACCCTGACCATCTCTGGGGTCCAGG
ATGAGGACGAGGCCGATTACTACTGCTCAGCGGGAGACATCAAGCTGAGAAGTACTGTGTTCGGCGGAGG
AACCCACCTGACCATCGCTGGTGGTCCCACGTCTACACCCTCGGTCTCTCTCTTCCCGCCCTCCTCTGAG
GAGCTCAGCGCCAACAAGGCCACAGTGGTGTGTCTCATCAGCGACTTCTCCCCCAGCGGCCTGGAGGTGA
TCTGGAAGGTAAATGACGCTGTCACCACCGACGGCGTCCAGACCACCAGGTCCTCGAAACAGAGCAACGG
CAAGTACGCGGCCAGCAGCTACCTGACGCGGACTTCCGCACAGTGGAAATCGTACAGCAGCGTCAGCTGC
CAGGTCAAGCACCAAGGGAAAACC
>KF985159.1 Equus caballus clone AdultC04 immmunoglobulin lambda light chain variable region (IGL) mRNA, partial cds
TTGGGGTCTCAGAAGGCAGTGCTGTTGGGGAATCTCCACCATGACCTGGGCTCTGCTCCTCATCACCCTC
TTCACTCAGGGCACAGGGTCCTGGGCGCAGTCTGCCCTGACTCAGCCTGCGTCAGTGTCCGGGACTCTGG
GACAGTCGGTCACCATCTCCTGTACTGGAAGCGGCAGCGACATTGGAAGTTATAACTTTGTTTCCTGGTA
CCAACAACGTCCGGGCACAGCCCCCAAACTCCTCATTAGTGCTGTCAGTCGTCGGGCTTCTGGGATCCCT
GATCGCTTCTCTGGCTCCAAGTCTGGGAACACGGCCTCTCTGACCATCTCGGGGCTCCAGGCTGAGGACG
AGGCCGATTATTACTGTACCTCAGCTGTCAGCTATGAACCTTATTTTGAGTTCGGCGGGGGCACCCACCT
GACCGTCGCAGGTGGTCCCACGTCTACACCCTCGGTCTCTCTCTTCCCGCCCTCCTCTGAGGAGCTCAGC
GCCAACAAGGCCACAGTGGTGTGTCTCATCAGTGACTTCTCCCCCAGCGGCCTGGAGGTGATCTGGAAGG
TAAATGACGCTGTCACCACCGACGGCGTCCAGACCACCAGGTCCTCGAAACAGAGCAACGGCAAGTACGC
GGCCAGCAGCTACCTGACGCGGACTTCCGCACAGTGGAAATCGTACAGCAGCGTCAGCTGCCAGGTCAAG
CACCAAGGGAAAACC
>KF985158.1 Equus caballus clone AdultC09 immmunoglobulin lambda light chain variable region (IGL) mRNA, partial cds
AGGGTTGGGGTCTCAGAAGGCAGTGCTCTTGGGGCGTCTCCACCATGGCCTGGACTCTGCTCCCTCTCAC
CCTCCTCATTCAGGGTACAGGGTCCTGGGCCCAGTCTGCCCTGACTCAGCCTGCGTCAGTGTCCGGGGCT
CTAGGACAGTCGGTCACAATCACCTGTACTGGAAGCAGCAGTGACGTCGGGGCATATAGTGCTGTCAGTT
GGTTGCAACAACACCCGGGCACAGCCCCCAAAGTTCTGATTTATAGTGTGAATGCTCGGGCCTCAGGAAT
CCCTGATCGCTTCTCTGGCAGCAAGTCTGGCAACACGGCCTCCCTGACCATATCTGGACTTCAGGTTGAG
GACGAGGCTATTTATTACTGCTATTCACTTGCGGCGGATTGGACTGGTGCATTCGGCGGAGGCACCCACC
TGACCATCGCAGGTGGTCCCACGTCTACACCCTCGGTCTCTCTCTTCCCGCCCTCCTCTGAGGAGCTCAG
CGCCAACAAGGCCACAGTGGTGTGTCTCATCAGTGACTTCTCCCCCAGCGGCTTGGAGGTGATCTGGAAG
GTAAATGACGCTGTCACCACCGACGGCGTCCAGACCACCAGGTCCTCGAAACAGAGCAACGGCAAGTACG
CGGCCAGCAGCTACCTGACGCGGACTTCCGCACAGTGGAAATCGTACAGCAGCGTCAGCTGCCAGGTCAA
GCACCAAGGGAAAACC
>KF985157.1 Equus caballus clone AdultA09 immmunoglobulin lambda light chain variable region (IGL) mRNA, partial cds
GAAGCTCTGCTTCAGCTGTGGGGCCACAGAAGGCAGGACTCGGTGAAGATCTCCACCATGGCCTGGTCCC
CTCTCCTCCTCACCCTCATCGCTCTCTGCACAGGATCCTGGGCCCAGTCTCTGACTCAGCCCGCCTCAGT
GTCTGGGACCCTGGGCCAGACAGTCACCATCTCCTGCACTGGAAGCAGCGATAGCAGAGATCATAATATG
GGCTGGTACCAACAGATCCCAGGGACAGCCCCCAAAACCCTCATCTACGCTAGTAACAAACGAGCCTCAG
GGGTCCCAGATCGATTCTCTGGCTCCAAGTCTGGCAACACAGCCACCCTGACCATCTCTGGGCTTCAGGC
TGAGGACGAGGCCGATTATTACTGTGGTTCCTATTACAGCAGTAATGAAGGTACATTCGGCGGGGGCACC
CACCTGACCATCGCAGGTGGTCCCACGTCTACACCCTCGGTCTCTCTCTTCCCGCCCTCCTCTGAGGAGC
TCAGCGCCAACAAGGCCACAGTGGTGTGTCTCATCAGTGACTTCTCCCCCAGCGGCTTGGAGGTGATCTG
GAAGGTAAATGACGCTGTCACCACCGACGGCGTCCAGACCACCAGGTCCTCGAAACAGAGCAACGGCAAG
TACGCGGCCAGCAGCTACCTGACGCGGACTTCCGCACAGTGGAAATCGTACAGCAGCGTCAGCTGCCAGG
TCAAGCACCAAGGGAAAACC
>KF985156.1 Equus caballus clone AdultA05 immmunoglobulin lambda light chain variable region (IGL) mRNA, partial cds
AGCTCTGCTTCAGCTGTGGGGCCACAGAAGGCAGGACTCGGTGAAGATCTCCACCATGGCCTGGTCCCCT

CTCCTCCTCACCCTCATCGCTCTCTGCACAGGATCCTGGGCCCAGTCTGTGACCCAGCCCGCCTCAGTGT
CTGGGACCCTGGGCCAGACAGTCACCATCTCCTGCTCTGGAAGCAGCTCCAACATCGGGTATAGTAGTAA
ATATGGGGGCTGGTACCAACAGATCCCAGGAACAGCCCCCAAACTCCTCATATATGAGGGTAACAAACGA
GCCTCAGGGGTCTCGGGTCGTTTCTCTGGCTCCAAGTCTGGCAACACAGTCACCCTGACCATCTCTGGGC
TCCAGGCTGAGGACGAGGCCGCGTATTACTGTGTTTCCTGGGACAGCAGCCTGACCAGCGTTGTGTTCGG
CGGAGGCACCCACCTGACCATCGCAGGTGGTCCCACGTCTACACCCTCGGTCTCTCTCTTCCCGCCCTCC
TCTGAGGAGCTCAGCGCCAACAAGGCCACAGTGGTGTGTCTCATCAGTGACTTCTCCCCCAGCGGCTTGG
AGGTGATCTGGAAGGTAAATGACGCTGTCACCACCGACGGCGTCCAGACCACCAGGTCCTCGAAACAGAG
CAACGGCAAGTACGCGGCCAGCAGCTACCTGACGCGGACTTCCGCACAGTGGAAATCGTACAGCAGCGTC
AGCTGCCAGGTCAAGCACCAAGGGAAAACC
>KF985155.1 Equus caballus clone AdultD06 immmunoglobulin lambda light chain variable region (IGL) mRNA, partial cds
CTGCTTCAGCTGTGGGGCCACAGAAGGCAGGACTCGGTGAAGATCTCCACCATGGCCTGGTCCCCGCTCC
TTCTCACCTTCATCGCTCTCTGCACAGGATCCTGGGCCCAGTCTCTGACTCAGCCCGCCTCAGTGTCTGG
GACCCTGGGCCAGACAGTCACCATCTCCTGCTCTGGAAGCAGCTCCAACATTGGAGGTAGTACCAGTTGG
GTGAACTGGTTCCAGCAGATCCCAGGAACAGCCCCCAAAACTCTCATCTATTATGCCACTAGTAGAGCGT
CCGGGGTCCCCGATCGATTCTCTGGCTCCAGGTCTGGCAACACAGCCACCCTGACCATCTCTGGGGTCCA
GGCTGAGGACGAGGCTGATTATTACTGCTCAGCAGTGGACAGCAGCCTGAGGAGTACTGTATTCGGCGGA
GGCACCCACCTGACCATCGCAGGTGGTCCCACGTCTACACCCTCGGTCTCTCTCTTCCCGCCCTCCTCTG
AGGAGCTCAGCGCCAACAAGGCCACAGTGGTGTGTCTCATCAGCGACTTCTCCCCCAGCGGCCTGGAGGT
GATCTGGAAGGTAAATGACGCTGTCACCACCGACGGCGTCCAGACCACCAGGTCCTCGAAACAGAGCAAC
GGCAAGTACGCGGCCAGCAGCTACCTGACGCGGACTTCCGCACAGTGGAAATCGTACAGCAGCGTCAGCT
GCCAGGTCAAGCACCAAGGGAAAACC
>KF985154.1 Equus caballus clone AdultC05 immmunoglobulin lambda light chain variable region (IGL) mRNA, partial cds
AAGCTCTGCTTCAGCTGTGGGGCCACAGAAGGCAGGACTCGGTGAAGATCTCCACCATGGCCTGGTCCCC
TCTCCTCCTCACCCTCATCGCTCTCTGCACAGGATCCTGGGCCCAGTCTGTGACTCAGCCCGCCTCTGTG
TCTGGGACCCTGGGCCAGACAGTCACCATCTCCTGCTCTGGAAGTAGTTCCAACATCGGGTTGAGTGGTA
GTTATGTGGGCTGGTTCCAACAGAACCCAGGAACAGCCCCCAAAACCCTCATCCATGATGTTAACGTGAG
AGTTTCAGGGGTCCCGGATCGCTTCTCCGGCTCCAAGTCTGGCAGCACAGCCACCCTGACCATCACTGGG
CTCCAGCCTGAGGACGAGGCCGATTATTACTGTGCAGCAGGAGACAACAAGATGAGGAACGCTGTATTCG
GCGGAGGCACCCAGCTGACCATCGCAGGTGGTCCCACGTCTACACCCTCGGTCTCTCTCTTCCCGCCCTC
CTCTGAGGAGCTCAGCGCCAACAAGGCCACAGTGGTGTGTCTCATCAGCGACTTCTCCCCCAGCGGCCTG
GAGGTGATCTGGAAGGTAAATGACGCTGTCACCACCGACTGCGTCCAGACCACCAGGTCCTCGAAACAGA
GCAACGGCAAGTACGCGGCCAGCAGCTACCTGACGCGGACTTCCGCACAGTGGAAATCGTACAGCAGCGT
CAGCTGCCAGGTCAAGCACCAAGGGAAAACC
>KF985153.1 Equus caballus clone AdultB01 immmunoglobulin lambda light chain variable region (IGL) mRNA, partial cds
AAGCTCTTCTTCAGCTCTGGGGCCACTGAAGGCAGGACTTGGTGACAATCTCCACCATGGCCTGGTCCCC
TCTCCTCCTCACCCTCATCGCTCTCTGCACAGGATCCTGGGCCCAGTCTTTGACCCAGCCCGCCTCAGTG
TCTGGGACCCTGGGCCAGACAGTCGCCATCTCCTGTACTGGAAGTAGCTCCAACATCGGTAACCCTTTTG
CTTATGTGGGCTGGTTCCAACACAACCCAGGAACCAACCCTAAAACCCTCATCCATGATACTGTCAAACG
GGGCTCAGGGGTCCCAGATCGATTCTCTGCCTCTAAGTCTGGCAACACAGCCACCCTGACCATCTCTGGG
GTCCAGGCTGAGGACGAGGGCGATTATTATTGCTCATCGGGAGACATCGATACTACTACTATGTTCGGCG
GAGGCACCCACCTGACCATCGAGGGTGGTCCCACGTCTACACCCTCGGTCTCTCTCTTCCCGCCCTCCTC
TGAGGAGCTCAGCGCCAACAAGGCCACAGTGGTGTGTCTCATCAGTGACTTCTCCCCCAGCGGCCTGGAG
GTGATCTGGAAGGTAAATGACGCTGTCACCACCGACGGCGTCCAGACCACCAGGTCCTCGAAACAGAGCA
ACGGCAAGTACGCGGCCAGCAGCTACCTGACGCGGACTTCCGCACAGTGGAAATCGTCCAGCAGCGTCAG
CTGCCAGGTCACGCACCAAGGGAAAACC
>KF985152.1 Equus caballus clone AdultC06 immmunoglobulin lambda light chain variable region (IGL) mRNA, partial cds
AAGCTCTGCTTCAGCTGTGGGGCCACAGAAGGCAGGACTCGGTGAAGATCTCCACCATGGCCTGGTCCCC
TCTCCTCCTCACCCTCATCGCTCTCTGCACAGGATCCTGGGCCCAGTCTGTGACCCAGCCCGCCTCAGTG
TCTGGGACCCTGGGCCAGACAGTCACCATCTCCTGCTCTGGAAGCGGCACCAATGTCGGTAGTGTGTATG
GTTTTGTGGGATGGTACCAACAGGTCCCAGGAGCAGCCCCCAAAACCCTGATCTATGATGATACAAAACG
AGCCTCAGGGGTCCCAGATCGATTCTCTGGTTCCAAGTCTGGCGACACAGCCACCCTGACCATTTCTGGC

GTGCAGGCTGAGGACGAGGCCGTATATTACTGTGGCTCCTATGACAGTACTGATAACGAAAGATTCGGCG
GAGGCACCCACCTGACCATAGTAGGTGGTCCCACGTCTACACCCTCGGTCTCTCTCTTCCCGCCCTCCTC
TGAGGAGCTCAGCGCCAACAAGGCCACAGTGGTGTGTCTCATCAGTGACTTCTCCCCCAGCGGCTTGGAG
GTGATCTGGAAGGTAAATGACGCTGTCACCACCGACGGCGTCCAGACCACCAGGTCCTCGAAACAGAGCA
ACGGCAAGTACGCGGCCAGCAGCTACCTGACGCGGACTTCCGCACAGTGGAAATCGTACAGCAGCGTCAG
CTGCCAGGTCAAGCACCAAGGGAAAACC
>KF985151.1 Equus caballus clone AdultD05 immmunoglobulin lambda light chain variable region (IGL) mRNA, partial cds
AGCTCTGCTTCAGCTGTGGGGCCACAGAAGGCAGGACTCGGTGACGATCTCCACCATGGCCTGGTGCCCT
CTCCTCCTCACCCTCATCGCTCTCTGCACAGGATCCTGGGCCCAGTCTGTGACTCAGCCCGCCTCAGTGT
CTGGGACCCTGGGCCAGACAGTCACCATCACCTGCACTGGAAGCAGCTCCAACATGTTTACTGGTGTGTG
CTGGTACCAACAGATCCCAGGAACAGCCCCCAAAACCCTCATCTATGGCGATGATAAGCTATTCTCAGGG
GTCCCAGATCGATTCTCTGGCTCCAAGTCTGGCACCACAGCCACCCTGACCATCACTGGGGTCCAGCCTG
AGGACGAGGCCGATTATTACTGTGCTGCCTGGAGCAGGACAGATGGTGTTAGATTCGGCGGAGGCACCCA
CCTGACCATCGCAGGTGGTCCCACGTCTACACCCTCGGTCTCTCTCTTCCCGCCCTCCTCTGAGGAGCTC
AGCGCCAACAAGGCCACAGTGGTGTGTCTCATCAGTGACTTCTCCCCCAGCGGCTTGGAGGTGATCTGGA
AGGTAAATGACGCTGTCACCACCGACGGCGTCCAGACCACCAGGTCCTCGAAACAGAGCAACGGCAAGTA
CGCGGCCAGCAGCTACCTGACGCGGACTTCCGCACAGTGGAAATCGTACAGCAGCGTCAGCTGCCAGGTC
AAGCACCAAGGGAAAACC
>KF985150.1 Equus caballus clone AdultD04 immmunoglobulin lambda light chain variable region (IGL) mRNA, partial cds
ACAGAAGGCAGGACTCGGTGAAGATCTCCACCATGGCCTGGTCCCCTCTCCTCCTCACCCTCATCGCTCT
CTGCACAGGATCCTGGGCCCAGTCTCTGACCCAGCCCGCCTCAGTGTCTGGGACCCTGGGCCAGACAGTC
ACCATCACCTGCACTCTGAGCAGCGCCGAAGCGAATGTTTATGCGGCCTGGTACCAACAGAGACCAGGAA
CAGGACCCAAAACCCTCTTCTATAATAATGTCGTGCGAGTCTCAGGGGTCCCAGATCGATTCTCGGCCTC
CAAGTCTGGCAGTACAGCCACCCTGACCATCACTGGGCTCCAGGGTGAGGACGAGGCCGATTATTACTGT
GGCACCACTAGCGTTAGTAACCTTAGTTTTGGGTTCGGCGGAGGCACCCACCTGACCATCGCAGGTGGTC
CCACGTCTACACCCTCGGTCTCTCTCTTCCCGCCCTCCTCTGAGGAGCTCAGCGCCAACAAGGCCACAGT
GGTGTGTCTCATCAGCGACTTCTCCCCCAGCGGCCTGGAGGTGATCTGGAAGGTAAATGACGCTGTCACC
ACCGACGGCGTCCAGACCACCAGGTCCTCGAAACAGAGCAACGGCAAGTACGCGGCCAGCAGCTACCTGA
CGCGGACTTCCGCACAGTGGAAATCGTACAGCAGCGTCAGCTGCCAGGTCAAGCACCAAGGGAAAACC
>KF985149.1 Equus caballus clone AdultC11 immmunoglobulin lambda light chain variable region (IGL) mRNA, partial cds
GCTTCAGCTGTGGGGCCACAGAAGGCAGGACTCGGTGACGATCTCCACCATGGCCTGGTGCCCTCTCCTC
CTCACCCTCATCGCTCTCTGCACAGGATCCTGGGCCCAGTCTGTGACTCAGCCCGCCTCAGTGTCTGGGA
CCCTGGGCCAGACAGTCACCATCACCTGCACTGGAAGAACGTCCGAATTAATTGCCTATGTGGGCTGGTA
CCAGCAGAAACCAGGCACAGCCCCCAAAACCCTCATCTATGACACCAACAAACGAACCTCAGGGGTCCCA
GATCGATTCTCCGGCTCCAAGTCTGGCAGCACAGCCACCCTGACCATCACTGGGCTCCAGGCTGAGGACG
AGGCCGATTATTACTGTGGTACTCATAGTAGTGGTGTTAGTGAGACATTCGGCGGAGGCACCCACCTGAC
CATTGTGGGTGGTCCCACGTCTACACCCTCGGTCTCTCTCTTCCCGCCCTCCTCTGAGGAGCTCAGCGCC
AACAAGGCCACAGTGGTGTGTCTCATCAGTGACTTCTCCCCCAGCGGCTTGGAGGTGATCTGGAAGGTAA
ATGACGCTGTCACCACCGACGGCGTCCAGACCACCAGGTCCTCGAAACAGAGCAACGGCAAGTACGCGGC
CAGCAGCTACCTGACGCGGACTTCCGCACAGTGGAAATCGTACAGCAGCGTCAGCTGCCAGGTCAAGCAC
CAAGGGAAAACC
>KF985148.1 Equus caballus clone AdultB07 immmunoglobulin lambda light chain variable region (IGL) mRNA, partial cds
AGCTCTGCTTCAGCTGTGGGGCCACAGAAGGCAGGACTCGGTGACGATCTCCACCATGGCCTGGTGCCCT
CTCCTCCTCACCCTCATCGCTCTCTGCACAGGATCCTGGGCCCAGTCTGTGACTCAGCCCGCCGAAGTGT
CTGGGACCCTGGGCCAGACAGTCGAGATCACCTGCACTGGGAGCAGCTCCGACGTGATACAGTCCGTGGG
CTGGTATCAACAAGTCCCAGGCGCAGCCCCCAAAACCCTCATTCATGGCATTAACGCACGACCCTCAGGG
GTCTCAGATCGATTCTCTGGCTCCAAGTCTGGCACTACAGCCACCCTGACCATTTTTTCGCTCCAGTATG
AGGACGAGGCCGATTATTACTGTGGTGTCACCGGCAGTAGTCACAGTCAAGCGTTCGGCGGAGGCACCCG
AGTGACCATCGTAGGTGGTCCCACGTCTACACCCTCGGTCTCTCTCTTCCCGCCCTCCTCTGAGGAGCTC
AGCGCCAACAAGGCCACAGTGGTGTGTCTCATCAGTGACTTCTCCCCCAGCGGCTTGGAGGTGATCTGGA
AGGTAAATGACGCTGTCACCACCGACGGCGTCCAGACCACCAGGTCCTCGAAACAGAGCAACGGCAAGTA

CGCGGCCAGCAGCTACCTGACGCGGACTTCCGCACAGTGGAAATCGTACAGCAGCGTCAGCTGCCAGGTC
AAGCACCAAGGGAAAACC
>KF985147.1 Equus caballus clone AdultB04 immmunoglobulin lambda light chain variable region (IGL) mRNA, partial cds
AGCTGTGGGGCCACAGAAGGCAGGACTCGGTGAAGATCTCCACCATGGCCTGGTGCCCTCTCCTCCTCAC
CCTCATCGCTCTCTGCACAGGATCCTGGGCCCAGTCTGTGACTCAGCCCGCCTCAGTGTCTGGGACCCTG
GGCCAGACAGTCACCATCACCTGTACTGGAAATATTATCAGAACGGCCTGGTACCGACAGATCCCAGGAA
CAGCCCCCAAATCCCTCATTTATGATATCTCCAAAAGACACTCAGGGGTCCCAGATCGCATCTCTGGCTC
CAGGTCTGGCAAGACAGCCACCCTGACCATCAGTGGGCTCCAGGCTGAGGACGAGGCCGTATATTACTGT
GGTACGGCTGACAGTACTAATGGTCGTCTCTATGTATTCGGCTCAGGCACCCACCTGACCATCGCGGGTG
GTCCCACGTCTACACCCTCGGTCTCTCTCTTCCCGCCCTCCTCTGAGGAGCTCAGCGCCAACAAGGCCAC
AGTGGTGTGTCTCATCAGTGACTTCTCCCCCAGCGGCTTGGAGGTGATCTGGAAGGTAAATGACGCTGTC
ACCACCGACGGCGTCCAGACCACCAGGTCCTCGAAACAGAGCAACGGCAAGTACGCGGCCAGCAGCTACC
TGACGCGGACTTCCGCACAGTGGAAATCGTACAGCAGCGTCAGCTGCCAGGTCAAGCACCAAGGGAAAAC
C
>KF985146.1 Equus caballus clone AdultA11 immmunoglobulin lambda light chain variable region (IGL) mRNA, partial cds
AAGCTCTGCTTCAGCTGTGGGGCCACAGAAGGCAGGACTCGGTGACGATCTCCACCATGGCCTGGTGCCC
TCTCCTCCTCACCCTCATCGCTCTCTGCACAGGATCCTGGGCCCAGTCTGTGACTCAGCCCGCCTCAGTG
TCTGGGACCCTGGGCCAGACAGTCACCATCACATGTACTGGAAGCGACTCCGACAGTGTTCGGAGTGTGG
GCTGGTACCAACAGATCCCAGGAACAGCCCCCAAAACCCTCATCTATGGCAGCACGAAACGTCCCTCAGG
GGTCCCAGATCGGTTCTCTGGCTCCAAGTCTGGTAGCAAAGCCACCCTGACCATCACTGGGCTCCAGGCG
GAGGACGAGGCCGATTATTATTGTGGGACAGTTAACATCAGGGGTAGTGGTGCGTTCGGCGGAGGCACCC
ACCTGACCATCGCAGGTGGTCCCACGTCTACACCCTCGGTCTCTCTCTTCCCGCCCTCCTCTGAGGAGCT
CAGCGCCAACAAGGCCACAGTGGTGTGTCTCATCAGTGACTTCTCCCCCAGCGGCTTGGAGGTGATCTGG
AAGGTAAATGACGCTGTCACCACCGACGGCGTCCAGACCACCAGGTCCTCGAAACAGAGCAACGGCAAGT
ACGCGGCCAGCAGCTACCTGACGCGGACTTCCGCACAGTGGAAATCGTACAGCAGCGTCAGCTGCCAGGT
CAAGCACCAAGGGAAAACC
>KF985145.1 Equus caballus clone AdultC02 immmunoglobulin lambda light chain variable region (IGL) mRNA, partial cds
CAGAAGGCAGGACTCGGTGAAGATCTCCACCATGGCCTGGTCCCCTCTCCTCCTCACCCTCATCGCTCTC
TGCACAGGATCCTGGGCCCAGTCTGTGACTCAGCCCGCCTCAGTGTCTGGGACCCTGGGCCAGACAGTCA
CCATCTCCTGCTCTGGAAGCAGCTCCAACATCGGACATACTTATAGTAAGGTGGGCTGGTTCCAACAGAT
CCCAGGAACAGCCCCCAAAACCCTCATCTATGGTGATAACAAACGAGCCTCAGGGGTCCCAGATCGATTC
TCTGGCTCCAAGTCTGGCAACACAGCCACCCTGACCATCTCTGGGGTCCAGGCTGAGGACGAGGCCGATT
ATTACTGTGGTTCCTATGACAGCAGTAGTAAGAGTGATCTTGCATTCGGCGGAGGCACCCACCTGACCAT
CGCAGGTGGTCCCACGTCTACACCCTCGGTCTCTCTCTTCCCGCCCTCCTCTGAGGAGCTCAGCGCCAAC
AAGGCCACAGTGGTGTGTCTCATCAGTGACTTCTCCCCCAGCGGCCTGGAGGTGATCTGGAAGGTAAATG
ACGCTGTCACCACCGACGGCGTCCAGACCACCAGGTCCTCGAAACAGAGCAACGGCAAGTACGCGGCCAG
CAGCTACCTGACGCGGACTTCCGCACAGTGGAAATCGTACAGCAGCGTCAGCTGCCAGGTCAAGCACCAA
GGGAAAACC
>KF985144.1 Equus caballus clone AdultC12 immmunoglobulin lambda light chain variable region (IGL) mRNA, partial cds
CTGCTTCAGCTGTGGGGCCACAGAAGGCAGGACTCGGTGAAGATCTCCACCATGGCCTGGTCCCCTCTCC
TCCTCACCCTCATCGCTCTCTACACAGGATCCTGGGCCCAGTCTCTGACTCAGCCCGCCTCAGTGTCTGG
GACCCTGGGCCAGACAGTCACCATCTCCTGCTCTGGAAGCCGCTCCAACGTCGGCGTTGATTATACTGTT
GTGGCCTGGTATCAACAGATCCCAGGAACAGCCCCCAAAACCCTCATCTACAGTGACAAACGAGCCTCAG
GGATCCCGGATCGATTCTCTGCATCCAAGTCTGGCAACACAGCCACCCTGACCATCTCTGGGGTCCAGTT
TGAGGACGAGGCCGATTATTACTGCAGTACTTACGACGAGAGTAGATGGACTGGTGTCTTCGGAGGAGGC
ACCCACCTGACCGTCGCAGGTGGTCCCACGTCTACACCCTCGGTCTCTCTCTTCCCGCCCTCCTCTGAGG
AGCTCAGCGCCAACAAGGCCACAGTGGTGTGTCTCATCAGTGACTTCTCCCCCAGCGGCTTGGAGGTGAT
CTGGAAGGTAAATGACGCTGTCACCACCGACGGCGTCCAGACCACCAGGTCCTCGAAACAGAGCAACGGC
AAGTACGCGGCCAGCAGCTACCTGACGCGGACTTCCGCACAGTGGAAATCGTACAGCAGCGTCAGCTGCC
AGGTCAAGCACCAAGGGAAAACC
>KF985143.1 Equus caballus clone AdultB11 immmunoglobulin lambda light chain variable region (IGL) mRNA, partial cds

AGCTCTGCTTCGGCTGTGGGGCCACAGAAGGCAGGACTCGGTGAAGATCTCCACCATGGCCTGGTCCCCT
CTCCTCCTCACCCTCATCGCTCTCTGCACAGGATCCTGGGCCCAATCTTTGACTCAGCCCGCCTCAGTGT
CTGGGACCCTGGGCCAGACAGTCACCATAGCTTGTTCTGGTGCCGGCTCCAACATCGGGTCCAGAGACTC
TTATGTGGGCTGGTTCAAGCAGGTCCCAGGCACAGCCCCCAAAACCATCATCTATTGGAACTCTAGACGG
GCCTCAGGGGTCCCTGATCGATTCTCTGGCCGTAAATCTGGTAACGTAGCCACCCTGACCATCTCTGGGG
TCCAGGCTGAGGACGAGGCTGATTATTACTGTTCATCGTCAGACGACAGCCTGAAGAGTGATGTATTCGG
CGGAGGCACCCACCTGACCATCGCAGGTGGTCCCACGTCTACACCCTCGGTCTCTCTCTTCCCGCCCTCC
TCTGAGGAGCTCAGCGCCGACAAGGCCACAGTGGTGTGTCTCATCAGCGACTTCTCCCCCAGCGGCCTGG
AGGTGATCTGGAAGGTAAATGACGCTGTCACCACCGACGGCGTCCAGACCACCAGGTCCTCGAAACAGAG
CAACGGCAAGTACGCGGCCAGCAGCTACCTGACGCGGACTTCCGCACAGTGGAAATCGTACAGCAGCGTC
AGCTGCCAGGTCAAGCACCAAGGGAAAACC
>KF985141.1 Equus caballus clone AdultA12 immmunoglobulin lambda light chain variable region (IGL) mRNA, partial cds
TCTCCACCATGGCCTGGTCCCCTCTCCTCCTCACCCTCATCGCTCTCTGCACAGGATCGTGGGCCCAGTC
TGTGACCCAGCCCGCCTCAGTGTCTGGGACCCTGGGCCAGACAGTCACCATCTCCTGTTCTGGGAGCAAA
TCCAACATCGGACATCGGACTAGTTATGTGGGATGGTTCCAACAGATCCCGGGAACAGCCCCCAGAACCC
TCATCTATGGCGGTAACGTACGAGCCTCAGGGGTTCCGGATCGATGGTCTGCCTCCAAAATTGGCAACAC
AGCCACCCTGACCATCTCTGGGGTCCAGGCTGAGGACGAGGCCGATTATTATTGCGTTACCTACGACAGC
GACCTTCGCACTGCGATATTCGGCGGAGGCACTCACCTGACCATCACAGGTGGTCCCACGTCTACACCCT
CGGTCTCTCTCTTCCCGCCCTCCTCTGAGGAGCTCAGCGCCAACAAGGCCACAGTGGTGTGTCTCATCAG
CGACTTCTCCCCCAGCGGCCTGGAGGTGATCTGGAAGGTAAATGACGCTGTCACCACCGACGGCGTCCAG
ACCACCAGGTCCTCGAAACAGAGCAACGGCAAGTACGCGGCCAGCAGCTACCTGACGCGGACTTCCGCAC
AGTGGAAATCGTACAGCAGCGTCAGCTGCCAGGTCAAGCACCAAGGGAAAACC
>KF985140.1 Equus caballus clone AdultA01 immmunoglobulin lambda light chain variable region (IGL) mRNA, partial cds
AAGCTCTGCTTCGGCTGTGGGGCCACAGAAGGCAGGACTCGGTGAAGATCTCCACCATGGCCTGGTCCCC
TCTCCTCCTCACCCTCATCGCTCTCTGCACAGGATCTTGGGCCCAGTCTGTGACTCAGCCCGCCTCAGTG
TCTGGAACCCTGGGCCAGACAGTCACCATCTCCTGTGCTGGCACCGACGACGACATCGGACATAGTTGGA
CGTATGTGGCCTGGCTCCAACAGTTCCCAGGAAGCGCCCCCAAGCCTCTATTCTATGCTAATAGGAAGCG
AGTCTCAGGGGTCCCAGATCGTTTCTCTGCTTCCAAGTCTGGCACTTCAGCCACCCTGACCATCTCTGGG
GTCCAGGCTGAGGACGAGGCCGATTTTTACTGTGCAGTGTATGGCGTCGACCAGGAAACAATATTCGGCG
GAGGTACCCACCTGACCGTCGCGGGTGGTCCCACGTCTACACCCTCGGTCTCTCTCTTCCCGCCCTCCTC
TGAGGAGCTCAGCGCCAACAAGGCCACAGTGGTGTGTCTCATCAGCGACTTCTCCCCCAGCGGCCTGGAG
GTGATCTGGAAGGTAAATGACGCTGTCACCACCGACGGCGTCCAGACCACCAGGTCCTCGAAACAGAGCA
ACGGCAAGTACGCGGCCAGCAGCTACCTGACGCGGACTTCCGCACAGTGGAAATCGTACAGCAGCGTCAG
CTGCCAGGTCACGCACCAAGGGAAAACC
>KF985139.1 Equus caballus clone AdultA02 immmunoglobulin lambda light chain variable region (IGL) mRNA, partial cds
TCTGCTTCAGCTGTGGGGCCACAGAAGGCAGGACTCGGTGAAGATCTCCACCATGGCCTGGTCCCCTCTC
CTCCTCACCCTCATCGCTCTCTGCACAGGATCCTGGGCCCAGTCTCTGACTCAGCCCGCCTCAGTGTCTG
GTACCCTGGGCCAGACAGTCACCATCTCGTGCACGGGAAATGCCACCGGCATAGGTTCCTATGCTGGCTG
GTACCAACAGATCCCGGGGACAGCCCCCAAAACCCTCATCCATGCAGGAACACAGGCACGGGCCTCAGGG
GTCACATCTCGATACTCTGCCTCCGCCTTCGGCTACACAGCCACCCTGACCATCACTGGGCTTCAGGCTG
GGGACGAGGCCGATTATTATTGTGGCTCCTTCGACGTCGAACTTGACGCCTATGTTTTCGGCGGAGGCAC
GCAATTGACCCTCGCAGGTGGTCCCACGTCTACACCCTCGGTCTCTCTCTTCCCGCCCTCCTCTGAGGAG
CTCAGCGCGAACAAGGCCACAGTGGTGTGTCTCATCAGTGACTTCTCCCCCAGCGACTTGACGGTGAGCT
GGAAGGTAAATGGCGCCGCCACCACCCAGGGCGTCCAGACCACCAAGCCCTCGAAACAGAGCAACGGCAA
GTACGCAGCCAGCAGCTACCTGTCGCTGACCCCCAGCCAGTGGAAATCGTCCAGCAGCGTCAGCTGCCAG
GTCACGCACCAAGGGAAAACC
>KF985138.1 Equus caballus clone AdultC01 immmunoglobulin lambda light chain variable region (IGL) mRNA, partial cds
AAGCTCTGCTTCAGCTGTGGGGCCACAGAAGGCAGGACTCGGTGACAATCTCCACCATGGCCTGGTCCCC
TCTCCTCCTCACCCTCATCGCTCTCTGCACAGGATCCTGGGCCCAGTCTCTGACACAGCCCGCCTCAGTG
TCTGGGACCCTGGGCCAGACAGTCACCATCTCCTGCTCTGGAAGCAGCTCCAACATCGGATATAGTCTAA
ATAATGGGGGCTGGTACCAACAGACCCCAGGAACAGCCCCCAAACTCCTCATATATGAGGGTAGCCTACG

AGCCTCAGGGGTCCCAGGTCGATTCTCTGGCTCCAAGTCTGGCAACACAAATACCCTGACCATCGCTGGG
CTCCAGGCTGAGGACGAGGCCGATTATTACTGTTATTCCTATGACAGCAACCGGAAGACAACGATATTCG
GCGGAGGCACCCACCTGACCATCGCAGGTGGTCCCACGTCTACACCCTCGGTCTCTCTCTTCCCGCCCTC
CTCTGAGGAGCTCAGCGCGAACAAGGCCACAGTGGTGTGTCTCATCAGTGACTTCTCCCCCAGCGACTTG
ACGGTGAGCTGGAAGGTAAATGGCGCCGCCACCACCCAGGGCGTCCAGACCACCAAGCCCTCGAAACAGA
GCAACGGCAAGTACGCAGCCAGCAGCTACCTGTCGCTGACCCCCAGCCAGTGGAAATCGTCCAGCAGCGT
CAGCTGCCAGGTCACGCACCAAGGGAAAACC
>KF985137.1 Equus caballus clone AdultB06 immmunoglobulin lambda light chain variable region (IGL) mRNA, partial cds
AGCTCTGCTTCAGCTGTGGGGCCACAGAAGGCAGGACTCGGTGACAATCTCCACCATGGCCTGGTCCCCT
CTCCTCCTCACCCTCATCGCTCTCTGCACAGGATCCTGGGCCCAGTCTCTGACTCAGCCCGCCTCAGTGT
CTGGGACCCTGGGCCAGACAGTCACCATCTCCTGCTCTGGAAGCAGCTCCAACATCGGGTATAGTAGTAA
ATATGGGGGCTGGTACCAACAGATCCCAGGAACAGCCCCCAAACTCCTCATATATGAGGGTAACAAACGA
GCCTCAGGGGTCCCAGATCAATTCTCTGGCTCCAAGTCTGGCAACACAGCCACCCTGATCATCTCTGGGC
TCCAGGCTGAGGACGAGGCCGATTATTACTGTGTTTCCTATGACAATAGCCTGAGTAGTGCTGTATTCGG
CGGAGGCACCCACCTGACCATCGCAGGTGGTCCCACGTCTACACCCTCGGTCTCTCTCTTCCCGCCCTCC
TCTGAGGAGCTCAGCGCGAACAAGGCCACAGTGGTGTGTCTCATCAGTGACTTCTCCCCCAGCGACTTGA
CGGTGAGCTGGAAGGTAAATGGCGCCGCCACCACCCAGGGCGTCCAGACCACCAAGCCCTCGAAACAGAG
CAACGGCAAGTACGCAGCCAGCAGCTACCTGTCGCTGACCCCCAGCCAGTGGAAATCGTCCAGCAGCGTC
AGCTGCCAGGTCACGCACCAAGGGAAAACC
>KF985136.1 Equus caballus clone AdultB09 immmunoglobulin lambda light chain variable region (IGL) mRNA, partial cds
TGCTTCAGCTGTGGGGCCACAGAAGGCAGGACTCGGTGACGATCTCCACCATGGCCTGGTCCCCTCTCCT
CCTCACCCTCATCGCTCTCTGCACAGGATCCTGGGCCCAGTCTCTGACTCAGCCCGCCTCAGTCTCTGGG
ACCCTGGGCCAGACAGTCACCATCACCTGCTCTGGAGAAAGCTCCAACATCGGGGAAGACGTTACTTCTG
TGGGCTGGTTCCAACAGTTCCCAGGAACAGCCCCCAAAACCCTCATCGGTAATAATAATGAACGAGCGTC
GGGGGTCCCCAATCGATTCTCCGGATCCAAGTCCGGTAACAAAGCCACCCTGACCATCTCTGGGGTCCAG
GCTGAGGACGAGGCCGACTATTACTGTGCAGCAGGAGACGACAGCCTCCATTCTTATGTGTTCGGCGGAG
GCACCCACCTGACCGTAGCAGGTGGTCCCACGTCTACACCCTCGGTCTCTCTCTTCCCGCCCTCCTCTGA
GGAGCTCAGCGCGAACAAGGCCACAGTGGTGTGTCTCATCAGTGACTTCTCCCCCAGCGACTTGACGGTG
AGCTGGAAGGTAAATGGCGCCGCCACCACCCAGGGCGTCCAGACCACCAAGCCCTCGAAACAGAGCAACG
GCAAGTACGCAGCCAGCAGCTACCTGTCGCTGACCCCCAGCCAGTGGAAATCGTCCAGCAGCGTCAGCTG
CCAGGTCACGCACCAAGGGAAAACC
>KF985135.1 Equus caballus clone AdultA10 immmunoglobulin lambda light chain variable region (IGL) mRNA, partial cds
AAGGCAGGACTCGGTGACGATCTCCACCATGGCCTGGTCCCCTCTCCTCCTCACCCTCATCGCTCTCTGC
ACAGGATCCTGGGCCCAGTCTCTGACTCAGCCCGCCTCAGTGTCTGGGACCCTGGGCCAGACAGTGTCCA
TCTCCTGCTCTGGAAGCAGAGCCAACATCGGATATACGTATAGTTCTGTGGGCTGGTTCCAACAGATCCC
AGGAACAGCCCCCAAAACCCTCATCTATAGTAATAACAAACGAGCCTCAGGGGTCCCAGATCGATTCTCT
GGCTCCAAGTCTGGCAACACAGCCACCCTGACCATCTCTGGGGTCCAGGCTGAGGACGAGGCTGCCTATT
ACTGTGGTACAAGAGACAGCAGTGTTAATGGCCTTGTATTCGGCGGAGGCACCCAGCTGACCATCGCAGG
TGGTCCCACGTCTACACCCTCGGTCTCTCTCTTCCCGCCCTCCTCTGAGGAGCTCAGCGCGAACAAGGCC
ACAGTGGTGTGTCTCATCAGTGACTTCTCCCCCAGCGACTTGACGGTGAGCTGGAAGGTAAATGGCGCCG
CCACCACCCAGGGCGTCCAGACCACCAAGCCCTCGAAACAGAGCAACGGCAAGTACGCAGCCAGCAGCTA
CCTGTCGCTGACCCCCAGCCAGTGGAAATCGTCCAGCAGCGTCAGCTGCCAGGTCACGCACCAAGGGAAA
ACC
>KF985134.1 Equus caballus clone AdultD01 immmunoglobulin lambda light chain variable region (IGL) mRNA, partial cds
AAGCTCTGCTTCAGCTGTGGGGCCACAGAAGGCAGGACTCGGTGACGATCTCCACCATGGCCTGGTGCCC
TCTCCTCCTCACCCTCATCGCTCTCTGCACAGGATCCTGGGCCCAGTCTGTGACTCAGCCCGCCTCAGTG
TCTGGGACCCTGGGCCAGACAGTCACCATCACCTGCACTGGAAGCAGCTCCAACATAGTTGCTTGGGTGG
GCTGGTACCAACAGGTCCCAGGAACAGCCCCCAAAACCCTCATCTATAATAATAACAAACGAGCCTCAGG
GGTCCCAGATCGATTCTCTGGCTCCAAGTCTGGCAGCATAGCCACCCTGACCATCAGTGGGCTCCAGGCT
GAGGACGAGGCCGATTATTACTGTGGTACCTTCAGTGCTGGATTCGCCGGAGGCACCCACCTGACCATCG
CAGGTGGTCCCACGTCTACACCCTCGGTCTCTCTCTTCCCGCCCTCCTCTGAGGAGCTCAGCGCGAACAA
GGCCACAGTGGTGTGTCTCATCAGTGACTTCTCCCCCAGCGACTTGACGGTGAGCTGGAAGGTAAATGGC

GCCGCCACCACCCAGGGCGTCCAGACCACCAAGCCCTCGAAACAGAGCAACGGCAAGTACGCAGCCAGCA
GCTACCTGTCGCTGACCCCCAGCCAGTGGAAATCGTCCAGCAGCGTCAGCTGCCAGGTCACGCACCAAGG
GAAAACC
>KF985133.1 Equus caballus clone AdultB03 immmunoglobulin lambda light chain variable region (IGL) mRNA, partial cds
AGCTCTGCTTCAGCTGTGGGGCCACAGAAGGCAGGACTCGGTGACGATCTCCACCATGGCCTGGTGCCCT
CTCCTCCTCACCCTCATCGCTCTCTGCACAGTTCCAGAATCCTGGGCCCAGTCTGTGACTCAGCCCGCCT
CAGTGTCTGGGACCCTGGGCCAGACAGTCACCATCACCTGCACTGGGAGCAGCTCCAACTTAGTTGCTTA
TGTGGGCTGGTACCAACAAATCCCAGGAACTGCCCCCAAAACCCTCATCTATGGTGTCAACAAAAGAGCC
TCAGGGGTCCCAGATCGATTCTCTGGCTCCAAGTCTGGCAGCACAGCCACCCTGACCATCACTGGGCTCC
AGTCTGAGGACGAGGCCGATTATTACTGTGGTGGCTCTAGCAGCGGTGGTGGTGTTGCTGTATTCGGCGG
AGGCACCCACCTGACCATCGCAGGTGGTCCCACGTCTACACCCTCGGTCTCTCTCTTCCCGCCCTCCTCT
GAGGAGCTCAGCGCGAACAAGGCCACAGTGGTGTGTCTCATCAGTGACTTCTCCCCCAGCGACTTGACGG
TGAGCTGGAAGGTAAATGGCGCCGCCACCACCCAGGGCGTCCAGACCACCAAGCCCTCGAAACAGAGCAA
CGGCAAGTACGCAGCCAGCAGCTACCTGTCGCTGACCCCCAGCCAGTGGAAATCGTCCAGCAGCGTCAGC
TGCCAGGTCACGCACCAAGGGAAAACC
>KF985132.1 Equus caballus clone AdultC08 immmunoglobulin lambda light chain variable region (IGL) mRNA, partial cds
AAGCTCTGCTTCAGCTGTGGGGCCACAGAAGGCAGGACTCGGTGAAGATCTCCACCATGGCCTGGTCCCC
TCTCCTCCTCACCCTCATCGCTCTCTGCACAGGATCCTGGGCCCAGTCTGTGACTCAGCCCGCCTCAGTG
TCTGGGACCCTGGGCCAGACAGTCACCATCTCCTGCTCTGGAAGCAGGTCCGACATCGGCTTCAGTGACA
ATCATGTGGCCTGGTTCCAACAGATCCCAGGAAAAGCCCCCAAAACCCTCATACATGCTGTAAACAAGCG
AGCCTCAGGGGTCCCAGATCGATTCTCTGGCTCGCAGTCTGGCAACACAGTCACCCTGACCATCTCTGGG
GTCCAGGCTGAGGACGAGGCCGATTATTACTGTGCTACTTATGACAGCAGCAGTACGACTGCTCTCTTCG
GCGGAGGCACCCGGCTGACCATCGCAGGTGGTCCCACGTCTACACCCTCGGTCTCTCTCTTCCCGCCCTC
CTCTGAGGAGCTCAGCGCGAACAAGGCCACAGTGGTGTGTCTCATCAGTGACTTCTCCCCCAGCGACTTG
ACGGTGAGCTGGAAGGTAAATGGCGCCGCCACCACCCAGGGCGTCCAGACCACCAAGCCCTCGAAACAGA
GCAACGGCAAGTACGCAGCCAGCAGCTACCTGTCGCTGACCCCCAGCCAGTGGAAATCGTCCAGCAGCGT
CAGCTGCCAGGTCACGCACCAAGGGAAAACC
>KF985131.1 Equus caballus clone AdultC07 immmunoglobulin lambda light chain variable region (IGL) mRNA, partial cds
ACAGAAGGCAGGACTCGGTGAAGATCTCCACCATGGCCTGGTCCCCTCTCCTCCTCACCCTCATCGCTCT
CTGCACAGGATCCTGGGCCCAGTCTGTGACTCAGCCCGCCTCAGTGACTGGGACCCTGGGCCATACAGTC
ACCATCTCCTGCTCTGGAAGCATCTGGAACATTGGGGGTGGTACAGGTGATGTGAGCTGGTTCCAACAAG
TCCCAGGAACAGCCCCCAGAACTCTCATTTATGGTGCCACTAATAAGTCGTCCGGGGTCCCCGATCGATT
CTCTGGATCCAGGTCTGGCAACACAGCCACCCTGACCATCTCTGGGGTCCAGCCTGAGGACGAGGCTGAT
TACTACTGTTCGTCACCTGACAGCAGCCTGGGGACTGTTCTCTTCGGCGGCGGGACCCGTCTCAGCGTCC
TGGGTGGTCCCACGTCTGCACCCTCGGTCTCTCTCTTCCCGCCCTCCTCTGAGGAGCTCAGCGCCAACAA
GGCCACAGTGGTGTGTCTCATCAGTGACTTCTCCCCCAGCGACTTGACGGTGAGCTGGAAGGTAAATGGC
GCCGCCATCAGCCAGGGAGTCCAGACCACCAAGCCCTCGAAACAGAGCAATGGCAAGTACGCGGCTAGCA
GCTACCTGACGCTGACCCCCGCCCAGTGGAAATCGTCCAGCAGCGTCAGCTGCCAGGTCACGCACCAAGG
GAAAACC
>KF985130.1 Equus caballus clone FoalT26 immmunoglobulin lambda light chain variable region (IGL) mRNA, partial cds
GTTGGGGTCTCAGAAGGCAGTGCTCTTGGGGCGTCTCCACCATGGCCTGGACTCTGCTCCTTCTCACCCT
CCTCACTCAGGGTACAGGGTCCTGGGCCCAGTCTGCCCTGACTCAGCCTGCGTCAGTGTCCGGGGCTCTA
GGACAGTCGGTCACCATCACCTGTGCTGGAAGCAGCAGTGACATTGGGGGATATAATGCTGTCAGCTGGT
TGCAACAGCACCCGGGCACAGCCCCCAAAGTTCTGATTTATAGTGTGAATGCTCGGGCCTCAGGGATCCC
TGATCGCTTCTCTGGCTCCAAGTCTGGCAACACGGCCTCCCTGACCATCTCTGGGCTCCAGGTTGAGGAC
GAGGCTGATTATTACTGTTACTCGCTTGTGAGTGATTACACACTTGCATTCGGCGGAGGCACCCACCTGA
CCATCACAGGTGGTCCCACGTCTACACCCTCGGTCTCTCTCTTCCCGCCCTCCTCTGAGGAGCTCAGCGC
CAACAAGGCCACAGTGGTGTGTCTCATCAGTGACTTCTCCCCCAGCGGCTTGGAGGTGATCTGGAAGGTA
AATGACGCTGTCACCACCGACGGCGTCCAGACCACCAGGTCCTCGAAACAGAGCAACGGCAAGTACGCGG
CCAGCAGCTACCTGACGCGGACTTCCGCACAGTGGAAATCGTACAGCAGCGTCAGCTGCCAGGTCAAGCA
CCAAGGGAAAACC

>KF985129.1 Equus caballus clone FoalA10 immmunoglobulin lambda light chain variable region (IGL) mRNA, partial cds
AGCTCTGCTTCAGCTGTGGGGCCACTGAAGGCAGGACTCGGTGACAATCTCCACCATGGCCTGGTCCCCT
CTCCTCCTCACCCTCATCGCTCTCTGCACAGGATCCCGGGCCCAGTCTGTGACTCAGCCTGCCTCAGTGT
CTGGGACCCTGGGCCAGACAGTCACCATCTCCTGCTCTGGAAGCAGCTCCAACGTTGGGAGTGGTTATGT
GTCCTGGTACCAACAGATCCCAGGAACAGCCCCCAAACTCCTCATCTATTATGCCACTAGTAGGGCTTCC
GGGGTCCCCGACCGATTCTCTGGCACCAGGTCTGGCAACACAGCCACCCTGACCATCTCTGGGCTCCAGG
CTGAGGATGAGGCCGATTATTACTGTGGTACCTCTGGCAGCAGTTGGAATAGTGATGGTGCATTCGGCGG
AGGCACCCACCTGACCATCGCAGGTGGTCCCACGTCTACACCCTCGGTCTCTCTCTTCCCGCCCTCCTCT
GAGGAGCTCAGCGCCAACAAGGCCACAGTGGTGTGTCTCATCAGTGACTTCTCCCCCAGCGGCTTGGAGG
TGATCTGGAAGGTAAATGACGCTGTCACCACCGACGGCGTCCAGACCACCAGGTCCTCGAAACAGAGCAA
CGGCAAGTACGCGGCCAGCAGCTACCTGACGCGGACTTCCGCACAGTGGAAATCGTACAGCAGCGTCAGC
TGCCAGGTCACGCACCAAGGGAAAACC
>KF985128.1 Equus caballus clone FoalA04 immmunoglobulin lambda light chain variable region (IGL) mRNA, partial cds
GAAGATCTGCTTCGGCTGTGGGGCCACAGAAGGCAGGACTCGGTGAAGATCTCCACCATGGCCTGGTCCC
CTCTCCTCCTCACCCTCATCGCTCTCTGCACAGGATCCTGGGCCCAGTCTCTGACTCAGCCCGCCTCAGT
GTCTGGGACCCTGGGCCAGACAGTCACCATCTCCTGCACTGGAAGCAGCTCCAGCATAGGTTCTTATATG
GGCTGGTACCAACAGATCCCAGGGACAGCCCCCAAAACCCTCATCTATGCTAATAACGAACGAGCCTCAG
GGGTCCCAGATCGATTCTCTGGCTCCAAGTCTGGCAACACAGCCACCCTGACCATCTCTGGGGTCCAGGC
TGAGGACGAGGCCGATTATTACTGTGGTTCCTATGACAGCAGTAGTAGTGGTACATTCGGCGGAGGCACC
CACCTGACCATCGCAGGTGGTCCCACGTCTACACCCTCGGTCTCTCTCTTCCCGCCCTCCTCTGAGGAGC
TCAGCGCCAACAAGGCCACAGTGGTGTGTCTCATCAGTGACTTCTCCCCCAGCGGCTTGGAGGTGATCTG
GAAGGTAAATGACGCTGTCACCACCGACGGCGTCCAGACCACCAGGTCCTCGAAACAGAGCAACGGCAAG
TACGCAGCCAGCAGCTACCTGACGCGGACTTCCGCACAGTGGAAATCGTACAGCAGCGTCAGCTGCCAGG
TCAAGCACCAAGGGAAAACC
>KF985127.1 Equus caballus clone FoalT08 immmunoglobulin lambda light chain variable region (IGL) mRNA, partial cds
AAGCTCTGCTTCGGCTGTGGGGCCACAGAAGGCAGGACTCGGTGAAGATCTCCACCATGGCCTGGTCCCC
TCTCCTCCTCACCCTCATCGCTCTCTGCACAGGATCCTGGGCCCAGTCTCTGACTCAGCCCGCCTCAGTG
TCTGGGACCCTGGGCCAGACAGTCACCATCTCCTGCACTGGAAGCAGCTCCAGCATAGGAGAATGGATGG
GCTGGTACCAACAGATCCCAGGGACAGCCCCCAAAACCCTCATCTATCATAATGACGAACGAGCCTCAGG
GGTCCCAGATCGATTCTCTGGCTCCAAGTCTGGCAACACAGCCACCCTGACCATCTCTGGGCTTCAGGCT
GAGGACGAGGCCGATTATTACTGTGGTTCCTATTACAGAAGTGATGAAAGTGGTGCATTCGGCGGAGGCA
CCCACCTGACCATCGCAGGTGGTCCCACGTCTACACCCTCGGTCTCTCTCTTCCCGCCCTCCTCTGAGGA
GCTCAGCGCCAACAAGGCCACAGTGGTGTGTCTCATCAGTGACTTCTCCCCCAGCGGCTTGGAGGTGATC
TGGAAGGTAAATGACGCTGTCACCACCGACGGCGTCCAGACCACCAGGTCCTCGAAACAGAGCAACGGCA
AGTACGCGGCCAGCAGCTACCTGACGCGGACTTCCGCACAGTGGAAATCGTACAGCAGCGTCAGCTGCCA
GGTCAAGCACCAAGGGAAAACC
>KF985126.1 Equus caballus clone FoalT15 immmunoglobulin lambda light chain variable region (IGL) mRNA, partial cds
GTGGCTTCAGAGGCAGGGCTTTGGGACATCTCCACCATGGCCTGGACCCCTCTCTTGTTAGCCTTCCTCA
CTCTCTGCACAGGTCCCGTGGCCTCTTCTAAGCTGACTCAGCCATCTTCAGTGTCTGTGGCCTTGGGACA
GACGGCCACCATCACCTGCAAGGGAGGCGACTTTGAAAGTTTTGTTGGTAGCTGGTACCAGCAGAAGCCA
GGCCAGGCCCCTGTGCTGGTCATCGATGCTAGTAATGAGCGGCCCTCAGGGATCCCTGAACGATTCTCTG
GCTCCAGCTCAGGAGACACAGCCACGCTGACCATCAGCGGGGCCCAGGCTGAGGACGAGGCTGACTATTA
CTGTCTGGCAGCAGATGCTTCTGATGGTGCATTCGGCGGAGGCACCCACCTGACCATCGCAGGTGGTCCC
ACGTCTACACCCTCGGTCTCTCTCTTCCCGCCCTCCTCTGAGGAGCTCAGCGCCAACAAGGCCACAGTGG
TGTGTCTCATCAGTGACTTCTCCCCCAGCGGCCTGGAGGTGATCTGGAAGGTAAATGACGCTGTCACCAC
CGACGGCGTCCAGACCACCAGGTCCTCGAAACAGAGCAACGGCAAGTACGCGGCCAGCAGCTACCTGACG
CGGACTTCCGCACAGTGGAAATCGTACAGCAGCGTCAGCTGCCAGGTCAAGCACCAAGGGAAAACC
>KF985124.1 Equus caballus clone FoalT06 immmunoglobulin lambda light chain variable region (IGL) mRNA, partial cds
GCTCTGCTTCAGCTGTGGGGCCACAGAAGGCAGGACTCGGTGACAATCTCCACCATGGCCTGGTGCCCTC
TCCTCCTCACCCTCATCGCTCTCTGCACAGGATCCTGGGCCCAGTCTGTGACTCAGCCCGCCTCAGTGTC
TGGGACCCTGGGCCAGACAGTCACCATCACCTGCACTGGAAGCAGCTCCAACATAGTTGCTTATGTGGGC

TGGTACCAACAGATCCCAGGAACAGCCCCCAAAATCCTCATCTATGCTAATAACAAACGAACCTCAGGGG
TTCCAGATCGATTCTCTGGCTCCAAGTCTGGCAGCACAGCCACCCTGACCATCACTGGGCTCCAGGCTGA
GGACGAGGCCGATTATTACTGTGGTACGTCTAGCAGCAGTGATAGTGGTGCATTCGGCGGAGGCACCCAC
CTGACCATCGCAGGTGGTCCCACGTCTACACCCTCGGTCTCTCTCTTCCCGCCCTCCTCTGAGGAGCTCA
GCGCCAACAAGGCCACAGTGGTGTGTCTCATCAGTGACTTCTCCCCCAGCGGCTTGGAGGTGATCTGGAA
GGTAAATGACGCTGTCACCACCGACGGCGTCCAGACCACCAGGTCCTCGAAACAGAGCAACGGCAAGTAC
GCGGCCAGCAGCTACCTGACGCGGACTTCCGCACAGTGGAAATCGTACAGCAGCGTCAGCTGCCAGGTCA
AGCACCAAGGGAAAACC
>KF985123.1 Equus caballus clone FoalT03 immmunoglobulin lambda light chain variable region (IGL) mRNA, partial cds
AAGCTCTGCTTCAGCTGTGGGGCCACAGAAGGCAGGACTCGGTGAAGATCTCCACCATGGCCTGGTCCCC
TCTCCTCCTCACCCTCATCGCTCTCTGCACAGGATCCTGGGCCCAGTCTGTGACTCAGCCCGCCTCAGTG
TCTGGGACCCTGGGCCAGACAGTCACCATCTCCTGCTCTGGAAGCAGCTCCAACATCGGGAATAGTATCA
GTTATGTGGGCTGGTACCAACAGATCCCAGGAACAGCCCCCAAAACCCTCATCTATGGTAATAACAAACG
AGCCTCAGGGGTCCCAGATCGATTCTCTGGCTCCAAGTCTGGCAACACAGCCACCCTGACCATCACTGGG
GTCCAGGCTGAGGACGAGGCTGACTATTACTGTAATACTTATGACGTAAGCCTGAGTAGTGATGCATTCG
GCGGAGGCACCCACCTGACCATCGCAGGTGGTCCCACGTCTACACCCTCGGTCTCTCTCTTCCCGCCCTC
CTCTGAGGAGCTCAGCGCCAACAAGGCCACAGTGGTGTGTCTCATCAGTGACTTCTCCCCCAGCGGCTTG
GAGGTGATCTGGAAGGTAAATGACGCTGTCACCACCGACGGCGTCCAGACCACCAGGTCCTCGAAACAGA
GCAACGGCAAGTACGCGGCCAGCAGCTACCTGACGCGGACTTCCGCACAGTGGAAATCGTACAGCAGCGT
CAGCTGCCAGGTCAAGCACCAAGGGAAAACC
>KF985122.1 Equus caballus clone FoalT02 immmunoglobulin lambda light chain variable region (IGL) mRNA, partial cds
AAGCTCTGCTTCAGCTGTGGGGCCACAGAAGGCAGGACTCGGTGAAGATCTCCACCATGGCCTGGTCCCC
TCTCCTCCTCACCCTCATCGCTCTCTGCACAGGATCCTGGGCCCAGTCTGTGACTCAGCCCGCCTCAGTG
TCTGGGACCCTGGGCCAGACAGTCACCATCTCCTGCTCTGGAACCAGCTCCAACATCGGGTATAGTAGTG
GTAGTAATGTGGGCTGGTTGCAACAGATCCCAGGAACAGCCCCCAAAGCCCTCATCTATAGTAGTAACAA
ACGAGCCTCAGGGGTCCCAGATCGATTCTCTGGCTCCACGTCTGGCAACACGGCCACCTTGACCATCTCT
GGGGTCCAGGCTGAGGACGACGCCGATTATTACTGTGGTTCCTGGGACACCAGCCTCATGGTTGTTGCAT
TCGGCGGAGGCACCCACCTGACCATCGCAGGTGGTCCCACGTCTACACCCTCGGTCTCTCTCTTCCCGCC
CTCCTCTGAGGAGCTCAGCGCCAACAAGGCCACAGTGGTGTGTCTCATCAGTGACTTCTCCCCCAGCGGC
TTGGAGGTGATCTGGAAGGTAAATGACGCTGTCACCACCGACGGCGTCCAGACCACCAGGTCCTCGAAAC
AGAGCAACGGCAAGTACGCGGCCAGCAGCTACCTGACGCGGACTTCCGCACAGTGGAAATCGTACAGCAG
CGTCAGCTGCCAGGTCAAGCACCAAGGGAAAACC
>KF985121.1 Equus caballus clone FoalA08 immmunoglobulin lambda light chain variable region (IGL) mRNA, partial cds
CTTCAGCTGTGGGGCCACAGAAGGCAGGACTCGGTGAAGATCTCCACCATGGCCTGGTCCCCTCTCCTCC
TCACCCTCATCGCTCTCTGCACAGGATCCTGGGCCCAGTCTGTGACTCAGCCCGCCTCAGTGTCTGGGAC
CCTGGGCCAGACAGTCACCATCTCCTGCTCTGGAAGCAGCTCCAACATCGGGTATAGTGATGGTACTAGT
GTGGGCTGGTTGCAACAGATCCCAGGAACAGCCCCCAAAGTCCTCATCTATAGTAGTAACAAACGAGACT
CAGGGGTCCCAGATCGATTCTCTGGCTCCAAGTCTGGCAACACAGTCACCCTGACCATCACTGGGGTCCA
GGCTGAGGACGAGGCTGACTATTACTGTGCTTATTATGACAGCAGCCTGAGTAGTTATGCATTCGGCGGA
GGCACCCACCTGACCATCGCAGGTGGTCCCACGTCTACACCCTCGGTCTCTCTCTTCCCGCCCTCCTCTG
AGGAGCTCAGCGCCAACAAGGCCACAGTGGTGTGTCTCATCAGTGACTTCTCCCCCAGCGGCTTGGAGGT
GATCTGGAAGGTAAATGACGCTGTCACCACCGACGGCGTCCAGACCACCAGGTCCTCGAAACAGAGCAAC
GGCAAGTACGCGGCCAGCAGCTACCTGACGCGGACTTCCGCACAGTGGAAATCGTACAGCAGCGTCAGCT
GCCAGGTCAAGCACCAAGGGAAAACC
>KF985119.1 Equus caballus clone FoalT19 immmunoglobulin lambda light chain variable region (IGL) mRNA, partial cds
GCCACTGAAGGCAGGACTCGGTGACAATCTCCACCATGGCCTGGTCCCCTCTCCTCCTCACCCTCATCGC
TCTCTGCACAGGATCCCGGGCCCAGTCTGTGACTCAGCCTGCCTCAGTGTCTGGGACCCTGGGCCAGACA
GTCACCATCTCCTGCTCTGGAAGCAGCTCCAACGTTGGGAGTAATTATGTGGCCTGGTACCAACAGATCC
CAGGAACAGCCCCCAAACTCCTCATCTATCGTGATAACAAACGACCCTCAGGGGTCCCAGATCGATTCTC
TGCCTCCAAGTCTGGGAACACAGCCACCCTGACCATCTCTGGGGTCCAGGCTGAGGACGAGGCCGATTAT
TACTGTGCAGCAGGAGACAGCAGCCTGAATAGTGCTGTATTCGGCGGAGGCACCCACCTGACCATCGCAG

GTGGTACCCCGTCTGCACCCTCGGTCTCTCTCTTCCCGCCCTCCTCTGAGGAGCTCAGCGCCAACAAGGC
CACAGTGGTGTGTCTCATCAGTGACTTCTCCCCCAGCGACTTGACGGTGAGCTGGAAGGTAAATGGCGCC
GCCACCACCCAGGGCGTCCAGACCACCAAGCCCTCGAAACAGAGCAACGGCAAGTACGCAGCCAGCAGCT
ACCTGTCGCTGACCCCCAGCCAGTGGAAATCGTCCAGCAGCGTCAGCTGCCAGGTCACGCACCAAGGGAA
AACC
>KF985118.1 Equus caballus clone FoalT29 immmunoglobulin lambda light chain variable region (IGL) mRNA, partial cds
AAGCTCTGCTTCAGCTGTGGGGCCACTGAAGGCAGGACTCGGTGACAATCTCCACCATGGCCTGGTCCCC
TCTCCTCCTCACCCTCATCGCTCTCTGCACAGGATCCCGGGCCCAGTCTGTGACTCAGCCTGCCTCAGTG
TCTGGGACCCTGGGCCAGACAGTCACCATCTCCTGCTCTGGAAGCAGCTCCAACGTTGGGAGTGGTAGAG
TGTCCTGGTACCAACAGATCCCAGGAACAGCCCCCAAACTCCTCATCTATTTAGCCACTAGTAGGGCTTC
CGGGGTCCCCGACCGATTCTCTGGCACCAGGTCTGGCAACACAGCCACCCTGACCATCTCTGGACTCCAG
GCTGAGGATGAGGCCGATTATTACTGTGGTACCTCTGACAGCAGTTGGAGTAGTGGTGTATTCGGCGGAG
GCACCCACCTGACCATCGCAGGTGGTCCCACGTCTACACCCTCGGTCTCTCTCTTCCCGCCCTCCTCTGA
GGAGCTCAGCGCGAACAAGGCCACAGTGGTGTGTCTCATCAGTGACTTCTCCCCCAGCGACTTGACGGTG
AGCTGGAAGGTAAATGGCGCCGCCACCACCCAGGGCGTCCAGACCACCAAGCCCTCGAAACAGAGCAACG
GCAAGTACGCAGCCAGCAGCTACCTGTCGCTGACCCCCAGCCAGTGGAAATCGTCCAGCAGCGTCAGCTG
CCAGGTCACGCACCAAGGGAAAACC
>KF985117.1 Equus caballus clone FoalT32 immmunoglobulin lambda light chain variable region (IGL) mRNA, partial cds
AGGGTTGGGGTCTCAGAAGGCAGTGCTCTTGGGGCGTCTCCACCATGGCCTGGACTCTGCTCCTTCTCAC
CCTCCTCATTCAGGGTACAGGGTCCTGGGCCCAGTCTGCCCTGACTCAGCCTGCGTCAGTGTCCGGGGCT
CTAGGACAGTCGGTCACCATCACCTGTGCTGGAAGCAGCAGTGACATTGGGGGTTATAATGCTGTCAGCT
GGTTGCAACAGCACCCGGGCACAGCCCCCAAAGTTCTGATTTATAGTGTGAATACTCGGGCCTCAGGGAT
CCCTGATCGCTTCTCTGGCTCCAAGTCTGGCAACACAGCCACCCTGACCATCTCTGGGGTCCAGGCTGAG
GACGAGGCCGATTATTACTGCTCAGCAGGAGACAGCAGTGGTAGTAGTGATGCTGTATTCGGCGGAGGCA
CCCACCTGACCATCGCAGGTGGTCCCACGTCTACACCCTCGGTCTCTCTCTTCCCGCCCTCCTCTGAGGA
GCTCAGCGCGAACAAGGCCACAGTGGTGTGTCTCATCAGTGACTTCTCCCCCAGCGACTTGACGGTGAGC
TGGAAGGTAAATGGCGCCGCCACCACCCAGGGCGTCCAGACCACCAAGCCCTCGAAACAGAGCAACGGCA
AGTACGCAGCCAGCAGCTACCTGTCGCTGACCCCCAGCCAGTGGAAATCGTCCAGCAGCGTCAGCTGCCA
GGTCACGCACCAAGGGAAAACC
>KF985116.1 Equus caballus clone FoalT20 immmunoglobulin lambda light chain variable region (IGL) mRNA, partial cds
AGGGTTGGGGTCTCAGAAGGCAGTGCTCTTGGGGCGTCTCCACCATGGCCTGGACTCTGCTCCTTCTCAC
CCTCCTCACTCAGGGTACAGGGTCCTGGGCCCAGTCTGCCCTGACTCAGCCTGCGTCAGTGTCCGGGGCT
CTAGGACAGTCGGTCACCATCACCTGTGCTGGAAGCAGCAGTGACATTGGGGGTTATAATGCTGTCAGCT
GGTTGCAACAGCACCCGGGCACAGCCCCCAAAGTTCTGATTTATAGTGTGAATACTCGGGCCTCAGGGAT
CCCTGATCGCTTCTCTGGCTCCAAGTCTGGCAACACGGCCTCCCTGACCATCTCTGGGCTCCAGGTTGAG
GACGAGGCTGATTATTACTGTTACTCGCTTGTGAGTGGTTACACTTTTGTATTCGGCGGAGGCACCCACC
TGACCATCGCAGGTGGTCCCACGTCTACACCCTCGGTCTCTCTCTTCCCGCCCTCCTCTGAGGAGCTCAG
CGCGAACAAGGCCACAGTGGTGTGTCTCATCAGTGACTTCTCCCCCAGCGACTTGACGGTGAGCTGGAAG
GTAAATGGCGCCGCCACCACCCAGGGCGTCCAGACCACCAAGCCCTCGAAACAGAGCAACGGCAAGTACG
CAGCCAGCAGCTACCTGTCGCTGACCCCCAGCCAGTGGAAATCGTCCAGCAGCGTCAGCTGCCAGGTCAC
GCACCAAGGGAAAACC
>KF985115.1 Equus caballus clone FoalT23 immmunoglobulin lambda light chain variable region (IGL) mRNA, partial cds
GTAGGCTCAGAGGCAGAAGTCTGGGGAATCTCCACCATGGCCTGGACCCCTCTCTTGTTAGCCTTCCTCT
CTCTCTGCACAGGTCCTGTTGTCTCTTCTGCAGTGACTCAGCCATCTGAGGTGTCCGTGGCCTTGGGACA
GAGAGCCACCCTCACCTGCCAGGGAAGCAACTTTGAATTTTTTTCTCCTAGCTGGTACCAGCAGAAGCCA
GGCCAGGCCCCTGTACTACTCATCAATATTAATAATGAGCGCCACTCAGGGATCCCTGAACGATTCTCCG
GCTCCAGCTCAGGAGACACGTCCACACTGACCATCAGTGGGGCCCAGGCTGAGGACGAGGCTGACTATTA
CTGTCTGGCAGTAGATGCTCTTAAAACTGTATTCGGCGGAGGCACCCACCTGACCATCGCAGGTGGTCCC
ACGTCTACACCCTCGGTCTCTCTCTTCCCGCCCTCCTCTGAGGAGCTCAGCGCGAACAAGGCCACAGTGG
TGTGTCTCATCAGTGACTTCTCCCCCAGCGACTTGACGGTGAGCTGGAAGGTAAATGGCGCCGCCACCAC
CCAGGGCGTCCAGACCACCAAGCCCTCGAAACAGAGCAACGGCAAGTACGCAGCCAGCAGCTACCTGTCG
CTGACCCCCAGCCAGTGGAAATCGTCCAGCAGCGTCAGCTGCCAGGTCACGCACCAAGGGAAAACC

>KF985114.1 Equus caballus clone FoalT18 immmunoglobulin lambda light chain variable region (IGL) mRNA, partial cds
TGGCTTCAGAGGCAGGGCTTTGGGACATCTCCACCATGGCCTGGACCCCTCTCTTGTTAGCCTTCCTCAC
TCTCTGCACAGGTCCCGTGGCCTCTTCTAAGCTGACTCAGCCATCTTCAGTGTCTGTGGCCTTGGGACAG
ACGGCCACCATCACCTGCAAGGGAGGCGACTTTGAAAGTTTTGTTGGTAGCTGGTACCAGCAGAAGCCAG
GCCAGGCCCCTGTGCTGGTCATCGATGCTAGTAATGAGCGGCCCTCAGGGATCCCTGAACGATTCTCTGG
CTCCAGCTCATTAGGCACATCCACGCTGACCATCAGCGGGGCCCAGGCTGAGGATGAGGCTGACTATTAC
TGTCAGCCAGCAGATACTCATAGTTCTGATGCTGTATTCGGCGGAGGCACCCACCTGACCATCGCAGGTG
GTCCCACGTCTACACCCTCGGTCTCTCTCTTCCCGCCCTCCTCTGAGGAGCTCAGCGCGAACAAGGCCAC
AGTGGTGTGTCTCATCAGTGACTTCTCCCCCAGCGACTTGACGGTGAGCTGGAAGGTAAATGGCGCCGCC
ACCACCCAGGGCGTCCAGACCACCAAGCCCTCGAAACAGAGCAACGGCAAGTACGCAGCCAGCAGCTACC
TGTCGCTGACCCCCAGCCAGTGGAAATCGTCCAGCAGCGTCAGCTGCCAGGTCACGCACCAAGGGAAAAC
C
>KF985112.1 Equus caballus clone FoalT25 immmunoglobulin lambda light chain variable region (IGL) mRNA, partial cds
GAAGCTCTGCTTCAGCTGTGGGGCCACAGAAGGCAGGACTCGGTGAAGATCTCCACCATGGCCTGGTCCC
CTCTCCTCCTCACCCTCATCGCTCTCTGCACAGGATCCTGGGCCCAGTCTCTGACTCAGCCCGCCTCAGT
GTCTGGGACCCTGGGCCAGACAGTCACCATCTCCTGCTCTGGAAGCAGCTCCAACATCGGGAATAGTTAT
AGTTATGTGGGCTGGTTCCAACAGATCCCAGGAACAGCCCCCAAAACCCTCATCTATGGTAATAACAAAC
GAGCCTCAGGGGTCCCAGATCGATTCTCTGGCTCCAAGTCTGGCAACACAGCCACCCTGACCATCTCTGG
GGTCCAGGCTGAGGACGAGGCCGATTATTACTGCTCAGCAGGAGACATGAGTGGTAGTGCTGTATTCGGC
GGAGGCACCCACCTGACCATCGCAGGTGGTCCCACGTCTACACCCTCGGTCTCTCTCTTCCCGCCCTCCT
CTGAGGAGCTCAGCGCGAACAAGGCCACAGTGGTGTGTCTCATCAGTGACTTCTCCCCCAGCGACTTGAC
GGTGAGCTGGAAGGTAAATGGCGCCGCCACCACCCAGGGCGTCCAGACCACCAAGCCCTCGAAACAGAGC
AACGGCAAGTACGCAGCCAGCAGCTACCTGTCGCTGACCCCCAGCCAGTGGAAATCGTCCAGCAGCGTCA
GCTGCCAGGTCACGCACCAAGGGAAAACC
>KF985111.1 Equus caballus clone FoalA01 immmunoglobulin lambda light chain variable region (IGL) mRNA, partial cds
AAGCTCTGCTTCGGCTGTGGGGCCACAGAAGGCAGGACTCGGTGAAGATCTCCACCATGGCCTGGTCCCC
TCTCCTCCTCACCCTCATCGCTCTCTGCACAGGATCCTGGGCCCAGTCTCTGACTCAGCCCGCCTCAGTG
TCTGGGACCCTGGGCCAGACAGTCACCATCTCCTGCACTGGAAGCAGCTCCAGCATAGGTTCTTATATGG
GCTGGTACCAACAGATCCCAGGGACAGCCCCCAAAACCCTCATCTATGCTAATAACAAACGAGCCTCAGG
GGTCCCAGATCGATTCTCTGGCTCCAAGTCTGGCAACACAGCCACCCTGACCATCTCTGGGCTTCAGGCT
GAGGACGAGGCCGATTATTACTGTGGTTCCTATTACAGCAGTGATAGTAGTGATCTTGCATTCGGCGGAG
GCACCCACCTGACCATCGCAGGTGGTCCCACGTCTACACCCTCGGTCTCTCTCTTCCCGCCCTCCTCTGA
GGAGCTCAGCGCCAACAAGGCCACAGTGGTGTGTCTCATTAGTGACTTCTCCCCCAGCGACTTGACGGTG
AGCTGGAAGGTAAATGGCGCCGCCACCACCCAGGGCGTCCAGACCACCAAGCCCTCGAAACAGAGCAACG
GCAAGTACGCAGCCAGCAGCTACCTGTCGCTGACCCCCAGCCAGTGGAAATCGTCCAGCAGCGTCAGCTG
CCAGGTCACGCACCAAGGGAAAACC
>KF985110.1 Equus caballus clone FoalA06 immmunoglobulin lambda light chain variable region (IGL) mRNA, partial cds
GCTCTGCTTCAGCTGTGGGGCCACAGAAGGCAGGACTCGGTGAAGATCTCCACCATGGCCTGGTCCCCTC
TCCTCCTCACCCTCATCGCTCTCTGCACAGGATCCTGGGCCCAGTCTGTGACTCAGCCCGCCTCAGTGTC
TGGGACCCTGGACCAGACAGTCACCATCTCCTGCTCTGGAAGCAGCTCCAATATCGGAGATAGTAGTGGT
GGTTATGTGGGCTGGTTGCAACAGACCCCAGGAACAGCCCCCAAAGCCCTCATCTATAGTAGTAACAAAC
GAGCCTCAGGGGTCCCAGATCGATTCTCTGGCTCCACGTCTGGCAACACAGCCACCTTGACCATCTCTGG
GGTCCAGGCTGAGGACGAGGCCGATTATTACTGTAGTTCCTATGACAGCAGCCTCAGTAGTTTTGTATTC
GGCGGAGGCACCCACCTGACCATCGCAGGTGGTCCCACGTCTACACCCTCGGTCTCTCTCTTCCCGCCCT
CCTCTGAGGAGCTCAGCGCGAACAAGGCCACAGTGGTGTGTCTCATCAGTGACTTCTCCCCCAGCGACTT
GACGGTGAGCTGGAAGGTAAATGGCGCCGCCACCACCCAGGGCGTCCAGACCACCAAGCCCTCGAAACAG
AGCAACGGCAAGTACGCAGCCAGCAGCTACCTGTCGCTGACCCCCAGCCAGTGGAAATCGTCCAGCAGCG
TCAGCTGCCAGGTCACGCACCAAGGGAAAACC
>KF985109.1 Equus caballus clone FoalA03 immmunoglobulin lambda light chain variable region (IGL) mRNA, partial cds
GCTTCAGCTGTGGGGCCACAGAAGGCAGGACTCGGTGAAGATCTCCACCATGGCCTGGTCCCCTCTCCTC

CTCACCCTCATCGCTCTCTGCACAGGATCCTGGGCCCAGTCTGTGACTCAGCCCGCCTCAGTGTCTGGGA
CCCTGGGCCAGACAGTCACCATCTCCTGCTCTGGAAGCAGCTCCAACATCGGGTATAGTAGTGGTAGTAG
TGTGGGCTGGTTGCAACAGATCCCAGGAACAGCCCCCAAAGCCCTCATCTATAGTAGTAACAAACGAGCC
TCAGGGGTCCCAGATCGATTCTCTGGCTCCACGTCTGGCAACACAGCCACCTTGACCATCTCTGGGGTCC
AGGCTGAGGACGAGGCCGATTATTACTGTGATTCCTGGGACAGCAGCCTCAGTAGTATTGTATTCGGCGG
AGGCACCCACCTGACCATCGCAGGTGGTCCCACGTCTACACCCTCGGTCTCTCTCTTCCCGCCCTCCTCT
GAGGAGCTCAGCGCGAACAAGGCCACAGTGGTGTGTCTCATCAGTGACTTCTCCCCCAGCGACTTGACGG
TGAGCTGGAAGGTAAATGGCGCCGCCACCACCCAGGGCGTCCAGACCACCAAGCCCTCGAAACAGAGCAA
CGGCAAGTACGCAGCCAGCAGCTACCTGTCGCTGACCCCCAGCCAGTGGAAATCGTCCAGCAGCGTCAGC
TGCCAGGTCACGCACCAAGGGAAAACC
>KF985108.1 Equus caballus clone FoalT30 immmunoglobulin lambda light chain variable region (IGL) mRNA, partial cds
GCTTCAGCTGTGGGGCCACAGAAGGCAGGACTCGGTGAAGATCTCCACCATGGCCTGGTCCCCTCTCCTC
CTCACCCTCATCGCTCTCTGCACAGGATCCTGGGCCCAGTCTGTGACTCAGCCCGCCTCAGTGTCTGGGA
CCCTGGGCCAGACAGTCACCATCTCCTGCTCTGGAAGCAGCTCCAACATCGGGTATAGTAGTGGTAGTTA
TGTGGGCTGGTTGCAACAGATCCCAGGAACAGCCCCCAAAGCCCTCATCTATAGTAGTAACAAACGAGCC
TCAGGGGTCCCAGATCGATTCTCTGGCTCCACGTCTGGCAACACAGCCACCTTGACCATCTCTGGGGTCC
AGGCTGAGGACGAGGCCGATTATTACTGTGGTTCCTATGACAGCAGCCTCAGTAGTGTTGTATTCGGCGG
AGGCACCCACCTGACCATCGCAGGTGGTCCCACGTCTACACCCTCGGTCTCTCTCTTCCCGCCCTCCTCT
GAGGAGCTCAGCGCCAACAAGGCCACAGTGGTGTGTCTCATCAGTGACTTCTCCCCCAGCGACTTGACGG
TGAGCTGGAAGGTAAATGGCGCCGCCACCACCCAGGGCGTCCAGACCACCAAGCCCTCGAAACAGAGCAA
CGGCAAGTACGCAGCCAGCAGCTACCTGTCGCTGACCCCCAGCCAGTGGAAATCGTCCAGCAGCGTCAGC
TGCCAGGTCACGCACCAAGGGAAAACC
>KF985107.1 Equus caballus clone FoalT24 immmunoglobulin lambda light chain variable region (IGL) mRNA, partial cds
AGCTCTGCTTCAGCTGTGGGGCCACAGAAGGCAGGACTCGGTGACGATCTCCACCATGGCCTGGTGCCCT
CTCCTCCTCACCCTCATCGCTCTCTGCACAGGATCCTGGGCCCAGTCTGTGACTCAGCCCGCCTCAGTGT
CTGGGACCCTGGGCCAGACAGTCACCATCTCCTGCTCTGGAAGCAGCTCCAACATCGGGAATATGGATTA
CGCTGTGGGCTGGTACCAACAGATCCCAGGAACAGCCCCCAAAATCCTCATCTATGGTAATAACAAACGA
GCCTCAGGGGTCCCAGATCGATTCTCTGGCTCCAAGTCTGGCAACACAGCCACCCTGACCATCTCTGGGG
TCCAGGCTGAGGACGAGGCCGATTATTACTGTGGTTCCTATGACAGCAGCAGTAGTAGTTTTGTATTCGG
CGGAGGCACCCACCTGACCATCGCAGGTGGTCCCACGTCTACACCCTCGGTCTCTCTCTTCCCGCCCTCC
TCTGAGGAGCTCAGCGCGAACAAGGCCACAGTGGTGTGTCTCATCAGTGACTTCTCCCCCAGCGACTTGA
CGGTGAGCTGGAAGGTAAATGGCGCCGCCACCACCCAGGGCGTCCAGACCACCAAGCCCTCGAAACAGAG
CAACGGCAAGTACGCAGCCAGCAGCTACCTGTCGCTGACCCCCAGCCAGTGGAAATCGTCCAGCAGCGTC
AGCTGCCAGGTCACGCACCAAGGGAAAACC
>KF985106.1 Equus caballus clone FoalT13 immmunoglobulin lambda light chain variable region (IGL) mRNA, partial cds
AAGCTCTGCTTCAGCTGTGGGGCCACAGAAGGCAGGACTCGGTGAAGATCTCCACCATGGCCTGGTCCCC
TCTCCTCCTCACCCTCATCGCTCTCTGCACAGGATCCTGGGCCCAGTCTGTGACTCAGCCCGCCTCAGTG
TCTGGGACCCTGGGCCAGACAGTCACCATCTCCTGCTCTGGAAGCAGCTCCAACATCGGGTGGAGTAGTG
GTAATTATGTGGGCTGGTTGCAACAGATCCCAGGAACAGCCCCCAAAGCCCTCATCTATAATAGTAACAA
ACGAGCCTCAGGGGTCCCAGATCGATTCTCTGGCTCCACGTCTGGCAACACAGCCACCTTGACCATCTCT
GGGGTCCAGGCTGAGGACGAGGCCGATTATTACTGTGGTGCCTATGACACGAGCCTCAGTAGTCCTGTAT
TCGGCGGAGGCACCCACCTGACCATCGCAGGTGGTCCCACGTCTACACCCTCGGTCTCTCTCTTCCCGCC
CTCCTCTGAGGAGCTCAGCGCGAACAAGGCCACAGTGGTGTGTCTCATCAGTGACTTCTCCCCCAGCGAC
TTGACGGTGAGCTGGAAGGTAAATGGCGCCGCCACCACCCAGGGCGTCCAGACCACCAAGCCCTCGAAAC
AGAGCAACGGCAAGTACGCAGCCAGCAGCTACCTGTCGCTGACCCCCAGCCAGTGGAAATCGTCCAGCAG
CGTCAGCTGCCAGGTCACGCACCAAGGGAAAACC
>KF985105.1 Equus caballus clone FoalT07 immmunoglobulin lambda light chain variable region (IGL) mRNA, partial cds
GCTTCAGCTGTGGGGCCACAGAAGGCAGGACTCGGTGAAGATCTCCACCATGGCCTGGTCCCCTCTCCTC
CTCACCCTCATCGCTCTCTGCACAGGATCCTGGGCCCAGTCTGTGACTCAGCCCGCCTCAGTGTCTGGGA
CCCTGGGCCAGACAGTCACCATCTCCTGCTCTGGAAGCAGCTCCAACATCGGGAATAGTTATAGTGATGT
GGGCTGGTACCAACAGATCCCAGGAACAGCCCCCAAAACCCTCATCTATGAGAGTAACAAGCGAGCCTCA
GGGGTCCCAGATCGATTCTCTGGCTCCAAGTCTGGCAACACAGCCACCCTGACCATCACTGGGGTCCAGG

CTGAGGACGAGGCTGACTATTACTGTGCTACTTGGGACAAAAGCCTGAGTAGTACTGTATTCGGCGGCGG
CACCCACCTGACCATCGCAGGTGGTCCCACGTCTACACCCTCGGTCTCTCTCTTCCCGCCCTCCTCTGAG
GAGCTCAGCGCGAACAAGGCCACAGTGGTGTGTCTCATCAGTGACTTCTCCCCCAGCGACTTGACGGTGA
GCTGGAAGGTAAATGGCGCCGCCACCACCCAGGGCGTCCAGACCACCAAGCCCTCGAAACAGAGCAACGG
CAAGTACGCAGCCAGCAGCTACCTGTCGCTGACCCCCAGCCAGTGGAAATCGTCCAGCAGCGTCAGCTGC
CAGGTCACGCACCAAGGGAAAACC
>KF985104.1 Equus caballus clone FoalT04 immmunoglobulin lambda light chain variable region (IGL) mRNA, partial cds
CTGCTTCAGCTGTGGGGCCACAGAAGGCAGGACTCGGTGAAGATCTCCACCATGGCCTGGTCCCCTCTCC
TCCTCACCCTCATCGCTCTCTGCACAGGATCCTGGGCCCAGTCTGTGACTCAGCCCGCCTCAGTGTCTGG
GACCCTGGGCCAGACAGTCACCATCTCCTGCTCTGGAAGCAGCTCCAACATCGGGAATAGTTATAGTTAT
GTGGGCTGGTACCAACAGATCCCAGGAACAGCCCCCAAAACCCTCATCTATGGTAATAACAAACGAGCCT
CAGGGGTCCCAGATCGATTCTCTGGCTCCAAGTCTGGCAACACAGCCACCCTGACCATCACTGGGGTCCA
GGCTGAGGACGAGGCTGACTATTACTGTGCTACTTATGACAGCAGCCTGAGTAGTGCTGTATTCGGCGGA
GGCACCCACCTGACCATCGCAGGTGGTCCCACGTCTACACCCTCGGTCTCTCTCTTCCCGCCCTCCTCTG
AGGAGCTCAGCGCGAACAAGGCCACAGTGGTGTGTCTCATCAGTGACTTCTCCCCCAGCGACTTGACGGT
GAGCTGGAAGGTAAATGGCGCCGCCACCACCCAGGGCGTCCAGACCACCAAGCCCTCGAAACAGAGCAAC
GGCAAGTACGCAGCCAGCAGCTACCTGTCGCTGACCCCCAGCCAGTGGAAATCGTCCAGCAGCGTCAGCT
GCCAGGTCACGCACCAAGGGAAAACC
>KF985103.1 Equus caballus clone FoalT17 immmunoglobulin lambda light chain variable region (IGL) mRNA, partial cds
AGGCTCAGAGGCAGAAGTCTGGGGAATCTCCACCATGGCCTGGACCCCTCTCTTGTTAGCCTTCCTCTCT
CTCTGCACAGGTCCTGTTGTCTCTTCTGCAGTGACTCAGCCATCTGAGGTGTCCGTGGCCTTGGGACAGA
GAGCCACCCTCACCTGCCAGGGAAGCAACTTTGAATTTTTTTCTCCTAGCTGGTACCAGCAGAAGCCAGG
CCAGGCCCCTGTACTGCTCATCAATATTAATTTTGAGCGCCACTCAGGGATCCCTGAACGATTCTCCGGC
TCCAGCTCAGGAGACACGTCCACACTGACCATCAGTGGGGCCCAGGCTGAGGACGAGGCTGACTATTACT
GTCTGGCAGTAGATGCTCTTAGTTCTGAAACTAGTATCTTCGGCGGCGGGACCCACCTCAGCGTCCTGGG
TGGTCCCACGTCTGCACCCTCGGTCTCTCTCTTCCCGCCCTCCTCTGAGGAGCTCAGCGCCAACAAGGCC
ACAGTGGTGTGTCTCATCAGTGACTTCTCCCCCAGCGACTTGACGGTGAGCTGGAAGGTAAATGGCGCCG
CCATCAGCCAGGGAGTCCAGACCACCAAGCCCTCGAAACAGAGCAATGGCAAGTACGCGGCTAGCAGCTA
CCTGACGCTGACCCCCGCCCAGTGGAAATCGTCCAGCAGCGTCAGCTGCCAGGTCACGCACCAAGGGAAA
ACC
>KF985102.1 Equus caballus clone FoalT14 immmunoglobulin lambda light chain variable region (IGL) mRNA, partial cds
GCTTCAGCTGTGGGGCCACAGAAGGCAGGACTCGGTGAAGATCTCCACCATGGCCTGGTCCCCTCTCCTC
CTCACCCTCATCGCTCTCTGCACAGGATCCTGGGCCCAGTCTCTGACTCAGCCCGCCTCAGTGTCTGGGA
CCCTGGGCCAGACAGTCACCATCTCCTGCTCTGGAAGCAGCTCCAACATCGGGATTAGTTATAGTTCTGT
GGGCTGGTTCCAACAGATCCCAGGAACAGCCCCCAAAACCCTCATCTATGGTAATAACAAACGAGCCTCA
GGGGTCCCAGATCGATTCTCTGGCTCCAAGTCTGGCAACACAGCCACCCTGACCATCTCTGGGGTCCAGG
CTGAGGACGAGGCTGACTATTACTGTGCAGCAGGAGACATGAGCCTTAATGTGGATATCTTCGGCGGCGG
GACCCACCTCAGCGTCCTGGGTGGTCCCACGTCTGCACCCTCGGTCTCTCTCTTCCCGCCCTCCTCTGAG
GAGCTCAGCGCCAACAAGGCCACAGTGGTGTGTCTCATCAGTGACTTCTCCCCCAGCGACTTGACGGTGA
GCTGGAAGGTAAATGGCGCCGCCATCAGCCAGGGAGTCCAGACCACCAAGCCCTCGAAACAGAGCAATGG
CAAGTACGCGGCTAGCAGCTACCTGACGCTGACCCCCGCCCAGTGGAAATCGTCCAGCAGCGTCAGCTGC
CAGGTCACGCACCAAGGGAAAACC
>KF985101.1 Equus caballus clone FoalT16 immmunoglobulin lambda light chain variable region (IGL) mRNA, partial cds
AAGCTCTGCTTCAGCTGTGGGGCCACAGAAGGCAGGACTCGGTGAAGATCTCCACCATGGCCTGGTCCCC
TCTCCTCCTCACCCTCATCGCTCTCTGCACAGGATCCTGGGCCCAGTCTGTGACTCAGCCCGCCTCAGTG
TCTGGGACCCTGGGCCAGACAGTCACCATCTCCTGCTCTGGAAGCAGCTCCAACATCGGTGATAGTTATA
GTTATGTGGGCTGGTACCAACAGATCCCAGGAACAGCCCCCAAAACCCTCATCTATGGTGATGACAAACG
AGCCTCAGGGGTCCCAGATCGATTCTCTGGCTCCAAGTCTGGCAACACAGCCACCCTGACCATCACTGGG
GTCCAGGCTGAGGACGAGGCTGACTATTACTGCTCTACTTATGACAGCAGCCTGAGTAGTGATATCTTCG
GCGGCGGGACCCACCTCAGCGTCCTGGGTGGTCCCACGTCTGCACCCTCGGTCTCTCTCTTCCCGCCCTC
CTCTGAGGAGCTCAGCGCCAACAAGGCCACAGTGGTGTGTCTCATCAGTGACTTCTCCCCCAGCGACTTG

ACGGTGAGCTGGAAGGTAAATGGCGCCGCCATCAGCCAGGGAGTCCAGACCACCAAGCCCTCGAAACAGA
GCAATGGCAAGTACGCGGCTAGCAGCTACCTGACGCTGACCCCCGCCCAGTGGAAATCGTCCAGCAGCGT
CAGCTGCCAGGTCACGCACCAAGGGAAAACC
>KF985100.1 Equus caballus clone NeonateT28 immmunoglobulin lambda light chain variable region (IGL) mRNA, partial cds
GGGCTCAGAGGCAGAGTTCTGGGGCATCTCCACCATGGCCTGGACCCCTCTCCTGCTCCCCCTCCTCACT
CTCTGTATAGGTTCTGTGGTCTCCTTGGAGCTGACTCAGCCAGCTTCAGTTTCTGTGGCCTTAGGACAGA
CTGCCACGATCACCTGCCAGGGAGGAATCTTTGACAAGAAGTATGTGTACTGGTACCAGCAGAAGCCCGG
CGGGACCCCTGTGACAGTGATTTATAAGGATAGTGAGCGGCCCTCCGGGATCCCTGACAGATTCTCTAGC
TCCAACTCAGGGAACACAGCCACCCTGACCATCAGCAGGGCCCAGGCAGAGGACGAGGCCGTCTATTACT
GCCACTCAGTAGATAGTGATAATGCTAGTGTATTCGGCGGAGGCACCCACCTGACCATCGCAGGTGGTCC
CACGTCTACACCCTCGGTCTCTCTCTTCCCGCCCTCCTCTGAGGAGCTCAGCGCCAACAAGGCCACAGTG
GTGTGTCTCATCAGCGACTTCTCCCCCAGCGGCCTGGAGGTGATCTGGAAGGTAAATGACGCTGTCACCA
CCGACGGCGTCCAGACCACCAGGTCCTCGAAACAGAGCAACGGCAAGTACGCGGCCAGCAGCTACCTGAC
GCGGACTTCCGCACAGTGGAAATCGTACAGCAGCGTCAGCTGCCAGGTCAAGCACCAAGGGAAAACC
>KF985099.1 Equus caballus clone NeonateT06 immmunoglobulin lambda light chain variable region (IGL) mRNA, partial cds
GGGATCCTGGGCCCAGTCTCTGACTCAGCCCGCCTCAGTGTCTGGGACCCTGGGCCAGACAGTCACCATC
TCCTGCTCTGGAAGCAGCTCCAACATCGGGTTGAGTAATAGTGGTGTGGGCTGGTACCAACAGCACCCGG
GCACAGCCCCCAAACTCCTCATTTATAGTGCCAGTTCTCGAGCCTCAGGGATCCCTGATCGCTTCTCTGG
CTCCAAGTCTGGGAACACGGCCTCTCTGACCATCTCGGGGCTCCAGGCTGAGGACGAGGCCGATTATTAC
TGTAGCTCATATAGCAATGCTGATCCTTGTGCATTCGGCGGAGGCACCCACCTGACCATCGCAGGTGGTC
CCACGTCTACACCCTCGGTCTCTCTCTTCCCGCCCTCCTCTGAGGAGCTCAGCGCCAACAAGGCCACAGT
GGTGTGTCTCATCAGTGACTTCTCCCCCAGCGGCCTGGAGGTGATCTGGAAGGTAAATGACGCTGTCACC
ACCGACGGCGTCCAGACCACCAGGTCCTCGAAACAGAGCAACGGCAAGTACGCGGCCAGCAGCTACCTGA
CGCGGACTTCCGCACAGTGGAAATCGTACAGCAGCGTCAGCTGCCAGGTCAAGCACCAAGGGAAAACC
>KF985098.1 Equus caballus clone NeonateT32 immmunoglobulin lambda light chain variable region (IGL) mRNA, partial cds
AAGCTCTGCTTCAGCTGTGGGGCCACAGAAGGCAGGACTCGGTGACAATCTCCACCATGGCCTGGTCCCC
TCTCCTCCTCACCCTCATCGCTCTCTGCACAGGATCCCGGGCCCAGTCTGTGACTCAGCCCGCCTCAGTG
TCTGGGACCCTGGGCCAGACAGTCACCATCTCCTGCTCTGGAAGCAGCTCCAACATCGGGAGTGGTCATG
TGTCCTGGTACCAACAGATCCTAGGAACAGCCCCCAAACGCCTCATCTATTCTTCCGTTAGCAGGGCTTC
CGGGGTCCCCGACCGATTCTCTGGCTCCAGGTCTGGCAACACAGCCACCCTGACCATCTCTGGGCTCCAG
GCTGAGGACGAGGCCGATTATTACTGTGGTACATTGTACATCAGTTGGAGTAGTGATGGTGCATTCGGCG
GAGGCACCCACCTGACCATCGCAGGTGGTCCCACGTCTACACCCTCGGTCTCTCTCTTCCCGCCCTCCTC
TGAGGAGCTCAGCGCCAACAAGGCCACAGTGGTGTGTCTCATCAGTGACTTCTCCCCCAGCGGCCTGGAG
GTGATCTGGAAGGTAAATGACGCTGTCACCACCGACGGCGTCCAGACCACCAGGTCCTCGAAACAGAGCA
ACGGCAAGTACGCGGCCAGCAGCTACCTGACGCGGACTTCCGCACAGTGGAAATCGTACAGCAGCGTCAG
CTGCCAGGTCAAGCACCAAGGGAAAACC
>KF985097.1 Equus caballus clone NeonateA01 immmunoglobulin lambda light chain variable region (IGL) mRNA, partial cds
AAGCTCTGCTTCAGCTGTGGGGCCACAGAAGGCAGGACTCGGTGAAGATCTCCACCATGGCCTGGTCCCC
TCTCCTCCTCACCCTCATCGCTCTCTGCACAGGATCCTGGGCCCAGTCTCTGACTCAGCCCGCCTCAGTG
TCTGGGACCCTGGGCCAGACAGTCACCATCTCCTGCACTGGAAGCAGCTCCAGCATAGTTTCTTATATGG
GGTGGTACCAACAGATCCCAGGGACAGCCCCCAAAACCCTCATCTATGGGACTAACAAACGAGCCTCAGG
GGTCCCAGATCGATTCTCTGGCTCCAAGTCTGGCAACACAGCCACCCTGACCATCTCTGGGCTTCAGGCT
GAGGACGAGGCCGATTATTACTGTGGTTCCGCCTACAGCAGTGATAGTGGTGATGGTGCATTCGGCGGAG
GCACCCACCTGACCATCGCAGGTGGTCCCACGTCTACACCCTCGGTCTCTCTCTTCCCGCCCTCCTCTGA
GGAGCTCAGCGCCAACAAGGCCACAGTGGTGTGTCTCATCAGTGACTTCTCCCCCAGCGGCCTGGAGGTG
ATCTGGAAGGTAAATGACGCTGTCACCACCGACGGCGTCCAGACCACCAGGTCCTCGAAACAGAGCAACG
GCAAGTACGCGGCCAGCAGCTACCTGACGCGGACTTCCGCACAGTGGAAATCGTACAGCAGCGTCAGCTG
CCAGGTCAAGCACCAAGGGAAAACC
>KF985095.1 Equus caballus clone NeonateT17 immmunoglobulin lambda light chain variable region (IGL) mRNA, partial cds
GGTTTGGGGTCTCAGAAGGCAGTGCTCTTGGGGCGTCTCCACCATGGCCTGGACTCTGCTCCTTCTCACC
CTCCTCATTCAGGGTACAGGGTCCTGGGCCCAGTCTGCCCTGACTCAGCCTGCGTCAGTGTCCGGGGCTC

TAGGACAGTCGGTCACCATCACCTGTGCTGGAAGCAGCAGTGACATTGGGGGTTATAATGCTGTCAGCTG
GTTGCAACAGCACCCGGGCACAGCCCCCAAAGTTCTGATTTATAGTGTGAATACTCGGGCCTCAGGGATC
CCTGATCGCTTCTCTGGCTCCAAGTCTGGCAACACGGCCTCCCTGACCATCTCTGGGCTCCAGGTTGAGG
ACGAGGCTGATTATTACTGTCACGCGTATGTTGGCAGTTACAATTATGTATTCGGCGGAGGCACCCACCT
GACCATCGCAGGTGGTCCCACGTCTACACCCTCGGTCTCTCTCTTCCCGCCCTCCTCTGAGGAGCTCAGC
GCCAACAAGGCCACAGTGGTGTGTCTCATCAGCGACTTCTCCCCCAGCGGCCTGGAGGTGATCTGGAAGG
TAAATGACGCTGTCACCACCGACGGCGTCCAGACCACCAGGTCCTCGAAACAGAGCAACGGCAAGTACGC
GGCCAGCAGCTACCTGACGCGGACTTCCGCACAGTGGAAATCGTACAGCAGCGTCAGCTGCCAGGTCAAG
CACCAAGGGAAAACC
>KF985094.1 Equus caballus clone NeonateT02 immmunoglobulin lambda light chain variable region (IGL) mRNA, partial cds
AGGGTTGGGGTCTCAGAAGGCAGTGCTCTTGGGGCGTCTCCACCATGGCCTGGACTCTGCTCCTTCTCAC
CCTCCTCATTCAGGGTACAGGGTCCTGGGCCCAGTCTGCCCTGACTCAGCCTGCGTCAGTGTCCGGGGCT
CTAGGACAGTCGGTCACCATCACCTGTGCTGGAAGCAGCAGTGACATTGGGGGTTATAATGCTGTCAGCT
GGTTGCAACAGCACCCGGGCACAGCCCCCAAAGTTCTGATTTATAGTGTGAATACTCGGGCCTCAGGGAT
CCCTGATCGCTTCTCTGGCTCCAAGTCTGGCAACACGGCCTCCCTGACCATCTCTGGGCTCCAGGTTGAG
GACGAGGCTGATTATTACTGTTACTCGCTTGTAAACGGTTTGCCTGCTGTATTCGGCGGAGGCACCCACC
TGACCATCGCAGGTGGTCCCACGTCTACACCCTCGGTCTCTCTCTTCCCGCCCTCCTCTGAGGAGCTCAG
CGCCAACAAGGCCACAGTGGTGTGTCTCATCAGCGACTTCTCCCCCAGCGGCCTGGAGGTGATCTGGAAG
GTAAATGACGCTGTCACCACCGACGGCGTCCAGACCACCAGGTCCTCGAAACAGAGCAACGGCAAGTACG
CGGCCAGCAGCTACCTGACGCGGACTTCCGCACAGTGGAAATCGTACAGCAGCGTCAGCTGCCAGGTCAA
GCACCAAGGGAAAACC
>KF985093.1 Equus caballus clone NeonateT07 immmunoglobulin lambda light chain variable region (IGL) mRNA, partial cds
GGGGAAGCTCTGCTTCAGCTGTGGGGCCACAGAAGGCAGGACTCGGTGAAGATCTCCACCATGGCCTGGT
CCCCTCTCCTCCTCACCCTCATCGCTCTCTGCACAGGATCCTGGGCCCAGTCTCTGACTCAGCCCGCCTC
AGTGTCTGGGACCCTGGGCCAGACAGTCACCATCTCCTGCACTGGAAGCAGCTCCAGCATAAGTTCTTAT
ATGGGCTGGTACCAACAGATCCCAGGGACAGCCCCCAAAACCCTCATCTATGCTACTAACAAACGAGCCT
CAGGGGTCCCAGATCGATTCTCTGGCTCCAAGTCTGGCAACACAGCCACCCTGACCATCTCTGGGCTTCA
GGCTGAGGACGAGGCCGATTATTACTGTGGTTCCTATTACATCAGTGATGACTATGCTGTATTCGGCGGA
GGCACCCACCTGACCATCGCAGGTGGTCCCACGTCTACACCCTCGGTCTCTCTCTTCCCGCCCTCCTCTG
AGGAGCTCAGCGCCAACAAGGCCACAGTGGTGTGTCTCATCAGCGACTTCTCCCCCAGCGGCCTGGAGGT
GATCTGGAAGGTAAATGACGCTGTCACCACCGACGGCGTCCAGACCACCAGGTCCTCGAAACAGAGCAAC
GGCAAGTACGCGGCCAGCAGCTACCTGACGCGGACTTCCGCACAGTGGAAATCGTACAGCAGCGTCAGCT
GCCAGGTCAAGCACCAAGGGAAAACC
>KF985092.1 Equus caballus clone NeonateT33 immmunoglobulin lambda light chain variable region (IGL) mRNA, partial cds
GCTTCAGCTGTGGGGCCACAGAAGGCAGGACTCGGTGAAGATCTCCACCATGGCCTGGTCCCCTCTCCTC
CTCACCCTCATCGCTCTCTGCACAGGATCCTGGGCCCAGTCTCTGACTCAGCCCGCCTCAGTGTCTGGGA
CCCTGGGCCAGACAGTCACCATCTCCTGCTCTGGAAGCAGCTCCAACATCGGGTATAGTTATAGTGCTGT
GGGCTGGTACCAACAGGTCCCAGGAACAGCCCTCAAAACCCTCATCTATGCTACTTACACACGAGCCTCA
GGGGTCCCAGATCGATTCTCTGGCTCCAAGTCTGGCAACACAGCCACCCTGACCATCTCTGGGGTCCAGG
CTGAGGACGAGGCGGATTATTACTGTGCAGCAGGAGACAGCAGCCTGAGGAGTGCTGTATTCGGCGGAGG
CACCCACCTGACCATCGCAGGTGGTCCCACGTCTACACCCTCGGTCTCTCTCTTCCCGCCCTCCTCTGAG
GAGCTCAGCGCCAACAAGGCCACAGTGGTGTGTCTCATCAGCGACTTCTCCCCCAGCGGCCTGGAGGTGA
TCTGGAAGGTAAATGACGCTGTCACCACCGACGGCGTCCAGACCACCAGGTCCTCGAAACAGAGCAACGG
CAAGTACGCGGCCAGCAGCTACCTGACGCGGACTTCCGCACAGTGGAAATCGTACAGCAGCGTCAGCTGC
CAGGTCAAGCACCAAGGGAAAACC
>KF985091.1 Equus caballus clone NeonateT24 immmunoglobulin lambda light chain variable region (IGL) mRNA, partial cds
AGCTCTGCTTCAGCTGTGGGGCCACAGAAGGCAGGACTCGGTGACGATCTCCACCATGGCCTGGTCCCCT
CTCCTCCTCACCCTCATCGCTCTCTGCACAGGATCCTGGGCCCAGTCTCTGACTCAGCCCGCCTCAGTGT
CTGGGACCCTGGGCCAGACAGTCACCATCTCCTGCTCTGGAAGCAGCTCCAACATCGGGAATAGTTATAG
TTCTGTGGGCTGGTTCCAACAGATCCCAGGAACAGCCCCCAAAACCCTCATCTATGGTAATAACAAACGA
GCCTCAGGGGTCCCAGATCGATTCTCTGGCTCCAAGTCTGGCAACACAGCCACCCTGACCATCTCTGGGG

TCCAGGCTGAGGACGAGGCTGACTATTACTGTGCAGCAGGAGACAGCAGCCTTAATGGTGCTGTATTCGG
CGGAGGCACCCACCTGACCATCGCAGGTGGTCCCACGTCTACACCCTCGGTCTCTCTCTTCCCGCCCTCC
TCTGAGGAGCTCAGCGCCAACAAGGCCACAGTGGTGTGTCTCATCAGCGACTTCTCCCCCAGCGGCCTGG
AGGTGATCTGGAAGGTAAATGACGCTGTCACCACCGACGGCGTCCAGACCACCAGGTCCTCGAAACAGAG
CAACGGCAAGTACGCGGCCAGCAGCTACCTGACGCGGACTTCCGCACAGTGGAAATCGTACAGCAGCGTC
AGCTGCCAGGTCAAGCACCAAGGGAAAACC
>KF985090.1 Equus caballus clone NeonateT12 immmunoglobulin lambda light chain variable region (IGL) mRNA, partial cds
AAGCTCTGCTTCAGCTGTGGGGCCACAGAAGGCAGGACTCGGTGAAGATCTCCACCATGGCCTGGTCCCC
TCTCCTCCTCACCCTCATCGCTCTCTGCACAGGATCCTGGGCCCAGTCTCTGACTCAGCCCGCCTCAGTG
TCTGGGACCCTGGGCCAGACAGTCACCATCTCCTGCTCTGGAAGCAGCTCCAACATCGGGTATAGATATA
ATGCTGTGGGCTGGTACCAACAGATCCCAGGAACAGCCCCCAAAACCCTCATCTATGGTAATAACAAACG
AGCCTCAGGGGTCCCAGATCGATTCTCTGGCTCCAAGTCTGGCAACACAGCCACCCTGACCATCTCTGGG
GTCCAGGCTGAGGACGAGGCCGATTATTACTGCTCAGCAGGAGACAGCAGTGGTAGTAGTGCATTCGGCG
GAGGCACCCACCTGACCATCGCAGGTGGTCCCACGTCTACACCCTCGGTCTCTCTCTTCCCGCCCTCCTC
TGAGGAGCTCAGCGCCAACAAGGCCACAGTGGTGTGTCTCATCAGTGACTTCTCCCCCAGCGGCCTGGAG
GTGATCTGGAAGGTAAATGACGCTGTCACCACCGACGGCGTCCAGACCACCAGGTCCTCGAAACAGAGCA
ACGGCAAGTACGCGGCCAGCAGCTACCTGACGCGGACTTCCGCACAGTGGAAATCGTACAGCAGCGTCAG
CTGCCAGGTCAAGCACCAAGGGAAAACC
>KF985089.1 Equus caballus clone NeonateT35 immmunoglobulin lambda light chain variable region (IGL) mRNA, partial cds
AGCTCTGCTTCAGCTGTGGGGCCACAGAAGGCAGGACTCGGTGAAGATCTCCACCATGGCCTGGTCCCCT
CTCCTCCTCACCCTCATCGCTCTCTGCACAGGATCCTGGGCCCAGTCTCTGACTCAGCCCGCCTCAGTGT
CTGGGACCCTGGGCCAGACAGTCACCATCTCCTGCTCTGGAAGCAGCTCCGACATCGGCCATAGTTATAC
TAGTGTGGGCTGGTACCAACAGATTCCAGGGACAGCCCCCAAAACCCTCATCTATGGTAATAGCAAACGA
GCCTCAGGGGTCCCAGATCGATTCTCTGGCTCCAAGTCTGGCAACACAGCCACCCTGACCATCTCTGGGC
TTCAGTCTGAGGACGAGGCCGATTATTACTGTGGTTCCTCACACAGCAGTGATACTAGTGTTGTATTCGG
CGGAGGCACCCACCTGACCATCGCAGGTGGTCCCACGTCTACACCCTCGGTCTCTCTCTTCCCGCCCTCC
TCTGAGGAGCTCAGCGCCAACAAGGCCACAGTGGTGTGTCTCATCAGCGACTTCTCCCCCAGCGGCCTGG
AGGTGATCTGGAAGGTAAATGACGCTGTCACCACCGACGGCGTCCAGACCACCAGGTCCTCGAAACAGAG
CAACGGCAAGTACGCGGCCAGCAGCTACCTGACGCGGACTTCCGCACAGTGGAAATCGTACAGCAGCGTC
AGCTGCCAGGTCAAGCACCAAGGGAAAACC
>KF985086.1 Equus caballus clone NeonateT04 immmunoglobulin lambda light chain variable region (IGL) mRNA, partial cds
AAGCTCTGCTTCAGCTGTGGGGCCACAGAAGGCAGGACTCGGTGAAGATCTCCACCATGGCCTGGTCCCC
TCTCCTCCTCACCCTCATCGCTCTCTGCACAGGATCCTGGGCCCAGTCTCTGACTCAGCCCGCCTCAGTG
TCTGGGACCCTGGGCCAGACAGTCACCATCTCCTGCTCTGGAAGCAGCTCCAACATCGGGTGGAGTGGTA
GTGCTGTGGGCTGGTACCAACAGATCCCAGGGACAGCCCCCAAAACCCTCATCTATGGTAATAACATGCG
AGCCTCAGGGGTCCCAGATCGATTCTCTGGCTCCAAGTCTGGCAACACAGCCACCCTGACCATCTCTGGG
CTTCAGGCTGAGGACGAGGCCGATTATTACTGTGGTTCCTCATACGGCAGTGATAGAAGTGCATTCGGCG
GAGGCACCCACCTGACCATCGCAGGTGGTCCCACGTCTACACCCTCGGTCTCTCTCTTCCCGCCCTCCTC
TGAGGAGCTCAGCGCCAACAAGGCCACAGTGGTGTGTCTCATCAGTGACTTCTCCCCCAGCGGCCTGGAG
GTGATCTGGAAGGTAAATGACGCTGTCACCACCGACGGCGTCCAGACCACCAGGTCCTCGAAACAGAGCA
ACGGCAAGTACGCGGCCAGCAGCTACCTGACGCGGACTTCCGCACAGTGGAAATCGTACAGCAGCGTCAG
CTGCCAGGTCAAGCACCAAGGGAAAACC
>KF985085.1 Equus caballus clone NeonateT34 immmunoglobulin lambda light chain variable region (IGL) mRNA, partial cds
TCTGCTTCAGCTGTGGGGCCACAGAAGGCAGGACTCGGTGACAATCTCCACCATGGCCTGGTGCCCTCTC
CTCCTCACCCTCATCGCTCTCTGCACAGGATCCTGGGCCCAGTCTGTGACTCAGCCCGCCTCAGTGTCTG
GGACCCTGGGCCAGACAGTCACCATCACCTGCACTGGAAGCAGCTCCAACATAGTTGCTTATGTGGGCTG
GTACCAACAGATCCCAGGAACAGCCCCCAAAACCCTCATCTACGTTAGTGACAAACGAGCCTCAGGGGTC
CCAGATCGATTCTCTGGCTCCAAGTCTGGCAGCACAGCCACCCTGACCATCACTGGGCTCCAGGCTGAGG
ACGAGGCCGATTATTACTGTGGTACCTCTGGCAGCAGTGGTAGTAGTGCTGTATTCGGCGGAGGCACCCA
CCTGACCATCGCAGGTGGTCCCACGTCTACACCCTCGGTCTCTCTCTTCCCGCCCTCCTCTGAGGAGCTC
AGCGCCAACAAGGCCACAGTGGTGTGTCTCATCAGCGACTTCTCCCCCAGCGGCCTGGAGGTGATCTGGA
AGGTAAATGACGCTGTCACCACCGACGGCGTCCAGACCACCAGGTCCTCGAAACAGAGCAACGGCAAGTA

CGCGGCCAGCAGCTACCTGACGCGGACTTCCGCACAGTGGAAATCGTACAGCAGCGTCAGCTGCCAGGTC
AAGCACCAAGGGAAAACC
>KF985084.1 Equus caballus clone NeonateT08 immmunoglobulin lambda light chain variable region (IGL) mRNA, partial cds
AGCTCTGCTTCAGCTGTGGGGCCACAGAAGGCAGGACTCGGTGACAATCTCCACCATGGCCTGGTGCCCT
CTCCTCCTCACCCTCATCGCTCTCTGCACAGGATCCTGGGCCCAGTCTGTGACTCAGCCCGCCTCAGTGT
CTGGGACCCTGGGCCAGACAGTCACCATCACCTGCACTGGAAGCAGCTCCAACATAGTTGCTTATGTGGG
CTGGTACCAACAGATCCCAGGAACAGCCCCCAAAACCCTCATCTACGCTAATAACAAACGAGCCTCAGGG
GTCCCAGATCGATTCTCTGGCTCCAAGTCTGGCAGCACAGCCACCCTGACCATCACTGGGCTCCAGGCTG
AGGACGAGGCCGATTATTACTGTGGTACCTCTAGCTTTAGTGGTAGTGATGTATTCGGCGGAGGCACCCA
CCTGACCATCGCAGGTGGTCCCACGTCTACACCCTCGGTCTCTCTCTTCCCGCCCTCCTCTGAGGAGCTC
AGCGCCAACAAGGCCACAGTGGTGTGTCTCATCAGCGACTTCTCCCCCAGCGGCCTGGAGGTGATCTGGA
AGGTAAATGACGCTGTCACCACCGACGGCGTCCAGACCACCAGGTCCTCGAAACAGAGCAACGGCAAGTA
CGCGGCCAGCAGCTACCTGACGCGGACTTCCGCACAGTGGAAATCGTACAGCAGCGTCAGCTGCCAGGTC
AAGCACCAAGGGAAAACC
>KF985083.1 Equus caballus clone NeonateT19 immmunoglobulin lambda light chain variable region (IGL) mRNA, partial cds
AGCTCTGCTTCAGCTGTGGGGCCACAGAAGGCAGGACTCGGTGAAGATCTCCACCATGGCCTGGTCCCCT
CTCCTCCTCACCCTCATCGCTCTCTGCACAGGATCCTGGGCCCAGTCTGTGACTCAGCCCGCCTCAGTGT
CTGGGACCCTGGGCCAGACAGTCACCATCACCTGCACTGGAAGCAGCTCCAACATAGTTGTTTATGTGGG
CTGGTACCAACAGATCCCAGGAACAGCCCCCGAAACCCTCATCTATAGTACTTACAAACGAGCCTCAGGG
GTCCCAGATCGATTCTCTGGCTCCAAGTCTGGCAGCACAGCCACCCTGACCATCACTGGGCTCCAGGCTG
AGGACGAGGCCGATTATTATTGTGGTACCTCTACCAGCAGTGGTAGTAGGGGTACATTCGGCGGAGGCAC
CCACCTGACCATCGCAGGTGGTCCCACGTCTACACCCTCGGTCTCTCTCTTCCCGCCCTCCTCTGAGGAG
CTCAGCGCCAACAAGGCCACAGTGGTGTGTCTCATCAGTGACTTCTCCCCCAGCGGCCTGGAGGTGATCT
GGAAGGTAAATGACGCTGTCACCACCGACGGCGTCCAGACCACCAGGTCCTCGAAACAGAGCAACGGCAA
GTACGCGGCCAGCAGCTACCTGACGCGGACTTCCGCACAGTGGAAATCGTACAGCAGCGTCAGCTGCCAG
GTCAAGCACCAAGGGAAAACC
>KF985082.1 Equus caballus clone NeonateT14 immmunoglobulin lambda light chain variable region (IGL) mRNA, partial cds
AGAAGGCAGGACTCGGTGAAGATCTCCACCATGGCCTGGTCCCCTCTCCTCCTCACCCTCATCGCTCTCT
GCACAGGATCCTGGGCCCAGTCTGTGACTCAGCCCGCCTCAGTGTCTGGGACCCTGGGCCAGACAGTCAC
CATCTCCTGCTCTGGAAGCAGCTCCAACATCGGGTATAACTATGATTATGTGGGCTGGTTCCAACAGATC
CCAGGAACAGCCCCCAAAACCCTCATCTGGGGTAATAACAAGCGAGCCTCAGGGGTCCCAGATCGATTCT
CTGGCTCCAAGTCTGGCAACACAGCCACCCTGACCATCTCTGGGGTCCAGGCTGAGGACGAGGCCGATTA
TTACTGTAGTTCCTACGACAGCAGCAGTAGTAGTACCGTATTCGGCGGAGGCACCCACCTGACCATCGCA
GGTGGTCCCACGTCTACACCCTCGGTCTCTCTCTTCCCGCCCTCCTCTGAGGAGCTCAGCGCCAACAAGG
CCACAGTGGTGTGTCTCATCAGCGACTTCTCCCCCAGCGGCCTGGAGGTGATCTGGAAGGTAAATGACGC
TGTCACCACCGACGGCGTCCAGACCACCAGGTCCTCGAAACAGAGCAACGGCAAGTACGCGGCCAGCAGC
TACCTGACGCGGACTTCCGCACAGTGGAAATCGTACAGCAGCGTCAGCTGCCAGGTCAAGCACCAAGGGA
AAACC
>KF985081.1 Equus caballus clone NeonateT29 immmunoglobulin lambda light chain variable region (IGL) mRNA, partial cds
GCTCTGCTTCAGCTGTGGGGCCACAGAAGGCAGGACTCGGTGAAGATCTCCACCATGGCCTGGTCCCCTC
TCCTCCTCACCCTCATCGCTTTCTGCACAGGATCCTGGGCCCAGTCTGTGACTCAGCCCGCCTCAGTGTC
TGGGACCCTGGGCCAGACAGTCACCATCTCCTGCTCTGGAAGCAGCTCCAACATCGGGTATAGTTATAGT
TATGTGGGCTGGTTCCAACAGATCCCAGGAACAGCCCCCAAAACCCTCATCTATGGTAGCAACAAACGAG
CCTCAGGGGTCCCAGATCGATTCTCTGGCTCCAAGTCTGGCAACACAGCCACCCTGACCATCTCTGGGGT
CCAGGCTGAGGACGAGGCCGATTATTACTGTGCTACTGATGACAGCAGCAGTAGTAGTGTTGTATTCGGC
GGAGGCACCCACCTGACCATCGCAGGTGGTCCCACGTCTACACCCTCGGTCTCTCTCTTCCCGCCCTCCT
CTGAGGAGCTCAGCGCCAACAAGGCCACAGTGGTGTGTCTCATCAGCGACTTCTCCCCCAGCGGCCTGGA
GGTGATCTGGAAGGTAAATGACGCTGTCACCACCGACGGCGTCCAGACCACCAGGTCCTCGAAACAGAGC
AACGGCAAGTACGCGGCCAGCAGCTACCTGACGCGGACTTCCGCACAGTGGAAATCGTACAGCAGCGTCA
GCTGCCAGGTCAAGCACCAAGGGAAAACC

>KF985080.1 Equus caballus clone NeonateT26 immmunoglobulin lambda light chain variable region (IGL) mRNA, partial cds
GCTCTGCTTCAGCTGTGGGGCCACAGAAGGCAGGACTCGGTGAAGATCTCCACCATGGCCTGGTCCCCTC
TCCTCCTCACCCTCATCGCTCTCTGCACAGGATCCTGGGCCCAGTCTGTGACTCAGCCCGCCTCAGTGTC
TGGGACCCTGGGCCAGACAGTCACCATCTCCTGCTCTGGAAGCAGCTCCAACATCGGGTATAGTTATAGT
GTTGTGGGCTGGTTCCAACAGATCCCAGGAACAGCCCCCAAAACCCTCATCTATGGTAATAACAAACGAG
CCTCAGGGGTCCCAGATCGATTCTCTGGCTCCAAGTCTGGCAACACAGCCACCCTGACCATCTCTGGGGT
CCAGGCTGAGGACGAGGCCGATTATTACTGTGGTTCCTATGACGGCAGCACTGGTAGTGCTGTATTCGGC
GGAGGCACCCACCTGACCATCGCAGGTGGTCCCACGTCTACACCCTCGGTCTCTCTCTTCCCGCCCTCCT
CTGAGGAGCTCAGCGCCAACAAGGCCACAGTGGTGTGTCTCATCAGCGACTTCTCCCCCAGCGGCCTGGA
GGTGATCTGGAAGGTAAATGACGCTGTCACCACCGACGGCGTCCAGACCACCAGGTCCTCGAAACAGAGC
AACGGCAAGTACGCGGCCAGCAGCTACCTGACGCGGACTTCCGCACAGTGGAAATCGTACAGCAGCGTCA
GCTGCCAGGTCAAGCACCAAGGGAAAACC
>KF985079.1 Equus caballus clone NeonateT20 immmunoglobulin lambda light chain variable region (IGL) mRNA, partial cds
GCTTCAGCTGTGGGGCCACAGAAGGCAGGACTCGGTGAAGATCTCCACCATGGCCTGGTCCCCTCTCCTC
CTCACCCTCATCGCTCTCTGCACAGGATCCTGGGCCCAGTCTGTGACTCAGCCCGCCTCAGTGTCTGGGA
CCCTGGGCCAGACAGTCACCATCTCCTGCTCTGGAAGCAGCTCCAACATCGGGTTGAGTAGTGGTAATGT
GGGCTGGTTCCAACAGATCCCAGGAACAGCCCCCAAAACCCTCATCTATGGTGCTAACAAACGAGCCTCA
GGGGTCCCAGATCGATTCTCTGGCTCCAAGTCTGGCAACACAGCCACCCTGACCATCTCTGGGGTCCAGG
CTGAGGACGAGGCCGATTATTACTGTGGTTCCTATGACAGCAGTAGTAGTAGTGCTGTATTCGGCGGAGG
CACCCACCTGACCATCGCAGGTGGTCCCACGTCTACACCCTCGGTCTCTCTCTTCCCGCCCTCCTCTGAG
GAGCTCAGCGCCAACAAGGCCACAGTGGTGTGTCTCATCAGCGACTTCTCCCCCAGCGGCCTGGAGGTGA
TCTGGAAGGTAAATGACGCTGTCACCACCGACGGCGTCCAGACCACCAGGTCCTCGAAACAGAGCAACGG
CAAGTACGCGGCCAGCAGCTACCTGACGCGGACTTCCGCACAGTGGAAATCGTACAGCAGCGTCAGCTGC
CAGGTCAAGCACCAAGGGAAAACC
>KF985078.1 Equus caballus clone NeonateT09 immmunoglobulin lambda light chain variable region (IGL) mRNA, partial cds
GCTCTGCTTCAGCTGTGGGGCCACAGAAGGCAGGACTCGGTGAAGATCTCCACCATGGCCTGGTCCCCTC
TCCTCCTCACCCTCATCGCTCTCTGCACAGGATCCTGGGCCCAGTCTGTGACTCAGCCCGCCTCAGTGTC
TGGGACCCTGGGCCAGACAGTCACCATCTCCTGCTCTGGAAGCAGCTCCAACGTCGGGAATAGTGCTAGT
TATGTGGGCTGGTTCCAACAGATCCCAGGAACAGCCCCCAAAACCCTCATCTATGGTAATAACAGACGAG
CCTCAGGGGTCCCAGATCGATTCTCTGCCTCCAAGTCTGGCAACACAGCCACCCTGACCATCTCTGGGGT
CCAGGCTGAGGACGAGGCCGATTATTACTGTGGTTCCACTGACAGCAGCAGTCATGGTGCATTCGGCGGA
GGCACCCACCTGACCATCGCAGGTGGTCCCACGTCTACACCCTCGGTCTCTCTCTTCCCGCCCTCCTCTG
AGGAGCTCAGCGCCAACAAGGCCACAGTGGTGTGTCTCATCAGTGACTTCTCCCCCAGCGGCCTGGAGGT
GATCTGGAAGGTAAATGACGCTGTCACCACCGACGGCGTCCAGACCACCAGGTCCTCGAAACAGAGCAAC
GGCAAGTACGCGGCCAGCAGCTACCTGACGCGGACTTCCGCACAGTGGAAATCGTACAGCAGCGTCAGCT
GCCAGGTCAAGCACCAAGGGAAAACC
>KF985076.1 Equus caballus clone NeonateT03 immmunoglobulin lambda light chain variable region (IGL) mRNA, partial cds
AGGGTTGGAGTCTCAGAAGACAGTGCTGTTGGGGAATCTCCACTATGGCCTGGGCTCTGCTCCTCATCAC
CCTCCTCACTCAGGGCACAGGGTCCTGGGCGCAGTCTGCCCTGACTCAGCCTGCGTCAGTCTCCGGGACT
CTGGGACAGTCGGTCACCATCACCTGTGCTGGAAGCACGGGATCTTATAAATATATTTCCTGGTACCAAC
AGCACCCAGGCACAGCCCCCAAACTCCTCATTTATAATGGGAATAATCGGGCCTCAGGGATCCCTGATCG
CTTCTCTGGCTCCACGTCTGGCAACACGATGTCCCTGACCATCTCTGGGCTCCAGGCTGAGGACGAGGCT
GATTATTACTGTCACGCGTATGTTGGCAGTTACAATTATATCTTCGGCGGCGGGACCCACCTCAGCGTCC
TGGGTGGTCCCACGTCTGCACCCTCGGTCTCTCTCTTCCCGCCCTCCTCTGAGGAGCTCAGCGCCAACAA
GGCCACAGTGGTGTGTCTCATCAGTGACTTCTCCCCCAGCGACTTGACGGTGAGCTGGAAGGTAAATGGC
GCCGCCATCAGCCAGGGAGTCCAGACCACCAAGCCCTCGAAACAGAGCAATGGCAAGTACGCGGCTAGCA
GCTACCTGACGCTGACCCCCGCCCAGTGGAAATCGTCCAGCAGCGTCAGCTGCCAGGTCACGCACCAAGG
GAAAACC
>KF985075.1 Equus caballus clone NeonateT13 immmunoglobulin lambda light chain variable region (IGL) mRNA, partial cds
TGGTCACAGAGGCAGAGCTCTGGGGCATCTCCACCATGGCCTGGACCCGTCTCCTGCTCCTCCTCCTCAC
TCTCTGCACAGGCTCTGTGGCTTCTTCTATGCTGACTCAGCCACTTACCGTGTCCGTGGCCTTTGGAAGC

ACAGTCACTATCACATGCCAGGGAGAGCTCCTAGACAGTTATTATGCTGAGTGGTACCAGCAGAAGCCAG
ACCAGGCTCCCGTGCTGGTCATATATTATGGAAGCAAACGTCCTTCGGGGATTTCTACCCGATTCTCTGG
CTCCTACTCAAGCAAGATGGCCACCCTGACCCTCAGTGGGGCCTGGGCCGAGGATGAGGCTGACTATTAC
TGTCAGGTGTGGGACAGCAGTGGTAACCAGCTTATCTTCGGCGGCGGGACCCACCTCAGCGTCCTGGGTG
GTCCCACGTCTGCACCCTCGGTCTCTCTCTTCCCGCCCTCCTCTGAGGAGCTCAGCGCCAACAAGGCCAC
AGTGGTGTGTCTCATCAGTGACTTCTCCCCCAGCGACTTGACGGTGAGCTGGAAGGTAAATGGCGCCGCC
ATCAGCCAGGGAGTCCAGACCACCAAGCCCTCGAAACAGAGCAATGGCAAGTACGCGGCTAGCAGCTACC
TGACGCTGACCCCCGCCCAGTGGAAATCGTCCAGCAGCGTCAGCTGCCAGGTCACGCACCAAGGGAAAAC
C
>KF985074.1 Equus caballus clone NeonateT22 immmunoglobulin lambda light chain variable region (IGL) mRNA, partial cds
AGGGCTTTGGGACATCTCCACCATGGCCTGGACCCCTCTCTTGTTAGCCTTCCTCACTCTCTGCACAGGT
CCCGTGGCCTCTTCTAAGCTGACTCAGCCATCTTCAGTGTCTGTGGCCTTGGGACAGACGGCCACCATCA
CCTGCAAGGGAGGCGACTTTGAAAGTTTTGTTGGTAGCTGGTACCAGCAGAAGCCAGGCCAGGCCCCTGT
GCTGGTCATCGATCCTAATAATGAGCGGCCCTCAGGGATCCCTGAACGATTCTCTGGCTCCAGCTCAGGA
GACACAGCCACGCTGACCATCAGCGGGGCCCAGGCTGAGGACGAGGCTGACTATTACTGTCTGGCAGAAG
ATGCTGATGATTATATCTTCGGCGGCGGGACCCACCTCAGCGTCCTGGGTGGTCCCACGTCTGCACCCTC
GGTCTCTCTCTTCCCGCCCTCCTCTGAGGAGCTCAGCGCCAACAAGGCCACAGTGGTGTGTCTCATCAGT
GACTTCTCCCCCAGCGACTTGACGGTGAGCTGGAAGGTAAATGGCGCCGCCATCAGCCAGGGAGTCCAGA
CCACCAAGCCCTCGAAACAGAGCAATGGCAAGTACGCGGCTAGCAGCTACCTGACGCTGACCCCCGCCCA
GTGGAAATCGTCCAGCAGCGTCAGCTGCCAGGTCACGCACCAAGGGAAAACC
>KF985073.1 Equus caballus clone NeonateT11 immmunoglobulin lambda light chain variable region (IGL) mRNA, partial cds
AGCTCTGCTTCAGCTGTGGGGCCACAGAAGGCAGGACTCGGTGACAATCTCCACCATGGCCTGGTGCCCT
CTCCTCCTCACCCTCATCGCTCTCTGCACAGGATCCTGGGCCCAGTCTGTGACTCAGCCCGCCTCAGTGT
CTGGGACCCTGGGCCAGGAAGTCACCATCACCTGCACTGTAAGCACGTCCAACATAGTTGCTTATGTGGG
CTGGTACCAACAGATCCCAGGAACAGCCCCCAAAACCCTCATCTACGCTACCAACAAACGAGCCTCAGGG
GTCCCAGATCGATTCTCTGGCTCCAAGTCTGGCAGCACAGCCACCCTGACCATCACTGGGCTCCAGGCTG
AGGACGAGGCCGATTATTACTGTGGTACCTCTTTTCTCAGTGTTATCTTCGGCGGCGGGACCCACCTCAG
CGTCCTGGGTGGTCCCACGTCTGCACCCTCGGTCTCTCTCTTCCCGCCCTCCTCTGAGGAGCTCAGCGCC
AACAAGGCCACAGTGGTGTGTCTCATCAGTGACTTCTCCCCCAGCGACTTGACGGTGAGCTGGAAGGTAA
ATGGCGCCGCCATCAGCCAGGGAGTCCAGACCACCAAGCCCTCGAAACAGAGCAATGGCAAGTACGCGGC
TAGCAGCTACCTGACGCTGACCCCCGCCCAGTGGAAATCGTCCAGCAGCGTCAGCTGCCAGGTCACGCAC
CAAGGGAAAACC
>KF985072.1 Equus caballus clone NeonateT27 immmunoglobulin lambda light chain variable region (IGL) mRNA, partial cds
AGCTCTGCTTCAGCTGTGGGGCCACAGAAGGCAGGACTCGGTGAAGATCTCCACCATGGCCTGGTCCCCT
CTCCTCCTCACCCTCATCGCTTTCTGCACAGGATCCTGGGCCCAGTCTGTGACTCAGCCCGCCTCAGTGT
CTGGGACCCTGGGCCAGACAGTCACCATCTCCTGCTCTGGAAGCAGCTCCAACATCGGATATAGTTATAG
TGTTGTGGGCTGGTTCCAACAGATCCCAGGAACAGCCCCCAAAACCCTCATCTATGGTAATAACAAACGA
GCCTCAGGGGTCCCAGATCGATTCTCTGGCTCCAAGTCTGGCAACACAGCCACCCTGACCATCTCTGGGG
TCCAGGCTGAGGACGAGGCCGATTATTACTGTGCTACTTATGACAGCAGCAGTAGTAGTGACATCTTCGG
CGGCGGGACCCACCTCAGCGTCCTGGGTGGTCCCACGTCTGCACCCTCGGTCTCTCTCTTCCCGCCCTCC
TCTGAGGAGCTCAGCGCCAACAAGGCCACAGTGGTGTGTCTCATCAGTGACTTCTCCCCCAGCGACTTGA
CGGTGAGCTGGAAGGTAAATGGCGCCGCCATCAGCCAGGGAGTCCAGACCACCAAGCCCTCGAAACAGAG
CAATGGCAAGTACGCGGCTAGCAGCTACCTGACGCTGACCCCCGCCCAGTGGAAATCGTCCAGCAGCGTC
AGCTGCCAGGTCACGCACCAAGGGAAAACC
>KF985071.1 Equus caballus clone NeonateT10 immmunoglobulin lambda light chain variable region (IGL) mRNA, partial cds
AGCTCTGCTTCAGCTGTGGGGCCACAGAAGGCAGGACTCGGTGAAGATCTCCACCATGGCCTGGTCCCCT
CTCCTCCTCACCCTCATCGCTTTCTGCACAGGATCCTGGGCCCAGTCTGTGACTCAGCCCGCCTCAGTGT
CTGGGACCCTGGGCCAGACAGTCACCATCTCCTGCTCTGGAAGCAGCTCCAACATCGGGTATAGTAGTAG
TTATGTGGGCTGGTTCCAACAGATCCCAGGAACAGCCCCCAAAACCCTCATCTATGGTAATAACAAACGA
GCCTCAGGGGTCCCAGATCGATTCTCTGGCTCCAAGTCTGGCAACACAGCCACCCTGACCATCTCTGGGG
TCCAGGCTGAGGACGAGGCCGACTATTACTGTGCTACTCATGACGTCAGCAGTAGTAGTGATATCTTCGG

CGGCGGGACCCACCTCAGCGTCCTGGGTGGTCCCACGTCTGCACCCTCGGTCTCTCTCTTCCCGCCCTCC
TCTGAGGAGCTCAGCGCCAACAAGGCCACAGTGGTGTGTCTCATCAGTGACTTCTCCCCCAGCGACTTGA
CGGTGAGCTGGAAGGTAAATGGCGCCGCCATCAGCCAGGGAGTCCAGACCACCAAGCCCTCGAAACAGAG
CAATGGCAAGTACGCGGCTAGCAGCTACCTGACGCTGACCCCCGCCCAGTGGAAATCGTCCAGCAGCGTC
AGCTGCCAGGTCACGCACCAAGGGAAAACC
>KF985070.1 Equus caballus clone FetusE06 immmunoglobulin lambda light chain variable region (IGL) mRNA, partial cds
ACAGAAGGCGGGACTCGGTGAAGATCTCCACCATGGCCTGGTCCCCTCTCCTCCTCACCCTCATCGCTCT
CTGCACAGGATCCTGGGCCCAGTCTCTGACCCAGCCCGCCTCAGTGTCTGGGACCCTGGGCCAGACAGTC
ACCATCTCCTGCTCTGGAAGCAGCTCCAACATCGGGTATAGTTATAGTGCTGTGGGCTGGTACCAACAGA
TCCCAGGAACAGCCCCCAAAACCCTCATCTATTATGCCACTAGTAGGGCTTCCGGGGTCCCCGACCGATT
CTCTGGCACCAGGTCTGGCAACACAGCCACCCTGACCATCTCTGGGCTCCAGGCTGAGGATGAGGCCGAT
TATTACTGTGGTACCTCTGGCAGCAGTTGGAGTAGTGGTGCATTCGGCGGAGGCACCCACCTGACCATCG
CAGGTGGTCCCACGTCTGCACCCTCGGTCTCTCTCTTCCCGCCCTCCTCTGAGGAGCTCAGCGCCAACAA
GGCCACAGTGGTGTGTCTCATCAGTGACTTCTCCCCCAGCGGCTTGGAGGTGATCTGGAAAGTAAATGAC
GCTGTCACCAACGACCGCGTCCAGACCACCAGGCCCTCGAAACAGAGCAACGGCAAGTACGCGGCCAGCA
GCTACCTGACACGGACCTCCACAGAGTGGAAATCGTACAGCAGCGTCAGCTGCCAGGTCAAGCACCAAGG
GAAAACC
>KF985069.1 Equus caballus clone FetusB02 immmunoglobulin lambda light chain variable region (IGL) mRNA, partial cds
GCTCTGCTTCAGCTGTGGGGACACAGAAGGCAGGACTCGGTGATGATCTCCACCATGGCCTGGTCCCCGC
TCCTTCTCACCTTCATCGCTCTCTGCACAGGATCCTGGGCCCAGTCTCTGACTCAGCCCGCCTCAGTGTC
TGGGACCCTGGGCCAGACAGTCACCATCTTCTGCTCTGGAAGCAGCTCCAACATTGGGTATAGTAGTAGT
TATGTGAGCTGGTTCCAACAGATCCCAGGAACAGCCCCCAAAACTCTCATCTATTATGCCACTAGTAGAG
CGTCCGGGGTCCCCGATCGATTCTCTGGCTCCAGGTCTGGCAACACAGCCACCCTGACCATCTCTGGGGT
CCAGGCTGAGGACGAGGCTGATTATTACTGCTCATCAGCAGACAGCAGCCTGAGGAGTGCTGTATTCGGC
GGAGGCACCCACCTGACCATCGCAGGTGGTCCCCTGTCTCCACCCTCGGTCTCTCTCTTCCCGCCCTCCT
CTGAGGAGCTCAGCGCCAACAAGGCCACAGTGGTGTGTCTCATCAGTGACTTCTCCCCCAGCGGCTTGGA
GGTGATCTGGAAGGTAAATGACGCTGTCACCAACGACCGCGTCCAGACCACCAGGCCCTCGAAACAGAGC
AACGGCAAGTACGCGGCCAGCAGCTACCTGACACGGACCTCCACAGAGTGGAAATCGTACAGCAGCGTCA
GCTGCCAGGTCACGCACCAAGGGAAAACC
>KF985068.1 Equus caballus clone FetusC08 immmunoglobulin lambda light chain variable region (IGL) mRNA, partial cds
GGGTTGGGGTCTCAGAAGGCAGTGCTCTTGGGGCGTCTCCACCATGGCCTGGACTCTGCTCCTTCTCACC
CTCCTCATTCAGGGTACAGGGTCCTGGGCCCAGTCTGCCCTGACTCAGCCTGCGTCAGTGTCCGGGGCTC
TAGGACAGTCGGTCACCATCACCTGTGCTGGAAGCAGCAGTGACATTGGGGGTTATAATGCTGTCAGCTG
GTTGCAACAGCACCCAGGCACAGCCCCCAAAGTTCTGATTTATAGTGTGAATACTCGGGCCTCAGGGATC
CCTGATCGCTTCTCTGGCTCCAAGTCTGGCAACACGGCCTCCCTGACCATCTCTGGGCTCCAGGTTGAGG
ACGAGGCTGATTATTACTGTTACTCGCTTGTGAGTGGTTACACTTATGGTGCATTCGGCGGAGGCACCCA
CCTGACCATCGCAGGTGGTCCCACGTCTGCACCCTCGGTCTCTCTCTTCCCGCCCTCCTCTGAGGAGCTC
AGCGCCAACAAGGCCACAGTGGTGTGTCTCATCAGTGACTTCTCCCCCAGCGGCTTGGAGGTGATCTGGA
AAGTAAATGACGCTGTCACCAACGACCGCGTCCAGACCACCAGGCCCTCGAAACAGAGCAACGGCAAGTA
CGCGGCCAGCAGCTACCTGACACGGACCTCCACAGAGTGGAAATCGTACAGCAGCGTCAGCTGCCAGGTC
AAGCACCAAGGGAAAACC
>KF985067.1 Equus caballus clone FetusB01 immmunoglobulin lambda light chain variable region (IGL) mRNA, partial cds
GCTTCAGCTGTGGGGCCACAGAAGGCAGGACTCGGTGACAATCTCCACCATGGCCTGGTCCCCTCTCCTC
CTCACCCTCATCGCTCTCTGCACAGGATCCTGGGCCCAGTCTGTGACTCAGCCCGCCTCAGTGTCTGGGA
CCCTGGGCCAGACAGTCACCATCTCCTGCTCTGGAAGCAGCTCCAACATCGGGTATAGTAGTAGTTATGG
GGGCTGGTACCAACAGATCCCAGGAACAGCCCCCAAACTCCTCATATATGAGGGTAACAAACGAGCCTCA
GGGGTCCCAGATCAATTCTCTGGCTCCAAGTCTGGCAACACAGCCACCCTGACCATCTCTGGGCTCCAGG
CTGAGGACGAGGCCGATTATTACTGTGTTTCCTATGACAGCAGCCTGAGTAGTGCTGTATTCGGCGGAGG
CACCCACCTGACCATCGCAGGTGGTCCCCTGTCTCCACCCTCGGTCTCTCTCTTCCCGCCCTCCTCTGAG
GAGCTCAGCGCCAACAAGGCCACAGTGGTGTGTCTCATCAGTGACTTCTCCCCCAGCGGCTTGGAGGTGA
TCTGGAAGGTAAATGACGCTGTCACCAACGACCGCGTCCAGACCACCAGGCCCTCGAAACAGAGCAACGG
CAAGTACGCGGCCAGCAGCTACCTGACACGGACCTCCACAGAGTGGAAATCGTACAGCAGCGTCAGCTGC

CAGGTCACGCACCAAGGGAAAACC
>KF985066.1 Equus caballus clone FetusC01 immmunoglobulin lambda light chain variable region (IGL) mRNA, partial cds
GGAAGCTCTGCTTCAGCTGTGGGGCCACAGAAGGCAGGACTCGGTGAAGATCTCCACCATGGCCTGGTCC
CCTCTCCTCCTCACCCTCATCGCTCTCTGCACAGGATCCTGGGCCCAGTCTCTGACTCAGCCCGCCTCAG
TGTCTGGGACCCTGGGCCAGACAGTCACCATCTCCTGCTCTGGAAGCAGCTCCAACATCGGGTATAGTTA
TAGTGCTGTGGGCTGGTACCAACAGATCCCAGGAACAGCCCCCAAAACCCTCATCTATGGTAATAACAAA
CGAGCCTCAGGGGTCCCAGATCGATTCTCTGGCTCCAAGTCTGGCAACACAGCCACCCTGACCATCTCTG
GGGTCCAGGCTGAGGACGAGGCCGATTATTACTGCTCAGCAGGAGACAGCAGTGGTAGTAGTGGTGCATT
CGGCGGAGGCACCCACCTGACCATCGCAGGTGGTCCCACGTCTGCACCCTCGGTCTCTCTCTTCCCGCCC
TCCTCTGAGGAGCTCAGCGCCAACAAGGCCACAGTGGTGTGTCTCATCAGTGACTTCTCCCCCAGCGGCT
TGGAGGTGATCTGGAAAGTAAATGACGCTGTCACCAACGACCGCGTCCAGACCACCAGGCCCTCGAAACA
GAGCAACGGCAAGTACGCGGCCAGCAGCTACCTGACACGGACCTCCACAGAGTGGAAATCGTACAGCAGC
GTCAGCTGCCAGGTCAAGCACCAAGGGAAAACC
>KF985065.1 Equus caballus clone FetusD08 immmunoglobulin lambda light chain variable region (IGL) mRNA, partial cds
CTCTGCTTCAGCTGTGGGGCCACAGAAGGCGGGACTCGGTGAAGATCTCCACCATGGCCTGGTCCCCTCT
CCTCCTCACCCTCATCGCTCTCTGCACAGGATCCTGGGCCCAGTCTCTGACCCAGCCCGCCTCAGTGTCT
GGGACCCTGGGCCAGACAGTCACCATCTCCTGCTCTGGAAGCAGCTCCAACATCGGGTATAGTTATAGTG
CTGTGGGCTGGTACCAACAGATCCCAGGAACAGCCCCCAAAACCCTCATCTATGGTAATAACAAACGAGC
CTCAGGGGTCCCAGATCGATTCTCTGGCTCCAAGTCTGGCAACACAGCCACCCTGACCATCTCTGGGCTT
CAGGCTGAGGACGAGGCCGATTATTACTGTGGTTCCTATTACAGCAGTGATAGTAGTGATGCTGTATTCG
GCGGAGGCACCCACCTGACCATCGCAGGTGGTCCCACGTCTGCACCCTCGGTCTCTCTCTTCCCGCCCTC
CTCTGAGGAGCTCAGCGCCAACAAGGCCACAGTGGTGTGTCTCATCAGTGACTTCTCCCCCAGCGGCTTG
GAGGTGATCTGGAAAGTAAATGACGCTGTCACCAACGACCGCGTCCAGACCACCAGGCCCTCGAAACAGA
GCAACGGCAAGTACGCGGCCAGCAGCTACCTGACACGGACCTCCACAGAGTGGAAATCGTACAGCAGCGT
CAGCTGCCAGGTCAAGCACCAAGGGAAAACC
>KF985064.1 Equus caballus clone FetusE10 immmunoglobulin lambda light chain variable region (IGL) mRNA, partial cds
AGCTCTGCTTCAGCTGTGGGGCCACAGAAGGCAGGACTCGGTGAAGATCTCCACCATGGCCTGGTCCCCT
CTCCTCCTCACCCTCATCGCTCTCTGCACAGGATCCTGGGCCCAGTCTCTGACTCAGCCCGCCTCAGTGT
CTGGGACCCTGGGCCAGACAGTCACCATCTCCTGCTCTGGAAGCAGCTCCAACATCGGGTATAGTTATAG
TGCTGTGGGCTGGTACCAACAGATCCCAGGGACAGCCCCCAAAACCCTCATCTATGCTACTAACAAACGA
GCCTCAGGGGTCCCAGATCGATTCTCTGGCTCCAAGTCTGGCAACACAGCCACCCTGACCATCTCTGGGC
TTCAGGCTGAGGACGAGGCCGATTATTACTGTGGTTCCTCATACAGCAGTGATAGTAGTGATGGTGCATT
CGGCGGAGGCACCCACCTGACCATCGCAGGTGGTCCCACGTCTGCACCCTCGGTCTCTCTCTTCCCGCCC
TCCTCTGAGGAGCTCAGCGCCAACAAGGCCACAGTGGTGTGTCTCATCAGTGACTTCTCCCCCAGCGGCT
TGGAGGTGATCTGGAAAGTAAATGACGCTGTCACCAACGACCGCGTCCAGACCACCAGGCCCTCGAAACA
GAGCAACGGCAAGTACGCGGCCAGCAGCTACCTGACACGGACCTCCACAGAGTGGAAATCGTACAGCAGC
GTCAGCTGCCAGGTCAAGCACCAAGGGAAAACC
>KF985063.1 Equus caballus clone FetusB08 immmunoglobulin lambda light chain variable region (IGL) mRNA, partial cds
AGCTCTGCTTCGGCTGTGGGGCCACAGAAGGCAGGACTCGGTGAAGATCTCCACCATGGCCTGGTCCCCT
CTCCTCCTCACCCTCATCGCTCTCTGCACAGGATCCTGGGCCCAGTCTCTGACCCAGCCCGCCTCAGTGT
CTGGGACCCTGGGCCAGACAGTCACCATCTCCTGCTCTGGAAGCAGCTCCAACATCGGGTATAGTTATAG
TGCTGTGGGCTGGTACCAACAGATCCCAGGAACAGCCCCCAAAACCCTCATCTATGGTAATAACAAACGA
GCCTCAGGGGTCCCAGATCGATTCTCTGGCTCCAAGTCTGGCAACACAGCCACCCTGACCATCTCTGGGC
TTCAGGCTGAGGACGAGGCCGATTATTACTGTGGTTCCTATTACAGCAGTGATAGTAGTGATGCTGTATT
CGGCGGAGGCACCCACCTGACCATCGCAGGTGGTCCCCTGTCTCCACCCTCGGTCTCTCTCTTCCCGCCC
TCCTCTGAGGAGCTCAGCGCCAACAAGGCCACAGTGGTGTGTCTCATCAGTGACTTCTCCCCCAGCGGCT
TGGAGGTGATCTGGAAGGTAAATGACGCTGTCACCAACGACCGCGTCCAGACCACCAGGCCCTCGAAACA
GAGCAACGGCAAGTACGCGGCCAGCAGCTACCTGACACGGACCTCCACAGAGTGGAAATCGTACAGCAGC
GTCAGCTGCCAGGTCACGCACCAAGGGAAAA
>KF985062.1 Equus caballus clone FetusB04 immmunoglobulin lambda light chain variable region (IGL) mRNA, partial cds

TCTGCTTCAGCTGTGGGGCCACAGAAGGCAGGACTCGGTGAAGATCTCCACCATGGCCTGGTCCCCTCTC
CTCCTCACCCTCATCGCTTTCTGCACAGGATCCTGGGCCCAGTCTGTGACTCAGCCCGCCTCAGTGTCTG
GGACCCTGGGCCAGACAGTCACCATCTCCTGCTCTGGAAGCAGCTCCAACATCGGGTATAGTTATAGTGC
TGTGGGCTGGTACCAACAGATCCCAGGAACAGCCCCCAAAACCCTCATCTATGGTAATAACAAACGAGCC
TCAGGGGTCCCAGATCGATTCTCTGGCTCCAAGTCTGGCAACACAGCCACCCTGACCATCTCTGGGCTTC
AGGCTGAGGACGAGGCCGATTATTACTGTGGTTCCTATTACAGCAGTGATAGTAGTGCATTCGGCGGAGG
CACCCACCTGACCATCGCAGGTGGTCCCACGTCTGCACCCTCGGTCTCTCTCTTCCCGCCCTCCTCTGAG
GAGCTCAGCGCCAACAAGGCCACAGTGGTGTGTCTCATCAGTGACTTCTCCCCCAGCGGCTTGGAGGTGA
TCTGGAAAGTAAATGACGCTGTCACCAACGACCGCGTCCAGACCACCAGGCCCTCGAAACAGAGCAACGG
CAAGTACGCGGCCAGCAGCTACCTGACACGGACCTCCACAGAGTGGAAATCGTACAGCAGCGTCAGCTGC
CAGGTCAAGCACCAAGGGAAAACC
>KF985060.1 Equus caballus clone FetusD03 immmunoglobulin lambda light chain variable region (IGL) mRNA, partial cds
GCTCTGCTTCAGCTGTGGGGCCACAGAAGGCAGGACTCGGTGACAATCTCCACCATGGCCTGGTGCCCTC
TCCTCCTCACCCTCATCGCTCTCTGCACAGGATCCTGGGCCCAGTCTGTGACTCAGCCCGCCTCAGTGTC
TGGGACCCTGGGCCAGACAGTCACCATCACCTGCACTGGAAGCAGCTCCAACATAGTTGCTTATGTGGGC
TGGTACCAACAGATCCCAGGAACAGCCCCCAAAACCCTCATCTACGCTAATAACAAACGAGCCTCAGGGG
TCCCAGATCGATTCTCTGGCTCCAAGTCTGGCAGCACAGCCACCCTGACCATCACTGGGCTCCAGGCTGA
GGACGAGGCCGATTATTACTGTGGTACCTCTAGCAGCAGTGGTAGTAGTGCTGTATTCGGCGGAGGCACC
CACCTGACCATCGCAGGTGGTCCCCTGTCTCCACCCTCGGTCTCTCTCTTCCCGCCCTCCTCTGAGGAGC
TCAGCGCCAACAAGGCCACAGTGGTGTGTCTCATCAGTGACTTCTCCCCCAGCGGCTTGGAGGTGATCTG
GAAGGTAAATGACGCTGTCACCAACGACCGCGTCCAGACCACCAGGCCCTCGAAACAGAGCAACGGCAAG
TACGCGGCCAGCAGCTACCTGACACGGACCTCCACAGAGTGGAAATCGTACAGCAGCGTCAGCTGCCAGG
TCACGCACCAAGGGAAAACC
>KF985059.1 Equus caballus clone FetusC11 immmunoglobulin lambda light chain variable region (IGL) mRNA, partial cds
AGCTCTGCTTCAGCTGTGGGGCCACAGAAGGCAGGACTCGGTGACAATCTCCACCATGGCCTGGTGCCCT
CTCCTCCTCACCCTCATCGCTCTCTGCACAGGATCCTGGGCCCAGTCTGTGACTCAGCCCGCCTCAGTGT
CTGGGACCCTGGGCCAGACAGTCACCATCACCTGCACTGGAAGCAGCTCCAACATAGTTGCTTATGTGGG
CTGGTACCAACAGATCCCAGGAACAGCCCCCAAAACCCTCATCTACGCTAATAACAAACGAGCCTCAGGG
GTCCCAGATCGATTCTCTGGCTCCAAGTCTGGCAGCACAGCCACCCTGACCATCACTGGGCTCCAGGCTG
AGGACGAGGCCGATTATTACTGTGGTACCTCTAGCAGCAGTGGTAGTAGTGATCCTGTATTCGGCGGAGG
CACCCACCTGACCATCGCAGGTGGTCCCACGTCTGCACCCTCGGTCTCTCTCTTCCCGCCCTCCTCTGAG
GAGCTCAGCGCCAACAAGGCCACAGTGGTGTGTCTCATCAGTGACTTCTCCCCCAGCGGCTTGGAGGTGA
TCTGGAAAGTAAATGACGCTGTCACCAACGACCGCGTCCAGACCACCAGGCCCTCGAAACAGAGCAACGG
CAAGTACGCGGCCAGCAGCTACCTGACACGGACCTCCACAGAGTGGAAATCGTACAGCAGCGTCAGCTGC
CAGGTCAAGCACCAAGGGAAAACC
>KF985058.1 Equus caballus clone FetusE08 immmunoglobulin lambda light chain variable region (IGL) mRNA, partial cds
CTCTGCTTCAGCTGTGGGGCCACAGAAGGCAGGACTCGGTGACAATCTCCACCATGGCCTGGTGCCCTCT
CCTCCTCACCCTCATCGCTCTCTGCACAGGATCCTGGGCCCAGTCTGTGACTCAGCCCGCCTCAGTGTCT
GGGACCCTGGGCCAGACAGTCACCATCACCTGCACTGGAAGCAGCTCCAACATCGGGAATAGTTATAGTT
ATGTGGGCTGGTTCCAACAGATCCCAGGAACAGCCCCCAAAACCCTCATCTATGGTAATAATAAACGAGC
CTCAGGGGTCCCAGATCGATTCTCTGGCTCCAAGTCTGGCAACACAGCCACCCTGACCATCTCTGGGGTC
CAGGCTGAGGACGAGGCCGATTATTACTGTGGTTCCTATGACAGCAGCAGTAGTAGTGCATTCGGCGGAG
GCACCCACCTGACCATCGCAGGTGGTCCCACGTCTGCACCCTCGGTCTCTCTCTTCCCGCCCTCCTCTGA
GGAGCTCAGCGCCAACAAGGCCACAGTGGTGTGTCTCATCAGTGACTTCTCCCCCAGCGGCTTGGAGGTG
ATCTGGAAGGTAAATGACGCTGTCACCAACGACCGCGTCCAGACCACCAGGCCCTCGAAACAGAGCAACG
GCAAGTACGCGGCCAGCAGCTACCTGACACGGACCTCCACAGAGTGGAAATCGTACAGCAGCGTCAGCTG
CCAGGTCACGCACCAAGGGAAAACC
>KF985057.1 Equus caballus clone FetusC09 immmunoglobulin lambda light chain variable region (IGL) mRNA, partial cds
AGCCCAGGAGCCTGAGGAGGTGACATCTGGGAGTGTCCCCACCATGGCCTGGACAGTGCTTCTTCTCTGG
CTCCTCACTTACAGCTCAGGGGCAGATTCTCAGGCTGTGGTGATCCAGGAGCCATCATTCTCTGTGTCCC
TAGGGGGGACGGTCATACTGACCTGTGGCCTTAGAACTGGGTCAGTCTCTACCAGTAACTATCCTAGATG
GTACCAGCAGACACCAGGCAAGGCTCCCCGTACACTCACCTACAGCACAAACAACCGCCCCTCTGGGATC

CCTGAACGCTTCTCTGGATCCATCTCAGGAAACAAAGCCGCCCTCACCATCACGGGGGCCCAGCCCGAGG
ACGAGGCCGACTATTACTGTGATCTGTATGTGGATCGTGGTGTTCATGGTGCATTCGGCGGAGGCACCCA
CCTGACCATCGCAGGTGGTCCCACGTCTGCACCCTCGGTCTCTCTCTTCCCGCCCTCCTCTGAGGAGCTC
AGCGCCAACAAGGCCACAGTGGTGTGTCTCATCAGTGACTTCTCCCCCAGCGACTTGACGGTGAGCTGGA
AGGTAAATGGCGCCGCCACCACCCAGGGCGTCCAGACCACCAGGCCCTCGAAACAGAGCAACGGCAAGTA
CGCGGCCAGCAGCTACCTGCCGCTGACCCCCACCCAGTGGAAATCGTCCAGCAGCGTCAGCTGCCAGGTC
ACGCACCAAGGGAAAACC
>KF985056.1 Equus caballus clone FetusA12 immmunoglobulin lambda light chain variable region (IGL) mRNA, partial cds
GTTGGAGGCTCAGAAGGCAGTGCTCTTGGGGCGTCTCCACTATGGCCTGGGCTCTGTTCCTCATCACCCT
CCTCACTCAGGGTACAGGGTCCTGGGGCCAGTCTGCCCTGATTCAGCCTTCTTCGGTGTCCGTGGCTCTA
GGACAGTCGGTCACCATCTCCTGTGCTGGAAGCAGCAGTGACATTGGGTATTATAACTCTATTTCCTGGT
ACCAACAGCACCCAGGCACAACCCCAAAGCTGCTGATTTACTATACCAATAAGAAGCACTCAGGGATCCC
TGATCGCTTCTCTGGCTCCAAGTCTGGGAACACGGCCTCCCTGACCATCTCTGGGCTCCAGGCTGAGGAT
GAGGCTGAGTATTACTGTTGCTCATATGCAGGCAGTGGCAATTTTGCATTCGGCGGAGGCACCCACCTGA
CCATCGCAGGTGGTCCCACGTCTGCACCCTCGGTCTCTCTCTTCCCGCCCTCCTCTGAGGAGCTCAGCGC
CAACAAGGCCACAGTGGTGTGTCTCATCAGTGACTTCTCCCCCAGCGACTTGACGGTGAGCTGGAAGGTA
AATGGCGCCGCCACCACCCAGGGCGTCCAGACCACCAGGCCCTCGAAACAGAGCAACGGCAAGTACGCGG
CCAGCAGCTACCTGCCGCTGACCCCCACCCAGTGGAAATCGTCCAGCAGCGTCAGCTGCCAGGTCACGCA
CCAAGGGAAAACC
>KF985055.1 Equus caballus clone FetusB10 immmunoglobulin lambda light chain variable region (IGL) mRNA, partial cds
TAGGCTCAGAGGCAGAAGTCTGGGGAATCTCCACCATGGCCTGGACCCCTCTCTTGTTAGCCTTCCTCTC
TCTCTGCACAGGTCCTGTTGTCTCTTCTGCAGTGACTCAGCCATCTGAGGTGTCCGTGGCCTTGGGACAG
AGAGCCACCCTCACCTGCCAGGGAAGCAACTTTGAATTTTTTTCTCCTAGCTGGTACCAGCAGAAGCCAG
GCCAGGCCCCTGTACTGCTCATCAATATTAATAATGAGCGCCACTCAGGGATCCCTGAACGATTCTCCGG
CTCCAGCTCAGGAGACACGTCCACACTGACCATCAGTGGGGCCCAGGCTGAGGACGAGGCTGACTATTAC
TGTCTGGCAGTAGATGCTCTTAGTTCTGCTGTATTCGGCGGAGGCACCCACCTGACCATCGCAGGTGGTC
CCCTGTCTCCACCCTCGGTCTCTCTCTTCCCGCCCTCCTCTGAGGAGCTCAGCGCCAACAAGGCCACAGT
GGTGTGTCTCATCAGTGACTTCTCCCCCAGCGACTTGACGGTGAGCTGGAAGGTAAATGGCGCCGCCATC
ACCCAGGGCGTCCAGACCACCAGGCCCTCGAAACAGAGCAACGGCAAGTACGCGGCCAGCAGCTACCTGC
CGCTGACCCCCACCCAGTGGAAATCGTCCAGCAGCGTCAGCTGCCAGGTCACGCACCAAGGGAAAACC
>KF985054.1 Equus caballus clone FetusA10 immmunoglobulin lambda light chain variable region (IGL) mRNA, partial cds
AGAGGCAGGGCTTTGGGACATCTCCACCATGGCCTGGACCCCTCTCTTGTTAGCCTTCCTCACTCTCTGC
ACAGGTCCCGTGGCCTCTTCTAAGCTGACTCAGCCATCTTCAGTGTCTGTGGCCTTGGGACAGACGGCCA
CCATCACCTGCAAGGGAGGCAACTTTGAAAGTTTTGTTGGTAGCTGGTACCAGCAGAAGCCAGGCCAGGC
CCCTGTGCTGGTCATCGATCCTAGTAATGAGCGGCCCTCAGGGATCCCTGAACGATTCTCTGGCTCCAGC
TCAGGAGACACAGCCACGCTGACCATCAGCGGGGCCCAGGCTGAGGACGAGGCTGACTATTACTGTCTGG
CAGCAGATGCTTCTGATTATGCATTCGGCGGAGGCACCCACCTGACCATCGCAGGTGGTCCCACGTCTGC
ACCCTCGGTCTCTCTCTTCCCGCCCTCCTCTGAGGAGCTCAGCACCAACAAGGCCACAGTGGTGTGTCTC
ATCAGTGACTTCTCCCCCAGCGACTTGACGGTGAGCTGGAAGGTAAATGGCGCCGCCATCACCCAGGGCG
TCCAGACCACCAGGCCCTCGAAACAGAGCAACGGCAAGTACGCGGCCAGCAGCTACCTGCCGCTGACCCC
CACCCAGTGGAAATCGTCCAGCAGCGTCAGCTGCCAGGTCACGCACCAAGGGAAAACC
>KF985053.1 Equus caballus clone FetusA04 immmunoglobulin lambda light chain variable region (IGL) mRNA, partial cds
CTTCAGCTGTGGGGCCACAGAAGGCAGGACTCGGTGAAGATCTCCACCATGGCCTGGTCCCCTCTCCTCC
TCACCCTCATCGCTCTCTGCACAGGATCCTGGGCCCAGTCTCTGACTCAGCCCGCCTCAGTGTCTGGGAC
CCTGGGCCAGACAGTCACCATCTCCTGCTCTGGAAGCAGCTCCAACATCGGGTATAGTTATAGTGCTGTG
GGCTGGTACCAACAGATCCCAGGAACAGCCCCCAAAACCCTCATCTATGGTAATAACAAACGAGCCTCAG
GGGTCCCAGATCGATTCTCTGGCTCCAAGTCTGGCAACACAGCCACCCTGACCATCTCTGGGGTCCAGGC
TGAGGACGAGGCCGATTATTACTGCTCAGCAGGAGACAGCAGTGGTAGTAGTGATGGTGCATTCGGCGGA
GGCACCCACCTGACCATCGCAGGTGGTCCCACGTCTGCACCCTCGGTCTCTCTCTTCCCGCCCTCCTCTG
AGGAGCTCAGCGCCAACAAGGCCACAGTGGTGTGTCTCATCAGTGACTTCTCCCCCAGCGACTTGACGGT
GAGCTGGAAGGTAAATGGCGCCGCCATCACCCAGGGCGTCCAGACCACCAGGCCCTCGAAACAGAGCAAC

GGCAAGTACGCGGCCAGCAGCTACCTGCCGCTGACCCCCACCCAGTGGAAATCGTCCAGCAGCGTCAGCT
GCCAGGTCACGCACCAAGGGAAAACC
>KF985052.1 Equus caballus clone FetusD04 immmunoglobulin lambda light chain variable region (IGL) mRNA, partial cds
AGCTCTGCTTCAGCTGTGGGGCCACAGAAGGCAGGACTCGGTGAAGATCTCCACCATGGCCTGGTCCCCT
CTCCTCCTCACCCTCATCGCTCTCTGCACAGGATCCTGGGCCCAGTCTCTGACTCAGCCCGCCTCAGTGT
CTGGGACCCTGGGCCAGACAGTCACCATCTCCTGCTCTGGAAGCAGCTCCAACATCGGGTATAGTTATAG
TGCTGTGGGCTGGTACCAACAGATCCCAGGGACAGCCCCCAAAACCCTCATCTATGCTACTAACAAACGA
GCCTCAGGGGTCCCAGATCGATTCTCTGGCTCCAAGTCTGGCAACACAGCCACCCTGACCATCTCTGGGC
TTCAGGCTGAGGACGAGGCCGATTATTACTGTGGTTCCTCATACAGCAGTGATAGTGGTGCATTCGGCGG
AGGCACCCACCTGACCATCGCAGGTGGTCCCACGTCTGCACCCTCGGTCTCTCTCTTCCCGCCCTCCTCT
GAGGAGCTCAGCGCCAACAAGGCCACAGTGGTGTGTCTCATCAGTGACTTCTCCCCCAGCGACTTGACGG
TGAGCTGGAAGGTAAATGGCGCCGCCACCACCCAGGGCGTCCAGACCACCAGGCCCTCGAAACAGAGCAA
CGGCAAGTACGCGGCCAGCAGCTACCTGCCGCTGACCCCCACCCAGTGGAAATCGTCCAGCAGCGTCAGC
TGCCAGGTCACGCACCAAGGGAAAACC
>KF985051.1 Equus caballus clone FetusE01 immmunoglobulin lambda light chain variable region (IGL) mRNA, partial cds
AGCTCTGCTTCAGCTGTGGGGCCACAGAAGGCAGGACTCGGTGACAATCTCCACCATGGCCTGGTGCCCT
CTCCTCCTCACCCTCATCGCTCTCTGCACAGGATCCTGGGCCCAGTCTGTGACTCAGCCCGCCTCAGTGT
CTGGGACCCTGGGCCAGACAGTCACCATCACCTGCACTGGAAGCAGCTCCAACATAGTTGCTTATGTGGG
CTGGTACCAACAGATCCCAGGAACAGCCCCCAAAACCCTCATCTACGCTAATAACAAACGAGCCTCAGGG
GTCCCAGATCGATTCTCTGGCTCCAAGTCTGGCAGCACAGCCACCCTGACCATCACTGGGCTCCAGGCTG
AGGACGAGGCCGATTATTACTGTGGTACCTCTAGCAGCAGTGGTAGTGCATTCGGCGGAGGCACCCACCT
GACCATCGCAGGTGGTCCCACGTCTGCACCCTCGGTCTCTCTCTTCCCGCCCTCCTCTGAGGAGCTCAGC
GCCAACAAGGCCACAGTGGTGTGTCTCATCAGTGACTTCTCCCCCAGCGACTTGACGGTGAGCTGGAAGG
TAAATGGCGCCGCCACCACCCAGGGCGTCCAGACCACCAGGCCCTCGAAACAGAGCAACGGCAAGTACGC
GGCCAGCAGCTACCTGCCGCTGACCCCCACCCAGTGGAAATCGTCCAGCAGCGTCAGCTGCCAGGTCACG
CACCAAGGGAAAACC
>KF985050.1 Equus caballus clone FetusB12 immmunoglobulin lambda light chain variable region (IGL) mRNA, partial cds
AGGGTTGGGGGCTCAGAAGGCAGTGCTCTTGGGGCGTCTCCACTATGGCCTGGGCTCTGTTCCTCATCAC
CCTCCTCACTCAGGGTACAGGGTCCTGGGGCCAGTCTGCCCTGATTCAGCCTTCTTCGGTGTCCGTGGCT
CTAGGACAGTCGGTCACCATCTCCTGTGCTGGAAGCAGCAGTGACATTGGGTATTATAACTCTATTTCCT
GGTACCAACAGCACCCAGGCACAACCCCAAAGCTGCTGATTTACTATACCAATAAGAAGCACTCAGGGAT
CCCTGATCGCTTCTCTGGCTCCAAGTCTGGGAACACGGCCTCCCTGACCATCTCTGGGCTCCAGGCTGAG
GATGAGGCTGAGTATTACTGTTGCTCATATGCAGGCAGTGGCAATTATATCTTCGGCAGCGGGACCCACC
TCAGCGTCCTGGGTGGTCCCCCGTCTGCACCCTCGGTCTCTCTCTTCCCGCCCTCCTCTGAGGAGCTCAG
CACCAACAAGGCCACAGTGGTGTGTCTCATCAGTGACTTCTCCCCCAGCGACTTGACGGTGAGCTGGAAG
GGAAATGGCGCCGCCATCAGCCAGGGCGTCCAGACCACCAAGCCCTCGAAACAGAGCAATGGCAAGTACG
CGGCTAGCAGCTACCTGACGCTGACCCCCGCCCAGTGGAAATCGTACAGCAGCGTCAGCTGCCAGGTCAC
GCACCAAGGGAAAACC
>KF985049.1 Equus caballus clone FetusA11 immmunoglobulin lambda light chain variable region (IGL) mRNA, partial cds
GTAGGCTCAGAGGCAGAAGTCTGGGGAATCTCCACCATGGCCTGGACCCCTCTCTTGTTAGCCTTCCTCT
CTCTCTGCACAGGTCCTGTTGTCTCTTCTGCAGTGACTCAGCCATCTGAGGTGTCCGTGGCCTTGGGACA
GAGAGCCACCCTCACCTGCCAGGGAAGCAACTTTGAATTTTTTTCTCCTAGCTGGTACCAGCAGAAGCCA
GGCCAGGCCCCTGTACTGCTCATCAATATTAATAATGAGCGCCACTCAGGGATCCCTGAACGATTCTCCG
GCTCCAGCTCAGGAGACACGTCCACACTGACCATCAGTGGGGCCCAGGCTGAGGACGAGGCTGACTATTA
CTGTCTGGCAGTAGATGCTCTTAGTTCTGAAACGTATATCTTCGGCGGCGGGACCCACCTCAGCGTCCTG
GGTGGTCCCCCGTCTGCACCCTCGGTCTCTCTCTTCCCGCCCTCCTCTGAGGAGCTCAGCACCAACAAGG
CCACAGTGGTGTGTCTCATCAGTGACTTCTCCCCCAGCGACTTGACGGTGAGCTGGAAGGGAAATGGCGC
CGCCATCAGCCAGGGCGTCCAGACCACCAAGCCCTCGAAACAGAGCAATGGCAAGTACGCGGCTAGCAGC
TACCTGACGCTGACCCCCGCCCAGTGGAAATCGTACAGCAGCGTCAGCTGCCAGGTCACGCACCAAGGGA
AAACC
>KF985048.1 Equus caballus clone FetusC03 immmunoglobulin lambda light chain variable region (IGL) mRNA, partial cds

GGAGCTAGATCTCTAGGCACCTCCACCATGGCCTGGACCCTTCTCCTGCTTCCCCTCCTCACTCTCTGCA
CAGGTTCTGTGACCGCCTATGACTTGACGCAACCACACTCAACTTCGGTGGCCCTAGGACAGACAGCGAC
AATCACCTGCTCTGGAGATAATCTCGAGGATGAATATGCTTACTGGTACCAGCAGAAGACAGGCCAGTCC
CCTGCCCTGGTCATTTATAAGGATAGTGAGCACCCCTCAGGGATCCCTGACCGGTTCTCTGGCTCAAACT
CAGGAAACACAGCCACGCTGACCATCAGAGGGGCCAAGACAGAGGACAAGGCTGACTATTACTGCCAATC
GTGGAGCAGTGCTAATGCTAGTATCTTCGGCGGCGGGACCCACCTCAGCGTCCTGGGTGGTCCCCCGTCT
GCACCCTCGGTCTCTCTCTTCCCGCCCTCCTCTGAGGAGCTCAGCACCAACAAGGCCACAGTGGTGTGTC
TCATCAGTGACTTCTCCCCCAGCGACTTGACGGTGAGCTGGAAGGGAAATGGCGCCGCCATCAGCCAGGG
CGTCCAGACCACCAAGCCCTCGAAACAGAGCAATGGCAAGTACGCGGCTAGCAGCTACCTGACGCTGACC
CCCGCCCAGTGGAAATCGTACAGCAGCGTCAGCTGCCAGGTCACGCACCAAGGGAAAACC
>KF985047.1 Equus caballus clone FetusE09 immmunoglobulin lambda light chain variable region (IGL) mRNA, partial cds
TTGGGGTCTCAGAAGGCAGTGCTCTTGGGGCGTCTCCACCATGGCCTGGACTCTGCTCCTTCTCACCCTC
CTCATTCAGGGTACAGGGTCCTGGGCCCAGTCTGCCCTGACTCAGCCTGCGTCAGTGTCCGGGGCTCTAG
GACAGTCGGTCACCATCACCTGTGCTGGAAGCAGCAGTGACATTGGGGGTTATAATGCTGTCAGTTGGTT
GCAACAGCACCCGGGCACAGCCCCCAAAGTTCTGATTTATAGTGTGAATACTCGGGCCTCAGGGATCCCT
GATCGCTTCTCTGGCTCCAAGTCTGGCAACACGGCCTCCCTGACCATCTCTGGGCTCCAGGTTGAGGACG
AGGCTGATTATTACTGTTACTCGCTTGTGAGTGGTTACACTTATATCTTCGGCGGCGGGACCCACCTCAG
CGTCCTGGGTGGTCCCCCGTCTGCACCCTCGGTCTCTCTCTTCCCGCCCTCCTCTGAGGAGCTCAGCACC
AACAAGGCCACAGTGGTGTGTCTCATCAGTGACTTCTCCCCCAGCGACTTGACGGTGAGCTGGAAGGGAA
ATGGCGCCGCCATCAGCCAGGGCGTCCAGACCACCAAGCCCTCGAAACAGAGCAATGGCAAGTACGCGGC
TAGCAGCTACCTGACGCTGACCCCCGCCCAGTGGAAATCGTACAGCAGCGTCAGCTGCCAGGTCACGCAC
CAAGGGAAAACC
>KF985046.1 Equus caballus clone FetusD06 immmunoglobulin lambda light chain variable region (IGL) mRNA, partial cds
ACCATGGCCTGGACCCCTCTCTTGTTAGCCTTCCTCACTCTCTGCACAGGTCCCGTGGCCTCTTCTAAGC
TGACTCAGCCATCTTCAGTGTCTGTGGCCTTGGGACAGACGGCCACCATCACCTGCAAGGGAGGCAACTT
TGAAAGTTTTGTTGGTAGCTGGTACCAGCAGAAGCCAGGCCAGGCCCCTGTGCTGGTCATCGATCCTAGT
AATGAGCGGCCCTCAGGGATCCCTGAACGATTCTCTGGCTCCAGCTCAGGAGACACAGCCACGCTGACCA
TCAGCGGGGCCCAGGCTGAGGACGAGGCTGACTATTACTGTCTGGCAGCAGATGCTTCTGATATCTTCGG
CAGCGGGACCCACCTCAGCGTCCTGGGTGGTCCCCCGTCTGCACCCTCGGTCTCTCTCTTCCCGCCCTCC
TCTGAGGAGCTCAGCACCAACAAGGCCACAGTGGTGTGTCTCATCAGTGACTTCTCCCCCAGCGACTTGA
CGGTGAGCTGGAAGGGAAATGGCGCCGCCATCAGCCAGGGCGTCCAGACCACCAAGCCCTCGAAACAGAG
CAATGGCAAGTACGCGGCTAGCAGCTACCTGACGCTGACCCCCGCCCAGTGGAAATCGTACAGCAGCGTC
AGCTGCCAGGTCACGCACCAAGGGAAAACC
>KF985045.1 Equus caballus clone FetusB09 immmunoglobulin lambda light chain variable region (IGL) mRNA, partial cds
GTGGCTTCAGAGGCAGGGCTTTGGGACATCTCCACCATGGCCTGGACCCCTCTCTTGTTAGCCTTCCTCA
CTCTCTGCACAGGTCCCGTGGCCTCTTCTAAGCTGACTCAGCCATCTTCAGTGTCTGTGGCCTTGGGACA
GACGGCCACCATCACCTGCAAGGGAGGCAACTTTGAAAGTTTTGTTGGTAGCTGGTACCAGCAGAAGCCA
GGCCAGGCCCCTGTGCTGGTCATCGATCCTAGTAATGAGCGGCCCTCAGGGATCCCTGAACGATTCTCTG
GCTCCAGCTCAGGAGACACAGCCACGCTGACCATCAGCGGGGCCCAGGCTGAGGACGAGGCTGACTATTA
CTGTCTGGCAGCAGATGCTTCTGATTATGAAGCTAGTATCTTCGGCAGCGGGACCCACCTCAGCGTCCTG
GGTGGTCCCCCGTCTGCACCCTCGGTCTCTCTCTTCCCGCCCTCCTCTGAGGAGCTCAGCACCAACAAGG
CCACAGTGGTGTGTCTCATCAGTGACTTCTCCCCCAGCGACTTGACGGTGAGCTGGAAGGGAAATGGCGC
CGCCATCAGCCAGGGCGTCCAGACCACCAAGCCCTCGAAACAGAGCAATGGCAAGTACGCGGCTAGCAGC
TACCTGACGCTGACCCCCGCCCAGTGGAAATCGTACAGCAGCGTCAGCTGCCAGGTCACGCACCAAGGGA
AAACC
>KF985044.1 Equus caballus clone FetusB06 immmunoglobulin lambda light chain variable region (IGL) mRNA, partial cds
TCAGAGGCAGAAGTCTGGGGAATCTCCACCATGGCCTGGACCCCTCTCTTGTTAGCCTTCCTCACTCTCT
GCACAGGTCCCGTGGCCTCTTCTAAGCTGACTCAGCCATCTTCAGTGTCTGTGGCCTTGGGACAGACGGC
CACCATCACCTGCAAGGGAGGCAACTTTGAAAGTTTTGTTGGTAGCTGGTACCAGCAGAAGCCAGGCCAG
GCCCCTGTGCTGGTCATCGATCCTAGTAATGAGCGGCCCTCAGGGATCCCTGAACGATTCTCTGGCTCCA
GCTCAGGAGACACAGCCACGCTGACCATCAGCGGGGCCCAGGCTGAGGACGAGGCTGACTATTACTGTCT

GGCAGCAGATGCTTCTGATTATATCTTCGGCAGCGGGACCCACCTCAGCGTCCTGGGTGGTCCCCCGTCT
GCACCCTCGGTCTCTCTCTTCCCGCCCTCCTCTGAGGAGCTCAGCACCAACAAGGCCACAGTGGTGTGTC
TCATCAGTGACTTCTCCCCCAGCGACTTGACGGTGAGCTGGAAGGGAAATGGCGCCGCCATCAGCCAGGG
CGTCCAGACCACCAAGCCCTCGAAACAGAGCAATGGCAAGTACGCGGCTAGCAGCTACCTGACGCTGACC
CCCGCCCAGTGGAAATCGTACAGCAGCGTCAGCTGCCAGGTCACGCACCAAGGGAAAACC
>KF985043.1 Equus caballus clone FetusC07 immmunoglobulin lambda light chain variable region (IGL) mRNA, partial cds
TCGGTGAAGATCTCCACCATGGCCTGGTCCCCTCTCCTCCTCACCCTCATCGCTCTCTGCACAGGATCCT
GGGCCCAGTCTGTGACTCAGCCCGCCTCAGTGTCTGGGACCCTGGGCCAGACAGTCACCATCTCCTGCTC
TGGAAGCAGCTCCAACATCGGGTATAGTTATAGTGCTGTGGGCTGGTACCAACAGATCCCAGGGACAGCC
CCCAAAACCCTCATCTATGCTACTAACAAACGAGCCTCAGGGGTCCCAGATCGATTCTCTGGCTCCAAGT
CTGGCAACACAGCCACCCTGACCATCTCTGGGCTTCAGGCTGAGGACGAGGCCGATTATTACTGTGGTTC
CTCATACAGCAGTGATATCTTCGGCGGCGGGACCCACCTCAGCGTCCTGGGTGGTCCCCCGTCTGCACCC
TCGGTCTCTCTCTTCCCGCCCTCCTCTGAGGAGCTCAGCACCAACAAGGCCACAGTGGTGTGTCTCATCA
GTGACTTCTCCCCCAGCGACTTGACGGTGAGCTGGAAGGGAAATGGCGCCGCCATCAGCCAGGGCGTCCA
GACCACCAAGCCCTCGAAACAGAGCAATGGCAAGTACGCGGCTAGCAGCTACCTGACGCTGACCCCCGCC
CAGTGGAAATCGTACAGCAGCGTCAGCTGCCAGGTCAAGCACCAAGGGAAAACC
>KF985041.1 Equus caballus clone FetusA02 immmunoglobulin lambda light chain variable region (IGL) mRNA, partial cds
GCTTCAGCTGTGGGGCCACAGAAGGCAGGACTCGGTGAAGATCTCCACCATGGCCTGGTCCCCTCTCCTC
CTCACCCTCATCGCTCTCTGCACAGGATCCTGGGCCCAGTCTCTGACTCAGCCCGCCTCAGTGTCTGGGA
CCCTGGGCCAGACAGTCACCATCTCCTGCTCTGGAAGCAGCTCCAACATCGGGTATAGTTATAGTGCTGT
GGGCTGGTACCAACAGATCCCAGGGACAGCCCCCAAAACCCTCATCTATGCTACTAACAAACGAGCCTCA
GGGGTCCCAGATCGATTCTCTGGCTCCAAGTCTGGCAACACAGCCACCCTGACCATCTCTGGGCTTCAGG
CTGAGGACGAGGCCGATTATTACTGTGGTTCCTCATACAGCAGTGATAGTTATATCTTCGGCGGCGGGAC
CCACCTCAGCGTCCTGGGTGGTCCCCCGTCTGCACCCTCGGTCTCTCTCTTCCCGCCCTCCTCTGAGGAG
CTCAGCACCAACAAGGCCACAGTGGTGTGTCTCATCAGTGACTTCTCCCCCAGCGACTTGACGGTGAGCT
GGAAGGGAAATGGCGCCGCCATCAGCCAGGGCGTCCAGACCACCAAGCCCTCGAAACAGAGCAATGGCAA
GTACGCGGCTAGCAGCTACCTGACGCTGACCCCCGCCCAGTGGAAATCGTACAGCAGCGTCAGCTGCCAG
GTCACGCACCAAGGGAAAACC
>KR190600.1 Equus caballus clone IGLV131 immunoglobulin lambda light chain variable region (IGL) mRNA, partial cds
ATGGCCTGGACCCCTCTCTTGTTAGCCTTCCTCACTCTCTGCACAGGTCCCGTGGCCTCTTCTAAGCTGA
CTCAGCCATCTTCAGTTTCTGTGGCCTTAGGACAGACTGCCACGATCACCTGCCAGGGAGGAATCTTTGA
CAAGAAGTATGTGAACTGGTACCAGCAGAAGCCCGGCGGGACCCCTGTGATAGTGATTTATAAGGATAGT
GAGCGGCCCTCCGGGATCCCTGACAGATTCTCTAGCTCCAGTTCAGGGAACACAGCCACCCTGACCATCA
GCAGGGCCCAGGCAGAGGACGAGGCCGTCTATTACTGCCACTCAGCAAATAGTGATAATGCTGTTGTATT
CGGCGGA
>KR190599.1 Equus caballus clone IGLV130 immunoglobulin lambda light chain variable region (IGL) mRNA, partial cds
ATGGCCTGGACCCCTCTCCTGCTCCCCCTCCTCACTCTCTGTATAGGTTCTGTGGTCTCCTTGGAGCTGA
CTCAGCCAGCTTCAGTTTCTGTGGCCTTAGGACAGACTGCCACGATCACCTGCCAGGGAGGAATCTTTGA
CAAGAAGTATATGAACTGGTACCAGCAGAAGCCCGGCGGGACCCCTGTGATAGTGATTTATATGGATAGT
GAGCGGCCCTCCGGGATCCCTGACAGATTCTCTAGCTCCAGTTCAGGGAACACAGCCACCCTGACCATCA
GCAGGGCCCAGGCAGAGGACGAGGCCGTCTATTACTGCCACTCTCAAGATAGTGATGATGTGGGTGTATT
CGGCGGA
>KR190598.1 Equus caballus clone IGLV129 immunoglobulin lambda light chain variable region (IGL) mRNA, partial cds
ATGGCCTGGACCCCTCTCCTGCTCCCCCTCCTCACTCTCTGTATAGGTTCTGTGGTCTCCTTGGAGCTGA
CTCAGCCAGCTTCAGTTTCTGTGGCCTTAGGACAGACTGCCACGATCACCTGCCAGGGAGGAATCTTTGA
CAAGAAGTATGTGAACTGGTACCAGCAGAAGCCCGGCGGGACCCCTGTGATAGTGATTTATAAGAATAGT
GAGCGGCCCTCCGGGATCCCTGACAGATTCTCTAGCTCCAGCTCAGGGAACACAGCCACCCTGACCATCA
GCAGGGCCCAGGCAGAGGACGAGGCCGTCTATTACTGCCACTCATATGACGATAATGGTAGTATCTTCGG
CGGC

>KR190597.1 Equus caballus clone IGLV128 immunoglobulin lambda light chain variable region (IGL) mRNA, partial cds
ATGGCCTGGACCCTTCTCCTGCTCCCCCTCCTCACTCTCTGTATAGGTTCTGTGGTCTCCTTGGAGCTGA
CTCAGCCAGCTTCAGTTTCTGTGGCCTTAGGACAGACTGCCACGATCACCTGCCAGGGAGGAATCTTTGA
CAAGCACTATGTGAACTGGTACCAGCAGAAGCCCGGCGGGACCCCTGTGATAGTGATTTATAAGGATAGT
GAGCGGCCCTCCGGGATCCCTGACAGATTCTCTAGCTCCAGCTCAGGGAACACAGCCACCCTGACCATCA
GCAGGGCCCAGGCAGAGGACGAGGCCGTCTATTACTGCCACTCAGCAGATAGTGATAACGCCAGTACATT
CGGCGGA
>KR190596.1 Equus caballus clone IGLV127 immunoglobulin lambda light chain variable region (IGL) mRNA, partial cds
ATGGCCTGGACCCCTCTCCTGCTCCCCCTCCTCACTCTCTGTATAGGTTCTGTGGTCTCCTTGGAGCTGA
CTCAGCCAGCTTCAGTTTCTGTGGCCTTAGGACAGACTGCCACGATCACCTGCCAGGGAGGAATCTTTGA
CGAGAAGTATGTGAACTGGTACCAGCAGAAGCCCGGCGGGACCCCTGTGATAGTGATTTATAAGGATAGT
GAGCGGCCCTCCGGGATCCCTGACAGATTCTCTAGCTCCAGCTCAGGGAACACAGCCACCCTGACCATCA
GCAGGGCCCAGGCAGAGGACGAGGCCGTCTATTACTGCCACTCATCAGATAGTGATAATGATTTATTCGG
CGGA
>KR190595.1 Equus caballus clone IGLV126 immunoglobulin lambda light chain variable region (IGL) mRNA, partial cds
ATGGCCTGGACCCCTCTCTTGTTAGCCTTCCTCACTCTCTGCACAGGTCCCGTGGCCACTTCTAAGCTGA
CTCAGCCATCTTCAGTGTCTGTGGCCTTGGGACAGACGGCCACCATCACCTGCAAGGGAGGCGACTTTGA
AAGTTTTGTTGTTAGCTGGTACCAGCAGAAGCCAGGCCAGGCCCCTGTGCTGGTCATCGGTGGTAGTAGT
GGGCGGCCCTCAGGGATCCCTGAACGATTCTCTGGCTCCAAATCAGGAGACACAGCCACCTTGACCATCA
GCGGGGCCCAGGCTGAGGACGAGGCTGACTATTACTGTCTGGCAGCAGATCGTTCTGATTATGAACTTGT
ATTCGGCGGA
>KR190594.1 Equus caballus clone IGLV125 immunoglobulin lambda light chain variable region (IGL) mRNA, partial cds
ATGGCCTGGACCCCTCTCCTGCTCCCCCTCCTCACTCTCTGTATAGGTTCTGTGGTCTCCTTGGAGCTGA
CTCAGCCAGCTTCAGTTTCTGTGGCCTTAGGACAGACTGTCACGATCACCTGCCAGGGAGGAATCTTTGA
CATTAAGTATGTGCACTGGTACCAGCAGAAGCCAGGCCAGGCCCCTGTGCTGGTCATCGATGCTAGTAAT
GAGCGGCCCTCAGGGATCCCTGAACGATTCTCTGGCTCCAGGTCAGGAGACACAGCCACGCTGACCATCA
GCGGGGCCCAGGCTGAGGACGAGGCTGACTATTACTGTCTGACAAGAGATGCTTCTACTTCTGATTATAT
CTTCGGCGGC
>KR190593.1 Equus caballus clone IGLV124 immunoglobulin lambda light chain variable region (IGL) mRNA, partial cds
ATGGCCTGGACCCCTCTCTTGTTAGCCTTCCTCACTCTCTGCACAGGTCCCGTGGCCTCTTCTAAGCTGA
CTCAGCCATCTTCAGTGTCTGTGGCCTTGGGACAGACGGCCACCATCACCTGCAAGGGAGGCGACTTTGA
AAGTTTTGTTGGTAGCTGGTACCAGCAGAAGCCAGGCCAGGCCCCTGTGCTGGTCATCGATGCTAGTAAT
GAGCGGCCCTCCGGGATCCCTGACAGATTCTCTGGCTCCAGCTCAGGGAACACAGCCACCCTGACCATCA
GCAGGGCCCAGACAGAGGACGAGGCCGTCTATTACTGCCACTCATTAGATAGTGATGCCGTTAGTAAATT
CGGCGGA
>KR190592.1 Equus caballus clone IGLV123 immunoglobulin lambda light chain variable region (IGL) mRNA, partial cds
ATGGCCTGGACCCCTCTCTTGTTAGCCTTCCTCACTCTCTGCACAGGTCCCGTGGCCTCTTCTAAGCTGA
CTCAGCCATCTTCAGTGTCTGTGGCCTTGGGACAGACGGCCACCATCACCTGCAAGGGAGGCGACTTTGA
AAGTTTTGTTGGTAGCTGGTACCAGCAGAAGCCCGGCGGGACCCCTGTGATAGTGATTTATAAGGATAGT
GAGCGGCCCTCCGGGATCCCTGACAGATTCTCTAGCTCCAGCTCAGGGAGCACAGCCACGCTGACCATCA
GTGGGGCCCAGGTTGAGGACGAGGCTGACTATTACTGTCTGGCAGCAGATGCTTCTGATTATATCTTCGG
CGGC
>KR190591.1 Equus caballus clone IGLV122 immunoglobulin lambda light chain variable region (IGL) mRNA, partial cds
ATGGCCTGGTCCCCTCTCTTGTTAGCCTTCCTCACTCTCTGCACAGGTCCCGTGGCCTCTTCTAAGCTGA
CTCAGCCATCTTCAGTGTCTGTGGCCTTGGGACAGACGGCCACCATCACCTGCAAGGGAGGCGACTTTGA
AAGTTTTGTTGGTAGCTGGTACCAGCAGAAGCCAGGCCAGGCCCCTGTGCTGGTCATCGATGCTAGTAAT
GAGCGGCCCTCAGGGATCCCTGAACGATTCTCTGGCTCCAGCTCAGGAGACACAGCCACGTTGACCATCA
GCGGGGCCCAGGCTGAGGACGAGGCTGACTATTATTTCCTGTTAACAGATGATTCTGGTTATATCTTCGG

CGGC
>KR190590.1 Equus caballus clone IGLV121 immunoglobulin lambda light chain variable region (IGL) mRNA, partial cds
ATGGCCTGGTCCCCTCTCCTCCTCACCCTCATCGCTCTCTGCACAGGATCCTGGGCCCAGTCTGTGACTC
AGCCATCTTCAGTGTCTGTGGCCTTGGGACAGACGGCCACCATCACCTGCAAGGGAGGCGACTTTGAAAG
TTTTGTTGGTAGCTGGTACCAGCAGAAGCCAGGCCAGGCCCCTGTGCTGGTCATCGATGCTAGTAATGAG
CGGCCCTCCGGGATCCCTGACAGATTCTCTAGCTCCAGCTCAGGGAACACAGCCACCCTGACCATCAACA
GGGCCCAGGCAGAGGACGAGGCCGTCTATTACTGCCACTCACAGGATAGTGATGATGCTACTGTATTCGG
CGGA
>KR190589.1 Equus caballus clone IGLV120 immunoglobulin lambda light chain variable region (IGL) mRNA, partial cds
ATGGCCTGGACCCCTCTCTTGTTAGCCTTCCTCACTCTCTGCACAGGTCCCGTGGCCTCTTCTAAGCTGA
CTCAGCCATCTTCAGTGTCTGTGGCCTTGGGACAGACGGCCACCATCACCTGCAAGGGAGGCGACTTTGA
AAGTTTTGTTGGTAGCTGGTACCAGCAGAAGCCAGGCCAGGCCCCTGTCGTGGTCATCGATGCTAGTAAT
GAGCGGCCCTCAGGGATCCCTGAACGATTCTCTGGGTCCAGTTCAGGAGAGACAGGTACGCTGACCATCA
GTGGAGCCCACGCTGAGGACGAGGCCGACTATTACTGTCTGGCAACAGATTATTATTTTCTTGAAACAGC
GTTCGGCGGC
>KR190588.1 Equus caballus clone IGLV119 immunoglobulin lambda light chain variable region (IGL) mRNA, partial cds
ATGGCCTGGACCCCTCTCTTGTTAGCCTTCCTCACTCTCTGCACAGGTCCCGTGGCCTCTTCTAAGCTGA
CTCAGCCATCTTCAGTGTCTGTGGCCTTGGGACAGACGGCCACCATCACCTGCAAGGGAGGCGACTTTGA
AAGTTTTGTTGGTAGTTGGTACCAGCAGAAGCCAGGCCAGGCCCCTGTGCTGGTCATCGATGCTAGTAAT
GAGCGGCCCTCAGGGATCCCTGAACGATTCTCTGGCTCCAGCTCAGGAGACACAGCCACGCTGACCATCA
GCGGGGCCCAGGCTGAGGACGAGGCTGACTATTACTGTCTGGCAAGAGATGCTTCTGATTATGAAGTTAG
TATCTTCGGCGGC
>KR190587.1 Equus caballus clone IGLV118 immunoglobulin lambda light chain variable region (IGL) mRNA, partial cds
ATGGCCTGGACCCCTCTCTTGTTAGCCTTCCTCACTCTCTGCACAGGTCCCGTGGCCTCTTCTAAGCTGA
CTCAGCCATCTTCAGTGTCTGTGGCCTTGGGACAGACGGCCACCATCACCTGCAAGGGAGGCGACTTTGA
AAGTTTTGTTGGTAGCTGGTACCAGCAGAAGCCAGGCCAGGCCCCTGTGCTGGTCATCGATGCTAGTAAT
GAGCGGCCCTCAGGGATCCCTGAACGATTCTCTGGCTCCAGGTCAGGAGACACAGCCACCCTGACCATCT
CTGGGCTTCAGGCTGAGGACGAGGCCGATTATTACTGTGTCTCCTATTACCCGAGTGAGCGTATTGATCC
TGTATTCGGCGGA
>KR190586.1 Equus caballus clone IGLV117 immunoglobulin lambda light chain variable region (IGL) mRNA, partial cds
ATGGCCTGGACCCCTCTCTTGTTAGCCTTCCTCACTCTCTGCACAGGTCCCGTGGCCTCTTCTAAGCTGA
CTCAGCCATCTTCAGTGTCTGTGGCCTTGGGACAGACGGCCACCATCACCTGCAAGGGAGGCGACTTTGA
AAGTTTTGTTGGTAGCTGGTACCAGCAGAAGCCAGGCCAGGCCCCTGTGCTGGTCATCGATGCTAGTAAT
GAGCGGCCCTCAGGGATCCCTGAACGATTCTCTGGGTCCAGTTCAGGAGAGACAGCTACGCTGACCATCA
GTGGAGCCCACGCTGAGGACGAGGCCGACTATTACTGTCTGGCAACAGATACTTATGTTGTTAAATTCGG
CGGA
>KR190585.1 Equus caballus clone IGLV116 immunoglobulin lambda light chain variable region (IGL) mRNA, partial cds
ATGGCCTGGTCCCCTCTCCTCCTCACCCTCATCGCTCTCTGCACAGGATCCTGGGCCCAGTCTCTGACCC
AGCCCGCCTCAGTGTCTGGGACCCTGGGCCAGACAGTCACCATCTCCTGCTCTGGAAGCAGCTCCAACAT
CGGAGATAGTTATAGTGTTGTGGGCTGGTACCAACAGATCCCAGGAACAGCCCCCAAAACCCTCATCTAT
GTGGATAACAAACGAGCCTCAGGGGTCCCAGATCGATTCTCTGGCTCCAAGTCTGGCAACACAGCCACCC
TGACCATCTCTGGGCTTCAGGCTGAGGACGAGGCCGATTATTACTGTGCTTACTATCTCGGCGGTGATGG
TGCATTCGGCGGA
>KR190584.1 Equus caballus clone IGLV115 immunoglobulin lambda light chain variable region (IGL) mRNA, partial cds
ATGGCCTGGTCCCCTCTCCTCCTCACCCTCATCGCTCTCTGCACAGGATCCTGGGCCCAGTCTCTGACCC
AGCCCGCCTCAGTGTCTGGGACCCTGGGCCAGACAGTCACCATCTCCTGCTCTGGAAGCAGCTCCAACAT
CGGATATACCTATAGTGCTGTGGTCTGGTACCAACAGGTCCCAGGAACAGCCCCCAAAACCCTCATCTAT
GATAGTAACAAACGAGTCTCAGGGGTCCCAGATCGATTCTCTGGCTCCAAGTCTGGCAACATAGCCACCC
TGACCATCTCTGGCCTTCAGGCTGAGGACGAGGCCGATTATTTTTGTGGTGCCGAAGATGGATTCGGCGG

A
>KR190583.1 Equus caballus clone IGLV114 immunoglobulin lambda light chain variable region (IGL) mRNA, partial cds
ATGGCCTGGTCCCCTCTCCTCCTCACCCTCATCGCTCTCTGCACAGGATCCTGGGCCCAGTCTCTGACCC
AGCCCGCCTCAGTGTCTGGGACCCTGGGCCAGACAGTCACCATCTCCTGCTCTGGAAGCAGCTCCAACAT
CGGCTATAGTTATAGTGCTGTGGGCTGGTACCAACAGATCCCAGGAACAGCCCCCAAAACCCTCATCTAT
GACAGTAACAAACGAGCCTCAGGGGTCCCAGATCGATTCTCTGGCTCCAAGTCTGGCAACACAGCCACCC
TGACCATCTCTGGGCTTCAGGCTGAGGACGAGGCCGATTATTACTGTGGTTCCTATGACAGCAGCAGTAG
TAGTGATGGTGCATTCGGCGGA
>KR190582.1 Equus caballus clone IGLV113 immunoglobulin lambda light chain variable region (IGL) mRNA, partial cds
ATGGCCTGGTCCCCTCTCCTCCTCACCCTCATCGCTTTCTGCACAGGATCCTGGGCCCAGTCTGTGACTC
AGCCCGCCTCAGTGTCTGGGACCCTGGGCCAGACAGTCACCATCTCCTGCTCTGGAAGCAGCTCCAACAA
TGTGGGCTGGTTCCAACAGATCCCAGGAACAGCCCCCAAAACCCTCATCTGGGGTAATAACAAACGAGCC
TCAGGGGTCCCAGATCGATTCTCTGGCTCCAAGTCTGGCAACACAGCCACCCTGACCATCTCTGGGGTCC
AGGCTGAGGACGAGGCCGATTATTACTGTGGTTCCTATGACAGCAGCAGTAGTAGTTATGTATTCGGCGG
A
>KR190581.1 Equus caballus clone IGLV112 immunoglobulin lambda light chain variable region (IGL) mRNA, partial cds
ATGGCCTGGTCCCCTCTCCTCCTCACCCTCATCGCTCTCTGCACAGGATCCTGGGCCCAGTCTGTGACTC
AGCCCGCCTCAGTGTCTGGGACCCTGGGCCAGACAGTCACCATCACCTGCACTGGAAGCAGCTCCAACAT
AGTTGCTTGGGTGGGCTGGTACCAACAGATCCCAGGAACAGCCCCCAAAACCCTCATCTATGCTAATAAC
AGACGAGCCTCAGGGGTCCCAGATCGATTCTCTGGCTCCAAGTCTGGCAACACAGCCACCCTGACCATCT
CTGGGGTCCAGGCTGAGGACGAGGCGGATTATTACTGTAGTTCCTATGACAGTAGCAGTGGTGTTGTATT
CGGCGGA
>KR190580.1 Equus caballus clone IGLV111 immunoglobulin lambda light chain variable region (IGL) mRNA, partial cds
ATGGCCTGGTCCCCTCTCCTCCTCACCCTCATCGCTCTCTGCACAGGATCCTGGGCCCAGTCTGTGACTC
AGCCCGCCTCAGTGTCTGGGACCCTGGGCCAGACAGTCACCATCTCCTGCTCTGGAAGCAGCTCCAACAT
CGGCTTTGATTATAGTTATGTGGGCTGGTTCCAACAGATCCCAGGAACAGCCCCCAAACTCCTCATCTAT
TCTTCCGCTAGCAGGGCTTCCGGGGTCCCCGACCGATTCTCTGGCTCCAGGTCTGGCAACACAGCCACCC
TGACCATCAGCAGGGCCCAGGCAGAGGACGAGGCCGTCTATTACTGCCACTCAGAAGATAGTGATAATAA
TGTTGTATTCGGCGGA
>KR190579.1 Equus caballus clone IGLV110 immunoglobulin lambda light chain variable region (IGL) mRNA, partial cds
ATGGCCTGGTCCCCTCTCCTCCTCACCCTCATCGCTCTCTGCACAGGATCCTGGGCCCAGTCTCTGACTC
AGCCCGCCTCAGTGTCTGGGACCCTGGGCCAGACAGTCACCATCTCCTGCACTGGAAGCAGCTCCAGCAT
AGGTTCTGGTATGGGCTGGTACCAACAGATCCCAGGGACAGCCCCCAAAACCCTCATCTATGATACTAAC
AAACGAGCCTCAGGGGTCCCAGATCGATTCTCTGGCTCCAAGTCTGGCGACACAGCCACCCTGACCATCA
CTGGGCTTCAGGCTGAGGACGAGGCCGATTATTATTGTGGTGCCTCTAGTAGCAGCGCTGATAGTCTATT
CGGCGGA
>KR190578.1 Equus caballus clone IGLV109 immunoglobulin lambda light chain variable region (IGL) mRNA, partial cds
ATGGCCTGGTCCCCTCTCCTCCTCACCCTCATCGCTCTCTGCACAGGATCCTGGGCCCAGTCTCTGACTC
AGCCCGCCTCAGTGTCTGGGACCCTGGGCCAGACAGTCACCATCTCCTGCACTGGAAGCAGCTCCAACAT
AGGTTCTGTAGTGGTATGGTACCAACAGATCCCAGGGACAGCCCCCAAAACCCTCATCTATGGTAATAAC
AAACGAGCCTCAGGGGTCCCAGATCGATTCTCTGGCTCCAAGTCTGGCAACACAGCCACCCTGACCATCT
CTGGGGTCCAGGCTGAGGACGAGGCCGATTATTACTGTGGTTCCTATTACAACAGTGATAGTACTGATGC
ATTCGGCGGA
>KR190577.1 Equus caballus clone IGLV108 immunoglobulin lambda light chain variable region (IGL) mRNA, partial cds
ATGGCCTGGTCCCCGCTCCTTCTCACCTTCATCGCTCTCTGCACAGGATCCTGGGCCCAGTCTCTGACTC
AGCCCGCCTCAGTGTCTGGGACCCTGGGCCAGACAGTCACCATCTCCTGCTCTGGAAGCAGCTCCAACAT
TGGATGGAGTGGTAGTTATGTGACCTGGTTCCAACAGATCCCAGGAACAGCCCCCAAACGTCTCATCTAT
GATGCCACTAGTAGAGCGTCCGGGGTCCCCGATCGATTCTCTGGCTCCAGGTCTGGCAACACAGCCACCC

TGACCATCTCTGGGGTCCAGGCTGAGGACGAGGCTGATTATTACTGCTCATCAGGAGACATGAGCCTGAC
GTTTGTATTCGGCGGA
>KR190576.1 Equus caballus clone IGLV107 immunoglobulin lambda light chain variable region (IGL) mRNA, partial cds
ATGGCCTGGTCCCCTCTCCTCCTCACCCTCATCGCTCTCTGCACAGGATCCCGGGCCCAGTCTGTGACTC
AGCCTGCCTCAGTGTCTGGGACCCTGGGCCAGACAGTCACCATCTCCTGCTCTGGAAGCAGCTCCAACGT
TGGGAGTGGTTATGTGTCCTGGTACCAACAGATCCCAGGAACAGCCCCCAAACTCCTCATCTTTCGTGCC
ACTAGTAGGGCTTCCGGGGTCCCCGACCGATTCTCTGGCACCAGGTCTGGCAACACAGCCACCCTGACCA
TCTCTGGGCTCCAGGCTGAGGATGAGGCCGATTATTACTGTGGTGCCTCTGGCAACAGTGGGGGTGGATT
CGGCGGA
>KR190575.1 Equus caballus clone IGLV106 immunoglobulin lambda light chain variable region (IGL) mRNA, partial cds
ATGGCCTGGTCCCCTCTCCTCCTCACCCTCATCGCTCTCTGCACAGGATCCTGGGCCCAGTCTGTGACTC
AGCCCGCCTCAGTGTCTGGGACCCTGGGCCAGACAGTCACCATCTCCTGCTCTGGAAGCAGCTCCAACAT
CGGAAATAGTTATAATTGGGTGGGCTGGTTCCAACAGATCCCAGGAACAGCCCCCAAAACCCTCATCTGG
GGTGGCAACGAACGAGCCTCAGGGGTCCCAGATCGATTCTCTGGCTCCAAGTCTGGCAACACAGCCACCC
TGACCATCTCTGGGGTCCAGGCTGAGGACGAGGCCGATTATTACTGTGGTTCCTATGACAGCAGCAGTAG
TAGTGATATCTTCGGCGGC
>KR190574.1 Equus caballus clone IGLV105 immunoglobulin lambda light chain variable region (IGL) mRNA, partial cds
ATGGCCTGGTCCCCTCTCCTCCTCACCCTCATCGCTTTCTGCACAGGATCCTGGGCCCAGTCTGTGACTC
AGCCCGCCTCAGTGTCTGGGACCCTGGGCCAGACAGTCACCATCTCCTGCTCTGGAAGCAGCTCCAACAT
CGGAAGTAGTTATAGTTATGTGGGCTGGTTCCAACAGATCCCAGGAACAGCCCCCAAAACCCTCATCTAT
GAAAATAACAAACGAGCCTCAGGGGTCCCAGATCGATTCTCTGGCTCCAAGTCTGGCAACACAGCCACCC
TGACCATCTCTGGGGTCCAGGCTGAGGACGAGGCCGATTATTACTGTGGTTCAGGTGACGTCAGCAGTAG
AAGTGCTGTATTCGGCGGA
>KR190573.1 Equus caballus clone IGLV104 immunoglobulin lambda light chain variable region (IGL) mRNA, partial cds
ATGGCCTGGTCCCCTCTCCTCCTCACCCTCATCGCTCTCTGCACAGGATCCTGGGCCCAGTCTGTGACTC
AGCCCGCCTCAGTGTCTGGGACCCTGGGCCAGACAGTCACCATCTCCTGCTCTGGAAGCAGCTCCAACAT
CGGAGATAGGTATAGTTATGTGGGCTGGTTCCAACAGATCCCAGGAACAGCCCCCAAAACCCTCATGCAT
GGTAATAACAAACGAGCCTCAGGGGTCCCAGATCGATTCTCTGGCTCCAAGTCTGGCAACACAGCCACCC
TGACCATCTCTGGGGTCCAGGCTGAGGACGAGGCCGATTATTACTGTGGTTCCTATGACACCAGCAGTAG
TAGTATTATATTCGGCGGA
>KR190572.1 Equus caballus clone IGLV103 immunoglobulin lambda light chain variable region (IGL) mRNA, partial cds
ATGGCCTGGTCCCCTCTCCTCCTCACCCTCATCGCTCTCTGCACAGGATCCTGGGCCCAGTCTCTGACTC
AGCCCGCCTCAGTGTCTGAGACCCTGGGCCAGACAGTCACCATCTCCTGCTCTGGAAGCAGCTCCAACAT
CGGCGATAGTTATGAAAGTGTGGGCTGGTACCAACAGATCCCAGGAACAGCCCCCAAAACCCTCATCTAT
GGTACTAACAAACGAGCCTCAGGGGTCCCAGATCGATTCTCTGGCTCCAAGTCTGGCAACACAGCCACCC
TGACCATCTCTGGGGTCCAGGCTGAGGACGAGGCCGATTATTACTGTGCTTCCTATGACAGCAGCAGTAG
TAGTGCTGTGTTCGGCGGA
>KR190571.1 Equus caballus clone IGLV102 immunoglobulin lambda light chain variable region (IGL) mRNA, partial cds
ATGGCCTGGTCCCCTCTCCTCCTCACCCTCATCGCTCTCTGCACAGGATCCTGGGCCCAGTCTGTGACTC
AGCCCGCCTCAGTGTCTGGGACCCTGGGCCAGACAGTCACCATCTCCTGCTCTGGAAGCAGCTCCAATAT
CGGACGTAGTAATGGTGATGTGGGCTGGTTCCAACAGATCCCAGGAACAGCCCCCAGCACCCTCATTTAT
GGTAATAACAAACGAGCCTCAGGGGTCCCAGATCGATTCTCTGGCTCCAAGTCTGGCAACACAGCCACCC
TGACCATCTCTGGGGTCCAGGCTGAGGACGAGGCCGATTATTACTGTGGTTCCTATGACAGCAGCAGTAG
TAGTTTTGTATTCGGCGGA
>KR190570.1 Equus caballus clone IGLV101 immunoglobulin lambda light chain variable region (IGL) mRNA, partial cds
ATGGCCTGGTCCCCGCTCCTTCTCACCTTCATCGCTCTCTGCACAGGATCCTGGGCCCAGTCTCTGACTC
AGCCCGCCTCAGTGTCTGGGACCCTGGGCCAGACAGTCACCATCTCCTGCTCTGGAAGCAGCTCCAACAT
TGGGTGGAGTGATAGTTATGTGAGCTGGTTCCAACAGATCCCAGGAACAACCCCCAAAATTCTCATCTAT
GTTGACACTAGTAGAGCGTCCGGGGTCCCCGATCGATTCTCTGGCTCCAGGTCTGGCAACACAGCCACCC

TGACCATCTCTGGGGTCCAGGCTGAGGACGAGGCTGATTATTACTGCTCATCAGCAGACAGCAGCCTGGG
GAGTGCTTTATTCGGCGGA
>KR190569.1 Equus caballus clone IGLV100 immunoglobulin lambda light chain variable region (IGL) mRNA, partial cds
ATGGCCTGGTCCCCTCTCCTCCTCACCCTCATCGCTCTCTGCACAGGATCCTGGGCCCAGTCTCTGACCC
AGCCCGCCTCAGTGTCTGGGACCCTGGGCCAGACAGTCACCATCTCCTGCTCTGGAAGCAGCTCCAACAT
TGGGTATAGTAGTAGTTATGTGAGCTGGTTCCAACAGATCCCAGGAACAGCCCCCAAAACTCTCATCTAT
TATGCCACTAGTAGAGCGTCCGGGGTCCCCGATCGATTCTCTGGCTCCAGGTCTGGCAACACAGCCACCC
TGACCATCTCTGGGGTCCAGGCTGAGGACGAGGCTGATTATTACTGCTCATCAGCAGACAGCAGCCTGAG
GATTATCTTCGGCGGC
>KR190568.1 Equus caballus clone IGLV99 immunoglobulin lambda light chain variable region (IGL) mRNA, partial cds
ATGGCCTGGTCCCCTCTCCTCCTCACCCTCATCGCTCTCTGCACAGGATCCTGGGCCCAGTCTCTGACTC
AGCCCGCCTCAGTGTCTGGGACCCTGGGCCAGACAGTCACCATCTCCTGCTCTGGAAGCAGCTCCAACAT
CGGTCATAGTAAGAGTTATGTGGCCTGGTTCCAACAGATCCCAGGAACAGCCCCCAAAACCCTCATCTAT
GGTAATAACAAACGAGCCTCAGGGGTCCCAGATCGATTCTCTGGCTCCAAGTCTGGCAACACAGCCACCC
TGACCATCTCTGGGGTCCAGGCTGAGGACGAGGCCGATTATTACTGCTCAGCAGGAGACAGCAGTGGCAA
TAATTGGGTATTCGGCGGG
>KR190567.1 Equus caballus clone IGLV98 immunoglobulin lambda light chain variable region (IGL) mRNA, partial cds
ATGGCCTGGTCCCCTCTCCTCCTCACCCTCATCGCTCTCTGCACAGGATCCTGGGCCCAGTCTCTGACTC
AGCCCGCCTCAGTGTCTGGGACCCTGGGCCAGACAGTCACCATCTCCTGCTCTGGAAGCAGCTCCAACAT
CGGAAATTGGTATAGTGATGTGGGCTGGTACCAACAGATCCCAGGAACAGCCCCCAAAACCCTCATCTAT
GGTGATAACAAACGAGTCTCAGGGGTCCCAGATCGATTCTCTGGCTCCAAGTCTGGCAACACAGCCACCC
TGACCATCTCTGGGGTCCAGGCTGAGGACGAGGCCGATTATTACTGCTCAGCAGAAGACAACAGTGGTAA
TAGTGATGTTATATTCGGCGGA
>KR190566.1 Equus caballus clone IGLV97 immunoglobulin lambda light chain variable region (IGL) mRNA, partial cds
ATGGCCTGGTCCCCTCTCCTCCTCACCCTCATCGCTCTCTGCACAGGATCCTGGGCCCAGTCTCTGACCC
AGCCCGCCTCAGTGTCTGGGACCCTGGGCCAGACAGTCACCATCTCCTGCTCTGGAAGCAGCTCCAACAT
CGGTTATAGTGAGAATACTGCTGTGGACTGGTACCAACAGATCCCAGGAACAGCCCCCAAAACCCTCATC
TATTTCAATAACAGACGAGCCTCAGGGGTCCCAGATCGATTCTCTGGCTCCAAGTCTGGCAACACAGCCA
CCCTGACCATCTCTGGGCTTCAGGCTGAGGACGAGGCCGATTATTACTGTGGAGCCGATGACGTCAGTGA
AGGTAGTACATTCGGCGGA
>KR190565.1 Equus caballus clone IGLV96 immunoglobulin lambda light chain variable region (IGL) mRNA, partial cds
ATGGCCTGGTCCCCTCTCCTCCTCACCCTCATCGCTCTCTGCACAGGATCCTGGGCCCAGTCTGTGACTC
AGCCCGCCTCAGTGTCTGGGACCCTGGGCCAGGAAGTCACCATCTCCTGCTCTGGAACGAACTCCAACAT
CGGACTGACTTGGAGTGCCGTGGCCTGGTTCCAACAGATCCCAGGAACAGCCCCCAAAACCCTCATCTAT
GATGATGATAGACGAGCCCCAGGGGTCCCAGATCGATTCTCTGGCTCCAAGTCTGGCAACACAGCCACCC
TGACCATCTCTGGGCTTCAGGCTGAGGACGAGGCCGAATATTACTGTGGTTGTTATTACAGCAGTGATAG
TGGTGCATTCGGCGGA
>KR190564.1 Equus caballus clone IGLV95 immunoglobulin lambda light chain variable region (IGL) mRNA, partial cds
ATGGCCTGGTCCCCTCTCCTCCTCACCCTCATCGCTCTCTGCACAGGATCCTGGGCCCAGTCTCTGACTC
AGCCCGCCTCAGTGTCTGGGACCCTGGGCCAGACAGTCACCATCTCCTGCTCTGGAAGCAGCTCCAACAT
CGGGTATAGTTATAGTACTGTGGGCTGGTACCAACAGATCCCAGGAACAGCCCCCAAAACCCTCATCTAT
GAGAATAACAAACGAGCCTCAGGGGTCCCAGATCGATTCTCTGGCTCCAAGTCTGGCAACACAGCCACCC
TGACCATCTCTGGGGTCCAGGCTGAGGACGAGGCCGATTATTACTGTGGTACCTCTAGCATCAGTGGTAG
TAGTGATGCTGTATTCGGCGGA
>KR190563.1 Equus caballus clone IGLV94 immunoglobulin lambda light chain variable region (IGL) mRNA, partial cds
ATGGCCTGGTCCCCTCTCCTCCTCACCCTCATCGCTCTCTGCACAGGATCCTGGGCCCAGTCTGTGACTC
AGCCCGCCTCAGTGTCTGGGACCCTGGGCCAGACAGTCACCATCACCTGCACTGGAAGCAGCTCCAACAT
AGTTGCTTATGTGGGCTGGTACCAACAGATCCCAGGAACAGCCCCCAAAACCCTCATCTATGATAATAAC

AAACGAGCCTCAGGGGTCCCAGATCGATTCTCTGGCTCCAAGTCTGGCAACACAGCCACCCTGACCATCT
CTGGGGTCCAGGCTGAGGACGAGGCCGATTATTACTGTGCATCAGGAGACACAAGCCTGAGGAGTGCTGT
ATTCGGCGGA
>KR190562.1 Equus caballus clone IGLV93 immunoglobulin lambda light chain variable region (IGL) mRNA, partial cds
ATGGCCTGGTCCCCTCTCCTCCTCACCCTCATCGCTCTCTGCACAGGATCCTGGGCCCAGTCTGTGACTC
AGCCCGCCTCAGTGTCTGGGACCCTGGGCCAGACAGTCACCATCTCCTGCTCTGGAAGCAGCTCCAACAT
AGTTGCTTATGTGGGCTGGTACCAACAGATCCCAGGAACAGCCCCCAAAACCCTCATCTATGGTAATAAC
AAACGAGCCTCAGGGGTCCCAGATCGATTCTCTGGCTCCAAGTCTGGCAACACAGCCACCCTGACCATCT
CTGGGGTCCAGGCTGAGGACGAGGCCGATTATTACTGCTCAGCAGGAGACATGAGTGGTAGTAGTGTCGT
ATTCGGCGGC
>KR190561.1 Equus caballus clone IGLV92 immunoglobulin lambda light chain variable region (IGL) mRNA, partial cds
ATGGCCTGGTCCCCTCTCCTCCTCACCCTCATCGCTCTCTGCACAGGTTCCTGGGCCCAGTCTGTGACTC
AGCCCGCCTCAGTGTCTGGGACCCTGGGCCAGACAGTCACCATCTCCTGCTCTGTCGGGGCGGCATACAC
GTTTGTGGCCTGGTTCCAACAGATCCCAGGAACAGCCCCCAAAACCCTCATCTATGGCAATGAATTGCGA
GCCTCAGGGGTCCCAGATCGATTCTCTGGCTCCAAGTCTGGCAACACAGCCACCCTGACCATCTCTGGGG
TCCAGGCTGAGGACGAGGCCGATTATTACTTCTCAGCAGGAGACATCAGTGGTAGTCCTGTATTCGGCGG
A
>KR190560.1 Equus caballus clone IGLV91 immunoglobulin lambda light chain variable region (IGL) mRNA, partial cds
ATGGCCTGGTCCCCTCTCCTCCTCACCCTCATCGCTCTCTGCACAGGATCCTGGGCCCAGTCTGTGACTC
AGCCCGCCTCAGTGTCTGGGACCCTGGGCCAGACAGTCACCATCACCTGCACTGGAAGCAGCTCCAACTC
ATTTGTTAATGTGGGATGGTACCAACAGATCCCAGGAACAGCCCCCAAAAGCCTCATCCATGGTAATAAC
AAACGAGCCTCAGGGGTCCCAGATCGATTCTCTGGCTCCAAGTCTGGCAACACAGCCACCCTGACCATCT
CTGGGGTCCAGGCTGAGGACGAGGCCGATTATTACTGTGGTTCCTATGACAGCAACAGTAGAAGTGTTGT
ATTCGGCGGA
>KR190559.1 Equus caballus clone IGLV90 immunoglobulin lambda light chain variable region (IGL) mRNA, partial cds
ATGGCCTGGTCCCCTCTCCTCCTCACCCTCATCGCTCTCTGCACAGGATCCTGGGCCCAGTCTCTGACTC
AGCCCGCCTCAGTGTCTGGGACCCTGGGCAAGACAGTCACCATCTCCTGCACTGGAACTAACTCCATTAT
TGGTTCATTTATGGCCTGGTACCAACAGATCCCAGGGACAGCCCCCAAAACCCTCATCTATGATAATAAC
AAACGAGCCTCAGGGGTCCCAGATCGATTCTCTGGCTCCAAGTCTGGCAACACAGCCACCCTGACCATCT
CTGGGCTTCAGACTGAGGACGAGGCCGATTATTTTTGTGGTTCCTATGAGAGTAGCAGTAGTAGTGTTGA
ATTCGGCGGA
>KR190558.1 Equus caballus clone IGLV89 immunoglobulin lambda light chain variable region (IGL) mRNA, partial cds
ATGGCCTGGTCCCCTCTCCTCCTCACCCTCATCGCTCTCTGCACAGGATCCTGGGCCCAGTCTCTGACTC
AGCCCGCCTCAGTGTCTGGGACCCTGGGCCAGACAGTCACCATCTCCTGCACTGGAATCAAGACCACCAT
AGGTTCTTATGTGGGCTGGTACCAACAGATCCCAGGGACAGCCCCCAAAACCCTCATCCTTGGTACGAAC
GAACGAGCCTCAGGGGTCCCAGATCGATTCTCTGGCTCCAAGTCTGGCAACACAGCCACCCTGACCATCT
CTGGGCTTCAGGCTGAGGACGAGGCCGAATATTACTGTGGTTCCTATTACAGTAGTGATAGTGGTGCATT
CGGCGGA
>KR190557.1 Equus caballus clone IGLV88 immunoglobulin lambda light chain variable region (IGL) mRNA, partial cds
ATGGCCTGGTCCCCTCTCCTCCTCACCCTCATCGCTCTCTGCACAGGATCCTGGGCCCAGTCTCTGACTC
AGCCCGCCTCAGTGTCTGGGACCCTGGGCCAGACAGTCACCATCTCCTGCACTGGAATCAGCTCCATCGG
AGGTTCTAGTATGGGCTGGTACCAACAGATCCCAGGGACAGCCCCCAAAACCCTCATCTATGCTACTAAC
ACACGAGCCTCAGGGGTCCCAGATCGATTCTCTGGCTCCAAGGTTGGCAACACAGCCACCCTGACCATCT
CTGGGGTCCAGGCTGAGGACGAGGCCGACTATTACTGTGCAGCAGGAGACAGAGACCTGAGGGCAGCTGT
GTTCGGCGGA
>KR190556.1 Equus caballus clone IGLV87 immunoglobulin lambda light chain variable region (IGL) mRNA, partial cds
ATGGCCTGGTCCCCTCTCCTCCTCACCCTCATCGCTCTCTGCACAGGATCCTGGGCCCAGTCTGTGACTC
AGCCCGCCTCAGTGTCTGGGACCCTGGGCCAGACAGTCACCATCTCCTGTTCTGGGAGCAGCTCCAACAT
CGGGTATAGGCGTAGTGATGTGGGCTGGTTCCGACAGATCCCAGGAACAGCCCCCAAAACCCTCATCTAT

GGTAGTAACAAACGAGCCTCAGGGGTCCCAGATCGATTCTCTGGCTCCAAGTCTGGCAACACAGCCACCC
TGACCATCTCTGGGGTCCAGGCTGAGGACGAGGCCGATTATTACTGTGGTTCCGGTTACAGCAGTGATAG
TAGTGATGATGTATTCGGCGGA
>KR190555.1 Equus caballus clone IGLV86 immunoglobulin lambda light chain variable region (IGL) mRNA, partial cds
ATGGCCTGGTCCCCTCTCCTCCTCACCCTCATCGCTCTCTGCACAGGCTCCTGGGCCCAGTCTGTGACTC
AGCCCGCCTCAGTGTCTGGGACCCTGGGCCAGACAGTCACCATCTCCTGCTCTGGAAGCAGCTCCAACAT
CGGACGTACAACGGCTAATGTGGCCTGGTACCTACAGATCCCAGGAACAGCCCCCAAAACCCTCATTTAT
GGTGATAACAAACGAGTCTCAGGGGTCCCAGATCGATTCTCTGGCTCCAAGTCTGGCAACACAGCCACCC
TGACCATCTCTGGGGTCCAGGCTGAGGACGAGGCCGCTTATTATTGTAGTTCCTGGGACACCAGCACTAT
GACCCTTATATTCGGCGGA
>KR190554.1 Equus caballus clone IGLV85 immunoglobulin lambda light chain variable region (IGL) mRNA, partial cds
ATGGCCTGGTCCCCTCTCCTCCTCACCCTCATCGCTCTCTGCACAGGATCCTGGGCCCAGTCTGTGACTC
AGCCCGCCTCAGTGTCTGGGACCCTGGGCCAGACAGTGACCATCTCCTGCTCTGGAAGCAGATCCAACAT
CGGGAATGATTATACTAATGTGGGCTGGTTCCAGCAGATCCCAGGAACAGCCCCCAAAACCCTCATCTAT
GGTAATAACAAGCGAGCCTCAGGGGTCCCAGATCGGTTCTCTGGCTCCAAGTCTGGCAACACAGCCACCC
TGACCATCTCTGGGCTTCAGGCTGAGGACGAGGCCGATTATTACTGTAGTTCCTATTACAGTAGTGATGG
TGCGTTCGGCGGA
>KR190553.1 Equus caballus clone IGLV84 immunoglobulin lambda light chain variable region (IGL) mRNA, partial cds
ATGGCCTGGTCCCCTCTCCTCCTCACCCTCATCGCTCTCTGCACAGGATCCTGGGCCCAGTCTGTGACTC
AGCCCGCCTCAGTGTCTGGGACCCTGGGCCAGACAGTCACCATCTCCTGTTTTGGAAGCAGCTCCAACAT
CGGGTCGACTTATAGTTATGTGGGCTGGTTCCAACAGATCCCAGGAACAGCCCCCAAAACCCTCATCTAT
GGTAGTGTCAAACGAGCCTCAGGGGTCCCAGATCGATTCTCTGGCTCCAAGTCTGGCAACACAGCCACCC
TGACCATCTCTGGGGTTCAGGCTGAGGACGAGGCCGATTATTACTGTGCTTCCTATTACAACAGTGATAA
TGGCGCATTCGGCGGA
>KR190552.1 Equus caballus clone IGLV83 immunoglobulin lambda light chain variable region (IGL) mRNA, partial cds
ATGGCCTGGTCCCCTCTCCTCCTCACCCTCATCGCTCTCTGCACAGGATCCTGGGCCCAGTCTGTGACTC
AGCCCGCCTCAGTGTCTGGGACCCTGGGCCAGACAGTCACCATCTTTTGTTCTGGAAGTGAGTCCAACAT
CGGATCTATGTATAGTTATGTGGCCTGGTTCCAACAGAGACCAGGGACAGCCCCCAAAACCCTCATTTAT
GGGGATGACAAACGTGCCTCAGGGGTCCCAGGCCGCTTCTCTGGCAACAAGTCTGGCAACACAGCCACCC
TGACCATCTCTAGGGCCCAGACTGAGGACGAGGCCGTTTATTACTGTGGTTCGCTGGACGAAGCCAGTAC
GAGTTTTGTATTCGGCGGA
>KR190551.1 Equus caballus clone IGLV82 immunoglobulin lambda light chain variable region (IGL) mRNA, partial cds
ATGGCCTGGTCCCCTCTCCTCCTCACCCTCATCGCTCTCTGCACAGGATCCTGGGCCCAGTCTGTGACTC
AGCCCGCCTCAGTGTCTGGGACCCTGGGCCAGACAGTCACCATCTCCTGCTCTGGAAGCAGCTCCAACAT
CGGATATAGTTATGTGGGCTGGTTCCAACAGATCCCAGGAACAGCCCCCAAAACCCTCATCTATGGTAAT
AACAAACGAGCCTCAGGGGTCCCAGATCGATTCTCTGGCTCCAAGTCTGGCAACACAGCCACCCTGACCC
TCTCTGGGGTCCAGGCTGAGGACGAGGCCGATTATTACTGTGGTTCCTGGGACACAAGCAGTAGATTTGT
TGTATTCGGCGGA
>KR190550.1 Equus caballus clone IGLV81 immunoglobulin lambda light chain variable region (IGL) mRNA, partial cds
ATGGCCTGGTCCCCTCTCCTCCTCACCCTCATCGCTCTCTGCACAGGATCCTGGGCCCAGTCTGTGACTC
AGCCCGCCTCAGTGTCTGGGACCCTGGGCCAGACAGTCACCATCTCCTGCTCTGGAAGCAGCTCCAACAT
CGGGAATGTTGTGGATAATTATGTGGTCTGGTTCCAACAGATCCCAGGAACAGCCCCCAAAACCCTCATC
TATGGTAATGACAAACGAGCCTCAGGGGTCCCAGATCGATTCTCTGGCTCCAAGTCTGGCAACACAGCCA
CCCTGACCATCTCTGGGGTCCAGGCTGAGGACGAGGCCGATTATTACTGTGGTTCCTGGGACAGCAGCAG
TAGTAGTGGTACGTTCGGCGGA
>KR190549.1 Equus caballus clone IGLV80 immunoglobulin lambda light chain variable region (IGL) mRNA, partial cds
ATGGCCTGGTCCCCTCTCCTCCTCACCCTCATCGCTCTCTGCACAGGATCCTGGGCCCAGTCTGTGACTC
AGCCCGCCTCAGTGTCTGGGACCCTGGGCCAGACAGTCACCATCTCCTGCTCTGGAAGCAGCTCCAACAT

CGGGTGGCTCAATAGTCATGTGGGCTGGTTCCAACAGAAAACAGGGACAGCCCCCAAAACCCTCGTCTAT
AATAATAACAAACGAGCCTCAGGGGTCCCAGATCGATTCTCTGGCTCCAAGTCTGGCAACACAGCCACCC
TGACCATCTCTGGGGTCCAGGCTGAGGACGAGGCCGATTATTACTGTGGTTCCCGTGACAGCGGAAGTGA
AATTTTTCTATTCGGCGGA
>KR190548.1 Equus caballus clone IGLV79 immunoglobulin lambda light chain variable region (IGL) mRNA, partial cds
ATGGCCTGGTCCCCTCTCCTCCTCACCCTCATCGCTCTCTGCACAGGATCCTGGGCCCAGTCTCTGACTC
AGCCCGCCTCAGTGTCTGGGACCCTGGGCCAGACAGTCACCATCTCCTGCTCTGGAAGCAGCTCCAATAT
CGGAGGGGCTAGTAGTGCTGTGGCCTGGTACCAACAGATCCCAGGAACAGCCCCCAAAACCCTCATCTAT
GGTGATAACTCACGAGCCTCAGGGGTCCCAGATCGATTCTCTGGCTCCAAGTCTGGCAACACAGCCACCC
TGACCATCTCTGGGGTCCAGGCTGAGGACGAGGCTGACTATTACTGTGGTTCCTATGACAGAAGCAGTAG
AAGTGATGCATTCGGCGGA
>KR190547.1 Equus caballus clone IGLV78 immunoglobulin lambda light chain variable region (IGL) mRNA, partial cds
ATGGCCTGGTCCCCTCTCCTCCTCACCCTCATCGCTCTCTGCACAGGATCCTGGGCCCAGTCTCTGACTC
AGCCCGCCTCAGTGTCTGGGACCCTGGGCCAGACAGTCACCATCTCCTGCTCTGGAAGCAGCTCCAACAT
CGGGTATAGTGCTAGTTATGTGGCCTGGTTCCAACAGATCCCAGGAACAGCCCCCAAAACCCTCATCTAT
GCTACTAACGCACGAGCCTCAGGGGTCCCAGATCGATTCTCTGGCTCCAAGTCTGGCAACACAGCCACCC
TGACCATCTCTGGGGTCCAGGCTGAGGACGAGGCCGATTATTACTGTGGTTCCTATGACAGCAGCAGTAG
TAGTGCTGTATTCGGCGGA
>KR190546.1 Equus caballus clone IGLV77 immunoglobulin lambda light chain variable region (IGL) mRNA, partial cds
ATGGCCTGGTCCCCTCTCCTCCTCACCCTCATCGCTCTCTGCACAGGATCCTGGGCCCAGTCTGTGACTC
AGCCCGCCTCAGTGTCTGGGACCCTGGGCCAGACAGTCACCATCTCCTGCTCTGGAAGCAGCTCCAACAT
TGGGTATAGTTATAGTTATGTGGGCTGGTTCCAACAGATCCCAGGAACAGCCCCCAAAACCCTCATCTAT
GGTAATAACAAACGAGCCTCAGGGGTCCCAGATCGATTCTCTGGCTCCAAGTCTGGCAACACAGCCACCC
TGACCATCTCTGGGGTCCAGGCTGAGGACGAGGCCGATTATTACTGTGGTTCCTATGACAGCAGCAGTAG
TAGTGATGAATTCGGCGGC
>KR190545.1 Equus caballus clone IGLV76 immunoglobulin lambda light chain variable region (IGL) mRNA, partial cds
ATGGCCTGGTCCCCTCTCCTCCTCACCCTCATCGCTCTCTGCACAGGATCCTGGGCCCAGTCTGTGACTC
AGCCCGCCTCAGTGTCTGGGACCCTGGACCAGACAGTCACCATCTCCTGCTCTGGAAGCAGCTACAACAT
CGGGTCTAATTATAAGTATGTGTGCTGGTTCCAACAGATCCCAGGAACAGCCCCCAGAACCCTCATCTAT
GGTAATAACAAACGAGCCTCAGGGGTCCCAGATCGATTCTCTGGCTCCACGTCTGGCGACACAGCCACCC
TGACCATCTCTGGGGTCCAGGCTGAGGACGAGGCCGATTATTACTGTGCAGCCTATGACTCAAGCATCAA
TATGGATGTATTCGGCGGA
>KR190544.1 Equus caballus clone IGLV75 immunoglobulin lambda light chain variable region (IGL) mRNA, partial cds
ATGGCCTGGTCCCCGCTCCTTCTCACCTTCATCGCTCTCTGCACAGGATCCTGGGCCCAGTCTCTGACTC
AGCCCGCCTCAGTGTCTGGGACCCTGGGCCAGACAGTCACCATCTCCTGCTCTGGAAGCAGCTCCAATGT
TGGGTCTTATACTGGTTGGATGACCTGGTTCCAACAGATCCCAGGAACAGCCCCCAAAACTCTCATCTAT
GGGGGCACTAGTAGAGCGTCCGGGGTCCCCGATCGATTCTCTGCCTCCAGGTCTGGCAACACAGCCACCC
TGACCATCTCTGGGGTCCAGGCTGAGGACGAGGCTGATTATTACTGTTCATCACCAGACAAGAGCCCGAA
TACTTTCGTATTCGGCGGA
>KR190543.1 Equus caballus clone IGLV74 immunoglobulin lambda light chain variable region (IGL) mRNA, partial cds
ATGGCCTGGTCCCCTCTCCTCCTCACCCTCATCGCTCTCTGCACAGGATCCTGGGCCCAGTCTCTGACTC
AGCCCGCCTCAGTGTCTGGGACCCTGGGCCAGACAGTCACCATCTCCTGCTCTGGAAGCCTGTCCAACAT
CGGGAATATTGATAGTGCTGTGGCCTGGTACCAGCAGGTCCCAGGAACAGCCCCCAAAACCCTCATCTAT
GATACTGTCACACGGGCCTCAGGGGTCCCAGATCGGTTCTCTGGCTCCAAGTCTGGCAACACAGCCACCC
TGACCATCTCTGGAGTCCAGGCTGAGGACGAGGCCGATTATTACTGTGCAGCAGGAGACAGGAGTCTGAG
GATTGCTATATTCGGCGGA
>KR190542.1 Equus caballus clone IGLV73 immunoglobulin lambda light chain variable region (IGL) mRNA, partial cds
ATGGCCTGGTCCCCTCTCCTCCTCACCCTCATCGCTCTCTGCACAGGATCCTGGGCCCAGTCTCTGACTC
AGCCCGCCTCAGTGTCTGGGACCCTGGGCCAGACAGTCACCATCTCCTGCTCTGGAAGCAGCTCCAACAT

CGGGTATAGTTATAGTTATGTGGGCTGGTACCAACAGATCCCAGGAACAGCCCCCAAAACCCTCATCTAT
GATAATAACAAACGAGTCTCAGGGGTCCCAGATCGATTCTCTGGCTCCAAGTCTGGCAACACAGCCACCC
TGACCATCTCTGGGGTCCAGGCTGAGGACGAGGCCGATTATTATTGCTCAGCAGGAGACAGCAGTGGTAG
TGGTGACGTATTCGGCGGA
>KR190541.1 Equus caballus clone IGLV72 immunoglobulin lambda light chain variable region (IGL) mRNA, partial cds
ATGGCCTGGTCCCCTCTCCTCCTCACCCTCATCGCTCTCTGCACAGGATCCTGGGCCCAGTCTGTGACTC
AGCCCGCCTCAGTGTCTGGGACCCTGGGCCAGCCAGTCACCATCTCCTGCTCTGGAAGCAGCTCCAACAT
CGGAAGAAGCGGTAGTGCTGTGGGCTGGTACCAACAGGTCCCAGGAACAGCCCTCAAAACCCTCATCTAT
AATACTAACACACGAACCTCAGGGGTCCCAGATCGATTCTCTGGATCCAAGTCTGGCAACACAGCCACCC
TGACCATCTCTGGGGTCCAGGCTGAGGACGAGGCCGATTATTACTGTGCAGCAGGGGACAACAGCCTGGA
TAGTCTTATATTCGGCGGA
>KR190540.1 Equus caballus clone IGLV71 immunoglobulin lambda light chain variable region (IGL) mRNA, partial cds
ATGGCCTGGTCCCCTCTCCTCCTCACCCTCATCGCTCTCTGCACAGGATCCTGGGCCCAGTCTGTGACTC
AGCCCGCCTCAGTGTCTGGGACCCTGGGCCAGACAGTCACCATCTCCTGCTCTGGAAGCACCTCCAATAT
CGGGTACAGTCGTAGTTTTGTGGGCTGGTTCCAACAGATCCCAGGAACAGCCCCCAAAACTCTCATCTAT
TATGCCACTAGTAGAGCGTCCGGGGTCCCCGATCGATTCTCTGGCTCCAGGTCTGGCAACACAGCCACCC
TGACCATCTCTGGGGTCCAGGCTGAGGACGAGGGTGATTATTACTGCTCATCAGCAGACAGCAGCCTGAG
GAGTCTTGTATTCGGCGGA
>KR190539.1 Equus caballus clone IGLV70 immunoglobulin lambda light chain variable region (IGL) mRNA, partial cds
ATGGCCTGGTCCCCTCTCCTCCTCACCCTCATCGCTCTCTGCACAGGATCCTGGGCCCAGTCTCTGACTC
AGCCCGCCTCAGTGTCTGGGACCCTGGGCCAGACAGTCACCATCTCCTGCTCTGGAAGCAACTCCAACAT
CGGTTATAGTTATAGTAATGTGGGCTGGTACCAACAAATCCCAGGAACAGCCCCCAAAACCCTCATCTCT
GGTAATGACAAACGACGTTCAGGGGTCCCAGATCGATTCTCTGGCTCCAAGTCTGGCAACACAGCCACCC
TGACCATCTCTGGGGTCCAGGCTGAGGACGAGGCCGATTATTACTGCGGGACAGGAGACATTACTGGTAG
CAGTGTTGTATTTGGCGGA
>KR190538.1 Equus caballus clone IGLV69 immunoglobulin lambda light chain variable region (IGL) mRNA, partial cds
ATGGCCTTGTCCCCTCTCCTCCTCACCCTCATCGCTCTCTGCACAGGATCCTGGGCCCAGTCTCTGACTC
AGCCCGCCTCAGTGTCTGGGACCCTGGGCCAGACAGTCACCATCTCCTGCTCTGGAAGCAGCTCCAACAT
CGGGTATGTTGCTAGTGCTGTGGGCTGGTACCAACAGATCCCAGGAACAGCCCCCAAAACCCTCATCTAT
GAGGATAACAAACGAATGTCAGGGGTGCCAGATCGATTCTCTGGGTCCAAGTCCGGCAACACAGCCACCC
TGACCATCTCTGGGGTCCGGCCTGAGGACGAGGCCGATTATTACTGCTCAGCAGGAGACAGCAGTGGTAG
TAGTATGATATTCGGCGGA
>KR190537.1 Equus caballus clone IGLV68 immunoglobulin lambda light chain variable region (IGL) mRNA, partial cds
ATGGCCTGGTCCCCTCTCCTCCTCACCCTCATCGCTCTCTGCACAGGATCCTGGGCCCAGTCTCTGACCC
AGCCCGCCTCAGTGTCTGGGACCCTGGGCCAGACAGTCACCATCTCCTGCTCTGGAAGCAGCTCCAACAT
CGGGTTCAGTTATAGTGCTGTGGGCTGGTACCAACAGATCCCAGGAACAGCCCCCAAAACCCTCATCTAT
GGTAATAACAAACGAGCCTCAGGGGTCCCAGATCGATTCTCTGGCTCCAAGTCTGGCAACACAGCCACCC
TGACCATCTCTGGGGTCCAGGCTGAGGACGAGGCCGATTATCACTGTTCAGCAGGAGACAGCAGTGGTAG
TAGTGCTGTATTCGGCGGA
>KR190536.1 Equus caballus clone IGLV67 immunoglobulin lambda light chain variable region (IGL) mRNA, partial cds
ATGGCCTGGTCCCCTCTCCTCCTCACCCTCATCGCTCTCTGCACAGGATCCTGGGCCCAGTCTCTGACTC
AGCCCGCCTCAGTGTCTGGGACCCTGGGCCAGACAGTCACCATCTCCTGCTCTGGAAGAGTGTCCAACAT
CGGGTGGACAGCTGTGGGCTGGTACCAACAGGTCCCAGAAACAGCCCTCAAAACCCTCATCAGTGGCCTT
AACACACGGGCCTCAGGGGTCCCAGACCGATTCTCTGCCTCCAAGTCTGACAACACAGCCACCCTGACCA
TCTCTGGGGTCCAGGCTGAGGACGAGGCGATTTATTACTGTGCAGCAGGAGACAGCAGCCTGGGGACTAC
ACTATTCGGCGGA
>KR190535.1 Equus caballus clone IGLV66 immunoglobulin lambda light chain variable region (IGL) mRNA, partial cds
ATGGCCTGGTCCCCTCTCCTCCTCACCCTCATCGCTCTCTGCACAGGATCCTGGGCCCAGTCTCTGACTC

AGCCCGCCTCAGTGTCTGGGACCCTGGGCCAGGGGGTCACCATCTCCTGCTCTGGAAGCAGGTCCAACAT
CGGGAGAGAGGGCAGTGATGTGGGCTGGTATCAACAGGTCCCAGGAACAGCCCTCAAAACCCTCATCTAT
GATACTAACACGCGACCGTCAGGGGTCCCAGATCGATTCTTTGGCTCCAAGTCTGGCAACATAGCCACCC
TGACCATTTCTGGGGTCCAGGCTGAGGACGAGGCCGATTATTACTGTGCAACAGGAGACGAAACGCTGAG
GAGCACAGTATTCGGCGGA
>KR190534.1 Equus caballus clone IGLV65 immunoglobulin lambda light chain variable region (IGL) mRNA, partial cds
ATGGCCTGGTCCCCTCTCCTCCTCACCCTCATCGCTCTCTGCACAGGATCCTGGGCCCAGTCTCTGACTC
AGCCCGCCTCAGTGTCTGGGACCCTGGGCCAGACAGTCACCATCTCCTGCTCTGGAAGCAGCTCCAACAT
CGGTTTTAGTGTGAGTAGTGTGGGCTGGTACCAACAGGTCCCAGGAACACAGCTCAAAACCCTCATCTAT
GACACCGACACACGACACTCAGGGGTCCCAGATCGATTCTCTGGCACCAAGTCTGGCAACACAGCCACCC
TGACCATCTCTGGGGTCCAGGCTGAGGACGAGGCCGATTATTACTGTTCAGCAGGAGACGCCAACCTGAG
GAGTTTTGTATTCGGCGGA
>KR190533.1 Equus caballus clone IGLV64 immunoglobulin lambda light chain variable region (IGL) mRNA, partial cds
ATGGCCTGGGCTCCGTTCTTCCTCATCATCCTAGCTCACTGCACAGAGTCTTGGTCTGAGGTTGTGCTGA
CTCAGCCTCGCTCTGTATCAGAGTCACTGGGACAGAAGGCCACCATGTCCTGTACCCGCAGCAGCGGCAA
CATTGGAAGCAGCTATGTGTACTGGTACCAGCAGCGCCCGGGCAGTGCCCCCACCACCATGATCTATGAT
GATGACGAAAGACTCTCTGGGGTACCCGATGGGTTCTCAGGGTCCATTGACAGCTCATCCAACGCTGCCT
ATCCGACCCTCTCTGGGCTGCAGCCTGAGGACGAGGCCGACAACTACTGTCAGTCTTATGACAGCAGCTA
TAATGGTGCATTCGGCGGA
>KR190532.1 Equus caballus clone IGLV63 immunoglobulin lambda light chain variable region (IGL) mRNA, partial cds
ATGGCCTGGACCCCTCTCCTGCTCCCCCTCCTCACTCTCTGTATAGGTTCTGTGGTCTCCTTGGAGCTGA
CTCAGCCAGCTTCAGTTTCTGTGGCCTTAGGACAGACTGCCACGATCACCTGCCAGGGAGGAATCTTTGA
CAAGAAGTATGTGAACTGGTACCAGCAGAAGCCCGGCGGGACCCCTGTGATAGTGATTTATAAGGATAGT
GAGCGGCCCTCCGGGATCCCTGACAGATTCTCTAGCTCCAGCTCAGGGAACACAGCCACCCTGACCATCA
GCAGGGCCCAGGCAGAGGACGAGGCCGTCTATTACTGCCACTCACTAGATAGTGATAATGCTAGTGCATT
CGGCGGA
>KR190531.1 Equus caballus clone IGLV62 immunoglobulin lambda light chain variable region (IGL) mRNA, partial cds
ATGGCCTGGACCCCTCTCCTGCTCCCCCTCCTCACTCTCTGTATAGGTTCTGTGGTCTCCTTGGAGCTGA
CTCAGCCAGCTTCAGTTTCTGTGGCCTTAGGACAGACTGCCACGATCACCTGCCAGGGAGGAATCTTTGA
CAAGAAGTATGTGAACTGGTACCAGCAGAAGCCCGGCGGGACCCCTGTGATAGTGATTTATAAGGATAGT
GAGCGGCCCTCCGGGATCCCTGACAGATTCTCTAGCTCCAGCTCAGGGAACACAGCCACCCTGACCATCA
GCAGGGCCCAGGCAGAGGACGAGGCCGTCTATTACTGCCACTCACTAGATAGTGAGGAGGCTACTATATT
CGGCGGA
>KR190530.1 Equus caballus clone IGLV61 immunoglobulin lambda light chain variable region (IGL) mRNA, partial cds
ATGGCCTGGACCCCTCTCCTGCTCCCCCTCCTCACTCTCTGTATAGGTTCTGTGGTCTCCTTGGAGCTGA
CTCAGCCAGCTTCAGTTTCTGTGGCCTTAGGACAGACTGCCACGATCACCTGCCAGGGAGGAATCTTTGA
CAAGAAGTATGTGAACTGGTACCAGCAGAAGCCCGGCGGGACCCCTGTGATAGTGATTTATAAGGATAGT
GAGCGGCCCTCCGGGATCCCTGACAGATTCTCTAGCTCCAGCTCAGGGAACACAGCCACCCTGACCATCA
GCAGGGCCCAGGCAGAGGACGAGGCCGTCTATTACTGCCACTCATTAGATACAGATAATGCTGCTGTATT
CGGCGGA
>KR190529.1 Equus caballus clone IGLV60 immunoglobulin lambda light chain variable region (IGL) mRNA, partial cds
ATGGCCTGGACCCCTCTCTTGTTAGCCTTCCTCTCTCTCTGCACAGGTCCTGTTGTCTCTTCTGCAGTGA
CTCAGCCATCTTCAGTGTCTGTGGCCTTGGGACAGAGAGCCACCCTCACCTGCCAGGGAAGCAACTTTGA
ATTTTTTTCTCCTAGCTGGTACCAGCAGAAGCCAGGCCAGGCCCCTGTACTGCTCATCAATATTAATAAT
GAGCGCCACTCAGGGATCCCTGAACGATTCTCCGGCTCCAGCTCAGGAGACACGTCCACACTGACCATCA
GTGGGGCCCAGGCTGAGGACGAGGCTGACTATTACTGTCTGGCAGTAGATGTTCTTAGTTCTGTTATCTT
CGGCGGC
>KR190528.1 Equus caballus clone IGLV59 immunoglobulin lambda light chain variable region (IGL) mRNA, partial cds
ATGGCCTGGACCCCTCTCTTGTTAGCCTTCCTCACTCTCTGCACAGGTCCCGTGGCCTCTTCTAAGCTGA

CTCAGCCATCTTCAGTGTCTGTGGCCTTGGGACAGACGGCCACCATCACCTGCAAGGGAGGCGACTTTGA
AAGTTTTGTGGGTAGCTGGTACCAGCAGAAGCCAGGCCAGGCCCCTGTGCTGGTCATCGATGCTGATAAT
GAGCGGCCCTCAGGGATCCCTGAACGATTCTCTGGCTCCAACTCAGGAGACACAGCCACGCTGACCATCA
GCGGGGCCCAGGCTGAGGACGAGGCTGACTATTACTGTCTGGCAGTAGATGCTCTTAGTTCTGAAACTAT
CTTCGGCGGC
>KR190527.1 Equus caballus clone IGLV58 immunoglobulin lambda light chain variable region (IGL) mRNA, partial cds
ATGGCCTGGTCCCCTCTCCTCCTCACCCTCATCGCTCTCTGCACAGGATCCTGGGCCCAGTCTCTGACCC
AGCCCGCCTCAGTGTCTGGGACCCTGGGCCAGACAGTCACCATCTCCTGCTCTGGAAGCAGCTCCAACAT
CGGGTATAGTCGTAGTTATGTGGGCTGGTTCCAACAGATCCCAGGAACAGCCCCCAAAACCCTCATCTAT
GCTACTAACAAACGAGCCTCAGGGGTCCCAGATCGATTCTCTGGCTCCAAGTCTGGCAACACAGCCACCC
TGACCATCACTGGGCTTCAGGCTGAGGACGAGGCCGATTATTACTGTGGTTCCTATTACAGCAGTGATAG
TAGTGGTGCATTCGGCGGA
>KR190526.1 Equus caballus clone IGLV57 immunoglobulin lambda light chain variable region (IGL) mRNA, partial cds
ATGGCCTGGTCCCCTCTCCTCCTCACCCTCATCGCTCTCTGCACAGGATCCTGGGCCCAGTCTCTGACCC
AGCCCGCCTCAGTGTCTGGGACCCTGGGCCAGACAGTCACCATCTCCTGCTCTGGAAACAGCTCCAACAT
CGGAAAGAGTGGGAGTGCTGTGGGCTGGTACCAACAGATCCCAGGAACAGCCCCCAAAACCCTCATCTAT
GGTAATAACAAACGAGCCTCAGGGGTCCCAGATCGATTCTCTGGCTCCAAGTCTGGCAACACAGCCACCC
TGACCATCTCTGGGCTTCAGGCTGAGGACGAGGCCGATTATTACTGTGGTTCCTATTACAACAGTGATAG
TGCTGTATTCGGCGGA
>KR190525.1 Equus caballus clone IGLV56 immunoglobulin lambda light chain variable region (IGL) mRNA, partial cds
ATGGCCTGGTCCCCTCTCCTCCTCACCCTCATCGCTCTCTGCACAGGATCCTGGGCCCAGTCTGTGACTC
AGCCCGCCTCAGTGTCTGGGACCCTGGGCCAGACAGTCACCATCACCTGCACTGGAAGCAGCTCCAACAT
AGTTAATTATGTGGGCTGGTACCAACAGATCCCAGGAACAGCCCCCAAAACCCTCATCTATAATAATGAC
AAACGACCCTCAGGGGTCCCAGATCGATTCTCTGGCTCCAAGTCTGGCAACACAGCCACCCTGACCATCT
CTGGGGTCCAGGCTGAGGACGAGGCCGATTATTACTGTGGTTCCTATGACAGCAGCAGTAGTAGTGCTGT
ATTCGGCGGA
>KR190524.1 Equus caballus clone IGLV55 immunoglobulin lambda light chain variable region (IGL) mRNA, partial cds
ATGGCCTGGTCCCCTCTCCTCCTCACCCTCATCGCTCTCTGCACAGGATCCTGGGCCCAGTCTGTGACTC
AGCCCGCCTCAGTGTCTGGGACCCTGGGCCAGACAGTCACCATCACCTGCACTGGAAGCAGCTCCAACAT
AGTTGCTTATGTGGGCTGGTACCAACAGATCCCAGGAACAGCCCCCAAAACCCTCATCTATGCTAATAAC
AAACGAGCCTCAGGGGTCCCAGATCGATTCTCTGGCTCCAAGTCTGGCAGCACAGCCACCCTGACCATCA
CTGGGCTCCAGGCTGAGGACGAGGCCGATTATTACTGTGGTACCTCTAGCAGCAGTGGTGGTGCATTCGG
CGGA
>KR190523.1 Equus caballus clone IGLV54 immunoglobulin lambda light chain variable region (IGL) mRNA, partial cds
ATGGCCTGGTCCCCTCTCCTCCTCACCCTCATCGCTCTCTGCACAGGATCCTGGGCCCAGTCTGTGACTC
AGCCCGCCTCAGTGTCTGGGACCCTGGGCCAGACAGTCACCATCACCTGCACTGGAAGCAGCATAGACAT
AGTTGCTTATGTGGGCTGGTACCAACAGATCCCAGGAACAGCCCCCAAAACCCTCATCTATGTTAATAAC
AAACGAGCCTCAGGACTCCCAGATCGATTCTCTGGCTCCAAGTCTGGCAGCACAGCCACCCTGACCATCA
CTGGGCTCCAGGCTGAGGACGAGGCCGATTATTACTGTGGTACCTCTAGCATCAGTGGTAGTAATGCTGT
ATTCGGCGGA
>KR190522.1 Equus caballus clone IGLV53 immunoglobulin lambda light chain variable region (IGL) mRNA, partial cds
ATGGCCTGGTCCCCTCTCCTCCTCACCCTCATCGCTCTCTGCACAGGATCCTGGGCCCAGTCTGTGACTC
AGCCCGCCTCAGTGTCTGGGGCCCTGGGCCAGACAGTCACCATCACCTGCACTGGAAGCAGCTCCGACAT
AGTTGCTTATGTGGCCTGGTACCAACAGATCCCAGGAACAGCCCCCAAAACCCTCATCTATGCTGATAAC
AAACGAGCCTCAGGGGTCCCAGATCGATTCTCTGGCTCCAAGTCTGGCAACACAGCCACCCTGACCATCT
CTGGGGTCCAGGCTGAGGACGAGGCCGATTATTACTGTGGTTCCTATGACAGCAGCAGTAGTAGTGCTGT
ATTCGGCGGA
>KR190521.1 Equus caballus clone IGLV52 immunoglobulin lambda light chain variable region (IGL) mRNA, partial cds

ATGGCCTGGTCCCCTCTCCTCCTCACCCTCATCGCTCTCTGCACAGGATCCTGGGCCCAGTCTGTGACTC
AGCCCGCCTCAGTGTCTGGGACCCTGGGCCAGACAGTCACCATCACCTGCACTGGAAGCAGCTCCAACAT
AGTTGCTTATGTGGGCTGGTACCAACAGATCCCAGGAACAGCCCCCAAAACCCTCATCTATGGTAATAAC
AAACGAGCCTCAGGGGTCCCAGATCGATTCTCTGGCTCCAAGTCTGGCAACACAGCCACCCTGACCATCT
CTGGGGTCCAGGCTGAGGACGAGGCCGATTATTACTGTGGTTCCTATGACAGCAGCAGTAGTAGTGATCA
TATCTTCGGCGGC
>KR190520.1 Equus caballus clone IGLV51 immunoglobulin lambda light chain variable region (IGL) mRNA, partial cds
ATGGCCTGGTCCCCTCTCCTCCTCACCCTCATCGCTCTCTGCACAGGATCCTGGGCCCAGTCTGTGACTC
AGCCCGCCTCAGTGTCTGGGACCCTGGGCCAGACAGTCACCATCACCTGCACTGGAAGCAGCTCCAACAT
AGTTGCTTATGTGGGCTGGTACCAACAGATCCCAGGAACAGCCCCCAAAACCCTCATCTATGCTAATAAC
AAACGAGCCTCAGGGGTCCCAGATCGATTCTCTGGCTCCAAGTCTGGCAGCACAGCCACCCTGACCATCA
CTGGGCTCCAGGCTGAGGACGAGGCCGATTATTACTGTGGTACCTCTAGCAGCAGTGGTAGTGCTGTATT
CGGCGGA
>KR190519.1 Equus caballus clone IGVL50 immunoglobulin lambda light chain variable region (IGL) mRNA, partial cds
ATGGCCTGGTCCCCTCTCCTCCTCACCCTCATCGCTCTCTGCACAGGATCCTGGGCCCAGTCTCTGACCC
AGCCCGCCTCAGTGTCTGGGACCCTGGGCCAGACAGTCACCATCTCCTGCTCTGGAAGCAGCTCCAACAT
CGGGAGTGGTCATGTGTCCTGGTACCAACAGATCCCAGGAACAGCCCCCAAACTCCTCATCTATTCTTCC
GCTAGCAGGGCTTCCGGGGTCCCCGATCGATTCTCTGGCTCCAGGTCTGGCAACACAGCCACCCTGACCA
TCTCTGGGGTCCAGGCTGAGGACGAGGCTGATTATTACTGCTCATCAGCAGACAGCAGCCTGAGGAGTGT
TTTATTCGGCGGA
>KR190518.1 Equus caballus clone IGLV49 immunoglobulin lambda light chain variable region (IGL) mRNA, partial cds
ATGGCCTGGTCCCCTCTCCTCCTCACCCTCATCGCTCTCTGCACAGGATCCCGGGCCCAGTCTGTGACTC
AGCCCGCCTCAGTGTCTGGGACCCTGGGCCAGACAGTCACCATCTCCTGCTCTGGAAGCAGCTCCAACAT
CGGGAGTGGTCATGTGTCCTGGTACCAACAGATCCCAGGAACAGCCCCCAAACTCCTCATCTATTCTTCC
GCTAGCAGGGCTTCCGGGGTCCCCGACCGATTCTCTGGCTCCAGGTCTGGCAACACAGCCACCCTGACCA
TCTCTGGGCTCCAGGCTGAGGATGAGGCCGATTATTACTGTGGTACATTGTATAGTAGTTGGAGTAGTGA
TGCTGTATTCGGCGGA
>KR190517.1 Equus caballus clone IGLV48 immunoglobulin lambda light chain variable region (IGL) mRNA, partial cds
ATGGCCTGGTCCCCTCTCCTCCTCACCCTCATCGCTCTCTGCACAGGATCCCGGGCCCAGTCTGTGACTC
AGCCCGCCTCAGTGTCTGGGACCCTGGGCCAGACAGTCACCATCTCCTGCTCTGGAAGCAGCTCCAACAT
CGGGAGTGGTCATGTGTCCTGGTACCAACAGATCCCAGGAACAGCCCCCAAACTCCTCATCTATTCTTCC
GCTAGCAGGGCTTCCGGGGTCCCCGACCGATTCTCTGGCTCCAGGTCTGGCAACACAGCCACCCTGACCA
TCTCTGGGCTCCAGGCTGAGGATGAGGCCGATTATTACTGTGGTACATTGTACAGCAGTTGGAGTAGTGT
CTTCGGCGGC
>KR190516.1 Equus caballus clone IGLV47 immunoglobulin lambda light chain variable region (IGL) mRNA, partial cds
ATGGCCTGGTCCCCTCTCCTCCTCACCCTCATCGCTCTCTGCACAGGATCCTGGGCCCAGTCTCTGACTC
AGCCCGCCTCAGTGTCTGGGACCCTGGGCCAGACAGTCACCATCTCCTGCACTGGAAGCAGCTCCAGCAT
AGGTTCTTATATGGGCTGGTACCAACAGATCCCAGGGACAGCCCCCAAACTCCTCATTTATAGTGCCAGT
TCTCGAGCCTCAGGGATCCCTGATCGCTTCTCTGGCTCCAAGTCTGGGAACACGGCCTCTCTGACCATCT
CGGGGCTCCAGGCTGAGGACGAGGCCGATTATTACTGTGGTACCTCTAGCAGCAGTGGTAGTAGTGATGC
ATTCGGCGGA
>KR190515.1 Equus caballus clone IGLV46 immunoglobulin lambda light chain variable region (IGL) mRNA, partial cds
ATGGCCTGGTCCCCTCTCCTCCTCACCCTCATCGCTCTCTGCACAGGATCCTGGGCCCAGTCTCTGACTC
AGCCCGCCTCAGTGTCTGGGACCCTGGGCCAGACAGTCACCATCTCCTGCACTGGAAGCAGCTCCAACAT
AGGTGCTTATGTGGACTGGTACCAACAGATCCCAGGAACAGCCCCCAAAACCCTCATCTATGGTAATAAC
AAACGAGCCTCAGGGGTCCCAGATCGATTCTCTGGCTCCAAGTCTGGCAACACAGCCACCCTGACCATCT
CTGGGGTCCAGGCTGAGGACGAGGCCGATTATTATTGTGGTTCCTATGACAGCAGCAGTAGTATTGTATT
CGGCGGA
>KR190514.1 Equus caballus clone IGLV45 immunoglobulin lambda light chain variable region (IGL) mRNA, partial cds

ATGGCCTGGTCCCCTCTCCTCCTCACCCTCATCGCTCTCTGCACAGGATCCTGGGCCCAGTCTCTGACTC
AGCCCGCCTCAGTGTCTGGGACCCTGGGCCAGACAGTCACCATCTCCTGCACTGGAAGCAGCTCCAGCAT
CACTTCTTATATGGGCTGGTACCAACAGATCCCAGGGACAGCCCCCAAAACCCTCATCTATGCTACTAAC
AAACGAGCCTCAGGGGTCCCAGATCGATTCTCTGGCTCCAAGTCTGGCAACACAGCCACCCTGACCATCA
CTGGGCTTCAGGCTGAGGACGAGGCCGATTATTACTGTGGTTCCTATTACAGCAGTGATATCTTCGGCGG
C
>KR190513.1 Equus caballus clone IGLV44 immunoglobulin lambda light chain variable region (IGL) mRNA, partial cds
ATGGCCTGGTCCCCTCTCCTCCTCACCCTCATCGCTCTCTGCACAGGATCCCGGGCCCAGTCTGTGACTC
AGCCTGCCTCAGTGTCTGGGACCCTGGGCCAGACAGTCACCATCTCCTGCTCTGGAAGCAGCTCCAACGT
TGGGAGTGGTTATGTGTCCTGGTACCAACAGATCCCAGGAACAGCCCCCAAACTCCTCATCTATTATGCC
ACTAGTAGGGCTTCCGGGGTCCCCGACCGATTCTCTGGCACCAGGTCTGGCAACACAGCCACCCTGACCA
TCTCTGGGCTCCAGGCTGAGGATGAGGCCGATTATTACTGTGGTGCCTCTGGCAGCAGTTGGAGTAGTGC
TGTATTCGGCGGA
>KR190512.1 Equus caballus clone IGLV43 immunoglobulin lambda light chain variable region (IGL) mRNA, partial cds
ATGGCCTGGTCCCCTCTCCTCCTCACCCTCATCGCTCTCTGCACAGGATCCCGGGCCCAGTCTGTGACTC
AGCCTGCCTCAGTGTCTGGGACCCTGGGCCAGACAGTCACCATCTCCTGCTCTGGAAGCAGCTCCAACGT
TGGGAGTGGTTATGTGTCCTGGTACCAACAGATCCCAGGAACAGCCCCCAAACTCCTCATCTATTATGCC
ACTAGTAGGGCTTCCGGGGTCCCCGACCGATTCTCTGGCACCAGGTCTGGCAACACAGCCACCCTGACCA
TCTCTGGGCTCCAGGCTGAGGATGAGGCCGATTATTACTGTGGTGCCTCTGGCAGCAGTTGGAGTAGTGC
ATTCGGCGGA
>KR190511.1 Equus caballus clone IGLV42 immunoglobulin lambda light chain variable region (IGL) mRNA, partial cds
ATGGCCTGGTCCCCTCTCCTCCTCACCCTCATCGCTCTCTGCACAGGATCCCGGGCCCAGTCTGTGACTC
AGCCTGCCTCAGTGTCTGGGACCCTGGGCCAGACAGTCACCATCTCCTGCTCTGGAAGCAGCTCCAACGT
TGGGACTGGTTATGTGTCCTGGTACCAACAGATCCCAGGAACAGCCCCCAAACTCCTCATCTATAGTGCC
ACTAGTAGGGCTTCCGGGGTCCCCGACCGATTCTCTGGCACCAGGTCTGGCAACACAGCCACCCTGACCA
TCTCTGGGCTCCAGGCTGAGGATGAGGCTGATTATTACTGCAAATCAGTAGACAGCAGCCTGAACACTGT
TGTATTCGGCGGA
>KR190510.1 Equus caballus clone IGLV41 immunoglobulin lambda light chain variable region (IGL) mRNA, partial cds
ATGGCCTGGTCCCCTCTCCTCCTCACCCTCATCGCTCTCTGCACAGGATCCTGGGCCCAGTCTCTGACTC
AGCCCGCCTCAGTGTCTGGGACCCTGGGCCAGACAGTCACCATCTCCTGCTCTGGAAGCAGCTCCAACAT
CGGGAATAGTGCTAGTTCTGTGGGCTGGTTCCAACAGATCCCAGGAACAGCCCCCAAAACCCTCATCTAT
GGTGATAACCAACGAGCCTCAGGGGTCCCAGATCGATTCTCTGGCTCCAAGTCTGGCAACACAGCCACCC
TGACCATCTCTGGGGTCCAGGCTGAGGACGAGGCCGATTATTACTGTGGTTTGTATGACAGCGCGGCTAG
TGTTGTATTCGGCGGA
>KR190509.1 Equus caballus clone IGLV40 immunoglobulin lambda light chain variable region (IGL) mRNA, partial cds
ATGGCCTGGTCCCCTCTCCTCCTCACCCTCATCGCTCTCTGCACAGGATCCTGGGCCCAGTCTCTGACTC
AGCCCGCCTCAGTGTCTGGGACCCTGGGCCAGACAGTCACCATCTCCTGCTCTGGAAGCAGCTCCAACAT
CGGGTATAGTTATAGTGCTGTGGGCTGGTACCAACAGATCCCAGGAACAGCCCCCAAAACCCTCATCTAT
GGTAATAACAAACGAGCCTCAGGGGTCCCAGATCGATTCTCTGGCTCCAAGTCTGGCAACACAGCCACCC
TGACCATCTCTGGGGTCCAGGCTGAGGACGAGGCCGATTATTACTGTGCTTCCTATGACAGCAGCAGTAG
TGTTGCATTCGGCGGA
>KR190508.1 Equus caballus clone IGLV39 immunoglobulin lambda light chain variable region (IGL) mRNA, partial cds
ATGGCCTGGTCCCCTCTCCTCCTCACCCTCATCGCTCTCTGCACAGGATCCTGGGCCCAGTCTGTGACTC
AGCCCGCCTCAGTGTCTGGGACCCTGGGCCAGACAGTCACCATCTCCTGCTCTGGAAGCAGCTCCAACAT
CGGGTATAGTTATAGTGCTGTGTACTGGTTCCAACAGATCCCAGGAACAGCCCCCAAAACCCTCATCTAT
GGTAATAACGGACGAGCCTCAGGGGTCCCAGATCGATTCTCTGGCTCCAAAAGTGGCAACACAGCCACCC
TGACCATCTCTGGGGTCCAGGCTGAGGACGAGGCCGATTATTACTGTGGTTCCTATGACAAAAGTAGTGA
TAGTCTTGTGTTCGGCGGA

>KR190507.1 Equus caballus clone IGLV38 immunoglobulin lambda light chain variable region (IGL) mRNA, partial cds
ATGGCCTGGTCCCCTCTCCTCCTCACCCTCATCGCTCTCTGCACAGGATCCTGGGCCCAGTCTGTGACTC
AGCCCGCCTCAGTGTCTGGGACCCTGGGCCAGACAGTCACCATCTCCTGCTCTGGAAGCAGCTCCAACAT
CGGCGATGATTATAGTTATGTGGGCTGGTTCCAACAGATCCCAGGAACAGCCCCCAAAACCCTCATCTAT
GCTGATAACAATCGAGCCTCAGGGGTCCCAGATCGATTCTCTGGCTCCAAGTCTGGCAACACAGCCACCC
TGACCATCTCTGGGGTCCAGGCTGAGGACGAGGCCGATTATTACTGTGGTTCCTATGACAGCAGCAGTGC
TGTATTCGGCGGA
>KR190506.1 Equus caballus clone IGLV37 immunoglobulin lambda light chain variable region (IGL) mRNA, partial cds
ATGGCCTGGTCCCCTCTCCTCCTCACCCTCATCGCTTTCTGCACAGGATCCTGGGCCCAGTCTGTGACTC
AGCCCGCCTCAGTGTCTGGGACCCTGGGCCAGACAGTCACCATCTCCTGCTCTGGAAGCAGCTCCAACAT
CGGACTTAGTTATATTTATGTGGGCTGGTTCCAACAGATCCCAGGAACAGCCCCCAAAACCCTCATCTAT
GGTCGTAACCAACGAGCCTCAGGGGTCCCAGATCGATTCTCTGGCTCCAAGTCTGGCAACACAGCCACCC
TGACCATCTCTGGGGTCCAGGCTGAGGACGAGGCCGATTATTACTGTGGTTCCTATGACAGCAGCAGTAA
TAGTGGAGTATTCGGCGGA
>KR190505.1 Equus caballus clone IGLV36 immunoglobulin lambda light chain variable region (IGL) mRNA, partial cds
ATGGCCTGGTCCCCGCTCCTTCTCACCTTCATCGCTCTCTGCACAGGATCCTGGGCCCAGTCTCTGACTC
AGCCCGCCTCAGTGTCTGGGACCCTGGGCCAGACAGTCACCATCTCCTGCTCTGGAAGCAGCTCCAACAT
TGGCGATAGTGATAGTTATGTGAGCTGGTTCCAACAGATCCCAGGAACAGCCCCCAAAACTCTCATCTAT
TATGCCACTAGTAGAGCGTCCGGGGTCCCCGATCGATTCTCTGGCTCCAGGTCTGGCAACACAGCCACCC
TGACCATCTCTGGGGTCCAGGCTGAGGACGAGGCTGATTATTACTGCTCATCAGCAGACAGCAGCCTGAG
GAGTGTTTTATTCGGCGGA
>KR190504.1 Equus caballus clone IGLV35 immunoglobulin lambda light chain variable region (IGL) mRNA, partial cds
ATGGCCTGGTCCCCTCTCCTCCTCACCCTCATCGCTCTCTGCACAGGATCCTGGGCCCAGTCTCTGACTC
AGCCCGCCTCAGTGTCTGGGACCCTGGGCCAGACAGTCACCATCTCCTGCTCTGGAAGCAGCTCCAACAT
CGGGTATAGTAGTAGTGCTGTGGGCTGGTACCAACAGATCCCAGGAACAGCCCCCAAAACCCTCATCTAT
AGTAATAACAAACGAGCCTCAGGGGTCCCAGATCGATTCTCTGGCTCCAAGTCTGGCAACACAGCCACCC
TGACCATCTCTGGGGTCCAGGCTGAGGACGAGGCCGATTATTACTGCTCAACAGGAGACAGTAGTGGTGC
TGAAATCTTCGGCGGC
>KR190503.1 Equus caballus clone IGLV34 immunoglobulin lambda light chain variable region (IGL) mRNA, partial cds
ATGGCCTGGTCCCCTCTCCTCCTCACCCTCATCGCTCTCTGCACAGGATCCTGGGCCCAGTCTCTGACCC
AGCCCGCCTCAGTGTCTGGGACCCTGGGCCAGACAGTCACCATCTCCTGCTCTGGAAGCAGCTCCAACAT
CGGACATCGTTATGGTTTCGTGGGCTGGTACCAACAGATCCCAGGAACAGCCCCCAAAACCCTCATCAAT
ACAAATAACAAACGAACCTCAGGGGTCCCAGATCGATTCTCTGGCTCCAAGTCTGGCAACACAGCCACCC
TGACCATCTCTGGGGTCCAGGCTGAGGACGAGGCCGATTATTACTGCTCAGCAGGAGACAGCAGTGGTAG
TAGTGCTGTATTCGGCGGA
>KR190502.1 Equus caballus clone IGLV33 immunoglobulin lambda light chain variable region (IGL) mRNA, partial cds
ATGGCCTGGGCTCTGCTCCTCATCACCCTCCTCACTCAGGGCACAGGGTCCTGGGCGCAGTCTGCCCTGA
CTCAGCCTGCGTCAGTCTCCGGGACTCTGGGACAGTCGGTCACCATCACCTGTGCTGGAAGCACGGGATC
TTATAAATATATTTCCTGGTACCAACAGCACCCAGGCACAGCCCCCAAACTCCTCATTTATAATGGGAAT
AATCGGGCCTCAGGGATCCCTGATCGCTTCTCCGGCTCCACGTCTGGCAACACGATGTCCCTGACCATCT
CTGGGCTCCAGGCTGAGGACGAGGCTGATTATTACTGTCACGCGTATGTTGGCAGTTACAATTATATCTT
CGGCGGC
>KR190501.1 Equus caballus clone IGLV32 immunoglobulin lambda light chain variable region (IGL) mRNA, partial cds
ATGGCCTGGTCCCCTCTCCTCCTCACCCTCATCGCTCTCTGCACAGGATCCTGGGCCCAGTCTGTGACTC
AGCCCGCCTCAGTGTCTGGGACCCTGGGCCAGACAGTCACCATCTCCTGCTCTGGAAGCAGCTCCAACAT
CGGGTATAGTGCTATCACTGTGGGCTGGTACCAACAGGTCCCAGGAACAGCCCTCAAAACCCTCATCTAT
AATACTAACACACGAGCCTCAGGGGTCCCAGATCGATTCTCTGGCTCCAAGTCTGGCAACACAGCCACCC
TGACCATCTCTGGGCTTCAGGCTGAGGACGAGGCCGATTATTACTGTGGTTCCTCTTACAGCAGTGATGC
TTTTGTATTCGGCGGA

>KR190500.1 Equus caballus clone IGLV31 immunoglobulin lambda light chain variable region (IGL) mRNA, partial cds
ATGGCCTGGTCCCCTCTCCTCCTCACCCTCATCGCTCTCTGCACAGGATCCTGGGCCCAGTCTCTGACTC
AGCCCGCCTCAGTGTCTGGGACCCTGGGCCAGACAGTCACCATCTCCTGCTCTGGAAGCAGCTCCAACAT
CGGGTATAGTTATAGTGCTGTGGGCTGGTACCAACAGATCCCAGGAACAGCCCCCAAAACCCTCATCTAT
GGTAATGACAAAAGAGCCTCAGGGGTTCCAGGACGATTCTCTGGCTCCAAGTCTGGCAACACAGCCACCC
TGACCATTTCTGGGGTCCAGGCTGAGGACGAGGCCGATTATTATTGTGGTTCCTGGGACGAGACCGCCCT
TTTTTTTCCATTCGGCGGA
>KR190499.1 Equus caballus clone IGLV30 immunoglobulin lambda light chain variable region (IGL) mRNA, partial cds
ATGGCCTGGTCCCCTCTCCTCCTCACCCTCATCGCTCTCTGCACAGGATCCTGGGCCCAGTCTGTGACTC
AGCCCGCCTCAGTGTCTGGGACCCTGGGCCAGACAGTCACCATCACCTGCACTGGAAGCAGCTCCAACGA
AGTTGGTTGGGTGGGCTGGTACCAACAGATCCCAGGAACAGCCCCCAAAACCCTCATCTATGATATTACT
AAACGCGCCTCAGGGGTCCCAGATCGATTCTCTGGCTCCAAGTCTGGCAACACAGCCACCCTGACCATCT
CTGGGGTCCAGGCTGAGGACGAGGCCGATTATTACTGTTGCGCCTATGACAGAAGCAGTAGTAGTTCTGT
ATTCGGCGGA
>KR190498.1 Equus caballus clone IGVL29 immunoglobulin lambda light chain variable region (IGL) mRNA, partial cds
ATGGCCTGGTCCCCTCTCCTCCTCACCCTCATCGCTCTCTGCACAGGATCCTGGGCCCAGTCTGTGACTC
AGCCCGCCTCAGTGTCTGGGACCCTGGGCCAGACAGTCACCATCACCTGTACTGGAAGCAGCTCCAACAT
AGTTGCTTATGTGGGCTGGTACCAACAGATCCCAGGAACAGCCCCCAAAACCCTCATCTATGATAACGAC
AAACGAGCCTCAGGGGTCCCAGATCGATTCTCTGGCTCCAAGTCTGGCAGCACAGCCACCCTGACCATCA
CGGGGCTCCAGGCTGAGGACGAGGCCGATTATTACTGTGGTACCTCTAGCACGAGTGGTTCTGTATTCGG
CGGA
>KR190497.1 Equus caballus clone IGLV28 immunoglobulin lambda light chain variable region (IGL) mRNA, partial cds
ATGGCCTGGTCCCCTCTCCTCCTCACCCTCATCGCTCTCTGCACAGGATCCTGGGCCCAGTCTGTGACTC
AGCCCGCCTCAGTGTCTGGGACCCTGGGCCAGACAGTCACCATCACCTGCACTGGAAGCAGCTCCAACAT
AGTTGCTTATGTGGCCTGGTACCAACAGATCCCAGGAACTGCCCCCAAAACCCTCTTCTATAATAATAAC
AAGCGAGCCTCAGGGGTCCCAGATCGATTCTCTGGCTCCAAGTCTGGCAACACAGCCACCCTGACCATCT
CTGGGGTCCTGCATGAGGACGAGGCCGATTATTACTGCTCAGCAGGAGACAGTAGTGGTAGTACTGAATT
CGGCGGA
>KR190496.1 Equus caballus clone IGLV27 immunoglobulin lambda light chain variable region (IGL) mRNA, partial cds
ATGGCCTGGTCCCCTCTCCTCCTCACCCTCATCGCTCTCTGCACAGGATCCTGGGCCCAGTCTGTGACTC
AGCCCGCCTCAGTGTCTGGGACCCTGGGCCAGACAGTCACCATCACCTGCACTGGAAGCAGTTCCGTGGC
TGTGGGCTGGTACCAACAGATCCCAGGAACAGCCCCCAAAACCCTCATCTATGCTGACGACAAACGAGCC
TCAGGGGTCCCAGATCGATTCTCTGGTTCCAGGTCTGGCAGCACAGCCACCCTGACCATCACTGGGCTCC
AGGCTGAGGACGAGGCCGATTATTACTGTGGTTCCTCTGCTGCATTCGGCGGA
>KR190495.1 Equus caballus clone IGLV26 immunoglobulin lambda light chain variable region (IGL) mRNA, partial cds
ATGGCCTGGTCCCCTCTCCTCCTCACCCTCATCGCTCTCTGCACAGGATCCTGGGCCCAGTCTGTGACTC
AGCCCGCCTCAGTGTCTGGGACCCTGGGCCAGACAGTCACCATCACCTGCACTAGAAGCAGCTCCGACAT
GTTTGCTTATGTGGGCTGGTACCAACAGATCCCAGGAACAGCCCCCAAAACCCTCATCTATACTAATAAC
AAACGAGCCTCAGGGGTCCCAGATCGATTCTCTGGCTCCAAGTCTGGCAGCACAGCCACCCTGACCATCA
CTGGGCTCCAGGCTGAGGACGAGGCCGATTATTACTGTACTTCCTATGACAGCAGCAGTGAGAGTGCTGT
ATTCGGCGGA
>KR190494.1 Equus caballus clone IGLV25 immunoglobulin lambda light chain variable region (IGL) mRNA, partial cds
ATGGCCTGGTCCCCTCTCCTCCTCACCCTCATCGCTCTCTGCACAGGATCCTGGGCCCAGTCTGTGACTC
AGCCCGCCTCAGTGTCTGGGACCCTGGGCCAGACAGTCACCATCACCTGCACTGGAAGCAGCTCCAACAT
AGTTGCTTTTGTGGGCTGGTACCAACAGATCCCAGGAACAGCCCCCAAAACCCTCATCTATAATAATAAC
AATCGAGCCTCAGGGGTCCCAGATCGATTCTCTGGCTCCAAGTCTGGCAGCACAGCCACCCTGACCATCA
CTGGGCTCCAGGCTGAGGACGAGGCCGATTATTACTGTGGTACCTCTAGCACGGGTGGTGAAACTAGTGG
CTTCGGCGGA

>KR190493.1 Equus caballus clone IGLV24 immunoglobulin lambda light chain variable region (IGL) mRNA, partial cds
ATGGCCTGGTCCCCTCTCCTCCTCACCCTCATCGCTCTCTGCACAGGATCCTGGGCCCAGTCTGTGACTC
AGCCCGCCTCAGTGTCTGGGACCCTGGGCCAGACAGTCACCATCACCTGCACTGGAAGCATGTCAGAGTT
TGTTGAAAGTGTGGCCTGGTACCAACACATCCCAGGAACAGCCCCCAAAGCCCTCATCTATGCTAATAAC
AAACGAGCCTCAGGGGTCCCAGATCGATTCTCTGGCTCCAAGTCTGCCAGCACAGCCACCCTGACCATCA
CTGGGCTCCAGGCTGAGGACGAGGCCGATTATCACTGTGGTACCTCTACCGGCACTGGTTATGACTTCGG
CGGC
>KR190492.1 Equus caballus clone IGLV23 immunoglobulin lambda light chain variable region (IGL) mRNA, partial cds
ATGGCCTGGTCCCCTCTCCTCCTCACCCTCATCGCTCTCTGCACAGGATCCTGGGCCCAGTCTCTGACTC
AGCCCGCCTCAGTGTCTGGGACCCTGGGCCAGACAGTCACCATCTCCTGCACTGGAAGCATGTCCAGCGA
GTGTTCTTGGATGGGCTGGTACCAACAGATCCCAGGGACAGCCCCCAAAACCCTCATCTTTGCTAATGAC
GTGCGAGCCTCAGGGGTGTCAGATCGATTCTCTGGCTCCAGGTCTGGCAACACAGCCACCCTGACCATCT
CTGGGCTTCAGGCTGAGGACGAGGCCGATTACTACTGTGGTTCCGGTTGCACTGAAGATAGGCGTGATGG
TGTATTCGGCGGA
>KR190491.1 Equus caballus clone IGLV22 immunoglobulin lambda light chain variable region (IGL) mRNA, partial cds
ATGGCCTGGTCCCCTCTCCTCCTCACCCTCATCGCTCTCTGCACAGGATCCTGGGCCCAGTCTCTGACTC
AGCCCGCCTCAGTGTCTGGGACCCTGGGCCAGACAGTCACCATCTCCTGCACTGGAAGCAGCTCCAAGAT
GGGAGTTTATATGAACTGGTACCAACAGATCCCAGGGACAGCCCCCAAAACTCTCATCTATAGTGCCAAT
AGTAGACCGTCCGGGGTCCCCGATCGATTCTCTGGCTCCAGGTCTGGCAACACAGCCACCCTGACCATCA
CTGGGCTCCAGGCTGAGGACGAGGCCGATTATTACTGTGGTGTCTATGACAGCAGCAGTAGTAGTAGTGT
ATTCGGCGGA
>KR190490.1 Equus caballus clone IGLV21 immunoglobulin lambda light chain variable region (IGL) mRNA, partial cds
ATGGCCTGGTCCCCTCTCCTCCTCACCCTCATCGCTCTCTGCACAGGATCCTGGGCCCAGTCTGTGACTC
AGCCCGCCTCAGTGTCTGGGACCCTGGGCCAGACAGTCACCATCTCCTGCTCTGGAATCAGCTCCAACAT
CGGATATAGTTATAGTTATGTGGGCTGGTTCCAACAGATCCCAGGAACAGCCCCCAAAACCCTCATCTAT
GGTAATGACAAACGAGCCTCAGGGGTCCCAGATCGATTCTCTGGCTCCAAGTCTGGCAACACAGCCACCC
TGACCATCTCTGGGGTCCAGGCTGAGGACGAGGCCGATTATTACTGTGGTTCCTATGACAGCGGCAGTAG
TAGTATTGTGTTCGGCGGA
>KR190489.1 Equus caballus clone IGLV20 immunoglobulin lambda light chain variable region (IGL) mRNA, partial cds
ATGGCCTGGTCCCCGCTCCTTCTCACCTTCATCGCTCTCTGCACAGGATCCTGGGCCCAGTCTGTGACTC
AGCCCGCCTCCGTGTCTGGGACCCTGGGCCAGACAGTCACCATCTCCTGCTCTGGAAGCAGCTCCAACAT
CGGGTATAGTAATAGTTATGTGGGCTGGTTCCAACAGATCCCAGGAACAGCCCCCAAAACCCTCATCTAT
GCATATAACAAACGAGCCGCAGGGATCCCAGGACGATTCTCTGGCTCCAAATCTGGCAACACAGCCACCC
TGACCATCTCTGGGGTCCAGGCTGAGGACGAGGCCGCTTATTACTGTGGTGCCTATGACAGCATTGGTAC
ATATGTATTCGGCGGA
>KR190488.1 Equus caballus clone IGLV19 immunoglobulin lambda light chain variable region (IGL) mRNA, partial cds
ATGGCCTGGTCCCCTCTCCTCCTCACCCTCATCGCTCTCTGCACAGCAGGATCCTGGGCCCAGTCTGTGA
CTCAGCCCGCCTCAGTGTCTGGGACCCTGGGCCAGACAGTCACCATCTCCTGCTCTGGAAGCAGTGATGA
CATCGGGAGAAGTGGTGCGCATGTGGCCTGGTTCCAACAGATCCCAGGAACAGCCCCCAAAACCCTCATC
TATGGTGATAACAAACGACTCTCAGGGGTCCCAGATCGATTCTCTGGCTCCAAGTCTGGCAACACAGCCA
CCCTGACCATCTCTGGGGTCCAGGCTGAGGACGAGGCCGATTATTACTGTGGTGGTCATGACACGGGCAC
TGGTACAATTGCATTCGGCGGA
>KR190487.1 Equus caballus clone IGLV18 immunoglobulin lambda light chain variable region (IGL) mRNA, partial cds
ATGGCCTGGTCCCCTCTCCTCCTCACCCTCATCGCTCTCTGCACAGGATCCTGGGCCCAGTCTGTGACTC
AGCCCGCCTCAGTGTCTGGGACCCTGGGCCAGACAGTCACCATCTCCTGCTCTGGAAGCAGCTCCGACAT
CGGTCTTAGTTATAATTATGTGGGCTGGTTCCAACAGATCCCAGGAACAGCCCCCAAAACCCTCATCTAT
GATAATAACAAACGAGCCTCAGGGGTCCCAGATCGATTCTCTGGCTCCAAGTCTGGCAACACAGGCACCC
TGACCATCTCTGGGGTCCAGGCTGAGGACGAGGCCGATTATTACTGTAGTTACTGGGACAACAGCAGGGG
TAGTAGTGACTTCGGCGGA

>KR190486.1 Equus caballus clone IGLV17 immunoglobulin lambda light chain variable region (IGL) mRNA, partial cds
ATGGCCTGGTCCCCTCTCCTCCTCACCCTCATCGCTCTCTGCACAGGATCCTGGGCCCAGTCTGTGACTC
AGCCCGCCTCAGTGTCTGGGACCCTGGGCCAGACAGTCACCATCTCCTGCTCTGGAAGCAGCTCCAACAT
CGGGTATACGTATAGTGATGTGGGCTGGTTCCAACAGTTCCCAGGAACAGCCCTCAAAACCCTCATCTAT
GCTACTAACACACGAGCCTCAGGGGTCCCAGATCGATTCTCTGGCTCCAAGTCTGGCAACACAGCCACCC
TGACCATCTCTGGGGTCCAGGCTGAGGACGAGGCCGATTATTATTGTGGTTCCTATGACGCCAGCAGTGA
TAGTAGTGTATTCGGCGGA
>KR190485.1 Equus caballus clone IGLV16 immunoglobulin lambda light chain variable region (IGL) mRNA, partial cds
ATGGCCTGGTCCCCTCTCCTCCTCACCCTCATCGCTCTCTGCACAGGATCCTGGGCCCAGTCTCTGACTC
AGCCCGCCTCAGTGTCTGGGACCCTGGGCCAGACAGTCACCATCTCCTGCTCTGGAAGCAGCTCCAACAT
CGGAAATAGTTATACAGCTGTGGGCTGGTACCAACAGATCCCAGGAACAGCCCCCAAAACCCTCATCTAT
GCTAACAAGCGAGCCTCAGGGGTCCCAGATCGATACTCTGGCTCCAAGTCTGGCAACACAGCCACCCTGA
CCATCTCTGGGGTCCAGGCTGAGGACGAGGCCGATTATTACTGTGGTTCCTATGACAGCAGCAGTAGTAG
TGTTGTATTCGGCGGA
>KR190484.1 Equus caballus clone IGLV15 immunoglobulin lambda light chain variable region (IGL) mRNA, partial cds
ATGGCCTGGTCCCCTCTCCTCCTCACCCTCATCGCTCTCTGCACAGGATCCTGGGCCCAGTCTGTGACTC
AGCCCGCCTCAGTGTCTGGGACCCTGGGCCAGACAGTCACCATCTCCTGCTCTGGAAGCAGCTCCAACAT
CGGGTATAGTTATGGTTATGTGGGCTGGTTCCAACAGATCCCAGGAACAGCCCCCAAAACCCTCATCTAT
GGTAATAACCTACGAGCCTCAGGGGTCCCAGATCGATTCTCTGGCTCCAAGTCTGGCAACACAGCCACCC
TGACCATCACTGGGCTCCAGGCTGAGGACGAGGCTGATTATTACTGTGGTATCTATGACAGCAGCCTGAG
TGGTGCATTCGGCGGA
>KR190483.1 Equus caballus clone IGLV14 immunoglobulin lambda light chain variable region (IGL) mRNA, partial cds
ATGGCCTGGTCCCCGCTCCTTCTCACCTTCATCGCTCTCTGCACAGGATCCTGGGCCCAGTCTCTGACTC
AGCCCGCCTCAGTGTCTGGGACCCTGGGCCAGACAGTCACCATCTCCTGCTCTGGAAGCAGATCCAACAT
TGGGTTAGGAGATGGTTATGTGAGCTGGTTCCAACAGATCCCAGGAACAGCCCCCAAAAGTCTCATCTAT
TATGCCAATTGGACCAGTTCCGGGGTCCCCGATCGATTCTCCGGCTCCAGGTCTGGCAACACAGCCACCC
TGACCATCTCTGGGGTCCAGGCTGAGGACGAGGCTGATTATTACTGCTCATCAGCAGACAGCAGCCTGAG
GAGTGCTGTATTCGGCGGA
>KR190482.1 Equus caballus clone IGLV13 immunoglobulin lambda light chain variable region (IGL) mRNA, partial cds
ATGGCCTGGTCCCCTCTCCTCCTCACCCTCATCGCTCTCTGCACAGGATCCTGGGCCCAGTCTGTGACTC
AGCCCGCCTCAGTGTCTGGGACCCTGGGCCAGACAGTCACCATCTCCTGCTCTGGAAGCAGCTCCAACAT
CGGCAATAGGAAAGTGGGCTGGTTCCAGCAGATCCCAGGAACAGCCCCCAAAACCCTCATCTATGGTGAT
AACCAACGAGCCTCAGGGGTCCCAGATCGATTCTCTGGCTCCAAGTCTGGCAACACAGCCACCCTGACCA
TCTCTGGGGTCCAGGCTGAGGACGAGGCCGATTATTACTGTGCAGCAGGAGACAGCAGCCTGAGGAGTGC
TGTATTCGGCGGA
>KR190481.1 Equus caballus clone IGLV12 immunoglobulin lambda light chain variable region (IGL) mRNA, partial cds
ATGGCCTGGTCCCCGCTCCTTCTCACCTTCATCGCTCTCTGCACAGGATCCTGGGCCCAGTCTCTGACTC
AGCCCGCCTCAGTGTCTGGGACCCTGGGCCAGACAGTCACCATCTCCTGCTCTGGAAGCAGCTCCAACAT
TGGGTATAGTAGTAGTTATGTGAGCTGGTTCCAACAGATTCCAGGAACAGCCCCCAAAACTCTCATCTAT
TATGCCACTAATAGAGCGTCCGGGGTCCCCGATCGATTCTCTGGCTCCAGGTCTGGCAACACAGCCACCC
TGACCATCTCTGGGGTCCAGGCTGAGGACGAGGCTGATTATTACTGCTCATCAGCAGACAGCAGCCTGAG
GAGTGTTGTATTCGGCGGA
>KR190480.1 Equus caballus clone IGLV11 immunoglobulin lambda light chain variable region (IGL) mRNA, partial cds
ATGGCCTGGTCCCCTCTCCTCCTCACCCTCATCGCTCTCTGCACAGGATCCTGGGCCCAGTCTGTGACTC
AGCCCGCCTCAGTGTCTGGGACCCTGGGCCAGACAGTCACCATCTCCTGCTCTGGAAGCAGCTCCAACAT
CGGAGGTAGTTATGTGAGCTGGTTCCAACAGATCCCAGGAACAGCCCCCAAAACCCTCATCTATGGTAAT
AACACACGAGCCTCAGGGGTCCCAGATCGATTCTCTGGCTCCAAGTCTGGCAACACAGCCACCCTGACCA
TCTCTGGGGTCCAGGCTGAGGACGAGGCCGATTATTACTGTGCAGCAGGAGACAGCAGCCTGAGCAGTGG

TGTATTCGGCGGA
>KR190479.1 Equus caballus clone IGLV10 immunoglobulin lambda light chain variable region (IGL) mRNA, partial cds
ATGGCCTGGTCCCCTCTCCTCCTCACCCTCATCGCTCTCTGCACAGGATCCTGGGCCCAGTCTCTGACTC
AGCCCGCCTCAGTGTCTGGGACCCTGGGCCAGACAGTCACCATCTCCTGCTCTGGAAGCAGCTCCAACAT
CGGGTATAGTGGTAGTGTTGTGGGCTGGTACCAACAGGTCCCAGGAACAGCCCTCAAAACCCTCATCTAT
AATACTGATACTCGAGCCTCAGGGGTCCCAGATCGATTCTCTGGCTCCAAGTCTGGCAACACAGCCACCC
TGACCATCTCTGGGGTCCAGGCTGAGGACGAGGCCGATTATTACTGTGTAGTAGGAGACAGCAGCCTGAG
GAGTGCTGTATTCGGCGGA
>KR190478.1 Equus caballus clone IGLV9 immunoglobulin lambda light chain variable region (IGL) mRNA, partial cds
ATGGCCTGGTCCCCTCTCCTCCTCACCCTCATCGCTCTCTGCACAGGATCCTGGGCCCAGTCTCTGACTC
AGCCCGCCTCAGTGTCTGGGACCCTGGGCCAGACAGTCACCATCTCCTGCTCTGGAAGCAGCTCCAACAT
CGGGTATACATATAGTGCTGTGGGCTGGTACCAACAGGTCCCAGGAACAGCCCTCAAAACCCTCATCTAT
GCTACTAACACACGAGCCTCAGGGGTCCCAGATCGATTCTCTGGCTCCAAGTCTGGCAACACAGCCACCC
TGACCATCTCTGGGGTCCAGGCTGAGGACGAGGCCGATTATTACTGTGCAGCAGGAGACAGCAGCCTGAG
GAGTGTTGTATTCGGCGGA
>KR190477.1 Equus caballus clone IGLV8 immunoglobulin lambda light chain variable region (IGL) mRNA, partial cds
ATGGCCTGGTCCCCTCTCCTCCTCACCCTCATCGCTCTCTGCACAGGATCCTGGGCCCAGTCTGTGACTC
AGCCCGCCTCAGTGTCTGGGACCCTGGGCCAGACAGTCACCATCTCCTGCTCTGGAAGCAGCTCCAACAT
CGGCAATGATTATAGTAATGTGGCCTGGTTCCAACAGATCCCAGGAACAGCCCCCAAAACCCTCATCTAT
AGTAATAACGCGCGAGCCTCAGGGGTCCCCGATCGATTCTCTGGCTCCAGGTCTGGCAACACAGCCACCC
TGACCATCTCTGGGGTCCAGGCTGAGGACGAGGCTGATTATTACTGCTCATCAGCAGACAGCAGCCTGAG
GAGTGCTGTATTCGGCGGA
>KR190476.1 Equus caballus clone IGLV7 immunoglobulin lambda light chain variable region (IGL) mRNA, partial cds
ATGGCCTGGTCCCCTCTCCTCCTCACCCTCATCGCTCTCTGCACAGGATCCTGGGCCCAGTCTGTGACTC
AGCCCGCCTCAGTGTCTGGGACCCTGGGCCAGACAGTCACCATCTCCTGCTCTGGAAGCAGCGCCAACAT
CGGGGATAGAACCAGTTATGTGGGCTGGTTCCAACAGATCCCAGGAACAGCCCCCAAAACCCTCATCTAT
GATAATAACAAACGAGCCTCAGGGGTCCCAGATCGATTCTCTGGCTCCAAGTCTGGCAACACAGCCACCC
TGACCATCTCTGGGGTCCAGGCTGAGGACGAGGCCGATTATTACTGTGCAGCAGGAGACAGCAGTCTGAG
AAGTGTTGTATTCGGCGGA
>KR190475.1 Equus caballus clone IGLV6 immunoglobulin lambda light chain variable region (IGL) mRNA, partial cds
ATGGCCTGGTCCCCTCTCCTCCTCACCCTCATCGAACTCTGCACAGGATCCTGGGCCCAGTCTCTGACTC
AGCCCGCCTCAGTGTCTGGGACCCTGGGCCAGACAGTCACCATCACCTGCTCTGGAAGCAGCTCCAACGT
CGGGTATAGTTATAGTAGTGTGGGCTGGTACCAACAGGTCCCAGGAACAGCCCTCAAAACCCTCATCTAT
AATACTAACACACGAGCCTCAGGGGTCCCAGATCGATTCTCTGGCTCCAAGTCTGGCAACACAGCCACCC
TGACCATCTCTGGGGTCCAGGCTGAGGACGAGGCCGATTATTACTGTGCAACAGGAGACATCAGCCTGAG
GAGTGGTGCATTCGGCGGA
>KR190474.1 Equus caballus clone IGLV5 immunoglobulin lambda light chain variable region (IGL) mRNA, partial cds
ATGGCCTGGTCCCCTCTCCTCCTCACCCTCATCGCTCTCTGCACAGGATCCTGGGCCCAGTCTCTGACTC
AGCCCGCCTCAGTGTCTGGGACCCTGGGCCAGACAGTCACCATCTCCTGCTCTGGAAGCAGCTCCAACAT
CGGGTATAGTTATAGTTTTGTGGGCTGGTACCAACAGGTCCCAGGAACAGCCCTCAAAACCCTCATCTGG
GATACTAACACACGAGTTTCAGGGGTCCCAGATCGATTCTCTGGCTCCAAGTCTGGCAACACAGCCACCC
TGACCATCTCTGGGGTCCAGGCTGAGGACGAGGCCGATTATTACTGTTCAGCAGGAGACAGCAGCCTGGG
GAATGCTGTATTCGGCGGA
>KR190473.1 Equus caballus clone IGLV4 immunoglobulin lambda light chain variable region (IGL) mRNA, partial cds
ATGGCCTGGTCCCCTCTCCTCCTCACCCTCATCGCTCTCTGCACAGGATCCTGGGCCCAGTCTGTGACTC
AGCCCGCCTCAGTGTCTGGGACCCTGGGCCAGACAGTCACCATCTCCTGCTCTGGAAGCAGTACCAACAT
CGGGTACTATTCTGTGGGCTGGTTCCAACAGATCCCAGGAACAGCCCCCAAAACCCTCGTCTATCGTGTG
AACAAACGAGCCGCAGGGGTCCCAGATCGATTCTCTGGCTCCAAGTCTGGCAGCACAGCCACCCTGACCA
TCTCTGGGGTCCAGGCTGAGGACGAGGCCGATTATTACTGTTCAGCAGGAGACAACAGCCTGAGGACTAC

TGTATTCGGCGGA
>KR190472.1 Equus caballus clone IGLV3 immunoglobulin lambda light chain variable region (IGL) mRNA, partial cds
ATGGCCTGGTCCCCTCTCCTCCTCACCCTCATCGCTCTCTGCACAGGATCCTGGGCCCAGTCTGTGACTC
AGCCCGCCTCAGTGTCTGGGACCCTGGGCCAGACAGTCACCATCTCCTGCTCTGGAAGCAGCTCCAACAT
CGGACATAAAGAGAGTTGGGTGGCCTGGTTCCAACAGATCCCCGGAACAGCCCCCAAAACCCTCATCTAT
GACGGTGACAAACGAGCCTCAGGGGTCCCAGATCGATTCTCTGGCTCCAAGTCTGGCAACACAGCCACCC
TGACCATTTCTGGGGTCCAGGCTGAGGACGAGGCCGATTATTACTGCTCAGCAGGAGACAGCAGTGGTAC
TTGTGTATTCGGCGGA
>KR190471.1 Equus caballus clone IGLV2 immunoglobulin lambda light chain variable region (IGL) mRNA, partial cds
ATGGCCTGGTCCCCGCTCCTTCTCACCTTCATCGCTCTCTGCACAGGATCCTGGGCCCAGTCTCTGACTC
AGCCCGCCTCAGTGTCTGGGACCCTGGGCCAGACAGTCACCATCTCCTGCTCTGGAAGCAGCTCCAACAT
TGGGTGGGCTGGCAATTATGTGAGCTGGTACCAACAGATCCCAGGAACAGCCCCCAAAACGATCATCTAC
TATGTCTCGAATAGACCGTCCGGGGTCCCCGATCGATTCTCTGGCTCCAAGTCTGGCAACACAGCCACCC
TGACCATCTCTGGGGTCCAGGCTGAGGACGAGGCTGATTATTACTGCTCATCAGCAGACAGCAGTCTGAG
GACTACTGTGTTCGGCGGA
>KR190470.1 Equus caballus clone IGLV1 immunoglobulin lambda light chain variable region (IGL) mRNA, partial cds
ATGGCCTGGTCCCCTCTCCTCCTCACCCTCATCGCTCTCTGCACAGGATCCTGGGCCCAGTCTCTGACTC
AGCCCGCCTCAGTGTCTGGGACCCTGGGCCAGACAGTCACCATCTCCTGCTCTGGAAGCAGCTCCAACAT
CGGTTATAGTGGTAGTCGTGTGGGCTGGTACCAACAGGTCCCAGGAACAGCCCTCAAAACCCTCATCTAT
GGTACTAGCGTACGACCCTCAGGGGTCCCAGATCGATTCTCTGGCTCCAAGTCTGGCAACACAGCCACCC
TGACCATCTCTGGGGTCCAGGCTGAGGACGAGGCCGATTATTACTGTGCAGCAGGAGACATGAGCCTGAG
GACAATTGTATTCGGCGGA
>KF748692.1 Equus caballus clone IGLVJ81 immunoglobulin lambda light chain variable region (IGL) mRNA, partial cds
ATGGCCTGGTCCCCTCTCCTCCTCACCCTCATCGCTTTCTGCGCAGGATCCTGGGCCCAGTCTGTCACTC
AGCCCGCTTCAGTGACTGGGACCCTGGGCGACACGGTCACCATCACCTGCATTGGCACCAGAAGGAACAT
TGGGGGAAGGCTGAATTATGTAGGATGGTACCAACAGATCCCAGGACACGCCCCCAAATGTCTCATCTAT
GGGACGGACAAGAGAGTCTCAGGGGTCCCGGATCGATTTTCTGCCGCCAAGTCGGACACAAGGGCGACCC
TGACCATCTCTGGGGTCCAGACTGAGGACGAGGCCGATTACTATTGTAGTACATATGACGAGGAGAACGA
CACGAACGTCTTCGGCGGAGGCACCCGACTGACCGTCACAG
>KF748691.1 Equus caballus clone IGLVJ80 immunoglobulin lambda light chain variable region (IGL) mRNA, partial cds
ATGGCCTGGTCCCCTCTCCTCCTCACCCTCATCGCTCTCTGCGCAGTTCCAGGATCCTGGACCCAGTCTT
TGATTCAGCCCGCCTCAAAGTCTGGGACCCTGGGGCAGACAGTCGAAATCTCCTGTTCTGGGCAAGCCTC
TAATATCGGACATGATGTGGCGGTGGGGGGTTGGTTTCAGAAGAGACCAGGGTCGGCCCCCAGACTCCTC
ACATATACGGGGAATCGGCGACCGTCAACGGTCCCGGACAGGTTCTCTGAGCGGAAGATTGGCAACGACT
ACATCCTAACCATTACTGGACTCCAGTCTGAGGACGAGGCCGAATACATCTGTGTCTCCGAGGACAAGGG
CCTGGAAAGTTATATTTTTGGCGATGGCACCTACCTGTCCATTTCAG
>KF748690.1 Equus caballus clone IGLVJ79 immunoglobulin lambda light chain variable region (IGL) mRNA, partial cds
ATGGCCTGGTCCCCTCTCCTCCTCACCCTCATCGCTTTCTGCACAGGATCCTGGGCCCAGTCTCTGACTC
AGCCCGCCTCAGTGTCTGGGACCCTGGGCCAGACAGTCACCATCTCCTGCTCTGGAAGCAACTCCAACAT
CGGGCATAGTGGAAATTATGTGGGCTGGTTCCAACAGAACCCAGGGACAGCCCCCAAAACCCTCATCTAT
GGTAATAACAAACGAGCCTCAGGGGTCCGAGATCGATTCTCTGCCGCCAGGTCTGGCAACACAGCCACCC
TGACTATCTCTGGGATCCAGCCTGAGGACGAGGCCGTTTATTATTGTGGTTCCTATGACACAGATAGTAG
TAGTGATGTATTCGGCGGAGGCACCCACCTGTCCATCGCAA
>KF748689.1 Equus caballus clone IGLVJ78 immunoglobulin lambda light chain variable region (IGL) mRNA, partial cds
ATGGCCTGGTCCCCTCTCCTCCTCACCCTCATCGCTCTCTGCACAGGATCCTGGGCCCAGTCTCTGACTC
AGCCCGCCTCAGTGTCGGCGACCCTGGGCCAGACAGTCACCATCTCCTGTTCTGGAAGCAGTAGTAACAT
CGGAGCTGAAGCGACTGAAATTGGCTGGTACCAGGAGGTCCCAGGAAAAGGCCTCAAAACGATATACTAT
CTTACAAACACACGAGCCTCAGGGGTCCCAACTCGCTTCTCTGCAAGGAAGTCTGGCAACACAGCCACCC

TGACCATCTCAGGGGTCCAGGCTGAGGACGAGGCCATTTATTATTGTACGGCACCAGACATCAATGAGGT
GACTGGTGTATTTGGCGGAGGCACCCACCTGACCGTCGTAG
>KF748688.1 Equus caballus clone IGLVJ77 immunoglobulin lambda light chain variable region (IGL) mRNA, partial cds
ATGGCCTGGTCCCCGCTCCTTCTCACCTTCATCGCTCTCTGCACAGGATCCTGGGCCCAGTCTCTGACTC
AGCCCGCCTCAGTGTCTGGGACCCTGGGCCAGACAGTCACCATCTCCTGCTCTGGAAGCAGGTCCAACAT
TGGCTGGGACGGTGATTATGTGACCTGGTTCCAACACGTCCCAGGAACAGCCCCCAAAACTCTCATCTAC
AGTGCCACGAGTAGAGCGTCCGGGGTCCCCGATCGATTCTCTGGGTCCAGGTCTGGCAACACAGCCACCC
TGACCATCTCTGGGGTCCAGGCTGAGGACGAGGCTGATTATTACTGCTCATCAGCAGACAGAAGCCTGAA
GACTGGGATTTTCGGCGGAGGCACCCACCTGACTATCGCGG
>KF748687.1 Equus caballus clone IGLVJ76 immunoglobulin lambda light chain variable region (IGL) mRNA, partial cds
ATGGCCTGGTCCCCTCTCCTCCTCACCCTCATCGCTCTCTGCACAGGATCCAGGGCCCAGTCTCTGACTC
AGCCCGCCTCATTGTCTGGGACCCTGGGCCAGACAGTCACCATTTCCTGCTCGGGAAGCAGCTCCAATAT
CGGGAGTGATGAGACTACAGTGTCCTGGTACCAACAGATCCCAGGAACAGCCCCCAAACTCCTCATTTAC
GAAGATTCCGTGCGAGGCTCAGGGGTCCCAGATCGCTTCTCTGGCTGGAAGTCTGGCAACACTGCTACCC
TGACCATTTCTGGGGCCCAGTCTGTGGACGAGGCCGATTATTATTGCTCAGTGGGGAACGAAAATGGGAA
TACAGAATTCGGTGGGCCCACCCACTTGACCATCGCTG
>KF748686.1 Equus caballus clone IGLVJ75 immunoglobulin lambda light chain variable region (IGL) mRNA, partial cds
ATGGCCTGGACTCTGCTCCTTCTCACCCTCCTCACTCAGGGTACAGGGTCCTGGGCCCAGTCTGCCCTGA
CTCAGCCTGCGTCAGTGTCCGGGGCTCTAGGCCAGTCGGTCACCATCACCTGTGGTGGCAGCAGCAGCGA
CATTGGGGCTTTCGATGCTGTCAGTTGGTTACAGCAGTACCCGGGCACAGCCCCCAAAGTTCTCATTTAT
AGTAAAGAGACTCGGGCCTCAGGGATCCCTGATCGCTTCTCTGGCTCCAAGTCTGGCAACACGGCCTACC
TGACCATCTCTGGGCTCCAGGTCGAAGACGAGGCTATTTATTACTGTTACTCCCTTGTTGAAAAGTACCA
TTACAAATTCGGCGGAGGGACCCACGTGATCGTCCTGG
>KF748685.1 Equus caballus clone IGLVJ74 immunoglobulin lambda light chain variable region (IGL) mRNA, partial cds
ATGGCCTGGTCCCCTCTCCTCCTCACCCTCATCGCTCTCTGCACAGGATCCTGGGCCGCGTCTGTGACTC
AGCCCGCCTCAGTGTCCGGGACCCTGGGCCAGACAGTCACCATCACCTGCACTGGAAGAAATCCCAATGT
TATTGAATATGTTGGCTGGTACCAACAGATCCCAGGAACACGCCCCAAAACTCTCATGTTTGGAAATAGG
CGAGCCGAAGGGGTCCCAGATCGCTTCTCTGCCTCCAAGTCTGGCAGCACAGCCACCCTGACCATCACTG
GGCTCCAGGCTGACGACGAGGCCGATTATTATTGTGGTGTGAGTGGCGAAGGCGGTGCATTTGGTGGAGG
CACTCAACTGACAGTCGCAG
>KF748684.1 Equus caballus clone IGLVJ73 immunoglobulin lambda light chain variable region (IGL) mRNA, partial cds
ATGGCCTGGTCCCCTCTCCTCCTCACCCTCATCGCTCTCTGCACAGCGCTGACAGGATCCTGGGCCCAGT
CTGTGACTCAGCCCGCCTCAGTGTCTGGGACCCTGGGCCAGACAGTCGAAATCGCCTGCACTGGAAGCGC
CTCCAGGGATGATTATTTGCTGAACGATGAGTATATGGTGAACTGGTACCAACAGGTTCCAGGAACCGCC
CCCAGAATCCTCATCTATAGCGTTGCGATTCGACCTGAAGGGGTCCCGAAACGATTCTCTGGCAGCAGGT
CTGGCAAGGTTTTCACCCTGACCATCAGTGGACTCCAGGCTGAGGACGAGGCCGTTTACTACTGTGCCAC
AACTAGCAGTGTCACGAAAACTGGAGTGTTCGGCGGGGGCACCCACCTAACTATCACAG
>KF748683.1 Equus caballus clone IGLVJ72 immunoglobulin lambda light chain variable region (IGL) mRNA, partial cds
ATGGCCTGGTCCCCTCTCCTCCTCACCCTCATCGCTCTCTGCACAGGATCCTGGGCCCAGTCTGTGACCC
AGCCCGCCTCAGTGTCTGGGACCCTGGGCCAGACAGTCACCATCTCCTGCTCCAACATCGGGGATAGTGA
TAGTGATGTGGCCTGGGTCCAACAGATCCCAGGAACAGCCCCCAAACTCCTCATTCGCGGTGTCACTGCA
CGGGCTTCTGGGATCCCTGATCGCTTCTCTGGCTCCAAGTCTGGGAACACGGCCTCTCTGACCATCTCGG
GACTCCAGGCTGAGGACGAGGCCGATTATTACTGCAGTTCCTATGACAGTGAAACGCGTAGTGCAGTGTT
CGGCGGGGGCACCCACCTGTCCATCGCAG
>KF748682.1 Equus caballus clone IGLVJ71 immunoglobulin lambda light chain variable region (IGL) mRNA, partial cds
ATGGCCTGGTCCCCTCTCCTCCTCACCCTCATCGCTCTCTGCACAGGATCTTGGGCCCAGTCTTTGACTC
AGCCCGCCTCAGTGTCTGGGACCCTGGGCCAGACAGTCACCATCTCCTGCTCTGGAAGTAGTTCCAACAT
CGGAAGTAATTATGTAGGCTGGTTCCAACAGATCCCAGGAACAGCCCCCAAGTTCCTCATTTATGAAACC
AACAAACGGGCCTCAGGGGTCCCAGATCGATTCTCTGCCTCCAAGTCTGGCAACACAGCCACCCTGACCA

TCTCTGGGATCCAGGCTGAGGACGAGGCCGATTATTACTGTGGTGCCGCTGACGACAGCAGTGGTTCCAC
TCACATCGGCGGAGGCACCCAGTTGACCATCGCAG
>KF748681.1 Equus caballus clone IGLVJ70 immunoglobulin lambda light chain variable region (IGL) mRNA, partial cds
ATGGCCTGGTCCCCTCTCCTCCTCACCCTCATCGCTCTCTGCACAGGATCCTGGGCCCAGTCTCTGACCC
AGCCCGCCTCAGTGTCTGGGACCCTGGGCCAGACAGTCACCATCTCCTGCTCTGGAAGCCGCTCCAACAT
CGGGTCGAGAGATGAAACTTATGTGAACTGGTTGGTCCAGGTCCCAGGAACAGCCCCCAAAGTCCTCATC
TACCACACGAACAAGCGAGCCCCCGGGGTCGCGGATCGATTCTCTGCCTCCACGTCTGACAACACAGCCA
CCTTGACCGTGTCTGGGGTCCAGGCTGAGGACGAGGCCGTTTATTATTGTGGTTCCTATGACAACAGTCT
CAGGATTTACGTGATCGGCGGAGGCACCCATCTGACTTTCGCAA
>KF748680.1 Equus caballus clone IGLVJ69 immunoglobulin lambda light chain variable region (IGL) mRNA, partial cds
ATGGCCTGGTCCCCTCTCCTCCTCACCCTCATCGCTCTCTGCACGGGCTCCTGGGCCCAGTCTGTGACTC
AGCCCGCCTCAGTGTCTGGGACCCTGGGCCAGTCAGTCACCATCTCCTGCTCGACAAGCAACTCCAACCT
CGGAAAAGATGATAGTGATGTGGCATGGTACCAACAGATCCCAGGAACGGCCCCCAAACTCCTCATCTAT
GGTGGGACGGGGCTAGGAGCAGGGGTCCCAGGTCGATTCTCTGGCTCCGTGTCTGGCAACACAGCCACCC
TCACCATCACTGGGGTCCAGGCTGAGGACGAGGCTGACTATTACTGTTCAACTTATGACAGCAGGTTGAA
TATTGAGTTCGGCGGAGGCACCCACCTGACCATCGTAG
>KF748679.1 Equus caballus clone IGLVJ68 immunoglobulin lambda light chain variable region (IGL) mRNA, partial cds
ATGGCCTGGTCCCCTCTCCTCCTCACCCTCATCGCTCTCTGCACAGGGTCCTGGGCCCAGCCTGTGACCC
AGTCCGCCTCAGTGTCTGGGACCCTCGGCCAGACAGTCACCATTTCCTGTTCTGGAAGTACTTCAAACAT
CGGGAATAGTTACGGATATGTCGCCTGGTTCCAGCAGATCACAGGAACAGTCCCCAAAACCCTCATCAGC
GCGAATAAGAAACGCGCCTCAGGTGTGCCGAGTCGATTCTCTGCCTCAAAGTCTGGCACAACAGCCACCC
TGACCATCTCTGGGGTCCAGGCTGAGGACGAGGCCGATTATTACTGTGCGTCCTATGACAACGTGGTCGA
GTCTGTGGTATTCGGCGGAGGCACCCACCTGACCATCGCAG
>KF748678.1 Equus caballus clone IGLVJ67 immunoglobulin lambda light chain variable region (IGL) mRNA, partial cds
ATGGCCTGGTCCCCTCTCCTCCTCACCCTCATCGCTCTCTGCACAGGGTCCTGGGCCCAGTCTGTGACTC
AGCCCGCCTCAGTGTCTGGGACCCTGGACCAGACAGTCACCATCTCCTGTTCTGGAAGCGGCCTCAATAC
AAGGGATTGGATTACGTATGTTGCCTGGTATCAAGTCGTCCCAGGAAGAGCCCCCAAAACCCTCATCTGG
GGTAACGACATTCGGGCCCAAGGGGTCCCAGATCGATTCTCTGGCAGGAAGTCTGGCAACACAGCCACTC
TGACCATAACGGAGCTCCAGGCTGAGGACGAGGCTGACTATTATTGTGGTACGGTTGACAACAATGTGCG
GAATGCTGATCTTTTCGGCGGAGGCACCCACCTGACCATCGAGG
>KF748677.1 Equus caballus clone IGLVJ66 immunoglobulin lambda light chain variable region (IGL) mRNA, partial cds
ATGGCCTGGTCCCCTCTCCTCCTCATCCTCATCGCTTCCTGCACAGGATCCTGGGCCCAGTCTCTGACTC
AGCCCGCCGATGTGTCTGGGACCCTGGGCCAGACAGTCACCATCTCCTGCTCTGGAACGAGCTCCAACAT
TGGATGGAGTAAAGATTATGTGAGCTGGTTCCAACAGAGACCAGGAACTGCCCCCAAAACACTCATCTAT
AGCGCCACTATCAGAGCGTCCGGGGTCCCCGATCGATTCTCTGGCTCCAGGTCTGGCAACACAATGACCC
TGACCATCTCTGGGGTCCAGGCTGAGGACGAGGCTGTTTATTACTGCTCATCACCAGACATTCTCCTAAG
GAGTGTTGTATTCGGCGGAGGCACCCACCTGACCGTCGCAG
>KF748676.1 Equus caballus clone IGLVJ65 immunoglobulin lambda light chain variable region (IGL) mRNA, partial cds
ATGACCTGGGCTCTGCTCCTCATCACCCTCTTCACTCAGGGCACAGGGTCCTGGGCGCAGTCTGCCCTGA
CTCAGCCTGCGTCAGTGTCCGGGACTCTGGGACAGTCGGTCACCATCTCCTGTGCTGGGAGCGGCGATGA
CATTGGAAAATGGGACCTTGTTTCCTGGTACCGACAGTATCCGGGCGCAGCCCCCAAACTCCTCATACAT
CAAGTCAGTCTAAGGGCTGGTGGGATCCCTGCCCGCTTCTCTGGCTCCAAGTCTGTGAATACGGCCTACC
TGGTCATCTCGGAACTCCAGGCTGAGGACGAGGCCGAATATTTCTGTGCGTCACGTGGAAGTGAAGGTTC
TGCCGTGTTCGGTGGAGGCACCCACTTGACCATTGCAG
>KF748675.1 Equus caballus clone IGLVJ64 immunoglobulin lambda light chain variable region (IGL) mRNA, partial cds
ATGGCCTGGTCCCCTCTCCTCCTCACCCTCATCGCTCTCTGCACAGGATCCTGGGCCCAGTCTCTGACTC
AGCCCGCCTCAGTGTCTGGGACCCTGGGCCAGACAGTCACCATCTCCTGCTCTGGAAGCAGCTCCAACAT
CGGCAGTGCTTTTACGACTGTGGGCTGGTACCAACAGGTCCCAGGAACAGCCCCCAAACTCCTCATTTAT

GGTGATGAGAAACGAGCCTCAGGGGTCCCAGATCGATTCTCTGGCTCCAAGTCAGGCAACACAGCCACCC
TGACCATCTCTGGGGTCCAGGCTGAGGACGAGGCTGACTATTATTGTGCTACTTTCGACGTAGATCAAAG
TATGCCTGTGTTCGGCGGAGGCACCCAACTGACCATCGCGA
>KF748674.1 Equus caballus clone IGLVJ63 immunoglobulin lambda light chain variable region (IGL) mRNA, partial cds
ATGGCCTGGTGCCCTCTCCTCCTCACCCTCATCGCTCTCTGCACAGAGGACTCCTGGGCCCAGTCTGTGA
CTCAGCCCGCCTCAGTGTCTGGGGCCCTGGGCCAGACAGTCACCATCACCTGCACTGGGATCGAGTCCGC
CGACATAGAAGATGTGGGTTGGTATCAACATGTCCCAGGAACAGTCCCCAAAACCCTCATCTGGGCTACG
AAAAGACGAGCCTCAGGGGTCCCAGATCGATTCTCTGGCTCCAAGACTGGCAACACAGCCACCCTAACCA
TCACTAGACTTAAGATTGAGGACGAGGCCGTTTATTATTGTGGGGCCTCGTCTGCCGACGGTGCGATCGC
ATTCGGCGGGGGCACAGACCTGACCCTCACAG
>KF748673.1 Equus caballus clone IGLVJ62 immunoglobulin lambda light chain variable region (IGL) mRNA, partial cds
ATGGCCTGGTCCCCTCTCCTCCTCACCCTCATCGCTCTCTGCACAGGATCCTGGGCCCAGTCTGTGACTC
AGCCCGCCTCAGTGTCTGGGACCCTGGGCCAGACAGTCACCATCACCTGCACTGGAAACGATGGGGGCAG
TGTTGATTATGTGGCCTGGTACCAGCAGATCCCAGGAACAGCGCCCAAAACCCTCTTCTGGGGTGGTGAT
AAACGGGCCTCAGGGGTCCCAGTAAGATTCTCTGCATCCAGATCTGGCACCACAGCCACCCTGACCATAA
CTGGGCTTCAGGCTGAGGACGAGGCCGATTATTTCTGTGGTGCCCGTGGCGTCGGTCAGACCTTCGGCGG
CGGGACCCACCTCACCGTCCTGG
>KF748672.1 Equus caballus clone IGLVJ61 immunoglobulin lambda light chain variable region (IGL) mRNA, partial cds
ATGGCCTGGTCCCCTCTCCTCCTCACCCTCATCGCTCTCTGCACAGGATCCCGGGCACAGTCTGTGACTC
AACCTGCCTCGGTGTCTGGGACCCTGGGCCAGACAGTCAATATCTCCTGTTCTGGAAGCAGCTCCAACAT
TGGGACGGATCTTGTGTCCTGGTACCAACAGATCCCAGGAACAGCCCCCAAAGTCCTCCTGTATTATGGC
GTTAGTAGGGCCTCCGGGGTCTCCGACCGATTCTCTGGCGTCAAGTCTGGCAACACAGCCACCCTGACCA
TCTCTGGGCTCCAGGCTGAGGATGAGGCCGTTTATTACTGTGGTGCCTCTGGAGACATGTGGAGTAGTGA
TTATGATGTATTCGGCGGAGGCACCTACTTGACCATCGCAG
>KF748670.1 Equus caballus clone IGLVJ59 immunoglobulin lambda light chain variable region (IGL) mRNA, partial cds
ATGGCCTGGTCCCCTCTCCTCCTCACCCTCATCGCTCTCTGCACAGGATCCTGGGCCCAGTCTTTGATTC
AGCCCGCCTCAGTGTCTGGGACCCTGGGCCAGACAGTCACCATCTCCTGCTCTGGAAGCAGCGCCAACAT
CGGAGATGGACCCTATAATGTGGGCTGGTTCCAGCAGAAGCCAGGAACAGCCCCCAAAACCCTCGTGCAT
TCGACAGACACGCGAGCCTCAGGGGTCCCCGATCGATTCTCTGGCTCCAGAACTGGGAACACTGCCACCC
TGACCATCTCTGGGGTCCAGGCTGAGGACGAGGCGGTTTATTACTGTGGTGCCTTTGACAGACGAGAAAG
TTATGTATTCGGCGGAGGCACCCACCTGGACATCGCAG
>KF748669.1 Equus caballus clone IGLVJ58 immunoglobulin lambda light chain variable region (IGL) mRNA, partial cds
ATGGCCTGGTCCCCTCTCCTCCTCACCCTCATCGCTCTCTGCACAGGATCCTGGGCCCAGTCTGTGACTC
AGCCCGCCTCAGTGTCGGGGACCCTGGGCCAGACAGTCACCATCTCCTGCTCTGGAAGCAGCTCCAACAT
CGGGAAGGGTCATAGTTATGTGGGCTGGTACCAACAGATCCCAGGAACAAGCCCCAAAACTCTCATCTAT
GGTAATAACAAACGAGCCTCAGGGGTGTCAAGTCGATTCTCTGGCTCCAAGTCTGGCAACACAGCCACCC
TGACCATCACTGGGGTCCAGGCTGACGACGAGGCTGACTATTACTGTAAGTCTTATGACAGCAGCCTGGA
TAGTGTGACTTTCGGCGGAGGCACCCACCTGACCATCGCAG
>KF748668.1 Equus caballus clone IGLVJ57 immunoglobulin lambda light chain variable region (IGL) mRNA, partial cds
ATGGCCTGGTCCCCTCTCCTCCTCACCACCATCGCTCTCTGCACAGGATCCTGGGCCCAGTCTCTGACTC
AGCCCGCCTCAGTGTCTGGGGCCCTGGGCCAGACAGTCACCATCTCCTGCTCTGGAAGCAGCTCCAACAT
CGGCGTAAGAGCTGTGGGCTGGTTCCAACAGATCCCAGGAACAGCCCCCAAAACCCTCATCTCTGGAAAT
AACAAACGAGCCTCAGGGGTCCCAGATCGATTCTCTGATTCCAAGTCTGGCAACACAGCCACCCTGACCA
TCATTGGGGTCCAGGCTGAGGACGAGGCTGACTATTACTGTGCTGTTTATGACGGCAGCCTGAGATACGT
TGTATTCGGCGGAGGCACCCACCTGACCATCGCAG
>KF748667.1 Equus caballus clone IGLVJ56 immunoglobulin lambda light chain variable region (IGL) mRNA, partial cds
ATGGCCTGGTCCCCTCTCCTCCTCACCCTCATCGCTCTCTGCACAGGATCCTGGGCCCAGTCTGTGACTC
AGCCCGCCTCAGTGTCTGGGACCCAGGGCCAGACAGTCACCATCTCGTGCTCTGGAAGCAGGTCCAGTAT
CGGGGCGTCTCGTAGTGGGCATGTGGGCTGGTATCAACAAATCCCAGGAACAGCCCCCAAAAATCTGATC

TATGGTAATGACAAACGAGCCTCAGGGGTCCCAGATCGATTCTCTGGCTCCAAGTCTGGCAACACAGCCA
CCCTGACCATCACTGGGGTCCAGGCTGAGGACGAGGCTGTCTATTACTGTTTGACTTATGACGTCGATCT
GAAAACTAATATATTCGGCGGAGGAACCCACCTGACCATCGCAG
>KF748666.1 Equus caballus clone IGLVJ55 immunoglobulin lambda light chain variable region (IGL) mRNA, partial cds
ATGGCCTGGTCCCCTCTCCTCCTCATCCTCATCGCTCTCTGCACAGGATCCTGGGCCCAGTCTGTGACGC
AGCCCGCCTCAGTGTCTGGGACCCTGGGCCAGACTGTCACCATCTCCTGCTCCGGTAGCGCTTCCAACAT
TGGTGGGAGTGGTAATTATGTGGCCTGGTACCAGCACAGACCAGGAGAAGCCCCCAAAACCCTCATCTAT
GGCAATGACCAGCGAGAGACAGGGGTCCCGTACCGATTCTCTGGCTCCAAGTCTGGCAGCACAGCCACCC
TGACCGTCACTGGGGTCCAGGATGAAGACGAGGCTGACTATTTCTGTGGTACTTGGGACAGCGACCAGAA
AACAGGTGTATTCGGCGGGGGCACCCACCTGACCGTCGCAG
>KF748665.1 Equus caballus clone IGLVJ54 immunoglobulin lambda light chain variable region (IGL) mRNA, partial cds
ATGGCCTGGTCCCCTCTCCTCCTCACCCTCATCGCTCTCTGCACAGGATCCTGGGCCCAGTCTCTGACTC
AGCCCGCCTCAGTGTCTGGGACCCTGGGCCAGACAGTCACCATCTCCTGCTCTGGAAGCAGCTCCAACAT
CGGGAATCATGACAGTATGGTGGGATGGTACCAGCAGGTCCCAGGAACAGCCCCCAAAACCCTCATCTAC
GGTGACAACAGACGAGCCTCAGGGACCCCAGATCGATTCTCTGGCTCCAAGTCTGGCAACGTTGCCACCC
TGACCATCTCTGGGGTCCAGGCTGAGGACGAGGCCGATTATTACTGTTCAGCAGGGGACAGCAGTCGGAG
TGCTGCATTCGGCGGAGGCACCCACCTGACCATCGCAG
>KF748664.1 Equus caballus clone IGLVJ53 immunoglobulin lambda light chain variable region (IGL) mRNA, partial cds
ATGGCCTGGTCCCCTCTCCTCCTCACCCTCATCGCTCTCTGCACAGGATCCTGGGCCCAGTCTCTGACCC
AGCCCGCCTCAGTGTCTGGGACCCTGGGCCAGACAGTCACCATCTCCTGCTCTGGAAGCAGCTCCAACAT
CGGGTATAGTTATAGTGCTGTGGGCTGGTACCAACAGATCCCAGGAACAGCCCCCAAAACCCTCATCTAT
GGTAATAACAAACGAGCCTCAGGGGTCCCAGATCGATTCTCTGGCTCCAAGTCTGGCAACACAGCCACCC
TGACCATCTCTGGGCTTCAGGCTGAGGACGAGGCCGATTATTACTGTGGTTCCTATTACAGCAGTGATGC
ATTCGGCGGAGGCACCCACCTGACCATCGCAG
>KF748663.1 Equus caballus clone IGLVJ52 immunoglobulin lambda light chain variable region (IGL) mRNA, partial cds
ATGGCCTGGTCCCCTCTCCTCCTCACCCTCATCGCTCTCTGCACAGGATCCTGGGCCCAGTCTCTGACTC
AGCCCGCCTCAGTGTCTGGGACCCTGGGCCAGACAGTCACCATCTCCTGCTCTGGAAGCAGCTCCAACAT
CGGGTATAGTTATAGTGCTGTGGGCTGGTACCAACAGATCCCAGGGACAGCCCCCAAAACCCTCATCTAT
GCTACTAACAAACGAGCCTCAGGGGTCCCAGATCGATTCTCTGGCTCCAAGTCTGGCAACACAGCCACCC
TGACCATCTCTGGGCTTCAGGCTGAGGACGAGGCCGATTATTACTGTGGTTCCTCATACAGCAGTGATAG
TAGTGATGGTGCATTCGGCGGAGGCACCCACCTGACCATCGCAG
>KF748662.1 Equus caballus clone IGLVJ51 immunoglobulin lambda light chain variable region (IGL) mRNA, partial cds
ATGGCCTGGTCCCCTCTCCTCCTCACCCTCATCGCTCTCTGCACAGGATCCTGGGCCCAGTCTCTGACTC
AGCCCGCCTCAGTGTCTGGGACCCTGGGCCAGACAGTCACCATCTCCTGCTCTGGAAGCAGCTCCAACAT
CGGGTATAGTTATAGTGCTGTGGGCTGGTACCAACAGATCCCAGGGACAGCCCCCAAAACCCTCATCTAT
GCTACTAACAAACGAGCCTCAGGGGTCCCAGATCGATTCTCTGGCTCCAAGTCTGGCAACACAGCCACCC
TGACCATCTCTGGGCTTCAGGCTGAGGACGAGGCCGATTATTACTGTGGTTCCTCATACAGCAGTGATAG
TGGTGCATTCGGCGGAGGCACCCACCTGACCATCGCAG
>KF748661.1 Equus caballus clone IGLVJ50 immunoglobulin lambda light chain variable region (IGL) mRNA, partial cds
ATGGCCTGGTGCCCTCTCCTCCTCACCCTCATCGCTCTCTGCACAGGATCCTGGGCCCAGTCTGTGACTC
AGCCCGCCTCAGTGTCTGGGACCCTGGGCCAGACAGTCACCATCACCTGCACTGGAAGCAGCTCCAACAT
AGTTGCTTATGTGGGCTGGTACCAACAGATCCCAGGAACAGCCCCCAAAACCCTCATCTATGCTAATAAC
AAACGAGCCTCAGGGGTCCCAGATCGATTCTCTGGCTCCAAGTCTGGCAGCACAGCCACCCTGACCATCA
CTGGGCTCCAGGCTGAGGACGAGGCCGATTATTACTGTGGTACCTCTAGCAGCAGTGGTAGTAGTGATCA
TGGTGCATTCGGCGGAGGCACCCACCTGACCATCGCAG
>KF748660.1 Equus caballus clone IGLVJ49 immunoglobulin lambda light chain variable region (IGL) mRNA, partial cds
ATGGCCTGGTCCCCTCTCCTCCTCACCCTCATCGCTCTCTGCACAGGATCCCGGGCCCAGTCTGTGACTC
AGCCCGCCTCAGTGTCTGGGACCCTGGGCCAGACAGTCACCATCTCCTGCTCTGGAAGCAGCTCCAACAT

CGGGAGTGGTCATGTGTCCTGGTACCAACAGATCCCAGGAACAGCCCCCAAACGCCTCATCTATTCTTCC
GCTAGCAGGGCTTCCGGGGTCCCCGACCGATTCTCTGGCTCCAGGTCTGGCAACACAGCCACCCTGACCA
TCTCTGGGCTCCAGGCTGAGGATGAGGCCGATTATTACTGTGGTACATTGTACAGCAGTTGGAGTAGTGC
TGTATTCGGCGGAGGCACCCACCTGACCATCGCAG
>KF748659.1 Equus caballus clone IGLVJ48 immunoglobulin lambda light chain variable region (IGL) mRNA, partial cds
ATGGCCTGGACCCCTCTCTTGTTAGCCTTCCTCTCTCTCTGCACAGGTCCTGTTGTCTCTTCTGCAGTGA
CTCAGCCATCTGAGGTGTCCGTGGCCTTGGGACAGAGAGCCACCCTCACCTGCCAGGGAAGCAACTTTGA
ATTTTTTTCTCCTAGCTGGTACCAGCAGAAGCCAGGCCAGGCCCCTGTACTGCTCATCAATATTAATAAT
GAGCGCCACTCAGGGATCCCTGAACGATTCTCCGGCTCCAGCTCAGGAGACACGTCCACACTGACCATCA
GTGGGGCCCAGGCTGAGGACGAGGCTGACTATTACTGTCTGGCAGTAGATGCTCTTAGTTCTGAAATTTT
TGTATTCGGCGGAGGCACCCACCTGACCATCGCAG
>KF748658.1 Equus caballus clone IGLVJ47 immunoglobulin lambda light chain variable region (IGL) mRNA, partial cds
ATGGCCTGGACCCCTCTCTTGTTAGCCTTCCTCTCTCTCTGCACAGGTCCTGTTGTCTCTTCTGCAGTGA
CTCAGCCATCTGAGGTGTCCGTGGCCTTGGGACAGAGAGCCACCCTCACCTGCCAGGGAAGCAACTTTGA
ATTTTTTTCTCCTAGCTGGTACCAGCAGAAGCCAGGCCAGGCCCCTGTACTGCTCATCAATATTAATAAT
GAGCGCCACTCAGGGATCCCTGAACGATTCTCCGGCTCCAGCTCAGGAGACACGTCCACACTGACCATCA
GTGGGGCCCAGGCTGAGGACGAGGCTGACTATTACTGTCTGGCAGTAGATGCTCTTAGTTCTGAAATTGT
ATTCGGCGGAGGCACCCACCTGACCATCGCAG
>KF748657.1 Equus caballus clone IGLVJ46 immunoglobulin lambda light chain variable region (IGL) mRNA, partial cds
ATGGCCTGGACCCCTCTCTTGTTAGCCTTCCTCACTCTCTGCACAGGTCCCGTGGCCTCTTCTAAGCTGA
CTCAGCCATCTTCAGTGTCTGTGGCCTTGGGACAGACGGCCACCATCACCTGCAAGGGAGGCAACTTTGA
AAGTTTTGTTGGTAGCTGGTACCAGCAGAAGCCAGGCCAGGCCCCTGTGCTGGTCATCGATCCTAGTAAT
GAGCGGCCCTCAGGGATCCCTGAACGATTCTCTGGCTCCAGCTCAGGAGACACAGCCACGCTGACCATCA
GCGGGGCCCAGGCTGAGGACGAGGCTGACTATTACTGTCTGGCAGCAGATGCTTCTGATTATGAAGCTGC
ATTCGGCGGAGGCACCCACCTGACCATCGCAG

>KF748656.1 Equus caballus clone IGLVJ45 immunoglobulin lambda light chain variable region (IGL) mRNA, partial cds
ATGGCCTGGACCCCTCTCTTGTTAGCCTTCCTCACTCTCTGCACAGGTCCCGTGGCCTCTTCTAAGCTGA
CTCAGCCATCTTCAGTGTCTGTGGCCTTGGGACAGACGGCCACCATCACCTGCAAGGGAGGCAACTTTGA
AAGTTTTGTTGGTAGCTGGTACCAGCAGAAGCCAGGCCAGGCCCCTGTGCTGGTCATCGATCCTAGTAAT
GAGCGGCCCTCAGGGATCCCTGAACGATTCTCTGGCTCCAGCTCAGGAGACACAGCCACGCTGACCATCA
GCGGGGCCCAGGCTGAGGACGAGGCTGACTATTACTGTCTGGCAGCAGATGCTTCTGATTATGAAGCTAG
TGCATTCGGCGGAGGCACCCACCTGACCATCGCAG
>KF748655.1 Equus caballus clone IGLVJ44 immunoglobulin lambda light chain variable region (IGL) mRNA, partial cds
ATGGCCTGGACCCCTCTCTTGTTAGCCTTCCTCACTCTCTGCACAGGTCCCGTGGCCTCTTCTAAGCTGA
CTCAGCCATCTTCAGTGTCTGTGGCCTTGGGACAGACGGCCACCATCACCTGCAAGGGAGGCAACTTTGA
AAGTTTTGTTGGTAGCTGGTACCAGCAGAAGCCAGGCCAGGCCCCTGTGCTGGTCATCGATCCTAGTAAT
GAGCGGCCCTCAGGGATCCCTGAACGATTCTCTGGCTCCAGCTCAGGAGACACAGCCACGCTGACCATCA
GCGGGGCCCAGGCTGAGGACGAGGCTGACTATTACTGTCTGGCAGCAGATGCTTCTGATTATGCTGTATT
CGGCGGAGGCACCCACCTGACCATCGCAG
>KF748654.1 Equus caballus clone IGLVJ43 immunoglobulin lambda light chain variable region (IGL) mRNA, partial cds
ATGGCCTGGACCCCTCTCTTGTTAGCCTTCCTCACTCTCTGCACAGGTCCCGTGGCCTCTTCTAAGCTGA
CTCAGCCATCTTCAGTGTCTGTGGCCTTGGGACAGACGGCCACCATCACCTGCAAGGGAGGCAACTTTGA
AAGTTTTGTTGGTAGCTGGTACCAGCAGAAGCCAGGCCAGGCCCCTGTGCTGGTCATCGATCCTAGTAAT
GAGCGGCCCTCAGGGATCCCTGAACGATTCTCTGGCTCCAGCTCAGGAGACACAGCCACGCTGACCATCA
GCGGGGCCCAGGCTGAGGACGAGGCTGACTATTACTGTCTGGCAGCAGATGCTTCTGATTATGAAGCTAG
TATCTTCGGCAGCGGGACCCACCTCAGCGTCCTGG
>KF748653.1 Equus caballus clone IGLVJ42 immunoglobulin lambda light chain variable region (IGL) mRNA, partial cds
ATGGCCTGGTGCCCTCTCCTCCTCACCCTCATCGCTCTCTGCACAGGATCCTGGGCCCAGTCTGTGACTC

AGCCCGCCTCAGTGTCTGGGACCCTGGGCCAGACAGTCACCATCACCTGCACTGGAAGCAGCTCCAACAT
AGTTGCTTATGTGGGCTGGTACCAACAGATCCCAGGAACAGCCCCCAAAACCCTCATCTATGGTAATAAC
AAACGAGCCTCAGGGGTCCCAGATCGATTCTCTGGCTCCAAGTCTGGCAGCACAGCCACCCTGACCATCA
CTGGGCTCCAGGCTGAGGACGAGGCCGATTATTACTGTGGTACCTCTAGCAGCAGTGGTAGTAGTGCTGT
ATTCGGCGGAGGCACCCACCTGACCATCGCAG
>KF748652.1 Equus caballus clone IGLVJ41 immunoglobulin lambda light chain variable region (IGL) mRNA, partial cds
ATGGCCTGGTGCCCTCTCCTCCTCACCCTCATCGCTCTCTGCACAGGATCCTGGGCCCAGTCTGTGACTC
AGCCCGCCTCAGTGTCTGGGACCCTGGGCCAGACAGTCACCATCACCTGCACTGGAAGCAGCTCCAACAT
AGTTGCTTATGTGGGCTGGTACCAACAGATCCCAGGAACAGCCCCCAAAACCCTCATCTATGCTAATAAC
AAACGAGCCTCAGGGGTCCCAGATCGATTCTCTGGCTCCAAGTCTGGCAGCACAGCCACCCTGACCATCA
CTGGGCTCCAGGCTGAGGACGAGGCCGATTATTACTGTGGTACCTCTAGCAGCAGTGGTAGTAGTGCTGT
ATTCGGCGGAGGCACCCACCTGACCATCGCAG
>KF748651.1 Equus caballus clone IGLVJ40 immunoglobulin lambda light chain variable region (IGL) mRNA, partial cds
ATGGCCTGGTCCCCTCTCCTCCTCACCCTCATCGCTCTCTGCACAGGATCCTGGGCCCAGTCTCTGACTC
AGCCCGCCTCAGTGTCTGGGACCCTGGGCCAGACAGTCACCATCTCCTGCACTGGAAGCAGCTCCAGCAT
AGGTTCTTATATGGGCTGGTACCAACAGATCCCAGGGACAGCCCCCAAAACCCTCATCTATGCTACTAAC
AAACGAGCCTCAGGGGTCCCAGATCGATTCTCTGGCTCCAAGTCTGGCAACACAGCCACCCTGACCATCA
CTGGGCTTCAGGCTGAGGACGAGGCCGATTATTACTGTGGTTCCTATTACAGCAGTGATAGTAGTGATGT
ATTCGGCGGAGGCACCCACCTGACCATCGCAG
>KF748650.1 Equus caballus clone IGLVJ39 immunoglobulin lambda light chain variable region (IGL) mRNA, partial cds
ATGGCCTGGTCCCCTCTCCTCCTCACCCTCATCGCTCTCTGCACAGGATCCTGGGCCCAGTCTCTGACTC
AGCCCGCCTCAGTGTCTGGGACCCTGGGCCAGACAGTCACCATCTCCTGCACTGGAAGCAGCTCCAGCAT
AGGTTCTTATATGGGCTGGTACCAACAGATCCCAGGGACAGCCCCCAAAACCCTCATCTATGCTACTAAC
AAACGAGCCTCAGGGGTCCCAGATCGATTCTCTGGCTCCAAGTCTGGCAACACAGCCACCCTGACCATCA
CTGGGCTTCAGGCTGAGGACGAGGCCGATTATTACTGTGGTTCCTATTACAGCAGTGATAGTGGTGCATT
CGGCGGAGGCACCCACCTGACCATCGCAG
>KF748648.1 Equus caballus clone IGLVJ37 immunoglobulin lambda light chain variable region (IGL) mRNA, partial cds
ATGGCCTGGTCCCCTCTCCTCCTCACCCTCATCGCTCTCTGCACAGGATCCTGGGCCCAGTCTGTGACTC
AGCCCGCCTCAGTGTCTGGGACCCTGGGCCAGACAGTCACCATCTCCTGCTCTGGAAGCAGCTCCAACAT
CGGGTATAGTTATAGTTATGTGGGCTGGTTCCAACAGATCCCAGGAACAGCCCCCAAAACCCTCATCTAT
GGTAATAACAAACGAGCCTCAGGGGTCCCAGATCGATTCTCTGGCTCCAAGTCTGGCAACACAGCCACCC
TGACCATCTCTGGGGTCCAGGCTGAGGACGAGGCCGATTATTACTGTGGTTCCTATTACAGCAGTGATAG
TAGTGTTGTATTCGGCGGAGGCACCCACCTGACCATCGCAG
>KF748647.1 Equus caballus clone IGLVJ36 immunoglobulin lambda light chain variable region (IGL) mRNA, partial cds
ATGGCCTGGTGCCCTCTCCTCCTCACCCTCATCGCTCTCTGCACAGGATCCTGGGCCCAGTCTGTGACTC
AGCCCGCCTCAGTGTCTGGGACCCTGGGCCAGACAGTCACCATCTCCTGCTCTGGAAGCAGCTCCAACAT
TGGGTATAGTAGTAGTTATGTGAGCTGGTTCCAACAGATCCCAGGAACAGCCCCCAAAACTCTCATCTAT
TATGCCACTAGTAGAGCGTCCGGGGTCCCCGATCGATTCTCTGGCTCCAGGTCTGGCAACACAGCCACCC
TGACCATCTCTGGGGTCCAGGCTGAGGACGAGGCTGATTATTACTGCTCATCAGCAGACAGCAGCCTGAG
GAGTGATGTATTCGGCGGAGGCACCCACCTGACCATCGCAG
>KF748646.1 Equus caballus clone IGLVJ35 immunoglobulin lambda light chain variable region (IGL) mRNA, partial cds
ATGGCCTGGTCCCCTCTCCTCCTCACCCTCATCGCTCTCTGCACAGGATCCTGGGCCCAGTCTCTGACTC
AGCCCGCCTCAGTGTCTGGGACCCTGGGCCAGACAGTCACCATCTCCTGCTCTGGAAGCAGCTCCAACAT
CGGGAATAGTTATAGTTCTGTGGGCTGGTTCCAACAGATCCCAGGAACAGCCCCCAAAACCCTCATCTAT
GGTAATAACAAACGAGCCTCAGGGGTCCCAGATCGATTCTCTGGCTCCAAGTCTGGCAACACAGCCACCC
TGACCATCTCTGGGGTCCAGGCTGAGGACGAGGCTGACTATTACTGTGCAGCAGGAGACAGCAGCCTTAA
TGGTGCTGTATTCGGCGGAGGCACCCACCTGACCATCGCAG
>KF748645.1 Equus caballus clone IGLVJ34 immunoglobulin lambda light chain variable region (IGL) mRNA, partial cds

ATGGCCTGGACCCCTCTCTTGTTAGCCTTCCTCACTCTCTGCACAGGTCCCGTGGCCTCTTCTAAGCTGA
CTCAGCCATCTTCAGTGTCTGTGGCCTTGGGACAGACGGCCACCATCACCTGCAAGGGAGGCGACTTTGA
AAGTTTTGTTGGTAGCTGGTACCAGCAGAAGCCAGGCCAGGCCCCTGTGCTGGTCATCGATGCTAGTAAT
GAGCGGCCCTCAGGGATCCCTGAACGATTCTCTGGCTCCAGCTCAGGAGACACAGCCACGCTGACCATCA
GCGGGGCCCAGGCTGAGGACGAGGCTGACTACTACTGTCTGGCAGCAGATGCTTCTGATATCTTCGGCGG
CGGGACCCACCTCAGCGTCCTGG
>KF748644.1 Equus caballus clone IGLVJ33 immunoglobulin lambda light chain variable region (IGL) mRNA, partial cds
ATGGCCTGGACCCCTCTCTTGTTAGCCTTCCTCACTCTCTGCACAGGTCCCGTGGCCTCTTCTAAGCTGA
CTCAGCCATCTTCAGTGTCTGTGGCCTTGGGACAGACGGCCACCATCACCTGCAAGGGAGGCGACTTTGA
AAGTTTTGTTGGTAGCTGGTACCAGCAGAAGCCAGGCCAGGCCCCTGTGCTGGTCATCGATGCTAGTAAT
GAGCGGCCCTCAGGGATCCCTGAACGATTCTCTGGCTCCAGCTCAGGAGACACAGCCACGCTGACCATCA
GCGGGGCCCAGGCTGAGGACGAGGCTGACTATTACTGTCTGGCAGCAGATGCTTCTGATATCTTCGGCGG
CGGGACCCACCTCAGCGTCCTGG
>KF748642.1 Equus caballus clone IGLVJ31 immunoglobulin lambda light chain variable region (IGL) mRNA, partial cds
ATGGCCTGGTGCCCTCTCCTCCTCACCCTCATCGCTCTCTGCACAGGATCCTGGGCCCAGTCTGTGACTC
AGCCCGCCTCAGTGTCTGGGACCCTGGGCCAGACAGTCACCATCACCTGCACTGGAAGCAGCTCCAACAT
AGTTGCTTATGTGGGCTGGTACCAACAGATCCCAGGAACAGCCCCCAAAACCCTCATCTACGCTAATAAC
AAACGAGCCTCAGGGGTCCCAGATCGATTCTCTGGCTCCAAGTCTGGCAGCACAGCCACCCTGACCATCA
CTGGGCTCCAGGCTGAGGACGAGGCCGATTATTACTGTGGTACCTCTAGCAGCAGTGGTAGTAGTGCATT
CGGCGGAGGCACCCACCTGACCATCGCAG
>KF748641.1 Equus caballus clone IGLVJ30 immunoglobulin lambda light chain variable region (IGL) mRNA, partial cds
ATGGCCTGGTGCCCTCTCCTCCTCACCCTCATCGCTCTCTGCACAGGATCCTGGGCCCAGTCTGTGACTC
AGCCCGCCTCAGTGTCTGGGACCCTGGGCCAGACAGTCACCATCACCTGCACTGGAAGCAGCTCCAACAT
AGTTGCTTATGTGGGCTGGTACCAACAGATCCCAGGAACAGCCCCCAAAACCCTCATCTACGCTAATAAC
AAACGAGCCTCAGGGGTCCCAGATCGATTCTCTGGCTCCAAGTCTGGCAGCACAGCCACCCTGACCATCA
CTGGGCTCCAGGCTGAGGACGAGGCCGATTATTACTGTGGTACCTCTAGCAGCAGTGGTAGTAGTGCTGT
ATTCGGCGGAGGCACCCACCTGACCATCGCAG
>KF748639.1 Equus caballus clone IGLVJ28 immunoglobulin lambda light chain variable region (IGL) mRNA, partial cds
ATGGCCTGGGCTCTGTTCCTCATCACCCTCCTCACTCAGGGTACAGGGTCCTGGGGCCAGTCTGCCCTGA
TTCAGCCTTCTTCGGTGTCCGTGGCTCTAGGACAGTCGGTCACCATCTCCTGTGCTGGAAGCAGCAGTGA
CATTGGGTATTATAACTCTATTTCCTGGTACCAACAGCACCCAGGCACAACCCCAAAGCTGCTGATTTAC
TATACCAATAAGAAGCACTCAGGGATCCCTGATCGCTTCTCTGGCTCCAAGTCTGGGAACACGGCCTCCC
TGACCATCTCTGGGCTCCAGGCTGAGGATGAGGCTGAGTATTACTGTTGCTCATATGCAGGCAGTGGCAA
TTTTGCATTCGGCGGAGGCACCCACCTGACCATCGCAG
>KF748638.1 Equus caballus clone IGLVJ27 immunoglobulin lambda light chain variable region (IGL) mRNA, partial cds
ATGGCCTGGACCCCTCTCTTGTTAGCCTTCCTCACTCTCTGCACAGGTCCCGTGGCCTCTTCTAAGCTGA
CTCAGCCATCTTCAGTGTCTGTGGCCTTGGGACAGACGGCCACCATCACCTGCAAGGGAGGCAACTTTGA
AAGTTTTGTTGGTAGCTGGTACCAGCAGAAGCCAGGCCAGGCCCCTGTGCTGGTCATCGATCCTAGTAAT
GAGCGGCCCTCAGGGATCCCTGAACGATTCTCTGGCTCCAGCTCAGGAGACACAGCCACGCTGACCATCA
GCGGGGCCCAGGCTGAGGACGAGGCTGACTATTACTGTCTGGCAGCAGATGCTTCTGATTATGCTGTATT
CGGCGGAGGCACCCACCTGACCATCGCAG
>KF748637.1 Equus caballus clone IGLVJ26 immunoglobulin lambda light chain variable region (IGL) mRNA, partial cds
ATGGCCTGGACCCCTCTCTTGTTAGCCTTCCTCACTCTCTGCACAGGTCCCGTGGCCTCTTCTAAGCTGA
CTCAGCCATCTTCAGTGTCTGTGGCCTTGGGACAGACGGCCACCATCACCTGCAAGGGAGGCAACTTTGA
AAGTTTTGTTGGTAGCTGGTACCAGCAGAAGCCAGGCCAGGCCCCTGTGCTGGTCATCGATCCTAGTAAT
GAGCGGCCCTCAGGGATCCCTGAACGATTCTCTGGCTCCAGCTCAGGAGACACAGCCACGCTGACCATCA
GCGGGGCCCAGGCTGAGGACGAGGCTGACTATTACTGTCTGGCAGCAGATGCTTCTGATGGTGCATTCGG
CGGAGGCACCCAACTGACCATCGCAG
>KF748636.1 Equus caballus clone IGLVJ25 immunoglobulin lambda light chain variable region (IGL) mRNA, partial cds

ATGGCCTGGACCCCTCTCTTGTTAGCCTTCCTCACTCTCTGCACAGGTCCCGTGGCCTCTTCTAAGCTGA
CTCAGCCATCTTCAGTGTCTGTGGCCTTGGGACAGACGGCCACCATCACCTGCAAGGGAGGCAACTTTGA
AAGTTTTGTTGGTAGCTGGTACCAGCAGAAGCCAGGCCAGGCCCCTGTGCTGGTCATCGATCCTAGTAAT
GAGCGGCCCTCAGGGATCCCTGAACGATTCTCTGGCTCCAGCTCAGGAGACACAGCCACGCTGACCATCA
GCGGGGCCCAGGCTGAGGACGAGGCTGACTATTACTGTCTGGCAGCAGATGCTTCTGATTATGGTGCATT
CGGCGGAGGCACCCACCTGACCATCGCAG
>KF748635.1 Equus caballus clone IGLVJ24 immunoglobulin lambda light chain variable region (IGL) mRNA, partial cds
ATGGCCTGGACCCCTCTCCTGCTCCTCCTCCTCACTCTCTGCACAGGCTCTGTGGCTTCTTCTATGCTGA
CTCAGCCACTTACCTTGTCCGTGGCCTTTGGAAGCACAGTCACTATCACATGCCAGGGAGAGCTCCTAGA
CAGTTATTATGCTGAGTGGTACCAGCAGAAGCCAGACCAGGCTCCCGTGCTGGTCATATATTATGGAAGC
AAACGTCCTTCGGGGATTTCTACCCGATTCTCTGGCTCCTACTCAAGCAAGATGGCCACCCTGACCCTCA
GTGGGGCCTTGGCCGAGGATGAGGCTGACTATTACTGTCAGGTGTGGGACAGCAGTGGTAACCAGTATAT
CTTCGGCAGCGGGACCCACCTCAGCGTCCTGG
>KF748634.1 Equus caballus clone IGLVJ23 immunoglobulin lambda light chain variable region (IGL) mRNA, partial cds
ATGGCCTGGACCCCTCTCCTGCTCCTCCTCCTCACTCTCTGCACAGGCTCTGTGGCTTCTTCTATGCTGA
CTCAGCCACTTACCTTGTCCGTGGCCTTTGGAAGCACAGTCACTATCACATGCCAGGGAGAGCTCCTAGA
CAGTTATTATGCTGAGTGGTACCAGCAGAAGCCAGACCAGGCTCCCGTGCTTGTCATATATTATGGAAGC
AAACGTCCTTCGGGGATTTCTACCCGATTCTCTGGCTCCTACTCAAGCAAGATGGCCACCCTGACCCTCA
GTGGGGCCTTGGCCGAGGATGAGGCTGACTATTACTGTCAGGTGTGGGACAGCAGTGGTAACCAGTATAT
CTTCGGCAGCGGGACCCACCTCAGCGTCCTGG
>KF748633.1 Equus caballus clone IGLVJ22 immunoglobulin lambda light chain variable region (IGL) mRNA, partial cds
ATGGCCTGGTCCCCTCTCCTCCTCACCCTCATCGCTCTCTGCACAGGATCCTGGGCCCAGTCTCTGACCC
AGCCCGCCTCAGTGTCTGGGACCCTGGGCCAGACAGTCACCATCTCCTGCTCTGGAAGCAGCTCCAACAT
CGGGTATAGTTATAGTGCTGTGGGCTGGTACCAACAGATCCCAGGAACAGCCCCCAAAACCCTCATCTAT
GGTAATAACAAACGAGCCTCAGGGGTCCCAGATCGATTCTCTGGCTCCAAGTCTGGCAACACAGCCACCC
TGACCATCTCTGGGCTTCAGGCTGAGGACGAGGCCGATTATTACTGTGGTTCCTATTACAGCAGTGATAG
TAGTGCATTCGGCGGAGGCACCCACCTGACCATCGCAG
>KF748632.1 Equus caballus clone IGLVJ21 immunoglobulin lambda light chain variable region (IGL) mRNA, partial cds
ATGGCCTGGTCCCCTCTCCTCCTCACCCTCATCGCTCTCTGCACAGGATCCTGGGCCCAGTCTCTGACCC
AGCCCGCCTCAGTGTCTGGGACCCTGGGCCAGACAGTCACCATCTCCTGCTCTGGAAGCAGCTCCAACAT
CGGGTATAGTTATAGTGCTGTGGGCTGGTACCAACAGATCCCAGGAACAGCCCCCAAAACCCTCATCTAT
GGTAATAACAAACGAGCCTCAGGGGTCCCAGATCGATTCTCTGGCTCCAAGTCTGGCAACACAGCCACCC
TGACCATCTCTGGGCTTCAGGCTGAGGACGAGGCCGATTATTACTGTGGTTCCTATTACAGCAGTGATAG
TGCTGTATTCGGCGGAGGCACCCACCTGACCATCGCAG
>KF748631.1 Equus caballus clone IGLVJ20 immunoglobulin lambda light chain variable region (IGL) mRNA, partial cds
ATGGCCTGGTGCCCTCTCCTCCTCACCCTCATCGCTCTCTGCACAGGATCCTGGGCCCAGTCTGTGACTC
AGCCCGCCTCAGTGTCTGGGACCCTGGGCCAGACAGTCACCATCACCTGCACTGGAAGCAGCTCCAACAT
AGTTGCTTATGTGGGCTGGTACCAACAGATCCCAGGAACAGCCCCCAAAACCCTCATCTATGGTAATAAC
AAACGAGCCTCAGGGGTCCCAGATCGATTCTCTGGCTCCAAGTCTGGCAACACAGCCACCCTGACCATCT
CTGGGCTTCAGGCTGAGGACGAGGCCGATTATTACTGTGGTTCCTATTACAGCAGTGATAGTAGTGTTGT
ATTCGGCGGAGGCACCCACCTGACCATCGCAG
>KF748630.1 Equus caballus clone IGLVJ19 immunoglobulin lambda light chain variable region (IGL) mRNA, partial cds
ATGGCCTGGTCCCCTCTCCTCCTCACCCTCATCGCTCTCTGCACAGGATCCTGGGCCCAGTCTCTGACCC
AGCCCGCCTCAGTGTCTGGGACCCTGGGCCAGACAGTCACCATCTCCTGCACTGGAAGCAGCTCCAACAT
AGTTGCTTATGTGGGCTGGTACCAACAGATCCCAGGAACAGCCCCCAAAACCCTCATCTACGCTAATAAC
AAACGAGCCTCAGGGGTCCCAGATCGATTCTCTGGCTCCAAGTCTGGCAGCACAGCCACCCTGACCATCA
CTGGGCTCCAGGCTGAGGACGAGGCCGATTATTACTGTGGTACCTCTAGCAGCAGTGGTAGTGCTGTATT
CGGCGGAGGCACCCACCTGACCATCGCAG

>KF748629.1 Equus caballus clone IGLVJ18 immunoglobulin lambda light chain variable region (IGL) mRNA, partial cds
ATGGCCTGGTGCCCTCTCCTCCTCACCCTCATCGCTCTCTGCACAGGATCCTGGGCCCAGTCTGTGACTC
AGCCCGCCTCAGTGTCTGGGACCCTGGGCCAGACAGTCACCATCACCTGCACTGGAAGCAGCTCCAACAT
AGTTGCTTATGTGGGCTGGTACCAACAGATCCCAGGAACAGCCCCCAAAACCCTCATCTATGCTAATAAC
AAACGAGCCTCAGGGGTCCCAGATCGATTCTCTGGCTCCAAGTCTGGCAGCACAGCCACCCTGACCATCA
CTGGGCTCCAGGCTGAGGACGAGGCCGATTATTACTGTGGTACCTCTAGCAGCAGTGGTAGTAGTGATAT
CTTCGGCGGCGGGACCCACCTCAGCGTCCTGG
>KF748628.1 Equus caballus clone IGLVJ17 immunoglobulin lambda light chain variable region (IGL) mRNA, partial cds
ATGGCCTGGTCCCCTCTCCTCCTCACCCTCATCGCTCTCTGCACAGGATCCCGGGCCCAGTCTGTGACTC
AGCCTGCCTCAGTGTCTGGGACCCTGGGCCAGACAGTCACCATCTCCTGCTCTGGAAGCAGCTCCAACGT
TGGGAGTGGTTATGTGTCCTGGTACCAACAGATCCCAGGAACAGCCCCCAAACTCCTCATCTATTATGCC
ACTAGTAGGGCTTCCGGGGTCCCCGACCGATTCTCTGGCACCAGGTCTGGCAACACAGCCACCCTGACCA
TCTCTGGGCTCCAGGCTGAGGATGAGGCCGATTATTACTGTGGTACCTCTGGCAGCAGTTGGAGTAGTGA
TGGTGCATTCGGCGGAGGCACCCACCTGACCATCGCAG
>KF748627.1 Equus caballus clone IGLVJ16 immunoglobulin lambda light chain variable region (IGL) mRNA, partial cds
ATGGCCTGGGCTCTGTTCCTCATCACCCTCCTCACTCAGGGTACAGGGTCCTGGGGCCAGTCTGCCCTGA
TTCAGCCTTCTTCGGTGTCCGTGGCTCTAGGACAGTCGGTCACCATCTCCTGTGCTGGAAGCAGCAGTGA
CATTGGGTATTATAACTCTATTTCCTGGTACCAACAGCACCCAGGCACAACCCCAAAGCTGCTGATTTAC
TATACCAATAAGAAGCACTCAGGGATCCCTGATCGCTTCTCTGGCTCCAAGTCTGGGAACACGGCCTCCC
TGACCATCTCTGGGCTCCAGGCTGAGGATGAGGCTGAGTATTACTGTTGCTCATATGCAGGCAGTGGCAA
TTTTGCATTCGGCGGAGGCACCCACCTGACCATCGCAG
>KF748626.1 Equus caballus clone IGLVJ15 immunoglobulin lambda light chain variable region (IGL) mRNA, partial cds
ATGGCCTGGACCCCTCTCTTGTTAGCCTTCCTCTCTCTCTGCACAGGTCCTGTTGTCTCTTCTGCAGTGA
CTCAGCCATCTGAGGTGTCCGTGGCCTTGGGACAGAGAGCCACCCTCACCTGCCAGGGAAGCAACTTTGA
ATTTTTTTCTCCTAGCTGGTACCAGCAGAAGCCAGGCCAGGCCCCTGTACTGCTCATCAATATTAATAAT
GAGCGCCACTCAGGGATCCCTGAACGATTCTCCGGCTCCAGCTCAGGAGACACGTCCACACTGACCATCA
GTGGGGCCCAGGCTGAGGACGAGGCTGACTATTACTGTCTGGCAGTAGATGCTCTTAGTTCTGAAAGTGC
ATTCGGCGGAGGCACCCACCTGACCATCGCAG
>KF748625.1 Equus caballus clone IGLVJ14 immunoglobulin lambda light chain variable region (IGL) mRNA, partial cds
ATGGCCTGGACCCCTCTCTTGTTAGCCTTCCTCTCTCTCTGCACAGGTCCTGTTGTCTCTTCTGCAGTGA
CTCAGCCATCTGAGGTGTCCGTGGCCTTGGGACAGAGAGCCACCCTCACCTGCCAGGGAAGCAACTTTGA
ATTTTTTTCTCCTAGCTGGTACCAGCAGAAGCCAGGCCAGGCCCCTGTACTGCTCATCAATATTAATAAT
GAGCGCCACTCAGGGATCCCTGAACGATTCTCCGGCTCCAGCTCAGGAGACACGTCCACACTGACCATCA
GTGGGGCCCAGGCTGAGGACGAGGCTGACTATTACTGTCTGGCAGTAGATGCTCTTAGTTCTGAAACTAT
CGGCGGAGGCACCCACCTGACCATCGCAG
>KF748624.1 Equus caballus clone IGLVJ13 immunoglobulin lambda light chain variable region (IGL) mRNA, partial cds
ATGGCCTGGACCCCTCTCCTGCTCCTCCTCCTCACTCTCTGCACAGGCTCTGTGGCTTCTTCTATGCTGA
CTCAGCCACTTACCTTGTCCGTGGCCTTTGGAAGCACAGTCACTATCACATGCCAGGGAGAGCTCCTAGA
CAGTTATTATGCTGAGTGGTACCAGCAGAAGCCAGACCAGGCTCCCGTGCTGGTCATATATTATGGAAGC
AAACGTCCTTCGGGGATTTCTACCCGATTCTCTGGCTCCTACTCAAGCAAGATGGCCACCCTGACCCTCA
GTGGGGCCTTGGCCGAGGATGAGGCTGACTATTACTGTCAGGTGTGGGACAGCAGTGGTAACCAGTATAT
CTTCGGCAGCGGGACCCACCTCAGCGTCCTGG
>KF748623.1 Equus caballus clone IGLVJ12 immunoglobulin lambda light chain variable region (IGL) mRNA, partial cds
ATGGCCTGGACCCCTCTCTTGTTAGCCTTCCTCACTCTCTGCACAGGTCCCGTGGCCTCTTCTAAGCTGA
CTCAGCCATCTTCAGTGTCTGTGGCCTTGGGACAGACGGCCACCATCACCTGCAAGGGAGGCGACTTTGA
AAGTTTTGTTGGTAGCTGGTACCAGCAGAAGCCAGGCCAGGCCCCTGTGCTGGTCATCGATGCTAGTAAT
GAGCGGCCCTCAGGGATCCCTGAACGATTCTCTGGCTCCAGCTCAGGAGACACAGCCACGCTGACCATCA
GCGGGGCCCAGGCTGAGGACGAGGCTGACTATTACTGTCTGGCAGCAGATGCTTCTGATTATGATATCTT
CGGCGGCGGGACCCACCTCAGCGTCCTGG

>KF748622.1 Equus caballus clone IGLVJ11 immunoglobulin lambda light chain variable region (IGL) mRNA, partial cds
ATGGCCTGGACCCCTCTCTTGTTAGCCTTCCTCACTCTCTGCACAGGTCCCGTGGCCTCTTCTAAGCTGA
CTCAGCCATCTTCAGTGTCTGTGGCCTTGGGACAGACGGCCACCATCACCTGCAAGGGAGGCGACTTTGA
AAGTTTTGTTGGTAGCTGGTACCAGCAGAAGCCAGGCCAGGCCCCTGTGCTGGTCATCGATGCTAGTAAT
GAGCGGCCCTCAGGGATCCCTGAACGATTCTCTGGCTCCAGCTCAGGAGACACAGCCACGCTGACCATCA
GCGGGGCCCAGGCTGAGGACGAGGCTGACTATTACTGTCTGGCAGCAGATGCTTCTGATATCTTCGGCGG
CGGGACCCACCTCAGCGTCCTGG
>KF748621.1 Equus caballus clone IGLVJ10 immunoglobulin lambda light chain variable region (IGL) mRNA, partial cds
ATGGCCTGGTCCCCTCTCCTCCTCACCCTCATCGCTCTCTGCACAGGATCCTGGGCCCAGTCTCTGACCC
AGCCCGCCTCAGTGTCTGGGACCCTGGGCCAGACAGTCACCATCTCCTGCTCTGGAAGCAGCTCCAACAT
CGGGTATAGTTATAGTGCTGTGGGCTGGTACCAACAGATCCCAGGAACAGCCCCCAAAACCCTCATCTAT
GGTAATAACAAACGAGCCTCAGGGGTCCCAGATCGATTCTCTGGCTCCAAGTCTGGCAACACAGCCACCC
TGACCATCTCTGGGCTTCAGGCTGAGGACGAGGCCGATTATTACTGTGGTTCCTATTACAGCAGTGATAG
TAGTGGTGCATTCGGCGGAGGCACCCACCTGACCATCGCAG
>KF748620.1 Equus caballus clone IGLVJ9 immunoglobulin lambda light chain variable region (IGL) mRNA, partial cds
ATGGCCTGGTCCCCTCTCCTCCTCACCCTCATCGCTCTCTGCACAGGATCCTGGGCCCAGTCTCTGACTC
AGCCCGCCTCAGTGTCTGGGACCCTGGGCCAGACAGTCACCATCTCCTGCTCTGGAAGCAGCTCCAACAT
CGGGTATAGTTATAGTGCTGTGGGCTGGTACCAACAGATCCCAGGGACAGCCCCCAAAACCCTCATCTAT
GCTACTAACAAACGAGCCTCAGGGGTCCCAGATCGATTCTCTGGCTCCAAGTCTGGCAACACAGCCACCC
TGACCATCTCTGGGCTTCAGGCTGAGGACGAGGCCGATTATTACTGTGGTTCCTCATACAGCAGTGATAG
TATTGCATTCGGCGGAGGCACCCACCTGACCATCGCAG
>KF748619.1 Equus caballus clone IGLVJ8 immunoglobulin lambda light chain variable region (IGL) mRNA, partial cds
ATGGCCTGGTCCCCTCTCCTCCTCACCCTCATCGCTCTCTGCACAGGATCCTGGGCCCAGTCTCTGACTC
AGCCCGCCTCAGTGTCTGGGACCCTGGGCCAGACAGTCACCATCTCCTGCTCTGGAAGCAGCTCCAACAT
CGGGTATAGTTATAGTGCTGTGGGCTGGTACCAACAGATCCCAGGGACAGCCCCCAAAACCCTCATCTAT
GCTACTAACAAACGAGCCTCAGGGGTCCCAGATCGATTCTCTGGCTCCAAGTCTGGCAACACAGCCACCC
TGACCATCTCTGGGCTTCAGGCTGAGGACGAGGCCGATTATTACTGTGGTTCCTCATACAGCAGTGATAG
TAGTGATGGTGCATTCGGCGGAGGCACCCACCTGACCATCGCAG
>KF748618.1 Equus caballus clone IGLVJ7 immunoglobulin lambda light chain variable region (IGL) mRNA, partial cds
ATGGCCTGGTCCCCTCTCCTCCTCACCCTCATCGCTCTCTGCACAGGATCCTGGGCCCAGTCTCTGACTC
AGCCCGCCTCAGTGTCTGGGACCCTGGGCCAGACAGTCACCATCTCCTGCTCTGGAAGCAGCTCCAACAT
CGGGTATAGTTATAGTGCTGTGGGCTGGTACCAACAGATCCCAGGGACAGCCCCCAAAACCCTCATCTAT
GCTACTAACAAACGAGCCTCAGGGGTCCCAGATCGATTCTCTGGCTCCAAGTCTGGCAACACAGCCACCC
TGACCATCTCTGGGCTTCAGGCTGAGGACGAGGCCGATTATTACTGTGGTTCCTCATACAGCAGTGATAG
TAGTGATATCTTCGGCAGCGGGACCCACCTCAGCGTCCTGG
>KF748617.1 Equus caballus clone IGLVJ6 immunoglobulin lambda light chain variable region (IGL) mRNA, partial cds
ATGGCCTGGTCCCCTCTCCTCCTCACCCTCATCGCTTTCTGCACAGGATCCTGGGCCCAGTCTGTGACTC
AGCCCGCCTCAGTGTCTGGGACCCTGGGCCAGACAGTCACCATCTCCTGCTCTGGAAGCAGCTCCAACAT
CGGGAATAGTTATAGTTATGTGGGCTGGTTCCAACAGATCCCAGGAACAGCCCCCAAAACCCTCATCTAT
GGTAATAATAAACGAGCCTCAGGGGTCCCAGATCGATTCTCTGGCTCCAAGTCTGGCAACACAGCCACCC
TGACCATCTCTGGGGTCCAGGCTGAGGACGAGGCCGATTATTACTGTGGTTCCTATGACAGCAGCAGTAG
TAGTGCATTCGGCGGAGGCACCCACCTGACCATCGCAG
>KF748616.1 Equus caballus clone IGLVJ5 immunoglobulin lambda light chain variable region (IGL) mRNA, partial cds
ATGGCCTGGACCCCTCTCTTGTTAGCCTTCCTCTCTCTCTGCACAGGTCCTGTTGTCTCTTCTGCAGTGA
CTCAGCCATCTGAGGTGTCCGTGGCCTTGGGACAGAGAGCCACCCTCACCTGCCAGGGAAGCAACTTTGA
ATTTTTTTCTCCTAGCTGGTACCAGCAGAAGCCAGGCCAGGCCCCTGTACTGCTCATCAATATTAATAAT
GAGCGCCACTCAGGGATCCCTGAACGATTCTCCGGCTCCAGCTCAGGAGACACGTCCACACTGACCATCA
GTGGGGCCCAGGCTGAGGACGAGGCTGACTATTACTGTCTGGCAGTAGATGCTCTTAGTTCTGAAACGTA

TATCTTCGGCGGCGGGACCCACCTCAGCGTCCTGG
>KF748615.1 Equus caballus clone IGLVJ4 immunoglobulin lambda light chain variable region (IGL) mRNA, partial cds
ATGGCCTGGACCCCTCTCTTGTTAGCCTTCCTCACTCTCTGCACAGGTCCTATGGCCTCTTCGGAGGTGA
CTCAGCCATCTGCGGTGTCTGTGGCCTTGGGACAGACAGCCACCCTCACCTGCCAGGGAGACTACTATGA
AAGATATATTGTCAACTGGTACCAGCAGAAGCCAGGCCAGGCACCTGTGCTGGTCATCTATGCTAATAGT
GAGCGGCCCTCAGGAATCCCTGAACGATTCTCTGGCTCCAGCTCATTAGGCACATCCACGCTGACCATCA
GCGGGGCCCAGGCTGAGGATGAGGCTGACTATTACTGTCAGCCAGCAGATGCTCATAGTTCTGATGGTGC
ATTCGGCGGAGGCACCCACCTGACCATCGCAG
>KF748614.1 Equus caballus clone IGLVJ3 immunoglobulin lambda light chain variable region (IGL) mRNA, partial cds
ATGGCCTGGACCCCTCTCTTGTTAGCCTTCCTCACTCTCTGCACAGGTCCCGTGGCCTCTTCTAAGCTGA
CTCAGCCATCTTCAGTGTCTGTGGCCTTGGGACAGACGGCCACCATCACCTGCAAGGGAGGCAACTTTGA
AAGTTTTGTTGGTAGCTGGTACCAGCAGAAGCCAGGCCAGGCCCCTGTGCTGGTCATCGATCCTAGTAAT
GAGCGGCCCTCAGGGATCCCTGAACGATTCTCTGGCTCCAGCTCAGGAGACACAGCCACGCTGACCATCA
GCGGGGCCCAGGCTGAGGACGAGGCTGACTATTACTGTCTGGCAGCAGATGCTTCTGTTGGTGCATTCGG
CGGAGGCACCCACCTGACCATCGCAG
>KF748613.1 Equus caballus clone IGLVJ2 immunoglobulin lambda light chain variable region (IGL) mRNA, partial cds
ATGGCCTGGACCCCTCTCTTGTTAGCCTTCCTCACTCTCTGCACAGGTCCCGTGGCCTCTTCTAAGCTGA
CTCAGCCATCTTCAGTGTCTGTGGCCTTGGGACAGACGGCCACCATCACCTGCAAGGGAGGCAACTTTGA
AAGTTTTGTTGGTAGCTGGTACCAGCAGAAGCCAGGCCAGGCCCCTGTGCTGGTCATCGATCCTAGTAAT
GAGCGGCCCTCAGGGATCCCTGAACGATTCTCTGGCTCCAGCTCAGGAGACACAGCCACGCTGACCATCA
GCGGGGCCCAGGCTGAGGACGAGGCTGACTATTACTGTCTGGCAGCAGATGCTTCTGATTATGAAGCTTA
TATCTTCGGCAGCGGGACCCACCTCAGCGTCCTGG
>KF748612.1 Equus caballus clone IGLVJ1 immunoglobulin lambda light chain variable region (IGL) mRNA, partial cds
ATGGCCTGGACCCCTCTCTTGTTAGCCTTCCTCACTCTCTGCACAGGTCCCGTGGCCTCTTCTAAGCTGA
CTCAGCCATCTTCAGTGTCTGTGGCCTTGGGACAGACGGCCACCATCACCTGCAAGGGAGGCAACTTTGA
AAGTTTTGTTGGTAGCTGGTACCAGCAGAAGCCAGGCCAGGCCCCTGTGCTGGTCATCGATCCTAGTAAT
GAGCGCCACTCAGGGATCCCTGAACGATTCTCCGGCTCCAGCTCAGGAGACACGTCCACACTGACCATCA
GTGGGGCCCAGGCTGAGGACGAGGCTGACTATTACTGTCTGGCAGTAGATGCTCTTAGTTCTGAAACGTA
TATCTTCGGCGGCGGGACCCACCTCAGCGTCCTGG
>KF748697.1 Equus caballus clone IGLVJ86 immunoglobulin lambda light chain variable region (IGL) mRNA, partial cds
ATGGCCTGGTCCCCTCTCCTCCTCACCCTCATCGCTCTCTGCACAGGATCCTGGGCCCAGTCTGTGACTC
AGCCCGCCTCAGTGTCTGGGGCCCTGGGCCAGACAGTCGTCATCACCTGTCGTGGAAGCAGCGCCAGTAT
AGTCGCTTATGTGGGTTGGTATCAACAAGTTCCAGGAACAGCCCCCAAAACCCTCATCTATAATGATGAC
AGGAGAGCCTCAGGGGTCTCAGAGCGATTTTCCGGCTCCAAGTCTGGTAGGACAGCCACCCTGACCATCA
CTGGGCTCCAGGCTGAGGACGAGGCCGATTATTGGTGTAGTGGCTCTGATGTCACCGGCACTGTATTCGG
CGGAGCCACCCACCTGACAGTCGAAG
>KF748696.1 Equus caballus clone IGLVJ85 immunoglobulin lambda light chain variable region (IGL) mRNA, partial cds
ATGGCCTGGTCCCCTCTCCTCCTCACCCTCATCGCTAGCTGCACAGGATCCTGGGCCCAGTCTCTGACTC
AGCCCGCAGAAGTGTCTGGGACCCTGGGCCAGACAGTCACCATCTCCTGCACTGGAAGCAGCTCCAGCAG
CGGTTCCCTTGTGGGTTGGTACCAACAAATCCCAGGGACACCCCCCAAACTCCTCATTTTCTCAAGTAAG
GAGCGAGCCTCAGGGGTCCCAGATCGATTCTCGGCCTCCAGGACTGGCGCGACAGCCACCCTGACCATCA
CTGGCCTACGGGCTGAGGACGAGGCCGATTATTATTGTGCCTCTGGAAGTTATTCATTCGGCGGTGGCAC
CCGCCTGACCATCGCAG
>KF748695.1 Equus caballus clone IGLVJ84 immunoglobulin lambda light chain variable region (IGL) mRNA, partial cds
ATGGCCTGGTCCCCTCTCCTCCTCACCCTCATCGCTTTCTGCACAGGATCCTGGGCCCAGTCTGTGACTC
AACCCGCCTCAGTGTCTGGGACCCTGGGCCAGACAGTCACCATCTCCTGCTCTGGAGATAGTACAAACAT
CGATAACAGATTTAATAAGGTGGGCTGGTTCCAACAGATGCCGGGAAAAGCCCCCAAGACCCTCATCTAT
GCGAATAGTCTGAGAGCAACAGGGGTCCCAGATCGATTCTCTGGCTCCAAGTCTGGCAACACAGCGACCC
TGACCATCTCTGGGGTCCAGTATGAGGACGAGGCCGATTATTACTGCACTACTTATGACACCAGTAGTAG

GAGTCTCGTATTCGGCGGAGGCACCCACCTGACCATCGCAG
>KF748694.1 Equus caballus clone IGLVJ83 immunoglobulin lambda light chain variable region (IGL) mRNA, partial cds
ATGGCCTGGTCCCCTCTCCTCCTCACCCTCATCGCTCTCTGCACAGGGTCCTGGGCCCAAGTTGTGACTC
AGCCCGCCTCAGTGTCTGGGACCCTGGGCCAGTCAGTCACCATCTCCTGCTCTGGGACAACAATGGACAT
CGGATGGAAGCATAATATTGTGGTCTGGTTCCAACAGAGTCCAGGAACTGCCCCCAAAACCCTCATCCAT
TATGGGAACGAACGCCTCTCAGGGGTTTCAGAACGATTCTCTGGATCTCAGTCCGGCAACACAGCCACCC
TGACCATCTCGGGGGTCCAGTCTGAGGACGAGGCGACCTACTATTGTGCCTGTTATGACAGAAGTAGTGG
GCTACTTCTATTCGGCGGGGGCACCTACCTGACCATCCTAG
>KF748693.1 Equus caballus clone IGLVJ82 immunoglobulin lambda light chain variable region (IGL) mRNA, partial cds
ATGGCCTGGTCCCCTCTCCTCCTCACCCTCATCGCTCTCTGCACAGGATCCTGGGCCCAGTCTGTGACTC
AGCCCGCCTCAGTGTCTGGGACCCTGGGCCAGACAGTCACCATCTCCTGCTCTGGAAGCATCTCCACTAT
CGGGACTAGTTCCGTGGGCTGGTTCCAGCAGATCCCAGGAACAGCCCCCAAAACCCTCTTTTATGGTCAA
AACAAACGAGCCTCAGGGGTCCCAGATCGATTCTCTGCCTCCAAGTCTGGCAACACAGCCACCCTGACCA
TCTCTGGAGTCCAGGCTGAGGACGAGGCCGATTATTACTGTGCTACCAGAGACAGTGGAAGTAGTACATC
TGTATTCGGCGGGGGCACCCACCTGACCATCGCAG
>KF992247.1 Equus caballus immunoglobulin lambda variable region (IGLV8S9) gene, IGLV8S9*02 allele, partial cds
CTCAGCTTTCCGTGAGGGATCACAGCTGCCACCAGCCACCTAGCACTGCCTGGTGCCCTCTGCTCAGGGC
TCACAGCTGTGAACGCCCCACCCCAGGGCCACGCTTAGGAAGTGGCACCTGAGCAAAGGCCAAGTGAGAG
ATCAGAAAGCGGGGGCTCTGCTTTGCATGTGTGGCCCCTCCCTCTCTCAGAGTATGAAGAAGGGGTGGGA
GAGATCAGGGGGAAGCTCTGCTTCGGCTGTGGGGCCACAGAAGGCAGGACTCGGTGAAGATCTCCACCAT
GGCCTGGTCCCCTCTCCTCCTCACCCTCATCGCTCTCTGCACAGGTGACTAGATATGGGGACAAGGGAAG
GGGCCTTGGGAAGATGCGTGGGACCCTGCTTTCTCCCTTTGTCTCTAGTCCCTGGAATCACCATGTCTGT
GTCTCTCTCACTTCCAGGATCCTGGGCCCAGTCTCTGACCCAGCCCGCCTCAGTGTCTGGGACCCTGGGC
CAGACAGTCACCATCTCCTGCTCTGGAAGCAGCTCCAACATCAGGTATAGTTATAGTGCTGTGGGCTGGT
ACCAACAGATCCCAGGAACAGCCCCCAAAACCCTCATCTATGGTAATAACAAACGAGCCTCAGGGGTCCC
AGATCGATTCTCTGGCTCCAAGTCTGGCAACACAGCCACCCTGACCATCTCTGGGCTTCAGGCTGAGGAC
GAGGCCGATTATTACTGTGGTTCCTATTACAGCAGTGATAGTAGTGACACAGTGATGCAGGCCCATGGGG
AAGTGAGACAAAAACCTGCTGTGCCCTCAGCCAAGGGGCTTCCCTGTGCAGCCCCCACTCCTCGGC
>KF992246.1 Equus caballus immunoglobulin lambda variable region (IGLV8S9) gene, IGLV8S9*01 allele, partial cds
TCACAGCTGGGACCAGCCACCTAGCACTGCCTGGTGCCCTCTGCTCAGGGCTCACAGCTGTGAACGCCCC
ACCCCAGGGCCACCCTTAGGAAGTGGCACCTGAGCAAAGGCCAAGTGAGAGATCAGAAAGCGGGGGCTCT
GATTTGCATGTGTGGCCCCTCCCTCTCTCAGAGTATGAAGAAGGGGTGGGAGAGATCAGGGGGAAGCTCT
GCTTCAGCTGTGGGGCCACAGAAGGCAGGACTCGGTGAAGATCTCCACCATGGCCTGGTCCCCTCTCCTC
CTCACCCTCATCGCTCTCTGCACAGGTGACTAGATATGGGGACAAGGGAAGGGGCCTTGGGAAGATGCGT
GGGACCCTGCTTTCTCCCTTTGTCTCTAGTCCCTGGAATCACCATCTCTGTGTCTCTCTCACTTCCAGGA
TCCTGGGCCCAGTCTCTGACTCAGCCCGCCTCAGTGTCTGGGACCCTGGGCCAGACAGTCACCATCTCCT
GCTCTGGAAGCAGCTCCAACATCGGGTATAGTTATAGTGCTGTGGGCTGGTACCAACAGATCCCAGGGAC
AGCCCCCAAAACCCTCATCTATGCTACTAACAAACGAGCCTCAGGGGTCCCAGATCGATTCTCTGGCTCC
AAGTCTGGCAACACAGCCACCCTGACCATCTCTGGGCTTCAGGCTGAGGACGAGGCCGATTATTACTGTG
GTTCCTCATACAGCAGTGATAGTAGTGACACAGTGATGCAGGCCAGTAGGGAAGTGAGACAAAAACCTGC
TGTGCCCTCAGCCATGGGGCTTCCCTGTGTAGCCCCCACTCCTCGGC
>KF992245.1 Equus caballus immunoglobulin lambda variable region (IGLV8S8) gene, IGLV8S8*02 allele, partial cds
ACAATTGTGGGTCTGTGCCTGGACACCAGGGTGCGCTCAGTGCTCATTCCTGAGGTTCCTGAGGCTGATC
ATAGCTATGAACAGCCACCTGGCACTGCCTGGTACCCTCTGCTCAGGGTTCACAGCTGTGAACTCCCCAC
CCCCTGGCCATCCTTAGGCAGTGGGACCTGACACAAGGCCAGTAAGAGATGATCAGAAAGCTGAGCACTC
TGCTTTGCATGTGTGGGCCCTCCCTCTCTCAGAGTATGAAGAAGGGGTGGGAGAGATCAGGGGAAGCTCT
GCTTCAGCTGTGGGGCCACAGAAGGCAGGACTCGGTGACAATCTCCACCATGGCCTGGTCCCCTCTCCTC
CTCACCCTCATCGCTCTCTGCACAGGTGACTAGATTGGGGGACAAGGGAAGGGGCCTTGGGAAGATGCGT
GGGACCCTGCTTTCTCCCCTTGTCTCTAGTCCCTGGAATCACCATCTCTGTGTCTCTCTCACTTCCAGGA
TCCTGGGCCCAGTCTGTGACTCAGCCCGCCTCAGTGTCTGGGACCCTGGGCCAGACAGTCACCATCTCCT

GCTCTGGAAGCAGCTCCAACATCGGGTATAGTAGTAGTTATGGGGGCTGGTACCAACAGATCCCAGGAAC
AGCCCCCAAACTCCTCATATATGAGGGTAACAAACGAGCCTCAGGGGTCCCAGATCAATTCTCTGGCTCC
AAGTCTGGCAACACAGCCACCCTGACCATCTCTGGGCTCCAGGCTGAGGACGAGGCCGATTATTACTGTG
TTTCCTATGACAGCAGCCTGAGTAGTGACACAGTGCTGCAGGCCCGTGGGGAAGTGAGACAAAAACC
>KF992244.1 Equus caballus immunoglobulin lambda variable region (IGLV8S8) gene, IGLV8S8*01 allele, partial cds
ACAATTGTGGGTCTGTGCCTGGACACCAGGGTGCGCTCAGTGCTCATTCCTGAGGTTCCTGAGGCTGATC
ATAGCTATGAACAGCCACCTGGCACTGCCTGGTACCCTCTGCTCAGGGTTCACAGCTGTGAACTCCCCAC
CCCCTGGCCATCCTTAGGCAGTGGGACCTGACACAAGGCCAGTAAGAGATGATCAGAAAGCTGAGCACTC
TGCTTTGCATGTGTGGGCCCTCCCTCTCTCAGAGTATGAAGAAGGGGTGGGAGAGATCAGGGGAAGCTCT
GCTTCAGCTGTGGGGCCACAGAAGGCAGGACTCGGTGACAATCTCCACCATGGCCTGGTTCCCTCTCCTC
CTCACCCTCATCGCTCTCTGCACAGGTGACTAGATTGGGGGACAAGGGAAGGGGCCTTGGGAAGATGCGT
GGGACCCTGCTTTCTCCCCTTGTCTCTAGTCCCTGGAATCACCATCTCTGTGTCTCTCTCACTTCCAGGA
TCCTGGGCCCAGTCTGTGACTCAGCCCGCCTCAGTGTCTGGGACCCTGGGCCAGACAGTCACCATCTCCT
GCTCTGGAAGCAGCTCCAACATCGGGTATAGTAGTAGTTATGGGGGCTGGTACCAACAGATCCCAGGAAC
AGCCCCCAAACTCCTCATATATGAGGGTAACAAACGAGCCTCAGGGGTCCCAGATCAATTCTCTGGCTCC
AAGTCTGGCAACACAGCCACCCTGACCATCTCTGGGCTCCAGGCTGAGGACGAGGCCGATTATTACTGTG
TTTCCTATGACAGCAGCCTGAGTAGTGACACAGTGCTGCAGGCCCGTGGGGAAGTGAGACAAAAACCTGC
TGTGCCCTC
>KF992243.1 Equus caballus immunoglobulin lambda variable region (IGLV8S7) gene, IGLV8S7*01 allele, partial cds
TGGCCCCTCCCTCTCTCAGAGTATGAAGAAGGGGTGGGAGAGATCAGGGGAAGCTCTGCTTCAGCTGTGG
GGCCACAGAAGGCAGGACTCGGTGACAATCTCCACCATGGCCTGGTGCCCTCTCCTCCTCACCCTCATCG
CTCTCTGCACAGGTGACTAGATATGGGGACAAGGGAAGGGGCCTTGGGAAGATGCGTGGGACCCTGCTTT
CTCCCCTTGCCTCTAGTCCCTGGAATCACCATCTCTGTGTCTCTCTCACTTCCAGGATCCTGGGCCCAGT
CTGTGACTCAGCCCGCCTCAGTGTCTGGGACCCTGGGCCAGACAGTCACCATCACCTGCACTGGAAGCAG
CTCCAACATAGTTGCTTATGTGGGCTGGTACCAACAGATCCCAGGAACAGCCCCCAAAACCCTCATCTAC
GCTAATAACAAACGAGCCTCAGGGGTCCCAGATCGATTCTCTGGCTCCAAGTCTGGCAGCACAGCCACCC
TGACCATCACTGGGCTCCAGGCTGAGGACGAGGCCGATTATTACTGTGGTACCTCTAGCAGCAGTGGTAG
TAGTGACACAGTGCTGCAGGCCTGTGGGGAAGTGAGACAAAAACCTGCTGTCCCCTCAGTGATGGGGCTT
CTCTGTGCAGCCCCCACTCCTCGGC
>KF992242.1 Equus caballus immunoglobulin lambda variable region (IGLV8S6) gene, IGLV8S6*01 allele, partial cds
TGGCCCCTCCCTCTCTCAGAGCATGAAGAAGGGGTGGGAGAGATCAGGGGGAAGCTCTGCTTCAGCTGTG
GGGCCACTGAAGGCAGGACTCGGTGACAATCTCCACCATGGCCTGGTCCCCTCTCCTCCTCACCCTCATC
GCTCTCTGCACAGGTGACTACATATGGGGATAAGAGAAGGGGACCGGGGAAGATGCCTGGGACCCTGCTT
TCTCTTGTCTCTAGACTCCAGAATCACCATCTCTGTGTCTCTCTCACTTCCAGGATCCCGGGCCCAGTCT
GTGACGCAGCCCGCCTCAGTGTCTGGGACCCTGGGCCAGACAGTCACCATCTCCTGCTCTGGAAGCAGCT
CCAACATCGGGAGTGGTCATGTGTCCTGGTACCAACAGATCCCAGGAACAGCCCCCAAACGCCTCATCTA
TTCTTCCGCTAGCAGGGCTTCCAGGGTCCCCGACCGATTCTCTGGCTCCAGGTCTGGCAACACAGCCACC
CTGACCATCTCTGGGCTCCAGGGTGAGGACGAGGCCGATTATTACTGTGGTACATTGTACAGCAGTTGGA
GTAGTGACACAGTGCTGCAGGCCTGTGGGGAAGTGAGACAAAAACCTGCTGTCCCCCCAGTGATGGGGCT
TCTCTGTGCAGCCCCCACTCCTCGGC
>KF992241.1 Equus caballus immunoglobulin lambda variable region (IGLV8S2) gene, IGLV8S2*03 allele, partial cds
TTCACAGCTGCGACCAGCCACCTAGCACTGCCTGGTGCCNTCTGCTCAGGGATCACAGCTGTGAACGCCC
CACCCCAGGGCCACCCTTAGGAAGTGGCACCTGAGCAAAGGCCAAGTGAGAGATCAGAAAGCGGGGGCTC
TGATTTGCATGTGTGGGCCCTCCCTCTCTCAGAGCATGAAGAAGGGGTGGGAGAGATCAGGGGGAAGCTC
TGCTTCAGCTGTGGGGCCACAGAAGGCAGGACTCGGTGAAGATCTCCACCATGGCCTGGTCCCCTCTCCT
CCTCACCCTCATCGCTCTCTGCACAGGTGACTAGATATGGGGACAAGGGAAGGGGCCTTGGGAAGATGCG
TGGGACCCTGCTTTCTCCCCTTGTCTCTAGTCCCTGGAATCACCATCTCTGTGTCTCTCTCACTTCCAGG
ATCCTGGGCCCAGTCTGTGACTCAGCCCGCCTCAGTGTCTGGGACCCTGGGCCAGACAGTCACCATCTCC
TGCTCTGGAAGCAGCTCCAACATCGGGAATAGTTATAGTTATGTGGGCTGGTACCAACAGATCCCAGGAA
CAGCCCCCAAAACCCTCATCTATGGTAATAACAAACGAGCCTCAGGGGTCCCAGATCGATTCTCTGGCTC
CAAGTCTGGCAACACAGCCACCCTGACCATCACTGGGGTCCAGGCTGAGGACGAGGCTGACTATTACTGT
GCTACTTATGACAGCAGCCTGAGTAGTGACACAGTGCTGCAGGCCCATGGGGAAGTGAGACAAAAACCTG

CTGTCCCCTCAGCAATGGGTCTTCCCTGTGGAGCCCCCACTCCTCGGC
>KF992240.1 Equus caballus immunoglobulin lambda variable region (IGLV8S2) gene, IGLV8S2*02 allele, partial cds
TGGGCCCTCCCTCTCTCAGAGTATGAAGAAGGGGTGGGAGAGATCAGGGGGAAGCTCTGCTTCAGCTGTG
GGGCCACAGAAGGCAGGACTCGGTGAAGATCTCCACCATGGCCTGGTCCCCTCTCCTCCTCACCCTCATC
GCTTTCTGCACAGGTGACTAGATATGGGGACAAGGGAAGGGGCCTTGGGAAGATGGGTGGGACCTTGATT
TCTCCCCTTGTCTCTAGTCCCTGGAATCACCATCTCTGTGTCTCTCTCACTTCCAGGATCCTGGGCCCAG
TCTGTGACTCAGCCCGCCTCAGTGTCTGGGACCCTGGGCCAGACAGTCACCATCTCCTGCTCTGGAAGCA
GCTCCAACATCGGGAATAGTTATAGTTATGTGGGCTGGTACCAACAGATCCCAGGAACAGCCCCCAAAAC
CCTCATCTATGGTAATAACAAACGAGCCTCAGGGGTCCCAGATCGATTCTCTGGCTCCAAGTCTGGCAAC
ACAGCCACCCTGACCATCACTGGGGTCCAGGCTGAGGACGAGGCTGACTATTACTGTGCTACTTATGACA
GCAGCCTGAGTAGTGACACAGTGCTGCAGGCCCATGGGGAAGTGAGACAAAAACCTGCTGTCCCCTCAGC
AATGGGTCTTCCCTGTGGAGCCCCCACTCCTCGGC
>KF992239.1 Equus caballus immunoglobulin lambda variable region (IGLV8S2) gene, IGLV8S2*01 allele, partial cds
TGGCCCCTCCCTCTCTCAGAGTATGAAGAAGGGGTGGGAGAGATCAGGGGGAAGCTCTGCTTCAGCTGTG
GGGCCACAGAAGGCAGGACTCGGTGAAGATCTCCACCATGGCCTGGTCCCCTCTCCTCCTCACCCTCATC
GCTTTCTGCACAGGTGACTAGATATGGGGACAAGGGAAGGGGCCTTGGGAAGATGGGTGGGACCTTGATT
TCTCCCCTTGTCTCTAGTCCCCGGAATCACCATCTCTGTGTCTCTCTCACTTCCAGGATCCTGGGCCCAG
TCTGTGACTCAGCCCGCCTCAGTGTCTGGGACCCTGGGCCAGACAGTCACCATCTCCTGCTCTGGAAGCA
GCTCCAACATCGGGAATAGTTATAGTTATGTGGGCTGGTTCCAACAGATCCCAGGAACAGCCCCCAAAAC
CCTCATCTATGGTAATAATAAACGAGCCTCAGGGGTCCCAGATCGATTCTCTGGCTCCAAGTCTGGCAAC
ACAGCCACCCTGACCATCTCTGGGGTCCAGGCTGAGGACGAGGCCGATTATTACTGTGGTTCCTATGACA
GCAGCAGTAGTAGTGACACAGTGCTGCAGGCCCGTGGGGAAGTGAGACAAAAACCTGCTGTCCCCTCAGC
CATAGGGCTTCCCTGTGCAGCCTCCACTCCTCCGC
>KF992238.1 Equus caballus immunoglobulin lambda variable region (IGLV8S1) gene, IGLV8S1*04 allele, partial cds
GGGGCTGTGATTTGCATGTGTGGCCCCTCCCTCTCTCAGAGTATGAAGAAGGGGTGGGAGAGATCAGGGG
GAAGCTCTGCTTCAGCTGTGGGGCCACAGAAGGCAGGACTCGGTGAAGATCTCCACCATGGCCTGGTCCC
CTCTCCTCCTCACCCTCATCGCTCTCTGCACAGGTGACTAGATATGGGGACAAGGGAAGGGGCCTTGGGA
AGATGCGTGGGACCCTGCTTTCTCCCTTTGTCTCTAGTCCCTGGAATCACCATCTCTGTGTCTCTCTCAC
TTCCAGGATCCTGGGCCCAGTCTCTGACTCAGCCCGCCTCAGTGTCTGGGACCCTGGGCCAGACAGTCAC
CATCTCCTGCTCTGGAAGCAGCTCCAACATCGGGTATAGTTATAGTGCTGTGGGCTGGTACCAACAGATC
CCAGGAACAGCCCCCAAAACCCTCATCTATGGTAATAACAAACGAGCCTCAGGGGTCCCAGATCGATTCT
CTGGCTCCAAGTCTGGCAACACAGCCACCCTGACCATCTCTGGGGTCCAGGCTGAGGACGAGGCCGATTA
TTACTGCTCAGCAGGAGACAGCAGTGGTAGTAGTGACACAGTGCTGCAGGCCCGTGGGGAAGTGAGACAA
AAACCTGCTGTGCCCTCAGCCATTGGGCTTCCCTGTGCAGCCCCCACTCCTCGGC
>KF992237.1 Equus caballus immunoglobulin lambda variable region (IGLV8S1) gene, IGLV8S1*03 allele, partial cds
TCACAGCTGTGACCACCCACCTAGCACTGCCTGGTGCCCTCTGCTCAGGGCTCATCCCTGTGAACGCCCC
ACCCCAGGGCCACCCTTAGGAAGTGGCACCTGAGCAAAGGCCAAGTGAGAGATCAGAAAGCGGGGGCTGT
GATTTGCATGTGTGGGCCCTCCCTCTCTCAGAGCATGAAGAAGGGGTGGGAGAGATCAGGGGGAAGCTCT
GCTTCAGCTGTGGGGCCACAGAAGGCAGGACTCGGTGAAGATCTCCACCATGGCCTGGTCCCCTCTCCTC
CTCACCCTCATCGCTCTCTGCACAGGTGACTAGATATGGGGACAAGGGAAGGGGCCTTGGGAAGATGCGT
GGGACCCTGCTTTCTCCCCTTGCCTCTAGTCCATTGAATCACCATCTCTGTGTCTCTCTCACTTCCAGGA
TCCTGGGCCCAGTCTCTGACTCAGCCCGCCTCAGTGTCTGGGACCCTGGGCCAGACAGTCACCATCTCCT
GCTCTGGAAGCAGCTCCAACATCGGGAATAGTTATAGTTCTGTGGGCTGGTTCCAACAGATCCCAGGAAC
AGCCCCCAAAACCCTCATCTATGGTAATAACAAACGAGCCTCAGGGGTCCCAGATCGATTCTCTGGCTCC
AAGTCTGGCAACACAGCCACCCTGACCATCTCTGGGGTCCAGGCTGAGGACGAGGCTGACTATTACTGTG
CAGCAGGAGACAGCAGCCTTAATGGTGACACAGTGCTGCAGGCCCGTGGGGAAGTGAGACAAAAACCTGC
TGTCCCCTCAGCCATGGGGTTTCCCTGTGCAGCCCCCACTCCTCGAC
>KF992236.1 Equus caballus immunoglobulin lambda variable region (IGLV8S1) gene, IGLV8S1*02 allele, partial cds
TGGGCCCTCCCTCTCTCAGAGCATGAAGAAGGGGTGGGAGAGATCAGGGGGAAGCTCTGCTTCAGCTGTG
GGGCCACAGAAGGCAGGACTCGGTGAAGATCTCCACCATGGCCTGGTCCCCTCTCCTCCTCACCCTCATC

GCTCTCTGCACAGGTGACTAGATATGGGGACAAGGGAAGGGGCCTTGGGAAGATGCGTGGGACTCTGCTT
TCTCCCCTTGCCTCTAGTCCATTGAATCACCATCTCTGTGTCTCTCTCACTTCCAGGATCCTGGGCCCAG
TCTCTGACTCAGCCCGCCTCAGTGTCTGGGACCCTGGGCCAGACAGTCACCATCTCCTGCTCTGGAAGCA
GCTCCAACATCGGGTATAGTTATAGTGCTGTGGGCTGGTACCAACAGATCCCAGGAACAGCCCCCAAAAC
CCTCATCTATGGTAATAACAAACGAGCCTCAGGGGTCCCAGATCGATTCTCTGGCTCCAAGTCTGGCAAC
ACAGCCACCCTGACCATCTCTGGGGTCCAGGCTGAGGACGAGGCCGATTATTACTGCTCAGCAGGAGACA
GCAGTGGTAGTAGTGACACAGTGCTGCAGGCCCGTGGGGAAGTGAGACAAAAACCTGCTGTGCCCTCAGC
CATTGGGCTTCCCTGTGCAGCCCCCACTCCTCGGC
>KF992235.1 Equus caballus immunoglobulin lambda variable region (IGLV8S1) gene, IGLV8S1*01 allele, partial cds
TGGGCCCTCCCTCTCTCAGAGCATGAAGAAGGGGTGGGAGAGATCAGGGGGAAGCTCTGCTTCAGCTGTG
GGGCCACAGAAGGCAGGACTCGGTGAAGATCTCCACCATGGCCTGGTCCCCTCTCCTCCTCACCCTCATC
GCTCTCTGCACAGGTGACTACATATGGGGACAAGGGAAGGGGCCTTGGGAAGATGCGTGGGACCCTGCTT
TCTCCCCTTGCCTCTAGTCCATTGAATCACCATCTCTGTGTCTCTCTCACTTCCAGGATCCTGGGCCCAG
TCTCTGACTCAGCCCGCCTCAGTGTCTGGGACCCTGGGCCAGACAGTCACCATCTCCTGCTCTGGAAGCA
GCTCCAACATCGGGTATAGTTATAGTGCTGTGGGCTGGTACCAACAGATCCCAGGAACAGCCCCCAAAAC
CCTCATCTATGGTAATAACAAACGAGCCTCAGGGGTCCCAGATCGATTCTCTGGCTCCAAGTCTGGCAAC
ACAGCCACCCTGACCATCTCTGGGGTCCAGGCTGAGGACGAGGCCGATTATTACTGCTCAGCAGGAGACA
GCAGTGGTAGTAGTGACACAGTGCTGCAGGCCCGTGGGGAAGTGAGACAAAAACCTGCTGTGCCCTCAGC
CATTGGGCTTCCCTGTGCAGCCCCCACTCCTCGGCCGTGTCAGCTGCTTCCTTTG
>KR190601.1 Equus caballus immunoglobulin lambda light chain variable region (IGLV8-128) gene, IGLV8-128*04 allele, partial cds
AGGGGGAAGCTCTGCTTCAGCTGTGGGGCCACAGAAGGCAGGACTCGGTGAAGATCTCCACCATGGCCTG
GTCCCCTCTCCTCCTCACCCTCATCGCTCTCTGCACAGGTGACTAGATATGGGGACAAGGGAAGGGGCCT
TGGGAAGATGGGTGGGACCTTGATTTCTCCCCTTGTCTCTAGTCCCTGGAATCACCATCTCTGTGTCTCT
CTCACTTCCAGGATCCTGGGCCCAGTCTGTGACTCAGCCCGCCTCAGTGTCTGGGACCCTGGGCCAGACA
GTCACCATCTCCTGCTCTGGAAGCAGCTCCAACATCGGGTATAGTTATAGTTATGTGGGCTGGTTCCAAC
AGATCCCAGGAACAGCCCCCAAAACCCTCATCTATGGTAATAACAAACGAGCCTCAGGGGTCCCAGATCG
ATTCTCTGGCTCCAAGTCTGGCAACACAGCCACCCTGACCATCTCTGGGGTCCAGGCTGAGGACGAGGCC
GATTATTACTGTGGTTCCTATGACAGCAGCAGTAGTAGTGACACAGTGCTGCAGGCCCGTGGGGAAGTGA
GACAAAAACCTG
>HM176304.1 Equus caballus clone 1L255 immunoglobulin lambda light chain V-J region mRNA, partial cds
ATGGCCTGGTCCCCTCTCCTCCTCACCCTCATCGCTTTCTGCACAGGATCCTGGGCCCAGTCCTTGACTC
AGCCCGCCTCAGTGTCTGGGACCCTGGGCCAGACAGTCACCATCACCTGCTCTGGAAGCAGTTCCAACAT
CGGGGCACATCATGTGGGCTGGTTCCAACAGATCCCAGGAACAGCCCCCAAAACCCTCATCTATAGAGAT
TATTTACGACCCTCAGGGGTCCCAGATCGATTTTCTGGCTCCAGGTCTGGCAACGTAGCCACCCTGACCA
TCTCTGGGGTCCAGGCTGAGGACGAGGCCGATTATTACTGTGGTTCCGCTGACAGCGGCAGCAGTGCTGT
ATTCGGCGGAGGCACCCACCTGACCATCGCAGGTGGTCCCACGTCTACACCCTCGGTCTCTCTCTTCCCG
CCCTCCTCTGAGGAGCTCAGCGCCAACAAGGCCACAGTGGTGTGTCTCATCAGTGACTTCTCCCCCAGCG
GCCTGGAGGTGATCTGGAAGGTAAATGACGCTGTCACCACCGACGGCGTCCAGACCACCAGGTCCTCGAA
ACAGAGCAACGGTAAGTACGCGGCCAGCAGCTACCTGACGCGGACTTCCGCACAGTGGAAATCGTACAGC
AGCGTCAGCTGCCAGG
>HM176291.1 Equus caballus clone 2LB67 immunoglobulin lambda light chain V-J region mRNA, partial cds
ATGGCCTGGTCCCCTCTCCTCCTCACCCTCATCGCTCTCTGCACAGCATCCTGGGCCCAGTCTGTGACTC
AGCCCGCCTCAGTGTCTGGGACCCTGGGCCAGACAGTCACCATCTCCTGCTCTGGAAGCAGCGGCAACAT
TGGTCGTTATGGTGTTGGCTGGTTCCAACAGATCCAAGGAAAAGCCCCCAAAACCGTCATCTATGGTAAT
AACAAACGACCCTCAGGGGTCCCAGATCGATTTTCTGGCTCCAAGTCTGGCAACACAGCCACCCTGACCA
TCTCTGGGCTCCAGGCTGAGGACGAGGGTGATTATTACTGTGGTACCCATGACACAAGTAGTGGTAGTCC
TGTATTCGGCGGGGGCACCCACCTGACCATCGCAGGTGGTCCCACGTCTACACCCTCGGTCTCTCTCTTC
CCGCCCTCCTCTGAGGAGCTCAGCGCGAACAAGGCCACAGTGGTGTGTCTCATCAGTGACTTCTCCCCCA
GCGACTTGACGGTGAGCTGGAAGGTAAATGGCGCCGCCACCACCCAGGGCGTCCAGACCACCAAGCCCTC
GAAACAGAGCAACGGCAAGTACGCAGCCAGCAGCTACCTGTCGCTGTCCCCCAGCCAGT

2 Supplementary Figures

2.1 Supplementary Figure S1

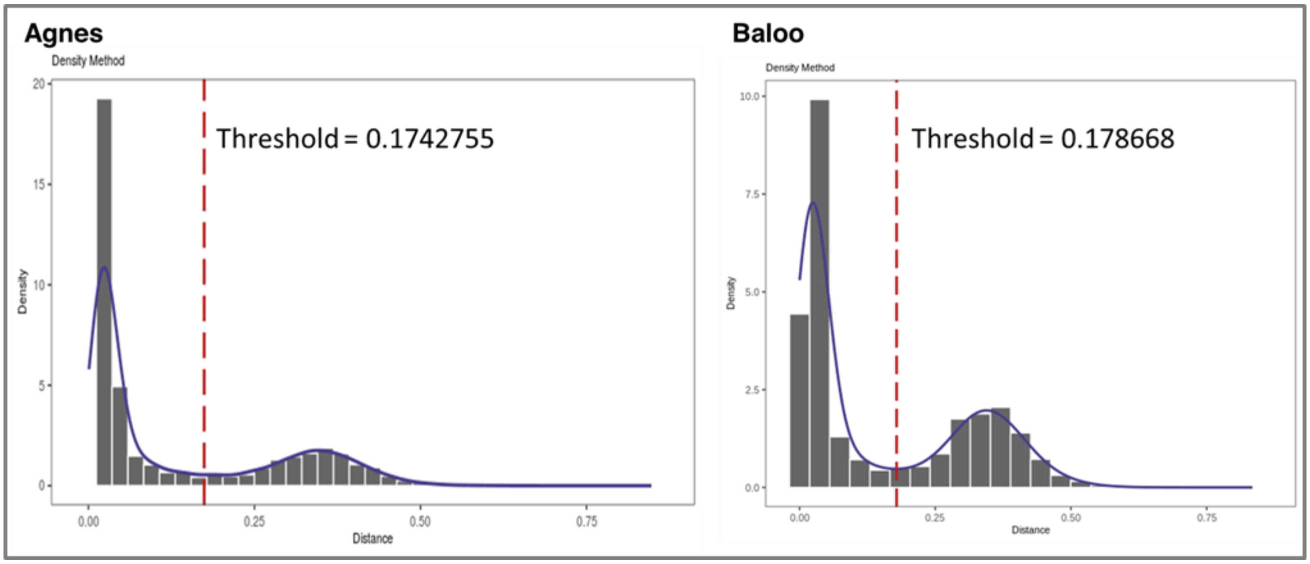

Supplementary Figure S1. Hamming distance histograms. The histograms represent the distance to nearest-neighbor distribution, for both Agnes and Baloo horse sequenced Ig repertoires in the libraries. The histograms were generated by the distToNearest function in the SHazaM R package (see Ref 25 in the main text). The clustering threshold is represented by the dotted red line, and its value, calculated by the findThreshold function in the SHazaM R package, is indicated.

2.2 Supplementary Figure S2

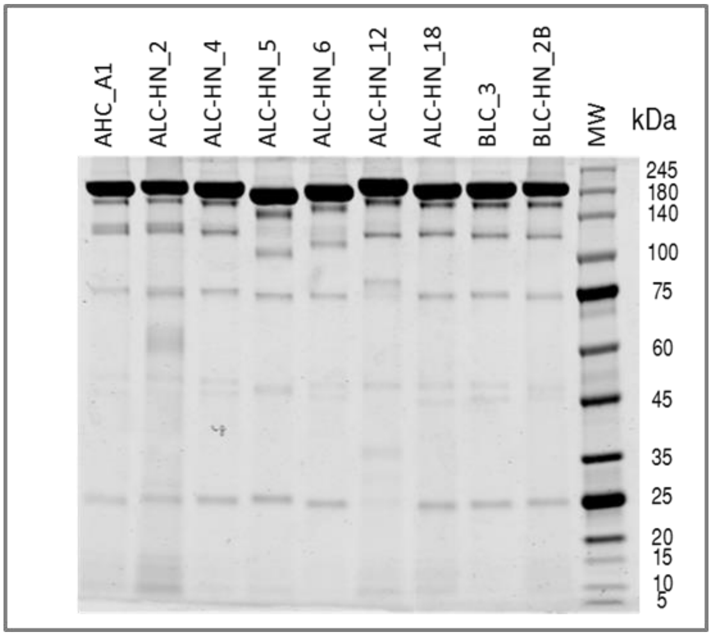

Supplementary Figure S2. SDS-PAGE analysis of purified Centaur mAbs. The indicated mAbs were expressed in CHO cells, purified on HiTrap Protein-A column and analyzed by SDS-PAGE. 7 µg of each mAb were boiled at 100°C for 5 min in sample buffer (Laemmli SB; BIORAD, USA) and loaded on each lane. Electrophoresis was performed in 1.5 mm thick NuPAGETM (4-12% Bis-Tris; Invitrogen, USA), run at 80V for 15 min and at 130 V for additional 60 min. The gel was stained with InstantBlue Coomassie (Expedeon, UK). PM2700 MW (Smobio, Taiwan) protein size markers, are indicated.

2.3 Supplementary Figure S3

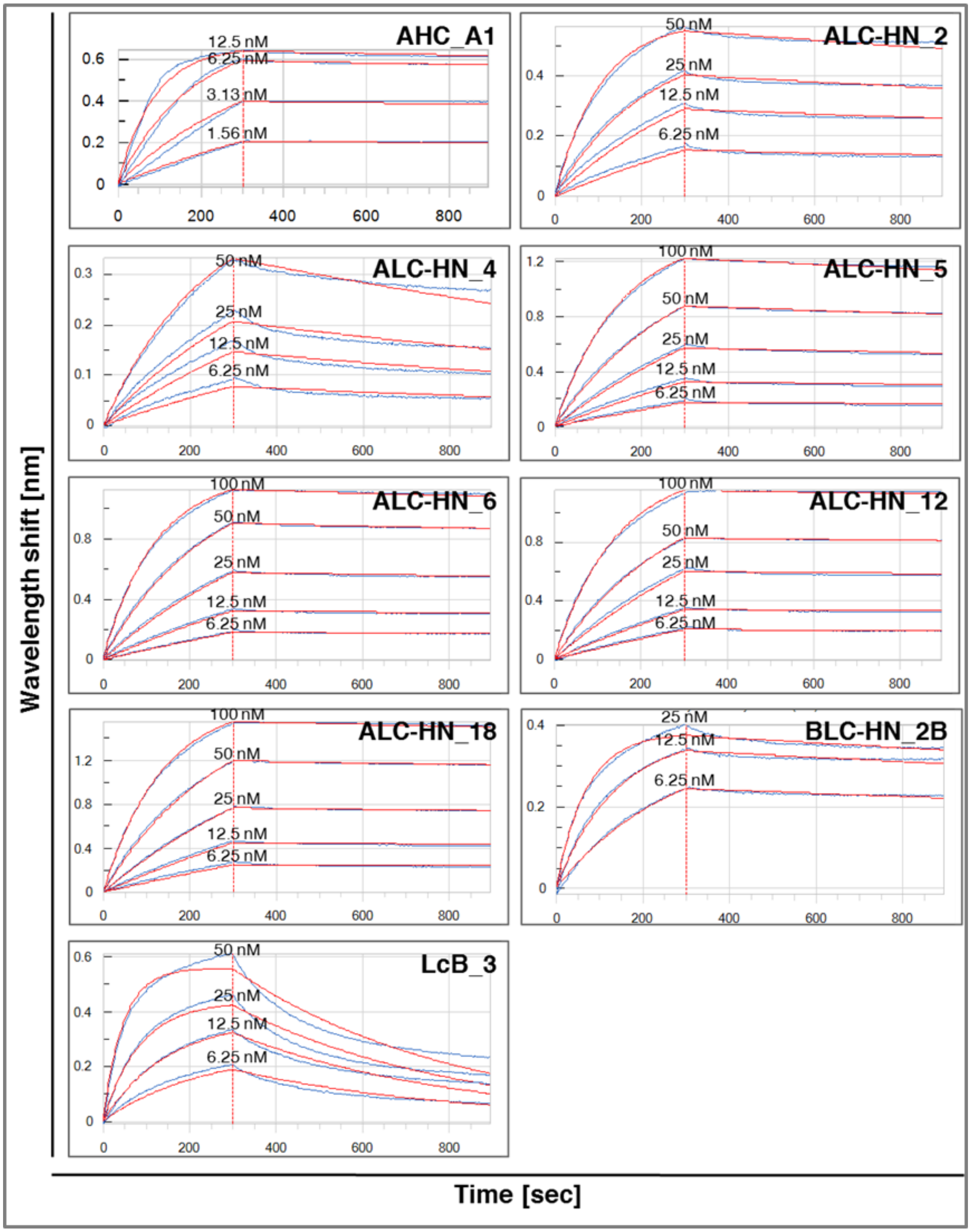

Supplementary Figure S3. Determination of binding kinetic parameters of the Centaur mAbs using BLI. Streptavidin-coated biosensors were loaded with each of the indicated biotinylated horse-human chimeric Centaur mAbs and reacted for 300 seconds with the indicated concentrations of targeted recombinant antigen (association phase) and then transferred to buffer-containing wells for another 600 seconds (dissociation phase). Sensorgrams (after subtraction of parallel measurements from unloaded biosensors) were fitted with a 1:1 binding model (red curves) using the Octet data analysis software 8.1. At least two independent measurement repeats performed for each mAb.

2.4 Supplementary Figure S4

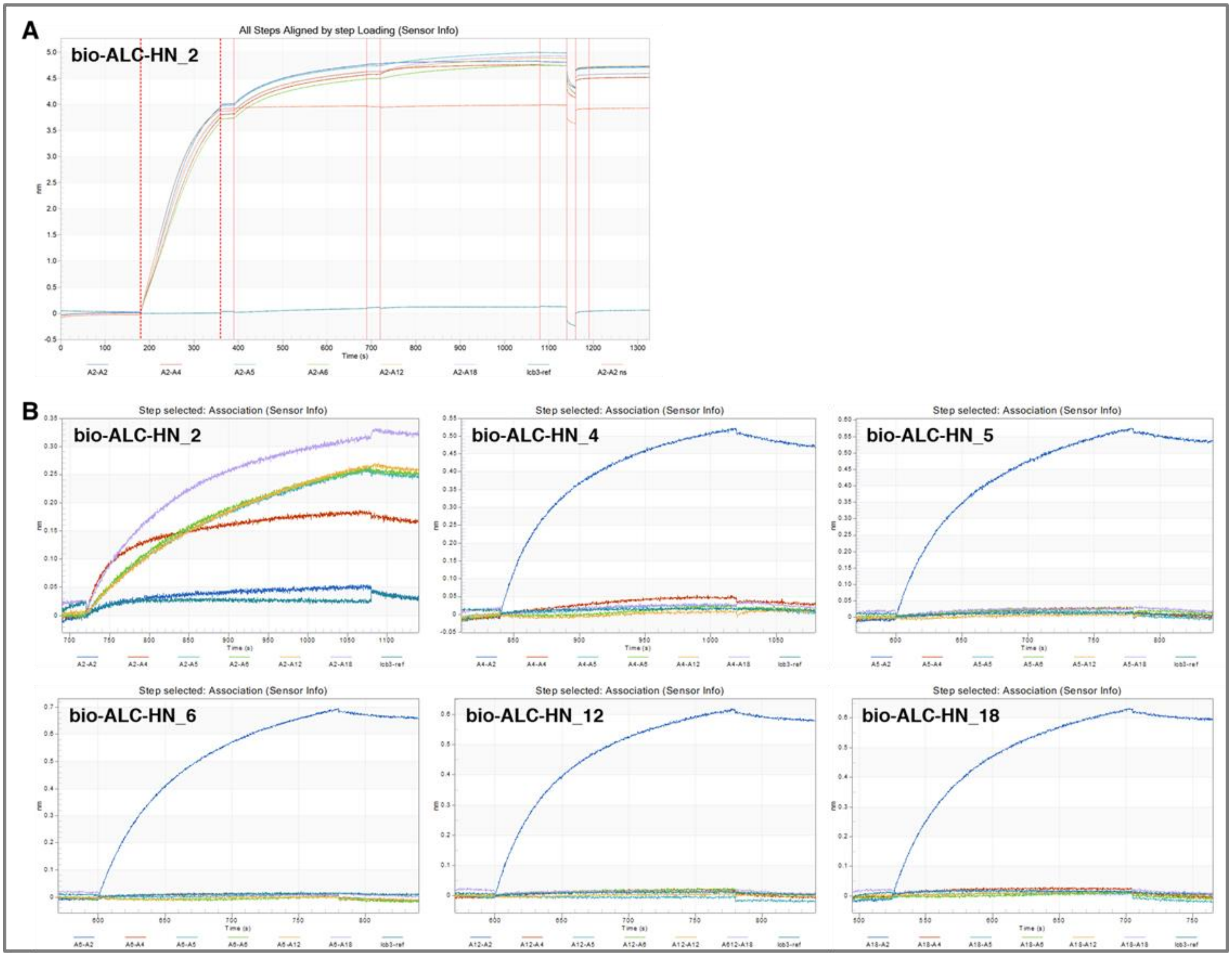

Supplementary Figure S4. Epitope binning of anti-LC-HN/A Centaur mAbs. (A) Streptavidin-coated biosensors were loaded with biotinylated ALC-HN_2 antibody (bio-ALC-HN_2) and reacted for 200 seconds with LC-HN/A recombinant protein, washed and then incubated with each of the six selected anti-LC-HN/A antibodies. (B) Presentation of the last step, representing the binding capacity of each mAb to a pre-complex of LC-HN/A with the indicated bio-mAb. Binding phase of 300 seconds applied for ALC-HN_2 and 180 seconds for the other mAbs. In each set of experiments, the background signal was obtained from a parallel sensor incubated with the homologous antibody (non-biotinylated) and sensograms of the last step are presented after subtraction of the background signal.
